# Proneural and mesenchymal glioma stem cells display major differences in splicing and lncRNA profiles

**DOI:** 10.1038/s41525-019-0108-5

**Published:** 2020-01-16

**Authors:** Gabriela D. A. Guardia, Bruna R. Correa, Patricia Rosa Araujo, Mei Qiao, Suzanne Burns, Luiz O. F. Penalva, Pedro A. F. Galante

**Affiliations:** 1 Centro de Oncologia Molecular, Hospital Sírio-Libanês, São Paulo, São Paulo 01309-060 Brazil; 2 Children’s Cancer Research Institute, UT Health San Antonio, San Antonio, TX 78229 USA; 3 Department of Cell Systems and Anatomy, UT Health San Antonio, San Antonio, TX 78229 USA; 4 Present Address: Centre for Genomic Regulation (CRG), The Barcelona Institute of Science and Technology, Barcelona, 08003 Catalonia Spain

**Keywords:** Cancer genomics, Cancer stem cells, Gene expression analysis, Gene regulatory networks

## Abstract

Therapy resistance and recurrence in high-grade gliomas are driven by their populations of glioma stem cells (GSCs). Thus, detailed molecular characterization of GSCs is needed to develop more effective therapies. We conducted a study to identify differences in the splicing profile and expression of long non-coding RNAs in proneural and mesenchymal GSC cell lines. Genes related to cell cycle, DNA repair, cilium assembly, and splicing showed the most differences between GSC subgroups. We also identified genes distinctly associated with survival among patients of mesenchymal or proneural subgroups. We determined that multiple long non-coding RNAs with increased expression in mesenchymal GSCs are associated with poor survival of glioblastoma patients. In summary, our study established critical differences between proneural and mesenchymal GSCs in splicing profiles and expression of long non-coding RNA. These splicing isoforms and lncRNA signatures may contribute to the uniqueness of GSC subgroups, thus contributing to cancer phenotypes and explaining differences in therapeutic responses.

## Introduction

High-grade (Grades III and IV) gliomas are the most common malignant brain tumors in adults. Glioblastoma (GBM, grade IV) in particular is highly invasive and refractory to conventional therapy; GBM patients have an average survival of 15 months.^1^ Therapy resistance and relapse are driven by glioma stem cells (GSCs), which comprise a small subpopulation of tumorigenic cells displaying stem-like properties: self-renewal, persistent proliferation, and ability to generate progeny of multiple lineages.^2^ Therefore, characterization of their biological properties, expression profile, and regulation is critical for creating new therapeutic strategies. GSCs are categorized based on molecular and phenotypic differences.^3^ For instance, mesenchymal (MES) GSCs have higher rates of proliferation in vitro,^4^ mice that received MES GSCs developed brain tumors at a much faster rate,^4^ and MES GSCs are more resistant to radiation than proneural (PN) GSCs.^4^ In addition, primary PN GBM, originally responsive to treatment, may relapse as MES tumors which become refractory to treatment. Two explanations for this change have been proposed: (i) PN-MES transition, in which PN GSCs are triggered to switch to a MES phenotype upon treatment; and (ii) tumor heterogeneity: MES GSCs already in primary PN tumors are more resistant to treatment and then take over, driving growth of secondary tumors.^5^

Transcriptomic analyses of GSCs have established a list of subtype-specific markers^6^ and defined changes in their expression levels in response to radiation.^7^ Additional studies have focused on mutation profiles and methylation status.^8^ Here we expand the characterization of GSCs by focusing on RNA-mediated mechanisms. Splicing profiles of MES and PN GSC lines show major differences affecting genes implicated in cell cycle regulation, DNA repair, cilium assembly, and RNA splicing. Additionally, we found long non-coding RNAs (lncRNAs) preferentially expressed in each GSC subgroup, with some exhibiting prognostic value.

## Results

### Splicing profiles define GSC subgroups

**Fig. 1:**
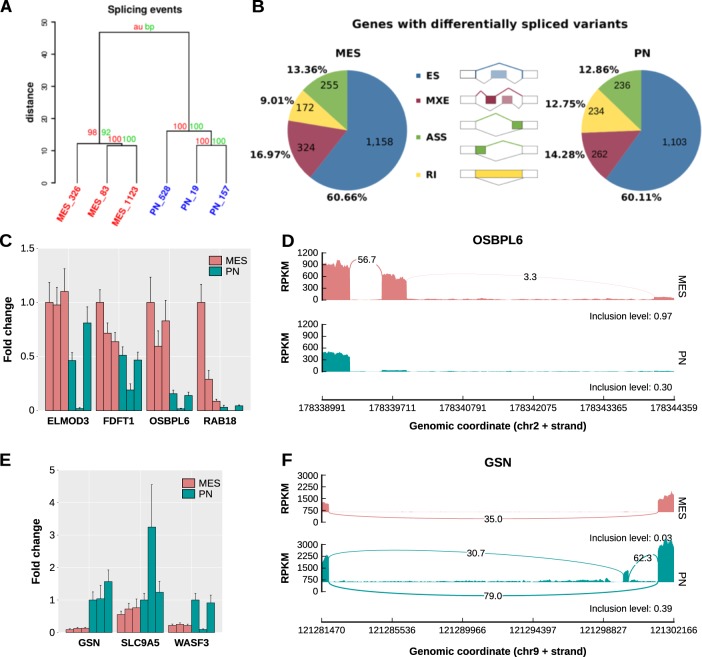
Mesenchymal and proneural GSCs have distinct alternative splicing profiles, but a similar number of genes harboring alternative splicing events. **a** Hierarchical cluster showing that GSCs can be grouped based on their alternative splicing profile (PSI values). Bootstrap (bp) and approximately unbiased (AU) probability from pvclust.^89^ **b** Percentage of events and number of genes harboring exon skipping (ES), mutually exclusive exons (MXE), alternative 3′ or 3′ splice site (ASS), and retained introns (RI) events in MES or PN GSCs. **c** Quantitative RT-PCR (qRT-PCR) validation of four exon-skipping events in PN and MES GSCs. ELMOD3, FDFT1, OSBPL6, and RAB18 are official gene names. **d** An exon- skipping event (gene OSBPL6; second exon) in PN and MES GSCs. **e** qRT-PCR validation of three exon-skipping events in MES and PN GSCs. GSN, SLC9A5, and WASF3 are official gene names. **d** An exon-skipping event (gene GSN; second exon) in MES and PN GSCs.

To assess the contribution of alternative splicing to GSC phenotypes and identify relevant differences between MES and PN GSCs, we performed RNA sequencing of three mesenchymal (MES-83, MES-326, MES-1123) and three PN GSC (PN-19, PN-157, and PN-528) cell lines (Supplementary Table 1). RNA-seq analysis revealed that MES and PN GSCs showed differences in 4934 splicing events affecting 3253 genes (|ΔPSI| > 0.1 and false discovery rate [FDR] < 0.05, likelihood-ratio test); among these, 1793 events were not reported in the reference transcriptome (GENCODE version 26; Supplementary Table 2). Using only the splicing profiling, we correctly clustered samples according to their subgroups (Fig. 1a). MES and PN GSCs showed a similar number of genes presenting alternative splicing (AS) events of exon skipping (ES), mutually exclusive exons (MXE), intron retention (IR), and alternative 5′/3′ splice sites (ASS; Fig. 1b). We randomly selected exon-skipping events to be validated by quantitative RT-PCR (Supplementary Table 3, Fig. 1c–f). All were in concordance (higher fold change, on average) with the ES “direction” indicated by RNA-seq data: ELMOD3, FDFT1, OSBPL6, and RAB18 have skipped exons in PN GSCs (Fig. 1c, d; Supplementary Fig. 1a), while GSN, SLC9A5, and WASF3 have skipped exons in MES GSCs (Fig. 1e, f; Supplementary Fig. 1b).

**Fig. 2:**
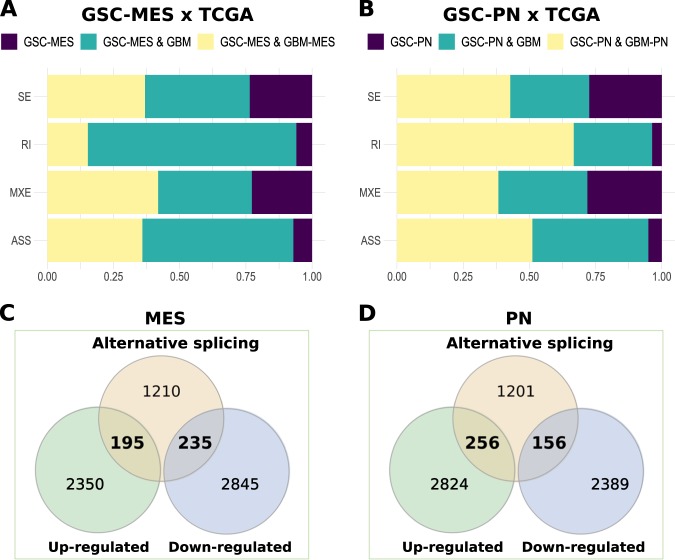
Proneural and mesenchymal GSCs share most of their AS events with GBM samples. **a** Percentage of alternative splicing events from GSCs in GBM samples. GSC-MES & GBM-MES contains AS events found in GSC MES and GBM (molecular subtype) mesenchymal. GSC-PN & GBM-PN contains AS events found in GSC PN and GBM (molecular subtype) proneural. GSC-MES & GBM and GSC-PN & GBM contains AS events found in GSC-MES and GSC-PN and GBM samples (all molecular subtypes), respectively. GSC-MES and GSC-PN contain AS events not found in GBM samples. **b** Intersection between differentially spliced and differentially expressed genes (up- and downregulated) in the two GSC subtypes.

Moreover, we used GBM samples from The Cancer Genome Atlas (TCGA) to determine which AS events found in PN GSC cell lines compared to MES are also confirmed in GBM. We found 78.7% (3,882/4,934; Supplementary Table 4) of AS isoforms from GSCs in GBM samples. Among the AS subtypes, IR and alternative splice sites (ASS) events were more highly match to GBM (>90%; Fig. 2a, b, classes in green plus yellow) than ES and MXE (~75%; Fig. 2a, b, classes in green plus yellow). Additionally, ~35% of AS events from MES GSC have agreement with GBM mesenchymal AS events (Fig. 2a, classes in yellow) and ~50% of AS events from PN GSC have agreement with GBM proneural AS events (Fig. 2b, classes in yellow). By performing gene ontology (GO) analysis with protein-coding genes sharing agreement in AS events (|ΔPSI| > 0.1 and FDR < 0.05, likelihood-ratio test) between PN GSC and GBM versus MES GSC and GBM, several biological processes commonly involved in brain tumors (e.g., neurogenesis, cell division and epithelial-mesenchymal transition)^9^ were enriched (FDR < 0.05, Fisher's exact test) (Supplementary Fig. 2), indicating that AS events in MES and PN GSCs are reliable and potentially important to GBM maintenance.

Next, we evaluated if differences in splicing profile between PN and MES GSCs affect mRNA levels. First, we identified 6393 differentially expressed genes between MES and PN GSCs, 5,625 of which were multi-exonic: 2545 were upregulated in MES GSCs and 3,080 were upregulated in PN GSCs (|log2FC| > 1 and *FDR* < 0.05, Wald test; Supplementary Table 5). We also explored differences in gene expression observed between MES and PN GSCs in MES and PN GBM samples from TCGA. We confirmed higher expression of 756 and 924 genes in MES and PN GBM (|log2FC| > 0; FDR < 0.05, Wald test; Supplementary Table 6), respectively. Considering the heterogeneity in GBM and that GSCs represent only a subpopulation of their cells, this low agreement (29.7%; (756 + 924)/5625)) in gene sets with differential expression between PN or MES GSCs and PN or MES GBMs was expected. Among splicing variants prevalent in MES GSCs (|ΔPSI| > 0.1 and FDR < 0.05, likelihood-ratio test), 195 genes (11.89%) had higher levels of expression, while 235 genes (14.33%) had lower levels of expression in MES compared to PN GSCs. In the case of splicing variants prevalent in PN GSCs (|ΔPSI| > 0.1 and FDR < 0.05, likelihood-ratio test), 256 genes (15.87%) were upregulated and 156 genes (9.67%) downregulated in PN GSCs compared to MES GSCs (Fig. 2c, d, Supplementary Table 7). Overall, splicing variants prevalent in MES GSCs were more often associated with down-regulated genes in the same subgroup compared to splicing variants prevalent in PN GSCs (*p*-value = 0.0001; chi-square 28.83; d.f. = 1).

### Genes displaying splicing differences between PN and MES GSCs are implicated in survival of GBM patients

**Fig. 3:**
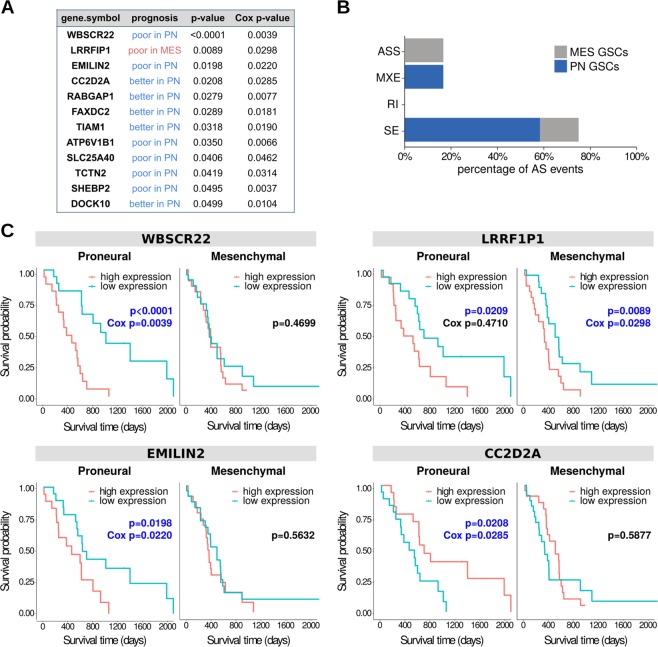
Differentially expressed genes in mesenchymal or proneural GSCs are related to GBM patient prognosis of patients with GBM. **a** Protein-coding genes with higher expression in proneural GSCs (showed in blue) or mesenchymal GSCs (showed in red) are exclusively associated with better versus poor prognosis in GBM patients with tumors of molecular subtypes proneural or mesenchymal subtypes of GBM, respectively. **b** Genes associated with survival in GBM patients are listed by type of alternative splicing event detected in the GSCs: ES, exon skipping; IR, intron retention; MXE, mutually exclusive exons; ASS, alternative splice sites 3′ or 5′. **c** Survival curves of four genes (presented in **a**) exclusively affecting patients with GBM proneural or mesenchymal GBM. Significant log-rank *p*-values and multivariate Cox *p*-values are shown in blue.

We investigated whether expression levels of protein-coding genes with splicing differences in MES vs. PN GSCs influence survival of GBM patients with one type of GBM but not the other one. Using data from TCGA, we created Kaplan-Meier survival curves to separately explore correlations between expression levels of each gene and patient survival. Genes exclusively associated with prognosis of MES or PN GBM were then included in multivariate Cox proportional-hazards regression models to adjust for the effects of clinical variables. Several genes exclusively affected prognosis of patients with PN versus MES GBM (log-rank *p*-value < 0.05 and multivariate Cox *p*-value < 0.05; Supplementary Table 8, Fig. 3a–c). Most genes predicted prognostic outcomes exclusively in PN GBM and present more splicing events in PN GSCs (Fig. 3b). In particular, 11 genes that are upregulated in PN GSCs are also associated with prognostic outcome in patients with PN but not MES GBM. Six genes were associated with poor prognostic outcomes, i.e., samples with higher expression of these genes (based on their median expression values) were associated with decreased survival compared to samples exhibiting lower expression of that gene. The other five genes were associated with increased survival when highly expressed. Co-expression analysis in TCGA GBM samples revealed weak or no correlations among these genes (|Spearman’s coefficient| < 0.8; Supplementary Table 9), suggesting that their impact on prognosis is independent. In the univariate analysis, only LRRFIP1, which is more highly expressed in MES GSCs, predicted prognosis in both GBM molecular subgroups. However, when considered with other clinical parameters in the multivariate Cox analysis, this gene remained significantly associated only with prognosis of MES GBM.

### Genes displaying splicing differences in PN and MES GSCs are implicated in mRNA splicing, DNA repair, cell division, and cilium assembly

To elucidate the biological function of genes with differences in splicing profiles between PN and MES GSCs, we first focused on cancer-related genes (Supplementary Fig. 3, Supplementary Table 10), using a consensus list of cancer driver genes identified in three studies.^10–12^ From this list, we identified 72 genes displaying splicing differences between PN and MES GSCs; 26 of these also showed differences in expression (Supplementary Table 11).

**Fig. 4:**
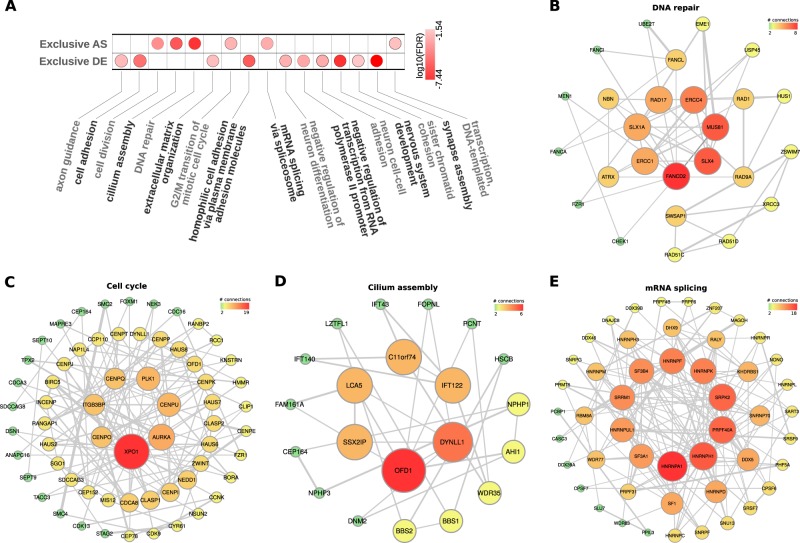
Proneural and mesenchymal GSCs have genes with alternative splicing and differential expression related to key biological processes in tumorigenesis and sets of highly connected networks of genes. **a** Gene Ontology (Biological Process) enrichment analysis of genes harboring alternative splicing events and genes differentially expressed genes in proneural versus mesenchymal types of GSCs. The networks based on protein-protein interactions display genes associated with **b** DNA repair; **b** cell cycle; **c** cilium assembly; and **d** mRNA splicing.

We also conducted GO analysis using the DAVID web tools^13^ and protein network analysis using the STRING database^14^ to determine if genes displaying differences in splicing levels are associated with particular biological processes. For genes displaying differences exclusively in splicing profiles and no significant changes in expression, the top enriched terms were cell cycle, cilium assembly, DNA repair, transcription, and mRNA splicing (FDR < 0.05, Fisher's exact test; Fig. 4a, Supplementary Table 12). Genes showing differences in expression levels without significant splicing events were associated with a different set of GO terms, in particular cell adhesion and neuronal function (FDR < 0.05, Fisher's exact test; Fig. 4a, Supplementary Table 13). Next, we conducted protein interaction analyses with gene sets associated with DNA repair, cell cycle, cilium assembly, and mRNA splicing; all showed a highly connected gene network (Fig. 4b–e).

### Splicing regulators potentially driving isoform preference

**Fig. 5:**
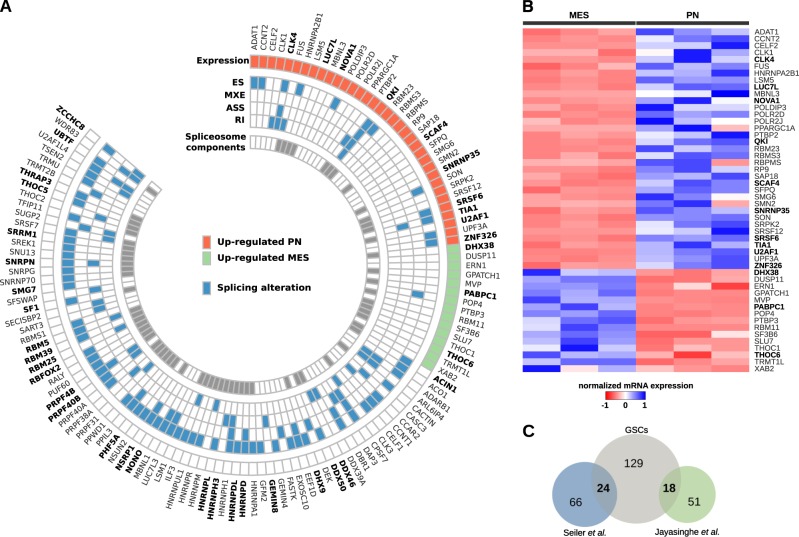
Mesenchymal and proneural GSCs have splicing-related genes with distinct profile of alternative splicing and/or differentially expressed. **a** Splicing-related genes differentially expressed and/or harboring alternative splicing events in mesenchymal versus proneural GSCs. Mutated genes are shown in bold. **b** Differentially expressed splicing-related genes in MES and PN GSCs. Genes reported to harbor mutations are shown in bold. **c** Number of splicing-related genes in our study that harbor mutations according to studies by Seiler and colleagues^16^ and Jayasinghe and colleagues^17^.

To determine which splicing regulators are potentially driving different splicing profiles in PN versus MES GSCs, we analyzed the expression and splicing profiles of 388 splicing-related RBPs, comprising 66 components of the spliceosome^15^ (Supplementary Table 14). Among them, 50 (12.9%) were differentially expressed: 35 were upregulated in PN GSCs while 15 were upregulated in MES GSCs (|log2FC| > 1 and *FDR* < 0.05, Wald test; Fig. 5a, b, Supplementary Table 15). From this group, 19 RBPs showed the same differences in expression in GBM MES vs. PN samples from TCGA (Supplementary Table 15). We also identified 188 events affecting 95 splicing-related RBPs that differed between PN and MES GSCs (Fig. 5a) (|ΔPSI| > 0.1 and FDR < 0.05, likelihood-ratio test; Supplementary Table 16). Moreover, 39 RBPs from the group of splicing factors showing expression and/or splicing differences between PN and MES GSCs were identified in recent meta-studies that used TCGA data to map mutations affecting multiple tumor types^16,17^ (Fig. 5c, Tables S17 and S18).

**Fig. 6:**
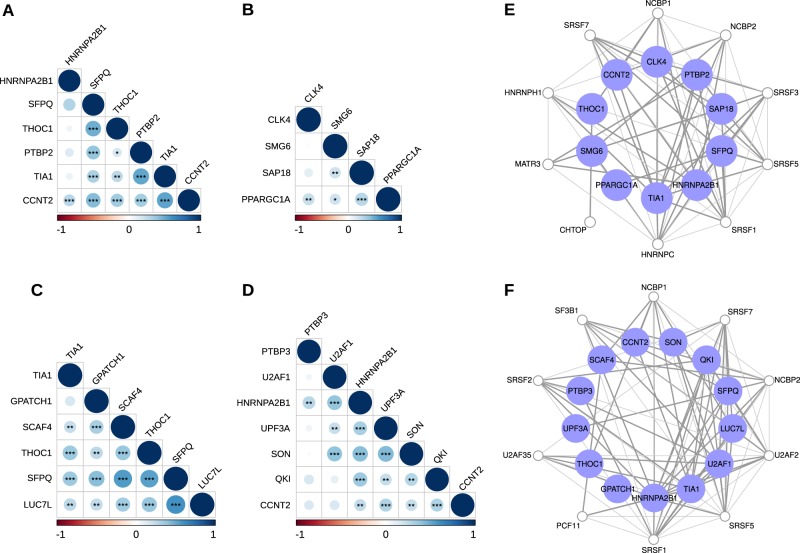
RBPs co-expressed in mesenchymal and proneural GBM samples. Correlation plots display RBPs positively correlated in (**a, b**) mesenchymal GBM; and (**c, d**) proneural GBM (Spearman’s significance: **p-value < 0.01; ***p-value < 0.001). Interaction networks show RBPs functionally related from (**e**) mesenchymal GBM and (**f**) proneural GBM. Blue nodes represent selected RBPs, while white nodes were additionally included in the network to give a broader context of associations between the RBPs. Only experimentally validated associations are shown in gray.

We investigated co-expression patterns among differentially expressed RBPs in MES and PN GSCs using Spearman’s rank correlation (Supplementary Fig. 4a, b, respectively). Correlation patterns were then confirmed in GBM samples from TCGA. We found two sets of positively correlated RBPs in MES GBM (Fig. 6a, b) and two other sets in PN GBM (Fig. 6c, d). Network analysis showed that these correlated RBPs also display functional interaction (Fig. 6e, f, respectively), indicating that these groups of related RBPs may act together to regulate distinct sets of splicing events and may regulate one another by modulating inclusion of some alternative exons.^18^

### lncRNAs display differences in expression and isoform preferences in PN vs. MES GSCs

**Fig. 7:**
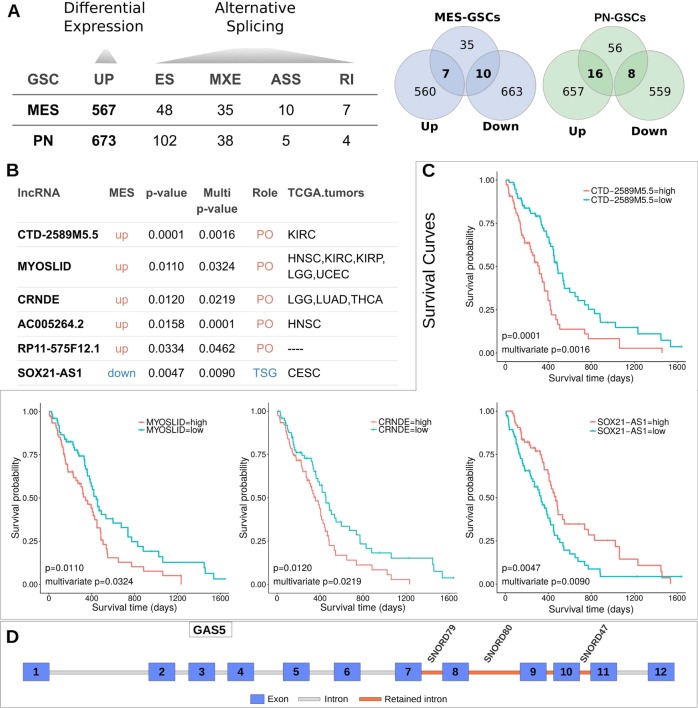
Mesenchymal and proneural GSCs present lncRNAs differentially expressed, harboring alternative splicing events, and associated with prognosis in GBM patients. **a** Number of lncRNAs presenting alternative splicing events or differentially expressed (left side) and their intersections (right side) in MES or PN GSCs. **b** Differentially expressed lncRNAs in GSCs with prognostic value to GBM and other cancer patients (“TCGA tumors” follows TCGA nomenclature for cancer types (e.g., LGG low grade glioma)). PO Proto-oncogenes; TSG tumor-suppressor genes. **c** Survival curves based on median expression values of lncRNAs in GBM patients. Corresponding log-rank p-values and multivariate Cox *p*-values are also shown. **d** Representation of lncRNA GAS5 and its retained introns (in red) in MES GSC. Retained introns host three small nucleolar RNAs: SNORD79, SNORD80, and SNORD57.

Aberrant expression of lncRNAs has been described in numerous cancer types, and a growing number of lncRNAs are implicated in malignant transformation.^19^ We investigated expression of lncRNAs in MES and PN GSCs. First, we found 1240 differentially expressed lncRNAs between MES and PN GSCs (Fig. 7a, Supplementary Table 19) and clustered GSCs based only on their lncRNA profile (Supplementary Fig. 5). Next, we investigated whether these differences are also observed in MES versus PN GBM tumors from TCGA (|log2FC| > 0 and *FDR* < 0.05, Wald test). We found 357 genes in concordance between GSCs and GBM tumors (i.e., upregulated in MES GSC and upregulated in MES GBM; downregulated in MES GSC and downregulated in MES GBM; same pattern as PN GSCs and PN GBM; Supplementary Table 20). The levels of agreement (28.7%; 357/1240) for differentially expressed lncRNAs were similar to those for coding genes (29.7%) and splicing isoforms between PN and MES GSCs and PN and MES GBM tumors.

We also queried all lncRNAs differentially expressed between MES and PN GSCs to determine if their expression levels might have prognostic value in GBM and other tumor types (Supplementary Table 21). We used univariate Cox proportional-hazards regression models to explore correlations between expression of lncRNAs and patient survival (Cox *p*-value < 0.05). Each lncRNA significantly correlated with GBM survival was then included in a multivariate Cox model with multiple covariates relevant to GBM prognosis. We created Kaplan-Meier survival curves for lncRNAs that remained significantly associated with patient survival in multivariate analyses (Cox *p*-value < 0.05). Six lncRNAs had significant association with prognosis of GBM (Cox *p*-value < 0.05; Supplementary Table 22, Supplementary Fig. 6). Of these, 5 (83.3%) displayed increased expression in MES GSCs and 5 (83.3%) were also linked to survival in other tumor types (Fig. 7b, c). In a second analysis, we evaluated the prognostic value of differentially expressed lncRNAs separately in MES and PN subgroups. Three and five lncRNAs were associated with survival rates in MES and PN GBM samples, respectively (multivariate Cox *p*-value < 0.05, log-rank *p*-value < 0.05; Supplementary Table 23, Figs. S7 and S8).

Despite having fewer exons per transcript on average compared to coding genes, alternative splicing occurs relatively often in lncRNAs.^20^We found 249 alternative splicing events (108 lncRNAs) that differed between MES and PN GSCs (|ΔPSI| > 0.1 and FDR < 0.05, likelihood-ratio test; Fig. 7A; Supplementary Table 24). When we compared differentially expressed and differentially spliced lncRNAs in MES and PN GSCs, 34 appeared on both lists (13 lncRNAs upregulated in MES GSCs and 21 upregulated in PN GSCs; Supplementary Table 25). A few lncRNAs displayed differences in multiple splicing events; these were GAS5 (23 events), PVT1 (5 events), TPT1-AS1 (14 events), PSMA3-AS1 (10 events), and CD27-AS1 (6 events).

Some lncRNAs are particularly complex. For example, GAS5 harbors several snoRNAs in its transcripts.^21^ The splicing differences observed between PN and MES (IR events) affect expression of snorD79, snorD80, and snorD47 (Fig. 7d). The latter has been described as a potential tumor suppressor in GBM, and associated with reduced proliferation, invasion and epithelial-mesenchymal transition, and induction of G2 cell-cycle arrest.^22^

## Discussion

Alternative splicing affects all hallmarks of cancer, and splicing regulators have been shown to function as drivers of glioblastoma development.^23^ Importantly, there are several examples of inhibitors of the splicing machinery and their use in cancer therapy has started to be explored.^24,25^ Here, we provide evidence that MES and PN GSCs display specific splicing profiles. Splicing differences preferentially affect genes implicated in cell cycle regulation, DNA repair, splicing, and cilium formation, which are all critical processes in therapy response and tumor relapse. We suggest that these sets of splicing events are important contributors to MES and PN features and differences in responding to radiation and temozolomide. In agreement, we observed that several genes whose splicing profiles differ between MES and PN GSCs show distinct association with survival in each subgroup.

GBM tumors contain radio-resistant GSCs that display increased pair capacity and, upon radiation, activate DNA damage checkpoint signals. MES GSCs are more resistant to radiation than PN GSCs, which can be explained based on differences in their expression profiles.^26–29^ A recent analysis with paired GBM samples (primary and recurrent) indicated that relapse was often associated with significant alterations in the expression of DNA repair and cell-cycle genes.^30^ In concordance, our analysis showed that multiple DNA repair genes display splicing differences between MES and PN GSCs. In particular, genes in DNA damage and integrity checkpoints are enriched among those with differences in splicing profiles. Regarding major nodes in the DNA repair network (Fig. 4a), ERCC1, FANCD2, and RAD17 have been associated with therapy resistance in gliomas and other tumor types. In a preclinical study, inhibition of ERCC1 significantly decreased tumor growth and sensitized cells to chemotherapy with cisplatin and temozolomide, increasing cell death by up to 25%.^31^ Also, methylation of the ERCC1 promoter was associated with sensitivity to radiation in glioma cell lines.^32^ Expression levels of FANCD2, a member of the Fanconi anemia DNA repair pathway, were strongly associated with glioma tumor grade, and inhibition of FANCD2 improved sensitivity to temozolomide and carmustine in glioma cells.^33^ Finally, certain splicing variants of gene RAD17 promote resistance to radiotherapy in cell lines.^34^

GSCs employ cell cycle regulation mechanisms to circumvent the effects of chemotherapy and radiotherapy.^35^ There are critical differences between PN and MES GBM regarding cell cycle regulation that could influence therapy response. For instance, CDK4/6 inhibition with palbociclib preferentially inhibited cell proliferation of PN GSCs.^36^ Alterations in cell cycle checkpoint can contribute to GSC resistance to radiation.^37^ Higher expression of RAD51 after exposure to radiation was observed in radio-resistant GSCs. GSCs resistant and sensitive to radiation have different cell cycle checkpoint responses when radiation was combined with RAD51 inhibition.^30^ RAD51, RAD51D, and RAD51AP1 show differences in their splicing profiles in PN vs. MES GSCs. The main nodes in the cell cycle network (Fig. 4b) contain critical players in glioma progression and response to therapy. For example, high expression of XPO1, also known as CRM1, correlates with malignancy and poor survival outcome in gliomas.^38^ High expression of XPO1 provides a growth advantage to glioma cells by promoting nuclear export of p27, a known cell cycle regulator with reduced expression in various tumors.^38^ Another main node, PLKI, has been associated with therapy resistance in gliomas and other brain tumors when highly expressed.^39^ In a recent study, Koncar et al. established PLK1 as a potential therapeutic target in IDH1-mutated gliomas.^40^ PLK1 bypasses the temozolomide-induced DNA damage checkpoint, limiting its effectiveness. Therefore, PLK1 inhibitors may improve temozolomide efficacy.

The protein kinase encoded by AURKA is a central regulator of mitotic processes, such as chromosomal segregation, chromatin condensation, and mitotic checkpoints. AURKA expression levels correlate with malignancy grade in gliomas. Its inhibition decreases cell proliferation, induces G2/M cell cycle arrest, and produces synergistic effects with radiation in GBM cell lines.^41^ Another study showed that AURKA regulates self-renewal and tumorigenicity of GSCs by activating the Wnt signaling pathway.^42^ Other nodes of the cell cycle network include centromere proteins (CENPU, CENPO, and CENPQ). Upregulation of CENPU has been reported in several malignancies, including GBM.^43^

We found that multiple genes implicated in cilia formation show splicing differences between PN and MES GSCs. There is growing evidence that cilia participate in gliomagenesis, including cell signaling activation, cell proliferation, apoptosis, and participation in therapeutic resistance.^44,45^ The proposed function of primary cilia is to limit GBM proliferation, and its loss leads to increased proliferation.^46^ However, others have proposed that cilia can induce or suppress tumorigenesis and is influenced by the oncogenic driver event.^44^ Between 1 and 30% of cells in glioblastoma samples are ciliated, and study in GBM cell lines shows that they rarely gave rise to cilia.^47^ Given that primary cilium has emerged as a key component in cancer development and alterations are observed during tumor development (including gliomas), the impact of splicing alterations on the function and expression of central nodes of the identified network (OFD1, DYNLL1, SSX2IP, LCA5, C11orf74, and IFT122) warrants further investigation.

We also determined that splicing differences between PN and MES GSCs preferentially affect genes implicated in splicing regulation. In the mRNA splicing network of genes with differences in splicing profiles (Fig. 4d), heterogeneous nuclear ribonucleoproteins (hnRNPs) occupy a central position. They regulate various post-transcriptional and translational processes, including alternative splicing and mRNA stabilization. hnRNPH is overexpressed in gliomas and regulates the splicing of RON and IG20, producing isoforms that promote survival, proliferation, and migration and invasion of GBM cells.^48^ hnRNPA1 has a central role in the let-7a/c-Myc/HNRNPA1/PKM2 signaling pathway via activating PKM2 expression and thus increasing aerobic glycolysis and cell proliferation in gliomas.^49^ hnRNPC overexpression in more aggressive glioma cells correlates with increased migratory and invasive activities through regulation of PDCD4.^50^ Similarly, hnRNPK has been associated with increased migration and invasion capabilities and MES transformation of GSCs through interactions with RTVP-1 and N-WASP.^51^ Finally, hnRNPM has been implicated in resistance to temozolomide in GBM.^52^

Differences in expression levels of RBPs implicated in splicing regulation are likely the main drivers of PN and MES splicing profiles. As indicated by expression correlation and network analyses, specific groups of associated RBPs might coordinately regulate distinct groups of splicing events. We highlight a specific set of RBPs: CLK4, PHF5A, PRPF40B, QKI, THOC6, TIA1, and U2AF1. These RBPs are frequently mutated in GBM and show differential splicing and expression in MES versus PN GSCs, and in GBM samples from TCGA compared to normal brain samples from the Genotype-Tissue Expression dataset.^53^ Expression variation of some of these RBPs has been associated with gliomas and other tumor types. In particular, CLK4 was included in a 6-gene signature that predicts cell proliferation of high-grade glioma cultures after in vitro treatment with the tyrosine kinase inhibitor sunitinib.^54^ PHF5A, a component of the spliceosome machinery, maintains proper exon recognition of C-rich 3′ splice sites in GSCs derived from GBM patients, and its knockdown leads to cell cycle arrest and loss of viability.^55^ Depletion of PRPF40B modulates ASS selection of apoptotic genes through direct interactions with SF1 and U2AF2, leading to decreased cell survival.^56^ Deletion of QKI, a known tumor suppressor, maintains stemness of GSCs and decreases differentiation in suboptimal environments.^57^ QKI influences splicing in many solid tumors,^58^ and is implicated in epithelial-to-mesenchymal transition.^59^ Finally, one of the most frequently mutated RBPs, U2AF1, affects alternative splicing in different tumor types. For example, in lung adenocarcinoma, alterations in splicing driven by U2AF1 induce cell cycle dysregulation and mitotic stress.^60^

Expression levels of splicing regulators preferentially affect genes implicated in RNA processing and other RNA-related processes.^61^ The spliceosome machinery may regulate itself by modulating inclusion of some alternative exons.^18^ Overall, our results indicate that alternative splicing signatures and the status of RNA processing components contribute to maintain the MES and PN phenotypes of GSCs. We have previously shown in a functional screening in GBM that several splicing regulators are among highly expressed RBPs affecting cancer phenotypes.^61^ Importantly, a growing number of specific splicing factor inhibitors are being identified, and their use in cancer therapy is gaining momentum.^24^

Differences in lncRNA expression and splicing profiles have been observed across tissues and during development, and impact several diseases, including cancer.^20^ In the second part of our study, we identified differences in expression and splicing profiles of lncRNAs between the two GSC subgroups. We established that the lncRNA profile also defines MES and PN GSCs. Among lncRNAs displaying expression differences between PN and MES GSCs, we identified six lncRNAs associated with survival in GBM and other cancers: CTD-2589M5.5, MYOSLID, CRNDE, AC005264.2, SOX21-AS1 and RP11-575F12.1. MYOSLID, CRNDE, and SOX21-AS1 have been characterized in the context of tumorigenesis. MYOSLID was correlated with tumor size, stage, invasion, and survival time in gastric cancer, and its knockdown inhibited tumorigenesis in mouse xenografts. MYOSLID acts as a ceRNA of miR-29c-3p, causing de-repression of anti-apoptotic gene MCL-1.^62^ MYOSLID was also defined as potential biomarker in head and neck squamous cell carcinoma, where it promotes invasion and metastasis by modulating the epithelial-mesenchymal transition.^63^ SOX21-AS1 has been linked to tumor progression and defined as a prognostic marker in cervical cancer, nephroblastoma, hepatocellular carcinoma, and lung and oral cancer.^64–69^ In glioblastoma, SOX21-AS1 was defined along other four lncRNAs as a signature that predicts survival.^70^ CRNDE is an important oncogenic lnRNA implicated in multiple malignancies including glioblastoma.^71–73^ Several relevant pathways, such as mTor, EGFR and TLR3-NF-κB-cytokine, are modulated by CRNDE.^74–76^ CRNDE also functions as a scaffold for chromatin-modifying complexes such as PRC2 and CoREST.^77^

Altogether, our study provides a novel RNA map to be explored regarding GBM progression, PN to MES transition, and treatment response and resistance.

## Methods

### Cell lines, cell culture and maintenance, RNA preparation and sequencing

We used six GSC lines previously described.^6^ Three were PN cell lines (PN-19, PN-157, and PN-528) and three were MES cell lines (MES-83, MES-326, MES-1123). PN and MES GSCs were maintained in DMEM/F12 supplemented with B27, heparin, bFGF, and EGF. Total RNA was: (i) extracted using TRIzol reagent (Life Technologies); (ii) purified with RNeasy (Qiagen), according to the manufacturer’s instructions; (iii) prepared for RNA-Seq according to the manufacturer’s instructions (Illumina); and (iv) sequenced using 101-bp paired-end chemistry on a HiSeq-2000 machine in the UTHSCSA Genomic Facility.

### Alternative splicing analysis

To identify splicing events differentially represented between PN and MES GSC cell lines, we first mapped raw RNA sequencing reads from all samples against the human reference genome (hg38/GRCh38) and the reference transcriptome (GENCODE version 26; www.gencodegenes.org; accessed on 30 Nov 2018) using GSNAP version 2016-09-23^78^ (parameters: -t 20; -B 4; -N 1; -E 1; -w 200000;–pairmax-rna 200000). Following this, reliable alignments against the genome (mapping quality score (Q) ≥ 20) were selected using SAMtools.^79^ To search for splicing differences between MES and PN cell lines, we used Replicate Multivariate Analysis of Transcript Splicing (rMATS),^80^ which reports splicing events already reported in the reference transcriptome, and those absent in the reference transcriptome (novel events). First, we compared the two GSC subgroups and selected all differentially represented splicing events (|ΔPSI| > 0.1 and FDR-adjusted *p*-value < 0.05, likelihood-ratio test). Novel and known splicing variants were then classified as follows: ES, MXE, retained intron (RI), alternative donor site (A5SS) or alternative acceptor site (A3SS). Outputs from rMATS were further processed using a set of locally created Python and R scripts. A hierarchical cluster was built based on inclusion levels of splicing events (PSI values) using *dist* and *hclust* R functions (https://www.r-project.org/; accessed 30 Jan 2019). We sought alternative isoforms for lncRNAs, selected according to GENCODE annotations.

To differentiate splicing events between PN and MES subtypes of GBM, we first downloaded aligned reads from 49 MES and 38 PN GBM samples from TCGA. Next, reads were aligned against the human reference genome (hg38/GRCh38 and the reference transcriptome (GENCODE version 22; https://www.gencodegenes.org/human/release_22.html; accessed 1 Oct 2019) using STAR.^81^ For GSCs, we selected reliable alignments against the genome (mapping quality score (Q) ≥ 20) using SAMtools, and searched for alternative splicing events using rMATs. Any batch-effect were detected or corrections were applied.

### Gene expression analyses

Differential expression analyses were performed using DESeq2.^82^ To compare MES-GSCs and PN-GSCs, only genomic mapped reads presenting mapping quality (Q) ≥ 20 (as described above) were considered. Read counts per gene were quantified using HTSeq-count,^83^ and GENCODE v26 was used as the reference for the human transcriptome. For comparisons between GBM and normal brain samples, read counts of 156 GBM samples were obtained from TCGA (GENCODE v22) and read counts of 287 samples from frontal cortex were obtained from the Genotype-Tissue Expression project (GENCODE v19). To compare MES and PN GBM samples, we used read counts from 50 and 45 samples from TCGA, respectively. For all comparisons, we selected as differentially expressed those genes presenting with a Benjamini-Hochberg (FDR) adjusted *p*-value < 0.05 and |log_2_FoldChange| ≥ 1 after the Wald test. Any batch effects were detected or corrections were applied.

### RNA-binding proteins (RBPs) and splicing regulator genes

We used a catalogue of 1542 human genes encoding RBPs^15^ to build a list of splicing regulators. We selected all genes associated with splice-related functional annotations in the catalog (388 genes) and identified the differentially spliced ones among them. Then we evaluated whether these RBPs represent known components of the spliceosomal machinery according to a comprehensive list of 404 splicing factor genes.^16^ We also sought alternative isoforms for lncRNAs, selected according to GENCODE annotations.

### Co-expression of RBP

We first analyzed co-expression patterns among differentially expressed RBPs in MES and PN GSCs using Spearman’s rank correlation. To identify groups of positively correlated RBPs (rho > 0.8), we performed hierarchical clustering of the correlation results. To confirm co-expression of the identified groups in a broader context, expression data (FPKM) from MES and PN GBM samples were downloaded from TCGA. Correlation analyses of selected groups were then performed separately for MES and PN GBM samples using Spearman’s rank correlation. Correlation plots were built for groups of RBPs exhibiting significant correlation patterns in GBM samples (Spearman's rank correlation *p*-values < 0.01). Correlation analyses were performed using R packages corrplot (github.com/taiyun/corrplot) and Hmisc (github.com/harrelfe/Hmisc).

### Survival analysis of protein-coding genes

Gene expression data (FPKM) and corresponding clinical data from GBM samples were downloaded from TCGA (https://portal.gdc.cancer.gov/). Expression data from TCGA were first filtered based on the list of protein-coding genes which were alternatively spliced and presented expression changes in PN versus MES GSCs. Based on the median value of gene expression levels, relevant survival differences between samples with high or low expression of each gene were determined using the log-rank test (*p*-value < 0.05). Associations of each gene with patient survival were separately assessed in MES and PN GBM samples, classified according to the GlioVis data portal.^84^ Genes exclusively associated with prognosis of MES or PN GBM (log-rank *p*-value < 0.05) were further included in a multivariate Cox proportional hazards model with the following covariates: age at diagnosis, gender, CIMP status, IDH1 mutation, MGMT methylation, chromosome 19/20 co-gain, and chromosome 7 gain/chromosome 10 loss. After adjusting for effects of these clinical variables, Kaplan-Meier survival curves were then built for genes that remained significantly associated with patient survival (multivariate Cox proportional-hazards regression *p*-value < 0.05).

### Functional annotation and interaction networks

GO categories and KEGG pathways were considered in the functional annotation, using the human genome as background in the DAVID web tool. Clusters of biological categories with FDR corrected *p*-values < 0.05 (Fisher's exact test) were considered enriched. Enriched clusters of GO categories were further processed to remove redundancy based on semantic similarities using the REVIGO web tool.^85^ Interaction analyses were performed based on protein-protein interaction data from STRING. Interaction networks of proteins exhibiting at least two interactions were then built using Cytoscape.^86^

### Survival analysis of lncRNAs

Data for expression of lncRNAs and corresponding clinical data from all tumor types in the study were downloaded from TCGA (https://portal.gdc.cancer.gov/). Expression data from TCGA were first filtered based on the list of differentially expressed lncRNAs previously obtained from our data (candidate lncRNAs). Expression data for each candidate lncRNA were then individually submitted to a separate survival analysis performed using univariate Cox proportional hazards models,^87^ with expression levels of lncRNAs as continuous variables. To adjust for other clinical variables, each lncRNA exhibiting significant correlation with patient survival in the univariate analysis (Cox proportional-hazards regression *p*-value < 0.05) was then included in a multivariate Cox proportional hazards model with the following covariates: age at diagnosis, gender, CIMP status, IDH1 mutation, MGMT methylation, chromosome 19/20 co-gain, and chromosome 7 gain/chromosome 10 loss. Kaplan-Meier survival curves were then built for lncRNAs that remained significantly associated with patient survival in the multivariate Cox proportional-hazards regression models (*p*-value < 0.05). Relevant survival differences between samples exhibiting high or low expression of each lncRNA (samples split based on the median value of lncRNA expression levels) were determined using the log-rank test (*p*-value < 0.05). We first considered all GBM samples in the survival analyses. Next, analyses were performed separately considering MES and PN subgroups. GBM samples were classified into these subgroups based on data from the GlioVis data portal.^48^

### Validation of splicing events by qRT-PCR

First, we selected a total of seven exon-skipping events to be tested by qRT-PCR. These candidates were randomly selected (using a random number generator) from a sorted list containing the top 500 exon-skipping events with the highest ΔPSI between GSC MES vs. PN. Next, total RNA was extracted using TRIzol reagent (Invitrogen) according to the manufacturer’s instructions. Reverse transcription of messenger RNAs was performed using a high-capacity cDNA reverse transcription kit (Applied Biosystems) with random priming. For mRNA analysis, quantitative PCR was performed using the primers listed in Supplementary Table 26 and Power SYBR green PCR master mix (Applied Biosystems). Real-time PCR was performed on a ViiA™ STRING7 Real-Time PCR System (Applied Biosystems). Data were acquired using the ViiA 7 RUO software package (Applied Biosystems) and analyzed using an adapted 2−ΔΔCT method with B2M as an endogenous control.

### Statistical analysis and figures

Statistical analyses were performed using R. Figures were built using R, Cytoscape,^86^ Circos Plot,^88^ Inkscape (https://inkscape.org/) and rmats2sashimiplot (https://github.com/Xinglab/rmats2sashimiplot/).

### Reporting summary

Further information on research design is available in the Nature Research Reporting Summary linked to this article.

## Supplementary information

Supplemental Material

Reporting Summary

## Data Availability

RNA sequencing data have been deposited in the European Nucleotide Archive [**ENA: PRJEB27943**].
