# Supplementary material for: Proneural and mesenchymal glioma stem cells display major differences in splicing and lncRNA profiles
Source: NPJ Genom Med. 2020 Jan 16;5:2. doi: 10.1038/s41525-019-0108-5 (PMC6965107; doi:10.1038/s41525-019-0108-5)
Supplement: Supplementary file 1 — Supplemental Material [file 41525_2019_108_MOESM1_ESM.pdf]

SUPPLEMENTARY INFORMATION

Proneural and Mesenchymal glioma stem cells display major differences in splicing and lncRNA profiles

Gabriela D.A. Guardia[1], Bruna R. Correa[1,2], Patricia Rosa Araujo[3], Mei Qiao[3], Suzanne Burns[3], Luiz O. F. Penalva[3,4*], Pedro A.F. Galante[1*]

1. Centro de Oncologia Molecular, Hospital Sírio-Libanês, São Paulo, São Paulo, 01309-060, Brazil
2. Current affiliation: Centre for Genomic Regulation (CRG), The Barcelona Institute of Science and Technology, Barcelona 08003, Catalonia, Spain
3. Children's Cancer Research Institute, UT Health San Antonio, San Antonio, Texas, 78229, USA
4. Department of Cell Systems and Anatomy, UT Health San Antonio, San Antonio, Texas, 78229, USA

*Corresponding authors: penalva@uthscsa.edu (L.O.F. P.), pgalante@mochsl.org.br (P.A.F.G.)

Supplementary Figures

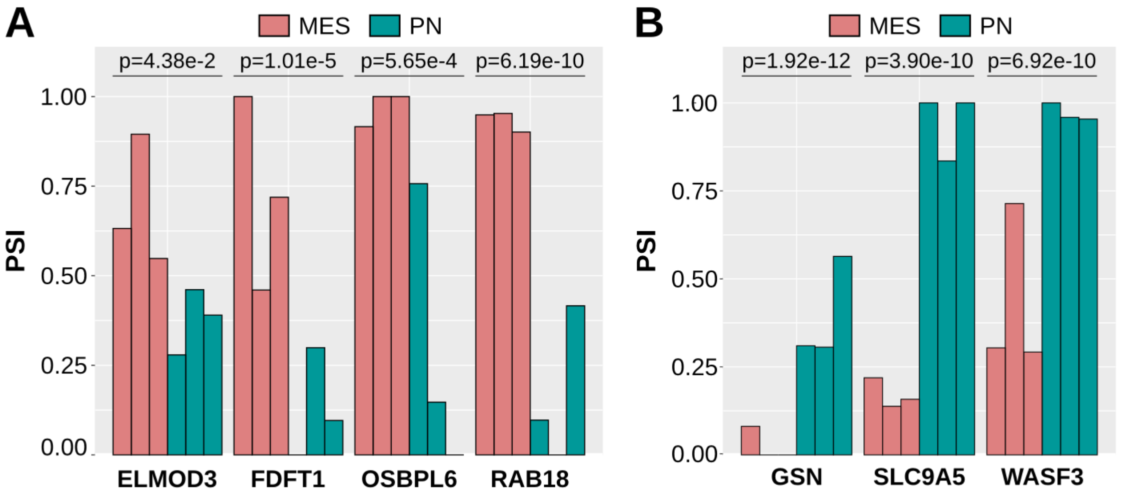

Supplementary Figure 1. RNA-Seq inclusion levels (PSI values) of **(A)** proneural GSCs and **(B)** mesenchymal GSCs exon skipping events further validated by qRT-PCR (Mann-Whitney Wilcoxon p-values).

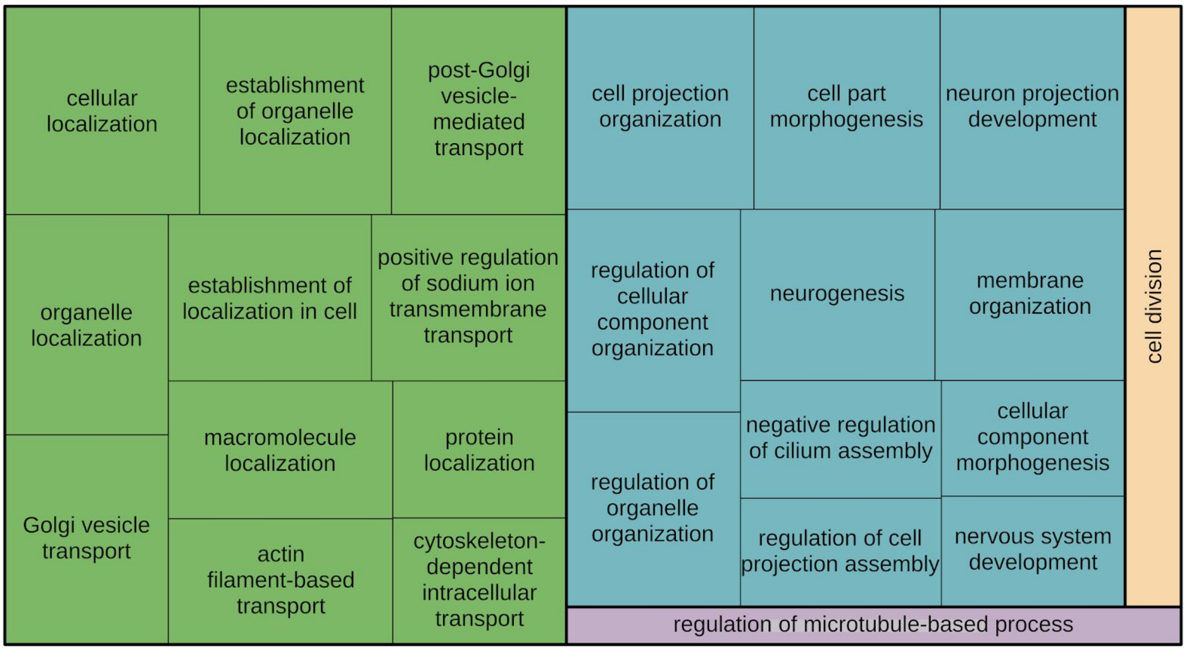

Supplementary Figure 2. Treemap of Gene ontology enriched processes for protein coding genes sharing AS events in proneural GSC and GBM versus mesenchymal GSC and GBM, respectively.

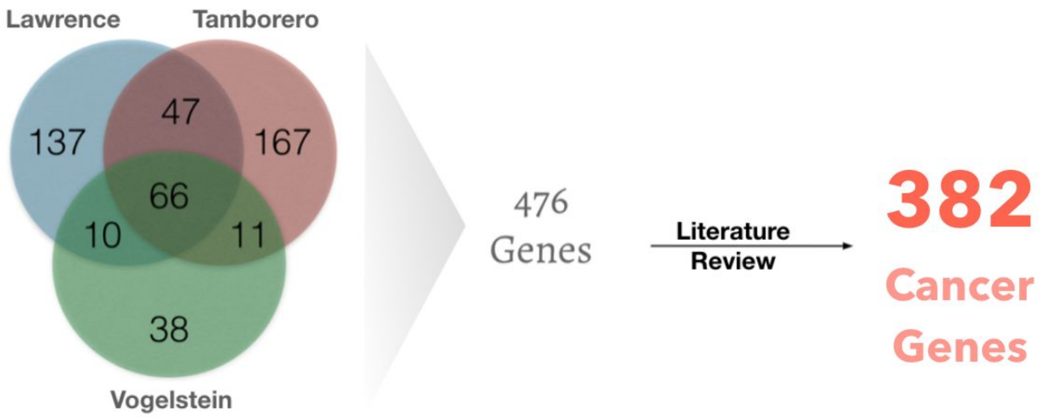

Supplementary Figure 3. Cancer genes. The set of cancer genes was defined using genes related to cancer presented in [1], [2] and [3]. We conducted a literature review for all genes and kept only those genes already described as cancer-related in at least one publication).

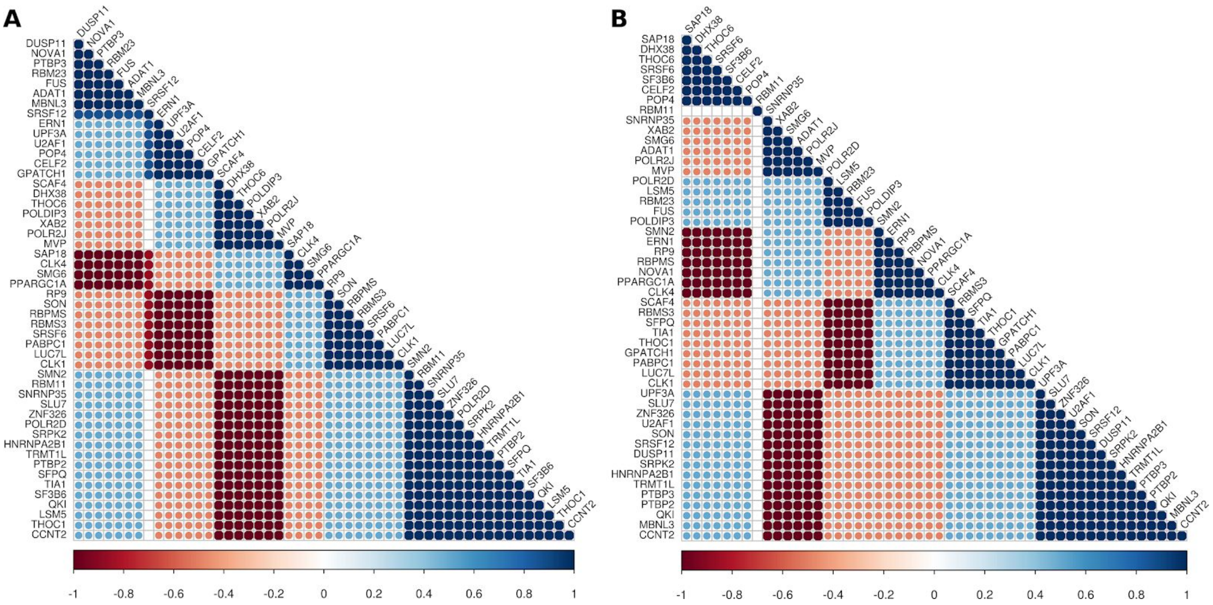

Supplementary Figure 4: Correlations among differentially expressed RBPs in A) mesenchymal GSCs; and B) proneural GSCs.

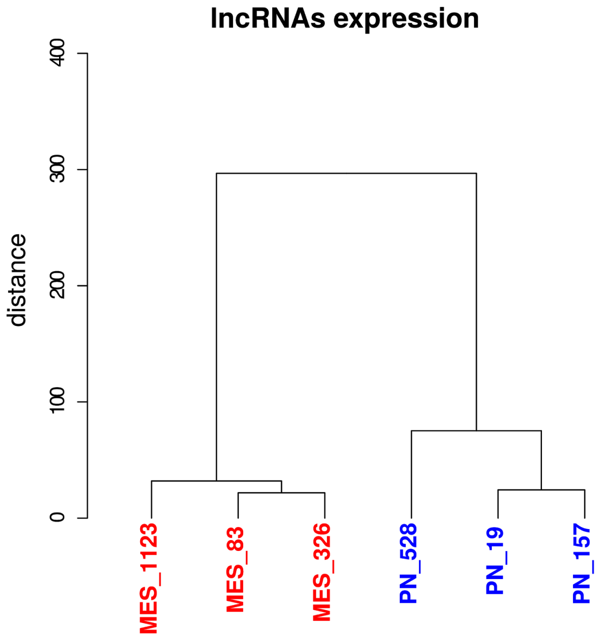

Supplementary Figure 5: Unsupervised hierarchical clustering of GSC samples based on expression levels of differentially expressed long non-coding RNAs (lncRNAs).

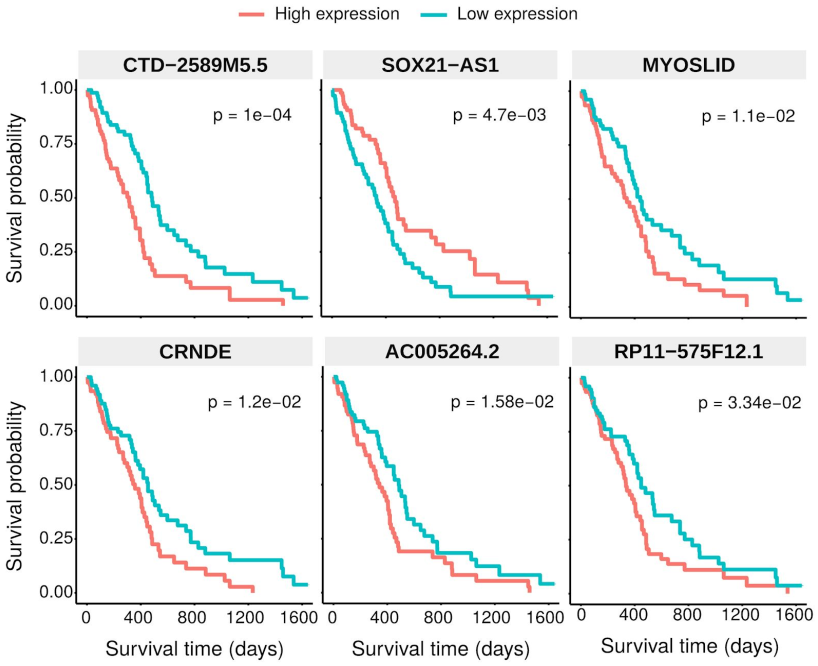

Supplementary Figure 6: Kaplan-Meier survival plots of differentially expressed lncRNAs significantly correlated with prognosis in GBM (p<0.05, univariate Cox proportional-hazards regression).

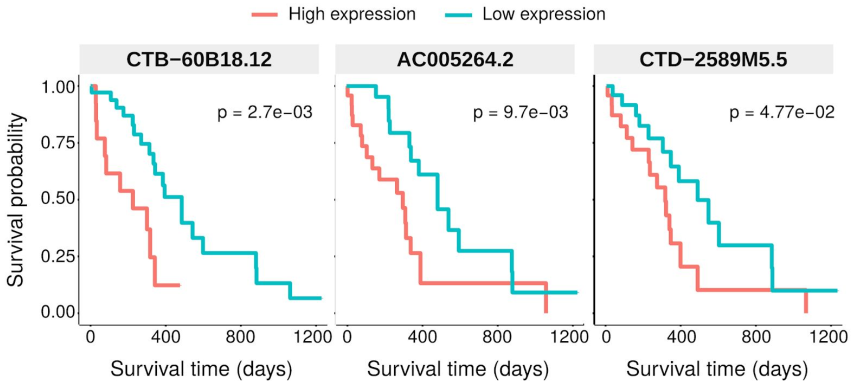

Supplementary Figure 7: Kaplan-Meier survival plots of differentially expressed lncRNAs significantly associated with prognosis in mesenchymal GBM (p<0.05, univariate Cox proportional-hazards regression).

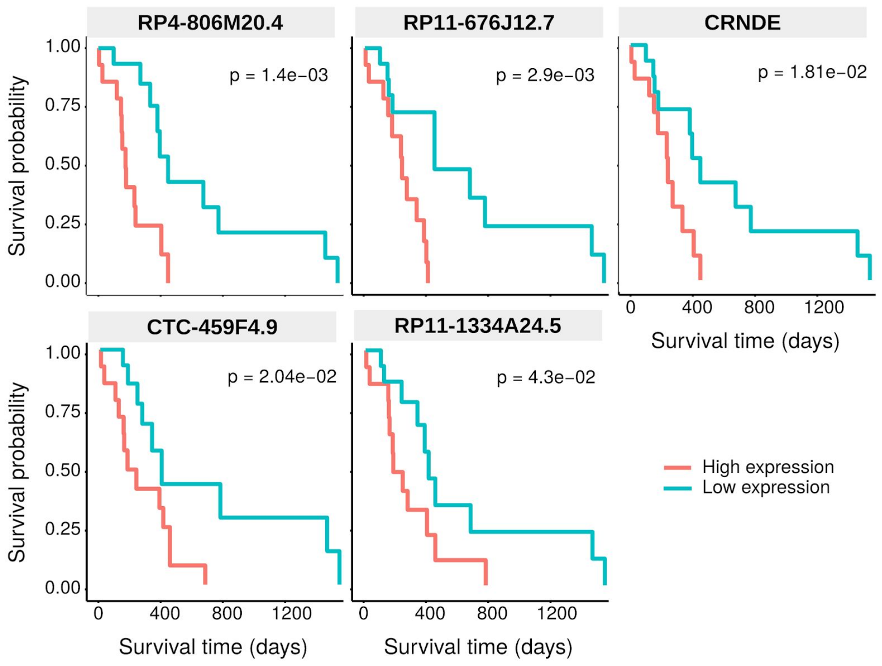

Supplementary Figure 8: Kaplan-Meier survival plots of differentially expressed lncRNAs significantly associated with prognosis in proneural GBM (p<0.05, univariate Cox proportional-hazards regression).

Supplementary Tables

Supplementary Table 1: Sequenced and reliable mapped reads per GSC sample.

sample	sequenced.reads	mapped.reads (>q20)	%mapped.reads (>q20)
**MES-83**	58,688,002	53,958,742	91.94
**MES-326**	61,481,484	56,192,789	91.40
**MES-1123**	61,517,920	52,705,388	85.67
**PRO-19**	69,594,474	66,841,359	96.04
**PRO-157**	62,056,008	59,633,152	96.10
**PRO-528**	58,363,862	55,228,567	94.63

Supplementary Table 2: Splicing events differing between mesenchymal (MES) and proneural (PN) GSCs.

Differentially [illegible]	MES		PN	
**Event type**	**# events**	**# spliced genes**	**# events**	**# spliced genes**
ASS	284	255	258	236
MXE	449	324	342	262
RI	202	172	250	234
ES	1,610	1,158	1,539	1,103
**Total**	2,545	1,640**	2,389	1,613**

*|PSI| > 0.1 and FDR < 0.05; ASS: alternative 5' or 3' splice site; MXE, mutually exclusive exon; RI: intron retention; ES: exon skipping

**Unique genes in all events of each condition

ASS (alternative splice site 5' or 3'): genomic coordinates of long and short exons (columns g.coord_1 and g.coord_2)

MXE (mutually exclusive exon):genomic coordinates of first and second exons (columns g.coord_1 and g.coord_2)

RI (retained intron): genomic coordinates of retained intron (column g.coord_1)

ES (exon skipping): genomic coordinates of skipped exon (column g.coord_1)

MES

event.type	gene	strand	g.coord_1	g.coord_2	inc.level.difference	FDR
ASS	RPL22L1	-	chr3:170868012-170868201	chr3:170868012-170868134	0.606	0
ASS	PFN2	-	chr3:149964903-149966586	chr3:149964903-149966264	0.392	0
ASS	GAS5	-	chr1:173865470-173865547	chr1:173865509-173865547	0.477	0
ASS	SMC4	+	chr3:160412325-160412849	chr3:160412325-160412453	0.115	0
ASS	DCUN1D4	+	chr4:51886567-51887244	chr4:51886567-51886638	0.307	0
ASS	ULK3	-	chr15:74837750-74837798	chr15:74837756-74837798	0.665	0
ASS	BTAF1	+	chr10:91959064-91959282	chr10:91959064-91959154	0.258	0
ASS	PARP6	-	chr15:72265269-72265473	chr15:72265412-72265473	0.279	0
ASS	KIAA1109	+	chr4:122334343-122334545	chr4:122334343-122334440	0.543	0
ASS	RPL17	-	chr18:49491735-49491833	chr18:49491745-49491833	0.271	0
ASS	THOC2	-	chrX:123620374-123620965	chrX:123620906-123620965	0.231	0
ASS	FAXDC2	-	chr5:154823307-154823592	chr5:154823386-154823592	0.969	1.44E-12
ASS	ECI2	-	chr6:4117307-4119275	chr6:4117307-4117451	0.327	2.46E-12
ASS	ANAPC5	-	chr12:121328315-121328497	chr12:121328315-121328458	0.198	5.53E-11
ASS	CDK11A	-	chr1:1707408-1707584	chr1:1707524-1707584	0.291	1.41E-10
ASS	INO80C	-	chr18:35487385-35487823	chr18:35487385-35487439	0.679	3.18E-10
ASS	YY1AP1	-	chr1:155668626-155668777	chr1:155668626-155668717	0.304	8.73E-10
ASS	FUZ	-	chr19:49812446-49812736	chr19:49812614-49812736	0.806	1.10E-09
ASS	QKI	+	chr6:163564666-163570145	chr6:163565945-163570145	0.545	3.14E-09
ASS	PSMG4	+	chr6:3264173-3264325	chr6:3264208-3264325	0.676	6.14E-09
ASS	CTNND1	+	chr11:57761796-57762119	chr11:57761796-57762046	0.285	6.49E-09
ASS	SNRPN	+	chr15:24974287-24974456	chr15:24974310-24974456	0.392	2.09E-08
ASS	SLC37A3	-	chr7:140351272-140351969	chr7:140351272-140351451	0.273	2.09E-08
ASS	MOV10	+	chr1:112699457-112699810	chr1:112699684-112699810	0.302	2.09E-08
ASS	RNF216	-	chr7:5739275-5739621	chr7:5739275-5739352	0.164	2.09E-08
ASS	NSFL1C	-	chr20:1455713-1455870	chr20:1455713-1455719	0.25	2.59E-08
ASS	DNAJC17	-	chr15:40776195-40776401	chr15:40776195-40776292	0.106	4.68E-08
ASS	EXOC7	-	chr17:76090328-76090481	chr17:76090328-76090397	0.63	5.30E-08
ASS	CNBP	-	chr3:129171633-129171771	chr3:129171654-129171771	0.231	5.77E-08
ASS	SFSWAP	+	chr12:131714070-131714240	chr12:131714070-131714193	0.163	1.54E-07
ASS	TIA1	-	chr2:70216752-70216994	chr2:70216885-70216994	0.191	1.98E-07
ASS	SASH1	+	chr6:148519546-148520382	chr6:148519546-148519893	0.102	2.40E-07
ASS	REPS1	-	chr6:138926400-138930098	chr6:138929976-138930098	0.146	2.51E-07
ASS	SNHG5	-	chr6:85677794-85678032	chr6:85677953-85678032	0.139	3.85E-07
ASS	GEMIN8	-	chrX:14026051-14026221	chrX:14026139-14026221	0.327	3.93E-07
ASS	ABHD3	-	chr18:21663677-21664230	chr18:21664117-21664230	0.388	4.71E-07
ASS	ZNF30	+	chr19:34926902-34927282	chr19:34926902-34927216	0.619	6.09E-07
ASS	ECI2	-	chr6:4133548-4133711	chr6:4133548-4133701	0.251	9.29E-07
ASS	PDLIM5	+	chr4:94575615-94576034	chr4:94575942-94576034	0.428	1.38E-06
ASS	ENGASE	+	chr17:79081897-79082877	chr17:79081897-79082063	0.365	1.43E-06
ASS	FUZ	-	chr19:49812446-49812736	chr19:49812637-49812736	0.508	1.43E-06
ASS	CDK5RAP3	+	chr17:47973518-47973693	chr17:47973518-47973650	0.131	1.44E-06
ASS	APRT	-	chr16:88809468-88809840	chr16:88809468-88809706	0.167	1.57E-06
ASS	PGGHG	+	chr11:290657-291113	chr11:290677-291113	0.517	2.86E-06
ASS	ITGA7	-	chr12:55700565-55701154	chr12:55700898-55701154	0.513	3.83E-06
ASS	GPN3	-	chr12:110468125-110468264	chr12:110468195-110468264	0.556	3.93E-06
ASS	TMEM198B	+	chr12:55832870-55833014	chr12:55832958-55833014	0.387	4.38E-06
ASS	MED24	-	chr17:40020272-40020482	chr17:40020272-40020353	0.193	4.61E-06
ASS	TCF4	-	chr18:55232508-55232671	chr18:55232520-55232671	0.431	5.01E-06
ASS	SPIN3	-	chrX:56978579-56978762	chrX:56978579-56978660	0.322	5.15E-06
ASS	ZNF226	+	chr19:44165587-44165807	chr19:44165756-44165807	0.275	5.46E-06
ASS	CENPK	-	chr5:65561462-65561564	chr5:65561496-65561564	0.15	5.79E-06
ASS	CXorf23	-	chrX:19937417-19937532	chrX:19937504-19937532	0.403	6.40E-06
ASS	CCHCR1	-	chr6:31157357-31157500	chr6:31157384-31157500	0.498	6.71E-06
ASS	SLC35B1	-	chr17:49706203-49706334	chr17:49706203-49706309	0.202	7.79E-06
ASS	COX11	-	chr17:54961118-54962915	chr17:54961118-54961312	0.114	7.79E-06
ASS	CERS5	-	chr12:50132961-50134068	chr12:50132961-50133064	0.323	1.11E-05
ASS	SERHL2	+	chr22:42571120-42571901	chr22:42571120-42571203	0.324	1.14E-05
ASS	SPIN3	-	chrX:56978579-56978747	chrX:56978579-56978660	0.322	1.29E-05
ASS	SPIN3	-	chrX:56978579-56978751	chrX:56978579-56978660	0.318	1.35E-05
ASS	BTN3A3	+	chr6:26443366-26443442	chr6:26443381-26443442	0.734	1.43E-05
ASS	SMARCC2	-	chr12:56164302-56164731	chr12:56164647-56164731	0.43	1.46E-05
ASS	ATP5O	-	chr21:33915135-33915724	chr21:33915651-33915724	0.422	1.59E-05
ASS	TPT1-AS1	+	chr13:45390713-45390902	chr13:45390757-45390902	0.12	1.61E-05
ASS	ATF6B	-	chr6:32117036-32117130	chr6:32117036-32117107	0.134	1.90E-05
ASS	QKI	+	chr6:163563419-163563719	chr6:163563443-163563719	0.127	1.90E-05
ASS	HNRNPH1	-	chr5:179623594-179623671	chr5:179623635-179623671	0.141	2.06E-05
ASS	METTL22	+	chr16:8644528-8644725	chr16:8644556-8644725	0.283	2.96E-05
ASS	NPHP3	-	chr3:132692653-132693865	chr3:132692653-132692818	0.122	3.53E-05
ASS	TMEM62	+	chr15:43149028-43149151	chr15:43149107-43149151	0.147	3.84E-05
ASS	SYNRG	-	chr17:37596222-37596344	chr17:37596222-37596341	0.311	4.07E-05
ASS	PAAF1	+	chr11:73886928-73887099	chr11:73887030-73887099	0.348	4.07E-05
ASS	CDKN2AIP	+	chr4:183445534-183445665	chr4:183445534-183445634	0.247	4.13E-05
ASS	ZNF740	+	chr12:53181676-53182190	chr12:53181676-53181992	0.305	4.35E-05
ASS	CLK4	-	chr5:178619837-178620673	chr5:178619837-178619896	0.302	4.40E-05
ASS	RASSF4	+	chr10:44983940-44984113	chr10:44984021-44984113	0.491	4.71E-05
ASS	UNKL	-	chr16:1369933-1370357	chr16:1370129-1370357	0.212	4.73E-05
ASS	TAX1BP1	+	chr7:27816348-27816520	chr7:27816348-27816394	0.103	5.39E-05
ASS	C11orf74	+	chr11:36594512-36594641	chr11:36594512-36594592	0.226	6.34E-05
ASS	MIGA2	+	chr9:129068018-129068332	chr9:129068197-129068332	0.231	7.43E-05
ASS	GIT2	-	chr12:109947255-109948846	chr12:109947255-109947504	0.382	7.50E-05
ASS	INPP4A	+	chr2:98555552-98555808	chr2:98555552-98555691	0.57	8.36E-05
ASS	ANKHD1-EIF4E	+	chr5:140537337-140537589	chr5:140537388-140537589	0.176	0.0001132589991
ASS	NUSAP1	+	chr15:41350987-41351129	chr15:41351032-41351129	0.123	0.000118969917
ASS	CASC3	+	chr17:40167370-40167612	chr17:40167497-40167612	0.116	0.0001438215556
ASS	GRHPR	+	chr9:37422713-37422833	chr9:37422713-37422805	0.238	0.000174827936
ASS	GSKIP	+	chr14:96379687-96379788	chr14:96379687-96379755	0.343	0.0001836014068
ASS	HSCB	+	chr22:28743881-28743978	chr22:28743881-28743923	0.124	0.0001921815736
ASS	TPT1-AS1	+	chr13:45377060-45377215	chr13:45377139-45377215	0.22	0.00019763891
ASS	ZCWPW1	-	chr7:100419001-100419189	chr7:100419110-100419189	0.476	0.0002101279767
ASS	MTMR2	-	chr11:95887611-95887759	chr11:95887611-95887684	0.249	0.0002271937679
ASS	ZC3HAV1	-	chr7:139079103-139080243	chr7:139079469-139080243	0.187	0.0002460105186
ASS	PLPP5	-	chr8:38268822-38268990	chr8:38268881-38268990	0.244	0.0002493600206
ASS	EFHC1	+	chr6:52454061-52454287	chr6:52454094-52454287	0.24	0.0002511876746
ASS	BCL2L11	+	chr2:111123745-111124139	chr2:111123745-111123869	0.154	0.0002762183682
ASS	CHD3	+	chr17:7909142-7909384	chr17:7909142-7909338	0.295	0.0002879218488
ASS	PHF20L1	+	chr8:132799082-132799172	chr8:132799094-132799172	0.563	0.0003262237303
ASS	DNAJC10	+	chr2:182739544-182740388	chr2:182740298-182740388	0.104	0.0003718189421
ASS	UNC50	+	chr2:98618145-98618515	chr2:98618167-98618515	0.127	0.0003777090284
ASS	GAA	+	chr17:80101810-80101925	chr17:80101810-80101890	0.217	0.0003941467045
ASS	BMP1	+	chr8:22201117-22201928	chr8:22201802-22201928	0.321	0.0003985755184

PN

event.type	gene	strand	g.coord_1	g.coord_2	inc.level.difference	FDR
ASS	RSRP1	-	chr1:25243549-25244224	chr1:25243549-25243633	-0.146	0
ASS	FAM122B	-	chrX:134769566-134772283	chrX:134769566-134771354	-0.244	0
ASS	QKI	+	chr6:163565945-163566795	chr6:163566720-163566795	-0.573	0
ASS	WSB1	+	chr17:27307743-27309272	chr17:27309099-27309272	-0.171	0
ASS	HLA-A	+	chr6:29944499-29944634	chr6:29944499-29944616	-0.528	0
ASS	KIF21A	-	chr12:39321347-39322882	chr12:39322667-39322882	-0.441	0
ASS	RSRP1	-	chr1:25244176-25245301	chr1:25245149-25245301	-0.218	0
ASS	NOP56	+	chr20:2655933-2656214	chr20:2655933-2656034	-0.26	0
ASS	BDH2	-	chr4:103085698-103086540	chr4:103086479-103086540	-0.241	0
ASS	MTX1	+	chr1:155210547-155212219	chr1:155210547-155210627	-0.6	0
ASS	PILRB	+	chr7:100353372-100354012	chr7:100353372-100353483	-0.397	0
ASS	LIAS	+	chr4:39467517-39468427	chr4:39467517-39467646	-0.23	0
ASS	SELENOF	-	chr1:86914027-86914424	chr1:86914314-86914424	-0.231	0
ASS	NDUFS5	+	chr1:39026317-39026402	chr1:39026317-39026398	-0.427	0
ASS	AP4M1	+	chr7:100101879-100102393	chr7:100101879-100101968	-0.269	0
ASS	LAS1L	-	chrX:65520701-65523707	chrX:65523559-65523707	-0.247	0
ASS	DAP3	+	chr1:155689090-155689243	chr1:155689090-155689174	-0.185	0
ASS	OSBPL3	-	chr7:24834086-24834736	chr7:24834485-24834736	-0.543	5.35E-13
ASS	ARL6IP4	+	chr12:122981570-122981879	chr12:122981594-122981879	-0.303	5.86E-13
ASS	DZANK1	-	chr20:18412645-18412853	chr20:18412645-18412835	-0.791	1.24E-12
ASS	CENPV	-	chr17:16348615-16349333	chr17:16348615-16348685	-0.676	2.77E-12
ASS	COMMD4	+	chr15:75338061-75338420	chr15:75338061-75338133	-0.366	4.88E-11
ASS	PNISR	-	chr6:99403828-99404702	chr6:99404602-99404702	-0.289	8.34E-11
ASS	TAF1D	-	chr11:93736693-93737239	chr11:93737063-93737239	-0.127	1.32E-10
ASS	NABP1	+	chr2:191683289-191683804	chr2:191683728-191683804	-0.552	2.14E-10
ASS	ARL6IP4	+	chr12:122981570-122981879	chr12:122981594-122981879	-0.254	3.67E-10
ASS	MGRN1	+	chr16:4686258-4690974	chr16:4688795-4690974	-0.593	3.75E-10
ASS	CDK13	+	chr7:40092784-40093237	chr7:40092964-40093237	-0.292	4.03E-10
ASS	C5orf42	-	chr5:37139339-37142480	chr5:37142309-37142480	-0.611	9.22E-10
ASS	PAM16	-	chr16:4341367-4341672	chr16:4341367-4341504	-0.529	6.69E-09
ASS	RAP1GAP	-	chr1:21602803-21603912	chr1:21602803-21602913	-0.117	7.66E-09
ASS	PRR13	+	chr12:53443390-53443773	chr12:53443486-53443773	-0.223	8.78E-09
ASS	C18orf54	+	chr18:54361642-54362431	chr18:54361642-54361948	-0.45	1.03E-08
ASS	SH3TC2	-	chr5:149041415-149041617	chr5:149041473-149041617	-0.733	1.14E-08
ASS	PARP2	+	chr14:20344931-20345126	chr14:20344931-20345087	-0.299	1.33E-08
ASS	TAZ	+	chrX:154419542-154419746	chrX:154419542-154419623	-0.441	3.46E-08
ASS	ZNF711	+	chrX:85246929-85247651	chrX:85247546-85247651	-0.271	7.29E-08
ASS	TKT	-	chr3:53237796-53237921	chr3:53237796-53237820	-0.501	1.07E-07
ASS	PCID2	-	chr13:113177610-113178008	chr13:113177610-113177976	-0.315	1.16E-07
ASS	C19orf60	+	chr19:18588994-18589683	chr19:18588994-18589077	-0.412	1.58E-07
ASS	MAP4K4	+	chr2:101887087-101887261	chr2:101887087-101887237	-0.102	2.13E-07
ASS	PKMYT1	-	chr16:2973137-2973445	chr16:2973137-2973215	-0.386	4.25E-07
ASS	RP11-49K24.6	-	chr18:47136301-47136449	chr18:47136338-47136449	-0.245	4.89E-07
ASS	C7orf50	-	chr7:1138188-1138257	chr7:1138217-1138257	-0.289	1.25E-06
ASS	PQBP1	+	chrX:48897911-48898093	chrX:48897911-48898082	-0.587	1.43E-06
ASS	NOL8	-	chr9:92324905-92325350	chr9:92325305-92325350	-0.176	1.63E-06
ASS	SUPT20H	-	chr13:37022010-37023044	chr13:37022010-37022317	-0.36	2.00E-06
ASS	MFF	+	chr2:227328677-227328899	chr2:227328677-227328789	-0.434	2.24E-06
ASS	FAM208A	-	chr3:56633043-56633966	chr3:56633043-56633843	-0.155	2.29E-06
ASS	DDX51	-	chr12:132141102-132141420	chr12:132141274-132141420	-0.411	2.38E-06
ASS	INPP5B	-	chr1:37931672-37932053	chr1:37931912-37932053	-0.173	3.09E-06
ASS	AP4M1	+	chr7:100101879-100102393	chr7:100101879-100101989	-0.585	3.09E-06
ASS	FAM208A	-	chr3:56633043-56633966	chr3:56633043-56633843	-0.224	4.33E-06
ASS	PHF20L1	+	chr8:132804611-132804740	chr8:132804614-132804740	-0.166	4.50E-06
ASS	GUCD1	-	chr22:24540437-24543100	chr22:24540437-24543097	-0.117	5.52E-06
ASS	DOCK1	+	chr10:127012231-127012374	chr10:127012294-127012374	-0.194	6.31E-06
ASS	TUBGCP4	+	chr15:43403682-43403842	chr15:43403682-43403799	-0.187	7.91E-06
ASS	APTX	-	chr9:32987543-32987846	chr9:32987543-32987731	-0.325	9.48E-06
ASS	B4GALT3	-	chr1:161175807-161176095	chr1:161175807-161176074	-0.292	1.36E-05
ASS	LRRC75A-AS1	+	chr17:16463163-16463317	chr17:16463166-16463317	-0.451	1.54E-05
ASS	SIPA1L1	+	chr14:71704720-71705340	chr14:71705221-71705340	-0.198	1.76E-05
ASS	NPAS2	+	chr2:100995399-100996055	chr2:100995795-100996055	-0.184	2.46E-05
ASS	ANXA4	+	chr2:69806384-69806498	chr2:69806384-69806432	-0.17	2.79E-05
ASS	NDUFAF6	+	chr8:95031994-95032098	chr8:95031994-95032094	-0.327	2.95E-05
ASS	CORO7-PAM16	-	chr16:4341367-4341583	chr16:4341367-4341504	-0.44	3.17E-05
ASS	PAM16	-	chr16:4341367-4341672	chr16:4341367-4341583	-0.336	3.17E-05
ASS	CLCN2	-	chr3:184354547-184354714	chr3:184354547-184354658	-0.555	3.27E-05
ASS	KMT2A	+	chr11:118471661-118474315	chr11:118474178-118474315	-0.201	3.34E-05
ASS	PRR13	+	chr12:53443390-53443773	chr12:53443540-53443773	-0.219	3.86E-05
ASS	HMGN3	-	chr6:79202275-79202389	chr6:79202316-79202389	-0.153	4.51E-05
ASS	CLEC16A	+	chr16:11003073-11003305	chr16:11003073-11003257	-0.473	4.65E-05
ASS	MSH5-SAPCD1	+	chr6:31761817-31761955	chr6:31761817-31761865	-0.151	4.80E-05
ASS	NEK3	-	chr13:52143914-52143987	chr13:52143915-52143987	-0.653	5.08E-05
ASS	CPM	-	chr12:68933141-68933202	chr12:68933159-68933202	-0.432	5.28E-05
ASS	NOL8	-	chr9:92324905-92325350	chr9:92325317-92325350	-0.431	6.68E-05
ASS	NRM	-	chr6:30690024-30690243	chr6:30690046-30690243	-0.66	7.95E-05
ASS	CLPTM1L	-	chr5:1331798-1331883	chr5:1331802-1331883	-0.269	8.36E-05
ASS	SLC25A19	-	chr17:75288387-75288591	chr17:75288497-75288591	-0.227	8.47E-05
ASS	NEO1	+	chr15:73270009-73270233	chr15:73270057-73270233	-0.215	0.0001021511285
ASS	PRPF38A	+	chr1:52405679-52405839	chr1:52405679-52405713	-0.175	0.0001084941665
ASS	PHB2	-	chr12:6969955-6970068	chr12:6969955-6970063	-0.437	0.0001139561149
ASS	RNF126	-	chr19:651610-651855	chr19:651691-651855	-0.369	0.000119055682
ASS	FNBP1	-	chr9:129923843-129924026	chr9:129923843-129923996	-0.447	0.0001222988585
ASS	CACNB4	-	chr2:151869176-151870611	chr2:151870530-151870611	-0.46	0.0001250994644
ASS	DDX39B	-	chr6:31540321-31540664	chr6:31540321-31540534	-0.123	0.000140885502
ASS	CPEB2	+	chr4:15007304-15007595	chr4:15007304-15007586	-0.18	0.0001514101156
ASS	CXorf38	-	chrX:40639008-40640320	chrX:40639008-40639128	-0.188	0.000157290213
ASS	ASIC1	+	chr12:50076967-50077060	chr12:50076967-50077049	-0.594	0.0001836014068
ASS	MYO19	-	chr17:36508896-36509135	chr17:36509061-36509135	-0.194	0.000193223543
ASS	FANCL	-	chr2:58198578-58198662	chr2:58198593-58198662	-0.201	0.0001938388517
ASS	FNBP1	-	chr9:129923843-129924026	chr9:129923843-129923939	-0.261	0.0001981096245
ASS	CENPT	-	chr16:67832227-67832545	chr16:67832454-67832545	-0.133	0.0002116162297
ASS	CACNB4	-	chr2:151869176-151870611	chr2:151870530-151870611	-0.378	0.000234553557
ASS	RAD51	+	chr15:40695173-40695425	chr15:40695173-40695321	-0.159	0.0002455544809
ASS	NAA60	+	chr16:3484698-3485083	chr16:3484698-3485061	-0.189	0.0002455544809
ASS	NEDD1	+	chr12:96907603-96907856	chr12:96907603-96907624	-0.158	0.0002711507675
ASS	DPAGT1	-	chr11:119100629-119100877	chr11:119100629-119100827	-0.241	0.0003131154122
ASS	PLK3	+	chr1:44804338-44804779	chr1:44804338-44804501	-0.426	0.0003250196594
ASS	CARD19	+	chr9:93107673-93107827	chr9:93107673-93107816	-0.246	0.0003422060407
ASS	TRIM37	-	chr17:59001597-59001741	chr17:59001597-59001714	-0.114	0.0003718189421
ASS	ATG16L2	+	chr11:72825301-72825407	chr11:72825348-72825407	-0.137	0.0003901111932

All genes with differential splicing
A1BG-AS1
AACS
AAK1
AASDH
ABCA11P
ABCB6
ABCD4
ABHD11
ABHD14A
ABHD14A-ACY1
ABHD14B
ABHD3
ABI1
ABL2
ABTB1
AC004076.9
AC004381.6
AC005154.6
AC007040.11
AC007566.10
AC024560.3
AC074286.1
AC091729.9
AC108488.4
AC138969.4
ACAA1
ACACA
ACAD10
ACAD11
ACADVL
ACAP2
ACAT1
ACER3
ACIN1
ACLY
ACO1
ACOT8
ACOX1
ACP1
ACSF3
ACSL3
ACSS1
ACTN1
ACTN4
ACTR10
ACTR3B
ACYP2
AD000671.6
ADAM22
ADAMTS13
ADAMTS6
ADAP2
ADARB1
ADAT1
ADCY10P1
ADCY6
ADD1
ADD3
ADGRB2
ADGRG1
ADGRG6
ADGRL1
ADGRL2
ADGRL3
ADRM1
AFDN
AFMID
AFTPH
AGAP1
AGAP3
AGAP4
AGO3
AGPAT4
AGRN
AGTPBP1
AGTRAP
AHI1
AHSA2
AIFM1
AK2
AKAP11
AKIP1
AKNA
ALDH3A2
ALDH3B1
ALDOA
ALG13
ALG2
ALG9
ALKBH3
ALKBH8
ALMS1
ALOX12-AS1
ALPK1
AMACR
AMBRA1
AMMECR1L
AMPD2
ANAPC10
ANAPC11
ANAPC16
ANAPC5

ASS	ARL16	-	chr17:81682442-81682675	chr17:81682490-81682675	0.291	0.0003991287134	ASS	ZRANB2-AS2	+	chr1:71237129-71237386	chr1:71237129-71237195	-0.531	0.0004010953459	ANGEL1
ASS	TMEM198B	+	chr12:55832958-55833038	chr12:55832958-55833014	0.385	0.0003991287134	ASS	SGO1	-	chr3:20174248-20175055	chr3:20175004-20175055	-0.175	0.000404362015	ANK3
ASS	MPRIP	+	chr17:17137915-17138429	chr17:17137915-17138075	0.242	0.0003991287134	ASS	PPP2R2D	+	chr10:131943967-131944145	chr10:131944094-131944145	-0.123	0.0004132445768	ANKAR
ASS	FAM111A	+	chr11:59148703-59148953	chr11:59148835-59148953	0.327	0.0004411870038	ASS	ZNF232	-	chr17:5109393-5109868	chr17:5109393-5109768	-0.175	0.0004438203268	ANKDD1A
ASS	ANKRD10	-	chr13:110900529-110900694	chr13:110900529-110900661	0.144	0.0004438203268	ASS	TMEM268	+	chr9:114624135-114624459	chr9:114624349-114624459	-0.432	0.0004438203268	ANKHD1
ASS	GAPVD1	+	chr9:125321432-125321562	chr9:125321432-125321499	0.203	0.0004619053114	ASS	DGKZ	+	chr11:46368001-46368079	chr11:46368004-46368079	-0.423	0.0004772283099	ANKHD1-EIF4EBP3
ASS	JKAMP	+	chr14:59486712-59486804	chr14:59486730-59486804	0.112	0.0004772283099	ASS	TRIM69	+	chr15:44758612-44758854	chr15:44758620-44758854	-0.228	0.0004772283099	ANKMY1
ASS	CEP70	-	chr3:138572858-138572984	chr3:138572858-138572932	0.362	0.0004772283099	ASS	BAHD1	+	chr15:40465334-40465435	chr15:40465343-40465435	-0.252	0.000491510266	ANKRD10
ASS	NUSAP1	+	chr15:41350990-41351129	chr15:41351032-41351129	0.162	0.0004781326667	ASS	FOPNL	-	chr16:15882948-15883048	chr16:15882948-15883020	-0.557	0.0005506993828	ANKRD11
ASS	LMBR1L	-	chr12:49106510-49106746	chr12:49106544-49106746	0.188	0.0004977651348	ASS	ARID1A	+	chr1:26772811-26772987	chr1:26772814-26772987	-0.205	0.0005909680069	ANKRD12
ASS	PIGG	+	chr4:521055-521273	chr4:521055-521249	0.116	0.0004977651348	ASS	NAGK	+	chr2:71072825-71073594	chr2:71073481-71073594	-0.117	0.0006627704568	ANKRD20A11P
ASS	FUZ	-	chr19:49812614-49812736	chr19:49812637-49812736	0.214	0.0005034348025	ASS	SLC25A14	+	chrX:130340106-130340353	chrX:130340106-130340344	-0.453	0.0008521869982	ANKRD27
ASS	KANK1	+	chr9:732377-734835	chr9:734747-734835	0.169	0.0005420927632	ASS	KAT6B	+	chr10:74975398-74976330	chr10:74975398-74975781	-0.395	0.0009030718475	ANKRD36
ASS	SSBP1	+	chr7:141741748-141741798	chr7:141741748-141741792	0.33	0.0005479620032	ASS	LRRC75B	-	chr22:24588213-24589393	chr22:24588213-24588329	-0.394	0.0009628756991	ANKRD39
ASS	HERC2P9	+	chr15:28655272-28655691	chr15:28655272-28655420	0.469	0.0006755196922	ASS	SNHG8	+	chr4:118278708-118279137	chr4:118278708-118278792	-0.114	0.0009901676837	ANKS3
ASS	SLC25A19	-	chr17:75288497-75288591	chr17:75288501-75288591	0.165	0.0007135351241	ASS	SH3D19	-	chr4:151176524-151176687	chr4:151176533-151176687	-0.127	0.001051640299	ANO10
ASS	PSEN1	+	chr14:73148019-73148106	chr14:73148019-73148094	0.258	0.0007399025448	ASS	ARHGEF40	+	chr14:21075331-21075765	chr14:21075331-21075499	-0.301	0.001128835062	ANO6
ASS	SART3	-	chr12:108544426-108544532	chr12:108544426-108544478	0.142	0.000777512798	ASS	EFNA1	+	chr1:155133502-155133780	chr1:155133729-155133780	-0.221	0.001140508688	ANXA2
ASS	NDOR1	+	chr9:137216066-137216188	chr9:137216093-137216188	0.142	0.0008449208851	ASS	CCHCR1	-	chr6:31144628-31144788	chr6:31144686-31144788	-0.201	0.001143198533	ANXA4
ASS	PEX1	-	chr7:92501506-92502079	chr7:92501506-92501673	0.104	0.0008732873205	ASS	TMEM94	+	chr17:75494626-75494808	chr17:75494709-75494808	-0.219	0.00121186621	AP1AR
ASS	MAPRE3	+	chr2:27024095-27024297	chr2:27024095-27024252	0.171	0.0008976924693	ASS	TOP3B	-	chr22:21975904-21976187	chr22:21975980-21976187	-0.44	0.001218214733	AP1G1
ASS	ZMYM5	-	chr13:19851354-19852190	chr13:19851688-19852190	0.216	0.0009282013644	ASS	RETSAT	-	chr2:85345512-85346094	chr2:85345974-85346094	-0.122	0.001218214733	AP1G2
ASS	ARHGAP26	+	chr5:143207197-143207473	chr5:143207197-143207308	0.25	0.0009743166039	ASS	PPT2-EGFL8	+	chr6:32167086-32167257	chr6:32167102-32167257	-0.136	0.001225588831	AP1S2
ASS	ARL13B	+	chr3:93996547-93996684	chr3:93996562-93996684	0.325	0.001110868338	ASS	ZNF596	+	chr8:243721-243858	chr8:243721-243805	-0.172	0.001472187424	AP2A2
ASS	CNOT3	+	chr19:54145541-54145817	chr19:54145594-54145817	0.135	0.001111191173	ASS	BZW2	+	chr7:16646130-16646304	chr7:16646130-16646288	-0.135	0.001551747794	AP2B1
ASS	ATP11A	+	chr13:112881812-112887168	chr13:112881875-112887168	0.39	0.001121865795	ASS	MRPL55	-	chr1:228108436-228109021	chr1:228108954-228109021	-0.126	0.001605495495	AP2M1
ASS	PBRM1	-	chr3:52679573-52679726	chr3:52679573-52679723	0.207	0.0011645449	ASS	PACRGL	+	chr4:20700457-20700747	chr4:20700457-20700611	-0.242	0.001751023104	AP3B1
ASS	BRF1	-	chr14:105219150-105219298	chr14:105219150-105219232	0.185	0.001386479338	ASS	NGLY1	-	chr3:25736308-25737455	chr3:25737333-25737455	-0.3	0.00181880726	AP3S2
ASS	STRN4	-	chr19:46727451-46727567	chr19:46727451-46727546	0.177	0.00157658726	ASS	CCDC66	+	chr3:56566593-56566759	chr3:56566593-56566740	-0.215	0.001851714863	AP4M1
ASS	CYLD	+	chr16:50749548-50750202	chr16:50749575-50750202	0.149	0.001576710913	ASS	PKMYT1	-	chr16:2973137-2973253	chr16:2973137-2973215	-0.365	0.00200752727	APAF1
ASS	SLC37A3	-	chr7:140352061-140352181	chr7:140352061-140352146	0.119	0.001576710913	ASS	PXN	-	chr12:120215102-120215417	chr12:120215102-120215273	-0.121	0.002194122714	APBA2
ASS	RAP1GAP	-	chr1:21597960-21598095	chr1:21597960-21598040	0.321	0.001576710913	ASS	ARPC1B	+	chr7:99377337-99377455	chr7:99377337-99377439	-0.21	0.002309111532	APBB2
ASS	MSTO1	+	chr1:155611688-155611852	chr1:155611688-155611827	0.319	0.001671111688	ASS	RFC5	+	chr12:118017689-118018053	chr12:118017689-118017815	-0.284	0.002352533445	APEH
ASS	MRNIP	-	chr5:179853196-179853437	chr5:179853377-179853437	0.124	0.001721902013	ASS	TLE2	-	chr19:3019282-3019466	chr19:3019282-3019463	-0.457	0.002391964041	APITD1-CORT
ASS	KMT2C	-	chr7:152156204-152156346	chr7:152156204-152156274	0.104	0.001747876875	ASS	KLC1	+	chr14:103679383-103679545	chr14:103679383-103679518	-0.222	0.002423306488	APLP1
ASS	APBA2	+	chr15:28921639-28921749	chr15:28921639-28921718	0.126	0.001751023104	ASS	CASP8	+	chr2:201266460-201266791	chr2:201266486-201266791	-0.127	0.002459197716	APLP2
ASS	SMTN	+	chr22:31088453-31088606	chr22:31088512-31088606	0.149	0.00185771104	ASS	CPSF7	-	chr11:61420469-61421608	chr11:61421389-61421608	-0.151	0.002518662501	APOM
ASS	PCBP1-AS1	-	chr2:70085815-70086273	chr2:70086123-70086273	0.138	0.001973456982	ASS	ADCY6	-	chr12:48773468-48774098	chr12:48773468-48773647	-0.286	0.002706536394	APOPT1
ASS	RAP1GAP	-	chr1:21597960-21598064	chr1:21597960-21598040	0.156	0.002071148324	ASS	NSD2	+	chr4:1952751-1953524	chr4:1953323-1953524	-0.107	0.002824582625	APRT
ASS	ZNF76	+	chr6:35290252-35290382	chr6:35290265-35290382	0.138	0.002194122714	ASS	RNF32	+	chr7:156676294-156677130	chr7:156676418-156677130	-0.385	0.002902268724	APTX
ASS	STIL	-	chr1:47312947-47313156	chr1:47312947-47313021	0.653	0.002250382184	ASS	TANGO2	+	chr22:20021068-20021246	chr22:20021068-20021134	-0.336	0.002947582078	ARAP1
ASS	SDR39U1	-	chr14:24442108-24442260	chr14:24442177-24442260	0.186	0.002257648074	ASS	NPHP1	-	chr2:110163047-110163135	chr2:110163047-110163132	-0.498	0.003019796635	AREL1
ASS	IVD	+	chr15:40407635-40407812	chr15:40407635-40407725	0.119	0.00226146107	ASS	ADARB1	+	chr21:45222017-45223577	chr21:45222017-45222107	-0.212	0.003150772569	ARF4-AS1
ASS	GALE	-	chr1:23796508-23796782	chr1:23796696-23796782	0.129	0.002262636209	ASS	SLC35A2	-	chrX:48904745-48905482	chrX:48904745-48904892	-0.268	0.003195651006	ARFGAP2
ASS	THOC2	-	chrX:123610917-123610963	chrX:123610917-123610957	0.113	0.002286623134	ASS	FCHSD1	-	chr5:141644858-141644942	chr5:141644858-141644938	-0.113	0.003195651006	ARFIP1
ASS	SECISBP2	+	chr9:89334521-89334730	chr9:89334635-89334730	0.158	0.002486846193	ASS	SLC52A2	+	chr8:144358592-144359065	chr8:144358592-144358900	-0.218	0.003268335568	ARHGAP11B
ASS	SETD5	+	chr3:9434276-9434485	chr3:9434333-9434485	0.181	0.002983828513	ASS	TRIQK	-	chr8:92885319-92886735	chr8:92885319-92886667	-0.188	0.003596342349	ARHGAP21
ASS	MRNIP	-	chr5:179853196-179853437	chr5:179853377-179853437	0.513	0.003083787964	ASS	DMAP1	+	chr1:44213662-44213858	chr1:44213708-44213858	-0.247	0.003715223759	ARHGAP23
ASS	IRF3	-	chr19:49661449-49662328	chr19:49661947-49662328	0.191	0.003142933795	ASS	HNRNPH3	+	chr10:68338502-68338687	chr10:68338502-68338642	-0.114	0.004040130954	ARHGAP26
ASS	CPSF7	-	chr11:61421053-61421130	chr11:61421053-61421127	0.301	0.003195651006	ASS	INTU	+	chr4:127685441-127687867	chr4:127687677-127687867	-0.203	0.004053514847	ARHGAP32
ASS	C1orf112	+	chr1:169804074-169804240	chr1:169804111-169804240	0.17	0.003340729773	ASS	CENPK	-	chr5:65554796-65554946	chr5:65554796-65554898	-0.103	0.004100974342	ARHGAP33
ASS	FAM111A	+	chr11:59148796-59148953	chr11:59148835-59148953	0.215	0.003383676778	ASS	PCBP1-AS1	-	chr2:70083506-70083687	chr2:70083537-70083687	-0.299	0.004120571796	ARHGEF1
ASS	CBWD5	+	chr9:65732946-65733122	chr9:65732992-65733122	0.431	0.003471603937	ASS	SLC2A11	+	chr22:23875084-23875241	chr22:23875120-23875241	-0.59	0.004351922584	ARHGEF10
ASS	MIGA2	+	chr9:129067994-129068332	chr9:129068197-129068332	0.18	0.003471603937	ASS	U2AF1L4	-	chr19:35744322-35744738	chr19:35744322-35744421	-0.35	0.004351922584	ARHGEF10L
ASS	ANK3	-	chr10:60059339-60060006	chr10:60059339-60059430	0.164	0.003617031386	ASS	ISG20	+	chr15:88650234-88652310	chr15:88652169-88652310	-0.128	0.004538961798	ARHGEF11
ASS	FKBP10	+	chr17:41819529-41820049	chr17:41819529-41819675	0.136	0.003748982002	ASS	CNOT8	+	chr5:154858638-154858816	chr5:154858638-154858768	-0.145	0.004634626811	ARHGEF12
ASS	PCBP1-AS1	-	chr2:70086054-70086269	chr2:70086123-70086269	0.295	0.003792655473	ASS	ZWINT	-	chr10:56358804-56358947	chr10:56358908-56358947	-0.184	0.004634626811	ARHGEF3
ASS	C11orf57	+	chr11:112075566-112075684	chr11:112075566-112075650	0.235	0.00395677552	ASS	MINK1	+	chr17:4890221-4890735	chr17:4890516-4890735	-0.395	0.004661268084	ARHGEF39
ASS	CAMK2D	-	chr4:113509637-113509675	chr4:113509637-113509672	0.216	0.004100974342	ASS	NABP1	+	chr2:191678135-191678705	chr2:191678135-191678241	-0.321	0.004867116873	ARHGEF40
ASS	FAM193B	-	chr5:177523158-177523265	chr5:177523162-177523265	0.368	0.004196039296	ASS	KB-1572G7.2	-	chr22:23686830-23686995	chr22:23686837-23686995	-0.224	0.005345968046	ARHGEF7
ASS	ZSCAN26	+	chr6:28271853-28272339	chr6:28271853-28272336	0.153	0.004252062559	ASS	PRR13	+	chr12:53443486-53443773	chr12:53443540-53443773	-0.226	0.005572013596	ARHGEF9
ASS	KIF2A	+	chr5:62352587-62352710	chr5:62352587-62352653	0.164	0.004282582856	ASS	FAM114A2	-	chr5:154038292-154038434	chr5:154038387-154038434	-0.106	0.005638572567	ARID1A
ASS	WDR26	-	chr1:224431476-224431581	chr1:224431524-224431581	0.166	0.004296445586	ASS	MRPL43	-	chr10:100983947-100984074	chr10:100984033-100984074	-0.163	0.005849655558	ARID4B
ASS	DPH5	-	chr1:101001466-101001587	chr1:101001466-101001544	0.225	0.004661268084	ASS	RREB1	+	chr6:7181123-7181289	chr6:7181123-7181246	-0.136	0.005879821613	ARIH2
ASS	ZBTB17	-	chr1:15946153-15946316	chr1:15946153-15946294	0.104	0.004732969007	ASS	ARHGEF1	+	chr19:41892024-41892373	chr19:41892330-41892373	-0.155	0.006062200051	ARL13B
ASS	PANK4	-	chr1:2510677-2510782	chr1:2510677-2510748	0.32	0.004748089327	ASS	C19orf66	+	chr19:10091475-10091630	chr19:10091583-10091630	-0.246	0.006127776705	ARL14EP
ASS	BPHL	+	chr6:3138040-3140509	chr6:3140385-3140509	0.145	0.004748089327	ASS	FZR1	+	chr19:3534420-3534522	chr19:3534420-3534513	-0.106	0.006456856362	ARL15
ASS	ATG16L2	+	chr11:72821198-72821741	chr11:72821667-72821741	0.398	0.00485576925	ASS	BPTF	+	chr17:67945408-67946325	chr17:67945408-67945896	-0.11	0.00665733317	ARL16
ASS	DOCK10	-	chr2:224805407-224805529	chr2:224805407-224805502	0.187	0.004936945245	ASS	SCAPER	-	chr15:76771723-76771954	chr15:76771741-76771954	-0.241	0.006799172032	ARL4A
ASS	PSMA3-AS1	-	chr14:58273978-58274135	chr14:58273978-58274010	0.23	0.00508214036	ASS	ZNF286A	+	chr17:15700047-15700366	chr17:15700134-15700366	-0.398	0.00692806339	ARL6IP4
ASS	CCDC90B	-	chr11:83273950-83274740	chr11:83273950-83273992	0.12	0.005087330558	ASS	NAT9	-	chr17:74773575-74773688	chr17:74773575-74773685	-0.19	0.00736127707	ARMC4
ASS	CHD3	+	chr17:7907890-7908019	chr17:7907893-7908019	0.274	0.005383440513	ASS	FNBP1	-	chr9:129923843-129923996	chr9:129923843-129923939	-0.161	0.007390962247	ARMCX5-GPRASP2
ASS	ST3GAL6-AS1	-	chr3:98718418-98718521	chr3:98718423-98718521	0.268	0.005488468139	ASS	AURKB	-	chr17:8207737-8207901	chr17:8207737-8207840	-0.343	0.007397341977	ARNT
ASS	OFD1	+	chrX:13752703-13753441	chrX:13753367-13753441	0.297	0.00566275157	ASS	RALGPS1	+	chr9:127212943-127213049	chr9:127212967-127213049	-0.109	0.007750127607	ARNTL2
ASS	PAM	+	chr5:103028181-103028238	chr5:103028184-103028238	0.116	0.005862508033	ASS	COA1	-	chr7:43650492-43650712	chr7:43650611-43650712	-0.196	0.007807705261	ARPC1B
ASS	TJAP1	+	chr6:43498922-43499100	chr6:43498974-43499100	0.158	0.005932310734	ASS	TANGO2	+	chr22:20061529-20061706	chr22:20061529-20061683	-0.657	0.007874013691	ARPC4
ASS	ATG16L2	+	chr11:72821547-72821741	chr11:72821667-72821741	0.453	0.006062200051	ASS	SMARCA4	+	chr19:11058254-11058363	chr19:11058257-11058363	-0.134	0.008425492765	ARPP19
ASS	NDUFAF7	+	chr2:37241577-37241791	chr2:37241577-37241637	0.116	0.00662797432	ASS	SLC22A17	-	chr14:23347459-23347731	chr14:23347513-23347731	-0.149	0.00896509776	ARRB1
ASS	TTC31	+	chr2:74483321-74483563	chr2:74483321-74483410	0.336	0.006799172032	ASS	MSI2	+	chr17:57674917-57675126	chr17:57674971-57675126	-0.372	0.009154824571	ARRDC1
ASS	PAAF1	+	chr11:73886928-73887099	chr11:73887039-73887099	0.125	0.006867038563	ASS	PARP14	+	chr3:122720254-122721653	chr3:122720254-122720388	-0.124	0.009250039851	ARVCF
ASS	CDK11A	-	chr1:1722587-1722831	chr1:1722707-1722831	0.114	0.006888488616	ASS	BCS1L	+	chr2:218660306-218660482	chr2:218660306-218660421	-0.338	0.009675218226	ASAH1
ASS	MARK3	+	chr14:103468032-103468186	chr14:103468080-103468186	0.136	0.007114890212	ASS	FANCA	-	chr16:89739128-89739289	chr16:89739132-89739289	-0.242	0.009715487683	ASAH2B
ASS	THNSL1	+	chr10:25016657-25016789	chr10:25016657-25016692	0.211	0.007391124316	ASS	PRKAG1	-	chr12:49005461-49005592	chr12:49005461-49005543	-0.39	0.01015531981	ASAP1
ASS	DNAJC10	+	chr2:182739544-182740388	chr2:182740298-182740388	0.235	0.00743209034	ASS	YPEL5	+	chr2:30155814-30155910	chr2:30155849-30155910	-0.389	0.01033981784	ASAP2
ASS	TIMM17B	-	chrX:48895351-48895501	chrX:48895351-48895454	0.121	0.00750782119	ASS	PI4KAP1	-	chr22:18547245-18547421	chr22:18547245-18547373	-0.163	0.01057437769	ASB8
ASS	HSF4	+	chr16:67167719-67167947	chr16:67167823-67167947	0.308	0.007648353669	ASS	BDP1	+	chr5:71541453-71541682	chr5:71541456-71541682	-0.113	0.01057437769	ASCC2
ASS	ZDHHC4	+	chr7:6577467-6577720	chr7:6577467-6577498	0.146	0.007745721211	ASS	DPM1	-	chr20:50941104-50941209	chr20:50941128-50941209	-0.303	0.01135523264	ASCC3
ASS	PBRM1	-	chr3:52587352-52587510	chr3:52587352-52587435	0.106	0.007750127607	ASS	RPL7L1	+	chr6:42880860-42881082	chr6:42880860-42880966	-0.144	0.0115195204	ASIC1
ASS	RNF181	+	chr2:85597103-85597581	chr2:85597444-85597581	0.288	0.007889084592	ASS	RUSC1	+	chr1:155326579-155327132	chr1:155326897-155327132	-0.112	0.01152357239	ASPH
ASS	POMT1	+	chr9:131509902-131510062	chr9:131509902-131509996	0.233	0.008187538655	ASS	DCAF11	+	chr14:24116916-24117044	chr14:24116971-24117044	-0.222	0.01159601821	ASPM
ASS	WHAMM	+	chr15:82826387-82826496	chr15:82826409-82826496	0.163	0.008521201378	ASS	ST5	-	chr11:8702571-8703611	chr11:8702571-8702720	-0.104	0.01220499947	ASPSCR1
ASS	DAG1	+	chr3:49476848-49476905	chr3:49476848-49476900	0.565	0.008667503029	ASS	HACE1	-	chr6:104777012-104777110	chr6:104777012-104777097	-0.242	0.01220499947	ATAD2
ASS	RPP25L	-	chr9:34611859-34612104	chr9:34612063-34612104	0.183	0.008976958899	ASS	SH3BP2	+	chr4:2831922-2832412	chr4:2832330-2832412	-0.38	0.01237140713	ATAD2B
ASS	KCTD9	-	chr8:25439605-25439792	chr8:25439605-25439664	0.234	0.00907788124	ASS	U2AF1L4	-	chr19:35744322-35744981	chr19:35744322-35744421	-0.329	0.01277456951	ATAD5
ASS	PABPC1L	+	chr20:44938044-44938191	chr20:44938060-44938191	0.124	0.009237124357	ASS	MTG1	+	chr10:133401528-133402245	chr10:133402148-133402245	-0.122	0.01355011343	ATAT1
ASS	SDHAP1	-	chr3:195965428-195965577	chr3:195965428-195965542	0.143	0.00934947157	ASS	C1orf159	-	chr1:1090352-1091374	chr1:1090352-1090428	-0.65	0.01370844678	ATF6B
ASS	ALG9	-	chr11:111838248-111838399	chr11:111838248-111838378	0.2	0.009557061151	ASS	KIZ	+	chr20:21205490-21205584	chr20:21205490-21205530	-0.295	0.01371813043	ATG10
ASS	PFAS	+	chr17:8266500-8266898	chr17:8266752-8266898	0.201	0.009613703767	ASS	MRRF	+	chr9:122285168-122285946	chr9:122285168-122285287	-0.116	0.01403880967	ATG13
ASS	ZNF341	+	chr20:33757147-33757343	chr20:33757147-33757322	0.258	0.009675218226	ASS	VPS29	-	chr12:110499456-110499546	chr12:110499534-110499546	-0.262	0.01416002077	ATG16L1
ASS	RMND1	-	chr6:151436419-151436554	chr6:151436445-151436554	0.147	0.009959142975	ASS	CPM	-	chr12:68933141-68933199	chr12:68933169-68933199	-0.431	0.01433723906	ATG16L2
ASS	TCTN1	+	chr12:110647195-110647336	chr12:110647210-110647336	0.456	0.009968353808	ASS	EPB41	+	chr1:28987430-28987905	chr1:28987447-28987905	-0.471	0.01505331047	ATG4D
ASS	DUXAP8	+	chr22:15790660-15790798	chr22:15790684-15790798	0.136	0.01002753622	ASS	ADCY10P1	+	chr6:41108948-41109904	chr6:41109712-41109904	-0.34	0.01505331047	ATG5
ASS	BMP1	+	chr8:22176923-22177139	chr8:22176960-22177139	0.119	0.01003596905	ASS	SHKBP1	+	chr19:40590729-40590853	chr19:40590732-40590853	-0.151	0.0151374011	ATP11A
ASS	C21orf58	-	chr21:46314715-46315049	chr21:46314715-46314880	0.528	0.01015531981	ASS	R3HDM1	+	chr2:135641535-135641790	chr2:135641535-135641604	-0.256	0.01629666727	ATP11B
ASS	FUBP1	-	chr1:77947452-77947552	chr1:77947452-77947547	0.12	0.01033981784	ASS	PIDD1	-	chr11:800984-801120	chr11:801001-801120	-0.139	0.01657875278	ATP11C
ASS	HSF4	+	chr16:67167460-67167599	chr16:67167474-67167599	0.154	0.01065698785	ASS	GPANK1	-	chr6:31663852-31664580	chr6:31663852-31664577	-0.155	0.01713879478	ATP13A1
ASS	PRKCSH	+	chr19:11447692-11447789	chr19:11447722-11447789	0.185	0.01102843369	ASS	PLEKHH3	-	chr17:42670993-42671130	chr17:42671002-42671130	-0.166	0.0177309439	ATP2A1
ASS	FAM219B	-	chr15:74904981-74905059	chr15:74904981-74905039	0.169	0.01124910275	ASS	CDK11A	-	chr1:1719327-1719455	chr1:1719357-1719455	-0.248	0.0177309439	ATP2B1
ASS	HUS1	-	chr7:47979409-47979553	chr7:47979420-47979553	0.233	0.01147637878	ASS	MVD	-	chr16:88656104-88656561	chr16:88656104-88656304	-0.376	0.01930354929	ATP5C1
ASS	ASB8	-	chr12:48150086-48150170	chr12:48150086-48150140	0.454	0.01183865018	ASS	RUSC1	+	chr1:155326609-155327132	chr1:155326897-155327132	-0.131	0.01930354929	ATP5D
ASS	CACNB3	+	chr12:48826244-48826518	chr12:48826366-48826518	0.105	0.01216881001	ASS	ATG16L1	+	chr2:233272965-233273777	chr2:233272965-233273052	-0.245	0.01938843415	ATP5G1
ASS	CDK10	+	chr16:89694568-89694788	chr16:89694664-89694788	0.186	0.01220499947	ASS	KLC1	+	chr14:103679383-103679545	chr14:103679383-103679518	-0.171	0.02000464033	ATP5H
ASS	IGSF8	-	chr1:160091339-160091631	chr1:160091339-160091608	0.108	0.01220499947	ASS	LIG1	-	chr19:48120166-48121322	chr19:48121169-48121322	-0.288	0.02059523257	ATP5O
ASS	UBE2D3	-	chr4:102809067-102809184	chr4:102809067-102809159	0.505	0.01254382835	ASS	CCDC189	-	chr16:30759653-30759809	chr16:30759653-30759724	-0.478	0.02067176633	ATP5SL
ASS	MPV17	-	chr2:27312993-27313154	chr2:27312993-27313074	0.194	0.01272076164	ASS	TMEM214	+	chr2:27037789-27037892	chr2:27037814-27037892	-0.32	0.0214154521	ATP6V0A1
ASS	PLEKHH3	-	chr17:42672085-42672392	chr17:42672085-42672212	0.295	0.01358674745	ASS	CCDC24	+	chr1:43993886-43993964	chr1:43993889-43993964	-0.492	0.02163235123	ATP6V0B
ASS	ZNF664	+	chr12:123973218-123973352	chr12:123973218-123973303	0.12	0.0156075419	ASS	RYK	-	chr3:134191848-134191983	chr3:134191848-134191974	-0.132	0.02228411796	ATP6V1B1
ASS	PROCA1	-	chr17:28704306-28704616	chr17:28704306-28704435	0.396	0.01603843118	ASS	IMPA2	+	chr18:12012169-12012349	chr18:12012169-12012215	-0.136	0.02239688901	ATP9B
ASS	IL17RC	+	chr3:9923880-9924137	chr3:9923880-9924020	0.159	0.01632096844	ASS	AURKA	-	chr20:56392153-56392295	chr20:56392167-56392295	-0.231	0.02306657183	ATPAF1
ASS	PIF1	-	chr15:64819846-64820019	chr15:64819846-64819986	0.253	0.01652397872	ASS	SYVN1	-	chr11:65132247-65132780	chr11:65132731-65132780	-0.118	0.02387795607	ATPAF2
ASS	CACNA2D1	-	chr7:81997178-81997250	chr7:81997178-81997235	0.334	0.01823872704	ASS	RECQL	-	chr12:21501169-21501372	chr12:21501169-21501215	-0.117	0.02462972179	ATRX

ASS	ZNF3	-	chr7:100077228-100077433	chr7:100077302-100077433	0.106	0.01848702629
ASS	GAS8	+	chr16:90031175-90031496	chr16:90031298-90031496	0.247	0.01930354929
ASS	RCAN3	+	chr1:24502896-24503150	chr1:24502896-24503117	0.534	0.01930630344
ASS	FOXRED1	+	chr11:126273315-126273454	chr11:126273338-126273454	0.153	0.02000335302
ASS	PKD1	-	chr16:2105321-2105707	chr16:2105321-2105474	0.196	0.02000335302
ASS	SFT2D1	-	chr6:166322856-166324595	chr6:166322856-166322886	0.146	0.02088288316
ASS	ZMAT1	-	chrX:101895813-101895882	chrX:101895813-101895879	0.243	0.02099159975
ASS	HADH	+	chr4:108023473-108027760	chr4:108023473-108023563	0.116	0.02124288484
ASS	SERGEF	-	chr11:18010070-18010139	chr11:18010074-18010139	0.309	0.02139215565
ASS	ZNF596	+	chr8:242886-243013	chr8:242917-243013	0.109	0.02146154903
ASS	TECR	+	chr19:14563140-14563257	chr19:14563173-14563257	0.126	0.02150085512
ASS	TJAP1	+	chr6:43498922-43499100	chr6:43498977-43499100	0.139	0.0218019065
ASS	RP4-639F20.1	+	chr1:94962924-94963270	chr1:94962978-94963270	0.159	0.02193666938
ASS	DPY19L3	+	chr19:32467473-32468813	chr19:32468730-32468813	0.183	0.02228411796
ASS	KMT5B	-	chr11:68171231-68171296	chr11:68171231-68171251	0.393	0.02228411796
ASS	VPS54	-	chr2:63981645-63981887	chr2:63981645-63981851	0.212	0.02258460249
ASS	HOOK2	-	chr19:12774560-12774741	chr19:12774634-12774741	0.197	0.02371124663
ASS	HDLBP	-	chr2:241269200-241269295	chr2:241269205-241269295	0.332	0.02387795607
ASS	PROSER3	+	chr19:35765033-35765176	chr19:35765066-35765176	0.148	0.02479012398
ASS	AP1G2	-	chr14:23564045-23564221	chr14:23564045-23564159	0.319	0.0254485429
ASS	TRIM33	-	chr1:114397607-114397990	chr1:114397607-114397860	0.173	0.0254485429
ASS	C21orf2	-	chr21:44334060-44335804	chr21:44335757-44335804	0.133	0.025564861
ASS	RBM6	+	chr3:49967197-49968748	chr3:49967469-49968748	0.154	0.02566195299
ASS	ZNF655	+	chr7:99561887-99562494	chr7:99562367-99562494	0.111	0.02597407973
ASS	RAD9A	+	chr11:67393690-67393790	chr11:67393722-67393790	0.407	0.02636430401
ASS	NSUN5P1	+	chr7:75415591-75415778	chr7:75415591-75415770	0.186	0.02639140838
ASS	VPS28	-	chr8:144425874-144426063	chr8:144425921-144426063	0.359	0.02683660206
ASS	ASAH1	-	chr8:18083980-18084980	chr8:18084675-18084980	0.215	0.02688224421
ASS	CLN6	-	chr15:68211555-68211863	chr15:68211674-68211863	0.117	0.0269999003
ASS	ENSA	-	chr1:150627311-150627592	chr1:150627466-150627592	0.106	0.02703202502
ASS	MSTO1	+	chr1:155613656-155613766	chr1:155613656-155613733	0.16	0.02717992751
ASS	NCOR2	-	chr12:124327408-124327633	chr12:124327546-124327633	0.164	0.02794282114
ASS	CNOT2	+	chr12:70294070-70294294	chr12:70294236-70294294	0.356	0.02871858636
ASS	POMZP3	-	chr7:76611453-76611627	chr7:76611453-76611591	0.25	0.02937565073
ASS	ZFAND2A	-	chr7:1152906-1153241	chr7:1152906-1153224	0.139	0.02999909818
ASS	C6orf48	+	chr6:31836294-31836517	chr6:31836423-31836517	0.111	0.02999909818
ASS	C6orf48	+	chr6:31836298-31836517	chr6:31836423-31836517	0.113	0.03010599228
ASS	HAUS4	-	chr14:22956745-22957094	chr14:22956915-22957094	0.171	0.03020364092
ASS	MTRF1L	-	chr6:152995135-152995319	chr6:152995135-152995294	0.242	0.03082514448
ASS	RABL2B	-	chr22:50769424-50769549	chr22:50769454-50769549	0.296	0.03112961493
ASS	USP33	-	chr1:77721830-77721925	chr1:77721830-77721901	0.164	0.03232140899
ASS	OCIAD1	+	chr4:48831226-48831433	chr4:48831226-48831398	0.283	0.03335973344
ASS	CCS	+	chr11:66605153-66605416	chr11:66605338-66605416	0.119	0.03445710622
ASS	SETD4	-	chr21:36058815-36058973	chr21:36058815-36058924	0.195	0.03505499569
ASS	C14orf159	+	chr14:91215404-91215793	chr14:91215404-91215556	0.236	0.03523702781
ASS	MUTYH	-	chr1:45332917-45333324	chr1:45333284-45333324	0.149	0.03557564081
ASS	ERBB2	+	chr17:39694680-39695181	chr17:39694987-39695181	0.372	0.03581629335
ASS	TMEM67	+	chr8:93758482-93758576	chr8:93758486-93758576	0.22	0.03659565077
ASS	FKBP1B	+	chr2:24063018-24063681	chr2:24063063-24063681	0.34	0.03680649749
ASS	TBC1D32	-	chr6:121115171-121115747	chr6:121115171-121115241	0.264	0.03686690828
ASS	ZBTB8OS	-	chr1:32634701-32634792	chr1:32634767-32634792	0.234	0.03728001552
ASS	PTBP2	+	chr1:96804784-96804939	chr1:96804799-96804939	0.389	0.03982276621
ASS	FUZ	-	chr19:49811362-49811491	chr19:49811362-49811467	0.594	0.03982276621
ASS	DDIT3	-	chr12:57517268-57517595	chr12:57517268-57517424	0.149	0.0401411959
ASS	PPP4R1L	-	chr20:58240514-58240710	chr20:58240514-58240610	0.14	0.04060591643
ASS	CMC2	-	chr16:80998284-80998473	chr16:80998284-80998354	0.193	0.04069570644
ASS	MSTO1	+	chr1:155611920-155612100	chr1:155611982-155612100	0.188	0.0463393071
ASS	TSACC	+	chr1:156346767-156346995	chr1:156346778-156346995	0.653	0.04759067616
ASS	SLC4A3	+	chr2:219633854-219633979	chr2:219633879-219633979	0.211	0.04759067616
ASS	HECTD4	-	chr12:112264083-112264212	chr12:112264089-112264212	0.156	0.04804249068
ASS	BICD1	+	chr12:32337498-32337816	chr12:32337498-32337706	0.166	0.04888897915
ASS	RAB15	-	chr14:64950578-64951151	chr14:64951073-64951151	0.116	0.04917885074
MXE	TPM3	-	chr1:154171412-154171488	chr1:154172028-154172104	0.121	0
MXE	PICALM	-	chr11:85983865-85983973	chr11:85990249-85990378	0.138	0
MXE	PICALM	-	chr11:85983865-85983973	chr11:85990249-85990399	0.281	0
MXE	ARIH2	+	chr3:48945135-48945181	chr3:48974957-48974979	0.774	0
MXE	ARIH2	+	chr3:48945135-48945181	chr3:48961611-48961679	0.948	0
MXE	ARIH2	+	chr3:48922747-48922811	chr3:48961611-48961679	0.828	0
MXE	ARIH2	+	chr3:48927461-48927813	chr3:48961611-48961679	0.347	0
MXE	ARIH2	+	chr3:48927461-48927813	chr3:48964918-48964982	0.314	0
MXE	ARIH2	+	chr3:48927461-48927813	chr3:48961611-48961679	0.349	0
MXE	OGDH	+	chr7:44647443-44647534	chr7:44647656-44647759	0.363	0
MXE	OGDH	+	chr7:44647443-44647534	chr7:44647656-44647759	0.358	0
MXE	ZFAND5	-	chr9:72356930-72357056	chr9:72360109-72360221	0.19	0
MXE	DNM2	+	chr19:10796060-10796199	chr19:10797379-10797518	0.455	0
MXE	SPAG9	-	chr17:50975862-50975901	chr17:50977107-50977221	0.251	0
MXE	LCORL	-	chr4:17873387-17878213	chr4:17886067-17886161	0.137	0
MXE	GAS5	-	chr1:173865509-173865547	chr1:173866176-173866206	0.233	0
MXE	GAS5	-	chr1:173865509-173865547	chr1:173865856-173866206	0.274	0
MXE	GAS5	-	chr1:173865856-173865894	chr1:173866527-173866796	0.254	0
MXE	GAS5	-	chr1:173865856-173865894	chr1:173866176-173866206	0.232	0
MXE	HMGN1	-	chr21:39345829-39345958	chr21:39347378-39347483	0.323	0
MXE	HMGN1	-	chr21:39345829-39345958	chr21:39347378-39347478	0.316	0
MXE	WASF3	+	chr13:26676548-26676724	chr13:26680034-26680201	0.44	0
MXE	EPB41L2	-	chr6:130870327-130870381	chr6:130880143-130880206	0.439	0
MXE	SYTL2	-	chr11:85709330-85709500	chr11:85714412-85714507	0.191	0
MXE	ASPH	-	chr8:61651049-61651124	chr8:61653567-61653660	0.132	0
MXE	MAP4K4	+	chr2:101860824-101860986	chr2:101863820-101864051	0.393	0
MXE	CHURC1-FNTB	+	chr14:64926009-64926080	chr14:65004248-65004313	0.234	0
MXE	ESYT2	-	chr7:158748193-158748280	chr7:158749648-158749723	0.142	0
MXE	FEZ2	-	chr2:36558437-36558513	chr2:36560784-36560865	0.131	0
MXE	FEZ2	-	chr2:36555682-36555748	chr2:36558437-36558513	0.176	0
MXE	PILRB	+	chr7:100353209-100353270	chr7:100353372-100354012	0.331	0
MXE	XPO1	-	chr2:61525491-61526521	chr2:61533771-61533903	0.12	0
MXE	ACTN4	+	chr19:38727945-38728026	chr19:38728315-38728381	0.24	0
MXE	MATR3	+	chr5:139314674-139314736	chr5:139315696-139315738	0.11	2.26E-14
MXE	AD000671.6	-	chr19:35744625-35744738	chr19:35745124-35745212	0.435	4.44E-14
MXE	CAST	+	chr5:96727488-96727530	chr5:96729152-96729209	0.368	6.23E-14
MXE	TPM2	-	chr9:35684731-35684807	chr9:35685063-35685139	0.365	8.39E-13
MXE	ZFAND5	-	chr9:72359417-72359521	chr9:72360109-72360221	0.191	1.68E-12
MXE	GSN	+	chr9:121281469-121281562	chr9:121300055-121300126	0.247	2.72E-12
MXE	MKKS	-	chr20:10412529-10413931	chr20:10420527-10420758	0.218	3.79E-12
MXE	TPM1	+	chr15:63061197-63061273	chr15:63061712-63061788	0.344	5.99E-12
MXE	CKLF	+	chr16:66558189-66558348	chr16:66563121-66563217	0.209	1.40E-11
MXE	AUP1	-	chr2:74528750-74529282	chr2:74529361-74529499	0.161	1.88E-11
MXE	GAS5	-	chr1:173865509-173865547	chr1:173865856-173865894	0.154	2.88E-11
MXE	ACTN4	+	chr19:38710256-38710342	chr19:38711275-38711361	0.262	4.35E-11
MXE	EED	+	chr11:86250295-86250448	chr11:86264171-86264263	0.118	4.38E-11
MXE	SLC29A1	+	chr6:44227262-44227342	chr6:44229389-44229471	0.274	4.53E-11
MXE	GAS5	-	chr1:173864674-173864704	chr1:173865228-173865282	0.206	5.01E-11
MXE	PILRB	+	chr7:100353372-100353483	chr7:100353894-100354012	0.349	1.28E-10
MXE	SRGAP1	+	chr12:64091275-64091378	chr12:64091892-64091926	0.576	3.66E-10
MXE	ARHGEF7	+	chr13:111205288-111205373	chr13:111209871-111210002	0.312	7.62E-10
MXE	MARK3	+	chr14:103491776-103492034	chr14:103498501-103498528	0.119	8.37E-10
MXE	DOCK9	-	chr13:98845328-98845372	chr13:98845923-98846060	0.468	1.11E-09
MXE	AGAP4	-	chr10:45841652-45841687	chr10:45844325-45844394	0.785	1.11E-09
MXE	AUP1	-	chr2:74528750-74529282	chr2:74529361-74529499	0.191	1.34E-09
MXE	EIF4G3	-	chr1:20997600-20997633	chr1:21001198-21001312	0.242	1.39E-09
MXE	CKLF-CMTM1	+	chr16:66558189-66558348	chr16:66563121-66563217	0.261	1.49E-09
MXE	FBXO22	+	chr15:75904490-75904629	chr15:75913202-75913290	0.364	1.52E-09
MXE	CARD8	-	chr19:48240961-48241063	chr19:48249522-48249633	0.292	2.00E-09

ASS	OPTN	+	chr10:13109096-13109288	chr10:13109111-13109288	-0.305	0.02544833074
ASS	MZF1	-	chr19:58573054-58573575	chr19:58573372-58573575	-0.21	0.025564861
ASS	DCAF4	+	chr14:72945883-72946027	chr14:72945887-72946027	-0.106	0.02566195299
ASS	RALGAPA1	-	chr14:35688458-35690003	chr14:35689835-35690003	-0.295	0.02598158189
ASS	ZSCAN5A	-	chr19:56314619-56314839	chr19:56314680-56314839	-0.126	0.02674759003
ASS	PBX4	-	chr19:19570585-19570833	chr19:19570686-19570833	-0.442	0.02703202502
ASS	CEP78	+	chr9:78251904-78252043	chr9:78251907-78252043	-0.161	0.02753281436
ASS	GABPB1	-	chr15:50301220-50301368	chr15:50301256-50301368	-0.122	0.02794282114
ASS	NUDT13	+	chr10:73126634-73126827	chr10:73126672-73126827	-0.103	0.02810489556
ASS	WDR31	-	chr9:113330983-113331116	chr9:113330983-113331113	-0.206	0.02812554226
ASS	XRN1	-	chr3:142332374-142332531	chr3:142332374-142332424	-0.119	0.02871858636
ASS	RABL2B	-	chr22:50782187-50782351	chr22:50782187-50782347	-0.333	0.02937565073
ASS	WDPCP	-	chr2:63439756-63439871	chr2:63439756-63439855	-0.142	0.02937565073
ASS	PRKCSH	+	chr19:11447438-11447618	chr19:11447438-11447597	-0.166	0.03010377877
ASS	ZNF559	+	chr19:9341616-9343843	chr19:9341694-9343843	-0.213	0.03031240378
ASS	RMND5B	+	chr5:178138107-178138396	chr5:178138107-178138258	-0.141	0.03086650433
ASS	UBE2D3	-	chr4:102826484-102826844	chr4:102826484-102826636	-0.14	0.0326899352
ASS	IP6K2	-	chr3:48715314-48715527	chr3:48715388-48715527	-0.414	0.03593330065
ASS	NPHP1	-	chr2:110164519-110164730	chr2:110164687-110164730	-0.238	0.0373288093
ASS	ZC3H11A	+	chr1:203796292-203796601	chr1:203796292-203796496	-0.116	0.03765181523
ASS	ZNF641	-	chr12:48347148-48347343	chr12:48347251-48347343	-0.276	0.03772791898
ASS	NEPRO	-	chr3:113012733-113013403	chr3:113013271-113013403	-0.264	0.03957103951
ASS	SENP6	+	chr6:75640683-75640704	chr6:75640686-75640704	-0.229	0.03982276621
ASS	TCAIM	+	chr3:44367455-44367708	chr3:44367531-44367708	-0.174	0.04017537365
ASS	PSMD6	-	chr3:64022642-64022801	chr3:64022738-64022801	-0.126	0.04120128176
ASS	ZCWPW1	-	chr7:100415974-100416097	chr7:100415974-100416094	-0.165	0.04156779975
ASS	HUWE1	-	chrX:53543840-53543968	chrX:53543885-53543968	-0.145	0.04156987741
ASS	BDH2	-	chr4:103085348-103086540	chr4:103086479-103086540	-0.231	0.04209827853
ASS	PIGA	-	chrX:15331215-15331992	chrX:15331873-15331992	-0.169	0.04313386635
ASS	ISG20	+	chr15:88650234-88652310	chr15:88652109-88652310	-0.225	0.0436367351
ASS	TAZ	+	chrX:154413206-154413358	chrX:154413206-154413339	-0.23	0.04391007992
ASS	WDR83	+	chr19:12670518-12670611	chr19:12670562-12670611	-0.299	0.04408854106
ASS	HAUS8	-	chr19:17059996-17060092	chr19:17059996-17060089	-0.131	0.04551241736
ASS	TIMM17B	-	chrX:48895351-48895972	chrX:48895351-48895501	-0.102	0.0463393071
ASS	FAHD2A	+	chr2:95410526-95411026	chr2:95410526-95410586	-0.122	0.04707260085
ASS	XRCC3	-	chr14:103711465-103711661	chr14:103711465-103711567	-0.26	0.04859579048
MXE	CTNND1	+	chr11:57789036-57789155	chr11:57794009-57794081	-0.371	0
MXE	FYN	-	chr6:111699514-111699670	chr6:111700103-111700268	-0.349	0
MXE	AP2M1	+	chr3:184178856-184179122	chr3:184180168-184180251	-0.238	0
MXE	PUDP	-	chrX:7077219-7077449	chrX:7105619-7105838	-0.462	0
MXE	PUDP	-	chrX:7077219-7077449	chrX:7105619-7105838	-0.583	0
MXE	GAS5	-	chr1:173865470-173865547	chr1:173865856-173865894	-0.266	0
MXE	GAS5	-	chr1:173864674-173864704	chr1:173865228-173865282	-0.224	0
MXE	MSL1	+	chr17:40129244-40129627	chr17:40132033-40132098	-0.237	0
MXE	NQO1	-	chr16:69713027-69713129	chr16:69714963-69715077	-0.394	0
MXE	SCHIP1	+	chr3:159866162-159866281	chr3:159886206-159886324	-0.247	0
MXE	TVP23C-CDRT4	-	chr17:15503097-15503232	chr17:15540432-15540561	-0.827	0
MXE	RP4-777O23.3	-	chr7:30500535-30500681	chr7:30524366-30524486	-0.115	0
MXE	SYTL2	-	chr11:85711112-85711232	chr11:85714412-85714507	-0.625	0
MXE	SYTL2	-	chr11:85714412-85714507	chr11:85717482-85717530	-0.512	0
MXE	NF2	+	chr22:29668332-29668446	chr22:29673268-29673486	-0.623	0
MXE	SLC29A1	+	chr6:44227262-44227342	chr6:44229389-44229471	-0.303	0
MXE	NUP93	+	chr16:56798475-56798538	chr16:56805503-56805632	-0.167	6.23E-14
MXE	RP4-777O23.3	-	chr7:30500535-30500681	chr7:30524366-30524486	-0.246	6.23E-14
MXE	CTNND1	+	chr11:57789036-57789155	chr11:57794009-57794081	-0.274	1.02E-13
MXE	KIAA1468	+	chr18:62279773-62279856	chr18:62280359-62280442	-0.495	1.61E-13
MXE	GPR158	+	chr10:25412249-25412473	chr10:25466650-25466719	-0.612	4.45E-12
MXE	SUN1	+	chr7:849519-849603	chr7:849922-850033	-0.397	5.58E-12
MXE	GAS5	-	chr1:173864483-173864506	chr1:173865228-173865282	-0.124	6.96E-12
MXE	SNX14	-	chr6:85541984-85542043	chr6:85543181-85543306	-0.275	9.64E-12
MXE	MRPS18C	+	chr4:83458345-83458429	chr4:83459739-83459797	-0.184	1.82E-11
MXE	DIABLO	-	chr12:122224511-122224644	chr12:122224644-122224748	-0.12	3.44E-11
MXE	PGAP2	+	chr11:3811249-3811424	chr11:3822908-3822993	-0.385	4.38E-11
MXE	SUN1	+	chr7:849519-849603	chr7:849922-850033	-0.266	1.97E-10
MXE	MRPL47	-	chr3:179598674-179598771	chr3:179601729-179601790	-0.101	3.57E-10
MXE	LINC01125	+	chr2:97699025-97699136	chr2:97701153-97701304	-0.638	3.98E-10
MXE	CNTLN	+	chr9:17273732-17273866	chr9:17298189-17298352	-0.54	6.62E-10
MXE	RASSF8-AS1	-	chr12:25952757-25953076	chr12:25956226-25956347	-0.53	9.32E-10
MXE	SEC31A	-	chr4:82861630-82861708	chr4:82862533-82862572	-0.328	1.11E-09
MXE	TRIQK	-	chr8:92921502-92921660	chr8:92954405-92954564	-0.132	1.64E-09
MXE	GAS5	-	chr1:173865470-173865547	chr1:173866176-173866206	-0.232	1.87E-09
MXE	CCDC88A	-	chr2:55301871-55302072	chr2:55303068-55303152	-0.243	1.99E-09
MXE	DST	-	chr6:56600068-56600221	chr6:56601442-56601676	-0.292	7.71E-09
MXE	LAS1L	-	chrX:65524055-65524262	chrX:65524563-65524614	-0.225	3.21E-08
MXE	FAM98A	-	chr2:33588334-33588519	chr2:33592079-33592214	-0.176	4.19E-08
MXE	CDC25C	-	chr5:138325814-138325904	chr5:138328483-138328529	-0.357	4.51E-08
MXE	SUN1	+	chr7:848417-848615	chr7:849922-850033	-0.231	1.08E-07
MXE	HNRNPAB	+	chr5:178209329-178209447	chr5:178210131-178210272	-0.117	1.08E-07
MXE	FANCI	+	chr15:89245194-89245311	chr15:89245755-89245839	-0.348	1.65E-07
MXE	CENPU	-	chr4:184697646-184697803	chr4:184700819-184700881	-0.15	1.65E-07
MXE	PPRC1	+	chr10:102144249-102144307	chr10:102146671-102147392	-0.277	2.09E-07
MXE	PPRC1	+	chr10:102144249-102144307	chr10:102146671-102147392	-0.142	3.20E-07
MXE	SYTL2	-	chr11:85714412-85714507	chr11:85717482-85717726	-0.242	3.60E-07
MXE	RP11-458D21.5	-	chr1:146136352-146136455	chr1:146142649-146142752	-0.66	4.61E-07
MXE	TPT1-AS1	+	chr13:45378529-45378671	chr13:45383090-45383235	-0.625	6.05E-07
MXE	ENO2	+	chr12:6917037-6917107	chr12:6917580-6917714	-0.153	6.05E-07
MXE	MTA1	+	chr14:105445666-105445852	chr14:105449358-105449409	-0.12	9.90E-07
MXE	FYN	-	chr6:111696276-111696456	chr6:111700103-111700268	-0.155	1.01E-06
MXE	EEF1D	-	chr8:143581228-143581328	chr8:143586218-143586290	-0.184	1.14E-06
MXE	HNRNPAB	+	chr5:178209329-178209432	chr5:178210131-178210272	-0.114	1.43E-06
MXE	SUZ12P1	+	chr17:30735032-30735097	chr17:30743300-30743369	-0.436	1.47E-06
MXE	DCUN1D4	+	chr4:51891759-51891851	chr4:51911069-51911174	-0.2	1.55E-06
MXE	MLLT10	+	chr10:21586293-21586348	chr10:21595330-21595440	-0.189	1.95E-06
MXE	SEC24B	+	chr4:109473003-109473186	chr4:109481676-109481781	-0.145	2.50E-06
MXE	RPAIN	+	chr17:5426235-5426299	chr17:5428070-5428211	-0.139	2.66E-06
MXE	MSRA	+	chr8:10207832-10207901	chr8:10245103-10245223	-0.15	2.77E-06
MXE	TNFSF13	+	chr17:7559623-7559702	chr17:7559850-7559893	-0.476	3.35E-06
MXE	TPT1-AS1	+	chr13:45379194-45379335	chr13:45383090-45383235	-0.547	3.37E-06
MXE	MSRA	+	chr8:10207832-10207901	chr8:10245103-10245223	-0.14	5.58E-06
MXE	SUZ12P1	+	chr17:30734896-30734943	chr17:30743300-30743369	-0.214	6.63E-06
MXE	SYTL2	-	chr11:85709330-85709500	chr11:85714412-85714507	-0.239	7.03E-06
MXE	SLCO4A1-AS1	-	chr20:62664923-62665497	chr20:62665859-62666010	-0.113	7.86E-06
MXE	C17orf80	+	chr17:73233219-73233325	chr17:73235475-73236995	-0.116	8.46E-06
MXE	TNFSF13	+	chr17:7559623-7559702	chr17:7559845-7559893	-0.405	9.38E-06
MXE	STRADA	-	chr17:63714005-63714108	chr17:63726637-63726695	-0.109	1.34E-05
MXE	SEPT2	+	chr2:241317498-241317599	chr2:241324408-241324662	-0.404	1.58E-05
MXE	SDCCAG3	-	chr9:136405117-136405202	chr9:136405904-136405978	-0.127	1.74E-05
MXE	DNM1	+	chr9:128224250-128224389	chr9:128226034-128226173	-0.438	2.11E-05
MXE	MFF	+	chr2:227342744-227342819	chr2:227347225-227347384	-0.134	2.19E-05
MXE	ETFA	-	chr15:76284364-76284460	chr15:76284804-76284927	-0.367	2.24E-05
MXE	MFF	+	chr2:227342744-227342819	chr2:227347225-227347384	-0.2	2.28E-05
MXE	GAS5	-	chr1:173865228-173865547	chr1:173866176-173866206	-0.198	2.31E-05
MXE	QKI	+	chr6:163455278-163455421	chr6:163534981-163535125	-0.168	3.05E-05
MXE	TPT1-AS1	+	chr13:45379194-45379335	chr13:45383090-45383235	-0.37	3.21E-05
MXE	YY1AP1	-	chr1:155679927-155680008	chr1:155680381-155680456	-0.342	3.79E-05
MXE	FOXK2	+	chr17:82582740-82582934	chr17:82584012-82584188	-0.175	3.95E-05
MXE	ISYNA1	-	chr19:18436972-18437105	chr19:18437598-18437760	-0.472	3.96E-05
MXE	LINC00630	+	chrX:102816991-102817082	chrX:102825992-102826169	-0.477	4.09E-05
MXE	TXNRD1	+	chr12:104290992-104291104	chr12:104309758-104309950	-0.697	4.20E-05
MXE	SUMF2	+	chr7:56074173-56074218	chr7:56074585-56074736	-0.12	4.97E-05
MXE	SH3GLB2	-	chr9:129012235-129012298	chr9:129014410-129014503	-0.113	5.31E-05

ATXN2
ATXN3
AUP1
AURKA
AURKB
B3GALNT1
B3GNTL1
B4GALNT1
B4GALT3
B4GALT4
BAG6
BAHD1
BAIAP2
BAX
BAZ1A
BAZ2A
BAZ2B
BBOF1
BBS1
BBS2
BBX
BCAS4
BCAT2
BCHE
BCKDK
BCL2L11
BCL2L12
BCL2L13
BCL2L2
BCL3
BCLAF1
BCOR
BCS1L
BDH2
BDNF-AS
BDP1
BEND6
BEST1
BICD1
BIRC5
BLOC1S1
BLOC1S5
BLOC1S6
BMP1
BMP2K
BNIP2
BORA
BORCS8-MEF2B
BPHL
BPTF
BRAT1
BRD7
BRD8
BRD9
BRF1
BROX
BRPF3
BTAF1
BTBD3
BTF3L4
BTN2A1
BTN2A2
BTN3A1
BTN3A2
BTN3A3
BZW2
C11orf1
C11orf49
C11orf54
C11orf57
C11orf65
C11orf74
C11orf80
C12orf4
C12orf65
C14orf159
C14orf166
C14orf79
C14orf93
C17orf53
C17orf62
C17orf80
C18orf54
C18orf8
C19orf12
C19orf60
C19orf66
C1orf112
C1orf159
C1orf162
C1orf35
C1orf43
C1orf50
C1orf52
C1QTNF1
C1QTNF6
C1RL
C20orf194
C21orf2
C21orf58
C21orf59
C21orf62-AS1
C2CD5
C2orf49
C2orf74
C2orf76
C3orf18
C4orf36
C5orf22
C5orf42
C6orf48
C6orf52
C7orf50
C8orf44
C8orf76
C9orf3
C9orf85
CA5B
CABIN1
CACNA2D1
CACNB3

MXE	H2AFY	-	chr5:135350822-135350913	chr5:135352945-135353045	0.155	2.56E-09
MXE	CENPO	+	chr2:24793851-24793965	chr2:24799674-24799844	0.222	3.30E-09
MXE	PRDM2	+	chr1:13773077-13773188	chr1:13778417-13782831	0.242	3.35E-09
MXE	SNHG17	-	chr20:38422091-38422241	chr20:38425934-38426052	0.256	5.21E-09
MXE	AUP1	-	chr2:74529131-74529282	chr2:74529361-74529499	0.241	6.67E-09
MXE	QKI	+	chr6:163534981-163535125	chr6:163561981-163562069	0.124	1.07E-08
MXE	CENPO	+	chr2:24793851-24793965	chr2:24799674-24799844	0.291	1.45E-08
MXE	AUP1	-	chr2:74529131-74529282	chr2:74529361-74529499	0.237	1.56E-08
MXE	ATG13	+	chr11:46630896-46630991	chr11:46632322-46632459	0.851	2.25E-08
MXE	RP11-421L21.3	+	chr1:101075394-101075716	chr1:101077362-101077508	0.439	3.74E-08
MXE	EPB41L2	-	chr6:130876673-130876775	chr6:130880143-130880206	0.244	4.51E-08
MXE	FBXO22	+	chr15:75904490-75904629	chr15:75913202-75913290	0.343	4.51E-08
MXE	DOCK9	-	chr13:98845328-98845372	chr13:98845923-98845991	0.55	4.87E-08
MXE	RP11-73M18.2	+	chr14:103574106-103574170	chr14:103587273-103587364	0.194	4.96E-08
MXE	SPAST	+	chr2:32098795-32098891	chr2:32114637-32114825	0.281	5.89E-08
MXE	SEC24B	+	chr4:109481676-109481781	chr4:109491326-109491407	0.23	6.93E-08
MXE	STAU1	-	chr20:49165996-49166285	chr20:49174194-49174269	0.153	8.71E-08
MXE	GAS5	-	chr1:173865856-173865894	chr1:173866760-173866796	0.229	1.36E-07
MXE	NDUFA3	+	chr19:54103113-54103188	chr19:54105933-54106011	0.116	1.38E-07
MXE	NOP56	+	chr20:2655933-2656034	chr20:2656400-2656549	0.117	1.38E-07
MXE	PPP4R1	-	chr18:9561979-9562075	chr18:9562921-9563046	0.15	1.60E-07
MXE	COMMD4	+	chr15:75338061-75338133	chr15:75338354-75338420	0.237	1.79E-07
MXE	PCBP1-AS1	-	chr2:70053730-70053792	chr2:70055713-70055910	0.505	1.79E-07
MXE	DCTN6	+	chr8:30164110-30164175	chr8:30175084-30175190	0.123	2.03E-07
MXE	RP11-33B1.1	+	chr4:119528739-119528789	chr4:119550378-119550443	0.639	2.06E-07
MXE	ASPH	-	chr8:61618977-61619019	chr8:61633682-61633727	0.21	2.91E-07
MXE	COMMD4	+	chr15:75338061-75338133	chr15:75338354-75338420	0.17	3.33E-07
MXE	USP45	-	chr6:99445796-99446463	chr6:99464603-99464747	0.271	4.26E-07
MXE	CCNYL1	+	chr2:207733946-207734047	chr2:207737410-207737446	0.386	4.44E-07
MXE	PCBP1-AS1	-	chr2:70053730-70053792	chr2:70055655-70055910	0.392	5.67E-07
MXE	PIGP	-	chr21:37069551-37069624	chr21:37072144-37072310	0.304	6.45E-07
MXE	MPHOSPH9	-	chr12:123226287-123226351	chr12:123227545-123227616	0.189	6.55E-07
MXE	PIGP	-	chr21:37069551-37069624	chr21:37072144-37072310	0.155	7.30E-07
MXE	SUZ12P1	+	chr17:30743300-30743369	chr17:30759490-30759539	0.242	8.81E-07
MXE	TAZ	+	chrX:154420031-154420094	chrX:154420211-154420264	0.272	9.79E-07
MXE	MPHOSPH9	-	chr12:123226287-123226351	chr12:123227462-123227616	0.117	9.96E-07
MXE	ARPC4	+	chr3:9797658-9797777	chr3:9800184-9800296	0.15	9.96E-07
MXE	PCBP1-AS1	-	chr2:70053730-70053792	chr2:70055713-70055910	0.382	1.18E-06
MXE	MPHOSPH9	-	chr12:123226287-123226351	chr12:123227462-123227572	0.18	1.39E-06
MXE	NBPF8	+	chr1:120447093-120447166	chr1:120447629-120447841	0.575	1.47E-06
MXE	HAGH	-	chr16:1819114-1819223	chr16:1819896-1820014	0.171	1.72E-06
MXE	COA1	-	chr7:43656032-43656153	chr7:43665657-43665722	0.198	1.72E-06
MXE	TMEM161B	-	chr5:88228444-88228528	chr5:88240812-88240916	0.238	1.82E-06
MXE	ASPH	-	chr8:61583943-61584029	chr8:61633682-61633727	0.178	1.90E-06
MXE	ANKRD36	+	chr2:97205939-97205968	chr2:97209680-97209709	0.596	1.97E-06
MXE	LRCH3	+	chr3:197854391-197854445	chr3:197858833-197858905	0.377	2.10E-06
MXE	ALG13	+	chrX:111689086-111689197	chrX:111708026-111708393	0.177	2.24E-06
MXE	HNRNPH1	-	chr5:179620891-179621397	chr5:179623036-179623164	0.114	2.46E-06
MXE	MCM3	-	chr6:52266074-52266144	chr6:52266610-52266696	0.113	2.57E-06
MXE	KIF21A	-	chr12:39315931-39315970	chr12:39318072-39318201	0.172	2.61E-06
MXE	VEGFA	+	chr6:43778459-43778536	chr6:43782010-43782087	0.25	2.69E-06
MXE	FAM208B	+	chr10:5714134-5714207	chr10:5717658-5717750	0.298	2.88E-06
MXE	QKI	+	chr6:163534981-163535125	chr6:163561981-163562069	0.124	2.92E-06
MXE	IFT88	+	chr13:20625749-20625849	chr13:20631015-20631102	0.315	2.93E-06
MXE	THUMPD2	-	chr2:39768900-39769093	chr2:39769709-39770119	0.133	2.96E-06
MXE	PPIL3	-	chr2:200882341-200882435	chr2:200885261-200885343	0.104	3.05E-06
MXE	PPRC1	+	chr10:102143044-102143098	chr10:102144249-102144307	0.231	3.13E-06
MXE	MPV17	-	chr2:27312993-27313074	chr2:27322447-27322522	0.188	3.87E-06
MXE	PCBP1-AS1	-	chr2:70053730-70053792	chr2:70059642-70059680	0.407	4.02E-06
MXE	LUC7L3	+	chr17:50737286-50737479	chr17:50738140-50738199	0.106	4.40E-06
MXE	NSMF	-	chr9:137452735-137452819	chr9:137453055-137453180	0.146	4.68E-06
MXE	FAM219B	-	chr15:74905153-74905231	chr15:74906277-74906365	0.451	4.92E-06
MXE	HMGN2	+	chr1:26473482-26473527	chr1:26473702-26473817	0.101	5.39E-06
MXE	CFLAR	+	chr2:201140356-201140439	chr2:201145377-201145432	0.193	5.62E-06
MXE	LUC7L3	+	chr17:50737286-50737479	chr17:50738136-50738199	0.109	5.64E-06
MXE	HLA-C	-	chr6:31270209-31270485	chr6:31271072-31271331	0.117	5.70E-06
MXE	PCBP1-AS1	-	chr2:70053730-70053792	chr2:70083537-70083687	0.148	5.92E-06
MXE	PCBP1-AS1	-	chr2:70053730-70053792	chr2:70083506-70083687	0.166	6.87E-06
MXE	SLC12A4	-	chr16:67954642-67954773	chr16:67963464-67963559	0.187	6.91E-06
MXE	RAD1	-	chr5:34911553-34911812	chr5:34913469-34913578	0.137	8.28E-06
MXE	ARHGEF7	+	chr13:111205288-111205373	chr13:111209871-111210002	0.219	1.07E-05
MXE	ARIH2	+	chr3:48924717-48924839	chr3:48945135-48945181	0.166	1.09E-05
MXE	ERBIN	+	chr5:66068876-66069020	chr5:66072168-66072291	0.469	1.14E-05
MXE	ALG13	+	chrX:111689086-111689197	chrX:111708964-111709048	0.279	1.16E-05
MXE	PPIL3	-	chr2:200882341-200882435	chr2:200885261-200885367	0.153	1.17E-05
MXE	MPV17	-	chr2:27312993-27313109	chr2:27322447-27322522	0.176	1.28E-05
MXE	ARHGAP11B	+	chr15:30638745-30638820	chr15:30644638-30644702	0.272	1.34E-05
MXE	NUTM2A-AS1	-	chr10:87246114-87246212	chr10:87288441-87288495	0.172	1.51E-05
MXE	GAS5	-	chr1:173865228-173865282	chr1:173865470-173865547	0.121	1.59E-05
MXE	KNSTRN	+	chr15:40383227-40383322	chr15:40386361-40386494	0.184	2.02E-05
MXE	ANKRD36	+	chr2:97198585-97198658	chr2:97200452-97200525	0.529	2.35E-05
MXE	RAPGEF1	-	chr9:131604930-131605188	chr9:131619050-131619209	0.455	2.38E-05
MXE	CFLAR	+	chr2:201140356-201140439	chr2:201149002-201149052	0.163	2.56E-05
MXE	NBPF9	-	chr1:149058924-149059097	chr1:149062092-149062265	0.221	2.56E-05
MXE	ACLY	-	chr17:41896619-41896649	chr17:41897748-41897839	0.174	2.86E-05
MXE	TNC	-	chr9:115048259-115048532	chr9:115059729-115060002	0.464	3.13E-05
MXE	DGUOK	+	chr2:73946718-73946906	chr2:73957124-73957240	0.163	3.19E-05
MXE	ADAP2	+	chr17:30926826-30926918	chr17:30931888-30931968	0.411	3.40E-05
MXE	EIF4A2	+	chr3:186784961-186785101	chr3:186786163-186786273	0.108	3.42E-05
MXE	KNSTRN	+	chr15:40383227-40383322	chr15:40386404-40386494	0.19	3.93E-05
MXE	WNK1	+	chr12:879572-880031	chr12:880720-880999	0.178	3.96E-05
MXE	POP5	-	chr12:120579773-120579923	chr12:120580995-120581257	0.117	3.96E-05
MXE	C8orf76	-	chr8:123231299-123231757	chr8:123239048-123239144	0.112	4.97E-05
MXE	TTC13	-	chr1:230936157-230936264	chr1:230939385-230939496	0.149	4.98E-05
MXE	TNC	-	chr9:115048259-115048532	chr9:115059729-115060002	0.416	5.63E-05
MXE	KRTCAP2	-	chr1:155169790-155169857	chr1:155172564-155172892	0.12	6.32E-05
MXE	GAS5	-	chr1:173865509-173865547	chr1:173866527-173866567	0.214	6.74E-05
MXE	HPS4	-	chr22:26465454-26465551	chr22:26466050-26466104	0.109	7.72E-05
MXE	HNRNPD	-	chr4:82355303-82355401	chr4:82356536-82356683	0.127	8.41E-05
MXE	LINC01184	-	chr5:127956803-127956921	chr5:128061143-128061244	0.313	8.49E-05
MXE	PGAP2	+	chr11:3823612-3823740	chr11:3823882-3824135	0.166	9.22E-05
MXE	DCUN1D4	+	chr4:51891759-51891851	chr4:51899269-51899378	0.271	9.22E-05
MXE	PRKRIP1	+	chr7:102395976-102396537	chr7:102397619-102397698	0.196	0.0001029012069
MXE	ZGRF1	-	chr4:112589723-112589874	chr4:112603523-112603697	0.206	0.0001070343972
MXE	PIGP	-	chr21:37067261-37067380	chr21:37072144-37072310	0.141	0.0001179796604
MXE	GAMT	-	chr19:1399523-1399587	chr19:1399792-1399938	0.185	0.0001202100974
MXE	ARIH2	+	chr3:48922747-48922811	chr3:48945135-48945181	0.183	0.0001227177574
MXE	MDM4	+	chr1:204526359-204526434	chr1:204537458-204537497	0.286	0.0001261302674
MXE	NBPF12	+	chr1:146987953-146988126	chr1:146989573-146989746	0.198	0.0001456783967
MXE	DGUOK	+	chr2:73946718-73946906	chr2:73950584-73950732	0.172	0.0001474551712
MXE	EFCAB13	+	chr17:47324453-47324523	chr17:47326225-47326387	0.192	0.0001479288917
MXE	SUN1	+	chr7:843340-843520	chr7:848417-848615	0.258	0.0001492596994
MXE	STRN3	-	chr14:30913523-30913657	chr14:30929200-30929311	0.128	0.0001502871092
MXE	TEX22	+	chr14:105445417-105445511	chr14:105449358-105449409	0.156	0.0001522249229
MXE	ZNF559-ZNF17	+	chr19:9324694-9324780	chr19:9338493-9338582	0.163	0.0001552386994
MXE	OSBPL6	+	chr2:178339671-178339764	chr2:178344285-178344360	0.48	0.0001659601603
MXE	PICALM	-	chr11:85996825-85996929	chr11:86000642-86000779	0.126	0.0001677012779
MXE	HLA-A	+	chr6:29944121-29944397	chr6:29945233-29945281	0.145	0.0001835004963
MXE	DLG1	-	chr3:197149742-197149796	chr3:197161639-197161738	0.152	0.000188737718
MXE	AGAP4	-	chr10:45834015-45834116	chr10:45841652-45841687	0.708	0.0001944930615
MXE	RBM6	+	chr3:50048244-50048319	chr3:50054334-50054395	0.131	0.000205403459

MXE	P4HA1	-	chr10:73043886-73043957	chr10:73044980-73045051	-0.157	5.46E-05	CACNB4
MXE	ARHGEF40	+	chr14:21087000-21087105	chr14:21087319-21087463	-0.249	6.58E-05	CACTIN
MXE	FRG1CP	-	chr20:28588642-28588757	chr20:28591393-28591519	-0.364	6.65E-05	CAD
MXE	RABGEF1	+	chr7:66739995-66740792	chr7:66771882-66772078	-0.192	7.65E-05	CADM2
MXE	FAM118B	+	chr11:126234994-126235087	chr11:126240791-126241044	-0.182	7.70E-05	CALU
MXE	FOXM1	-	chr12:2865354-2865399	chr12:2866392-2866521	-0.146	7.70E-05	CAMK1
MXE	GAS5	-	chr1:173865470-173865547	chr1:173866527-173866567	-0.194	8.27E-05	CAMK2D
MXE	ELP2	+	chr18:36138794-36138872	chr18:36141136-36141201	-0.137	8.74E-05	CAMK2G
MXE	TPT1-AS1	+	chr13:45379925-45380001	chr13:45383090-45383235	-0.293	8.84E-05	CAMTA1
MXE	FAM45A	+	chr10:119123468-119123569	chr10:119129514-119129622	-0.327	8.86E-05	CAMTA2
MXE	SS18	-	chr18:26080304-26080423	chr18:26084051-26084175	-0.557	9.01E-05	CANT1
MXE	TPT1-AS1	+	chr13:45379925-45380001	chr13:45383090-45383235	-0.308	9.27E-05	CAPN7
MXE	NDE1	+	chr16:15687374-15687511	chr16:15691143-15691323	-0.145	9.27E-05	CAPRIN2
MXE	EMILIN2	+	chr18:2884963-2885139	chr18:2890560-2892486	-0.309	9.76E-05	CARD19
MXE	PVT1	+	chr8:127939507-127939676	chr8:127983903-127984204	-0.137	0.0001007016067	CARD8
MXE	RNF146	+	chr6:127280515-127280604	chr6:127285207-127285348	-0.284	0.0001091448679	CARF
MXE	NSRP1	+	chr17:30118079-30118173	chr17:30163066-30163162	-0.106	0.0001113518188	CARM1
MXE	FAM3A	-	chrX:154511847-154511871	chrX:154512822-154512936	-0.138	0.0001121731905	CARS2
MXE	DPH5	-	chr1:100995109-100995149	chr1:101001466-101001544	-0.167	0.0001213418567	CASC3
MXE	GUSBP11	-	chr22:23695172-23695517	chr22:23700725-23700845	-0.284	0.0001265450782	CASC4
MXE	DLEU1	+	chr13:50433412-50433550	chr13:50527865-50528064	-0.517	0.0001288175153	CASK
MXE	UGGT2	-	chr13:95859590-95859675	chr13:95860787-95860883	-0.253	0.0001298389853	CASP4
MXE	FIP1L1	+	chr4:53399729-53399839	chr4:53452919-53453133	-0.103	0.0001310168527	CASP8
MXE	LARP1	+	chr5:154793594-154793723	chr5:154793799-154794000	-0.104	0.0001568303725	CAST
MXE	SS18	-	chr18:26080304-26080423	chr18:26082362-26082528	-0.419	0.0001659601603	CATSPER2
MXE	DST	-	chr6:56598475-56598709	chr6:56600068-56600221	-0.19	0.0001855333334	CBLB
MXE	TPT1-AS1	+	chr13:45379194-45380001	chr13:45383090-45383235	-0.377	0.0001881439659	CBWD1
MXE	ZGRF1	-	chr4:112603523-112603697	chr4:112606007-112606091	-0.197	0.0001962090093	CBWD5
MXE	HDAC11	+	chr3:13498512-13498555	chr3:13500712-13500789	-0.384	0.0001997038677	CBY1
MXE	RP11-175P13.3	-	chr12:100177042-100177120	chr12:100180552-100180716	-0.478	0.0002414008361	CC2D1B
MXE	GK	+	chrX:30677374-30677452	chrX:30691122-30691199	-0.43	0.0002590266131	CC2D2A
MXE	PSMA4	+	chr15:78542176-78542219	chr15:78542482-78542645	-0.115	0.0002604984889	CCAR2
MXE	SH3D19	-	chr4:151147921-151148186	chr4:151149499-151149561	-0.154	0.0002641670792	CCDC125
MXE	PCBP1-AS1	-	chr2:70055713-70055910	chr2:70059642-70059680	-0.379	0.0002725051906	CCDC136
MXE	NAT1	+	chr8:18212390-18212774	chr8:18216876-18216994	-0.582	0.0002815207596	CCDC138
MXE	RCC1	+	chr1:28508829-28508901	chr1:28516724-28516867	-0.187	0.0002881438691	CCDC14
MXE	DONSON	-	chr21:33583487-33583666	chr21:33584589-33584768	-0.122	0.0002959732077	CCDC148
MXE	CALU	+	chr7:128754261-128754455	chr7:128754528-128754722	-0.125	0.0002985715716	CCDC15
MXE	TKFC	+	chr11:61335839-61335937	chr11:61336203-61336390	-0.446	0.0003061337011	CCDC150
MXE	PDGFRA	+	chr4:54258756-54258817	chr4:54261094-54261412	-0.168	0.0003061919067	CCDC159
MXE	NQO2	+	chr6:3003668-3003789	chr6:3004494-3004651	-0.629	0.0003073158693	CCDC18
MXE	SLCO5A1	-	chr8:69738039-69738204	chr8:69755423-69755641	-0.288	0.000307498296	CCDC183
MXE	TPT1-AS1	+	chr13:45379194-45380001	chr13:45383090-45383235	-0.397	0.0003101837007	CCDC189
MXE	RP11-421L21.3	+	chr1:101075275-101075394	chr1:101075394-101075716	-0.198	0.0003131589947	CCDC18-AS1
MXE	CD27-AS1	-	chr12:6448639-6448737	chr12:6450340-6450980	-0.223	0.0003131589947	CCDC24
MXE	PTTG1IP	-	chr21:44856192-44856364	chr21:44861162-44861271	-0.116	0.0003146550775	CCDC57
MXE	ANKRD11	-	chr16:89305205-89305344	chr16:89316932-89317078	-0.148	0.0003367841868	CCDC66
MXE	AGTPBP1	-	chr9:85681267-85681335	chr9:85692688-85692813	-0.124	0.0003822624243	CCDC7
MXE	B3GNTL1	-	chr17:82965650-82965746	chr17:83005114-83005210	-0.181	0.000398856445	CCDC88A
MXE	SUGP2	-	chr19:18995143-18995280	chr19:19001612-19001674	-0.153	0.0004015508016	CCDC90B
MXE	OFD1	+	chrX:13763744-13763855	chrX:13767126-13767284	-0.138	0.0004426164455	CCDC91
MXE	ELOVL5	-	chr6:53290094-53290189	chr6:53290400-53290535	-0.319	0.0004536946538	CCDC92
MXE	STAG3L5P-PVRI(	+	chr7:100349798-100349887	chr7:100351251-100351410	-0.142	0.0004594839116	CCHCR1
MXE	ZCWPW2	+	chr3:28492126-28492173	chr3:28514063-28514122	-0.527	0.0004781924829	CCM2
MXE	GALNT7	+	chr4:173297794-173297977	chr4:173298114-173298297	-0.185	0.0004884841784	CCNC
MXE	CENPI	+	chrX:101098443-101098539	chrX:101099808-101100065	-0.149	0.0005529783214	CCNE1
MXE	LINC00894	+	chrX:149942183-149942329	chrX:149943650-149943762	-0.445	0.0005601986855	CCNF
MXE	MEF2A	+	chr15:99671322-99671454	chr15:99671519-99671657	-0.174	0.0005770143277	CCNI
MXE	FAM221A	+	chr7:23692182-23692253	chr7:23698191-23698299	-0.105	0.0006007034903	CCNJL
MXE	DNAH6	+	chr2:84528903-84529166	chr2:84544232-84544500	-0.408	0.0006161325847	CCNK
MXE	ST3GAL5	-	chr2:85851263-85851703	chr2:85852859-85853099	-0.153	0.0006198488713	CCNL1
MXE	TCF7L2	+	chr10:113159919-113159992	chr10:113160618-113160691	-0.684	0.0007076008699	CCNT1
MXE	TFDP1	+	chr13:113610995-113611062	chr13:113631622-113631744	-0.13	0.0007625425829	CCNT2
MXE	TADA2A	+	chr17:37426949-37427009	chr17:37437737-37437922	-0.353	0.0007720291368	CCNYL1
MXE	ACAA1	-	chr3:38129289-38129388	chr3:38131595-38131638	-0.251	0.0007803381414	CCP110
MXE	TRMT2B	-	chrX:101042041-101042312	chrX:101051643-101051733	-0.147	0.0008186054836	CCS
MXE	ST3GAL5	-	chr2:85851263-85851703	chr2:85852859-85853091	-0.148	0.0008362175432	CCSER2
MXE	FAM122C	+	chrX:134847882-134847971	chrX:134849166-134849228	-0.69	0.0008449722938	CD274
MXE	NCK2	+	chr2:105855047-105855289	chr2:105881327-105882049	-0.129	0.0008495928534	CD27-AS1
MXE	ERCC1	-	chr19:45421177-45421393	chr19:45423269-45423381	-0.124	0.0008495928534	CD320
MXE	CLIP1	-	chr12:122347374-122347479	chr12:122351110-122351143	-0.208	0.0009627121469	CD46
MXE	TXLNA	+	chr1:32184524-32184616	chr1:32187953-32188124	-0.196	0.001031064798	CD99L2
MXE	TACC3	+	chr4:1723727-1723870	chr4:1727707-1728787	-0.115	0.001047468692	CDC14B
MXE	ATP9B	+	chr18:79154503-79154555	chr18:79176812-79176907	-0.167	0.001097782761	CDC16
MXE	MPPE1	-	chr18:11897525-11897620	chr18:11905737-11905953	-0.218	0.0011580361	CDC25C
MXE	PMF1-BGLAP	+	chr1:156233627-156233728	chr1:156236287-156236483	-0.129	0.001301445097	CDC42SE1
MXE	UGGT2	-	chr13:95856157-95856340	chr13:95859590-95859675	-0.204	0.001400276335	CDC45
MXE	TMBIM6	+	chr12:49741956-49742280	chr12:49743416-49743565	-0.442	0.001459892005	CDCA3
MXE	GTPBP10	+	chr7:90355085-90355230	chr7:90372154-90372228	-0.185	0.001463632539	CDCA7
MXE	ST3GAL5	-	chr2:85851263-85851703	chr2:85852859-85853000	-0.212	0.001463632539	CDCA7L
MXE	ARMCX5-GPRAS	+	chrX:102601463-102601509	chrX:102601879-102601977	-0.371	0.001699714909	CDK10
MXE	TFDP1	+	chr13:113610995-113611062	chr13:113623179-113623286	-0.147	0.001750191041	CDK11A
MXE	SDHC	+	chr1:161323613-161323670	chr1:161328395-161328497	-0.213	0.001810025378	CDK13
MXE	NUP88	-	chr17:5388801-5388960	chr17:5391560-5391662	-0.12	0.001837403073	CDK16
MXE	CTNND1	+	chr11:57789036-57789155	chr11:57791384-57791673	-0.185	0.001855811067	CDK20
MXE	LINC00894	+	chrX:149941789-149941847	chrX:149943650-149943762	-0.4	0.002052545047	CDK4
MXE	MEF2A	+	chr15:99671322-99671454	chr15:99671519-99671657	-0.169	0.002084043817	CDK5RAP2
MXE	LINC00630	+	chrX:102816991-102817082	chrX:102825992-102826169	-0.413	0.002188454797	CDK5RAP3
MXE	PIGQ	+	chr16:580182-580263	chr16:580857-580972	-0.13	0.002220645897	CDK7
MXE	PXN	-	chr12:120215559-120215661	chr12:120216272-120217116	-0.148	0.002279950226	CDKL1
MXE	KIF21A	-	chr12:39311414-39311553	chr12:39319905-39320013	-0.432	0.002295330497	CDKL3
MXE	ZC3H18	+	chr16:88608933-88609051	chr16:88611267-88611536	-0.152	0.00231520176	CDKN2AIP
MXE	TMEM63B	+	chr6:44150563-44150629	chr6:44151845-44152008	-0.191	0.002658984414	CEBPZOS
MXE	USP15	+	chr12:62325871-62325933	chr12:62349220-62349307	-0.168	0.002700201888	CELF1
MXE	ACTR10	+	chr14:58215204-58215284	chr14:58219693-58219729	-0.106	0.002706536064	CELSR3
MXE	TFPI	-	chr2:187513615-187513646	chr2:187529363-187529485	-0.201	0.002783607971	CENPA
MXE	GBA	-	chr1:155240629-155240717	chr1:155241085-155241180	-0.124	0.002845465673	CENPE
MXE	PRKDC	-	chr8:47893138-47893387	chr8:47897160-47897294	-0.12	0.002892471462	CENPI
MXE	FAM228B	+	chr2:24139369-24139450	chr2:24146929-24147086	-0.281	0.00294268215	CENPJ
MXE	ARNTL2	+	chr12:27370128-27370230	chr12:27376345-27376387	-0.224	0.002977500621	CENPK
MXE	DLG1	-	chr3:197075836-197075870	chr3:197076585-197076682	-0.212	0.003045392827	CENPO
MXE	TANGO2	+	chr22:20055942-20056013	chr22:20063337-20063442	-0.397	0.00308776571	CENPT
MXE	NUDT13	+	chr10:73125117-73125243	chr10:73125397-73125509	-0.4	0.003352362165	CENPU
MXE	DLG1	-	chr3:197075836-197075870	chr3:197076585-197076685	-0.165	0.003356174234	CENPV
MXE	ZGRF1	-	chr4:112603523-112603697	chr4:112606007-112606091	-0.193	0.003456374552	CENPX
MXE	FEZ2	-	chr2:36555682-36555748	chr2:36558437-36558513	-0.108	0.003486529079	CEP152
MXE	RWDD3	+	chr1:95244210-95244698	chr1:95246541-95246657	-0.166	0.003664600071	CEP164
MXE	FASTKD1	-	chr2:169538012-169538141	chr2:169540050-169540179	-0.128	0.003836887588	CEP192
MXE	RP11-507M3.1	+	chr2:24139369-24139450	chr2:24146929-24147086	-0.408	0.004156507974	CEP41
MXE	PGAP2	+	chr11:3811249-3811424	chr11:3823612-3823740	-0.123	0.004195565611	CEP57L1
MXE	CARF	+	chr2:202924356-202924415	chr2:202941859-202941980	-0.149	0.004239967383	CEP63
MXE	TRMT2B	-	chrX:101051250-101051366	chrX:101051643-101051733	-0.474	0.004271075024	CEP70
MXE	CCNJL	-	chr5:160269462-160269544	chr5:160276268-160276412	-0.321	0.004337264385	CEP72
MXE	JMJD4	-	chr1:227733413-227733681	chr1:227733906-227734032	-0.213	0.004384953289	CEP78
MXE	DGKZ	+	chr11:46368001-46368079	chr11:46369493-46369550	-0.279	0.004410053941	CEP85L
MXE	OSBPL1A	-	chr18:24341546-24341658	chr18:24366891-24366966	-0.154	0.004423317853	CERS5
MXE	ANKRD27	-	chr19:32607634-32607832	chr19:32615657-32615780	-0.209	0.004476786939	CFAP44
MXE	HSF4	+	chr16:67167474-67167599	chr16:67167823-67167947	-0.201	0.004540987033	CFLAR
MXE	CTNND1	+	chr11:57789036-57789155	chr11:57791384-57791673	-0.219	0.004696136325	CHAF1A
MXE	ZNF559-ZNF177	+	chr19:9338493-9338582	chr19:9339192-9339319	-0.188	0.004810240806	CHD3
MXE	ANKMY1	-	chr2:240509347-240509455	chr2:240511860-240512001	-0.477	0.005009216857	CHD9

MXE	BTF3L4	+	chr1:52064824-52064938	chr1:52083339-52083541	0.158	0.0002261884385
MXE	IMMP1L	-	chr11:31460625-31460714	chr11:31473723-31473786	0.349	0.0002261884385
MXE	BCL2L13	+	chr22:17683213-17683321	chr22:17702242-17702386	0.148	0.0002387901891
MXE	DGUOK	+	chr2:73946718-73946906	chr2:73957124-73957240	0.158	0.0002387901891
MXE	RFWD2	-	chr1:176116623-176116681	chr1:176135009-176135086	0.237	0.0002536424228
MXE	C1orf50	+	chr1:42767508-42767624	chr1:42773562-42773649	0.118	0.0002590266131
MXE	PMS2	-	chr7:5982823-5982991	chr7:5986758-5987620	0.113	0.0002590266131
MXE	NFIC	+	chr19:3381711-3382243	chr19:3425105-3425177	0.258	0.0002641670792
MXE	GSAP	-	chr7:77329332-77329391	chr7:77330238-77330367	0.103	0.0002698227188
MXE	DUXAP10	-	chr14:19307020-19307111	chr14:19331481-19331617	0.469	0.0002766239401
MXE	MTCL1	+	chr18:8806892-8807060	chr18:8812978-8813233	0.212	0.0002977314871
MXE	HNRNPH1	-	chr5:179621241-179621397	chr5:179623036-179623164	0.106	0.0002986804302
MXE	TIAL1	-	chr10:119580470-119580846	chr10:119581921-119582009	0.126	0.0003079895426
MXE	UBA2	+	chr19:34430575-34430659	chr19:34431860-34431931	0.116	0.0003332621248
MXE	MFF	+	chr2:227347225-227347384	chr2:227352513-227352573	0.182	0.0003367841868
MXE	IFRD1	+	chr7:112450507-112450782	chr7:112455762-112455867	0.193	0.000362523698
MXE	GAS5	-	chr1:173865856-173865894	chr1:173866527-173866567	0.25	0.0003771806936
MXE	SLC26A11	+	chr17:80220918-80221115	chr17:80221547-80221794	0.399	0.0003818203054
MXE	HJURP	-	chr2:233844204-233844283	chr2:233849762-233849859	0.145	0.0003822624243
MXE	GLS	+	chr2:190913192-190913364	chr2:190920214-190920248	0.17	0.0003834698295
MXE	NVL	-	chr1:224232088-224232182	chr1:224232894-224232972	0.615	0.000384726908
MXE	ZGRF1	-	chr4:112589723-112589874	chr4:112603523-112603697	0.213	0.0003964893019
MXE	ZNRD1ASP	-	chr6:30021666-30021761	chr6:30034907-30035110	0.253	0.0004121135196
MXE	SOD1	+	chr21:31663789-31663886	chr21:31666448-31666518	0.107	0.000440024471
MXE	SLMAP	+	chr3:57907883-57908006	chr3:57909075-57909150	0.2	0.000440024471
MXE	CCDC15	+	chr11:124993168-124993243	chr11:125003866-125003959	0.188	0.0004781924829
MXE	ABI1	-	chr10:26755654-26755741	chr10:26759061-26759238	0.17	0.0004781924829
MXE	MAPT	+	chr17:45971858-45971945	chr17:45978374-45978440	0.149	0.0005296925756
MXE	GIT2	-	chr12:109947255-109947504	chr12:109951211-109951316	0.184	0.0005428233416
MXE	ATAT1	+	chr6:30642767-30643011	chr6:30645894-30645974	0.4	0.0005709668944
MXE	C11orf80	+	chr11:66788159-66788269	chr11:66796280-66796364	0.206	0.0005733001896
MXE	RPS6KB2	+	chr11:67431367-67431515	chr11:67432352-67432492	0.107	0.0005752714278
MXE	ACOX1	-	chr17:75960214-75960375	chr17:75973624-75973785	0.184	0.0006007034903
MXE	ESYT2	-	chr7:158737047-158737179	chr7:158739022-158739121	0.124	0.0006139817884
MXE	AGPAT4	-	chr6:161154148-161154310	chr6:161165576-161165713	0.108	0.0006171062672
MXE	ZGRF1	-	chr4:112589723-112589874	chr4:112603523-112603697	0.211	0.0006512876139
MXE	LSM14B	+	chr20:62126225-62126439	chr20:62127584-62127701	0.276	0.000694920693
MXE	RPS6KB2	+	chr11:67431367-67431515	chr11:67432355-67432492	0.108	0.0007294478413
MXE	NINL	-	chr20:25469990-25470095	chr20:25476042-25477089	0.11	0.0007599285581
MXE	HLA-A	+	chr6:29943267-29943543	chr6:29944121-29944397	0.135	0.0007623981442
MXE	PKD1P6-NPIPP	-	chr16:15118925-15119054	chr16:15125690-15125807	0.146	0.0007720928532
MXE	NSMF	-	chr9:137453055-137453180	chr9:137453730-137453820	0.157	0.0007803381414
MXE	CENPO	+	chr2:24793851-24793965	chr2:24799674-24799844	0.114	0.0008017307644
MXE	SFI1	+	chr22:31604308-31604404	chr22:31604868-31604945	0.276	0.0008459114573
MXE	ELOVL5	-	chr6:53287861-53287942	chr6:53290094-53290189	0.217	0.0009267743125
MXE	KYAT1	-	chr9:128846679-128846859	chr9:128847432-128847643	0.365	0.0009655421884
MXE	HJURP	-	chr2:233845727-233845820	chr2:233847396-233847461	0.167	0.0009825921946
MXE	ABI1	-	chr10:26755654-26755741	chr10:26759061-26759235	0.179	0.001001354063
MXE	TFRC	-	chr3:196075158-196075360	chr3:196077063-196077122	0.101	0.001018090401
MXE	FBXL2	+	chr3:33297868-33297962	chr3:33364624-33364719	0.214	0.001031064798
MXE	MYEF2	-	chr15:48151472-48151571	chr15:48151873-48151942	0.163	0.001055792768
MXE	HMG20B	+	chr19:3575539-3575660	chr19:3576260-3576307	0.118	0.001067898129
MXE	FAM219B	-	chr15:74904981-74905059	chr15:74905153-74905231	0.127	0.001096382874
MXE	FAM49B	-	chr8:129904498-129904585	chr8:129970942-129970995	0.151	0.001100968158
MXE	NUP50	+	chr22:45170987-45171177	chr22:45171599-45171683	0.179	0.001119222119
MXE	DYNC1I2	+	chr2:171690146-171690263	chr2:171692776-171692894	0.13	0.001121487013
MXE	C17orf62	-	chr17:82446622-82446696	chr17:82449169-82449292	0.208	0.001134691147
MXE	MTHFD1L	+	chr6:150887841-150887981	chr6:150905649-150905761	0.172	0.001134691147
MXE	PSMA3-AS1	-	chr14:58273978-58274010	chr14:58285590-58285756	0.377	0.001136202548
MXE	MDC1	-	chr6:30707565-30708357	chr6:30711411-30711504	0.13	0.001143797693
MXE	POLE2	-	chr14:49646569-49646694	chr14:49646993-49647092	0.387	0.001174947873
MXE	SLC9B2	-	chr4:103057800-103057971	chr4:103066326-103066507	0.136	0.001204409351
MXE	CAST	+	chr5:96722638-96722698	chr5:96729152-96729209	0.177	0.00121263946
MXE	ZNF480	+	chr19:52300393-52300484	chr19:52314152-52314279	0.278	0.001318100309
MXE	CNOT2	+	chr12:70285399-70285586	chr12:70294236-70294294	0.41	0.001318100309
MXE	DEK	-	chr6:18258303-18258405	chr6:18263842-18263996	0.121	0.001323759902
MXE	SNX8	-	chr7:2271849-2271971	chr7:2278099-2278305	0.111	0.001378247514
MXE	FBXL4	-	chr6:98917373-98917719	chr6:98934761-98934879	0.106	0.001380734764
MXE	CASK	-	chrX:41555599-41555635	chrX:41557031-41557100	0.134	0.001407486282
MXE	ACSL3	+	chr2:222887829-222887888	chr2:222900673-222900780	0.18	0.001419689146
MXE	TFDP2	-	chr3:142005440-142005544	chr3:142101734-142101841	0.124	0.00143780418
MXE	KLHDC1	+	chr14:49725685-49725769	chr14:49728925-49729009	0.409	0.001455552303
MXE	CUEDC2	-	chr10:102423797-102423859	chr10:102423995-102424178	0.107	0.001455705933
MXE	TMEM184B	-	chr22:38245934-38246100	chr22:38247769-38248019	0.12	0.001647547866
MXE	SDCCAG8	+	chr1:243304712-243304777	chr1:243307988-243308177	0.157	0.001679077248
MXE	COL27A1	+	chr9:114245865-114245910	chr9:114250614-114250668	0.486	0.001750191041
MXE	C1orf43	-	chr1:154213892-154213946	chr1:154214456-154214574	0.101	0.001794772579
MXE	ARPC1B	+	chr7:99376432-99376574	chr7:99377357-99377455	0.136	0.001794772579
MXE	HMGN2	+	chr1:26473482-26473527	chr1:26473702-26473732	0.132	0.001794772579
MXE	BCL2L13	+	chr22:17688985-17689142	chr22:17696140-17696210	0.108	0.001814591819
MXE	DPH5	-	chr1:100995109-100995149	chr1:101001466-101001587	0.104	0.001814591819
MXE	ZMAT1	-	chrX:101887197-101887304	chrX:101895813-101895882	0.262	0.001875902704
MXE	DGUOK	+	chr2:73938909-73939022	chr2:73957124-73957240	0.133	0.00191291694
MXE	PHF7	+	chr3:52422221-52422338	chr3:52422759-52422881	0.14	0.00222168987
MXE	TMEM161A	-	chr19:19133129-19133210	chr19:19134783-19134887	0.142	0.002295330497
MXE	XKR9	+	chr8:70674817-70674899	chr8:70680780-70681330	0.185	0.002440740444
MXE	ANKRD20A11P	-	chr21:13915186-13915409	chr21:13916746-13916958	0.288	0.002470228289
MXE	EPB41L2	-	chr6:130870327-130870381	chr6:130876673-130876775	0.306	0.002539822157
MXE	CACNB4	-	chr2:151860710-151860820	chr2:151870530-151870611	0.462	0.002614877735
MXE	OGDH	+	chr7:44645326-44645518	chr7:44647656-44647759	0.168	0.002859687472
MXE	RELA	-	chr11:65661686-65661835	chr11:65661936-65662088	0.167	0.002859687472
MXE	STAG3L5P-PVF	+	chr7:100338772-100338889	chr7:100345864-100345968	0.111	0.00296195312
MXE	DPY19L3	+	chr19:32467473-32467626	chr19:32468730-32468813	0.123	0.002975638097
MXE	EED	+	chr11:86257514-86257596	chr11:86264171-86264263	0.151	0.003132359724
MXE	ANAPC16	+	chr10:72223887-72224056	chr10:72230365-72230440	0.11	0.003363953235
MXE	AP2A2	+	chr11:977094-977224	chr11:981197-981299	0.149	0.003517278317
MXE	SCLY	+	chr2:238068064-238068165	chr2:238091217-238091254	0.11	0.003682682
MXE	C17orf62	-	chr17:82447579-82447621	chr17:82449173-82449292	0.236	0.003734037699
MXE	FAM122B	-	chrX:134781820-134781917	chrX:134785885-134785947	0.173	0.003764948527
MXE	ARPC1B	+	chr7:99376432-99376574	chr7:99377337-99377455	0.114	0.003775870552
MXE	GRB10	-	chr7:50732271-50732368	chr7:50755886-50756056	0.133	0.003775870552
MXE	POMGNT1	-	chr1:46194552-46194651	chr1:46194843-46194961	0.121	0.003915883207
MXE	POP5	-	chr12:120579513-120579597	chr12:120581114-120581257	0.18	0.004097329756
MXE	LRRCC1	+	chr8:85115374-85115584	chr8:85124791-85124939	0.137	0.004174444603
MXE	NUDT17	+	chr1:145846012-145846196	chr1:145846597-145846690	0.18	0.004181133627
MXE	DLG1	-	chr3:197076585-197076685	chr3:197081050-197081117	0.12	0.004195565611
MXE	PIGF	-	chr2:46588074-46588179	chr2:46591802-46591925	0.224	0.004271075024
MXE	PDE9A	+	chr21:42731769-42731949	chr21:42732069-42732124	0.216	0.004302566949
MXE	PPIL3	-	chr2:200882341-200882382	chr2:200885261-200885343	0.286	0.004322405941
MXE	POLM	-	chr7:44079570-44079741	chr7:44079860-44079959	0.279	0.004328527956
MXE	RABEPK	+	chr9:125220538-125220700	chr9:125227909-125228059	0.116	0.004332221339
MXE	TBL1X	+	chrX:9534899-9535034	chrX:9640272-9640367	0.131	0.004619036225
MXE	TEX9	+	chr15:56412301-56412436	chr15:56428366-56428473	0.163	0.004726296243
MXE	MRNIP	-	chr5:179847977-179848066	chr5:179853196-179853276	0.159	0.004780932254
MXE	MELK	+	chr9:36589535-36589652	chr9:36594627-36594771	0.128	0.004831103709
MXE	B4GALT4	-	chr3:119229846-119230244	chr3:119236852-119237070	0.106	0.004953620754
MXE	RFWD2	-	chr1:176136487-176136547	chr1:176149005-176149074	0.28	0.005073756977
MXE	UBE3A	-	chr15:25407066-25407212	chr15:25408619-25408684	0.199	0.005157906822
MXE	FAM228B	+	chr2:24146929-24147086	chr2:24164197-24164335	0.315	0.005315538034
MXE	CTU2	+	chr16:88707135-88707210	chr16:88709937-88710016	0.271	0.005693884603
MXE	PPP4C	+	chr16:30081258-30081310	chr16:30082483-30082534	0.164	0.005808692927

MXE	TRIM5	-	chr11:5665980-5666081	chr11:5667688-5667711	-0.204	0.005212261631
MXE	TMA16	+	chr4:163514073-163514158	chr4:163515312-163515461	-0.108	0.005271854269
MXE	ZGRF1	-	chr4:112603523-112603697	chr4:112606007-112606091	-0.186	0.005366809164
MXE	ZMYND8	-	chr20:47236325-47236516	chr20:47249286-47249439	-0.139	0.005434224012
MXE	CCDC7	+	chr10:32463016-32463049	chr10:32471063-32471230	-0.421	0.0054461904
MXE	PSMA3-AS1	-	chr14:58285590-58285756	chr14:58291634-58291707	-0.248	0.005483540331
MXE	RUFY3	+	chr4:70789494-70789592	chr4:70793764-70793904	-0.252	0.005649545712
MXE	RPAIN	+	chr17:5425970-5426299	chr17:5428070-5428211	-0.102	0.005830025374
MXE	LRP8	-	chr1:53271027-53271153	chr1:53271226-53271346	-0.148	0.006052763706
MXE	RWDD3	+	chr1:95244210-95244698	chr1:95246541-95246657	-0.142	0.006065508786
MXE	ACER3	+	chr11:76985642-76985724	chr11:76990538-76990574	-0.21	0.006065508786
MXE	FAM92A	+	chr8:93709770-93709875	chr8:93718674-93718788	-0.105	0.006065508786
MXE	RASSF4	+	chr10:44982520-44982663	chr10:44984021-44984113	-0.181	0.006290006577
MXE	NUDT13	+	chr10:73125117-73125243	chr10:73125397-73125509	-0.384	0.006397404286
MXE	FAM86C1	+	chr11:71791738-71791819	chr11:71793379-71793481	-0.395	0.00664473448
MXE	CACNB4	-	chr2:151855223-151855375	chr2:151870841-151870861	-0.198	0.006726053455
MXE	TRPC1	+	chr3:142736378-142736533	chr3:142743484-142743586	-0.146	0.006802253715
MXE	ACAA1	-	chr3:38131925-38132005	chr3:38133951-38134009	-0.213	0.006804735893
MXE	IGF2BP3	-	chr7:23319137-23319254	chr7:23342063-23342189	-0.106	0.00699945538
MXE	CTNND1	+	chr11:57789036-57789155	chr11:57791493-57791673	-0.237	0.007137831095
MXE	CTNND1	+	chr11:57789036-57789155	chr11:57791491-57791673	-0.238	0.007189633564
MXE	FLCN	-	chr17:17222500-17222661	chr17:17223921-17224143	-0.238	0.00723383536
MXE	RPL9	-	chr4:39454863-39454944	chr4:39456405-39456538	-0.102	0.007314278166
MXE	HSD11B1L	+	chr19:5684818-5684905	chr19:5684964-5685119	-0.557	0.007513422672
MXE	TTC28-AS1	+	chr22:27935150-27935230	chr22:27993123-27993535	-0.166	0.007663048107
MXE	FAM53C	+	chr5:138333432-138333528	chr5:138335384-138335485	-0.129	0.007877138958
MXE	TOGARAM1	+	chr14:45006007-45006267	chr14:45008912-45009145	-0.172	0.007886016626
MXE	ZGRF1	-	chr4:112603523-112603697	chr4:112606007-112606091	-0.178	0.008014771864
MXE	SYTL2	-	chr11:85714412-85714507	chr11:85717482-85717530	-0.199	0.008014771864
MXE	LINC00174	-	chr7:66455658-66455853	chr7:66467681-66467719	-0.442	0.008104896287
MXE	SIPA1L3	+	chr19:38119305-38119882	chr19:38130497-38130772	-0.122	0.008360219703
MXE	ADGRL3	+	chr4:61676825-61676935	chr4:61732753-61733554	-0.138	0.008473719203
MXE	CHEK2	-	chr22:28695709-28695873	chr22:28696900-28696987	-0.111	0.008687271641
MXE	AURKA	-	chr20:56388311-56388506	chr20:56388866-56388784	-0.126	0.008758570512
MXE	GALNT7	+	chr4:173297794-173297977	chr4:173298114-173298297	-0.152	0.008807758448
MXE	GAS5	-	chr1:173866760-173866796	chr1:173866990-173867043	-0.166	0.008854126289
MXE	CAMK2G	-	chr10:73824039-73824084	chr10:73825278-73825347	-0.176	0.009092240333
MXE	ANKMY1	-	chr2:240507559-240507691	chr2:240511860-240512001	-0.18	0.009147376957
MXE	SPOP	-	chr17:49636758-49636809	chr17:49668026-49668078	-0.254	0.009456335949
MXE	CAPRIN2	-	chr12:30719078-30719225	chr12:30720810-30720915	-0.157	0.009621429537
MXE	SENP7	-	chr3:101398860-101399055	chr3:101417592-101417790	-0.117	0.009790410867
MXE	CCDC14	-	chr3:123955842-123955965	chr3:123956354-123956427	-0.103	0.01001908489
MXE	ZNF562	-	chr19:9656546-9656653	chr19:9660719-9660874	-0.177	0.01002915357
MXE	LINC01125	+	chr2:97694446-97694599	chr2:97701153-97701304	-0.331	0.0101820342
MXE	PCBP1-AS1	-	chr2:70055655-70055910	chr2:70059642-70059680	-0.27	0.01020578741
MXE	FASTK	-	chr7:151076912-151077027	chr7:151077100-151077236	-0.121	0.01029295253
MXE	KIF13A	-	chr6:17789871-17789910	chr6:17794248-17794395	-0.232	0.01033725682
MXE	CBWD1	-	chr9:147900-148040	chr9:151304-151427	-0.365	0.01047188361
MXE	PQLC3	+	chr2:11164203-11164261	chr2:11171924-11172045	-0.19	0.0108490355
MXE	RNF146	+	chr6:127280515-127280604	chr6:127282286-127282395	-0.382	0.01094845149
MXE	LINC01125	+	chr2:97690255-97690327	chr2:97694446-97694599	-0.519	0.01103826341
MXE	SFI1	+	chr22:31556941-31557059	chr22:31561289-31561392	-0.182	0.01122483829
MXE	ABHD14A-ACY1	+	chr3:51978258-51978374	chr3:51980392-51980628	-0.239	0.0113272972
MXE	BMP2K	+	chr4:78878733-78878891	chr4:78887173-78887294	-0.153	0.01147700303
MXE	SCAI	-	chr9:124974174-124974276	chr9:124975246-124975389	-0.139	0.01228392512
MXE	RFWD2	-	chr1:176149005-176149074	chr1:176175909-176176007	-0.212	0.01239959315
MXE	GPD2	+	chr2:156476097-156476207	chr2:156496043-156496215	-0.115	0.01261593438
MXE	RFWD2	-	chr1:176149005-176149074	chr1:176184632-176184692	-0.34	0.01289228184
MXE	KIAA1328	+	chr18:37066889-37067545	chr18:37084124-37084232	-0.178	0.01313028342
MXE	MTMR2	-	chr11:95882373-95882496	chr11:95887611-95887684	-0.388	0.01313028342
MXE	THAP9-AS1	-	chr4:82895691-82895774	chr4:82897988-82898062	-0.118	0.01323461605
MXE	HSD11B1L	+	chr19:5684818-5684905	chr19:5684964-5685119	-0.541	0.01372157933
MXE	LIAS	+	chr4:39463524-39463605	chr4:39465045-39465202	-0.112	0.0137419117
MXE	PCBP1-AS1	-	chr2:70055713-70055910	chr2:70059642-70059680	-0.246	0.01388670621
MXE	PCBP1-AS1	-	chr2:70055713-70055910	chr2:70059642-70059680	-0.246	0.01388670621
MXE	GTPBP10	+	chr7:90355085-90355230	chr7:90372154-90372228	-0.153	0.01403485768
MXE	SH3D19	-	chr4:151149499-151149561	chr4:151159239-151159352	-0.189	0.0144354842
MXE	PCNT	+	chr21:46421969-46422124	chr21:46425830-46425971	-0.26	0.01452950685
MXE	DOPEY1	+	chr6:83147235-83147291	chr6:83148758-83148863	-0.179	0.01456348202
MXE	C1QTNF6	-	chr22:37191671-37191737	chr22:37195380-37195458	-0.134	0.01470907625
MXE	CARD8	-	chr19:48249522-48249633	chr19:48249750-48249847	-0.132	0.01479498131
MXE	CACNB4	-	chr2:151870530-151870611	chr2:151870841-151870861	-0.263	0.01553418096
MXE	SAP30BP	+	chr17:75703310-75703371	chr17:75703807-75703859	-0.134	0.01563724167
MXE	CROCCP2	-	chr1:16635026-16635168	chr1:16642766-16642850	-0.24	0.01637989376
MXE	MOK	-	chr14:102263545-102263616	chr14:102278605-102278694	-0.108	0.01710508937
MXE	ZHX3	-	chr20:41213733-41213899	chr20:41267462-41267612	-0.408	0.01738238435
MXE	WDR27	-	chr6:169665485-169665556	chr6:169670568-169670693	-0.225	0.01847367209
MXE	LIAS	+	chr4:39463524-39463605	chr4:39467517-39467646	-0.102	0.01876642424
MXE	NBPF12	+	chr1:146989573-146989746	chr1:146991112-146991285	-0.243	0.01900691102
MXE	SH3D19	-	chr4:151147921-151148186	chr4:151149499-151149561	-0.136	0.01943201436
MXE	FAM86C1	+	chr11:71791735-71791819	chr11:71793379-71793481	-0.231	0.01960597291
MXE	DPY19L4	+	chr8:94756035-94756159	chr8:94761699-94761834	-0.12	0.01971974686
MXE	BBOF1	+	chr14:74046059-74046130	chr14:74047929-74048074	-0.127	0.01977590355
MXE	MELTF	-	chr3:197021403-197021471	chr3:197022956-197023113	-0.268	0.02020366208
MXE	CUZD1	-	chr10:122836177-122836350	chr10:122836830-122836899	-0.162	0.02039991834
MXE	ZC3H11A	+	chr1:203796450-203803016	chr1:203818569-203818689	-0.122	0.02053261644
MXE	PMS2P1	-	chr7:100328835-100329019	chr7:100330181-100330284	-0.11	0.02066836334
MXE	DHRS4L2	+	chr14:23995031-23995133	chr14:24001383-24001517	-0.307	0.02072749562
MXE	CACNB4	-	chr2:151870530-151870611	chr2:151870841-151870861	-0.273	0.02085288607
MXE	FAM228B	+	chr2:24135118-24135187	chr2:24164197-24164335	-0.133	0.02193360829
MXE	PIGP	-	chr21:37067261-37067380	chr21:37069551-37069624	-0.126	0.02367950919
MXE	HDAC8	-	chrX:72488932-72489041	chrX:72495155-72495268	-0.134	0.0247604586
MXE	SRBD1	-	chr2:45476992-45477075	chr2:45488239-45488331	-0.18	0.02536826635
MXE	USP53	+	chr4:119235289-119235411	chr4:119239217-119239903	-0.112	0.02547112692
MXE	KIF13A	-	chr6:17873376-17873437	chr6:17898167-17898180	-0.19	0.02565955525
MXE	IFI27L1	+	chr14:94100738-94100771	chr14:94101813-94102059	-0.137	0.02602007324
MXE	SETD1A	+	chr16:30961266-30961537	chr16:30963432-30963554	-0.139	0.02646674035
MXE	MOK	-	chr14:102263545-102263616	chr14:102265822-102265912	-0.197	0.02719533086
MXE	SHMT1	-	chr17:18333165-18333288	chr17:18335558-18335675	-0.112	0.0274075858
MXE	WDR4	-	chr21:42859661-42859722	chr21:42862281-42862394	-0.158	0.02770680963
MXE	CCDC14	-	chr3:123955842-123955965	chr3:123956045-123956115	-0.121	0.02785913751
MXE	RGL1	+	chr1:183806374-183806485	chr1:183847565-183847774	-0.141	0.02946769912
MXE	CLN3	-	chr16:28486346-28486490	chr16:28486577-28486650	-0.172	0.03019109549
MXE	TPT1-AS1	+	chr13:45379898-45380001	chr13:45383090-45383235	-0.116	0.03030437867
MXE	HPS5	-	chr11:18311913-18312024	chr11:18317750-18317907	-0.14	0.03038613536
MXE	TRABD2A	-	chr2:84839148-84839323	chr2:84841860-84842007	-0.25	0.03053270545
MXE	SEC24C	+	chr10:73751107-73751243	chr10:73759621-73759794	-0.118	0.03147663073
MXE	ABHD14A	+	chr3:51978258-51978374	chr3:51980392-51980628	-0.203	0.0318128299
MXE	UCKL1	-	chr20:63942469-63942492	chr20:63943652-63943669	-0.169	0.0337906073
MXE	TTC25	+	chr17:41935470-41935543	chr17:41935598-41935749	-0.215	0.03441493153
MXE	PCBP1-AS1	-	chr2:70055713-70055910	chr2:70059642-70059680	-0.431	0.03469929876
MXE	NDUFAF6	+	chr8:95036301-95036494	chr8:95040705-95040807	-0.13	0.03547198855
MXE	NDE1	+	chr16:15651468-15651584	chr16:15651930-15652050	-0.124	0.03549421484
MXE	PAQR3	-	chr4:78920533-78920681	chr4:78923856-78923947	-0.147	0.03575113456
MXE	STX2	-	chr12:130806981-130807090	chr12:130808630-130808704	-0.116	0.03649123668
MXE	CCDC66	+	chr3:56563683-56564125	chr3:56566593-56566740	-0.109	0.0372602753
MXE	ZUFSP	-	chr6:116660735-116660846	chr6:116666633-116666912	-0.12	0.03791778075
MXE	TBC1D5	-	chr3:17508473-17508605	chr3:17623848-17623913	-0.117	0.03804740112
MXE	ARL14EP	+	chr11:30330885-30331374	chr11:30332865-30332993	-0.109	0.03819192811
MXE	ASPSCR1	+	chr17:82016475-82016527	chr17:82016799-82016869	-0.327	0.03891190073
MXE	MVK	+	chr12:109579801-109579946	chr12:109581394-109581550	-0.105	0.03891190073

CHEK1
CHEK2
CHFR
CHKA
CHKB
CHMP2B
CHN1
CHORDC1
CHPF2
CHRM3
CHTF8
CHURC1
CHURC1-FNTB
CIB1
CIB2
CIRBP
CITED1
CIZ1
CKLF
CKLF-CMTM1
CLASP1
CLASP2
CLCN2
CLCN3
CLEC16A
CLEC2B
CLEC2D
CLHC1
CLIP1
CLK1
CLK3
CLK4
CLN3
CLN6
CLOCK
CLPTM1L
CLSTN1
CLTC
CLYBL
CMC1
CMC2
CNBP
CNNM3
CNOT2
CNOT3
CNOT8
CNTLN
CNTRL
COA1
COG4
COG7
COL12A1
COL27A1
COL4A3BP
COMMD3
COMMD4
COMMD7
COPG1
COQ3
COQ5
CORO7
CORO7-PAM16
COX11
COX20
CPEB2
CPM
CPNE3
CPSF7
CPT1B
CRBN
CREB1
CRELD1
CRELD2
CREM
CRIM1
CRLS1
CROCCP2
CROCCP3
CRYZ
CS
CSAD
CSNK1D
CSNK1G3
CSPP1
CTAGE5
CTBP2
CTC1
CTC-308K20.1
CTC-444N24.6
CTD-2116N17.1
CTD-2341M24.1
CTD-2378E12.1
CTD-2583A14.10
CTD-2651B20.3
CTDP1
CTNND1
CTSC
CTU2
CUEDC2
CUL5
CUL9
CUTC
CUZD1
CWF19L1
CXorf23
CXorf38
CYB5R2
CYLD
CYP20A1
DAG1
DALRD3
DAP3
DBR1
DCAF1
DCAF10
DCAF11
DCAF4
DCAF8
DCLK2
DCTD
DCTN6

MXE	TMPO	+	chr12:98544950-98545061	chr12:98546358-98546447	0.108	0.006041335787	MXE	FAM135A	+	chr6:70426438-70426532	chr6:70426569-70426709	-0.159	0.03932366509	DCUN1D4
MXE	FUZ	-	chr19:49812250-49812335	chr19:49812446-49812736	0.511	0.006041335787	MXE	PRPF4B	+	chr6:4061654-4061700	chr6:4062212-4062299	-0.24	0.03970723868	DCXR
MXE	CAPN7	+	chr3:15242177-15242253	chr3:15245525-15245671	0.27	0.006346642534	MXE	WBSCR22	+	chr7:73693623-73693669	chr7:73693991-73694050	-0.103	0.03980685599	DDHD1
MXE	RCL1	+	chr9:4826857-4827033	chr9:4833153-4833228	0.175	0.006396217123	MXE	MX1	+	chr21:41449136-41449295	chr21:41451166-41451243	-0.419	0.04197901362	DDIAS
MXE	TNFSF12-TNFS	+	chr17:7559623-7559702	chr17:7559845-7559893	0.19	0.006396217123	MXE	TNC	-	chr9:115046409-115046682	chr9:115048259-115048532	-0.285	0.04224761506	DDIT3
MXE	GTSE1	+	chr22:46326435-46326654	chr22:46328687-46328889	0.122	0.006468149765	MXE	RP11-421L21.3	+	chr1:101077362-101077508	chr1:101083460-101083574	-0.235	0.04252502176	DDR1
MXE	RABL2B	-	chr22:50769454-50769552	chr22:50775771-50775851	0.246	0.006656664683	MXE	NDUFAF6	+	chr8:95036370-95036494	chr8:95040705-95040927	-0.109	0.04341779919	DDX11
MXE	RPL21	+	chr13:27253764-27253843	chr13:27255241-27255349	0.201	0.006700381109	MXE	LINC01778	+	chr1:30826554-30826652	chr1:30829747-30829882	-0.203	0.04357236015	DDX17
MXE	HSCB	+	chr22:28745863-28746008	chr22:28751240-28751288	0.191	0.00674097484	MXE	MOK	-	chr14:102265822-102265912	chr14:102278605-102278694	-0.135	0.04446147507	DDX39A
MXE	RPL21	+	chr13:27253764-27253843	chr13:27255241-27255354	0.205	0.006823004384	MXE	SFI1	+	chr22:31556941-31557059	chr22:31561289-31561392	-0.221	0.04505429643	DDX39B
MXE	UBE3A	-	chr15:25407066-25407137	chr15:25408619-25408684	0.246	0.006838125131	MXE	WDR4	-	chr21:42855681-42855780	chr21:42859661-42859722	-0.189	0.04529641663	DDX42
MXE	KRIT1	-	chr7:92235402-92236542	chr7:92237666-92237759	0.128	0.006841950359	MXE	U2AF1L5	-	chr21:6492130-6492197	chr21:6493043-6493110	-0.334	0.04701157616	DDX46
MXE	DCXR	-	chr17:82036383-82036448	chr17:82036543-82036643	0.123	0.007049774526	MXE	IQCK	+	chr16:19735352-19735450	chr16:19788837-19788922	-0.216	0.04786024144	DDX50
MXE	PARPBP	+	chr12:102148229-102148463	chr12:102165728-102165883	0.121	0.007314278166	MXE	GARNL3	+	chr9:127264951-127265021	chr9:127291167-127291242	-0.161	0.04806034241	DDX51
MXE	POP4	+	chr19:29610408-29610457	chr19:29611861-29611939	0.229	0.007333096958	MXE	MARK2	+	chr11:63903060-63903158	chr11:63903985-63904147	-0.139	0.04895904011	DDX58
MXE	PGAP3	-	chr17:39676613-39676755	chr17:39684596-39684749	0.199	0.007333096958	RI	RSRP1	-	chr1:25243549-25245301	NA	-0.168	0	DECR2
MXE	LETMD1	+	chr12:51052091-51052207	chr12:51055834-51056021	0.126	0.007378020695	RI	NOC3L	-	chr10:94357173-94358215	NA	-0.182	0	DEF8
MXE	ATP2B1	-	chr12:89627677-89627716	chr12:89630504-89630645	0.152	0.007378547302	RI	CEP192	+	chr18:13057584-13059312	NA	-0.287	0	DEK
MXE	RPL9	-	chr4:39456405-39456538	chr4:39457585-39457681	0.37	0.007466712483	RI	MCM7	-	chr7:100099022-100099753	NA	-0.108	0	DENND4A
MXE	MFF	+	chr2:227347225-227347384	chr2:227352513-227352573	0.151	0.007663048107	RI	BDH2	-	chr4:103085348-103086540	NA	-0.253	0	DENND4C
MXE	RMDN3	-	chr15:40738500-40738576	chr15:40745119-40745259	0.11	0.007869364411	RI	ANAPC5	-	chr12:121345838-121347005	NA	-0.144	0	DENND5A
MXE	MTHFD1L	+	chr6:150887844-150887981	chr6:150905649-150905761	0.142	0.008350531521	RI	ANAPC5	-	chr12:121327095-121328497	NA	-0.144	0	DEPDC1
MXE	LDLR	+	chr19:11123173-11123344	chr19:11128007-11128085	0.134	0.008448894889	RI	BBS2	-	chr16:56502316-56502808	NA	-0.191	0	DEPDC5
MXE	ETNK1	+	chr12:22671271-22671355	chr12:22673499-22673660	0.112	0.008574289804	RI	CD46	+	chr1:207767012-207770362	NA	-0.477	0	DERL2
MXE	PHACTR4	+	chr1:28407409-28407463	chr1:28457831-28457855	0.149	0.00857806831	RI	RPL17	-	chr18:49491531-49491833	NA	-0.324	0	DET1
MXE	ARHGEF7	+	chr13:111153904-111153991	chr13:111209871-111210002	0.14	0.008703836928	RI	TSEN2	+	chr3:12529761-12531659	NA	-0.169	0	DGKA
MXE	ZNF83	-	chr19:52635065-52635153	chr19:52655560-52655687	0.184	0.008711948191	RI	COG4	-	chr16:70508405-70509388	NA	-0.192	1.14E-14	DGKH
MXE	FAM228B	+	chr2:24146929-24147086	chr2:24167626-24167683	0.308	0.008954576075	RI	MLST8	+	chr16:2206357-2206659	NA	-0.766	1.14E-14	DGKZ
MXE	DGUOK	+	chr2:73938909-73939022	chr2:73950584-73950732	0.157	0.00925802358	RI	MATR3	+	chr5:139314674-139316188	NA	-0.388	8.68E-14	DGUOK
MXE	PLAT	-	chr8:42185080-42185158	chr8:42187397-42187572	0.116	0.009587266545	RI	CLK4	-	chr5:178616881-178618778	NA	-0.481	1.16E-13	DHFR
MXE	SLC9B1	-	chr4:102949256-102949427	chr4:102989799-102989941	0.474	0.01001908489	RI	SUGP2	-	chr19:18994365-18995280	NA	-0.322	1.97E-13	DHODH
MXE	KIAA1551	+	chr12:31960776-31960871	chr12:31980877-31985957	0.111	0.01001908489	RI	FUS	+	chr16:31184938-31188357	NA	-0.141	6.33E-13	DHPS
MXE	CRLS1	+	chr20:6026170-6026238	chr20:6028671-6028725	0.369	0.01002816107	RI	TAMM41	-	chr3:11807832-11809682	NA	-0.108	6.68E-13	DHRS1
MXE	ACP1	+	chr2:272036-272150	chr2:272191-272305	0.13	0.01002816107	RI	MAN2C1	-	chr15:75358461-75358808	NA	-0.337	1.41E-12	DHRS4-AS1
MXE	MELK	+	chr9:36589535-36589652	chr9:36594627-36594771	0.127	0.01013902407	RI	HMGN1	-	chr21:39345145-39348339	NA	-0.329	1.73E-12	DHRS4L2
MXE	DLST	+	chr14:74882590-74882624	chr14:74885585-74885634	0.149	0.01029295253	RI	TPM1	+	chr15:63061197-63062277	NA	-0.14	1.57E-11	DHTKD1
MXE	DBR1	-	chr3:138163777-138163858	chr3:138167080-138167305	0.171	0.01047188361	RI	CIRBP	+	chr19:1271980-1274440	NA	-0.142	5.48E-11	DHX33
MXE	FBXL2	+	chr3:33297868-33297962	chr3:33359282-33359357	0.262	0.01098908084	RI	CLK1	-	chr2:200859679-200861466	NA	-0.38	5.85E-11	DHX34
MXE	ASCC3	-	chr6:100848147-100848707	chr6:100864063-100864214	0.126	0.01102991524	RI	BAZ2A	-	chr12:56615013-56615607	NA	-0.151	1.89E-10	DHX9
MXE	ZGRF1	-	chr4:112587279-112587929	chr4:112603523-112603697	0.107	0.01126235261	RI	RPS27	+	chr1:153990762-153991034	NA	-0.166	2.22E-10	DIABLO
MXE	HPS4	-	chr22:26465454-26465551	chr22:26466225-26466262	0.161	0.01126235261	RI	SLC25A3	+	chr12:98593974-98595848	NA	-0.124	3.27E-10	DIAPH2
MXE	ARNT	-	chr1:150842423-150842468	chr1:150846262-150846307	0.101	0.01126235261	RI	MFN1	+	chr3:179378340-179378814	NA	-0.137	4.80E-10	DIEXF
MXE	ACOT8	-	chr20:45848449-45848675	chr20:45855158-45855292	0.153	0.01128144421	RI	NELFE	-	chr6:31953728-31954134	NA	-0.144	1.11E-09	DLEU1
MXE	ANKRD27	-	chr19:32605834-32605954	chr19:32607634-32607832	0.209	0.01128144421	RI	HAUS7	-	chrX:153454393-153455766	NA	-0.157	1.59E-09	DLEU2
MXE	TALDO1	+	chr11:755878-756002	chr11:759008-759057	0.114	0.01128144421	RI	PLSCR1	-	chr3:146521543-146522053	NA	-0.279	1.65E-09	DLG1
MXE	FAM49B	-	chr8:129879596-129879789	chr8:129896748-129896854	0.146	0.01128144421	RI	NAGS	+	chr17:44006528-44007494	NA	-0.583	2.47E-09	DLG3
MXE	ZNF655	+	chr7:99561887-99561973	chr7:99562367-99562494	0.168	0.01140485557	RI	SPAG5	-	chr17:28579750-28580120	NA	-0.101	2.69E-09	DLG4
MXE	PCNX1	+	chr14:70988566-70988699	chr14:71009633-71009724	0.145	0.01152016078	RI	March6	+	chr5:10400783-10402139	NA	-0.253	4.85E-09	DLGAP1-AS1
MXE	CPSF7	-	chr11:61421053-61421127	chr11:61421191-61421257	0.378	0.01173911421	RI	DNHD1	+	chr11:6497584-6498961	NA	-0.604	1.49E-08	DLGAP4
MXE	MBNL1	+	chr3:152455541-152455577	chr3:152456266-152456361	0.163	0.01194955016	RI	ZC3H11A	+	chr1:203796450-203803016	NA	-0.23	1.55E-08	DLGAP5
MXE	TRAPPC3	-	chr1:36139719-36139819	chr1:36140068-36140166	0.111	0.01217057872	RI	FAH	+	chr15:80152793-80153135	NA	-0.544	3.92E-08	DLST
MXE	FAM228B	+	chr2:24146929-24147086	chr2:24164197-24164335	0.263	0.01231089299	RI	ARHGAP32	-	chr11:128972452-128975002	NA	-0.203	4.53E-08	DMAP1
MXE	CIB1	-	chr15:90232218-90232327	chr15:90233668-90233703	0.112	0.01287962329	RI	MLST8	+	chr16:2206357-2206659	NA	-0.198	5.59E-08	DMPK
MXE	TMEM161B-AS	+	chr5:88287435-88287622	chr5:88410072-88410194	0.224	0.0129113373	RI	BTN3A1	+	chr6:26409893-26410032	NA	-0.15	5.62E-08	DMTF1
MXE	PCBP1-AS1	-	chr2:70059642-70059680	chr2:70083537-70083687	0.181	0.01308646593	RI	TUBGCP4	+	chr15:43401715-43403799	NA	-0.278	7.93E-08	DMXL2
MXE	TLK1	-	chr2:171028338-171028405	chr2:171046173-171046362	0.105	0.01313028342	RI	HNRNPM	+	chr19:8465323-8465515	NA	-0.161	7.93E-08	DNAAF2
MXE	PLEKHA4	-	chr19:48839204-48839263	chr19:48841148-48841310	0.396	0.01313028342	RI	CENPT	-	chr16:67832011-67832545	NA	-0.428	8.43E-08	DNAH6
MXE	DCTN6	+	chr8:30164110-30164175	chr8:30175084-30175190	0.221	0.01355535248	RI	CLK4	-	chr5:178617343-178618778	NA	-0.336	9.82E-08	DNAJB6
MXE	RP11-274B21.1	+	chr7:128578889-128578933	chr7:128579998-128580094	0.251	0.01368807562	RI	EXOSC9	+	chr4:121813865-121816447	NA	-0.124	9.95E-08	DNAJC10
MXE	UBE3A	-	chr15:25407066-25407262	chr15:25408619-25408684	0.154	0.01414466032	RI	MTF2	+	chr1:93110229-93110428	NA	-0.264	1.02E-07	DNAJC13
MXE	MELK	+	chr9:36589535-36589652	chr9:36594627-36594771	0.139	0.01456348202	RI	PACS2	+	chr14:105383358-105384463	NA	-0.184	1.17E-07	DNAJC14
MXE	CACNB4	-	chr2:151855223-151855375	chr2:151870530-151870611	0.41	0.01456348202	RI	CTC-308K20.1	-	chr5:172957153-172958257	NA	-0.484	1.27E-07	DNAJC17
MXE	SNX14	-	chr6:85549722-85549879	chr6:85557975-85558060	0.146	0.01475123239	RI	YBX3	-	chr12:10709907-10713333	NA	-0.112	1.32E-07	DNAJC19
MXE	C8orf76	-	chr8:123237797-123237941	chr8:123239048-123239144	0.205	0.01478857271	RI	ILF3	+	chr19:10681218-10682192	NA	-0.143	1.78E-07	DNAJC4
MXE	HSCB	+	chr22:28743881-28743923	chr22:28751240-28751288	0.233	0.01546534984	RI	RBM6	+	chr3:50061461-50062108	NA	-0.347	1.98E-07	DNAJC5
MXE	PCBP1-AS1	-	chr2:70059642-70059680	chr2:70083505-70083687	0.183	0.01562606437	RI	TPM2	-	chr9:35684487-35685139	NA	-0.633	3.04E-07	DNAJC7
MXE	N4BP2	+	chr4:40097226-40097569	chr4:40100035-40100126	0.26	0.01590946898	RI	CARD19	+	chr9:93110567-93112289	NA	-0.301	3.15E-07	DNAJC8
MXE	DHRS4L2	+	chr14:24001383-24001517	chr14:24004336-24004392	0.22	0.01613726558	RI	WDR55	+	chr5:140668233-140668502	NA	-0.188	3.74E-07	DNAL1
MXE	C11orf65	-	chr11:108405428-108405559	chr11:108431745-108431838	0.19	0.01613726558	RI	BCKDK	+	chr16:31110400-31111219	NA	-0.469	3.95E-07	DNHD1
MXE	CIZ1	-	chr9:128187862-128187934	chr9:128190328-128190444	0.137	0.0168551311	RI	COMMD4	+	chr15:75338984-75339878	NA	-0.366	6.58E-07	DNM1
MXE	PKD1P6-NPIPP	-	chr16:15125690-15125807	chr16:15127469-15127596	0.32	0.01727736852	RI	DRG2	+	chr17:18099016-18100435	NA	-0.13	6.99E-07	DNM2
MXE	RABEPK	+	chr9:125220538-125220700	chr9:125227909-125228059	0.102	0.01742082734	RI	UBXN4	+	chr2:135753538-135754277	NA	-0.168	8.43E-07	DNM3
MXE	TPT1-AS1	+	chr13:45383090-45383235	chr13:45389734-45389820	0.107	0.018188216	RI	A1BG-AS1	+	chr19:58353713-58355183	NA	-0.563	1.09E-06	DNMT3A
MXE	FAM228B	+	chr2:24146929-24147086	chr2:24164197-24164335	0.271	0.01854545969	RI	ARRDC1	+	chr9:137612895-137613510	NA	-0.299	1.29E-06	DOCK1
MXE	ZGRF1	-	chr4:112587279-112587929	chr4:112603523-112603697	0.108	0.01901870816	RI	EPS8L2	+	chr11:709552-710486	NA	-0.571	1.48E-06	DOCK10
MXE	ZGRF1	-	chr4:112586444-112586583	chr4:112603523-112603697	0.176	0.01943201436	RI	RPP21	+	chr6:30345297-30346557	NA	-0.118	1.90E-06	DOCK7
MXE	IRF3	-	chr19:49660712-49660828	chr19:49661947-49662328	0.146	0.02026289817	RI	CLK3	+	chr15:74622119-74622560	NA	-0.15	1.92E-06	DOCK9
MXE	SLC38A9	-	chr5:55656714-55656774	chr5:55664692-55664863	0.106	0.02053550709	RI	ETV4	-	chr17:43545273-43545668	NA	-0.136	2.47E-06	DOK1
MXE	HMGN1	-	chr21:39347246-39347292	chr21:39347378-39347478	0.122	0.02072749562	RI	WDR83	+	chr19:12669754-12670080	NA	-0.576	5.75E-06	DOLPP1
MXE	GUSB	-	chr7:65976014-65976202	chr7:65979833-65979911	0.116	0.02072749562	RI	WDR46	-	chr6:33280427-33280987	NA	-0.152	6.22E-06	DONSON
MXE	AC005154.6	-	chr7:30576563-30576710	chr7:30584710-30584808	0.278	0.02127345004	RI	DDX51	-	chr12:132140830-132141420	NA	-0.49	7.41E-06	DOPEY1
MXE	CRELD2	+	chr22:49923233-49923317	chr22:49924359-49924455	0.137	0.02184438566	RI	EXOSC8	+	chr13:37002933-37004561	NA	-0.106	7.73E-06	DPAGT1
MXE	CBWD1	-	chr9:146101-146158	chr9:151304-151427	0.103	0.02204786988	RI	DENND4C	+	chr9:19336269-19336832	NA	-0.222	9.18E-06	DPH1
MXE	ZC3H7B	+	chr22:41346002-41346208	chr22:41348066-41348167	0.112	0.02216666014	RI	KRIT1	-	chr7:92235402-92236542	NA	-0.14	9.18E-06	DPH3
MXE	CHKA	-	chr11:68074716-68074830	chr11:68081403-68081457	0.108	0.02218226445	RI	E2F4	+	chr16:67192760-67193170	NA	-0.136	9.34E-06	DPH5
MXE	ARMCX5-GPRA	+	chrX:102601879-102601977	chrX:102639399-102639533	0.154	0.02227107106	RI	TERF2	-	chr16:69368375-69368595	NA	-0.523	1.08E-05	DPM1
MXE	POLL	-	chr10:101582762-101582891	chr10:101584601-101584919	0.125	0.02262017329	RI	CCDC136	+	chr7:128806236-128806858	NA	-0.455	1.38E-05	DPM2
MXE	METTL6	-	chr3:15414020-15414162	chr3:15415497-15415583	0.174	0.02355341548	RI	RPGR	-	chrX:38287093-38288041	NA	-0.212	1.39E-05	DPY19L2
MXE	C6orf52	-	chr6:10683186-10683232	chr6:10686965-10687164	0.145	0.02470719013	RI	SNHG14	+	chr15:25087466-25089866	NA	-0.204	1.55E-05	DPY19L3
MXE	CTSC	-	chr11:88312387-88312554	chr11:88334936-88335082	0.108	0.0247604586	RI	TLE2	-	chr19:3019282-3019773	NA	-0.546	1.93E-05	DPY19L4
MXE	CDKL3	-	chr5:134308573-134308727	chr5:134312291-134312380	0.291	0.02508081161	RI	SNX15	+	chr11:65035519-65038829	NA	-0.182	2.03E-05	DRAM2
MXE	MCTP1	-	chr5:94714776-94714886	chr5:94779109-94779163	0.198	0.02584102084	RI	CCHCR1	-	chr6:31144686-31145073	NA	-0.388	2.14E-05	DRG2
MXE	KIAA1429	-	chr8:94530962-94531085	chr8:94534838-94535007	0.112	0.02615574639	RI	PINK1	+	chr1:20648504-20649231	NA	-0.23	2.52E-05	DSN1
MXE	CDKL3	-	chr5:134308573-134308727	chr5:134312291-134312380	0.292	0.02648226135	RI	PEX1	-	chr7:92492952-92494396	NA	-0.21	2.53E-05	DST
MXE	EPS8	-	chr12:15670855-15670923	chr12:15681225-15681302	0.137	0.02834580514	RI	SRSF7	-	chr2:38748578-38749705	NA	-0.138	2.58E-05	DTNB
MXE	SH3GLB2	-	chr9:129010669-129010693	chr9:129012235-129012298	0.133	0.02873734411	RI	NDUFAF7	+	chr2:37241577-37242693	NA	-0.318	2.72E-05	DTWD1
MXE	RNF19B	-	chr1:32942251-32942459	chr1:32944018-32944159	0.179	0.03033891155	RI	ENDOV	+	chr17:80429772-80430343	NA	-0.266	2.91E-05	DUS1L
MXE	IKZF4	+	chr12:56019317-56019415	chr12:56023670-56023764	0.295	0.03038613536	RI	RBM8A	-	chr1:145926481-145927077	NA	-0.199	3.44E-05	DUS4L
MXE	AKIP1	+	chr11:8911443-8911671	chr11:8914825-8914930	0.172	0.0304034326	RI	ISCU	+	chr12:108564059-108564392	NA	-0.255	3.67E-05	DUSP12
MXE	LRP8	-	chr1:53275630-53275753	chr1:53280586-53280715	0.224	0.0304034326	RI	CROCCP3	-	chr1:16476929-16478437	NA	-0.584	3.92E-05	DUSP18
MXE	RANBP3	-	chr19:5931418-5931531	chr19:5932451-5932544	0.161	0.03047068983	RI	PRPF40B	+	chr12:49631859-49632880	NA	-0.355	4.01E-05	DUXAP10
MXE	POLL	-	chr10:101582762-101582891	chr10:101585861-101586156	0.139	0.03049002289	RI	KANSL2	-	chr12:48681381-48682225	NA	-0.201	4.32E-05	DUXAP8
MXE	ALG13	+	chrX:111708964-111709048	chrX:111711674-111711725	0.116	0.03083217036	RI	PRPF4B	+	chr6:4060412-4061147	NA	-0.11	4.91E-05	DUXAP9
MXE	ANKRD39	-	chr2:96848268-96848444	chr2:96853400-96853604	0.176	0.03138932416	RI	THAP7	-	chr22:21000646-21001411	NA	-0.17	5.29E-05	DVL3
MXE	HMGN1	-	chr21:39348291-39348339	chr21:39348421-39348577	0.151	0.03247308498	RI	NKIRAS2	+	chr17:42022398-42022640	NA	-0.163	5.64E-05	DXO
MXE	TNC	-	chr9:115048259-115048532	chr9:115064646-115064919	0.187	0.03274388609	RI	RPS9	+	chr19:54206552-54207647	NA	-0.319	6.28E-05	DYNC1I2
MXE	LETMD1	+	chr12:51052091-51052207	chr12:51056143-51056245	0.154	0.03327346733	RI	MPP2	-	chr17:43882902-43883355	NA	-0.23	8.14E-05	DYNC2H1
MXE	C6orf52	-	chr6:10683186-10683232	chr6:10686965-10687164	0.123	0.03336528488	RI	SIRT7	-	chr17:81914293-81915512	NA	-0.388	8.29E-05	DYNLL1
MXE	GRB10	-	chr7:50710874-50710990	chr7:50755886-50756056	0.18	0.03407818423	RI	SH3TC2	-	chr5:149026859-149028718	NA	-0.387	8.54E-05	DYRK4
MXE	P4HTM	+	chr3:49004860-49005046	chr3:49005717-49005867	0.125	0.03416242048	RI	AMBRA1	-	chr11:46541944-46543398	NA	-0.38	8.95E-05	DZANK1
MXE	PMS2	-	chr7:5978595-5978696	chr7:5982823-5982991	0.135	0.03417749302	RI	PEX1	-	chr7:92501506-92502079	NA	-0.198	9.31E-05	DZIP3
MXE	DAG1	+	chr3:49470964-49471049	chr3:49476848-49476905	0.277	0.03468750359	RI	BRAT1	-	chr7:2543203-2543962	NA	-0.283	9.46E-05	E2F4
MXE	NASP	+	chr1:45594693-45594773	chr1:45600384-45600457	0.277	0.03478906968	RI	EWSR1	+	chr22:29298732-29299280	NA	-0.354	9.93E-05	E2F6
MXE	ZC3H11A	+	chr1:203796450-203796601	chr1:203801574-203801628	0.199	0.03537717391	RI	ZCCHC8	-	chr12:122483278-122483563	NA	-0.216	0.0001105704463	EBPL
MXE	DHTKD1	+	chr10:12088985-12089255	chr10:12091512-12091684	0.142	0.03547198855	RI	LRRC41	-	chr1:46279181-46279614	NA	-0.122	0.0001182452421	ECHDC2
MXE	SENP7	-	chr3:101417592-101417790	chr3:101458954-101459052	0.124	0.03571358068	RI	GALE	-	chr1:23798116-23798730	NA	-0.109	0.0001267152438	ECI2
MXE	ST7L	-	chr1:112583971-112584126	chr1:112591524-112591603	0.177	0.03649123668	RI	IDH3A	+	chr15:78156907-78157631	NA	-0.239	0.0001490791649	ECSIT
MXE	NBPF26	+	chr1:120834494-120834546	chr1:120836516-120836568	0.438	0.03649123668	RI	DALRD3	-	chr3:49015818-49016086	NA	-0.283	0.0001551860719	EDC3
MXE	CAPN7	+	chr3:15240753-15240853	chr3:15245525-15245671	0.139	0.03674380634	RI	NSD1	+	chr5:177135086-177136030	NA	-0.175	0.0001641005066	EED
MXE	PPIL3	-	chr2:200882341-200882382	chr2:200885261-200885367	0.277	0.03684497474	RI	MTMR11	-	chr1:149930364-149930965	NA	-0.421	0.000164408632	EEF1AKMT1
MXE	RP11-421L21.3	+	chr1:101075394-101075716	chr1:101083460-101083574	0.215	0.03685287948	RI	NAGK	-	chr2:71072640-71073594	NA	-0.128	0.000164408632	EEF1D

MXE	ZRANB3	-	chr2:135390801-135390820	chr2:135504328-135504496	0.14	0.0369310243
MXE	TMEM241	-	chr18:23368714-23368750	chr18:23370215-23370261	0.262	0.0380235146
MXE	P4HTM	+	chr3:49004860-49005046	chr3:49005776-49005867	0.126	0.03840447679
MXE	SPAG1	+	chr8:100213818-100213918	chr8:100220278-100220431	0.178	0.03855224577
MXE	PCBP1-AS1	-	chr2:70018014-70018131	chr2:70051202-70051305	0.165	0.04040093942
MXE	ANKS3	-	chr16:4726658-4726780	chr16:4726978-4727177	0.242	0.04053130872
MXE	PCBP1-AS1	-	chr2:70018014-70018131	chr2:70051202-70051305	0.198	0.04071530613
MXE	FAM228B	+	chr2:24146929-24147086	chr2:24167626-24167683	0.261	0.0408781115
MXE	CEP85L	-	chr6:118491685-118491865	chr6:118511297-118511415	0.153	0.04132591343
MXE	NT5C3A	-	chr7:33024038-33024108	chr7:33026816-33026915	0.177	0.04140530147
MXE	MPDZ	-	chr9:13113930-13114021	chr9:13115247-13115334	0.123	0.04158436659
MXE	MSRB3	+	chr12:65308528-65308655	chr12:65326825-65326934	0.241	0.0416374328
MXE	SLC6A8	+	chrX:153693904-153694017	chrX:153694203-153694267	0.117	0.04184520844
MXE	ATG10	+	chr5:82164398-82164537	chr5:82178489-82178587	0.141	0.04224761506
MXE	C2orf76	-	chr2:119317465-119317503	chr2:119321153-119321204	0.248	0.04224843685
MXE	CPSF7	-	chr11:61421053-61421130	chr11:61421191-61421257	0.32	0.0423514077
MXE	ANXA2	-	chr15:60390212-60390317	chr15:60396150-60396427	0.449	0.04267432066
MXE	COG7	-	chr16:23418699-23418827	chr16:23424748-23424947	0.173	0.04308153903
MXE	PPP4R1L	-	chr20:58251756-58251864	chr20:58271362-58271505	0.273	0.04380838209
MXE	ZCWPW2	+	chr3:28478813-28478931	chr3:28492126-28492173	0.365	0.04454817012
MXE	TMEM14B	+	chr6:10749201-10749268	chr6:10770076-10770204	0.143	0.04701157616
MXE	SCP2	+	chr1:52948008-52948080	chr1:52950754-52950886	0.123	0.04701157616
MXE	FAM228B	+	chr2:24135118-24135187	chr2:24167626-24167683	0.377	0.04707829882
MXE	GK	+	chrX:30668011-30668118	chrX:30677374-30677452	0.34	0.0486093457
MXE	ATAD2B	-	chr2:23799824-23799993	chr2:23808988-23809176	0.258	0.04900841136
MXE	CCDC18-AS1	-	chr1:93315148-93315269	chr1:93318167-93318228	0.386	0.04922057688
MXE	CHKB	-	chr22:50579726-50579830	chr22:50580189-50580271	0.132	0.04985936646
RI	GAS5	-	chr1:173864256-173865282	NA	0.15	0
RI	GAS5	-	chr1:173865228-173865547	NA	0.436	0
RI	HLA-A	+	chr6:29944499-29945091	NA	0.911	0
RI	PCYT2	-	chr17:81906099-81906546	NA	0.195	0
RI	STX10	-	chr19:13144061-13144671	NA	0.17	0
RI	BAX	+	chr19:48960809-48961798	NA	0.233	0
RI	ZNF582-AS1	+	chr19:56398276-56399168	NA	0.809	0
RI	ENO2	+	chr12:6916670-6917107	NA	0.353	0
RI	FNBP1L	+	chr1:93550946-93554659	NA	0.47	0
RI	QKI	+	chr6:163563419-163570145	NA	0.602	3.34E-14
RI	EMD	+	chrX:154379941-154380367	NA	0.117	2.22E-13
RI	C5orf42	-	chr5:37139339-37142480	NA	0.11	3.58E-13
RI	TPT1	-	chr13:45340711-45341158	NA	0.802	4.16E-13
RI	NRBP2	-	chr8:143839508-143840030	NA	0.219	1.41E-12
RI	GAS5	-	chr1:173864483-173864704	NA	0.206	4.03E-10
RI	AP1G2	-	chr14:23563583-23563856	NA	0.577	6.09E-10
RI	CDK5RAP3	+	chr17:47974399-47975337	NA	0.199	6.86E-10
RI	SZT2	+	chr1:43448611-43450171	NA	0.464	1.23E-09
RI	MUS81	+	chr11:65865810-65866439	NA	0.242	2.06E-09
RI	PNISR	-	chr6:99403828-99404702	NA	0.328	6.32E-09
RI	RHOT2	+	chr16:672253-672825	NA	0.134	1.09E-08
RI	GAS5	-	chr1:173864256-173864506	NA	0.171	1.09E-08
RI	ARL6IP4	+	chr12:122981128-122981879	NA	0.401	1.61E-08
RI	TMEM198B	+	chr12:55830840-55831349	NA	0.33	1.84E-08
RI	ATAT1	+	chr6:30642767-30644679	NA	0.271	2.34E-08
RI	MAGED2	+	chrX:54815247-54816007	NA	0.171	3.60E-08
RI	MUS81	+	chr11:65864719-65865323	NA	0.2	4.10E-08
RI	TMEM94	+	chr17:75492928-75493593	NA	0.355	4.12E-08
RI	SZT2	+	chr1:43447848-43448484	NA	0.491	4.39E-08
RI	POMT1	+	chr9:131509902-131510415	NA	0.364	5.98E-08
RI	AP1G2	-	chr14:23563379-23563638	NA	0.518	9.00E-08
RI	CC2D1B	-	chr1:52360423-52361132	NA	0.202	1.15E-07
RI	COMMD7	-	chr20:32702702-32704071	NA	0.127	2.13E-07
RI	GOLGA2	-	chr9:128262562-128263092	NA	0.313	2.48E-07
RI	AP1G2	-	chr14:23563715-23564221	NA	0.412	3.17E-07
RI	RP13-794C1.1	+	chr3:48465486-48467645	NA	0.483	3.17E-07
RI	SYVN1	-	chr11:65132247-65132780	NA	0.109	3.46E-07
RI	PGGHG	+	chr11:290677-292095	NA	0.381	3.71E-07
RI	QKI	+	chr6:163563419-163570145	NA	0.403	4.78E-07
RI	MFSD10	-	chr4:2931374-2931936	NA	0.328	5.32E-07
RI	ATP5D	+	chr19:1244314-1244825	NA	0.328	7.42E-07
RI	DPM2	-	chr9:127936552-127937523	NA	0.183	9.37E-07
RI	WDR6	+	chr3:49011634-49014293	NA	0.245	9.44E-07
RI	TTC28-AS1	+	chr22:27997410-27998838	NA	0.378	9.64E-07
RI	CRELD1	+	chr3:9943076-9943515	NA	0.132	9.79E-07
RI	NID2	-	chr14:52005736-52006660	NA	0.884	1.29E-06
RI	DVL3	+	chr3:184164795-184165206	NA	0.14	1.86E-06
RI	MED22	-	chr9:133340904-133344333	NA	0.106	2.59E-06
RI	C21orf58	-	chr21:46301129-46302154	NA	0.265	3.09E-06
RI	EFEMP2	-	chr11:65870120-65870658	NA	0.135	3.56E-06
RI	EIF4A2	+	chr3:186784563-186786051	NA	0.153	4.57E-06
RI	CDK5RAP3	+	chr17:47973518-47974031	NA	0.107	6.43E-06
RI	PLCD4	+	chr2:218633604-218634221	NA	0.245	8.18E-06
RI	TMEM80	+	chr11:698868-700235	NA	0.241	9.73E-06
RI	IZUMO4	+	chr19:2098051-2098334	NA	0.667	1.07E-05
RI	TPM3	-	chr1:154170648-154172978	NA	0.14	1.13E-05
RI	FAM50A	+	chrX:154449680-154449931	NA	0.165	1.14E-05
RI	OGT	+	chrX:71544566-71548023	NA	0.104	1.60E-05
RI	RALGAPA1	-	chr14:35688458-35690003	NA	0.26	2.23E-05
RI	TPRN	-	chr9:137191616-137192365	NA	0.157	2.31E-05
RI	UBXN1	-	chr11:62676497-62677005	NA	0.114	5.34E-05
RI	WBP1	+	chr2:74459437-74459745	NA	0.268	5.64E-05
RI	CIRBP	+	chr19:1271980-1273172	NA	0.145	7.01E-05
RI	TCF4	-	chr18:55228846-55232671	NA	0.753	7.37E-05
RI	MUS81	+	chr11:65864719-65865145	NA	0.292	7.45E-05
RI	TJAP1	+	chr6:43503622-43506553	NA	0.22	7.65E-05
RI	ZNF517	+	chr8:144802869-144804238	NA	0.542	8.14E-05
RI	BDH2	-	chr4:103085348-103086540	NA	0.223	8.25E-05
RI	ERCC3	-	chr2:127289336-127289824	NA	0.142	8.44E-05
RI	SERHL2	+	chr22:42555663-42556083	NA	0.355	8.44E-05
RI	ACAA1	-	chr3:38126161-38126700	NA	0.156	8.83E-05
RI	ARL16	-	chr17:81682033-81682675	NA	0.167	8.83E-05
RI	CENPT	-	chr16:67829764-67830548	NA	0.195	8.95E-05
RI	U2AF1L4	-	chr19:35744322-35744738	NA	0.397	9.78E-05
RI	RPL12	-	chr9:127449609-127451405	NA	0.177	0.0001196031007
RI	HLA-F	+	chr6:29726010-29726526	NA	0.379	0.0001249613788
RI	PRPF31	+	chr19:54129056-54129370	NA	0.131	0.0001267919825
RI	PER3	+	chr1:7829836-7830161	NA	0.148	0.0001435736878
RI	AP1G2	-	chr14:23565605-23566160	NA	0.306	0.000143996723
RI	BEST1	+	chr11:61958145-61959578	NA	0.12	0.0001484207919
RI	TTLL3	+	chr3:9817644-9820741	NA	0.276	0.0001504685719
RI	PRPF4B	+	chr6:4056320-4057187	NA	0.118	0.0001702677729
RI	ARHGAP33	+	chr19:35787167-35788822	NA	0.324	0.0001837228863
RI	MAU2	+	chr19:19344848-19345369	NA	0.213	0.000191588729
RI	RNF213	+	chr17:80388611-80389367	NA	0.128	0.0002137736042
RI	CCDC183	+	chr9:136806503-136806867	NA	0.442	0.0002420757095
RI	PI4KAP2	-	chr22:21475210-21476764	NA	0.423	0.0002582586347
RI	SAT2	-	chr17:7627142-7627414	NA	0.136	0.0002695271887
RI	MEN1	-	chr11:64805634-64806368	NA	0.15	0.0002725309832
RI	WDR90	+	chr16:655057-655468	NA	0.76	0.0002730825593
RI	AP1G2	-	chr14:23562503-23563638	NA	0.27	0.000308896551
RI	PPIL2	+	chr22:21694936-21695496	NA	0.507	0.000377647128
RI	NAT9	-	chr17:74771959-74772277	NA	0.266	0.0003929852813
RI	TMEM198B	+	chr12:55832958-55833541	NA	0.151	0.0004005612502

RI	RRNAD1	+	chr1:156731990-156732473	NA	-0.303	0.000167746949
RI	MTX1	+	chr1:155210547-155212219	NA	-0.118	0.0001735682727
RI	RP11-295P9.3	+	chr10:13654352-13655482	NA	-0.475	0.0001735682727
RI	THOP1	+	chr19:2807441-2808444	NA	-0.64	0.0001902823536
RI	ELP5	+	chr17:7258587-7259940	NA	-0.113	0.0002144640796
RI	CDK20	-	chr9:87969193-87970897	NA	-0.259	0.0002155787581
RI	NIT2	+	chr3:100345584-100346255	NA	-0.108	0.0002330445727
RI	COMMD3	+	chr10:22318271-22318713	NA	-0.116	0.0002480556874
RI	RPP30	+	chr10:90894774-90895483	NA	-0.194	0.0002573288381
RI	TMEM208	+	chr16:67228796-67229278	NA	-0.109	0.0002668533231
RI	CACNB4	-	chr2:151870530-151872493	NA	-0.426	0.0002817565338
RI	NUDT17	+	chr1:145846432-145846690	NA	-0.388	0.0002993961125
RI	BTBD3	+	chr20:11917864-11918601	NA	-0.352	0.0003142423491
RI	B4GALNT1	-	chr12:57631199-57631364	NA	-0.155	0.000336563563
RI	TMEM138	+	chr11:61364249-61364518	NA	-0.186	0.0003481439514
RI	RBM41	-	chrX:107088435-107088839	NA	-0.104	0.0003658835225
RI	DDX46	+	chr5:134816429-134817714	NA	-0.105	0.0003968948543
RI	CENPT	-	chr16:67832011-67832545	NA	-0.43	0.0004376056157
RI	MYO15B	+	chr17:75590906-75591246	NA	-0.606	0.0004401943641
RI	CD274	+	chr9:5462833-5463121	NA	-0.167	0.0004473236184
RI	DPH1	+	chr17:2041767-2043424	NA	-0.39	0.0004699056745
RI	PHF7	+	chr3:52421647-52422881	NA	-0.515	0.0004977363804
RI	GNG5	-	chr1:84506010-84506565	NA	-0.566	0.0005832107347
RI	PLEKHH2	+	chr2:43710488-43712383	NA	-0.327	0.0005898240881
RI	ARHGEF40	+	chr14:21087000-21087463	NA	-0.404	0.0005943848687
RI	NUDT8	-	chr11:67628918-67629921	NA	-0.504	0.000634117199
RI	MOK	-	chr14:102232534-102233789	NA	-0.205	0.0006345139613
RI	WDR45	-	chrX:49076644-49077911	NA	-0.337	0.0006389898037
RI	TRABD	+	chr22:50197822-50198186	NA	-0.409	0.000651342608
RI	TCF4	-	chr18:55228846-55232671	NA	-0.136	0.000666479782
RI	RNH1	-	chr11:499014-499999	NA	-0.105	0.0006770723973
RI	NEPRO	-	chr3:113012733-113013403	NA	-0.103	0.0006844104956
RI	TIMM21	+	chr18:74155144-74155403	NA	-0.104	0.0006993832697
RI	BCL3	+	chr19:44758245-44758841	NA	-0.546	0.0007097597659
RI	B4GALT3	-	chr1:161176433-161177106	NA	-0.298	0.0007328348363
RI	CHKB	-	chr22:50580189-50580416	NA	-0.177	0.0007579141903
RI	ATP5SL	-	chr19:41431318-41433434	NA	-0.28	0.0007734376691
RI	LIN37	+	chr19:35753086-35754157	NA	-0.312	0.0007935197621
RI	RPL3	-	chr22:39313190-39314208	NA	-0.286	0.0007965341189
RI	MED23	-	chr6:131592387-131593171	NA	-0.386	0.0008916825081
RI	PIDD1	-	chr11:802681-803647	NA	-0.317	0.001045349009
RI	CCDC57	-	chr17:82193755-82194139	NA	-0.603	0.001120005346
RI	RPS9	+	chr19:54206275-54206724	NA	-0.234	0.001162103423
RI	QTRT1	+	chr19:10701949-10702254	NA	-0.138	0.001162103423
RI	MIF4GD	-	chr17:75267745-75268192	NA	-0.123	0.001166852681
RI	MINK1	+	chr17:4889646-4890735	NA	-0.431	0.001258786667
RI	HARS2	+	chr5:140693490-140693665	NA	-0.235	0.001275168466
RI	C2orf49	+	chr2:105342847-105343223	NA	-0.14	0.001365874141
RI	ACAD11	-	chr3:132578795-132579558	NA	-0.11	0.00157883001
RI	CCAR2	+	chr8:22619149-22620468	NA	-0.169	0.001791555223
RI	LMNA	+	chr1:156137653-156138757	NA	-0.152	0.001867343234
RI	BTN3A2	+	chr6:26373275-26373413	NA	-0.101	0.001925892701
RI	ARL6IP4	+	chr12:122981128-122981879	NA	-0.104	0.001961330751
RI	GNRHR2	+	chr1:145919343-145919900	NA	-0.257	0.002073595908
RI	TTC8	+	chr14:88841036-88841514	NA	-0.153	0.002168953809
RI	ORMDL1	-	chr2:189782421-189783123	NA	-0.168	0.002200528562
RI	DDX39A	-	chr19:14409741-14410334	NA	-0.119	0.002219711602
RI	C19orf66	+	chr19:10091249-10091630	NA	-0.243	0.002219711602
RI	ADARB1	+	chr21:45222017-45226560	NA	-0.208	0.002219711602
RI	DUS1L	-	chr17:82061208-82061721	NA	-0.208	0.002439817932
RI	POP5	-	chr12:120579513-120579923	NA	-0.106	0.002482369946
RI	RPAIN	+	chr17:5425970-5428211	NA	-0.106	0.002482770348
RI	SAMD4A	+	chr14:54784339-54784620	NA	-0.417	0.002500459043
RI	NSUN5	-	chr7:73307582-73308553	NA	-0.246	0.002672952032
RI	NAPEPLD	-	chr7:103099775-103103554	NA	-0.376	0.002761239845
RI	KCNAB3	-	chr17:7923967-7924265	NA	-0.341	0.002842735607
RI	ABTB1	+	chr3:127676535-127677286	NA	-0.262	0.002857952109
RI	POLD1	+	chr19:50401998-50402373	NA	-0.509	0.003199637451
RI	CYB5R2	-	chr11:7668477-7672523	NA	-0.382	0.003244746418
RI	RBM33	+	chr7:155738059-155740026	NA	-0.159	0.003244746418
RI	RCHY1	-	chr4:75490580-75491782	NA	-0.482	0.003299762828
RI	RP11-435I10.4	-	chr16:28482476-28482672	NA	-0.18	0.003804598835
RI	RABGGTA	-	chr14:24270836-24271169	NA	-0.487	0.004579326249
RI	C18orf8	+	chr18:23530228-23530612	NA	-0.144	0.004901189048
RI	IMP4	+	chr2:130342893-130343194	NA	-0.588	0.004901189048
RI	FAM3A	-	chrX:154515759-154516242	NA	-0.192	0.00491760265
RI	TJAP1	+	chr6:43500743-43501687	NA	-0.124	0.005092959178
RI	CAD	+	chr2:27233311-27233808	NA	-0.17	0.005154888944
RI	MRNIP	-	chr5:179837275-179840959	NA	-0.163	0.005154888944
RI	SCYL1	+	chr11:65538269-65538704	NA	-0.15	0.005191038741
RI	RUSC1	+	chr1:155325863-155327132	NA	-0.221	0.005394362141
RI	AKNA	-	chr9:114343707-114346009	NA	-0.174	0.005410896005
RI	POLD1	+	chr19:50415723-50416528	NA	-0.266	0.005472713134
RI	NAGK	+	chr2:71075554-71076701	NA	-0.162	0.006239792161
RI	POLR1C	+	chr6:43519705-43520185	NA	-0.137	0.006280059241
RI	CLK4	-	chr5:178616881-178618778	NA	-0.325	0.00639004427
RI	PCNX2	-	chr1:232984135-232986540	NA	-0.155	0.006928555429
RI	SLC9A5	+	chr16:67255392-67255930	NA	-0.326	0.0072366921
RI	PIDD1	-	chr11:800761-801120	NA	-0.135	0.007244724238
RI	DET1	-	chr15:88530622-88531715	NA	-0.216	0.007987937871
RI	ARAP1	-	chr11:72712437-72713243	NA	-0.108	0.008307631995
RI	MIIP	+	chr1:12029222-12030124	NA	-0.328	0.008465546508
RI	U2AF1L4	-	chr19:35744322-35745187	NA	-0.332	0.0095896582
RI	PNKP	-	chr19:49864178-49864403	NA	-0.142	0.0095896582
RI	AFTPH	+	chr2:64579485-64581273	NA	-0.262	0.009886221094
RI	GALE	-	chr1:23796508-23796782	NA	-0.111	0.01001845849
RI	TANC1	+	chr2:159219237-159219867	NA	-0.182	0.01003146764
RI	CC2D1B	-	chr1:52357780-52358461	NA	-0.203	0.01047160473
RI	SREBF1	-	chr17:17817547-17817916	NA	-0.223	0.01127882976
RI	ORC2	-	chr2:200913294-200913992	NA	-0.167	0.01229678921
RI	CDCA7	+	chr2:173363817-173364989	NA	-0.16	0.01251638125
RI	NELFE	-	chr6:31954554-31955096	NA	-0.139	0.01266779508
RI	TEN1-CDK3	+	chr17:76002510-76003398	NA	-0.193	0.01292108692
RI	BPHL	+	chr6:3138040-3140509	NA	-0.198	0.01313973159
RI	DERL2	-	chr17:5481100-5481389	NA	-0.118	0.01314365759
RI	INTS11	-	chr1:1312225-1312700	NA	-0.462	0.01464113754
RI	NPHP1	-	chr2:110163047-110164730	NA	-0.268	0.01528618861
RI	LLGL1	+	chr17:18235469-18236760	NA	-0.389	0.01561183787
RI	MAEA	+	chr4:1336860-1338617	NA	-0.323	0.01580040133
RI	SPATA7	+	chr14:88427629-88429463	NA	-0.114	0.01608781478
RI	STAT2	-	chr12:56354777-56355351	NA	-0.129	0.0174877641
RI	ALDH3B1	+	chr11:68015296-68018637	NA	-0.385	0.01778902412
RI	NEK8	+	chr17:28737305-28737736	NA	-0.113	0.0180385365
RI	RBM25	+	chr14:73110830-73111802	NA	-0.103	0.01817881467
RI	CHKB	-	chr22:50581748-50582854	NA	-0.109	0.01817881467
RI	FBF1	-	chr17:75914121-75914932	NA	-0.219	0.01867064045
RI	N6AMT1	-	chr21:28872190-28876491	NA	-0.205	0.01873364361
RI	NECAB3	-	chr20:33659884-33660395	NA	-0.22	0.01873719104
RI	WRAP73	-	chr1:3630766-3631657	NA	-0.219	0.0189131366
RI	IKBKB	+	chr8:42319269-42320844	NA	-0.132	0.01928890763
RI	WDR45	-	chrX:49076644-49077911	NA	-0.12	0.01992583731

EEF1E1-BLOC1S5
EEF2KMT
EFCAB13
EFCAB14
EFEMP2
EFHC1
EFNA1
EHBP1
EIF1AX
EIF2B5
EIF2D
EIF3E
EIF4A2
EIF4G3
ELF2
ELFN2
ELMOD3
ELOVL5
ELP2
ELP5
EMC10
EMC3-AS1
EMC8
EMD
EME1
EMILIN2
EML1
EMSY
ENDOV
ENGASE
ENO2
ENO3
ENOSF1
ENSA
ENTPD1
ENTPD5
ENTPD6
EOGT
EP400NL
EPB41
EPB41L1
EPB41L2
EPS15
EPS8
EPS8L2
ERBB2
ERBIN
ERCC1
ERCC3
ERCC6L2
ERLEC1
ERMARD
ESCO2
ESYT2
ETFA
ETFDH
ETNK1
ETNK2
ETV1
ETV4
EVI5
EVI5L
EWSR1
EXO1
EXO5
EXOC7
EXOSC10
EXOSC3
EXOSC8
EXOSC9
EYA3
EZH2
FAAP20
FADS3
FAH
FAHD2A
FAM107B
FAM111A
FAM111B
FAM114A2
FAM118B
FAM120B
FAM120C
FAM122B
FAM122C
FAM126B
FAM131A
FAM134C
FAM135A
FAM13B
FAM161A
FAM168A
FAM173B
FAM175A
FAM189B
FAM192A
FAM193B
FAM200B
FAM204A
FAM208A
FAM208B
FAM210A
FAM219B
FAM221A
FAM227B
FAM228B
FAM24B
FAM35A
FAM3A
FAM45A
FAM49B
FAM50A
FAM53C
FAM66C
FAM76B
FAM86B3P
FAM86C1
FAM86DP
FAM86EP
FAM86FP
FAM92A

RI	ASAH1	-	chr8:18064456-18067298	NA	0.112	0.0004079568349	RI	CAPRIN2	-	chr12:30719078-30720915	NA	-0.116	0.02160256185	FAM98A
RI	TIAL1	-	chr10:119576610-119577203	NA	0.175	0.0004445519329	RI	ZNF10	+	chr12:133130610-133131014	NA	-0.378	0.02210096143	FANCA
RI	RGL2	-	chr6:33293794-33294066	NA	0.105	0.000485827391	RI	ABHD11	-	chr7:73737220-73737735	NA	-0.285	0.0236309712	FANCD2
RI	RBM5	+	chr3:50100531-50103166	NA	0.214	0.0005495594221	RI	ZSWIM7	-	chr17:15977578-15977913	NA	-0.191	0.02434367906	FANCI
RI	ATP5G1	+	chr17:48893408-48895334	NA	0.256	0.0005832107347	RI	ABHD14A-ACY1	+	chr3:51987555-51988603	NA	-0.115	0.02534479944	FANCL
RI	C11orf49	+	chr11:47161126-47162247	NA	0.261	0.0006560085309	RI	YDJC	-	chr22:21629307-21629761	NA	-0.158	0.02566034074	FASTK
RI	WBP1	+	chr2:74459437-74459745	NA	0.226	0.0007736868659	RI	PTGES2	-	chr9:128122361-128123134	NA	-0.117	0.02567180123	FASTKD1
RI	CDK11A	-	chr1:1703475-1703940	NA	0.208	0.0007969360077	RI	NKTR	+	chr3:42619019-42619708	NA	-0.12	0.02667604946	FAT1
RI	BBS1	+	chr11:66531655-66532219	NA	0.388	0.0009446880095	RI	MAMDC4	+	chr9:136856463-136856826	NA	-0.348	0.0271123135	FAXDC2
RI	HSF4	+	chr16:67165521-67165846	NA	0.187	0.0009475934077	RI	BRAT1	-	chr7:2539785-2541052	NA	-0.181	0.02743375478	FBF1
RI	CCNK	+	chr14:99500765-99502376	NA	0.166	0.0009695331436	RI	FAM200B	+	chr4:15686235-15687148	NA	-0.143	0.02854546606	FBRS
RI	TMEM175	+	chr4:948115-948615	NA	0.244	0.001050415336	RI	ZNF232	-	chr17:5108925-5109868	NA	-0.142	0.02997087908	FBXL12
RI	XPNPEP1	-	chr10:109875527-109877867	NA	0.116	0.001120005346	RI	DECR2	+	chr16:406684-406952	NA	-0.175	0.03089148023	FBXL2
RI	BTN3A2	+	chr6:26367989-26368267	NA	0.308	0.001170885484	RI	DALRD3	-	chr3:49015972-49016340	NA	-0.234	0.03426009204	FBXL4
RI	NPR2	+	chr9:35808508-35808853	NA	0.544	0.001284150908	RI	ATG16L1	+	chr2:233289853-233290353	NA	-0.153	0.03527976138	FBXL6
RI	MOV10	+	chr1:112698286-112699810	NA	0.143	0.001314429458	RI	HMBS	+	chr11:119092124-119092523	NA	-0.227	0.03751352006	FBXO22
RI	CTC1	-	chr17:8229890-8230468	NA	0.204	0.001464590268	RI	NAPRT	-	chr8:143576077-143576572	NA	-0.331	0.03947238461	FBXO24
RI	RELT	+	chr11:73394235-73394734	NA	0.126	0.001605230086	RI	EOGT	-	chr3:68988505-68989017	NA	-0.162	0.04266975874	FBXO38
RI	ING4	-	chr12:6652270-6652770	NA	0.184	0.001649350636	RI	C11orf57	+	chr11:112074315-112074916	NA	-0.109	0.04557377175	FBXO44
RI	NOP56	+	chr20:2657080-2658393	NA	0.109	0.001723300719	RI	HDLBP	-	chr2:241268953-241269295	NA	-0.137	0.04657411543	FBXO9
RI	DGKA	+	chr12:55940081-55940722	NA	0.103	0.001744039268	RI	DCXR	-	chr17:82036858-82037547	NA	-0.268	0.04849882675	FCHSD1
RI	HCN3	+	chr1:155283973-155284757	NA	0.266	0.001850082181	RI	HDAC6	+	chrX:48814961-48815490	NA	-0.158	0.04960482811	FDFT1
RI	ZNF76	+	chr6:35286127-35286399	NA	0.123	0.001893949787	RI	PILRB	+	chr7:100353372-100354012	NA	-0.115	0.04986683364	FECH
RI	ABCB6	-	chr2:219210380-219210823	NA	0.538	0.001910688849	SE	ADGRG6	+	chr6:142383759-142383843	NA	-0.871	0	FERMT2
RI	HTRA2	+	chr2:74531338-74531702	NA	0.13	0.001981135288	SE	HMGCR	+	chr5:75355055-75355214	NA	-0.112	0	FEZ2
RI	DHRS1	-	chr14:24292183-24292784	NA	0.237	0.002376863758	SE	BRD9	-	chr5:868606-868804	NA	-0.151	0	FGFR1OP2
RI	ELMOD3	+	chr2:85355075-85357252	NA	0.322	0.002439817932	SE	SDHAP2	+	chr3:195663623-195663767	NA	-0.572	0	FGFR2
RI	ZSWIM8	+	chr10:73794146-73794639	NA	0.284	0.002500567157	SE	NUMB	-	chr14:73279280-73279424	NA	-0.272	0	FHL1
RI	MIB2	+	chr1:1628018-1628399	NA	0.252	0.002590851266	SE	COA1	-	chr7:43650611-43650712	NA	-0.256	0	FHL2
RI	PDZD7	-	chr10:101021797-101022385	NA	0.162	0.002698189981	SE	COA1	-	chr7:43650492-43650712	NA	-0.171	0	FHOD3
RI	VPS28	-	chr8:144423616-144424132	NA	0.101	0.002759449052	SE	FIS1	-	chr7:101244006-101244139	NA	-0.152	0	FIP1L1
RI	ASPSCR1	+	chr17:82016799-82017113	NA	0.125	0.002774257415	SE	DPH3	-	chr3:16264154-16264229	NA	-0.651	0	FIRRE
RI	PPOX	+	chr1:161170906-161171218	NA	0.241	0.003244746418	SE	SYTL2	-	chr11:85711112-85711232	NA	-0.66	0	FIS1
RI	RNF215	-	chr22:30379481-30379780	NA	0.152	0.003341924059	SE	OSBPL6	+	chr2:178339671-178339764	NA	-0.635	0	FKBP10
RI	DDIT3	-	chr12:57517268-57517753	NA	0.147	0.00342269305	SE	DCUN1D4	+	chr4:51887122-51887244	NA	-0.285	0	FKBP1B
RI	JMJD8	-	chr16:683321-683598	NA	0.146	0.004143798929	SE	ANAPC11	+	chr17:81899241-81899542	NA	-0.106	0	FKTN
RI	ING4	-	chr12:6652661-6653050	NA	0.142	0.004201701249	SE	HMGXB4	+	chr22:35263981-35264096	NA	-0.501	0	FLCN
RI	ARL6IP4	+	chr12:122981128-122981879	NA	0.229	0.00421474628	SE	SLMAP	+	chr3:57907883-57908006	NA	-0.337	0	FLOT1
RI	CDK11A	-	chr1:1704222-1704655	NA	0.134	0.004818730187	SE	SLC3A2	+	chr11:62870744-62870837	NA	-0.475	0	FLT3LG
RI	TNFRSF25	-	chr1:6462862-6463127	NA	0.18	0.004890547527	SE	ANAPC16	+	chr10:72220053-72220379	NA	-0.512	0	FLVCR1
RI	SNHG17	-	chr20:38421005-38422241	NA	0.299	0.004901189048	SE	ANAPC16	+	chr10:72223887-72224056	NA	-0.218	0	FLYWCH1
RI	MOSPD3	+	chr7:100612729-100613269	NA	0.105	0.005192877926	SE	FOXM1	-	chr12:2865354-2865399	NA	-0.4	0	FNBP1
RI	CDK11A	-	chr1:1704038-1704344	NA	0.116	0.00555582277	SE	PCTP	+	chr17:55787565-55787654	NA	-0.874	0	FNBP1L
RI	U2AF1L4	-	chr19:35744322-35745187	NA	0.282	0.005964038928	SE	ELOVL5	-	chr6:53273219-53273344	NA	-0.12	0	FNBP4
RI	ATP13A1	-	chr19:19656659-19656916	NA	0.138	0.006156541073	SE	DENND4C	+	chr9:19336685-19336832	NA	-0.52	0	FNIP2
RI	FBXL6	-	chr8:144356299-144356713	NA	0.333	0.00642391228	SE	ASPH	-	chr8:61646749-61646878	NA	-0.112	0	FNTB
RI	PRMT2	+	chr21:46660832-46661936	NA	0.205	0.006535676432	SE	CENPK	-	chr5:65551105-65551242	NA	-0.156	0	FOPNL
RI	EFHC1	+	chr6:52452687-52454287	NA	0.172	0.006772555752	SE	SEPT2	+	chr2:241324408-241324662	NA	-0.718	0	FOXD3-AS1
RI	IL17RC	+	chr3:9923880-9924291	NA	0.18	0.007244724238	SE	JAM2	+	chr21:25711366-25711568	NA	-0.238	0	FOXK2
RI	ZSWIM8	+	chr10:73794146-73794639	NA	0.147	0.007527884364	SE	STX2	-	chr12:130795994-130796120	NA	-0.43	0	FOXM1
RI	RBM8A	-	chr1:145926808-145927077	NA	0.208	0.007696141702	SE	RPLP0	-	chr12:120198553-120198739	NA	-0.112	0	FOXRED1
RI	C1orf35	-	chr1:228102079-228102382	NA	0.111	0.007696141702	SE	RPLP0	-	chr12:120198553-120198631	NA	-0.14	0	FRG1
RI	C21orf59	-	chr21:32601666-32603292	NA	0.204	0.007746832051	SE	RPLP0	-	chr12:120198553-120198770	NA	-0.125	0	FRG1BP
RI	SAT2	-	chr17:7627362-7627802	NA	0.127	0.007818289167	SE	TCF25	+	chr16:89878460-89878627	NA	-0.179	0	FRG1CP
RI	TUBGCP6	-	chr22:50219287-50219791	NA	0.225	0.007846724902	SE	FKTN	+	chr9:105574197-105574332	NA	-0.692	0	FRG1HP
RI	PAN2	-	chr12:56319087-56319487	NA	0.186	0.008317953933	SE	ESYT2	-	chr7:158752780-158752843	NA	-0.166	0	FRS2
RI	LIN37	+	chr19:35754245-35754519	NA	0.239	0.00873127551	SE	MAP4	-	chr3:47867245-47867338	NA	-0.246	0	FUBP1
RI	RP11-252A24.2	-	chr16:74352039-74352378	NA	0.16	0.009084561186	SE	MAP4	-	chr3:47869213-47869327	NA	-0.264	0	FUK
RI	PIAS3	-	chr1:145848521-145850269	NA	0.106	0.0095896582	SE	EIF4A2	+	chr3:186784961-186785101	NA	-0.29	0	FUS
RI	FADS3	-	chr11:61876358-61876963	NA	0.185	0.009886221094	SE	IMMP1L	-	chr11:31473723-31473786	NA	-0.43	0	FUT8
RI	EWSR1	+	chr22:29298732-29299851	NA	0.181	0.009886221094	SE	FEZ2	-	chr2:36560784-36560865	NA	-0.225	0	FUZ
RI	CUL9	+	chr6:43196068-43196862	NA	0.205	0.009992530522	SE	RPL17-C18orf32	-	chr18:49488499-49488566	NA	-0.596	0	FXR1
RI	CCDC150	+	chr2:196729192-196729861	NA	0.155	0.00999475111	SE	PTPMT1	+	chr11:47569699-47569891	NA	-0.32	0	FYN
RI	AP1G2	-	chr14:23561511-23562415	NA	0.266	0.01014875315	SE	INTS3	+	chr1:153761019-153761109	NA	-0.275	0	FZR1
RI	CCDC159	+	chr19:11354579-11354943	NA	0.208	0.01046368681	SE	KIF13A	-	chr6:17789871-17789910	NA	-0.706	0	GAA
RI	POMZP3	-	chr7:76618182-76618615	NA	0.259	0.01055453037	SE	RAI14	+	chr5:34813573-34813660	NA	-0.649	0	GAB1
RI	LDLRAP1	+	chr1:25563660-25565207	NA	0.131	0.01068294572	SE	MTIF2	-	chr2:55266380-55266518	NA	-0.23	0	GABBR1
RI	ADD1	+	chr4:2908514-2909431	NA	0.284	0.01160406502	SE	SGCE	-	chr7:94599696-94599723	NA	-0.512	0	GABPB1
RI	NR2C2	+	chr3:15037999-15039227	NA	0.148	0.01160759958	SE	EXO1	+	chr1:241849108-241849216	NA	-0.555	0	GABPB2
RI	USP21	+	chr1:161164533-161164942	NA	0.162	0.01262240708	SE	MFF	+	chr2:227329605-227329783	NA	-0.159	0	GAK
RI	HSF4	+	chr16:67169278-67169945	NA	0.151	0.01288428282	SE	ARHGEF9	-	chrX:63656509-63658064	NA	-0.148	0	GALE
RI	PLXNB1	-	chr3:48410454-48411036	NA	0.641	0.01297814223	SE	PICALM	-	chr11:85990249-85990399	NA	-0.458	0	GALNT7
RI	EFNA1	+	chr1:155133502-155133780	NA	0.136	0.01309155262	SE	PICALM	-	chr11:85990249-85990378	NA	-0.387	0	GAMT
RI	DSN1	-	chr20:36770872-36771473	NA	0.155	0.01437449705	SE	ARIH2	+	chr3:48945135-48945181	NA	-0.852	0	GAPVD1
RI	ULK3	-	chr15:74842074-74842420	NA	0.109	0.0146694743	SE	SDHAP1	-	chr3:195968218-195968385	NA	-0.49	0	GARNL3
RI	MUTYH	-	chr1:45332762-45333324	NA	0.151	0.01473339108	SE	MCM3	-	chr6:52265234-52265385	NA	-0.335	0	GAS5
RI	SYVN1	-	chr11:65130001-65130379	NA	0.323	0.01538941007	SE	SLCO4A1	+	chr20:62668011-62668184	NA	-0.3	0	GAS8
RI	TMEM234	-	chr1:32216196-32217351	NA	0.169	0.01538941007	SE	ERBIN	+	chr5:66075023-66075230	NA	-0.523	0	GATAD2A
RI	DDX11	+	chr12:31103576-31104791	NA	0.21	0.01676346818	SE	FRG1BP	+	chr20:30391196-30391308	NA	-0.796	0	GATC
RI	FBXO9	+	chr6:53092733-53093561	NA	0.135	0.01685875146	SE	YPEL5	+	chr2:30148244-30148541	NA	-0.348	0	GBA
RI	ZNF7	+	chr8:144829042-144829604	NA	0.209	0.01867064045	SE	YPEL5	+	chr2:30148244-30148305	NA	-0.242	0	GCSH
RI	MAGED4	+	chrX:52186396-52187845	NA	0.307	0.01903661337	SE	APBB2	-	chr4:40935076-40935139	NA	-0.711	0	GDAP2
RI	EIF2B5	+	chr3:184143051-184143565	NA	0.105	0.01970885861	SE	ITGB3BP	-	chr1:63510064-63510181	NA	-0.17	0	GEMIN4
RI	TMEM218	-	chr11:125102131-125102809	NA	0.156	0.02020371516	SE	EHBP1	+	chr2:62987930-62988038	NA	-0.692	0	GEMIN8
RI	FAM219B	-	chr15:74899991-74902786	NA	0.109	0.02030767124	SE	C2orf74	+	chr2:61162841-61162955	NA	-0.143	0	GFM2
RI	TTC31	+	chr2:74490024-74490473	NA	0.117	0.02129946588	SE	ADD3	+	chr10:110132304-110132400	NA	-0.694	0	GGACT
RI	DXO	-	chr6:31970605-31971147	NA	0.165	0.02210643183	SE	RPL17	-	chr18:49491735-49491833	NA	-0.518	0	GGCX
RI	WDR24	-	chr16:685868-686186	NA	0.15	0.02211554839	SE	EXOSC3	-	chr9:37781985-37782137	NA	-0.295	0	GGT1
RI	KIAA1217	+	chr10:24544980-24547843	NA	0.335	0.02220739931	SE	ADGRG6	+	chr6:142440907-142440953	NA	-0.595	2.89E-14	GIGYF2
RI	PCSK7	-	chr11:117223207-117224216	NA	0.199	0.02272113874	SE	NQO2	+	chr6:3004494-3004651	NA	-0.183	2.89E-14	GINS3
RI	BRF1	-	chr14:105211121-105212164	NA	0.167	0.0236309712	SE	CRBN	-	chr3:3175162-3175266	NA	-0.303	2.89E-14	GIT2
RI	MOV10	+	chr1:112698286-112698789	NA	0.132	0.02573388838	SE	PCYT2	-	chr17:81907203-81907257	NA	-0.221	2.89E-14	GK
RI	BBS1	+	chr11:66515692-66515933	NA	0.106	0.02792705927	SE	NADK	-	chr1:1756778-1756882	NA	-0.34	2.89E-14	GLMP
RI	NICN1	-	chr3:49424948-49425438	NA	0.264	0.02849421127	SE	PPA2	-	chr4:105456680-105456745	NA	-0.126	2.89E-14	GLS
RI	CENPT	-	chr16:67829764-67831358	NA	0.126	0.03113327069	SE	CRBN	-	chr3:3175162-3175269	NA	-0.193	5.59E-14	GLT8D1
RI	CCDC189	-	chr16:30759011-30759247	NA	0.176	0.03403927258	SE	SPAST	+	chr2:32098795-32098891	NA	-0.697	5.59E-14	GMIP
RI	ACADVL	+	chr17:7224320-7224552	NA	0.266	0.03643105134	SE	ZFAS1	+	chr20:49280484-49280570	NA	-0.112	5.59E-14	GNAS
RI	TRIM22	+	chr11:5697247-5698545	NA	0.236	0.03643105134	SE	TMPO	+	chr12:98544441-98544537	NA	-0.257	5.59E-14	GNE
RI	OBSL1	-	chr2:219550728-219551798	NA	0.388	0.03714903893	SE	NADK	-	chr1:1756738-1756882	NA	-0.352	5.59E-14	GNG5
RI	ZFYVE19	+	chr15:40813942-40814569	NA	0.164	0.03791551547	SE	TEAD1	+	chr11:12878888-12878900	NA	-0.599	8.25E-14	GNPDA1
RI	CDK10	+	chr16:89693273-89693467	NA	0.13	0.03804967276	SE	WBP1	+	chr2:74458831-74458919	NA	-0.968	1.34E-13	GNRHR2
RI	SLC12A4	-	chr16:67945086-67945553	NA	0.105	0.03906439299	SE	BAG6	-	chr6:31639499-31639646	NA	-0.338	1.58E-13	GOLGA2
RI	SAT2	-	chr17:7627362-7627805	NA	0.124	0.04004330286	SE	PSMA3-AS1	-	chr14:58293065-58293138	NA	-0.417	1.58E-13	GOLGA4
RI	DDIT3	-	chr12:57517268-57517753	NA	0.12	0.04122650279	SE	EMC8	-	chr16:85780378-85780473	NA	-0.164	1.58E-13	GOLGA6L9
RI	CELSR3	-	chr3:48641850-48642467	NA	0.33	0.04364710769	SE	PPRC1	+	chr10:102146671-102147392	NA	-0.347	2.35E-13	GOLGB1
RI	CAPRIN2	-	chr12:30714958-30716676	NA	0.173	0.04460971474	SE	HNRNPAB	+	chr5:178210131-178210272	NA	-0.136	2.85E-13	GOLT1B
RI	CCDC189	-	chr16:30759341-30759724	NA	0.231	0.04587055951	SE	CTD-2116N17.1	-	chr15:64376742-64376905	NA	-0.113	3.81E-13	GORAB
RI	PSME1	+	chr14:24136984-24137431	NA	0.265	0.04795993741	SE	IMMP1L	-	chr11:31473723-31473786	NA	-0.512	3.81E-13	GOSR2
SE	BCAS4	+	chr20:50840536-50840930	NA	0.605	0	SE	MGEA5	-	chr10:101793912-101793998	NA	-0.159	4.00E-13	GPANK1
SE	TTC13	-	chr1:230921420-230921504	NA	0.993	0	SE	CPT1B	-	chr22:50573807-50573921	NA	-0.61	4.70E-13	GPATCH2
SE	FNBP1	-	chr9:129923843-129923996	NA	0.541	0	SE	XPNPEP3	+	chr22:40860677-40860793	NA	-0.881	5.32E-13	GPATCH8
SE	FNBP1	-	chr9:129923843-129924026	NA	0.604	0	SE	TCEAL8	-	chrX:103254604-103254677	NA	-0.212	6.41E-13	GPBP1
SE	CAST	+	chr5:96726793-96726859	NA	0.698	0	SE	PRPF4B	+	chr6:4056526-4056688	NA	-0.255	6.63E-13	GPBP1L1
SE	CAST	+	chr5:96726750-96726859	NA	0.557	0	SE	PPRC1	+	chr10:102145019-102145090	NA	-0.558	7.52E-13	GPCPD1
SE	ST20	-	chr15:79907628-79907684	NA	1	0	SE	TMEM161B-AS1	+	chr5:88287435-88287622	NA	-0.534	8.81E-13	GPD2
SE	SPAG9	-	chr17:50975862-50975901	NA	0.73	0	SE	MRPL47	-	chr3:179602651-179602737	NA	-0.139	1.07E-12	GPM6A
SE	FHL1	+	chrX:136209242-136209442	NA	0.176	0	SE	SMC4	+	chr3:160412529-160412849	NA	-0.105	1.36E-12	GPN3
SE	SNRNP70	+	chr19:49102113-49103587	NA	0.141	0	SE	PARD3	-	chr10:34336198-34336243	NA	-0.427	1.53E-12	GPR155
SE	MXRA7	-	chr17:76683846-76683927	NA	0.604	0	SE	EXO1	+	chr1:241849111-241849216	NA	-0.46	1.86E-12	GPR157
SE	TBC1D9B	-	chr5:179867444-179867495	NA	0.158	0	SE	DENND5A	-	chr11:9176771-9176902	NA	-0.13	3.99E-12	GPR158
SE	FAM98A	-	chr2:33592079-33592214	NA	0.153	0	SE	CBY1	+	chr22:38665545-38665685	NA	-0.274	5.38E-12	GPR180

SE	HMGN1	-	chr21:39345829-39345958	NA	0.417	0	SE	NONO	+	chrX:71284983-71285097	NA	-0.185	7.49E-12	GPX4
SE	HMGN1	-	chr21:39345829-39347292	NA	0.323	0	SE	DOCK7	-	chr1:62544946-62545039	NA	-0.402	7.63E-12	GRAMD4
SE	ADRM1	+	chr20:62304460-62304577	NA	0.131	0	SE	COA1	-	chr7:43650611-43650712	NA	-0.536	8.02E-12	GRB10
SE	CLSTN1	-	chr1:9737497-9737554	NA	0.288	0	SE	KIAA1468	+	chr18:62280359-62280442	NA	-0.631	9.58E-12	GRHPR
SE	CLSTN1	-	chr1:9756480-9756510	NA	0.516	0	SE	SNU13	-	chr22:41682355-41682587	NA	-0.101	1.11E-11	GS1-124K5.12
SE	NQO1	-	chr16:69714963-69715077	NA	0.647	0	SE	EIF2D	-	chr1:206599462-206599612	NA	-0.125	1.15E-11	GSAP
SE	ABI1	-	chr10:26771074-26771089	NA	0.644	0	SE	ZC3H11A	+	chr1:203796292-203796496	NA	-0.271	1.93E-11	GSKIP
SE	ABI1	-	chr10:26755654-26755741	NA	0.371	0	SE	MAP4K4	+	chr2:101860824-101860986	NA	-0.35	1.98E-11	GSN
SE	PRPF40A	-	chr2:152679055-152679109	NA	0.309	0	SE	ATRX	-	chrX:77595711-77595785	NA	-0.196	2.07E-11	GTF2E2
SE	PIN4	+	chrX:72186460-72186534	NA	0.25	0	SE	SEC24B	+	chr4:109481676-109481781	NA	-0.425	2.17E-11	GTF2IP1
SE	PIN4	+	chrX:72186233-72186534	NA	0.554	0	SE	TCOF1	+	chr5:150379228-150379408	NA	-0.127	2.19E-11	GTF2IP12
SE	CCDC88A	-	chr2:55303068-55303152	NA	0.561	0	SE	MAPKAPK5-AS1	-	chr12:111840332-111840477	NA	-0.213	3.75E-11	GTPBP10
SE	RAPGEF2	+	chr4:159344035-159344059	NA	0.824	0	SE	GTF2E2	-	chr8:30612298-30612481	NA	-0.115	3.77E-11	GTSE1
SE	TVP23C-CDRT	-	chr17:15540432-15540561	NA	0.897	0	SE	SNHG5	-	chr6:85677790-85677875	NA	-0.106	4.04E-11	GUCD1
SE	RPRD2	+	chr1:150441880-150441958	NA	0.591	0	SE	ERLEC1	+	chr2:53808298-53808460	NA	-0.181	4.53E-11	GUF1
SE	SUMF2	+	chr7:56074173-56074218	NA	0.13	0	SE	METTL2B	+	chr7:128477081-128477173	NA	-0.255	4.97E-11	GUK1
SE	SUMF2	+	chr7:56074113-56074218	NA	0.315	0	SE	ZFAND6	+	chr15:80098415-80098578	NA	-0.352	5.86E-11	GULP1
SE	CMC2	-	chr16:81001247-81001334	NA	0.202	0	SE	AC074286.1	-	chr2:177332141-177332251	NA	-0.616	6.59E-11	GUSB
SE	DST	-	chr6:56463045-56463156	NA	0.309	0	SE	SEPT7P2	-	chr7:45728831-45729026	NA	-0.255	7.82E-11	GUSBP11
SE	DST	-	chr6:56468981-56468999	NA	0.827	0	SE	SEPT6	-	chrX:119625334-119625379	NA	-0.275	8.27E-11	GZF1
SE	KIF21A	-	chr12:39330241-39330262	NA	0.766	0	SE	TMEM161B	-	chr5:88240812-88240916	NA	-0.239	8.98E-11	H2AFY
SE	RSRP1	-	chr1:25244453-25244498	NA	0.639	0	SE	DUSP18	-	chr22:30664954-30665121	NA	-0.738	1.14E-10	HACE1
SE	SYTL2	-	chr11:85717482-85717530	NA	0.76	0	SE	AHSA2	+	chr2:61183546-61183662	NA	-0.209	1.32E-10	HACL1
SE	SYTL2	-	chr11:85717482-85717726	NA	0.857	0	SE	ZNF667-AS1	+	chr19:56491576-56491766	NA	-0.819	1.32E-10	HADH
SE	PNISR	-	chr6:99403828-99403882	NA	0.633	0	SE	MAX	-	chr14:65077562-65077643	NA	-0.553	1.66E-10	HAGH
SE	SAP30BP	+	chr17:75671815-75671863	NA	0.183	0	SE	RABEPK	+	chr9:125213369-125213522	NA	-0.256	2.92E-10	HARS2
SE	TMEM18	-	chr2:675757-676238	NA	0.545	0	SE	RNF14	+	chr5:141970703-141970877	NA	-0.175	3.01E-10	HAUS2
SE	TMEM18	-	chr2:675757-676238	NA	0.523	0	SE	IQCB1	-	chr3:121828469-121828632	NA	-0.169	3.04E-10	HAUS4
SE	PFDN5	+	chr12:53295838-53295941	NA	0.139	0	SE	DHX9	+	chr1:182855576-182855713	NA	-0.121	3.15E-10	HAUS7
SE	PFDN5	+	chr12:53296243-53296275	NA	0.31	0	SE	ADGRL2	+	chr1:81986900-81987029	NA	-0.227	3.49E-10	HAUS8
SE	CENPU	-	chr4:184700819-184700881	NA	0.484	0	SE	DLGAP5	-	chr14:55148593-55148684	NA	-0.132	3.50E-10	HCFC1R1
SE	ARHGAP11B	+	chr15:30644638-30644702	NA	0.474	0	SE	MIS12	+	chr17:5488195-5488589	NA	-0.277	3.75E-10	HCG18
SE	FERMT2	-	chr14:52861013-52861034	NA	0.155	0	SE	CDC25C	-	chr5:138329552-138329647	NA	-0.277	4.21E-10	HCN3
SE	VLDLR	+	chr9:2651414-2651498	NA	0.626	0	SE	HSCB	+	chr22:28743881-28743978	NA	-0.496	5.71E-10	HDAC11
SE	BTN3A3	+	chr6:26443381-26443442	NA	0.872	0	SE	TBC1D5	-	chr3:17428449-17428519	NA	-0.186	6.16E-10	HDAC6
SE	ANKRD12	+	chr18:9204475-9204544	NA	0.597	0	SE	RAB18	+	chr10:27531528-27531615	NA	-0.763	6.19E-10	HDAC8
SE	AP1G1	-	chr16:71767876-71767885	NA	0.557	0	SE	BRD7	-	chr16:50326283-50326391	NA	-0.114	6.79E-10	HDAC9
SE	H2AFY	-	chr5:135350822-135350913	NA	0.205	0	SE	DCLK2	+	chr4:150253473-150253556	NA	-0.488	7.92E-10	HDHD2
SE	LRRFIP1	+	chr2:237751199-237751271	NA	0.265	0	SE	TPM1	+	chr15:63061197-63061273	NA	-0.419	8.14E-10	HDLBP
SE	ARL4A	+	chr7:12687634-12687728	NA	0.602	0	SE	ZNF189	+	chr9:101399394-101399530	NA	-0.554	9.33E-10	HEATR3
SE	EXOC7	-	chr17:76091142-76091235	NA	0.387	0	SE	C2orf74	+	chr2:61157861-61158012	NA	-0.743	1.09E-09	HEATR6
SE	RAPGEF6	-	chr5:131450022-131450046	NA	0.797	0	SE	PPA2	-	chr4:105456695-105456745	NA	-0.34	1.28E-09	HECTD4
SE	ENSA	-	chr1:150625641-150625802	NA	0.185	0	SE	SERHL2	+	chr22:42573633-42573745	NA	-0.73	1.31E-09	HERC2P9
SE	ENSA	-	chr1:150625641-150625808	NA	0.156	0	SE	ANKRD11	-	chr16:89313316-89313589	NA	-0.138	1.55E-09	HGSNAT
SE	MAP4K4	+	chr2:101868028-101868037	NA	0.441	0	SE	U2AF1L4	-	chr19:35744490-35744540	NA	-0.55	1.56E-09	HHLA3
SE	RBM5	+	chr3:50104247-50104308	NA	0.637	0	SE	RPE	+	chr2:210016203-210016257	NA	-0.235	1.56E-09	HIVEP2
SE	RBM5	+	chr3:50110378-50110463	NA	0.247	0	SE	OSBPL6	+	chr2:178324176-178324269	NA	-0.323	1.60E-09	HJURP
SE	SEC22C	-	chr3:42563764-42563980	NA	0.211	0	SE	RPL17	-	chr18:49491735-49491833	NA	-0.518	1.66E-09	HKR1
SE	DNAJC8	-	chr1:28228921-28229023	NA	0.104	0	SE	ZC3H11A	+	chr1:203796292-203796601	NA	-0.272	1.68E-09	HLA-A
SE	CLTC	+	chr17:59687000-59687021	NA	0.189	0	SE	TCAF1	-	chr7:143881922-143882099	NA	-0.792	1.72E-09	HLA-C
SE	PPFIBP1	+	chr12:27658815-27658848	NA	0.773	0	SE	LPP	+	chr3:188341662-188341719	NA	-0.778	1.83E-09	HLA-F
SE	YY1AP1	-	chr1:155688070-155688201	NA	0.617	0	SE	CAST	+	chr5:96740744-96740783	NA	-0.662	2.11E-09	HLA-L
SE	NSFL1C	-	chr20:1455713-1455719	NA	0.582	0	SE	FAM111B	+	chr11:59109539-59109706	NA	-0.236	2.18E-09	HM13
SE	TSSC4	+	chr11:2401838-2402147	NA	0.535	0	SE	NUMA1	-	chr11:72012400-72012442	NA	-0.417	2.52E-09	HMBS
SE	SEPT7	+	chr7:35831491-35832034	NA	0.326	0	SE	CENPO	+	chr2:24793851-24793965	NA	-0.316	2.58E-09	HMG20B
SE	CUL5	+	chr11:108104189-108104902	NA	0.466	0	SE	PARPBP	+	chr12:102151569-102151800	NA	-0.18	2.79E-09	HMGCR
SE	FGFR1OP2	+	chr12:26960514-26960628	NA	0.58	0	SE	STX16	+	chr20:58669290-58669453	NA	-0.389	3.14E-09	HMGCS1
SE	ZNF277	+	chr7:112286872-112287074	NA	0.364	0	SE	RPS3A	+	chr4:151100484-151100588	NA	-0.359	3.45E-09	HMGN1
SE	ZNF277	+	chr7:112286876-112287074	NA	0.497	0	SE	KMT2E	+	chr7:105110476-105110602	NA	-0.199	3.54E-09	HMGN2
SE	MYO5A	-	chr15:52337809-52337884	NA	0.362	0	SE	ARHGEF9	-	chrX:63656509-63658064	NA	-0.538	3.67E-09	HMGN3
SE	POLG	-	chr15:89317938-89318037	NA	0.2	0	SE	TMEM161B-AS1	+	chr5:88287435-88287622	NA	-0.436	4.27E-09	HMGN5
SE	FAM221A	+	chr7:23692182-23694913	NA	0.453	0	SE	C2orf76	-	chr2:119321153-119321204	NA	-0.196	4.71E-09	HMGXB4
SE	PLEKHA1	+	chr10:122428275-122428420	NA	0.806	0	SE	EME1	+	chr17:50378594-50378681	NA	-0.194	5.20E-09	HMMR
SE	PLEKHA1	+	chr10:122428275-122428316	NA	0.778	0	SE	FNTB	+	chr14:65032358-65032521	NA	-0.679	5.36E-09	HNRNPA1
SE	NIN	-	chr14:50756491-50758630	NA	0.581	0	SE	EME1	+	chr17:50378599-50378681	NA	-0.275	5.50E-09	HNRNPA2B1
SE	EMC10	+	chr19:50481860-50481947	NA	0.162	0	SE	LNPK	-	chr2:175994133-175994280	NA	-0.134	5.77E-09	HNRNPAB
SE	SEPT9	+	chr17:77482143-77482335	NA	1	0	SE	PIGP	-	chr21:37072144-37072310	NA	-0.267	6.49E-09	HNRNPD
SE	PSMG4	+	chr6:3264208-3264325	NA	0.225	0	SE	DNM2	+	chr19:10808568-10808580	NA	-0.173	6.70E-09	HNRNPDL
SE	MARK3	+	chr14:103498501-103498528	NA	0.574	0	SE	MRPL33	+	chr2:27774423-27774530	NA	-0.221	6.94E-09	HNRNPH1
SE	MARK3	+	chr14:103500155-103500200	NA	0.369	0	SE	SNX19	-	chr11:130880621-130880806	NA	-0.191	7.19E-09	HNRNPH3
SE	RPS24	+	chr10:78040203-78040225	NA	0.876	0	SE	FAM192A	-	chr16:57174619-57174688	NA	-0.132	7.91E-09	HNRNPL
SE	CSNK1D	-	chr17:82249697-82250179	NA	0.196	0	SE	TMEM237	-	chr2:201634803-201634893	NA	-0.126	8.05E-09	HNRNPM
SE	MTX1	+	chr1:155212126-155212219	NA	0.196	0	SE	PUM2	-	chr2:20331716-20331937	NA	-0.248	8.33E-09	HNRNPR
SE	GULP1	+	chr2:188584264-188584403	NA	0.787	0	SE	NTAN1	-	chr16:15047854-15047920	NA	-0.181	9.24E-09	HNRNPUL1
SE	BNIP2	-	chr15:59668102-59668138	NA	0.435	0	SE	TPGS2	-	chr18:36805373-36805397	NA	-0.211	9.62E-09	HOOK2
SE	SON	+	chr21:33572550-33572630	NA	0.263	0	SE	CRIM1	+	chr2:36537351-36537546	NA	-0.151	1.02E-08	HP1BP3
SE	MYL6	+	chr12:56160625-56160670	NA	0.427	0	SE	MACF1	+	chr1:39427960-39428287	NA	-0.149	1.08E-08	HPS1
SE	WDFY3	-	chr4:84726860-84726911	NA	0.733	0	SE	USP32P3	+	chr17:20420378-20420605	NA	-0.582	1.10E-08	HPS4
SE	SNX14	-	chr6:85538837-85538864	NA	0.554	0	SE	NEO1	+	chr15:73293389-73293548	NA	-0.392	1.17E-08	HPS5
SE	UBE2E1	+	chr3:23811459-23811510	NA	0.124	0	SE	VPS41	-	chr7:38796414-38796526	NA	-0.11	1.23E-08	HSCB
SE	PKIG	+	chr20:44582584-44582731	NA	0.416	0	SE	CARS2	-	chr13:110648317-110649297	NA	-0.116	1.25E-08	HSD11B1L
SE	ORMDL1	-	chr2:189783013-189783123	NA	0.422	0	SE	PLA2G12A	-	chr4:109718682-109718759	NA	-0.304	1.31E-08	HSF4
SE	ITGAE	-	chr17:3723287-3723383	NA	0.116	0	SE	TAF2	-	chr8:119744880-119745036	NA	-0.283	1.62E-08	HSPA14
SE	PET100	+	chr19:7631231-7631321	NA	0.132	0	SE	FRG1	+	chr4:189955036-189955151	NA	-0.158	1.68E-08	HTRA2
SE	HNRNPA2B1	-	chr7:26190992-26191128	NA	0.402	0	SE	NFE2L1	+	chr17:48057031-48057121	NA	-0.245	1.71E-08	HUS1
SE	SPTAN1	+	chr9:128627924-128627942	NA	0.186	0	SE	CBY1	+	chr22:38656955-38657143	NA	-0.14	1.72E-08	HUWE1
SE	SPTAN1	+	chr9:128609650-128609665	NA	0.759	0	SE	MLLT10	+	chr10:21617111-21617207	NA	-0.197	1.81E-08	HYI
SE	CTNND1	+	chr11:57789036-57789155	NA	0.578	0	SE	CASC4	+	chr15:44402886-44403054	NA	-0.205	1.92E-08	HYKK
SE	CTNND1	+	chr11:57789036-57789155	NA	0.57	0	SE	ERBIN	+	chr5:66068876-66069020	NA	-0.112	1.96E-08	IAH1
SE	SNHG6	-	chr8:66922613-66922725	NA	0.151	0	SE	RPAIN	+	chr17:5428070-5428211	NA	-0.141	2.10E-08	ICA1
SE	PICALM	-	chr11:85978069-85978093	NA	0.361	0	SE	KIF21A	-	chr12:39318072-39318201	NA	-0.303	2.13E-08	ICA1L
SE	MYO18A	-	chr17:29085603-29085648	NA	0.652	0	SE	SEC31A	-	chr4:82861630-82861708	NA	-0.183	2.27E-08	IDH3A
SE	CCSER2	+	chr10:84499874-84499959	NA	0.444	0	SE	SLC4A7	-	chr3:27409355-27409530	NA	-0.163	2.31E-08	IFFO2
SE	PHF14	+	chr7:11061790-11061841	NA	0.17	0	SE	ACTN1	-	chr14:68878988-68879069	NA	-0.131	2.38E-08	IFI27L1
SE	ARIH2	+	chr3:48974957-48974979	NA	1	0	SE	B4GALT3	-	chr1:161176911-161177106	NA	-0.112	2.40E-08	IFNGR2
SE	MTA1	+	chr14:105445666-105445852	NA	0.292	0	SE	MYEF2	-	chr15:48151099-48151171	NA	-0.243	2.46E-08	IFRD1
SE	SMAP1	+	chr6:70791688-70791769	NA	0.294	0	SE	ANKRD36	+	chr2:97189086-97189115	NA	-0.886	2.93E-08	IFT122
SE	SZRD1	+	chr1:16391374-16391424	NA	0.283	0	SE	EMC3-AS1	+	chr3:9988371-9988477	NA	-0.782	3.23E-08	IFT43
SE	AP2M1	+	chr3:184180644-184180650	NA	0.458	0	SE	DRAM2	-	chr1:111139500-111139666	NA	-0.159	3.27E-08	IFT80
SE	NUDT2	+	chr9:34336228-34336341	NA	0.193	0	SE	FANCI	+	chr15:89245755-89245839	NA	-0.504	3.29E-08	IFT88
SE	DBR1	-	chr3:138163777-138163858	NA	0.426	0	SE	CEP78	+	chr9:78265858-78265906	NA	-0.275	3.30E-08	IGF2BP2
SE	FAM173B	-	chr5:10235210-10235261	NA	0.584	0	SE	SNHG5	-	chr6:85677794-85677868	NA	-0.124	3.33E-08	IGF2BP3
SE	ITGB3BP	-	chr1:63446805-63446856	NA	0.274	0	SE	CDCA3	-	chr12:6849322-6849413	NA	-0.116	3.55E-08	IGFLR1
SE	ITGB3BP	-	chr1:63447564-63447614	NA	0.545	0	SE	PEX2	-	chr8:76988306-76988338	NA	-0.111	3.97E-08	IGSF8
SE	GOLGA4	+	chr3:37266841-37266940	NA	0.487	0	SE	LRP8	-	chr1:53257239-53257464	NA	-0.134	3.98E-08	IKBKB
SE	ZFAND5	-	chr9:72363469-72363606	NA	0.426	0	SE	CAPRIN2	-	chr12:30719078-30719225	NA	-0.298	4.20E-08	IKZF4
SE	QKI	+	chr6:163455278-163455421	NA	0.424	0	SE	PRC1	-	chr15:90969078-90969120	NA	-0.291	4.34E-08	IL11RA
SE	OFD1	+	chrX:13763744-13763855	NA	0.423	0	SE	TWF1	-	chr12:43805523-43805637	NA	-0.789	4.38E-08	IL17RC
SE	AK2	-	chr1:33031543-33031661	NA	0.171	0	SE	STX16	+	chr20:58669301-58669453	NA	-0.4	4.53E-08	ILF3
SE	FXR1	+	chr3:180971074-180971155	NA	0.216	0	SE	DMTF1	+	chr7:87194683-87194828	NA	-0.205	4.66E-08	IMMP1L
SE	FXR1	+	chr3:180971074-180971155	NA	0.132	0	SE	NUP50	+	chr22:45170987-45171177	NA	-0.207	4.66E-08	IMP4
SE	PUDP	-	chrX:7105748-7105838	NA	1	0	SE	RNF146	+	chr6:127282286-127282395	NA	-0.658	4.86E-08	IMPA2
SE	PUDP	-	chrX:7105619-7105838	NA	1	0	SE	APEH	+	chr3:49674488-49674621	NA	-0.425	5.13E-08	INCENP
SE	PTK2	-	chr8:140803542-140803650	NA	0.167	0	SE	ETFA	-	chr15:76295590-76295737	NA	-0.141	5.37E-08	INF2
SE	PRMT2	+	chr21:46636438-46636547	NA	0.729	0	SE	APOPT1	+	chr14:103574106-103574170	NA	-0.175	5.73E-08	ING4
SE	JKAMP	+	chr14:59486712-59486804	NA	0.233	2.89E-14	SE	UBTF	-	chr17:44208618-44209001	NA	-0.104	6.26E-08	INIP
SE	CLIP1	-	chr12:122351110-122351143	NA	0.924	2.89E-14	SE	AMACR	-	chr5:33994077-33994120	NA	-0.197	6.29E-08	INO80C
SE	AK2	-	chr1:33012073-33012122	NA	0.113	2.89E-14	SE	NARFL	-	chr16:740021-740103	NA	-0.156	6.49E-08	INPP4A
SE	CCAR2	+	chr8:22613016-22613136	NA	0.147	8.25E-14	SE	RBM23	-	chr14:22905605-22905659	NA	-0.136	6.52E-08	INPP5B
SE	PRKDC	-	chr8:47782161-47782254	NA	0.118	1.08E-13	SE	CHD3	+	chr17:7907600-7907702	NA	-0.246	6.56E-08	INTS11
SE	CHEK2	-	chr22:28696900-28696987	NA	0.25	1.08E-13	SE	SNHG5	-	chr6:85677794-85677899	NA	-0.128	7.18E-08	INTS3

SE	ACTR3B	+	chr7:152811696-152811733	NA	0.328	1.08E-13	SE	MAP4K4	+	chr2:101871493-101871685	NA	-0.161	7.79E-08	INTS4
SE	TIA1	-	chr2:70229058-70229091	NA	0.349	1.08E-13	SE	CHURC1-FNTB	+	chr14:64926006-64926080	NA	-0.159	8.36E-08	INTS6
SE	ANKAR	+	chr2:189693073-189693177	NA	0.91	1.34E-13	SE	SBF2	-	chr11:9992414-9992543	NA	-0.223	9.19E-08	INTU
SE	BCHE	-	chr3:165829516-165831041	NA	0.372	1.84E-13	SE	CHURC1	+	chr14:64926009-64926080	NA	-0.109	9.60E-08	INVS
SE	DHPS	-	chr19:12677107-12677211	NA	0.104	2.35E-13	SE	IMMP1L	-	chr11:31473723-31473786	NA	-0.579	1.01E-07	IP6K2
SE	MINDY3	-	chr10:15838227-15838279	NA	0.52	2.85E-13	SE	CENPI	+	chrX:101099808-101100065	NA	-0.245	1.04E-07	IQCB1
SE	SORBS1	-	chr10:95434621-95434717	NA	0.934	3.34E-13	SE	YWHAZ	-	chr8:100951928-100952125	NA	-0.141	1.20E-07	IQCK
SE	PIP5K1C	-	chr19:3633436-3633520	NA	0.96	3.34E-13	SE	PHTF2	+	chr7:77937709-77937838	NA	-0.121	1.21E-07	IQGAP3
SE	MRPS18C	+	chr4:83458345-83458429	NA	0.342	3.58E-13	SE	DENND4A	-	chr15:65700543-65700672	NA	-0.35	1.22E-07	IRAK4
SE	LCORL	-	chr4:17873387-17878213	NA	0.225	4.00E-13	SE	HCG18	-	chr6:30314268-30314482	NA	-0.478	1.30E-07	IRF3
SE	PPP5C	+	chr19:46383410-46383476	NA	0.117	4.00E-13	SE	CCNC	-	chr6:99544205-99544273	NA	-0.12	1.47E-07	ISCU
SE	ARL14EP	+	chr11:30330885-30331374	NA	0.239	4.00E-13	SE	KYAT1	-	chr9:128847432-128847643	NA	-0.293	1.47E-07	ISG20
SE	KIF23	+	chr15:69425281-69425323	NA	0.356	4.47E-13	SE	RBFOX2	-	chr22:35836319-35836439	NA	-0.189	1.49E-07	IST1
SE	ACAP2	-	chr3:195312946-195312967	NA	0.323	4.91E-13	SE	ALDH3A2	+	chr17:19673150-19673275	NA	-0.125	1.60E-07	ISYNA1
SE	HMGCS1	-	chr5:43307765-43307824	NA	0.166	5.14E-13	SE	SLC35F5	-	chr2:113717752-113717850	NA	-0.167	1.64E-07	ITGA7
SE	TEX22	+	chr14:105449358-105449409	NA	0.444	5.32E-13	SE	RSU1	-	chr10:16816972-16817084	NA	-0.133	1.64E-07	ITGAE
SE	VEGFA	+	chr6:43781955-43782087	NA	0.199	6.27E-13	SE	RPAIN	+	chr17:5428070-5428211	NA	-0.25	1.74E-07	ITGB1BP1
SE	COA1	-	chr7:43656032-43656153	NA	0.166	6.41E-13	SE	CKLF	+	chr16:66558189-66558348	NA	-0.27	1.89E-07	ITGB3BP
SE	TPM1	+	chr15:63061712-63061788	NA	0.268	6.41E-13	SE	DNAJC13	+	chr3:132484362-132484493	NA	-0.177	1.90E-07	ITPR1
SE	HMGN1	-	chr21:39345829-39345958	NA	0.412	7.73E-13	SE	TMEM161B-AS1	+	chr5:88283009-88283086	NA	-0.54	1.95E-07	ITPRIPL1
SE	KLHDC4	-	chr16:87730551-87730644	NA	0.27	8.40E-13	SE	PARPBP	+	chr12:102175482-102175666	NA	-0.397	1.97E-07	IVD
SE	CHEK1	+	chr11:125637448-125637544	NA	0.198	8.61E-13	SE	ARMCX5-GPRASP2	+	chrX:102601879-102601977	NA	-0.266	1.98E-07	IZUMO4
SE	PUDP	-	chrX:7105619-7105838	NA	1	9.24E-13	SE	SPTAN1	+	chr9:128592982-128593042	NA	-0.286	2.07E-07	JAM2
SE	U2AF1L4	-	chr19:35744625-35744692	NA	0.725	9.67E-13	SE	SUN1	+	chr7:849922-850033	NA	-0.238	2.07E-07	JKAMP
SE	JKAMP	+	chr14:59486730-59486804	NA	0.305	1.06E-12	SE	RGMB-AS1	-	chr5:98771655-98771766	NA	-0.667	2.10E-07	JMJD1C
SE	LIMCH1	+	chr4:41638931-41638967	NA	0.554	1.32E-12	SE	UBA2	+	chr19:34430092-34430186	NA	-0.122	2.15E-07	JMJD4
SE	AP4M1	+	chr7:100102245-100102393	NA	0.292	1.62E-12	SE	HDLBP	-	chr2:241269360-241269561	NA	-0.129	2.19E-07	JMJD8
SE	GSN	+	chr9:121300055-121300126	NA	0.366	1.92E-12	SE	FAM76B	-	chr11:95779077-95779135	NA	-0.347	2.25E-07	JOSD2
SE	JKAMP	+	chr14:59486680-59486804	NA	0.307	2.33E-12	SE	CSPP1	+	chr8:67172768-67172973	NA	-0.222	2.44E-07	KANK1
SE	NUP58	+	chr13:25309246-25309282	NA	0.193	2.36E-12	SE	ANAPC16	+	chr10:72220053-72220379	NA	-0.373	2.52E-07	KANK2
SE	USP3-AS1	-	chr15:63593748-63593877	NA	0.925	4.00E-12	SE	FKBP10	+	chr17:41819840-41820023	NA	-0.12	2.58E-07	KANSL2
SE	AC024560.3	-	chr3:197624471-197624720	NA	0.423	4.05E-12	SE	LAS1L	-	chrX:65528259-65528369	NA	-0.125	2.61E-07	KAT6A
SE	TMEM126B	+	chr11:85631144-85631316	NA	0.31	4.34E-12	SE	NBR2	+	chr17:43132955-43133137	NA	-0.583	2.65E-07	KAT6B
SE	CLASP2	-	chr3:33596710-33596734	NA	0.815	4.89E-12	SE	SNHG14	+	chr15:25053265-25053363	NA	-0.65	2.78E-07	KATNAL1
SE	COA1	-	chr7:43665657-43665722	NA	0.299	5.25E-12	SE	WNK1	+	chr12:879572-880031	NA	-0.109	2.78E-07	KATNAL2
SE	EPB41L2	-	chr6:130870327-130870381	NA	0.576	5.39E-12	SE	KLHL42	+	chr12:27797246-27797349	NA	-0.27	2.88E-07	KB-1572G7.2
SE	BTN3A3	+	chr6:26443366-26443442	NA	0.881	5.48E-12	SE	NQO2	+	chr6:3002049-3002286	NA	-0.131	2.94E-07	KBTBD3
SE	SNX25	+	chr4:185351444-185351609	NA	0.389	5.65E-12	SE	ROBO1	-	chr3:78693347-78693356	NA	-0.306	2.98E-07	KCNAB3
SE	SEC22C	-	chr3:42563764-42563880	NA	0.108	5.82E-12	SE	RABEPK	+	chr9:125220538-125220700	NA	-0.194	3.05E-07	KCTD17
SE	SEC31A	-	chr4:82862533-82862572	NA	0.298	6.25E-12	SE	HNRNPDL	-	chr4:82425562-82425667	NA	-0.185	3.12E-07	KCTD9
SE	PPIP5K2	+	chr5:103176865-103176910	NA	0.24	7.34E-12	SE	C1orf50	+	chr1:42767508-42767624	NA	-0.527	3.15E-07	KDM4C
SE	NRM	-	chr6:30689275-30689452	NA	0.259	8.50E-12	SE	LINC02210	+	chr17:45640388-45640455	NA	-0.754	3.26E-07	KHK
SE	SEPT10	-	chr2:109545384-109545542	NA	0.137	9.50E-12	SE	TAF1	+	chrX:71459149-71459251	NA	-0.38	3.53E-07	KIAA0141
SE	BPTF	+	chr17:67875555-67875744	NA	0.314	9.73E-12	SE	FBXO22	+	chr15:75904490-75904629	NA	-0.222	3.54E-07	KIAA0430
SE	EEF1D	-	chr8:143592646-143592893	NA	0.522	1.10E-11	SE	DUS4L	+	chr7:107573703-107573821	NA	-0.237	3.75E-07	KIAA0586
SE	EEF1D	-	chr8:143592646-143592942	NA	0.497	1.12E-11	SE	CARD8	-	chr19:48249522-48249633	NA	-0.243	4.17E-07	KIAA0753
SE	CTNND1	+	chr11:57789036-57789155	NA	0.495	1.21E-11	SE	RP11-849H4.2	-	chr11:71925932-71926104	NA	-0.787	4.30E-07	KIAA1109
SE	OXR1	+	chr8:106737519-106737600	NA	0.389	1.25E-11	SE	PAQR6	-	chr1:156247509-156247632	NA	-0.619	4.35E-07	KIAA1191
SE	HMMR	+	chr5:163467700-163467748	NA	0.338	1.27E-11	SE	CPNE3	+	chr8:86524876-86524997	NA	-0.406	4.50E-07	KIAA1211
SE	COMMD4	+	chr15:75338354-75338420	NA	0.133	1.29E-11	SE	SEPT6	-	chrX:119625334-119625379	NA	-0.414	4.59E-07	KIAA1217
SE	ILF3	+	chr19:10684415-10684476	NA	0.323	1.34E-11	SE	RP11-705C15.2	+	chr12:9656567-9656697	NA	-0.542	4.86E-07	KIAA1328
SE	CASK	-	chrX:41555599-41555635	NA	0.278	1.34E-11	SE	DONSON	-	chr21:33583487-33583666	NA	-0.127	4.88E-07	KIAA1429
SE	TMEM259	-	chr19:1011095-1011195	NA	0.136	1.43E-11	SE	PHYKPL	-	chr5:178223319-178223451	NA	-0.526	4.88E-07	KIAA1468
SE	CTNND1	+	chr11:57791384-57791673	NA	0.43	1.62E-11	SE	COX20	+	chr1:244841943-244842058	NA	-0.408	5.10E-07	KIAA1551
SE	ANKRD36	+	chr2:97209680-97209709	NA	0.869	1.65E-11	SE	MACF1	+	chr1:39465094-39465112	NA	-0.145	5.30E-07	KIAA2026
SE	AURKB	-	chr17:8210176-8210249	NA	0.232	1.97E-11	SE	MACF1	+	chr1:39436481-39436490	NA	-0.428	5.56E-07	KIF13A
SE	TTC3	+	chr21:37150819-37150884	NA	0.141	2.10E-11	SE	WDFY3	-	chr4:84808333-84808417	NA	-0.281	6.05E-07	KIF16B
SE	ARFIP1	+	chr4:152870752-152870848	NA	0.234	2.20E-11	SE	NAP1L4	-	chr11:2989128-2989271	NA	-0.394	6.18E-07	KIF1B
SE	PCBP1-AS1	-	chr2:70053730-70053815	NA	0.84	2.34E-11	SE	CARD8	-	chr19:48242539-48242644	NA	-0.334	6.60E-07	KIF21A
SE	U2AF1L4	-	chr19:35744625-35744738	NA	0.662	2.54E-11	SE	DLEU2	-	chr13:50049583-50049662	NA	-0.345	6.76E-07	KIF21B
SE	MYO6	+	chr6:75911671-75911698	NA	0.315	3.49E-11	SE	RBCK1	+	chr20:417219-417342	NA	-0.183	7.29E-07	KIF23
SE	TNIK	-	chr3:171139469-171139556	NA	0.604	3.94E-11	SE	EED	+	chr11:86266082-86266216	NA	-0.22	7.62E-07	KIF2A
SE	LTBP4	+	chr19:40622949-40623021	NA	0.229	4.13E-11	SE	PHF3	+	chr6:63678827-63679020	NA	-0.103	7.80E-07	KIF3A
SE	SYNJ1	-	chr21:32634860-32634884	NA	0.634	4.13E-11	SE	HKR1	+	chr19:37344609-37344774	NA	-0.782	7.85E-07	KIZ
SE	PAAF1	+	chr11:73887030-73887099	NA	0.188	4.40E-11	SE	EPB41L2	-	chr6:130880143-130880206	NA	-0.166	7.87E-07	KLC1
SE	TIA1	-	chr2:70228343-70228462	NA	0.465	4.53E-11	SE	BDNF-AS	+	chr11:27658240-27658462	NA	-0.274	1.00E-06	KLHDC1
SE	LRCH3	+	chr3:197858833-197858905	NA	0.521	5.20E-11	SE	HKR1	+	chr19:37346887-37347052	NA	-0.712	1.03E-06	KLHDC4
SE	RPS24	+	chr10:78040200-78040225	NA	0.57	5.37E-11	SE	RP11-421L21.3	+	chr1:101075394-101075716	NA	-0.362	1.04E-06	KLHL24
SE	PPIL3	-	chr2:200882341-200882435	NA	0.454	5.63E-11	SE	DNAJC19	-	chr3:180986942-180987022	NA	-0.262	1.04E-06	KLHL42
SE	GNAS	+	chr20:58898940-58898985	NA	0.217	5.70E-11	SE	RWDD3	+	chr1:95239776-95239909	NA	-0.532	1.07E-06	KLHL7
SE	MCTP1	-	chr5:94917895-94917973	NA	0.457	5.72E-11	SE	FYN	-	chr6:111846588-111846629	NA	-0.25	1.07E-06	KMT2A
SE	GSN	+	chr9:121300055-121300126	NA	0.788	5.86E-11	SE	DLEU2	-	chr13:50044650-50044768	NA	-0.404	1.13E-06	KMT2C
SE	FANCA	-	chr16:89762637-89762793	NA	0.287	5.88E-11	SE	ALDOA	+	chr16:30065875-30065927	NA	-0.23	1.22E-06	KMT2D
SE	HMGN1	-	chr21:39347246-39347292	NA	0.375	7.19E-11	SE	ZC3H11A	+	chr1:203796450-203796607	NA	-0.325	1.25E-06	KMT2E
SE	CTNND1	+	chr11:57791384-57791673	NA	0.299	7.26E-11	SE	ZNF302	+	chr19:34679827-34679959	NA	-0.131	1.32E-06	KMT5B
SE	SLMAP	+	chr3:57925844-57925934	NA	0.285	7.79E-11	SE	ALKBH3	+	chr11:43901515-43901725	NA	-0.16	1.33E-06	KMT5C
SE	MPHOSPH9	-	chr12:123226287-123226351	NA	0.322	8.53E-11	SE	EMSY	+	chr11:76535894-76536059	NA	-0.231	1.35E-06	KNSTRN
SE	PILRB	+	chr7:100353894-100354012	NA	0.41	9.00E-11	SE	TMTC4	-	chr13:100670359-100670569	NA	-0.222	1.46E-06	KNTC1
SE	MICAL3	-	chr22:17803810-17803861	NA	0.695	9.30E-11	SE	SGSM2	+	chr17:2367270-2367405	NA	-0.365	1.48E-06	KPNA5
SE	LIAS	+	chr4:39468118-39468427	NA	0.184	9.48E-11	SE	TTC3	+	chr21:37094004-37094090	NA	-0.175	1.50E-06	KRIT1
SE	RP4-798P15.3	-	chr1:177984205-177984418	NA	0.86	9.70E-11	SE	BCHE	-	chr3:165777716-165777795	NA	-0.11	1.52E-06	KRTCAP2
SE	GNAS	+	chr20:58898940-58898985	NA	0.209	1.00E-10	SE	RPS3A	+	chr4:151100974-151101085	NA	-0.371	1.58E-06	KTN1
SE	C11orf49	+	chr11:47157022-47157218	NA	0.276	1.06E-10	SE	TMTC4	-	chr13:100670282-100670569	NA	-0.327	1.64E-06	KYAT1
SE	SACS	-	chr13:23341444-23341690	NA	0.213	1.17E-10	SE	SLC50A1	+	chr1:155136827-155136951	NA	-0.332	1.70E-06	L3HYPDH
SE	SENP7	-	chr3:101417592-101417790	NA	0.314	1.18E-10	SE	LMAN2L	-	chr2:96737136-96737217	NA	-0.169	1.72E-06	L3MBTL3
SE	SLC25A36	+	chr3:140966933-140967036	NA	0.326	1.18E-10	SE	SEMA4F	+	chr2:74657852-74657951	NA	-0.406	1.72E-06	LAMP2
SE	NSUN2	-	chr5:6631872-6631977	NA	0.189	1.23E-10	SE	NSUN4	+	chr1:46346920-46347075	NA	-0.182	1.73E-06	LARP1
SE	PMS2CL	+	chr7:6733398-6733527	NA	0.48	1.31E-10	SE	AGPAT4	-	chr6:161165576-161165713	NA	-0.184	1.73E-06	LAS1L
SE	WARS	-	chr14:100375282-100375403	NA	0.232	1.37E-10	SE	MRPS18C	+	chr4:83458345-83458429	NA	-0.13	1.79E-06	LCA5L
SE	HARS2	+	chr5:140696102-140696201	NA	0.175	1.39E-10	SE	CCNC	-	chr6:99544178-99544273	NA	-0.138	1.82E-06	LCAT
SE	UBA1	+	chrX:47198166-47198355	NA	0.128	1.49E-10	SE	HNRNPD	-	chr4:82356536-82356683	NA	-0.181	1.83E-06	LCORL
SE	RBM6	+	chr3:49972058-49972148	NA	0.301	1.54E-10	SE	COA1	-	chr7:43650611-43650712	NA	-0.425	1.83E-06	LDLR
SE	GAPVD1	+	chr9:125342218-125342299	NA	0.172	1.64E-10	SE	SPG7	+	chr16:89545871-89546030	NA	-0.139	1.91E-06	LDLRAP1
SE	MIS12	+	chr17:5487556-5487694	NA	0.225	1.66E-10	SE	CDC25C	-	chr5:138325814-138325904	NA	-0.312	1.94E-06	LETM2
SE	VTI1B	-	chr14:67663028-67663190	NA	0.183	1.67E-10	SE	ZEB1	+	chr10:31387123-31387266	NA	-0.24	1.97E-06	LETMD1
SE	CEP78	+	chr9:78246668-78246782	NA	0.163	1.69E-10	SE	MAZ	+	chr16:29809539-29809764	NA	-0.262	1.98E-06	LIAS
SE	UGGT2	-	chr13:95860787-95860883	NA	0.462	1.74E-10	SE	SOS1	-	chr2:38989269-38989314	NA	-0.553	2.09E-06	LIG1
SE	SDR39U1	-	chr14:24442177-24442260	NA	0.422	1.80E-10	SE	ZGRF1	-	chr4:112548252-112548380	NA	-0.346	2.11E-06	LIG4
SE	PTK2	-	chr8:140925660-140925749	NA	0.299	1.81E-10	SE	LINC00969	+	chr3:195663623-195663832	NA	-0.32	2.17E-06	LIMCH1
SE	MAPK3	-	chr16:30117136-30117285	NA	0.141	1.99E-10	SE	CCNYL1	+	chr2:207740654-207740706	NA	-0.26	2.18E-06	LIMK2
SE	FAM122B	-	chrX:134772142-134772283	NA	0.264	2.18E-10	SE	KIF21A	-	chr12:39318072-39318201	NA	-0.269	2.19E-06	LIN37
SE	PAAF1	+	chr11:73886928-73887099	NA	0.173	2.20E-10	SE	KIAA1109	+	chr4:122316347-122316548	NA	-0.112	2.22E-06	LIN9
SE	HAGH	-	chr16:1819114-1819223	NA	0.183	2.21E-10	SE	SLCO4A1-AS1	-	chr20:62664923-62665497	NA	-0.273	2.23E-06	LINC00174
SE	PUF60	-	chr8:143820665-143820716	NA	0.435	2.28E-10	SE	RP11-421L21.3	+	chr1:101080388-101080613	NA	-0.634	2.30E-06	LINC00630
SE	WARS	-	chr14:100375282-100375406	NA	0.236	2.43E-10	SE	RP11-9G1.3	-	chr4:133140585-133140659	NA	-0.468	2.41E-06	LINC00894
SE	TMEM39A	-	chr3:119435754-119435935	NA	0.321	2.44E-10	SE	PCBP1-AS1	-	chr2:70055713-70055910	NA	-0.436	2.42E-06	LINC00910
SE	GPM6A	-	chr4:175812190-175812249	NA	0.649	2.67E-10	SE	ZEB1	+	chr10:31373017-31373173	NA	-0.459	2.43E-06	LINC00963
SE	RASSF8-AS1	-	chr12:25956226-25956347	NA	0.633	2.67E-10	SE	DCUN1D4	+	chr4:51905188-51905320	NA	-0.109	2.74E-06	LINC00969
SE	PSMG1	-	chr21:39179923-39179986	NA	0.236	2.86E-10	SE	CCHCR1	-	chr6:31154495-31154799	NA	-0.274	2.81E-06	LINC01001
SE	ARHGAP11B	+	chr15:30644638-30644702	NA	0.392	2.89E-10	SE	TMEM161B-AS1	+	chr5:88285740-88285851	NA	-0.317	2.86E-06	LINC01021
SE	TLE2	-	chr19:3017839-3017859	NA	0.769	3.07E-10	SE	MCOLN3	-	chr1:85036709-85036868	NA	-0.648	3.05E-06	LINC01125
SE	HNRNPD	-	chr4:82371527-82371584	NA	0.29	3.50E-10	SE	AC007566.10	+	chr7:92462957-92463026	NA	-0.228	3.06E-06	LINC01128
SE	SLC9A5	+	chr16:67262211-67262289	NA	0.773	3.90E-10	SE	SNAPC5	-	chr15:66495329-66495419	NA	-0.142	3.19E-06	LINC01184
SE	MTCL1	+	chr18:8809447-8809561	NA	0.295	3.95E-10	SE	IMMP1L	-	chr11:31456259-31456386	NA	-0.223	3.19E-06	LINC01778
SE	CTNND1	+	chr11:57806460-57806478	NA	0.266	4.23E-10	SE	YWHAZ	-	chr8:100951928-100952308	NA	-0.173	3.26E-06	LINC02210
SE	AFDN	+	chr6:167913402-167913423	NA	0.579	4.69E-10	SE	NEDD1	+	chr12:96907603-96907624	NA	-0.596	3.39E-06	LIPT1
SE	THUMPD3-AS1	-	chr3:9395505-9395579	NA	0.324	4.80E-10	SE	NUP54	-	chr4:76134174-76134362	NA	-0.118	3.46E-06	LL22NC03-80A10.6
SE	FAM168A	-	chr11:73425021-73425048	NA	0.237	5.74E-10	SE	CCM2	+	chr7:45068442-45068579	NA	-0.28	3.67E-06	LLGL1
SE	C17orf62	-	chr17:82446622-82446692	NA	0.348	5.97E-10	SE	DHX33	-	chr17:5463528-5463689	NA	-0.177	3.95E-06	LMAN2L
SE	RWDD1	+	chr6:116572857-116572985	NA	0.105	6.80E-10	SE	ARHGEF9	-	chrX:63724531-63724711	NA	-0.104	4.07E-06	LMBR1L
SE	WASF3	+	chr13:26680034-26680201	NA	0.534	6.92E-10	SE	DLGAP1-AS1	+	chr18:3596578-3596675	NA	-0.304	4.07E-06	LMNA

SE	ARL4A	+	chr7:12687634-12687728	NA	0.499	7.00E-10	SE	ALG13	+	chrX:111689086-111689197	NA	-0.261	4.16E-06	LNPK
SE	WARS	-	chr14:100375282-100375350	NA	0.205	7.15E-10	SE	PLD3	+	chr19:40365717-40365930	NA	-0.551	4.25E-06	LPCAT4
SE	SNHG17	-	chr20:38422091-38422241	NA	0.386	7.64E-10	SE	TMEM260	+	chr14:56621530-56621702	NA	-0.384	4.25E-06	LPIN1
SE	ZNF706	-	chr8:101204649-101204693	NA	0.718	1.02E-09	SE	SLC12A2	+	chr5:128177104-128177152	NA	-0.117	4.35E-06	LPP
SE	MICAL3	-	chr22:17826453-17826516	NA	0.5	1.06E-09	SE	CLEC2B	-	chr12:9862498-9862573	NA	-0.149	4.64E-06	LRCH3
SE	MED6	-	chr14:70585755-70585783	NA	0.114	1.29E-09	SE	SIL1	-	chr5:139127943-139128052	NA	-0.447	4.68E-06	LRP2BP
SE	TOP1MT	-	chr8:143332486-143332657	NA	0.317	1.32E-09	SE	ZHX3	-	chr20:41213733-41213899	NA	-0.16	4.68E-06	LRP8
SE	SUN1	+	chr7:849519-849603	NA	0.107	1.48E-09	SE	DCAF10	+	chr9:37842088-37842286	NA	-0.185	4.77E-06	LRRC23
SE	AURKB	-	chr17:8210176-8210225	NA	0.33	1.60E-09	SE	RAC1	+	chr7:6398661-6398718	NA	-0.261	4.82E-06	LRRC27
SE	FAM24B	-	chr10:122850423-122850520	NA	0.515	1.61E-09	SE	ALKBH3	+	chr11:43892040-43892129	NA	-0.233	4.97E-06	LRRC37A4P
SE	RP11-274B21.1	+	chr7:128577963-128578033	NA	0.682	1.63E-09	SE	CENPO	+	chr2:24793851-24793965	NA	-0.334	5.09E-06	LRRC37B
SE	C19orf60	+	chr19:18589478-18589683	NA	0.647	1.65E-09	SE	CLHC1	-	chr2:55181569-55181744	NA	-0.302	5.10E-06	LRRC41
SE	HMMR	+	chr5:163467703-163467748	NA	0.199	1.66E-09	SE	MED23	-	chr6:131602217-131602381	NA	-0.459	5.22E-06	LRRC75A-AS1
SE	PRKAG1	-	chr12:49005742-49005852	NA	0.109	1.93E-09	SE	POC1B	-	chr12:89425160-89425379	NA	-0.142	5.41E-06	LRRC75B
SE	NOL8	-	chr9:92311145-92311259	NA	0.186	1.93E-09	SE	CHTF8	-	chr16:69121052-69121170	NA	-0.201	5.45E-06	LRRCC1
SE	BTF3L4	+	chr1:52083339-52083541	NA	0.252	2.12E-09	SE	IMMP1L	-	chr11:31463171-31463305	NA	-0.266	5.79E-06	LRRFIP1
SE	NGLY1	-	chr3:25732318-25732483	NA	0.146	2.13E-09	SE	TCTN2	+	chr12:123694841-123694976	NA	-0.273	5.84E-06	LRRK2
SE	SRGAP1	+	chr12:64091892-64091926	NA	0.904	2.23E-09	SE	CADM2	+	chr3:85979163-85979283	NA	-0.535	5.93E-06	LRTOMT
SE	KIF3A	-	chr5:132706450-132706459	NA	0.49	2.45E-09	SE	PPP2R3C	-	chr14:35108138-35108236	NA	-0.611	5.99E-06	LSM1
SE	NKTR	+	chr3:42627199-42630575	NA	0.222	2.54E-09	SE	UACA	-	chr15:70684264-70684446	NA	-0.129	6.02E-06	LSM14B
SE	PRDM2	+	chr1:13778417-13782831	NA	0.289	2.58E-09	SE	RPP40	-	chr6:4998715-4998841	NA	-0.204	6.11E-06	LTA4H
SE	HSPA14	+	chr10:14851218-14851323	NA	0.196	2.58E-09	SE	ZBTB1	+	chr14:64516599-64516751	NA	-0.31	6.58E-06	LTB4R2
SE	MAP4K4	+	chr2:101863820-101864051	NA	0.319	2.64E-09	SE	MBD5	+	chr2:148233244-148233395	NA	-0.204	6.62E-06	LTBP1
SE	SBF1	-	chr22:50457033-50457111	NA	0.784	2.68E-09	SE	IQCB1	-	chr3:121795456-121795566	NA	-0.18	6.62E-06	LTBP4
SE	PSMG4	+	chr6:3264173-3264325	NA	0.147	2.71E-09	SE	NIN	-	chr14:50766306-50766396	NA	-0.116	6.67E-06	LUC7L2
SE	NUDT22	+	chr11:64227567-64227666	NA	0.367	3.01E-09	SE	CCDC18-AS1	-	chr1:93318167-93318228	NA	-0.794	6.70E-06	LUC7L3
SE	MTTP	+	chr4:99574808-99574970	NA	0.745	3.17E-09	SE	TMEM45A	+	chr3:100519076-100519316	NA	-0.11	6.71E-06	LUZP1
SE	LUC7L3	+	chr17:50738136-50738199	NA	0.274	3.31E-09	SE	ZC3H14	+	chr14:88602827-88603060	NA	-0.23	6.94E-06	LYPLAL1
SE	ZCWPW2	+	chr3:28492126-28492173	NA	0.602	4.72E-09	SE	SLC35F5	-	chr2:113717752-113717848	NA	-0.29	6.95E-06	LYRM1
SE	HNRNPH1	-	chr5:179619268-179619360	NA	0.129	5.52E-09	SE	UBA3	-	chr3:69080111-69080153	NA	-0.14	7.08E-06	LZTFL1
SE	AASDH	-	chr4:56342834-56342966	NA	0.252	5.97E-09	SE	ADGRB2	-	chr1:31728024-31728081	NA	-0.325	7.12E-06	MACF1
SE	GOLT1B	+	chr12:21508382-21508561	NA	0.101	6.35E-09	SE	CEP41	-	chr7:130400038-130400254	NA	-0.189	7.13E-06	MADD
SE	SNAPIN	+	chr1:153659137-153659184	NA	0.214	7.04E-09	SE	PLA2G12A	-	chr4:109718688-109718759	NA	-0.67	7.20E-06	MAEA
SE	FUBP1	-	chr1:77947452-77947547	NA	0.814	7.18E-09	SE	NDUFB2	+	chr7:140700869-140700959	NA	-0.623	7.31E-06	MAGED2
SE	APTX	-	chr9:32988082-32988129	NA	0.332	7.79E-09	SE	TCF25	+	chr16:89908965-89909083	NA	-0.201	7.43E-06	MAGED4
SE	USP45	-	chr6:99445796-99446463	NA	0.455	7.84E-09	SE	OFD1	+	chrX:13751248-13751368	NA	-0.203	7.43E-06	MAGI1
SE	FANCI	+	chr15:89245194-89245311	NA	0.123	8.00E-09	SE	NME6	-	chr3:48296118-48296158	NA	-0.375	7.94E-06	MAMDC4
SE	SRSF7	-	chr2:38748897-38749346	NA	0.118	8.21E-09	SE	PHF8	-	chrX:53993600-53993903	NA	-0.284	8.23E-06	MAN2B2
SE	CDC42SE1	-	chr1:151056650-151056786	NA	0.108	8.21E-09	SE	PUM2	-	chr2:20278582-20278819	NA	-0.248	8.29E-06	MAN2C1
SE	TTYH3	+	chr7:2647958-2648054	NA	0.238	8.35E-09	SE	SNRPG	-	chr2:70289349-70289372	NA	-0.177	8.30E-06	MANBA
SE	C17orf62	-	chr17:82446622-82446696	NA	0.26	9.88E-09	SE	DRAM2	-	chr1:111139587-111139711	NA	-0.351	8.31E-06	MAP3K6
SE	TMEM9B	-	chr11:8962091-8962183	NA	0.247	1.02E-08	SE	FAM86DP	-	chr3:75427426-75427692	NA	-0.221	8.51E-06	MAP3K8
SE	TSSC4	+	chr11:2401975-2402147	NA	0.429	1.03E-08	SE	PARPBP	+	chr12:102178591-102178770	NA	-0.445	8.63E-06	MAP4
SE	PAAF1	+	chr11:73887039-73887099	NA	0.177	1.10E-08	SE	BNIP2	-	chr15:59665349-59665442	NA	-0.234	8.71E-06	MAP4K4
SE	DGUOK	+	chr2:73950626-73950732	NA	0.113	1.12E-08	SE	CDC25C	-	chr5:138326020-138326054	NA	-0.343	8.74E-06	MAP7D1
SE	GPBP1	+	chr5:57237112-57237172	NA	0.19	1.14E-08	SE	SEC24C	+	chr10:73746629-73746716	NA	-0.106	8.85E-06	MAP9
SE	POLR1B	+	chr2:112549266-112549399	NA	0.141	1.16E-08	SE	C2CD5	-	chr12:22519114-22519239	NA	-0.69	9.33E-06	MAPK3
SE	C19orf60	+	chr19:18589412-18589683	NA	0.546	1.17E-08	SE	C5orf22	+	chr5:31548489-31548687	NA	-0.15	9.78E-06	MAPK8IP3
SE	ZFYVE19	+	chr15:40809155-40809240	NA	0.239	1.17E-08	SE	AK2	-	chr1:33011447-33011710	NA	-0.312	9.96E-06	MAPKAPK5-AS1
SE	FAM24B	-	chr10:122850423-122850550	NA	0.361	1.17E-08	SE	FDFT1	+	chr8:11803090-11803225	NA	-0.595	1.01E-05	MAPRE3
SE	GUK1	+	chr1:228141123-228141288	NA	0.395	1.21E-08	SE	PVT1	+	chr8:127983903-127984204	NA	-0.173	1.02E-05	MAPT
SE	RP11-51F16.8	-	chr17:63726637-63726695	NA	0.422	1.24E-08	SE	HEATR6	-	chr17:60073745-60074159	NA	-0.259	1.02E-05	March2
SE	CDC16	+	chr13:114261886-114261948	NA	0.175	1.45E-08	SE	ORC4	-	chr2:147975901-147975975	NA	-0.107	1.06E-05	March6
SE	MCTP1	-	chr5:94799012-94799132	NA	0.365	1.48E-08	SE	BPHL	+	chr6:3119284-3119512	NA	-0.475	1.12E-05	March7
SE	MCTP1	-	chr5:94917895-94917973	NA	0.575	1.56E-08	SE	RPL17	-	chr18:49491745-49491833	NA	-0.198	1.13E-05	MARK2
SE	CARD19	+	chr9:93111878-93111938	NA	0.278	1.56E-08	SE	IQCB1	-	chr3:121799195-121799374	NA	-0.148	1.16E-05	MARK3
SE	EEF1D	-	chr8:143592646-143592893	NA	0.499	1.57E-08	SE	GSAP	-	chr7:77328605-77328637	NA	-0.358	1.17E-05	MAST1
SE	NAP1L1	-	chr12:76074202-76074239	NA	0.129	1.61E-08	SE	ANKHD1	+	chr5:140472238-140472295	NA	-0.238	1.18E-05	MATR3
SE	CDC14B	-	chr9:96509672-96509789	NA	0.526	1.71E-08	SE	PSMA3-AS1	-	chr14:58285590-58285756	NA	-0.308	1.18E-05	MAU2
SE	DCAF8	-	chr1:160261284-160261358	NA	0.259	1.88E-08	SE	EPB41L2	-	chr6:130878103-130878250	NA	-0.114	1.20E-05	MAX
SE	KIAA1109	+	chr4:122275971-122276022	NA	0.408	1.95E-08	SE	SLTM	-	chr15:58912562-58912610	NA	-0.667	1.22E-05	MAZ
SE	MFF	+	chr2:227342744-227342819	NA	0.112	2.01E-08	SE	MRNIP	-	chr5:179851242-179851424	NA	-0.604	1.26E-05	MBD1
SE	CTNND1	+	chr11:57791493-57791673	NA	0.434	2.12E-08	SE	NDUFB2	+	chr7:140700869-140700959	NA	-0.561	1.31E-05	MBD5
SE	DTWD1	+	chr15:49630955-49631058	NA	0.101	2.38E-08	SE	GAB1	+	chr4:143457679-143457769	NA	-0.485	1.31E-05	MBNL1
SE	SLK	+	chr10:104002171-104003527	NA	0.124	2.43E-08	SE	ENTPD6	+	chr20:25207075-25207397	NA	-0.27	1.31E-05	MBTD1
SE	CTNND1	+	chr11:57791491-57791673	NA	0.434	2.44E-08	SE	TRAPPC13	+	chr5:65655635-65655653	NA	-0.396	1.37E-05	MCAT
SE	THOC5	-	chr22:29550727-29550843	NA	0.218	2.53E-08	SE	LRCH3	+	chr3:197866111-197866219	NA	-0.12	1.39E-05	MCCC2
SE	CARM1	+	chr19:10921374-10921443	NA	0.381	2.60E-08	SE	LRRCC1	+	chr8:85123412-85123606	NA	-0.193	1.49E-05	MCFD2
SE	PRKAG1	-	chr12:49005742-49005881	NA	0.205	2.77E-08	SE	CC2D1B	-	chr1:52354364-52354454	NA	-0.441	1.52E-05	MCM3
SE	RNF146	+	chr6:127280515-127280604	NA	0.538	2.89E-08	SE	ENTPD6	+	chr20:25207078-25207397	NA	-0.274	1.54E-05	MCM7
SE	CHAF1A	+	chr19:4418019-4418076	NA	0.137	3.24E-08	SE	SLC25A30	-	chr13:45411732-45411938	NA	-0.245	1.54E-05	MCM9
SE	ZFYVE19	+	chr15:40809118-40809240	NA	0.147	3.27E-08	SE	DENND4A	-	chr15:65700543-65700675	NA	-0.267	1.60E-05	MCOLN3
SE	MRPL22	+	chr5:154950820-154950938	NA	0.222	3.60E-08	SE	KRIT1	-	chr7:92244001-92244149	NA	-0.288	1.62E-05	MCRIP2
SE	PTPRS	-	chr19:5216719-5216767	NA	0.236	3.60E-08	SE	ARHGEF12	+	chr11:120429711-120429831	NA	-0.145	1.69E-05	MCTP1
SE	ZNF584	+	chr19:58409940-58410091	NA	0.579	3.95E-08	SE	WASF1	-	chr6:110178597-110178742	NA	-0.264	1.70E-05	MDC1
SE	FAM13B	-	chr5:138018954-138019146	NA	0.522	3.97E-08	SE	ZNF761	+	chr19:53445000-53445123	NA	-0.322	1.76E-05	MDM1
SE	GABBR1	-	chr6:29609228-29609379	NA	0.14	4.05E-08	SE	COA1	-	chr7:43665657-43665722	NA	-0.29	1.78E-05	MDM4
SE	UBE2W	-	chr8:73870268-73870301	NA	0.19	4.28E-08	SE	ZNF202	-	chr11:123727475-123727595	NA	-0.121	1.81E-05	MED12L
SE	SEPT9	+	chr17:77402336-77402495	NA	0.519	4.46E-08	SE	TPT1-AS1	+	chr13:45390757-45390902	NA	-0.148	1.99E-05	MED22
SE	PPWD1	+	chr5:65571838-65572286	NA	0.179	4.50E-08	SE	TMEM63B	+	chr6:44135327-44135366	NA	-0.149	2.00E-05	MED23
SE	PAQR3	-	chr4:78935120-78935283	NA	0.324	4.59E-08	SE	CDC45	+	chr22:19482689-19482827	NA	-0.101	2.01E-05	MED24
SE	CASK	-	chrX:41557031-41557100	NA	0.608	4.65E-08	SE	NOD1	-	chr7:30437572-30437656	NA	-0.262	2.01E-05	MED6
SE	CENPV	-	chr17:16349139-16349333	NA	0.682	5.11E-08	SE	IGF2BP3	-	chr7:23418775-23418824	NA	-0.245	2.05E-05	MEF2A
SE	SCML1	+	chrX:17744070-17744219	NA	0.304	5.26E-08	SE	LSM1	-	chr8:38169801-38169917	NA	-0.102	2.05E-05	MEIG1
SE	ZNF584	+	chr19:58409964-58410091	NA	0.59	5.49E-08	SE	PLEKHM1	-	chr17:45468208-45468593	NA	-0.287	2.06E-05	MEIS3
SE	FAM126B	-	chr2:201022058-201022114	NA	0.549	5.59E-08	SE	WIPI2	+	chr7:5193117-5193171	NA	-0.549	2.10E-05	MELK
SE	KMT2D	-	chr12:49024052-49024100	NA	0.182	5.73E-08	SE	DAG1	+	chr3:49470964-49471049	NA	-0.562	2.12E-05	MELTF
SE	SUZ12P1	+	chr17:30734896-30734943	NA	0.696	5.79E-08	SE	ZNF283	+	chr19:43827775-43827940	NA	-0.29	2.15E-05	MEN1
SE	NHLRC3	+	chr13:39042104-39042305	NA	0.36	5.79E-08	SE	WDR27	-	chr6:169613558-169613656	NA	-0.437	2.16E-05	METTL14
SE	MBNL1	+	chr3:152456266-152456361	NA	0.13	5.88E-08	SE	PVT1	+	chr8:128099369-128099573	NA	-0.182	2.16E-05	METTL22
SE	NKTR	+	chr3:42630545-42630575	NA	0.346	6.05E-08	SE	LRRCC1	+	chr8:85115099-85115275	NA	-0.246	2.17E-05	METTL25
SE	FAAP20	-	chr1:2192844-2192975	NA	0.417	6.06E-08	SE	MON2	+	chr12:62470700-62470778	NA	-0.183	2.20E-05	METTL26
SE	SLC35C2	-	chr20:46355801-46355874	NA	0.143	6.22E-08	SE	ADARB1	+	chr21:45184473-45184593	NA	-0.343	2.24E-05	METTL2B
SE	ZNF584	+	chr19:58410005-58410091	NA	0.589	6.35E-08	SE	ANO10	-	chr3:43551471-43551588	NA	-0.115	2.25E-05	METTL4
SE	TJAP1	+	chr6:43478658-43478718	NA	0.136	6.56E-08	SE	PRKRIP1	+	chr7:102395976-102396537	NA	-0.361	2.37E-05	METTL6
SE	SH3YL1	-	chr2:242797-242871	NA	0.127	6.59E-08	SE	TMEM178A	+	chr2:39707048-39707186	NA	-0.269	2.40E-05	MFF
SE	RAB3IP	+	chr12:69812777-69812877	NA	0.262	6.76E-08	SE	CLCN3	+	chr4:169717791-169717867	NA	-0.136	2.41E-05	MFN1
SE	ZNF711	+	chrX:85246929-85247183	NA	0.32	7.32E-08	SE	C1RL	-	chr12:7108250-7108479	NA	-0.435	2.43E-05	MFSD10
SE	KLHL24	+	chr3:183636220-183636311	NA	0.354	7.54E-08	SE	ZNF226	+	chr19:44165587-44165613	NA	-0.248	2.43E-05	MFSD12
SE	USP8	+	chr15:50484274-50484361	NA	0.316	7.79E-08	SE	THOC5	-	chr22:29531077-29531110	NA	-0.132	2.46E-05	MFSD6
SE	RALGAPA1	-	chr14:35548507-35548538	NA	0.594	8.12E-08	SE	LINC00910	-	chr17:43387935-43388057	NA	-0.294	2.51E-05	MGAT5
SE	GPX4	+	chr19:1106180-1106266	NA	0.138	8.15E-08	SE	NSD2	+	chr4:1954536-1954685	NA	-0.11	2.54E-05	MGEA5
SE	FAT1	-	chr4:186590367-186590403	NA	0.184	8.32E-08	SE	TMEM241	-	chr18:23370215-23370261	NA	-0.469	2.54E-05	MGRN1
SE	MLF1	+	chr3:158588851-158588924	NA	0.118	8.38E-08	SE	CHTF8	-	chr16:69121052-69121170	NA	-0.142	2.57E-05	MIA3
SE	GUK1	+	chr1:228141123-228141507	NA	0.459	8.84E-08	SE	LYRM1	+	chr16:20902485-20902717	NA	-0.338	2.59E-05	MIATNB
SE	MTA1	+	chr14:105445666-105445852	NA	0.152	9.06E-08	SE	TPT1-AS1	+	chr13:45390713-45390902	NA	-0.109	2.67E-05	MIB2
SE	SLC7A11	-	chr4:138185120-138185244	NA	0.542	9.09E-08	SE	RBM6	+	chr3:49967197-49968748	NA	-0.109	2.69E-05	MICAL1
SE	FAAP20	-	chr1:2192646-2192975	NA	0.387	9.33E-08	SE	ANKHD1-EIF4EBI	+	chr5:140496519-140497278	NA	-0.155	2.70E-05	MICAL2
SE	SORBS1	-	chr10:95414493-95414655	NA	0.777	9.52E-08	SE	UBE3A	-	chr15:25415709-25415842	NA	-0.241	2.84E-05	MICAL3
SE	SF1	-	chr11:64771463-64771537	NA	0.208	1.08E-07	SE	BIRC5	+	chr17:78222827-78222992	NA	-0.278	2.86E-05	MIER2
SE	ATP2A1	+	chr16:28894504-28894607	NA	0.824	1.09E-07	SE	CDKL1	-	chr14:50332261-50332432	NA	-0.34	2.97E-05	MIF4GD
SE	NADK2	-	chr5:36208624-36208690	NA	0.185	1.16E-07	SE	FAM193B	-	chr5:177531281-177531521	NA	-0.319	2.98E-05	MIGA2
SE	MRPL22	+	chr5:154950802-154950938	NA	0.289	1.18E-07	SE	UPF3A	+	chr13:114298839-114299000	NA	-0.129	3.01E-05	MIIP
SE	PPA2	-	chr4:105453597-105453642	NA	0.601	1.19E-07	SE	SLC27A5	-	chr19:58481063-58481187	NA	-0.897	3.04E-05	MINDY3
SE	TANC2	+	chr17:63412679-63412709	NA	0.348	1.26E-07	SE	BCLAF1	-	chr6:136269140-136269303	NA	-0.166	3.04E-05	MINK1
SE	C17orf62	-	chr17:82449173-82449292	NA	0.309	1.37E-07	SE	UBP1	-	chr3:33396744-33396878	NA	-0.143	3.09E-05	MIOS
SE	MGRN1	+	chr16:4686258-4686303	NA	0.582	1.44E-07	SE	CCDC18	+	chr1:93183977-93184146	NA	-0.208	3.11E-05	MIR22HG
SE	ARHGAP21	-	chr10:24622732-24622762	NA	0.367	1.48E-07	SE	IGF2BP3	-	chr7:23361533-23361597	NA	-0.188	3.26E-05	MIR99AHG
SE	FAM135A	+	chr6:70486166-70486244	NA	0.442	1.50E-07	SE	C14orf93	-	chr14:23000958-23001031	NA	-0.667	3.29E-05	MIS12
SE	PRKD1	-	chr14:29656475-29656499	NA	0.259	1.68E-07	SE	CENPE	-	chr4:103143247-103143406	NA	-0.101	3.31E-05	MKKS
SE	CEP72	+	chr5:665940-666099	NA	0.538	1.80E-07	SE	VCAN	+	chr5:83537006-83542268	NA	-0.471	3.38E-05	MLF1

SE	FHL2	-	chr2:105396646-105396697	NA	0.659	1.87E-07	SE	FBXL12	-	chr19:9813230-9813274	NA	-0.533	3.50E-05	MLLT10
SE	CTNND1	+	chr11:57789036-57789155	NA	0.187	1.92E-07	SE	KIF23	+	chr15:69440767-69441079	NA	-0.258	3.51E-05	MLLT6
SE	NPHP3	-	chr3:132682702-132682818	NA	0.261	2.50E-07	SE	GGT1	+	chr22:24608989-24609231	NA	-0.515	3.61E-05	MLST8
SE	GLS	+	chr2:190913192-190913364	NA	0.119	2.51E-07	SE	CREM	+	chr10:35188199-35188388	NA	-0.257	3.64E-05	MLX
SE	JOSD2	-	chr19:50507573-50507699	NA	0.407	2.52E-07	SE	ZNF90	+	chr19:20125005-20126166	NA	-0.155	3.70E-05	MLXIP
SE	DNAJC7	-	chr17:42003358-42003557	NA	0.624	2.54E-07	SE	NR2C2AP	-	chr19:19202469-19202575	NA	-0.166	3.85E-05	MOK
SE	PDLIM5	+	chr4:94584985-94585003	NA	0.172	2.58E-07	SE	SETD9	+	chr5:56911168-56911536	NA	-0.363	3.97E-05	MON2
SE	KNTC1	+	chr12:122597730-122597938	NA	0.198	2.64E-07	SE	SUZ12P1	+	chr17:30743300-30743369	NA	-0.245	4.02E-05	MORC2
SE	CMC2	-	chr16:80980769-80980879	NA	0.286	3.19E-07	SE	PHKA1	-	chrX:72620724-72620901	NA	-0.252	4.07E-05	MORF4L1
SE	EPB41L2	-	chr6:130870327-130870381	NA	0.474	3.36E-07	SE	RP11-705C15.2	+	chr12:9656567-9656649	NA	-0.49	4.12E-05	MOSPD3
SE	SZRD1	+	chr1:16391374-16391424	NA	0.224	3.38E-07	SE	ITPRIPL1	+	chr2:96325746-96325849	NA	-0.45	4.21E-05	MOV10
SE	WDR35	-	chr2:19960553-19960614	NA	0.295	3.39E-07	SE	PSMA3-AS1	-	chr14:58293765-58295811	NA	-0.201	4.24E-05	MPDZ
SE	DCAF8	-	chr1:160261284-160261350	NA	0.367	3.58E-07	SE	NBPF8	+	chr1:120447093-120447166	NA	-0.448	4.38E-05	MPHOSPH9
SE	TMBIM6	+	chr12:49741956-49742115	NA	0.658	3.62E-07	SE	BMP2K	+	chr4:78887173-78887284	NA	-0.409	4.44E-05	MPP2
SE	MSH5-SAPCD1	+	chr6:31762789-31763168	NA	0.326	3.62E-07	SE	UBE3A	-	chr15:25408619-25408684	NA	-0.217	4.55E-05	MPPE1
SE	APLP1	+	chr19:35878081-35878108	NA	0.448	3.77E-07	SE	POLL	-	chr10:101584601-101584919	NA	-0.187	4.65E-05	MPRIP
SE	AC024560.3	-	chr3:197622186-197622330	NA	0.281	3.78E-07	SE	CLASP1	-	chr2:121445448-121445496	NA	-0.3	4.70E-05	MPV17
SE	MED23	-	chr6:131615323-131615341	NA	0.436	3.90E-07	SE	POLL	-	chr10:101585315-101585478	NA	-0.378	4.71E-05	MRM2
SE	POLE2	-	chr14:49674349-49674427	NA	0.345	4.09E-07	SE	SUOX	+	chr12:55999332-55999458	NA	-0.531	4.75E-05	MRNIP
SE	JKAMP	+	chr14:59484989-59485116	NA	0.442	4.09E-07	SE	KIAA0586	+	chr14:58543882-58543967	NA	-0.36	4.82E-05	MROH8
SE	PCBP1-AS1	-	chr2:70053730-70053792	NA	0.227	4.19E-07	SE	HNRNPR	-	chr1:23340851-23341017	NA	-0.125	4.88E-05	MRPL18
SE	METTL4	-	chr18:2544652-2544759	NA	0.245	4.20E-07	SE	PTAR1	-	chr9:69750780-69750950	NA	-0.122	4.91E-05	MRPL22
SE	SUN1	+	chr7:832504-832601	NA	0.164	4.30E-07	SE	ZNF680	-	chr7:64543706-64543802	NA	-0.377	4.99E-05	MRPL33
SE	NEXN	+	chr1:77925187-77925229	NA	0.687	4.41E-07	SE	ANKDD1A	+	chr15:64944651-64944747	NA	-0.33	5.01E-05	MRPL43
SE	ADAM22	+	chr7:88186614-88186701	NA	0.22	4.44E-07	SE	GDAP2	-	chr1:117877775-117877841	NA	-0.303	5.13E-05	MRPL47
SE	MAGI1	-	chr3:65387142-65387226	NA	0.5	4.47E-07	SE	CLEC2D	+	chr12:9693824-9693948	NA	-0.461	5.17E-05	MRPL52
SE	DGKH	+	chr13:42221263-42221394	NA	0.615	4.55E-07	SE	DOK1	+	chr2:74555893-74556078	NA	-0.164	5.43E-05	MRPL55
SE	CAST	+	chr5:96729152-96729209	NA	0.178	4.62E-07	SE	FAM86EP	-	chr4:3946625-3946939	NA	-0.559	5.49E-05	MRPS15
SE	DCAF8	-	chr1:160261284-160261358	NA	0.334	4.67E-07	SE	METTL14	+	chr4:118705610-118705821	NA	-0.105	5.49E-05	MRPS18C
SE	C17orf62	-	chr17:82447579-82447621	NA	0.405	4.76E-07	SE	NAP1L4	-	chr11:2949226-2949264	NA	-0.143	5.54E-05	MRPS28
SE	LMBR1L	-	chr12:49106653-49106746	NA	0.177	4.77E-07	SE	RSG1	-	chr1:16232892-16233017	NA	-0.557	5.59E-05	MRPS31P4
SE	RWDD1	+	chr6:116574057-116574171	NA	0.198	4.92E-07	SE	EPB41L2	-	chr6:130876673-130876775	NA	-0.296	5.60E-05	MRPS6
SE	CSNK1G3	+	chr5:123605338-123605362	NA	0.597	5.04E-07	SE	PTPN4	+	chr2:119881785-119881830	NA	-0.222	5.64E-05	MRRF
SE	C17orf62	-	chr17:82446622-82446696	NA	0.197	5.07E-07	SE	ANKAR	+	chr2:189705022-189705224	NA	-0.38	6.03E-05	MSH5-SAPCD1
SE	ARHGEF11	-	chr1:156938417-156938513	NA	0.352	5.07E-07	SE	PATZ1	-	chr22:31328786-31328924	NA	-0.176	6.08E-05	MSI2
SE	ZFYVE27	+	chr10:97743093-97743164	NA	0.398	5.08E-07	SE	MAP4K4	+	chr2:101860824-101860986	NA	-0.384	6.13E-05	MSL1
SE	GOLT1B	+	chr12:21507943-21508109	NA	0.315	5.26E-07	SE	ZNF680	-	chr7:64544305-64544432	NA	-0.37	6.13E-05	MSRA
SE	NFYA	+	chr6:41080810-41080897	NA	0.521	5.31E-07	SE	ZEB1	+	chr10:31387123-31387266	NA	-0.257	6.18E-05	MSRB3
SE	C17orf62	-	chr17:82449169-82449292	NA	0.264	5.41E-07	SE	CEP63	+	chr3:134547334-134547472	NA	-0.173	6.22E-05	MSTO1
SE	MYO19	-	chr17:36501851-36501961	NA	0.2	5.91E-07	SE	HNRNPR	-	chr1:23340851-23341020	NA	-0.127	6.33E-05	MTA1
SE	TOM1L2	-	chr17:17857768-17857855	NA	0.335	6.07E-07	SE	UBE2Q2P2	+	chr15:82408287-82408367	NA	-0.354	6.56E-05	MTCL1
SE	SH3GLB2	-	chr9:129010669-129010693	NA	0.201	6.34E-07	SE	PCNX1	+	chr14:70995740-70995925	NA	-0.362	6.66E-05	MTERF4
SE	MRPL43	-	chr10:100983947-100984074	NA	0.71	6.77E-07	SE	VPS8	+	chr3:184922428-184922483	NA	-0.122	6.66E-05	MTF2
SE	TTC28	-	chr22:27990788-27990812	NA	0.572	6.78E-07	SE	PRC1	-	chr15:90969078-90969120	NA	-0.172	6.70E-05	MTG1
SE	WDR27	-	chr6:169665485-169665556	NA	0.439	6.79E-07	SE	HAUS2	+	chr15:42560781-42560870	NA	-0.212	6.71E-05	MTHFD1L
SE	ZNF391	+	chr6:27395016-27395095	NA	0.698	7.31E-07	SE	RP11-42I10.1	+	chr16:48671860-48672033	NA	-0.342	6.74E-05	MTIF2
SE	PFDN5	+	chr12:53296429-53296551	NA	0.175	7.32E-07	SE	DUS4L	+	chr7:107573703-107573883	NA	-0.291	6.90E-05	MTIF3
SE	DLEU1	+	chr13:50083141-50083194	NA	0.268	8.10E-07	SE	LL22NC03-80A10	+	chr22:22308696-22308819	NA	-0.224	6.91E-05	MTMR11
SE	RCL1	+	chr9:4833153-4833228	NA	0.141	8.10E-07	SE	FKTN	+	chr9:105619933-105620061	NA	-0.164	7.32E-05	MTMR2
SE	COQ5	-	chr12:120526456-120526546	NA	0.116	8.30E-07	SE	LIG4	-	chr13:108214537-108214807	NA	-0.237	7.32E-05	MTMR3
SE	WDR35	-	chr2:19932382-19932447	NA	0.27	8.36E-07	SE	CDKL3	-	chr5:134312291-134312380	NA	-0.456	7.45E-05	MTMR4
SE	PIBF1	+	chr13:72783422-72783721	NA	0.156	9.12E-07	SE	PRR34-AS1	+	chr22:46055598-46055733	NA	-0.455	7.54E-05	MTMR9LP
SE	CIRBP	+	chr19:1273493-1273715	NA	0.369	9.30E-07	SE	POLL	-	chr10:101585861-101586156	NA	-0.208	7.54E-05	MTRF1L
SE	CLASP1	-	chr2:121409023-121409047	NA	0.444	9.89E-07	SE	PARPBP	+	chr12:102195311-102195447	NA	-0.408	7.60E-05	MTSS1L
SE	ACO1	+	chr9:32386370-32386438	NA	0.159	9.98E-07	SE	SLC37A3	-	chr7:140345215-140345263	NA	-0.127	7.67E-05	MTTP
SE	STXBP1	+	chr9:127684367-127684493	NA	0.11	1.02E-06	SE	PSMA3-AS1	-	chr14:58273978-58274135	NA	-0.237	7.74E-05	MTX1
SE	SORBS1	-	chr10:95414493-95414862	NA	0.681	1.03E-06	SE	CNOT2	+	chr12:70285399-70285586	NA	-0.556	8.05E-05	MTX3
SE	PPM1K	-	chr4:88278143-88278642	NA	0.38	1.05E-06	SE	PGPEP1	+	chr19:18357382-18357615	NA	-0.297	8.09E-05	MUS81
SE	TNRC6A	+	chr16:24793472-24793649	NA	0.254	1.07E-06	SE	TWF1	-	chr12:43805830-43805885	NA	-0.612	8.15E-05	MUTYH
SE	SUZ12P1	+	chr17:30734896-30734943	NA	0.583	1.11E-06	SE	HM13	+	chr20:31568077-31568224	NA	-0.252	8.23E-05	MVD
SE	ARIH2	+	chr3:48945135-48945181	NA	0.362	1.11E-06	SE	GOSR2	+	chr17:46931104-46931207	NA	-0.498	8.57E-05	MVK
SE	CCHCR1	-	chr6:31157384-31157500	NA	0.328	1.20E-06	SE	ZSCAN31	-	chr6:28329302-28329714	NA	-0.38	8.71E-05	MX1
SE	ARHGAP11B	+	chr15:30641521-30641617	NA	0.456	1.21E-06	SE	TMEM218	-	chr11:125110643-125110746	NA	-0.631	8.71E-05	MXRA7
SE	NUMB	-	chr14:73316389-73316422	NA	0.209	1.27E-06	SE	ZNF254	+	chr19:24105939-24106066	NA	-0.269	8.74E-05	MYCBP2
SE	BCL2L2	+	chr14:23308815-23308932	NA	0.394	1.28E-06	SE	RFFL	-	chr17:35021370-35021781	NA	-0.159	8.79E-05	MYEF2
SE	NDUFS8	+	chr11:68035627-68035788	NA	0.114	1.32E-06	SE	PHF5A	-	chr22:41468019-41468147	NA	-0.127	8.80E-05	MYH14
SE	PPIL3	-	chr2:200882341-200882382	NA	0.525	1.32E-06	SE	ELMOD3	+	chr2:85355554-85355598	NA	-0.498	9.02E-05	MYL6
SE	MPHOSPH9	-	chr12:123163013-123163134	NA	0.157	1.33E-06	SE	FANCD2	+	chr3:10047916-10048051	NA	-0.29	9.02E-05	MYLK
SE	TOP1MT	-	chr8:143334107-143334285	NA	0.35	1.38E-06	SE	TRIM5	-	chr11:5665980-5666081	NA	-0.147	9.07E-05	MYNN
SE	LETM2	+	chr8:38406945-38407038	NA	0.437	1.41E-06	SE	ETV1	-	chr7:13989267-13989464	NA	-0.291	9.08E-05	MYO15B
SE	METTL4	-	chr18:2544194-2544286	NA	0.201	1.41E-06	SE	ADAMTS13	+	chr9:133438245-133438366	NA	-0.333	9.27E-05	MYO18A
SE	SREK1	+	chr5:66156064-66158932	NA	0.327	1.42E-06	SE	CLHC1	-	chr2:55208625-55208710	NA	-0.498	9.57E-05	MYO19
SE	PCMTD1	-	chr8:51839446-51839638	NA	0.325	1.42E-06	SE	MRNIP	-	chr5:179851242-179851424	NA	-0.145	9.74E-05	MYO5A
SE	SORBS3	+	chr8:22565825-22565872	NA	0.697	1.53E-06	SE	DDIAS	+	chr11:82913282-82913386	NA	-0.22	9.98E-05	MYO6
SE	CIRBP	+	chr19:1273600-1273715	NA	0.456	1.72E-06	SE	MAN2B2	+	chr4:6609396-6609574	NA	-0.313	0.0001010478751	MYO9A
SE	DDX50	+	chr10:68906225-68906337	NA	0.106	1.74E-06	SE	STAG2	+	chrX:124021366-124021431	NA	-0.307	0.0001010478751	MYT1
SE	ZCCHC8	-	chr12:122490461-122490567	NA	0.176	1.79E-06	SE	CAMTA1	+	chr1:6820180-6820250	NA	-0.2	0.0001018934292	MZF1
SE	GGCX	-	chr2:85552415-85552567	NA	0.196	1.86E-06	SE	WASF1	-	chr6:110160634-110160732	NA	-0.222	0.0001020999114	N4BP2
SE	PCBP1-AS1	-	chr2:70053730-70053792	NA	0.359	2.03E-06	SE	EED	+	chr11:86268455-86268561	NA	-0.104	0.0001028447021	N4BP2L2
SE	SCAMP3	-	chr1:155261656-155261734	NA	0.181	2.09E-06	SE	PTK2	-	chr8:140948665-140948723	NA	-0.211	0.0001040508253	N6AMT1
SE	ZDHHC6	-	chr10:112440533-112440695	NA	0.146	2.19E-06	SE	SLC50A1	+	chr1:155136827-155136951	NA	-0.296	0.0001103751914	NAA16
SE	CAMK2G	-	chr10:73819531-73819645	NA	0.182	2.25E-06	SE	BCS1L	+	chr2:218660036-218660166	NA	-0.25	0.0001103973794	NAA60
SE	CRBN	-	chr3:3153959-3154075	NA	0.177	2.26E-06	SE	ATP11B	+	chr3:182902480-182902566	NA	-0.436	0.0001130729132	NAB1
SE	MPPE1	-	chr18:11897525-11897620	NA	0.665	2.32E-06	SE	DDR1	+	chr6:30885624-30885680	NA	-0.447	0.0001146660205	NABP1
SE	BPTF	+	chr17:67875555-67875744	NA	0.174	2.33E-06	SE	ANGEL1	-	chr14:76810088-76810254	NA	-0.124	0.0001146705376	NADK
SE	PHACTR4	+	chr1:28457831-28457855	NA	0.538	2.41E-06	SE	ZSCAN31	-	chr6:28329302-28329778	NA	-0.367	0.0001149006239	NADK2
SE	NFASC	+	chr1:204962107-204962158	NA	0.41	2.42E-06	SE	CA5B	+	chrX:15772495-15772614	NA	-0.418	0.0001159793671	NADSYN1
SE	CKLF	+	chr16:66563121-66563217	NA	0.109	2.44E-06	SE	TMEM234	-	chr1:32217436-32217577	NA	-0.441	0.000117787783	NAGK
SE	RBM6	+	chr3:49975322-49975392	NA	0.313	2.49E-06	SE	ELMOD3	+	chr2:85387097-85387225	NA	-0.375	0.0001184888955	NAGLU
SE	LAS1L	-	chrX:65524563-65524614	NA	0.365	2.58E-06	SE	SEPT2	+	chr2:241324408-241324662	NA	-0.489	0.0001196803573	NAGPA
SE	AP3B1	-	chr5:78175625-78175697	NA	0.109	2.62E-06	SE	RPAIN	+	chr17:5425970-5428211	NA	-0.166	0.0001203924305	NAGS
SE	SVIL	-	chr10:29532001-29532172	NA	0.376	2.63E-06	SE	AC004076.9	-	chr19:57475659-57475786	NA	-0.498	0.0001214203275	NAP1L1
SE	PPIP5K2	+	chr5:103187313-103187376	NA	0.314	2.71E-06	SE	ZNF155	+	chr19:43990023-43990112	NA	-0.207	0.0001224929477	NAP1L4
SE	WDR27	-	chr6:169670568-169670693	NA	0.297	2.79E-06	SE	STRN3	-	chr14:30929200-30929311	NA	-0.205	0.0001232390093	NAPEPLD
SE	ZNF519	-	chr18:14080188-14080266	NA	0.373	2.79E-06	SE	RSRC2	-	chr12:122526169-122526293	NA	-0.517	0.0001240921588	NAPRT
SE	OMA1	-	chr1:58530600-58530729	NA	0.167	3.05E-06	SE	SEC14L2	+	chr22:30415947-30416087	NA	-0.154	0.0001264740287	NARFL
SE	AGTPBP1	-	chr9:85678334-85678398	NA	0.197	3.06E-06	SE	OSBP2	+	chr22:30889489-30889636	NA	-0.246	0.0001277040789	NASP
SE	PKP4	+	chr2:158676738-158676867	NA	0.165	3.10E-06	SE	LRRC37B	+	chr17:32045699-32045818	NA	-0.181	0.0001290126533	NAT1
SE	GSN	+	chr9:121300055-121300126	NA	0.835	3.15E-06	SE	NAA16	+	chr13:41336649-41336756	NA	-0.191	0.000130332972	NAT9
SE	PLD3	+	chr19:40365552-40365585	NA	0.65	3.15E-06	SE	TCAF1	-	chr7:143881922-143882099	NA	-0.57	0.0001331782875	NBEAL1
SE	MLLT6	+	chr17:38721877-38722227	NA	0.413	3.20E-06	SE	ERBIN	+	chr5:66068876-66069020	NA	-0.454	0.0001331799491	NBN
SE	TNFSF12-TNFS	+	chr17:7559623-7559702	NA	0.503	3.25E-06	SE	FAM114A2	-	chr5:154038292-154038434	NA	-0.185	0.0001340157811	NBPF1
SE	PARP8	+	chr5:50668070-50668125	NA	0.293	3.37E-06	SE	STK16	+	chr2:219247112-219247246	NA	-0.101	0.0001349078205	NBPF11
SE	SLC29A1	+	chr6:44225972-44226167	NA	0.228	3.37E-06	SE	INIP	-	chr9:112701787-112701880	NA	-0.115	0.0001351758142	NBPF12
SE	PXN	-	chr12:120215929-120216090	NA	0.416	3.39E-06	SE	CLHC1	-	chr2:55209403-55209516	NA	-0.431	0.00013661978	NBPF26
SE	AGAP1	+	chr2:235750353-235750488	NA	0.18	3.67E-06	SE	CCDC150	+	chr2:196677114-196677192	NA	-0.528	0.0001382276306	NBPF8
SE	N4BP2L2	-	chr13:32538617-32538684	NA	0.145	3.80E-06	SE	CHFR	-	chr12:132869618-132869834	NA	-0.261	0.0001382408096	NBPF9
SE	RAB34	-	chr17:28717212-28717291	NA	0.418	4.03E-06	SE	TFR2	-	chr7:100629252-100629372	NA	-0.294	0.0001387535683	NBR2
SE	BCL2L13	+	chr22:17696140-17696210	NA	0.207	4.22E-06	SE	RB1CC1	-	chr8:52685936-52686156	NA	-0.105	0.0001409018816	NCK2
SE	EEF1D	-	chr8:143592646-143592797	NA	0.512	4.25E-06	SE	DDR1	+	chr6:30884537-30884710	NA	-0.449	0.0001410645129	NCOA1
SE	RTN4	-	chr2:55028163-55028220	NA	0.106	4.37E-06	SE	YEATS4	+	chr12:69365789-69365884	NA	-0.172	0.0001418200435	NCOA2
SE	TEX30	-	chr13:102770011-102770086	NA	0.4	4.43E-06	SE	ISCU	+	chr12:108564059-108564155	NA	-0.321	0.0001418305399	NCOR2
SE	EPB41L2	-	chr6:130870327-130870381	NA	0.548	4.47E-06	SE	GINS3	+	chr16:58395101-58395218	NA	-0.228	0.0001444542877	NDE1
SE	ZCWPW2	+	chr3:28478813-28478931	NA	0.377	4.60E-06	SE	PLEKHH1	+	chr14:67586809-67586949	NA	-0.12	0.0001479566393	NDEL1
SE	KTN1	+	chr14:55663954-55664041	NA	0.262	4.64E-06	SE	TWF1	-	chr12:43805830-43806002	NA	-0.36	0.0001485744595	NDOR1
SE	ABHD14B	-	chr3:51971811-51971892	NA	0.435	4.64E-06	SE	BBS1	+	chr11:66523171-66523365	NA	-0.462	0.0001503356103	NDUFA3
SE	PGAP2	+	chr11:3811249-3811424	NA	0.332	4.64E-06	SE	ENTPD1	+	chr10:95842343-95842494	NA	-0.413	0.0001503356103	NDUFAF6
SE	ZNF326	+	chr1:90007611-90007750	NA	0.173	5.06E-06	SE	CCDC15	+	chr11:124991460-124991583	NA	-0.46	0.0001503356103	NDUFAF7
SE	ZNF33B	-	chr10:42636919-42636972	NA	0.245	5.20E-06	SE	IGF2BP3	-	chr7:23361533-23361597	NA	-0.178	0.0001511590587	NDUFB2
SE	PNPLA3	+	chr22:43934605-43934666	NA	0.268	5.34E-06	SE	CSAD	-	chr12:53171176-53171441	NA	-0.51	0.0001524546588	NDUFC1
SE	UBA2	+	chr19:34430092-34430186	NA	0.399	5.38E-06	SE	MIOS	+	chr7:7567606-7567688	NA	-0.497	0.0001530389937	NDUFS5

SE	ICA1	-	chr7:8138984-8139047	NA	0.216	5.39E-06	SE	TCF7L2	+	chr10:113160618-113160691	NA	-0.805	0.0001537967219	NDUFS8
SE	SRRM1	+	chr1:24663182-24663224	NA	0.254	5.46E-06	SE	ZC3H14	+	chr14:88601923-88602083	NA	-0.325	0.0001541382425	NECAB3
SE	TMEM135	+	chr11:87071522-87071615	NA	0.729	5.59E-06	SE	PICALM	-	chr11:85990249-85990399	NA	-0.349	0.0001596972278	NEDD1
SE	MFF	+	chr2:227352513-227352573	NA	0.145	5.60E-06	SE	SUV39H2	+	chr10:14881499-14881645	NA	-0.195	0.0001620470404	NEDD4
SE	TSSC4	+	chr11:2401838-2401979	NA	0.319	5.66E-06	SE	HDAC9	+	chr7:18629349-18629481	NA	-0.279	0.0001629656818	NEK3
SE	MPHOSPH9	-	chr12:123226287-123226351	NA	0.235	5.93E-06	SE	TNRC18	-	chr7:5361593-5361722	NA	-0.177	0.0001631757984	NEK6
SE	CLIP1	-	chr12:122347374-122347479	NA	0.242	5.93E-06	SE	RPS3A	+	chr4:151100974-151101012	NA	-0.255	0.0001633148417	NEK7
SE	XRRA1	-	chr11:74940784-74940882	NA	0.728	5.96E-06	SE	PLCB4	+	chr20:9373046-9373104	NA	-0.482	0.0001633148417	NEK8
SE	DTWD1	+	chr15:49631000-49631058	NA	0.137	6.02E-06	SE	POLL	-	chr10:101584601-101584919	NA	-0.155	0.0001700015266	NELFE
SE	ZNF83	-	chr19:52617245-52617335	NA	0.838	6.02E-06	SE	IGF2BP2	-	chr3:185672540-185672669	NA	-0.175	0.0001714835135	NEMP1
SE	DDX42	+	chr17:63799588-63799625	NA	0.121	6.10E-06	SE	RNASEH1-AS1	+	chr2:3559447-3559729	NA	-0.187	0.0001717190751	NENF
SE	FBXO24	+	chr7:100595101-100595223	NA	0.414	6.11E-06	SE	CACNB4	-	chr2:151841667-151841739	NA	-0.193	0.0001723696749	NEO1
SE	STIM1	+	chr11:4084672-4084765	NA	0.381	6.19E-06	SE	NISCH	+	chr3:52480878-52481037	NA	-0.453	0.0001737618393	NEPRO
SE	APTX	-	chr9:32988082-32988124	NA	0.321	6.31E-06	SE	FAM210A	-	chr18:13671861-13671973	NA	-0.142	0.000175916401	NETO2
SE	BLOC1S1	+	chr12:55716932-55717005	NA	0.104	6.58E-06	SE	LRRK2	+	chr12:40313971-40314173	NA	-0.211	0.0001763959562	NEXN
SE	FANCD2	+	chr3:10064355-10064429	NA	0.229	6.59E-06	SE	DIAPH2	+	chrX:96763099-96763120	NA	-0.514	0.0001769287926	NF1
SE	ASAP2	+	chr2:9391061-9391196	NA	0.539	6.77E-06	SE	FAM45A	+	chr10:119105310-119105495	NA	-0.304	0.0001800778006	NF2
SE	POLA1	+	chrX:24725980-24726055	NA	0.155	6.82E-06	SE	ZNF253	+	chr19:19880050-19880146	NA	-0.377	0.0001810450478	NFASC
SE	SEC31A	-	chr4:82862533-82862572	NA	0.268	7.13E-06	SE	MANBA	-	chr4:102753822-102753995	NA	-0.228	0.0001816263906	NFATC3
SE	TAZ	+	chrX:154419704-154419746	NA	0.431	7.20E-06	SE	MVK	+	chr12:109581394-109581550	NA	-0.22	0.0001835601539	NFE2L1
SE	PSMA3-AS1	-	chr14:58288717-58288813	NA	0.432	7.31E-06	SE	TRIQK	-	chr8:92921502-92921660	NA	-0.286	0.000185566505	NFIB
SE	MRPL18	+	chr6:159790667-159790787	NA	0.156	7.43E-06	SE	SNAP23	+	chr15:42529674-42529819	NA	-0.129	0.0001857822036	NFIC
SE	SVIL	-	chr10:29526960-29527056	NA	0.202	7.64E-06	SE	NFRKB	-	chr11:129893296-129893385	NA	-0.497	0.0001859770855	NFRKB
SE	CACNB4	-	chr2:151869176-151869235	NA	0.65	7.65E-06	SE	BLOC1S6	+	chr15:45592134-45592276	NA	-0.153	0.0001872045859	NFYA
SE	TEAD4	+	chr12:2959947-2960353	NA	0.59	7.68E-06	SE	C12orf4	-	chr12:4517060-4517189	NA	-0.142	0.0001872045859	NFYC
SE	FBRS	+	chr16:30661303-30661333	NA	0.439	8.18E-06	SE	MRPS31P4	+	chr13:52629218-52629359	NA	-0.323	0.0001872472194	NGLY1
SE	DET1	-	chr15:88512350-88513140	NA	0.232	8.30E-06	SE	LPCAT4	-	chr15:34361399-34361532	NA	-0.164	0.0001905212064	NHLRC3
SE	ARHGEF7	+	chr13:111288353-111288443	NA	0.162	8.37E-06	SE	POLL	-	chr10:101585861-101586156	NA	-0.26	0.0001954185169	NHSL1
SE	GAA	+	chr17:80101810-80101890	NA	0.367	8.37E-06	SE	MBD5	+	chr2:148053932-148054050	NA	-0.366	0.0001956341426	NICN1
SE	MTMR3	+	chr22:30023456-30023483	NA	0.277	8.40E-06	SE	SNX10	+	chr7:26357006-26357127	NA	-0.215	0.0001989304633	NID2
SE	PARD3	-	chr10:34372497-34372536	NA	0.263	8.40E-06	SE	PVR	+	chr19:44658741-44658765	NA	-0.478	0.0002048526225	NIN
SE	GATC	+	chr12:120454932-120455030	NA	0.123	8.48E-06	SE	LINC00630	+	chrX:102825992-102826169	NA	-0.467	0.0002076409456	NINL
SE	GUSBP11	-	chr22:23700725-23700845	NA	0.608	8.54E-06	SE	HEATR6	-	chr17:60073745-60073886	NA	-0.144	0.000218129136	NIPA2
SE	STRADA	-	chr17:63726637-63726695	NA	0.364	9.33E-06	SE	SH3PXD2A	-	chr10:103622469-103622553	NA	-0.25	0.000219648813	NISCH
SE	SLC2A11	+	chr22:23877384-23877869	NA	0.319	9.42E-06	SE	NUDT22	+	chr11:64229849-64230070	NA	-0.145	0.000222018372	NIT2
SE	PHACTR4	+	chr1:28457831-28457855	NA	0.626	9.42E-06	SE	ZMYM5	-	chr13:19838699-19838985	NA	-0.124	0.0002257543833	NKIRAS2
SE	XPO6	-	chr16:28155701-28155771	NA	0.514	1.03E-05	SE	FAM204A	-	chr10:118341726-118341927	NA	-0.26	0.00022583759	NKTR
SE	CEP192	+	chr18:13058293-13058435	NA	0.104	1.04E-05	SE	IST1	+	chr16:71903164-71903258	NA	-0.742	0.0002334056595	NLE1
SE	TMEM219	+	chr16:29972041-29972140	NA	0.129	1.05E-05	SE	EBPL	-	chr13:49669776-49669846	NA	-0.257	0.0002342420853	NLRX1
SE	NPHP3	-	chr3:132696730-132696813	NA	0.34	1.09E-05	SE	RAB28	-	chr4:13369870-13369965	NA	-0.154	0.0002377188118	NME4
SE	SUN1	+	chr7:849519-849603	NA	0.489	1.12E-05	SE	MPDZ	-	chr9:13115247-13115334	NA	-0.152	0.0002389659815	NME6
SE	MFF	+	chr2:227352513-227352573	NA	0.165	1.13E-05	SE	NFIC	+	chr19:3453762-3453916	NA	-0.281	0.0002392046182	NMNAT3
SE	RBM23	-	chr14:22908332-22908380	NA	0.228	1.15E-05	SE	CAPRIN2	-	chr12:30719078-30720915	NA	-0.305	0.0002400924652	NMT2
SE	EPB41L2	-	chr6:130876673-130876775	NA	0.387	1.18E-05	SE	CHFR	-	chr12:132869618-132869798	NA	-0.231	0.000242143471	NOC3L
SE	PILRB	+	chr7:100353372-100354012	NA	0.217	1.18E-05	SE	TPRKB	-	chr2:73732583-73732700	NA	-0.164	0.0002432746919	NOD1
SE	SLC29A1	+	chr6:44226060-44226167	NA	0.312	1.18E-05	SE	BCL2L11	+	chr2:111124049-111124139	NA	-0.168	0.0002445589411	NOL8
SE	UBE2G2	-	chr21:44774566-44774741	NA	0.299	1.20E-05	SE	SLC4A7	-	chr3:27379248-27379356	NA	-0.315	0.0002462880971	NOMO2
SE	TBC1D32	-	chr6:121090852-121091041	NA	0.271	1.21E-05	SE	NEK3	-	chr13:52135728-52135863	NA	-0.128	0.000253614227	NONO
SE	PCBP1-AS1	-	chr2:70053730-70053792	NA	0.315	1.22E-05	SE	PPP4R1L	-	chr20:58271362-58271505	NA	-0.253	0.0002564344844	NOP56
SE	ZNF83	-	chr19:52616717-52616815	NA	0.291	1.25E-05	SE	CLHC1	-	chr2:55181569-55181744	NA	-0.264	0.0002588502674	NOVA1-AS1
SE	GUSB	-	chr7:65974918-65975071	NA	0.188	1.27E-05	SE	FAM76B	-	chr11:95779077-95779135	NA	-0.393	0.0002600015158	NPAS2
SE	HPS1	-	chr10:98431130-98431291	NA	0.43	1.28E-05	SE	USP25	+	chr21:15849776-15849872	NA	-0.116	0.0002637844158	NPHP1
SE	RAD51AP1	+	chr12:4545218-4545312	NA	0.258	1.28E-05	SE	KATNAL1	-	chr13:30255446-30255615	NA	-0.107	0.0002645918642	NPHP3
SE	SPATS2	+	chr12:49371227-49371290	NA	0.28	1.33E-05	SE	SOX9-AS1	-	chr17:72074548-72076733	NA	-0.145	0.0002651228345	NPR2
SE	MED24	-	chr17:40020527-40020619	NA	0.695	1.34E-05	SE	PRKRIP1	+	chr7:102395976-102396537	NA	-0.301	0.000271826994	NPRL3
SE	PEX3	+	chr6:143472159-143472328	NA	0.146	1.34E-05	SE	MFF	+	chr2:227328677-227328789	NA	-0.276	0.0002739113856	NQO1
SE	ATAD5	+	chr17:30879422-30879487	NA	0.194	1.38E-05	SE	CHEK1	+	chr11:125656205-125656335	NA	-0.313	0.0002812419842	NQO2
SE	ZNF83	-	chr19:52616717-52616875	NA	0.245	1.38E-05	SE	E2F6	-	chr2:11455341-11455474	NA	-0.203	0.000284447168	NR2C2
SE	UGP2	+	chr2:63842538-63842599	NA	0.155	1.39E-05	SE	PARPBP	+	chr12:102182548-102182627	NA	-0.311	0.0002851607735	NR2C2AP
SE	EEF1D	-	chr8:143590607-143590729	NA	0.51	1.39E-05	SE	STX16-NPEPL1	+	chr20:58691404-58691562	NA	-0.512	0.0002874981515	NR2F1-AS1
SE	GUK1	+	chr1:228148370-228148539	NA	0.187	1.39E-05	SE	CSAD	-	chr12:53171272-53171441	NA	-0.52	0.0002923670232	NR4A1
SE	WASHC2C	+	chr10:45751481-45751553	NA	0.143	1.48E-05	SE	SEC14L2	+	chr22:30415758-30416087	NA	-0.117	0.0002925652383	NRBP2
SE	LTBP4	+	chr19:40622949-40623021	NA	0.236	1.52E-05	SE	RWDD3	+	chr1:95239776-95239909	NA	-0.503	0.0002931707329	NRG2
SE	DYNC2H1	+	chr11:103287532-103287605	NA	0.228	1.53E-05	SE	RHNO1	+	chr12:2885282-2885534	NA	-0.109	0.000293221581	NRM
SE	STAMBP	+	chr2:73829404-73829510	NA	0.245	1.54E-05	SE	RWDD3	+	chr1:95237347-95237460	NA	-0.386	0.0002933051974	NSD1
SE	ARHGEF7	+	chr13:111209871-111210002	NA	0.238	1.63E-05	SE	PVR	+	chr19:44658741-44658765	NA	-0.441	0.0002963289862	NSD2
SE	TICRR	+	chr15:89592046-89592176	NA	0.22	1.65E-05	SE	NRG2	-	chr5:139871043-139871148	NA	-0.776	0.0003004649703	NSFL1C
SE	ADGRL2	+	chr1:81987288-81987341	NA	0.256	1.66E-05	SE	MCAT	-	chr22:43137080-43137298	NA	-0.307	0.0003041334185	NSMF
SE	STAU1	-	chr20:49165996-49166285	NA	0.133	1.66E-05	SE	FANCA	-	chr16:89742799-89742938	NA	-0.113	0.000310718969	NSRP1
SE	CCNE1	+	chr19:29821995-29822130	NA	0.164	1.69E-05	SE	LAMP2	-	chrX:120439075-120439293	NA	-0.194	0.000310718969	NSUN2
SE	ELP2	+	chr18:36138794-36138872	NA	0.133	1.86E-05	SE	SMARCC2	-	chr12:56164302-56164368	NA	-0.483	0.0003110222918	NSUN4
SE	TMEM116	-	chr12:112003799-112003863	NA	0.416	1.88E-05	SE	HACL1	-	chr3:15574976-15575082	NA	-0.111	0.0003159746893	NSUN5
SE	MPHOSPH9	-	chr12:123162114-123162218	NA	0.166	1.93E-05	SE	ZC3H14	+	chr14:88602827-88603045	NA	-0.265	0.000317236808	NSUN5P1
SE	CDCA7	+	chr2:173359254-173359491	NA	0.168	1.93E-05	SE	BCS1L	+	chr2:218661405-218661545	NA	-0.112	0.000321897375	NT5C3A
SE	PPIL3	-	chr2:200885261-200885367	NA	0.339	1.93E-05	SE	ORC2	-	chr2:200925835-200925932	NA	-0.175	0.0003271679084	NTAN1
SE	ARHGEF10	+	chr8:1926376-1926463	NA	0.138	1.97E-05	SE	TPRKB	-	chr2:73734527-73734588	NA	-0.182	0.0003277096277	NUDT13
SE	EEF1D	-	chr8:143590607-143590735	NA	0.471	1.99E-05	SE	FAM200B	+	chr4:15684849-15684959	NA	-0.284	0.0003323528096	NUDT17
SE	SMARCC2	-	chr12:56172936-56173029	NA	0.191	2.01E-05	SE	CHPF2	+	chr7:151236955-151237172	NA	-0.137	0.0003362019884	NUDT2
SE	PAQR3	-	chr4:78935120-78935176	NA	0.61	2.01E-05	SE	PROCA1	-	chr17:28706679-28706763	NA	-0.381	0.0003369731875	NUDT22
SE	NBN	-	chr8:89981952-89982002	NA	0.189	2.01E-05	SE	RP11-10K16.1	-	chr4:173166644-173166790	NA	-0.284	0.000342014704	NUDT8
SE	PILRB	+	chr7:100353372-100354012	NA	0.245	2.05E-05	SE	C1orf43	-	chr1:154219835-154219937	NA	-0.126	0.0003434288658	NUMA1
SE	SDR39U1	-	chr14:24442108-24442260	NA	0.447	2.11E-05	SE	SLC50A1	+	chr1:155137560-155137722	NA	-0.133	0.0003522126727	NUMB
SE	SYTL4	-	chrX:100703092-100703185	NA	0.392	2.13E-05	SE	MRNIP	-	chr5:179853196-179853276	NA	-0.526	0.0003544351238	NUP214
SE	SYTL2	-	chr11:85704857-85705028	NA	0.229	2.15E-05	SE	SEC14L2	+	chr22:30415758-30415865	NA	-0.109	0.0003667018751	NUP50
SE	MCCC2	+	chr5:71626639-71626753	NA	0.267	2.16E-05	SE	TPM1	+	chr15:63064063-63064190	NA	-0.394	0.0003706949144	NUP54
SE	CRYZ	-	chr1:74707102-74707204	NA	0.138	2.18E-05	SE	EZH2	-	chr7:148836821-148837000	NA	-0.269	0.000373023368	NUP58
SE	RFWD2	-	chr1:176136487-176136547	NA	0.29	2.21E-05	SE	ST3GAL6-AS1	-	chr3:98715649-98715777	NA	-0.232	0.0003731111346	NUP88
SE	FLVCR1	+	chr1:212863724-212863869	NA	0.221	2.23E-05	SE	CCNF	+	chr16:2432960-2433067	NA	-0.145	0.0003781219961	NUP93
SE	RP11-452F19.3	+	chr1:222823855-222823945	NA	0.305	2.24E-05	SE	PVR	+	chr19:44658741-44658900	NA	-0.204	0.0003788723075	NUSAP1
SE	MADD	+	chr11:47326737-47326807	NA	0.171	2.26E-05	SE	ZNF141	+	chr4:343781-343908	NA	-0.248	0.000379898374	NUTM2A-AS1
SE	USP36	-	chr17:78797785-78797879	NA	0.382	2.27E-05	SE	LPIN1	+	chr2:11776085-11776193	NA	-0.162	0.0003811031107	NVL
SE	BORA	+	chr13:72731280-72731387	NA	0.306	2.29E-05	SE	ORC2	-	chr2:200926767-200926900	NA	-0.171	0.0003867219596	OARD1
SE	CORO7	-	chr16:4415714-4415852	NA	0.117	2.30E-05	SE	RP4-614C10.2	-	chr2:89580557-89580635	NA	-0.337	0.0003980473053	OBSL1
SE	SNX21	+	chr20:45840321-45840458	NA	0.621	2.35E-05	SE	SLC16A4	-	chr1:110378852-110379356	NA	-0.19	0.0004009114888	OCIAD1
SE	COL4A3BP	-	chr5:75399309-75399387	NA	0.146	2.38E-05	SE	ODF2L	-	chr1:86356443-86356602	NA	-0.303	0.0004028475812	ODF2L
SE	TJP1	-	chr15:29719776-29720016	NA	0.238	2.38E-05	SE	PREPL	-	chr2:44359496-44360038	NA	-0.263	0.000403764681	OFD1
SE	SERPINB6	-	chr6:2968778-2968913	NA	0.103	2.38E-05	SE	BCS1L	+	chr2:218660938-218661307	NA	-0.11	0.0004065369557	OGDH
SE	SUPT20H	-	chr13:37010551-37010655	NA	0.261	2.46E-05	SE	RP13-968A2.1	-	chr7:99299463-99299666	NA	-0.667	0.0004067926128	OGT
SE	CD46	+	chr1:207767778-207767823	NA	0.246	2.53E-05	SE	ACYP2	+	chr2:54050957-54051050	NA	-0.395	0.0004126182547	OMA1
SE	ZNF267	+	chr16:31890206-31890250	NA	0.165	2.55E-05	SE	RHNO1	+	chr12:2885262-2885534	NA	-0.135	0.0004129172434	OPA1
SE	TEX15	-	chr8:30874936-30875102	NA	0.357	2.61E-05	SE	ZSCAN23	-	chr6:28435858-28436343	NA	-0.305	0.0004182202028	OPTN
SE	IFT88	+	chr13:20663262-20663386	NA	0.116	2.64E-05	SE	TMEM254	+	chr10:80086230-80086258	NA	-0.532	0.0004276980026	ORC2
SE	NADSYN1	+	chr11:71476032-71477438	NA	0.443	2.65E-05	SE	GOSR2	+	chr17:46931101-46931207	NA	-0.261	0.0004282073017	ORC4
SE	CCDC14	-	chr3:123956739-123956795	NA	0.133	2.67E-05	SE	ATP5C1	+	chr10:7806973-7807010	NA	-0.379	0.0004310721539	ORMDL1
SE	TARBP1	-	chr1:234427316-234427388	NA	0.149	2.67E-05	SE	CLEC2D	+	chr12:9693415-9693948	NA	-0.21	0.0004320131833	OSBP2
SE	DGKH	+	chr13:42221161-42221394	NA	0.471	2.68E-05	SE	PLSCR1	-	chr3:146528613-146528831	NA	-0.271	0.0004347064404	OSBPL1A
SE	MAPK8IP3	+	chr16:1758147-1758159	NA	0.225	2.71E-05	SE	ZSWIM7	-	chr17:15986799-15986892	NA	-0.184	0.0004348505466	OSBPL3
SE	CACTIN	-	chr19:3611228-3611364	NA	0.444	2.72E-05	SE	RHNO1	+	chr12:2885161-2885534	NA	-0.157	0.0004617321275	OSBPL6
SE	PAPD7	+	chr5:6743711-6743840	NA	0.104	2.72E-05	SE	TMUB2	+	chr17:44187906-44188009	NA	-0.306	0.0004694339575	OSER1-AS1
SE	RPL13A	+	chr19:49489849-49490090	NA	0.206	2.84E-05	SE	CCNYL1	+	chr2:207733946-207734047	NA	-0.293	0.0004783507939	OXR1
SE	NF1	+	chr17:31252937-31253000	NA	0.338	2.85E-05	SE	RPL17	-	chr18:49491735-49491833	NA	-0.366	0.0004843497337	P4HA1
SE	ARPP19	-	chr15:52564189-52564246	NA	0.288	3.04E-05	SE	PMS2CL	+	chr7:6737075-6737936	NA	-0.111	0.0004879258515	P4HTM
SE	PRKAR1A	+	chr17:68514986-68515119	NA	0.445	3.04E-05	SE	NIPA2	+	chr15:22839654-22839790	NA	-0.147	0.000496439426	PAAF1
SE	UHRF2	+	chr9:6495584-6497103	NA	0.259	3.07E-05	SE	LIG4	-	chr13:108214537-108214610	NA	-0.167	0.0005007711641	PABPC1L
SE	ZNF484	-	chr9:92875014-92875059	NA	0.552	3.08E-05	SE	RP11-33B1.1	+	chr4:119543752-119543845	NA	-0.635	0.0005024837576	PACRGL
SE	ZCWPW1	-	chr7:100407227-100407303	NA	0.552	3.10E-05	SE	PARP12	-	chr7:140027275-140027406	NA	-0.167	0.0005031149632	PACS2
SE	EFCAB13	+	chr17:47347807-47347951	NA	0.634	3.12E-05	SE	RHNO1	+	chr12:2885282-2885492	NA	-0.151	0.0005058911378	PAK4
SE	CNNM3	+	chr2:96831968-96832068	NA	0.242	3.12E-05	SE	ABCD4	-	chr14:74297638-74298069	NA	-0.168	0.0005076552538	PAM
SE	P4HA1	-	chr10:73044980-73045051	NA	0.19	3.13E-05	SE	CCDC138	+	chr2:108788031-108788089	NA	-0.158	0.0005103633731	PAM16
SE	FNBP1	-	chr9:129923843-129923939	NA	0.534	3.18E-05	SE	PHF20L1	+	chr8:132825034-132825157	NA	-0.128	0.0005152712216	PAN2
SE	TREX2	-	chrX:153454876-153455053	NA	0.29	3.19E-05	SE	ZKSCAN3	+	chr6:28363691-28363815	NA	-0.198	0.0005156781645	PANK4

SE	EFNA1	+	chr1:155133502-155133568	NA	0.178	3.25E-05
SE	GNPDA1	-	chr5:142012287-142012377	NA	0.102	3.27E-05
SE	CHORDC1	-	chr11:90206747-90206810	NA	0.12	3.29E-05
SE	CACNB4	-	chr2:151870841-151870861	NA	0.542	3.46E-05
SE	TMEM62	+	chr15:43178606-43178711	NA	0.244	3.47E-05
SE	PPP1R12C	-	chr19:55096261-55096335	NA	0.162	3.49E-05
SE	FAM13B	-	chr5:137946227-137946311	NA	0.339	3.76E-05
SE	MLF1	+	chr3:158588851-158588924	NA	0.16	3.81E-05
SE	TSPAN4	+	chr11:850287-850367	NA	0.239	3.83E-05
SE	RFX3	-	chr9:3301545-3301620	NA	0.269	3.88E-05
SE	CDK5RAP2	-	chr9:120460571-120460667	NA	0.271	3.89E-05
SE	APLP1	+	chr19:35878084-35878108	NA	0.253	4.07E-05
SE	MDM4	+	chr1:204537429-204537497	NA	0.494	4.14E-05
SE	PLEKHA8	+	chr7:30050433-30050474	NA	0.397	4.16E-05
SE	TMEM161A	-	chr19:19134783-19134916	NA	0.602	4.20E-05
SE	TBC1D8B	+	chrX:106839325-106839457	NA	0.271	4.34E-05
SE	MFSD12	-	chr19:3542775-3542977	NA	0.339	4.34E-05
SE	MDM4	+	chr1:204537458-204537497	NA	0.403	4.41E-05
SE	RAD51AP1	+	chr12:4545781-4545832	NA	0.335	4.54E-05
SE	RECQL5	-	chr17:75650516-75650718	NA	0.192	4.71E-05
SE	RHOC	-	chr1:112705099-112705123	NA	0.252	4.77E-05
SE	SNX11	+	chr17:48110707-48110767	NA	0.122	4.93E-05
SE	EEF1D	-	chr8:143590607-143590738	NA	0.346	5.02E-05
SE	SNRPN	+	chr15:24974641-24974957	NA	0.514	5.03E-05
SE	FUBP1	-	chr1:77947452-77947552	NA	0.606	5.11E-05
SE	FAM161A	-	chr2:61836582-61836872	NA	0.345	5.12E-05
SE	PCOLCE	+	chr7:100605675-100605812	NA	0.33	5.14E-05
SE	RMND1	-	chr6:151422540-151422605	NA	0.159	5.32E-05
SE	CENPA	+	chr2:26792755-26792833	NA	0.12	5.47E-05
SE	CTD-2341M24.'	+	chr14:86128664-86128773	NA	0.612	5.70E-05
SE	ATXN2	-	chr12:111464661-111464715	NA	0.397	5.72E-05
SE	RAB3GAP1	+	chr2:135169733-135169848	NA	0.527	5.95E-05
SE	TPCN2	+	chr11:69067502-69067605	NA	0.34	6.03E-05
SE	ALG13	+	chrX:111721602-111721711	NA	0.145	6.15E-05
SE	ZNF326	+	chr1:90007344-90007750	NA	0.107	6.21E-05
SE	METTL26	-	chr16:634888-634956	NA	0.131	6.42E-05
SE	CTNND1	+	chr11:57789036-57789155	NA	0.274	6.52E-05
SE	MAEA	+	chr4:1327626-1327703	NA	0.161	6.59E-05
SE	PQLC3	+	chr2:11174967-11175009	NA	0.307	6.68E-05
SE	ADAMTS6	-	chr5:65470777-65471142	NA	0.295	6.69E-05
SE	March2	+	chr19:8418741-8418807	NA	0.335	6.70E-05
SE	March2	+	chr19:8418536-8418807	NA	0.264	6.70E-05
SE	PTPRD	-	chr9:8437197-8437239	NA	0.283	6.82E-05
SE	SYNJ1	-	chr21:32634860-32634881	NA	0.289	6.83E-05
SE	MTMR2	-	chr11:95887611-95887684	NA	0.172	6.89E-05
SE	MAEA	+	chr4:1322380-1322503	NA	0.166	6.91E-05
SE	ACAA1	-	chr3:38131595-38131638	NA	0.191	6.97E-05
SE	PAQR3	-	chr4:78923856-78923947	NA	0.126	6.97E-05
SE	ZNF83	-	chr19:52616717-52616841	NA	0.225	7.13E-05
SE	PILRB	+	chr7:100353894-100354012	NA	0.284	7.18E-05
SE	SUN1	+	chr7:848417-848615	NA	0.104	7.24E-05
SE	NSD2	+	chr4:1942338-1942432	NA	0.179	7.24E-05
SE	ATP6V0A1	+	chr17:42507519-42507627	NA	0.106	7.40E-05
SE	ETFDH	+	chr4:158680466-158680607	NA	0.132	7.40E-05
SE	CDK5RAP2	-	chr9:120460571-120460747	NA	0.404	7.74E-05
SE	KIF21A	-	chr12:39315931-39315970	NA	0.222	7.86E-05
SE	FAM219B	-	chr15:74904981-74905059	NA	0.208	7.91E-05
SE	COA1	-	chr7:43656032-43656153	NA	0.194	8.02E-05
SE	ULK4	-	chr3:41819422-41819506	NA	0.301	8.15E-05
SE	TOM1L1	+	chr17:54905488-54905567	NA	0.157	8.32E-05
SE	TOP1MT	-	chr8:143332486-143332601	NA	0.232	8.45E-05
SE	AP2B1	+	chr17:35670856-35670898	NA	0.163	8.61E-05
SE	GPR155	-	chr2:174482872-174482918	NA	0.246	8.71E-05
SE	GPBP1L1	-	chr1:45685575-45685620	NA	0.195	8.74E-05
SE	SPAG9	-	chr17:50975862-50975901	NA	0.187	8.78E-05
SE	NLRX1	+	chr11:119172355-119172425	NA	0.323	8.79E-05
SE	SEPT7	+	chr7:35831352-35831496	NA	0.389	8.87E-05
SE	CTAGE5	+	chr14:39320927-39321056	NA	0.384	9.02E-05
SE	SPEF2	+	chr5:35644354-35644525	NA	0.32	9.18E-05
SE	DCAF8	-	chr1:160261284-160261358	NA	0.188	9.23E-05
SE	WARS	-	chr14:100375282-100375350	NA	0.573	9.26E-05
SE	RPL13A	+	chr19:49489849-49489912	NA	0.166	9.38E-05
SE	DCAF8	-	chr1:160261284-160261325	NA	0.329	9.41E-05
SE	RIN3	+	chr14:92615406-92615479	NA	0.363	9.41E-05
SE	NENF	+	chr1:212442545-212442625	NA	0.143	9.57E-05
SE	FNBP4	-	chr11:47725736-47725836	NA	0.165	9.68E-05
SE	ZNF684	+	chr1:40533142-40533181	NA	0.39	9.79E-05
SE	GTPBP10	+	chr7:90355085-90355230	NA	0.181	9.97E-05
SE	AGRN	+	chr1:1051031-1051043	NA	0.332	1.00E-04
SE	TARS2	+	chr1:150504631-150504733	NA	0.14	0.0001016278257
SE	WNK1	+	chr12:880720-880999	NA	0.224	0.0001029798356
SE	CAST	+	chr5:96726793-96726859	NA	0.543	0.0001035888519
SE	BPTF	+	chr17:67886127-67886316	NA	0.15	0.0001037401653
SE	SGCE	-	chr7:94629718-94629841	NA	0.15	0.0001040508253
SE	METTL26	-	chr16:634888-635063	NA	0.117	0.0001053612437
SE	SPATA4	-	chr4:176192957-176193076	NA	0.321	0.00010612589
SE	MYLK	-	chr3:123732895-123733102	NA	0.244	0.0001112824625
SE	OARD1	-	chr6:41070075-41070134	NA	0.133	0.0001114496143
SE	NBPF1	-	chr1:16589898-16590001	NA	0.243	0.0001130815542
SE	FAM208A	-	chr3:56638705-56638765	NA	0.106	0.0001130893075
SE	DGUOK	+	chr2:73950600-73950732	NA	0.168	0.0001134949235
SE	FNIP2	+	chr4:158833800-158833890	NA	0.447	0.0001145993843
SE	HDHD2	-	chr18:47137234-47137338	NA	0.192	0.0001146885166
SE	AGAP4	-	chr10:45834015-45834116	NA	0.8	0.0001149006239
SE	NPHP3	-	chr3:132717954-132718117	NA	0.3	0.0001161877562
SE	TEPSIN	-	chr17:81232314-81232518	NA	0.331	0.0001166155859
SE	VEGFA	+	chr6:43781955-43782052	NA	0.179	0.000117787783
SE	CACNB4	-	chr2:151869176-151869735	NA	0.455	0.0001178626348
SE	PDK1	+	chr2:172568740-172568817	NA	0.257	0.0001189696158
SE	TOM1L1	+	chr17:54903707-54903792	NA	0.141	0.0001213482772
SE	POLB	+	chr8:42352518-42352568	NA	0.146	0.0001215527779
SE	LRRC23	+	chr12:6909889-6910026	NA	0.254	0.0001253084563
SE	CSNK1G3	+	chr5:123595038-123595134	NA	0.14	0.0001322429857
SE	SH3D19	-	chr4:151144219-151144288	NA	0.199	0.0001322429857
SE	XRRA1	-	chr11:74945017-74945085	NA	0.438	0.0001331087106
SE	TMEM230	-	chr20:5111499-5111605	NA	0.177	0.0001350148226
SE	ATP9B	+	chr18:79375393-79375426	NA	0.2	0.0001362768567
SE	BRD8	-	chr5:138159554-138159599	NA	0.262	0.0001421619531
SE	MVD	-	chr16:88657145-88657247	NA	0.523	0.0001426700148
SE	NSD2	+	chr4:1942338-1942565	NA	0.246	0.0001438727222
SE	C14orf166	+	chr14:52004239-52004242	NA	0.55	0.0001448755793
SE	ERMARD	+	chr6:169753037-169753181	NA	0.329	0.0001454345137
SE	NAB1	+	chr2:190655976-190656153	NA	0.328	0.0001454345137
SE	UBE3A	-	chr15:25407066-25407137	NA	0.213	0.0001454345137
SE	MFF	+	chr2:227342744-227342819	NA	0.192	0.000146113945
SE	UBXN2B	+	chr8:58413298-58413371	NA	0.307	0.0001467205675
SE	GPR155	-	chr2:174482872-174482918	NA	0.231	0.0001494592795
SE	KLHL24	+	chr3:183636220-183636311	NA	0.322	0.0001503356103
SE	PPP1R3E	-	chr14:23300106-23300325	NA	0.399	0.0001503356103
SE	NENF	+	chr1:212442564-212442625	NA	0.115	0.0001526398926
SE	KIAA1191	-	chr5:176359480-176359567	NA	0.544	0.0001530389937

SE	PSMA3-AS1	-	chr14:58285590-58285756	NA	-0.289	0.0005255286629
SE	DCUN1D4	+	chr4:51908919-51908975	NA	-0.168	0.0005258881182
SE	ASCC2	-	chr22:29825621-29825780	NA	-0.158	0.0005291269653
SE	AKAP11	+	chr13:42285985-42286035	NA	-0.284	0.0005294509158
SE	FBXO38	+	chr5:148427212-148427947	NA	-0.176	0.000531989971
SE	CDC14B	-	chr9:96551795-96551872	NA	-0.195	0.0005340109262
SE	KAT6A	-	chr8:42049619-42049707	NA	-0.254	0.0005347540089
SE	SUCO	+	chr1:172577538-172577559	NA	-0.276	0.0005405865695
SE	RALY	+	chr20:34065043-34065160	NA	-0.352	0.0005418065682
SE	GABBR1	-	chr6:29608600-29608733	NA	-0.105	0.0005418065682
SE	THAP5	-	chr7:108565829-108566022	NA	-0.157	0.0005452233171
SE	SLC35D2	-	chr9:96336716-96336784	NA	-0.163	0.0005475375873
SE	MRNIP	-	chr5:179853196-179853276	NA	-0.182	0.0005585912991
SE	HNRNPUL1	+	chr19:41305831-41305867	NA	-0.111	0.000558909809
SE	OSBPL6	+	chr2:178339671-178339764	NA	-0.671	0.0005646569594
SE	SEPT6	-	chrX:119625334-119625396	NA	-0.208	0.0005682274055
SE	ZNF655	+	chr7:99563187-99563266	NA	-0.171	0.0005704224736
SE	BTN2A2	+	chr6:26385014-26385362	NA	-0.176	0.0005716012452
SE	FAM228B	+	chr2:24137908-24138100	NA	-0.463	0.0005866349904
SE	LRRC37A4P	-	chr17:45542763-45542835	NA	-0.448	0.0006039756834
SE	SLCO4A1	+	chr20:62668154-62668184	NA	-0.556	0.0006103010624
SE	MED12L	+	chr3:151409242-151409332	NA	-0.451	0.0006243958793
SE	TBL1X	+	chrX:9534899-9535034	NA	-0.128	0.0006349605137
SE	ATPAF1	-	chr1:46643193-46643301	NA	-0.18	0.0006417798024
SE	DHRS4L2	+	chr14:24001383-24001517	NA	-0.461	0.0006418050778
SE	TRIM9	-	chr14:51000682-51000828	NA	-0.253	0.0006452214125
SE	C11orf74	+	chr11:36636050-36636117	NA	-0.164	0.000648428039
SE	SETX	-	chr9:132269402-132269489	NA	-0.176	0.0006607809519
SE	SLC50A1	+	chr1:155137578-155137722	NA	-0.148	0.0006659499029
SE	ARMCX5-GPRAS	+	chrX:102601463-102601509	NA	-0.358	0.0006660333427
SE	FAM227B	-	chr15:49577171-49577305	NA	-0.42	0.0006714121578
SE	KANSL2	-	chr12:48679035-48679150	NA	-0.132	0.0006849077524
SE	PRPSAP2	+	chr17:18857783-18857853	NA	-0.205	0.0006885416923
SE	FRG1HP	+	chr9:41008880-41009006	NA	-0.132	0.0006889078724
SE	SAMD4A	+	chr14:54737023-54737287	NA	-0.201	0.000707373342
SE	ST5	-	chr11:8729949-8731209	NA	-0.325	0.0007143248076
SE	ZGRF1	-	chr4:112603523-112603697	NA	-0.291	0.0007152943645
SE	CXorf23	-	chrX:19935808-19935898	NA	-0.182	0.0007224857652
SE	C3orf18	-	chr3:50566005-50566094	NA	-0.523	0.0007237793159
SE	MFSD10	-	chr4:2931554-2931663	NA	-0.108	0.0007475297347
SE	EP400NL	+	chr12:132109767-132109832	NA	-0.301	0.0007481903903
SE	DMXL2	-	chr15:51453549-51453641	NA	-0.143	0.0007499683931
SE	SMUG1	-	chr12:54186846-54187011	NA	-0.312	0.0007555329084
SE	CD27-AS1	-	chr12:6443545-6443717	NA	-0.321	0.0007580514648
SE	BLOC1S6	+	chr15:45592134-45592276	NA	-0.123	0.0007611946758
SE	ATPAF1	-	chr1:46645160-46645256	NA	-0.171	0.0007654461304
SE	CDKL3	-	chr5:134306608-134306702	NA	-0.255	0.0007711754357
SE	SHF	-	chr15:45171882-45172078	NA	-0.508	0.0007745711686
SE	THTPA	+	chr14:23556742-23557304	NA	-0.189	0.0007750139882
SE	CCNI	-	chr4:77056248-77056323	NA	-0.374	0.0007768817232
SE	PPP4R1	-	chr18:9562921-9563046	NA	-0.304	0.0007881069043
SE	RP11-156P1.3	-	chr17:47072471-47072559	NA	-0.494	0.0007881069043
SE	RAD1	-	chr5:34913469-34913578	NA	-0.262	0.0008025627237
SE	NUP50	+	chr22:45170987-45171143	NA	-0.125	0.000806993149
SE	ATP6V1B1	+	chr2:70963161-70963261	NA	-0.253	0.0008122141714
SE	MFF	+	chr2:227347225-227347384	NA	-0.189	0.0008205256852
SE	RP11-371E8.4	+	chr14:93188892-93188997	NA	-0.557	0.0008231927538
SE	NUDT13	+	chr10:73125397-73125509	NA	-0.517	0.0008479198092
SE	TMPO	+	chr12:98544950-98545061	NA	-0.24	0.0008503980827
SE	ASAP1	-	chr8:130091972-130092143	NA	-0.139	0.0008506612306
SE	LZTFL1	-	chr3:45837926-45838047	NA	-0.249	0.0008526729915
SE	DTNB	-	chr2:25383835-25383889	NA	-0.491	0.0008538554437
SE	ABCA11P	-	chr4:437445-437511	NA	-0.39	0.0008550215213
SE	LINC00174	-	chr7:66400321-66400437	NA	-0.314	0.0008590836179
SE	CCDC57	-	chr17:82107401-82107621	NA	-0.242	0.0008632009723
SE	ZMIZ1	+	chr10:79162052-79162133	NA	-0.14	0.000864542017
SE	TCTN1	+	chr12:110644966-110645129	NA	-0.225	0.0008678622588
SE	SPRY1	+	chr4:123397610-123397856	NA	-0.155	0.0008703111406
SE	TIMM8B	-	chr11:112085966-112086310	NA	-0.109	0.0008728679574
SE	MCFD2	-	chr2:46909022-46909177	NA	-0.11	0.000879782463
SE	WEE2-AS1	-	chr7:141714188-141714374	NA	-0.275	0.0008913910942
SE	RPAIN	+	chr17:5426235-5426299	NA	-0.117	0.0008985289339
SE	PCBP1-AS1	-	chr2:70085815-70085903	NA	-0.291	0.0008998411314
SE	RP11-274B21.14	+	chr7:128578889-128578933	NA	-0.501	0.0009029980055
SE	ZNF592	+	chr15:84778182-84778312	NA	-0.241	0.000908503465
SE	NFIB	-	chr9:14116207-14116346	NA	-0.389	0.0009191385584
SE	INTS4	-	chr11:77910969-77911152	NA	-0.154	0.0009230389767
SE	TTC13	-	chr1:230945388-230945454	NA	-0.256	0.0009232480333
SE	MLLT10	+	chr10:21534644-21534804	NA	-0.187	0.0009232480333
SE	MIOS	+	chr7:7568025-7568123	NA	-0.159	0.0009319807982
SE	C8orf44	+	chr8:66676744-66676902	NA	-0.455	0.0009339975889
SE	SYNE2	+	chr14:64098746-64098821	NA	-0.232	0.0009384681377
SE	THAP5	-	chr7:108565951-108566022	NA	-0.278	0.0009421571947
SE	ZSCAN23	-	chr6:28435459-28435607	NA	-0.301	0.0009603740643
SE	ZNF254	+	chr19:24106547-24106643	NA	-0.187	0.0009638969816
SE	APOM	+	chr6:31656471-31656626	NA	-0.162	0.0009638969816
SE	STAG3L4	+	chr7:67308576-67308652	NA	-0.331	0.0009659206584
SE	ALMS1	+	chr2:73550266-73550437	NA	-0.164	0.0009874780662
SE	CLEC2D	+	chr12:9692827-9693948	NA	-0.153	0.0009874780662
SE	DNAAF2	-	chr14:49628011-49628155	NA	-0.201	0.000992479765
SE	POLL	-	chr10:101585315-101585478	NA	-0.325	0.0009943830497
SE	DNAJC14	-	chr12:55829488-55829636	NA	-0.185	0.0009943830497
SE	SHF	-	chr15:45171882-45172002	NA	-0.471	0.001004510926
SE	GAPVD1	+	chr9:125335171-125335252	NA	-0.178	0.001005069542
SE	TMPO	+	chr12:98544950-98545061	NA	-0.13	0.001012684496
SE	THTPA	+	chr14:23556742-23557039	NA	-0.24	0.001013018677
SE	RP11-421L21.3	+	chr1:101083460-101083574	NA	-0.185	0.001013602768
SE	ZNF814	-	chr19:57885882-57885969	NA	-0.212	0.001014446317
SE	AGPAT4	-	chr6:161165576-161165713	NA	-0.441	0.001023745599
SE	PAM	+	chr5:103025133-103025334	NA	-0.125	0.001027895403
SE	PATZ1	-	chr22:31328786-31328859	NA	-0.158	0.001028859064
SE	NISCH	+	chr3:52480878-52481109	NA	-0.395	0.001052296447
SE	ABCD4	-	chr14:74299547-74299675	NA	-0.145	0.001061603759
SE	LYPLAL1	+	chr1:219193081-219193251	NA	-0.156	0.001062493224
SE	TANC2	+	chr17:63418306-63418407	NA	-0.138	0.00106274245
SE	THTPA	+	chr14:23556742-23557034	NA	-0.239	0.001064705924
SE	GLS	+	chr2:190913192-190913364	NA	-0.115	0.001084209801
SE	CLEC2D	+	chr12:9692827-9692931	NA	-0.167	0.001097891832
SE	ZNF789	+	chr7:99478273-99478356	NA	-0.412	0.001102013063
SE	RPRD2	+	chr1:150464526-150464727	NA	-0.105	0.001107474483
SE	IST1	+	chr16:71924090-71924281	NA	-0.123	0.001113102966
SE	SLC25A29	-	chr14:100298026-100298190	NA	-0.417	0.001127335634
SE	ZNF76	+	chr6:35293750-35293915	NA	-0.302	0.001129571315
SE	PVT1	+	chr8:127983903-127984204	NA	-0.228	0.001136224867
SE	SLC47A1	+	chr17:19571477-19571572	NA	-0.191	0.001141438719
SE	ZNF37BP	-	chr10:42525539-42525660	NA	-0.132	0.001151705187
SE	BTN2A1	+	chr6:26458606-26458718	NA	-0.142	0.001159125414
SE	IFT80	-	chr3:160366092-160366152	NA	-0.242	0.001172941124
SE	LYPLAL1	+	chr1:219193129-219193251	NA	-0.168	0.001177240212
SE	LYRM1	+	chr16:20902485-20902717	NA	-0.246	0.001177240212
SE	KIAA1217	+	chr10:24542882-24544481	NA	-0.141	0.001183334333

PAPD4
PAPD7
PAQR3
PAQR6
PARD3
PARP12
PARP14
PARP2
PARP6
PARP8
PARPBP
PATJ
PATZ1
PBRM1
PBX4
PCBP1-AS1
PCBP4
PCDHGC3
PCGF3
PCGF6
PCID2
PCMTD1
PCNT
PCNX1
PCNX2
PCOLCE
PCSK7
PCTP
PCYT2
PDCD10
PDCD2L
PDE9A
PDGFRA
PDK1
PDLIM5
PDPR
PDZD7
PER3
PET100
PEX1
PEX2
PEX3
PEX5
PEX7
PFAS
PFDN5
PFKL
PFKM
PFN2
PGAP1
PGAP2
PGAP3
PGGHG
PGPEP1
PHACTR4
PHB2
PHC1
PHF14
PHF20L1
PHF3
PHF5A
PHF7
PHF8
PHKA1
PHKB
PHTF2
PHYKPL
PI4KAP1
PI4KAP2
PIAS3
PIBF1
PICALM
PIDD1
PIF1
PIGA
PIGF
PIGG
PIGN
PIGP
PIGQ
PIGT
PILRB
PIN4
PINK1
PIP5K1C
PKD1
PKD1P6-NPIPP1
PKIG
PKMYT1
PKP4
PLA2G12A
PLA2G6
PLAT
PLCB4
PLCD4
PLD3
PLEKHA1
PLEKHA4
PLEKHA8
PLEKHH1
PLEKHH2
PLEKHH3
PLEKHM1
PLEKHM1P1
PLGRKT
PLK3
PLOD2
PLPP5
PLSCR1
PLXNB1
PMF1-BGLAP
PMS2
PMS2CL
PMS2P1
PNISR
PNKP
PNPLA3
PNPLA6
POC1A
POC1B
POC5

SE	TEAD2	-	chr19:49355958-49355970	NA	0.145	0.0001549461537	SE	CREB1	+	chr2:207577109-207577224	NA	-0.381	0.001185968339	POGLUT1
SE	EIF3E	-	chr8:108242111-108242346	NA	0.195	0.0001580415189	SE	INVS	+	chr9:100131869-100131954	NA	-0.479	0.00118875574	POLA1
SE	PNISR	-	chr6:99416348-99416428	NA	0.196	0.0001655884713	SE	TMEM222	+	chr1:27330984-27331137	NA	-0.165	0.001213990935	POLB
SE	C5orf42	-	chr5:37139339-37139370	NA	0.206	0.0001656045859	SE	SBF2	-	chr11:9828125-9828221	NA	-0.181	0.001230254427	POLD1
SE	SYTL2	-	chr11:85717482-85717530	NA	0.596	0.0001661427625	SE	DYNLL1	+	chr12:120495283-120495437	NA	-0.512	0.001232062067	POLDIP3
SE	SP140L	+	chr2:230328756-230328831	NA	0.363	0.0001674844062	SE	C11orf74	+	chr11:36633283-36633438	NA	-0.18	0.001232514114	POLE2
SE	MDC1	-	chr6:30707565-30708357	NA	0.192	0.0001681652016	SE	OARD1	-	chr6:41068840-41068953	NA	-0.107	0.001236636279	POLG
SE	C19orf12	-	chr19:29708253-29708423	NA	0.347	0.0001709470325	SE	ZNF821	-	chr16:71861775-71861942	NA	-0.292	0.001272523681	POLL
SE	ARPP19	-	chr15:52568847-52568902	NA	0.12	0.0001747922282	SE	MROH8	-	chr20:37155062-37155167	NA	-0.609	0.001277328111	POLM
SE	HLA-L	+	chr6:30262294-30262342	NA	0.674	0.0001824986839	SE	ZNF692	-	chr1:248856372-248856422	NA	-0.103	0.001282264471	POLQ
SE	JMJD1C	-	chr10:63215552-63215617	NA	0.149	0.0001826002476	SE	SNAPC5	-	chr15:66494902-66495419	NA	-0.399	0.001312004339	POLR1B
SE	MAP3K6	-	chr1:27364660-27364684	NA	0.66	0.0001833146153	SE	RABGAP1	+	chr9:122945331-122945432	NA	-0.112	0.001312112384	POLR1C
SE	VPS53	-	chr17:661808-661895	NA	0.212	0.0001833798291	SE	ATP6V1B1	+	chr2:70963161-70963312	NA	-0.147	0.001312492684	POLR2J4
SE	VKORC1	-	chr16:31093311-31093482	NA	0.134	0.0001852608114	SE	SREBF1	-	chr17:17817547-17817591	NA	-0.375	0.0013265261	POMGNT1
SE	ALKBH8	-	chr11:107549752-107549823	NA	0.271	0.0001857822036	SE	PEX1	-	chr7:92509328-92509411	NA	-0.17	0.001359664745	POMT1
SE	ZC2HC1A	+	chr8:78711989-78712106	NA	0.185	0.0001857822036	SE	RPE	+	chr2:210009638-210009736	NA	-0.277	0.001384659396	POMZP3
SE	ABI1	-	chr10:26755654-26755741	NA	0.129	0.0001864371792	SE	RIDA	-	chr8:98108645-98108713	NA	-0.106	0.00140484798	POP4
SE	PRPF4B	+	chr6:4062556-4062760	NA	0.163	0.0001935444919	SE	BDNF-AS	+	chr11:27675576-27676140	NA	-0.343	0.001406429329	POP5
SE	MAP9	-	chr4:155375775-155375914	NA	0.149	0.0001944976715	SE	POLR2J4	-	chr7:43986600-43986677	NA	-0.327	0.001407114613	PORCN
SE	TBC1D23	+	chr3:100311832-100311877	NA	0.132	0.0001948670864	SE	XIAP	+	chrX:123860166-123860293	NA	-0.377	0.001426975572	POT1
SE	GNPDA1	-	chr5:142012287-142012339	NA	0.127	0.0001952559675	SE	B3GALNT1	-	chr3:161089943-161090062	NA	-0.188	0.001439021672	PPA2
SE	MTIF3	-	chr13:27441016-27441077	NA	0.327	0.0001959215647	SE	HERC2P9	+	chr15:28655494-28655691	NA	-0.447	0.001439456619	PPCDC
SE	MYO9A	-	chr15:71902940-71903063	NA	0.191	0.0001969559049	SE	UBA2	+	chr19:34431860-34431931	NA	-0.474	0.001456868059	PPFIBP1
SE	SLC7A11	-	chr4:138223198-138223324	NA	0.163	0.0001997450176	SE	UBE2F	+	chr2:237987962-237987992	NA	-0.108	0.001458988257	PPHLN1
SE	ARNT	-	chr1:150842423-150842468	NA	0.123	0.0001999884726	SE	NME6	-	chr3:48296118-48296236	NA	-0.388	0.001460213182	PPIL2
SE	UFD1L	-	chr22:19475495-19475602	NA	0.185	0.0002027578632	SE	ENSA	-	chr1:150626478-150626526	NA	-0.374	0.001478634489	PPIL3
SE	ENTPD6	+	chr20:25221231-25221333	NA	0.148	0.0002037373271	SE	LINC00969	+	chr3:195696360-195696589	NA	-0.207	0.001482125936	PPIP5K2
SE	UFD1L	-	chr22:19475469-19475602	NA	0.155	0.0002051225455	SE	SUOX	+	chr12:55999332-55999590	NA	-0.449	0.001493573318	PPM1A
SE	PPIP5K2	+	chr5:103186319-103186439	NA	0.322	0.0002061758732	SE	DNMT3A	-	chr2:25239314-25239513	NA	-0.13	0.001496409421	PPM1K
SE	SMARCAL1	+	chr2:216438419-216438485	NA	0.161	0.0002091071405	SE	CBLB	-	chr3:105681478-105681610	NA	-0.127	0.001497862413	PPOX
SE	FEZ2	-	chr2:36560784-36560865	NA	0.201	0.0002096174479	SE	POLL	-	chr10:101582762-101582891	NA	-0.123	0.001498880467	PPP1R12B
SE	MRPL55	-	chr1:228108954-228109021	NA	0.223	0.0002096174479	SE	CAMK1	-	chr3:9765758-9765890	NA	-0.122	0.001499769913	PPP1R12C
SE	HPS1	-	chr10:98435271-98435414	NA	0.245	0.0002122238175	SE	FAM86EP	-	chr4:3948089-3948220	NA	-0.499	0.001505065008	PPP1R3E
SE	BORA	+	chr13:72734959-72735005	NA	0.361	0.0002217088096	SE	ITGB1BP1	-	chr2:9419989-9420100	NA	-0.267	0.001505065008	PPP2R2D
SE	EVI5	-	chr1:92663419-92663452	NA	0.515	0.0002266272164	SE	ZNF778	+	chr16:89222091-89222183	NA	-0.224	0.001520288195	PPP2R3C
SE	NBEAL1	+	chr2:203115984-203116070	NA	0.355	0.0002383144115	SE	GK	+	chrX:30727465-30727552	NA	-0.313	0.001523891106	PPP2R5C
SE	GLS	+	chr2:190920214-190920248	NA	0.395	0.0002389327734	SE	CFAP44	-	chr3:113344515-113344712	NA	-0.248	0.001534015222	PPP3CB
SE	PIGQ	+	chr16:581198-581341	NA	0.524	0.0002419316532	SE	PAM	+	chr5:103025130-103025334	NA	-0.106	0.001547919879	PPP4C
SE	PAK4	+	chr19:39168229-39168312	NA	0.166	0.0002419316532	SE	C11orf65	-	chr11:108431745-108431838	NA	-0.536	0.001578796476	PPP4R1
SE	UPRT	+	chrX:75294562-75294620	NA	0.227	0.0002487257324	SE	PAM	+	chr5:102974115-102974436	NA	-0.124	0.001610289966	PPP4R1L
SE	DNAJB6	+	chr7:157409794-157415529	NA	0.461	0.0002552904057	SE	ING4	-	chr12:6655637-6655913	NA	-0.405	0.001631986303	PPP5C
SE	TBC1D8B	+	chrX:106839307-106839457	NA	0.193	0.0002577584141	SE	ZNF260	-	chr19:36525154-36525373	NA	-0.156	0.001648135725	PPP6R2
SE	IFNGR2	+	chr21:33410840-33410892	NA	0.178	0.000258114977	SE	ZNF655	+	chr7:99561887-99561973	NA	-0.273	0.001673590734	PPRC1
SE	LRRC23	+	chr12:6907314-6907445	NA	0.123	0.0002615336253	SE	MAPT	+	chr17:45971858-45971945	NA	-0.225	0.001677676425	PPT2-EGFL8
SE	PCOLCE	+	chr7:100605504-100605812	NA	0.334	0.000261928969	SE	KANK2	-	chr19:11196049-11196227	NA	-0.329	0.00167889463	PPWD1
SE	SMG7	+	chr1:183547102-183547252	NA	0.216	0.0002682286217	SE	FUZ	-	chr19:49812446-49812685	NA	-0.212	0.001680905192	PQBP1
SE	DSN1	-	chr20:36771424-36771473	NA	0.253	0.0002691945079	SE	TNIK	-	chr3:171128713-171128878	NA	-0.129	0.00168795208	PQLC1
SE	NME4	+	chr16:406684-406777	NA	0.59	0.0002851559216	SE	CWF19L1	-	chr10:100250247-100250332	NA	-0.101	0.00170288151	PQLC3
SE	PHACTR4	+	chr1:28457831-28457859	NA	0.402	0.0002880114652	SE	FBXO38	+	chr5:148427212-148427722	NA	-0.173	0.00170797872	PRC1
SE	IFT88	+	chr13:20615792-20615879	NA	0.231	0.0002900265419	SE	TPRKB	-	chr2:73734527-73734591	NA	-0.127	0.001712917184	PRDM2
SE	PAK4	+	chr19:39168229-39168302	NA	0.16	0.0002900265419	SE	PGAP2	+	chr11:3823612-3823740	NA	-0.366	0.001732186964	PRDM5
SE	SUZ12P1	+	chr17:30743300-30743369	NA	0.278	0.0002914239142	SE	ZC3H18	+	chr16:88611267-88611536	NA	-0.365	0.001733304234	PREPL
SE	AMPD2	+	chr1:109622184-109622353	NA	0.554	0.0002935232439	SE	AREL1	-	chr14:74701668-74701774	NA	-0.502	0.001735749298	PRIMPOL
SE	CITED1	-	chrX:72305301-72305422	NA	0.169	0.00029790281	SE	DTNB	-	chr2:25455404-25455494	NA	-0.289	0.001751966261	PRKAB1
SE	ARNTL2	+	chr12:27370128-27370230	NA	0.173	0.0002979307257	SE	BDNF-AS	+	chr11:27675296-27676140	NA	-0.368	0.00177508727	PRKAG1
SE	RAB34	-	chr17:28716902-28716994	NA	0.215	0.0002981241961	SE	FUZ	-	chr19:49812446-49812736	NA	-0.142	0.001793405002	PRKAR1A
SE	RPUSD4	-	chr11:126206253-126206553	NA	0.101	0.0003073383043	SE	CLEC16A	+	chr16:11174153-11174277	NA	-0.601	0.001796423469	PRKCSH
SE	CBWD5	+	chr9:65732946-65733122	NA	0.237	0.000307827365	SE	MRPS6	+	chr21:34103496-34103519	NA	-0.428	0.001796423469	PRKD1
SE	RFX2	-	chr19:6026162-6026237	NA	0.324	0.0003095361672	SE	ZNF506	-	chr19:19793833-19795660	NA	-0.147	0.001824574982	PRKDC
SE	TYSND1	-	chr10:70143841-70143972	NA	0.289	0.000311701948	SE	TRMO	-	chr9:97916163-97916338	NA	-0.189	0.001827589487	PRKRIP1
SE	R3HDM2	-	chr12:57297327-57297387	NA	0.127	0.0003145254367	SE	ESYT2	-	chr7:158752780-158752843	NA	-0.346	0.001829980388	PRMT2
SE	R3HDM2	-	chr12:57305639-57305663	NA	0.105	0.0003186670567	SE	TIMM8B	-	chr11:112085977-112086310	NA	-0.121	0.001838520626	PROCA1
SE	RANBP10	-	chr16:67772033-67772086	NA	0.208	0.0003211554556	SE	DPY19L2	-	chr12:63569223-63569349	NA	-0.127	0.001838520626	PROSER3
SE	C11orf1	+	chr11:111883135-111883202	NA	0.166	0.0003246175549	SE	LCA5L	-	chr21:39427758-39427905	NA	-0.462	0.001839490346	PRPF31
SE	TOP3B	-	chr22:21975904-21976187	NA	0.134	0.0003277096277	SE	PLGRKT	-	chr9:5381862-5382031	NA	-0.183	0.001850489502	PRPF38A
SE	DPM1	-	chr20:50941104-50941209	NA	0.474	0.0003324026152	SE	TOPORS-AS1	+	chr9:32565396-32565628	NA	-0.351	0.001855130247	PRPF40A
SE	JMJD1C	-	chr10:63215552-63215696	NA	0.103	0.0003440367549	SE	NMT2	-	chr10:15130141-15130312	NA	-0.104	0.001857369881	PRPF40B
SE	WASH2P	+	chr2:113595062-113595161	NA	0.555	0.000350375615	SE	CLHC1	-	chr2:55180509-55180712	NA	-0.136	0.001870402945	PRPF4B
SE	ZNF384	-	chr12:6678662-6678710	NA	0.196	0.0003506228985	SE	SEPT6	-	chrX:119625334-119625379	NA	-0.408	0.001889786218	PRPSAP2
SE	RTN4	-	chr2:55025085-55027485	NA	0.231	0.0003550262261	SE	SNX14	-	chr6:85565331-85565419	NA	-0.184	0.001898049618	PRR13
SE	ZMYND8	-	chr20:47212641-47212725	NA	0.25	0.0003587716172	SE	YAP1	+	chr11:102209516-102209564	NA	-0.148	0.001899552138	PRR3
SE	FAM219B	-	chr15:74904981-74905039	NA	0.191	0.0003588759518	SE	TMBIM6	+	chr12:49749617-49749856	NA	-0.271	0.001910715501	PRR34-AS1
SE	TEAD2	-	chr19:49359843-49360081	NA	0.101	0.0003628054664	SE	CBY1	+	chr22:38665545-38665685	NA	-0.324	0.001921405105	PRR4
SE	TANC2	+	chr17:63151269-63151380	NA	0.203	0.0003699467386	SE	CNTRL	+	chr9:121171407-121171548	NA	-0.173	0.001925312558	PRUNE1
SE	ZNF195	-	chr11:3373576-3373656	NA	0.149	0.0003772197851	SE	WIPI1	-	chr17:68434555-68434626	NA	-0.101	0.00192549168	PSEN1
SE	ASAH2B	+	chr10:50745127-50745274	NA	0.359	0.000381490862	SE	PPP1R12B	+	chr1:202445045-202445228	NA	-0.302	0.001936037329	PSMA3-AS1
SE	AGTRAP	+	chr1:11746126-11746223	NA	0.184	0.0003826530531	SE	UBE2F	+	chr2:237994743-237994809	NA	-0.194	0.001936623701	PSMA4
SE	SERPINB6	-	chr6:2968778-2968913	NA	0.414	0.0003879148638	SE	POLL	-	chr10:101584877-101584919	NA	-0.548	0.001950407967	PSMD6
SE	CBWD5	+	chr9:65732992-65733122	NA	0.214	0.0003888102782	SE	RP11-1228E12.1	-	chr17:71365-71556	NA	-0.667	0.001952422279	PSME1
SE	SLC25A22	-	chr11:796042-796367	NA	0.298	0.0003906458635	SE	XRRA1	-	chr11:74909888-74910012	NA	-0.121	0.00199678348	PSMG1
SE	CAMTA2	-	chr17:4982088-4982160	NA	0.239	0.0003906458635	SE	APITD1-CORT	+	chr1:10440346-10440413	NA	-0.673	0.001998768924	PSMG3-AS1
SE	OSBPL3	-	chr7:24863199-24863292	NA	0.18	0.0004067926128	SE	ZNF253	+	chr19:19878480-19878607	NA	-0.297	0.00201485337	PSMG4
SE	RNMT	+	chr18:13759941-13760208	NA	0.162	0.0004093805217	SE	RPE	+	chr2:210009656-210009736	NA	-0.116	0.002023216657	PSTK
SE	CDCA7	+	chr2:173359342-173359491	NA	0.106	0.0004100811703	SE	IKZF4	+	chr12:56019317-56019415	NA	-0.422	0.002024138061	PTAR1
SE	METTL26	-	chr16:635517-635774	NA	0.353	0.0004141608316	SE	DDIAS	+	chr11:82913286-82913386	NA	-0.122	0.002044719189	PTBP2
SE	MLLT10	+	chr10:21557620-21557824	NA	0.143	0.00041663617	SE	NFIB	-	chr9:14116180-14116346	NA	-0.374	0.002058724474	PTGES2
SE	ABCD4	-	chr14:74295147-74295198	NA	0.467	0.0004186868464	SE	TPM1	+	chr15:63064063-63064142	NA	-0.35	0.002058724474	PTK2
SE	CLK4	-	chr5:178619837-178619896	NA	0.17	0.0004320131833	SE	FAM227B	-	chr15:49589775-49590007	NA	-0.191	0.002064161024	PTPMT1
SE	STAG3L3	-	chr7:73000357-73000481	NA	0.569	0.0004383795297	SE	AFTPH	+	chr2:64579485-64579546	NA	-0.213	0.002066660603	PTPN4
SE	ZMYM1	+	chr1:35093393-35093441	NA	0.292	0.0004387441602	SE	ZNF211	+	chr19:57633785-57633905	NA	-0.403	0.002075918316	PTPRA
SE	METTL26	-	chr16:635611-635774	NA	0.303	0.0004387441602	SE	NTAN1	-	chr16:15047996-15048099	NA	-0.137	0.002085721271	PTPRD
SE	SEMA4F	+	chr2:74662731-74662825	NA	0.419	0.0004418183214	SE	CTDP1	+	chr18:79736354-79736521	NA	-0.177	0.002088014931	PTPRS
SE	TBC1D31	+	chr8:123082701-123082817	NA	0.101	0.0004520812027	SE	RANGAP1	-	chr22:41284900-41285153	NA	-0.167	0.002089000179	PTTG1IP
SE	NOD1	-	chr7:30463608-30463698	NA	0.431	0.0004537657274	SE	PBRM1	-	chr3:52554723-52554879	NA	-0.487	0.002131254622	PUDP
SE	RNF10	+	chr12:120539367-120539468	NA	0.101	0.0004537657274	SE	FBXL2	+	chr3:33297868-33297962	NA	-0.342	0.002142500663	PUF60
SE	CACNB4	-	chr2:151869176-151869735	NA	0.339	0.0004627385666	SE	ANK3	-	chr10:60200128-60200263	NA	-0.135	0.002225080387	PUM2
SE	UBE3A	-	chr15:25407066-25407212	NA	0.154	0.0004742376075	SE	TRIM16	-	chr17:15677175-15677280	NA	-0.495	0.002239049543	PVR
SE	LRRC75B	-	chr22:24588678-24589265	NA	0.438	0.0004754203316	SE	COPG1	+	chr3:129250681-129250894	NA	-0.143	0.002239049543	PVT1
SE	MARK3	+	chr14:103498501-103498528	NA	0.529	0.0004916542622	SE	NFYC	+	chr1:40766917-40766974	NA	-0.117	0.002262356732	PXMP2
SE	MTMR4	-	chr17:58514991-58515083	NA	0.257	0.0005045853247	SE	OPA1	+	chr3:193617783-193617837	NA	-0.396	0.002268274405	PXN
SE	YLPM1	+	chr14:74824255-74824307	NA	0.119	0.0005060879067	SE	HM13	+	chr20:31568077-31568280	NA	-0.212	0.002292667923	PYM1
SE	NUDT13	+	chr10:73124213-73124320	NA	0.313	0.0005127634016	SE	DAG1	+	chr3:49487253-49487415	NA	-0.199	0.002308161594	QKI
SE	CENPA	+	chr2:26792501-26792833	NA	0.254	0.0005141992172	SE	C14orf93	-	chr14:23000958-23001037	NA	-0.576	0.002308460508	QRICH1
SE	GAS5	-	chr1:173864674-173864704	NA	0.223	0.0005287084667	SE	ZNF655	+	chr7:99561887-99561973	NA	-0.307	0.002332507155	QTRT1
SE	OARD1	-	chr6:41068840-41068953	NA	0.132	0.0005294281457	SE	ZNF814	-	chr19:57868457-57868579	NA	-0.647	0.002347592929	R3HCC1L
SE	SHMT1	-	chr17:18335558-18335675	NA	0.197	0.0005294509158	SE	SCMH1	-	chr1:41151613-41151684	NA	-0.202	0.002354333127	R3HDM1
SE	IFNGR2	+	chr21:33410835-33410892	NA	0.174	0.0005294509158	SE	IFFO2	-	chr1:18918361-18918502	NA	-0.158	0.002376499677	R3HDM2
SE	SCLY	+	chr2:238083247-238083354	NA	0.155	0.0005316991493	SE	ZNF571	-	chr19:37586667-37586745	NA	-0.57	0.002377389601	R3HDM4
SE	SPG11	-	chr15:44606024-44606091	NA	0.11	0.0005365370169	SE	RP11-33B1.1	+	chr4:119488644-119488717	NA	-0.108	0.002384550275	RAB15
SE	TNC	-	chr9:115048259-115048532	NA	0.601	0.0005415202019	SE	TRIM16	-	chr17:15682853-15682964	NA	-0.196	0.002384579214	RAB18
SE	RSRC2	-	chr12:122520503-122520581	NA	0.157	0.000549099964	SE	KB-1572G7.2	-	chr22:23686837-23686995	NA	-0.382	0.002398430612	RAB26
SE	PGAP2	+	chr11:3811249-3811424	NA	0.149	0.0005535177013	SE	TCTN1	+	chr12:110644966-110645143	NA	-0.283	0.002401590692	RAB28
SE	KNTC1	+	chr12:122597730-122597938	NA	0.153	0.0005646569594	SE	CLOCK	-	chr4:55486392-55486549	NA	-0.197	0.002404395753	RAB34
SE	TBC1D32	-	chr6:121299445-121299505	NA	0.161	0.0005716012452	SE	ARID4B	-	chr1:235213768-235214026	NA	-0.111	0.002459905923	RAB3GAP1
SE	RP11-319G6.1	+	chr3:139419556-139419680	NA	0.16	0.0005716012452	SE	TRPC1	+	chr3:142791018-142791158	NA	-0.158	0.002527404038	RAB3IP
SE	KIAA1551	+	chr12:31987238-31987322	NA	0.143	0.0005753242167	SE	GRB10	-	chr7:50732271-50732368	NA	-0.158	0.002538626055	RAB5B
SE	CMC2	-	chr16:80981805-80981877	NA	0.148	0.0005766288072	SE	UBR3	+	chr2:169877493-169877637	NA	-0.104	0.002544829601	RABEPK
SE	MOK	-	chr14:102265822-102265912	NA	0.346	0.0005890502783	SE	KLHL7	+	chr7:23117833-23117964	NA	-0.334	0.002563569625	RABGAP1
SE	UBE3A	-	chr15:25407066-25407228	NA	0.146	0.0005917140631	SE	C17orf53	+	chr17:44154852-44154938	NA	-0.13	0.002587792456	RABGEF1
SE	KTN1	+	chr14:55673171-55673255	NA	0.144	0.0006166132953	SE	UBE2F	+	chr2:237987962-237987992	NA	-0.164	0.002623975802	RABGGTA
SE	METTL26	-	chr16:634888-634956	NA	0.133	0.0006246456998	SE	RP11-421L21.3	+	chr1:101075394-101075716	NA	-0.238	0.002624894621	RABL2B

SE	EEF1E1-BLOC1	-	chr6:8026366-8026425	NA	0.111	0.0006252997307	SE	KIAA0430	-	chr16:15624768-15624927	NA	-0.102	0.002706069451	RAC1
SE	YIPF1	-	chr1:53855000-53855051	NA	0.335	0.0006432768657	SE	ZC3H14	+	chr14:88596733-88596808	NA	-0.164	0.002711949392	RAD1
SE	EFHC1	+	chr6:52488556-52488675	NA	0.223	0.0006454851872	SE	HACE1	-	chr6:104824813-104824907	NA	-0.259	0.00276043376	RAD51
SE	CD320	-	chr19:8303854-8304088	NA	0.114	0.0006456606726	SE	TATDN1	-	chr8:124522150-124522200	NA	-0.101	0.002762253283	RAD51AP1
SE	MTX3	-	chr5:79990593-79990663	NA	0.13	0.0006469923546	SE	PLEKHM1P1	-	chr17:64814862-64815245	NA	-0.363	0.002766188792	RAD51D
SE	TRMU	+	chr22:46355989-46356072	NA	0.401	0.0006531052105	SE	POC1A	-	chr3:52096568-52096712	NA	-0.174	0.002778487747	RAD51L3-RFFL
SE	ENOSF1	-	chr18:691203-691276	NA	0.295	0.0006544003966	SE	DYRK4	+	chr12:4580823-4580977	NA	-0.667	0.002780083212	RAD54B
SE	BAZ2B	-	chr2:159397073-159397100	NA	0.236	0.0006556996521	SE	ZDHHC17	+	chr12:76821057-76821167	NA	-0.148	0.002782691887	RAD9A
SE	WWP2	+	chr16:69783031-69783152	NA	0.18	0.0006598131779	SE	SEC61A2	+	chr10:12167752-12167893	NA	-0.231	0.002784788117	RAI14
SE	PRDM2	+	chr1:13778417-13782831	NA	0.191	0.0006640598814	SE	WNK1	+	chr12:879572-880031	NA	-0.223	0.002784788117	RALGAPA1
SE	ZFAND6	+	chr15:80073265-80073330	NA	0.155	0.0006650621728	SE	NAT9	-	chr17:74773865-74774055	NA	-0.21	0.002803673059	RALGAPA2
SE	GZF1	+	chr20:23367002-23367097	NA	0.152	0.0006774829026	SE	RP11-286N22.8	+	chr11:61438003-61438113	NA	-0.152	0.002815028299	RALGPS1
SE	PARP6	-	chr15:72250844-72250954	NA	0.18	0.000680065943	SE	GK	+	chrX:30668011-30668118	NA	-0.365	0.002839945502	RALY
SE	PPP6R2	+	chr22:50436987-50437068	NA	0.202	0.0006841654985	SE	PLD3	+	chr19:40365717-40365930	NA	-0.211	0.002858947029	RANBP10
SE	CENPT	-	chr16:67832227-67832354	NA	0.107	0.0006888228651	SE	FASTKD1	-	chr2:169538012-169538141	NA	-0.147	0.002881470132	RANBP2
SE	MARK3	+	chr14:103498501-103498528	NA	0.341	0.0006888228651	SE	FAM120C	-	chrX:54087754-54087964	NA	-0.163	0.002895587267	RANBP3
SE	SLC35B1	-	chr17:49707342-49707444	NA	0.41	0.0006971692881	SE	KCTD17	+	chr22:37060822-37060922	NA	-0.226	0.00289981091	RANGAP1
SE	PHKB	+	chr16:47463898-47463993	NA	0.204	0.0007058046358	SE	AP1AR	+	chr4:112262987-112263086	NA	-0.121	0.002927110877	RAP1GAP
SE	PFDN5	+	chr12:53296429-53296551	NA	0.453	0.0007066383201	SE	PIGN	-	chr18:62163530-62163656	NA	-0.345	0.002929997565	RAPGEF1
SE	CENPT	-	chr16:67832227-67832350	NA	0.108	0.0007237793159	SE	ZNF561	-	chr19:9617620-9617758	NA	-0.122	0.002940491554	RAPGEF2
SE	CEP57L1	+	chr6:109106034-109106090	NA	0.427	0.0007319614165	SE	RAB26	+	chr16:2151129-2151315	NA	-0.161	0.002964538658	RAPGEF6
SE	TRIM35	-	chr8:27290155-27290178	NA	0.26	0.0007441076698	SE	ATPAF2	-	chr17:18021421-18021663	NA	-0.21	0.002988281976	RARS2
SE	ARPP19	-	chr15:52568847-52568900	NA	0.143	0.0007495632856	SE	GFM2	-	chr5:74730259-74730398	NA	-0.123	0.003000752999	RASSF4
SE	HNRNPA1	+	chr12:54281385-54281502	NA	0.112	0.0007498733205	SE	ZBED5-AS1	+	chr11:10865260-10865327	NA	-0.129	0.003016790343	RASSF8-AS1
SE	CCDC15	+	chr11:125003866-125003959	NA	0.204	0.0007539629551	SE	KCNAB3	-	chr17:7925096-7925183	NA	-0.145	0.003016887814	RB1CC1
SE	C9orf3	+	chr9:94928424-94928531	NA	0.575	0.0007666247027	SE	PEX5	+	chr12:7202240-7202351	NA	-0.238	0.003024317095	RBCK1
SE	ARHGEF40	+	chr14:21075644-21075765	NA	0.169	0.000780430881	SE	MORF4L1	+	chr15:78887268-78887349	NA	-0.129	0.003025881065	RBFOX2
SE	PUF60	-	chr8:143827384-143827536	NA	0.128	0.000780430881	SE	MYL6	+	chr12:56159586-56159622	NA	-0.114	0.003043669782	RBM23
SE	HIVEP2	-	chr6:142769551-142775125	NA	0.156	0.0007818481423	SE	TACC2	+	chr10:122143571-122143706	NA	-0.197	0.003050330045	RBM25
SE	TRIM16	-	chr17:15651090-15651946	NA	0.319	0.0007828582725	SE	CFAP44	-	chr3:113409105-113409322	NA	-0.236	0.003084990753	RBM26-AS1
SE	EEF1D	-	chr8:143590644-143590738	NA	0.311	0.0007911413037	SE	MIR99AHG	+	chr21:16537378-16537439	NA	-0.119	0.003127023316	RBM27
SE	PDPR	+	chr16:70114869-70114930	NA	0.311	0.0008000210449	SE	MYH14	+	chr19:50224153-50224177	NA	-0.159	0.003134453167	RBM33
SE	SEPT7	+	chr7:35831424-35831496	NA	0.371	0.0008025627237	SE	C14orf93	-	chr14:22999956-23000117	NA	-0.453	0.003140202336	RBM39
SE	CLK4	-	chr5:178619837-178620673	NA	0.125	0.0008055929555	SE	CARF	+	chr2:202974333-202974496	NA	-0.101	0.003230019984	RBM41
SE	MLX	+	chr17:42567618-42567655	NA	0.164	0.0008121704195	SE	SS18	-	chr18:26035830-26035923	NA	-0.123	0.003304618959	RBM5
SE	AMACR	-	chr5:34004573-34004734	NA	0.195	0.0008221747762	SE	KAT6A	-	chr8:42049575-42049707	NA	-0.266	0.003321961312	RBM6
SE	SENP3	+	chr17:7565694-7565764	NA	0.13	0.0008458178938	SE	KHK	+	chr2:27092331-27092448	NA	-0.403	0.003344420368	RBM8A
SE	INIP	-	chr9:112689526-112689617	NA	0.193	0.0008535483712	SE	SPIN1	+	chr9:88416643-88416766	NA	-0.452	0.003349315801	RBMS1
SE	ECHDC2	-	chr1:52905033-52906611	NA	0.209	0.0008643964118	SE	LYRM1	+	chr16:20902485-20902717	NA	-0.261	0.003349315801	RBPJ
SE	STX16	+	chr20:58659622-58659634	NA	0.138	0.0008643964118	SE	RAB5B	+	chr12:55979371-55979474	NA	-0.173	0.003363584424	RCAN3
SE	UPF3A	+	chr13:114286301-114286400	NA	0.217	0.0008678622588	SE	TCOF1	+	chr5:150372005-150372236	NA	-0.13	0.003432257142	RCBTB2
SE	ARHGAP32	-	chr11:129040927-129041009	NA	0.125	0.0008706042036	SE	STYXL1	-	chr7:76021850-76021992	NA	-0.112	0.003498861351	RCC1
SE	ARHGEF10	+	chr8:1880047-1880164	NA	0.324	0.000880675005	SE	SGCE	-	chr7:94639340-94639448	NA	-0.38	0.003512996048	RCHY1
SE	ZCWPW1	-	chr7:100402515-100402576	NA	0.541	0.0008828287501	SE	C1QTNF1	+	chr17:79043954-79044123	NA	-0.132	0.003512996048	RCL1
SE	ARHGEF40	+	chr14:21075622-21075765	NA	0.248	0.0008843185609	SE	RPAIN	+	chr17:5425970-5426299	NA	-0.151	0.00352321067	RCN1
SE	CCNE1	+	chr19:29812531-29812578	NA	0.23	0.0008983920389	SE	STYXL1	-	chr7:76013741-76013887	NA	-0.105	0.003523797492	RECQL
SE	RNF146	+	chr6:127286049-127286177	NA	0.264	0.0008983920389	SE	ZNF559-ZNF177	+	chr19:9339192-9339319	NA	-0.117	0.003549029187	RECQL5
SE	FBXL4	-	chr6:98880552-98880624	NA	0.299	0.0008985289339	SE	CCDC7	+	chr10:32567669-32567891	NA	-0.407	0.00357102587	RELA
SE	THUMPD2	-	chr2:39768900-39769093	NA	0.229	0.0009046763203	SE	ACACA	-	chr17:37345252-37345382	NA	-0.667	0.003589770542	RELT
SE	MBD1	-	chr18:50271468-50271540	NA	0.415	0.0009073322787	SE	IQCB1	-	chr3:121797117-121797227	NA	-0.338	0.003619441762	REPS1
SE	POC5	-	chr5:75712853-75712951	NA	0.104	0.0009107826143	SE	RTN2	-	chr19:45493159-45493378	NA	-0.366	0.00365172218	RETSAT
SE	PHC1	+	chr12:8921600-8921750	NA	0.284	0.0009122921912	SE	ZSWIM7	-	chr17:15986799-15986886	NA	-0.15	0.003676103307	RFC5
SE	SH3GLB2	-	chr9:129010669-129010693	NA	0.367	0.0009146336722	SE	ZNF611	-	chr19:52707391-52707538	NA	-0.506	0.00374323208	RFFL
SE	TTLL3	+	chr3:9818821-9818920	NA	0.357	0.0009150251426	SE	GRB10	-	chr7:50710874-50710990	NA	-0.197	0.00375709255	RFWD2
SE	PRR4	-	chr12:11121009-11121180	NA	0.119	0.0009347379716	SE	IAH1	+	chr2:9475986-9476039	NA	-0.21	0.003840014914	RFWD3
SE	TBC1D32	-	chr6:121255327-121255410	NA	0.174	0.0009385159804	SE	RPAIN	+	chr17:5425970-5426082	NA	-0.164	0.003868841766	RFX2
SE	SRSF6	+	chr20:43459152-43459420	NA	0.11	0.0009388080305	SE	ARF4-AS1	+	chr3:57600058-57600169	NA	-0.46	0.003887141779	RFX3
SE	PPM1K	-	chr4:88278535-88278642	NA	0.544	0.0009616700701	SE	XRRA1	-	chr11:74927390-74927488	NA	-0.244	0.003894370242	RGL1
SE	MON2	+	chr12:62566013-62566031	NA	0.421	0.0009638969816	SE	CYP20A1	+	chr2:203272669-203272748	NA	-0.143	0.003905914187	RGL2
SE	TCEA1	-	chr8:54002891-54003092	NA	0.308	0.0009906262505	SE	USP53	+	chr4:119268267-119268420	NA	-0.123	0.00393903467	RGMB-AS1
SE	ST7L	-	chr1:112540778-112540872	NA	0.192	0.0009928791088	SE	STK19	+	chr6:31972620-31972919	NA	-0.289	0.00395611319	RGS19
SE	EIF4A2	+	chr3:186788309-186788416	NA	0.195	0.0009928791088	SE	NAT1	+	chr8:18211192-18211342	NA	-0.138	0.003968071478	RGS5
SE	DEPDC5	+	chr22:31861367-31861433	NA	0.208	0.0009982071298	SE	GIT2	-	chr12:109951166-109951316	NA	-0.131	0.003988133396	RHEB
SE	GTPBP10	+	chr7:90354457-90354549	NA	0.163	0.001003586949	SE	WIPF1	-	chr2:174677044-174677124	NA	-0.321	0.004005348732	RHNO1
SE	TRIM65	-	chr17:75892754-75892850	NA	0.15	0.001012684496	SE	C9orf85	+	chr9:71971504-71971618	NA	-0.131	0.004011400497	RHOC
SE	DMPK	-	chr19:45780322-45780381	NA	0.189	0.001052296447	SE	MATR3	+	chr5:139293452-139293575	NA	-0.31	0.004029235655	RHOQ
SE	EXO5	+	chr1:40509732-40509790	NA	0.274	0.001054160684	SE	SLC35B3	-	chr6:8417401-8417494	NA	-0.187	0.00405415197	RHOT1
SE	MRPS28	-	chr8:80028694-80028796	NA	0.333	0.00106274245	SE	PICALM	-	chr11:85990249-85990378	NA	-0.41	0.004066478452	RHOT2
SE	HKR1	+	chr19:37322184-37322232	NA	0.262	0.001068047104	SE	AASDH	-	chr4:56345126-56345290	NA	-0.133	0.004080108744	RIC8B
SE	TNFRSF12A	+	chr16:3021188-3021319	NA	0.144	0.001101883147	SE	SETD2	-	chr3:47080800-47081083	NA	-0.136	0.004085034607	RIDA
SE	PPIP5K2	+	chr5:103135992-103136114	NA	0.577	0.001101883147	SE	TRIQK	-	chr8:92921502-92921660	NA	-0.19	0.00409329242	RIF1
SE	RXRB	-	chr6:33199168-33199416	NA	0.163	0.001107009695	SE	ADGRL2	+	chr1:81952986-81953025	NA	-0.368	0.004113255113	RIN2
SE	ECSIT	-	chr19:11519074-11519193	NA	0.11	0.001119481664	SE	TPT1-AS1	+	chr13:45360140-45360239	NA	-0.619	0.004168487247	RIN3
SE	ELFN2	-	chr22:37417768-37417919	NA	0.417	0.001125297341	SE	DLG4	-	chr17:7191892-7192002	NA	-0.116	0.004221254915	RIPK2
SE	APAF1	+	chr12:98703370-98703499	NA	0.278	0.001135475143	SE	DHFR	-	chr5:80637882-80638009	NA	-0.441	0.004239439132	RMDN1
SE	PRPF40B	+	chr12:49632595-49632623	NA	0.329	0.001138009614	SE	ZNF107	+	chr7:64691864-64691960	NA	-0.133	0.004266924805	RMDN3
SE	CENPX	-	chr17:82019640-82019694	NA	0.221	0.001167154583	SE	CARD8	-	chr19:48218870-48219012	NA	-0.107	0.004268594052	RMND1
SE	MTSS1L	-	chr16:70665465-70665540	NA	0.25	0.001167925376	SE	UBE2Q2	+	chr15:75890434-75890483	NA	-0.102	0.004301470106	RMND5B
SE	WAC-AS1	-	chr10:28525947-28526008	NA	0.149	0.001169338867	SE	VAPB	+	chr20:58434601-58434705	NA	-0.303	0.004308658414	RNASEH1-AS1
SE	DNM3	+	chr1:172093695-172093725	NA	0.149	0.001174848614	SE	UACA	-	chr15:70677107-70677140	NA	-0.119	0.004314478065	RNF10
SE	NEDD1	+	chr12:96907701-96907856	NA	0.23	0.001184309423	SE	NEK6	+	chr9:124292848-124293013	NA	-0.319	0.004357067677	RNF126
SE	ENOSF1	-	chr18:677344-677444	NA	0.104	0.001190248648	SE	URB2	+	chr1:229645858-229645969	NA	-0.118	0.004374083708	RNF14
SE	ZNF19	-	chr16:71482081-71482143	NA	0.476	0.001195407288	SE	CAPRIN2	-	chr12:30720810-30720915	NA	-0.234	0.004393856425	RNF146
SE	ACSL3	+	chr2:222900673-222900780	NA	0.192	0.00120846	SE	FAM35A	+	chr10:87175888-87176095	NA	-0.146	0.00447617254	RNF157
SE	BRD8	-	chr5:138157641-138157894	NA	0.218	0.001234249565	SE	LINC01125	+	chr2:97701153-97701304	NA	-0.424	0.00448074171	RNF181
SE	SIDT2	+	chr11:117186989-117187001	NA	0.102	0.001234249565	SE	MRRF	+	chr9:122286038-122286166	NA	-0.124	0.004509801696	RNF19B
SE	NDEL1	+	chr17:8463319-8463354	NA	0.323	0.001234956404	SE	FIP1L1	+	chr4:53391429-53391498	NA	-0.163	0.004672475626	RNF213
SE	SLC25A10	+	chr17:81713261-81713506	NA	0.269	0.001237237216	SE	YAF2	-	chr12:42210354-42210454	NA	-0.213	0.004674892494	RNF215
SE	FBXO44	+	chr1:11655327-11655699	NA	0.302	0.001244028305	SE	NR2F1-AS1	-	chr5:93438702-93438741	NA	-0.288	0.004687272022	RNF216
SE	AP1G1	-	chr16:71806685-71806728	NA	0.594	0.00126477858	SE	PTPRA	+	chr20:2947981-2948024	NA	-0.158	0.004696244476	RNF32
SE	ARHGEF3	-	chr3:57007181-57007336	NA	0.264	0.001289105061	SE	UBL7-AS1	+	chr15:74466035-74466130	NA	-0.286	0.00472504306	RNFT1
SE	THAP6	+	chr4:75542408-75542486	NA	0.404	0.001289105061	SE	MRPL52	+	chr14:22830963-22831037	NA	-0.361	0.004738364736	RNH1
SE	METTL26	-	chr16:634888-635063	NA	0.174	0.001291710649	SE	CWF19L1	-	chr10:100261978-100262063	NA	-0.149	0.004766249955	RNMT
SE	PRIMPOL	+	chr4:184682247-184682336	NA	0.3	0.001312112384	SE	FAM200B	+	chr4:15684849-15684959	NA	-0.173	0.004900798307	ROBO1
SE	TSC1	-	chr9:132921362-132921436	NA	0.143	0.001322173813	SE	ENO3	+	chr17:4952024-4952147	NA	-0.139	0.00490899262	RP11-10K16.1
SE	SLC35B3	-	chr6:8419579-8419677	NA	0.143	0.001346225949	SE	RAD54B	-	chr8:94432610-94432656	NA	-0.445	0.00493556984	RP11-1212A22.4
SE	BORCS8-MEF2	-	chr19:19180685-19180761	NA	0.197	0.001347769055	SE	ABCD4	-	chr14:74300149-74300268	NA	-0.162	0.004938847597	RP11-1228E12.1
SE	EEF1D	-	chr8:143590644-143590735	NA	0.479	0.001367554607	SE	ADGRG1	+	chr16:57641590-57641708	NA	-0.318	0.005039214886	RP11-156P1.3
SE	ZNF419	+	chr19:57490146-57490185	NA	0.458	0.001367971256	SE	TRPC1	+	chr3:142743484-142743586	NA	-0.25	0.005039214886	RP11-175P13.3
SE	BAZ1A	-	chr14:34786125-34786221	NA	0.32	0.001379584947	SE	CCNT1	-	chr12:48695998-48696162	NA	-0.117	0.005099606958	RP11-216L13.19
SE	SCML1	+	chrX:17744070-17744219	NA	0.339	0.001379584947	SE	TET2	+	chr4:105241338-105241429	NA	-0.147	0.005143745186	RP11-250B2.3
SE	RHEB	-	chr7:151498088-151498180	NA	0.227	0.001382000474	SE	CRELD1	+	chr3:9943901-9944095	NA	-0.229	0.00516164134	RP11-252A24.2
SE	RIN2	+	chr20:19964951-19965024	NA	0.162	0.001397451056	SE	TCTN1	+	chr12:110634395-110634463	NA	-0.353	0.005171587045	RP11-274B21.1
SE	SPEG	+	chr2:219444024-219444059	NA	0.281	0.001400638034	SE	FAM189B	-	chr1:155251094-155251164	NA	-0.295	0.005179405283	RP11-274B21.14
SE	GATAD2A	+	chr19:19436130-19436219	NA	0.313	0.00140484798	SE	ZNF654	+	chr3:88126133-88126269	NA	-0.197	0.005235334937	RP11-286N22.8
SE	FAM189B	-	chr1:155250275-155250929	NA	0.18	0.001414702212	SE	XRRA1	-	chr11:74851988-74852082	NA	-0.417	0.005245556191	RP11-295P9.3
SE	ADCY6	-	chr12:48773939-48774098	NA	0.182	0.001421550924	SE	POLL	-	chr10:101585315-101585478	NA	-0.186	0.005335128453	RP11-303E16.10
SE	SPIRE1	-	chr18:12459754-12459928	NA	0.108	0.001432568516	SE	NAA16	+	chr13:41336649-41336756	NA	-0.165	0.005376307953	RP11-315D16.2
SE	DUSP12	+	chr1:161751131-161751377	NA	0.39	0.001440110946	SE	ALOX12-AS1	-	chr17:7010058-7010137	NA	-0.167	0.005410559041	RP11-319G6.1
SE	USP36	-	chr17:78796316-78796494	NA	0.128	0.001449853653	SE	CCNL1	-	chr3:157156935-157157083	NA	-0.156	0.005432987046	RP11-33B1.1
SE	PARP8	+	chr5:50760291-50760362	NA	0.282	0.001454317555	SE	FLOT1	-	chr6:30741613-30741704	NA	-0.292	0.005445824529	RP11-345J4.5
SE	ABL2	-	chr1:179133311-179133374	NA	0.173	0.001458185891	SE	ARNTL2	+	chr12:27376345-27376387	NA	-0.204	0.005462729449	RP11-371E8.4
SE	ENOSF1	-	chr18:691067-691106	NA	0.182	0.001473628693	SE	ANK3	-	chr10:60059697-60060033	NA	-0.119	0.00550678331	RP11-421L21.3
SE	CAPN7	+	chr3:15245525-15245671	NA	0.287	0.001480159749	SE	FAM120C	-	chrX:54085714-54085916	NA	-0.142	0.005509184309	RP11-42I10.1
SE	MATR3	+	chr5:139314674-139314736	NA	0.14	0.001482217519	SE	GOSR2	+	chr17:46935028-46935225	NA	-0.111	0.005554562012	RP11-435I10.4
SE	SBNO2	-	chr19:1147308-1147420	NA	0.3	0.001484505828	SE	PSMA3-AS1	-	chr14:58293313-58293544	NA	-0.102	0.005554562012	RP11-452F19.3
SE	DPY19L2	-	chr12:63590998-63591127	NA	0.364	0.001505065008	SE	ZBTB8OS	-	chr1:32633261-32633354	NA	-0.104	0.005563206351	RP11-458D21.5
SE	MAP7D1	+	chr1:36174897-36175008	NA	0.155	0.001511022672	SE	MYNN	+	chr3:169774030-169774097	NA	-0.201	0.00559327814	RP11-499E18.1
SE	SLC38A6	+	chr14:61034018-61034113	NA	0.104	0.001528473657	SE	KANSL2	-	chr12:48679035-48679238	NA	-0.226	0.005663637832	RP11-49K24.6
SE	SEPT2	+	chr2:241316421-241316540	NA	0.447	0.001538786731	SE	RWDD3	+	chr1:95246541-95246657	NA	-0.14	0.005692088547	RP11-507M3.1
SE	IQGAP3	-	chr1:156554234-156554392	NA	0.251	0.001556186387	SE	THAP6	+	chr4:75521735-75521861	NA	-0.129	0.005749812137	RP11-51F16.8

SE	NETO2	-	chr16:47086225-47086339	NA	0.128	0.001562775525	SE	LIMK2	+	chr22:31225719-31225819	NA	-0.168	0.005763158056	RP11-580I16.2
SE	EPB41L1	+	chr20:36195328-36195364	NA	0.293	0.001588997599	SE	CSAD	-	chr12:53171325-53171441	NA	-0.452	0.00576997794	RP11-705C15.2
SE	ARHGEF39	-	chr9:35662945-35663074	NA	0.169	0.001596040472	SE	TJP2	+	chr9:69249374-69249485	NA	-0.187	0.005796639247	RP11-73M18.2
SE	LIN9	-	chr1:226295841-226295946	NA	0.198	0.001604315093	SE	ULBP2	+	chr6:149947319-149947451	NA	-0.15	0.005873187661	RP11-762I7.5
SE	GPBP1L1	-	chr1:45685575-45685620	NA	0.241	0.001604980824	SE	KIF21B	-	chr1:200983055-200983094	NA	-0.388	0.005890845208	RP11-849H4.2
SE	RAD51L3-RFFL	-	chr17:35107365-35107447	NA	0.493	0.001624246109	SE	LTBP4	+	chr19:40617099-40617225	NA	-0.159	0.005927429303	RP11-9G1.3
SE	WARS	-	chr14:100375282-100375406	NA	0.432	0.001631060496	SE	ZNF561	-	chr19:9617620-9617768	NA	-0.105	0.006032195647	RP13-794C1.1
SE	RP5-1021I20.4	+	chr14:73922065-73922206	NA	0.198	0.001649349498	SE	L3HYPDH	-	chr14:59474438-59474526	NA	-0.268	0.006032195647	RP13-968A2.1
SE	CEP57L1	+	chr6:109155790-109155877	NA	0.116	0.001692970305	SE	MATR3	+	chr5:139279934-139280058	NA	-0.199	0.006055130299	RP4-614C10.2
SE	ELF2	-	chr4:139116648-139116807	NA	0.572	0.001701782054	SE	MORC2	-	chr22:30963224-30963334	NA	-0.537	0.006121948866	RP4-639F20.1
SE	KIF1B	+	chr1:10326110-10326359	NA	0.345	0.001701782054	SE	TNRC6A	+	chr16:24776932-24777358	NA	-0.105	0.006129789789	RP4-714D9.5
SE	RGS5	-	chr1:163319181-163319291	NA	0.123	0.001702146197	SE	TRIM16	-	chr17:15682860-15682951	NA	-0.131	0.006132731773	RP4-777O23.3
SE	RIF1	+	chr2:151468473-151468551	NA	0.219	0.001720428133	SE	TRIM16	-	chr17:15681050-15681222	NA	-0.577	0.006143326895	RP4-798P15.3
SE	TBL1XR1	-	chr3:177098465-177098541	NA	0.273	0.001724332248	SE	CARD8	-	chr19:48218870-48219012	NA	-0.144	0.006207750042	RP5-1021I20.4
SE	MPRIP	+	chr17:17180606-17180669	NA	0.41	0.00173447688	SE	IFI27L1	+	chr14:94097494-94097678	NA	-0.265	0.006289332786	RPAIN
SE	MLXIP	+	chr12:122132291-122132383	NA	0.121	0.001737699878	SE	FBXL12	-	chr19:9818544-9818617	NA	-0.14	0.006348023345	RPE
SE	ZFYVE21	+	chr14:103729792-103729846	NA	0.185	0.001742917457	SE	ANXA2	-	chr15:60396150-60396427	NA	-0.362	0.006386827087	RPGR
SE	UPP1	+	chr7:48103296-48103411	NA	0.155	0.001796423469	SE	RBCK1	+	chr20:409883-410025	NA	-0.12	0.006423577048	RPL12
SE	ADGRL3	+	chr4:61730621-61730636	NA	0.224	0.001796423469	SE	FRG1HP	+	chr9:40993252-40993434	NA	-0.165	0.006454968809	RPL13A
SE	MYO9A	-	chr15:71892964-71893177	NA	0.389	0.001802867873	SE	KIAA0753	-	chr17:6611918-6612148	NA	-0.124	0.006509517624	RPL17
SE	FAM131A	+	chr3:184337310-184337371	NA	0.177	0.001823831485	SE	TRIM69	+	chr15:44759747-44759872	NA	-0.144	0.006509517624	RPL17-C18orf32
SE	RPS3	+	chr11:75401639-75401733	NA	0.255	0.001839342785	SE	FEZ2	-	chr2:36560784-36560865	NA	-0.386	0.006549900596	RPL21
SE	NEDD1	+	chr12:96924832-96924942	NA	0.202	0.001854735332	SE	LZTFL1	-	chr3:45837926-45838051	NA	-0.136	0.006670359649	RPL22L1
SE	URB1	-	chr21:32378444-32378541	NA	0.101	0.001888259153	SE	ERCC6L2	+	chr9:95967458-95967625	NA	-0.229	0.006683548413	RPL3
SE	TNFRSF12A	+	chr16:3021214-3021319	NA	0.123	0.001898049618	SE	SLCO4A1	+	chr20:62668157-62668184	NA	-0.51	0.006698640151	RPL7L1
SE	SDCCAG8	+	chr1:243293090-243293219	NA	0.255	0.001899552138	SE	DYNC1I2	+	chr2:171707232-171707377	NA	-0.218	0.006705666254	RPL9
SE	MIER2	-	chr19:336082-336173	NA	0.29	0.001904359178	SE	ANAPC10	-	chr4:145097687-145097760	NA	-0.592	0.006742072944	RPLP0
SE	TTC28-AS1	+	chr22:27989881-27990026	NA	0.514	0.001921405105	SE	SH2B1	+	chr16:28873169-28873269	NA	-0.129	0.006763217863	RPP21
SE	UBA3	-	chr3:69071534-69071617	NA	0.622	0.001965233616	SE	PDE9A	+	chr21:42731769-42731949	NA	-0.281	0.006781962229	RPP25L
SE	RP11-315D16.2	-	chr15:68218535-68218650	NA	0.377	0.001966429415	SE	PCNX1	+	chr14:70988566-70988699	NA	-0.316	0.006891588316	RPP30
SE	RIC8B	+	chr12:106874480-106874567	NA	0.257	0.001966429415	SE	SEPSECS	-	chr4:25159627-25159764	NA	-0.194	0.006939518803	RPP40
SE	KMT5C	+	chr19:55343977-55343997	NA	0.336	0.001968293186	SE	DLG3	+	chrX:70497179-70497221	NA	-0.148	0.006943205476	RPRD2
SE	NPHP3	-	chr3:132699352-132699450	NA	0.298	0.002000095534	SE	SMUG1	-	chr12:54187818-54187905	NA	-0.443	0.006943253512	RPS24
SE	CAMTA2	-	chr17:4982088-4982227	NA	0.274	0.002049001138	SE	KCTD9	-	chr8:25446128-25446250	NA	-0.335	0.007073635801	RPS27
SE	CPEB2	+	chr4:15040463-15040487	NA	0.139	0.002054465018	SE	BRPF3	+	chr6:36225270-36225364	NA	-0.263	0.00709572117	RPS28
SE	PFKM	+	chr12:48118481-48118577	NA	0.295	0.002062230091	SE	TADA2A	+	chr17:37423508-37423615	NA	-0.112	0.007108285273	RPS3
SE	VPS13B	+	chr8:99507769-99507911	NA	0.44	0.00206399437	SE	TRDMT1	-	chr10:17168840-17168917	NA	-0.296	0.007123014945	RPS3A
SE	CEP152	-	chr15:48748442-48748610	NA	0.127	0.002066660603	SE	APOPT1	+	chr14:103579370-103579491	NA	-0.184	0.00718773629	RPS6KB2
SE	URI1	+	chr19:29971192-29971227	NA	0.253	0.002066660603	SE	PCDHGC3	+	chr5:141478393-141478546	NA	-0.206	0.007191723509	RPS9
SE	ADGRL1	-	chr19:14167015-14167030	NA	0.204	0.002071693895	SE	ZDHHC4	+	chr7:6577589-6577720	NA	-0.177	0.007214427814	RPTOR
SE	SGO1	-	chr3:20174248-20175055	NA	0.231	0.002075918316	SE	SLC33A1	-	chr3:155833467-155833585	NA	-0.117	0.00728625754	RPUSD3
SE	HGSNAT	+	chr8:43173712-43173743	NA	0.232	0.002089000179	SE	PRKAB1	+	chr12:119671494-119671668	NA	-0.152	0.007309215227	RPUSD4
SE	PPM1A	+	chr14:60250379-60250481	NA	0.136	0.002092126104	SE	RPUSD3	-	chr3:9838984-9839171	NA	-0.136	0.007378662604	RREB1
SE	NSRP1	+	chr17:30118079-30118173	NA	0.239	0.002111221232	SE	IFI27L1	+	chr14:94097491-94097678	NA	-0.249	0.007405164285	RRNAD1
SE	DGKA	+	chr12:55932560-55932909	NA	0.472	0.002122617065	SE	NHSL1	-	chr6:138441982-138442114	NA	-0.476	0.007436656522	RRP12
SE	UGGT1	+	chr2:128156391-128156415	NA	0.114	0.002140032246	SE	C1orf159	-	chr1:1083914-1084011	NA	-0.411	0.00746974798	RSG1
SE	SPEG	+	chr2:219444029-219444059	NA	0.302	0.002168602909	SE	ATXN2	-	chr12:111516153-111516363	NA	-0.261	0.007572864754	RSRC2
SE	PKIG	+	chr20:44585099-44585204	NA	0.289	0.002174269931	SE	AC138969.4	+	chr16:16335860-16336137	NA	-0.26	0.007599994575	RSRP1
SE	AGTPBP1	-	chr9:85660935-85660973	NA	0.203	0.002196361706	SE	STK40	-	chr1:36367851-36368084	NA	-0.157	0.007647461604	RSU1
SE	TMEM25	+	chr11:118533419-118533551	NA	0.48	0.002210906602	SE	SNHG14	+	chr15:24998969-24999078	NA	-0.19	0.007705740173	RTKN
SE	ZNF75D	-	chrX:135293729-135294258	NA	0.316	0.002220951479	SE	EYA3	-	chr1:28027788-28027926	NA	-0.142	0.007720184609	RTN2
SE	DZIP3	+	chr3:108605334-108605438	NA	0.116	0.002236110888	SE	KBTBD3	-	chr11:106076506-106076707	NA	-0.16	0.007742020846	RTN4
SE	PEX7	+	chr6:136869889-136870003	NA	0.32	0.002238644159	SE	TMEM94	+	chr17:75492238-75492337	NA	-0.168	0.007872163172	RUFY3
SE	NAGLU	+	chr17:42537397-42537545	NA	0.267	0.002270954097	SE	MTA1	+	chr14:105466706-105466742	NA	-0.111	0.007911333933	RUNX2
SE	CENPX	-	chr17:82019509-82019694	NA	0.172	0.002280843257	SE	GLT8D1	-	chr3:52704723-52704952	NA	-0.203	0.00793029098	RUSC1
SE	IL11RA	+	chr9:34660803-34660936	NA	0.179	0.00233625466	SE	ABHD11	-	chr7:73737220-73737360	NA	-0.193	0.007980648665	RWDD1
SE	NAGPA	-	chr16:5028296-5028398	NA	0.287	0.00234344699	SE	TRPC1	+	chr3:142792823-142792967	NA	-0.13	0.007980648665	RWDD3
SE	TKFC	+	chr11:61343341-61343458	NA	0.109	0.002348355715	SE	SLC25A26	+	chr3:66236543-66236700	NA	-0.363	0.00798304603	RXRB
SE	UBE3A	-	chr15:25407066-25407262	NA	0.117	0.002354815036	SE	ZGRF1	-	chr4:112623816-112623876	NA	-0.145	0.008185219315	RYK
SE	VPS9D1	-	chr16:89708426-89708531	NA	0.817	0.002386013963	SE	POLQ	-	chr3:121473349-121473487	NA	-0.124	0.008199787959	SACS
SE	BCOR	-	chrX:40071636-40071690	NA	0.268	0.002441790178	SE	HMGN1	-	chr21:39347378-39347483	NA	-0.241	0.008201186754	SAMD4A
SE	FAM13B	-	chr5:137956476-137956542	NA	0.265	0.002442117802	SE	STK40	-	chr1:36367847-36368084	NA	-0.133	0.008231467309	SAMD4B
SE	SMTN	+	chr22:31080449-31080754	NA	0.259	0.002442197371	SE	PICALM	-	chr11:85976622-85976682	NA	-0.151	0.008270511682	SAP30BP
SE	PRKAR1A	+	chr17:68514986-68515119	NA	0.137	0.002459905923	SE	KB-1572G7.2	-	chr22:23686830-23686995	NA	-0.362	0.008304198588	SART3
SE	NLE1	-	chr17:35134942-35135059	NA	0.179	0.002462945607	SE	ZNF75A	+	chr16:3313048-3313175	NA	-0.223	0.00831961416	SASH1
SE	CPSF7	-	chr11:61420469-61420530	NA	0.111	0.002471171293	SE	DHODH	+	chr16:72021123-72021311	NA	-0.152	0.00832100594	SAT2
SE	GAK	-	chr4:851749-851974	NA	0.131	0.002471213769	SE	LTBP1	+	chr2:33342837-33342963	NA	-0.376	0.008353634004	SBF1
SE	RTKN	-	chr2:74439550-74439977	NA	0.359	0.002527404038	SE	ZNF211	+	chr19:57633712-57633905	NA	-0.257	0.008374122316	SBF2
SE	PNPLA6	+	chr19:7541983-7542067	NA	0.137	0.002534240441	SE	C6orf52	-	chr6:10686965-10687056	NA	-0.117	0.0084424949	SBNO2
SE	POLG	-	chr15:89332217-89332374	NA	0.207	0.002540358248	SE	SREBF1	-	chr17:17823517-17823607	NA	-0.302	0.008461191506	SCAI
SE	CATSPER2	-	chr15:43647049-43647118	NA	0.467	0.002544829601	SE	ITGB1BP1	-	chr2:9419989-9420100	NA	-0.285	0.008497664699	SCAMP3
SE	DDX58	-	chr9:32492390-32492538	NA	0.106	0.002559520408	SE	PBRM1	-	chr3:52554723-52554879	NA	-0.286	0.008665320904	SCAPER
SE	CCDC14	-	chr3:123956354-123956427	NA	0.525	0.002598953941	SE	CDK4	-	chr12:57750441-57750563	NA	-0.44	0.008685505951	SCHIP1
SE	CTD-2583A14.1	+	chr19:57820493-57820549	NA	0.225	0.00260091679	SE	TTC38	+	chr22:46281970-46282151	NA	-0.168	0.008685505951	SCLT1
SE	KIAA1217	+	chr10:24542692-24542770	NA	0.293	0.00260091679	SE	MFF	+	chr2:227328677-227330846	NA	-0.184	0.008685505951	SCLY
SE	ARAP1	-	chr11:72732514-72732597	NA	0.29	0.002607277755	SE	AURKA	-	chr20:56390555-56390665	NA	-0.227	0.008722123107	SCMH1
SE	IFT88	+	chr13:20631015-20631102	NA	0.152	0.002607315509	SE	GGACT	-	chr13:100533562-100533774	NA	-0.122	0.008751623156	SCML1
SE	FAM134C	-	chr17:42587833-42587864	NA	0.151	0.002611775813	SE	CLHC1	-	chr2:55208625-55208710	NA	-0.228	0.008763410401	SCP2
SE	TMEM230	-	chr20:5111763-5111808	NA	0.104	0.002611775813	SE	CARD8	-	chr19:48206383-48206520	NA	-0.225	0.008844746833	SCPEP1
SE	WASHC2A	+	chr10:50092161-50092233	NA	0.141	0.002623125021	SE	MYO19	-	chr17:36512657-36512779	NA	-0.114	0.008854836282	SCRN3
SE	PARP6	-	chr15:72250844-72250957	NA	0.195	0.002626254247	SE	TAB3	-	chrX:30842965-30843049	NA	-0.131	0.0088897773	SCYL1
SE	PDCD10	-	chr3:167725400-167725473	NA	0.136	0.002643161609	SE	CHEK2	-	chr22:28703504-28703566	NA	-0.105	0.008912603064	SDCCAG3
SE	MCM9	-	chr6:118856370-118856545	NA	0.342	0.002662136025	SE	ICA1L	-	chr2:202870529-202870656	NA	-0.618	0.008958063124	SDCCAG8
SE	ZNF140	+	chr12:133081272-133081329	NA	0.172	0.002671777406	SE	DDIAS	+	chr11:82930156-82930274	NA	-0.128	0.009033374603	SDHAP1
SE	PHKB	+	chr16:47463881-47463993	NA	0.207	0.002682815417	SE	ATG5	-	chr6:106308363-106308491	NA	-0.132	0.009067089434	SDHAP2
SE	ZBTB14	-	chr18:5293971-5294001	NA	0.26	0.002715613574	SE	CHFR	-	chr12:132870723-132870783	NA	-0.113	0.009067303247	SDHC
SE	FOXD3-AS1	-	chr1:63321664-63322080	NA	0.279	0.002721675563	SE	ADAT1	-	chr16:75618585-75618698	NA	-0.123	0.009096233477	SDR39U1
SE	FUK	+	chr16:70469151-70469323	NA	0.247	0.002737544214	SE	KTN1	+	chr14:55601756-55601880	NA	-0.221	0.009128552165	SEC14L2
SE	SLC25A42	+	chr19:19104912-19104938	NA	0.188	0.00274477048	SE	FAM200B	+	chr4:15684885-15684959	NA	-0.144	0.009167109226	SEC22C
SE	WDR45	-	chrX:49077642-49077747	NA	0.23	0.002782540749	SE	GCSH	-	chr16:81084462-81084534	NA	-0.473	0.009167109226	SEC24B
SE	RWDD1	+	chr6:116572857-116572985	NA	0.236	0.002782540749	SE	CYP20A1	+	chr2:203272645-203272748	NA	-0.229	0.00918764879	SEC24C
SE	CCP110	+	chr16:19524779-19525019	NA	0.315	0.002784788117	SE	WDR20	+	chr14:102197754-102197847	NA	-0.174	0.009240772418	SEC31A
SE	C4orf36	-	chr4:86932221-86932319	NA	0.427	0.002784788117	SE	MFF	+	chr2:227347225-227347384	NA	-0.14	0.009275745962	SEC61A2
SE	SGCE	-	chr7:94644605-94644667	NA	0.295	0.002784788117	SE	COQ3	-	chr6:99383007-99383130	NA	-0.107	0.009287960857	SECISBP2
SE	DGUOK	+	chr2:73957124-73957240	NA	0.204	0.002786142805	SE	ATG4D	+	chr19:10546838-10547253	NA	-0.185	0.009304957146	SELENOF
SE	NCOR2	-	chr12:124327546-124327633	NA	0.149	0.002803673059	SE	ARL13B	+	chr3:93996562-93996684	NA	-0.413	0.009312346136	SEMA3D
SE	SGCE	-	chr7:94639340-94639448	NA	0.116	0.002822164336	SE	DPY19L3	+	chr19:32467473-32467626	NA	-0.201	0.009323277483	SEMA4F
SE	GTPBP10	+	chr7:90355085-90355230	NA	0.115	0.002839945502	SE	PPHLN1	+	chr12:42351884-42352049	NA	-0.174	0.009409870388	SEMA6C
SE	PQLC3	+	chr2:11174967-11175009	NA	0.106	0.00284845948	SE	NAA16	+	chr13:41355143-41355216	NA	-0.106	0.009473724616	SENP3
SE	KNTC1	+	chr12:122601535-122601625	NA	0.121	0.002873453659	SE	MBD1	-	chr18:50273333-50273471	NA	-0.124	0.009485303594	SENP5
SE	RHOC	-	chr1:112705099-112705201	NA	0.18	0.002921158496	SE	GS1-124K5.12	-	chr7:66576754-66576929	NA	-0.184	0.009495284401	SENP6
SE	RIF1	+	chr2:151480097-151480227	NA	0.489	0.002957482326	SE	ADGRG1	+	chr16:57641586-57641708	NA	-0.443	0.009505385205	SENP7
SE	KIAA1551	+	chr12:31970188-31970356	NA	0.106	0.002983483307	SE	DNAJC19	-	chr3:180986997-180987022	NA	-0.198	0.009511077212	SEPSECS
SE	RECQL	-	chr12:21501169-21501372	NA	0.113	0.002987512401	SE	ZMYM5	-	chr13:19837655-19837821	NA	-0.131	0.009523405635	SEPT10
SE	UBE2Q2P2	+	chr15:82415527-82415675	NA	0.258	0.002987989726	SE	MRNIP	-	chr5:179851242-179851337	NA	-0.108	0.009537224812	SEPT2
SE	TCF7L2	+	chr10:113159919-113159992	NA	0.415	0.002988281976	SE	ALG2	-	chr9:99220978-99221137	NA	-0.252	0.009618423766	SEPT6
SE	ATP11A	+	chr13:112878216-112878303	NA	0.461	0.002993621228	SE	ZBED3-AS1	+	chr5:77100235-77100391	NA	-0.276	0.009747054499	SEPT7
SE	CDCA7	+	chr2:173363999-173364161	NA	0.102	0.003022493822	SE	EML1	+	chr14:99914565-99914697	NA	-0.141	0.01019963675	SEPT7P2
SE	PQLC1	-	chr18:79933968-79934022	NA	0.39	0.003043669782	SE	SLC37A3	-	chr7:140345868-140345970	NA	-0.111	0.01020257768	SEPT9
SE	EMC3-AS1	+	chr3:9987905-9988007	NA	0.589	0.003046176652	SE	WASF1	-	chr6:110178597-110178742	NA	-0.166	0.01033646562	SERGEF
SE	SNAP47	+	chr1:227731584-227731747	NA	0.402	0.00308346604	SE	TRAPPC6B	-	chr14:39154210-39154294	NA	-0.118	0.01034319119	SERHL2
SE	CREM	+	chr10:35201450-35201486	NA	0.132	0.003127472154	SE	MTERF4	-	chr2:241099395-241099894	NA	-0.153	0.01052679412	SERPINB6
SE	RBM39	-	chr20:35740524-35740597	NA	0.154	0.003153225159	SE	NEDD4	-	chr15:55991925-55992005	NA	-0.318	0.01072761332	SETD1A
SE	ARVCF	-	chr22:19972357-19972411	NA	0.124	0.003171110219	SE	L3HYPDH	-	chr14:59474438-59474526	NA	-0.206	0.01072927059	SETD2
SE	C3orf18	-	chr3:50566005-50566094	NA	0.3	0.003176882036	SE	DOLPP1	+	chr9:129086138-129086267	NA	-0.101	0.01074850663	SETD4
SE	FAM118B	+	chr11:126229224-126229293	NA	0.261	0.003200271846	SE	C1orf52	-	chr1:85258934-85259061	NA	-0.229	0.01080748181	SETD5
SE	NSUN5P1	+	chr7:75411654-75411759	NA	0.317	0.003212828364	SE	C12orf65	+	chr12:123253646-123255033	NA	-0.119	0.01080983012	SETD9
SE	TCF7	+	chr5:134144796-134144869	NA	0.3	0.003218971638	SE	RP11-216L13.19	+	chr9:136809254-136809429	NA	-0.207	0.01082847443	SETX
SE	MRPS15	-	chr1:36461263-36461312	NA	0.47	0.003230017772	SE	LINC01001	-	chr11:134563-134796	NA	-0.533	0.01082847443	SF1
SE	GNE	-	chr9:36236831-36236984	NA	0.127	0.003266110278	SE	TFDP1	+	chr13:113631622-113631905	NA	-0.13	0.01092216805	SFI1
SE	CLYBL	+	chr13:99862990-99863092	NA	0.159	0.003291280702	SE	TRIM16	-	chr17:15677175-15677280	NA	-0.403	0.01092216805	SFSWAP

SE	BROX	+	chr1:222715144-222715269	NA	0.353	0.00330261625	SE	EPB41L1	+	chr20:36173763-36173954	NA	-0.442	0.0111182907	SFT2D1
SE	MCRIP2	+	chr16:646471-646608	NA	0.358	0.003349315801	SE	SNHG3	+	chr1:28508127-28508160	NA	-0.173	0.0111445589	SFXN2
SE	ZNF569	-	chr19:37454833-37455073	NA	0.129	0.003350576238	SE	PRR3	+	chr6:30558149-30558212	NA	-0.208	0.01114936122	SGCE
SE	IP6K2	-	chr3:48694458-48694525	NA	0.133	0.003363630737	SE	C11orf54	+	chr11:93741962-93742094	NA	-0.24	0.01116962224	SGK494
SE	PCBP1-AS1	-	chr2:70059642-70059680	NA	0.334	0.003387004345	SE	ACAD10	+	chr12:111729805-111729956	NA	-0.108	0.01123275744	SGO1
SE	LRRC27	+	chr10:132342212-132342271	NA	0.468	0.003432257142	SE	ATG10	+	chr5:82164398-82164537	NA	-0.157	0.01125548831	SGSM2
SE	PAPD4	+	chr5:79615074-79615163	NA	0.112	0.003439201947	SE	LYRM1	+	chr16:20902485-20902717	NA	-0.221	0.01134847022	SH2B1
SE	VRK3	-	chr19:50009235-50009385	NA	0.166	0.003445306513	SE	MUTYH	-	chr1:45332917-45332959	NA	-0.303	0.01134847022	SH3BP2
SE	THAP9	+	chr4:82905860-82905926	NA	0.179	0.003447264686	SE	RNFT1	-	chr17:59962539-59962616	NA	-0.248	0.01146618412	SH3D19
SE	RBM27	+	chr5:146251710-146251875	NA	0.2	0.003498229501	SE	ZSCAN32	-	chr16:3390009-3390183	NA	-0.271	0.01146618412	SH3GLB2
SE	RGS19	-	chr20:64076856-64076954	NA	0.313	0.003517135917	SE	ZC3H13	-	chr13:46051931-46051989	NA	-0.187	0.01151561822	SH3PXD2A
SE	RP11-295P9.3	+	chr10:13654088-13654258	NA	0.33	0.003522034591	SE	RP11-849H4.2	-	chr11:71923308-71923475	NA	-0.195	0.01157165107	SH3TC2
SE	LUC7L2	+	chr7:139375508-139375583	NA	0.459	0.003536476151	SE	HUWE1	-	chrX:53625785-53626034	NA	-0.207	0.01157165107	SH3YL1
SE	EPB41	+	chr1:29058810-29058852	NA	0.139	0.003539628202	SE	ZEB1	+	chr10:31373017-31373173	NA	-0.149	0.0115916586	SHARPIN
SE	NAGK	+	chr2:71070740-71070839	NA	0.422	0.003548756426	SE	DLG1	-	chr3:197161639-197161738	NA	-0.183	0.01173470505	SHF
SE	AIFM1	-	chrX:130155155-130155286	NA	0.488	0.003549029187	SE	LRP2BP	-	chr4:185378080-185378207	NA	-0.311	0.01178140079	SHKBP1
SE	CAMTA2	-	chr17:4982088-4982175	NA	0.265	0.003568838564	SE	AFMID	+	chr17:78204827-78204900	NA	-0.196	0.01185604305	SHMT1
SE	ARFGAP2	-	chr11:47172280-47172333	NA	0.138	0.00357102587	SE	MGAT5	+	chr2:134253094-134253218	NA	-0.373	0.01190796755	SIDT2
SE	PER3	+	chr1:7787197-7787246	NA	0.168	0.00357297092	SE	POLL	-	chr10:101585861-101586156	NA	-0.179	0.01196858008	SIL1
SE	WARS	-	chr14:100375282-100375350	NA	0.109	0.003593871272	SE	OSER1-AS1	+	chr20:44214887-44215003	NA	-0.236	0.01202849288	SIN3B
SE	VEPH1	-	chr3:157495211-157495506	NA	0.107	0.003593871272	SE	OPA1	+	chr3:193667370-193667455	NA	-0.506	0.01205057511	SIPA1L1
SE	EXO5	+	chr1:40509450-40509625	NA	0.215	0.003593871272	SE	MICAL1	-	chr6:109447874-109447963	NA	-0.132	0.01210955425	SIPA1L3
SE	EIF4A2	+	chr3:186788309-186788420	NA	0.222	0.00365172218	SE	RP11-849H4.2	-	chr11:71925932-71926104	NA	-0.261	0.01221869075	SIRT7
SE	SPAG1	+	chr8:100220278-100220431	NA	0.111	0.003664403339	SE	ZNF468	-	chr19:52853871-52854126	NA	-0.214	0.01228624385	SLC11A2
SE	CLIP1	-	chr12:122351110-122351143	NA	0.111	0.003673171316	SE	METTL4	-	chr18:2547354-2547529	NA	-0.114	0.01235782639	SLC12A2
SE	GPCPD1	-	chr20:5586193-5586269	NA	0.46	0.003724555554	SE	TFDP2	-	chr3:141995019-141995141	NA	-0.144	0.01243222895	SLC12A4
SE	FAM120B	+	chr6:170358225-170358318	NA	0.141	0.003756883991	SE	CTD-2651B20.3	-	chr15:45264626-45264809	NA	-0.287	0.01243842924	SLC16A4
SE	PER3	+	chr1:7787197-7787246	NA	0.575	0.003807338828	SE	FBF1	-	chr17:75935631-75935673	NA	-0.31	0.01257062869	SLC20A2
SE	RPS6KB2	+	chr11:67432355-67432492	NA	0.228	0.003857761711	SE	DNAL1	+	chr14:73653169-73653215	NA	-0.118	0.01260941449	SLC22A17
SE	FLYWCH1	+	chr16:2913005-2913250	NA	0.23	0.003866750586	SE	KLC1	+	chr14:103684985-103685036	NA	-0.248	0.01272844939	SLC23A2
SE	PCBP1-AS1	-	chr2:70053730-70053792	NA	0.657	0.003866796193	SE	FAM92A	+	chr8:93718674-93718788	NA	-0.125	0.01275158129	SLC24A1
SE	SLC29A1	+	chr6:44226060-44226167	NA	0.22	0.003873995416	SE	CDKL3	-	chr5:134308573-134308727	NA	-0.188	0.0127687169	SLC25A10
SE	PVT1	+	chr8:128070159-128070272	NA	0.138	0.003876184563	SE	TLE1	-	chr9:81652183-81652288	NA	-0.191	0.01278775258	SLC25A14
SE	RALGAPA1	-	chr14:35700161-35700302	NA	0.434	0.003894370242	SE	SH3YL1	-	chr2:256206-256690	NA	-0.149	0.0128107561	SLC25A19
SE	ELOVL5	-	chr6:53290400-53290535	NA	0.395	0.003902246943	SE	ZNF317	+	chr19:9157979-9158075	NA	-0.114	0.01281397378	SLC25A22
SE	KLC1	+	chr14:103692358-103692425	NA	0.228	0.003905473379	SE	KCTD9	-	chr8:25446079-25446250	NA	-0.323	0.01287710281	SLC25A26
SE	IL17RC	+	chr3:9930399-9930459	NA	0.205	0.003957427051	SE	WDR89	-	chr14:63641298-63641407	NA	-0.131	0.01313831589	SLC25A29
SE	PYM1	-	chr12:55927075-55927267	NA	0.329	0.003959039809	SE	NCOA1	+	chr2:24768078-24768135	NA	-0.171	0.01318176908	SLC25A3
SE	DNAJC13	+	chr3:132462466-132462523	NA	0.13	0.003988133396	SE	MRNIP	-	chr5:179853196-179853276	NA	-0.36	0.01335046322	SLC25A30
SE	SENP5	+	chr3:196927795-196927879	NA	0.201	0.003991670538	SE	REPS1	-	chr6:138926400-138926481	NA	-0.27	0.01380120963	SLC25A36
SE	ULK3	-	chr15:74838151-74838192	NA	0.127	0.004060614688	SE	ZNF678	+	chr1:227646543-227646670	NA	-0.293	0.01382788928	SLC25A40
SE	TTC7A	+	chr2:46958494-46958552	NA	0.432	0.004065365915	SE	KCTD17	+	chr22:37061538-37061629	NA	-0.228	0.01385980222	SLC25A42
SE	RARS2	-	chr6:87545615-87545699	NA	0.112	0.004068455867	SE	SEC24B	+	chr4:109481676-109481781	NA	-0.216	0.01392337102	SLC26A11
SE	QRICH1	-	chr3:49092396-49092456	NA	0.337	0.004077983224	SE	POLL	-	chr10:101584877-101584919	NA	-0.34	0.01394848916	SLC26A6
SE	CENPE	-	chr4:103182761-103182891	NA	0.102	0.004168487247	SE	EPB41	+	chr1:29060421-29060484	NA	-0.303	0.01397229194	SLC27A5
SE	FNBP1	-	chr9:129923843-129924026	NA	0.282	0.004212179557	SE	PRKRIP1	+	chr7:102397619-102397698	NA	-0.122	0.0140171851	SLC29A1
SE	TPCN2	+	chr11:69079833-69079883	NA	0.302	0.004265354996	SE	BCL2L11	+	chr2:111123732-111123869	NA	-0.111	0.01404100243	SLC2A11
SE	RARS2	-	chr6:87548590-87548646	NA	0.101	0.004302882599	SE	SLC39A13	+	chr11:47413399-47413507	NA	-0.126	0.01405882068	SLC33A1
SE	FAM189B	-	chr1:155250275-155250821	NA	0.133	0.004324272947	SE	SHARPIN	-	chr8:144099080-144099205	NA	-0.192	0.01419024747	SLC35A2
SE	IL11RA	+	chr9:34660853-34660936	NA	0.131	0.004334562675	SE	LRP8	-	chr1:53272603-53272642	NA	-0.257	0.01439386568	SLC35A3
SE	CANT1	-	chr17:79000099-79000270	NA	0.441	0.004358181142	SE	REPS1	-	chr6:138926400-138926478	NA	-0.299	0.01444851823	SLC35B1
SE	ALPK1	+	chr4:112315800-112315852	NA	0.153	0.004367829994	SE	RNF181	+	chr2:85596856-85596931	NA	-0.235	0.01446539393	SLC35B3
SE	TTC28-AS1	+	chr22:27935150-27935230	NA	0.145	0.004416989367	SE	TBC1D7	-	chr6:13325093-13325174	NA	-0.194	0.01448293753	SLC35C2
SE	SLC26A6	-	chr3:48632244-48632396	NA	0.139	0.004427904581	SE	RNF157	-	chr17:76155251-76155317	NA	-0.122	0.0145371809	SLC35D2
SE	FAM86EP	-	chr4:3954959-3955033	NA	0.25	0.00443138389	SE	TMEM14B	+	chr6:10749621-10749698	NA	-0.238	0.0145521764	SLC35F5
SE	ZNF440	+	chr19:11830273-11830409	NA	0.23	0.00448074171	SE	B3GALNT1	-	chr3:161089959-161090062	NA	-0.113	0.01458009455	SLC37A3
SE	ZNF195	-	chr11:3361975-3362073	NA	0.117	0.004491791827	SE	MRPS28	-	chr8:80028694-80028796	NA	-0.387	0.01459665929	SLC38A6
SE	ZMYM1	+	chr1:35093319-35093441	NA	0.328	0.004503457102	SE	MIR22HG	-	chr17:1714370-1714453	NA	-0.418	0.01461642436	SLC38A9
SE	CCDC125	-	chr5:69294792-69294900	NA	0.188	0.004507877005	SE	DUXAP9	+	chr14:19064582-19064693	NA	-0.436	0.01476653649	SLC39A13
SE	SUZ12P1	+	chr17:30735032-30735097	NA	0.355	0.004564802919	SE	TRDMT1	-	chr10:17161482-17161548	NA	-0.166	0.01480028911	SLC3A2
SE	PPP3CB	-	chr10:73439871-73439901	NA	0.239	0.004570387656	SE	ENGASE	+	chr17:79083481-79083590	NA	-0.201	0.0149867324	SLC47A1
SE	IL17RC	+	chr3:9932819-9932858	NA	0.284	0.004594439537	SE	IMMP1L	-	chr11:31460625-31460714	NA	-0.299	0.01505945115	SLC4A1AP
SE	TMEM45A	+	chr3:100519461-100519608	NA	0.2	0.004619682531	SE	ZNF271P	+	chr18:35291009-35291232	NA	-0.134	0.01506849521	SLC4A3
SE	RALGPS1	+	chr9:126977694-126977745	NA	0.155	0.00469384981	SE	KIAA1211	+	chr4:56298213-56298349	NA	-0.234	0.01513160793	SLC4A7
SE	ZNF507	+	chr19:32347244-32347338	NA	0.214	0.004718480332	SE	SMUG1	-	chr12:54187818-54187905	NA	-0.206	0.01517451728	SLC50A1
SE	IKZF4	+	chr12:56024661-56024905	NA	0.279	0.004728450157	SE	TMEM44-AS1	+	chr3:194589473-194589626	NA	-0.4	0.01519053624	SLC52A2
SE	CTD-2583A14.1	+	chr19:57820493-57820549	NA	0.198	0.004759894371	SE	DEPDC1	-	chr1:68482045-68482897	NA	-0.205	0.01524058518	SLC6A8
SE	RANBP2	+	chr2:108762100-108762195	NA	0.158	0.00482716635	SE	FAM86B3P	+	chr8:8234482-8234584	NA	-0.64	0.01525821969	SLC7A11
SE	GIGYF2	+	chr2:232703423-232703489	NA	0.119	0.004832928255	SE	MBD1	-	chr18:50273333-50273426	NA	-0.161	0.01539580262	SLC9A5
SE	DHRS4-AS1	-	chr14:23953773-23954033	NA	0.213	0.004865530267	SE	CC2D2A	+	chr4:15479218-15479328	NA	-0.369	0.01564723304	SLC9B1
SE	TMEM87B	+	chr2:112074911-112074962	NA	0.152	0.004874200486	SE	SLC11A2	-	chr12:51006173-51006271	NA	-0.224	0.01585603976	SLC9B2
SE	CDK16	+	chrX:47222260-47222380	NA	0.28	0.004926977751	SE	TBL1X	+	chrX:9653544-9653689	NA	-0.276	0.01594697474	SLCO4A1
SE	SPOP	-	chr17:49636758-49636809	NA	0.119	0.004939913488	SE	METTL6	-	chr3:15414729-15414914	NA	-0.183	0.01601132017	SLCO4A1-AS1
SE	ZNF2	+	chr2:95176187-95176259	NA	0.249	0.00494140747	SE	CC2D2A	+	chr4:15479222-15479328	NA	-0.366	0.01617330057	SLCO5A1
SE	RFFL	-	chr17:35014739-35014763	NA	0.172	0.005094539813	SE	MOK	-	chr14:102231706-102231821	NA	-0.188	0.01617330354	SLK
SE	GPR157	-	chr1:9105485-9105680	NA	0.412	0.00512657653	SE	C8orf44	+	chr8:66677641-66677954	NA	-0.321	0.01632161566	SLMAP
SE	RPS6KB2	+	chr11:67432352-67432492	NA	0.222	0.00513160654	SE	ATAT1	+	chr6:30642767-30643011	NA	-0.236	0.01637108017	SLTM
SE	NPRL3	-	chr16:117300-117375	NA	0.206	0.005136856933	SE	CDK11A	-	chr1:1722587-1722634	NA	-0.123	0.01643350428	SMAP1
SE	THRB	-	chr3:24297225-24297371	NA	0.304	0.005335128453	SE	RP11-303E16.10	-	chr16:81087600-81087664	NA	-0.322	0.01648327918	SMARCA4
SE	KIAA1109	+	chr4:122226706-122226835	NA	0.29	0.005433588589	SE	POGLUT1	+	chr3:119478333-119478474	NA	-0.363	0.01651821476	SMARCAL1
SE	HAUS2	+	chr15:42558197-42558290	NA	0.134	0.005475061472	SE	TMEM161B-AS1	+	chr5:88283009-88283086	NA	-0.3	0.01655644229	SMARCC2
SE	KPNA5	+	chr6:116692054-116692156	NA	0.101	0.005599612143	SE	MDM1	-	chr12:68325440-68325575	NA	-0.31	0.01658132986	SMC4
SE	NSUN5P1	+	chr7:75410605-75410726	NA	0.256	0.005608000496	SE	ZSCAN32	-	chr16:3390009-3390133	NA	-0.279	0.01658132986	SMG7
SE	ZNF419	+	chr19:57490146-57490185	NA	0.322	0.005610157684	SE	RAD51D	-	chr17:35118500-35118619	NA	-0.325	0.01663502083	SMTN
SE	POLDIP3	-	chr22:42601969-42602056	NA	0.229	0.005634242534	SE	PPCDC	+	chr15:75044385-75044514	NA	-0.207	0.01663502083	SMUG1
SE	ARRB1	-	chr11:75271700-75271724	NA	0.161	0.005673378401	SE	PTK2	-	chr8:140948665-140948723	NA	-0.159	0.01676869911	SNAP23
SE	CACNB4	-	chr2:151860710-151860820	NA	0.251	0.005673378401	SE	KATNAL2	+	chr18:47046390-47046527	NA	-0.121	0.01677792067	SNAP47
SE	ENTPD5	-	chr14:73972883-73973024	NA	0.112	0.005732405631	SE	TMEM38B	+	chr9:105722533-105722621	NA	-0.24	0.01679686198	SNAPC5
SE	ADGRB2	-	chr1:31736572-31736723	NA	0.192	0.005745634186	SE	USP54	-	chr10:73504849-73504990	NA	-0.122	0.0168943985	SNAPIN
SE	KIAA2026	-	chr9:5933750-5933881	NA	0.125	0.005769454447	SE	TMEM62	+	chr15:43146492-43146634	NA	-0.341	0.01692620548	SNHG11
SE	IFT122	+	chr3:129461227-129461304	NA	0.398	0.005789128017	SE	GFM2	-	chr5:74738501-74738642	NA	-0.102	0.01693677111	SNHG14
SE	RHOQ	+	chr2:46543753-46543812	NA	0.264	0.005789128017	SE	IRAK4	+	chr12:43772911-43772986	NA	-0.137	0.01711610116	SNHG17
SE	FRS2	+	chr12:69486201-69486297	NA	0.184	0.005841165759	SE	SNX14	-	chr6:85567533-85567577	NA	-0.289	0.01711655461	SNHG3
SE	ERBIN	+	chr5:66072168-66072291	NA	0.439	0.005843897436	SE	NDUFAF7	+	chr2:37242634-37242693	NA	-0.166	0.01724334818	SNHG5
SE	CASP4	-	chr11:104951895-104952063	NA	0.144	0.005857179572	SE	PGAP2	+	chr11:3823612-3823740	NA	-0.434	0.01725172355	SNHG6
SE	SH2B1	+	chr16:28866011-28867033	NA	0.113	0.005860391112	SE	RHOT1	+	chr17:32209349-32209445	NA	-0.194	0.01725573761	SNHG8
SE	RAB34	-	chr17:28717212-28717593	NA	0.118	0.005954277431	SE	ZNF625-ZNF20	-	chr19:12145257-12146224	NA	-0.209	0.01755228438	SNRNP70
SE	PTPRS	-	chr19:5218786-5218798	NA	0.259	0.005967428301	SE	UROS	-	chr10:125789282-125789528	NA	-0.157	0.01755232888	SNRPG
SE	PQLC3	+	chr2:11172750-11172824	NA	0.284	0.005969129601	SE	NFIB	-	chr9:14102421-14102510	NA	-0.31	0.01760896452	SNRPN
SE	SEPT10	-	chr2:109585720-109585838	NA	0.12	0.006104791791	SE	IFT43	+	chr14:76076596-76076691	NA	-0.188	0.01764911282	SNU13
SE	RAB34	-	chr17:28717212-28717620	NA	0.115	0.006132731773	SE	ATXN3	-	chr14:92047895-92048089	NA	-0.328	0.01781677028	SNX10
SE	EEF2KMT	-	chr16:5097279-5097353	NA	0.221	0.006146579901	SE	CDC25C	-	chr5:138289479-138289563	NA	-0.118	0.01782449163	SNX11
SE	FAM49B	-	chr8:129904498-129904585	NA	0.157	0.006151912774	SE	MIATNB	+	chr22:26718139-26718231	NA	-0.307	0.01784243899	SNX14
SE	RP11-580I16.2	-	chr17:47626546-47626627	NA	0.524	0.006170354981	SE	UBQLN1	-	chr9:83666349-83666394	NA	-0.116	0.01785569141	SNX15
SE	TMEM117	+	chr12:44143524-44143624	NA	0.285	0.006170354981	SE	SPPL2A	-	chr15:50726072-50726126	NA	-0.135	0.01789049185	SNX19
SE	HDAC11	+	chr3:13496735-13496852	NA	0.252	0.006200467481	SE	RBM26-AS1	+	chr13:79417834-79417916	NA	-0.207	0.0179359004	SNX21
SE	DLGAP4	+	chr20:36465264-36465367	NA	0.113	0.006207750042	SE	ULK4	-	chr3:41915976-41916052	NA	-0.403	0.01803272989	SNX25
SE	RAB34	-	chr17:28717212-28717647	NA	0.114	0.006207750042	SE	MTRF1L	-	chr6:152991184-152991321	NA	-0.237	0.01806842543	SNX8
SE	COL12A1	-	chr6:75138319-75138340	NA	0.133	0.006341438047	SE	IMMP1L	-	chr11:31456259-31456386	NA	-0.126	0.01808880893	SOD1
SE	SLC9B1	-	chr4:102949256-102949427	NA	0.311	0.006378477914	SE	RP11-849H4.2	-	chr11:71925932-71926104	NA	-0.363	0.01809089575	SON
SE	ZNF195	-	chr11:3373576-3373656	NA	0.579	0.006463478574	SE	C3orf18	-	chr3:50565525-50565700	NA	-0.372	0.01820919836	SORBS1
SE	RMDN1	-	chr8:86484871-86484961	NA	0.114	0.006463478574	SE	GPR180	+	chr13:94619149-94619330	NA	-0.211	0.01825893441	SORBS3
SE	ZDHHC16	+	chr10:97448189-97448350	NA	0.309	0.006465468267	SE	HCG18	-	chr6:30314268-30314482	NA	-0.426	0.01832264645	SOS1
SE	C17orf62	-	chr17:82447579-82447621	NA	0.184	0.006498475566	SE	BCAT2	-	chr19:48807755-48808236	NA	-0.345	0.01860693698	SOX9-AS1
SE	ANO6	+	chr12:45302013-45302093	NA	0.148	0.006518544222	SE	POT1	-	chr7:124870910-124871041	NA	-0.104	0.01884693048	SP110
SE	ZBTB43	+	chr9:126808792-126808915	NA	0.229	0.006518544222	SE	C6orf52	-	chr6:10672546-10672636	NA	-0.505	0.01885208174	SP140L
SE	TBL2	-	chr7:73576540-73576701	NA	0.221	0.006525933172	SE	DDR1	+	chr6:30895403-30895514	NA	-0.192	0.01899892488	SPAG1
SE	EPB41	+	chr1:29060421-29060484	NA	0.14	0.006560213909	SE	RP11-345J4.5	-	chr16:29450111-29450276	NA	-0.448	0.01900449467	SPAG5
SE	ZNF485	+	chr10:43609254-43609350	NA	0.164	0.006739092745	SE	RP11-250B2.3	+	chr6:80454109-80454227	NA	-0.281	0.01900862158	SPAG9
SE	MYO19	-	chr17:36501851-36501901	NA	0.107	0.00682352165	SE	MVK	+	chr12:109579801-109579946	NA	-0.113	0.019011357	SPAST
SE	ZNF615	-	chr19:52007292-52007330	NA	0.242	0.006828710607	SE	LIPT1	+	chr2:99156300-99156426	NA	-0.342	0.01909479024	SPATA4

SE	TRIM41	+	chr5:181233412-181233435	NA	0.132	0.00683098948	SE	PREPL	-	chr2:44359496-44359750	NA	-0.217	0.01939041588	SPATA6L
SE	PFDN5	+	chr12:53296453-53296551	NA	0.125	0.006837178035	SE	LRRCC1	+	chr8:85109594-85109800	NA	-0.297	0.01949475042	SPATA7
SE	DRAM2	-	chr1:111122615-111122643	NA	0.241	0.006855151817	SE	MADD	+	chr11:47288967-47289027	NA	-0.124	0.01952163363	SPATS2
SE	FAM118B	+	chr11:126229224-126229336	NA	0.294	0.006913625577	SE	AP3S2	-	chr15:89877305-89877424	NA	-0.351	0.01954407125	SPATS2L
SE	LSM14B	+	chr20:62127584-62127701	NA	0.213	0.00694961065	SE	ZNF468	-	chr19:52853871-52854107	NA	-0.181	0.01962932789	SPEF2
SE	NUP214	+	chr9:131189052-131189131	NA	0.564	0.006949996165	SE	ZNF343	-	chr20:2486640-2486763	NA	-0.189	0.01973132719	SPEG
SE	AAK1	-	chr2:69495984-69496080	NA	0.316	0.006955259997	SE	CDK7	+	chr5:69254601-69254669	NA	-0.114	0.01973663004	SPG11
SE	TMEM63A	-	chr1:225866573-225866682	NA	0.112	0.006984479321	SE	ZMYM5	-	chr13:19851354-19851448	NA	-0.105	0.01973663004	SPG7
SE	FAM120B	+	chr6:170348150-170348323	NA	0.108	0.006986795451	SE	CMC1	+	chr3:28263290-28263380	NA	-0.137	0.01974238758	SPIN1
SE	SUPT20H	-	chr13:37048563-37048599	NA	0.207	0.006997668845	SE	TUG1	+	chr22:30972047-30972172	NA	-0.204	0.02023209143	SPIN3
SE	DGUOK	+	chr2:73950584-73950732	NA	0.101	0.007035617093	SE	CLEC2D	+	chr12:9693033-9693115	NA	-0.395	0.02024804826	SPIRE1
SE	IKZF4	+	chr12:56023670-56023764	NA	0.341	0.007045125414	SE	BPTF	+	chr17:67963332-67963506	NA	-0.201	0.02039831761	SPOP
SE	HCFC1R1	-	chr16:3023473-3023530	NA	0.28	0.007123014945	SE	FAM66C	+	chr12:8195276-8195412	NA	-0.396	0.02043424899	SPPL2A
SE	LINC00969	+	chr3:195685817-195685939	NA	0.268	0.007193032486	SE	LINC00963	+	chr9:129502396-129503139	NA	-0.244	0.02048193694	SPRY1
SE	FRS2	+	chr12:69537915-69537983	NA	0.102	0.007194573322	SE	NUP50	+	chr22:45170987-45171152	NA	-0.108	0.0205273931	SPTAN1
SE	ZNF485	+	chr10:43608613-43608740	NA	0.166	0.00723312539	SE	SUOX	+	chr12:55999332-55999458	NA	-0.483	0.02072133492	SRBD1
SE	TMEM87B	+	chr2:112077191-112077282	NA	0.121	0.007276760501	SE	GMIP	-	chr19:19634506-19634703	NA	-0.187	0.02076062402	SREBF1
SE	ZBTB25	-	chr14:64487113-64487196	NA	0.142	0.007295871206	SE	KIAA0141	+	chr5:141937197-141937357	NA	-0.104	0.02077964043	SREBF2
SE	CDC14B	-	chr9:96509672-96509789	NA	0.275	0.00744813648	SE	MEIS3	-	chr19:47415050-47415101	NA	-0.259	0.02092964191	SREK1
SE	TCEA1	-	chr8:54002891-54003086	NA	0.261	0.0075854768	SE	ARHGEF10L	+	chr1:17634547-17634562	NA	-0.27	0.02106431358	SRGAP1
SE	GLMP	-	chr1:156293319-156293429	NA	0.122	0.007592639129	SE	NUDT13	+	chr10:73125397-73125509	NA	-0.448	0.02109524081	SRRM1
SE	LTB4R2	+	chr14:24310170-24310255	NA	0.239	0.007647461604	SE	FAHD2A	+	chr2:95410526-95410586	NA	-0.306	0.0211341988	SRSF6
SE	ZNF383	+	chr19:37219598-37219749	NA	0.392	0.007705740173	SE	C8orf44	+	chr8:66676744-66676902	NA	-0.267	0.02133016112	SRSF7
SE	MYCBP2	-	chr13:77103208-77103310	NA	0.138	0.007740262278	SE	AMMECR1L	-	chr2:127871248-127871359	NA	-0.193	0.02137621621	SS18
SE	POLDIP3	-	chr22:42601969-42602107	NA	0.263	0.007773580358	SE	LTA4H	-	chr12:96003837-96003981	NA	-0.141	0.02140667551	SSBP1
SE	C1orf162	+	chr1:111477650-111477775	NA	0.327	0.007797388202	SE	TM7SF3	-	chr12:26980565-26980646	NA	-0.234	0.02164296605	SSBP4
SE	DIAPH2	+	chrX:96735757-96735790	NA	0.176	0.007803028724	SE	MICAL2	+	chr11:12249183-12249246	NA	-0.136	0.02165860992	SSH1
SE	PRPF4B	+	chr6:4062556-4062633	NA	0.151	0.007818435325	SE	ZDHHC16	+	chr10:97453798-97453846	NA	-0.151	0.02165860992	SSH2
SE	RAB34	-	chr17:28717212-28717398	NA	0.101	0.007840343391	SE	TOP3B	-	chr22:21970209-21970406	NA	-0.103	0.02168639396	SSX2IP
SE	TSEN2	+	chr3:12516610-12516661	NA	0.173	0.007851399504	SE	FAM192A	-	chr16:57175538-57175700	NA	-0.171	0.02184055867	ST20
SE	ARHGEF7	+	chr13:111209871-111210002	NA	0.205	0.007873662431	SE	CTD-2378E12.1	-	chr15:50890855-50891077	NA	-0.655	0.02204339488	ST3GAL5
SE	RCBTB2	-	chr13:48524658-48524757	NA	0.139	0.007914741791	SE	ST3GAL5	-	chr2:85870071-85870258	NA	-0.324	0.02209414026	ST3GAL6
SE	SUN1	+	chr7:848417-848615	NA	0.122	0.007925773942	SE	ZNF655	+	chr7:99562348-99562494	NA	-0.219	0.02239654989	ST3GAL6-AS1
SE	RIPK2	+	chr8:89762828-89762982	NA	0.303	0.007952215332	SE	ZNF814	-	chr19:57876915-57877042	NA	-0.234	0.02257980147	ST5
SE	SDCCAG8	+	chr1:243293398-243293494	NA	0.172	0.007959369707	SE	TOM1	+	chr22:35321958-35322019	NA	-0.112	0.02259035035	ST7L
SE	PLOD2	-	chr3:146077861-146077924	NA	0.18	0.007990153349	SE	CCDC189	-	chr16:30759341-30759724	NA	-0.199	0.02284834663	STAG2
SE	TIAL1	-	chr10:119580470-119580846	NA	0.251	0.008171877175	SE	EDC3	-	chr15:74687090-74687179	NA	-0.111	0.0231839421	STAG3L3
SE	CCDC92	-	chr12:123944271-123944364	NA	0.16	0.008185219315	SE	TCF3	-	chr19:1612206-1612433	NA	-0.134	0.02319335102	STAG3L4
SE	PUF60	-	chr8:143824312-143824396	NA	0.228	0.008185219315	SE	CCDC148	-	chr2:158345214-158345318	NA	-0.458	0.02324707802	STAG3L5P-PVRIG2P-PILRB
SE	FAM228B	+	chr2:24167626-24167683	NA	0.353	0.00823389498	SE	LIAS	+	chr4:39462195-39462289	NA	-0.12	0.02343194834	STAMBP
SE	IMMP1L	-	chr11:31460625-31460714	NA	0.426	0.00831961416	SE	EIF1AX	-	chrX:20132181-20132263	NA	-0.231	0.02363008235	STARD4
SE	MBTD1	-	chr17:51202021-51202077	NA	0.178	0.008345359488	SE	BAX	+	chr19:48955547-48955599	NA	-0.209	0.02365356143	STAT2
SE	LSM14B	+	chr20:62127584-62127701	NA	0.157	0.008345604047	SE	ANK3	-	chr10:60145967-60146021	NA	-0.18	0.02371629734	STAU1
SE	FGFR2	-	chr10:121565636-121565704	NA	0.539	0.008407926613	SE	LINC01128	+	chr1:850177-850351	NA	-0.51	0.02372588051	STIL
SE	DHX34	+	chr19:47376914-47376989	NA	0.308	0.008437561811	SE	GTF2IP1	-	chr7:75202541-75202613	NA	-0.111	0.0239620042	STIM1
SE	FAM189B	-	chr1:155253858-155253978	NA	0.24	0.008470168692	SE	TET2	+	chr4:105190359-105190505	NA	-0.155	0.02398359823	STK16
SE	CENPJ	-	chr13:24885605-24885670	NA	0.667	0.008553574934	SE	CCDC91	+	chr12:28225794-28225861	NA	-0.195	0.02409243924	STK19
SE	SIN3B	+	chr19:16862883-16862979	NA	0.164	0.008586619979	SE	AC004381.6	+	chr16:20813189-20813302	NA	-0.334	0.0241083796	STK40
SE	ZNF772	-	chr19:57475659-57475786	NA	0.322	0.008723427672	SE	WBP1	+	chr2:74459477-74459562	NA	-0.219	0.02421023616	STRADA
SE	C20orf194	-	chr20:3343675-3343731	NA	0.169	0.0087482388	SE	ZNF611	-	chr19:52711206-52711304	NA	-0.152	0.02423789603	STRN3
SE	MRM2	-	chr7:2238014-2238937	NA	0.164	0.008753276415	SE	ASAH1	-	chr8:18083980-18084286	NA	-0.213	0.02432113571	STRN4
SE	MAP3K8	+	chr10:30437175-30437406	NA	0.224	0.008761827026	SE	BICD1	+	chr12:32338785-32338979	NA	-0.249	0.02450190234	STX10
SE	MYT1	+	chr20:64223290-64223359	NA	0.213	0.008775297312	SE	OSBPL6	+	chr2:178230425-178230513	NA	-0.254	0.02472987573	STX16
SE	TLE2	-	chr19:3017839-3017859	NA	0.158	0.008775297312	SE	RP11-156P1.3	-	chr17:47033277-47033381	NA	-0.171	0.02472987573	STX16-NPEPL1
SE	ARHGEF1	+	chr19:41892024-41892123	NA	0.126	0.008891331548	SE	ZCCHC8	-	chr12:122483278-122483344	NA	-0.213	0.0247351972	STX2
SE	NT5C3A	-	chr7:33035933-33035988	NA	0.264	0.008891469354	SE	CFAP44	-	chr3:113333405-113333583	NA	-0.174	0.02483827348	STXBP1
SE	ITPR1	+	chr3:4653841-4653886	NA	0.351	0.008916593862	SE	UBAP2L	+	chr1:154269361-154269412	NA	-0.251	0.0249882848	STYXL1
SE	DEF8	+	chr16:89952561-89952682	NA	0.18	0.00897994775	SE	SPATS2L	+	chr2:200308886-200309090	NA	-0.459	0.02523595813	SUCO
SE	MELK	+	chr9:36594627-36594771	NA	0.142	0.008988988263	SE	USP28	-	chr11:113830866-113830943	NA	-0.126	0.02524154486	SUGP2
SE	SYNRG	-	chr17:37577379-37577613	NA	0.107	0.009170669219	SE	NFIC	+	chr19:3453762-3453916	NA	-0.197	0.02539293364	SUMF2
SE	EVI5L	+	chr19:7857091-7857124	NA	0.253	0.009226021974	SE	CLHC1	-	chr2:55228031-55228204	NA	-0.305	0.02539319847	SUN1
SE	BTN3A3	+	chr6:26443569-26443659	NA	0.229	0.009259011861	SE	BLOC1S5	-	chr6:8027206-8027399	NA	-0.176	0.02561488547	SUOX
SE	TGFB3	-	chr14:75960268-75960386	NA	0.128	0.009293494939	SE	AACS	+	chr12:125136661-125136864	NA	-0.11	0.02563440273	SUPT20H
SE	R3HDM4	-	chr19:901419-901546	NA	0.164	0.009304957146	SE	SLC25A40	-	chr7:87860071-87860169	NA	-0.144	0.02566581821	SUSD1
SE	CHN1	-	chr2:174945169-174945261	NA	0.138	0.009304957146	SE	FUT8	+	chr14:65430215-65430309	NA	-0.114	0.02578183739	SUV39H2
SE	TMEM218	-	chr11:125102131-125102317	NA	0.306	0.009312346136	SE	CDK7	+	chr5:69254601-69254669	NA	-0.131	0.02599262688	SUZ12P1
SE	IL17RC	+	chr3:9930027-9930149	NA	0.152	0.009316263501	SE	DHFR	-	chr5:80633876-80633992	NA	-0.241	0.02602011014	SVIL
SE	GOLGA6L9	+	chr15:82432571-82432631	NA	0.341	0.009416548364	SE	LINC00969	+	chr3:195689338-195689438	NA	-0.151	0.02605534879	SYNE1
SE	SYVN1	-	chr11:65132247-65132308	NA	0.167	0.009427282182	SE	NSRP1	+	chr17:30163066-30163162	NA	-0.453	0.02613073178	SYNE2
SE	FLT3LG	+	chr19:49480297-49480476	NA	0.21	0.009473724616	SE	TMEM161B-AS1	+	chr5:88285740-88285851	NA	-0.2	0.02616662861	SYNJ1
SE	RP11-499E18.1	-	chr4:102419696-102419794	NA	0.535	0.009473724616	SE	ARL15	-	chr5:54154579-54154639	NA	-0.151	0.02617319012	SYNRG
SE	DEF8	+	chr16:89952557-89952682	NA	0.177	0.009482340098	SE	ZNF655	+	chr7:99561887-99562494	NA	-0.14	0.02620422848	SYTL2
SE	TMBIM6	+	chr12:49742437-49742607	NA	0.143	0.009482340098	SE	CUTC	+	chr10:99750368-99750396	NA	-0.249	0.02620422848	SYTL4
SE	ASPM	-	chr1:197100430-197105185	NA	0.123	0.009515144024	SE	PPP2R5C	+	chr14:101917830-101917947	NA	-0.147	0.02623640526	SYVN1
SE	RNF146	+	chr6:127282286-127282395	NA	0.411	0.009704814921	SE	C11orf80	+	chr11:66822563-66822674	NA	-0.151	0.02626010702	SZRD1
SE	ANKMY1	-	chr2:240557189-240557352	NA	0.295	0.009947313719	SE	LCA5L	-	chr21:39428171-39428503	NA	-0.499	0.0264779633	SZT2
SE	PCGF6	-	chr10:103347237-103347297	NA	0.116	0.01001026167	SE	TARBP1	-	chr1:234463836-234463934	NA	-0.21	0.02650525741	TAB3
SE	NR4A1	+	chr12:52052473-52052645	NA	0.472	0.01007221058	SE	HEATR3	+	chr16:50072604-50072714	NA	-0.119	0.02655662668	TACC2
SE	ATP6V0B	+	chr1:43975664-43975848	NA	0.169	0.01015259463	SE	ZDHHC24	-	chr11:66526935-66527006	NA	-0.258	0.02660810651	TACC3
SE	TAPT1	-	chr4:16170652-16170729	NA	0.159	0.01024382028	SE	RNFT1	-	chr17:59960067-59960168	NA	-0.284	0.02664983959	TADA2A
SE	ATP5H	-	chr17:75039871-75040041	NA	0.283	0.01033447427	SE	CS	-	chr12:56298608-56298691	NA	-0.251	0.0267903489	TAF1
SE	DDHD1	-	chr14:53093315-53093444	NA	0.18	0.01034250695	SE	HP1BP3	-	chr1:20780344-20780540	NA	-0.131	0.0274504833	TAF1D
SE	ANKRD10	-	chr13:110894284-110894393	NA	0.459	0.01041046655	SE	TTLL3	+	chr3:9833103-9833273	NA	-0.192	0.0275139992	TAF2
SE	BBOF1	+	chr14:74046059-74046130	NA	0.302	0.01050857481	SE	ZNF559-ZNF177	+	chr19:9333014-9333113	NA	-0.265	0.0275869585	TALDO1
SE	SUSD1	-	chr9:112042100-112042165	NA	0.11	0.01062440517	SE	CCDC18-AS1	-	chr1:93318167-93318228	NA	-0.312	0.02759347156	TAMM41
SE	SLC4A1AP	+	chr2:27688699-27688767	NA	0.174	0.01068668249	SE	PRUNE1	+	chr1:151024610-151024795	NA	-0.144	0.02759566031	TANC1
SE	METTL26	-	chr16:635611-635774	NA	0.278	0.01074595393	SE	STARD4	-	chr5:111501961-111502088	NA	-0.133	0.02770700481	TANC2
SE	ZDHHC15	-	chrX:75429077-75429198	NA	0.334	0.01080928689	SE	TMEM67	+	chr8:93791881-93792004	NA	-0.114	0.02775592997	TANGO2
SE	ABCD4	-	chr14:74288206-74288259	NA	0.172	0.01087266728	SE	ATAD2	-	chr8:123400742-123401175	NA	-0.254	0.02776636468	TAPT1
SE	NEK3	-	chr13:52141019-52141069	NA	0.503	0.01092365486	SE	ULK4P2	+	chr15:30582893-30582957	NA	-0.462	0.02777981206	TARBP1
SE	HYKK	+	chr15:78513083-78513425	NA	0.122	0.01097352416	SE	ST3GAL6	+	chr3:98756368-98756540	NA	-0.151	0.02805536072	TARS2
SE	ATP11C	-	chrX:139737730-139737796	NA	0.111	0.01111677213	SE	SCMH1	-	chr1:41159724-41159933	NA	-0.234	0.02810714143	TATDN1
SE	ST3GAL6-AS1	-	chr3:98718423-98718521	NA	0.384	0.01134847022	SE	ZNF551	+	chr19:57685261-57685385	NA	-0.277	0.02831425901	TAX1BP1
SE	MRPS28	-	chr8:80028694-80028796	NA	0.205	0.01136970053	SE	CREM	+	chr10:35179133-35179276	NA	-0.135	0.02831425901	TAZ
SE	C14orf93	-	chr14:23000958-23001037	NA	0.336	0.01137520954	SE	FBXO22	+	chr15:75913202-75913290	NA	-0.115	0.02857103996	TBC1D23
SE	RAB34	-	chr17:28717212-28717647	NA	0.126	0.01139775805	SE	TLE1	-	chr9:81652213-81652288	NA	-0.133	0.02872169449	TBC1D31
SE	PIGQ	+	chr16:582247-582309	NA	0.229	0.01159964214	SE	BORA	+	chr13:72734959-72735005	NA	-0.114	0.02872793168	TBC1D32
SE	FGFR2	-	chr10:121565437-121565704	NA	0.411	0.01160808791	SE	PLD3	+	chr19:40365661-40365930	NA	-0.186	0.0290614872	TBC1D5
SE	FAM107B	-	chr10:14553321-14553387	NA	0.202	0.01165258035	SE	LETMD1	+	chr12:51058074-51058128	NA	-0.149	0.0290614872	TBC1D7
SE	TTLL3	+	chr3:9818554-9818920	NA	0.103	0.01165258035	SE	DIEXF	+	chr1:209842582-209842695	NA	-0.158	0.02912579184	TBC1D8B
SE	ISYNA1	-	chr19:18437598-18437760	NA	0.257	0.01175358981	SE	RBPJ	+	chr4:26362445-26362628	NA	-0.121	0.02915758566	TBC1D9B
SE	INTS3	+	chr1:153753700-153753818	NA	0.218	0.01176581464	SE	IKZF4	+	chr12:56023670-56023764	NA	-0.31	0.02968938387	TBL1X
SE	ABCD4	-	chr14:74299547-74299675	NA	0.244	0.01184031581	SE	USP28	-	chr11:113830866-113830946	NA	-0.125	0.0296972396	TBL1XR1
SE	CEP164	+	chr11:117363428-117363506	NA	0.215	0.01185604305	SE	CDKL3	-	chr5:134350248-134350427	NA	-0.32	0.02984907237	TBL2
SE	AHI1	-	chr6:135302711-135302817	NA	0.327	0.01187945836	SE	AC108488.4	-	chr2:3533107-3533190	NA	-0.211	0.02986419007	TBXAS1
SE	SLC26A6	-	chr3:48632244-48632337	NA	0.235	0.01192185245	SE	RP11-762I7.5	-	chr12:55815886-55815939	NA	-0.268	0.02987985873	TCAF1
SE	PPP2R3C	-	chr14:35109818-35109901	NA	0.112	0.01192661118	SE	FAHD2A	+	chr2:95410526-95410586	NA	-0.335	0.02993461557	TCAIM
SE	SETD5	+	chr3:9429826-9430051	NA	0.16	0.0119410651	SE	LCORL	-	chr4:17886067-17886161	NA	-0.2	0.03026946122	TCEA1
SE	TPK1	-	chr7:144681806-144681864	NA	0.484	0.01202735009	SE	CDCA7L	-	chr7:21911616-21911754	NA	-0.123	0.03029140932	TCEAL8
SE	PDCD2L	+	chr19:34404648-34404990	NA	0.19	0.01204751673	SE	LETMD1	+	chr12:51058005-51058128	NA	-0.143	0.03029140932	TCF20
SE	GPATCH8	-	chr17:44467096-44467114	NA	0.136	0.01205057511	SE	FAM175A	-	chr4:83470202-83470396	NA	-0.107	0.03069639954	TCF25
SE	TRAF3IP2-AS1	+	chr6:111574620-111574658	NA	0.317	0.01210955425	SE	TMEM161B	-	chr5:88197668-88197765	NA	-0.157	0.03072258172	TCF3
SE	C17orf62	-	chr17:82447579-82447646	NA	0.304	0.01212190181	SE	GUF1	+	chr4:44691665-44691799	NA	-0.139	0.03109214455	TCF4
SE	FAM192A	-	chr16:57178501-57178852	NA	0.15	0.01213319028	SE	CABIN1	+	chr22:24041138-24041273	NA	-0.101	0.03112960624	TCF7
SE	SLC23A2	-	chr20:4885820-4885909	NA	0.147	0.01214445282	SE	FAM208B	+	chr10:5709529-5709663	NA	-0.318	0.0313341614	TCF7L2
SE	ZDHHC16	+	chr10:97450356-97450536	NA	0.275	0.01221799436	SE	RP11-274B21.14	+	chr7:128579998-128580094	NA	-0.156	0.0314556154	TCOF1
SE	ZGRF1	-	chr4:112589723-112589874	NA	0.105	0.01226428593	SE	EEF1AKMT1	-	chr13:20731840-20732121	NA	-0.116	0.03161887069	TCTN1
SE	LRP8	-	chr1:53249379-53249556	NA	0.15	0.01237418479	SE	BAG6	-	chr6:31644306-31644414	NA	-0.101	0.03174855014	TCTN2
SE	CAMK2G	-	chr10:73818208-73818895	NA	0.221	0.01243956146	SE	TIMM23B	+	chr10:49943234-49943358	NA	-0.251	0.03179732384	TEAD1
SE	PRR3	+	chr6:30561327-30561508	NA	0.264	0.01244086474	SE	BRPF3	+	chr6:36225266-36225364	NA	-0.162	0.03182618393	TEAD2
SE	MBTD1	-	chr17:51193427-51193510	NA	0.119	0.01245768692	SE	ACSF3	+	chr16:89114338-89114487	NA	-0.173	0.03184982237	TEAD4

SE	SUN1	+	chr7:849519-849603	NA	0.204	0.01249196002
SE	ZFX	+	chrX:24152719-24152830	NA	0.404	0.01252426593
SE	ZNF142	-	chr2:218651700-218651822	NA	0.397	0.01252426593
SE	CROCCP2	-	chr1:16642766-16642850	NA	0.292	0.0125683966
SE	TTLL4	+	chr2:218727268-218727347	NA	0.133	0.01265510166
SE	PCBP4	-	chr3:51961748-51961894	NA	0.367	0.01272273568
SE	SLC29A1	+	chr6:44226060-44226167	NA	0.201	0.01275158129
SE	THRAP3	+	chr1:36225411-36225463	NA	0.218	0.01278202333
SE	TFIP11	-	chr22:26511118-26511317	NA	0.127	0.0127859952
SE	SSBP4	+	chr19:18425908-18426083	NA	0.143	0.01287323078
SE	GCSH	-	chr16:81084462-81084534	NA	0.376	0.01290998373
SE	PLK3	+	chr1:44804649-44804779	NA	0.219	0.01310929813
SE	CDK16	+	chrX:47222260-47222342	NA	0.245	0.01326260223
SE	GSAP	-	chr7:77353571-77353641	NA	0.111	0.01348537548
SE	CCDC7	+	chr10:32463016-32463049	NA	0.147	0.01351304657
SE	MTMR3	+	chr22:29970975-29971062	NA	0.133	0.01356089458
SE	ARMC4	-	chr10:27968922-27969018	NA	0.369	0.01357652802
SE	EXOSC10	-	chr1:11080498-11080514	NA	0.137	0.01358618517
SE	XRRA1	-	chr11:74930299-74930372	NA	0.246	0.01359356348
SE	PPP2R3C	-	chr14:35109818-35109847	NA	0.157	0.01371870462
SE	NOVA1-AS1	+	chr14:26797913-26798030	NA	0.122	0.01371870462
SE	PCBP4	-	chr3:51961748-51962088	NA	0.211	0.01378706426
SE	PDPR	+	chr16:70114820-70114930	NA	0.362	0.01384958618
SE	BCL2L12	+	chr19:49667018-49667161	NA	0.105	0.01389440019
SE	ZNF195	-	chr11:3361975-3362032	NA	0.142	0.01403856436
SE	SAMD4B	+	chr19:39351816-39352374	NA	0.354	0.01422507111
SE	MFSD6	+	chr2:190415291-190415413	NA	0.262	0.01422507111
SE	SREK1	+	chr5:66158808-66158932	NA	0.212	0.01430870942
SE	NQO2	+	chr6:3015529-3015643	NA	0.158	0.01432424694
SE	RUNX2	+	chr6:45545216-45545282	NA	0.331	0.01433428446
SE	CHD9	+	chr16:53319812-53319951	NA	0.176	0.01436473974
SE	PPP6R2	+	chr22:50391866-50392015	NA	0.121	0.01437782339
SE	SSH2	-	chr17:29848848-29848929	NA	0.284	0.01446539393
SE	TLK2	+	chr17:62523133-62523177	NA	0.139	0.01462858414
SE	KIAA1109	+	chr4:122325854-122325917	NA	0.27	0.01494049834
SE	NOMO2	-	chr16:18528012-18528071	NA	0.117	0.01502360744
SE	BEST1	+	chr11:61951770-61951958	NA	0.448	0.01511068212
SE	TRMO	-	chr9:97912872-97912975	NA	0.12	0.01516291276
SE	TCEA1	-	chr8:54002891-54002940	NA	0.315	0.01533786035
SE	NASP	+	chr1:45607320-45608337	NA	0.258	0.01542938513
SE	GPATCH2	-	chr1:217495014-217495075	NA	0.155	0.01558075653
SE	NQO2	+	chr6:3002049-3002286	NA	0.152	0.01579734626
SE	ZNF815P	+	chr7:5833526-5833568	NA	0.226	0.016092165
SE	SLC25A36	+	chr3:140966999-140967036	NA	0.166	0.01612752722
SE	SUOX	+	chr12:55997614-55997723	NA	0.141	0.01614921425
SE	TOP3B	-	chr22:21975980-21976187	NA	0.112	0.0164236481
SE	KIF16B	-	chr20:16335925-16336015	NA	0.107	0.016488075
SE	CD27-AS1	-	chr12:6450340-6450980	NA	0.27	0.01669192307
SE	C21orf62-AS1	+	chr21:32788611-32788701	NA	0.385	0.01673793337
SE	CD27-AS1	-	chr12:6450869-6450980	NA	0.313	0.01675843214
SE	EFCAB14	-	chr1:46688352-46688544	NA	0.149	0.01675843214
SE	PEX7	+	chr6:136866626-136866733	NA	0.108	0.01676038414
SE	PCNX2	-	chr1:233160282-233160433	NA	0.131	0.01676038414
SE	GOLGB1	-	chr3:121719645-121719768	NA	0.423	0.01706419273
SE	RPS28	+	chr19:8321655-8321703	NA	0.124	0.01756076189
SE	CCHCR1	-	chr6:31157357-31157500	NA	0.171	0.01760896452
SE	VPS13B	+	chr8:99507136-99507203	NA	0.175	0.01780311254
SE	ZNF133	+	chr20:18289898-18290074	NA	0.305	0.01781677028
SE	LUZP1	-	chr1:23109584-23109650	NA	0.253	0.017863027
SE	AC007040.11	-	chr2:70991829-70991983	NA	0.101	0.01868342172
SE	R3HDM1	+	chr2:135616151-135616193	NA	0.265	0.01888167559
SE	CCDC18	+	chr1:93183359-93183495	NA	0.179	0.01918166239
SE	PATJ	+	chr1:62100318-62100408	NA	0.5	0.01919460471
SE	USP33	-	chr1:77715741-77715868	NA	0.116	0.01924510814
SE	AGAP3	+	chr7:151120519-151120745	NA	0.19	0.01944727044
SE	SCPEP1	+	chr17:56998373-56998498	NA	0.128	0.01954785441
SE	March7	+	chr2:159715680-159715766	NA	0.241	0.01974476361
SE	PCBP4	-	chr3:51962809-51962892	NA	0.169	0.01989487174
SE	ERBB2	+	chr17:39719786-39719834	NA	0.122	0.02037278548
SE	TMEM234	-	chr1:32216888-32216947	NA	0.237	0.02050635879
SE	BEND6	+	chr6:57018420-57018557	NA	0.22	0.02050644615
SE	RBM5	+	chr3:50100531-50100605	NA	0.228	0.02074446813
SE	EED	+	chr11:86264171-86264263	NA	0.359	0.02081308966
SE	LRTOMT	+	chr11:72089416-72089596	NA	0.107	0.02085996974
SE	CDK10	+	chr16:89689251-89689324	NA	0.19	0.02101009346
SE	APLP2	+	chr11:130123611-130123779	NA	0.109	0.02102855911
SE	SYNE1	-	chr6:152145486-152145555	NA	0.343	0.02105099548
SE	RHOT1	+	chr17:32172936-32173060	NA	0.167	0.02106816711
SE	ZNF33B	-	chr10:42632294-42632439	NA	0.176	0.02109524081
SE	ZNF440	+	chr19:11830282-11830409	NA	0.165	0.02119091855
SE	TOX2	+	chr20:44065711-44066107	NA	0.576	0.02126672239
SE	CTC-444N24.6	-	chr19:57278115-57278182	NA	0.214	0.02156029198
SE	ARNTL2	+	chr12:27370128-27370230	NA	0.309	0.02166060778
SE	NBN	-	chr8:89981952-89982002	NA	0.266	0.02189479798
SE	AP2A2	+	chr11:1000431-1000598	NA	0.186	0.02196082209
SE	BAZ2B	-	chr2:159430862-159431156	NA	0.282	0.02202842779
SE	TTC8	+	chr14:88875025-88875109	NA	0.135	0.02217804804
SE	CENPJ	-	chr13:24886234-24886385	NA	0.102	0.02219822181
SE	ZNF195	-	chr11:3362545-3362602	NA	0.142	0.02225064706
SE	TMEM62	+	chr15:43138573-43138619	NA	0.119	0.02228420277
SE	FHL2	-	chr2:105396646-105396697	NA	0.59	0.02228946003
SE	MIA3	+	chr1:222662887-222663031	NA	0.106	0.02231683261
SE	MTMR11	-	chr1:149931259-149931426	NA	0.184	0.0223742272
SE	METTL25	+	chr12:82456726-82456820	NA	0.303	0.02240571123
SE	GORAB	+	chr1:170536309-170536421	NA	0.325	0.02241166849
SE	FAM227B	-	chr15:49611214-49611268	NA	0.104	0.02259035035
SE	ZNF444	+	chr19:56142275-56142386	NA	0.162	0.02259035035
SE	SLC35A3	+	chr1:99999260-99999415	NA	0.193	0.02279519203
SE	DENND5A	-	chr11:9143402-9143485	NA	0.105	0.02294288612
SE	CCNT2	+	chr2:134945786-134945982	NA	0.132	0.02298175873
SE	ZNF561-AS1	+	chr19:9627775-9627806	NA	0.149	0.02302160623
SE	WDR27	-	chr6:169664165-169664286	NA	0.149	0.02319335102
SE	ATAT1	+	chr6:30640534-30640603	NA	0.265	0.02320561358
SE	PCBP4	-	chr3:51961940-51962088	NA	0.125	0.02326580329
SE	CELF1	-	chr11:47500860-47500932	NA	0.237	0.02326580329
SE	FECH	-	chr18:57585863-57585973	NA	0.304	0.02354481015
SE	THAP9-AS1	-	chr4:82897988-82898062	NA	0.122	0.02355317783
SE	NR4A1	+	chr12:52052495-52052645	NA	0.417	0.02371629734
SE	LMBR1L	-	chr12:49106544-49106746	NA	0.175	0.02376495083
SE	INCENP	+	chr11:62141499-62141511	NA	0.323	0.02376597087
SE	BCL2L12	+	chr19:49667021-49667161	NA	0.145	0.02383511682
SE	TSEN2	+	chr3:12505153-12505231	NA	0.135	0.02398359823
SE	TAZ	+	chrX:154414100-154414190	NA	0.19	0.024007703
SE	NBPF1	-	chr1:16594930-16595009	NA	0.188	0.02406623634
SE	UBE2D3	-	chr4:102825238-102825317	NA	0.142	0.02414968727
SE	AGO3	+	chr1:36005718-36005858	NA	0.17	0.02438269561
SE	HYI	-	chr1:43452902-43453004	NA	0.24	0.02445759739
SE	CENPJ	-	chr13:24886233-24886385	NA	0.106	0.02450060409
SE	CD27-AS1	-	chr12:6450892-6450980	NA	0.337	0.02475961249
SE	TIAL1	-	chr10:119580470-119580538	NA	0.17	0.02493720771
SE	SSH1	-	chr12:108802321-108802368	NA	0.175	0.0250286001

SE	TMCO3	+	chr13:113520615-113520733	NA	-0.109	0.03199306257
SE	ZNF605	-	chr12:132948147-132948270	NA	-0.227	0.03204901676
SE	PDPR	+	chr16:70131301-70131419	NA	-0.126	0.03206756502
SE	SCMH1	-	chr1:41152598-41152741	NA	-0.202	0.03233185005
SE	TUBD1	-	chr17:59886082-59886230	NA	-0.11	0.03243601941
SE	CYP20A1	+	chr2:203246754-203246921	NA	-0.234	0.03260507664
SE	SLC24A1	+	chr15:65638127-65638181	NA	-0.326	0.0326161793
SE	SCLT1	-	chr4:128942995-128943188	NA	-0.136	0.03286776765
SE	ZNF655	+	chr7:99561887-99561973	NA	-0.183	0.03287761207
SE	ZCCHC11	-	chr1:52458335-52458449	NA	-0.13	0.03293364916
SE	GIGYF2	+	chr2:232722553-232722658	NA	-0.35	0.03294531518
SE	SCRN3	+	chr2:174397335-174397404	NA	-0.284	0.03300582981
SE	DUXAP9	+	chr14:19064582-19064693	NA	-0.478	0.03303492877
SE	RGS5	-	chr1:163248529-163248598	NA	-0.186	0.03328327983
SE	SDHAP1	-	chr3:195986518-195986604	NA	-0.178	0.03354692153
SE	PPCDC	+	chr15:75043440-75043536	NA	-0.124	0.03357411761
SE	ZNF655	+	chr7:99563193-99563266	NA	-0.177	0.03398103924
SE	UBA7	-	chr3:49809389-49809464	NA	-0.212	0.03434244633
SE	TTC8	+	chr14:88841424-88841514	NA	-0.273	0.03451558164
SE	TTLL11	-	chr9:122031722-122031856	NA	-0.221	0.03455480269
SE	ZNF619	+	chr3:40481862-40482016	NA	-0.294	0.0345748495
SE	GEMIN4	-	chr17:749813-749909	NA	-0.119	0.03462897539
SE	ZNF562	-	chr19:9658008-9658135	NA	-0.103	0.03489005793
SE	HHLA3	+	chr1:70366145-70366328	NA	-0.332	0.03495076767
SE	SETD5	+	chr3:9434044-9434181	NA	-0.169	0.03496582331
SE	TJP2	+	chr9:69251034-69251364	NA	-0.124	0.03536904157
SE	CIB2	-	chr15:78123704-78123739	NA	-0.171	0.03553450655
SE	IRAK4	+	chr12:43771219-43771365	NA	-0.229	0.03571655976
SE	C17orf80	+	chr17:73242243-73242351	NA	-0.198	0.03616841176
SE	LCAT	-	chr16:67940736-67940831	NA	-0.264	0.03618639737
SE	PSMG3-AS1	+	chr7:1579286-1579404	NA	-0.294	0.03625932837
SE	NFATC3	+	chr16:68221195-68221284	NA	-0.116	0.0364768483
SE	ZNF772	-	chr19:57476633-57476672	NA	-0.347	0.03664495534
SE	MINDY3	-	chr10:15816834-15816915	NA	-0.114	0.03671992905
SE	DECR2	+	chr16:405512-405665	NA	-0.244	0.03712636787
SE	LTBP4	+	chr19:40616888-40617042	NA	-0.21	0.03749535962
SE	PDPR	+	chr16:70130422-70130544	NA	-0.119	0.03781600207
SE	LINC00963	+	chr9:129502396-129503139	NA	-0.268	0.03797039936
SE	PPP1R12B	+	chr1:202501858-202502005	NA	-0.611	0.03813114754
SE	NBPF11	-	chr1:148124898-148125001	NA	-0.3	0.03825624706
SE	MEF2A	+	chr15:99620890-99621080	NA	-0.166	0.03836748222
SE	SLC3A2	+	chr11:62871576-62871669	NA	-0.132	0.03843822808
SE	PPP1R12B	+	chr1:202443086-202443127	NA	-0.25	0.03843822808
SE	HERC2P9	+	chr15:28612058-28612173	NA	-0.119	0.0384989077
SE	NUP50	+	chr22:45166308-45166430	NA	-0.213	0.03850020803
SE	AP1S2	-	chrX:15833443-15833524	NA	-0.284	0.03860979484
SE	R3HCC1L	+	chr10:98186466-98186563	NA	-0.183	0.03879465813
SE	L3MBTL3	+	chr6:130049650-130049830	NA	-0.114	0.03879465813
SE	STK19	+	chr6:31972620-31972757	NA	-0.102	0.03881355157
SE	ZBTB21	-	chr21:42002349-42002503	NA	-0.3	0.03885696071
SE	PIGN	-	chr18:62163530-62163656	NA	-0.226	0.03897967352
SE	FANCA	-	chr16:89758576-89758705	NA	-0.158	0.03898426062
SE	GABPB2	+	chr1:151073038-151073157	NA	-0.108	0.03900299778
SE	CCDC7	+	chr10:32471063-32471230	NA	-0.41	0.03915855325
SE	C11orf80	+	chr11:66748378-66748517	NA	-0.19	0.03972173331
SE	SYTL2	-	chr11:85711112-85711232	NA	-0.188	0.04030865465
SE	PXN	-	chr12:120226838-120227117	NA	-0.27	0.04038676185
SE	DDX17	-	chr22:38489227-38489458	NA	-0.125	0.04076256011
SE	RFWD3	-	chr16:74664623-74664745	NA	-0.289	0.04080879163
SE	MARK2	+	chr11:63903985-63904147	NA	-0.336	0.04088100642
SE	NCOA2	-	chr8:70144641-70144848	NA	-0.19	0.0410268245
SE	CHRM3	+	chr1:239826852-239827081	NA	-0.298	0.04103024877
SE	TBXAS1	+	chr7:139782487-139782564	NA	-0.293	0.04145970596
SE	RASSF8-AS1	-	chr12:25947518-25947622	NA	-0.397	0.0415224856
SE	RP4-714D9.5	+	chr1:100043072-100043229	NA	-0.178	0.04156260787
SE	CEP57L1	+	chr6:109145218-109145381	NA	-0.161	0.04168586939
SE	ZSCAN32	-	chr16:3393648-3393814	NA	-0.136	0.04185356045
SE	PRR3	+	chr6:30558149-30558212	NA	-0.227	0.04197130637
SE	KYAT1	-	chr9:128846679-128846859	NA	-0.234	0.04202003307
SE	ZBTB24	-	chr6:109476762-109476930	NA	-0.224	0.0423681903
SE	DNAJC5	+	chr20:63931108-63931182	NA	-0.186	0.04246695346
SE	SLC20A2	-	chr8:42535171-42535335	NA	-0.177	0.04253479721
SE	FAM86FP	-	chr12:8235252-8235518	NA	-0.454	0.04253479721
SE	PSTK	+	chr10:122986299-122986375	NA	-0.104	0.04261741669
SE	AC091729.9	+	chr7:1162351-1162543	NA	-0.173	0.04292658489
SE	RPS27	+	chr1:153990913-153991034	NA	-0.186	0.04298664594
SE	ACOX1	-	chr17:75973624-75973785	NA	-0.152	0.04320406396
SE	MTMR9LP	-	chr1:32234035-32234301	NA	-0.18	0.04339536956
SE	CHTF8	-	chr16:69120106-69120208	NA	-0.143	0.04354642454
SE	NEMP1	-	chr12:57069233-57069306	NA	-0.112	0.043757897
SE	ELMOD3	+	chr2:85355554-85355784	NA	-0.315	0.04376813577
SE	VEZT	+	chr12:95256549-95256622	NA	-0.146	0.04376813577
SE	LINC00630	+	chrX:102839800-102839863	NA	-0.285	0.04393571592
SE	TBC1D5	-	chr3:17622647-17622765	NA	-0.131	0.0439700115
SE	GTF2IP12	-	chr4:119442157-119442217	NA	-0.254	0.04405182501
SE	PHYKPL	-	chr5:178224615-178224729	NA	-0.302	0.04422443977
SE	C2orf76	-	chr2:119340158-119340361	NA	-0.263	0.04431440655
SE	RPTOR	+	chr17:80925369-80925480	NA	-0.262	0.04458274527
SE	RAD51D	-	chr17:35116858-35117037	NA	-0.364	0.04505876384
SE	ZBTB38	+	chr3:141438242-141438365	NA	-0.146	0.04507777634
SE	ZC3H14	+	chr14:88601923-88602083	NA	-0.154	0.04519826484
SE	CCP110	+	chr16:19548514-19548600	NA	-0.181	0.04543028697
SE	PFKL	+	chr21:44305799-44305935	NA	-0.253	0.04584038807
SE	GUF1	+	chr4:44690716-44690860	NA	-0.155	0.04593373253
SE	SFXN2	+	chr10:102729318-102729394	NA	-0.106	0.04627864623
SE	THUMPD2	-	chr2:39768900-39769093	NA	-0.213	0.04630611355
SE	EEF2KMT	-	chr16:5091793-5091895	NA	-0.234	0.04630611355
SE	MBNL1	+	chr3:152446703-152446757	NA	-0.235	0.04669502336
SE	TRPC1	+	chr3:142743484-142743586	NA	-0.213	0.04709255475
SE	CHMP2B	+	chr3:87240322-87240453	NA	-0.102	0.04720535292
SE	ZCWPW2	+	chr3:28478813-28478931	NA	-0.318	0.04732471362
SE	ARAP1	-	chr11:72692752-72692785	NA	-0.131	0.04752424816
SE	ZNF680	-	chr7:64560945-64561273	NA	-0.153	0.04800831293
SE	ZNF180	-	chr19:44485035-44485157	NA	-0.207	0.04841923256
SE	PGAP2	+	chr11:3823616-3823740	NA	-0.337	0.04930931816
SE	CCDC57	-	chr17:82179026-82179189	NA	-0.265	0.04941263439
SE	ACAD10	+	chr12:111729805-111729956	NA	-0.186	0.04957365061
SE	CD27-AS1	-	chr12:6450473-6450708	NA	-0.232	0.0496313164
SE	UFD1L	-	chr22:19451877-19452000	NA	-0.188	0.04996473423
SE	ACAT1	+	chr11:108129102-108129201	NA	-0.17	0.04997449951

TECR
TEN1-CDK3
TEPSIN
TERF2
TET2
TEX15
TEX22
TEX30
TEX9
TFDP1
TFDP2
TFIP11
TFPI
TFR2
TFRC
TGFB3
THAP5
THAP6
THAP7
THAP9
THAP9-AS1
THNSL1
THOC2
THOC5
THOP1
THRAP3
THRB
THTPA
THUMPD2
THUMPD3-AS1
TIA1
TIAL1
TIAM1
TICRR
TIMM17B
TIMM21
TIMM23B
TIMM8B
TJAP1
TJP1
TJP2
TKFC
TKT
TLE1
TLE2
TLK1
TLK2
TM7SF3
TMA16
TMBIM6
TMCO3
TMEM116
TMEM117
TMEM126B
TMEM135
TMEM138
TMEM143
TMEM14B
TMEM161A
TMEM161B
TMEM161B-AS1
TMEM175
TMEM178A
TMEM18
TMEM184B
TMEM198B
TMEM208
TMEM214
TMEM218
TMEM219
TMEM222
TMEM230
TMEM234
TMEM237
TMEM241
TMEM25
TMEM254
TMEM259
TMEM260
TMEM267
TMEM268
TMEM38B
TMEM39A
TMEM44-AS1
TMEM45A
TMEM62
TMEM63A
TMEM63B
TMEM67
TMEM80
TMEM87B
TMEM94
TMEM9B
TMPO
TMTC4
TMUB2
TNC
TNFRSF12A
TNFRSF25
TNFSF12-TNFSF13
TNFSF13
TNIK
TNRC18
TNRC6A
TOGARAM1
TOM1
TOM1L1
TOM1L2
TOP1MT
TOP3B
TOPORS-AS1
TOX2
TPCN2
TPGS2
TPK1
TPM1
TPM2
TPM3
TPRKB
TPRN
TPT1

SE	NDUFC1	-	chr4:139297384-139297443	NA	0.122	0.02506932821	TPT1-AS1
SE	CARD8	-	chr19:48223718-48223868	NA	0.111	0.02523536706	TRABD
SE	CD99L2	-	chrX:150814861-150814936	NA	0.114	0.02523595813	TRABD2A
SE	SREBF2	+	chr22:41904534-41904732	NA	0.353	0.02530794582	TRAF3IP2-AS1
SE	RP11-295P9.3	+	chr10:13652845-13653148	NA	0.259	0.0255521065	TRAPPC13
SE	RALGAPA2	-	chr20:20396694-20396734	NA	0.421	0.0255521065	TRAPPC3
SE	ELMOD3	+	chr2:85371439-85371562	NA	0.326	0.02558045263	TRAPPC6B
SE	MIOS	+	chr7:7567606-7567688	NA	0.224	0.02561488547	TRDMT1
SE	TRMT1	-	chr19:13109914-13110001	NA	0.135	0.02576294352	TREX2
SE	PPIP5K2	+	chr5:103187313-103187376	NA	0.267	0.02576515749	TRIM16
SE	DENND5A	-	chr11:9142721-9143485	NA	0.117	0.02602112914	TRIM22
SE	CCDC91	+	chr12:28225794-28225861	NA	0.197	0.02611709641	TRIM33
SE	SEPT2	+	chr2:241317498-241317599	NA	0.488	0.02613073178	TRIM35
SE	RRP12	-	chr10:97424800-97424893	NA	0.17	0.02624280943	TRIM37
SE	HMGN5	-	chrX:81197705-81197778	NA	0.137	0.02630321839	TRIM41
SE	DCAF10	+	chr9:37857240-37857351	NA	0.214	0.02641179415	TRIM5
SE	SPOP	-	chr17:49668026-49668078	NA	0.119	0.02643844487	TRIM65
SE	SSX2IP	-	chr1:84671188-84671308	NA	0.154	0.02654969046	TRIM69
SE	CEBPZOS	+	chr2:37198493-37198672	NA	0.177	0.02655662668	TRIM9
SE	ACSS1	-	chr20:25015137-25015230	NA	0.146	0.02664983959	TRIQK
SE	AHI1	-	chr6:135297431-135297492	NA	0.127	0.02667616491	TRMO
SE	ZNF3	-	chr7:100077302-100077433	NA	0.173	0.02672533686	TRMT1
SE	HDHD2	-	chr18:47137234-47137296	NA	0.159	0.02675171691	TRMT2B
SE	SEC61A2	+	chr10:12155777-12155931	NA	0.189	0.02675707532	TRMU
SE	ULK3	-	chr15:74838151-74838173	NA	0.603	0.02679711654	TRPC1
SE	CD320	-	chr19:8305030-8305805	NA	0.291	0.02730924673	TSACC
SE	ARL16	-	chr17:81683012-81683126	NA	0.101	0.02738999534	TSC1
SE	SNHG11	+	chr20:38447929-38448093	NA	0.161	0.02738999534	TSEN2
SE	RBMS1	-	chr2:160282256-160282304	NA	0.217	0.02751345708	TSPAN4
SE	SEMA6C	-	chr1:151138315-151138406	NA	0.248	0.02759566031	TSSC4
SE	UAP1L1	+	chr9:137078049-137078254	NA	0.132	0.02820264394	TTC13
SE	OCIAD1	+	chr4:48831226-48831496	NA	0.132	0.02831425901	TTC23
SE	ACIN1	-	chr14:23089981-23090101	NA	0.233	0.02847082484	TTC25
SE	TMEM260	+	chr14:56593114-56593317	NA	0.108	0.02900749021	TTC28
SE	SPATA6L	-	chr9:4626425-4626555	NA	0.323	0.02903051114	TTC28-AS1
SE	TIAM1	-	chr21:31276731-31276908	NA	0.388	0.02905195337	TTC3
SE	UFD1L	-	chr22:19475067-19475100	NA	0.209	0.0290614872	TTC31
SE	CROCCP2	-	chr1:16643027-16643145	NA	0.19	0.0290614872	TTC38
SE	PGAP1	-	chr2:196892345-196892401	NA	0.204	0.02951502951	TTC7A
SE	IFT88	+	chr13:20589810-20589867	NA	0.139	0.02953166679	TTC8
SE	SNHG14	+	chr15:25111764-25111894	NA	0.173	0.02967749663	TTLL11
SE	RHOC	-	chr1:112705099-112705168	NA	0.157	0.02991615115	TTLL3
SE	DCAF1	-	chr3:51466805-51466876	NA	0.15	0.03019391919	TTLL4
SE	PQLC1	-	chr18:79919183-79919400	NA	0.234	0.03019391919	TTYH3
SE	PXMP2	+	chr12:132695883-132696046	NA	0.16	0.03033077178	TUBD1
SE	FIRRE	-	chrX:131795808-131796331	NA	0.578	0.03072258172	TUBGCP4
SE	KDM4C	+	chr9:7105401-7105500	NA	0.224	0.03074448238	TUBGCP6
SE	SH3YL1	-	chr2:260084-260702	NA	0.155	0.0307508689	TUG1
SE	KIAA1468	+	chr18:62279773-62279856	NA	0.309	0.03090604922	TVP23C-CDRT4
SE	PCGF3	+	chr4:732399-732498	NA	0.258	0.03122303962	TWF1
SE	MEIG1	+	chr10:14970247-14970546	NA	0.313	0.03122985027	TXLNA
SE	CDKL3	-	chr5:134359896-134360091	NA	0.194	0.03199306257	TXNRD1
SE	MRPL55	-	chr1:228108436-228108474	NA	0.184	0.03254129005	TYSND1
SE	ERMARD	+	chr6:169753286-169753372	NA	0.179	0.03259045842	U2AF1L4
SE	PLSCR1	-	chr3:146536539-146536565	NA	0.146	0.03266616291	U2AF1L5
SE	ZBTB21	-	chr21:42002896-42002961	NA	0.134	0.03287761207	UACA
SE	SEMA3D	-	chr7:85097804-85097965	NA	0.135	0.03294531518	UAP1L1
SE	IFT122	+	chr3:129458598-129458677	NA	0.346	0.03306288922	UBA1
SE	USP13	+	chr3:179681877-179682003	NA	0.101	0.03322861598	UBA2
SE	MIATNB	+	chr22:26693680-26693836	NA	0.193	0.0333244848	UBA3
SE	C14orf79	+	chr14:104989631-104989707	NA	0.313	0.03336016912	UBA7
SE	TMEM161A	-	chr19:19134783-19134887	NA	0.228	0.03346111353	UBAP2L
SE	C17orf53	+	chr17:44150960-44151044	NA	0.173	0.03360945903	UBE2D3
SE	RECQL	-	chr12:21501169-21501215	NA	0.14	0.03400412644	UBE2E1
SE	CDKL3	-	chr5:134319357-134319497	NA	0.137	0.03403990512	UBE2F
SE	ZFX	+	chrX:24161714-24161842	NA	0.357	0.03417336386	UBE2G2
SE	GRAMD4	+	chr22:46676599-46676668	NA	0.121	0.0341951532	UBE2Q2
SE	CA5B	+	chrX:15745630-15745710	NA	0.117	0.0345544535	UBE2Q2P2
SE	PLA2G6	-	chr22:38125638-38125691	NA	0.237	0.03472346189	UBE2W
SE	ETNK2	-	chr1:204134514-204134694	NA	0.161	0.03472346189	UBE3A
SE	IMMP1L	-	chr11:31456259-31456386	NA	0.156	0.03497307275	UBL7-AS1
SE	NME4	+	chr16:398207-398385	NA	0.28	0.0349971465	UBP1
SE	TNRC18	-	chr7:5329936-5329969	NA	0.341	0.03499961163	UBQLN1
SE	TTC23	-	chr15:99245388-99245510	NA	0.187	0.03499961163	UBR3
SE	PIGT	+	chr20:45416516-45418979	NA	0.135	0.03536904157	UBTF
SE	BAIAP2	+	chr17:81036862-81036961	NA	0.133	0.03537587177	UBXN1
SE	FAM66C	+	chr12:8187731-8188225	NA	0.229	0.03557962837	UBXN2B
SE	ARHGAP23	+	chr17:38498413-38498510	NA	0.185	0.03589513837	UBXN4
SE	TPCN2	+	chr11:69067502-69067605	NA	0.119	0.03637629393	UCKL1
SE	THAP9-AS1	-	chr4:82897988-82898062	NA	0.201	0.03668129886	UFD1L
SE	PUF60	-	chr8:143824312-143824399	NA	0.166	0.0374094293	UGGT1
SE	HNRNPL	-	chr19:38841612-38841682	NA	0.156	0.03764037285	UGGT2
SE	TARBP1	-	chr1:234400852-234400948	NA	0.192	0.03775197242	UGP2
SE	RP11-421L21.3	+	chr1:101077362-101077508	NA	0.196	0.03803544067	UHRF2
SE	LINC01021	+	chr5:27477638-27477801	NA	0.121	0.03820251916	ULBP2
SE	IGFLR1	-	chr19:35740379-35740564	NA	0.271	0.03824379223	ULK3
SE	MAST1	+	chr19:12865315-12865481	NA	0.28	0.03825624706	ULK4
SE	DNAJC4	+	chr11:64231031-64231147	NA	0.445	0.03833779699	ULK4P2
SE	RP11-1212A22.	-	chr16:18372577-18372854	NA	0.263	0.03838602055	UNC50
SE	BBX	+	chr3:107789786-107789876	NA	0.148	0.03881092694	UNKL
SE	SGK494	-	chr17:28612261-28612366	NA	0.113	0.03885696071	UPF3A
SE	N4BP2	+	chr4:40100035-40100126	NA	0.335	0.03898426062	UPP1
SE	FHOD3	+	chr18:36687127-36687178	NA	0.331	0.0391410956	UPRT
SE	PRDM5	-	chr4:120754552-120754638	NA	0.142	0.03926733927	URB1
SE	TKFC	+	chr11:61335839-61335937	NA	0.113	0.03969952441	URB2
SE	TMEM218	-	chr11:125102131-125102302	NA	0.343	0.03972173331	URI1
SE	CTBP2	-	chr10:125110989-125111093	NA	0.106	0.04007048318	UROS
SE	CDC16	+	chr13:114236644-114236699	NA	0.131	0.04044128526	USP13
SE	GOLGA2	-	chr9:128272784-128272865	NA	0.197	0.04068278322	USP15
SE	WDR54	+	chr2:74422119-74422375	NA	0.2	0.04089304347	USP21
SE	INTS6	-	chr13:51423029-51423090	NA	0.181	0.04127709796	USP25
SE	PPOX	+	chr1:161167370-161167486	NA	0.156	0.04131087203	USP28
SE	GABBR1	-	chr6:29627485-29627646	NA	0.158	0.0415224856	USP32P3
SE	EPS15	-	chr1:51459040-51459148	NA	0.216	0.04158645599	USP33
SE	ZNF786	-	chr7:149080590-149080717	NA	0.354	0.04214164797	USP36
SE	DHRS1	-	chr14:24291555-24291625	NA	0.126	0.04221843917	USP3-AS1
SE	NEK7	+	chr1:198292944-198293039	NA	0.196	0.04223369146	USP45
SE	NMNAT3	-	chr3:139637962-139638062	NA	0.251	0.0425199426	USP53
SE	PHC1	+	chr12:8936855-8936964	NA	0.372	0.04253479721	USP54
SE	ESCO2	+	chr8:27783999-27784057	NA	0.118	0.04261741669	USP8
SE	SP110	-	chr2:230216780-230216821	NA	0.134	0.04267665388	VAPB
SE	SVIL	-	chr10:29535988-29536069	NA	0.202	0.04292658489	VCAN
SE	ESCO2	+	chr8:27780173-27780267	NA	0.105	0.04297087789	VEGFA
SE	LRRC75A-AS1	+	chr17:16460892-16461245	NA	0.218	0.04305333938	VEPH1
SE	PCGF3	+	chr4:732443-732498	NA	0.204	0.04316493501	VEZT
SE	INF2	+	chr14:104715283-104715340	NA	0.294	0.04353087844	VKORC1
SE	GOLGB1	-	chr3:121719645-121719753	NA	0.427	0.04360660297	VLDLR
SE	ANO6	+	chr12:45228167-45228273	NA	0.332	0.04369154184	VPS13B
SE	ADGRL2	+	chr1:81987259-81987341	NA	0.144	0.0439700115	VPS28
SE	HPS5	-	chr11:18317750-18317907	NA	0.306	0.04407811637	VPS29
SE	NBPF1	-	chr1:16594930-16595013	NA	0.147	0.04422144556	VPS41

SE	KLHDC1	+	chr14:49728925-49729009	NA	0.363	0.04492330258
SE	TMEM143	-	chr19:48360071-48360176	NA	0.29	0.04513725149
SE	ACIN1	-	chr14:23071124-23071186	NA	0.194	0.04533534914
SE	CDKL3	-	chr5:134359896-134360091	NA	0.385	0.04552554741
SE	DOCK9	-	chr13:98825871-98825940	NA	0.149	0.04570926204
SE	SMUG1	-	chr12:54188514-54188596	NA	0.17	0.0459673085
SE	TCF20	-	chr22:42168608-42168736	NA	0.28	0.04608462819
SE	TFIP11	-	chr22:26511118-26511195	NA	0.144	0.04611867135
SE	DNM1	+	chr9:128240986-128241047	NA	0.22	0.04612861764
SE	DCTD	-	chr4:182916418-182916539	NA	0.153	0.0464612947
SE	RCN1	+	chr11:32103003-32103202	NA	0.332	0.04655359236
SE	PORCN	+	chrX:48512834-48512852	NA	0.154	0.04729117939
SE	SDCCAG3	-	chr9:136408998-136409067	NA	0.183	0.0474914878
SE	WARS	-	chr14:100375282-100375406	NA	0.185	0.04751030201
SE	PAQR3	-	chr4:78922140-78922421	NA	0.317	0.04751030201
SE	TMEM267	-	chr5:43479889-43479987	NA	0.131	0.04751393873
SE	NFIC	+	chr19:3456549-3456635	NA	0.109	0.04788326124
SE	HPS4	-	chr22:26466050-26466104	NA	0.314	0.04795517943
SE	ABCD4	-	chr14:74300149-74300268	NA	0.157	0.04822228261
SE	RCHY1	-	chr4:75491582-75491637	NA	0.129	0.04831464141
SE	SNX21	+	chr20:45840447-45840458	NA	0.535	0.04920193968
SE	ZFAND1	-	chr8:81717248-81717288	NA	0.224	0.04922594537
SE	CTNND1	+	chr11:57794009-57794081	NA	0.138	0.04953027008
SE	ZNF133	+	chr20:18297984-18298062	NA	0.116	0.04998063542

VPS53
VPS54
VPS8
VPS9D1
VRK3
VTI1B
WAC-AS1
WARS
WASF1
WASF3
WASH2P
WASHC2A
WASHC2C
WBP1
WBSCR22
WDFY3
WDPCP
WDR20
WDR24
WDR26
WDR27
WDR31
WDR35
WDR4
WDR45
WDR46
WDR54
WDR55
WDR6
WDR83
WDR89
WDR90
WEE2-AS1
WHAMM
WIPF1
WIPI1
WIPI2
WNK1
WRAP73
WSB1
WWP2
XIAP
XKR9
XPNPEP1
XPNPEP3
XPO1
XPO6
XRCC3
XRN1
XRRA1
YAF2
YAP1
YBX3
YDJC
YEATS4
YIPF1
YLPM1
YPEL5
YWHAZ
YY1AP1
ZBED3-AS1
ZBED5-AS1
ZBTB1
ZBTB14
ZBTB17
ZBTB21
ZBTB24
ZBTB25
ZBTB38
ZBTB43
ZBTB8OS
ZC2HC1A
ZC3H11A
ZC3H13
ZC3H14
ZC3H18
ZC3H7B
ZC3HAV1
ZCCHC11
ZCCHC8
ZCWPW1
ZCWPW2
ZDHHC15
ZDHHC16
ZDHHC17
ZDHHC24
ZDHHC4
ZDHHC6
ZEB1
ZFAND1
ZFAND2A
ZFAND5
ZFAND6
ZFAS1
ZFX
ZFYVE19
ZFYVE21
ZFYVE27
ZGRF1
ZHX3
ZKSCAN3
ZMAT1
ZMIZ1
ZMYM1
ZMYM5
ZMYND8
ZNF10
ZNF107
ZNF133
ZNF140
ZNF141
ZNF142
ZNF155
ZNF180
ZNF189
ZNF19
ZNF195
ZNF2
ZNF202
ZNF211
ZNF226

ZNF232
ZNF253
ZNF254
ZNF260
ZNF267
ZNF271P
ZNF277
ZNF283
ZNF286A
ZNF3
ZNF30
ZNF302
ZNF317
ZNF326
ZNF33B
ZNF341
ZNF343
ZNF37BP
ZNF383
ZNF384
ZNF391
ZNF419
ZNF440
ZNF444
ZNF468
ZNF480
ZNF484
ZNF485
ZNF506
ZNF507
ZNF517
ZNF519
ZNF551
ZNF559
ZNF559-ZNF177
ZNF561
ZNF561-AS1
ZNF562
ZNF569
ZNF571
ZNF582-AS1
ZNF584
ZNF592
ZNF596
ZNF605
ZNF611
ZNF615
ZNF619
ZNF625-ZNF20
ZNF641
ZNF654
ZNF655
ZNF664
ZNF667-AS1
ZNF678
ZNF680
ZNF684
ZNF692
ZNF7
ZNF706
ZNF711
ZNF740
ZNF75A
ZNF75D
ZNF76
ZNF761
ZNF772
ZNF778
ZNF786
ZNF789
ZNF814
ZNF815P
ZNF821
ZNF83
ZNF90
ZNRD1ASP
ZRANB2-AS2
ZRANB3
ZSCAN23
ZSCAN26
ZSCAN31
ZSCAN32
ZSCAN5A
ZSWIM7
ZSWIM8
ZUFSP
ZWINT

Supplementary Table 3: Exon skipping events in mesenchymal (MES) and proneural GSC samples from which events were randomly selected for PCR validations. Validated events are highlighted in blue.

Exon skipping events in MES GSCs		
geneSymbol	deltaPSI	FDR
WBP1	0.968	1.34E-13
SLC27A5	0.897	3.04E-05
ANKRD36	0.886	2.93E-08
XPNPEP3	0.881	5.32E-13
PCTP	0.874	0
ADGRG6	0.871	0
ARIH2	0.852	0
ZNF667-AS1	0.819	1.32E-10
TCF7L2	0.805	0.000153796721
FRG1BP	0.796	0
CCDC18-AS1	0.794	6.70E-06
TCAF1	0.792	1.72E-09
TWF1	0.789	4.38E-08
RP11-849H4.2	0.787	4.30E-07
EMC3-AS1	0.782	3.23E-08
HKR1	0.782	7.85E-07
LPP	0.778	1.83E-09
NRG2	0.776	0.000300464970
RAB18	0.763	6.19E-10
LINC02210	0.754	3.26E-07
C2orf74	0.743	1.09E-09
IST1	0.742	0.000233405659
DUSP18	0.738	1.14E-10
SERHL2	0.73	1.31E-09
SEPT2	0.718	0
HKR1	0.712	1.03E-06
APBB2	0.711	0
KIF13A	0.706	0
SPAST	0.697	5.59E-14
ADD3	0.694	0
FKTN	0.692	0
EHBP1	0.692	0
C2CD5	0.69	9.33E-06
FNTB	0.679	5.36E-09
APITD1-CORT	0.673	0.001998768924
OSBPL6	0.671	0.000564656959
PLA2G12A	0.67	7.20E-06
RGMB-AS1	0.667	2.10E-07
SLTM	0.667	1.22E-05
C14orf93	0.667	3.29E-05
RP13-968A2.1	0.667	0.000406792612
RP11-1228E12.1	0.667	0.001952422279
DYRK4	0.667	0.002780083212
ACACA	0.667	0.003589770542

Exon skipping events in PN GSCs		
geneSymbol	deltaPSI	FDR
ST20	1	0
SEPT9	1	0
ARIH2	1	0
PUDP	1	0
PUDP	1	0
PUDP	1	9.24E-13
TTC13	0.993	0
PIP5K1C	0.96	3.34E-13
SORBS1	0.934	3.34E-13
USP3-AS1	0.925	4.00E-12
CLIP1	0.924	2.89E-14
ANKAR	0.91	1.34E-13
SRGAP1	0.904	2.23E-09
TVP23C-CDRT4	0.897	0
BTN3A3	0.881	5.48E-12
RPS24	0.876	0
BTN3A3	0.872	0
ANKRD36	0.869	1.65E-11
RP4-798P15.3	0.86	9.70E-11
SYTL2	0.857	0
PCBP1-AS1	0.84	2.34E-11
ZNF83	0.838	6.02E-06
GSN	0.835	3.15E-06
DST	0.827	0
RAPGEF2	0.824	0
ATP2A1	0.824	1.09E-07
VPS9D1	0.817	0.002386013963
CLASP2	0.815	4.89E-12
FUBP1	0.814	7.18E-09
PLEKHA1	0.806	0
AGAP4	0.8	0.000114900623
RAPGEF6	0.797	0
GSN	0.788	5.86E-11
GULP1	0.787	0
SBF1	0.784	2.68E-09
PLEKHA1	0.778	0
SORBS1	0.777	9.52E-08
PPFIBP1	0.773	0
SLC9A5	0.773	3.90E-10
TLE2	0.769	3.07E-10
KIF21A	0.766	0
SYTL2	0.76	0
SPTAN1	0.759	0
MTTP	0.745	3.17E-09

CAST	0.662	2.11E-09	WDFY3	0.733	0
SYTL2	0.66	0	SPAG9	0.73	0
RNF146	0.658	4.86E-08	PRMT2	0.729	0
CTD-2378E12.1	0.655	0.02204339488	TMEM135	0.729	5.59E-06
DPH3	0.651	0	XRRA1	0.728	5.96E-06
SNHG14	0.65	2.78E-07	U2AF1L4	0.725	9.67E-13
RAI14	0.649	0	ZNF706	0.718	1.02E-09
MCOLN3	0.648	3.05E-06	MRPL43	0.71	6.77E-07
ZNF814	0.647	0.002347592929	CAST	0.698	0
FAM86B3P	0.64	0.01525821969	ZNF391	0.698	7.31E-07
OSBPL6	0.635	0	SORBS3	0.697	1.53E-06
RP11-33B1.1	0.635	0.000502483757	SUZ12P1	0.696	5.79E-08
RP11-421L21.3	0.634	2.30E-06	MICAL3	0.695	9.30E-11
KIAA1468	0.631	9.58E-12	MED24	0.695	1.34E-05
TMEM218	0.631	8.71E-05	NEXN	0.687	4.41E-07
NDUFB2	0.623	7.31E-06	RP11-274B21.1	0.682	1.63E-09
PAQR6	0.619	4.35E-07	CENPV	0.682	5.11E-08
TPT1-AS1	0.619	0.004168487247	SORBS1	0.681	1.03E-06
ICA1L	0.618	0.008958063124	HLA-L	0.674	0.000182498683
AC074286.1	0.616	6.59E-11	CENPJ	0.667	0.008553574934
TWF1	0.612	8.15E-05	MPPE1	0.665	2.32E-06
PPP2R3C	0.611	5.99E-06	U2AF1L4	0.662	2.54E-11
PPP1R12B	0.611	0.03813114754	MAP3K6	0.66	0.000183314615
CPT1B	0.61	4.70E-13	FHL2	0.659	1.87E-07
MROH8	0.609	0.001277328111	TMBIM6	0.658	3.62E-07
MRNIP	0.604	1.26E-05	PCBP1-AS1	0.657	0.003866796193
CLEC16A	0.601	0.001796423469	MYO18A	0.652	0
TEAD1	0.599	8.25E-14	PLD3	0.65	3.15E-06
RPL17-C18orf32	0.596	0	CACNB4	0.65	7.65E-06
NEDD1	0.596	3.39E-06	GPM6A	0.649	2.67E-10
ADGRG6	0.595	2.89E-14	NQO1	0.647	0
FDFT1	0.595	1.01E-05	C19orf60	0.647	1.65E-09
ANAPC10	0.592	0.006742072944	ABI1	0.644	0
NBR2	0.583	2.65E-07	RSRP1	0.639	0
USP32P3	0.582	1.10E-08	RBM5	0.637	0
IMMP1L	0.579	1.01E-07	SYNJ1	0.634	4.13E-11
TRIM16	0.577	0.006143326895	EFCAB13	0.634	3.12E-05
C14orf93	0.576	0.002308460508	PNISR	0.633	0
SDHAP2	0.572	0	RASSF8-AS1	0.633	2.67E-10
TCAF1	0.57	0.000133178287	VLDLR	0.626	0
ZNF571	0.57	0.002377389601	PHACTR4	0.626	9.42E-06
DAG1	0.562	2.12E-05	DNAJC7	0.624	2.54E-07
NDUFB2	0.561	1.31E-05	UBA3	0.622	0.001965233616
FAM86EP	0.559	5.49E-05	SNX21	0.621	2.35E-05
PPRC1	0.558	7.52E-13	YY1AP1	0.617	0
RSG1	0.557	5.59E-05	DGKH	0.615	4.55E-07
RP11-371E8.4	0.557	0.000823192753	CTD-2341M24.1	0.612	5.70E-05
CNOT2	0.556	8.05E-05	PAQR3	0.61	2.01E-05
SLCO4A1	0.556	0.000610301062	CASK	0.608	4.65E-08

**EXO1**	0.555	0	**GUSBP11**	0.608	8.54E-06
**ZNF189**	0.554	9.33E-10	**FUBP1**	0.606	5.11E-05
**MAX**	0.553	1.66E-10	**BCAS4**	0.605	0
**SOS1**	0.553	2.09E-06	**FNBP1**	0.604	0
**PLD3**	0.551	4.25E-06	**MXRA7**	0.604	0
**U2AF1L4**	0.55	1.56E-09	**TNIK**	0.604	3.94E-11
**WIPI2**	0.549	2.10E-05	**ULK3**	0.603	0.02679711654
**POLL**	0.548	0.001950407967	**ARL4A**	0.602	0
**RP11-705C15.2**	0.542	4.86E-07	**ZCWPW2**	0.602	4.72E-09
**TMEM161B-AS1**	0.54	1.95E-07	**TMEM161A**	0.602	4.20E-05
**ARHGEF9**	0.538	3.67E-09	**PPA2**	0.601	1.19E-07
**MORC2**	0.537	0.006121948866	**TNC**	0.601	0.000541520201
**COA1**	0.536	8.02E-12	**ANKRD12**	0.597	0
**C11orf65**	0.536	0.001578796476	**CSNK1G3**	0.597	5.04E-07
**CADM2**	0.535	5.93E-06	**SYTL2**	0.596	0.000166142762
**TMEM161B-AS1**	0.534	8.81E-13	**RALGAPA1**	0.594	8.12E-08
**FBXL12**	0.533	3.50E-05	**AP1G1**	0.594	0.00126477858
**LINC01001**	0.533	0.01082847443	**RPRD2**	0.591	0
**RWDD3**	0.532	1.07E-06	**ZNF584**	0.59	5.49E-08
**TMEM254**	0.532	0.000427698002	**TEAD4**	0.59	7.68E-06
**SUOX**	0.531	4.75E-05	**NME4**	0.59	0.000285155921
**CCDC150**	0.528	0.000138227630	**FHL2**	0.59	0.02228946003
**C1orf50**	0.527	3.15E-07	**ZNF584**	0.589	6.35E-08
**PHYKPL**	0.526	4.88E-07	**EMC3-AS1**	0.589	0.003046176652
**MRNIP**	0.526	0.000354435123	**FAM173B**	0.584	0
**ERBIN**	0.523	0	**SUZ12P1**	0.583	1.11E-06
**C3orf18**	0.523	0.000723779315	**NSFL1C**	0.582	0
**DENND4C**	0.52	0	**MGRN1**	0.582	1.44E-07
**CSAD**	0.52	0.000292367023	**NIN**	0.581	0
**RPL17**	0.518	0	**FGFR1OP2**	0.58	0
**RPL17**	0.518	1.66E-09	**AFDN**	0.579	4.69E-10
**RSRC2**	0.517	0.000124092158	**ZNF584**	0.579	3.95E-08
**NUDT13**	0.517	0.000847919809	**ZNF195**	0.579	0.006463478574
**GGT1**	0.515	3.61E-05	**CTNND1**	0.578	0
**DIAPH2**	0.514	0.000176928792	**FIRRE**	0.578	0.03072258172
**ANAPC16**	0.512	0	**PPIP5K2**	0.577	0.001101883147
**SGCE**	0.512	0	**EPB41L2**	0.576	5.39E-12
**IMMP1L**	0.512	3.81E-13	**TOX2**	0.576	0.02126672239
**STX16-NPEPL1**	0.512	0.000287498151	**MCTP1**	0.575	1.56E-08
**DYNLL1**	0.512	0.001232062067	**C9orf3**	0.575	0.000766624702
**CSAD**	0.51	0.000152454658	**PER3**	0.575	0.003807338828
**SLCO4A1**	0.51	0.006698640151	**MARK3**	0.574	0
**LINC01128**	0.51	0.02372588051	**WARS**	0.573	9.26E-05
**SHF**	0.508	0.000774571168	**TTC28**	0.572	6.78E-07
**ZNF611**	0.506	0.00374323208	**ELF2**	0.572	0.001701782054
**OPA1**	0.506	0.01205057511	**CTNND1**	0.57	0
**C6orf52**	0.505	0.01885208174	**RPS24**	0.57	5.37E-11
**FANCI**	0.504	3.29E-08	**STAG3L3**	0.569	0.000438379529
**RWDD3**	0.503	0.000293170732	**NUP214**	0.564	0.006949996165

**AREL1**	0.502	0.001735749298
**HMGXB4**	0.501	0
**RP11-274B21.1[illegible]**	0.501	0.000902998005
**FAM86EP**	0.499	0.001505065008
**LCA5L**	0.499	0.0264779633
**GOSR2**	0.498	8.57E-05
**ELMOD3**	0.498	9.02E-05
**CLHC1**	0.498	9.57E-05
**AC004076.9**	0.498	0.000121420327
**MIOS**	0.497	0.000153038993
**NFRKB**	0.497	0.000185977085
**HSCB**	0.496	5.71E-10
**TRIM16**	0.495	0.002239049543
**RP11-156P1.3**	0.494	0.000788106904
**DTNB**	0.491	0.000853855443
**SDHAP1**	0.49	0
**RP11-705C15.2**	0.49	4.12E-05
**SEPT2**	0.489	0.000119680357
**DCLK2**	0.488	7.92E-10
**PBRM1**	0.487	0.002131254622
**GAB1**	0.485	1.31E-05
**SMARCC2**	0.483	0.000311022291
**SUOX**	0.483	0.02072133492
**PLCB4**	0.482	0.000163314841
**INVS**	0.479	0.00118875574
**HCG18**	0.478	1.30E-07
**PVR**	0.478	0.000204852622
**DUXAP9**	0.478	0.03303492877
**NHSL1**	0.476	0.007436656522
**SLC3A2**	0.475	0
**BPHL**	0.475	1.12E-05
**UBA2**	0.474	0.001456868059
**GCSH**	0.473	0.009167109226
**VCAN**	0.471	3.38E-05
**SHF**	0.471	0.001004510926
**TMEM241**	0.469	2.54E-05
**RP11-9G1.3**	0.468	2.41E-06
**LINC00630**	0.467	0.000207640945
**FAM228B**	0.463	0.000586634990
**BBS1**	0.462	0.000150335610
**LCA5L**	0.462	0.001839490346
**ULK4P2**	0.462	0.02777981206
**CLEC2D**	0.461	5.17E-05
**DHRS4L2**	0.461	0.000641805077
**EXO1**	0.46	1.86E-12
**CCDC15**	0.46	0.000150335610
**ARF4-AS1**	0.46	0.003887141779
**ZEB1**	0.459	2.43E-06
**MED23**	0.459	5.22E-06
**CCDC88A**	0.561	0
**CAST**	0.557	0
**AP1G1**	0.557	0
**WASH2P**	0.555	0.000350375615
**PIN4**	0.554	0
**SNX14**	0.554	0
**LIMCH1**	0.554	1.32E-12
**AMPD2**	0.554	0.000293523243
**ZNF484**	0.552	3.08E-05
**ZCWPW1**	0.552	3.10E-05
**C14orf166**	0.55	0.000144875579
**FAM126B**	0.549	5.59E-08
**EPB41L2**	0.548	4.47E-06
**C19orf60**	0.546	1.17E-08
**TMEM18**	0.545	0
**ITGB3BP**	0.545	0
**KIAA1191**	0.544	0.000153038993
**PPM1K**	0.544	0.000961670070
**CAST**	0.543	0.000103588851
**SLC7A11**	0.542	9.09E-08
**CACNB4**	0.542	3.46E-05
**FNBP1**	0.541	0
**ZCWPW1**	0.541	0.000882828750
**ASAP2**	0.539	6.77E-06
**FGFR2**	0.539	0.008407926613
**RNF146**	0.538	2.89E-08
**CEP72**	0.538	1.80E-07
**PHACTR4**	0.538	2.41E-06
**TSSC4**	0.535	0
**RP11-499E18.1**	0.535	0.009473724616
**SNX21**	0.535	0.04920193968
**WASF3**	0.534	6.92E-10
**FNBP1**	0.534	3.18E-05
**MARK3**	0.529	0.000491654262
**RAB3GAP1**	0.527	5.95E-05
**CDC14B**	0.526	1.71E-08
**PPIL3**	0.525	1.32E-06
**CCDC14**	0.525	0.002598953941
**PIGQ**	0.524	0.000241931653
**RP11-580I16.2**	0.524	0.006170354981
**TMEM18**	0.523	0
**MVD**	0.523	0.000142670014
**EEF1D**	0.522	1.10E-11
**FAM13B**	0.522	3.97E-08
**LRCH3**	0.521	5.20E-11
**NFYA**	0.521	5.31E-07
**MINDY3**	0.52	2.85E-13
**SEPT9**	0.519	4.46E-08
**CLSTN1**	0.516	0

SPATS2L	0.459	0.02523595813
PICALM	0.458	0
CCDC148	0.458	0.02324707802
CDKL3	0.456	7.45E-05
PRR34-AS1	0.455	7.54E-05
C8orf44	0.455	0.000933997588
ERBIN	0.454	0.000133179949
FAM86FP	0.454	0.04253479721
NISCH	0.453	0.000173761839
C14orf93	0.453	0.003140202336
NSRP1	0.453	0.02613073178
SPIN1	0.452	0.003349315801
CSAD	0.452	0.00576997794
MED12L	0.451	0.000624395879
ITPRIPL1	0.45	4.21E-05
DDR1	0.449	0.000141064512
SUOX	0.449	0.001493573318
NBPF8	0.448	4.38E-05
LRRC37A4P	0.448	0.000603975683
RP11-345J4.5	0.448	0.01900449467
NUDT13	0.448	0.02109524081
SIL1	0.447	4.68E-06
DDR1	0.447	0.000114666020
HERC2P9	0.447	0.001439456619
PARPBP	0.445	8.63E-06
RAD54B	0.445	0.00493556984
SMUG1	0.443	0.006943253512
ADGRG1	0.443	0.009505385205
EPB41L1	0.442	0.0111182907
CC2D1B	0.441	1.52E-05
TMEM234	0.441	0.000117787783
PVR	0.441	0.000296328986
AGPAT4	0.441	0.001023745599
DHFR	0.441	0.004239439132
CDK4	0.44	0.008685505951
WDR27	0.437	2.16E-05
TMEM161B-AS1	0.436	4.27E-09
PCBP1-AS1	0.436	2.42E-06
ATP11B	0.436	0.000113072913
DUXAP9	0.436	0.01476653649
C1RL	0.435	2.43E-05
PGAP2	0.434	0.01725172355
CLHC1	0.431	0.00013661978
STX2	0.43	0
IMMP1L	0.43	0
MACF1	0.428	5.56E-07
MRPS6	0.428	0.001796423469
PARD3	0.427	1.53E-12
HCG18	0.426	0.01832264645

FAM24B	0.515	1.61E-09
EVI5	0.515	0.000226627216
XPO6	0.514	1.03E-05
SNRPN	0.514	5.03E-05
TTC28-AS1	0.514	0.001921405105
EEF1D	0.512	4.25E-06
EEF1D	0.51	1.39E-05
TNFSF12-TNFSI	0.503	3.25E-06
NEK3	0.503	0.01092365486
MICAL3	0.5	1.06E-09
MAGI1	0.5	4.47E-07
PATJ	0.5	0.01919460471
ARL4A	0.499	7.00E-10
EEF1D	0.499	1.57E-08
ZNF277	0.497	0
EEF1D	0.497	1.12E-11
CTNND1	0.495	1.21E-11
MDM4	0.494	4.14E-05
RAD51L3-RFFL	0.493	0.001624246109
KIF3A	0.49	2.45E-09
SUN1	0.489	1.12E-05
RIF1	0.489	0.002957482326
AIFM1	0.488	0.003549029187
SEPT2	0.488	0.02613073178
GOLGA4	0.487	0
CENPU	0.484	0
TPK1	0.484	0.01202735009
PMS2CL	0.48	1.31E-10
TMEM25	0.48	0.002210906602
EEF1D	0.479	0.001367554607
ZNF19	0.476	0.001195407288
ARHGAP11B	0.474	0
EPB41L2	0.474	3.36E-07
DPM1	0.474	0.000332402615
DGKA	0.472	0.002122617065
NR4A1	0.472	0.01007221058
EEF1D	0.471	1.99E-05
DGKH	0.471	2.68E-05
MRPS15	0.47	0.003230017772
LRRC27	0.468	0.003432257142
ABCD4	0.467	0.000418686846
CATSPER2	0.467	0.002544829601
CUL5	0.466	0
TIA1	0.465	4.53E-11
UGGT2	0.462	1.74E-10
DNAJB6	0.461	0.000255290405
ATP11A	0.461	0.002993621228
GPCPD1	0.46	0.003724555554
GUK1	0.459	8.84E-08

**SEC24B**	0.425	2.17E-11
**APEH**	0.425	5.13E-08
**COA1**	0.425	1.83E-06
**LINC01125**	0.424	0.00448074171
**IKZF4**	0.422	0.002024138061
**FAM227B**	0.42	0.000671412157
**TPM1**	0.419	8.14E-10
**CA5B**	0.418	0.000115979367
**MIR22HG**	0.418	0.01461642436
**PSMA3-AS1**	0.417	1.58E-13
**NUMA1**	0.417	2.52E-09
**SLC25A29**	0.417	0.001127335634
**XRRA1**	0.417	0.005245556191
**SEPT6**	0.414	4.59E-07
**ENTPD1**	0.413	0.000150335610
**ARL13B**	0.413	0.009312346136
**ZNF789**	0.412	0.001102013063
**C1orf159**	0.411	0.00746974798
**PICALM**	0.41	0.004066478452
**CCDC7**	0.41	0.03915855325
**BMP2K**	0.409	4.44E-05
**COX20**	0.408	5.10E-07
**PARPBP**	0.408	7.60E-05
**SEPT6**	0.408	0.001889786218
**CCDC7**	0.407	0.00357102587
**CPNE3**	0.406	4.50E-07
**SEMA4F**	0.406	1.72E-06
**ING4**	0.405	0.001631986303
**DLEU2**	0.404	1.13E-06
**ZNF211**	0.403	0.002075918316
**KHK**	0.403	0.003344420368
**TRIM16**	0.403	0.01092216805
**ULK4**	0.403	0.01803272989
**DOCK7**	0.402	7.63E-12
**FOXM1**	0.4	0
**STX16**	0.4	4.53E-08
**TMEM44-AS1**	0.4	0.01519053624
**SNAPC5**	0.399	0.001312004339
**PARPBP**	0.397	1.97E-07
**RASSF8-AS1**	0.397	0.0415224856
**TRAPPC13**	0.396	1.37E-05
**OPA1**	0.396	0.002268274405
**FAM66C**	0.396	0.02043424899
**ACYP2**	0.395	0.000412618254
**NISCH**	0.395	0.001052296447
**CLEC2D**	0.395	0.02024804826
**NAP1L4**	0.394	6.18E-07
**TPM1**	0.394	0.000370694914
**FAM76B**	0.393	0.000260001515

**LUC7L2**	0.459	0.003536476151
**ANKRD10**	0.459	0.01041046655
**AP2M1**	0.458	0
**ZNF419**	0.458	0.001367971256
**MCTP1**	0.457	5.72E-11
**ARHGAP11B**	0.456	1.21E-06
**CIRBP**	0.456	1.72E-06
**USP45**	0.455	7.84E-09
**CACNB4**	0.455	0.000117862634
**PPIL3**	0.454	5.63E-11
**FAM221A**	0.453	0
**PFDN5**	0.453	0.000706638320
**APLP1**	0.448	3.77E-07
**BEST1**	0.448	0.01511068212
**SDR39U1**	0.447	2.11E-05
**FNIP2**	0.447	0.000114599384
**SEPT2**	0.447	0.001538786731
**PRKAR1A**	0.445	3.04E-05
**DNAJC4**	0.445	0.03833779699
**CCSER2**	0.444	0
**TEX22**	0.444	5.32E-13
**CLASP1**	0.444	9.89E-07
**CACTIN**	0.444	2.72E-05
**NADSYN1**	0.443	2.65E-05
**FAM135A**	0.442	1.50E-07
**JKAMP**	0.442	4.09E-07
**MAP4K4**	0.441	0
**CANT1**	0.441	0.004358181142
**VPS13B**	0.44	0.00206399437
**WDR27**	0.439	6.79E-07
**FBRS**	0.439	8.18E-06
**ERBIN**	0.439	0.005843897436
**XRRA1**	0.438	0.000133108710
**LRRC75B**	0.438	0.000475420331
**LETM2**	0.437	1.41E-06
**MED23**	0.436	3.90E-07
**BNIP2**	0.435	0
**PUF60**	0.435	2.28E-10
**ABHD14B**	0.435	4.64E-06
**CTNND1**	0.434	2.12E-08
**CTNND1**	0.434	2.44E-08
**RALGAPA1**	0.434	0.003894370242
**PSMA3-AS1**	0.432	7.31E-06
**WARS**	0.432	0.001631060496
**TTC7A**	0.432	0.004065365915
**TAZ**	0.431	7.20E-06
**NOD1**	0.431	0.000453765727
**CTNND1**	0.43	1.62E-11
**HPS1**	0.43	1.28E-05

**NEO1**	0.392	1.17E-08	**TSSC4**	0.429	1.03E-08
**ABCA11P**	0.39	0.000855021521	**MYL6**	0.427	0
**STX16**	0.389	3.14E-09	**CEP57L1**	0.427	0.000731961416
**NFIB**	0.389	0.000919138558	**C4orf36**	0.427	0.002784788117
**NME6**	0.388	0.001460213182	**GOLGB1**	0.427	0.04360660297
**KIF21B**	0.388	0.005890845208	**DBR1**	0.426	0
**PICALM**	0.387	0	**ZFAND5**	0.426	0
**MRPS28**	0.387	0.01459665929	**IMMP1L**	0.426	0.00831961416
**RWDD3**	0.386	0.000293305197	**QKI**	0.424	0
**FEZ2**	0.386	0.006549900596	**OFD1**	0.423	0
**TMEM260**	0.384	4.25E-06	**AC024560.3**	0.423	4.05E-12
**MAP4K4**	0.384	6.13E-05	**GOLGB1**	0.423	0.01706419273
**KB-1572G7.2**	0.382	0.002398430612	**ORMDL1**	0.422	0
**PROCA1**	0.381	0.000336973187	**SDR39U1**	0.422	1.80E-10
**CREB1**	0.381	0.001185968339	**RP11-51F16.8**	0.422	1.24E-08
**TAF1**	0.38	3.53E-07	**NAGK**	0.422	0.003548756426
**ANKAR**	0.38	6.03E-05	**MON2**	0.421	0.000963896981
**ZSCAN31**	0.38	8.71E-05	**RALGAPA2**	0.421	0.0255521065
**SGCE**	0.38	0.003512996048	**SEMA4F**	0.419	0.000441818321
**ATP5C1**	0.379	0.000431072153	**RAB34**	0.418	4.03E-06
**POLL**	0.378	4.71E-05	**HMGN1**	0.417	0
**ZNF680**	0.377	4.99E-05	**FAAP20**	0.417	6.06E-08
**ZNF253**	0.377	0.000181045047	**ELFN2**	0.417	0.001125297341
**XIAP**	0.377	0.001426975572	**NR4A1**	0.417	0.02371629734
**LTBP1**	0.376	0.008353634004	**PKIG**	0.416	0
**NME6**	0.375	7.94E-06	**PXN**	0.416	3.39E-06
**ELMOD3**	0.375	0.000118488895	**TMEM116**	0.416	1.88E-05
**SREBF1**	0.375	0.0013265261	**MBD1**	0.415	0.000907332278
**CCNI**	0.374	0.000776881723	**TCF7L2**	0.415	0.002988281976
**ENSA**	0.374	0.001478634489	**FBXO24**	0.414	6.11E-06
**NFIB**	0.374	0.002058724474	**SERPINB6**	0.414	0.000387914863
**ANAPC16**	0.373	2.52E-07	**MLLT6**	0.413	3.20E-06
**MGAT5**	0.373	0.01190796755	**HMGN1**	0.412	7.73E-13
**C3orf18**	0.372	0.01820919836	**GPR157**	0.412	0.00512657653
**RPS3A**	0.371	1.58E-06	**RNF146**	0.411	0.009704814921
**ZNF680**	0.37	6.13E-05	**FGFR2**	0.411	0.01160808791
**CC2D2A**	0.369	0.01564723304	**PILRB**	0.41	9.00E-11
**BDNF-AS**	0.368	0.00177508727	**NFASC**	0.41	2.42E-06
**ADGRL2**	0.368	0.004113255113	**SLC35B1**	0.41	0.000697169288
**ZSCAN31**	0.367	0.000114900623	**MPRIP**	0.41	0.00173447688
**MBD5**	0.366	0.000195634142	**KIAA1109**	0.408	1.95E-08
**RPL17**	0.366	0.000484349733	**JOSD2**	0.407	2.52E-07
**PGAP2**	0.366	0.001732186964	**C17orf62**	0.405	4.76E-07
**RTN2**	0.366	0.00365172218	**CDK5RAP2**	0.404	7.74E-05
**CC2D2A**	0.366	0.01617330057	**THAP6**	0.404	0.001289105061
**SGSM2**	0.365	1.48E-06	**ZFX**	0.404	0.01252426593
**ZC3H18**	0.365	0.001733304234	**MDM4**	0.403	4.41E-05
**GK**	0.365	0.002839945502	**HNRNPA2B1**	0.402	0
**RAD51D**	0.364	0.04505876384	**PHACTR4**	0.402	0.000288011465

**SETD9**	0.363	3.97E-05
**PLEKHM1P1**	0.363	0.002766188792
**SLC25A26**	0.363	0.00798304603
**POGLUT1**	0.363	0.01651821476
**RP11-849H4.2**	0.363	0.01809089575
**RP11-421L21.3**	0.362	1.04E-06
**PCNX1**	0.362	6.66E-05
**ANXA2**	0.362	0.006386827087
**KB-1572G7.2**	0.362	0.008304198588
**PRKRIP1**	0.361	2.37E-05
**MRPL52**	0.361	0.004738364736
**KIAA0586**	0.36	4.82E-05
**TWF1**	0.36	0.000148574459
**MRNIP**	0.36	0.01335046322
**RPS3A**	0.359	3.45E-09
**GSAP**	0.358	1.17E-05
**ARMCX5-GPRA**	0.358	0.000666033342
**UBE2Q2P2**	0.354	6.56E-05
**TCTN1**	0.353	0.005171587045
**NADK**	0.352	5.59E-14
**ZFAND6**	0.352	5.86E-11
**RALY**	0.352	0.000541806568
**DRAM2**	0.351	8.31E-06
**TOPORS-AS1**	0.351	0.001855130247
**AP3S2**	0.351	0.01954407125
**MAP4K4**	0.35	1.98E-11
**DENND4A**	0.35	1.22E-07
**TPM1**	0.35	0.002058724474
**GIGYF2**	0.35	0.03294531518
**PICALM**	0.349	0.000159697227
**YPEL5**	0.348	0
**PPRC1**	0.347	2.35E-13
**FAM76B**	0.347	2.25E-07
**ZNF772**	0.347	0.03664495534
**ZGRF1**	0.346	2.11E-06
**ESYT2**	0.346	0.001829980388
**DLEU2**	0.345	6.76E-07
**PIGN**	0.345	0.002929997565
**BCAT2**	0.345	0.01860693698
**CDC25C**	0.343	8.74E-06
**ADARB1**	0.343	2.24E-05
**BDNF-AS**	0.343	0.001406429329
**RP11-42I10.1**	0.342	6.74E-05
**FBXL2**	0.342	0.002142500663
**LIPT1**	0.342	0.01909479024
**TMEM62**	0.341	0.01692620548
**NADK**	0.34	2.89E-14
**PPA2**	0.34	1.28E-09
**CDKL1**	0.34	2.97E-05

**SNAP47**	0.402	0.00308346604
**TRMU**	0.401	0.000653105210
**TEX30**	0.4	4.43E-06
**UBA2**	0.399	5.38E-06
**PPP1R3E**	0.399	0.000150335610
**ZFYVE27**	0.398	5.08E-07
**IFT122**	0.398	0.005789128017
**PLEKHA8**	0.397	4.16E-05
**ATXN2**	0.397	5.72E-05
**ZNF142**	0.397	0.01252426593
**GUK1**	0.395	1.21E-08
**GLS**	0.395	0.000238932773
**ELOVL5**	0.395	0.003902246943
**BCL2L2**	0.394	1.28E-06
**ARHGAP11B**	0.392	2.89E-10
**SYTL4**	0.392	2.13E-05
**ZNF383**	0.392	0.007705740173
**ZNF684**	0.39	9.79E-05
**DUSP12**	0.39	0.001440110946
**PQLC1**	0.39	0.003043669782
**SNX25**	0.389	5.65E-12
**OXR1**	0.389	1.25E-11
**SEPT7**	0.389	8.87E-05
**MYO9A**	0.389	0.001802867873
**TIAM1**	0.388	0.02905195337
**EXOC7**	0.387	0
**FAAP20**	0.387	9.33E-08
**EPB41L2**	0.387	1.18E-05
**SNHG17**	0.386	7.64E-10
**C21orf62-AS1**	0.385	0.01673793337
**CDKL3**	0.385	0.04552554741
**CTAGE5**	0.384	9.02E-05
**ST3GAL6-AS1**	0.384	0.01134847022
**USP36**	0.382	2.27E-05
**CARM1**	0.381	2.60E-08
**STIM1**	0.381	6.19E-06
**PPM1K**	0.38	1.05E-06
**ZCWPW2**	0.377	4.60E-06
**RP11-315D16.2**	0.377	0.001966429415
**SVIL**	0.376	2.63E-06
**GCSH**	0.376	0.01290998373
**HMGN1**	0.375	7.19E-11
**ZNF519**	0.373	2.79E-06
**BCHE**	0.372	1.84E-13
**PHC1**	0.372	0.04253479721
**ABI1**	0.371	0
**SEPT7**	0.371	0.000802562723
**MARK3**	0.369	0
**CIRBP**	0.369	9.30E-07

**POLL**	0.34	0.01394848916
**BAG6**	0.338	1.58E-13
**LYRM1**	0.338	2.59E-05
**IQCB1**	0.338	0.003619441762
**SLMAP**	0.337	0
**RP4-614C10.2**	0.337	0.000398047305
**PGAP2**	0.337	0.04930931816
**MARK2**	0.336	0.04088100642
**MCM3**	0.335	0
**KCTD9**	0.335	0.007073635801
**FAHD2A**	0.335	0.02993461557
**CARD8**	0.334	6.60E-07
**CENPO**	0.334	5.09E-06
**KLHL7**	0.334	0.002563569625
**AC004381.6**	0.334	0.0241083796
**ADAMTS13**	0.333	9.27E-05
**SLC50A1**	0.332	1.70E-06
**HHLA3**	0.332	0.03495076767
**STAG3L4**	0.331	0.000965920658
**ANKDD1A**	0.33	5.01E-05
**KANK2**	0.329	0.00167889463
**ATXN3**	0.328	0.01781677028
**TMTC4**	0.327	1.64E-06
**POLR2J4**	0.327	0.001407114613
**SLC24A1**	0.326	0.0326161793
**ZC3H11A**	0.325	1.25E-06
**ADGRB2**	0.325	7.12E-06
**ZC3H14**	0.325	0.000154138242
**ST5**	0.325	0.000714324807
**POLL**	0.325	0.000994383049
**RAD51D**	0.325	0.01663502083
**CBY1**	0.324	0.001921405105
**ST3GAL5**	0.324	0.02209414026
**OSBPL6**	0.323	1.60E-09
**MRPS31P4**	0.323	0.000187247219
**KCTD9**	0.323	0.01287710281
**ZNF761**	0.322	1.76E-05
**RP11-303E16.1(**	0.322	0.01648327918
**ISCU**	0.321	0.000141830539
**CD27-AS1**	0.321	0.000758051464
**WIPF1**	0.321	0.004005348732
**C8orf44**	0.321	0.01632161566
**PTPMT1**	0.32	0
**LINC00969**	0.32	2.17E-06
**CDKL3**	0.32	0.02984907237
**FAM193B**	0.319	2.98E-05
**NEK6**	0.319	0.004357067677
**ADGRG1**	0.318	0.005039214886
**NEDD4**	0.318	0.01072761332
**ARMC4**	0.369	0.01357652802
**NUDT22**	0.367	3.01E-09
**ARHGAP21**	0.367	1.48E-07
**DCAF8**	0.367	3.58E-07
**GAA**	0.367	8.37E-06
**SH3GLB2**	0.367	0.000914633672
**PCBP4**	0.367	0.01272273568
**GSN**	0.366	1.92E-12
**MCTP1**	0.365	1.48E-08
**LAS1L**	0.365	2.58E-06
**ZNF277**	0.364	0
**STRADA**	0.364	9.33E-06
**DPY19L2**	0.364	0.001505065008
**RIN3**	0.363	9.41E-05
**SP140L**	0.363	0.000167484406
**KLHDC1**	0.363	0.04492330258
**MYO5A**	0.362	0
**ARIH2**	0.362	1.11E-06
**PDPR**	0.362	0.01384958618
**PICALM**	0.361	0
**FAM24B**	0.361	1.17E-08
**BORA**	0.361	0.000221708809
**NHLRC3**	0.36	5.79E-08
**PCBP1-AS1**	0.359	2.03E-06
**ASAH2B**	0.359	0.000381490862
**RTKN**	0.359	0.002527404038
**EED**	0.359	0.02081308966
**MCRIP2**	0.358	0.003349315801
**TEX15**	0.357	2.61E-05
**TTLL3**	0.357	0.000915025142
**ZFX**	0.357	0.03417336386
**KIF23**	0.356	4.47E-13
**NBEAL1**	0.355	0.000238314411
**SUZ12P1**	0.355	0.004564802919
**KLHL24**	0.354	7.54E-08
**SAMD4B**	0.354	0.01422507111
**ZNF786**	0.354	0.04214164797
**METTL26**	0.353	0.000414160831
**BROX**	0.353	0.00330261625
**FAM228B**	0.353	0.00823389498
**SREBF2**	0.353	0.02530794582
**ARHGEF11**	0.352	5.07E-07
**ITPR1**	0.351	0.008916593862
**TOP1MT**	0.35	1.38E-06
**TIA1**	0.349	1.08E-13
**C17orf62**	0.348	5.97E-10
**TANC2**	0.348	1.26E-07
**C19orf12**	0.347	0.000170947032
**NKTR**	0.346	6.05E-08

**FAM208B**	0.318	0.0313341614
**ZCWPW2**	0.318	0.04732471362
**TMEM161B-AS1**	0.317	2.86E-06
**CENPO**	0.316	2.58E-09
**PCNX1**	0.316	0.006891588316
**SLC4A7**	0.315	0.000246288097
**ELMOD3**	0.315	0.04376813577
**LINC00174**	0.314	0.000859083617
**MAN2B2**	0.313	0.000101047875
**CHEK1**	0.313	0.000281241984
**GK**	0.313	0.001523891106
**CDC25C**	0.312	1.94E-06
**AK2**	0.312	9.96E-06
**SMUG1**	0.312	0.000755532908
**CCDC18-AS1**	0.312	0.02759347156
**PARPBP**	0.311	0.000285160773
**LRP2BP**	0.311	0.01178140079
**ZBTB1**	0.31	6.58E-06
**MATR3**	0.31	0.004029235655
**FBF1**	0.31	0.01257062869
**MDM1**	0.31	0.01658132986
**NFIB**	0.31	0.01760896452
**IKZF4**	0.31	0.02968938387
**PSMA3-AS1**	0.308	1.18E-05
**STAG2**	0.307	0.000101047875
**MCAT**	0.307	0.000304133418
**ZNF655**	0.307	0.002332507155
**MIATNB**	0.307	0.01784243899
**ROBO1**	0.306	2.98E-07
**TMUB2**	0.306	0.000469433957
**FAHD2A**	0.306	0.0211341988
**CAPRIN2**	0.305	0.000240092465
**ZSCAN23**	0.305	0.000418220202
**CLHC1**	0.305	0.02539319847
**PLA2G12A**	0.304	1.31E-08
**DLGAP1-AS1**	0.304	4.07E-06
**FAM45A**	0.304	0.000180077800
**PPP4R1**	0.304	0.000788106904
**CRBN**	0.303	2.89E-14
**KIF21A**	0.303	2.13E-08
**GDAP2**	0.303	5.13E-05
**ODF2L**	0.303	0.000402847581
**VAPB**	0.303	0.004308658414
**MUTYH**	0.303	0.01134847022
**EPB41**	0.303	0.01397229194
**CLHC1**	0.302	5.10E-06
**ZNF76**	0.302	0.001129571315
**PPP1R12B**	0.302	0.001936037329
**SREBF1**	0.302	0.008461191506

**EEF1D**	0.346	5.02E-05
**MOK**	0.346	0.000589050278
**IFT122**	0.346	0.03306288922
**POLE2**	0.345	4.09E-07
**FAM161A**	0.345	5.12E-05
**KIF1B**	0.345	0.001701782054
**SYNE1**	0.343	0.02105099548
**TMEM218**	0.343	0.03972173331
**MRPS18C**	0.342	3.58E-13
**MCM9**	0.342	0.002662136025
**MARK3**	0.341	0.000688822865
**IKZF4**	0.341	0.007045125414
**GOLGA6L9**	0.341	0.009416548364
**TNRC18**	0.341	0.03499961163
**NPHP3**	0.34	1.09E-05
**TPCN2**	0.34	6.03E-05
**PPIL3**	0.339	1.93E-05
**FAM13B**	0.339	3.76E-05
**MFSD12**	0.339	4.34E-05
**CACNB4**	0.339	0.000462738566
**SCML1**	0.339	0.001379584947
**HMMR**	0.338	1.27E-11
**NF1**	0.338	2.85E-05
**QRICH1**	0.337	0.004077983224
**CD27-AS1**	0.337	0.02475961249
**KMT5C**	0.336	0.001968293186
**C14orf93**	0.336	0.01137520954
**TOM1L2**	0.335	6.07E-07
**RAD51AP1**	0.335	4.54E-05
**March2**	0.335	6.70E-05
**YIPF1**	0.335	0.000643276865
**N4BP2**	0.335	0.03898426062
**DCAF8**	0.334	4.67E-07
**PCOLCE**	0.334	0.000261928969
**PCBP1-AS1**	0.334	0.003387004345
**ZDHHC15**	0.334	0.01080928689
**MRPS28**	0.333	0.00106274245
**APTX**	0.332	7.79E-09
**PGAP2**	0.332	4.64E-06
**AGRN**	0.332	1.00E-04
**ANO6**	0.332	0.04369154184
**RCN1**	0.332	0.04655359236
**TEPSIN**	0.331	0.000116615585
**RUNX2**	0.331	0.01433428446
**FHOD3**	0.331	0.0391410956
**AURKB**	0.33	1.60E-09
**PCOLCE**	0.33	5.14E-05
**RP11-295P9.3**	0.33	0.003522034591
**DCAF8**	0.329	9.41E-05

**PHYKPL**	0.302	0.04422443977
**PRKRIP1**	0.301	0.000271826994
**EP400NL**	0.301	0.000748190390
**ZSCAN23**	0.301	0.000960374064

**ERMARD**	0.329	0.000145434513
**PRPF40B**	0.329	0.001138009614
**PYM1**	0.329	0.003959039809
**ACTR3B**	0.328	1.08E-13
**CCHCR1**	0.328	1.20E-06
**NAB1**	0.328	0.000145434513
**ZMYM1**	0.328	0.004503457102
**SREK1**	0.327	1.42E-06
**MTIF3**	0.327	0.000195921564
**C1orf162**	0.327	0.007797388202
**AHI1**	0.327	0.01187945836
**SEPT7**	0.326	0
**SLC25A36**	0.326	1.18E-10
**MSH5-SAPCD1**	0.326	3.62E-07
**ELMOD3**	0.326	0.02558045263
**PCMTD1**	0.325	1.42E-06
**GORAB**	0.325	0.02241166849
**THUMPD3-AS1**	0.324	4.80E-10
**PAQR3**	0.324	4.59E-08
**RFX2**	0.324	0.000309536167
**ARHGEF10**	0.324	0.000880675005
**HMGN1**	0.323	0
**ACAP2**	0.323	4.91E-13
**ILF3**	0.323	1.34E-11
**NLRX1**	0.323	8.79E-05
**NDEL1**	0.323	0.001234956404
**INCENP**	0.323	0.02376597087
**SPATA6L**	0.323	0.02903051114
**MPHOSPH9**	0.322	8.53E-11
**KLHL24**	0.322	0.000150335610
**PPIP5K2**	0.322	0.000206175873
**ZNF419**	0.322	0.005610157684
**ZNF772**	0.322	0.008723427672
**TMEM39A**	0.321	2.44E-10
**APTX**	0.321	6.31E-06
**SPATA4**	0.321	0.00010612589
**ZNF711**	0.32	7.32E-08
**SPEF2**	0.32	9.18E-05
**BAZ1A**	0.32	0.001379584947
**PEX7**	0.32	0.002238644159
**MAP4K4**	0.319	2.64E-09
**TSSC4**	0.319	5.66E-06
**SLC2A11**	0.319	9.42E-06
**TRIM16**	0.319	0.000782858272
**TOP1MT**	0.317	1.32E-09
**NSUN5P1**	0.317	0.003212828364
**TRAF3IP2-AS1**	0.317	0.01210955425
**PAQR3**	0.317	0.04751030201
**USP8**	0.316	7.79E-08

**ZNF75D**	0.316	0.002220951479
**AAK1**	0.316	0.006955259997
**SUMF2**	0.315	0
**MYO6**	0.315	3.49E-11
**GOLT1B**	0.315	5.26E-07
**PCBP1-AS1**	0.315	1.22E-05
**CCP110**	0.315	0.002784788117
**TCEA1**	0.315	0.01533786035
**BPTF**	0.314	9.73E-12
**SENP7**	0.314	1.18E-10
**PPIP5K2**	0.314	2.71E-06
**HPS4**	0.314	0.04795517943
**RBM6**	0.313	2.49E-06
**NUDT13**	0.313	0.000512763401
**GATAD2A**	0.313	0.00140484798
**RGS19**	0.313	0.003517135917
**CD27-AS1**	0.313	0.01675843214
**MEIG1**	0.313	0.03122985027
**C14orf79**	0.313	0.03336016912
**SLC29A1**	0.312	1.18E-05
**EEF1D**	0.311	0.000791141303
**PDPR**	0.311	0.000800021044
**SLC9B1**	0.311	0.006378477914
**PFDN5**	0.31	0
**TMEM126B**	0.31	4.34E-12
**PRPF40A**	0.309	0
**DST**	0.309	0
**C17orf62**	0.309	1.37E-07
**ZDHHC16**	0.309	0.006465468267
**ARNTL2**	0.309	0.02166060778
**KIAA1468**	0.309	0.03090604922
**TCEA1**	0.308	0.000990626250
**DHX34**	0.308	0.008437561811
**JKAMP**	0.307	2.33E-12
**PQLC3**	0.307	6.68E-05
**UBXN2B**	0.307	0.000146720567
**BORA**	0.306	2.29E-05
**TMEM218**	0.306	0.009312346136
**HPS5**	0.306	0.04407811637
**JKAMP**	0.305	1.06E-12
**RP11-452F19.3**	0.305	2.24E-05
**ZNF133**	0.305	0.01781677028
**SCML1**	0.304	5.26E-08
**THRB**	0.304	0.005335128453
**C17orf62**	0.304	0.01212190181
**FECH**	0.304	0.02354481015
**METTL26**	0.303	0.000438744160
**RIPK2**	0.303	0.007952215332
**METTL25**	0.303	0.02240571123

**FBXO44**	0.302	0.001244028305
**SPEG**	0.302	0.002168602909
**TPCN2**	0.302	0.004265354996
**BBOF1**	0.302	0.01050857481
**RBM6**	0.301	1.54E-10
**ULK4**	0.301	8.15E-05

Supplementary Table 4: Splicing events differing between mesenchymal (MES) and proneural (PN) GSCs, and between MES and PN GBM.

ASS (alternative splice site 5' or 3'): genomic coordinates of long and short exons (columns g.coord_1 and g.coord_2)
MXE (mutually exclusive exon):genomic coordinates of first and second exons (columns g.coord_1 and g.coord_2)
RI (retained intron): genomic coordinates of retained intron (column g.coord_1)
ES (exon skipping): genomic coordinates of skipped exon (column g.coord_1)

					GSCs		GBM	
**event.type**	**gene**	**strand**	**g.coord_1**	**g.coord_2**	**inc.level.difference**	**FDR**	**inc.level.difference**	**FDR**
ASS	FAXDC2	-	chr5:154823307-154823592	chr5:154823386-154823592	0.969	1.44E-12	-0.011	0.7552485584
ASS	FUZ	-	chr19:49812446-49812736	chr19:49812614-49812736	0.806	1.10E-09	0.002	0.03863816794
ASS	BTN3A3	+	chr6:26443366-26443442	chr6:26443381-26443442	0.734	1.43E-05	-0.006	1
ASS	INO80C	-	chr18:35487385-35487823	chr18:35487385-35487439	0.679	3.18E-10	0.039	1
ASS	PSMG4	+	chr6:3264173-3264325	chr6:3264208-3264325	0.676	6.14E-09	0.016	1
ASS	ULK3	-	chr15:74837750-74837798	chr15:74837756-74837798	0.665	0	0.006	1
ASS	STIL	-	chr1:47312947-47313156	chr1:47312947-47313021	0.653	0.002250382184	0.009	1
ASS	TSACC	+	chr1:156346767-156346995	chr1:156346778-156346995	0.653	0.04759067616	0.132	1
ASS	EXOC7	-	chr17:76090328-76090481	chr17:76090328-76090397	0.63	5.30E-08	-0.023	1
ASS	ZNF30	+	chr19:34926902-34927282	chr19:34926902-34927216	0.619	6.09E-07	-0.032	1
ASS	FUZ	-	chr19:49811362-49811491	chr19:49811362-49811467	0.594	0.03982276621	-0.034	6.05E-07
ASS	INPP4A	+	chr2:98555552-98555808	chr2:98555552-98555691	0.57	8.36E-05	0.108	5.73E-06
ASS	DAG1	+	chr3:49476848-49476905	chr3:49476848-49476900	0.565	0.008667503029	0.02	1
ASS	PHF20L1	+	chr8:132799082-132799172	chr8:132799094-132799172	0.563	0.0003262237303	-0.148	1
ASS	GPN3	-	chr12:110468125-110468264	chr12:110468195-110468264	0.556	3.93E-06	0.099	0.9966788445
ASS	QKI	+	chr6:163564666-163570145	chr6:163565945-163570145	0.545	3.14E-09	-0.038	0.001031669967
ASS	KIAA1109	+	chr4:122334343-122334545	chr4:122334343-122334440	0.543	0	-0.036	5.72E-06
ASS	RCAN3	+	chr1:24502896-24503150	chr1:24502896-24503117	0.534	0.01930630344	0.067	1
ASS	C21orf58	-	chr21:46314715-46315049	chr21:46314715-46314880	0.528	0.01015531981	-0.076	1
ASS	ATHL1	+	chr11:290657-291113	chr11:290677-291113	0.517	2.86E-06	-0.077	1
ASS	ITGA7	-	chr12:55700565-55701154	chr12:55700898-55701154	0.513	3.83E-06	-0.061	0.0449696624
ASS	C5orf45	-	chr5:179853196-179853437	chr5:179853377-179853437	0.513	0.003083787964	0.018	1
ASS	FUZ	-	chr19:49812446-49812736	chr19:49812637-49812736	0.508	1.43E-06	0.034	1
ASS	UBE2D3	-	chr4:102809067-102809184	chr4:102809067-102809159	0.505	0.01254382835	-0.003	1
ASS	CCHCR1	-	chr6:31157357-31157500	chr6:31157384-31157500	0.498	6.71E-06	-0.063	1
ASS	RASSF4	+	chr10:44983940-44984113	chr10:44984021-44984113	0.491	4.71E-05	0.036	1
ASS	GAS5	-	chr1:173865470-173865547	chr1:173865509-173865547	0.477	0	-0.009	1
ASS	ZCWPW1	-	chr7:100419001-100419189	chr7:100419110-100419189	0.476	0.0002101279767	-0.078	1
ASS	HERC2P9	+	chr15:28655272-28655691	chr15:28655272-28655420	0.469	0.0006755196922	-0.007	1
ASS	TCTN1	+	chr12:110647195-110647336	chr12:110647210-110647336	0.456	0.009968353808	0.059	1
ASS	ASB8	-	chr12:48150086-48150170	chr12:48150086-48150140	0.454	0.01183865018	0.054	1
ASS	ATG16L2	+	chr11:72821547-72821741	chr11:72821667-72821741	0.453	0.006062200051	-0.067	1
ASS	CBWD5	+	chr9:65732946-65733122	chr9:65732992-65733122	0.431	0.003471603937	-0.016	1
ASS	TCF4	-	chr18:55232508-55232671	chr18:55232520-55232671	0.431	5.01E-06	0.001	1
ASS	SMARCC2	-	chr12:56164302-56164731	chr12:56164647-56164731	0.43	1.46E-05	0.085	1.84E-11
ASS	PDLIM5	+	chr4:94575615-94576034	chr4:94575942-94576034	0.428	1.38E-06	0.04	0.676965413
ASS	ATP5O	-	chr21:33915135-33915724	chr21:33915651-33915724	0.422	1.59E-05	0.028	1
ASS	RAD9A	+	chr11:67393690-67393790	chr11:67393722-67393790	0.407	0.02636430401	0.002	1
ASS	CXorf23	-	chrX:19937417-19937532	chrX:19937504-19937532	0.403	6.40E-06	0.042	1
ASS	ATG16L2	+	chr11:72821198-72821741	chr11:72821667-72821741	0.398	0.00485576925	-0.073	0.5324033143
ASS	PROCA1	-	chr17:28704306-28704616	chr17:28704306-28704435	0.396	0.01603843118	-0.126	0.01850640356
ASS	SUV420H1	-	chr11:68171231-68171296	chr11:68171231-68171251	0.393	0.02228411796	0.017	1
ASS	PFN2	-	chr3:149964903-149966586	chr3:149964903-149966264	0.392	0	-0.026	0.07954730297
ASS	SNRPN	+	chr15:24974287-24974456	chr15:24974310-24974456	0.392	2.09E-08	0.002	0.9902235134
ASS	ATP11A	+	chr13:112881812-112887168	chr13:112881875-112887168	0.39	0.001121865795	0.057	1
ASS	PTBP2	+	chr1:96804784-96804939	chr1:96804799-96804939	0.389	0.03982276621	0.014	1
ASS	ABHD3	-	chr18:21663677-21664230	chr18:21664117-21664230	0.388	4.71E-07	0.001	0.235248041
ASS	GIT2	-	chr12:109947255-109948846	chr12:109947255-109947504	0.382	7.50E-05	0.036	0.1930077518
ASS	ERBB2	+	chr17:39694680-39695181	chr17:39694987-39695181	0.372	0.03581629335	0.113	1
ASS	FAM193B	-	chr5:177523158-177523265	chr5:177523162-177523265	0.368	0.004196039296	0.014	1
ASS	ENGASE	+	chr17:79081897-79082877	chr17:79081897-79082063	0.365	1.43E-06	0.016	1
ASS	CEP70	-	chr3:138572858-138572984	chr3:138572858-138572932	0.362	0.0004772283099	-0.021	0.609275072
ASS	VPS28	-	chr8:144425874-144426063	chr8:144425921-144426063	0.359	0.02683660206	-0.049	1
ASS	CNOT2	+	chr12:70294070-70294294	chr12:70294236-70294294	0.356	0.02871858636	-0.068	1
ASS	PAAF1	+	chr11:73886928-73887099	chr11:73887030-73887099	0.348	4.07E-05	-0.031	1

ASS	GSKIP	+	chr14:96379687-96379788	chr14:96379687-96379755	0.343	0.0001836014068	-0.047	1
ASS	FKBP1B	+	chr2:24063018-24063681	chr2:24063063-24063681	0.34	0.03680649749	-0.043	0.653044201
ASS	TTC31	+	chr2:74483321-74483563	chr2:74483321-74483410	0.336	0.006799172032	0.118	1
ASS	CACNA2D1	-	chr7:81997178-81997250	chr7:81997178-81997235	0.334	0.01823872704	-0.038	1
ASS	HDLBP	-	chr2:241269200-241269295	chr2:241269205-241269295	0.332	0.02387795607	0.068	1
ASS	SSBP1	+	chr7:141741748-141741798	chr7:141741748-141741792	0.33	0.0005479620032	0.133	1
ASS	GEMIN8	-	chrX:14026051-14026221	chrX:14026139-14026221	0.327	3.93E-07	-0.006	1
ASS	FAM111A	+	chr11:59148703-59148953	chr11:59148835-59148953	0.327	0.0004411870038	-0.017	1
ASS	ECI2	-	chr6:4117307-4119275	chr6:4117307-4117451	0.327	2.46E-12	0.032	1
ASS	ARL13B	+	chr3:93996547-93996684	chr3:93996562-93996684	0.325	0.001110868338	0.033	1
ASS	SERHL2	+	chr22:42571120-42571901	chr22:42571120-42571203	0.324	1.14E-05	-0.245	1.17E-12
ASS	CERS5	-	chr12:50132961-50134068	chr12:50132961-50133064	0.323	1.11E-05	0.078	0.8570516629
ASS	RAP1GAP	-	chr1:21597960-21598095	chr1:21597960-21598040	0.321	0.001576710913	-0.056	0.5037900999
ASS	BMP1	+	chr8:22201117-22201928	chr8:22201802-22201928	0.321	0.0003985755184	0.024	1
ASS	PANK4	-	chr1:2510677-2510782	chr1:2510677-2510748	0.32	0.004748089327	-0.004	1
ASS	MSTO1	+	chr1:155611688-155611852	chr1:155611688-155611827	0.319	0.001671111688	-0.063	1
ASS	AP1G2	-	chr14:23564045-23564221	chr14:23564045-23564159	0.319	0.0254485429	-0.111	0.0005465986558
ASS	SYNRG	-	chr17:37596222-37596344	chr17:37596222-37596341	0.311	4.07E-05	-0.067	1
ASS	SERGEF	-	chr11:18010070-18010139	chr11:18010074-18010139	0.309	0.02139215565	-0.06	1
ASS	HSF4	+	chr16:67167719-67167947	chr16:67167823-67167947	0.308	0.007648353669	0.027	1
ASS	DCUN1D4	+	chr4:51886567-51887244	chr4:51886567-51886638	0.307	0	-0.022	0.01679940389
ASS	ZNF740	+	chr12:53181676-53182190	chr12:53181676-53181992	0.305	4.35E-05	0.024	1
ASS	YY1AP1	-	chr1:155668626-155668777	chr1:155668626-155668717	0.304	8.73E-10	-0.043	0.0380951098
ASS	CLK4	-	chr5:178619837-178620673	chr5:178619837-178619896	0.302	4.40E-05	0.037	1
ASS	MOV10	+	chr1:112699457-112699810	chr1:112699684-112699810	0.302	2.09E-08	-0.031	0.003361713201
ASS	CPSF7	-	chr11:61421053-61421130	chr11:61421053-61421127	0.301	0.003195651006	0.054	1
ASS	OFD1	+	chrX:13752703-13753441	chrX:13753367-13753441	0.297	0.00566275157	-0.103	0.1720331179
ASS	RABL2B	-	chr22:50769424-50769549	chr22:50769454-50769549	0.296	0.03112961493	0.011	1
ASS	PLEKHH3	-	chr17:42672085-42672392	chr17:42672085-42672212	0.295	0.01358674745	0	1
ASS	CHD3	+	chr17:7909142-7909384	chr17:7909142-7909338	0.295	0.0002879218488	-0.038	0.02484334521
ASS	CDK11A	-	chr1:1707408-1707584	chr1:1707524-1707584	0.291	1.41E-10	0.091	0.0556495864
ASS	ARL16	-	chr17:81682442-81682675	chr17:81682490-81682675	0.291	0.0003991287134	0.034	1
ASS	RNF181	+	chr2:85597103-85597581	chr2:85597444-85597581	0.288	0.007889084592	0.049	0.0484292929
ASS	CTNND1	+	chr11:57761796-57762119	chr11:57761796-57762046	0.285	6.49E-09	0.009	0.02081041728
ASS	METTL22	+	chr16:8644528-8644725	chr16:8644556-8644725	0.283	2.96E-05	0.019	1
ASS	OCIAD1	+	chr4:48831226-48831433	chr4:48831226-48831398	0.283	0.03335973344	-0.027	1
ASS	PARP6	-	chr15:72265269-72265473	chr15:72265412-72265473	0.279	0	0	1
ASS	ZNF226	+	chr19:44165587-44165807	chr19:44165756-44165807	0.275	5.46E-06	-0.088	0.0002121826412
ASS	CHD3	+	chr17:7907890-7908019	chr17:7907893-7908019	0.274	0.005383440513	-0.005	0.1664054621
ASS	SLC37A3	-	chr7:140351272-140351969	chr7:140351272-140351451	0.273	2.09E-08	-0.033	0
ASS	RPL17	-	chr18:49491735-49491833	chr18:49491745-49491833	0.271	0	0.022	0.1087311279
ASS	ST3GAL6-A:	-	chr3:98718418-98718521	chr3:98718423-98718521	0.268	0.005488468139	-0.099	1
ASS	TBC1D32	-	chr6:121115171-121115747	chr6:121115171-121115241	0.264	0.03686690828	-0.023	1
ASS	BTAF1	+	chr10:91959064-91959282	chr10:91959064-91959154	0.258	0	-0.036	1
ASS	ZNF341	+	chr20:33757147-33757343	chr20:33757147-33757322	0.258	0.009675218226	-0.068	1
ASS	PIF1	-	chr15:64819846-64820019	chr15:64819846-64819986	0.253	0.01652397872	-0.094	1
ASS	ECI2	-	chr6:4133548-4133711	chr6:4133548-4133701	0.251	9.29E-07	-0.007	1
ASS	NSFL1C	-	chr20:1455713-1455870	chr20:1455713-1455719	0.25	2.59E-08	0.011	1
ASS	ARHGAP26	+	chr5:143207197-143207473	chr5:143207197-143207308	0.25	0.0009743166039	-0.078	0.09728090801
ASS	POMZP3	-	chr7:76611453-76611627	chr7:76611453-76611591	0.25	0.02937565073	-0.008	1
ASS	MTMR2	-	chr11:95887611-95887759	chr11:95887611-95887684	0.249	0.0002271937679	-0.002	1
ASS	GAS8	+	chr16:90031175-90031496	chr16:90031298-90031496	0.247	0.01930354929	-0.11	6.26E-06
ASS	CDKN2AIP	+	chr4:183445534-183445665	chr4:183445534-183445634	0.247	4.13E-05	0.008	1
ASS	PPAPDC1B	-	chr8:38268822-38268990	chr8:38268881-38268990	0.244	0.0002493600206	-0.037	0.000823726726
ASS	ZMAT1	-	chrX:101895813-101895882	chrX:101895813-101895879	0.243	0.02099159975	-0.095	1
ASS	MTRF1L	-	chr6:152995135-152995319	chr6:152995135-152995294	0.242	0.03082514448	0.001	1
ASS	C14orf159	+	chr14:91215404-91215793	chr14:91215404-91215556	0.236	0.03523702781	0	1
ASS	DNAJC10	+	chr2:182739544-182740388	chr2:182740298-182740388	0.235	0.00743209034	-0.085	0.003064074245
ASS	C11orf57	+	chr11:112075566-112075684	chr11:112075566-112075650	0.235	0.00395677552	0.024	1
ASS	KCTD9	-	chr8:25439605-25439792	chr8:25439605-25439664	0.234	0.00907788124	-0.016	1
ASS	ZBTB8OS	-	chr1:32634701-32634792	chr1:32634767-32634792	0.234	0.03728001552	0.016	1
ASS	POMT1	+	chr9:131509902-131510062	chr9:131509902-131509996	0.233	0.008187538655	-0.06	3.81E-06
ASS	HUS1	-	chr7:47979409-47979553	chr7:47979420-47979553	0.233	0.01147637878	0.03	1

ASS	THOC2	-	chrX:123620374-123620965	chrX:123620906-123620965	0.231	0	-0.007	0.0003434966716
ASS	FAM73B	+	chr9:129068018-129068332	chr9:129068197-129068332	0.231	7.43E-05	0.005	0.8570516629
ASS	CNBP	-	chr3:129171633-129171771	chr3:129171654-129171771	0.231	5.77E-08	0.026	0.03863816794
ASS	PSMA3-AS1	-	chr14:58273978-58274135	chr14:58273978-58274010	0.23	0.00508214036	-0.052	0.7447194854
ASS	C11orf74	+	chr11:36594512-36594641	chr11:36594512-36594592	0.226	6.34E-05	-0.042	1
ASS	DPH5	-	chr1:101001466-101001587	chr1:101001466-101001544	0.225	0.004661268084	0.009	1
ASS	TMEM67	+	chr8:93758482-93758576	chr8:93758486-93758576	0.22	0.03659565077	-0.017	1
ASS	TPT1-AS1	+	chr13:45377060-45377215	chr13:45377139-45377215	0.22	0.00019763891	0.011	1
ASS	GAA	+	chr17:80101810-80101925	chr17:80101810-80101890	0.217	0.0003941467045	-0.026	0.2346365999
ASS	ZMYM5	-	chr13:19851354-19852190	chr13:19851688-19852190	0.216	0.0009282013644	-0.021	1
ASS	CAMK2D	-	chr4:113509637-113509675	chr4:113509637-113509672	0.216	0.004100974342	-0.108	0.0003760779331
ASS	FAM111A	+	chr11:59148796-59148953	chr11:59148835-59148953	0.215	0.003383676778	-0.02	1
ASS	FUZ	-	chr19:49812614-49812736	chr19:49812637-49812736	0.214	0.0005034348025	0.026	0.003903921844
ASS	UNKL	-	chr16:1369933-1370357	chr16:1370129-1370357	0.212	4.73E-05	-0.009	1
ASS	VPS54	-	chr2:63981645-63981887	chr2:63981645-63981851	0.212	0.02258460249	-0.047	1
ASS	SLC4A3	+	chr2:219633854-219633979	chr2:219633879-219633979	0.211	0.04759067616	0.002	1
ASS	THNSL1	+	chr10:25016657-25016789	chr10:25016657-25016692	0.211	0.007391124316	0.12	1
ASS	PBRM1	-	chr3:52679573-52679726	chr3:52679573-52679723	0.207	0.0011645449	0.016	1
ASS	GAPVD1	+	chr9:125321432-125321562	chr9:125321432-125321499	0.203	0.0004619053114	-0.065	0.05966462151
ASS	SLC35B1	-	chr17:49706203-49706334	chr17:49706203-49706309	0.202	7.79E-06	-0.01	1
ASS	PFAS	+	chr17:8266500-8266898	chr17:8266752-8266898	0.201	0.009613703767	-0.002	1
ASS	ALG9	-	chr11:111838248-111838399	chr11:111838248-111838378	0.2	0.009557061151	-0.027	0.2889115731
ASS	ANAPC5	-	chr12:121328315-121328497	chr12:121328315-121328458	0.198	5.53E-11	-0.087	0
ASS	HOOK2	-	chr19:12774560-12774741	chr19:12774634-12774741	0.197	0.02371124663	-0.037	1
ASS	PKD1	-	chr16:2105321-2105707	chr16:2105321-2105474	0.196	0.02000335302	-0.048	0.0009523146041
ASS	SETD4	-	chr21:36058815-36058973	chr21:36058815-36058924	0.195	0.03505499569	-0.023	1
ASS	MPV17	-	chr2:27312993-27313154	chr2:27312993-27313074	0.194	0.01272076164	-0.007	1
ASS	MED24	-	chr17:40020272-40020482	chr17:40020272-40020353	0.193	4.61E-06	-0.032	0
ASS	CMC2	-	chr16:80998284-80998473	chr16:80998284-80998354	0.193	0.04069570644	0.025	1
ASS	TIA1	-	chr2:70216752-70216994	chr2:70216885-70216994	0.191	1.98E-07	-0.046	6.41E-05
ASS	IRF3	-	chr19:49661449-49662328	chr19:49661947-49662328	0.191	0.003142933795	0.005	1
ASS	LMBR1L	-	chr12:49106510-49106746	chr12:49106544-49106746	0.188	0.0004977651348	0.043	1
ASS	ZC3HAV1	-	chr7:139079103-139080243	chr7:139079469-139080243	0.187	0.0002460105186	-0.022	1
ASS	DOCK10	-	chr2:224805407-224805529	chr2:224805407-224805502	0.187	0.004936945245	0.168	3.62E-06
ASS	SDR39U1	-	chr14:24442108-24442260	chr14:24442177-24442260	0.186	0.002257648074	-0.005	1
ASS	CDK10	+	chr16:89694568-89694788	chr16:89694664-89694788	0.186	0.01220499947	-0.026	0.02428250175
ASS	NSUN5P1	+	chr7:75415591-75415778	chr7:75415591-75415770	0.186	0.02639140838	-0.007	1
ASS	PRKCSH	+	chr19:11447692-11447789	chr19:11447722-11447789	0.185	0.01102843369	-0.013	1
ASS	BRF1	-	chr14:105219150-105219298	chr14:105219150-105219232	0.185	0.001386479338	0.004	1
ASS	RPP25L	-	chr9:34611859-34612104	chr9:34612063-34612104	0.183	0.008976958899	-0.021	0.4656072724
ASS	DPY19L3	+	chr19:32467473-32468813	chr19:32468730-32468813	0.183	0.02228411796	-0.003	0.01410483595
ASS	SETD5	+	chr3:9434276-9434485	chr3:9434333-9434485	0.181	0.002983828513	0.017	1
ASS	FAM73B	+	chr9:129067994-129068332	chr9:129068197-129068332	0.18	0.003471603937	0.002	1
ASS	STRN4	-	chr19:46727451-46727567	chr19:46727451-46727546	0.177	0.00157658726	-0.085	1.23E-12
ASS	TRIM33	-	chr1:114397607-114397990	chr1:114397607-114397860	0.173	0.0254485429	0.015	1
ASS	MAPRE3	+	chr2:27024095-27024297	chr2:27024095-27024252	0.171	0.0008976924693	0.154	4.02E-12
ASS	HAUS4	-	chr14:22956745-22957094	chr14:22956915-22957094	0.171	0.03020364092	-0.028	0.0732778143
ASS	C1orf112	+	chr1:169804074-169804240	chr1:169804111-169804240	0.17	0.003340729773	-0.015	1
ASS	KANK1	+	chr9:732377-734835	chr9:734747-734835	0.169	0.0005420927632	0.031	1
ASS	FAM219B	-	chr15:74904981-74905059	chr15:74904981-74905039	0.169	0.01124910275	0.027	1
ASS	APRT	-	chr16:88809468-88809840	chr16:88809468-88809706	0.167	1.57E-06	0.03	0.002294258572
ASS	BICD1	+	chr12:32337498-32337816	chr12:32337498-32337706	0.166	0.04888897915	-0.011	1
ASS	WDR26	-	chr1:224431476-224431581	chr1:224431524-224431581	0.166	0.004296445586	-0.029	0.4588527966
ASS	SLC25A19	-	chr17:75288497-75288591	chr17:75288501-75288591	0.165	0.0007135351241	-0.003	0.9595103662
ASS	RNF216	-	chr7:5739275-5739621	chr7:5739275-5739352	0.164	2.09E-08	-0.007	0.003577665493
ASS	KIF2A	+	chr5:62352587-62352710	chr5:62352587-62352653	0.164	0.004282582856	-0.039	0.6032882041
ASS	USP33	-	chr1:77721830-77721925	chr1:77721830-77721901	0.164	0.03232140899	0.001	1
ASS	ANK3	-	chr10:60059339-60060006	chr10:60059339-60059430	0.164	0.003617031386	0.24	7.50E-11
ASS	NCOR2	-	chr12:124327408-124327633	chr12:124327546-124327633	0.164	0.02794282114	-0.176	0
ASS	SFSWAP	+	chr12:131714070-131714240	chr12:131714070-131714193	0.163	1.54E-07	0.007	1
ASS	WHAMM	+	chr15:82826387-82826496	chr15:82826409-82826496	0.163	0.008521201378	-0.016	1
ASS	NUSAP1	+	chr15:41350990-41351129	chr15:41351032-41351129	0.162	0.0004781326667	-0.005	1
ASS	MSTO1	+	chr1:155613656-155613766	chr1:155613656-155613733	0.16	0.02717992751	-0.057	0.3210570112

ASS	IL17RC	+	chr3:9923880-9924137	chr3:9923880-9924020	0.159	0.01632096844	-0.053	7.56E-09
ASS	RP4-639F20	+	chr1:94962924-94963270	chr1:94962978-94963270	0.159	0.02193666938	-0.018	1
ASS	TJAP1	+	chr6:43498922-43499100	chr6:43498974-43499100	0.158	0.005932310734	-0.025	0.1036896878
ASS	SECISBP2	+	chr9:89334521-89334730	chr9:89334635-89334730	0.158	0.002486846193	-0.014	1
ASS	RAP1GAP	-	chr1:21597960-21598064	chr1:21597960-21598040	0.156	0.002071148324	-0.018	1
ASS	HECTD4	-	chr12:112264083-112264212	chr12:112264089-112264212	0.156	0.04804249068	-0.051	1
ASS	RBM6	+	chr3:49967197-49968748	chr3:49967469-49968748	0.154	0.02566195299	0.028	1
ASS	HSF4	+	chr16:67167460-67167599	chr16:67167474-67167599	0.154	0.01065698785	0.002	1
ASS	FOXRED1	+	chr11:126273315-126273454	chr11:126273338-126273454	0.153	0.02000335302	-0.118	1
ASS	ZSCAN26	+	chr6:28271853-28272339	chr6:28271853-28272336	0.153	0.004252062559	-0.037	1
ASS	CENPK	-	chr5:65561462-65561564	chr5:65561496-65561564	0.15	5.79E-06	0.002	1
ASS	CYLD	+	chr16:50749548-50750202	chr16:50749575-50750202	0.149	0.001576710913	-0.037	1
ASS	MUTYH	-	chr1:45332917-45333324	chr1:45333284-45333324	0.149	0.03557564081	0.075	0
ASS	DDIT3	-	chr12:57517268-57517595	chr12:57517268-57517424	0.149	0.0401411959	-0.031	0.4700854447
ASS	SMTN	+	chr22:31088453-31088606	chr22:31088512-31088606	0.149	0.00185771104	-0.017	0.001441947888
ASS	PROSER3	+	chr19:35765033-35765176	chr19:35765066-35765176	0.148	0.02479012398	-0.042	1
ASS	TMEM62	+	chr15:43149028-43149151	chr15:43149107-43149151	0.147	3.84E-05	0.004	1
ASS	RMND1	-	chr6:151436419-151436554	chr6:151436445-151436554	0.147	0.009959142975	-0.013	0.4403719306
ASS	REPS1	-	chr6:138926400-138930098	chr6:138929976-138930098	0.146	2.51E-07	-0.009	0
ASS	ZDHHC4	+	chr7:6577467-6577720	chr7:6577467-6577498	0.146	0.007745721211	0.074	0.0006772200526
ASS	SFT2D1	-	chr6:166322856-166324595	chr6:166322856-166322886	0.146	0.02088288316	-0.007	1
ASS	BPHL	+	chr6:3138040-3140509	chr6:3140385-3140509	0.145	0.004748089327	-0.011	0.1581460708
ASS	ANKRD10	-	chr13:110900529-110900694	chr13:110900529-110900661	0.144	0.0004438203268	-0.049	1
ASS	SDHAP1	-	chr3:195965428-195965577	chr3:195965428-195965542	0.143	0.00934947157	-0.016	1
ASS	SART3	-	chr12:108544426-108544532	chr12:108544426-108544478	0.142	0.000777512798	-0.033	0.1930077518
ASS	NDOR1	+	chr9:137216066-137216188	chr9:137216093-137216188	0.142	0.0008449208851	-0.013	1
ASS	PPP4R1L	-	chr20:58240514-58240710	chr20:58240514-58240610	0.14	0.04060591643	-0.044	1
ASS	TJAP1	+	chr6:43498922-43499100	chr6:43498977-43499100	0.139	0.0218019065	-0.021	0.4092659801
ASS	ZFAND2A	-	chr7:1152906-1153241	chr7:1152906-1153224	0.139	0.02999909818	0.001	1
ASS	SNHG5	-	chr6:85677794-85678032	chr6:85677953-85678032	0.139	3.85E-07	0.158	2.28E-13
ASS	ZNF76	+	chr6:35290252-35290382	chr6:35290265-35290382	0.138	0.002194122714	-0.006	1
ASS	MARK3	+	chr14:103468032-103468186	chr14:103468080-103468186	0.136	0.007114890212	-0.042	0.02818569323
ASS	FKBP10	+	chr17:41819529-41820049	chr17:41819529-41819675	0.136	0.003748982002	-0.031	0
ASS	DUXAP8	+	chr22:15790660-15790798	chr22:15790684-15790798	0.136	0.01002753622	-0.095	1
ASS	CNOT3	+	chr19:54145541-54145817	chr19:54145594-54145817	0.135	0.001111191173	-0.038	0.0001534372107
ASS	ATF6B	-	chr6:32117036-32117130	chr6:32117036-32117107	0.134	1.90E-05	-0.019	4.89E-05
ASS	C21orf2	-	chr21:44334060-44335804	chr21:44335757-44335804	0.133	0.025564861	-0.022	0.0183057067
ASS	CDK5RAP3	+	chr17:47973518-47973693	chr17:47973518-47973650	0.131	1.44E-06	-0.03	0
ASS	GALE	-	chr1:23796508-23796782	chr1:23796696-23796782	0.129	0.002262636209	0.186	0
ASS	QKI	+	chr6:163563419-163563719	chr6:163563443-163563719	0.127	1.90E-05	0.02	0.01917737047
ASS	UNC50	+	chr2:98618145-98618515	chr2:98618167-98618515	0.127	0.0003777090284	-0.008	0.01434919428
ASS	APBA2	+	chr15:28921639-28921749	chr15:28921639-28921718	0.126	0.001751023104	0.018	0.1786536796
ASS	TECR	+	chr19:14563140-14563257	chr19:14563173-14563257	0.126	0.02150085512	-0.006	1
ASS	PAAF1	+	chr11:73886928-73887099	chr11:73887039-73887099	0.125	0.006867038563	-0.045	1
ASS	HSCB	+	chr22:28743881-28743978	chr22:28743881-28743923	0.124	0.0001921815736	0	1
ASS	PABPC1L	+	chr20:44938044-44938191	chr20:44938060-44938191	0.124	0.009237124357	0.002	1
ASS	C5orf45	-	chr5:179853196-179853437	chr5:179853377-179853437	0.124	0.001721902013	-0.035	6.13E-10
ASS	NUSAP1	+	chr15:41350987-41351129	chr15:41351032-41351129	0.123	0.000118969917	-0.001	1
ASS	NPHP3	-	chr3:132692653-132693865	chr3:132692653-132692818	0.122	3.53E-05	0.018	1
ASS	TIMM17B	-	chrX:48895351-48895501	chrX:48895351-48895454	0.121	0.00750782119	-0.001	1
ASS	CCDC90B	-	chr11:83273950-83274740	chr11:83273950-83273992	0.12	0.005087330558	0.033	0.7861625944
ASS	FUBP1	-	chr1:77947452-77947552	chr1:77947452-77947547	0.12	0.01033981784	0.011	1
ASS	TPT1-AS1	+	chr13:45390713-45390902	chr13:45390757-45390902	0.12	1.61E-05	0.018	1
ASS	ZNF664	+	chr12:123973218-123973352	chr12:123973218-123973303	0.12	0.0156075419	0.023	1
ASS	IVD	+	chr15:40407635-40407812	chr15:40407635-40407725	0.119	0.00226146107	-0.021	1.23E-14
ASS	SLC37A3	-	chr7:140352061-140352181	chr7:140352061-140352146	0.119	0.001576710913	-0.004	0.0001172081813
ASS	BMP1	+	chr8:22176923-22177139	chr8:22176960-22177139	0.119	0.01003596905	-0.076	5.14E-07
ASS	CCS	+	chr11:66605153-66605416	chr11:66605338-66605416	0.119	0.03445710622	-0.02	2.78E-07
ASS	CLN6	-	chr15:68211555-68211863	chr15:68211674-68211863	0.117	0.0269999003	-0.044	0
ASS	NDUFAF7	+	chr2:37241577-37241791	chr2:37241577-37241637	0.116	0.00662797432	-0.018	1
ASS	CASC3	+	chr17:40167370-40167612	chr17:40167497-40167612	0.116	0.0001438215556	0.002	1
ASS	RAB15	-	chr14:64950578-64951151	chr14:64951073-64951151	0.116	0.04917885074	-0.006	0.1808345877
ASS	PIGG	+	chr4:521055-521273	chr4:521055-521249	0.116	0.0004977651348	-0.032	1

ASS	SMC4	+	chr3:160412325-160412849	chr3:160412325-160412453	0.115	0	-0.001	1
ASS	CDK11A	-	chr1:1722587-1722831	chr1:1722707-1722831	0.114	0.006888488616	-0.016	0.08616336822
ASS	COX11	-	chr17:54961118-54962915	chr17:54961118-54961312	0.114	7.79E-06	0.11	0
ASS	THOC2	-	chrX:123610917-123610963	chrX:123610917-123610957	0.113	0.002286623134	0.052	0.0007886757372
ASS	C6orf48	+	chr6:31836298-31836517	chr6:31836423-31836517	0.113	0.03010599228	-0.006	1
ASS	JKAMP	+	chr14:59486712-59486804	chr14:59486730-59486804	0.112	0.0004772283099	0.038	0.06903160736
ASS	ZNF655	+	chr7:99561887-99562494	chr7:99562367-99562494	0.111	0.02597407973	-0.032	1
ASS	C6orf48	+	chr6:31836294-31836517	chr6:31836423-31836517	0.111	0.02999909818	-0.005	1
ASS	ZNF596	+	chr8:242886-243013	chr8:242917-243013	0.109	0.02146154903	-0.023	1
ASS	IGSF8	-	chr1:160091339-160091631	chr1:160091339-160091608	0.108	0.01220499947	-0.01	0.1785591081
ASS	DNAJC17	-	chr15:40776195-40776401	chr15:40776195-40776292	0.106	4.68E-08	-0.017	5.68E-07
ASS	ENSA	-	chr1:150627311-150627592	chr1:150627466-150627592	0.106	0.02703202502	0.018	1
ASS	PBRM1	-	chr3:52587352-52587510	chr3:52587352-52587435	0.106	0.007750127607	0.013	0.09767448422
ASS	ZNF3	-	chr7:100077228-100077433	chr7:100077302-100077433	0.106	0.01848702629	0.014	1
ASS	CACNB3	+	chr12:48826244-48826518	chr12:48826366-48826518	0.105	0.01216881001	-0.02	3.16E-06
ASS	KMT2C	-	chr7:152156204-152156346	chr7:152156204-152156274	0.104	0.001747876875	-0.024	0.3652827441
ASS	DNAJC10	+	chr2:182739544-182740388	chr2:182740298-182740388	0.104	0.0003718189421	-0.04	4.59E-07
ASS	ZBTB17	-	chr1:15946153-15946316	chr1:15946153-15946294	0.104	0.004732969007	-0.007	1
ASS	PEX1	-	chr7:92501506-92502079	chr7:92501506-92501673	0.104	0.0008732873205	0.036	1
ASS	TAX1BP1	+	chr7:27816348-27816520	chr7:27816348-27816394	0.103	5.39E-05	-0.017	0.05541473235
ASS	MAP4K4	+	chr2:101887087-101887261	chr2:101887087-101887237	-0.102	2.13E-07	0.031	1.16E-05
ASS	TIMM17B	-	chrX:48895351-48895972	chrX:48895351-48895501	-0.102	0.0463393071	0.005	1
ASS	CENPK	-	chr5:65554796-65554946	chr5:65554796-65554898	-0.103	0.004100974342	-0.011	0.1356658922
ASS	NUDT13	+	chr10:73126634-73126827	chr10:73126672-73126827	-0.103	0.02810489556	-0.113	1
ASS	ST5	-	chr11:8702571-8703611	chr11:8702571-8702720	-0.104	0.01220499947	-0.005	1.01E-06
ASS	FAM114A2	-	chr5:154038292-154038434	chr5:154038387-154038434	-0.106	0.005638572567	0.024	1
ASS	FZR1	+	chr19:3534420-3534522	chr19:3534420-3534513	-0.106	0.006456856362	0.011	0.9931016041
ASS	DCAF4	+	chr14:72945883-72946027	chr14:72945887-72946027	-0.106	0.02566195299	-0.01	1
ASS	WHSC1	+	chr4:1952751-1953524	chr4:1953323-1953524	-0.107	0.002824582625	-0.007	0.1770369432
ASS	RALGPS1	+	chr9:127212943-127213049	chr9:127212967-127213049	-0.109	0.007750127607	-0.008	1
ASS	BPTF	+	chr17:67945408-67946325	chr17:67945408-67945896	-0.11	0.00665733317	0.016	1
ASS	RUSC1	+	chr1:155326579-155327132	chr1:155326897-155327132	-0.112	0.01152357239	-0.012	0.01001965199
ASS	BDP1	+	chr5:71541453-71541682	chr5:71541456-71541682	-0.113	0.01057437769	0.019	1
ASS	FCHSD1	-	chr5:141644858-141644942	chr5:141644858-141644938	-0.113	0.003195651006	-0.013	1
ASS	HNRNPH3	+	chr10:68338502-68338687	chr10:68338502-68338642	-0.114	0.004040130954	0.002	1
ASS	TRIM37	-	chr17:59001597-59001741	chr17:59001597-59001714	-0.114	0.0003718189421	-0.002	1
ASS	SNHG8	+	chr4:118278708-118279137	chr4:118278708-118278792	-0.114	0.0009901676837	0.045	0.8071787139
ASS	ZC3H11A	+	chr1:203796292-203796601	chr1:203796292-203796496	-0.116	0.03765181523	0.033	1
ASS	MRRF	+	chr9:122285168-122285946	chr9:122285168-122285287	-0.116	0.01403880967	-0.002	0.253174273
ASS	RECQL	-	chr12:21501169-21501372	chr12:21501169-21501215	-0.117	0.02462972179	0.013	1
ASS	RAP1GAP	-	chr1:21602803-21603912	chr1:21602803-21602913	-0.117	7.66E-09	-0.016	0.000110843787
ASS	NAGK	+	chr2:71072825-71073594	chr2:71073481-71073594	-0.117	0.0006627704568	-0.025	0
ASS	GUCD1	-	chr22:24540437-24543100	chr22:24540437-24543097	-0.117	5.52E-06	-0.005	0.9902235134
ASS	SYVN1	-	chr11:65132247-65132780	chr11:65132731-65132780	-0.118	0.02387795607	0.006	1
ASS	XRN1	-	chr3:142332374-142332531	chr3:142332374-142332424	-0.119	0.02871858636	0.012	1
ASS	PXN	-	chr12:120215102-120215417	chr12:120215102-120215273	-0.121	0.002194122714	0.093	0.09087061813
ASS	RETSAT	-	chr2:85345512-85346094	chr2:85345974-85346094	-0.122	0.001218214733	-0.02	0
ASS	GABPB1	-	chr15:50301220-50301368	chr15:50301256-50301368	-0.122	0.02794282114	0.024	0.6345367851
ASS	FAHD2A	+	chr2:95410526-95411026	chr2:95410526-95410586	-0.122	0.04707260085	0.048	0.001080396776
ASS	MTG1	+	chr10:133401528-133402245	chr10:133402148-133402245	-0.122	0.01355011343	-0.012	1
ASS	PPP2R2D	+	chr10:131943967-131944145	chr10:131944094-131944145	-0.123	0.0004132445768	0.022	0.002816004248
ASS	DDX39B	-	chr6:31540321-31540664	chr6:31540321-31540534	-0.123	0.000140885502	-0.09	1.25E-06
ASS	PARP14	+	chr3:122720254-122721653	chr3:122720254-122720388	-0.124	0.009250039851	-0.012	8.44E-09
ASS	ZSCAN5A	-	chr19:56314619-56314839	chr19:56314680-56314839	-0.126	0.02674759003	-0.011	1
ASS	MRPL55	-	chr1:228108436-228109021	chr1:228108954-228109021	-0.126	0.001605495495	-0.061	0.03088046436
ASS	PSMD6	-	chr3:64022642-64022801	chr3:64022738-64022801	-0.126	0.04120128176	0.005	1
ASS	CASP8	+	chr2:201266460-201266791	chr2:201266486-201266791	-0.127	0.002459197716	-0.024	1
ASS	SH3D19	-	chr4:151176524-151176687	chr4:151176533-151176687	-0.127	0.001051640299	0.018	1
ASS	TAF1D	-	chr11:93736693-93737239	chr11:93737063-93737239	-0.127	1.32E-10	0.019	1
ASS	ISG20	+	chr15:88650234-88652310	chr15:88652169-88652310	-0.128	0.004538961798	-0.111	0.2212579832
ASS	HAUS8	-	chr19:17059996-17060092	chr19:17059996-17060089	-0.131	0.04551241736	-0.007	1
ASS	RUSC1	+	chr1:155326609-155327132	chr1:155326897-155327132	-0.131	0.01930354929	-0.015	0.01418407996
ASS	RYK	-	chr3:134191848-134191983	chr3:134191848-134191974	-0.132	0.02228411796	-0.021	1

ASS	CENPT	-	chr16:67832227-67832545	chr16:67832454-67832545	-0.133	0.0002116162297	0.007	1
ASS	SMARCA4	+	chr19:11058254-11058363	chr19:11058257-11058363	-0.134	0.008425492765	0.027	0.1906372409
ASS	BZW2	+	chr7:16646130-16646304	chr7:16646130-16646288	-0.135	0.001551747794	0.024	1
ASS	RREB1	+	chr6:7181123-7181289	chr6:7181123-7181246	-0.136	0.005879821613	0.095	1
ASS	IMPA2	+	chr18:12012169-12012349	chr18:12012169-12012215	-0.136	0.02239688901	-0.021	1
ASS	PPT2-EGFL	+	chr6:32167086-32167257	chr6:32167102-32167257	-0.136	0.001225588831	0.032	1.85E-07
ASS	ATG16L2	+	chr11:72825301-72825407	chr11:72825348-72825407	-0.137	0.0003901111932	-0.013	1
ASS	PIDD1	-	chr11:800984-801120	chr11:801001-801120	-0.139	0.01657875278	-0.007	1
ASS	UBE2D3	-	chr4:102826484-102826844	chr4:102826484-102826636	-0.14	0.0326899352	0.027	0.4551225185
ASS	RMND5B	+	chr5:178138107-178138396	chr5:178138107-178138258	-0.141	0.03086650433	-0.023	0.01044156558
ASS	WDPCP	-	chr2:63439756-63439871	chr2:63439756-63439855	-0.142	0.02937565073	0.047	1
ASS	RPL7L1	+	chr6:42880860-42881082	chr6:42880860-42880966	-0.144	0.0115195204	0	1
ASS	HUWE1	-	chrX:53543840-53543968	chrX:53543885-53543968	-0.145	0.04156987741	-0.005	1
ASS	CNOT8	+	chr5:154858638-154858816	chr5:154858638-154858768	-0.145	0.004634626811	-0.03	0.330382506
ASS	RSRP1	-	chr1:25243549-25244224	chr1:25243549-25243633	-0.146	0	0.025	1
ASS	SLC22A17	-	chr14:23347459-23347731	chr14:23347513-23347731	-0.149	0.00896509776	-0.014	0.008337512171
ASS	CPSF7	-	chr11:61420469-61421608	chr11:61421389-61421608	-0.151	0.002518662501	0.039	7.21E-10
ASS	SHKBP1	+	chr19:40590729-40590853	chr19:40590732-40590853	-0.151	0.0151374011	-0.001	1
ASS	MSH5-SAP(	+	chr6:31761817-31761955	chr6:31761817-31761865	-0.151	4.80E-05	-0.002	0.7085476287
ASS	HMGN3	-	chr6:79202275-79202389	chr6:79202316-79202389	-0.153	4.51E-05	-0.019	1
ASS	ARHGEF1	+	chr19:41892024-41892373	chr19:41892330-41892373	-0.155	0.006062200051	0.064	1.01E-08
ASS	FAM208A	-	chr3:56633043-56633966	chr3:56633043-56633843	-0.155	2.29E-06	-0.007	1
ASS	GPANK1	-	chr6:31663852-31664580	chr6:31663852-31664577	-0.155	0.01713879478	0.039	0.3562912458
ASS	NEDD1	+	chr12:96907603-96907856	chr12:96907603-96907624	-0.158	0.0002711507675	0.047	1
ASS	RAD51	+	chr15:40695173-40695425	chr15:40695173-40695321	-0.159	0.0002455544809	0	1
ASS	CEP78	+	chr9:78251904-78252043	chr9:78251907-78252043	-0.161	0.02753281436	-0.007	1
ASS	FNBP1	-	chr9:129923843-129923996	chr9:129923843-129923939	-0.161	0.007390962247	-0.005	1
ASS	MRPL43	-	chr10:100983947-100984074	chr10:100984033-100984074	-0.163	0.005849655558	-0.021	1
ASS	PI4KAP1	-	chr22:18547245-18547421	chr22:18547245-18547373	-0.163	0.01057437769	0.019	1
ASS	ZCWPW1	-	chr7:100415974-100416097	chr7:100415974-100416094	-0.165	0.04156779975	0.009	1
ASS	PLEKHH3	-	chr17:42670993-42671130	chr17:42671002-42671130	-0.166	0.0177309439	-0.013	1
ASS	PHF20L1	+	chr8:132804611-132804740	chr8:132804614-132804740	-0.166	4.50E-06	-0.017	1
ASS	PRKCSH	+	chr19:11447438-11447618	chr19:11447438-11447597	-0.166	0.03010377877	-0.048	0.001154053307
ASS	ANXA4	+	chr2:69806384-69806498	chr2:69806384-69806432	-0.17	2.79E-05	0.005	1
ASS	WSB1	+	chr17:27307743-27309272	chr17:27309099-27309272	-0.171	0	-0.089	0.002565534185
ASS	KLC1	+	chr14:103679383-103679545	chr14:103679383-103679518	-0.171	0.02000464033	0.046	0.08950478072
ASS	ZNF596	+	chr8:243721-243858	chr8:243721-243805	-0.172	0.001472187424	0.001	1
ASS	INPP5B	-	chr1:37931672-37932053	chr1:37931912-37932053	-0.173	3.09E-06	-0.014	0.2820841183
ASS	TCAIM	+	chr3:44367455-44367708	chr3:44367531-44367708	-0.174	0.04017537365	0.007	1
ASS	SGOL1	-	chr3:20174248-20175055	chr3:20175004-20175055	-0.175	0.000404362015	0.034	0.1819771004
ASS	PRPF38A	+	chr1:52405679-52405839	chr1:52405679-52405713	-0.175	0.0001084941665	0.01	0.001810073749
ASS	ZNF232	-	chr17:5109393-5109868	chr17:5109393-5109768	-0.175	0.0004438203268	0.011	2.76E-05
ASS	NOL8	-	chr9:92324905-92325350	chr9:92325305-92325350	-0.176	1.63E-06	-0.046	1
ASS	CPEB2	+	chr4:15007304-15007595	chr4:15007304-15007586	-0.18	0.0001514101156	0.014	1
ASS	ZWINT	-	chr10:56358804-56358947	chr10:56358908-56358947	-0.184	0.004634626811	-0.013	1
ASS	NPAS2	+	chr2:100995399-100996055	chr2:100995795-100996055	-0.184	2.46E-05	-0.038	0.2567807888
ASS	DAP3	+	chr1:155689090-155689243	chr1:155689090-155689174	-0.185	0	0.004	1
ASS	TUBGCP4	+	chr15:43403682-43403842	chr15:43403682-43403799	-0.187	7.91E-06	-0.026	1
ASS	CXorf38	-	chrX:40639008-40640320	chrX:40639008-40639128	-0.188	0.000157290213	-0.03	1
ASS	TRIQK	-	chr8:92885319-92886735	chr8:92885319-92886667	-0.188	0.003596342349	0.044	0.08095202563
ASS	NAA60	+	chr16:3484698-3485083	chr16:3484698-3485061	-0.189	0.0002455544809	0.027	0.1856844325
ASS	NAT9	-	chr17:74773575-74773688	chr17:74773575-74773685	-0.19	0.00736127707	0.009	1
ASS	DOCK1	+	chr10:127012231-127012374	chr10:127012294-127012374	-0.194	6.31E-06	-0.004	1
ASS	MYO19	-	chr17:36508896-36509135	chr17:36509061-36509135	-0.194	0.000193223543	-0.034	0.001388486115
ASS	COA1	-	chr7:43650492-43650712	chr7:43650611-43650712	-0.196	0.007807705261	-0.035	1
ASS	SIPA1L1	+	chr14:71704720-71705340	chr14:71705221-71705340	-0.198	1.76E-05	0.002	1
ASS	FANCL	-	chr2:58198578-58198662	chr2:58198593-58198662	-0.201	0.0001938388517	0.016	1
ASS	KMT2A	+	chr11:118471661-118474315	chr11:118474178-118474315	-0.201	3.34E-05	-0.03	1
ASS	CCHCR1	-	chr6:31144628-31144788	chr6:31144686-31144788	-0.201	0.001143198533	-0.005	1
ASS	INTU	+	chr4:127685441-127687867	chr4:127687677-127687867	-0.203	0.004053514847	-0.119	0.0337448331
ASS	ARID1A	+	chr1:26772811-26772987	chr1:26772814-26772987	-0.205	0.0005909680069	-0.003	1
ASS	WDR31	-	chr9:113330983-113331116	chr9:113330983-113331113	-0.206	0.02812554226	-0.037	1
ASS	MZF1	-	chr19:58573054-58573575	chr19:58573372-58573575	-0.21	0.025564861	-0.003	1

ASS	ARPC1B	+	chr7:99377337-99377455	chr7:99377337-99377439	-0.21	0.002309111532	-0.015	1
ASS	ADARB1	+	chr21:45222017-45223577	chr21:45222017-45222107	-0.212	0.003150772569	-0.032	1
ASS	ZNF559	+	chr19:9341616-9343843	chr19:9341694-9343843	-0.213	0.03031240378	-0.048	0.2212579832
ASS	NEO1	+	chr15:73270009-73270233	chr15:73270057-73270233	-0.215	0.0001021511285	-0.266	0
ASS	CCDC66	+	chr3:56566593-56566759	chr3:56566593-56566740	-0.215	0.001851714863	0.027	0.253174273
ASS	RSRP1	-	chr1:25244176-25245301	chr1:25245149-25245301	-0.218	0	0.029	1
ASS	SLC52A2	+	chr8:144358592-144359065	chr8:144358592-144358900	-0.218	0.003268335568	0.017	1
ASS	KIAA0195	+	chr17:75494626-75494808	chr17:75494709-75494808	-0.219	0.00121186621	-0.016	1
ASS	PRR13	+	chr12:53443390-53443773	chr12:53443540-53443773	-0.219	3.86E-05	-0.013	0.001193237389
ASS	EFNA1	+	chr1:155133502-155133780	chr1:155133729-155133780	-0.221	0.001140508688	0.036	1
ASS	KLC1	+	chr14:103679383-103679545	chr14:103679383-103679518	-0.222	0.002423306488	0.026	0.4847738017
ASS	PRR13	+	chr12:53443390-53443773	chr12:53443486-53443773	-0.223	8.78E-09	-0.01	0.9902235134
ASS	FAM208A	-	chr3:56633043-56633966	chr3:56633043-56633843	-0.224	4.33E-06	-0.001	1
ASS	KB-1572G7.	-	chr22:23686830-23686995	chr22:23686837-23686995	-0.224	0.005345968046	0.008	1
ASS	ISG20	+	chr15:88650234-88652310	chr15:88652109-88652310	-0.225	0.0436367351	-0.013	0
ASS	PRR13	+	chr12:53443486-53443773	chr12:53443540-53443773	-0.226	0.005572013596	-0.029	0.06438414149
ASS	SLC25A19	-	chr17:75288387-75288591	chr17:75288497-75288591	-0.227	8.47E-05	0.009	0.7951186121
ASS	TRIM69	+	chr15:44758612-44758854	chr15:44758620-44758854	-0.228	0.0004772283099	0.02	1
ASS	SENP6	+	chr6:75640683-75640704	chr6:75640686-75640704	-0.229	0.03982276621	0.047	1
ASS	TAZ	+	chrX:154413206-154413358	chrX:154413206-154413339	-0.23	0.04391007992	-0.036	1
ASS	AURKA	-	chr20:56392153-56392295	chr20:56392167-56392295	-0.231	0.02306657183	0.071	1
ASS	BDH2	-	chr4:103085348-103086540	chr4:103086479-103086540	-0.231	0.04209827853	0.024	0.3286128168
ASS	Sep15	-	chr1:86914027-86914424	chr1:86914314-86914424	-0.231	0	0.028	0
ASS	NPHP1	-	chr2:110164519-110164730	chr2:110164687-110164730	-0.238	0.0373288093	-0.121	1
ASS	SCAPER	-	chr15:76771723-76771954	chr15:76771741-76771954	-0.241	0.006799172032	-0.025	1
ASS	BDH2	-	chr4:103085698-103086540	chr4:103086479-103086540	-0.241	0	0.015	0.07530626706
ASS	HACE1	-	chr6:104777012-104777110	chr6:104777012-104777097	-0.242	0.01220499947	0.021	6.83E-08
ASS	PACRGL	+	chr4:20700457-20700747	chr4:20700457-20700611	-0.242	0.001751023104	-0.066	0.009106652149
ASS	FANCA	-	chr16:89739128-89739289	chr16:89739132-89739289	-0.242	0.009715487683	-0.003	1
ASS	FAM122B	-	chrX:134769566-134772283	chrX:134769566-134771354	-0.244	0	-0.019	1
ASS	ATG16L1	+	chr2:233272965-233273777	chr2:233272965-233273052	-0.245	0.01938843415	-0.046	0.00493616634
ASS	C19orf66	+	chr19:10091475-10091630	chr19:10091583-10091630	-0.246	0.006127776705	-0.013	0.4633519523
ASS	C9orf89	+	chr9:93107673-93107827	chr9:93107673-93107816	-0.246	0.0003422060407	-0.025	0.9166047911
ASS	LAS1L	-	chrX:65520701-65523707	chrX:65523559-65523707	-0.247	0	-0.138	0
ASS	DMAP1	+	chr1:44213662-44213858	chr1:44213708-44213858	-0.247	0.003715223759	-0.029	1
ASS	CDK11A	-	chr1:1719327-1719455	chr1:1719357-1719455	-0.248	0.0177309439	-0.026	1
ASS	BAHD1	+	chr15:40465334-40465435	chr15:40465343-40465435	-0.252	0.000491510266	0.051	0.8460730171
ASS	ARL6IP4	+	chr12:122981570-122981879	chr12:122981594-122981879	-0.254	3.67E-10	-0.021	0.3143760161
ASS	R3HDM1	+	chr2:135641535-135641790	chr2:135641535-135641604	-0.256	0.01629666727	-0.04	0.5894882427
ASS	NOP56	+	chr20:2655933-2656214	chr20:2655933-2656034	-0.26	0	-0.039	0.002273725252
ASS	XRCC3	-	chr14:103711465-103711661	chr14:103711465-103711567	-0.26	0.04859579048	-0.014	1
ASS	FNBP1	-	chr9:129923843-129924026	chr9:129923843-129923939	-0.261	0.0001981096245	-0.003	1
ASS	VPS29	-	chr12:110499456-110499546	chr12:110499534-110499546	-0.262	0.01416002077	0.02	1
ASS	SLC35A2	-	chrX:48904745-48905482	chrX:48904745-48904892	-0.268	0.003195651006	-0.016	1
ASS	CLPTM1L	-	chr5:1331798-1331883	chr5:1331802-1331883	-0.269	8.36E-05	0.014	1
ASS	AP4M1	+	chr7:100101879-100102393	chr7:100101879-100101968	-0.269	0	-0.015	1.56E-05
ASS	ZNF711	+	chrX:85246929-85247651	chrX:85247546-85247651	-0.271	7.29E-08	-0.02	1
ASS	ZNF641	-	chr12:48347148-48347343	chr12:48347251-48347343	-0.276	0.03772791898	0.085	1
ASS	RFC5	+	chr12:118017689-118018053	chr12:118017689-118017815	-0.284	0.002352533445	-0.077	0.5448320262
ASS	ADCY6	-	chr12:48773468-48774098	chr12:48773468-48773647	-0.286	0.002706536394	0.012	0.5681061732
ASS	LIG1	-	chr19:48120166-48121322	chr19:48121169-48121322	-0.288	0.02059523257	-0.039	0.06844812466
ASS	PNISR	-	chr6:99403828-99404702	chr6:99404602-99404702	-0.289	8.34E-11	0.025	0.699386043
ASS	C7orf50	-	chr7:1138188-1138257	chr7:1138217-1138257	-0.289	1.25E-06	0.12	0.06516334069
ASS	CDK13	+	chr7:40092784-40093237	chr7:40092964-40093237	-0.292	4.03E-10	0.019	1
ASS	B4GALT3	-	chr1:161175807-161176095	chr1:161175807-161176074	-0.292	1.36E-05	-0.167	0
ASS	KIZ	+	chr20:21205490-21205584	chr20:21205490-21205530	-0.295	0.01371813043	0.036	0.00973255938
ASS	WDR83	+	chr19:12670518-12670611	chr19:12670562-12670611	-0.299	0.04408854106	-0.018	0.02042970593
ASS	PARP2	+	chr14:20344931-20345126	chr14:20344931-20345087	-0.299	1.33E-08	-0.028	1
ASS	PCBP1-AS1	-	chr2:70083506-70083687	chr2:70083537-70083687	-0.299	0.004120571796	-0.135	1
ASS	NGLY1	-	chr3:25736308-25737455	chr3:25737333-25737455	-0.3	0.00181880726	0.041	1
ASS	ARHGEF40	+	chr14:21075331-21075765	chr14:21075331-21075499	-0.301	0.001128835062	0.049	1.23E-14
ASS	DPM1	-	chr20:50941104-50941209	chr20:50941128-50941209	-0.303	0.01135523264	-0.02	1
ASS	ARL6IP4	+	chr12:122981570-122981879	chr12:122981594-122981879	-0.303	5.86E-13	-0.004	1

ASS	OPTN	+	chr10:13109096-13109288	chr10:13109111-13109288	-0.305	0.02544833074	-0.058	1
ASS	PCID2	-	chr13:113177610-113178008	chr13:113177610-113177976	-0.315	1.16E-07	0.027	0.5094209076
ASS	TMEM214	+	chr2:27037789-27037892	chr2:27037814-27037892	-0.32	0.0214154521	-0.11	1
ASS	NABP1	+	chr2:191678135-191678705	chr2:191678135-191678241	-0.321	0.004867116873	-0.001	1
ASS	APTX	-	chr9:32987543-32987846	chr9:32987543-32987731	-0.325	9.48E-06	0.002	1
ASS	NDUFAF6	+	chr8:95031994-95032098	chr8:95031994-95032094	-0.327	2.95E-05	0.015	1
ASS	U2AF1L4	-	chr19:35744322-35744981	chr19:35744322-35744421	-0.329	0.01277456951	0.102	0.01436035444
ASS	RABL2B	-	chr22:50782187-50782351	chr22:50782187-50782347	-0.333	0.02937565073	0.014	1
ASS	TANGO2	+	chr22:20021068-20021246	chr22:20021068-20021134	-0.336	0.002947582078	0.021	1
ASS	PAM16	-	chr16:4341367-4341672	chr16:4341367-4341583	-0.336	3.17E-05	0.048	0.0132344605
ASS	BCS1L	+	chr2:218660306-218660482	chr2:218660306-218660421	-0.338	0.009675218226	0.054	0.9201442443
ASS	ADCY10P1	+	chr6:41108948-41109904	chr6:41109712-41109904	-0.34	0.01505331047	-0.042	1
ASS	AURKB	-	chr17:8207737-8207901	chr17:8207737-8207840	-0.343	0.007397341977	-0.132	1
ASS	U2AF1L4	-	chr19:35744322-35744738	chr19:35744322-35744421	-0.35	0.004351922584	0.106	0.00613842183
ASS	SUPT20H	-	chr13:37022010-37023044	chr13:37022010-37022317	-0.36	2.00E-06	-0.01	1
ASS	PKMYT1	-	chr16:2973137-2973253	chr16:2973137-2973215	-0.365	0.00200752727	0.074	0.0007379598445
ASS	COMMD4	+	chr15:75338061-75338420	chr15:75338061-75338133	-0.366	4.88E-11	0.041	0.01204700972
ASS	RNF126	-	chr19:651610-651855	chr19:651691-651855	-0.369	0.000119055682	0.062	0.0002124678132
ASS	MSI2	+	chr17:57674917-57675126	chr17:57674971-57675126	-0.372	0.009154824571	-0.036	0.755732217
ASS	MVD	-	chr16:88656104-88656561	chr16:88656104-88656304	-0.376	0.01930354929	-0.011	0.1256515688
ASS	SH3BP2	+	chr4:2831922-2832412	chr4:2832330-2832412	-0.38	0.01237140713	0.003	1
ASS	RNF32	+	chr7:156676294-156677130	chr7:156676418-156677130	-0.385	0.002902268724	0.184	1
ASS	PKMYT1	-	chr16:2973137-2973445	chr16:2973137-2973215	-0.386	4.25E-07	0.055	6.18E-06
ASS	YPEL5	+	chr2:30155814-30155910	chr2:30155849-30155910	-0.389	0.01033981784	0.012	1
ASS	PRKAG1	-	chr12:49005461-49005592	chr12:49005461-49005543	-0.39	0.01015531981	0.104	0.305961467
ASS	LRRC75B	-	chr22:24588213-24589393	chr22:24588213-24588329	-0.394	0.0009628756991	0.062	0.1959219425
ASS	MINK1	+	chr17:4890221-4890735	chr17:4890516-4890735	-0.395	0.004661268084	-0.039	0.04915354714
ASS	KAT6B	+	chr10:74975398-74976330	chr10:74975398-74975781	-0.395	0.0009030718475	0.082	1
ASS	ZNF286A	+	chr17:15700047-15700366	chr17:15700134-15700366	-0.398	0.00692806339	-0.06	1
ASS	DDX51	-	chr12:132141102-132141420	chr12:132141274-132141420	-0.411	2.38E-06	-0.006	0.8632958686
ASS	C19orf60	+	chr19:18588994-18589683	chr19:18588994-18589077	-0.412	1.58E-07	0.013	0.1819771004
ASS	IP6K2	-	chr3:48715314-48715527	chr3:48715388-48715527	-0.414	0.03593330065	0.006	1
ASS	DGKZ	+	chr11:46368001-46368079	chr11:46368004-46368079	-0.423	0.0004772283099	-0.012	1
ASS	PLK3	+	chr1:44804338-44804779	chr1:44804338-44804501	-0.426	0.0003250196594	-0.002	1
ASS	NDUFS5	+	chr1:39026317-39026402	chr1:39026317-39026398	-0.427	0	0.034	0.2769346244
ASS	CPM	-	chr12:68933141-68933199	chr12:68933169-68933199	-0.431	0.01433723906	-0.011	1
ASS	NOL8	-	chr9:92324905-92325350	chr9:92325317-92325350	-0.431	6.68E-05	-0.076	1
ASS	CPM	-	chr12:68933141-68933202	chr12:68933159-68933202	-0.432	5.28E-05	-0.003	1
ASS	C9orf91	+	chr9:114624135-114624459	chr9:114624349-114624459	-0.432	0.0004438203268	-0.034	1
ASS	MFF	+	chr2:227328677-227328899	chr2:227328677-227328789	-0.434	2.24E-06	0.008	1
ASS	PHB2	-	chr12:6969955-6970068	chr12:6969955-6970063	-0.437	0.0001139561149	-0.001	1
ASS	TOP3B	-	chr22:21975904-21976187	chr22:21975980-21976187	-0.44	0.001218214733	-0.038	1
ASS	CORO7-PAI	-	chr16:4341367-4341583	chr16:4341367-4341504	-0.44	3.17E-05	-0.03	0.001056435653
ASS	TAZ	+	chrX:154419542-154419746	chrX:154419542-154419623	-0.441	3.46E-08	0.068	0.02235414444
ASS	PBX4	-	chr19:19570585-19570833	chr19:19570686-19570833	-0.442	0.02703202502	-0.063	1
ASS	FNBP1	-	chr9:129923843-129924026	chr9:129923843-129923996	-0.447	0.0001222988585	0.006	1
ASS	C18orf54	+	chr18:54361642-54362431	chr18:54361642-54361948	-0.45	1.03E-08	-0.002	1
ASS	SLC25A14	+	chrX:130340106-130340353	chrX:130340106-130340344	-0.453	0.0008521869982	-0.067	1
ASS	TLE2	-	chr19:3019282-3019466	chr19:3019282-3019463	-0.457	0.002391964041	-0.001	1
ASS	EPB41	+	chr1:28987430-28987905	chr1:28987447-28987905	-0.471	0.01505331047	-0.249	1
ASS	CLEC16A	+	chr16:11003073-11003305	chr16:11003073-11003257	-0.473	4.65E-05	0.083	1
ASS	C16orf93	-	chr16:30759653-30759809	chr16:30759653-30759724	-0.478	0.02067176633	-0.032	1
ASS	CCDC24	+	chr1:43993886-43993964	chr1:43993889-43993964	-0.492	0.02163235123	0.017	1
ASS	NPHP1	-	chr2:110163047-110163135	chr2:110163047-110163132	-0.498	0.003019796635	0.019	1
ASS	TKT	-	chr3:53237796-53237921	chr3:53237796-53237820	-0.501	1.07E-07	0.015	1
ASS	HLA-A	+	chr6:29944499-29944634	chr6:29944499-29944616	-0.528	0	-0.009	0.663760545
ASS	PAM16	-	chr16:4341367-4341672	chr16:4341367-4341504	-0.529	6.69E-09	-0.025	0.09247298319
ASS	ZRANB2-AS	+	chr1:71237129-71237386	chr1:71237129-71237195	-0.531	0.0004010953459	-0.109	1
ASS	OSBPL3	-	chr7:24834086-24834736	chr7:24834485-24834736	-0.543	5.35E-13	0.015	1
ASS	NABP1	+	chr2:191683289-191683804	chr2:191683728-191683804	-0.552	2.14E-10	-0.097	1
ASS	FOPNL	-	chr16:15882948-15883048	chr16:15882948-15883020	-0.557	0.0005506993828	-0.007	1
ASS	QKI	+	chr6:163565945-163566795	chr6:163566720-163566795	-0.573	0	-0.041	0.2989539018
ASS	AP4M1	+	chr7:100101879-100102393	chr7:100101879-100101989	-0.585	3.09E-06	-0.027	1

ASS	PQBP1	+	chrX:48897911-48898093	chrX:48897911-48898082	-0.587	1.43E-06	-0.015	1
ASS	SLC2A11	+	chr22:23875084-23875241	chr22:23875120-23875241	-0.59	0.004351922584	0.008	1
ASS	MGRN1	+	chr16:4686258-4690974	chr16:4688795-4690974	-0.593	3.75E-10	-0.042	8.72E-06
ASS	ASIC1	+	chr12:50076967-50077060	chr12:50076967-50077049	-0.594	0.0001836014068	0.127	1
ASS	MTX1	+	chr1:155210547-155212219	chr1:155210547-155210627	-0.6	0	0.121	3.06E-08
ASS	C5orf42	-	chr5:37139339-37142480	chr5:37142309-37142480	-0.611	9.22E-10	0.032	1
ASS	C1orf159	-	chr1:1090352-1091374	chr1:1090352-1090428	-0.65	0.01370844678	0.005	1
ASS	NEK3	-	chr13:52143914-52143987	chr13:52143915-52143987	-0.653	5.08E-05	0.222	1
ASS	TANGO2	+	chr22:20061529-20061706	chr22:20061529-20061683	-0.657	0.007874013691	-0.024	0.8148565953
ASS	NRM	-	chr6:30690024-30690243	chr6:30690046-30690243	-0.66	7.95E-05	-0.176	1
ASS	CENPV	-	chr17:16348615-16349333	chr17:16348615-16348685	-0.676	2.77E-12	0.058	0.5158640232
ASS	SH3TC2	-	chr5:149041415-149041617	chr5:149041473-149041617	-0.733	1.14E-08	0.06	1
ASS	DZANK1	-	chr20:18412645-18412853	chr20:18412645-18412835	-0.791	1.24E-12	0.025	1
MXE	ARIH2	+	chr3:48945135-48945181	chr3:48961611-48961679	0.948	0	0.02	0.2684251991
MXE	ATG13	+	chr11:46630896-46630991	chr11:46632322-46632459	0.851	2.25E-08	-0.053	1
MXE	ARIH2	+	chr3:48922747-48922811	chr3:48961611-48961679	0.828	0	-0.01	1
MXE	AGAP4	-	chr10:45841652-45841687	chr10:45844325-45844394	0.785	1.11E-09	0.034	1
MXE	RP11-33B1.	+	chr4:119528739-119528789	chr4:119550378-119550443	0.639	2.06E-07	-0.037	0.8998473395
MXE	NVL	-	chr1:224232088-224232182	chr1:224232894-224232972	0.615	0.000384726908	-0.039	1
MXE	SRGAP1	+	chr12:64091275-64091378	chr12:64091892-64091926	0.576	3.66E-10	0.247	1
MXE	DOCK9	-	chr13:98845328-98845372	chr13:98845923-98845991	0.55	4.87E-08	-0.002	1
MXE	ANKRD36	+	chr2:97198585-97198658	chr2:97200452-97200525	0.529	2.35E-05	0.06	1
MXE	FUZ	-	chr19:49812250-49812335	chr19:49812446-49812736	0.511	0.006041335787	0.014	1
MXE	PCBP1-AS1	-	chr2:70053730-70053792	chr2:70055713-70055910	0.505	1.79E-07	-0.072	0.7337601527
MXE	OSBPL6	+	chr2:178339671-178339764	chr2:178344285-178344360	0.48	0.0001659601603	0.182	1
MXE	SLC9B1	-	chr4:102949256-102949427	chr4:102989799-102989941	0.474	0.01001908489	0.108	1
MXE	ERBB2IP	+	chr5:66068876-66069020	chr5:66072168-66072291	0.469	1.14E-05	0.032	1
MXE	DOCK9	-	chr13:98845328-98845372	chr13:98845923-98846060	0.468	1.11E-09	0.004	1
MXE	TNC	-	chr9:115048259-115048532	chr9:115059729-115060002	0.464	3.13E-05	0.026	0.4060670565
MXE	DNM2	+	chr19:10796060-10796199	chr19:10797379-10797518	0.455	0	0.168	0
MXE	RAPGEF1	-	chr9:131604930-131605188	chr9:131619050-131619209	0.455	2.38E-05	0.08	1
MXE	ANXA2	-	chr15:60390212-60390317	chr15:60396150-60396427	0.449	0.04267432066	0.029	1
MXE	WASF3	+	chr13:26676548-26676724	chr13:26680034-26680201	0.44	0	-0.153	1.26E-05
MXE	RP11-421L2	+	chr1:101075394-101075716	chr1:101077362-101077508	0.439	3.74E-08	0.105	7.02E-07
MXE	NBPF26	+	chr1:120834494-120834546	chr1:120836516-120836568	0.438	0.03649123668	0.667	1
MXE	AD000671.6	-	chr19:35744625-35744738	chr19:35745124-35745212	0.435	4.44E-14	-0.083	0.002127710418
MXE	TNC	-	chr9:115048259-115048532	chr9:115059729-115060002	0.416	5.63E-05	0.021	0.7244533484
MXE	ADAP2	+	chr17:30926826-30926918	chr17:30931888-30931968	0.411	3.40E-05	0.007	1
MXE	CNOT2	+	chr12:70285399-70285586	chr12:70294236-70294294	0.41	0.001318100309	0.017	1
MXE	KLHDC1	+	chr14:49725685-49725769	chr14:49728925-49729009	0.409	0.001455592303	0.034	1
MXE	PCBP1-AS1	-	chr2:70053730-70053792	chr2:70059642-70059680	0.407	4.02E-06	0.003	1
MXE	ATAT1	+	chr6:30642767-30643011	chr6:30645894-30645974	0.4	0.0005709668944	-0.143	0
MXE	SLC26A11	+	chr17:80220918-80221115	chr17:80221547-80221794	0.399	0.0003818203054	0.015	0.9970477496
MXE	PLEKHA4	-	chr19:48839204-48839263	chr19:48841148-48841310	0.396	0.01313028342	-0.011	1
MXE	MAP4K4	+	chr2:101860824-101860986	chr2:101863820-101864051	0.393	0	0.248	0
MXE	PCBP1-AS1	-	chr2:70053730-70053792	chr2:70055655-70055910	0.392	5.67E-07	-0.04	1
MXE	POLE2	-	chr14:49646569-49646694	chr14:49646993-49647092	0.387	0.001174947873	-0.116	1
MXE	CCNYL1	+	chr2:207733946-207734047	chr2:207737410-207737446	0.386	4.44E-07	0.061	0.6835350824
MXE	RP4-717I23.	-	chr1:93315148-93315269	chr1:93318167-93318228	0.386	0.04922057688	0.035	1
MXE	PCBP1-AS1	-	chr2:70053730-70053792	chr2:70055713-70055910	0.382	1.18E-06	-0.056	1
MXE	CPSF7	-	chr11:61421053-61421127	chr11:61421191-61421257	0.378	0.01173911421	-0.014	1
MXE	FAM228B	+	chr2:24135118-24135187	chr2:24167626-24167683	0.377	0.04707829882	0.018	1
MXE	RPL9	-	chr4:39456405-39456538	chr4:39457585-39457681	0.37	0.007466712483	0.05	0.3088808078
MXE	CRLS1	+	chr20:6026170-6026238	chr20:6028671-6028725	0.369	0.01002816107	0.056	1
MXE	CAST	+	chr5:96727488-96727530	chr5:96729152-96729209	0.368	6.23E-14	-0.003	1
MXE	CCBL1	-	chr9:128846679-128846859	chr9:128847432-128847643	0.365	0.0009655421884	0.092	1
MXE	TPM2	-	chr9:35684731-35684807	chr9:35685063-35685139	0.365	8.39E-13	0.005	1
MXE	ZCWPW2	+	chr3:28478813-28478931	chr3:28492126-28492173	0.365	0.04454817012	-0.019	1
MXE	FBXO22	+	chr15:75904490-75904629	chr15:75913202-75913290	0.364	1.52E-09	0.01	1
MXE	OGDH	+	chr7:44647443-44647534	chr7:44647656-44647759	0.363	0	-0.046	1.20E-09
MXE	OGDH	+	chr7:44647443-44647534	chr7:44647656-44647759	0.358	0	-0.021	0.0001402925284
MXE	PILRB	+	chr7:100353372-100353483	chr7:100353894-100354012	0.349	1.28E-10	0.034	0.7555031296
MXE	IMMP1L	-	chr11:31460625-31460714	chr11:31473723-31473786	0.349	0.0002261884385	-0.017	1

MXE	ARIH2	+	chr3:48927461-48927813	chr3:48961611-48961679	0.349	0	-0.005	1
MXE	ARIH2	+	chr3:48927461-48927813	chr3:48961611-48961679	0.347	0	-0.005	1
MXE	TPM1	+	chr15:63061197-63061273	chr15:63061712-63061788	0.344	5.99E-12	0.08	1.35E-05
MXE	FBXO22	+	chr15:75904490-75904629	chr15:75913202-75913290	0.343	4.51E-08	0.01	1
MXE	HMGN1	-	chr21:39345829-39345958	chr21:39347378-39347483	0.323	0	0.023	1
MXE	CPSF7	-	chr11:61421053-61421130	chr11:61421191-61421257	0.32	0.0423514077	-0.034	1
MXE	PKD1P6	-	chr16:15125690-15125807	chr16:15127469-15127596	0.32	0.01727736852	0.003	1
MXE	HMGN1	-	chr21:39345829-39345958	chr21:39347378-39347478	0.316	0	0.01	1
MXE	IFT88	+	chr13:20625749-20625849	chr13:20631015-20631102	0.315	2.93E-06	-0.034	1
MXE	ARIH2	+	chr3:48927461-48927813	chr3:48964918-48964982	0.314	0	-0.007	1
MXE	LINC01184	-	chr5:127956803-127956921	chr5:128061143-128061244	0.313	8.49E-05	-0.032	0.291262272
MXE	FAM228B	+	chr2:24146929-24147086	chr2:24167626-24167683	0.308	0.008954576075	0.007	1
MXE	EPB41L2	-	chr6:130870327-130870381	chr6:130876673-130876775	0.306	0.002539822157	0.163	7.51E-11
MXE	PIGP	-	chr21:37069551-37069624	chr21:37072144-37072310	0.304	6.45E-07	0.012	0.8697005245
MXE	FAM208B	+	chr10:5714134-5714207	chr10:5717658-5717750	0.298	2.88E-06	-0.034	1
MXE	IKZF4	+	chr12:56019317-56019415	chr12:56023670-56023764	0.295	0.03038613536	0.106	1
MXE	CDKL3	-	chr5:134308573-134308727	chr5:134312291-134312380	0.292	0.02648226135	-0.01	0.9363930543
MXE	CDKL3	-	chr5:134308573-134308727	chr5:134312291-134312380	0.291	0.02508081161	-0.008	1
MXE	MDM4	+	chr1:204526359-204526434	chr1:204537458-204537497	0.286	0.0001261302674	-0.024	1
MXE	PPIL3	-	chr2:200882341-200882382	chr2:200885261-200885343	0.286	0.004322405941	-0.021	0.1276900912
MXE	SPAST	+	chr2:32098795-32098891	chr2:32114637-32114825	0.281	5.89E-08	0.108	4.52E-10
MXE	PICALM	-	chr11:85983865-85983973	chr11:85990249-85990399	0.281	0	-0.014	0.00188858072
MXE	RFWD2	-	chr1:176136487-176136547	chr1:176149005-176149074	0.28	0.005073756977	0.003	1
MXE	ALG13	+	chrX:111689086-111689197	chrX:111708964-111709048	0.279	1.16E-05	0.041	1
MXE	AC005154.6	-	chr7:30576563-30576710	chr7:30584710-30584808	0.278	0.02127345004	0.017	1
MXE	NASP	+	chr1:45594693-45594773	chr1:45600384-45600457	0.277	0.03478906968	-0.02	0.7249845197
MXE	DAG1	+	chr3:49470964-49471049	chr3:49476848-49476905	0.277	0.03468750359	0.037	1
MXE	PPIL3	-	chr2:200882341-200882382	chr2:200885261-200885367	0.277	0.03684497474	-0.025	0.6695285476
MXE	LSM14B	+	chr20:62126225-62126439	chr20:62127584-62127701	0.276	0.000694920693	0.096	1.02E-05
MXE	SFI1	+	chr22:31604308-31604404	chr22:31604868-31604945	0.276	0.0008459114573	-0.017	1
MXE	SLC29A1	+	chr6:44227262-44227342	chr6:44229389-44229471	0.274	4.53E-11	-0.03	1.01E-06
MXE	GAS5	-	chr1:173865509-173865547	chr1:173865856-173866206	0.274	0	-0.013	0.9066165008
MXE	DCUN1D4	+	chr4:51891759-51891851	chr4:51899269-51899378	0.271	9.22E-05	0.008	1
MXE	USP45	-	chr6:99445796-99446463	chr6:99464603-99464747	0.271	4.26E-07	-0.023	1
MXE	CTU2	+	chr16:88707135-88707210	chr16:88709937-88710016	0.271	0.005693884603	-0.01	1
MXE	FAM228B	+	chr2:24146929-24147086	chr2:24164197-24164335	0.271	0.01854545969	0.012	1
MXE	FAM228B	+	chr2:24146929-24147086	chr2:24164197-24164335	0.263	0.01231089299	0.008	1
MXE	ACTN4	+	chr19:38710256-38710342	chr19:38711275-38711361	0.262	4.35E-11	0.252	0
MXE	TMEM241	-	chr18:23368714-23368750	chr18:23370215-23370261	0.262	0.0380235146	-0.005	1
MXE	FBXL2	+	chr3:33297868-33297962	chr3:33359282-33359357	0.262	0.01098908084	-0.006	1
MXE	ZMAT1	-	chrX:101887197-101887304	chrX:101895813-101895882	0.262	0.001875902704	-0.058	1
MXE	FAM228B	+	chr2:24146929-24147086	chr2:24167626-24167683	0.261	0.0408781115	0	1
MXE	N4BP2	+	chr4:40097226-40097569	chr4:40100035-40100126	0.26	0.01590946898	0.164	1
MXE	NFIC	+	chr19:3381711-3382243	chr19:3425105-3425177	0.258	0.0002641670792	0.011	1
MXE	SUN1	+	chr7:843340-843520	chr7:848417-848615	0.258	0.0001492596994	-0.075	8.72E-07
MXE	SNHG17	-	chr20:38422091-38422241	chr20:38425934-38426052	0.256	5.21E-09	0.014	1
MXE	ZNRD1-AS1	-	chr6:30021666-30021761	chr6:30034907-30035110	0.253	0.0004121135196	0.062	1
MXE	SPAG9	-	chr17:50975862-50975901	chr17:50977107-50977221	0.251	0	-0.001	1
MXE	VEGFA	+	chr6:43778459-43778536	chr6:43782010-43782087	0.25	2.69E-06	0.022	0.854414688
MXE	C2orf76	-	chr2:119317465-119317503	chr2:119321153-119321204	0.248	0.04224843685	-0.043	0.2210888422
MXE	GSN	+	chr9:121281469-121281562	chr9:121300055-121300126	0.247	2.72E-12	0.022	3.32E-06
MXE	RABL2B	-	chr22:50769454-50769552	chr22:50775771-50775851	0.246	0.006656664683	0.005	1
MXE	UBE3A	-	chr15:25407066-25407137	chr15:25408619-25408684	0.246	0.006838125131	-0.011	1
MXE	EIF4G3	-	chr1:20997600-20997633	chr1:21001198-21001312	0.242	1.39E-09	0.052	1
MXE	PRDM2	+	chr1:13773077-13773188	chr1:13778417-13782831	0.242	3.35E-09	0.021	1
MXE	ANKS3	-	chr16:4726658-4726780	chr16:4726978-4727177	0.242	0.04053130872	-0.013	1
MXE	SUZ12P1	+	chr17:30743300-30743369	chr17:30759490-30759539	0.242	8.81E-07	0.004	1
MXE	AUP1	-	chr2:74529131-74529282	chr2:74529361-74529499	0.241	6.67E-09	0	1
MXE	MSRB3	+	chr12:65308528-65308655	chr12:65326825-65326934	0.241	0.0416374328	0	1
MXE	ACTN4	+	chr19:38727945-38728026	chr19:38728315-38728381	0.24	0	0.086	0
MXE	TMEM161B	-	chr5:88228444-88228528	chr5:88240812-88240916	0.238	1.82E-06	0.001	1
MXE	AUP1	-	chr2:74529131-74529282	chr2:74529361-74529499	0.237	1.56E-08	-0.002	1
MXE	C17orf62	-	chr17:82447579-82447621	chr17:82449173-82449292	0.236	0.003734037699	0.003	1

MXE	HSCB	+	chr22:28743881-28743923	chr22:28751240-28751288	0.233	0.01546534984	0.029	0.859825515
MXE	GAS5	-	chr1:173865509-173865547	chr1:173866176-173866206	0.233	0	-0.008	1
MXE	SEC24B	+	chr4:109481676-109481781	chr4:109491326-109491407	0.23	6.93E-08	-0.058	0.0007635296406
MXE	POP4	+	chr19:29610408-29610457	chr19:29611861-29611939	0.229	0.007333096958	0.015	0.9668803811
MXE	PIGF	-	chr2:46588074-46588179	chr2:46591802-46591925	0.224	0.004271075024	-0.094	6.72E-05
MXE	LRP8	-	chr1:53275630-53275753	chr1:53280586-53280715	0.224	0.0304034326	0.032	0.8275904879
MXE	TMEM161B-	+	chr5:88287435-88287622	chr5:88410072-88410194	0.224	0.0129113373	0.101	0.0003093883312
MXE	CENPO	+	chr2:24793851-24793965	chr2:24799674-24799844	0.222	3.30E-09	0.002	1
MXE	DCTN6	+	chr8:30164110-30164175	chr8:30175084-30175190	0.221	0.01355535248	0.016	0.9854573988
MXE	NBPF9	-	chr1:149058924-149059097	chr1:149062092-149062265	0.221	2.56E-05	0.02	0.3997409494
MXE	MKKS	-	chr20:10412529-10413931	chr20:10420527-10420758	0.218	3.79E-12	0.027	0.01347495685
MXE	ELOVL5	-	chr6:53287861-53287942	chr6:53290094-53290189	0.217	0.0009267743125	-0.108	1
MXE	PDE9A	+	chr21:42731769-42731949	chr21:42732069-42732124	0.216	0.004302566949	0.028	0.1385328075
MXE	RP11-421L2	+	chr1:101075394-101075716	chr1:101083460-101083574	0.215	0.03685287948	0.014	1
MXE	FBXL2	+	chr3:33297868-33297962	chr3:33364624-33364719	0.214	0.001031064798	-0.014	1
MXE	GAS5	-	chr1:173865509-173865547	chr1:173866527-173866567	0.214	6.74E-05	-0.012	0.9763670398
MXE	ZGRF1	-	chr4:112589723-112589874	chr4:112603523-112603697	0.213	0.0003964893019	-0.05	1
MXE	MTCL1	+	chr18:8806892-8807060	chr18:8812978-8813233	0.212	0.0002977314871	0.033	1
MXE	ZGRF1	-	chr4:112589723-112589874	chr4:112603523-112603697	0.211	0.0006512876139	-0.055	1
MXE	ASPH	-	chr8:61618977-61619019	chr8:61633682-61633727	0.21	2.91E-07	-0.055	0.000493692975
MXE	ANKRD27	-	chr19:32605834-32605954	chr19:32607634-32607832	0.209	0.01128144421	-0.017	1
MXE	CKLF	+	chr16:66558189-66558348	chr16:66563121-66563217	0.209	1.40E-11	0.04	1.02E-05
MXE	ZGRF1	-	chr4:112589723-112589874	chr4:112603523-112603697	0.206	0.0001070343972	-0.064	1
MXE	C11orf80	+	chr11:66788159-66788269	chr11:66796280-66796364	0.206	0.0005733001896	-0.025	1
MXE	GAS5	-	chr1:173864674-173864704	chr1:173865228-173865282	0.206	5.01E-11	-0.009	1
MXE	RPL21	+	chr13:27253764-27253843	chr13:27255241-27255354	0.205	0.006823004384	0.001	1
MXE	RPL21	+	chr13:27253764-27253843	chr13:27255241-27255349	0.201	0.006700381109	0.007	1
MXE	SLMAP	+	chr3:57907883-57908006	chr3:57909075-57909150	0.2	0.000440024471	0	1
MXE	ZC3H11A	+	chr1:203796450-203796601	chr1:203801574-203801628	0.199	0.03537717391	0.089	1
MXE	PGAP3	-	chr17:39676613-39676755	chr17:39684596-39684749	0.199	0.007333096958	0.008	0.02862868358
MXE	COA1	-	chr7:43656032-43656153	chr7:43665657-43665722	0.198	1.72E-06	-0.025	0.8598337163
MXE	PCBP1-AS1	-	chr2:70018014-70018131	chr2:70051202-70051305	0.198	0.04071530613	-0.019	1
MXE	NBPF12	+	chr1:146987953-146988126	chr1:146989573-146989746	0.198	0.0001456783967	0	1
MXE	PRKRIP1	+	chr7:102395976-102396537	chr7:102397619-102397698	0.196	0.0001029012069	-0.018	0.1966846816
MXE	RP11-73M1[illegible]	+	chr14:103574106-103574170	chr14:103587273-103587364	0.194	4.96E-08	0.039	0.1026254872
MXE	CFLAR	+	chr2:201140356-201140439	chr2:201145377-201145432	0.193	5.62E-06	-0.007	1
MXE	ZFAND5	-	chr9:72359417-72359521	chr9:72360109-72360221	0.191	1.68E-12	-0.006	1
MXE	KNSTRN	+	chr15:40383227-40383322	chr15:40386404-40386494	0.19	3.93E-05	0.003	1
MXE	TNFSF12-TI	+	chr17:7559623-7559702	chr17:7559845-7559893	0.19	0.006396217123	-0.011	1
MXE	MPHOSPH9	-	chr12:123226287-123226351	chr12:123227545-123227616	0.189	6.55E-07	0.039	1
MXE	MPV17	-	chr2:27312993-27313074	chr2:27322447-27322522	0.188	3.87E-06	-0.015	0.442229027
MXE	CCDC15	+	chr11:124993168-124993243	chr11:125003866-125003959	0.188	0.0004781924829	0.007	1
MXE	TNC	-	chr9:115048259-115048532	chr9:115064646-115064919	0.187	0.03274388609	0.042	0.2240421397
MXE	XKR9	+	chr8:70674817-70674899	chr8:70680780-70681330	0.185	0.002440740444	-0.053	1
MXE	GIT2	-	chr12:109947255-109947504	chr12:109951211-109951316	0.184	0.0005428233416	0.004	1
MXE	ACOX1	-	chr17:75960214-75960375	chr17:75973624-75973785	0.184	0.0006007034903	-0.018	0.1839333804
MXE	ZNF83	-	chr19:52635065-52635153	chr19:52655560-52655687	0.184	0.008711948191	-0.046	0.238757624
MXE	ARIH2	+	chr3:48922747-48922811	chr3:48945135-48945181	0.183	0.0001227177574	-0.042	0.4385128411
MXE	PCBP1-AS1	-	chr2:70059642-70059680	chr2:70083506-70083687	0.183	0.01562606437	0.016	1
MXE	MFF	+	chr2:227347225-227347384	chr2:227352513-227352573	0.182	0.0003367841868	0.056	5.72E-05
MXE	MPHOSPH9	-	chr12:123226287-123226351	chr12:123227462-123227572	0.18	1.39E-06	0.037	1
MXE	GRB10	-	chr7:50710874-50710990	chr7:50755886-50756056	0.18	0.03407818423	-0.003	1
MXE	ACSL3	+	chr2:222887829-222887888	chr2:222900673-222900780	0.18	0.001419689146	0.043	1
MXE	POP5	-	chr12:120579513-120579597	chr12:120581114-120581257	0.18	0.004097329756	-0.003	1
MXE	NUDT17	+	chr1:145846012-145846196	chr1:145846597-145846690	0.18	0.004181133627	0.034	1
MXE	RNF19B	-	chr1:32942251-32942459	chr1:32944018-32944159	0.179	0.03033891155	0.005	1
MXE	ABI1	-	chr10:26755654-26755741	chr10:26759061-26759235	0.179	0.001001354063	0.049	0.004433906038
MXE	WNK1	+	chr12:879572-880031	chr12:880720-880999	0.178	3.96E-05	-0.023	1
MXE	SPAG1	+	chr8:100213818-100213918	chr8:100220278-100220431	0.178	0.03855224577	-0.018	1
MXE	ST7L	-	chr1:112583971-112584126	chr1:112591524-112591603	0.177	0.03649123668	-0.005	1
MXE	ALG13	+	chrX:111689086-111689197	chrX:111708026-111708393	0.177	2.24E-06	0.011	1
MXE	NT5C3A	-	chr7:33024038-33024108	chr7:33026816-33026915	0.177	0.04140530147	-0.01	1
MXE	CAST	+	chr5:96722638-96722698	chr5:96729152-96729209	0.177	0.00121263946	0.035	0.4484738992

MXE	ZGRF1	-	chr4:112586444-112586583	chr4:112603523-112603697	0.176	0.01943201436	0.022	1
MXE	ANKRD39	-	chr2:96848268-96848444	chr2:96853400-96853604	0.176	0.03138932416	-0.002	1
MXE	RCL1	+	chr9:4826857-4827033	chr9:4833153-4833228	0.175	0.006396217123	-0.019	1
MXE	ACLY	-	chr17:41896619-41896649	chr17:41897748-41897839	0.174	2.86E-05	-0.022	0.0470832219
MXE	METTL6	-	chr3:15414020-15414162	chr3:15415497-15415583	0.174	0.02355341548	-0.024	1
MXE	FAM122B	-	chrX:134781820-134781917	chrX:134785885-134785947	0.173	0.003764948527	-0.016	1
MXE	COG7	-	chr16:23418699-23418827	chr16:23424748-23424947	0.173	0.04308153903	0	1
MXE	DGUOK	+	chr2:73946718-73946906	chr2:73950584-73950732	0.172	0.0001474551712	0.057	2.75E-10
MXE	MTHFD1L	+	chr6:150887841-150887981	chr6:150905649-150905761	0.172	0.001134691147	0.035	0.3570144628
MXE	KIF21A	-	chr12:39315931-39315970	chr12:39318072-39318201	0.172	2.61E-06	0.125	2.28E-07
MXE	AKIP1	+	chr11:8911443-8911671	chr11:8914825-8914930	0.172	0.0304034326	0.012	0.9920590038
MXE	NUTM2A-AS	-	chr10:87246114-87246212	chr10:87288441-87288495	0.172	1.51E-05	-0.074	0.3277942775
MXE	HAGH	-	chr16:1819114-1819223	chr16:1819896-1820014	0.171	1.72E-06	0.003	1
MXE	DBR1	-	chr3:138163777-138163858	chr3:138167080-138167305	0.171	0.01047188361	-0.048	0.1460140654
MXE	GLS	+	chr2:190913192-190913364	chr2:190920214-190920248	0.17	0.0003834698295	0.058	1
MXE	ABI1	-	chr10:26755654-26755741	chr10:26759061-26759238	0.17	0.0004781924829	0.05	0.0005222918194
MXE	COMMD4	+	chr15:75338061-75338133	chr15:75338354-75338420	0.17	3.33E-07	-0.017	0.0001138023157
MXE	OGDH	+	chr7:44645326-44645518	chr7:44647656-44647759	0.168	0.002859687472	-0.01	1
MXE	ZNF655	+	chr7:99561887-99561973	chr7:99562367-99562494	0.168	0.01140485557	0.003	1
MXE	HJURP	-	chr2:233845727-233845820	chr2:233847396-233847461	0.167	0.0009825921946	0.042	0.9535636341
MXE	RELA	-	chr11:65661686-65661835	chr11:65661936-65662088	0.167	0.002859687472	0.006	1
MXE	PGAP2	+	chr11:3823612-3823740	chr11:3823882-3824135	0.166	9.22E-05	-0.018	4.55E-07
MXE	ARIH2	+	chr3:48924717-48924839	chr3:48945135-48945181	0.166	1.09E-05	-0.031	0.6250065823
MXE	PCBP1-AS1	-	chr2:70053730-70053792	chr2:70083506-70083687	0.166	6.87E-06	0.009	1
MXE	PCBP1-AS1	-	chr2:70018014-70018131	chr2:70051202-70051305	0.165	0.04040093942	-0.022	1
MXE	PPP4C	+	chr16:30081258-30081310	chr16:30082483-30082534	0.164	0.005808692927	0.029	0.001191643273
MXE	CFLAR	+	chr2:201140356-201140439	chr2:201149002-201149052	0.163	2.56E-05	-0.026	0.6534637752
MXE	MYEF2	-	chr15:48151472-48151571	chr15:48151873-48151942	0.163	0.001055792768	-0.024	0.8311654373
MXE	DGUOK	+	chr2:73946718-73946906	chr2:73957124-73957240	0.163	3.19E-05	0.067	2.63E-14
MXE	MBNL1	+	chr3:152455541-152455577	chr3:152456266-152456361	0.163	0.01194955016	-0.004	1
MXE	RANBP3	-	chr19:5931418-5931531	chr19:5932451-5932544	0.161	0.03047068983	0.002	1
MXE	C5orf45	-	chr5:179847977-179848066	chr5:179853196-179853276	0.159	0.004780932254	-0.003	0.443059214
MXE	DGUOK	+	chr2:73946718-73946906	chr2:73957124-73957240	0.158	0.0002387901891	0.079	0
MXE	BTF3L4	+	chr1:52064824-52064938	chr1:52083339-52083541	0.158	0.0002261884385	0.007	1
MXE	DGUOK	+	chr2:73938909-73939022	chr2:73950584-73950732	0.157	0.00925802358	0.082	0
MXE	NSMF	-	chr9:137453055-137453180	chr9:137453730-137453820	0.157	0.0007803381414	0.003	1
MXE	TEX22	+	chr14:105445417-105445511	chr14:105449358-105449409	0.156	0.0001522249229	-0.038	0.2537068609
MXE	H2AFY	-	chr5:135350822-135350913	chr5:135352945-135353045	0.155	2.56E-09	-0.054	0.1211480961
MXE	PIGP	-	chr21:37069551-37069624	chr21:37072144-37072310	0.155	7.30E-07	0.008	0.3247655327
MXE	LETMD1	+	chr12:51052091-51052207	chr12:51056143-51056245	0.154	0.03327346733	0.034	0.226484228
MXE	UBE3A	-	chr15:25407066-25407262	chr15:25408619-25408684	0.154	0.01414466032	-0.011	1
MXE	GAS5	-	chr1:173865509-173865547	chr1:173865856-173865894	0.154	2.88E-11	-0.01	1
MXE	RP4-769N1:	+	chrX:102601879-102601977	chrX:102639399-102639533	0.154	0.02227107106	-0.011	1
MXE	ACOT8	-	chr20:45848449-45848675	chr20:45855158-45855292	0.153	0.01128144421	0.023	0.03473123519
MXE	CEP85L	-	chr6:118491685-118491865	chr6:118511297-118511415	0.153	0.04132591343	0.034	1
MXE	STAU1	-	chr20:49165996-49166285	chr20:49174194-49174269	0.153	8.71E-08	0.047	0.00166323334
MXE	PPIL3	-	chr2:200882341-200882435	chr2:200885261-200885367	0.153	1.17E-05	-0.023	0.09285044144
MXE	ATP2B1	-	chr12:89627677-89627716	chr12:89630504-89630645	0.152	0.007378547302	0.008	1
MXE	DLG1	-	chr3:197149742-197149796	chr3:197161639-197161738	0.152	0.000188737718	0.008	1
MXE	EED	+	chr11:86257514-86257596	chr11:86264171-86264263	0.151	0.003132359724	0.001	1
MXE	FAM49B	-	chr8:129904498-129904585	chr8:129970942-129970995	0.151	0.001100968158	0.128	1.82E-10
MXE	MFF	+	chr2:227347225-227347384	chr2:227352513-227352573	0.151	0.007663048107	0.048	0.0001226727659
MXE	HMGN1	-	chr21:39348291-39348339	chr21:39348421-39348577	0.151	0.03247308498	-0.006	0.2194692576
MXE	DLST	+	chr14:74882590-74882624	chr14:74885585-74885634	0.149	0.01029295253	0.02	1
MXE	TTC13	-	chr1:230936157-230936264	chr1:230939385-230939496	0.149	4.98E-05	-0.069	0.009095902606
MXE	AP2A2	+	chr11:977094-977224	chr11:981197-981299	0.149	0.003517278317	0.005	1
MXE	MAPT	+	chr17:45971858-45971945	chr17:45978374-45978440	0.149	0.0005296925756	0.056	2.63E-14
MXE	BCL2L13	+	chr22:17683213-17683321	chr22:17702242-17702386	0.148	0.0002387901891	-0.011	1
MXE	PCBP1-AS1	-	chr2:70053730-70053792	chr2:70083537-70083687	0.148	5.92E-06	0.016	0.6302690865
MXE	IRF3	-	chr19:49660712-49660828	chr19:49661947-49662328	0.146	0.02026289817	0.009	1
MXE	SNX14	-	chr6:85549722-85549879	chr6:85557975-85558060	0.146	0.01475123239	0.006	1
MXE	FAM49B	-	chr8:129879596-129879789	chr8:129896748-129896854	0.146	0.01128144421	-0.042	1
MXE	NSMF	-	chr9:137452735-137452819	chr9:137453055-137453180	0.146	4.68E-06	0.004	1

MXE	PCNX	+	chr14:70988566-70988699	chr14:71009633-71009724	0.145	0.01152016078	0.002	1
MXE	HJURP	-	chr2:233844204-233844283	chr2:233849762-233849859	0.145	0.0003822624243	0.005	1
MXE	C6orf52	-	chr6:10683186-10683232	chr6:10686965-10687164	0.145	0.02470719013	0.014	1
MXE	TMEM14B	+	chr6:10749201-10749268	chr6:10770076-10770204	0.143	0.04701157616	0.043	0.03497287862
MXE	TMEM161A	-	chr19:19133129-19133210	chr19:19134783-19134887	0.142	0.002295330497	0.003	1
MXE	MTHFD1L	+	chr6:150887844-150887981	chr6:150905649-150905761	0.142	0.008350531521	0.027	0.7920307084
MXE	DHTKD1	+	chr10:12088985-12089255	chr10:12091512-12091684	0.142	0.03547198855	-0.001	1
MXE	ATG10	+	chr5:82164398-82164537	chr5:82178489-82178587	0.141	0.04224761506	0.019	1
MXE	PIGP	-	chr21:37067261-37067380	chr21:37072144-37072310	0.141	0.0001179796604	0.008	0.9584830328
MXE	PHF7	+	chr3:52422221-52422338	chr3:52422759-52422881	0.14	0.00222168987	0.013	1
MXE	ZRANB3	-	chr2:135390801-135390820	chr2:135504328-135504496	0.14	0.0369310243	0.015	1
MXE	CAPN7	+	chr3:15240753-15240853	chr3:15245525-15245671	0.139	0.03674380634	-0.021	0.7925114743
MXE	POLL	-	chr10:101582762-101582891	chr10:101585861-101586156	0.139	0.03049002289	0.008	1
MXE	PICALM	-	chr11:85983865-85983973	chr11:85990249-85990378	0.138	0	-0.007	0.3621489362
MXE	RAD1	-	chr5:34911553-34911812	chr5:34913469-34913578	0.137	8.28E-06	0.014	1
MXE	LRRCC1	+	chr8:85115374-85115584	chr8:85124791-85124939	0.137	0.004174444603	0.038	0.8759998953
MXE	CIZ1	-	chr9:128187862-128187934	chr9:128190328-128190444	0.137	0.0168551311	0.005	1
MXE	EPS8	-	chr12:15670855-15670923	chr12:15681225-15681302	0.137	0.02834580514	0.02	1
MXE	CRELD2	+	chr22:49923233-49923317	chr22:49924359-49924455	0.137	0.02184438566	0.005	1
MXE	ARPC1B	+	chr7:99376432-99376574	chr7:99377357-99377455	0.136	0.001794772579	-0.003	0.05702319254
MXE	PMS2	-	chr7:5978595-5978696	chr7:5982823-5982991	0.135	0.03417749302	-0.024	1
MXE	LDLR	+	chr19:11123173-11123344	chr19:11128007-11128085	0.134	0.008448894889	0.009	1
MXE	CASK	-	chrX:41555599-41555635	chrX:41557031-41557100	0.134	0.001407486282	-0.043	0.0001035333759
MXE	GRB10	-	chr7:50732271-50732368	chr7:50755886-50756056	0.133	0.003775870552	0.009	1
MXE	DGUOK	+	chr2:73938909-73939022	chr2:73957124-73957240	0.133	0.00191291694	0.069	0
MXE	THUMPD2	-	chr2:39768900-39769093	chr2:39769709-39770119	0.133	2.96E-06	0.012	1
MXE	SH3GLB2	-	chr9:129010669-129010693	chr9:129012235-129012298	0.133	0.02873734411	0.039	0.164683156
MXE	RBM6	+	chr3:50048244-50048319	chr3:50054334-50054395	0.131	0.000205403459	-0.011	1
MXE	DYNC1I2	+	chr2:171690146-171690263	chr2:171692776-171692894	0.13	0.001121487013	-0.02	0.3455800053
MXE	MDC1	-	chr6:30707565-30708357	chr6:30711411-30711504	0.13	0.001143797693	0.015	0.946859693
MXE	ACP1	+	chr2:272036-272150	chr2:272191-272305	0.13	0.01002816107	-0.006	1
MXE	KRIT1	-	chr7:92235402-92236542	chr7:92237666-92237759	0.128	0.006841950359	-0.004	1
MXE	MELK	+	chr9:36589535-36589652	chr9:36594627-36594771	0.128	0.004831103709	-0.032	1
MXE	STRN3	-	chr14:30913523-30913657	chr14:30929200-30929311	0.128	0.0001502871092	-0.005	0.9587829415
MXE	MELK	+	chr9:36589535-36589652	chr9:36594627-36594771	0.127	0.01013902407	-0.038	1
MXE	LETMD1	+	chr12:51052091-51052207	chr12:51055834-51056021	0.126	0.007378020695	0.034	0.04226380702
MXE	ASCC3	-	chr6:100848147-100848707	chr6:100864063-100864214	0.126	0.01102991524	0.008	1
MXE	P4HTM	+	chr3:49004860-49005046	chr3:49005776-49005867	0.126	0.03840447679	-0.003	1
MXE	POLL	-	chr10:101582762-101582891	chr10:101584601-101584919	0.125	0.02262017329	0.014	1
MXE	P4HTM	+	chr3:49004860-49005046	chr3:49005717-49005867	0.125	0.03416242048	0.005	1
MXE	QKI	+	chr6:163534981-163535125	chr6:163561981-163562069	0.124	2.92E-06	0.013	0.7253696346
MXE	ESYT2	-	chr7:158737047-158737179	chr7:158739022-158739121	0.124	0.0006139817884	-0.007	1
MXE	DCTN6	+	chr8:30164110-30164175	chr8:30175084-30175190	0.123	2.03E-07	0.006	1
MXE	MPDZ	-	chr9:13113930-13114021	chr9:13115247-13115334	0.123	0.04158436659	0.005	1
MXE	SCP2	+	chr1:52948008-52948080	chr1:52950754-52950886	0.123	0.04701157616	0.002	1
MXE	C6orf52	-	chr6:10683186-10683232	chr6:10686965-10687164	0.123	0.03336528488	-0.015	1
MXE	DCXR	-	chr17:82036383-82036448	chr17:82036543-82036643	0.123	0.007049774526	-0.009	0.865728919
MXE	DPY19L3	+	chr19:32467473-32467626	chr19:32468730-32468813	0.123	0.002975638097	-0.009	0.01860732592
MXE	GTSE1	+	chr22:46326435-46326654	chr22:46328687-46328889	0.122	0.006468149765	-0.005	1
MXE	HMGN1	-	chr21:39347246-39347292	chr21:39347378-39347478	0.122	0.02072749562	-0.026	1
MXE	POMGNT1	-	chr1:46194552-46194651	chr1:46194843-46194961	0.121	0.003915883207	-0.022	0.0896573496
MXE	TPM3	-	chr1:154171412-154171488	chr1:154172028-154172104	0.121	0	0.146	0
MXE	DLG1	-	chr3:197076585-197076685	chr3:197081050-197081117	0.12	0.004195565611	-0.032	0.6917497094
MXE	XPO1	-	chr2:61525491-61526521	chr2:61533771-61533903	0.12	0	-0.004	1
MXE	KRTCAP2	-	chr1:155169790-155169857	chr1:155172564-155172892	0.12	6.32E-05	0.005	1
MXE	MARK3	+	chr14:103491776-103492034	chr14:103498501-103498528	0.119	8.37E-10	0.031	0.0001760395163
MXE	HMG20B	+	chr19:3575539-3575660	chr19:3576260-3576307	0.118	0.001067898129	-0.003	1
MXE	EED	+	chr11:86250295-86250448	chr11:86264171-86264263	0.118	4.38E-11	-0.007	1
MXE	C1orf50	+	chr1:42767508-42767624	chr1:42773562-42773649	0.118	0.0002590266131	0.029	0.2873861516
MXE	MPHOSPH9	-	chr12:123226287-123226351	chr12:123227462-123227616	0.117	9.96E-07	0.021	1
MXE	NOP56	+	chr20:2655933-2656034	chr20:2656400-2656549	0.117	1.38E-07	0.015	0.2786831776
MXE	SLC6A8	+	chrX:153693904-153694017	chrX:153694203-153694267	0.117	0.04184520844	0.009	1
MXE	POP5	-	chr12:120579773-120579923	chr12:120580995-120581257	0.117	3.96E-05	-0.002	1

MXE	HLA-C	-	chr6:31270209-31270485	chr6:31271072-31271331	0.117	5.70E-06	0.019	1
MXE	ALG13	+	chrX:111708964-111709048	chrX:111711674-111711725	0.116	0.03083217036	-0.028	1
MXE	PLAT	-	chr8:42185080-42185158	chr8:42187397-42187572	0.116	0.009587266545	-0.002	1
MXE	UBA2	+	chr19:34430575-34430659	chr19:34431860-34431931	0.116	0.0003332621248	-0.03	0.01524426996
MXE	RABEPK	+	chr9:125220538-125220700	chr9:125227909-125228059	0.116	0.004332221339	0.021	0.1005484389
MXE	GUSB	-	chr7:65976014-65976202	chr7:65979833-65979911	0.116	0.02072749562	0.014	1
MXE	ARPC1B	+	chr7:99376432-99376574	chr7:99377337-99377455	0.114	0.003775870552	0	0.06377427603
MXE	CENPO	+	chr2:24793851-24793965	chr2:24799674-24799844	0.114	0.0008017307644	-0.002	1
MXE	HNRNPH1	-	chr5:179620891-179621397	chr5:179623036-179623164	0.114	2.46E-06	-0.013	0.7854809476
MXE	TALDO1	+	chr11:755878-756002	chr11:759008-759057	0.114	0.01128144421	0.005	1
MXE	PMS2	-	chr7:5982823-5982991	chr7:5986758-5987620	0.113	0.0002590266131	0.003	1
MXE	ETNK1	+	chr12:22671271-22671355	chr12:22673499-22673660	0.112	0.008574289804	-0.001	1
MXE	KIAA1429	-	chr8:94530962-94531085	chr8:94534838-94535007	0.112	0.02615574639	-0.012	1
MXE	CIB1	-	chr15:90232218-90232327	chr15:90233668-90233703	0.112	0.01287962329	0.017	0.3584000157
MXE	TRAPPC3	-	chr1:36139719-36139819	chr1:36140068-36140166	0.111	0.01217057872	-0.004	1
MXE	KIAA1551	+	chr12:31960776-31960871	chr12:31980877-31985957	0.111	0.01001908489	-0.023	1
MXE	STAG3L5P-I	+	chr7:100338772-100338889	chr7:100345864-100345968	0.111	0.00296195312	0.012	1
MXE	RMDN3	-	chr15:40738500-40738576	chr15:40745119-40745259	0.11	0.007869364411	-0.001	1
MXE	ANAPC16	+	chr10:72223887-72224056	chr10:72230365-72230440	0.11	0.003363953235	-0.007	1
MXE	LUC7L3	+	chr17:50737286-50737479	chr17:50738136-50738199	0.109	5.64E-06	-0.061	1
MXE	AGPAT4	-	chr6:161154148-161154310	chr6:161165576-161165713	0.108	0.0006171062672	-0.019	0.005268690489
MXE	BCL2L13	+	chr22:17688985-17689142	chr22:17696140-17696210	0.108	0.001814591819	0.009	1
MXE	CTSC	-	chr11:88312387-88312554	chr11:88334936-88335082	0.108	0.0247604586	0.021	0.771352608
MXE	CHKA	-	chr11:68074716-68074830	chr11:68081403-68081457	0.108	0.02218226445	-0.068	0.0005110467477
MXE	RPS6KB2	+	chr11:67431367-67431515	chr11:67432355-67432492	0.108	0.0007294478413	0.058	0
MXE	CUEDC2	-	chr10:102423797-102423859	chr10:102423995-102424178	0.107	0.001455705933	0.001	1
MXE	ZGRF1	-	chr4:112587279-112587929	chr4:112603523-112603697	0.107	0.01126235261	0.026	1
MXE	SOD1	+	chr21:31663789-31663886	chr21:31666448-31666518	0.107	0.000440024471	0.002	1
MXE	TPT1-AS1	+	chr13:45383090-45383235	chr13:45389734-45389820	0.107	0.018188216	-0.026	1
MXE	RPS6KB2	+	chr11:67431367-67431515	chr11:67432352-67432492	0.107	0.0005752714278	0.057	0
MXE	LUC7L3	+	chr17:50737286-50737479	chr17:50738140-50738199	0.106	4.40E-06	-0.059	1
MXE	FBXL4	-	chr6:98917373-98917719	chr6:98934761-98934879	0.106	0.001380734764	-0.006	1
MXE	B4GALT4	-	chr3:119229846-119230244	chr3:119236852-119237070	0.106	0.004953620754	-0.008	1
MXE	HNRNPH1	-	chr5:179621241-179621397	chr5:179623036-179623164	0.106	0.0002986804302	-0.016	0.5513892917
MXE	SLC38A9	-	chr5:55656714-55656774	chr5:55664692-55664863	0.106	0.02053550709	0.005	0.9214862729
MXE	TLK1	-	chr2:171028338-171028405	chr2:171046173-171046362	0.105	0.01313028342	0.015	1
MXE	DPH5	-	chr1:100995109-100995149	chr1:101001466-101001587	0.104	0.001814591819	0.003	1
MXE	PPIL3	-	chr2:200882341-200882435	chr2:200885261-200885343	0.104	3.05E-06	-0.016	0.001421949682
MXE	CBWD1	-	chr9:146101-146158	chr9:151304-151427	0.103	0.02204786988	-0.005	0.8649321474
MXE	GSAP	-	chr7:77329332-77329391	chr7:77330238-77330367	0.103	0.0002698227188	0.022	1
MXE	RABEPK	+	chr9:125220538-125220700	chr9:125227909-125228059	0.102	0.01742082734	0.01	1
MXE	TFRC	-	chr3:196075158-196075360	chr3:196077063-196077122	0.101	0.001018090401	-0.004	1
MXE	ARNT	-	chr1:150842423-150842468	chr1:150846262-150846307	0.101	0.01126235261	-0.043	0.3623723167
MXE	C1orf43	-	chr1:154213892-154213946	chr1:154214456-154214574	0.101	0.001794772579	0.012	0.6985415487
MXE	HMGN2	+	chr1:26473482-26473527	chr1:26473702-26473817	0.101	5.39E-06	0.001	0.01181436369
MXE	MRPL47	-	chr3:179598674-179598771	chr3:179601729-179601790	-0.101	3.57E-10	-0.005	1
MXE	RPAIN	+	chr17:5425970-5426299	chr17:5428070-5428211	-0.102	0.005830025374	-0.016	1
MXE	FIP1L1	+	chr4:53399729-53399839	chr4:53452919-53453133	-0.103	0.0001310168527	0.007	1
MXE	CCDC14	-	chr3:123955842-123955965	chr3:123956354-123956427	-0.103	0.01001908489	0.044	0.6057130972
MXE	LARP1	+	chr5:154793594-154793723	chr5:154793799-154794000	-0.104	0.0001568303725	-0.033	0.2190766925
MXE	MVK	+	chr12:109579801-109579946	chr12:109581394-109581550	-0.105	0.03891190073	0.006	1
MXE	FAM92A1	+	chr8:93709770-93709875	chr8:93718674-93718788	-0.105	0.006065508786	-0.016	0.9582556869
MXE	FAM221A	+	chr7:23692182-23692253	chr7:23698191-23698299	-0.105	0.0006007034903	-0.042	2.32E-06
MXE	NSRP1	+	chr17:30118079-30118173	chr17:30163066-30163162	-0.106	0.0001113518188	0.011	0.8547258525
MXE	ACTR10	+	chr14:58215204-58215284	chr14:58219693-58219729	-0.106	0.002706536064	-0.007	1
MXE	IGF2BP3	-	chr7:23319137-23319254	chr7:23342063-23342189	-0.106	0.00699945538	-0.029	1
MXE	MOK	-	chr14:102263545-102263616	chr14:102278605-102278694	-0.108	0.01710508937	0.025	1
MXE	ARL14EP	+	chr11:30330885-30331374	chr11:30332865-30332993	-0.109	0.03819192811	-0.033	0.04351879584
MXE	NDUFAF6	+	chr8:95036370-95036494	chr8:95040705-95040927	-0.109	0.04341779919	0.047	0.01612042262
MXE	CCDC66	+	chr3:56563683-56564125	chr3:56566593-56566740	-0.109	0.0372602753	0.021	1
MXE	STRADA	-	chr17:63714005-63714108	chr17:63726637-63726695	-0.109	1.34E-05	-0.047	2.28E-05
MXE	CHEK2	-	chr22:28695709-28695873	chr22:28696900-28696987	-0.111	0.008687271641	0.015	1
MXE	USP53	+	chr4:119235289-119235411	chr4:119239217-119239903	-0.112	0.02547112692	0.051	0.112898373

MXE	SHMT1	-	chr17:18333165-18333288	chr17:18335558-18335675	-0.112	0.0274075858	-0.001	1
MXE	SH3GLB2	-	chr9:129012235-129012298	chr9:129014410-129014503	-0.113	5.31E-05	-0.002	0.9066802714
MXE	SLCO4A1-A	-	chr20:62664923-62665497	chr20:62665859-62666010	-0.113	7.86E-06	-0.042	5.69E-06
MXE	HNRNPAB	+	chr5:178209329-178209432	chr5:178210131-178210272	-0.114	1.43E-06	-0.017	0.08268356326
MXE	TACC3	+	chr4:1723727-1723870	chr4:1727707-1728787	-0.115	0.001047468692	-0.018	0.5328406679
MXE	PSMA4	+	chr15:78542176-78542219	chr15:78542482-78542645	-0.115	0.0002604984889	-0.008	1
MXE	GPD2	+	chr2:156476097-156476207	chr2:156496043-156496215	-0.115	0.01261593438	-0.029	1
MXE	RP4-777O2:	-	chr7:30500535-30500681	chr7:30524366-30524486	-0.115	0	-0.065	7.85E-08
MXE	STX2	-	chr12:130806981-130807090	chr12:130808630-130808704	-0.116	0.03649123668	-0.027	1
MXE	C17orf80	+	chr17:73233219-73233325	chr17:73235475-73236995	-0.116	8.46E-06	0.014	1
MXE	TPT1-AS1	+	chr13:45379898-45380001	chr13:45383090-45383235	-0.116	0.03030437867	0	1
MXE	PTTG1IP	-	chr21:44856192-44856364	chr21:44861162-44861271	-0.116	0.0003146550775	-0.004	1
MXE	TBC1D5	-	chr3:17508473-17508605	chr3:17623848-17623913	-0.117	0.03804740112	-0.021	1
MXE	SENP7	-	chr3:101398860-101399055	chr3:101417592-101417790	-0.117	0.009790410867	0.011	1
MXE	HNRNPAB	+	chr5:178209329-178209447	chr5:178210131-178210272	-0.117	1.08E-07	-0.017	0.03188244244
MXE	SEC24C	+	chr10:73751107-73751243	chr10:73759621-73759794	-0.118	0.03147663073	0.005	1
MXE	THAP9-AS1	-	chr4:82895691-82895774	chr4:82897988-82898062	-0.118	0.01323461605	-0.054	0.8140679126
MXE	SUMF2	+	chr7:56074173-56074218	chr7:56074585-56074736	-0.12	4.97E-05	-0.028	0.07792216463
MXE	ZUFSP	-	chr6:116660735-116660846	chr6:116666633-116666912	-0.12	0.03791778075	0.037	0.5922684338
MXE	DPY19L4	+	chr8:94756035-94756159	chr8:94761699-94761834	-0.12	0.01971974686	0.03	0.8433385351
MXE	MTA1	+	chr14:105445666-105445852	chr14:105449358-105449409	-0.12	9.90E-07	-0.004	0.5868038912
MXE	DIABLO	-	chr12:122224511-122224644	chr12:122224644-122224748	-0.12	3.44E-11	-0.009	0.6105059873
MXE	FASTK	-	chr7:151076912-151077027	chr7:151077100-151077236	-0.121	0.01029295253	0.004	1
MXE	CCDC14	-	chr3:123955842-123955965	chr3:123956045-123956115	-0.121	0.02785913751	0.003	1
MXE	SIPA1L3	+	chr19:38119305-38119882	chr19:38130497-38130772	-0.122	0.008360219703	0.007	1
MXE	DONSON	-	chr21:33583487-33583666	chr21:33584589-33584768	-0.122	0.0002959732077	0.033	0.1163465324
MXE	PGAP2	+	chr11:3811249-3811424	chr11:3823612-3823740	-0.123	0.004195565611	0.039	1.89E-05
MXE	ERCC1	-	chr19:45421177-45421393	chr19:45423269-45423381	-0.124	0.0008495928534	-0.026	0.005620356807
MXE	NDE1	+	chr16:15651468-15651584	chr16:15651930-15652050	-0.124	0.03549421484	0.004	1
MXE	AGTPBP1	-	chr9:85681267-85681335	chr9:85692688-85692813	-0.124	0.0003822624243	0.032	1
MXE	GBA	-	chr1:155240629-155240717	chr1:155241085-155241180	-0.124	0.002845465673	-0.008	0.9785565686
MXE	CALU	+	chr7:128754261-128754455	chr7:128754528-128754722	-0.125	0.0002985715716	-0.036	3.80E-13
MXE	AURKA	-	chr20:56388311-56388506	chr20:56388686-56388784	-0.126	0.008758570512	0.01	1
MXE	CCDC176	+	chr14:74046059-74046130	chr14:74047929-74048074	-0.127	0.01977590355	-0.093	1
MXE	SDCCAG3	-	chr9:136405117-136405202	chr9:136405904-136405978	-0.127	1.74E-05	0.01	0.8346712181
MXE	FASTKD1	-	chr2:169538012-169538141	chr2:169540050-169540179	-0.128	0.003836887588	0.003	1
MXE	NCK2	+	chr2:105855047-105855289	chr2:105881327-105882049	-0.129	0.0008495928534	0.004	1
MXE	PMF1-BGLA	+	chr1:156233627-156233728	chr1:156236287-156236483	-0.129	0.001301445097	0.004	1
MXE	NDUFAF6	+	chr8:95036301-95036494	chr8:95040705-95040927	-0.13	0.03547198855	0.036	0.04183150021
MXE	TFDP1	+	chr13:113610995-113611062	chr13:113631622-113631744	-0.13	0.0007625425829	-0.02	0.03652199859
MXE	CARD8	-	chr19:48249522-48249633	chr19:48249750-48249847	-0.132	0.01479498131	0.009	1
MXE	TRIQK	-	chr8:92921502-92921660	chr8:92954405-92954564	-0.132	1.64E-09	-0.015	0.4796691057
MXE	FAM228B	+	chr2:24135118-24135187	chr2:24164197-24164335	-0.133	0.02193360829	-0.007	1
MXE	HDAC8	-	chrX:72488932-72489041	chrX:72495155-72495268	-0.134	0.0247604586	-0.012	1
MXE	SAP30BP	+	chr17:75703310-75703371	chr17:75703807-75703859	-0.134	0.01563724167	0	1
MXE	MFF	+	chr2:227342744-227342819	chr2:227347225-227347384	-0.134	2.19E-05	-0.014	0.3929611123
MXE	MOK	-	chr14:102265822-102265912	chr14:102278605-102278694	-0.135	0.04446147507	0.034	1
MXE	SH3D19	-	chr4:151147921-151148186	chr4:151149499-151149561	-0.136	0.01943201436	0.025	1
MXE	ELP2	+	chr18:36138794-36138872	chr18:36141136-36141201	-0.137	8.74E-05	0.034	1.76E-11
MXE	IFI27L1	+	chr14:94100738-94100771	chr14:94101813-94102059	-0.137	0.02602007324	-0.006	1
MXE	PVT1	+	chr8:127939507-127939676	chr8:127983903-127984204	-0.137	0.0001007016067	-0.01	1
MXE	OFD1	+	chrX:13763744-13763855	chrX:13767126-13767284	-0.138	0.0004426164455	0.004	1
MXE	FAM3A	-	chrX:154511847-154511871	chrX:154512822-154512936	-0.138	0.0001121731905	-0.007	1
MXE	LPHN3	+	chr4:61676825-61676935	chr4:61732753-61733554	-0.138	0.008473719203	0.016	1
MXE	MARK2	+	chr11:63903060-63903158	chr11:63903985-63904147	-0.139	0.04895904011	0.055	5.31E-07
MXE	ZMYND8	-	chr20:47236325-47236516	chr20:47249286-47249439	-0.139	0.005434224012	0.007	1
MXE	RPAIN	+	chr17:5426235-5426299	chr17:5428070-5428211	-0.139	2.66E-06	-0.003	1
MXE	SCAI	-	chr9:124974174-124974276	chr9:124975246-124975389	-0.139	0.01228392512	0.036	1
MXE	HPS5	-	chr11:18311913-18312024	chr11:18317750-18317907	-0.14	0.03038613536	-0.047	0.7408635736
MXE	MSRA	+	chr8:10207832-10207901	chr8:10245103-10245223	-0.14	5.58E-06	-0.053	0
MXE	RGL1	+	chr1:183806374-183806485	chr1:183847565-183847774	-0.141	0.02946769912	0.021	0.9019539908
MXE	RWDD3	+	chr1:95244210-95244698	chr1:95246541-95246657	-0.142	0.006065508786	-0.027	0.6016057985
MXE	SEC24B	+	chr4:109473003-109473186	chr4:109481676-109481781	-0.145	2.50E-06	0.042	0.01875358126

MXE	FOXM1	-	chr12:2865354-2865399	chr12:2866392-2866521	-0.146	7.70E-05	-0.128	0
MXE	TRPC1	+	chr3:142736378-142736533	chr3:142743484-142743586	-0.146	0.006802253715	0.038	1
MXE	PAQR3	-	chr4:78920533-78920681	chr4:78923856-78923947	-0.147	0.03575113456	0.018	1
MXE	PXN	-	chr12:120215559-120215661	chr12:120216272-120217116	-0.148	0.002279950226	0.015	1
MXE	LRP8	-	chr1:53271027-53271153	chr1:53271226-53271346	-0.148	0.006052763706	-0.019	1
MXE	ANKRD11	-	chr16:89305205-89305344	chr16:89316932-89317078	-0.148	0.0003367841868	0.024	0.6690858706
MXE	CARF	+	chr2:202924356-202924415	chr2:202941859-202941980	-0.149	0.004239967383	0.05	0.9952485255
MXE	CENPU	-	chr4:184697646-184697803	chr4:184700819-184700881	-0.15	1.65E-07	0.033	0.7284504163
MXE	MSRA	+	chr8:10207832-10207901	chr8:10245103-10245223	-0.15	2.77E-06	-0.047	0
MXE	GALNT7	+	chr4:173297794-173297977	chr4:173298114-173298297	-0.152	0.008807758448	-0.055	0.2018823108
MXE	ZC3H18	+	chr16:88608933-88609051	chr16:88611267-88611536	-0.152	0.00231520176	-0.027	0.69030266
MXE	GTPBP10	+	chr7:90355085-90355230	chr7:90372154-90372228	-0.153	0.01403485768	-0.001	1
MXE	ENO2	+	chr12:6917037-6917107	chr12:6917580-6917714	-0.153	6.05E-07	-0.002	1
MXE	SH3D19	-	chr4:151147921-151148186	chr4:151149499-151149561	-0.154	0.0002641670792	0.021	1
MXE	OSBPL1A	-	chr18:24341546-24341658	chr18:24366891-24366966	-0.154	0.004423317853	0.03	1
MXE	FYN	-	chr6:111696276-111696456	chr6:111700103-111700268	-0.155	1.01E-06	-0.018	0.05560903508
MXE	CAPRIN2	-	chr12:30719078-30719225	chr12:30720810-30720915	-0.157	0.009621429537	-0.007	1
MXE	FAM135A	+	chr6:70426438-70426532	chr6:70426569-70426709	-0.159	0.03932366509	0.032	1
MXE	GARNL3	+	chr9:127264951-127265021	chr9:127291167-127291242	-0.161	0.04806034241	-0.014	1
MXE	DLG1	-	chr3:197075836-197075870	chr3:197076585-197076685	-0.165	0.003356174234	0.051	0.2483918727
MXE	RWDD3	+	chr1:95244210-95244698	chr1:95246541-95246657	-0.166	0.003664600071	-0.035	0.2305103564
MXE	TTC28-AS1	+	chr22:27935150-27935230	chr22:27993123-27993535	-0.166	0.007663048107	0.003	1
MXE	ATP9B	+	chr18:79154503-79154555	chr18:79176812-79176907	-0.167	0.001097782761	-0.066	1
MXE	QKI	+	chr6:163455278-163455421	chr6:163534981-163535125	-0.168	3.05E-05	0.004	1
MXE	USP15	+	chr12:62325871-62325933	chr12:62349220-62349307	-0.168	0.002700201888	-0.03	1
MXE	MEF2A	+	chr15:99671322-99671454	chr15:99671519-99671657	-0.169	0.002084043817	0.005	1
MXE	MEF2A	+	chr15:99671322-99671454	chr15:99671519-99671657	-0.174	0.0005770143277	-0.004	1
MXE	FOXK2	+	chr17:82582740-82582934	chr17:82584012-82584188	-0.175	3.95E-05	-0.011	1
MXE	CAMK2G	-	chr10:73824039-73824084	chr10:73825278-73825347	-0.176	0.009092240333	-0.018	1
MXE	ZNF562	-	chr19:9656546-9656653	chr19:9660719-9660874	-0.177	0.01002915357	0.007	1
MXE	DOPEY1	+	chr6:83147235-83147291	chr6:83148758-83148863	-0.179	0.01456348202	-0.015	1
MXE	SRBD1	-	chr2:45476992-45477075	chr2:45488239-45488331	-0.18	0.02536826635	-0.011	1
MXE	ANKMY1	-	chr2:240507559-240507691	chr2:240511860-240512001	-0.18	0.009147376957	0.002	1
MXE	RASSF4	+	chr10:44982520-44982663	chr10:44984021-44984113	-0.181	0.006290006577	0.01	0.7969899087
MXE	B3GNTL1	-	chr17:82965650-82965746	chr17:83005114-83005210	-0.181	0.000398856445	-0.004	1
MXE	FAM118B	+	chr11:126234994-126235087	chr11:126240791-126241044	-0.182	7.70E-05	0.017	1
MXE	SFI1	+	chr22:31556941-31557059	chr22:31561289-31561392	-0.182	0.01122483829	-0.009	1
MXE	MRPS18C	+	chr4:83458345-83458429	chr4:83459739-83459797	-0.184	1.82E-11	0.002	1
MXE	GTPBP10	+	chr7:90355085-90355230	chr7:90372154-90372228	-0.185	0.001463632539	0.008	1
MXE	GALNT7	+	chr4:173297794-173297977	chr4:173298114-173298297	-0.185	0.0004884841784	-0.061	0.2428842518
MXE	ZGRF1	-	chr4:112603523-112603697	chr4:112606007-112606091	-0.186	0.005366809164	-0.045	1
MXE	RCC1	+	chr1:28508829-28508901	chr1:28516724-28516867	-0.187	0.0002881438691	0.002	1
MXE	MLLT10	+	chr10:21586293-21586348	chr10:21595330-21595440	-0.189	1.95E-06	-0.048	0.5245300014
MXE	SH3D19	-	chr4:151149499-151149561	chr4:151159239-151159352	-0.189	0.0144354842	0.041	1
MXE	KIF13A	-	chr6:17873376-17873437	chr6:17898167-17898180	-0.19	0.02565955525	-0.012	1
MXE	PQLC3	+	chr2:11164203-11164261	chr2:11171924-11172045	-0.19	0.0108490355	-0.022	1
MXE	TMEM63B	+	chr6:44150563-44150629	chr6:44151845-44152008	-0.191	0.002658984414	-0.023	1
MXE	RABGEF1	+	chr7:66739995-66740792	chr7:66771882-66772078	-0.192	7.65E-05	-0.04	0.3079977626
MXE	ZGRF1	-	chr4:112603523-112603697	chr4:112606007-112606091	-0.193	0.003456374552	-0.049	1
MXE	GAS5	-	chr1:173865470-173865547	chr1:173866527-173866567	-0.194	8.27E-05	-0.004	1
MXE	TXLNA	+	chr1:32184524-32184616	chr1:32187953-32188124	-0.196	0.001031064798	0.003	1
MXE	MOK	-	chr14:102263545-102263616	chr14:102265822-102265912	-0.197	0.02719533086	-0.028	1
MXE	RP11-421L2	+	chr1:101075275-101075394	chr1:101075394-101075716	-0.198	0.0003131589947	-0.042	0.4348041261
MXE	SYTL2	-	chr11:85714412-85714507	chr11:85717482-85717530	-0.199	0.008014771864	0.038	1
MXE	DCUN1D4	+	chr4:51891759-51891851	chr4:51911069-51911174	-0.2	1.55E-06	0.015	1
MXE	MFF	+	chr2:227342744-227342819	chr2:227347225-227347384	-0.2	2.28E-05	-0.015	0.8673176874
MXE	TFPI	-	chr2:187513615-187513646	chr2:187529363-187529485	-0.201	0.002783607971	-0.008	1
MXE	HSF4	+	chr16:67167474-67167599	chr16:67167823-67167947	-0.201	0.004540987033	0.008	1
MXE	ABHD14A	+	chr3:51978258-51978374	chr3:51980392-51980628	-0.203	0.0318128299	-0.014	1
MXE	UGGT2	-	chr13:95856157-95856340	chr13:95859590-95859675	-0.204	0.001400276335	0.026	0.2681968531
MXE	TRIM5	-	chr11:5665980-5666081	chr11:5667688-5667711	-0.204	0.005212261631	-0.008	1
MXE	CLIP1	-	chr12:122347374-122347479	chr12:122351110-122351143	-0.208	0.0009627121469	0.038	1
MXE	ANKRD27	-	chr19:32607634-32607832	chr19:32615657-32615780	-0.209	0.004476786939	0.019	1

MXE	ACER3	+	chr11:76985642-76985724	chr11:76990538-76990574	-0.21	0.006065508786	-0.036	1
MXE	DLG1	-	chr3:197075836-197075870	chr3:197076585-197076682	-0.212	0.003045392827	0.06	0.2112286043
MXE	ACAA1	-	chr3:38131925-38132005	chr3:38133951-38134009	-0.213	0.006804735893	0.037	0.007783566267
MXE	SDHC	+	chr1:161323613-161323670	chr1:161328395-161328497	-0.213	0.001810025378	0.006	1
MXE	SUZ12P1	+	chr17:30734896-30734943	chr17:30743300-30743369	-0.214	6.63E-06	0.016	1
MXE	TTC25	+	chr17:41935470-41935543	chr17:41935598-41935749	-0.215	0.03441493153	-0.073	1
MXE	MPPE1	-	chr18:11897525-11897620	chr18:11905737-11905953	-0.218	0.0011580361	-0.004	1
MXE	CTNND1	+	chr11:57789036-57789155	chr11:57791384-57791673	-0.219	0.004696136325	-0.002	1
MXE	SFI1	+	chr22:31556941-31557059	chr22:31561289-31561392	-0.221	0.04505429643	-0.042	1
MXE	CD27-AS1	-	chr12:6448639-6448737	chr12:6450340-6450980	-0.223	0.0003131589947	0.011	1
MXE	ARNTL2	+	chr12:27370128-27370230	chr12:27376345-27376387	-0.224	0.002977500621	-0.017	1
MXE	GAS5	-	chr1:173864674-173864704	chr1:173865228-173865282	-0.224	0	-0.004	1
MXE	WDR27	-	chr6:169665485-169665556	chr6:169670568-169670693	-0.225	0.01847367209	0.026	1
MXE	FAM86C1	+	chr11:71791735-71791819	chr11:71793379-71793481	-0.231	0.01960597291	-0.004	1
MXE	SUN1	+	chr7:848417-848615	chr7:849922-850033	-0.231	1.08E-07	0.017	0.8312730181
MXE	KIF13A	-	chr6:17789871-17789910	chr6:17794248-17794395	-0.232	0.01033725682	-0.045	1
MXE	RP11-421L2	+	chr1:101077362-101077508	chr1:101083460-101083574	-0.235	0.04252502176	-0.1	2.15E-06
MXE	MSL1	+	chr17:40129244-40129627	chr17:40132033-40132098	-0.237	0	0.051	8.80E-07
MXE	CTNND1	+	chr11:57789036-57789155	chr11:57791493-57791673	-0.237	0.007137831095	-0.012	1
MXE	FLCN	-	chr17:17222500-17222661	chr17:17223921-17224143	-0.238	0.00723383536	-0.006	1
MXE	CTNND1	+	chr11:57789036-57789155	chr11:57791491-57791673	-0.238	0.007189633564	-0.013	1
MXE	SYTL2	-	chr11:85714412-85714507	chr11:85717482-85717726	-0.242	3.60E-07	0.005	1
MXE	PCBP1-AS1	-	chr2:70055713-70055910	chr2:70059642-70059680	-0.246	0.01388670621	0.088	0.4140041386
MXE	PCBP1-AS1	-	chr2:70055713-70055910	chr2:70059642-70059680	-0.246	0.01388670621	0.099	0.1388438884
MXE	SCHIP1	+	chr3:159866162-159866281	chr3:159886206-159886324	-0.247	0	-0.017	0.6671294199
MXE	ARHGEF40	+	chr14:21087000-21087105	chr14:21087319-21087463	-0.249	6.58E-05	-0.095	8.37E-13
MXE	RUFY3	+	chr4:70789494-70789592	chr4:70793764-70793904	-0.252	0.005649545712	-0.195	0
MXE	UGGT2	-	chr13:95859590-95859675	chr13:95860787-95860883	-0.253	0.0001298389853	0.039	0.8375223012
MXE	SPOP	-	chr17:49636758-49636809	chr17:49668026-49668078	-0.254	0.009456335949	-0.035	0.5066287568
MXE	PCNT	+	chr21:46421969-46422124	chr21:46425830-46425971	-0.26	0.01452950685	-0.016	0.9719564839
MXE	SUN1	+	chr7:849519-849603	chr7:849922-850033	-0.266	1.97E-10	0.041	0.1766295962
MXE	PCBP1-AS1	-	chr2:70055655-70055910	chr2:70059642-70059680	-0.27	0.01020578741	0.056	0.9571047309
MXE	CTNND1	+	chr11:57789036-57789155	chr11:57794009-57794081	-0.274	1.02E-13	-0.038	0.4669800982
MXE	SNX14	-	chr6:85541984-85542043	chr6:85543181-85543306	-0.275	9.64E-12	-0.031	0.02285440182
MXE	PPRC1	+	chr10:102144249-102144307	chr10:102146671-102147392	-0.277	2.09E-07	0.039	0.2473813842
MXE	DGKZ	+	chr11:46368001-46368079	chr11:46369493-46369550	-0.279	0.004410053941	0.003	1
MXE	FAM228B	+	chr2:24139369-24139450	chr2:24146929-24147086	-0.281	0.00294268215	-0.013	1
MXE	RNF146	+	chr6:127280515-127280604	chr6:127285207-127285348	-0.284	0.0001091448679	-0.077	0.0004303877121
MXE	GUSBP11	-	chr22:23695172-23695517	chr22:23700725-23700845	-0.284	0.0001265450782	-0.018	1
MXE	TNC	-	chr9:115046409-115046682	chr9:115048259-115048532	-0.285	0.04224761506	-0.045	3.59E-07
MXE	SLCO5A1	-	chr8:69738039-69738204	chr8:69755423-69755641	-0.288	0.000307498296	-0.05	1
MXE	SLC29A1	+	chr6:44227262-44227342	chr6:44229389-44229471	-0.303	0	0.044	0.001575429063
MXE	DHRS4L2	+	chr14:23995031-23995133	chr14:24001383-24001517	-0.307	0.02072749562	0.052	0.07792216463
MXE	TPT1-AS1	+	chr13:45379925-45380001	chr13:45383090-45383235	-0.308	9.27E-05	-0.012	1
MXE	EMILIN2	+	chr18:2884963-2885139	chr18:2890560-2892486	-0.309	9.76E-05	0.017	1
MXE	C1QTNF6	-	chr22:37191671-37191737	chr22:37195380-37195458	-0.314	0.01470907625	-0.001	1
MXE	ELOVL5	-	chr6:53290094-53290189	chr6:53290400-53290535	-0.319	0.0004536946538	-0.06	1
MXE	CCNJL	-	chr5:160269462-160269544	chr5:160276268-160276412	-0.321	0.004337264385	-0.018	1
MXE	FAM45A	+	chr10:119123468-119123569	chr10:119129514-119129622	-0.327	8.86E-05	-0.01	1
MXE	SEC31A	-	chr4:82861630-82861708	chr4:82862533-82862572	-0.328	1.11E-09	-0.01	1
MXE	U2AF1	-	chr21:6492130-6492197	chr21:6493043-6493110	-0.334	0.04701157616	-0.078	1
MXE	RFWD2	-	chr1:176149005-176149074	chr1:176184632-176184692	-0.34	0.01289228184	-0.014	1
MXE	YY1AP1	-	chr1:155679927-155680008	chr1:155680381-155680456	-0.342	3.79E-05	0.004	1
MXE	FANCI	+	chr15:89245194-89245311	chr15:89245755-89245839	-0.348	1.65E-07	-0.007	1
MXE	FYN	-	chr6:111699514-111699670	chr6:111700103-111700268	-0.349	0	-0.056	0
MXE	TADA2A	+	chr17:37426949-37427009	chr17:37437737-37437922	-0.353	0.0007720291368	0.06	0.9317169586
MXE	CBWD1	-	chr9:147900-148040	chr9:151304-151427	-0.365	0.01047188361	-0.039	1
MXE	ETFA	-	chr15:76284364-76284460	chr15:76284804-76284927	-0.367	2.24E-05	-0.008	1
MXE	CTNND1	+	chr11:57789036-57789155	chr11:57794009-57794081	-0.371	0	-0.045	0.2954947816
MXE	PCBP1-AS1	-	chr2:70055713-70055910	chr2:70059642-70059680	-0.379	0.0002725051906	0.095	1
MXE	RNF146	+	chr6:127280515-127280604	chr6:127282286-127282395	-0.382	0.01094845149	-0.174	1.52E-11
MXE	HDAC11	+	chr3:13498512-13498555	chr3:13500712-13500789	-0.384	0.0001997038677	0.023	1
MXE	PGAP2	+	chr11:3811249-3811424	chr11:3822908-3822993	-0.385	4.38E-11	0.01	0.01935677844

MXE	MTMR2	-	chr11:95882373-95882496	chr11:95887611-95887684	-0.388	0.01313028342	-0.067	1
MXE	NQO1	-	chr16:69713027-69713129	chr16:69714963-69715077	-0.394	0	0.016	1
MXE	FAM86C1	+	chr11:71791738-71791819	chr11:71793379-71793481	-0.395	0.00664473448	0.04	1
MXE	SUN1	+	chr7:849519-849603	chr7:849922-850033	-0.397	5.58E-12	-0.011	1
MXE	TANGO2	+	chr22:20055942-20056013	chr22:20063337-20063442	-0.397	0.00308776571	0.022	0.547983957
MXE	LINC00894	+	chrX:149941789-149941847	chrX:149943650-149943762	-0.4	0.002052545047	0.063	1
MXE	SEPT2	+	chr2:241317498-241317599	chr2:241324408-241324662	-0.404	1.58E-05	0.052	1
MXE	ZHX3	-	chr20:41213733-41213899	chr20:41267462-41267612	-0.408	0.01738238435	-0.087	1
MXE	RP11-507M:	+	chr2:24139369-24139450	chr2:24146929-24147086	-0.408	0.004156507974	0.017	1
MXE	LINC00630	+	chrX:102816991-102817082	chrX:102825992-102826169	-0.413	0.002188454797	-0.126	1
MXE	SS18	-	chr18:26080304-26080423	chr18:26082362-26082528	-0.419	0.0001659601603	0.244	1
MXE	MX1	+	chr21:41449136-41449295	chr21:41451166-41451243	-0.419	0.04197901362	0.008	1
MXE	PCBP1-AS1	-	chr2:70055713-70055910	chr2:70059642-70059680	-0.431	0.03469929876	0.11	0.1021250984
MXE	DNM1	+	chr9:128224250-128224389	chr9:128226034-128226173	-0.438	2.11E-05	0.155	3.08E-07
MXE	TMBIM6	+	chr12:49741956-49742280	chr12:49743416-49743565	-0.442	0.001459892005	0.072	0.1378272727
MXE	LINC00894	+	chrX:149942183-149942329	chrX:149943650-149943762	-0.445	0.0005601986855	0.13	1
MXE	DAK	+	chr11:61335839-61335937	chr11:61336203-61336390	-0.446	0.0003061337011	0.036	1
MXE	ISYNA1	-	chr19:18436972-18437105	chr19:18437598-18437760	-0.472	3.96E-05	-0.001	1
MXE	TRMT2B	-	chrX:101051250-101051366	chrX:101051643-101051733	-0.474	0.004271075024	0.044	1
MXE	TNFSF13	+	chr17:7559623-7559702	chr17:7559850-7559893	-0.476	3.35E-06	0.011	1
MXE	ANKMY1	-	chr2:240509347-240509455	chr2:240511860-240512001	-0.477	0.005009216857	0.046	1
MXE	RP11-175P1	-	chr12:100177042-100177120	chr12:100180552-100180716	-0.478	0.0002414008361	-0.073	1
MXE	KIAA1468	+	chr18:62279773-62279856	chr18:62280359-62280442	-0.495	1.61E-13	-0.055	0.01431104557
MXE	SYTL2	-	chr11:85714412-85714507	chr11:85717482-85717530	-0.512	0	-0.016	1
MXE	DLEU1	+	chr13:50433412-50433550	chr13:50527865-50528064	-0.517	0.0001288175153	-0.168	1
MXE	ZCWPW2	+	chr3:28492126-28492173	chr3:28514063-28514122	-0.527	0.0004781924829	0.044	1
MXE	CNTLN	+	chr9:17273732-17273866	chr9:17298189-17298352	-0.54	6.62E-10	0.022	1
MXE	HSD11B1L	+	chr19:5684818-5684905	chr19:5684964-5685119	-0.541	0.01372157933	-0.02	0.790753828
MXE	SS18	-	chr18:26080304-26080423	chr18:26084051-26084175	-0.557	9.01E-05	0.142	1
MXE	HSD11B1L	+	chr19:5684818-5684905	chr19:5684964-5685119	-0.557	0.007513422672	-0.03	0.3387484737
MXE	NAT1	+	chr8:18212390-18212774	chr8:18216876-18216994	-0.582	0.0002815207596	-0.008	1
MXE	HDHD1	-	chrX:7077219-7077449	chrX:7105619-7105838	-0.583	0	0	1
MXE	SYTL2	-	chr11:85711112-85711232	chr11:85714412-85714507	-0.625	0	-0.022	1
MXE	NQO2	+	chr6:3003668-3003789	chr6:3004494-3004651	-0.629	0.0003073158693	-0.037	1
MXE	NOTCH2NL	-	chr1:146136352-146136455	chr1:146142649-146142752	-0.66	4.61E-07	0.096	1
MXE	TCF7L2	+	chr10:113159919-113159992	chr10:113160618-113160691	-0.684	0.0007076008699	-0.061	1
MXE	FAM122C	+	chrX:134847882-134847971	chrX:134849166-134849228	-0.69	0.0008449722938	0.015	1
MXE	TXNRD1	+	chr12:104290992-104291104	chr12:104309758-104309950	-0.697	4.20E-05	0.135	1
MXE	TVP23C-CD	-	chr17:15503097-15503232	chr17:15540432-15540561	-0.827	0	-0.06	0.6267284598
RI	HLA-A	+	chr6:29944499-29945091	NA	0.911	0	0.041	0.5203650265
RI	NID2	-	chr14:52005736-52006660	NA	0.884	1.29E-06	-0.022	4.39E-08
RI	ZNF582-AS1	+	chr19:56398276-56399168	NA	0.809	0	-0.003	8.31E-05
RI	TPT1	-	chr13:45340711-45341158	NA	0.802	4.16E-13	-0.045	0.009230892338
RI	WDR90	+	chr16:655057-655468	NA	0.76	0.0002730825593	-0.015	1
RI	TCF4	-	chr18:55228846-55232671	NA	0.753	7.37E-05	-0.018	0.0002409891459
RI	PLXNB1	-	chr3:48410454-48411036	NA	0.641	0.01297814223	-0.015	0
RI	QKI	+	chr6:163563419-163570145	NA	0.602	3.34E-14	-0.026	1.78E-06
RI	AP1G2	-	chr14:23563583-23563856	NA	0.577	6.09E-10	-0.009	0.5361133281
RI	NPR2	+	chr9:35808508-35808853	NA	0.544	0.001284150908	-0.042	0.006974750714
RI	ZNF517	+	chr8:144802869-144804238	NA	0.542	8.14E-05	-0.005	1
RI	ABCB6	-	chr2:219210380-219210823	NA	0.538	0.001910688849	-0.03	0
RI	AP1G2	-	chr14:23563379-23563638	NA	0.518	9.00E-08	-0.052	1
RI	PPIL2	+	chr22:21694936-21695496	NA	0.507	0.000377647128	-0.031	1
RI	FNBP1L	+	chr1:93550946-93554659	NA	0.47	0	0.113	1.85E-13
RI	GAS5	-	chr1:173865228-173865547	NA	0.436	0	-0.012	0.002626283484
RI	PI4KAP2	-	chr22:21475210-21476764	NA	0.423	0.0002582586347	0.142	1
RI	AP1G2	-	chr14:23563715-23564221	NA	0.412	3.17E-07	-0.088	0.002836188205
RI	QKI	+	chr6:163563419-163570145	NA	0.403	4.78E-07	0.008	1
RI	ARL6IP4	+	chr12:122981128-122981879	NA	0.401	1.61E-08	-0.042	0.007097848044
RI	U2AF1L4	-	chr19:35744322-35744738	NA	0.397	9.78E-05	-0.004	0.9339013671
RI	OBSL1	-	chr2:219550728-219551798	NA	0.388	0.03714903893	-0.067	3.75E-06
RI	BBS1	+	chr11:66531655-66532219	NA	0.388	0.0009446880095	-0.029	2.07E-11
RI	ATHL1	+	chr11:290677-292095	NA	0.381	3.71E-07	-0.051	0.2222305185

RI	HLA-F	+	chr6:29726010-29726526	NA	0.379	0.0001249613788	-0.023	1.84E-09
RI	TTC28-AS1	+	chr22:27997410-27998838	NA	0.378	9.64E-07	0.013	0.9865056133
RI	POMT1	+	chr9:131509902-131510415	NA	0.364	5.98E-08	-0.062	1.81E-07
RI	KIAA0195	+	chr17:75492928-75493593	NA	0.355	4.12E-08	-0.024	4.79E-05
RI	SERHL2	+	chr22:42555663-42556083	NA	0.355	8.44E-05	-0.049	1
RI	ENO2	+	chr12:6916670-6917107	NA	0.353	0	-0.003	0
RI	KIAA1217	+	chr10:24544980-24547843	NA	0.335	0.02220739931	-0.008	1
RI	FBXL6	-	chr8:144356299-144356713	NA	0.333	0.00642391228	-0.021	0.1849841908
RI	CELSR3	-	chr3:48641850-48642467	NA	0.33	0.04364710769	0.001	1
RI	ATP5D	+	chr19:1244314-1244825	NA	0.328	7.42E-07	-0.035	0.01190386311
RI	MFSD10	-	chr4:2931374-2931936	NA	0.328	5.32E-07	0.073	0.007813240274
RI	PNISR	-	chr6:99403828-99404702	NA	0.328	6.32E-09	0.003	1
RI	ARHGAP33	+	chr19:35787167-35788822	NA	0.324	0.0001837228863	-0.015	1
RI	SYVN1	-	chr11:65130001-65130379	NA	0.323	0.01538941007	-0.02	0.1608661574
RI	ELMOD3	+	chr2:85355075-85357252	NA	0.322	0.002439817932	-0.01	1
RI	GOLGA2	-	chr9:128262562-128263092	NA	0.313	2.48E-07	-0.013	2.35E-08
RI	BTN3A2	+	chr6:26367989-26368267	NA	0.308	0.001170885484	-0.009	0.7901193723
RI	MAGED4	+	chrX:52186396-52187845	NA	0.307	0.01903661337	0.087	1
RI	AP1G2	-	chr14:23565605-23566160	NA	0.306	0.000143996723	-0.014	1
RI	SNHG17	-	chr20:38421005-38422241	NA	0.299	0.004901189048	-0.03	1
RI	MUS81	+	chr11:65864719-65865145	NA	0.292	7.45E-05	-0.032	0.004995050927
RI	ADD1	+	chr4:2908514-2909431	NA	0.284	0.01160406502	0.007	1
RI	ZSWIM8	+	chr10:73794146-73794639	NA	0.284	0.002500567157	0.01	1
RI	U2AF1L4	-	chr19:35744322-35745187	NA	0.282	0.005964038928	0.018	1
RI	TTLL3	+	chr3:9817644-9820741	NA	0.276	0.0001504685719	-0.032	0.4762847514
RI	ATAT1	+	chr6:30642767-30644679	NA	0.271	2.34E-08	0.125	0
RI	AP1G2	-	chr14:23562503-23563638	NA	0.27	0.000308896551	-0.01	1
RI	WBP1	+	chr2:74459437-74459745	NA	0.268	5.64E-05	0.04	0.3367828665
RI	ACADVL	+	chr17:7224320-7224552	NA	0.266	0.03643105134	-0.006	1
RI	NAT9	-	chr17:74771959-74772277	NA	0.266	0.0003929852813	-0.11	5.20E-08
RI	HCN3	+	chr1:155283973-155284757	NA	0.266	0.001850082181	-0.031	0.5948979623
RI	AP1G2	-	chr14:23561511-23562415	NA	0.266	0.01014875315	0.032	0.05209917345
RI	PSME1	+	chr14:24136984-24137431	NA	0.265	0.04795993741	0.059	0.3903378356
RI	C21orf58	-	chr21:46301129-46302154	NA	0.265	3.09E-06	-0.064	0.05204595944
RI	NICN1	-	chr3:49424948-49425438	NA	0.264	0.02849421127	-0.042	0.00314863413
RI	C11orf49	+	chr11:47161126-47162247	NA	0.261	0.0006560085309	-0.034	0.147638566
RI	POMZP3	-	chr7:76618182-76618615	NA	0.259	0.01055453037	-0.061	0.15525877
RI	ATP5G1	+	chr17:48893408-48895334	NA	0.256	0.0005832107347	0.039	1
RI	MIB2	+	chr1:1628018-1628399	NA	0.252	0.002590851266	-0.012	0.003114513445
RI	PLCD4	+	chr2:218633604-218634221	NA	0.245	8.18E-06	-0.019	1
RI	WDR6	+	chr3:49011634-49014293	NA	0.245	9.44E-07	-0.005	1
RI	TMEM175	+	chr4:948115-948615	NA	0.244	0.001050415336	-0.03	1
RI	MUS81	+	chr11:65865810-65866439	NA	0.242	2.06E-09	-0.033	0.0001614240489
RI	TMEM80	+	chr11:698868-700235	NA	0.241	9.73E-06	-0.016	0.03065871459
RI	LIN37	+	chr19:35754245-35754519	NA	0.239	0.00873127551	-0.003	1
RI	DHRS1	-	chr14:24292183-24292784	NA	0.237	0.002376863758	-0.052	0.0008685476598
RI	TRIM22	+	chr11:5697247-5698545	NA	0.236	0.03643105134	-0.003	0.4342450058
RI	BAX	+	chr19:48960809-48961798	NA	0.233	0	-0.028	2.03E-05
RI	C16orf93	-	chr16:30759341-30759724	NA	0.231	0.04587055951	-0.103	0.02202247675
RI	ARL6IP4	+	chr12:122981128-122981879	NA	0.229	0.00421474628	-0.031	0.4367982126
RI	WBP1	+	chr2:74459437-74459745	NA	0.226	0.0007736868659	0.037	0.1526846262
RI	TUBGCP6	-	chr22:50219287-50219791	NA	0.225	0.007846724902	0	0.0007867004752
RI	BDH2	-	chr4:103085348-103086540	NA	0.223	8.25E-05	-0.106	0.0006568547472
RI	TJAP1	+	chr6:43503622-43506553	NA	0.22	7.65E-05	-0.008	0.003719528183
RI	NRBP2	-	chr8:143839508-143840030	NA	0.219	1.41E-12	-0.008	1.90E-06
RI	RBM5	+	chr3:50100531-50103166	NA	0.214	0.0005495594221	-0.012	1
RI	MAU2	+	chr19:19344848-19345369	NA	0.213	0.000191588729	-0.015	1.42E-05
RI	DDX11	+	chr12:31103576-31104791	NA	0.21	0.01676346818	-0.051	0.004456518969
RI	ZNF7	+	chr8:144829042-144829604	NA	0.209	0.01867064045	-0.057	0.06075179784
RI	CDK11A	-	chr1:1703475-1703940	NA	0.208	0.0007969360077	-0.041	1.45E-07
RI	CCDC159	+	chr19:11354579-11354943	NA	0.208	0.01046368681	-0.002	1
RI	RBM8A	-	chr1:145926808-145927077	NA	0.208	0.007696141702	-0.03	0.2455024824
RI	GAS5	-	chr1:173864483-173864704	NA	0.206	4.03E-10	-0.017	4.93E-08

RI	CUL9	+	chr6:43196068-43196862	NA	0.205	0.009992530522	-0.036	5.12E-05
RI	PRMT2	+	chr21:46660832-46661936	NA	0.205	0.006535676432	-0.007	4.34E-05
RI	C21orf59	-	chr21:32601666-32603292	NA	0.204	0.007746832051	0.072	0.01577654258
RI	CTC1	-	chr17:8229890-8230468	NA	0.204	0.001464590268	-0.057	1.81E-06
RI	CC2D1B	-	chr1:52360423-52361132	NA	0.202	1.15E-07	-0.023	8.67E-14
RI	MUS81	+	chr11:65864719-65865323	NA	0.2	4.10E-08	-0.007	0.2614932913
RI	CDK5RAP3	+	chr17:47974399-47975337	NA	0.199	6.86E-10	-0.025	0.3129948839
RI	PCSK7	-	chr11:117223207-117224216	NA	0.199	0.02272113874	0.002	0.1947017111
RI	CENPT	-	chr16:67829764-67830548	NA	0.195	8.95E-05	-0.06	0.06005234483
RI	PCYT2	-	chr17:81906099-81906546	NA	0.195	0	-0.015	3.27E-12
RI	HSF4	+	chr16:67165521-67165846	NA	0.187	0.0009475934077	-0.014	1
RI	PAN2	-	chr12:56319087-56319487	NA	0.186	0.008317953933	-0.045	0.03870315637
RI	FADS3	-	chr11:61876358-61876963	NA	0.185	0.009886221094	-0.085	1.03E-07
RI	ING4	-	chr12:6652270-6652770	NA	0.184	0.001649350636	0	1
RI	DPM2	-	chr9:127936552-127937523	NA	0.183	9.37E-07	-0.054	0
RI	EWSR1	+	chr22:29298732-29299851	NA	0.181	0.009886221094	-0.027	0.1021827972
RI	IL17RC	+	chr3:9923880-9924291	NA	0.18	0.007244724238	-0.054	2.35E-08
RI	TNFRSF25	-	chr1:6462862-6463127	NA	0.18	0.004890547527	-0.072	0.3375541643
RI	RPL12	-	chr9:127449609-127451405	NA	0.177	0.0001196031007	-0.003	1
RI	C16orf93	-	chr16:30759011-30759247	NA	0.176	0.03403927258	-0.063	1
RI	TIAL1	-	chr10:119576610-119577203	NA	0.175	0.0004445519329	-0.01	0.0007022138268
RI	CAPRIN2	-	chr12:30714958-30716676	NA	0.173	0.04460971474	-0.078	0.0002060574012
RI	EFHC1	+	chr6:52452687-52454287	NA	0.172	0.006772555752	-0.016	0.5857241083
RI	MAGED2	+	chrX:54815247-54816007	NA	0.171	3.60E-08	-0.037	0
RI	GAS5	-	chr1:173864256-173864506	NA	0.171	1.09E-08	-0.015	1.05E-13
RI	STX10	-	chr19:13144061-13144671	NA	0.17	0	-0.021	2.70E-10
RI	TMEM234	-	chr1:32216196-32217351	NA	0.169	0.01538941007	-0.035	3.41E-08
RI	BRF1	-	chr14:105211121-105212164	NA	0.167	0.0236309712	-0.014	0
RI	ARL16	-	chr17:81682033-81682675	NA	0.167	8.83E-05	0.014	1
RI	CCNK	+	chr14:99500765-99502376	NA	0.166	0.0009695331436	-0.049	0.4311923144
RI	FAM50A	+	chrX:154449680-154449931	NA	0.165	1.14E-05	-0.012	1.14E-12
RI	DXO	-	chr6:31970605-31971147	NA	0.165	0.02210643183	-0.035	0.06841266514
RI	ZFYVE19	+	chr15:40813942-40814569	NA	0.164	0.03791551547	0.002	1
RI	USP21	+	chr1:161164533-161164942	NA	0.162	0.01262240708	-0.073	0.0002098949126
RI	PDZD7	-	chr10:101021797-101022385	NA	0.162	0.002698189981	-0.049	1
RI	RP11-252A2	-	chr16:74352039-74352378	NA	0.16	0.009084561186	0.045	1
RI	TPRN	-	chr9:137191616-137192365	NA	0.157	2.31E-05	-0.002	0.3809034212
RI	ACAA1	-	chr3:38126161-38126700	NA	0.156	8.83E-05	-0.028	6.65E-13
RI	TMEM218	-	chr11:125102131-125102809	NA	0.156	0.02020371516	-0.062	7.28E-05
RI	CCDC150	+	chr2:196729192-196729861	NA	0.155	0.00999475111	0.082	1
RI	DSN1	-	chr20:36770872-36771473	NA	0.155	0.01437449705	-0.032	1
RI	EIF4A2	+	chr3:186784563-186786051	NA	0.153	4.57E-06	0.015	0.1015092422
RI	RNF215	-	chr22:30379481-30379780	NA	0.152	0.003341924059	-0.015	0.1179909992
RI	HSF4	+	chr16:67169278-67169945	NA	0.151	0.01288428282	-0.049	1
RI	MUTYH	-	chr1:45332762-45333324	NA	0.151	0.01473339108	0.093	0
RI	WDR24	-	chr16:685868-686186	NA	0.15	0.02211554839	-0.036	6.14E-06
RI	MEN1	-	chr11:64805634-64806368	NA	0.15	0.0002725309832	-0.014	0.003925373802
RI	GAS5	-	chr1:173864256-173865282	NA	0.15	0	-0.012	0
RI	NR2C2	+	chr3:15037999-15039227	NA	0.148	0.01160759958	-0.005	0.1156182394
RI	DDIT3	-	chr12:57517268-57517753	NA	0.147	0.00342269305	-0.036	0.08978042819
RI	ZSWIM8	+	chr10:73794146-73794639	NA	0.147	0.007527884364	-0.003	0.4762847514
RI	JMJD8	-	chr16:683321-683598	NA	0.146	0.004143798929	-0.1	0
RI	CIRBP	+	chr19:1271980-1273172	NA	0.145	7.01E-05	-0.001	1
RI	MOV10	+	chr1:112698286-112699810	NA	0.143	0.001314429458	-0.028	6.57E-05
RI	ING4	-	chr12:6652661-6653050	NA	0.142	0.004201701249	-0.009	0.002957063449
RI	ERCC3	-	chr2:127289336-127289824	NA	0.142	8.44E-05	-0.015	6.33E-06
RI	TPM3	-	chr1:154170648-154172978	NA	0.14	1.13E-05	0.093	0.04270665674
RI	DVL3	+	chr3:184164795-184165206	NA	0.14	1.86E-06	-0.001	0.7797013353
RI	ATP13A1	-	chr19:19656659-19656916	NA	0.138	0.006156541073	-0.036	0.00349910865
RI	SAT2	-	chr17:7627142-7627414	NA	0.136	0.0002695271887	-0.037	0
RI	EFNA1	+	chr1:155133502-155133780	NA	0.136	0.01309155262	-0.033	8.54E-09
RI	FBXO9	+	chr6:53092733-53093561	NA	0.135	0.01685875146	-0.016	0.005485746792
RI	EFEMP2	-	chr11:65870120-65870658	NA	0.135	3.56E-06	-0.005	0.0003296405564

RI	CDK11A	-	chr1:1704222-1704655	NA	0.134	0.004818730187	-0.041	0.01994143287
RI	RHOT2	+	chr16:672253-672825	NA	0.134	1.09E-08	-0.019	0.02641073325
RI	MOV10	+	chr1:112698286-112698789	NA	0.132	0.02573388838	-0.05	1.32E-05
RI	CRELD1	+	chr3:9943076-9943515	NA	0.132	9.79E-07	-0.034	1.96E-13
RI	PRPF31	+	chr19:54129056-54129370	NA	0.131	0.0001267919825	-0.006	3.97E-05
RI	LDLRAP1	+	chr1:25563660-25565207	NA	0.131	0.01068294572	-0.013	1
RI	HTRA2	+	chr2:74531338-74531702	NA	0.13	0.001981135288	-0.122	0.001332338633
RI	CDK10	+	chr16:89693273-89693467	NA	0.13	0.03804967276	-0.076	0.0139818314
RI	RNF213	+	chr17:80388611-80389367	NA	0.128	0.0002137736042	-0.016	2.21E-08
RI	SAT2	-	chr17:7627362-7627802	NA	0.127	0.007818289167	-0.024	0
RI	COMMD7	-	chr20:32702702-32704071	NA	0.127	2.13E-07	-0.013	5.23E-14
RI	RELT	+	chr11:73394235-73394734	NA	0.126	0.001605230086	0.001	1
RI	CENPT	-	chr16:67829764-67831358	NA	0.126	0.03113327069	-0.02	0.007089067466
RI	ASPSCR1	+	chr17:82016799-82017113	NA	0.125	0.002774257415	-0.029	2.69E-06
RI	SAT2	-	chr17:7627362-7627805	NA	0.124	0.04004330286	-0.034	0.0265299928
RI	ZNF76	+	chr6:35286127-35286399	NA	0.123	0.001893949787	-0.006	0.001043272011
RI	BEST1	+	chr11:61958145-61959578	NA	0.12	0.0001484207919	-0.037	1
RI	DDIT3	-	chr12:57517268-57517753	NA	0.12	0.04122650279	-0.004	0.03653090247
RI	PRPF4B	+	chr6:4056320-4057187	NA	0.118	0.0001702677729	-0.024	1.40E-14
RI	EMD	+	chrX:154379941-154380367	NA	0.117	2.22E-13	-0.025	0
RI	TTC31	+	chr2:74490024-74490473	NA	0.117	0.02129946588	-0.033	0.3483858373
RI	CDK11A	-	chr1:1704038-1704344	NA	0.116	0.00555582277	-0.008	0.6643929134
RI	XPNPEP1	-	chr10:109875527-109877867	NA	0.116	0.001120005346	-0.006	0.03012675355
RI	UBXN1	-	chr11:62676497-62677005	NA	0.114	5.34E-05	-0.008	2.69E-06
RI	C1orf35	-	chr1:228102079-228102382	NA	0.111	0.007696141702	-0.057	4.08E-09
RI	C5orf42	-	chr5:37139339-37142480	NA	0.11	3.58E-13	-0.009	0.004241921815
RI	NOP56	+	chr20:2657080-2658393	NA	0.109	0.001723300719	-0.009	0.000914828258
RI	ULK3	-	chr15:74842074-74842420	NA	0.109	0.0146694743	-0.038	4.93E-08
RI	SYVN1	-	chr11:65132247-65132780	NA	0.109	3.46E-07	-0.016	6.51E-12
RI	FAM219B	-	chr15:74899991-74902786	NA	0.109	0.02030767124	0.027	0.03057139956
RI	CDK5RAP3	+	chr17:47973518-47974031	NA	0.107	6.43E-06	-0.009	1.28E-11
RI	PIAS3	-	chr1:145848521-145850269	NA	0.106	0.0095896582	0.001	1
RI	MED22	-	chr9:133340904-133344333	NA	0.106	2.59E-06	0.214	0
RI	BBS1	+	chr11:66515692-66515933	NA	0.106	0.02792705927	-0.019	5.27E-09
RI	MOSPD3	+	chr7:100612729-100613269	NA	0.105	0.005192877926	-0.003	0.0038948208
RI	SLC12A4	-	chr16:67945086-67945553	NA	0.105	0.03906439299	-0.014	2.48E-15
RI	EIF2B5	+	chr3:184143051-184143565	NA	0.105	0.01970885861	-0.041	7.77E-05
RI	RGL2	-	chr6:33293794-33294066	NA	0.105	0.000485827391	-0.045	0
RI	OGT	+	chrX:71544566-71548023	NA	0.104	1.60E-05	-0.005	1
RI	DGKA	+	chr12:55940081-55940722	NA	0.103	0.001744039268	-0.059	0
RI	SPAG5	-	chr17:28579750-28580120	NA	-0.101	2.69E-09	-0.024	2.62E-06
RI	BTN3A2	+	chr6:26373275-26373413	NA	-0.101	0.001925892701	-0.005	1
RI	RBM25	+	chr14:73110830-73111802	NA	-0.103	0.01817881467	-0.01	0.08530603406
RI	C3orf17	-	chr3:113012733-113013403	NA	-0.103	0.0006844104956	-0.007	0.04836579501
RI	TIMM21	+	chr18:74155144-74155403	NA	-0.104	0.0006993832697	0.001	0.1088174894
RI	RBM41	-	chrX:107088435-107088839	NA	-0.104	0.0003658835225	0	1
RI	ARL6IP4	+	chr12:122981128-122981879	NA	-0.104	0.001961330751	-0.033	6.01E-08
RI	RNH1	-	chr11:499014-499999	NA	-0.105	0.0006770723973	-0.01	0
RI	DDX46	+	chr5:134816429-134817714	NA	-0.105	0.0003968948543	0.007	1
RI	EXOSC8	+	chr13:37002933-37004561	NA	-0.106	7.73E-06	-0.002	1
RI	RPAIN	+	chr17:5425970-5428211	NA	-0.106	0.002482770348	-0.027	0
RI	POP5	-	chr12:120579513-120579923	NA	-0.106	0.002482369946	-0.025	4.93E-12
RI	NIT2	+	chr3:100345584-100346255	NA	-0.108	0.0002330445727	-0.005	0.7014034958
RI	TAMM41	-	chr3:11807832-11809682	NA	-0.108	6.68E-13	-0.042	0
RI	MCM7	-	chr7:100099022-100099753	NA	-0.108	0	-0.002	0.1176138885
RI	ARAP1	-	chr11:72712437-72713243	NA	-0.108	0.008307631995	-0.042	0
RI	GALE	-	chr1:23798116-23798730	NA	-0.109	0.0001267152438	-0.022	6.08E-07
RI	C11orf57	+	chr11:112074315-112074916	NA	-0.109	0.04557377175	-0.009	0.5437975795
RI	TMEM208	+	chr16:67228796-67229278	NA	-0.109	0.0002668533231	0.008	0.4610517445
RI	PRPF4B	+	chr6:4060412-4061147	NA	-0.11	4.91E-05	0.023	0.0002323265305
RI	ACAD11	-	chr3:132578795-132579558	NA	-0.11	0.00157883001	-0.055	0.003829939189
RI	GALE	-	chr1:23796508-23796782	NA	-0.111	0.01001845849	-0.044	3.88E-08
RI	YBX3	-	chr12:10709907-10713333	NA	-0.112	1.32E-07	-0.003	0.024518083

RI	NEK8	+	chr17:28737305-28737736	NA	-0.113	0.0180385365	-0.145	1
RI	ELP5	+	chr17:7258587-7259940	NA	-0.113	0.0002144640796	0.007	0.001423666323
RI	SPATA7	+	chr14:88427629-88429463	NA	-0.114	0.01608781478	-0.007	0.2948560283
RI	PILRB	+	chr7:100353372-100354012	NA	-0.115	0.04986683364	-0.014	0.1987536026
RI	CAPRIN2	-	chr12:30719078-30720915	NA	-0.116	0.02160256185	-0.022	0.001718528215
RI	COMMD3	+	chr10:22318271-22318713	NA	-0.116	0.0002480556874	-0.014	1.37E-10
RI	PTGES2	-	chr9:128122361-128123134	NA	-0.117	0.02567180123	0.016	0.669622968
RI	DERL2	-	chr17:5481100-5481389	NA	-0.118	0.01314365759	0.017	1
RI	MTX1	+	chr1:155210547-155212219	NA	-0.118	0.0001735682727	-0.036	1.61E-14
RI	RPP21	+	chr6:30345297-30346557	NA	-0.118	1.90E-06	-0.002	0.06402147554
RI	DDX39A	-	chr19:14409741-14410334	NA	-0.119	0.002219711602	-0.004	0.9200018801
RI	NKTR	+	chr3:42619019-42619708	NA	-0.12	0.02667604946	0.003	1
RI	WDR45	-	chrX:49076644-49077911	NA	-0.12	0.01992583731	0.012	0.5739021329
RI	LRRC41	-	chr1:46279181-46279614	NA	-0.122	0.0001182452421	-0.007	0.004490061065
RI	MIF4GD	-	chr17:75267745-75268192	NA	-0.123	0.001166852681	-0.031	4.23E-10
RI	SLC25A3	+	chr12:98593974-98595848	NA	-0.124	3.27E-10	-0.004	6.96E-07
RI	EXOSC9	+	chr4:121813865-121816447	NA	-0.124	9.95E-08	-0.007	0.01466038292
RI	TJAP1	+	chr6:43500743-43501687	NA	-0.124	0.005092959178	-0.018	0.01770379103
RI	NAGK	+	chr2:71072640-71073594	NA	-0.128	0.000164408632	-0.028	0
RI	STAT2	-	chr12:56354777-56355351	NA	-0.129	0.0174877641	-0.125	8.90E-06
RI	DRG2	+	chr17:18099016-18100435	NA	-0.13	6.99E-07	0	0.2576807227
RI	IKBKB	+	chr8:42319269-42320844	NA	-0.132	0.01928890763	0.078	2.48E-15
RI	PIDD1	-	chr11:800761-801120	NA	-0.135	0.007244724238	-0.001	1
RI	ETV4	-	chr17:43545273-43545668	NA	-0.136	2.47E-06	-0.029	1
RI	TCF4	-	chr18:55228846-55232671	NA	-0.136	0.000666479782	-0.013	1.46E-08
RI	E2F4	+	chr16:67192760-67193170	NA	-0.136	9.34E-06	-0.042	2.32E-05
RI	HDLBP	-	chr2:241268953-241269295	NA	-0.137	0.04657411543	0.12	1
RI	MFN1	+	chr3:179378340-179378814	NA	-0.137	4.80E-10	-0.005	0.1793576111
RI	POLR1C	+	chr6:43519705-43520185	NA	-0.137	0.006280059241	-0.027	5.93E-05
RI	SRSF7	-	chr2:38748578-38749705	NA	-0.138	2.58E-05	0.007	0.9650266323
RI	QTRT1	+	chr19:10701949-10702254	NA	-0.138	0.001162103423	-0.025	0
RI	NELFE	-	chr6:31954554-31955096	NA	-0.139	0.01266779508	0.026	0.2484620488
RI	KRIT1	-	chr7:92235402-92236542	NA	-0.14	9.18E-06	-0.005	0.6493889757
RI	C2orf49	+	chr2:105342847-105343223	NA	-0.14	0.001365874141	0.01	1
RI	TPM1	+	chr15:63061197-63062277	NA	-0.14	1.57E-11	-0.024	0
RI	FUS	+	chr16:31184938-31188357	NA	-0.141	6.33E-13	0.008	1
RI	PNKP	-	chr19:49864178-49864403	NA	-0.142	0.0095896582	-0.039	7.98E-05
RI	CIRBP	+	chr19:1271980-1274440	NA	-0.142	5.48E-11	0.001	1
RI	ZNF232	-	chr17:5108925-5109868	NA	-0.142	0.02997087908	-0.117	6.68E-07
RI	ILF3	+	chr19:10681218-10682192	NA	-0.143	1.78E-07	-0.016	0.09343721961
RI	FAM200B	+	chr4:15686235-15687148	NA	-0.143	0.02854546606	0.018	1
RI	ANAPC5	-	chr12:121327095-121328497	NA	-0.144	0	0.006	0.05237580454
RI	ANAPC5	-	chr12:121345838-121347005	NA	-0.144	0	0.013	1.05E-08
RI	C18orf8	+	chr18:23530228-23530612	NA	-0.144	0.004901189048	0	1
RI	NELFE	-	chr6:31953728-31954134	NA	-0.144	1.11E-09	-0.013	0.0002534816591
RI	BTN3A1	+	chr6:26409893-26410032	NA	-0.15	5.62E-08	-0.038	5.81E-06
RI	SCYL1	+	chr11:65538269-65538704	NA	-0.15	0.005191038741	-0.045	0.07108288609
RI	CLK3	+	chr15:74622119-74622560	NA	-0.15	1.92E-06	-0.007	0.2069400463
RI	BAZ2A	-	chr12:56615013-56615607	NA	-0.151	1.89E-10	-0.003	1
RI	LMNA	+	chr1:156137653-156138757	NA	-0.152	0.001867343234	-0.045	0.0001998731416
RI	WDR46	-	chr6:33280427-33280987	NA	-0.152	6.22E-06	0.005	1
RI	ATG16L1	+	chr2:233289853-233290353	NA	-0.153	0.03527976138	-0.02	0.08041337484
RI	TTC8	+	chr14:88841036-88841514	NA	-0.153	0.002168953809	0.005	1
RI	B4GALNT1	-	chr12:57631199-57631364	NA	-0.155	0.000336563563	0.024	5.47E-12
RI	PCNXL2	-	chr1:232984135-232986540	NA	-0.155	0.006928555429	-0.084	1.63E-09
RI	HAUS7	-	chrX:153454393-153455766	NA	-0.157	1.59E-09	-0.002	0.7311315753
RI	HDAC6	+	chrX:48814961-48815490	NA	-0.158	0.04960482811	-0.048	0.01542143747
RI	RBM33	+	chr7:155738059-155740026	NA	-0.159	0.003244746418	-0.008	1
RI	CDCA7	+	chr2:173363817-173364989	NA	-0.16	0.01251638125	-0.014	0.01265986631
RI	HNRNPM	+	chr19:8465323-8465515	NA	-0.161	7.93E-08	-0.006	1
RI	NAGK	+	chr2:71075554-71076701	NA	-0.162	0.006239792161	-0.007	2.75E-06
RI	EOGT	-	chr3:68988505-68989017	NA	-0.162	0.04266975874	-0.058	0.3511442743
RI	C5orf45	-	chr5:179837275-179840959	NA	-0.163	0.005154888944	0.015	1

RI	NKIRAS2	+	chr17:42022398-42022640	NA	-0.163	5.64E-05	-0.007	0.2847463068
RI	RPS27	+	chr1:153990762-153991034	NA	-0.166	2.22E-10	-0.016	0.007689539258
RI	ORC2	-	chr2:200913294-200913992	NA	-0.167	0.01229678921	-0.011	0.7308035463
RI	CD274	+	chr9:5462833-5463121	NA	-0.167	0.0004473236184	-0.003	1
RI	RSRP1	-	chr1:25243549-25245301	NA	-0.168	0	0.027	0.9699067261
RI	ORMDL1	-	chr2:189782421-189783123	NA	-0.168	0.002200528562	-0.017	0.8767054258
RI	UBXN4	+	chr2:135753538-135754277	NA	-0.168	8.43E-07	-0.023	2.57E-09
RI	TSEN2	+	chr3:12529761-12531659	NA	-0.169	0	0.026	0.008442343124
RI	CCAR2	+	chr8:22619149-22620468	NA	-0.169	0.001791555223	-0.013	4.85E-09
RI	CAD	+	chr2:27233311-27233808	NA	-0.17	0.005154888944	-0.071	2.10E-10
RI	THAP7	-	chr22:21000646-21001411	NA	-0.17	5.29E-05	-0.094	0
RI	AKNA	-	chr9:114343707-114346009	NA	-0.174	0.005410896005	-0.006	0.08557171304
RI	NSD1	+	chr5:177135086-177136030	NA	-0.175	0.0001641005066	0.022	1
RI	DECR2	+	chr16:406684-406952	NA	-0.175	0.03089148023	-0.029	1
RI	CHKB	-	chr22:50580189-50580416	NA	-0.177	0.0007579141903	-0.068	4.74E-05
RI	BRAT1	-	chr7:2539785-2541052	NA	-0.181	0.02743375478	-0.037	2.72E-08
RI	SNX15	+	chr11:65035519-65038829	NA	-0.182	2.03E-05	0	0.4979325872
RI	TANC1	+	chr2:159219237-159219867	NA	-0.182	0.01003146764	-0.033	0.07769260489
RI	NOC3L	-	chr10:94357173-94358215	NA	-0.182	0	-0.007	1
RI	PACS2	+	chr14:105383358-105384463	NA	-0.184	1.17E-07	-0.001	1
RI	TMEM138	+	chr11:61364249-61364518	NA	-0.186	0.0003481439514	-0.006	0.5105583676
RI	WDR55	+	chr5:140668233-140668502	NA	-0.188	3.74E-07	-0.079	1.39E-07
RI	BBS2	-	chr16:56502316-56502808	NA	-0.191	0	-0.014	1.84E-06
RI	ZSWIM7	-	chr17:15977578-15977913	NA	-0.191	0.02434367906	0.019	0.9015479658
RI	FAM3A	-	chrX:154515759-154516242	NA	-0.192	0.00491760265	-0.026	0.2187702092
RI	COG4	-	chr16:70508405-70509388	NA	-0.192	1.14E-14	0.026	0.2730210341
RI	TEN1-CDK3	+	chr17:76002510-76003398	NA	-0.193	0.01292108692	0.026	1
RI	RPP30	+	chr10:90894774-90895483	NA	-0.194	0.0002573288381	-0.009	7.68E-05
RI	PEX1	-	chr7:92501506-92502079	NA	-0.198	9.31E-05	-0.034	0.03800830747
RI	BPHL	+	chr6:3138040-3140509	NA	-0.198	0.01313973159	0.074	0.1329040777
RI	MLST8	+	chr16:2206357-2206659	NA	-0.198	5.59E-08	0.011	0.01410214697
RI	RBM8A	-	chr1:145926481-145927077	NA	-0.199	3.44E-05	-0.002	1
RI	KANSL2	-	chr12:48681381-48682225	NA	-0.201	4.32E-05	0.014	8.74E-06
RI	ARHGAP32	-	chr11:128972452-128975002	NA	-0.203	4.53E-08	0.01	0.2827202429
RI	CC2D1B	-	chr1:52357780-52358461	NA	-0.203	0.01047160473	-0.034	5.67E-05
RI	MOK	-	chr14:102232534-102233789	NA	-0.205	0.0006345139613	-0.062	0.002836188205
RI	N6AMT1	-	chr21:28872190-28876491	NA	-0.205	0.01873364361	0.005	1
RI	DUS1L	-	chr17:82061208-82061721	NA	-0.208	0.002439817932	-0.018	2.92E-14
RI	ADARB1	+	chr21:45222017-45226560	NA	-0.208	0.002219711602	-0.04	0.4312666656
RI	PEX1	-	chr7:92492952-92494396	NA	-0.21	2.53E-05	-0.066	0.0003051041912
RI	RPGR	-	chrX:38287093-38288041	NA	-0.212	1.39E-05	-0.003	1
RI	DET1	-	chr15:88530622-88531715	NA	-0.216	0.007987937871	0.012	0.2614932913
RI	WRAP73	-	chr1:3630766-3631657	NA	-0.219	0.0189131366	-0.079	6.18E-11
RI	FBF1	-	chr17:75914121-75914932	NA	-0.219	0.01867064045	-0.04	0.0001056579432
RI	NECAB3	-	chr20:33659884-33660395	NA	-0.22	0.01873719104	0.061	1
RI	RUSC1	+	chr1:155325863-155327132	NA	-0.221	0.005394362141	-0.03	0.03198465532
RI	DENND4C	+	chr9:19336269-19336832	NA	-0.222	9.18E-06	-0.055	1
RI	SREBF1	-	chr17:17817547-17817916	NA	-0.223	0.01127882976	-0.032	1
RI	HMBS	+	chr11:119092124-119092523	NA	-0.227	0.03751352006	-0.071	1.69E-05
RI	MPP2	-	chr17:43882902-43883355	NA	-0.23	8.14E-05	0.035	1
RI	PINK1	+	chr1:20648504-20649231	NA	-0.23	2.52E-05	-0.005	0.0003067980742
RI	RPS9	+	chr19:54206275-54206724	NA	-0.234	0.001162103423	-0.04	0.04059299619
RI	DALRD3	-	chr3:49015972-49016340	NA	-0.234	0.03426009204	-0.077	2.81E-08
RI	HARS2	+	chr5:140693490-140693665	NA	-0.235	0.001275168466	-0.004	1
RI	IDH3A	+	chr15:78156907-78157631	NA	-0.239	0.0001490791649	-0.053	1
RI	C19orf66	+	chr19:10091249-10091630	NA	-0.243	0.002219711602	-0.027	0.0314046826
RI	NSUN5	-	chr7:73307582-73308553	NA	-0.246	0.002672952032	-0.018	1
RI	March6	+	chr5:10400783-10402139	NA	-0.253	4.85E-09	0.004	1
RI	BDH2	-	chr4:103085348-103086540	NA	-0.253	0	0.013	0.4026289619
RI	ISCU	+	chr12:108564059-108564392	NA	-0.255	3.67E-05	-0.081	0.03298130095
RI	GNRHR2	+	chr1:145919343-145919900	NA	-0.257	0.002073595908	-0.002	1
RI	CDK20	-	chr9:87969193-87970897	NA	-0.259	0.0002155787581	-0.002	1
RI	ABTB1	+	chr3:127676535-127677286	NA	-0.262	0.002857952109	-0.021	9.27E-14

RI	AFTPH	+	chr2:64579485-64581273	NA	-0.262	0.009886221094	-0.015	0.8648963937
RI	MTF2	+	chr1:93110229-93110428	NA	-0.264	1.02E-07	-0.007	1
RI	POLD1	+	chr19:50415723-50416528	NA	-0.266	0.005472713134	-0.069	2.48E-15
RI	ENDOV	+	chr17:80429772-80430343	NA	-0.266	2.91E-05	-0.03	1
RI	NPHP1	-	chr2:110163047-110164730	NA	-0.268	0.01528618861	-0.134	0.05663681961
RI	DCXR	-	chr17:82036858-82037547	NA	-0.268	0.04849882675	0.022	0.0003818895414
RI	TUBGCP4	+	chr15:43401715-43403799	NA	-0.278	7.93E-08	-0.018	0.8599833433
RI	PLSCR1	-	chr3:146521543-146522053	NA	-0.279	1.65E-09	-0.005	1
RI	ATP5SL	-	chr19:41431318-41433434	NA	-0.28	0.0007734376691	-0.015	0.01736526612
RI	BRAT1	-	chr7:2543203-2543962	NA	-0.283	9.46E-05	-0.034	3.24E-11
RI	DALRD3	-	chr3:49015818-49016086	NA	-0.283	0.0001551860719	-0.102	3.08E-09
RI	ABHD11	-	chr7:73737220-73737735	NA	-0.285	0.0236309712	-0.141	4.76E-09
RI	RPL3	-	chr22:39313190-39314208	NA	-0.286	0.0007965341189	0.033	2.47E-10
RI	CEP192	+	chr18:13057584-13059312	NA	-0.287	0	0.009	0.09509219872
RI	B4GALT3	-	chr1:161176433-161177106	NA	-0.298	0.0007328348363	-0.055	1
RI	ARRDC1	+	chr9:137612895-137613510	NA	-0.299	1.29E-06	-0.098	1.42E-06
RI	C9orf89	+	chr9:93110567-93112289	NA	-0.301	3.15E-07	-0.003	1
RI	RRNAD1	+	chr1:156731990-156732473	NA	-0.303	0.000167746949	-0.098	7.76E-10
RI	LIN37	+	chr19:35753086-35754157	NA	-0.312	0.0007935197621	-0.014	0.002626283484
RI	PIDD1	-	chr11:802681-803647	NA	-0.317	0.001045349009	-0.016	1
RI	NDUFAF7	+	chr2:37241577-37242693	NA	-0.318	2.72E-05	-0.044	0.1580231666
RI	RPS9	+	chr19:54206552-54207647	NA	-0.319	6.28E-05	-0.042	0.04129831848
RI	SUGP2	-	chr19:18994365-18995280	NA	-0.322	1.97E-13	0.02	0.2272518209
RI	MAEA	+	chr4:1336860-1338617	NA	-0.323	0.01580040133	0.021	0.2669472176
RI	RPL17	-	chr18:49491531-49491833	NA	-0.324	0	0.016	0.0002667903333
RI	CLK4	-	chr5:178616881-178618778	NA	-0.325	0.00639004427	0.011	1
RI	SLC9A5	+	chr16:67255392-67255930	NA	-0.326	0.0072366921	0.07	1
RI	PLEKHH2	+	chr2:43710488-43712383	NA	-0.327	0.0005898240881	-0.013	1
RI	MIIP	+	chr1:12029222-12030124	NA	-0.328	0.008465546508	-0.024	1.32E-08
RI	HMGN1	-	chr21:39345145-39348339	NA	-0.329	1.73E-12	-0.028	0
RI	NAPRT	-	chr8:143576077-143576572	NA	-0.331	0.03947238461	-0.012	5.15E-05
RI	U2AF1L4	-	chr19:35744322-35745187	NA	-0.332	0.0095896582	0.107	0.001914407062
RI	CLK4	-	chr5:178617343-178618778	NA	-0.336	9.82E-08	0.046	0.002583436711
RI	MAN2C1	-	chr15:75358461-75358808	NA	-0.337	1.41E-12	-0.015	0.1499871015
RI	WDR45	-	chrX:49076644-49077911	NA	-0.337	0.0006389898037	0.034	0.7736195789
RI	KCNAB3	-	chr17:7923967-7924265	NA	-0.341	0.002842735607	0.072	1
RI	RBM6	+	chr3:50061461-50062108	NA	-0.347	1.98E-07	0.014	1
RI	MAMDC4	+	chr9:136856463-136856826	NA	-0.348	0.0271123135	-0.044	1
RI	EWSR1	+	chr22:29298732-29299280	NA	-0.354	9.93E-05	-0.018	1
RI	PRPF40B	+	chr12:49631859-49632880	NA	-0.355	4.01E-05	0.112	0.0003839233133
RI	COMMD4	+	chr15:75338984-75339878	NA	-0.366	6.58E-07	0.025	0.003388821628
RI	NAPEPLD	-	chr7:103099775-103103554	NA	-0.376	0.002761239845	0.026	1.53E-05
RI	ZNF10	+	chr12:133130610-133131014	NA	-0.378	0.02210096143	0.074	1
RI	CLK1	-	chr2:200859679-200861466	NA	-0.38	5.85E-11	-0.049	0.3040324742
RI	AMBRA1	-	chr11:46541944-46543398	NA	-0.38	8.95E-05	-0.008	1
RI	CYB5R2	-	chr11:7668477-7672523	NA	-0.382	0.003244746418	0.051	1
RI	ALDH3B1	+	chr11:68015296-68018637	NA	-0.385	0.01778902412	-0.003	1
RI	MED23	-	chr6:131592387-131593171	NA	-0.386	0.0008916825081	-0.017	0.0111019012
RI	SH3TC2	-	chr5:149026859-149028718	NA	-0.387	8.54E-05	0.117	1
RI	MATR3	+	chr5:139314674-139316188	NA	-0.388	8.68E-14	-0.028	1
RI	NUDT17	+	chr1:145846432-145846690	NA	-0.388	0.0002993961125	-0.051	0.1983694294
RI	SIRT7	-	chr17:81914293-81915512	NA	-0.388	8.29E-05	-0.041	6.14E-06
RI	CCHCR1	-	chr6:31144686-31145073	NA	-0.388	2.14E-05	-0.019	0.5164150801
RI	LLGL1	+	chr17:18235469-18236760	NA	-0.389	0.01561183787	0.001	1
RI	DPH1	+	chr17:2041767-2043424	NA	-0.39	0.0004699056745	-0.013	0.709521158
RI	ARHGEF40	+	chr14:21087000-21087463	NA	-0.404	0.0005943848687	-0.085	4.06E-12
RI	TRABD	+	chr22:50197822-50198186	NA	-0.409	0.000651342608	-0.102	1.24E-05
RI	SAMD4A	+	chr14:54784339-54784620	NA	-0.417	0.002500459043	-0.035	1
RI	MTMR11	-	chr1:149930364-149930965	NA	-0.421	0.000164408632	-0.048	0.02194223989
RI	CENPT	-	chr16:67832011-67832545	NA	-0.428	8.43E-08	0.028	9.59E-08
RI	CENPT	-	chr16:67832011-67832545	NA	-0.43	0.0004376056157	-0.076	1
RI	MINK1	+	chr17:4889646-4890735	NA	-0.431	0.001258786667	-0.03	0.007493007502
RI	CCDC136	+	chr7:128806236-128806858	NA	-0.455	1.38E-05	-0.008	1

RI	CPSF3L	-	chr1:1312225-1312700	NA	-0.462	0.01464113754	0.069	0.04946945316
RI	BCKDK	+	chr16:31110400-31111219	NA	-0.469	3.95E-07	0.093	0.05338492089
RI	RP11-295P9	+	chr10:13654352-13655482	NA	-0.475	0.0001735682727	-0.027	1
RI	CD46	+	chr1:207767012-207770362	NA	-0.477	0	-0.142	3.34E-09
RI	CLK4	-	chr5:178616881-178618778	NA	-0.481	1.16E-13	0.042	0.09158095123
RI	RCHY1	-	chr4:75490580-75491782	NA	-0.482	0.003299762828	-0.007	1
RI	CTC-308K20	-	chr5:172957153-172958257	NA	-0.484	1.27E-07	0.013	0.6521013934
RI	RABGGTA	-	chr14:24270836-24271169	NA	-0.487	0.004579326249	-0.048	1
RI	DDX51	-	chr12:132140830-132141420	NA	-0.49	7.41E-06	0.002	0.6720834607
RI	NUDT8	-	chr11:67628918-67629921	NA	-0.504	0.000634117199	-0.013	1
RI	POLD1	+	chr19:50401998-50402373	NA	-0.509	0.003199637451	-0.022	0.005934433671
RI	PHF7	+	chr3:52421647-52422881	NA	-0.515	0.0004977363804	-0.087	1
RI	TERF2	-	chr16:69368375-69368595	NA	-0.523	1.08E-05	-0.018	1
RI	FAH	+	chr15:80152793-80153135	NA	-0.544	3.92E-08	-0.093	0.7285943532
RI	TLE2	-	chr19:3019282-3019773	NA	-0.546	1.93E-05	-0.039	1
RI	BCL3	+	chr19:44758245-44758841	NA	-0.546	0.0007097597659	-0.014	1.07E-05
RI	A1BG-AS1	+	chr19:58353713-58355183	NA	-0.563	1.09E-06	-0.04	0.3358816376
RI	GNG5	-	chr1:84506010-84506565	NA	-0.566	0.0005832107347	-0.021	1
RI	EPS8L2	+	chr11:709552-710486	NA	-0.571	1.48E-06	-0.059	1
RI	WDR83	+	chr19:12669754-12670080	NA	-0.576	5.75E-06	-0.017	0.9366484804
RI	NAGS	+	chr17:44006528-44007494	NA	-0.583	2.47E-09	0.099	0.03185542832
RI	CROCCP3	-	chr1:16476929-16478437	NA	-0.584	3.92E-05	0.04	1
RI	IMP4	+	chr2:130342893-130343194	NA	-0.588	0.004901189048	-0.062	0.1505246326
RI	CCDC57	-	chr17:82193755-82194139	NA	-0.603	0.001120005346	-0.052	1
RI	DNHD1	+	chr11:6497584-6498961	NA	-0.604	1.49E-08	0.005	1
RI	MYO15B	+	chr17:75590906-75591246	NA	-0.606	0.0004401943641	0.024	1
RI	TPM2	-	chr9:35684487-35685139	NA	-0.633	3.04E-07	-0.018	1
RI	THOP1	+	chr19:2807441-2808444	NA	-0.64	0.0001902823536	-0.023	3.65E-06
RI	MLST8	+	chr16:2206357-2206659	NA	-0.766	1.14E-14	0.026	0.4706446529
SE	HDHD1	-	chrX:7105748-7105838	NA	1	0	0.003	1
SE	PIP5K1C	-	chr19:3633436-3633520	NA	0.96	3.34E-13	0.136	7.35E-10
SE	SORBS1	-	chr10:95434621-95434717	NA	0.934	3.34E-13	0.047	0.3523006826
SE	USP3-AS1	-	chr15:63593748-63593877	NA	0.925	4.00E-12	0.25	1
SE	CLIP1	-	chr12:122351110-122351143	NA	0.924	2.89E-14	0.03	1
SE	ANKAR	+	chr2:189693073-189693177	NA	0.91	1.34E-13	0.004	1
SE	SRGAP1	+	chr12:64091892-64091926	NA	0.904	2.23E-09	0.001	1
SE	BTN3A3	+	chr6:26443366-26443442	NA	0.881	5.48E-12	0.061	1
SE	RPS24	+	chr10:78040203-78040225	NA	0.876	0	0.028	0
SE	ANKRD36	+	chr2:97209680-97209709	NA	0.869	1.65E-11	0.007	1
SE	RP4-798P15	-	chr1:177984205-177984418	NA	0.86	9.70E-11	0.02	1
SE	SYTL2	-	chr11:85717482-85717726	NA	0.857	0	-0.047	1
SE	PCBP1-AS1	-	chr2:70053730-70053815	NA	0.84	2.34E-11	0	1
SE	ZNF83	-	chr19:52617245-52617335	NA	0.838	6.02E-06	0.159	1
SE	DST	-	chr6:56468981-56468999	NA	0.827	0	0.001	0.01236024759
SE	RAPGEF2	+	chr4:159344035-159344059	NA	0.824	0	0.052	1
SE	ATP2A1	+	chr16:28894504-28894607	NA	0.824	1.09E-07	-0.085	1
SE	VPS9D1	-	chr16:89708426-89708531	NA	0.817	0.002386013963	-0.011	1
SE	CLASP2	-	chr3:33596710-33596734	NA	0.815	4.89E-12	0.076	1
SE	FUBP1	-	chr1:77947452-77947547	NA	0.814	7.18E-09	0	1
SE	PLEKHA1	+	chr10:122428275-122428420	NA	0.806	0	0.018	1
SE	SBF1	-	chr22:50457033-50457111	NA	0.784	2.68E-09	0.013	1
SE	PLEKHA1	+	chr10:122428275-122428316	NA	0.778	0	-0.002	1
SE	SORBS1	-	chr10:95414493-95414655	NA	0.777	9.52E-08	-0.107	1
SE	PPFIBP1	+	chr12:27658815-27658848	NA	0.773	0	0.101	1
SE	SLC9A5	+	chr16:67262211-67262289	NA	0.773	3.90E-10	-0.088	1
SE	TLE2	-	chr19:3017839-3017859	NA	0.769	3.07E-10	-0.033	0.1059516518
SE	KIF21A	-	chr12:39330241-39330262	NA	0.766	0	0.223	1
SE	SYTL2	-	chr11:85717482-85717530	NA	0.76	0	-0.014	0.4684459028
SE	SPTAN1	+	chr9:128609650-128609665	NA	0.759	0	0.102	3.38E-08
SE	WDFY3	-	chr4:84726860-84726911	NA	0.733	0	-0.011	1
SE	SPAG9	-	chr17:50975862-50975901	NA	0.73	0	-0.003	1
SE	PRMT2	+	chr21:46636438-46636547	NA	0.729	0	0.067	0.2319464078
SE	XRRA1	-	chr11:74940784-74940882	NA	0.728	5.96E-06	0.119	1

SE	ZNF706	-	chr8:101204649-101204693	NA		0.718	1.02E-09	0.046	1
SE	MRPL43	-	chr10:100983947-100984074	NA		0.71	6.77E-07	-0.039	1
SE	ZNF391	+	chr6:27395016-27395095	NA		0.698	7.31E-07	-0.199	1
SE	CAST	+	chr5:96726793-96726859	NA		0.698	0	0.031	1
SE	SUZ12P1	+	chr17:30734896-30734943	NA		0.696	5.79E-08	0.022	1
SE	MED24	-	chr17:40020527-40020619	NA		0.695	1.34E-05	0.043	1
SE	MICAL3	-	chr22:17803810-17803861	NA		0.695	9.30E-11	0.081	0.9403152009
SE	NEXN	+	chr1:77925187-77925229	NA		0.687	4.41E-07	-0.062	1
SE	CENPV	-	chr17:16349139-16349333	NA		0.682	5.11E-08	-0.063	0.9854954385
SE	RP11-274B2	+	chr7:128577963-128578033	NA		0.682	1.63E-09	0.03	1
SE	SORBS1	-	chr10:95414493-95414862	NA		0.681	1.03E-06	-0.112	1
SE	HLA-L	+	chr6:30262294-30262342	NA		0.674	0.0001824986839	-0.02	1
SE	MAP3K6	-	chr1:27364660-27364684	NA		0.66	0.0001833146153	0.005	1
SE	FHL2	-	chr2:105396646-105396697	NA		0.659	1.87E-07	-0.054	1
SE	MYO18A	-	chr17:29085603-29085648	NA		0.652	0	0.031	1
SE	PLD3	+	chr19:40365552-40365585	NA		0.65	3.15E-06	0.038	1
SE	CACNB4	-	chr2:151869176-151869235	NA		0.65	7.65E-06	-0.057	1
SE	GPM6A	-	chr4:175812190-175812249	NA		0.649	2.67E-10	0.168	2.56E-10
SE	C19orf60	+	chr19:18589478-18589683	NA		0.647	1.65E-09	-0.02	0.02956170982
SE	NQO1	-	chr16:69714963-69715077	NA		0.647	0	-0.019	0.3824801379
SE	ABI1	-	chr10:26771074-26771089	NA		0.644	0	0.038	0.8846931441
SE	RSRP1	-	chr1:25244453-25244498	NA		0.639	0	-0.024	1
SE	SYNJ1	-	chr21:32634860-32634884	NA		0.634	4.13E-11	0.12	3.29E-05
SE	EFCAB13	+	chr17:47347807-47347951	NA		0.634	3.12E-05	0.039	1
SE	PNISR	-	chr6:99403828-99403882	NA		0.633	0	-0.005	0.8137340216
SE	VLDLR	+	chr9:2651414-2651498	NA		0.626	0	0.005	1
SE	DNAJC7	-	chr17:42003358-42003557	NA		0.624	2.54E-07	0.03	1
SE	UBA3	-	chr3:69071534-69071617	NA		0.622	0.001965233616	-0.054	1.51E-07
SE	DGKH	+	chr13:42221263-42221394	NA		0.615	4.55E-07	0.211	1
SE	CTD-2341M	+	chr14:86128664-86128773	NA		0.612	5.70E-05	-0.013	1
SE	PAQR3	-	chr4:78935120-78935176	NA		0.61	2.01E-05	-0.076	0.0001321555703
SE	CASK	-	chrX:41557031-41557100	NA		0.608	4.65E-08	0.034	1
SE	GUSBP11	-	chr22:23700725-23700845	NA		0.608	8.54E-06	0.005	1
SE	FUBP1	-	chr1:77947452-77947552	NA		0.606	5.11E-05	-0.001	1
SE	BCAS4	+	chr20:50840536-50840930	NA		0.605	0	-0.124	0.0005746395579
SE	TNIK	-	chr3:171139469-171139556	NA		0.604	3.94E-11	0.077	1
SE	FNBP1	-	chr9:129923843-129924026	NA		0.604	0	0.071	0.0002937363512
SE	ULK3	-	chr15:74838151-74838173	NA		0.603	0.02679711654	-0.039	0.1239974608
SE	TMEM161A	-	chr19:19134783-19134916	NA		0.602	4.20E-05	-0.014	1
SE	ARL4A	+	chr7:12687634-12687728	NA		0.602	0	0.047	1
SE	ZCWPW2	+	chr3:28492126-28492173	NA		0.602	4.72E-09	-0.008	1
SE	TNC	-	chr9:115048259-115048532	NA		0.601	0.0005415202019	0.124	0.006188894532
SE	ANKRD12	+	chr18:9204475-9204544	NA		0.597	0	0.183	1
SE	SYTL2	-	chr11:85717482-85717530	NA		0.596	0.0001661427625	0.035	1
SE	RALGAPA1	-	chr14:35548507-35548538	NA		0.594	8.12E-08	0.041	1
SE	RPRD2	+	chr1:150441880-150441958	NA		0.591	0	-0.09	0.0888300337
SE	NME4	+	chr16:406684-406777	NA		0.59	0.0002851559216	0.003	1
SE	FHL2	-	chr2:105396646-105396697	NA		0.59	0.02228946003	0.016	1
SE	ZNF584	+	chr19:58409964-58410091	NA		0.59	5.49E-08	-0.002	1
SE	TEAD4	+	chr12:2959947-2960353	NA		0.59	7.68E-06	-0.125	1
SE	ZNF584	+	chr19:58410005-58410091	NA		0.589	6.35E-08	-0.01	1
SE	EMC3-AS1	+	chr3:9987905-9988007	NA		0.589	0.003046176652	0.002	1
SE	SUZ12P1	+	chr17:30734896-30734943	NA		0.583	1.11E-06	0.02	1
SE	NSFL1C	-	chr20:1455713-1455719	NA		0.582	0	0.031	0.01245407629
SE	MGRN1	+	chr16:4686258-4686303	NA		0.582	1.44E-07	0.08	0.001210876276
SE	NIN	-	chr14:50756491-50758630	NA		0.581	0	0.004	1
SE	FGFR1OP2	+	chr12:26960514-26960628	NA		0.58	0	-0.019	1
SE	ZNF195	-	chr11:3373576-3373656	NA		0.579	0.006463478574	-0.055	1
SE	MLLT4	+	chr6:167913402-167913423	NA		0.579	4.69E-10	-0.104	1
SE	ZNF584	+	chr19:58409940-58410091	NA		0.579	3.95E-08	-0.006	1
SE	CTNND1	+	chr11:57789036-57789155	NA		0.578	0	0.05	1.32E-06
SE	FIRRE	-	chrX:131795808-131796331	NA		0.578	0.03072258172	-0.083	1
SE	PPIP5K2	+	chr5:103135992-103136114	NA		0.577	0.001101883147	-0.059	1

SE	EPB41L2	-	chr6:130870327-130870381	NA	0.576	5.39E-12	0.247	0
SE	TOX2	+	chr20:44065711-44066107	NA	0.576	0.02126672239	-0.051	1
SE	PER3	+	chr1:7787197-7787246	NA	0.575	0.003807338828	0.149	1
SE	C9orf3	+	chr9:94928424-94928531	NA	0.575	0.0007666247027	-0.073	1
SE	MCTP1	-	chr5:94917895-94917973	NA	0.575	1.56E-08	0.068	1
SE	WARS	-	chr14:100375282-100375350	NA	0.573	9.26E-05	-0.014	1
SE	TTC28	-	chr22:27990788-27990812	NA	0.572	6.78E-07	0.082	1
SE	ELF2	-	chr4:139116648-139116807	NA	0.572	0.001701782054	0.14	0.4389218514
SE	RPS24	+	chr10:78040200-78040225	NA	0.57	5.37E-11	0.104	0
SE	STAG3L3	-	chr7:73000357-73000481	NA	0.569	0.0004383795297	-0.004	1
SE	NUP214	+	chr9:131189052-131189131	NA	0.564	0.006949996165	-0.019	1
SE	CCDC88A	-	chr2:55303068-55303152	NA	0.561	0	0.064	0.05755593527
SE	CAST	+	chr5:96726753-96726859	NA	0.557	0	0.032	1
SE	AP1G1	-	chr16:71767876-71767885	NA	0.557	0	0.148	0.0005668235355
SE	WASH2P	+	chr2:113595062-113595161	NA	0.555	0.000350375615	-0.084	0.02421113543
SE	LIMCH1	+	chr4:41638931-41638967	NA	0.554	1.32E-12	-0.006	1
SE	PIN4	+	chrX:72186233-72186534	NA	0.554	0	-0.029	1
SE	AMPD2	+	chr1:109622184-109622353	NA	0.554	0.0002935232439	0.007	1
SE	SNX14	-	chr6:85538837-85538864	NA	0.554	0	0.052	0.0002910068944
SE	ZCWPW1	-	chr7:100407227-100407303	NA	0.552	3.10E-05	-0.048	1
SE	ZNF484	-	chr9:92875014-92875059	NA	0.552	3.08E-05	-0.017	1
SE	C14orf166	+	chr14:52004239-52004242	NA	0.55	0.0001448755793	0.005	0.005065734623
SE	EPB41L2	-	chr6:130870327-130870381	NA	0.548	4.47E-06	0.244	0
SE	C19orf60	+	chr19:18589412-18589683	NA	0.546	1.17E-08	-0.016	0.03770047692
SE	ITGB3BP	-	chr1:63447564-63447614	NA	0.545	0	0.156	1.01E-12
SE	KIAA1191	-	chr5:176359480-176359567	NA	0.544	0.0001530389937	0	1
SE	PPM1K	-	chr4:88278535-88278642	NA	0.544	0.0009616700701	0.114	0.01644211319
SE	CAST	+	chr5:96726793-96726859	NA	0.543	0.0001035888519	-0.001	1
SE	SLC7A11	-	chr4:138185120-138185244	NA	0.542	9.09E-08	-0.011	1
SE	ZCWPW1	-	chr7:100402515-100402576	NA	0.541	0.0008828287501	-0.087	7.43E-08
SE	FNBP1	-	chr9:129923843-129923996	NA	0.541	0	0.081	0.0009275162984
SE	ASAP2	+	chr2:9391061-9391196	NA	0.539	6.77E-06	0.072	1
SE	CEP72	+	chr5:665940-666099	NA	0.538	1.80E-07	0.045	1
SE	RNF146	+	chr6:127280515-127280604	NA	0.538	2.89E-08	0.118	8.93E-12
SE	TSSC4	+	chr11:2401838-2402147	NA	0.535	0	0.007	1
SE	WASF3	+	chr13:26680034-26680201	NA	0.534	6.92E-10	0.007	1
SE	FNBP1	-	chr9:129923843-129923939	NA	0.534	3.18E-05	-0.021	1
SE	MARK3	+	chr14:103498501-103498528	NA	0.529	0.0004916542622	-0.055	1
SE	RAB3GAP1	+	chr2:135169733-135169848	NA	0.527	5.95E-05	0	1
SE	CCDC14	-	chr3:123956354-123956427	NA	0.525	0.002598953941	-0.005	1
SE	PPIL3	-	chr2:200882341-200882382	NA	0.525	1.32E-06	-0.041	0.1038708471
SE	PIGQ	+	chr16:581198-581341	NA	0.524	0.0002419316532	0.001	1
SE	RP11-580I1(	-	chr17:47626546-47626627	NA	0.524	0.006170354981	-0.014	1
SE	MVD	-	chr16:88657145-88657247	NA	0.523	0.0001426700148	0.013	1
SE	FAM13B	-	chr5:138018954-138019146	NA	0.522	3.97E-08	-0.026	1
SE	NFYA	+	chr6:41080810-41080897	NA	0.521	5.31E-07	0.012	1
SE	LRCH3	+	chr3:197858833-197858905	NA	0.521	5.20E-11	0.132	1.39E-09
SE	FAM188A	-	chr10:15838227-15838279	NA	0.52	2.85E-13	0	1
SE	SEPT9	+	chr17:77402336-77402495	NA	0.519	4.46E-08	0	1
SE	CLSTN1	-	chr1:9756480-9756510	NA	0.516	0	0.04	0.1189545023
SE	FAM24B	-	chr10:122850423-122850520	NA	0.515	1.61E-09	-0.013	1
SE	SNRPN	+	chr15:24974641-24974957	NA	0.514	5.03E-05	0.028	1
SE	XPO6	-	chr16:28155701-28155771	NA	0.514	1.03E-05	0.029	1
SE	TTC28-AS1	+	chr22:27989881-27990026	NA	0.514	0.001921405105	0.011	1
SE	EEF1D	-	chr8:143592646-143592797	NA	0.512	4.25E-06	0.046	1
SE	EEF1D	-	chr8:143590607-143590729	NA	0.51	1.39E-05	-0.009	1
SE	TNFSF12-TI	+	chr17:7559623-7559702	NA	0.503	3.25E-06	-0.022	1
SE	INADL	+	chr1:62100318-62100408	NA	0.5	0.01919460471	-0.013	1
SE	MAGI1	-	chr3:65387142-65387226	NA	0.5	4.47E-07	0.237	3.26E-11
SE	MICAL3	-	chr22:17826453-17826516	NA	0.5	1.06E-09	-0.146	1
SE	ARL4A	+	chr7:12687634-12687728	NA	0.499	7.00E-10	0.109	0.2419974143
SE	EEF1D	-	chr8:143592646-143592942	NA	0.497	1.12E-11	0.038	1
SE	ZNF277	+	chr7:112286876-112287074	NA	0.497	0	0.002	1

SE	CTNND1	+	chr11:57789036-57789155	NA	0.495	1.21E-11	0.021	1
SE	MDM4	+	chr1:204537429-204537497	NA	0.494	4.14E-05	-0.063	1
SE	KIF3A	-	chr5:132706450-132706459	NA	0.49	2.45E-09	0.152	1
SE	RIF1	+	chr2:151480097-151480227	NA	0.489	0.002957482326	-0.051	1
SE	SUN1	+	chr7:849519-849603	NA	0.489	1.12E-05	-0.129	0.0748433781
SE	AIFM1	-	chrX:130155155-130155286	NA	0.488	0.003549029187	0.02	1
SE	GOLGA4	+	chr3:37266841-37266940	NA	0.487	0	-0.063	1
SE	TPK1	-	chr7:144681806-144681864	NA	0.484	0.01202735009	0.081	1
SE	TMEM25	+	chr11:118533419-118533551	NA	0.48	0.002210906602	-0.136	3.15E-14
SE	PMS2CL	+	chr7:6733398-6733527	NA	0.48	1.31E-10	0.153	0
SE	EEF1D	-	chr8:143590644-143590735	NA	0.479	0.001367554607	0.011	1
SE	DPM1	-	chr20:50941104-50941209	NA	0.474	0.0003324026152	-0.027	1
SE	EPB41L2	-	chr6:130870327-130870381	NA	0.474	3.36E-07	0.076	1
SE	DGKA	+	chr12:55932560-55932909	NA	0.472	0.002122617065	0.206	1
SE	NR4A1	+	chr12:52052473-52052645	NA	0.472	0.01007221058	0.044	1
SE	DGKH	+	chr13:42221161-42221394	NA	0.471	2.68E-05	0.235	1
SE	MRPS15	-	chr1:36461263-36461312	NA	0.47	0.003230017772	0.013	1
SE	LRRC27	+	chr10:132342212-132342271	NA	0.468	0.003432257142	-0.017	1
SE	CATSPER2	-	chr15:43647049-43647118	NA	0.467	0.002544829601	0.044	1
SE	CUL5	+	chr11:108104189-108104902	NA	0.466	0	-0.001	1
SE	TIA1	-	chr2:70228343-70228462	NA	0.465	4.53E-11	0.015	1
SE	UGGT2	-	chr13:95860787-95860883	NA	0.462	1.74E-10	0.001	1
SE	DNAJB6	+	chr7:157409794-157415529	NA	0.461	0.0002552904057	-0.014	1
SE	GPCPD1	-	chr20:5586193-5586269	NA	0.46	0.003724555554	-0.053	0.0005656385185
SE	ANKRD10	-	chr13:110894284-110894393	NA	0.459	0.01041046655	0.049	1
SE	GUK1	+	chr1:228141123-228141507	NA	0.459	8.84E-08	0.028	0.04222877495
SE	C7orf55-LU(	+	chr7:139375508-139375583	NA	0.459	0.003536476151	-0.022	1
SE	MCTP1	-	chr5:94917895-94917973	NA	0.457	5.72E-11	-0.056	1
SE	CIRBP	+	chr19:1273600-1273715	NA	0.456	1.72E-06	0.02	1
SE	USP45	-	chr6:99445796-99446463	NA	0.455	7.84E-09	-0.082	1
SE	PPIL3	-	chr2:200882341-200882435	NA	0.454	5.63E-11	-0.03	0.002990973779
SE	PFDN5	+	chr12:53296429-53296551	NA	0.453	0.0007066383201	-0.069	0.09450408021
SE	FAM221A	+	chr7:23692182-23694913	NA	0.453	0	0.037	1
SE	APLP1	+	chr19:35878081-35878108	NA	0.448	3.77E-07	0.027	6.52E-13
SE	FNIP2	+	chr4:158833800-158833890	NA	0.447	0.0001145993843	-0.039	1
SE	SEPT2	+	chr2:241316421-241316540	NA	0.447	0.001538786731	0.028	1
SE	DNAJC4	+	chr11:64231031-64231147	NA	0.445	0.03833779699	0.01	1
SE	CLASP1	-	chr2:121409023-121409047	NA	0.444	9.89E-07	0.166	5.97E-05
SE	CACTIN	-	chr19:3611228-3611364	NA	0.444	2.72E-05	0.006	1
SE	TEX22	+	chr14:105449358-105449409	NA	0.444	5.32E-13	-0.065	8.91E-05
SE	NADSYN1	+	chr11:71476032-71477438	NA	0.443	2.65E-05	0.064	4.34E-07
SE	FAM135A	+	chr6:70486166-70486244	NA	0.442	1.50E-07	-0.03	1
SE	MAP4K4	+	chr2:101868028-101868037	NA	0.441	0	0.268	0
SE	VPS13B	+	chr8:99507769-99507911	NA	0.44	0.00206399437	-0.009	1
SE	ERBB2IP	+	chr5:66072168-66072291	NA	0.439	0.005843897436	0.137	0.02706632387
SE	FBRS	+	chr16:30661303-30661333	NA	0.439	8.18E-06	-0.027	0.4139295319
SE	WDR27	-	chr6:169665485-169665556	NA	0.439	6.79E-07	-0.009	1
SE	XRRA1	-	chr11:74945017-74945085	NA	0.438	0.0001331087106	0.079	1
SE	LRRC75B	-	chr22:24588678-24589265	NA	0.438	0.0004754203316	-0.078	0.2099330039
SE	LETM2	+	chr8:38406945-38407038	NA	0.437	1.41E-06	0.028	1
SE	MED23	-	chr6:131615323-131615341	NA	0.436	3.90E-07	0.144	1
SE	ABHD14B	-	chr3:51971811-51971892	NA	0.435	4.64E-06	0.033	0.5163550836
SE	PUF60	-	chr8:143820665-143820716	NA	0.435	2.28E-10	0.041	0.0002170931953
SE	RALGAPA1	-	chr14:35700161-35700302	NA	0.434	0.003894370242	0.301	1
SE	CTNND1	+	chr11:57791491-57791673	NA	0.434	2.44E-08	0.043	1
SE	CTNND1	+	chr11:57791493-57791673	NA	0.434	2.12E-08	0.043	1
SE	TTC7A	+	chr2:46958494-46958552	NA	0.432	0.004065365915	0.005	1
SE	WARS	-	chr14:100375282-100375406	NA	0.432	0.001631060496	-0.014	1
SE	PSMA3-AS1	-	chr14:58288717-58288813	NA	0.432	7.31E-06	-0.008	1
SE	NOD1	-	chr7:30463608-30463698	NA	0.431	0.0004537657274	0.097	1
SE	HPS1	-	chr10:98431130-98431291	NA	0.43	1.28E-05	-0.008	1
SE	TSSC4	+	chr11:2401975-2402147	NA	0.429	1.03E-08	0.003	1
SE	C4orf36	-	chr4:86932221-86932319	NA	0.427	0.002784788117	-0.044	1

SE	GOLGB1	-	chr3:121719645-121719753	NA	0.427	0.04360660297	0.092	1
SE	CEP57L1	+	chr6:109106034-109106090	NA	0.427	0.0007319614165	0.009	1
SE	ZFAND5	-	chr9:72363469-72363606	NA	0.426	0	0.023	0.5651198145
SE	DBR1	-	chr3:138163777-138163858	NA	0.426	0	-0.017	1
SE	IMMP1L	-	chr11:31460625-31460714	NA	0.426	0.00831961416	-0.017	1
SE	OFD1	+	chrX:13763744-13763855	NA	0.423	0	-0.022	0.6703107187
SE	GOLGB1	-	chr3:121719645-121719768	NA	0.423	0.01706419273	0.084	1
SE	AC024560.3	-	chr3:197624471-197624720	NA	0.423	4.05E-12	0.029	1
SE	NAGK	+	chr2:71070740-71070839	NA	0.422	0.003548756426	-0.047	1
SE	ORMDL1	-	chr2:189783013-189783123	NA	0.422	0	-0.041	0.9341657548
SE	MON2	+	chr12:62566013-62566031	NA	0.421	0.0009638969816	0.045	1
SE	RALGAPA2	-	chr20:20396694-20396734	NA	0.421	0.0255521065	-0.094	3.15E-14
SE	SEMA4F	+	chr2:74662731-74662825	NA	0.419	0.0004418183214	0.036	1
SE	RAB34	-	chr17:28717212-28717291	NA	0.418	4.03E-06	0.012	1
SE	C1orf86	-	chr1:2192844-2192975	NA	0.417	6.06E-08	-0.005	1
SE	ELFN2	-	chr22:37417768-37417919	NA	0.417	0.001125297341	-0.007	1
SE	HMGN1	-	chr21:39345829-39345958	NA	0.417	0	0.033	1
SE	PKIG	+	chr20:44582584-44582731	NA	0.416	0	-0.101	3.41E-05
SE	MBD1	-	chr18:50271468-50271540	NA	0.415	0.0009073322787	-0.06	0.04015298357
SE	FBXO24	+	chr7:100595101-100595223	NA	0.414	6.11E-06	0.03	1
SE	SERPINB6	-	chr6:2968778-2968913	NA	0.414	0.0003879148638	0.021	1
SE	MLLT6	+	chr17:38721877-38722227	NA	0.413	3.20E-06	-0.089	1
SE	GPR157	-	chr1:9105485-9105680	NA	0.412	0.00512657653	0.078	1
SE	HMGN1	-	chr21:39345829-39345958	NA	0.412	7.73E-13	0.047	0
SE	SLC35B1	-	chr17:49707342-49707444	NA	0.41	0.0006971692881	0.01	1
SE	PILRB	+	chr7:100353894-100354012	NA	0.41	9.00E-11	0.032	0.2268086619
SE	MPRIP	+	chr17:17180606-17180669	NA	0.41	0.00173447688	-0.075	0.02200380162
SE	NFASC	+	chr1:204962107-204962158	NA	0.41	2.42E-06	0.178	8.88E-09
SE	KIAA1109	+	chr4:122275971-122276022	NA	0.408	1.95E-08	0.153	1
SE	C17orf62	-	chr17:82447579-82447621	NA	0.405	4.76E-07	-0.007	1
SE	CDK5RAP2	-	chr9:120460571-120460747	NA	0.404	7.74E-05	-0.019	1
SE	THAP6	+	chr4:75542408-75542486	NA	0.404	0.001289105061	-0.1	1
SE	MDM4	+	chr1:204537458-204537497	NA	0.403	4.41E-05	-0.058	1
SE	HNRNPA2B	-	chr7:26190992-26191128	NA	0.402	0	-0.001	1
SE	TEX30	-	chr13:102770011-102770086	NA	0.4	4.43E-06	-0.002	0.6358331318
SE	UBA2	+	chr19:34430092-34430186	NA	0.399	5.38E-06	-0.018	1
SE	PPP1R3E	-	chr14:23300106-23300325	NA	0.399	0.0001503356103	0.009	1
SE	ZFYVE27	+	chr10:97743093-97743164	NA	0.398	5.08E-07	-0.01	1
SE	IFT122	+	chr3:129461227-129461304	NA	0.398	0.005789128017	-0.073	1
SE	PLEKHA8	+	chr7:30050433-30050474	NA	0.397	4.16E-05	-0.004	1
SE	ZNF142	-	chr2:218651700-218651822	NA	0.397	0.01252426593	0.089	1
SE	ATXN2	-	chr12:111464661-111464715	NA	0.397	5.72E-05	0.005	1
SE	ELOVL5	-	chr6:53290400-53290535	NA	0.395	0.003902246943	-0.129	1
SE	GLS	+	chr2:190920214-190920248	NA	0.395	0.0002389327734	-0.044	1
SE	GUK1	+	chr1:228141123-228141288	NA	0.395	1.21E-08	0.032	1
SE	BCL2L2	+	chr14:23308815-23308932	NA	0.394	1.28E-06	-0.002	1
SE	SYTL4	-	chrX:100703092-100703185	NA	0.392	2.13E-05	0.038	1
SE	ZNF383	+	chr19:37219598-37219749	NA	0.392	0.007705740173	-0.042	1
SE	DUSP12	+	chr1:161751131-161751377	NA	0.39	0.001440110946	-0.062	1
SE	MYO9A	-	chr15:71892964-71893177	NA	0.389	0.001802867873	-0.135	1
SE	SNX25	+	chr4:185351444-185351609	NA	0.389	5.65E-12	-0.003	1
SE	OXR1	+	chr8:106737519-106737600	NA	0.389	1.25E-11	0.169	3.60E-09
SE	TIAM1	-	chr21:31276731-31276908	NA	0.388	0.02905195337	0.014	1
SE	EPB41L2	-	chr6:130876673-130876775	NA	0.387	1.18E-05	0.1	0.2015795536
SE	C1orf86	-	chr1:2192646-2192975	NA	0.387	9.33E-08	0.008	0.001859775651
SE	EXOC7	-	chr17:76091142-76091235	NA	0.387	0	0.128	0.0001621404627
SE	SNHG17	-	chr20:38422091-38422241	NA	0.386	7.64E-10	-0.025	1
SE	CDKL3	-	chr5:134359896-134360091	NA	0.385	0.04552554741	-0.095	1
SE	C21orf62-AS	+	chr21:32788611-32788701	NA	0.385	0.01673793337	-0.091	1
SE	CTAGE5	+	chr14:39320927-39321056	NA	0.384	9.02E-05	0.038	0.2046867693
SE	ST3GAL6-A	-	chr3:98718423-98718521	NA	0.384	0.01134847022	0.016	1
SE	USP36	-	chr17:78797785-78797879	NA	0.382	2.27E-05	0.033	1
SE	STIM1	+	chr11:4084672-4084765	NA	0.381	6.19E-06	-0.006	1

SE	ZCWPW2	+	chr3:28478813-28478931	NA		0.377	4.60E-06	0.007	1
SE	SVIL	-	chr10:29532001-29532172	NA		0.376	2.63E-06	-0.058	1
SE	HMGN1	-	chr21:39347246-39347292	NA		0.375	7.19E-11	0.041	1
SE	ZNF519	-	chr18:14080188-14080266	NA		0.373	2.79E-06	-0.051	1
SE	CIRBP	+	chr19:1273493-1273715	NA		0.369	9.30E-07	0.015	1
SE	ARMC4	-	chr10:27968922-27969018	NA		0.369	0.01357652802	0.072	1
SE	PCBP4	-	chr3:51961748-51961894	NA		0.367	0.01272273568	0.008	0.05908775887
SE	ARHGAP21	-	chr10:24622732-24622762	NA		0.367	1.48E-07	0.028	1
SE	DCAF8	-	chr1:160261284-160261350	NA		0.367	3.58E-07	0.043	0.3245857962
SE	SH3GLB2	-	chr9:129010669-129010693	NA		0.367	0.0009146336722	-0.014	1
SE	NUDT22	+	chr11:64227567-64227666	NA		0.367	3.01E-09	-0.046	6.37E-07
SE	GAA	+	chr17:80101810-80101890	NA		0.367	8.37E-06	-0.036	1
SE	LAS1L	-	chrX:65524563-65524614	NA		0.365	2.58E-06	0.054	0.2567188962
SE	MCTP1	-	chr5:94799012-94799132	NA		0.365	1.48E-08	0.06	1
SE	DPY19L2	-	chr12:63590998-63591127	NA		0.364	0.001505065008	-0.02	1
SE	STRADA	-	chr17:63726637-63726695	NA		0.364	9.33E-06	0.015	1
SE	RIN3	+	chr14:92615406-92615479	NA		0.363	9.41E-05	-0.022	1
SE	SP140L	+	chr2:230328756-230328831	NA		0.363	0.0001674844062	0.006	1
SE	KLHDC1	+	chr14:49728925-49729009	NA		0.363	0.04492330258	0.006	1
SE	PDPR	+	chr16:70114820-70114930	NA		0.362	0.01384958618	-0.086	1
SE	MYO5A	-	chr15:52337809-52337884	NA		0.362	0	-0.191	1
SE	PICALM	-	chr11:85978069-85978093	NA		0.361	0	-0.134	1.09E-12
SE	BORA	+	chr13:72734959-72735005	NA		0.361	0.0002217088096	-0.043	1
SE	FAM24B	-	chr10:122850423-122850550	NA		0.361	1.17E-08	-0.007	1
SE	NHLRC3	+	chr13:39042104-39042305	NA		0.36	5.79E-08	0.05	0.8245268707
SE	EED	+	chr11:86264171-86264263	NA		0.359	0.02081308966	-0.052	1
SE	ASAH2B	+	chr10:50745127-50745274	NA		0.359	0.000381490862	-0.038	1
SE	FAM195A	+	chr16:646471-646608	NA		0.358	0.003349315801	0.022	1
SE	ZFX	+	chrX:24161714-24161842	NA		0.357	0.03417336386	0.161	1
SE	TEX15	-	chr8:30874936-30875102	NA		0.357	2.61E-05	-0.077	1
SE	TTLL3	+	chr3:9818821-9818920	NA		0.357	0.0009150251426	-0.02	1
SE	KIF23	+	chr15:69425281-69425323	NA		0.356	4.47E-13	-0.081	0.01190529972
SE	NBEAL1	+	chr2:203115984-203116070	NA		0.355	0.0002383144115	-0.046	1
SE	KLHL24	+	chr3:183636220-183636311	NA		0.354	7.54E-08	0.031	1
SE	SAMD4B	+	chr19:39351816-39352374	NA		0.354	0.01422507111	0.042	1
SE	ZNF786	-	chr7:149080590-149080717	NA		0.354	0.04214164797	-0.03	1
SE	C16orf13	-	chr16:635517-635774	NA		0.353	0.0004141608316	-0.024	1
SE	BROX	+	chr1:222715144-222715269	NA		0.353	0.00330261625	0.055	1
SE	SREBF2	+	chr22:41904534-41904732	NA		0.353	0.02530794582	0.002	4.37E-07
SE	ARHGEF11	-	chr1:156938417-156938513	NA		0.352	5.07E-07	0.013	1
SE	ITPR1	+	chr3:4653841-4653886	NA		0.351	0.008916593862	-0.112	1
SE	TOP1MT	-	chr8:143334107-143334285	NA		0.35	1.38E-06	0.021	1
SE	TIA1	-	chr2:70229058-70229091	NA		0.349	1.08E-13	-0.024	1
SE	TANC2	+	chr17:63412679-63412709	NA		0.348	1.26E-07	0.311	1
SE	C17orf62	-	chr17:82446622-82446692	NA		0.348	5.97E-10	-0.007	1
SE	MOK	-	chr14:102265822-102265912	NA		0.346	0.0005890502783	-0.022	1
SE	IFT122	+	chr3:129458598-129458677	NA		0.346	0.03306288922	-0.086	0.341561334
SE	KIF1B	+	chr1:10326110-10326359	NA		0.345	0.001701782054	0.184	3.19E-09
SE	POLE2	-	chr14:49674349-49674427	NA		0.345	4.09E-07	-0.001	0.537773333
SE	FAM161A	-	chr2:61836582-61836872	NA		0.345	5.12E-05	0.056	1
SE	SYNE1	-	chr6:152145486-152145555	NA		0.343	0.02105099548	-0.205	8.16E-11
SE	TMEM218	-	chr11:125102131-125102302	NA		0.343	0.03972173331	0.001	1
SE	MCM9	-	chr6:118856370-118856545	NA		0.342	0.002662136025	0.033	1
SE	MRPS18C	+	chr4:83458345-83458429	NA		0.342	3.58E-13	-0.007	1
SE	TNRC18	-	chr7:5329936-5329969	NA		0.341	0.03499961163	-0.086	0.07802427692
SE	GOLGA6L9	+	chr15:82432571-82432631	NA		0.341	0.009416548364	-0.139	1
SE	NPHP3	-	chr3:132696730-132696813	NA		0.34	1.09E-05	0.034	1
SE	TPCN2	+	chr11:69067502-69067605	NA		0.34	6.03E-05	-0.052	1
SE	FAM13B	-	chr5:137946227-137946311	NA		0.339	3.76E-05	-0.061	0.00986286595
SE	SCML1	+	chrX:17744070-17744219	NA		0.339	0.001379584947	-0.062	1
SE	MFSD12	-	chr19:3542775-3542977	NA		0.339	4.34E-05	0.04	1
SE	PPIL3	-	chr2:200885261-200885367	NA		0.339	1.93E-05	-0.034	1
SE	HMMR	+	chr5:163467700-163467748	NA		0.338	1.27E-11	-0.051	1

SE	NF1	+	chr17:31252937-31253000	NA	0.338	2.85E-05	-0.015	1
SE	CD27-AS1	-	chr12:6450892-6450980	NA	0.337	0.02475961249	0.024	1
SE	SUV420H2	+	chr19:55343977-55343997	NA	0.336	0.001968293186	-0.035	1
SE	YIPF1	-	chr1:53855000-53855051	NA	0.335	0.0006432768657	0.023	1
SE	N4BP2	+	chr4:40100035-40100126	NA	0.335	0.03898426062	0.172	1
SE	March2	+	chr19:8418741-8418807	NA	0.335	6.70E-05	-0.03	1
SE	RAD51AP1	+	chr12:4545781-4545832	NA	0.335	4.54E-05	-0.086	1
SE	TOM1L2	-	chr17:17857768-17857855	NA	0.335	6.07E-07	0.04	1
SE	ZDHHC15	-	chrX:75429077-75429198	NA	0.334	0.01080928689	-0.012	1
SE	PCOLCE	+	chr7:100605504-100605812	NA	0.334	0.000261928969	-0.017	0.4364746126
SE	PCBP1-AS1	-	chr2:70059642-70059680	NA	0.334	0.003387004345	0.025	1
SE	MRPS28	-	chr8:80028694-80028796	NA	0.333	0.00106274245	0.081	1
SE	RCN1	+	chr11:32103003-32103202	NA	0.332	0.04655359236	0.005	1
SE	ANO6	+	chr12:45228167-45228273	NA	0.332	0.04369154184	-0.007	1
SE	AGRN	+	chr1:1051031-1051043	NA	0.332	1.00E-04	0.256	0
SE	FHOD3	+	chr18:36687127-36687178	NA	0.331	0.0391410956	0.056	1
SE	ENTHD2	-	chr17:81232314-81232518	NA	0.331	0.0001166155859	-0.065	0.02667018268
SE	PCOLCE	+	chr7:100605675-100605812	NA	0.33	5.14E-05	-0.01	1
SE	AURKB	-	chr17:8210176-8210225	NA	0.33	1.60E-09	0.156	7.96E-09
SE	RP11-295P9	+	chr10:13654088-13654258	NA	0.33	0.003522034591	0.012	1
SE	PRPF40B	+	chr12:49632595-49632623	NA	0.329	0.001138009614	-0.077	0.07353316925
SE	ERMARD	+	chr6:169753037-169753181	NA	0.329	0.0001454345137	0.035	1
SE	DCAF8	-	chr1:160261284-160261325	NA	0.329	9.41E-05	0.019	1
SE	WIBG	-	chr12:55927075-55927267	NA	0.329	0.003959039809	0.088	1
SE	ACTR3B	+	chr7:152811696-152811733	NA	0.328	1.08E-13	-0.062	0.06486921955
SE	ZMYM1	+	chr1:35093319-35093441	NA	0.328	0.004503457102	0.114	1
SE	CCHCR1	-	chr6:31157384-31157500	NA	0.328	1.20E-06	0.035	1
SE	MTIF3	-	chr13:27441016-27441077	NA	0.327	0.0001959215647	0.084	1
SE	AHI1	-	chr6:135302711-135302817	NA	0.327	0.01187945836	0.069	0.2343642136
SE	C1orf162	+	chr1:111477650-111477775	NA	0.327	0.007797388202	-0.035	1
SE	SREK1	+	chr5:66156064-66158932	NA	0.327	1.42E-06	0.009	1
SE	SLC25A36	+	chr3:140966933-140967036	NA	0.326	1.18E-10	-0.019	1
SE	ELMOD3	+	chr2:85371439-85371562	NA	0.326	0.02558045263	-0.018	1
SE	SEPT7	+	chr7:35831491-35832034	NA	0.326	0	0.096	0.003911619295
SE	MSH5-SAPC	+	chr6:31762789-31763168	NA	0.326	3.62E-07	-0.06	0.06333810809
SE	GORAB	+	chr1:170536309-170536421	NA	0.325	0.02241166849	0.006	1
SE	PCMTD1	-	chr8:51839446-51839638	NA	0.325	1.42E-06	0.04	3.17E-07
SE	RFX2	-	chr19:6026162-6026237	NA	0.324	0.0003095361672	-0.076	1
SE	ARHGEF10	+	chr8:1880047-1880164	NA	0.324	0.000880675005	0.082	1
SE	ILF3	+	chr19:10684415-10684476	NA	0.323	1.34E-11	0.002	1
SE	INCENP	+	chr11:62141499-62141511	NA	0.323	0.02376597087	-0.121	0.0004036382164
SE	NLRX1	+	chr11:119172355-119172425	NA	0.323	8.79E-05	-0.011	1
SE	NDEL1	+	chr17:8463319-8463354	NA	0.323	0.001234956404	-0.03	0.4336648067
SE	HMGN1	-	chr21:39345829-39347292	NA	0.323	0	0.039	1
SE	MPHOSPH9	-	chr12:123226287-123226351	NA	0.322	8.53E-11	0.094	1
SE	KLHL24	+	chr3:183636220-183636311	NA	0.322	0.0001503356103	0.018	1
SE	PPIP5K2	+	chr5:103186319-103186439	NA	0.322	0.0002061758732	0.035	1
SE	ZNF772	-	chr19:57475659-57475786	NA	0.322	0.008723427672	0.14	1
SE	SPATA4	-	chr4:176192957-176193076	NA	0.321	0.00010612589	0.027	1
SE	TMEM39A	-	chr3:119435754-119435935	NA	0.321	2.44E-10	0.155	1
SE	PEX7	+	chr6:136869889-136870003	NA	0.32	0.002238644159	-0.017	1
SE	ZNF711	+	chrX:85246929-85247183	NA	0.32	7.32E-08	0.024	1
SE	SPEF2	+	chr5:35644354-35644525	NA	0.32	9.18E-05	-0.102	1
SE	BAZ1A	-	chr14:34786125-34786221	NA	0.32	0.001379584947	-0.027	1
SE	SLC2A11	+	chr22:23877384-23877869	NA	0.319	9.42E-06	-0.002	0.6223901432
SE	TSSC4	+	chr11:2401838-2401979	NA	0.319	5.66E-06	-0.001	1
SE	TRIM16	-	chr17:15651090-15651946	NA	0.319	0.0007828582725	-0.066	0.005247877485
SE	TOP1MT	-	chr8:143332486-143332657	NA	0.317	1.32E-09	0.052	0.08678612438
SE	TRAF3IP2-A	+	chr6:111574620-111574658	NA	0.317	0.01210955425	-0.03	1
SE	AAK1	-	chr2:69495984-69496080	NA	0.316	0.006955259997	0.164	1
SE	USP8	+	chr15:50484274-50484361	NA	0.316	7.79E-08	-0.012	1
SE	CCP110	+	chr16:19524779-19525019	NA	0.315	0.002784788117	-0.075	1
SE	GOLT1B	+	chr12:21507943-21508109	NA	0.315	5.26E-07	0.16	1

SE	SUMF2	+	chr7:56074113-56074218	NA	0.315	0	-0.024	0.03608106686
SE	TCEA1	-	chr8:54002891-54002940	NA	0.315	0.01533786035	0.01	0.05019875989
SE	MYO6	+	chr6:75911671-75911698	NA	0.315	3.49E-11	0.034	1
SE	SENP7	-	chr3:101417592-101417790	NA	0.314	1.18E-10	0.012	1
SE	PPIP5K2	+	chr5:103187313-103187376	NA	0.314	2.71E-06	0.043	0.2318864067
SE	BPTF	+	chr17:67875555-67875744	NA	0.314	9.73E-12	0.016	1
SE	RBM6	+	chr3:49975322-49975392	NA	0.313	2.49E-06	-0.019	1
SE	C14orf79	+	chr14:104989631-104989707	NA	0.313	0.03336016912	-0.064	1
SE	NUDT13	+	chr10:73124213-73124320	NA	0.313	0.0005127634016	-0.006	1
SE	GATAD2A	+	chr19:19436130-19436219	NA	0.313	0.00140484798	-0.062	1
SE	RGS19	-	chr20:64076856-64076954	NA	0.313	0.003517135917	-0.015	1
SE	MEIG1	+	chr10:14970247-14970546	NA	0.313	0.03122985027	0.076	1
SE	CD27-AS1	-	chr12:6450869-6450980	NA	0.313	0.01675843214	0.003	1
SE	SLC29A1	+	chr6:44226060-44226167	NA	0.312	1.18E-05	-0.047	1
SE	PDPR	+	chr16:70114869-70114930	NA	0.311	0.0008000210449	-0.086	1
SE	EEF1D	-	chr8:143590644-143590738	NA	0.311	0.0007911413037	-0.013	1
SE	SLC9B1	-	chr4:102949256-102949427	NA	0.311	0.006378477914	0.17	1
SE	ARNTL2	+	chr12:27370128-27370230	NA	0.309	0.02166060778	0.007	1
SE	KIAA1468	+	chr18:62279773-62279856	NA	0.309	0.03090604922	0.109	2.86E-13
SE	DST	-	chr6:56463045-56463156	NA	0.309	0	-0.009	0.0004247547399
SE	ZDHHC16	+	chr10:97448189-97448350	NA	0.309	0.006465468267	0.003	1
SE	C17orf62	-	chr17:82449173-82449292	NA	0.309	1.37E-07	-0.014	1
SE	PRPF40A	-	chr2:152679055-152679109	NA	0.309	0	0.017	1
SE	DHX34	+	chr19:47376914-47376989	NA	0.308	0.008437561811	-0.001	1
SE	TCEA1	-	chr8:54002891-54003092	NA	0.308	0.0009906262505	0.006	0.002119978404
SE	JKAMP	+	chr14:59486680-59486804	NA	0.307	2.33E-12	0.003	1
SE	UBXN2B	+	chr8:58413298-58413371	NA	0.307	0.0001467205675	0.006	1
SE	BORA	+	chr13:72731280-72731387	NA	0.306	2.29E-05	-0.032	2.61E-06
SE	TMEM218	-	chr11:125102131-125102317	NA	0.306	0.009312346136	-0.001	1
SE	JKAMP	+	chr14:59486730-59486804	NA	0.305	1.06E-12	0.002	1
SE	ZNF133	+	chr20:18289898-18290074	NA	0.305	0.01781677028	-0.051	1
SE	RP11-452F1	+	chr1:222823855-222823945	NA	0.305	2.24E-05	0.053	1
SE	SCML1	+	chrX:17744070-17744219	NA	0.304	5.26E-08	0.079	1
SE	FECH	-	chr18:57585863-57585973	NA	0.304	0.02354481015	-0.01	1
SE	THRB	-	chr3:24297225-24297371	NA	0.304	0.005335128453	-0.153	1
SE	C17orf62	-	chr17:82447579-82447646	NA	0.304	0.01212190181	-0.058	5.68E-07
SE	RIPK2	+	chr8:89762828-89762982	NA	0.303	0.007952215332	-0.015	1
SE	METTL25	+	chr12:82456726-82456820	NA	0.303	0.02240571123	0.006	1
SE	SPEG	+	chr2:219444029-219444059	NA	0.302	0.002168602909	-0.005	1
SE	CCDC176	+	chr14:74046059-74046130	NA	0.302	0.01050857481	0.054	1
SE	FBXO44	+	chr1:11655327-11655699	NA	0.302	0.001244028305	-0.007	1
SE	TPCN2	+	chr11:69079833-69079883	NA	0.302	0.004265354996	-0.156	8.11E-05
SE	RBM6	+	chr3:49972058-49972148	NA	0.301	1.54E-10	-0.019	1
SE	ULK4	-	chr3:41819422-41819506	NA	0.301	8.15E-05	-0.019	1
SE	SBNO2	-	chr19:1147308-1147420	NA	0.3	0.001484505828	-0.001	1
SE	TCF7	+	chr5:134144796-134144869	NA	0.3	0.003218971638	0.242	2.65E-09
SE	NPHP3	-	chr3:132717954-132718117	NA	0.3	0.0001161877562	0.042	1
SE	PRIMPOL	+	chr4:184682247-184682336	NA	0.3	0.001312112384	-0.063	1
SE	COA1	-	chr7:43665657-43665722	NA	0.299	5.25E-12	0.171	3.71E-07
SE	FBXL4	-	chr6:98880552-98880624	NA	0.299	0.0008985289339	-0.029	0.06462676519
SE	PTK2	-	chr8:140925660-140925749	NA	0.299	1.81E-10	-0.015	1
SE	UBE2G2	-	chr21:44774566-44774741	NA	0.299	1.20E-05	0.01	1
SE	NPHP3	-	chr3:132699352-132699450	NA	0.298	0.002000095534	-0.089	0.6590073355
SE	SEC31A	-	chr4:82862533-82862572	NA	0.298	6.25E-12	-0.015	1
SE	SLC25A22	-	chr11:796042-796367	NA	0.298	0.0003906458635	-0.071	1
SE	WDR27	-	chr6:169670568-169670693	NA	0.297	2.79E-06	0.007	1
SE	ADAMTS6	-	chr5:65470777-65471142	NA	0.295	6.69E-05	-0.013	1
SE	WDR35	-	chr2:19960553-19960614	NA	0.295	3.39E-07	-0.011	1
SE	SGCE	-	chr7:94644605-94644667	NA	0.295	0.002784788117	0.061	1
SE	ENOSF1	-	chr18:691203-691276	NA	0.295	0.0006544003966	-0.093	1
SE	ANKMY1	-	chr2:240557189-240557352	NA	0.295	0.009947313719	-0.203	1
SE	MTCL1	+	chr18:8809447-8809561	NA	0.295	3.95E-10	-0.035	1
SE	FAM118B	+	chr11:126229224-126229336	NA	0.294	0.006913625577	-0.064	1

SE	EPB41L1	+	chr20:36195328-36195364	NA	0.293	0.001588997599	0.136	7.06E-06
SE	MTA1	+	chr14:105445666-105445852	NA	0.292	0	-0.047	1
SE	ZMYM1	+	chr1:35093393-35093441	NA	0.292	0.0004387441602	0.15	1
SE	AP4M1	+	chr7:100102245-100102393	NA	0.292	1.62E-12	0.016	0.01138816182
SE	ZNF83	-	chr19:52616717-52616815	NA	0.291	1.25E-05	0.035	1
SE	CD320	-	chr19:8305030-8305805	NA	0.291	0.02730924673	-0.073	0.05327408743
SE	KIAA1109	+	chr4:122226706-122226835	NA	0.29	0.005433588589	0.055	1
SE	RFWD2	-	chr1:176136487-176136547	NA	0.29	2.21E-05	0.009	1
SE	TMEM143	-	chr19:48360071-48360176	NA	0.29	0.04513725149	-0.053	1
SE	ARAP1	-	chr11:72732514-72732597	NA	0.29	0.002607277755	-0.012	1
SE	MRPL22	+	chr5:154950802-154950938	NA	0.289	1.18E-07	-0.09	1.19E-13
SE	PRDM2	+	chr1:13778417-13782831	NA	0.289	2.58E-09	0.016	1
SE	TYSND1	-	chr10:70143841-70143972	NA	0.289	0.000311701948	0.001	1
SE	SYNJ1	-	chr21:32634860-32634881	NA	0.289	6.83E-05	0.129	1
SE	PKIG	+	chr20:44585099-44585204	NA	0.289	0.002174269931	-0.012	2.33E-10
SE	ARPP19	-	chr15:52564189-52564246	NA	0.288	3.04E-05	-0.018	1
SE	CLSTN1	-	chr1:9737497-9737554	NA	0.288	0	0.088	0
SE	NAGPA	-	chr16:5028296-5028398	NA	0.287	0.00234344699	0.026	0.1222207181
SE	CAPN7	+	chr3:15245525-15245671	NA	0.287	0.001480159749	-0.027	1.99E-08
SE	FANCA	-	chr16:89762637-89762793	NA	0.287	5.88E-11	0.013	1
SE	TMEM117	+	chr12:44143524-44143624	NA	0.285	0.006170354981	-0.014	1
SE	PILRB	+	chr7:100353894-100354012	NA	0.284	7.18E-05	0.056	0.05251272139
SE	PQLC3	+	chr2:11172750-11172824	NA	0.284	0.005969129601	0.138	1
SE	PTPRD	-	chr9:8437197-8437239	NA	0.283	6.82E-05	0.04	1
SE	ATP5H	-	chr17:75039871-75040041	NA	0.283	0.01033447427	-0.118	1
SE	BAZ2B	-	chr2:159430862-159431156	NA	0.282	0.02202842779	0.033	1
SE	FNBP1	-	chr9:129923843-129924026	NA	0.282	0.004212179557	-0.012	1
SE	SPEG	+	chr2:219444024-219444059	NA	0.281	0.001400638034	0	1
SE	AC024560.3	-	chr3:197622186-197622330	NA	0.281	3.78E-07	-0.124	2.03E-05
SE	TCF20	-	chr22:42168608-42168736	NA	0.28	0.04608462819	0.218	0
SE	CDK16	+	chrX:47222260-47222380	NA	0.28	0.004926977751	0.032	0.9951118393
SE	NME4	+	chr16:398207-398385	NA	0.28	0.0349971465	0.01	1
SE	MAST1	+	chr19:12865315-12865481	NA	0.28	0.03825624706	0.001	1
SE	SPATS2	+	chr12:49371227-49371290	NA	0.28	1.33E-05	0.122	0.002406835435
SE	IKZF4	+	chr12:56024661-56024905	NA	0.279	0.004728450157	0.158	1
SE	FOXD3-AS1	-	chr1:63321664-63322080	NA	0.279	0.002721675563	0.068	0.04617524532
SE	APAF1	+	chr12:98703370-98703499	NA	0.278	0.001135475143	-0.039	0.9007699827
SE	CASK	-	chrX:41555599-41555635	NA	0.278	1.34E-11	0.011	1
SE	C9orf89	+	chr9:93111878-93111938	NA	0.278	1.56E-08	-0.073	3.47E-06
SE	SUZ12P1	+	chr17:30743300-30743369	NA	0.278	0.0002914239142	0.008	1
SE	CAMTA2	-	chr17:4982088-4982227	NA	0.274	0.002049001138	0.018	1
SE	LUC7L3	+	chr17:50738136-50738199	NA	0.274	3.31E-09	-0.171	1
SE	ITGB3BP	-	chr1:63446805-63446856	NA	0.274	0	-0.009	1
SE	TBC1D8B	+	chrX:106839325-106839457	NA	0.271	4.34E-05	-0.054	1
SE	CDK5RAP2	-	chr9:120460571-120460667	NA	0.271	3.89E-05	-0.023	1
SE	ALKBH8	-	chr11:107549752-107549823	NA	0.271	0.0001857822036	-0.059	1
SE	WDR35	-	chr2:19932382-19932447	NA	0.27	8.36E-07	-0.002	1
SE	KIAA1109	+	chr4:122325854-122325917	NA	0.27	0.01494049834	-0.056	0.0001767220318
SE	CD27-AS1	-	chr12:6450340-6450980	NA	0.27	0.01669192307	0.009	1
SE	RFX3	-	chr9:3301545-3301620	NA	0.269	3.88E-05	0.042	0.0003000116087
SE	SLC25A10	+	chr17:81713261-81713506	NA	0.269	0.001237237216	-0.015	1
SE	SEC31A	-	chr4:82862533-82862572	NA	0.268	7.13E-06	0.022	1
SE	TPM1	+	chr15:63061712-63061788	NA	0.268	6.41E-13	0.07	5.59E-09
SE	DLEU1	+	chr13:50083141-50083194	NA	0.268	8.10E-07	-0.098	0.003387663196
SE	BCOR	-	chrX:40071636-40071690	NA	0.268	0.002441790178	0.064	1
SE	NAGLU	+	chr17:42537397-42537545	NA	0.267	0.002270954097	-0.064	4.33E-07
SE	MCCC2	+	chr5:71626639-71626753	NA	0.267	2.16E-05	-0.016	1
SE	PPIP5K2	+	chr5:103187313-103187376	NA	0.267	0.02576515749	0.085	0.08718094723
SE	NBN	-	chr8:89981952-89982002	NA	0.266	0.02189479798	-0.016	1
SE	CTNND1	+	chr11:57806460-57806478	NA	0.266	4.23E-10	0.196	0
SE	FAM13B	-	chr5:137956476-137956542	NA	0.265	0.002442117802	-0.064	0.9867544371
SE	R3HDM1	+	chr2:135616151-135616193	NA	0.265	0.01888167559	-0.014	1
SE	CAMTA2	-	chr17:4982088-4982175	NA	0.265	0.003568838564	0.013	1

SE	LRRFIP1	+	chr2:237751199-237751271	NA	0.265	0	0.059	0
SE	ATAT1	+	chr6:30640534-30640603	NA	0.265	0.02320561358	-0.094	1.11E-12
SE	RHOQ	+	chr2:46543753-46543812	NA	0.264	0.005789128017	-0.002	1
SE	FAM122B	-	chrX:134772142-134772283	NA	0.264	2.18E-10	0.016	1
SE	ARHGEF3	-	chr3:57007181-57007336	NA	0.264	0.001289105061	-0.045	1
SE	PRR3	+	chr6:30561327-30561508	NA	0.264	0.01244086474	-0.031	0.3827413428
SE	POLDIP3	-	chr22:42601969-42602107	NA	0.263	0.007773580358	-0.067	0.07531317234
SE	PARD3	-	chr10:34372497-34372536	NA	0.263	8.40E-06	0.039	0.2716532777
SE	SON	+	chr21:33572550-33572630	NA	0.263	0	-0.024	1
SE	RP11-1212A	-	chr16:18372577-18372854	NA	0.263	0.03838602055	-0.072	1
SE	BRD8	-	chr5:138159554-138159599	NA	0.262	0.0001421619531	0.046	6.12E-06
SE	MFSD6	+	chr2:190415291-190415413	NA	0.262	0.01422507111	0.016	1
SE	TCEA1	-	chr8:54002891-54003086	NA	0.261	0.0075854768	0.005	0.4694765891
SE	FAM118B	+	chr11:126229224-126229293	NA	0.261	0.003200271846	-0.031	1
SE	TRIM35	-	chr8:27290155-27290178	NA	0.26	0.0007441076698	-0.052	1
SE	ZBTB14	-	chr18:5293971-5294001	NA	0.26	0.002715613574	-0.021	1
SE	PTPRS	-	chr19:5218786-5218798	NA	0.259	0.005967428301	-0.049	1.09E-05
SE	UHRF2	+	chr9:6495584-6497103	NA	0.259	3.07E-05	0.011	0.0004981566378
SE	SMTN	+	chr22:31080449-31080754	NA	0.259	0.002442197371	-0.008	1
SE	PRKD1	-	chr14:29656475-29656499	NA	0.259	1.68E-07	0.131	1.22E-06
SE	RP11-295P9	+	chr10:13652845-13653148	NA	0.259	0.0255521065	0.011	1
SE	RAD51AP1	+	chr12:4545218-4545312	NA	0.258	1.28E-05	0.026	1
SE	NASP	+	chr1:45607320-45608337	NA	0.258	0.01542938513	0.122	0.0001609554291
SE	PCGF3	+	chr4:732399-732498	NA	0.258	0.03122303962	-0.024	1
SE	UBE2Q2P2	+	chr15:82415527-82415675	NA	0.258	0.002987989726	-0.074	1
SE	ISYNA1	-	chr19:18437598-18437760	NA	0.257	0.01175358981	-0.029	1
SE	MTMR4	-	chr17:58514991-58515083	NA	0.257	0.0005045853247	-0.007	1
SE	RIC8B	+	chr12:106874480-106874567	NA	0.257	0.001966429415	-0.019	1
SE	LPHN2	+	chr1:81987288-81987341	NA	0.256	1.66E-05	-0.047	0.2525992523
SE	LRRC23	+	chr12:6909889-6910026	NA	0.254	0.0001253084563	-0.056	1.94E-06
SE	TNRC6A	+	chr16:24793472-24793649	NA	0.254	1.07E-06	0.191	1.41E-06
SE	CENPA	+	chr2:26792501-26792833	NA	0.254	0.0005141992172	-0.009	1
SE	SRRM1	+	chr1:24663182-24663224	NA	0.254	5.46E-06	0.061	0.05116587074
SE	APLP1	+	chr19:35878084-35878108	NA	0.253	4.07E-05	0.02	0
SE	EVI5L	+	chr19:7857091-7857124	NA	0.253	0.009226021974	0.165	1.47E-10
SE	LUZP1	-	chr1:23109584-23109650	NA	0.253	0.017863027	-0.144	1
SE	BTF3L4	+	chr1:52083339-52083541	NA	0.252	2.12E-09	0.007	1
SE	HDAC11	+	chr3:13496735-13496852	NA	0.252	0.006200467481	-0.002	1
SE	TIAL1	-	chr10:119580470-119580846	NA	0.251	0.008171877175	0.012	0.0005019871937
SE	CACNB4	-	chr2:151860710-151860820	NA	0.251	0.005673378401	-0.068	1
SE	IQGAP3	-	chr1:156554234-156554392	NA	0.251	0.001556186387	-0.05	1
SE	ZMYND8	-	chr20:47212641-47212725	NA	0.25	0.0003587716172	-0.105	4.56E-06
SE	MTSS1L	-	chr16:70665465-70665540	NA	0.25	0.001167925376	0.007	1
SE	CHEK2	-	chr22:28696900-28696987	NA	0.25	1.08E-13	-0.001	1
SE	FAM86EP	-	chr4:3954959-3955033	NA	0.25	0.00443138389	-0.024	1
SE	SEMA6C	-	chr1:151138315-151138406	NA	0.248	0.02759566031	-0.044	0.002697885149
SE	ARHGEF40	+	chr14:21075622-21075765	NA	0.248	0.0008843185609	-0.034	0
SE	RBM5	+	chr3:50110378-50110463	NA	0.247	0	-0.036	0
SE	FUK	+	chr16:70469151-70469323	NA	0.247	0.002737544214	-0.038	0.00249957137
SE	TMEM9B	-	chr11:8962091-8962183	NA	0.247	1.02E-08	-0.014	0.01444928176
SE	WHSC1	+	chr4:1942338-1942565	NA	0.246	0.0001438727222	0.062	0.2939917334
SE	CD46	+	chr1:207767778-207767823	NA	0.246	2.53E-05	-0.029	1
SE	GPR155	-	chr2:174482872-174482918	NA	0.246	8.71E-05	0.034	1
SE	XRRA1	-	chr11:74930299-74930372	NA	0.246	0.01359356348	-0.011	1
SE	METTL4	-	chr18:2544652-2544759	NA	0.245	4.20E-07	0.02	1
SE	CDK16	+	chrX:47222260-47222342	NA	0.245	0.01326260223	0.01	1
SE	STAMBP	+	chr2:73829404-73829510	NA	0.245	1.54E-05	0.096	0.00306661764
SE	ZNF83	-	chr19:52616717-52616875	NA	0.245	1.38E-05	0.054	0.9530455421
SE	MYLK	-	chr3:123732895-123733102	NA	0.244	0.0001112824625	0.131	1
SE	TMEM62	+	chr15:43178606-43178711	NA	0.244	3.47E-05	-0.033	1
SE	CNNM3	+	chr2:96831968-96832068	NA	0.242	3.12E-05	0.14	1.16E-12
SE	ZNF615	-	chr19:52007292-52007330	NA	0.242	0.006828710607	-0.026	1
SE	March7	+	chr2:159715680-159715766	NA	0.241	0.01974476361	-0.119	1

SE	DRAM2	-	chr1:111122615-111122643	NA	0.241	0.006855151817	-0.025	1
SE	PPIP5K2	+	chr5:103176865-103176910	NA	0.24	7.34E-12	0	1
SE	FAM189B	-	chr1:155253858-155253978	NA	0.24	0.008470168692	-0.031	1
SE	HYI	-	chr1:43452902-43453004	NA	0.24	0.02445759739	0.001	1
SE	CAMTA2	-	chr17:4982088-4982160	NA	0.239	0.0003906458635	0.005	1
SE	NSRP1	+	chr17:30118079-30118173	NA	0.239	0.002111221232	0.026	1
SE	ARL14EP	+	chr11:30330885-30331374	NA	0.239	4.00E-13	0.032	5.08E-07
SE	ZFYVE19	+	chr15:40809155-40809240	NA	0.239	1.17E-08	-0.016	1
SE	LTB4R2	+	chr14:24310170-24310255	NA	0.239	0.007647461604	-0.148	1
SE	TJP1	-	chr15:29719776-29720016	NA	0.238	2.38E-05	-0.019	1
SE	TTYH3	+	chr7:2647958-2648054	NA	0.238	8.35E-09	0	1
SE	FAM168A	-	chr11:73425021-73425048	NA	0.237	5.74E-10	0.122	1
SE	CBWD5	+	chr9:65732946-65733122	NA	0.237	0.000307827365	-0.009	1
SE	CELF1	-	chr11:47500860-47500932	NA	0.237	0.02326580329	-0.048	1
SE	PLA2G6	-	chr22:38125638-38125691	NA	0.237	0.03472346189	-0.14	9.74E-12
SE	LTBP4	+	chr19:40622949-40623021	NA	0.236	1.52E-05	-0.084	3.01E-05
SE	PTPRS	-	chr19:5216719-5216767	NA	0.236	3.60E-08	0.06	0.3354210696
SE	BAZ2B	-	chr2:159397073-159397100	NA	0.236	0.0006556996521	-0.048	1
SE	PSMG1	-	chr21:39179923-39179986	NA	0.236	2.86E-10	-0.001	1
SE	MPHOSPH9	-	chr12:123226287-123226351	NA	0.235	5.93E-06	0.125	1
SE	SLC26A6	-	chr3:48632244-48632337	NA	0.235	0.01192185245	-0.04	1
SE	ARFIP1	+	chr4:152870752-152870848	NA	0.234	2.20E-11	-0.077	0.1913637849
SE	JKAMP	+	chr14:59486712-59486804	NA	0.233	2.89E-14	0.001	1
SE	ACIN1	-	chr14:23089981-23090101	NA	0.233	0.02847082484	-0.092	0.1548117789
SE	WARS	-	chr14:100375282-100375403	NA	0.232	1.37E-10	-0.081	0.02690079185
SE	HGSNAT	+	chr8:43173712-43173743	NA	0.232	0.002089000179	-0.022	1
SE	TOP1MT	-	chr8:143332486-143332601	NA	0.232	8.45E-05	0.065	0.05902137746
SE	IFT88	+	chr13:20615792-20615879	NA	0.231	0.0002900265419	-0.005	0.004249519972
SE	RTN4	-	chr2:55025085-55027485	NA	0.231	0.0003550262261	0.333	0
SE	SGOL1	-	chr3:20174248-20175055	NA	0.231	0.002075918316	0.004	1
SE	GPR155	-	chr2:174482872-174482918	NA	0.231	0.0001494592795	-0.13	1
SE	FLYWCH1	+	chr16:2913005-2913250	NA	0.23	0.003866750586	-0.029	0.11362415
SE	NEDD1	+	chr12:96907701-96907856	NA	0.23	0.001184309423	-0.05	1
SE	ZNF440	+	chr19:11830273-11830409	NA	0.23	0.00448074171	-0.009	1
SE	WDR45	-	chrX:49077642-49077747	NA	0.23	0.002782540749	-0.023	2.85E-05
SE	LTBP4	+	chr19:40622949-40623021	NA	0.229	4.13E-11	-0.039	0
SE	POLDIP3	-	chr22:42601969-42602056	NA	0.229	0.005634242534	-0.038	0.005666031768
SE	SYTL2	-	chr11:85704857-85705028	NA	0.229	2.15E-05	-0.006	1
SE	THUMPD2	-	chr2:39768900-39769093	NA	0.229	0.0009046763203	0.027	1
SE	FANCD2	+	chr3:10064355-10064429	NA	0.229	6.59E-06	-0.008	1
SE	ZBTB43	+	chr9:126808792-126808915	NA	0.229	0.006518544222	0.001	1
SE	FAM66C	+	chr12:8187731-8188225	NA	0.229	0.03557962837	-0.021	1
SE	RBM23	-	chr14:22908332-22908380	NA	0.228	1.15E-05	-0.041	0.01356018259
SE	SLC29A1	+	chr6:44225972-44226167	NA	0.228	3.37E-06	-0.113	1
SE	KLC1	+	chr14:103692358-103692425	NA	0.228	0.003905473379	-0.031	0.03628582128
SE	RPS6KB2	+	chr11:67432355-67432492	NA	0.228	0.003857761711	0.094	1.96E-10
SE	PUF60	-	chr8:143824312-143824396	NA	0.228	0.008185219315	-0.04	0.0002717723216
SE	DYNC2H1	+	chr11:103287532-103287605	NA	0.228	1.53E-05	-0.004	1
SE	UPRT	+	chrX:75294562-75294620	NA	0.227	0.0002487257324	0.023	1
SE	RHEB	-	chr7:151498088-151498180	NA	0.227	0.001382000474	0	1
SE	ZNF815P	+	chr7:5833526-5833568	NA	0.226	0.016092165	-0.008	1
SE	MAPK8IP3	+	chr16:1758147-1758159	NA	0.225	2.71E-05	0	1
SE	ZNF83	-	chr19:52616717-52616841	NA	0.225	7.13E-05	0.036	1
SE	MIS12	+	chr17:5487556-5487694	NA	0.225	1.66E-10	0.046	1
SE	PSMG4	+	chr6:3264208-3264325	NA	0.225	0	0	1
SE	WNK1	+	chr12:880720-880999	NA	0.224	0.0001029798356	0.018	1
SE	ZFAND1	-	chr8:81717248-81717288	NA	0.224	0.04922594537	0.012	1
SE	KDM4C	+	chr9:7105401-7105500	NA	0.224	0.03074448238	0.042	0.06438158474
SE	LPHN3	+	chr4:61730621-61730636	NA	0.224	0.001796423469	0.049	1
SE	MIOS	+	chr7:7567606-7567688	NA	0.224	0.02561488547	0.079	1
SE	GAS5	-	chr1:173864674-173864704	NA	0.223	0.0005287084667	0.131	1
SE	MRPL22	+	chr5:154950820-154950938	NA	0.222	3.60E-08	-0.07	0
SE	KIF21A	-	chr12:39315931-39315970	NA	0.222	7.86E-05	0.229	1

SE	EIF4A2	+	chr3:186788309-186788420	NA		0.222	0.00365172218	-0.009	1
SE	RPS6KB2	+	chr11:67432352-67432492	NA		0.222	0.00513160654	0.094	1.32E-10
SE	TBL2	-	chr7:73576540-73576701	NA		0.221	0.006525933172	0.014	0.3044475248
SE	EEF2KMT	-	chr16:5097279-5097353	NA		0.221	0.006146579901	0.019	1
SE	CAMK2G	-	chr10:73818208-73818895	NA		0.221	0.01243956146	0.008	1
SE	FLVCR1	+	chr1:212863724-212863869	NA		0.221	2.23E-05	-0.041	1
SE	ADAM22	+	chr7:88186614-88186701	NA		0.22	4.44E-07	0.098	8.84E-06
SE	DNM1	+	chr9:128240986-128241047	NA		0.22	0.04612861764	-0.016	1
SE	SLC29A1	+	chr6:44226060-44226167	NA		0.22	0.003873995416	-0.112	1
SE	TICRR	+	chr15:89592046-89592176	NA		0.22	1.65E-05	-0.005	1
SE	BEND6	+	chr6:57018420-57018557	NA		0.22	0.02050644615	0.002	1
SE	RIF1	+	chr2:151468473-151468551	NA		0.219	0.001720428133	0.044	1
SE	PLK3	+	chr1:44804649-44804779	NA		0.219	0.01310929813	-0.009	1
SE	THRAP3	+	chr1:36225411-36225463	NA		0.218	0.01278202333	-0.001	1
SE	THOC5	-	chr22:29550727-29550843	NA		0.218	2.53E-08	-0.011	1
SE	BRD8	-	chr5:138157641-138157894	NA		0.218	0.001234249565	0.16	5.27E-06
SE	INTS3	+	chr1:153753700-153753818	NA		0.218	0.01176581464	0.095	1
SE	LRRC75A-A	+	chr17:16460892-16461245	NA		0.218	0.04305333938	-0.12	1
SE	RBMS1	-	chr2:160282256-160282304	NA		0.217	0.02751345708	-0.031	1
SE	UPF3A	+	chr13:114286301-114286400	NA		0.217	0.0008678622588	0.02	1
SE	ICA1	-	chr7:8138984-8139047	NA		0.216	5.39E-06	-0.003	1
SE	EPS15	-	chr1:51459040-51459148	NA		0.216	0.04158645599	0.064	1
SE	FXR1	+	chr3:180971074-180971155	NA		0.216	0	0.049	0.0001362988217
SE	SMG7	+	chr1:183547102-183547252	NA		0.216	0.0002682286217	-0.004	1
SE	CEP164	+	chr11:117363428-117363506	NA		0.215	0.01185604305	0.037	1
SE	DCAF10	+	chr9:37857240-37857351	NA		0.214	0.02641179415	-0.03	1
SE	SNAPIN	+	chr1:153659137-153659184	NA		0.214	7.04E-09	-0.048	0
SE	CBWD5	+	chr9:65732992-65733122	NA		0.214	0.0003888102782	-0.009	1
SE	ZNF507	+	chr19:32347244-32347338	NA		0.214	0.004718480332	0.016	1
SE	CTC-444N2[illegible]	-	chr19:57278115-57278182	NA		0.214	0.02156029198	0.021	1
SE	UBE3A	-	chr15:25407066-25407137	NA		0.213	0.0001454345137	0.007	1
SE	LSM14B	+	chr20:62127584-62127701	NA		0.213	0.00694961065	0.129	1.21E-05
SE	SACS	-	chr13:23341444-23341690	NA		0.213	1.17E-10	-0.08	1
SE	MYT1	+	chr20:64223290-64223359	NA		0.213	0.008775297312	0.156	1
SE	DHRS4-AS1	-	chr14:23953773-23954033	NA		0.213	0.004865530267	0.007	1
SE	VPS53	-	chr17:661808-661895	NA		0.212	0.0001833798291	-0.009	1
SE	SREK1	+	chr5:66158808-66158932	NA		0.212	0.01430870942	0.02	1
SE	PCBP4	-	chr3:51961748-51962088	NA		0.211	0.01378706426	-0.014	1
SE	SEC22C	-	chr3:42563764-42563980	NA		0.211	0	-0.011	0.01905582287
SE	FLT3LG	+	chr19:49480297-49480476	NA		0.21	0.009473724616	0.004	1
SE	ECHDC2	-	chr1:52905033-52906611	NA		0.209	0.0008643964118	0.013	1
SE	NUMB	-	chr14:73316389-73316422	NA		0.209	1.27E-06	0.032	1
SE	DEPDC5	+	chr22:31861367-31861433	NA		0.208	0.0009982071298	0.311	0
SE	FAM219B	-	chr15:74904981-74905059	NA		0.208	7.91E-05	-0.009	1
SE	BCL2L13	+	chr22:17696140-17696210	NA		0.207	4.22E-06	-0.004	1
SE	PHKB	+	chr16:47463881-47463993	NA		0.207	0.002682815417	0.065	1
SE	POLG	-	chr15:89332217-89332374	NA		0.207	0.002540358248	-0.007	1
SE	NPRL3	-	chr16:117300-117375	NA		0.206	0.005136856933	-0.035	0.09544121561
SE	RPL13A	+	chr19:49489849-49490090	NA		0.206	2.84E-05	0	1
SE	H2AFY	-	chr5:135350822-135350913	NA		0.205	0	-0.006	0.08520446241
SE	WARS	-	chr14:100375282-100375350	NA		0.205	7.15E-10	-0.097	0.011127145
SE	MRPS28	-	chr8:80028694-80028796	NA		0.205	0.01136970053	0.061	1
SE	IL17RC	+	chr3:9930399-9930459	NA		0.205	0.003957427051	-0.025	0.07451454225
SE	PRKAG1	-	chr12:49005742-49005881	NA		0.205	2.77E-08	-0.059	1.54E-07
SE	LPHN1	-	chr19:14167015-14167030	NA		0.204	0.002071693895	0.138	0.0002093392385
SE	PHKB	+	chr16:47463898-47463993	NA		0.204	0.0007058046358	0.047	1
SE	DGUOK	+	chr2:73957124-73957240	NA		0.204	0.002786142805	0.146	0
SE	CCDC15	+	chr11:125003866-125003959	NA		0.204	0.0007539629551	-0.004	1
SE	SUN1	+	chr7:849519-849603	NA		0.204	0.01249196002	-0.094	1
SE	PCGF3	+	chr4:732443-732498	NA		0.204	0.04316493501	-0.023	1
SE	PGAP1	-	chr2:196892345-196892401	NA		0.204	0.02951502951	-0.002	1
SE	AGTPBP1	-	chr9:85660935-85660973	NA		0.203	0.002196361706	-0.006	1
SE	TANC2	+	chr17:63151269-63151380	NA		0.203	0.0003699647386	0.104	1

SE	FAM107B	-	chr10:14553321-14553387	NA	0.202	0.01165258035	-0.092	1
SE	PPP6R2	+	chr22:50436987-50437068	NA	0.202	0.0006841654985	-0.039	0.8502105905
SE	CMC2	-	chr16:81001247-81001334	NA	0.202	0	0.046	0.05756834268
SE	NEDD1	+	chr12:96924832-96924942	NA	0.202	0.001854735332	0.021	1
SE	SVIL	-	chr10:29526960-29527056	NA	0.202	7.64E-06	0.097	1
SE	SVIL	-	chr10:29535988-29536069	NA	0.202	0.04292658489	0.007	1
SE	METTL4	-	chr18:2544194-2544286	NA	0.201	1.41E-06	0.014	1
SE	SLC29A1	+	chr6:44226060-44226167	NA	0.201	0.01275158129	-0.013	1
SE	SENP5	+	chr3:196927795-196927879	NA	0.201	0.003991670538	-0.019	0.1202433306
SE	SH3GLB2	-	chr9:129010669-129010693	NA	0.201	6.34E-07	0	1
SE	FEZ2	-	chr2:36560784-36560865	NA	0.201	0.0002096174479	0.001	1
SE	THAP9-AS1	-	chr4:82897988-82898062	NA	0.201	0.03668129886	0.036	1
SE	WDR54	+	chr2:74422119-74422375	NA	0.2	0.04089304347	0.058	0.04076956037
SE	RBM27	+	chr5:146251710-146251875	NA	0.2	0.003498229501	0.002	1
SE	POLG	-	chr15:89317938-89318037	NA	0.2	0	0.047	5.09E-06
SE	ATP9B	+	chr18:79375393-79375426	NA	0.2	0.0001362768567	0.16	8.67E-11
SE	TMEM45A	+	chr3:100519461-100519608	NA	0.2	0.004619682531	-0.007	1
SE	MYO19	-	chr17:36501851-36501961	NA	0.2	5.91E-07	0.219	2.54E-11
SE	HMMR	+	chr5:163467703-163467748	NA	0.199	1.66E-09	-0.062	1
SE	SH3D19	-	chr4:151144219-151144288	NA	0.199	0.0001322429857	0.029	1
SE	RWDD1	+	chr6:116574057-116574171	NA	0.198	4.92E-07	-0.009	1
SE	CHEK1	+	chr11:125637448-125637544	NA	0.198	8.61E-13	-0.006	1
SE	LIN9	-	chr1:226295841-226295946	NA	0.198	0.001604315093	-0.081	1
SE	RP5-1021I2	+	chr14:73922065-73922206	NA	0.198	0.001649349498	0.036	0.02743546281
SE	CTD-2583A	+	chr19:57820493-57820549	NA	0.198	0.004759894371	-0.022	1
SE	MEF2BNB-M	-	chr19:19180685-19180761	NA	0.197	0.001347769055	-0.012	0.1330025421
SE	CCDC91	+	chr12:28225794-28225861	NA	0.197	0.02611709641	0.029	1
SE	AGTPBP1	-	chr9:85678334-85678398	NA	0.197	3.06E-06	-0.026	0.07907275112
SE	GOLGA2	-	chr9:128272784-128272865	NA	0.197	0.04068278322	-0.099	0.002359423705
SE	SHMT1	-	chr17:18335558-18335675	NA	0.197	0.0005294509158	-0.031	0.4412670492
SE	GGCX	-	chr2:85552415-85552567	NA	0.196	1.86E-06	-0.03	3.43E-05
SE	ZNF384	-	chr12:6678662-6678710	NA	0.196	0.0003506228985	-0.062	1.87E-05
SE	CSNK1D	-	chr17:82249697-82250179	NA	0.196	0	0.014	0
SE	MTX1	+	chr1:155212126-155212219	NA	0.196	0	-0.035	0
SE	HSPA14	+	chr10:14851218-14851323	NA	0.196	2.58E-09	-0.005	1
SE	EIF3E	-	chr8:108242111-108242346	NA	0.195	0.0001580415189	-0.046	1
SE	PARP6	-	chr15:72250844-72250957	NA	0.195	0.002626254247	0.04	0.6378306673
SE	EIF4A2	+	chr3:186788309-186788416	NA	0.195	0.0009928791088	-0.01	0.5902580187
SE	AMACR	-	chr5:34004573-34004734	NA	0.195	0.0008221747762	-0.039	1
SE	CDKL3	-	chr5:134359896-134360091	NA	0.194	0.03199306257	-0.036	1
SE	ACIN1	-	chr14:23071124-23071186	NA	0.194	0.04533534914	-0.069	0.04489001631
SE	COA1	-	chr7:43656032-43656153	NA	0.194	8.02E-05	-0.021	1
SE	ATAD5	+	chr17:30879422-30879487	NA	0.194	1.38E-05	0.002	1
SE	SLC35A3	+	chr1:99999260-99999415	NA	0.193	0.02279519203	-0.025	1
SE	TBC1D8B	+	chrX:106839307-106839457	NA	0.193	0.0002577584141	-0.047	1
SE	INIP	-	chr9:112689526-112689617	NA	0.193	0.0008535483712	-0.011	1
SE	NUDT2	+	chr9:34336228-34336341	NA	0.193	0	-0.036	0.9487342534
SE	TARBP1	-	chr1:234400852-234400948	NA	0.192	0.03775197242	0.105	0.0002920998039
SE	RECQL5	-	chr17:75650516-75650718	NA	0.192	4.71E-05	-0.019	1
SE	BAI2	-	chr1:31736572-31736723	NA	0.192	0.005745634186	-0.003	1
SE	MDC1	-	chr6:30707565-30708357	NA	0.192	0.0001681652016	0.026	1
SE	HDHD2	-	chr18:47137234-47137338	NA	0.192	0.0001146885166	0.037	1.49E-13
SE	MFF	+	chr2:227342744-227342819	NA	0.192	0.000146113945	-0.006	1
SE	MYO9A	-	chr15:71902940-71903063	NA	0.191	0.0001969559049	-0.004	1
SE	SMARCC2	-	chr12:56172936-56173029	NA	0.191	2.01E-05	0.102	7.48E-05
SE	FAM219B	-	chr15:74904981-74905039	NA	0.191	0.0003588759518	-0.005	1
SE	GPBP1	+	chr5:57237112-57237172	NA	0.19	1.14E-08	0.025	1
SE	UBE2W	-	chr8:73870268-73870301	NA	0.19	4.28E-08	0.026	1
SE	P4HA1	-	chr10:73044980-73045051	NA	0.19	3.13E-05	-0.015	0.02562999478
SE	PDCD2L	+	chr19:34404648-34404990	NA	0.19	0.01204751673	-0.054	0
SE	AGAP3	+	chr7:151120519-151120745	NA	0.19	0.01944727044	0.083	0.0009914770566
SE	NSUN2	-	chr5:6631872-6631977	NA	0.189	1.23E-10	0.006	1
SE	SEC61A2	+	chr10:12155777-12155931	NA	0.189	0.02675707532	0.047	9.76E-05

SE	NBN	-	chr8:89981952-89982002	NA	0.189	2.01E-05	-0.034	1
SE	DMPK	-	chr19:45780322-45780381	NA	0.189	0.001052296447	0.015	1
SE	CLTC	+	chr17:59687000-59687021	NA	0.189	0	0.154	0
SE	DCAF8	-	chr1:160261284-160261358	NA	0.188	9.23E-05	0.008	1
SE	GUSB	-	chr7:65974918-65975071	NA	0.188	1.27E-05	-0.042	0.06188305526
SE	PAAF1	+	chr11:73887030-73887099	NA	0.188	4.40E-11	0.124	3.37E-05
SE	SLC25A42	+	chr19:19104912-19104938	NA	0.188	0.00274477048	-0.001	1
SE	CCDC125	-	chr5:69294792-69294900	NA	0.188	0.004507877005	0.003	1
SE	NBPF1	-	chr1:16594930-16595009	NA	0.188	0.02406623634	-0.028	1
SE	SPAG9	-	chr17:50975862-50975901	NA	0.187	8.78E-05	0	1
SE	TTC23	-	chr15:99245388-99245510	NA	0.187	0.03499961163	0.008	1
SE	GUK1	+	chr1:228148370-228148539	NA	0.187	1.39E-05	-0.011	1
SE	AP2A2	+	chr11:1000431-1000598	NA	0.186	0.02196082209	-0.027	2.03E-07
SE	SPTAN1	+	chr9:128627924-128627942	NA	0.186	0	0.018	0
SE	NOL8	-	chr9:92311145-92311259	NA	0.186	1.93E-09	-0.008	1
SE	UFD1L	-	chr22:19475495-19475602	NA	0.185	0.0002027578632	0.059	7.80E-13
SE	ZFYVE21	+	chr14:103729792-103729846	NA	0.185	0.001742917457	0.166	0
SE	ZC2HC1A	+	chr8:78711989-78712106	NA	0.185	0.0001857822036	-0.015	1
SE	NADK2	-	chr5:36208624-36208690	NA	0.185	1.16E-07	0.002	1
SE	ARHGAP23	+	chr17:38498413-38498510	NA	0.185	0.03589513837	0.007	1
SE	MTMR11	-	chr1:149931259-149931426	NA	0.184	0.0223742272	-0.001	1
SE	FAT1	-	chr4:186590367-186590403	NA	0.184	8.32E-08	-0.05	0.005454289519
SE	MRPL55	-	chr1:228108436-228108474	NA	0.184	0.03254129005	-0.044	0.03034242192
SE	FRS2	+	chr12:69486201-69486297	NA	0.184	0.005841165759	-0.025	1
SE	C17orf62	-	chr17:82447579-82447621	NA	0.184	0.006498475566	-0.028	2.15E-11
SE	HAGH	-	chr16:1819114-1819223	NA	0.183	2.21E-10	0.007	1
SE	VTI1B	-	chr14:67663028-67663190	NA	0.183	1.67E-10	-0.004	0.5757117115
SE	SAP30BP	+	chr17:75671815-75671863	NA	0.183	0	-0.003	0.05352239286
SE	SDCCAG3	-	chr9:136408998-136409067	NA	0.183	0.0474914878	0.018	1
SE	CAMK2G	-	chr10:73819531-73819645	NA	0.182	2.25E-06	0.011	1
SE	KMT2D	-	chr12:49024052-49024100	NA	0.182	5.73E-08	-0.019	0.05994834723
SE	ADCY6	-	chr12:48773939-48774098	NA	0.182	0.001421550924	-0.002	1
SE	INTS6	-	chr13:51423029-51423090	NA	0.181	0.04127709796	0.066	1
SE	GTPBP10	+	chr7:90355085-90355230	NA	0.181	9.97E-05	-0.019	1
SE	SCAMP3	-	chr1:155261656-155261734	NA	0.181	2.09E-06	-0.03	0.002395562845
SE	OSBPL3	-	chr7:24863199-24863292	NA	0.18	0.0004067926128	0.012	1
SE	DDHD1	-	chr14:53093315-53093444	NA	0.18	0.01034250695	0.017	1
SE	PARP6	-	chr15:72250844-72250954	NA	0.18	0.000680065943	0.036	0.6407210674
SE	DEF8	+	chr16:89952561-89952682	NA	0.18	0.00897994775	0.002	1
SE	PLOD2	-	chr3:146077861-146077924	NA	0.18	0.007990153349	0.008	1
SE	RHOC	-	chr1:112705099-112705201	NA	0.18	0.002921158496	-0.027	0.5802092128
SE	AGAP1	+	chr2:235750353-235750488	NA	0.18	3.67E-06	-0.012	1
SE	FAM189B	-	chr1:155250275-155250929	NA	0.18	0.001414702212	-0.003	1
SE	NLE1	-	chr17:35134942-35135059	NA	0.179	0.002462945607	-0.009	1
SE	WHSC1	+	chr4:1942338-1942432	NA	0.179	7.24E-05	0.058	0.409967346
SE	VEGFA	+	chr6:43781955-43782052	NA	0.179	0.000117787783	0.089	1.24E-07
SE	CCDC18	+	chr1:93183359-93183495	NA	0.179	0.01918166239	0.017	1
SE	ERMARD	+	chr6:169753286-169753372	NA	0.179	0.03259045842	0.024	1
SE	IL11RA	+	chr9:34660803-34660936	NA	0.179	0.00233625466	-0.016	1
SE	THAP9	+	chr4:82905860-82905926	NA	0.179	0.003447264686	0.044	1
SE	MBTD1	-	chr17:51202021-51202077	NA	0.178	0.008345359488	0.03	1
SE	IFNGR2	+	chr21:33410840-33410892	NA	0.178	0.000258114977	0.001	1
SE	TMEM230	-	chr20:5111499-5111605	NA	0.177	0.0001350148226	0.016	1
SE	CRBN	-	chr3:3153959-3154075	NA	0.177	2.26E-06	0.003	1
SE	LMBR1L	-	chr12:49106653-49106746	NA	0.177	4.77E-07	0.001	1
SE	DEF8	+	chr16:89952557-89952682	NA	0.177	0.009482340098	0.001	1
SE	FAM131A	+	chr3:184337310-184337371	NA	0.177	0.001823831485	-0.016	1
SE	CEBPZOS	+	chr2:37198493-37198672	NA	0.177	0.02655662668	0.047	1
SE	FHL1	+	chrX:136209242-136209442	NA	0.176	0	0.011	0.7426068118
SE	ZCCHC8	-	chr12:122490461-122490567	NA	0.176	1.79E-06	0.002	1
SE	CHD9	+	chr16:53319812-53319951	NA	0.176	0.01436473974	0.001	0.1433774672
SE	SSH1	-	chr12:108802321-108802368	NA	0.175	0.0250286001	0.026	1
SE	HARS2	+	chr5:140696102-140696201	NA	0.175	1.39E-10	-0.023	1

SE	PFDN5	+	chr12:53296429-53296551	NA	0.175	7.32E-07	0.05	2.75E-11
SE	VPS13B	+	chr8:99507136-99507203	NA	0.175	0.01780311254	-0.044	1
SE	LMBR1L	-	chr12:49106544-49106746	NA	0.175	0.02376495083	-0.001	1
SE	C16orf13	-	chr16:634888-635063	NA	0.174	0.001291710649	0.003	1
SE	TBC1D32	-	chr6:121255327-121255410	NA	0.174	0.0009385159804	0.026	1
SE	IFNGR2	+	chr21:33410835-33410892	NA	0.174	0.0005294509158	-0.002	1
SE	SLC4A1AP	+	chr2:27688699-27688767	NA	0.174	0.01068668249	-0.002	1
SE	BPTF	+	chr17:67875555-67875744	NA	0.174	2.33E-06	-0.001	1
SE	ARNTL2	+	chr12:27370128-27370230	NA	0.173	0.0002979307257	-0.057	1
SE	C17orf53	+	chr17:44150960-44151044	NA	0.173	0.03360945903	-0.057	1
SE	TSEN2	+	chr3:12516610-12516661	NA	0.173	0.007851399504	-0.013	1
SE	ZNF326	+	chr1:90007611-90007750	NA	0.173	5.06E-06	0.006	1
SE	PAAF1	+	chr11:73886928-73887099	NA	0.173	2.20E-10	0.127	8.56E-07
SE	SNHG14	+	chr15:25111764-25111894	NA	0.173	0.02967749663	0.001	1
SE	SDCCAG8	+	chr1:243293398-243293494	NA	0.172	0.007959369707	-0.02	1
SE	MTMR2	-	chr11:95887611-95887684	NA	0.172	6.89E-05	0.007	1
SE	ABCD4	-	chr14:74288206-74288259	NA	0.172	0.01087266728	-0.013	0.08165659186
SE	PDLIM5	+	chr4:94584985-94585003	NA	0.172	2.58E-07	0.076	0.0002059159903
SE	GAPVD1	+	chr9:125342218-125342299	NA	0.172	1.64E-10	-0.019	0.02188844353
SE	STRA13	-	chr17:82019509-82019694	NA	0.172	0.002280843257	-0.006	1
SE	AK2	-	chr1:33031543-33031661	NA	0.171	0	0.032	5.97E-11
SE	MADD	+	chr11:47326737-47326807	NA	0.171	2.26E-05	-0.034	0.2160637101
SE	CCHCR1	-	chr6:31157357-31157500	NA	0.171	0.01760896452	0.057	1
SE	RRP12	-	chr10:97424800-97424893	NA	0.17	0.02624280943	-0.113	1
SE	PHF14	+	chr7:11061790-11061841	NA	0.17	0	-0.002	1
SE	CLK4	-	chr5:178619837-178619896	NA	0.17	0.0004320131833	-0.003	1
SE	TIAL1	-	chr10:119580470-119580538	NA	0.17	0.02493720771	0.022	2.51E-05
SE	C20orf194	-	chr20:3343675-3343731	NA	0.169	0.0087482388	-0.019	1
SE	PCBP4	-	chr3:51962809-51962892	NA	0.169	0.01989487174	0.022	1
SE	ATP6V0B	+	chr1:43975664-43975848	NA	0.169	0.01015259463	-0.014	1
SE	CITED1	-	chrX:72305301-72305422	NA	0.169	0.00029790281	-0.027	1
SE	ARHGEF39	-	chr9:35662945-35663074	NA	0.169	0.001596040472	0.061	1
SE	ARHGEF40	+	chr14:21075644-21075765	NA	0.169	0.000780430881	-0.022	1
SE	PER3	+	chr1:7787197-7787246	NA	0.168	0.00357297092	0.016	1
SE	DGUOK	+	chr2:73950600-73950732	NA	0.168	0.0001134949235	0.148	0
SE	CDCA7	+	chr2:173359254-173359491	NA	0.168	1.93E-05	0.121	1.81E-06
SE	RHOT1	+	chr17:32172936-32173060	NA	0.167	0.02106816711	-0.006	1
SE	SYVN1	-	chr11:65132247-65132308	NA	0.167	0.009427282182	-0.005	1
SE	OMA1	-	chr1:58530600-58530729	NA	0.167	3.05E-06	-0.014	1
SE	PTK2	-	chr8:140803542-140803650	NA	0.167	0	-0.004	1
SE	MPHOSPH9	-	chr12:123162114-123162218	NA	0.166	1.93E-05	-0.001	1
SE	MAEA	+	chr4:1322380-1322503	NA	0.166	6.91E-05	-0.034	1
SE	VRK3	-	chr19:50009235-50009385	NA	0.166	0.003445306513	-0.041	3.53E-10
SE	COA1	-	chr7:43656032-43656153	NA	0.166	6.41E-13	0.001	1
SE	HMGCS1	-	chr5:43307765-43307824	NA	0.166	5.14E-13	0.015	1
SE	SLC25A36	+	chr3:140966999-140967036	NA	0.166	0.01612752722	-0.01	1
SE	C11orf1	+	chr11:111883135-111883202	NA	0.166	0.0003246175549	0.001	0.8240834163
SE	RPL13A	+	chr19:49489849-49489912	NA	0.166	9.38E-05	0	1
SE	FNBP4	-	chr11:47725736-47725836	NA	0.165	9.68E-05	0.032	1
SE	PKP4	+	chr2:158676738-158676867	NA	0.165	3.10E-06	0.163	5.75E-13
SE	MFF	+	chr2:227352513-227352573	NA	0.165	1.13E-05	0.006	0.2366737735
SE	ZNF440	+	chr19:11830282-11830409	NA	0.165	0.02119091855	0	1
SE	CCNE1	+	chr19:29821995-29822130	NA	0.164	1.69E-05	-0.021	1
SE	FTSJ2	-	chr7:2238014-2238937	NA	0.164	0.008753276415	0.012	1
SE	SIN3B	+	chr19:16862883-16862979	NA	0.164	0.008586619979	0.047	0.02216388922
SE	SUN1	+	chr7:832504-832601	NA	0.164	4.30E-07	-0.017	0.01627928816
SE	R3HDM4	-	chr19:901419-901546	NA	0.164	0.009304957146	0.002	1
SE	AP2B1	+	chr17:35670856-35670898	NA	0.163	8.61E-05	0.07	0.0001450373156
SE	GTPBP10	+	chr7:90354457-90354549	NA	0.163	0.001003586949	-0.022	1
SE	PRPF4B	+	chr6:4062556-4062760	NA	0.163	0.0001935444919	0.029	1
SE	CEP78	+	chr9:78246668-78246782	NA	0.163	1.69E-10	-0.012	1
SE	SLC7A11	-	chr4:138223198-138223324	NA	0.163	0.0001997450176	0.005	1
SE	RNMT	+	chr18:13759941-13760208	NA	0.162	0.0004093805217	-0.005	1

SE	ARHGEF7	+	chr13:111288353-111288443	NA	0.162	8.37E-06	-0.007	0.1515690427
SE	PPP1R12C	-	chr19:55096261-55096335	NA	0.162	3.49E-05	-0.008	1
SE	RIN2	+	chr20:19964951-19965024	NA	0.162	0.001397451056	-0.028	1
SE	EMC10	+	chr19:50481860-50481947	NA	0.162	0	-0.031	5.89E-05
SE	ZNF444	+	chr19:56142275-56142386	NA	0.162	0.02259035035	-0.018	1
SE	ARRB1	-	chr11:75271700-75271724	NA	0.161	0.005673378401	0.123	0.0005740650471
SE	SMARCAL1	+	chr2:216438419-216438485	NA	0.161	0.0002091071405	-0.004	1
SE	ETNK2	-	chr1:204134514-204134694	NA	0.161	0.03472346189	-0.015	1
SE	TBC1D32	-	chr6:121299445-121299505	NA	0.161	0.0005716012452	-0.032	1
SE	SNHG11	+	chr20:38447929-38448093	NA	0.161	0.02738999534	0.021	1
SE	CCDC92	-	chr12:123944271-123944364	NA	0.16	0.008185219315	-0.015	1
SE	PAK4	+	chr19:39168229-39168302	NA	0.16	0.0002900265419	-0.012	1
SE	SETD5	+	chr3:9429826-9430051	NA	0.16	0.0119410651	0.067	0.06221824574
SE	PXMP2	+	chr12:132695883-132696046	NA	0.16	0.03033077178	-0.005	0.4102931695
SE	RP11-319G	+	chr3:139419556-139419680	NA	0.16	0.0005716012452	0.038	1
SE	ACO1	+	chr9:32386370-32386438	NA	0.159	9.98E-07	-0.029	0.1239584912
SE	RMND1	-	chr6:151422540-151422605	NA	0.159	5.32E-05	-0.006	1
SE	TAPT1	-	chr4:16170652-16170729	NA	0.159	0.01024382028	-0.007	1
SE	NQO2	+	chr6:3015529-3015643	NA	0.158	0.01432424694	-0.05	1.17E-06
SE	TBC1D9B	-	chr5:179867444-179867495	NA	0.158	0	0.124	0
SE	GABBR1	-	chr6:29627485-29627646	NA	0.158	0.0415224856	0.002	1
SE	PPP2R3C	-	chr14:35109818-35109847	NA	0.157	0.01371870462	-0.007	1
SE	RSRC2	-	chr12:122520503-122520581	NA	0.157	0.000549099964	-0.043	1
SE	TOM1L1	+	chr17:54905488-54905567	NA	0.157	8.32E-05	0.03	1
SE	LSM14B	+	chr20:62127584-62127701	NA	0.157	0.008345604047	-0.009	1
SE	HIVEP2	-	chr6:142769551-142775125	NA	0.156	0.0007818481423	-0.013	1
SE	PIBF1	+	chr13:72783422-72783721	NA	0.156	9.12E-07	-0.027	1
SE	HNRNPL	-	chr19:38841612-38841682	NA	0.156	0.03764037285	0.002	1
SE	MRPL18	+	chr6:159790667-159790787	NA	0.156	7.43E-06	0.015	0.02825197921
SE	PPOX	+	chr1:161167370-161167486	NA	0.156	0.04131087203	0	1
SE	IMMP1L	-	chr11:31456259-31456386	NA	0.156	0.03497307275	0.01	1
SE	SH3YL1	-	chr2:260084-260702	NA	0.155	0.0307508689	-0.021	1
SE	FERMT2	-	chr14:52861013-52861034	NA	0.155	0	0.071	2.63E-07
SE	ZFAND6	+	chr15:80073265-80073330	NA	0.155	0.0006650621728	0.008	0.3473770622
SE	GPATCH2	-	chr1:217495014-217495075	NA	0.155	0.01558075653	-0.004	1
SE	POLA1	+	chrX:24725980-24726055	NA	0.155	6.82E-06	-0.007	1
SE	RALGPS1	+	chr9:126977694-126977745	NA	0.155	0.00469384981	-0.008	1
SE	UGP2	+	chr2:63842538-63842599	NA	0.155	1.39E-05	0.078	0.002278911379
SE	UPP1	+	chr7:48103296-48103411	NA	0.155	0.001796423469	-0.003	1
SE	PORCN	+	chrX:48512834-48512852	NA	0.154	0.04729117939	0.075	0.0009153999046
SE	SSX2IP	-	chr1:84671188-84671308	NA	0.154	0.02654969046	0.046	1
SE	RBM39	-	chr20:35740524-35740597	NA	0.154	0.003153225159	0.01	1
SE	DCTD	-	chr4:182916418-182916539	NA	0.153	0.0464612947	0.01	1
SE	IFT88	+	chr13:20631015-20631102	NA	0.152	0.002607315509	0.001	1
SE	NQO2	+	chr6:3002049-3002286	NA	0.152	0.01579734626	-0.018	1
SE	TMEM87B	+	chr2:112074911-112074962	NA	0.152	0.004874200486	-0.014	1
SE	MTA1	+	chr14:105445666-105445852	NA	0.152	9.06E-08	0.003	1
SE	FAM134C	-	chr17:42587833-42587864	NA	0.151	0.002611775813	0.03	1
SE	SNHG6	-	chr8:66922613-66922725	NA	0.151	0	0.023	0
SE	VPRBP	-	chr3:51466805-51466876	NA	0.15	0.03019391919	0.006	1
SE	BPTF	+	chr17:67886127-67886316	NA	0.15	0.0001037401653	0.011	1
SE	FAM192A	-	chr16:57178501-57178852	NA	0.15	0.01213319028	-0.007	1
SE	TARBP1	-	chr1:234427316-234427388	NA	0.149	2.67E-05	0.034	0.01321933004
SE	DOCK9	-	chr13:98825871-98825940	NA	0.149	0.04570926204	-0.013	1
SE	EFCAB14	-	chr1:46688352-46688544	NA	0.149	0.01675843214	0.07	0.00251862826
SE	JMJD1C	-	chr10:63215552-63215617	NA	0.149	0.0001826002476	-0.01	1
SE	WDR27	-	chr6:169664165-169664286	NA	0.149	0.02319335102	-0.024	1
SE	DNM3	+	chr1:172093695-172093725	NA	0.149	0.001174848614	-0.025	1
SE	WAC-AS1	-	chr10:28525947-28526008	NA	0.149	0.001169338867	0.026	1
SE	ZNF561-AS	+	chr19:9627715-9627806	NA	0.149	0.02302160623	0.061	0.6809108068
SE	BBX	+	chr3:107789786-107789876	NA	0.148	0.03881092694	-0.046	1
SE	SLC23A2	-	chr20:4885820-4885909	NA	0.147	0.01214445282	-0.012	0.02707584673
SE	CCAR2	+	chr8:22613016-22613136	NA	0.147	8.25E-14	-0.001	1

SE	PSMG4	+	chr6:3264173-3264325	NA	0.147	2.71E-09	0.002	1
SE	NBPF1	-	chr1:16594930-16595013	NA	0.147	0.04422144556	-0.017	1
SE	ZDHHC6	-	chr10:112440533-112440695	NA	0.146	2.19E-06	-0.016	1
SE	PEX3	+	chr6:143472159-143472328	NA	0.146	1.34E-05	-0.011	1
SE	POLB	+	chr8:42352518-42352568	NA	0.146	0.0001215527779	-0.026	5.91E-10
SE	COL4A3BP	-	chr5:75399309-75399387	NA	0.146	2.38E-05	0.121	3.64E-06
SE	NGLY1	-	chr3:25732318-25732483	NA	0.146	2.13E-09	0.044	4.43E-13
SE	ACSS1	-	chr20:25015137-25015230	NA	0.146	0.02664983959	0.001	1
SE	ALG13	+	chrX:111721602-111721711	NA	0.145	6.15E-05	-0.015	1
SE	BCL2L12	+	chr19:49667021-49667161	NA	0.145	0.02383511682	-0.028	1
SE	MFF	+	chr2:227352513-227352573	NA	0.145	5.60E-06	0.005	1
SE	N4BP2L2	-	chr13:32538617-32538684	NA	0.145	3.80E-06	0.009	1
SE	TNFRSF12A	+	chr16:3021188-3021319	NA	0.144	0.001101883147	0.002	1
SE	TFIP11	-	chr22:26511118-26511195	NA	0.144	0.04611867135	0.012	1
SE	LPHN2	+	chr1:81987259-81987341	NA	0.144	0.0439700115	-0.032	0.5058044519
SE	KTN1	+	chr14:55673171-55673255	NA	0.144	0.0006166132953	-0.105	0
SE	MLLT10	+	chr10:21557620-21557824	NA	0.143	0.00041663617	0.095	1
SE	SLC35C2	-	chr20:46355801-46355874	NA	0.143	6.22E-08	-0.003	1
SE	NENF	+	chr1:212442545-212442625	NA	0.143	9.57E-05	-0.028	0.0001670778283
SE	SLC35B3	-	chr6:8419579-8419677	NA	0.143	0.001346225949	-0.018	1
SE	ARPP19	-	chr15:52568847-52568900	NA	0.143	0.0007495632856	-0.003	1
SE	SSBP4	+	chr19:18425908-18426083	NA	0.143	0.01287323078	0.001	1
SE	TMBIM6	+	chr12:49742437-49742607	NA	0.143	0.009482340098	0.01	1
SE	TSC1	-	chr9:132921362-132921436	NA	0.143	0.001322173813	-0.016	1
SE	FAM21C	+	chr10:45751481-45751553	NA	0.143	1.48E-05	0.014	1
SE	ZNF195	-	chr11:3361975-3362032	NA	0.142	0.01403856436	0.037	1
SE	ZNF195	-	chr11:3362545-3362602	NA	0.142	0.02225064706	0.03	1
SE	ZBTB25	-	chr14:64487113-64487196	NA	0.142	0.007295871206	-0.04	1
SE	UBE2D3	-	chr4:102825238-102825317	NA	0.142	0.02414968727	-0.02	1
SE	PRDM5	-	chr4:120754552-120754638	NA	0.142	0.03926733927	-0.01	1
SE	MELK	+	chr9:36594627-36594771	NA	0.142	0.008988988263	-0.04	1
SE	FAM21A	+	chr10:50092161-50092233	NA	0.141	0.002623125021	-0.012	0.09679735232
SE	MAPK3	-	chr16:30117136-30117285	NA	0.141	1.99E-10	0.003	0.5815311423
SE	SNRNP70	+	chr19:49102113-49103587	NA	0.141	0	0.033	0.8188259869
SE	FAM120B	+	chr6:170358225-170358318	NA	0.141	0.003756883991	-0.009	1
SE	RCL1	+	chr9:4833153-4833228	NA	0.141	8.10E-07	0.005	1
SE	RECQL	-	chr12:21501169-21501215	NA	0.14	0.03400412644	-0.036	1
SE	TARS2	+	chr1:150504631-150504733	NA	0.14	0.0001016278257	-0.038	3.19E-11
SE	CSNK1G3	+	chr5:123595038-123595134	NA	0.14	0.0001322429857	0.122	2.21E-11
SE	EPB41	+	chr1:29060421-29060484	NA	0.14	0.006560213909	0.007	1
SE	GABBR1	-	chr6:29609228-29609379	NA	0.14	4.05E-08	0.21	2.71E-10
SE	IFT88	+	chr13:20589810-20589867	NA	0.139	0.02953166679	-0.07	0.1152574686
SE	RCBTB2	-	chr13:48524658-48524757	NA	0.139	0.007914741791	0.008	1
SE	CPEB2	+	chr4:15040463-15040487	NA	0.139	0.002054465018	0.148	0.01776847332
SE	TLK2	+	chr17:62523133-62523177	NA	0.139	0.01462858414	-0.011	1
SE	EPB41	+	chr1:29058810-29058852	NA	0.139	0.003539628202	0.013	1
SE	SLC26A6	-	chr3:48632244-48632396	NA	0.139	0.004427904581	-0.034	1
SE	MYCBP2	-	chr13:77103208-77103310	NA	0.138	0.007740262278	0.018	1
SE	ARHGEF10	+	chr8:1926376-1926463	NA	0.138	1.97E-05	-0.012	1
SE	CRYZ	-	chr1:74707102-74707204	NA	0.138	2.18E-05	-0.015	0.02631510705
SE	STX16	+	chr20:58659622-58659634	NA	0.138	0.0008643964118	0.102	0.0003486423568
SE	CHN1	-	chr2:174945169-174945261	NA	0.138	0.009304957146	0.005	4.23E-06
SE	ARFGAP2	-	chr11:47172280-47172333	NA	0.138	0.00357102587	-0.006	1
SE	GPX4	+	chr19:1106180-1106266	NA	0.138	8.15E-08	-0.022	3.15E-14
SE	CTNND1	+	chr11:57794009-57794081	NA	0.138	0.04953027008	0.01	1
SE	PVT1	+	chr8:128070159-128070272	NA	0.138	0.003876184563	-0.004	1
SE	CDKL3	-	chr5:134319357-134319497	NA	0.137	0.03403990512	-0.018	0.7335603905
SE	DTWD1	+	chr15:49631000-49631058	NA	0.137	6.02E-06	-0.013	0.7843853241
SE	CHAF1A	+	chr19:4418019-4418076	NA	0.137	3.24E-08	-0.023	1
SE	EXOSC10	-	chr1:11080498-11080514	NA	0.137	0.01358618517	-0.045	7.07E-09
SE	SEPT10	-	chr2:109545384-109545542	NA	0.137	9.50E-12	0.069	4.54E-07
SE	HMGN5	-	chrX:81197705-81197778	NA	0.137	0.02630321839	0.001	1
SE	PPM1A	+	chr14:60250379-60250481	NA	0.136	0.002092126104	0.003	1

SE	PDCD10	-	chr3:167725400-167725473	NA	0.136	0.002643161609	0.024	1
SE	TJAP1	+	chr6:43478658-43478718	NA	0.136	6.56E-08	0.004	1
SE	TMEM259	-	chr19:1011095-1011195	NA	0.136	1.43E-11	-0.003	1
SE	GPATCH8	-	chr17:44467096-44467114	NA	0.136	0.01205057511	-0.023	1
SE	TRMT1	-	chr19:13109914-13110001	NA	0.135	0.02576294352	-0.08	0.001079766575
SE	SEMA3D	-	chr7:85097804-85097965	NA	0.135	0.03294531518	0.002	1
SE	TSEN2	+	chr3:12505153-12505231	NA	0.135	0.02398359823	-0.015	1
SE	TOP3B	-	chr22:21975904-21976187	NA	0.134	0.0003277096277	-0.02	1
SE	SP110	-	chr2:230216780-230216821	NA	0.134	0.04267665388	-0.019	1
SE	VKORC1	-	chr16:31093311-31093482	NA	0.134	0.0001852608114	-0.058	3.77E-12
SE	IP6K2	-	chr3:48694458-48694525	NA	0.133	0.003363630737	0.013	1
SE	MTMR3	+	chr22:29970975-29971062	NA	0.133	0.01356089458	-0.007	1
SE	COL12A1	-	chr6:75138319-75138340	NA	0.133	0.006341438047	0.017	1
SE	STAU1	-	chr20:49165996-49166285	NA	0.133	1.66E-05	0.053	0.005303244308
SE	C16orf13	-	chr16:634888-634956	NA	0.133	0.0006246456998	-0.022	1
SE	FAM189B	-	chr1:155250275-155250821	NA	0.133	0.004324272947	-0.002	1
SE	TBC1D23	+	chr3:100311832-100311877	NA	0.132	0.0001948670864	0.026	1.62E-07
SE	CCNT2	+	chr2:134945786-134945982	NA	0.132	0.02298175873	0.056	6.14E-05
SE	CREM	+	chr10:35201450-35201486	NA	0.132	0.003127472154	-0.069	0.001607562557
SE	OCIAD1	+	chr4:48831226-48831496	NA	0.132	0.02831425901	0.019	0.08659141384
SE	FXR1	+	chr3:180971074-180971155	NA	0.132	0	0.014	0.0009531020857
SE	TRIM41	+	chr5:181233412-181233435	NA	0.132	0.00683098948	0.019	1
SE	UAP1L1	+	chr9:137078049-137078254	NA	0.132	0.02820264394	0.015	1
SE	PET100	+	chr19:7631231-7631321	NA	0.132	0	-0.005	0.4987957518
SE	ADRM1	+	chr20:62304460-62304577	NA	0.131	0	0.003	1
SE	GAK	-	chr4:851749-851974	NA	0.131	0.002471213769	0	1
SE	DNAJC13	+	chr3:132462466-132462523	NA	0.13	0.003988133396	-0.001	1
SE	SENP3	+	chr17:7565694-7565764	NA	0.13	0.0008458178938	-0.041	0.0001097197167
SE	MTX3	-	chr5:79990593-79990663	NA	0.13	0.0006469923546	-0.012	1
SE	ABI1	-	chr10:26755654-26755741	NA	0.129	0.0001864371792	0.096	1.45E-05
SE	TMEM219	+	chr16:29972041-29972140	NA	0.129	1.05E-05	0.007	1
SE	RCHY1	-	chr4:75491582-75491637	NA	0.129	0.04831464141	-0.039	0.9821681081
SE	HNRNPH1	-	chr5:179619268-179619360	NA	0.129	5.52E-09	0.023	0.1229639939
SE	ZNF569	-	chr19:37454833-37455073	NA	0.129	0.003350576238	0.017	1
SE	USP36	-	chr17:78796316-78796494	NA	0.128	0.001449853653	0.026	1
SE	TGFB3	-	chr14:75960268-75960386	NA	0.128	0.009293494939	0.008	0.1390942615
SE	SCPEP1	+	chr17:56998373-56998498	NA	0.128	0.01954785441	-0.004	1
SE	UBA1	+	chrX:47198166-47198355	NA	0.128	1.49E-10	0.015	0.0002299308617
SE	PUF60	-	chr8:143827384-143827536	NA	0.128	0.000780430881	-0.007	0.001798425144
SE	SH3YL1	-	chr2:242797-242871	NA	0.127	6.59E-08	0.033	0.01598709411
SE	TFIP11	-	chr22:26511118-26511317	NA	0.127	0.0127859952	0.015	1
SE	CEP152	-	chr15:48748442-48748610	NA	0.127	0.002066660603	-0.087	1
SE	GNPDA1	-	chr5:142012287-142012339	NA	0.127	0.0001952559675	-0.077	5.36E-11
SE	AHI1	-	chr6:135297431-135297492	NA	0.127	0.02667616491	0.015	1
SE	GNE	-	chr9:36236831-36236984	NA	0.127	0.003266110278	-0.063	0
SE	R3HDM2	-	chr12:57297327-57297387	NA	0.127	0.0003145254367	-0.027	0
SE	ARHGEF1	+	chr19:41892024-41892123	NA	0.126	0.008891331548	-0.029	6.36E-10
SE	RAB34	-	chr17:28717212-28717647	NA	0.126	0.01139775805	0.004	1
SE	DHRS1	-	chr14:24291555-24291625	NA	0.126	0.04221843917	0.008	1
SE	PCBP4	-	chr3:51961940-51962088	NA	0.125	0.02326580329	-0.038	1
SE	PFDN5	+	chr12:53296453-53296551	NA	0.125	0.006837178035	0.049	1.20E-11
SE	ARHGAP32	-	chr11:129040927-129041009	NA	0.125	0.0008706042036	-0.031	1
SE	KIAA2026	-	chr9:5933750-5933881	NA	0.125	0.005769454447	-0.062	1
SE	SLK	+	chr10:104002171-104003527	NA	0.124	2.43E-08	0.008	1
SE	ARVCF	-	chr22:19972357-19972411	NA	0.124	0.003171110219	-0.015	1
SE	UBE2E1	+	chr3:23811459-23811510	NA	0.124	0	0.004	1
SE	RPS28	+	chr19:8321655-8321703	NA	0.124	0.01756076189	0	1
SE	TNFRSF12A	+	chr16:3021214-3021319	NA	0.123	0.001898049618	0.001	1
SE	LRRC23	+	chr12:6907314-6907445	NA	0.123	0.0002615336253	-0.023	6.91E-09
SE	ASPM	-	chr1:197100430-197105185	NA	0.123	0.009515144024	-0.006	0.05706057739
SE	FANCI	+	chr15:89245194-89245311	NA	0.123	8.00E-09	-0.012	1
SE	ARNT	-	chr1:150842423-150842468	NA	0.123	0.0001999884726	-0.046	0.1877618683
SE	GATC	+	chr12:120454932-120455030	NA	0.123	8.48E-06	0.012	1

SE	SNX11	+	chr17:48110707-48110767	NA		0.122	4.93E-05	-0.026	0.0522783913
SE	NDUFC1	-	chr4:139297384-139297443	NA		0.122	0.02506932821	-0.057	0.0002301436447
SE	HYKK	+	chr15:78513083-78513425	NA		0.122	0.01097352416	0.001	1
SE	GLMP	-	chr1:156293319-156293429	NA		0.122	0.007592639129	-0.03	0.0004217889346
SE	THAP9-AS1	-	chr4:82897988-82898062	NA		0.122	0.02355317783	0.048	1
SE	GRAMD4	+	chr22:46676599-46676668	NA		0.121	0.0341951532	-0.026	1.83E-10
SE	PPP6R2	+	chr22:50391866-50392015	NA		0.121	0.01437782339	-0.073	1
SE	TMEM87B	+	chr2:112077191-112077282	NA		0.121	0.007276760501	-0.012	1
SE	KNTC1	+	chr12:122601535-122601625	NA		0.121	0.002873453659	0.008	1
SE	DDX42	+	chr17:63799588-63799625	NA		0.121	6.10E-06	-0.022	0.0004885254591
SE	LINC01021	+	chr5:27477638-27477801	NA		0.121	0.03820251916	0.1	1
SE	CHORDC1	-	chr11:90206747-90206810	NA		0.12	3.29E-05	0.068	9.72E-06
SE	CENPA	+	chr2:26792755-26792833	NA		0.12	5.47E-05	-0.003	1
SE	C9orf156	-	chr9:97912872-97912975	NA		0.12	0.01516291276	-0.01	1
SE	PRR4	-	chr12:11121009-11121180	NA		0.119	0.0009347379716	-0.038	0.9228271422
SE	GLS	+	chr2:190913192-190913364	NA		0.119	2.51E-07	0.002	1
SE	YLPM1	+	chr14:74824255-74824307	NA		0.119	0.0005060879067	0.005	1
SE	SPOP	-	chr17:49636758-49636809	NA		0.119	0.004939913488	0.001	1
SE	TMEM62	+	chr15:43138573-43138619	NA		0.119	0.02228420277	-0.089	1
SE	TPCN2	+	chr11:69067502-69067605	NA		0.119	0.03637629393	0	1
SE	RAB34	-	chr17:28717212-28717593	NA		0.118	0.005954277431	0	1
SE	SRSF7	-	chr2:38748897-38749346	NA		0.118	8.21E-09	-0.002	1
SE	ESCO2	+	chr8:27783999-27784057	NA		0.118	0.04261741669	0.01	1
SE	MLF1	+	chr3:158588851-158588924	NA		0.118	8.38E-08	0.011	1
SE	PRKDC	-	chr8:47782161-47782254	NA		0.118	1.08E-13	0.066	0.001044918815
SE	ZNF195	-	chr11:3361975-3362073	NA		0.117	0.004491791827	0.037	0.2244350862
SE	PPP5C	+	chr19:46383410-46383476	NA		0.117	4.00E-13	-0.053	1.26E-05
SE	UBE3A	-	chr15:25407066-25407262	NA		0.117	0.002354815036	0.006	1
SE	C16orf13	-	chr16:634888-635063	NA		0.117	0.0001053612437	-0.01	1
SE	DENND5A	-	chr11:9142721-9143485	NA		0.117	0.02602112914	-0.033	1.83E-08
SE	NOMO2	-	chr16:18528012-18528071	NA		0.117	0.01502360744	0.096	1
SE	CORO7	-	chr16:4415714-4415852	NA		0.117	2.30E-05	-0.005	1
SE	IFT88	+	chr13:20663262-20663386	NA		0.116	2.64E-05	-0.013	1
SE	USP33	-	chr1:77715741-77715868	NA		0.116	0.01924510814	-0.023	1
SE	ITGAE	-	chr17:3723287-3723383	NA		0.116	0	0.011	1
SE	COQ5	-	chr12:120526456-120526546	NA		0.116	8.30E-07	0.108	4.46E-09
SE	ZNF133	+	chr20:18297984-18298062	NA		0.116	0.04998063542	-0.059	1
SE	SGCE	-	chr7:94639340-94639448	NA		0.116	0.002822164336	0.032	1
SE	PCGF6	-	chr10:103347237-103347297	NA		0.116	0.01001026167	-0.102	0.0002471633823
SE	CEP57L1	+	chr6:109155790-109155877	NA		0.116	0.001692970305	0.004	1
SE	DZIP3	+	chr3:108605334-108605438	NA		0.116	0.002236110888	-0.045	1
SE	RAB34	-	chr17:28717212-28717620	NA		0.115	0.006132731773	0	1
SE	NENF	+	chr1:212442564-212442625	NA		0.115	0.0001526398926	-0.018	9.75E-06
SE	RAB34	-	chr17:28717212-28717647	NA		0.114	0.006207750042	0	1
SE	NDUFS8	+	chr11:68035627-68035788	NA		0.114	1.32E-06	0.003	1
SE	MED6	-	chr14:70585755-70585783	NA		0.114	1.29E-09	0.003	1
SE	UGGT1	+	chr2:128156391-128156415	NA		0.114	0.002140032246	0.042	1
SE	CD320	-	chr19:8303854-8304088	NA		0.114	0.0006458606726	-0.025	2.09E-09
SE	AK2	-	chr1:33012073-33012122	NA		0.113	2.89E-14	0.032	2.68E-06
SE	RECQL	-	chr12:21501169-21501372	NA		0.113	0.002987512401	-0.03	1
SE	DLGAP4	+	chr20:36465264-36465367	NA		0.113	0.006207750042	0.011	1
SE	DGUOK	+	chr2:73950626-73950732	NA		0.113	1.12E-08	0.138	0
SE	SGK494	-	chr17:28612261-28612366	NA		0.113	0.03885696071	-0.029	1
SE	SH2B1	+	chr16:28866011-28867033	NA		0.113	0.005860391112	0.002	1
SE	PPP2R3C	-	chr14:35109818-35109901	NA		0.112	0.01192661118	-0.004	1
SE	TOP3B	-	chr22:21975980-21976187	NA		0.112	0.0164236481	-0.031	1
SE	RARS2	-	chr6:87545615-87545699	NA		0.112	0.004068455867	-0.025	1
SE	PAPD4	+	chr5:79615074-79615163	NA		0.112	0.003439201947	-0.004	1
SE	MFF	+	chr2:227342744-227342819	NA		0.112	2.01E-08	-0.018	0.3763213742
SE	ENTPD5	-	chr14:73972883-73973024	NA		0.112	0.005732405631	0	1
SE	TMEM63A	-	chr1:225866573-225866682	NA		0.112	0.006984479321	0.001	1
SE	ATP11C	-	chrX:139737730-139737796	NA		0.111	0.01111677213	0.18	1
SE	SPAG1	+	chr8:100220278-100220431	NA		0.111	0.003664403339	-0.012	1

SE	CARD8	-	chr19:48223718-48223868	NA	0.111	0.02523536706	-0.019	1
SE	CLIP1	-	chr12:122351110-122351143	NA	0.111	0.003673171316	0.078	1
SE	CPSF7	-	chr11:61420469-61420530	NA	0.111	0.002471171293	-0.016	5.46E-05
SE	GSAP	-	chr7:77353571-77353641	NA	0.111	0.01348537548	0.014	1
SE	SUSD1	-	chr9:112042100-112042165	NA	0.11	0.01062440517	-0.002	1
SE	SRSF6	+	chr20:43459152-43459420	NA	0.11	0.0009388080305	0.069	0.08187010603
SE	ECSIT	-	chr19:11519074-11519193	NA	0.11	0.001119481664	-0.054	2.61E-07
SE	STXBP1	+	chr9:127684367-127684493	NA	0.11	1.02E-06	0.024	4.37E-10
SE	APLP2	+	chr11:130123611-130123779	NA	0.109	0.02102855911	-0.054	0.06573600478
SE	AP3B1	-	chr5:78175625-78175697	NA	0.109	2.62E-06	0.006	1
SE	WARS	-	chr14:100375282-100375350	NA	0.109	0.003593871272	-0.127	0.009775879358
SE	DAK	+	chr11:61343341-61343458	NA	0.109	0.002348355715	-0.017	0.009262246572
SE	PRKAG1	-	chr12:49005742-49005852	NA	0.109	1.93E-09	-0.038	3.69E-11
SE	CKLF	+	chr16:66563121-66563217	NA	0.109	2.44E-06	-0.007	0.4918071069
SE	TMEM260	+	chr14:56593114-56593317	NA	0.108	0.02900749021	0.019	1
SE	SEC22C	-	chr3:42563764-42563880	NA	0.108	5.82E-12	-0.01	0.03225444425
SE	CENPT	-	chr16:67832227-67832350	NA	0.108	0.0007237793159	-0.005	1
SE	PEX7	+	chr6:136866626-136866733	NA	0.108	0.01676038414	-0.007	1
SE	FAM120B	+	chr6:170348150-170348323	NA	0.108	0.006986795451	-0.008	1
SE	SPIRE1	-	chr18:12459754-12459928	NA	0.108	0.001432568516	-0.043	1
SE	KIF16B	-	chr20:16335925-16336015	NA	0.107	0.016488075	0.013	1
SE	CENPT	-	chr16:67832227-67832354	NA	0.107	0.0006888228651	-0.006	1
SE	ZNF326	+	chr1:90007344-90007750	NA	0.107	6.21E-05	0.003	1
SE	SUN1	+	chr7:849519-849603	NA	0.107	1.48E-09	-0.009	0.1970997238
SE	LRTOMT	+	chr11:72089416-72089596	NA	0.107	0.02085996974	-0.022	0.01970884038
SE	SYNRG	-	chr17:37577379-37577613	NA	0.107	0.009170669219	-0.053	2.99E-11
SE	MYO19	-	chr17:36501851-36501901	NA	0.107	0.00682352165	0.196	1.40E-11
SE	ATP6V0A1	+	chr17:42507519-42507627	NA	0.106	7.40E-05	-0.003	1
SE	DDX58	-	chr9:32492390-32492538	NA	0.106	0.002559520408	-0.012	1
SE	DDX50	+	chr10:68906225-68906337	NA	0.106	1.74E-06	0.021	1
SE	RTN4	-	chr2:55028163-55028220	NA	0.106	4.37E-06	0.032	4.56E-09
SE	CDCA7	+	chr2:173359342-173359491	NA	0.106	0.0004100811703	0.101	7.40E-06
SE	CENPJ	-	chr13:24886233-24886385	NA	0.106	0.02450060409	0.02	0.2310102641
SE	MIA3	+	chr1:222662887-222663031	NA	0.106	0.02231683261	-0.004	1
SE	FAM208A	-	chr3:56638705-56638765	NA	0.106	0.0001130893075	0.003	1
SE	CTBP2	-	chr10:125110989-125111093	NA	0.106	0.04007048318	-0.008	1
SE	BCL2L12	+	chr19:49667018-49667161	NA	0.105	0.01389440019	-0.024	0.4661532477
SE	ZGRF1	-	chr4:112589723-112589874	NA	0.105	0.01226428593	-0.026	1
SE	ESCO2	+	chr8:27780173-27780267	NA	0.105	0.04297087789	0.004	1
SE	TMEM230	-	chr20:5111763-5111808	NA	0.104	0.002611775813	0.01	1
SE	DHPS	-	chr19:12677107-12677211	NA	0.104	2.35E-13	-0.02	1.13E-06
SE	CEP192	+	chr18:13058293-13058435	NA	0.104	1.04E-05	0.007	1
SE	PAPD7	+	chr5:6743711-6743840	NA	0.104	2.72E-05	-0.004	1
SE	ENOSF1	-	chr18:677344-677444	NA	0.104	0.001190248648	-0.009	1
SE	SLC38A6	+	chr14:61034018-61034113	NA	0.104	0.001528473657	0.018	1
SE	FAM227B	-	chr15:49611214-49611268	NA	0.104	0.02259035035	0.005	1
SE	SERPINB6	-	chr6:2968778-2968913	NA	0.103	2.38E-05	0.007	1
SE	GNPDA1	-	chr5:142012287-142012377	NA	0.102	3.27E-05	-0.065	3.15E-14
SE	CDCA7	+	chr2:173363999-173364161	NA	0.102	0.003022493822	0.021	0.01067769121
SE	SIDT2	+	chr11:117186989-117187001	NA	0.102	0.001234249565	0.102	2.31E-05
SE	CENPJ	-	chr13:24886234-24886385	NA	0.102	0.02219822181	0.027	0.06661899907
SE	FRS2	+	chr12:69537915-69537983	NA	0.102	0.007194573322	0.024	1
SE	RNF10	+	chr12:120539367-120539468	NA	0.101	0.0004537657274	0.019	0.0003902832663
SE	TEAD2	-	chr19:49359843-49360081	NA	0.101	0.0003628054664	0.003	1
SE	DTWD1	+	chr15:49630955-49631058	NA	0.101	2.38E-08	-0.012	0.1724486627
SE	RAB34	-	chr17:28717212-28717398	NA	0.101	0.007840343391	-0.001	1
SE	GOLT1B	+	chr12:21508382-21508561	NA	0.101	6.35E-09	0.011	1
SE	DGUOK	+	chr2:73950584-73950732	NA	0.101	0.007035617093	0.019	0.04222875936
SE	RARS2	-	chr6:87548590-87548646	NA	0.101	0.004302882599	-0.029	1.49E-13
SE	TBC1D31	+	chr8:123082701-123082817	NA	0.101	0.0004520812027	-0.055	1
SE	RPUSD4	-	chr11:126206253-126206553	NA	0.101	0.0003073383043	0.004	0.3340085523
SE	KPNA5	+	chr6:116692054-116692156	NA	0.101	0.005599612143	0.004	1
SE	AC007040.1	-	chr2:70991829-70991983	NA	0.101	0.01868342172	-0.044	3.91E-13

SE	CWF19L1	-	chr10:100250247-100250332	NA	-0.101	0.00170288151	-0.005	1
SE	CABIN1	+	chr22:24041138-24041273	NA	-0.101	0.03112960624	-0.035	1
SE	NHP2L1	-	chr22:41682355-41682587	NA	-0.101	1.11E-11	0.02	3.20E-08
SE	CARF	+	chr2:202974333-202974496	NA	-0.101	0.003230019984	-0.027	1
SE	CENPE	-	chr4:103143247-103143406	NA	-0.101	3.31E-05	-0.022	1
SE	DOLPP1	+	chr9:129086138-129086267	NA	-0.101	0.01074850663	-0.004	1
SE	BAG6	-	chr6:31644306-31644414	NA	-0.101	0.03174855014	-0.102	0
SE	CHMP2B	+	chr3:87240322-87240453	NA	-0.102	0.04720535292	-0.005	0.267483413
SE	UBE2Q2	+	chr15:75890434-75890483	NA	-0.102	0.004301470106	-0.009	1
SE	GFM2	-	chr5:74738501-74738642	NA	-0.102	0.01693677111	-0.043	6.60E-06
SE	KIAA0430	-	chr16:15624768-15624927	NA	-0.102	0.002706069451	0.004	1
SE	PSMA3-AS1	-	chr14:58293313-58293544	NA	-0.102	0.005554562012	0.001	1
SE	TOP3B	-	chr22:21970209-21970406	NA	-0.103	0.02168639396	0.003	1
SE	PHF3	+	chr6:63678827-63679020	NA	-0.103	7.80E-07	0.033	1
SE	ZNF692	-	chr1:248856372-248856422	NA	-0.103	0.001282264471	-0.019	1
SE	ZNF562	-	chr19:9658008-9658135	NA	-0.103	0.03489005793	0.004	1
SE	UBTF	-	chr17:44208618-44209001	NA	-0.104	6.26E-08	-0.003	1
SE	POT1	-	chr7:124870910-124871041	NA	-0.104	0.01884693048	-0.005	1
SE	UBR3	+	chr2:169877493-169877637	NA	-0.104	0.002544829601	0.021	1
SE	NMT2	-	chr10:15130141-15130312	NA	-0.104	0.001857369881	-0.003	1
SE	ZBTB8OS	-	chr1:32633261-32633354	NA	-0.104	0.005563206351	0.008	1
SE	PSTK	+	chr10:122986299-122986375	NA	-0.104	0.04261741669	-0.051	1.21E-07
SE	RB1CC1	-	chr8:52685936-52686156	NA	-0.105	0.0001409018816	0.037	1
SE	TNRC6A	+	chr16:24776932-24777358	NA	-0.105	0.006129789789	-0.01	1
SE	SMC4	+	chr3:160412529-160412849	NA	-0.105	1.36E-12	0	1
SE	METTL14	+	chr4:118705610-118705821	NA	-0.105	5.49E-05	-0.016	1
SE	RPRD2	+	chr1:150464526-150464727	NA	-0.105	0.001107474483	-0.019	1
SE	ZNF561	-	chr19:9617620-9617768	NA	-0.105	0.006032195647	-0.002	1
SE	GABBR1	-	chr6:29608600-29608733	NA	-0.105	0.0005418065682	-0.016	1
SE	HRSP12	-	chr8:98108645-98108713	NA	-0.106	0.00140484798	-0.013	0.09155746834
SE	ANAPC11	+	chr17:81899241-81899542	NA	-0.106	0	0	1
SE	PAM	+	chr5:103025130-103025334	NA	-0.106	0.001547919879	0.005	1
SE	SFXN2	+	chr10:102729318-102729394	NA	-0.106	0.04627864623	-0.153	5.96E-10
SE	NAA16	+	chr13:41355143-41355216	NA	-0.106	0.009473724616	0.015	1
SE	SEC24C	+	chr10:73746629-73746716	NA	-0.106	8.85E-06	-0.008	1
SE	SNHG5	-	chr6:85677790-85677875	NA	-0.106	4.04E-11	-0.135	3.15E-14
SE	KATNAL1	-	chr13:30255446-30255615	NA	-0.107	0.0002645918642	-0.019	1
SE	COQ3	-	chr6:99383007-99383130	NA	-0.107	0.009287960857	0.009	1
SE	FAM175A	-	chr4:83470202-83470396	NA	-0.107	0.03069639954	-0.004	1
SE	NUP50	+	chr22:45170987-45171152	NA	-0.108	0.0205273931	0.018	0.2460414665
SE	MFSD10	-	chr4:2931554-2931663	NA	-0.108	0.0007475297347	-0.065	3.15E-14
SE	ACAD10	+	chr12:111729805-111729956	NA	-0.108	0.01123275744	-0.056	1
SE	GABPB2	+	chr1:151073038-151073157	NA	-0.108	0.03900299778	0.001	1
SE	C5orf45	-	chr5:179851242-179851337	NA	-0.108	0.009537224812	0	1
SE	RP11-33B1.	+	chr4:119488644-119488717	NA	-0.108	0.002384550275	-0.01	1
SE	RBM6	+	chr3:49967197-49968748	NA	-0.109	2.69E-05	-0.006	1
SE	DCUN1D4	+	chr4:51905188-51905320	NA	-0.109	2.74E-06	0.015	1
SE	TIMM8B	-	chr11:112085966-112086310	NA	-0.109	0.0008728679574	-0.003	1
SE	CHURC1	+	chr14:64926009-64926080	NA	-0.109	9.60E-08	0	1
SE	VPS41	-	chr7:38796414-38796526	NA	-0.11	1.23E-08	0.001	1
SE	TUBD1	-	chr17:59886082-59886230	NA	-0.11	0.03243601941	-0.046	1
SE	WHSC1	+	chr4:1954536-1954685	NA	-0.11	2.54E-05	0.004	1
SE	BCHE	-	chr3:165777716-165777795	NA	-0.11	1.52E-06	0.006	7.54E-09
SE	TMEM45A	+	chr3:100519076-100519316	NA	-0.11	6.71E-06	0.005	1
SE	ARID4B	-	chr1:235213768-235214026	NA	-0.111	0.002459905923	0.036	1
SE	HNRNPUL1	+	chr19:41305831-41305867	NA	-0.111	0.000558909809	-0.011	0.5809769352
SE	HACL1	-	chr3:15574976-15575082	NA	-0.111	0.0003159746893	-0.045	1
SE	BCL2L11	+	chr2:111123732-111123869	NA	-0.111	0.01404100243	0.046	1
SE	SLC37A3	-	chr7:140345868-140345970	NA	-0.111	0.01020257768	-0.035	1
SE	EDC3	-	chr15:74687090-74687179	NA	-0.111	0.0231839421	-0.005	1
SE	PMS2CL	+	chr7:6737075-6737936	NA	-0.111	0.0004879258515	0.033	1
SE	GTF2IP1	-	chr7:75202541-75202613	NA	-0.111	0.0239620042	0.016	1
SE	RABGAP1	+	chr9:122945331-122945432	NA	-0.112	0.001312112384	-0.011	1

SE	RPLP0	-	chr12:120198553-120198739	NA	-0.112	0	-0.009	0
SE	TOM1	+	chr22:35321958-35322019	NA	-0.112	0.02259035035	-0.042	0.00244859248
SE	HMGCR	+	chr5:75355055-75355214	NA	-0.112	0	0.012	1
SE	B4GALT3	-	chr1:161176911-161177106	NA	-0.112	2.40E-08	0.002	0.3906935861
SE	TMEM194A	-	chr12:57069233-57069306	NA	-0.112	0.043757897	-0.018	1
SE	ZFAS1	+	chr20:49280484-49280570	NA	-0.112	5.59E-14	-0.074	0
SE	ASPH	-	chr8:61646749-61646878	NA	-0.112	0	-0.002	1
SE	TADA2A	+	chr17:37423508-37423615	NA	-0.112	0.007108285273	0.012	1
SE	CHFR	-	chr12:132870723-132870783	NA	-0.113	0.009067303247	-0.033	1
SE	MVK	+	chr12:109579801-109579946	NA	-0.113	0.019011357	-0.089	0.0005025009757
SE	B3GALNT1	-	chr3:161089959-161090062	NA	-0.113	0.01458009455	0.058	4.50E-12
SE	FUT8	+	chr14:65430215-65430309	NA	-0.114	0.02578183739	0.006	1
SE	EPB41L2	-	chr6:130878103-130878250	NA	-0.114	1.20E-05	-0.222	2.05E-13
SE	MYL6	+	chr12:56159586-56159622	NA	-0.114	0.003043669782	-0.013	0
SE	ZNF317	+	chr19:9157979-9158075	NA	-0.114	0.01281397378	-0.031	1
SE	CDK7	+	chr5:69254601-69254669	NA	-0.114	0.01973663004	-0.014	1
SE	BORA	+	chr13:72734959-72735005	NA	-0.114	0.02872793168	-0.105	0
SE	FAM188A	-	chr10:15816834-15816915	NA	-0.114	0.03671992905	-0.026	0.4593155239
SE	TMEM67	+	chr8:93791881-93792004	NA	-0.114	0.02775592997	0.005	1
SE	BRD7	-	chr16:50326283-50326391	NA	-0.114	6.79E-10	0.001	1
SE	L3MBTL3	+	chr6:130049650-130049830	NA	-0.114	0.03879465813	0.039	1
SE	MYO19	-	chr17:36512657-36512779	NA	-0.114	0.008854836282	0.062	0.2365470521
SE	GLS	+	chr2:190913192-190913364	NA	-0.115	0.001084209801	-0.053	1
SE	INIP	-	chr9:112701787-112701880	NA	-0.115	0.0001351758142	-0.007	1
SE	ANO10	-	chr3:43551471-43551588	NA	-0.115	2.25E-05	0.005	0.9816504588
SE	FBXO22	+	chr15:75913202-75913290	NA	-0.115	0.02857103996	-0.004	1
SE	GTF2E2	-	chr8:30612298-30612481	NA	-0.115	3.77E-11	0.013	1
SE	NFATC3	+	chr16:68221195-68221284	NA	-0.116	0.0364768483	-0.006	1
SE	CDCA3	-	chr12:6849322-6849413	NA	-0.116	3.55E-08	-0.103	0.004971439658
SE	DLG4	-	chr17:7191892-7192002	NA	-0.116	0.004221254915	-0.007	1
SE	CARS2	-	chr13:110648317-110649297	NA	-0.116	1.25E-08	0.003	1
SE	UBQLN1	-	chr9:83666349-83666394	NA	-0.116	0.01785569141	-0.051	0.07701091082
SE	N6AMT2	-	chr13:20731840-20732121	NA	-0.116	0.03161887069	0.028	0.2821223924
SE	USP25	+	chr21:15849776-15849872	NA	-0.116	0.0002637844158	0.004	1
SE	SLC12A2	+	chr5:128177104-128177152	NA	-0.117	4.35E-06	-0.051	0.1017851617
SE	NFYC	+	chr1:40766917-40766974	NA	-0.117	0.002262356732	-0.008	0.01352635303
SE	SEC14L2	+	chr22:30415758-30416087	NA	-0.117	0.0002925652383	-0.038	7.19E-12
SE	RPAIN	+	chr17:5426235-5426299	NA	-0.117	0.0008985289339	-0.123	1.10E-11
SE	CCNT1	-	chr12:48695998-48696162	NA	-0.117	0.005099606958	-0.051	1
SE	C6orf52	-	chr6:10686965-10687056	NA	-0.117	0.0084424949	-0.004	1
SE	SLC33A1	-	chr3:155833467-155833585	NA	-0.117	0.00728625754	-0.006	1
SE	URB2	+	chr1:229645858-229645969	NA	-0.118	0.004374083708	0.002	1
SE	NUP54	-	chr4:76134174-76134362	NA	-0.118	3.46E-06	0.009	1
SE	CDC25C	-	chr5:138289479-138289563	NA	-0.118	0.01782449163	-0.038	1
SE	TRAPPC6B	-	chr14:39154210-39154294	NA	-0.118	0.01034319119	-0.003	1
SE	C12orf65	+	chr12:123253646-123255033	NA	-0.119	0.01080983012	0.011	1
SE	ANK3	-	chr10:60059697-60060033	NA	-0.119	0.00550678331	-0.196	3.65E-13
SE	HEATR3	+	chr16:50072604-50072714	NA	-0.119	0.02655662668	-0.026	1
SE	GEMIN4	-	chr17:749813-749909	NA	-0.119	0.03462897539	0.038	1
SE	HERC2P9	+	chr15:28612058-28612173	NA	-0.119	0.0384989077	-0.019	1
SE	ELOVL5	-	chr6:53273219-53273344	NA	-0.12	0	-0.031	0.0170225618
SE	PLEKHH1	+	chr14:67586809-67586949	NA	-0.12	0.0001479566393	0.017	3.40E-11
SE	CCNC	-	chr6:99544205-99544273	NA	-0.12	1.47E-07	0.013	2.72E-06
SE	LIAS	+	chr4:39462195-39462289	NA	-0.12	0.02343194834	-0.02	1
SE	RBCK1	+	chr20:409883-410025	NA	-0.12	0.006423577048	-0.003	1
SE	FKBP10	+	chr17:41819840-41820023	NA	-0.12	2.58E-07	0.019	0
SE	LRCH3	+	chr3:197866111-197866219	NA	-0.12	1.39E-05	-0.075	5.01E-05
SE	PHTF2	+	chr7:77937709-77937838	NA	-0.121	1.21E-07	-0.014	1
SE	DHX9	+	chr1:182855576-182855713	NA	-0.121	3.15E-10	0.008	0.2382460916
SE	AP1AR	+	chr4:112262987-112263086	NA	-0.121	0.002927110877	-0.172	4.17E-13
SE	TIMM8B	-	chr11:112085977-112086310	NA	-0.121	0.001838520626	-0.004	1
SE	ZNF202	-	chr11:123727475-123727595	NA	-0.121	1.81E-05	-0.026	1
SE	XRRA1	-	chr11:74909888-74910012	NA	-0.121	0.00199678348	-0.034	1

SE	KATNAL2	+	chr18:47046390-47046527	NA		-0.121	0.01677792067	0.016	1
SE	UBA2	+	chr19:34430092-34430186	NA		-0.122	2.15E-07	0.03	6.66E-08
SE	PRKRIP1	+	chr7:102397619-102397698	NA		-0.122	0.0140171851	-0.039	0.04239164553
SE	CAMK1	-	chr3:9765758-9765890	NA		-0.122	0.001499769913	-0.038	2.09E-07
SE	GGACT	-	chr13:100533562-100533774	NA		-0.122	0.008751623156	0.063	1
SE	RNF157	-	chr17:76155251-76155317	NA		-0.122	0.0145371809	-0.027	3.60E-08
SE	VPS8	+	chr3:184922428-184922483	NA		-0.122	6.66E-05	-0.011	1
SE	USP54	-	chr10:73504849-73504990	NA		-0.122	0.0168943985	-0.023	1
SE	ZNF561	-	chr19:9617620-9617758	NA		-0.122	0.002940491554	0.001	1
SE	CDK11A	-	chr1:1722587-1722634	NA		-0.123	0.01643350428	-0.002	1
SE	ADAT1	-	chr16:75618585-75618698	NA		-0.123	0.009096233477	-0.038	1
SE	USP53	+	chr4:119268267-119268420	NA		-0.123	0.00393903467	-0.013	1
SE	GFM2	-	chr5:74730259-74730398	NA		-0.123	0.003000752999	-0.027	0.180170117
SE	POLL	-	chr10:101582762-101582891	NA		-0.123	0.001498880467	0.006	1
SE	IST1	+	chr16:71924090-71924281	NA		-0.123	0.001113102966	0.01	2.26E-06
SE	ANGEL1	-	chr14:76810088-76810254	NA		-0.124	0.0001146705376	-0.008	1
SE	POLQ	-	chr3:121473349-121473487	NA		-0.124	0.008199787959	-0.001	1
SE	MADD	+	chr11:47288967-47289027	NA		-0.124	0.01952163363	0.008	1
SE	TJP2	+	chr9:69251034-69251364	NA		-0.124	0.03536904157	-0.048	0.001909610936
SE	ZMYM5	-	chr13:19838699-19838985	NA		-0.124	0.0002257543833	0.023	1
SE	PPCDC	+	chr15:75043440-75043536	NA		-0.124	0.03357411761	0.005	1
SE	PAM	+	chr5:102974115-102974436	NA		-0.124	0.001610289966	0.028	1
SE	MRRF	+	chr9:122286038-122286166	NA		-0.124	0.004509801696	0.066	0.1442757745
SE	KIAA0753	-	chr17:6611918-6612148	NA		-0.124	0.006509517624	0.006	1
SE	SNHG5	-	chr6:85677794-85677868	NA		-0.124	3.33E-08	-0.136	0
SE	LAS1L	-	chrX:65528259-65528369	NA		-0.125	2.61E-07	-0.023	1.65E-10
SE	USP28	-	chr11:113830866-113830946	NA		-0.125	0.0296972396	-0.014	1
SE	ALDH3A2	+	chr17:19673150-19673275	NA		-0.125	1.60E-07	0.041	1
SE	RPLP0	-	chr12:120198553-120198770	NA		-0.125	0	-0.013	2.05E-13
SE	NUP50	+	chr22:45170987-45171143	NA		-0.125	0.000806993149	0.012	1
SE	EIF2D	-	chr1:206599462-206599612	NA		-0.125	1.15E-11	-0.089	0
SE	PAM	+	chr5:103025133-103025334	NA		-0.125	0.001027895403	0.005	1
SE	PDPR	+	chr16:70131301-70131419	NA		-0.126	0.03206756502	0.079	1
SE	IMMP1L	-	chr11:31456259-31456386	NA		-0.126	0.01808880893	-0.02	1
SE	TMEM237	-	chr2:201634803-201634893	NA		-0.126	8.05E-09	0.008	1
SE	PHF5A	-	chr22:41468019-41468147	NA		-0.127	8.80E-05	-0.031	2.63E-07
SE	CBLB	-	chr3:105681478-105681610	NA		-0.127	0.001497862413	-0.031	1
SE	HNRNPR	-	chr1:23340851-23341020	NA		-0.127	6.33E-05	-0.002	1
SE	TPRKB	-	chr2:73734527-73734591	NA		-0.127	0.001712917184	0.003	1
SE	TBL1X	+	chrX:9534899-9535034	NA		-0.128	0.0006349605137	0.005	1
SE	PHF20L1	+	chr8:132825034-132825157	NA		-0.128	0.0005152712216	0.036	0.07618515052
SE	SNHG5	-	chr6:85677794-85677899	NA		-0.128	7.18E-08	-0.147	9.07E-14
SE	SNAP23	+	chr15:42529674-42529819	NA		-0.129	0.0001857822036	0.001	1
SE	HDLBP	-	chr2:241269360-241269561	NA		-0.129	2.19E-07	-0.007	1
SE	TNIK	-	chr3:171128713-171128878	NA		-0.129	0.00168795208	0.006	1
SE	THAP6	+	chr4:75521735-75521861	NA		-0.129	0.005749812137	-0.064	4.17E-13
SE	MORF4L1	+	chr15:78887268-78887349	NA		-0.129	0.003025881065	-0.002	1
SE	TCOF1	+	chr5:150372005-150372236	NA		-0.13	0.003432257142	0.001	1
SE	DNMT3A	-	chr2:25239314-25239513	NA		-0.13	0.001496409421	0.001	1
SE	ZCCHC11	-	chr1:52458335-52458449	NA		-0.13	0.03293364916	0.008	1
SE	TRPC1	+	chr3:142792823-142792967	NA		-0.13	0.007980648665	0.007	1
SE	MRPS18C	+	chr4:83458345-83458429	NA		-0.13	1.79E-06	-0.013	0.2516003699
SE	DENND5A	-	chr11:9176771-9176902	NA		-0.13	3.99E-12	0.004	1
SE	TFDP1	+	chr13:113631622-113631905	NA		-0.13	0.01092216805	-0.112	1.87E-11
SE	ACTN1	-	chr14:68878988-68879069	NA		-0.131	2.38E-08	-0.019	1.92E-05
SE	ZNF302	+	chr19:34679827-34679959	NA		-0.131	1.32E-06	0.001	0.0002950735094
SE	NQO2	+	chr6:3002049-3002286	NA		-0.131	2.94E-07	0.003	1
SE	HP1BP3	-	chr1:20780344-20780540	NA		-0.131	0.0274504833	-0.054	0.05102852609
SE	TBC1D5	-	chr3:17622647-17622765	NA		-0.131	0.0439700115	-0.037	1
SE	ZMYM5	-	chr13:19837655-19837821	NA		-0.131	0.009523405635	0	1
SE	CDK7	+	chr5:69254601-69254669	NA		-0.131	0.02599262688	0.061	1
SE	GIT2	-	chr12:109951166-109951316	NA		-0.131	0.003988133396	-0.004	1
SE	WDR89	-	chr14:63641298-63641407	NA		-0.131	0.01313831589	-0.002	1

SE	C9orf85	+	chr9:71971504-71971618	NA	-0.131	0.004011400497	-0.018	1
SE	TAB3	-	chrX:30842965-30843049	NA	-0.131	0.0088897773	0.02	1
SE	ARAP1	-	chr11:72692752-72692785	NA	-0.131	0.04752424816	-0.018	0.7127745885
SE	TRIM16	-	chr17:15682860-15682951	NA	-0.131	0.006132731773	-0.144	0.0002239694333
SE	ATG5	-	chr6:106308363-106308491	NA	-0.132	0.009067089434	-0.067	0.0003631199993
SE	THOC5	-	chr22:29531077-29531110	NA	-0.132	2.46E-05	-0.013	0.3101146591
SE	DLGAP5	-	chr14:55148593-55148684	NA	-0.132	3.50E-10	0.039	1
SE	MICAL1	-	chr6:109447874-109447963	NA	-0.132	0.01210955425	-0.046	2.05E-13
SE	KANSL2	-	chr12:48679035-48679150	NA	-0.132	0.0006849077524	-0.031	1
SE	FAM192A	-	chr16:57174619-57174688	NA	-0.132	7.91E-09	-0.017	2.03E-05
SE	ZNF37BP	-	chr10:42525539-42525660	NA	-0.132	0.001151705187	0.084	1
SE	RP11-87H9.	+	chr9:41008880-41009006	NA	-0.132	0.0006889078724	-0.016	1
SE	RSU1	-	chr10:16816972-16817084	NA	-0.133	1.64E-07	-0.025	0.7525329271
SE	AASDH	-	chr4:56345126-56345290	NA	-0.133	0.004080108744	-0.046	1
SE	STARD4	-	chr5:111501961-111502088	NA	-0.133	0.02770700481	0.013	1
SE	STK40	-	chr1:36367847-36368084	NA	-0.133	0.008231467309	-0.032	0.0006167935661
SE	TLE1	-	chr9:81652213-81652288	NA	-0.133	0.02872169449	-0.015	1
SE	KIAA1715	-	chr2:175994133-175994280	NA	-0.134	5.77E-09	-0.002	1
SE	LRP8	-	chr1:53257239-53257464	NA	-0.134	3.98E-08	0.004	1
SE	ZNF271P	+	chr18:35291009-35291232	NA	-0.134	0.01506849521	-0.005	1
SE	SPPL2A	-	chr15:50726072-50726126	NA	-0.135	0.01789049185	-0.024	0.8359812358
SE	ANK3	-	chr10:60200128-60200263	NA	-0.135	0.002225080387	-0.022	1
SE	RHNO1	+	chr12:2885262-2885534	NA	-0.135	0.0004129172434	-0.024	0.04322393452
SE	RBM23	-	chr14:22905605-22905659	NA	-0.136	6.52E-08	-0.001	1
SE	CLCN3	+	chr4:169717791-169717867	NA	-0.136	2.41E-05	-0.022	0.09189064949
SE	MICAL2	+	chr11:12249183-12249246	NA	-0.136	0.02165860992	-0.015	1
SE	SCLT1	-	chr4:128942995-128943188	NA	-0.136	0.03286776765	-0.008	1
SE	RPUSD3	-	chr3:9838984-9839171	NA	-0.136	0.007378662604	-0.022	0.04035963457
SE	CLHC1	-	chr2:55180509-55180712	NA	-0.136	0.001870402945	0.01	1
SE	SETD2	-	chr3:47080800-47081083	NA	-0.136	0.004085034607	-0.003	1
SE	HNRNPAB	+	chr5:178210131-178210272	NA	-0.136	2.85E-13	-0.026	0.221852334
SE	CHPF2	+	chr7:151236955-151237172	NA	-0.137	0.0003362019884	0.079	0.2590016045
SE	NTAN1	-	chr16:15047996-15048099	NA	-0.137	0.002085721271	-0.026	1
SE	IRAK4	+	chr12:43772911-43772986	NA	-0.137	0.01711610116	-0.014	1
SE	CCNC	-	chr6:99544178-99544273	NA	-0.138	1.82E-06	0.019	1.57E-09
SE	ANKRD11	-	chr16:89313316-89313589	NA	-0.138	1.55E-09	0.002	1
SE	ENO3	+	chr17:4952024-4952147	NA	-0.139	0.00490899262	0.012	0.4798648571
SE	MRPL47	-	chr3:179602651-179602737	NA	-0.139	1.07E-12	-0.014	1
SE	GUF1	+	chr4:44691665-44691799	NA	-0.139	0.03109214455	0.023	1
SE	ASAP1	-	chr8:130091972-130092143	NA	-0.139	0.0008506612306	-0.032	0.6580130316
SE	SPG7	+	chr16:89545871-89546030	NA	-0.139	1.91E-06	0.028	0
SE	RPLP0	-	chr12:120198553-120198631	NA	-0.14	0	-0.015	8.93E-12
SE	ZMIZ1	+	chr10:79162052-79162133	NA	-0.14	0.000864542017	-0.035	1
SE	MFF	+	chr2:227347225-227347384	NA	-0.14	0.009275745962	-0.084	6.66E-09
SE	ZNF655	+	chr7:99561887-99562494	NA	-0.14	0.02620422848	-0.114	0.0009465271811
SE	EML1	+	chr14:99914565-99914697	NA	-0.141	0.01019963675	-0.008	1
SE	LTA4H	-	chr12:96003837-96003981	NA	-0.141	0.02140667551	-0.038	0.5455110131
SE	YWHAZ	-	chr8:100951928-100952125	NA	-0.141	1.20E-07	0.009	1
SE	FUZ	-	chr19:49812446-49812736	NA	-0.142	0.001793405002	-0.067	0.01336889041
SE	C12orf4	-	chr12:4517060-4517189	NA	-0.142	0.0001872045859	0.005	1
SE	BTN2A1	+	chr6:26458606-26458718	NA	-0.142	0.001159125414	-0.023	0.007301125562
SE	POC1B	-	chr12:89425160-89425379	NA	-0.142	5.41E-06	0.037	0.01190586918
SE	EYA3	-	chr1:28027788-28027926	NA	-0.142	0.007720184609	-0.019	1
SE	CHTF8	-	chr16:69121052-69121170	NA	-0.142	2.57E-05	-0.055	1
SE	FAM210A	-	chr18:13671861-13671973	NA	-0.142	0.000175916401	-0.016	1
SE	FAM120C	-	chrX:54085714-54085916	NA	-0.142	0.005509184309	0.022	1
SE	LETMD1	+	chr12:51058005-51058128	NA	-0.143	0.03029140932	0.009	1
SE	CYP20A1	+	chr2:203272669-203272748	NA	-0.143	0.003905914187	-0.047	0.09191980553
SE	CHTF8	-	chr16:69120106-69120208	NA	-0.143	0.04354642454	0.026	1
SE	COPG1	+	chr3:129250681-129250894	NA	-0.143	0.002239049543	-0.027	0.60943402
SE	C2orf74	+	chr2:61162841-61162955	NA	-0.143	0	-0.016	1
SE	HEATR6	-	chr17:60073745-60073886	NA	-0.144	0.000218129136	0.006	1
SE	SLC25A40	-	chr7:87860071-87860169	NA	-0.144	0.02566581821	-0.006	1

SE	CARD8	-	chr19:48218870-48219012	NA	-0.144	0.006207750042	-0.041	0.5131149699
SE	PRUNE	+	chr1:151024610-151024795	NA	-0.144	0.02759566031	-0.01	1
SE	FAM200B	+	chr4:15684885-15684959	NA	-0.144	0.009167109226	-0.003	1
SE	ABCD4	-	chr14:74299547-74299675	NA	-0.145	0.001061603759	0.004	1
SE	MACF1	+	chr1:39465094-39465112	NA	-0.145	5.30E-07	-0.008	1
SE	ZGRF1	-	chr4:112623816-112623876	NA	-0.145	0.008185219315	-0.096	1
SE	NUDT22	+	chr11:64229849-64230070	NA	-0.145	0.000222018372	-0.039	0.007436051979
SE	C5orf45	-	chr5:179851242-179851424	NA	-0.145	9.74E-05	0.004	0.5676840759
SE	KCNAB3	-	chr17:7925096-7925183	NA	-0.145	0.003016887814	-0.01	1
SE	ARHGEF12	+	chr11:120429711-120429831	NA	-0.145	1.69E-05	-0.021	1
SE	SOX9-AS1	-	chr17:72074548-72076733	NA	-0.145	0.0002651228345	-0.027	1
SE	VEZT	+	chr12:95256549-95256622	NA	-0.146	0.04376813577	-0.025	1
SE	FAM35A	+	chr10:87175888-87176095	NA	-0.146	0.00447617254	-0.045	1
SE	ZBTB38	+	chr3:141438242-141438365	NA	-0.146	0.04507777634	-0.035	1
SE	ZNF506	-	chr19:19793833-19795660	NA	-0.147	0.001824574982	-0.001	1
SE	ATP6V1B1	+	chr2:70963161-70963312	NA	-0.147	0.001312492684	-0.061	1
SE	TRIM5	-	chr11:5665980-5666081	NA	-0.147	9.07E-05	-0.016	1
SE	TET2	+	chr4:105241338-105241429	NA	-0.147	0.005143745186	0.001	1
SE	DLG3	+	chrX:70497179-70497221	NA	-0.148	0.006943205476	0	1
SE	YAP1	+	chr11:102209516-102209564	NA	-0.148	0.001899552138	-0.004	1
SE	SLC50A1	+	chr1:155137578-155137722	NA	-0.148	0.0006659499029	-0.032	0.3558783218
SE	TPT1-AS1	+	chr13:45390757-45390902	NA	-0.148	1.99E-05	-0.042	1
SE	IQCB1	-	chr3:121799195-121799374	NA	-0.148	1.16E-05	-0.023	1
SE	ZDHHC17	+	chr12:76821057-76821167	NA	-0.148	0.002782691887	-0.003	1
SE	SH3YL1	-	chr2:256206-256690	NA	-0.149	0.0128107561	0.035	0.3725291074
SE	LETMD1	+	chr12:51058074-51058128	NA	-0.149	0.0290614872	0.007	1
SE	MACF1	+	chr1:39427960-39428287	NA	-0.149	1.08E-08	-0.139	0
SE	TMEM63B	+	chr6:44135327-44135366	NA	-0.149	2.00E-05	-0.244	0
SE	C5orf22	+	chr5:31548489-31548687	NA	-0.15	9.78E-06	0.001	1
SE	ULBP2	+	chr6:149947319-149947451	NA	-0.15	0.005873187661	-0.067	0.4059025373
SE	ZSWIM7	-	chr17:15986799-15986886	NA	-0.15	0.003676103307	0.006	0.2490285615
SE	BRD9	-	chr5:868606-868804	NA	-0.151	0	0.002	1
SE	ST3GAL6	+	chr3:98756368-98756540	NA	-0.151	0.02805536072	0.051	1
SE	RPAIN	+	chr17:5425970-5426299	NA	-0.151	0.00352321067	-0.074	1.17E-05
SE	CRIM1	+	chr2:36537351-36537546	NA	-0.151	1.02E-08	0.011	1
SE	RHNO1	+	chr12:2885282-2885492	NA	-0.151	0.0005058911378	-0.031	0.04539760873
SE	C11orf80	+	chr11:66822563-66822674	NA	-0.151	0.02626010702	-0.07	1
SE	DHODH	+	chr16:72021123-72021311	NA	-0.152	0.00832100594	-0.006	1
SE	MPDZ	-	chr9:13115247-13115334	NA	-0.152	0.0002389659815	-0.004	1
SE	PRKAB1	+	chr12:119671494-119671668	NA	-0.152	0.007309215227	-0.006	1
SE	ACOX1	-	chr17:75973624-75973785	NA	-0.152	0.04320406396	0.01	0.03035785603
SE	ZNF611	-	chr19:52711206-52711304	NA	-0.152	0.02423789603	0.019	1
SE	FIS1	-	chr7:101244006-101244139	NA	-0.152	0	-0.018	3.15E-14
SE	CLEC2D	+	chr12:9692827-9693948	NA	-0.153	0.0009874780662	-0.004	1
SE	BLOC1S6	+	chr15:45592134-45592276	NA	-0.153	0.0001872045859	-0.074	2.62E-11
SE	ZNF680	-	chr7:64560945-64561273	NA	-0.153	0.04800831293	0.003	1
SE	ZC3H14	+	chr14:88601923-88602083	NA	-0.154	0.04519826484	-0.002	1
SE	INTS4	-	chr11:77910969-77911152	NA	-0.154	0.0009230389767	0.012	0.00310164235
SE	GUF1	+	chr4:44690716-44690860	NA	-0.155	0.04593373253	0.019	1
SE	SPRY1	+	chr4:123397610-123397856	NA	-0.155	0.0008703111406	-0.013	1
SE	POLL	-	chr10:101584601-101584919	NA	-0.155	0.0001700015266	-0.006	1
SE	NARFL	-	chr16:740021-740103	NA	-0.156	6.49E-08	0.007	1
SE	CENPK	-	chr5:65551105-65551242	NA	-0.156	0	-0.01	1
SE	LYPLAL1	+	chr1:219193081-219193251	NA	-0.156	0.001062493224	0.006	1
SE	ZNF260	-	chr19:36525154-36525373	NA	-0.156	0.001648135725	0.005	1
SE	ATG10	+	chr5:82164398-82164537	NA	-0.157	0.01125548831	-0.05	0.005542284501
SE	THAP5	-	chr7:108565829-108566022	NA	-0.157	0.0005452233171	-0.036	0.007207114976
SE	UROS	-	chr10:125789282-125789528	NA	-0.157	0.01755232888	0.003	1
SE	STK40	-	chr1:36367851-36368084	NA	-0.157	0.007647461604	-0.039	0.0001717830389
SE	PATZ1	-	chr22:31328786-31328859	NA	-0.158	0.001028859064	-0.087	9.07E-11
SE	ASCC2	-	chr22:29825621-29825780	NA	-0.158	0.0005291269653	-0.017	1
SE	GRB10	-	chr7:50732271-50732368	NA	-0.158	0.002538626055	0.056	1
SE	FRG1	+	chr4:189955036-189955151	NA	-0.158	1.68E-08	0.02	0.7399231888

SE	DIEXF	+	chr1:209842582-209842695	NA	-0.158	0.02912579184	-0.029	1
SE	PTPRA	+	chr20:2947981-2948024	NA	-0.158	0.004696244476	-0.033	0.970570909
SE	TRPC1	+	chr3:142791018-142791158	NA	-0.158	0.002527404038	-0.031	1
SE	FANCA	-	chr16:89758576-89758705	NA	-0.158	0.03898426062	-0.005	1
SE	RFFL	-	chr17:35021370-35021781	NA	-0.159	8.79E-05	0.031	0.5929753256
SE	MYH14	+	chr19:50224153-50224177	NA	-0.159	0.003134453167	0.054	1
SE	CHURC1-FN	+	chr14:64926006-64926080	NA	-0.159	8.36E-08	0.001	1
SE	MGEA5	-	chr10:101793912-101793998	NA	-0.159	4.00E-13	-0.022	0.0008377962418
SE	ZHX3	-	chr20:41213733-41213899	NA	-0.16	4.68E-06	-0.007	1
SE	KBTBD3	-	chr11:106076506-106076707	NA	-0.16	0.007742020846	0.01	1
SE	MAP4K4	+	chr2:101871493-101871685	NA	-0.161	7.79E-08	-0.071	4.82E-06
SE	MBD1	-	chr18:50273333-50273426	NA	-0.161	0.01539580262	-0.094	8.44E-06
SE	RAB26	+	chr16:2151129-2151315	NA	-0.161	0.002964538658	-0.008	1
SE	BRPF3	+	chr6:36225266-36225364	NA	-0.162	0.03182618393	-0.031	1
SE	LPIN1	+	chr2:11776085-11776193	NA	-0.162	0.0003811031107	0.025	1
SE	APOM	+	chr6:31656471-31656626	NA	-0.162	0.0009638969816	-0.003	1
SE	SLC4A7	-	chr3:27409355-27409530	NA	-0.163	2.31E-08	-0.063	1
SE	SLC35D2	-	chr9:96336716-96336784	NA	-0.163	0.0005475375873	-0.004	1
SE	FIP1L1	+	chr4:53391429-53391498	NA	-0.163	0.004672475626	0.04	0.5459772561
SE	FAM120C	-	chrX:54087754-54087964	NA	-0.163	0.002895587267	0.016	1
SE	ZC3H14	+	chr14:88596733-88596808	NA	-0.164	0.002711949392	-0.03	1
SE	ALMS1	+	chr2:73550266-73550437	NA	-0.164	0.0009874780662	-0.029	1
SE	RPAIN	+	chr17:5425970-5426082	NA	-0.164	0.003868841766	-0.073	0.0003832155032
SE	C11orf74	+	chr11:36636050-36636117	NA	-0.164	0.000648428039	0.02	1
SE	LPCAT4	-	chr15:34361399-34361532	NA	-0.164	0.0001905212064	-0.037	5.01E-05
SE	UBE2F	+	chr2:237987962-237987992	NA	-0.164	0.002623975802	-0.014	1
SE	TMEM222	+	chr1:27330984-27331137	NA	-0.165	0.001213990935	-0.004	1
SE	RP11-87H9.	+	chr9:40993252-40993434	NA	-0.165	0.006454968809	0.046	1
SE	NDUFAF7	+	chr2:37242634-37242693	NA	-0.166	0.01724334818	0.029	1
SE	BCLAF1	-	chr6:136269140-136269303	NA	-0.166	3.04E-05	0.01	0.1681799919
SE	MEF2A	+	chr15:99620890-99621080	NA	-0.166	0.03836748222	-0.058	1
SE	TRDMT1	-	chr10:17161482-17161548	NA	-0.166	0.01480028911	-0.024	1
SE	ESYT2	-	chr7:158752780-158752843	NA	-0.166	0	-0.025	3.10E-07
SE	RPAIN	+	chr17:5425970-5428211	NA	-0.166	0.0001203924305	-0.044	1.16E-05
SE	NR2C2AP	-	chr19:19202469-19202575	NA	-0.166	3.85E-05	-0.105	4.87E-10
SE	RANGAP1	-	chr22:41284900-41285153	NA	-0.167	0.002089000179	-0.001	1
SE	LIG4	-	chr13:108214537-108214610	NA	-0.167	0.0005007711641	0.007	1
SE	ALOX12-AS	-	chr17:7010058-7010137	NA	-0.167	0.005410559041	-0.008	1
SE	TTC38	+	chr22:46281970-46282151	NA	-0.168	0.008685505951	0.002	1
SE	DCUN1D4	+	chr4:51908919-51908975	NA	-0.168	0.0005258881182	0.026	1
SE	ABCD4	-	chr14:74297638-74298069	NA	-0.168	0.0005076552538	0.019	1
SE	LYPLAL1	+	chr1:219193129-219193251	NA	-0.168	0.001177240212	0.002	1
SE	KIAA0195	+	chr17:75492238-75492337	NA	-0.168	0.007872163172	-0.01	1
SE	LMAN2L	-	chr2:96737136-96737217	NA	-0.169	1.72E-06	0.008	1
SE	SETD5	+	chr3:9434044-9434181	NA	-0.169	0.03496582331	0.066	1
SE	IQCB1	-	chr3:121828469-121828632	NA	-0.169	3.04E-10	-0.01	1
SE	ACAT1	+	chr11:108129102-108129201	NA	-0.17	0.04997449951	0.045	1
SE	PEX1	-	chr7:92509328-92509411	NA	-0.17	0.001359664745	-0.08	0.03926674578
SE	ITGB3BP	-	chr1:63510064-63510181	NA	-0.17	0	-0.002	1
SE	NCOA1	+	chr2:24768078-24768135	NA	-0.171	0.01318176908	-0.031	1
SE	COA1	-	chr7:43650492-43650712	NA	-0.171	0	-0.041	0
SE	ORC2	-	chr2:200926767-200926900	NA	-0.171	0.0003867219596	-0.005	1
SE	ATPAF1	-	chr1:46645160-46645256	NA	-0.171	0.0007654461304	-0.019	1
SE	FAM192A	-	chr16:57175538-57175700	NA	-0.171	0.02184055867	0.016	1
SE	RP11-156P1	-	chr17:47033277-47033381	NA	-0.171	0.02472987573	-0.099	1
SE	YEATS4	+	chr12:69365789-69365884	NA	-0.172	0.0001418200435	0.005	1
SE	DNM2	+	chr19:10808568-10808580	NA	-0.173	6.70E-09	-0.125	3.49E-05
SE	CNTRL	+	chr9:121171407-121171548	NA	-0.173	0.001925312558	-0.017	1
SE	FBXO38	+	chr5:148427212-148427722	NA	-0.173	0.00170797872	0.099	1.15E-07
SE	ACSF3	+	chr16:89114338-89114487	NA	-0.173	0.03184982237	-0.074	2.35E-08
SE	CEP63	+	chr3:134547334-134547472	NA	-0.173	6.22E-05	0.025	0.1996070004
SE	AC091729.9	+	chr7:1162351-1162543	NA	-0.173	0.04292658489	-0.073	1
SE	PVT1	+	chr8:127983903-127984204	NA	-0.173	1.02E-05	0.007	1

SE	POC1A	-	chr3:52096568-52096712	NA	-0.174	0.002778487747	-0.044	1
SE	CFAP44	-	chr3:113333405-113333583	NA	-0.174	0.02483827348	-0.048	1
SE	IGF2BP2	-	chr3:185672540-185672669	NA	-0.175	0.0001714835135	-0.115	1
SE	ORC2	-	chr2:200925835-200925932	NA	-0.175	0.0003271679084	-0.003	1
SE	TTC3	+	chr21:37094004-37094090	NA	-0.175	1.50E-06	-0.003	1
SE	PATZ1	-	chr22:31328786-31328924	NA	-0.176	6.08E-05	-0.059	1.94E-06
SE	SETX	-	chr9:132269402-132269489	NA	-0.176	0.0006607809519	0	1
SE	FBXO38	+	chr5:148427212-148427947	NA	-0.176	0.000531989971	0.098	2.29E-08
SE	BLOC1S5	-	chr6:8027206-8027399	NA	-0.176	0.02561488547	-0.021	1
SE	DHX33	-	chr17:5463528-5463689	NA	-0.177	3.95E-06	-0.064	0.05454204889
SE	DNAJC13	+	chr3:132484362-132484493	NA	-0.177	1.90E-07	-0.002	1
SE	SLC20A2	-	chr8:42535171-42535335	NA	-0.177	0.04253479721	0.069	0.5310320296
SE	TNRC18	-	chr7:5361593-5361722	NA	-0.177	0.0001631757984	-0.114	1.57E-09
SE	ZNF655	+	chr7:99563193-99563266	NA	-0.177	0.03398103924	0.032	0.0006725046737
SE	IGF2BP3	-	chr7:23361533-23361597	NA	-0.178	0.0001511590587	-0.022	1
SE	GAPVD1	+	chr9:125335171-125335252	NA	-0.178	0.001005069542	0.038	0.007320527099
SE	TCF25	+	chr16:89878460-89878627	NA	-0.179	0	0.001	0.3228993575
SE	C11orf74	+	chr11:36633283-36633438	NA	-0.18	0.001232514114	0.021	1
SE	IQCB1	-	chr3:121795456-121795566	NA	-0.18	6.62E-06	-0.03	1
SE	PARPBP	+	chr12:102151569-102151800	NA	-0.18	2.79E-09	0.032	1
SE	MTMR9LP	-	chr1:32234035-32234301	NA	-0.18	0.04339536956	0.051	1
SE	ERLEC1	+	chr2:53808298-53808460	NA	-0.181	4.53E-11	0.027	0.1371881116
SE	SBF2	-	chr11:9828125-9828221	NA	-0.181	0.001230254427	-0.002	1
SE	HNRNPD	-	chr4:82356536-82356683	NA	-0.181	1.83E-06	0.001	1
SE	ZNF468	-	chr19:52853871-52854107	NA	-0.181	0.01962932789	0.03	1
SE	TPRKB	-	chr2:73734527-73734588	NA	-0.182	0.0003277096277	0.006	1
SE	C5orf45	-	chr5:179853196-179853276	NA	-0.182	0.0005585912991	0.016	0.03078998763
SE	CXorf23	-	chrX:19935808-19935898	NA	-0.182	0.0007224857652	-0.004	1
SE	PVT1	+	chr8:128099369-128099573	NA	-0.182	2.16E-05	-0.007	1
SE	MON2	+	chr12:62470700-62470778	NA	-0.183	2.20E-05	0.058	1
SE	DLG1	-	chr3:197161639-197161738	NA	-0.183	0.01173470505	-0.024	1
SE	PLGRKT	-	chr9:5381862-5382031	NA	-0.183	0.001850489502	-0.115	1
SE	NQO2	+	chr6:3004494-3004651	NA	-0.183	2.89E-14	0.014	0.8136727638
SE	RBCK1	+	chr20:417219-417342	NA	-0.183	7.29E-07	0	1
SE	SEC31A	-	chr4:82861630-82861708	NA	-0.183	2.27E-08	-0.004	1
SE	METTL6	-	chr3:15414729-15414914	NA	-0.183	0.01601132017	0.083	0.0001102242049
SE	AGPAT4	-	chr6:161165576-161165713	NA	-0.184	1.73E-06	0.007	0.004403524414
SE	SNX14	-	chr6:85565331-85565419	NA	-0.184	0.001898049618	0.037	1
SE	MFF	+	chr2:227328677-227330846	NA	-0.184	0.008685505951	0.09	0.002578297385
SE	ZSWIM7	-	chr17:15986799-15986892	NA	-0.184	0.0004348505466	0.011	0.0647562408
SE	APOPT1	+	chr14:103579370-103579491	NA	-0.184	0.00718773629	-0.082	4.33E-05
SE	FAM114A2	-	chr5:154038292-154038434	NA	-0.185	0.0001340157811	-0.01	1
SE	DCAF10	+	chr9:37842088-37842286	NA	-0.185	4.77E-06	0.013	1
SE	ATG4D	+	chr19:10546838-10547253	NA	-0.185	0.009304957146	-0.046	1
SE	DNAJC14	-	chr12:55829488-55829636	NA	-0.185	0.0009943830497	0.088	0.02081818664
SE	HNRNPDL	-	chr4:82425562-82425667	NA	-0.185	3.12E-07	0.003	1
SE	RP11-421L2	+	chr1:101083460-101083574	NA	-0.185	0.001013602768	-0.114	0.0001433071412
SE	DNAJC5	+	chr20:63931108-63931182	NA	-0.186	0.04246695346	-0.006	1
SE	PLD3	+	chr19:40365661-40365930	NA	-0.186	0.0290614872	-0.166	7.56E-13
SE	ACAD10	+	chr12:111729805-111729956	NA	-0.186	0.04957365061	-0.046	1
SE	TBC1D5	-	chr3:17428449-17428519	NA	-0.186	6.16E-10	-0.028	1
SE	RGS5	-	chr1:163248529-163248598	NA	-0.186	0.03328327983	-0.041	1
SE	RPS27	+	chr1:153990913-153991034	NA	-0.186	0.04298664594	0.003	3.43E-11
SE	GMIP	-	chr19:19634506-19634703	NA	-0.187	0.02076062402	-0.01	0.8869189082
SE	TJP2	+	chr9:69249374-69249485	NA	-0.187	0.005796639247	-0.193	5.22E-13
SE	ZC3H13	-	chr13:46051931-46051989	NA	-0.187	0.01151561822	0.053	1
SE	SLC35B3	-	chr6:8417401-8417494	NA	-0.187	0.00405415197	-0.04	2.87E-09
SE	POLL	-	chr10:101584601-101584919	NA	-0.187	4.65E-05	-0.058	0.009125994487
SE	RNASEH1-A	+	chr2:3559447-3559729	NA	-0.187	0.0001717190751	-0.018	1
SE	CDKL3	-	chr5:134308573-134308727	NA	-0.188	0.0127687169	0.024	1
SE	UFD1L	-	chr22:19451877-19452000	NA	-0.188	0.04996473423	0.005	1
SE	IFT43	+	chr14:76076596-76076691	NA	-0.188	0.01764911282	-0.115	1
SE	IGF2BP3	-	chr7:23361533-23361597	NA	-0.188	3.26E-05	0.025	1

SE	SYTL2	-	chr11:85711112-85711232	NA	-0.188	0.04030865465	-0.046	1
SE	ZNF343	-	chr20:2486640-2486763	NA	-0.189	0.01973132719	-0.152	0.04312643186
SE	RBFOX2	-	chr22:35836319-35836439	NA	-0.189	1.49E-07	-0.024	1
SE	MFF	+	chr2:227347225-227347384	NA	-0.189	0.0008205256852	-0.031	0.01051332156
SE	THTPA	+	chr14:23556742-23557304	NA	-0.189	0.0007750139882	-0.086	0.0001347111507
SE	NCOA2	-	chr8:70144641-70144848	NA	-0.19	0.0410268245	0.029	1
SE	SLC16A4	-	chr1:110378852-110379356	NA	-0.19	0.0004009114888	0.044	0.002967562341
SE	TRIQK	-	chr8:92921502-92921660	NA	-0.19	0.00409329242	-0.065	7.38E-05
SE	SNX19	-	chr11:130880621-130880806	NA	-0.191	7.19E-09	0.004	1
SE	SLC47A1	+	chr17:19571477-19571572	NA	-0.191	0.001141438719	0.078	1
SE	FAM227B	-	chr15:49589775-49590007	NA	-0.191	0.002064161024	0.008	1
SE	NAA16	+	chr13:41336649-41336756	NA	-0.191	0.000130332972	-0.011	1
SE	TLE1	-	chr9:81652183-81652288	NA	-0.191	0.01278775258	-0.006	2.29E-06
SE	SHARPIN	-	chr8:144099080-144099205	NA	-0.192	0.01419024747	-0.065	6.52E-08
SE	DDR1	+	chr6:30895403-30895514	NA	-0.192	0.01899892488	-0.067	5.99E-09
SE	TTLL3	+	chr3:9833103-9833273	NA	-0.192	0.0275139992	-0.029	1
SE	ABHD11	-	chr7:73737220-73737360	NA	-0.193	0.007980648665	-0.114	0.005031905239
SE	LRRCC1	+	chr8:85123412-85123606	NA	-0.193	1.49E-05	-0.033	1
SE	AMMECR1L	-	chr2:127871248-127871359	NA	-0.193	0.02137621621	-0.082	1
SE	LAMP2	-	chrX:120439075-120439293	NA	-0.194	0.000310718969	0.054	0.005295519913
SE	SEPSECS	-	chr4:25159627-25159764	NA	-0.194	0.006939518803	-0.037	1
SE	RHOT1	+	chr17:32209349-32209445	NA	-0.194	0.01725573761	-0.032	0.1023117668
SE	RABEPK	+	chr9:125220538-125220700	NA	-0.194	3.05E-07	-0.034	0.004062852733
SE	EME1	+	chr17:50378594-50378681	NA	-0.194	5.20E-09	0.019	1
SE	UBE2F	+	chr2:237994743-237994809	NA	-0.194	0.001936623701	-0.03	1
SE	CDC14B	-	chr9:96551795-96551872	NA	-0.195	0.0005340109262	-0.041	1
SE	CCDC91	+	chr12:28225794-28225861	NA	-0.195	0.02409243924	-0.04	1
SE	ATRX	-	chrX:77595711-77595785	NA	-0.196	2.07E-11	0.013	1
SE	AFMID	+	chr17:78204827-78204900	NA	-0.196	0.01185604305	-0.023	1
SE	C2orf76	-	chr2:119321153-119321204	NA	-0.196	4.71E-09	0.049	0.1967951618
SE	TRIM16	-	chr17:15682853-15682964	NA	-0.196	0.002384579214	-0.16	1
SE	MLLT10	+	chr10:21617111-21617207	NA	-0.197	1.81E-08	-0.027	1
SE	GRB10	-	chr7:50710874-50710990	NA	-0.197	0.00375709255	0.02	1
SE	CLOCK	-	chr4:55486392-55486549	NA	-0.197	0.002404395753	-0.068	1
SE	TACC2	+	chr10:122143571-122143706	NA	-0.197	0.003050330045	0.025	1
SE	NFIC	+	chr19:3453762-3453916	NA	-0.197	0.02539293364	-0.053	0.06164307927
SE	AMACR	-	chr5:33994077-33994120	NA	-0.197	6.29E-08	-0.004	1
SE	C17orf80	+	chr17:73242243-73242351	NA	-0.198	0.03616841176	-0.026	1
SE	DNAJC19	-	chr3:180986997-180987022	NA	-0.198	0.009511077212	0.004	1
SE	RPL17	-	chr18:49491745-49491833	NA	-0.198	1.13E-05	0.018	6.40E-07
SE	KMT2E	+	chr7:105110476-105110602	NA	-0.199	3.54E-09	-0.1	1.12E-09
SE	C16orf93	-	chr16:30759341-30759724	NA	-0.199	0.02284834663	-0.031	0.3996345431
SE	MATR3	+	chr5:139279934-139280058	NA	-0.199	0.006055130299	-0.04	1
SE	CAMTA1	+	chr1:6820180-6820250	NA	-0.2	0.0001018934292	-0.002	1
SE	LCORL	-	chr4:17886067-17886161	NA	-0.2	0.03026946122	0.033	1
SE	TMEM161B-	+	chr5:88285740-88285851	NA	-0.2	0.02616662861	0.01	1
SE	SAMD4A	+	chr14:54737023-54737287	NA	-0.201	0.000707373342	-0.046	1
SE	MYNN	+	chr3:169774030-169774097	NA	-0.201	0.00559327814	-0.023	1
SE	TCF25	+	chr16:89908965-89909083	NA	-0.201	7.43E-06	-0.008	0.3340085523
SE	DNAAF2	-	chr14:49628011-49628155	NA	-0.201	0.000992479765	-0.03	1
SE	BPTF	+	chr17:67963332-67963506	NA	-0.201	0.02039831761	0.025	1
SE	DPY19L3	+	chr19:32467473-32467626	NA	-0.201	0.009323277483	-0.003	0.8430130572
SE	PSMA3-AS1	-	chr14:58293765-58295811	NA	-0.201	4.24E-05	-0.01	0.4096347567
SE	SCMH1	-	chr1:41151613-41151684	NA	-0.202	0.002354333127	-0.105	0.05240945867
SE	SCMH1	-	chr1:41152598-41152741	NA	-0.202	0.03233185005	-0.03	1
SE	GLT8D1	-	chr3:52704723-52704952	NA	-0.203	0.00793029098	0.078	5.35E-05
SE	OFD1	+	chrX:13751248-13751368	NA	-0.203	7.43E-06	0.045	1
SE	E2F6	-	chr2:11455341-11455474	NA	-0.203	0.000284447168	0	1
SE	ARNTL2	+	chr12:27376345-27376387	NA	-0.204	0.005462739449	0.046	1
SE	PVR	+	chr19:44658741-44658900	NA	-0.204	0.0003788723075	0.015	1
SE	RPP40	-	chr6:4998715-4998841	NA	-0.204	6.11E-06	-0.008	1
SE	MBD5	+	chr2:148233244-148233395	NA	-0.204	6.62E-06	-0.003	1
SE	DEPDC1	-	chr1:68482045-68482897	NA	-0.205	0.01524058518	0.213	7.38E-06

SE	PRPSAP2	+	chr17:18857783-18857853	NA	-0.205	0.0006885416923	-0.024	1
SE	CASC4	+	chr15:44402886-44403054	NA	-0.205	1.92E-08	0.03	1
SE	STRN3	-	chr14:30929200-30929311	NA	-0.205	0.0001232390093	0.029	1
SE	SMUG1	-	chr12:54187818-54187905	NA	-0.206	0.01517451728	0.003	1
SE	HUWE1	-	chrX:53625785-53626034	NA	-0.207	0.01157165107	0.073	1
SE	PPCDC	+	chr15:75044385-75044514	NA	-0.207	0.01663502083	0.018	0.0001170558574
SE	ZNF180	-	chr19:44485035-44485157	NA	-0.207	0.04841923256	0.027	1
SE	ZNF155	+	chr19:43990023-43990112	NA	-0.207	0.0001224929477	-0.019	1
SE	RBM26-AS1	+	chr13:79417834-79417916	NA	-0.207	0.0179359004	-0.125	6.15E-14
SE	RP11-216L1	+	chr9:136809254-136809429	NA	-0.207	0.01082847443	-0.143	1
SE	SEPT6	-	chrX:119625334-119625396	NA	-0.208	0.0005682274055	0.016	0.3152060732
SE	AHSA2	+	chr2:61183546-61183662	NA	-0.209	1.32E-10	-0.004	1
SE	ZNF625-ZNI	-	chr19:12145257-12146224	NA	-0.209	0.01755228438	-0.012	1
SE	TARBP1	-	chr1:234463836-234463934	NA	-0.21	0.02650525741	-0.168	0
SE	CLEC2D	+	chr12:9693415-9693948	NA	-0.21	0.0004320131833	0.023	1
SE	LTBP4	+	chr19:40616888-40617042	NA	-0.21	0.03749535962	0.008	1
SE	NAT9	-	chr17:74773865-74774055	NA	-0.21	0.002803673059	-0.01	1
SE	IAH1	+	chr2:9475986-9476039	NA	-0.21	0.003840014914	-0.004	1
SE	ATPAF2	-	chr17:18021421-18021663	NA	-0.21	0.002988281976	0.003	1
SE	TPGS2	-	chr18:36805373-36805397	NA	-0.211	9.62E-09	-0.065	5.84E-08
SE	GPR180	+	chr13:94619149-94619330	NA	-0.211	0.01825893441	0.023	1
SE	LRRK2	+	chr12:40313971-40314173	NA	-0.211	0.0001763959562	-0.055	1
SE	FUZ	-	chr19:49812446-49812685	NA	-0.212	0.001680905192	-0.069	1
SE	HM13	+	chr20:31568077-31568280	NA	-0.212	0.002292667923	-0.042	1
SE	HAUS2	+	chr15:42560781-42560870	NA	-0.212	6.71E-05	0.055	1
SE	UBA7	-	chr3:49809389-49809464	NA	-0.212	0.03434244633	-0.018	1
SE	ZNF814	-	chr19:57885882-57885969	NA	-0.212	0.001014446317	-0.009	1
SE	ZCCHC8	-	chr12:122483278-122483344	NA	-0.213	0.0247351972	0.002	1
SE	AFTPH	+	chr2:64579485-64579546	NA	-0.213	0.002066660603	-0.057	0.1318557848
SE	THUMPD2	-	chr2:39768900-39769093	NA	-0.213	0.04630611355	0.012	1
SE	TRPC1	+	chr3:142743484-142743586	NA	-0.213	0.04709255475	0.022	1
SE	ZNF468	-	chr19:52853871-52854126	NA	-0.214	0.01228624385	0.054	1
SE	SNX10	+	chr7:26357006-26357127	NA	-0.215	0.0001989304633	-0.005	1
SE	SEC24B	+	chr4:109481676-109481781	NA	-0.216	0.01392337102	0.014	1
SE	UBE3A	-	chr15:25408619-25408684	NA	-0.217	4.55E-05	0.024	1
SE	PREPL	-	chr2:44359496-44359750	NA	-0.217	0.01939041588	0.037	1
SE	ANAPC16	+	chr10:72223887-72224056	NA	-0.218	0	-0.021	0.001566799978
SE	ZNF655	+	chr7:99562348-99562494	NA	-0.219	0.02239654989	-0.09	0.3484447737
SE	WBP1	+	chr2:74459477-74459562	NA	-0.219	0.02421023616	0.041	1
SE	MVK	+	chr12:109581394-109581550	NA	-0.22	0.0001835601539	-0.083	0.002006938341
SE	DDIAS	+	chr11:82913282-82913386	NA	-0.22	9.98E-05	-0.026	1
SE	TTLL11	-	chr9:122031722-122031856	NA	-0.221	0.03455480269	-0.047	1
SE	PCYT2	-	chr17:81907203-81907257	NA	-0.221	2.89E-14	-0.014	0.03479523062
SE	MRPL33	+	chr2:27774423-27774530	NA	-0.221	6.94E-09	-0.038	0.008284518975
SE	FAM86DP	-	chr3:75427426-75427692	NA	-0.221	8.51E-06	-0.015	1
SE	CSPP1	+	chr8:67172768-67172973	NA	-0.222	2.44E-07	0.028	3.92E-06
SE	TMTC4	-	chr13:100670359-100670569	NA	-0.222	1.46E-06	0.009	1
SE	SBF2	-	chr11:9992414-9992543	NA	-0.223	9.19E-08	0.008	1
SE	IMMP1L	-	chr11:31456259-31456386	NA	-0.223	3.19E-06	-0.003	1
SE	SLC11A2	-	chr12:51006173-51006271	NA	-0.224	0.01585603976	-0.08	1
SE	ZBTB24	-	chr6:109476762-109476930	NA	-0.224	0.0423681903	-0.118	1.58E-10
SE	LL22NC03-8	+	chr22:22308696-22308819	NA	-0.224	6.91E-05	-0.048	1
SE	CARD8	-	chr19:48206383-48206520	NA	-0.225	0.008844746833	-0.061	1
SE	FEZ2	-	chr2:36560784-36560865	NA	-0.225	0	0.197	0
SE	TCTN1	+	chr12:110644966-110645129	NA	-0.225	0.0008678622588	-0.05	2.93E-05
SE	KCTD17	+	chr22:37060822-37060922	NA	-0.226	0.00289981091	-0.153	1.32E-06
SE	KANSL2	-	chr12:48679035-48679238	NA	-0.226	0.005663637832	-0.045	1
SE	AURKA	-	chr20:56390555-56390665	NA	-0.227	0.008722123107	-0.063	1
SE	LPHN2	+	chr1:81986900-81987029	NA	-0.227	3.49E-10	0.057	3.85E-11
SE	KCTD17	+	chr22:37061538-37061629	NA	-0.228	0.01385980222	-0.128	4.10E-06
SE	MANBA	-	chr4:102753822-102753995	NA	-0.228	0.0001816263906	0.102	1
SE	CLHC1	-	chr2:55208625-55208710	NA	-0.228	0.008763410401	-0.025	1
SE	GINS3	+	chr16:58395101-58395218	NA	-0.228	0.0001444542877	0.012	1

SE	AC007566.1	+	chr7:92462957-92463026	NA	-0.228	3.06E-06	-0.056	0.03277997034
SE	PVT1	+	chr8:127983903-127984204	NA	-0.228	0.001136224867	-0.089	1
SE	CYP20A1	+	chr2:203272645-203272748	NA	-0.229	0.00918764879	-0.077	0.128834362
SE	C1orf52	-	chr1:85258934-85259061	NA	-0.229	0.01080748181	0.027	1
SE	CRELD1	+	chr3:9943901-9944095	NA	-0.229	0.00516164134	0.015	0.05719650957
SE	ERCC6L2	+	chr9:95967458-95967625	NA	-0.229	0.006683548413	-0.004	0.001909610936
SE	IRAK4	+	chr12:43771219-43771365	NA	-0.229	0.03571655976	-0.131	1
SE	MTIF2	-	chr2:55266380-55266518	NA	-0.23	0	0.008	1
SE	ZC3H14	+	chr14:88602827-88603060	NA	-0.23	6.94E-06	-0.033	1
SE	ALDOA	+	chr16:30065875-30065927	NA	-0.23	1.22E-06	-0.022	1
SE	SEC61A2	+	chr10:12167752-12167893	NA	-0.231	0.002784788117	-0.112	1
SE	CHFR	-	chr12:132869618-132869798	NA	-0.231	0.000242143471	-0.013	1
SE	C11orf30	+	chr11:76535894-76536059	NA	-0.231	1.35E-06	-0.031	1
SE	EIF1AX	-	chrX:20132181-20132263	NA	-0.231	0.02363008235	0.002	1
SE	CD27-AS1	-	chr12:6450473-6450708	NA	-0.232	0.0496313164	-0.045	1
SE	SCMH1	-	chr1:41159724-41159933	NA	-0.234	0.02810714143	-0.105	0.1131855418
SE	CAPRIN2	-	chr12:30720810-30720915	NA	-0.234	0.004393856425	-0.068	0.8406346425
SE	BNIP2	-	chr15:59665349-59665442	NA	-0.234	8.71E-06	0.005	1
SE	CCBL1	-	chr9:128846679-128846859	NA	-0.234	0.04202003307	-0.054	1
SE	MBNL1	+	chr3:152446703-152446757	NA	-0.235	0.04669502336	0.184	4.02E-08
SE	RNF181	+	chr2:85596856-85596931	NA	-0.235	0.01446539393	-0.044	0.0007419914904
SE	ATAT1	+	chr6:30642767-30643011	NA	-0.236	0.01637108017	0.047	5.33E-05
SE	FAM111B	+	chr11:59109539-59109706	NA	-0.236	2.18E-09	-0.032	1
SE	CFAP44	-	chr3:113409105-113409322	NA	-0.236	0.003084990753	-0.103	1
SE	OSER1-AS1	+	chr20:44214887-44215003	NA	-0.236	0.01202849288	0.067	0.07979102748
SE	MTRF1L	-	chr6:152991184-152991321	NA	-0.237	0.01806842543	0.032	1
SE	LIG4	-	chr13:108214537-108214807	NA	-0.237	7.32E-05	0.01	1
SE	PSMA3-AS1	-	chr14:58273978-58274135	NA	-0.237	7.74E-05	0.056	1
SE	ANKHD1	+	chr5:140472238-140472295	NA	-0.238	1.18E-05	0.004	1
SE	TMEM14B	+	chr6:10749621-10749698	NA	-0.238	0.0145521764	-0.057	1
SE	PEX5	+	chr12:7202240-7202351	NA	-0.238	0.003024317095	-0.094	3.12E-07
SE	JAM2	+	chr21:25711366-25711568	NA	-0.238	0	0	1
SE	RP11-421L2	+	chr1:101075394-101075716	NA	-0.238	0.002624894621	-0.026	1
SE	THTPA	+	chr14:23556742-23557034	NA	-0.239	0.001064705924	-0.084	0.0001353553277
SE	TMPO	+	chr12:98544950-98545061	NA	-0.24	0.0008503980827	-0.055	0.144092348
SE	ZEB1	+	chr10:31387123-31387266	NA	-0.24	1.97E-06	-0.103	1
SE	C11orf54	+	chr11:93741962-93742094	NA	-0.24	0.01116962224	0.066	1
SE	THTPA	+	chr14:23556742-23557039	NA	-0.24	0.001013018677	-0.085	0.0001048126062
SE	ZNF592	+	chr15:84778182-84778312	NA	-0.241	0.000908503465	0.005	1
SE	HMGN1	-	chr21:39347378-39347483	NA	-0.241	0.008201186754	0.007	1
SE	IFT80	-	chr3:160366092-160366152	NA	-0.242	0.001172941124	-0.057	0.001087131773
SE	YPEL5	+	chr2:30148244-30148305	NA	-0.242	0	0.006	1
SE	CCDC57	-	chr17:82107401-82107621	NA	-0.242	0.0008632009723	-0.008	1
SE	MYEF2	-	chr15:48151099-48151171	NA	-0.243	2.46E-08	0.048	0.02383342557
SE	XRRA1	-	chr11:74927390-74927488	NA	-0.244	0.003894370242	-0.043	1
SE	LINC00963	+	chr9:129502396-129503139	NA	-0.244	0.02048193694	0.064	0.6082805099
SE	DECR2	+	chr16:405512-405665	NA	-0.244	0.03712636787	0.017	6.96E-06
SE	NFE2L1	+	chr17:48057031-48057121	NA	-0.245	1.71E-08	-0.078	0
SE	CENPI	+	chrX:101099808-101100065	NA	-0.245	1.04E-07	-0.112	1
SE	SLC25A30	-	chr13:45411732-45411938	NA	-0.245	1.54E-05	-0.003	1
SE	SUZ12P1	+	chr17:30743300-30743369	NA	-0.245	4.02E-05	-0.055	0.8659334431
SE	MAP4	-	chr3:47867245-47867338	NA	-0.246	0	-0.129	0
SE	LRRCC1	+	chr8:85115099-85115275	NA	-0.246	2.17E-05	-0.047	0.916041012
SE	CHD3	+	chr17:7907600-7907702	NA	-0.246	6.56E-08	-0.065	0.08178015237
SE	PUM2	-	chr2:20278582-20278819	NA	-0.248	8.29E-06	-0.006	1
SE	PUM2	-	chr2:20331716-20331937	NA	-0.248	8.33E-09	0.005	1
SE	KLC1	+	chr14:103684985-103685036	NA	-0.248	0.01272844939	0.024	0.8524029425
SE	CFAP44	-	chr3:113344515-113344712	NA	-0.248	0.001534015222	0.038	1
SE	BICD1	+	chr12:32338785-32338979	NA	-0.249	0.02450190234	0.043	1
SE	LZTFL1	-	chr3:45837926-45838047	NA	-0.249	0.0008526729915	0	1
SE	IFI27L1	+	chr14:94097491-94097678	NA	-0.249	0.007405164285	0.019	2.87E-07
SE	BCS1L	+	chr2:218660036-218660166	NA	-0.25	0.0001103973794	-0.105	0.001970275672
SE	PPP1R12B	+	chr1:202443086-202443127	NA	-0.25	0.03843822808	-0.16	1

SE	SH3PXD2A	-	chr10:103622469-103622553	NA	-0.25	0.000219648813	-0.131	1
SE	RPAIN	+	chr17:5428070-5428211	NA	-0.25	1.74E-07	-0.096	2.67E-05
SE	TRPC1	+	chr3:142743484-142743586	NA	-0.25	0.005039214886	0.032	1
SE	UBAP2L	+	chr1:154269361-154269412	NA	-0.251	0.0249882848	-0.074	3.44E-08
SE	TIMM23B	+	chr10:49943234-49943358	NA	-0.251	0.03179732384	0.05	1
SE	PHKA1	-	chrX:72620724-72620901	NA	-0.252	4.07E-05	0.002	0.0003193279855
SE	HM13	+	chr20:31568077-31568224	NA	-0.252	8.23E-05	-0.049	1
SE	ALG2	-	chr9:99220978-99221137	NA	-0.252	0.009618423766	0.001	1
SE	TRIM9	-	chr14:51000682-51000828	NA	-0.253	0.0006452214125	-0.027	1
SE	ATP6V1B1	+	chr2:70963161-70963261	NA	-0.253	0.0008122141714	-0.056	1
SE	PFKL	+	chr21:44305799-44305935	NA	-0.253	0.04584038807	-0.031	1
SE	OSBPL6	+	chr2:178230425-178230513	NA	-0.254	0.02472987573	0.023	1
SE	KAT6A	-	chr8:42049619-42049707	NA	-0.254	0.0005347540089	0.17	1
SE	ATAD2	-	chr8:123400742-123401175	NA	-0.254	0.02776636468	-0.018	1
SE	CDKL3	-	chr5:134306608-134306702	NA	-0.255	0.0007711754357	-0.068	1
SE	PRPF4B	+	chr6:4056526-4056688	NA	-0.255	6.63E-13	0.023	3.15E-14
SE	RPS3A	+	chr4:151100974-151101012	NA	-0.255	0.0001633148417	-0.005	0.3892592243
SE	SEPT7P2	-	chr7:45728831-45729026	NA	-0.255	7.82E-11	0.006	1
SE	RABEPK	+	chr9:125213369-125213522	NA	-0.256	2.92E-10	-0.055	1.46E-05
SE	TMPO	+	chr12:98544441-98544537	NA	-0.257	5.59E-14	-0.037	0.4772502517
SE	EBPL	-	chr13:49669776-49669846	NA	-0.257	0.0002342420853	-0.023	0.2089693241
SE	ZEB1	+	chr10:31387123-31387266	NA	-0.257	6.18E-05	-0.129	0
SE	LRP8	-	chr1:53272603-53272642	NA	-0.257	0.01439386568	-0.127	1
SE	KIF23	+	chr15:69440767-69441079	NA	-0.258	3.51E-05	0.129	4.24E-05
SE	ZDHHC24	-	chr11:66526935-66527006	NA	-0.258	0.02660810651	-0.088	1
SE	HEATR6	-	chr17:60073745-60074159	NA	-0.259	1.02E-05	0.028	1
SE	HACE1	-	chr6:104824813-104824907	NA	-0.259	0.00276043376	-0.096	1
SE	MEIS3	-	chr19:47415050-47415101	NA	-0.259	0.02092964191	-0.011	1
SE	CCNYL1	+	chr2:207740654-207740706	NA	-0.26	2.18E-06	-0.029	1
SE	AC138969.4	+	chr16:16335860-16336137	NA	-0.26	0.007599994575	-0.037	1
SE	CHFR	-	chr12:132869618-132869834	NA	-0.261	0.0001382408096	-0.017	1
SE	ALG13	+	chrX:111689086-111689197	NA	-0.261	4.16E-06	-0.091	1
SE	RAC1	+	chr7:6398661-6398718	NA	-0.261	4.82E-06	-0.004	0.5875483802
SE	RP11-849H4	-	chr11:71925932-71926104	NA	-0.261	0.01221869075	-0.062	1
SE	MAZ	+	chr16:29809539-29809764	NA	-0.262	1.98E-06	-0.01	1
SE	NOD1	-	chr7:30437572-30437656	NA	-0.262	2.01E-05	0.051	0.8351505569
SE	RAD1	-	chr5:34913469-34913578	NA	-0.262	0.0008025627237	-0.024	1
SE	RPTOR	+	chr17:80925369-80925480	NA	-0.262	0.04458274527	0.001	1
SE	DNAJC19	-	chr3:180986942-180987022	NA	-0.262	1.04E-06	0.001	1
SE	BRPF3	+	chr6:36225270-36225364	NA	-0.263	0.00709572117	-0.049	5.10E-12
SE	PREPL	-	chr2:44359496-44360038	NA	-0.263	0.000403764681	0.03	1
SE	C2orf76	-	chr2:119340158-119340361	NA	-0.263	0.04431440655	-0.078	0.7458835289
SE	MAP4	-	chr3:47869213-47869327	NA	-0.264	0	-0.152	0
SE	WASF1	-	chr6:110178597-110178742	NA	-0.264	1.70E-05	-0.071	0.04264150054
SE	CLHC1	-	chr2:55181569-55181744	NA	-0.264	0.0002588502674	0.022	1
SE	LCAT	-	chr16:67940736-67940831	NA	-0.264	0.03618639737	0.011	1
SE	ZC3H14	+	chr14:88602827-88603045	NA	-0.265	0.000317236808	-0.038	1
SE	IFI27L1	+	chr14:94097494-94097678	NA	-0.265	0.006289332786	0.023	7.85E-06
SE	CCDC57	-	chr17:82179026-82179189	NA	-0.265	0.04941263439	-0.099	0.0544597215
SE	IMMP1L	-	chr11:31463171-31463305	NA	-0.266	5.79E-06	-0.025	0.01631422618
SE	RP4-769N13	+	chrX:102601879-102601977	NA	-0.266	1.98E-07	0.035	1
SE	ITGB1BP1	-	chr2:9419989-9420100	NA	-0.267	0.001505065008	0.025	1
SE	PIGP	-	chr21:37072144-37072310	NA	-0.267	6.49E-09	-0.007	1
SE	C8orf44	+	chr8:66676744-66676902	NA	-0.267	0.02133016112	0.031	1
SE	LINC00963	+	chr9:129502396-129503139	NA	-0.268	0.03797039936	0.041	0.001474455026
SE	RP11-762I7.	-	chr12:55815886-55815939	NA	-0.268	0.02987985873	-0.001	1
SE	EZH2	-	chr7:148836821-148837000	NA	-0.269	0.000373023368	-0.15	1
SE	KIF21A	-	chr12:39318072-39318201	NA	-0.269	2.19E-06	-0.08	1
SE	ARHGEF10I	+	chr1:17634547-17634562	NA	-0.27	0.02106431358	-0.03	1.35E-10
SE	KLHL42	+	chr12:27797246-27797349	NA	-0.27	2.88E-07	0.05	0.2378188244
SE	PXN	-	chr12:120226838-120227117	NA	-0.27	0.04038676185	0.004	1
SE	REPS1	-	chr6:138926400-138926481	NA	-0.27	0.01380120963	0.093	0.0005225978947
SE	TMBIM6	+	chr12:49749617-49749856	NA	-0.271	0.001910715501	0.018	1

SE	PLSCR1	-	chr3:146528613-146528831	NA		-0.271	0.0004347064404	-0.054	0.0210189815
SE	ZC3H11A	+	chr1:203796292-203796601	NA		-0.272	1.68E-09	-0.025	1
SE	NUMB	-	chr14:73279280-73279424	NA		-0.272	0	0.002	1
SE	TTC8	+	chr14:88841424-88841514	NA		-0.273	0.03451558164	-0.022	1
SE	TCTN2	+	chr12:123694841-123694976	NA		-0.273	5.84E-06	-0.008	1
SE	SLCO4A1-A	-	chr20:62664923-62665497	NA		-0.273	2.23E-06	-0.144	0.1614596839
SE	BDNF-AS	+	chr11:27658240-27658462	NA		-0.274	1.00E-06	-0.036	1
SE	SEPT6	-	chrX:119625334-119625379	NA		-0.275	8.27E-11	-0.023	1
SE	INTS3	+	chr1:153761019-153761109	NA		-0.275	0	-0.009	1
SE	CEP78	+	chr9:78265858-78265906	NA		-0.275	3.30E-08	0.046	0.6253280454
SE	EME1	+	chr17:50378599-50378681	NA		-0.275	5.50E-09	0.013	1
SE	SUCO	+	chr1:172577538-172577559	NA		-0.276	0.0005405865695	0.127	7.27E-05
SE	ZBED3-AS1	+	chr5:77100235-77100391	NA		-0.276	0.009747054499	0.082	1
SE	MIS12	+	chr17:5488195-5488589	NA		-0.277	3.75E-10	-0.001	1
SE	RPE	+	chr2:210009638-210009736	NA		-0.277	0.001384659396	-0.076	9.71E-11
SE	BIRC5	+	chr17:78222827-78222992	NA		-0.278	2.86E-05	0.005	1
SE	HDAC9	+	chr7:18629349-18629481	NA		-0.279	0.0001629656818	0.037	1
SE	ZSCAN32	-	chr16:3390009-3390133	NA		-0.279	0.01658132986	-0.054	1
SE	NFIC	+	chr19:3453762-3453916	NA		-0.281	0.0002392046182	-0.027	1
SE	PDE9A	+	chr21:42731769-42731949	NA		-0.281	0.006781962229	-0.06	0.1118308266
SE	WDFY3	-	chr4:84808333-84808417	NA		-0.281	6.05E-07	-0.025	1
SE	TAF2	-	chr8:119744880-119745036	NA		-0.283	1.62E-08	0.009	1
SE	TCTN1	+	chr12:110644966-110645143	NA		-0.283	0.002401590692	-0.063	0.003957862823
SE	AKAP11	+	chr13:42285985-42286035	NA		-0.284	0.0005294509158	0.011	1
SE	SCRN3	+	chr2:174397335-174397404	NA		-0.284	0.03300582981	0.057	1
SE	PHF8	-	chrX:53993600-53993903	NA		-0.284	8.23E-06	-0.055	0.3846135774
SE	AP1S2	-	chrX:15833443-15833524	NA		-0.284	0.03860979484	0.046	1
SE	RP11-10K16	-	chr4:173166644-173166790	NA		-0.284	0.000342014704	-0.091	0.02088168224
SE	DCUN1D4	+	chr4:51887122-51887244	NA		-0.285	0	0.023	0.2942302704
SE	ITGB1BP1	-	chr2:9419989-9420100	NA		-0.285	0.008497664699	-0.095	0.2003718815
SE	LINC00630	+	chrX:102839800-102839863	NA		-0.285	0.04393571592	-0.011	1
SE	PBRM1	-	chr3:52554723-52554879	NA		-0.286	0.008665320904	-0.132	0.001071403471
SE	SPTAN1	+	chr9:128592982-128593042	NA		-0.286	2.07E-07	-0.039	1
SE	TRIQK	-	chr8:92921502-92921660	NA		-0.286	0.000185566505	-0.016	0.4813644051
SE	UBL7-AS1	+	chr15:74466035-74466130	NA		-0.286	0.00472504306	0.007	1
SE	PLEKHM1	-	chr17:45468208-45468593	NA		-0.287	2.06E-05	-0.016	1
SE	CTD-2651B2	-	chr15:45264626-45264809	NA		-0.287	0.01243842924	0.093	1
SE	NR2F1-AS1	-	chr5:93438702-93438741	NA		-0.288	0.004687272022	0.006	1
SE	SNX14	-	chr6:85567533-85567577	NA		-0.289	0.01711655461	0.08	0.7878538332
SE	DTNB	-	chr2:25455404-25455494	NA		-0.289	0.001751966261	-0.199	0
SE	STK19	+	chr6:31972620-31972919	NA		-0.289	0.00395611319	-0.022	1
SE	PSMA3-AS1	-	chr14:58285590-58285756	NA		-0.289	0.0005255286629	0.03	1
SE	COA1	-	chr7:43665657-43665722	NA		-0.29	1.78E-05	0.025	0.932701491
SE	SLC35F5	-	chr2:113717752-113717848	NA		-0.29	6.95E-06	0.011	1
SE	ZNF283	+	chr19:43827775-43827940	NA		-0.29	2.15E-05	0.048	1
SE	ETV1	-	chr7:13989267-13989464	NA		-0.291	9.08E-05	-0.092	1
SE	DUS4L	+	chr7:107573703-107573883	NA		-0.291	6.90E-05	0.034	1
SE	PCBP1-AS1	-	chr2:70085815-70085903	NA		-0.291	0.0008998411314	0.018	1
SE	TBXAS1	+	chr7:139782487-139782564	NA		-0.293	0.04145970596	-0.032	1
SE	CCNYL1	+	chr2:207733946-207734047	NA		-0.293	0.0004783507939	-0.063	0.2653902291
SE	CCBL1	-	chr9:128847432-128847643	NA		-0.293	1.47E-07	-0.059	1
SE	ZNF678	+	chr1:227646543-227646670	NA		-0.293	0.01382788928	-0.031	1
SE	FAM189B	-	chr1:155251094-155251164	NA		-0.295	0.005179405283	0.056	1
SE	EPB41L2	-	chr6:130876673-130876775	NA		-0.296	5.60E-05	-0.17	1
SE	TRDMT1	-	chr10:17168840-17168917	NA		-0.296	0.007123014945	-0.048	1
SE	SLC50A1	+	chr1:155136827-155136951	NA		-0.296	0.0001103751914	-0.039	0.5783982015
SE	LRRCC1	+	chr8:85109594-85109800	NA		-0.297	0.01949475042	-0.132	1
SE	CHRM3	+	chr1:239826852-239827081	NA		-0.298	0.04103024877	0.157	1
SE	REPS1	-	chr6:138926400-138926478	NA		-0.299	0.01444851823	0.092	0.0001449755893
SE	IMMP1L	-	chr11:31460625-31460714	NA		-0.299	0.01505945115	-0.13	0.005720682925
SE	CLASP1	-	chr2:121445448-121445496	NA		-0.3	4.70E-05	-0.236	1
SE	SLCO4A1	+	chr20:62668011-62668184	NA		-0.3	0	-0.118	1.48E-06
SE	ZBTB21	-	chr21:42002349-42002503	NA		-0.3	0.03885696071	0.002	1

SE	TMEM161B-	+	chr5:88283009-88283086	NA	-0.3	0.01655644229	0.004	1
SE	NBPF11	-	chr1:148124898-148125001	NA	-0.3	0.03825624706	-0.001	1
SE	PRKRIP1	+	chr7:102395976-102396537	NA	-0.301	0.000271826994	0.007	1
SE	EP400NL	+	chr12:132109767-132109832	NA	-0.301	0.0007481903903	0.059	1
SE	ZSCAN23	-	chr6:28435459-28435607	NA	-0.301	0.0009603740643	-0.011	1
SE	SREBF1	-	chr17:17823517-17823607	NA	-0.302	0.008461191506	0	1
SE	CLHC1	-	chr2:55181569-55181744	NA	-0.302	5.10E-06	-0.03	0.1269639185
SE	PHYKPL	-	chr5:178224615-178224729	NA	-0.302	0.04422443977	-0.023	1
SE	CRBN	-	chr3:3175162-3175266	NA	-0.303	2.89E-14	-0.052	0.1757137416
SE	ODF2L	-	chr1:86356443-86356602	NA	-0.303	0.0004028475812	0.046	1
SE	VAPB	+	chr20:58434601-58434705	NA	-0.303	0.004308658414	-0.063	0.08639686555
SE	MUTYH	-	chr1:45332917-45332959	NA	-0.303	0.01134847022	-0.078	0
SE	KIF21A	-	chr12:39318072-39318201	NA	-0.303	2.13E-08	0.012	1
SE	EPB41	+	chr1:29060421-29060484	NA	-0.303	0.01397229194	0.095	0.1710696772
SE	GDAP2	-	chr1:117877775-117877841	NA	-0.303	5.13E-05	-0.015	0.775169044
SE	FAM45A	+	chr10:119105310-119105495	NA	-0.304	0.0001800778006	-0.01	1
SE	PLA2G12A	-	chr4:109718682-109718759	NA	-0.304	1.31E-08	-0.025	0.03889098255
SE	PPP4R1	-	chr18:9562921-9563046	NA	-0.304	0.0007881069043	0.011	1
SE	DLGAP1-AS	+	chr18:3596578-3596675	NA	-0.304	4.07E-06	0.028	1
SE	CAPRIN2	-	chr12:30719078-30720915	NA	-0.305	0.0002400924652	-0.047	1
SE	CLHC1	-	chr2:55228031-55228204	NA	-0.305	0.02539319847	0.074	1
SE	FAHD2A	+	chr2:95410526-95410586	NA	-0.306	0.0211341988	-0.027	0.000765265166
SE	MCAT	-	chr22:43137080-43137298	NA	-0.307	0.0003041334185	-0.02	1
SE	STAG2	+	chrX:124021366-124021431	NA	-0.307	0.0001010478751	-0.159	1
SE	MIATNB	+	chr22:26718139-26718231	NA	-0.307	0.01784243899	-0.016	1
SE	MDM1	-	chr12:68325440-68325575	NA	-0.31	0.01658132986	-0.016	1
SE	IKZF4	+	chr12:56023670-56023764	NA	-0.31	0.02968938387	0.117	1
SE	ZBTB1	+	chr14:64516599-64516751	NA	-0.31	6.58E-06	0.022	1
SE	MATR3	+	chr5:139293452-139293575	NA	-0.31	0.004029235655	0.046	1
SE	LRP2BP	-	chr4:185378080-185378207	NA	-0.311	0.01178140079	-0.037	1
SE	PARPBP	+	chr12:102182548-102182627	NA	-0.311	0.0002851607735	-0.071	1
SE	AK2	-	chr1:33011447-33011710	NA	-0.312	9.96E-06	-0.002	1
SE	CDC25C	-	chr5:138325814-138325904	NA	-0.312	1.94E-06	-0.06	1
SE	MAN2B2	+	chr4:6609396-6609574	NA	-0.313	0.0001010478751	0.001	1
SE	CHEK1	+	chr11:125656205-125656335	NA	-0.313	0.0002812419842	0.064	1
SE	LINC00174	-	chr7:66400321-66400437	NA	-0.314	0.0008590836179	0.068	1
SE	ELMOD3	+	chr2:85355554-85355784	NA	-0.315	0.04376813577	0.012	1
SE	PCNX	+	chr14:70988566-70988699	NA	-0.316	0.006891588316	-0.042	1
SE	TMEM161B-	+	chr5:88285740-88285851	NA	-0.317	2.86E-06	0.002	1
SE	NEDD4	-	chr15:55991925-55992005	NA	-0.318	0.01072761332	-0.05	1
SE	FAM208B	+	chr10:5709529-5709663	NA	-0.318	0.0313341614	-0.105	1
SE	ZCWPW2	+	chr3:28478813-28478931	NA	-0.318	0.04732471362	0.017	1
SE	NEK6	+	chr9:124292848-124293013	NA	-0.319	0.004357067677	-0.028	0.01472867009
SE	FAM193B	-	chr5:177531281-177531521	NA	-0.319	2.98E-05	-0.011	1
SE	CDKL3	-	chr5:134350248-134350427	NA	-0.32	0.02984907237	-0.062	1
SE	LINC00969	+	chr3:195663623-195663832	NA	-0.32	2.17E-06	0.06	1
SE	WIPF1	-	chr2:174677044-174677124	NA	-0.321	0.004005348732	-0.027	1
SE	ISCU	+	chr12:108564059-108564155	NA	-0.321	0.0001418305399	-0.007	0.5714972179
SE	C8orf44	+	chr8:66677641-66677954	NA	-0.321	0.01632161566	0.026	1
SE	ZNF761	+	chr19:53445000-53445123	NA	-0.322	1.76E-05	0.021	1
SE	OSBPL6	+	chr2:178324176-178324269	NA	-0.323	1.60E-09	-0.043	1
SE	KCTD9	-	chr8:25446079-25446250	NA	-0.323	0.01287710281	0.039	1
SE	MRPS31P4	+	chr13:52629218-52629359	NA	-0.323	0.0001872472194	-0.164	0.002335329708
SE	ZC3H14	+	chr14:88601923-88602083	NA	-0.325	0.0001541382425	-0.024	1
SE	ST5	-	chr11:8729949-8731209	NA	-0.325	0.0007143248076	-0.038	0.9077938238
SE	TMTC4	-	chr13:100670282-100670569	NA	-0.327	1.64E-06	0.015	1
SE	POLR2J4	-	chr7:43986600-43986677	NA	-0.327	0.001407114613	-0.09	0.02530429799
SE	ATXN3	-	chr14:92047895-92048089	NA	-0.328	0.01781677028	0.025	1
SE	KANK2	-	chr19:11196049-11196227	NA	-0.329	0.00167889463	0.12	1
SE	ANKDD1A	+	chr15:64944651-64944747	NA	-0.33	5.01E-05	-0.048	0.2380220377
SE	STAG3L4	+	chr7:67308576-67308652	NA	-0.331	0.0009659206584	-0.21	1
SE	SLC50A1	+	chr1:155136827-155136951	NA	-0.332	1.70E-06	-0.02	1
SE	HHLA3	+	chr1:70366145-70366328	NA	-0.332	0.03495076767	0.033	1.29E-05

SE	ADAMTS13	+	chr9:133438245-133438366	NA	-0.333	9.27E-05	0.03	1
SE	AC004381.6	+	chr16:20813189-20813302	NA	-0.334	0.0241083796	0.041	1
SE	CARD8	-	chr19:48242539-48242644	NA	-0.334	6.60E-07	0.002	1
SE	KLHL7	+	chr7:23117833-23117964	NA	-0.334	0.002563569625	-0.174	1.47E-05
SE	KCTD9	-	chr8:25446128-25446250	NA	-0.335	0.007073635801	0.035	1
SE	MCM3	-	chr6:52265234-52265385	NA	-0.335	0	0.004	0.3051416962
SE	MARK2	+	chr11:63903985-63904147	NA	-0.336	0.04088100642	0.111	1.17E-06
SE	PGAP2	+	chr11:3823616-3823740	NA	-0.337	0.04930931816	0.079	1
SE	SLMAP	+	chr3:57907883-57908006	NA	-0.337	0	-0.046	1
SE	IQCB1	-	chr3:121797117-121797227	NA	-0.338	0.003619441762	-0.041	1
SE	BAG6	-	chr6:31639499-31639646	NA	-0.338	1.58E-13	0	1
SE	NADK	-	chr1:1756778-1756882	NA	-0.34	2.89E-14	0.002	1
SE	CDKL1	-	chr14:50332261-50332432	NA	-0.34	2.97E-05	0.083	0.6450388627
SE	PPA2	-	chr4:105456695-105456745	NA	-0.34	1.28E-09	-0.065	0.08720728937
SE	POLL	-	chr10:101584877-101584919	NA	-0.34	0.01394848916	-0.09	1
SE	LIPT1	+	chr2:99156300-99156426	NA	-0.342	0.01909479024	-0.012	1
SE	FBXL2	+	chr3:33297868-33297962	NA	-0.342	0.002142500663	-0.024	1
SE	RP11-42I10.	+	chr16:48671860-48672033	NA	-0.342	6.74E-05	0.011	1
SE	CDC25C	-	chr5:138326020-138326054	NA	-0.343	8.74E-06	-0.101	1
SE	ADARB1	+	chr21:45184473-45184593	NA	-0.343	2.24E-05	0.039	1
SE	BDNF-AS	+	chr11:27675576-27676140	NA	-0.343	0.001406429329	0.001	1
SE	BCAT2	-	chr19:48807755-48808236	NA	-0.345	0.01860693698	-0.136	0.008072673115
SE	ZGRF1	-	chr4:112548252-112548380	NA	-0.346	2.11E-06	-0.122	0.005489998185
SE	FAM76B	-	chr11:95779077-95779135	NA	-0.347	2.25E-07	-0.064	0.1550974639
SE	PPRC1	+	chr10:102146671-102147392	NA	-0.347	2.35E-13	0.031	1
SE	PICALM	-	chr11:85990249-85990399	NA	-0.349	0.0001596972278	0.009	1
SE	MAP4K4	+	chr2:101860824-101860986	NA	-0.35	1.98E-11	0.024	0.6076199675
SE	DENND4A	-	chr15:65700543-65700672	NA	-0.35	1.22E-07	0.079	1
SE	GIGYF2	+	chr2:232722553-232722658	NA	-0.35	0.03294531518	0.048	1
SE	DRAM2	-	chr1:111139587-111139711	NA	-0.351	8.31E-06	-0.108	0.007389615969
SE	AP3S2	-	chr15:89877305-89877424	NA	-0.351	0.01954407125	-0.166	0.002541734369
SE	TOPORS-AS	+	chr9:32565396-32565628	NA	-0.351	0.001855130247	-0.099	1
SE	NADK	-	chr1:1756738-1756882	NA	-0.352	5.59E-14	0.005	0.6692838042
SE	RALY	+	chr20:34065043-34065160	NA	-0.352	0.0005418065682	-0.04	0.4734481098
SE	TCTN1	+	chr12:110634395-110634463	NA	-0.353	0.005171587045	-0.107	0.006492979708
SE	UBE2Q2P2	+	chr15:82408287-82408367	NA	-0.354	6.56E-05	0.022	1
SE	GSAP	-	chr7:77328605-77328637	NA	-0.358	1.17E-05	-0.107	1
SE	RPS3A	+	chr4:151100484-151100588	NA	-0.359	3.45E-09	-0.003	1
SE	KIAA0586	+	chr14:58543882-58543967	NA	-0.36	4.82E-05	-0.031	0.05872442611
SE	TWF1	-	chr12:43805830-43806002	NA	-0.36	0.0001485744595	0.008	0.009466461147
SE	C5orf45	-	chr5:179853196-179853276	NA	-0.36	0.01335046322	-0.098	1
SE	PRKRIP1	+	chr7:102395976-102396537	NA	-0.361	2.37E-05	-0.032	1
SE	MRPL52	+	chr14:22830963-22831037	NA	-0.361	0.004738364736	0.041	0.005021442539
SE	PCNX	+	chr14:70995740-70995925	NA	-0.362	6.66E-05	0.016	1
SE	ANXA2	-	chr15:60396150-60396427	NA	-0.362	0.006386827087	0.079	1
SE	RP11-421L2	+	chr1:101075394-101075716	NA	-0.362	1.04E-06	-0.113	0.0005273259685
SE	KB-1572G7.	-	chr22:23686830-23686995	NA	-0.362	0.008304198588	-0.082	1
SE	SLC25A26	+	chr3:66236543-66236700	NA	-0.363	0.00798304603	-0.013	1
SE	POGLUT1	+	chr3:119478333-119478474	NA	-0.363	0.01651821476	-0.045	1
SE	RP11-849H4	-	chr11:71925932-71926104	NA	-0.363	0.01809089575	0.07	1
SE	RAD51D	-	chr17:35116858-35117037	NA	-0.364	0.04505876384	0.023	1
SE	SGSM2	+	chr17:2367270-2367405	NA	-0.365	1.48E-06	-0.048	0.0007408437771
SE	ZC3H18	+	chr16:88611267-88611536	NA	-0.365	0.001733304234	-0.015	1
SE	GK	+	chrX:30668011-30668118	NA	-0.365	0.002839945502	-0.015	1
SE	CC2D2A	+	chr4:15479222-15479328	NA	-0.366	0.01617330057	0.108	3.17E-10
SE	RTN2	-	chr19:45493159-45493378	NA	-0.366	0.00365172218	-0.11	0.0003053249332
SE	PGAP2	+	chr11:3823612-3823740	NA	-0.366	0.001732186964	0.039	0.001668824333
SE	ZSCAN31	-	chr6:28329302-28329778	NA	-0.367	0.0001149006239	0.043	1
SE	LPHN2	+	chr1:81952986-81953025	NA	-0.368	0.004113255113	0.037	1
SE	BDNF-AS	+	chr11:27675296-27676140	NA	-0.368	0.00177508727	0	1
SE	RPS3A	+	chr4:151100974-151101085	NA	-0.371	1.58E-06	-0.005	1
SE	C3orf18	-	chr3:50565525-50565700	NA	-0.372	0.01820919836	0.038	0.8353793742
SE	MGAT5	+	chr2:134253094-134253218	NA	-0.373	0.01190796755	-0.015	1

SE	ANAPC16	+	chr10:72220053-72220379	NA		-0.373	2.52E-07	-0.011	1
SE	CCNI	-	chr4:77056248-77056323	NA		-0.374	0.0007768817232	0.057	1
SE	NFIB	-	chr9:14116180-14116346	NA		-0.374	0.002058724474	-0.036	2.82E-05
SE	SREBF1	-	chr17:17817547-17817591	NA		-0.375	0.0013265261	-0.002	1
SE	ELMOD3	+	chr2:85387097-85387225	NA		-0.375	0.0001184888955	0.004	1
SE	NME6	-	chr3:48296118-48296158	NA		-0.375	7.94E-06	0.038	1
SE	ZNF680	-	chr7:64543706-64543802	NA		-0.377	4.99E-05	-0.043	1
SE	ATP5C1	+	chr10:7806973-7807010	NA		-0.379	0.0004310721539	0.019	1
SE	SGCE	-	chr7:94639340-94639448	NA		-0.38	0.003512996048	0.006	1
SE	ANKAR	+	chr2:189705022-189705224	NA		-0.38	6.03E-05	-0.008	1
SE	ZSCAN31	-	chr6:28329302-28329714	NA		-0.38	8.71E-05	0.043	1
SE	CREB1	+	chr2:207577109-207577224	NA		-0.381	0.001185968339	-0.111	1
SE	TMEM260	+	chr14:56621530-56621702	NA		-0.384	4.25E-06	-0.055	0.02941186272
SE	MAP4K4	+	chr2:101860824-101860986	NA		-0.384	6.13E-05	-0.195	1.41E-10
SE	RWDD3	+	chr1:95237347-95237460	NA		-0.386	0.0002933051974	-0.004	1
SE	PICALM	-	chr11:85990249-85990378	NA		-0.387	0	0.015	4.24E-05
SE	MRPS28	-	chr8:80028694-80028796	NA		-0.387	0.01459665929	-0.1	0.002399977568
SE	KIF21B	-	chr1:200983055-200983094	NA		-0.388	0.005890845208	0.08	1
SE	NME6	-	chr3:48296118-48296236	NA		-0.388	0.001460213182	0.087	0.364791001
SE	ABCA11P	-	chr4:437445-437511	NA		-0.39	0.0008550215213	-0.059	1
SE	FAM76B	-	chr11:95779077-95779135	NA		-0.393	0.0002600015158	-0.104	1
SE	TPM1	+	chr15:63064063-63064190	NA		-0.394	0.0003706949144	0.005	1
SE	NAP1L4	-	chr11:2989128-2989271	NA		-0.394	6.18E-07	-0.023	1
SE	NISCH	+	chr3:52480878-52481109	NA		-0.395	0.001052296447	-0.052	1
SE	CLEC2D	+	chr12:9693033-9693115	NA		-0.395	0.02024804826	-0.036	1
SE	ACYP2	+	chr2:54050957-54051050	NA		-0.395	0.0004126182547	0.003	1
SE	TRAPPC13	+	chr5:65655635-65655653	NA		-0.396	1.37E-05	-0.068	0.1112287712
SE	OPA1	+	chr3:193617783-193617837	NA		-0.396	0.002268274405	-0.047	1
SE	FAM66C	+	chr12:8195276-8195412	NA		-0.396	0.02043424899	-0.009	1
SE	RASSF8-AS	-	chr12:25947518-25947622	NA		-0.397	0.0415224856	-0.036	1
SE	SNAPC5	-	chr15:66494902-66495419	NA		-0.399	0.001312004339	-0.029	1
SE	FOXM1	-	chr12:2865354-2865399	NA		-0.4	0	-0.291	0
SE	STX16	+	chr20:58669301-58669453	NA		-0.4	4.53E-08	-0.033	1
SE	DOCK7	-	chr1:62544946-62545039	NA		-0.402	7.63E-12	0.028	0.02735058732
SE	ZNF211	+	chr19:57633785-57633905	NA		-0.403	0.002075918316	0.058	1
SE	ULK4	-	chr3:41915976-41916052	NA		-0.403	0.01803272989	0.028	1
SE	TRIM16	-	chr17:15677175-15677280	NA		-0.403	0.01092216805	-0.054	1
SE	DLEU2	-	chr13:50044650-50044768	NA		-0.404	1.13E-06	-0.047	1
SE	ING4	-	chr12:6655637-6655913	NA		-0.405	0.001631986303	-0.094	1
SE	CPNE3	+	chr8:86524876-86524997	NA		-0.406	4.50E-07	-0.131	1
SE	SEMA4F	+	chr2:74657852-74657951	NA		-0.406	1.72E-06	0.186	1
SE	SEPT6	-	chrX:119625334-119625379	NA		-0.408	0.001889786218	0.017	0.4065025993
SE	PARPBP	+	chr12:102195311-102195447	NA		-0.408	7.60E-05	-0.044	1
SE	PICALM	-	chr11:85990249-85990378	NA		-0.41	0.004066478452	0.001	1
SE	CCDC7	+	chr10:32471063-32471230	NA		-0.41	0.03915855325	-0.056	1
SE	C1orf159	-	chr1:1083914-1084011	NA		-0.411	0.00746974798	0.037	0.291894514
SE	ZNF789	+	chr7:99478273-99478356	NA		-0.412	0.001102013063	-0.044	1
SE	ENTPD1	+	chr10:95842343-95842494	NA		-0.413	0.0001503356103	0.002	1
SE	ARL13B	+	chr3:93996562-93996684	NA		-0.413	0.009312346136	0.034	1
SE	NUMA1	-	chr11:72012400-72012442	NA		-0.417	2.52E-09	-0.102	0
SE	SLC25A29	-	chr14:100298026-100298190	NA		-0.417	0.001127335634	-0.003	1
SE	PSMA3-AS1	-	chr14:58293065-58293138	NA		-0.417	1.58E-13	-0.004	0.9650937482
SE	CA5B	+	chrX:15772495-15772614	NA		-0.418	0.0001159793671	-0.003	1
SE	TPM1	+	chr15:63061197-63061273	NA		-0.419	8.14E-10	-0.075	0.01630012279
SE	FAM227B	-	chr15:49577171-49577305	NA		-0.42	0.0006714121578	0.051	1
SE	IKZF4	+	chr12:56019317-56019415	NA		-0.422	0.002024138061	0.056	1
SE	SEC24B	+	chr4:109481676-109481781	NA		-0.425	2.17E-11	0.107	0.005586626612
SE	APEH	+	chr3:49674488-49674621	NA		-0.425	5.13E-08	-0.067	2.69E-05
SE	HCG18	-	chr6:30314268-30314482	NA		-0.426	0.01832264645	-0.023	1
SE	PARD3	-	chr10:34336198-34336243	NA		-0.427	1.53E-12	0.037	1
SE	MACF1	+	chr1:39436481-39436490	NA		-0.428	5.56E-07	-0.19	1.61E-07
SE	MRPS6	+	chr21:34103496-34103519	NA		-0.428	0.001796423469	0.047	1
SE	STX2	-	chr12:130795994-130796120	NA		-0.43	0	0.063	0.2859101726

SE	PGAP2	+	chr11:3823612-3823740	NA		-0.434	0.01725172355	0.087	1
SE	ATP11B	+	chr3:182902480-182902566	NA		-0.436	0.0001130729132	-0.115	1
SE	TMEM161B-	+	chr5:88287435-88287622	NA		-0.436	4.27E-09	-0.073	0.5167260806
SE	WDR27	-	chr6:169613558-169613656	NA		-0.437	2.16E-05	-0.002	1
SE	CDK4	-	chr12:57750441-57750563	NA		-0.44	0.008685505951	-0.044	1
SE	AGPAT4	-	chr6:161165576-161165713	NA		-0.441	0.001023745599	-0.12	1
SE	PVR	+	chr19:44658741-44658765	NA		-0.441	0.0002963289862	0.023	1
SE	CC2D1B	-	chr1:52354364-52354454	NA		-0.441	1.52E-05	-0.001	1
SE	TMEM234	-	chr1:32217436-32217577	NA		-0.441	0.000117787783	-0.024	0.0003342243668
SE	DHFR	-	chr5:80637882-80638009	NA		-0.441	0.004239439132	-0.018	1
SE	EPB41L1	+	chr20:36173763-36173954	NA		-0.442	0.0111182907	0.024	1
SE	GPR56	+	chr16:57641586-57641708	NA		-0.443	0.009505385205	0.124	1.73E-12
SE	SIL1	-	chr5:139127943-139128052	NA		-0.447	4.68E-06	0.011	1
SE	DDR1	+	chr6:30885624-30885680	NA		-0.447	0.0001146660205	-0.177	3.91E-13
SE	HERC2P9	+	chr15:28655494-28655691	NA		-0.447	0.001439456619	-0.001	1
SE	NUDT13	+	chr10:73125397-73125509	NA		-0.448	0.02109524081	0.006	1
SE	LRRC37A4F	-	chr17:45542763-45542835	NA		-0.448	0.0006039756834	0.013	1
SE	RP11-345J4	-	chr16:29450111-29450276	NA		-0.448	0.01900449467	0.091	1
SE	NBPF8P	+	chr1:120447093-120447166	NA		-0.448	4.38E-05	0.5	1
SE	SUOX	+	chr12:55999332-55999590	NA		-0.449	0.001493573318	-0.019	1
SE	ITPRIPL1	+	chr2:96325746-96325849	NA		-0.45	4.21E-05	-0.053	1
SE	MED12L	+	chr3:151409242-151409332	NA		-0.451	0.0006243958793	0.012	1
SE	SPIN1	+	chr9:88416643-88416766	NA		-0.452	0.003349315801	0.12	1
SE	CSAD	-	chr12:53171325-53171441	NA		-0.452	0.00576997794	-0.052	1
SE	NISCH	+	chr3:52480878-52481037	NA		-0.453	0.0001737618393	-0.049	1
SE	C14orf93	-	chr14:22999956-23000117	NA		-0.453	0.003140202336	0.046	1
SE	NSRP1	+	chr17:30163066-30163162	NA		-0.453	0.02613073178	0.058	1
SE	FAM86FP	-	chr12:8235252-8235518	NA		-0.454	0.04253479721	0.235	1.12E-06
SE	C8orf44	+	chr8:66676744-66676902	NA		-0.455	0.0009339975889	0.055	1
SE	PRR34-AS1	+	chr22:46055598-46055733	NA		-0.455	7.54E-05	0.122	1
SE	CDKL3	-	chr5:134312291-134312380	NA		-0.456	7.45E-05	0.05	1
SE	PICALM	-	chr11:85990249-85990399	NA		-0.458	0	0.019	0.0002234820154
SE	CCDC148	-	chr2:158345214-158345318	NA		-0.458	0.02324707802	-0.162	1
SE	SPATS2L	+	chr2:200308886-200309090	NA		-0.459	0.02523595813	-0.055	0.003101143011
SE	CCDC15	+	chr11:124991460-124991583	NA		-0.46	0.0001503356103	0.117	1
SE	EXO1	+	chr1:241849111-241849216	NA		-0.46	1.86E-12	-0.09	1
SE	CLEC2D	+	chr12:9693824-9693948	NA		-0.461	5.17E-05	-0.004	1
SE	DHRS4L2	+	chr14:24001383-24001517	NA		-0.461	0.0006418050778	0.071	0.4484524402
SE	LCA5L	-	chr21:39427758-39427905	NA		-0.462	0.001839490346	0.086	1
SE	BBS1	+	chr11:66523171-66523365	NA		-0.462	0.0001503356103	0.023	1.21E-09
SE	FAM228B	+	chr2:24137908-24138100	NA		-0.463	0.0005866349904	0.003	1
SE	LINC00630	+	chrX:102825992-102826169	NA		-0.467	0.0002076409456	-0.152	1
SE	RP11-9G1.3	-	chr4:133140585-133140659	NA		-0.468	2.41E-06	0.043	1
SE	TMEM241	-	chr18:23370215-23370261	NA		-0.469	2.54E-05	0.057	1
SE	VCAN	+	chr5:83537006-83542268	NA		-0.471	3.38E-05	-0.202	0
SE	SHF	-	chr15:45171882-45172002	NA		-0.471	0.001004510926	0.027	1
SE	UBA2	+	chr19:34431860-34431931	NA		-0.474	0.001456868059	0.044	1
SE	BPHL	+	chr6:3119284-3119512	NA		-0.475	1.12E-05	0.035	1
SE	SLC3A2	+	chr11:62870744-62870837	NA		-0.475	0	-0.164	0.0004743134009
SE	NHSL1	-	chr6:138441982-138442114	NA		-0.476	0.007436656522	0.027	1
SE	PVR	+	chr19:44658741-44658765	NA		-0.478	0.0002048526225	0.045	1
SE	HCG18	-	chr6:30314268-30314482	NA		-0.478	1.30E-07	0.039	1
SE	INVS	+	chr9:100131869-100131954	NA		-0.479	0.00118875574	-0.12	1
SE	PLCB4	+	chr20:9373046-9373104	NA		-0.482	0.0001633148417	-0.178	1
SE	SUOX	+	chr12:55999332-55999458	NA		-0.483	0.02072133492	-0.063	1
SE	SMARCC2	-	chr12:56164302-56164368	NA		-0.483	0.0003110222918	-0.065	0
SE	PBRM1	-	chr3:52554723-52554879	NA		-0.487	0.002131254622	-0.026	0.3753665405
SE	DCLK2	+	chr4:150253473-150253556	NA		-0.488	7.92E-10	-0.024	0
SE	SDHAP1	-	chr3:195968218-195968385	NA		-0.49	0	0.009	1
SE	DTNB	-	chr2:25383835-25383889	NA		-0.491	0.0008538554437	-0.053	0.02369477492
SE	TRIM16	-	chr17:15677175-15677280	NA		-0.495	0.002239049543	0.047	1
SE	MIOS	+	chr7:7567606-7567688	NA		-0.497	0.0001530389937	0.068	1
SE	CLHC1	-	chr2:55208625-55208710	NA		-0.498	9.57E-05	0.06	1

SE	AC004076.9	-	chr19:57475659-57475786	NA	-0.498	0.0001214203275	-0.003	1
SE	FAM86EP	-	chr4:3948089-3948220	NA	-0.499	0.001505065008	-0.011	1
SE	AREL1	-	chr14:74701668-74701774	NA	-0.502	0.001735749298	-0.193	1
SE	RWDD3	+	chr1:95239776-95239909	NA	-0.503	0.0002931707329	-0.008	1
SE	FANCI	+	chr15:89245755-89245839	NA	-0.504	3.29E-08	-0.002	1
SE	C6orf52	-	chr6:10672546-10672636	NA	-0.505	0.01885208174	-0.064	1
SE	OPA1	+	chr3:193667370-193667455	NA	-0.506	0.01205057511	-0.239	1
SE	ZNF611	-	chr19:52707391-52707538	NA	-0.506	0.00374323208	-0.027	1
SE	SHF	-	chr15:45171882-45172078	NA	-0.508	0.0007745711686	0.034	0.9718710115
SE	SLCO4A1	+	chr20:62668157-62668184	NA	-0.51	0.006698640151	-0.133	1
SE	CSAD	-	chr12:53171176-53171441	NA	-0.51	0.0001524546588	-0.043	1
SE	LINC01128	+	chr1:850177-850351	NA	-0.51	0.02372588051	0.083	1
SE	DYNLL1	+	chr12:120495283-120495437	NA	-0.512	0.001232062067	0.073	1
SE	ANAPC16	+	chr10:72220053-72220379	NA	-0.512	0	-0.043	0.2418983119
SE	STX16-NPE	+	chr20:58691404-58691562	NA	-0.512	0.0002874981515	-0.089	1
SE	DIAPH2	+	chrX:96763099-96763120	NA	-0.514	0.0001769287926	-0.011	1
SE	GGT1	+	chr22:24608989-24609231	NA	-0.515	3.61E-05	0.016	1
SE	RSRC2	-	chr12:122526169-122526293	NA	-0.517	0.0001240921588	0.017	1
SE	NUDT13	+	chr10:73125397-73125509	NA	-0.517	0.0008479198092	0.034	1
SE	RPL17	-	chr18:49491735-49491833	NA	-0.518	1.66E-09	0.066	0.003111975725
SE	RPL17	-	chr18:49491735-49491833	NA	-0.518	0	0.024	5.34E-05
SE	DENND4C	+	chr9:19336685-19336832	NA	-0.52	0	0.028	1
SE	CSAD	-	chr12:53171272-53171441	NA	-0.52	0.0002923670232	-0.041	1
SE	ERBB2IP	+	chr5:66075023-66075230	NA	-0.523	0	-0.006	1
SE	C5orf45	-	chr5:179853196-179853276	NA	-0.526	0.0003544351238	-0.12	1
SE	PHYKPL	-	chr5:178223319-178223451	NA	-0.526	4.88E-07	0.015	1
SE	C1orf50	+	chr1:42767508-42767624	NA	-0.527	3.15E-07	-0.082	0.008228621344
SE	CCDC150	+	chr2:196677114-196677192	NA	-0.528	0.0001382276306	-0.06	1
SE	SUOX	+	chr12:55999332-55999458	NA	-0.531	4.75E-05	-0.019	1
SE	RWDD3	+	chr1:95239776-95239909	NA	-0.532	1.07E-06	-0.022	1
SE	TMEM254	+	chr10:80086230-80086258	NA	-0.532	0.0004276980026	-0.025	1
SE	FBXL12	-	chr19:9813230-9813274	NA	-0.533	3.50E-05	-0.005	1
SE	TMEM161B-	+	chr5:88287435-88287622	NA	-0.534	8.81E-13	-0.039	1
SE	CADM2	+	chr3:85979163-85979283	NA	-0.535	5.93E-06	-0.15	1
SE	MORC2	-	chr22:30963224-30963334	NA	-0.537	0.006121948866	-0.054	1
SE	TMEM161B-	+	chr5:88283009-88283086	NA	-0.54	1.95E-07	-0.031	1
SE	RP11-705C1	+	chr12:9656567-9656697	NA	-0.542	4.86E-07	-0.017	1
SE	POLL	-	chr10:101584877-101584919	NA	-0.548	0.001950407967	0.128	1
SE	U2AF1L4	-	chr19:35744490-35744540	NA	-0.55	1.56E-09	-0.001	1
SE	SOS1	-	chr2:38989269-38989314	NA	-0.553	2.09E-06	-0.225	2.43E-11
SE	MAX	-	chr14:65077562-65077643	NA	-0.553	1.66E-10	-0.031	1
SE	ZNF189	+	chr9:101399394-101399530	NA	-0.554	9.33E-10	-0.028	1
SE	EXO1	+	chr1:241849108-241849216	NA	-0.555	0	-0.111	1
SE	SLCO4A1	+	chr20:62668154-62668184	NA	-0.556	0.0006103010624	-0.115	1
SE	CNOT2	+	chr12:70285399-70285586	NA	-0.556	8.05E-05	0.02	0.7382928969
SE	RSG1	-	chr1:16232892-16233017	NA	-0.557	5.59E-05	-0.135	0.000196399903
SE	RP11-371E8	+	chr14:93188892-93188997	NA	-0.557	0.0008231927538	-0.117	1
SE	PPRC1	+	chr10:102145019-102145090	NA	-0.558	7.52E-13	0.008	1
SE	FAM86EP	-	chr4:3946625-3946939	NA	-0.559	5.49E-05	0.004	1
SE	NDUFB2	+	chr7:140700869-140700959	NA	-0.561	1.31E-05	-0.02	1
SE	SDHAP2	+	chr3:195663623-195663767	NA	-0.572	0	0.056	1
SE	TRIM16	-	chr17:15681050-15681222	NA	-0.577	0.006143326895	-0.231	1
SE	NBR2	+	chr17:43132955-43133137	NA	-0.583	2.65E-07	0.028	1
SE	FDFT1	+	chr8:11803090-11803225	NA	-0.595	1.01E-05	-0.125	1
SE	RPL17-C18o	-	chr18:49488499-49488566	NA	-0.596	0	-0.006	0
SE	TEAD1	+	chr11:12878888-12878900	NA	-0.599	8.25E-14	-0.099	1
SE	CLEC16A	+	chr16:11174153-11174277	NA	-0.601	0.001796423469	0.044	0.00696067509
SE	C5orf45	-	chr5:179851242-179851424	NA	-0.604	1.26E-05	-0.111	1
SE	MROH8	-	chr20:37155062-37155167	NA	-0.609	0.001277328111	0.013	1
SE	CPT1B	-	chr22:50573807-50573921	NA	-0.61	4.70E-13	-0.076	0.03505798078
SE	PPP2R3C	-	chr14:35108138-35108236	NA	-0.611	5.99E-06	-0.033	1
SE	TWF1	-	chr12:43805830-43805885	NA	-0.612	8.15E-05	-0.003	1
SE	AC074286.1	-	chr2:177332141-177332251	NA	-0.616	6.59E-11	0.033	0.7292808135

SE	PAQR6	-	chr1:156247509-156247632	NA	-0.619	4.35E-07	0.008	1
SE	TPT1-AS1	+	chr13:45360140-45360239	NA	-0.619	0.004168487247	-0.044	1
SE	NDUFB2	+	chr7:140700869-140700959	NA	-0.623	7.31E-06	-0.014	1
SE	KIAA1468	+	chr18:62280359-62280442	NA	-0.631	9.58E-12	-0.034	1
SE	OSBPL6	+	chr2:178339671-178339764	NA	-0.635	0	0.009	1
SE	RP11-33B1.	+	chr4:119543752-119543845	NA	-0.635	0.0005024837576	0.071	0.3209474617
SE	FAM86B3P	+	chr8:8234482-8234584	NA	-0.64	0.01525821969	-0.058	1
SE	MCOLN3	-	chr1:85036709-85036868	NA	-0.648	3.05E-06	0.051	1
SE	RAI14	+	chr5:34813573-34813660	NA	-0.649	0	-0.022	1
SE	SNHG14	+	chr15:25053265-25053363	NA	-0.65	2.78E-07	-0.092	1
SE	CTD-2378E1	-	chr15:50890855-50891077	NA	-0.655	0.02204339488	-0.014	1
SE	SYTL2	-	chr11:85711112-85711232	NA	-0.66	0	-0.056	1
SE	DYRK4	+	chr12:4580823-4580977	NA	-0.667	0.002780083212	0.06	1
SE	C14orf93	-	chr14:23000958-23001031	NA	-0.667	3.29E-05	0.011	1
SE	SLTM	-	chr15:58912562-58912610	NA	-0.667	1.22E-05	0.03	1
SE	RGMB-AS1	-	chr5:98771655-98771766	NA	-0.667	2.10E-07	0.219	1
SE	ACACA	-	chr17:37345252-37345382	NA	-0.667	0.003589770542	0.457	1
SE	RP11-1228E	-	chr17:71365-71556	NA	-0.667	0.001952422279	-0.065	1
SE	PLA2G12A	-	chr4:109718688-109718759	NA	-0.67	7.20E-06	-0.047	0.7537550146
SE	OSBPL6	+	chr2:178339671-178339764	NA	-0.671	0.0005646569594	-0.099	1
SE	APITD1-COI	+	chr1:10440346-10440413	NA	-0.673	0.001998768924	-0.151	1
SE	FNTB	+	chr14:65032358-65032521	NA	-0.679	5.36E-09	0.005	0.001500651941
SE	C2CD5	-	chr12:22519114-22519239	NA	-0.69	9.33E-06	0.135	1
SE	FKTN	+	chr9:105574197-105574332	NA	-0.692	0	-0.095	1
SE	ADD3	+	chr10:110132304-110132400	NA	-0.694	0	-0.006	1
SE	SPAST	+	chr2:32098795-32098891	NA	-0.697	5.59E-14	-0.233	2.79E-10
SE	KIF13A	-	chr6:17789871-17789910	NA	-0.706	0	-0.13	0.0001637023178
SE	HKR1	+	chr19:37346887-37347052	NA	-0.712	1.03E-06	0.023	0.17022336
SE	SERHL2	+	chr22:42573633-42573745	NA	-0.73	1.31E-09	0.082	1
SE	DUSP18	-	chr22:30664954-30665121	NA	-0.738	1.14E-10	-0.022	1
SE	IST1	+	chr16:71903164-71903258	NA	-0.742	0.0002334056595	0.029	1
SE	C2orf74	+	chr2:61157861-61158012	NA	-0.743	1.09E-09	-0.012	1
SE	CRHR1-IT1	+	chr17:45640388-45640455	NA	-0.754	3.26E-07	-0.012	1
SE	NRG2	-	chr5:139871043-139871148	NA	-0.776	0.0003004649703	0.166	1
SE	LPP	+	chr3:188341662-188341719	NA	-0.778	1.83E-09	-0.094	1
SE	EMC3-AS1	+	chr3:9988371-9988477	NA	-0.782	3.23E-08	-0.089	1
SE	HKR1	+	chr19:37344609-37344774	NA	-0.782	7.85E-07	-0.013	1
SE	RP11-849H4	-	chr11:71925932-71926104	NA	-0.787	4.30E-07	0.021	1
SE	TWF1	-	chr12:43805523-43805637	NA	-0.789	4.38E-08	-0.001	1
SE	FRG1B	+	chr20:30391196-30391308	NA	-0.796	0	0	1
SE	TCF7L2	+	chr10:113160618-113160691	NA	-0.805	0.0001537967219	-0.105	0.5033618553
SE	ARIH2	+	chr3:48945135-48945181	NA	-0.852	0	-0.041	0.8091615025
SE	GPR126	+	chr6:142383759-142383843	NA	-0.871	0	0.102	1
SE	PCTP	+	chr17:55787565-55787654	NA	-0.874	0	-0.116	1
SE	XPNPEP3	+	chr22:40860677-40860793	NA	-0.881	5.32E-13	0.019	1
SE	SLC27A5	-	chr19:58481063-58481187	NA	-0.897	3.04E-05	-0.007	1
SE	WBP1	+	chr2:74458831-74458919	NA	-0.968	1.34E-13	-0.05	1

Supplementary Table 5: Differentially expressed (multi-exon) genes between mesenchymal (MES) and proneural (PN) GSCs.

Up-regulated genes in MES (down-regulated in PN)			Down-regulated genes in MES (up-regulated in PN)		
gene	log2FoldChange	FDR	gene	log2FoldChange	FDR
ANPEP	10.37672335	1.99E-70	GPM6B	-10.7108866	3.53E-127
CDCP1	10.40751179	4.38E-70	XIST	-10.22135421	1.92E-94
SLFN11	10.60639843	5.23E-70	S100B	-11.42490738	4.02E-86
FOSL1	6.627260714	6.26E-67	PTPN12	-9.999474471	2.84E-81
TGM2	8.0496362	2.47E-62	PDE4B	-9.699042881	7.76E-74
ALDH1A3	9.693668267	4.46E-58	PMP2	-10.52753001	1.69E-69
CTSZ	9.232888351	3.20E-56	COL11A1	-9.852666093	2.15E-68
FGF5	9.914601859	9.77E-56	ARNT2	-9.154545309	4.87E-65
MDFIC	8.245710663	1.49E-53	PTPRZ1	-10.28835018	1.65E-64
DNAH14	9.92104056	1.48E-51	PRTFDC1	-10.00667187	1.29E-63
HTATIP2	9.580435866	1.73E-51	KCNJ10	-9.87928151	2.84E-63
NPTX1	9.466385946	5.90E-48	GLUL	-10.26958145	1.42E-61
PROCR	6.359394042	3.17E-47	KCNA2	-10.10043006	1.60E-61
C4orf32	9.223157055	2.46E-46	NRN1	-6.728159335	6.09E-58
PLBD1	9.361245145	1.24E-45	FEZ1	-10.17148472	4.86E-57
MAGEB2	10.47706157	4.67E-45	BCAN	-10.28913039	4.13E-56
DGKE	4.657100071	1.82E-42	EDNRB	-10.41573472	1.47E-55
TNFRSF10D	9.844729806	5.15E-42	PRRX1	-10.14033503	1.39E-54
EGR1	5.868025559	5.36E-42	ZNF532	-10.70904731	7.97E-54
MAGEA6	8.868855162	2.48E-41	ARL10	-8.203124291	9.15E-54
STEAP2	10.03667625	3.45E-41	THY1	-10.7581076	3.55E-53
SAGE1	9.807227223	3.65E-41	PCDH15	-9.315194192	3.32E-52
TUSC3	10.13746076	3.85E-41	LAMA4	-10.27567243	3.49E-52
BAIAP2L1	6.367793984	1.04E-40	FXYD6	-9.91321044	1.22E-51
MAGEA12	8.887703501	2.63E-40	OLIG2	-10.26687569	3.17E-50
PLAC8	8.325223787	1.25E-39	C16orf62	-5.362219583	8.72E-50
SSH3	6.341574541	1.85E-39	NLGN3	-7.881961442	1.09E-49
PCDHGA2	6.176337738	7.07E-39	GJA1	-10.83161779	4.49E-46
ME1	9.99547059	1.63E-37	TUBB2B	-10.34902154	4.63E-46
MAGEA3	9.20938161	2.18E-37	PDE7B	-7.554988903	1.01E-45
AIM1	4.484574169	2.27E-37	LSAMP	-11.09310763	2.38E-45
KRT8	9.674670956	7.60E-37	JAM3	-10.09854089	3.23E-45
DSP	8.731780312	1.50E-36	VCAN	-10.59791853	6.34E-45
ADIRF-AS1	8.146945972	3.93E-36	GNG2	-10.37731504	2.35E-44
EFEMP1	10.94104935	1.18E-35	SEMA5A	-8.620146667	2.59E-44
TMEM200A	8.154277392	2.21E-35	LPL	-10.86010733	5.94E-44
PRAME	11.30299762	2.28E-34	SLC35F1	-9.913492707	1.09E-43
CENPV	6.07890014	5.41E-34	OLIG1	-10.4094014	1.77E-43
LY6K	9.042398443	1.69E-33	CSPG4	-9.981876495	1.87E-43
TNNT1	7.367167158	2.31E-33	MATN2	-5.727630561	5.55E-43
AP1M2	8.676689761	2.52E-33	SOX2-OT	-9.101572388	5.55E-43
CARD10	3.768033105	3.49E-33	ANGPTL2	-10.21874552	2.80E-42
TPTEP1	8.710320898	4.75E-33	LOXL3	-8.960420753	6.74E-42
NFE4	9.976464198	6.41E-33	MMP2	-10.58258811	1.13E-41
TRIML2	11.22848393	7.02E-33	ZNF608	-8.890494985	3.60E-41
CCNA1	7.873729163	3.98E-32	ZNF354C	-6.988507908	3.01E-40
KCNC4	5.727273687	8.91E-32	FREM2	-5.34289419	1.36E-39
LRRK1	8.571496039	1.06E-31	ZNF300	-11.00747094	4.33E-39
MTUS2	10.68290418	9.40E-31	SALL1	-10.58666471	5.44E-39
RP11-169F17.1	9.033911847	1.37E-30	SFRP2	-10.44066802	7.92E-39
SH2D5	6.059762317	1.63E-30	PLEKHG1	-5.313309705	1.25E-38
VRK2	8.89492723	2.36E-30	CADM1	-10.13968566	2.92E-38
KIAA1324L	6.036329732	2.36E-30	MAP2	-8.550779268	1.24E-37
FAT4	6.403587108	2.76E-30	SEMA6D	-10.74443082	2.38E-37
CDYL2	4.09893842	3.66E-30	MPHOSPH8	-9.960201482	2.46E-37
DOCK5	9.951895733	4.36E-30	ZNF334	-9.167076066	2.52E-37
TRIM59	8.908310705	4.97E-30	MDFI	-9.446982379	2.61E-37
KATNAL1	3.923730963	2.47E-29	CELF2	-9.197588986	3.31E-37
ANKRD19P	9.505126862	4.11E-29	ZNF736	-10.01302976	3.31E-37
HYI	4.717152425	4.55E-29	SULF2	-9.432349657	3.40E-37
RAB11FIP1	7.786360457	5.32E-29	ARRDC4	-9.922042555	9.19E-37
BNC1	9.628933752	7.03E-29	ZNF542P	-8.241290774	1.08E-36
SLC6A15	10.63090322	8.52E-29	FGFBP3	-4.890933756	1.31E-36

**RP11-29H23.5**	5.503680744	1.10E-28
**EGFR**	8.054984625	2.38E-28
**GALNT6**	9.520782009	5.22E-28
**KRT18**	10.48646723	8.62E-28
**SYT1**	9.182934052	9.78E-28
**STEAP1B**	7.740812364	1.01E-27
**ZDHHC23**	5.825985955	1.38E-27
**MEIOC**	10.28027997	2.24E-27
**ADM2**	8.178556601	3.47E-27
**RUNX3**	7.918046976	1.00E-26
**FAM83G**	8.138353029	2.19E-26
**TES**	11.5895949	2.98E-26
**CASP4**	5.47407291	5.91E-26
**MIPEP**	3.630105065	6.82E-26
**ANKRD27**	3.621652629	2.55E-25
**NIPAL2**	8.36324125	1.11E-24
**ATP1A3**	6.239698946	1.15E-24
**CADPS2**	8.128795909	1.94E-24
**STAMBPL1**	6.804542538	2.61E-24
**FAM167A**	6.998362133	4.32E-24
**MAP3K21**	4.424497619	5.59E-24
**ASPHD1**	6.064175804	9.89E-24
**SPAG1**	4.676396214	1.16E-23
**MAT1A**	7.790193181	1.41E-23
**FRG1CP**	5.838530862	2.66E-23
**MIR222HG**	4.064738431	3.46E-23
**IER3**	8.701544953	4.32E-23
**CHAC1**	5.281574911	4.60E-23
**PRKCH**	6.334595303	7.52E-23
**DUSP23**	7.170341232	7.62E-23
**RP11-66B24.7**	8.978200339	8.13E-23
**GBP3**	5.910045439	1.02E-22
**CATSPER1**	7.986893223	1.24E-22
**ECHDC2**	6.681644148	1.60E-22
**DENND2C**	5.835339136	2.82E-22
**ALS2CL**	6.818265014	3.48E-22
**GALNT14**	9.829325991	3.89E-22
**LTBR**	11.6375772	4.79E-22
**G0S2**	7.105369181	5.12E-22
**ZYG11A**	9.298223962	8.93E-22
**AOX1**	7.880765933	9.45E-22
**TPM2**	4.718007161	1.31E-21
**DMKN**	10.85795198	2.40E-21
**NME7**	3.184804872	4.21E-21
**ZFP69**	6.679942664	5.57E-21
**RASGEF1A**	11.32825721	6.39E-21
**SELENOM**	6.401009234	7.38E-21
**MAP7**	5.298952006	9.52E-21
**SIK1**	4.775276041	1.51E-20
**IL4R**	8.482273784	1.84E-20
**EMB**	11.61506186	2.13E-20
**SERPINB9**	8.0080179	2.24E-20
**PPARG**	8.888484141	2.51E-20
**KCNG1**	6.44977673	2.70E-20
**MPP7**	5.703107201	3.90E-20
**TNFRSF10A**	11.17295269	6.54E-20
**CTGF**	5.860050234	1.69E-19
**PMAIP1**	5.786364675	1.71E-19
**CSAG1**	10.39534479	2.24E-19
**LINC00960**	10.26330846	2.66E-19
**IL7**	6.924790947	2.84E-19
**RERG**	6.463962203	2.84E-19
**NIPAL1**	5.786889744	3.62E-19
**PTPRH**	6.884909544	3.71E-19
**PARP4**	3.664220502	3.83E-19
**TPBG**	10.9432167	3.92E-19
**RP11-420A23.1**	3.684649121	4.13E-19
**FLT4**	11.1406747	5.10E-19

**SETD6**	-7.381658324	1.68E-36
**SCRG1**	-11.00534551	2.13E-36
**ANK3**	-5.405543463	3.28E-36
**KBTBD2**	-10.16136065	3.63E-36
**VANGL2**	-10.05992989	4.66E-36
**DZIP1**	-10.41702861	7.91E-36
**EMILIN2**	-9.817925615	1.18E-35
**MLLT11**	-3.661895834	1.41E-35
**LINC00511**	-4.72758947	8.94E-35
**PCDHGC3**	-8.35313208	1.06E-34
**ARMCX4**	-9.554212362	1.39E-34
**ZNF883**	-7.861048753	2.51E-34
**CPXM1**	-10.59310884	2.90E-34
**GAS7**	-10.61413097	3.24E-34
**TTYH1**	-9.274951487	3.37E-34
**ZNF426**	-6.550993203	3.83E-34
**RFTN2**	-9.486571459	3.83E-34
**SALL3**	-9.484263493	7.54E-34
**SOX5**	-10.44884347	8.88E-34
**TNS2**	-5.274985934	1.11E-33
**ELOVL2**	-10.50214959	1.29E-33
**FAXDC2**	-8.26761745	6.82E-33
**LINC00665**	-6.508442983	2.47E-32
**COL15A1**	-9.540643455	3.01E-32
**ZNF93**	-6.992893798	5.30E-32
**ZCCHC24**	-3.633322916	5.98E-32
**DISC1**	-7.749702677	6.46E-32
**AEBP1**	-10.00012064	7.06E-32
**SALL2**	-10.13047648	7.18E-32
**CHFR**	-4.3815182	1.70E-31
**ZNF585B**	-5.896946498	2.98E-31
**GNG7**	-3.592546789	5.58E-31
**COL9A3**	-10.84611674	7.89E-31
**SV2A**	-10.50705791	8.45E-31
**PNMAL1**	-10.66805759	1.01E-30
**GPRC5B**	-6.25402465	1.37E-30
**PCDH17**	-7.346332956	1.62E-30
**SNTB1**	-10.72263333	1.78E-30
**METTL7A**	-10.00097875	2.29E-30
**PPP1R9A**	-10.27095545	3.18E-30
**CGREF1**	-8.270353768	5.04E-30
**PROM1**	-9.594418494	2.08E-29
**ZNF431**	-10.57495506	5.15E-29
**SOX8**	-10.61991431	6.31E-29
**CALD1**	-6.980993502	7.17E-29
**CCDC149**	-9.605882251	1.74E-28
**FRMD5**	-3.470142481	2.15E-28
**DNAH9**	-10.38475554	3.92E-28
**AIF1L**	-6.580842703	5.14E-28
**IGFBP2**	-8.857058782	6.29E-28
**TP73-AS1**	-8.380778075	9.40E-28
**DDX19B**	-7.00083471	1.38E-27
**ZNF43**	-10.46143831	1.44E-27
**TNS1**	-7.553817513	1.59E-27
**PTN**	-7.544921187	9.51E-27
**PAG1**	-6.013691688	1.28E-26
**FLRT3**	-9.404052833	1.57E-26
**ELMO1**	-10.38645852	1.57E-26
**HUNK**	-8.59894225	1.94E-26
**GTF2I**	-3.536858275	1.97E-26
**ARHGEF7**	-3.169599061	2.19E-26
**CERS6**	-11.63716116	2.20E-26
**FAM222A**	-4.446160383	2.27E-26
**GREB1**	-4.58141535	6.44E-26
**COL20A1**	-8.507407137	2.20E-25
**RP11-43F13.1**	-5.493523604	2.46E-25
**LINC00461**	-4.071773964	3.20E-25
**GATM**	-6.081612298	3.42E-25

TRIM58	9.157004469	5.22E-19
SLC7A1	4.685882218	6.19E-19
PCK2	4.053455228	9.70E-19
LINC00470	10.06050549	1.29E-18
CECR7	6.053854562	1.36E-18
LINC01123	7.706435703	1.45E-18
SERPINB1	6.875822023	1.82E-18
LRRC3	10.06294273	2.15E-18
BCL2A1	9.578396942	2.28E-18
MYPN	7.765391721	2.87E-18
RANBP17	9.991459436	3.49E-18
LINC01436	9.962790407	3.64E-18
CHMP4C	10.6741186	4.88E-18
PCBD1	2.576167751	4.88E-18
LINC00707	8.816289946	5.03E-18
RCN3	4.914377718	5.39E-18
NMU	5.767828016	5.93E-18
SLFN12	9.859558309	6.30E-18
PLA2G16	3.456964396	6.33E-18
BTNL9	10.55411173	6.77E-18
CTC-459F4.3	2.972995332	7.19E-18
MGST1	8.323798165	7.62E-18
CT45A10	9.842596013	8.52E-18
ADAMTS20	6.395726259	8.90E-18
CD40	9.848257012	9.59E-18
ANKRD18A	9.840397959	9.59E-18
MLPH	9.97898411	1.03E-17
AC005301.9	10.40930029	1.21E-17
SPAG4	4.83178205	1.24E-17
POPDC3	10.70298984	1.47E-17
H2AFJ	10.10846556	1.58E-17
TNFAIP2	4.579302989	1.82E-17
ELL2	4.370510164	1.84E-17
TBX18	10.55129888	1.88E-17
RP11-30P6.6	5.681131516	2.14E-17
LDHC	7.827302255	2.32E-17
AC003092.1	10.50044544	3.23E-17
PDE2A	7.539356435	3.24E-17
MAN1A1	5.994690759	3.81E-17
LINC00857	8.351658274	5.01E-17
PPP4R4	6.434039894	5.47E-17
EPHA6	10.32105983	5.55E-17
HS3ST3A1	8.286553592	5.75E-17
WDR66	6.426849601	7.31E-17
RP5-884M6.1	9.72557049	8.11E-17
TRHDE	10.225134	1.02E-16
MME	7.580851253	1.13E-16
IL31RA	10.29331539	1.31E-16
GATA2	7.564511382	1.87E-16
GFPT2	3.789043114	2.39E-16
CXCL5	9.88726771	2.48E-16
MYEOV	6.72909767	2.78E-16
POP4	2.558240582	3.06E-16
ZC3H12B	10.03520897	3.13E-16
RP11-366H4.1	10.03231099	3.19E-16
LYPD5	6.057885385	3.45E-16
DMC1	4.532516677	3.59E-16
ZNF215	10.02543514	3.62E-16
EXT1	3.434408515	3.92E-16
CCDC68	8.224374394	3.99E-16
ALOXE3	7.381877954	4.18E-16
UQCRFS1	2.88582042	4.45E-16
TMEM156	9.559318585	5.31E-16
IRF5	7.00966284	5.34E-16
FAM201A	9.935176618	5.54E-16
LINC01249	9.35697513	5.98E-16
ARHGAP44	9.385450595	6.31E-16
CSAG3	9.420240009	6.95E-16

ZNF521	-9.053771583	6.87E-25
TCF4	-6.396944488	9.15E-25
PCYT1B	-9.739303728	1.14E-24
STK32B	-9.948259043	1.30E-24
C8orf46	-7.552860078	1.30E-24
MAPK10	-5.577763238	2.41E-24
SATB1	-8.151615527	2.44E-24
SLIT1	-8.858801125	2.97E-24
C3orf70	-9.879811433	4.32E-24
DGKI	-8.056519559	4.98E-24
ID4	-9.676126145	5.08E-24
FUZ	-5.489171703	6.01E-24
ASIC4	-7.363346329	6.89E-24
MEGF10	-7.723854206	8.88E-24
KCNIP1	-10.4081515	1.00E-23
FAM49A	-9.702722105	1.06E-23
TMEM246	-9.921678381	1.46E-23
BMPR1B	-8.276208211	1.75E-23
NLGN4X	-11.23965927	2.53E-23
DTX3	-9.417946781	2.67E-23
PPP1R14C	-11.01184836	2.80E-23
TET1	-9.264971076	4.27E-23
IFFO1	-8.216836297	5.09E-23
ZNF738	-11.13900474	6.12E-23
ZNF382	-7.315329042	6.23E-23
SEMA5B	-8.997057749	6.86E-23
LGI2	-9.662332607	6.86E-23
HSD17B7P2	-4.537758875	7.26E-23
NIM1K	-6.896650699	7.95E-23
PEG3	-9.771631465	9.70E-23
U2AF1	-4.008162325	1.11E-22
KLHL13	-7.731430076	1.55E-22
LRRN3	-9.781411133	1.99E-22
ZNF506	-9.665650226	3.80E-22
JAKMIP2	-9.286124584	4.60E-22
HR	-8.47755102	4.64E-22
B3GAT1	-10.73660002	7.25E-22
ZNF558	-10.88040244	8.30E-22
BCHE	-6.773798862	2.14E-21
SPARC	-9.925643879	2.19E-21
GOLGA8A	-4.239484651	2.24E-21
MIR34AHG	-7.151118111	2.77E-21
PDGFRA	-7.789961156	3.32E-21
UNC80	-6.892903517	3.32E-21
FYN	-2.80300083	3.86E-21
FABP7	-10.09407029	4.62E-21
COL25A1	-9.871205098	5.67E-21
LRRC17	-9.852956937	7.20E-21
RNF175	-10.6522121	7.91E-21
NID2	-10.74102599	8.89E-21
EPDR1	-10.62044559	9.54E-21
CD200	-7.121530413	1.07E-20
ATCAY	-8.890513603	1.24E-20
ARHGEF6	-8.800450871	1.25E-20
TBCEL	-10.51166637	1.63E-20
LRRTM2	-8.257057854	1.96E-20
PTPRO	-9.49242516	2.01E-20
SEMA6A	-3.721909589	2.06E-20
BRSK2	-9.124951221	2.23E-20
TNFAIP6	-11.87449937	2.51E-20
MBNL3	-7.840253782	2.85E-20
DPYSL2	-3.285516338	3.22E-20
ZNF577	-4.719904165	4.14E-20
NFATC4	-9.281561952	4.66E-20
RHOJ	-8.207722309	4.68E-20
AC074289.1	-6.611724699	5.13E-20
DOCK10	-5.317664304	5.21E-20
ZNF254	-3.300071439	5.92E-20

**FBXL13**	4.073630031	7.04E-16	**SHISA4**	-8.252074567	6.07E-20
**RP11-497G19.1**	7.366430786	7.63E-16	**BAALC**	-9.8394667	8.66E-20
**RP11-108M9.4**	6.808368938	8.17E-16	**MAF**	-9.360820864	9.09E-20
**IRX2**	9.879670091	1.04E-15	**SCN4B**	-7.319101004	1.11E-19
**TM4SF19**	8.107684931	1.12E-15	**SLITRK2**	-10.04752805	1.22E-19
**UBR5-AS1**	3.568858858	1.20E-15	**CHDH**	-8.279875292	1.35E-19
**ARNTL2**	3.112129098	1.27E-15	**ZNF737**	-7.790571748	1.36E-19
**CD55**	6.416076487	1.55E-15	**MAGI2**	-4.699332574	1.77E-19
**HLA-F**	3.785726115	1.74E-15	**EFS**	-11.71719437	2.19E-19
**ECHDC3**	9.916313073	1.95E-15	**ZNF529**	-10.37140739	2.19E-19
**LINC00944**	9.821144795	2.02E-15	**ZNF85**	-8.923181435	2.41E-19
**HHEX**	9.284678213	2.09E-15	**CEP41**	-2.976284071	2.58E-19
**RP11-148B18.4**	9.230758474	2.71E-15	**KLHDC8B**	-5.177604003	2.70E-19
**TEC**	9.788127266	2.87E-15	**SHROOM2**	-9.25792332	3.70E-19
**MPC2**	2.217026581	2.91E-15	**TRPM3**	-9.023382272	4.07E-19
**LINC01139**	9.226548995	3.83E-15	**ZNF853**	-9.488504741	4.76E-19
**ARHGAP18**	2.894816636	4.55E-15	**CENPBD1P1**	-6.754699469	5.26E-19
**FBXO2**	6.161180216	4.82E-15	**SIK3**	-2.593062937	5.61E-19
**MSC-AS1**	7.978350409	5.06E-15	**ZNF100**	-10.16308062	6.51E-19
**SRGN**	8.999051767	5.33E-15	**CTSF**	-10.82814364	8.15E-19
**PCDHB16**	3.639258312	7.40E-15	**ZNF813**	-5.755383586	8.26E-19
**SNCA**	9.822429782	7.71E-15	**PDK2**	-8.066321843	9.06E-19
**HTR7**	9.07563604	8.97E-15	**GALM**	-7.069916342	1.19E-18
**KIRREL2**	5.612867901	9.91E-15	**DPYSL3**	-5.057738842	1.22E-18
**LINC01605**	7.765975334	1.00E-14	**FAM185A**	-6.1123909	1.32E-18
**PDCD5**	2.815378657	1.13E-14	**GRIA3**	-9.675971287	1.44E-18
**TOR4A**	5.508333968	1.13E-14	**HS6ST2**	-11.41136831	1.53E-18
**CASC9**	9.550356712	1.20E-14	**ZNF714**	-4.260582069	2.14E-18
**RP11-66B24.2**	9.683498973	1.23E-14	**SLC1A3**	-3.63981199	2.29E-18
**GABRG2**	9.665870915	1.75E-14	**LGR5**	-7.870737827	2.81E-18
**ANKRD29**	5.047227526	1.75E-14	**SCN1A**	-9.357026789	2.95E-18
**SLC6A17**	9.499196799	1.77E-14	**GPC4**	-11.45651654	2.98E-18
**CTB-140J7.2**	9.055864721	1.87E-14	**MYO5C**	-6.118861437	3.15E-18
**SLC37A1**	3.021648762	1.89E-14	**PDE1C**	-8.354175503	3.44E-18
**PLCG2**	9.590450465	2.05E-14	**GAL3ST4**	-6.443570621	3.64E-18
**DSE**	2.704223603	2.09E-14	**ZNF677**	-9.188854358	3.86E-18
**MIR137HG**	9.463462249	2.91E-14	**NACAD**	-10.5508776	4.41E-18
**RP4-647C14.2**	9.440803875	3.12E-14	**ZNF528**	-10.1800793	8.36E-18
**HES7**	4.361735502	3.25E-14	**LGALS3BP**	-6.121070095	1.03E-17
**RBM11**	9.634986879	3.26E-14	**PYROXD2**	-6.317394309	1.11E-17
**ADGRE1**	9.915077612	3.80E-14	**CTNND2**	-9.188019381	1.14E-17
**WBP2NL**	6.031877741	3.81E-14	**ZNF649**	-4.239487276	1.14E-17
**RPS14**	2.69839837	4.43E-14	**CYTL1**	-11.17613217	1.15E-17
**PEAR1**	9.651199139	4.78E-14	**C1orf106**	-10.54470501	1.19E-17
**LINC00910**	4.052061325	4.78E-14	**HNMT**	-8.373712742	1.46E-17
**ZNF185**	4.038606758	4.79E-14	**STS**	-6.984349731	1.60E-17
**ANKRD20A5P**	9.060929706	5.04E-14	**ZNF493**	-9.795868508	1.61E-17
**ZNF507**	2.576833563	5.28E-14	**GAB1**	-3.007152327	1.63E-17
**SLC35F3**	7.725858952	5.52E-14	**LINGO1**	-7.211888039	1.66E-17
**CCL28**	9.472655039	5.61E-14	**TNFRSF21**	-3.469843197	2.52E-17
**TRHDE-AS1**	9.426571735	5.89E-14	**MEST**	-10.35274885	2.57E-17
**MVP**	4.607741975	6.03E-14	**TMEM100**	-10.92996911	2.82E-17
**VEGFC**	8.836172741	6.20E-14	**GRIA2**	-6.415433296	3.08E-17
**LRRC8E**	8.858383051	6.47E-14	**RNF165**	-7.209967682	3.16E-17
**XAGE1A**	8.842575962	6.51E-14	**RNF182**	-10.33654376	3.24E-17
**MTMR11**	3.003439045	8.69E-14	**LRRC4**	-10.7217206	3.37E-17
**TBC1D31**	2.526802507	8.82E-14	**NR2F1**	-4.215252249	3.50E-17
**ADTRP**	9.315486173	9.03E-14	**SETD9**	-4.251404359	3.77E-17
**SYDE2**	2.786296789	9.42E-14	**ITPKB**	-9.904710761	3.79E-17
**EIF3H**	2.355237204	9.92E-14	**KCNK2**	-7.243138293	3.93E-17
**RP11-129M6.1**	9.283071256	1.02E-13	**GPR158**	-2.873095031	4.71E-17
**CRLF1**	8.337542336	1.05E-13	**NPAS3**	-7.214867527	5.70E-17
**ANKRD33B**	3.577189315	1.07E-13	**NDRG4**	-6.076663384	6.00E-17
**CT83**	9.346767662	1.11E-13	**PHYHIPL**	-10.89254433	6.17E-17
**SPOCD1**	6.738843561	1.11E-13	**FCGRT**	-6.761377092	6.89E-17
**FOXF1**	6.367552262	1.21E-13	**IL17D**	-4.924958106	7.23E-17
**ENPEP**	7.734195951	1.37E-13	**CDH11**	-8.197407409	7.76E-17
**SLC27A2**	6.13916901	1.78E-13	**ZNF90**	-10.21007468	8.19E-17

**PCAT7**	7.012366968	1.83E-13
**URI1**	2.834677087	1.93E-13
**TBX3**	8.766174026	2.04E-13
**PCDHGA11**	4.423236618	2.15E-13
**AC009237.8**	9.329693341	2.55E-13
**CHMP1B2P**	9.57475411	2.71E-13
**CHRNB1**	3.381862282	2.84E-13
**POMP**	3.077207618	2.86E-13
**ABL2**	1.946649981	3.09E-13
**GREB1L**	5.654658124	3.32E-13
**PI15**	9.287295526	3.53E-13
**UAP1**	3.917844275	3.56E-13
**TYMP**	7.789511082	4.06E-13
**TRDN**	7.458389294	4.29E-13
**SYTL3**	4.49682492	4.53E-13
**EOGT**	2.54815863	4.75E-13
**PAX9**	5.974857725	6.11E-13
**RP11-390F4.3**	4.822466039	6.69E-13
**LINC01533**	9.351719175	6.69E-13
**GABRA3**	8.575160557	6.74E-13
**ADAMTSL5**	4.541447974	6.83E-13
**STARD4**	2.836661212	7.11E-13
**NEXN**	3.064731182	7.17E-13
**CYR61**	5.2672121	7.41E-13
**STARD8**	4.215153879	8.20E-13
**TINAGL1**	9.507350859	8.94E-13
**C9orf84**	8.657146418	9.74E-13
**XDH**	9.82222514	1.07E-12
**POLD4**	4.083277395	1.07E-12
**MICAL2**	3.586544564	1.11E-12
**LINC00662**	2.658992173	1.17E-12
**SP140L**	2.932572231	1.28E-12
**SULT1B1**	9.066396323	1.36E-12
**PDLIM1**	6.279512668	1.36E-12
**RP11-262H14.3**	4.33804083	1.57E-12
**C2orf61**	6.773942963	1.57E-12
**PIK3CD**	4.158851226	1.58E-12
**TATDN1**	3.26461441	1.65E-12
**MELTF**	3.497884867	1.83E-12
**MARVELD2**	6.100614067	1.88E-12
**NPAS1**	4.601118761	1.93E-12
**S100A11**	7.009811445	1.93E-12
**RP11-221N13.3**	5.221276331	1.97E-12
**SPHK1**	4.437044625	2.09E-12
**ARMC4**	5.531166913	2.15E-12
**MED30**	2.878150653	2.31E-12
**AP001065.15**	8.370716353	2.71E-12
**CASC8**	7.391618522	2.71E-12
**RSPH9**	6.504984186	2.87E-12
**RP11-121P12.1**	9.043223723	2.97E-12
**NEURL1**	4.506955651	3.17E-12
**KCNH5**	8.870059735	3.35E-12
**STK26**	6.445434363	3.42E-12
**RBP7**	7.112883305	3.84E-12
**KC6**	8.731874238	4.18E-12
**SP100**	5.294358713	4.33E-12
**LINC00668**	8.742740586	4.45E-12
**PERP**	4.800936489	4.47E-12
**VSTM1**	8.855953601	4.48E-12
**DAW1**	9.25006084	4.94E-12
**PCBP3**	5.969916999	5.81E-12
**SLC27A6**	8.464113724	5.81E-12
**RP11-879F14.2**	7.167672853	5.85E-12
**FMNL1**	5.775497124	6.05E-12
**GFI1**	3.369899347	6.06E-12
**PABPC1**	2.380206452	6.33E-12
**ADRA2C**	7.735334101	6.79E-12
**FOXL1**	8.909237677	7.98E-12

**NCALD**	-8.930482188	9.75E-17
**FOXG1**	-10.62131767	9.94E-17
**HOXD10**	-6.527447426	1.09E-16
**ZNF788**	-8.068120567	1.13E-16
**EDA2R**	-9.853769986	1.18E-16
**SCARA3**	-9.682658406	1.23E-16
**AC018647.3**	-10.62948487	1.29E-16
**EHD3**	-10.17479461	1.42E-16
**L3MBTL1**	-8.284658022	1.44E-16
**AUTS2**	-6.38015083	1.64E-16
**NLGN1**	-5.059927971	1.80E-16
**HAGLR**	-10.37607784	1.88E-16
**CLIP3**	-5.945129955	1.96E-16
**CSGALNACT1**	-8.232399915	2.10E-16
**PALD1**	-10.90680831	2.15E-16
**ATP1B2**	-9.504524542	2.62E-16
**ZNF682**	-10.42077159	3.19E-16
**NTRK3**	-9.197252806	3.19E-16
**EFNB3**	-8.591446455	3.21E-16
**IL17RB**	-8.654065736	3.24E-16
**ZNF660**	-10.0699578	3.32E-16
**RAB33A**	-8.576585853	3.40E-16
**P2RX7**	-9.187600357	3.74E-16
**SOX21-AS1**	-10.67844791	4.24E-16
**PPFIBP2**	-3.621657396	4.66E-16
**PRMT2**	-3.181143107	5.87E-16
**UGT2B7**	-8.956885073	6.58E-16
**ST8SIA5**	-7.849818162	7.58E-16
**ZIC1**	-11.0272921	7.94E-16
**LEF1**	-7.382403235	8.04E-16
**ZNF329**	-6.00381647	9.07E-16
**SDHAP3**	-3.905372433	9.40E-16
**ZNF880**	-8.091083864	1.21E-15
**GRIA4**	-11.20919008	1.27E-15
**COLGALT2**	-7.359081654	1.30E-15
**ZNF829**	-9.859562407	1.31E-15
**SNX32**	-7.353942281	1.40E-15
**COL9A2**	-4.675710053	1.53E-15
**ZNF626**	-10.13870792	1.63E-15
**SBDSP1**	-2.872161777	1.91E-15
**RCBTB2**	-4.409763263	2.01E-15
**DNALI1**	-10.66378167	2.06E-15
**CX3CL1**	-9.218951742	2.23E-15
**ZNF513**	-4.810389562	2.35E-15
**C2orf72**	-3.585382675	2.43E-15
**HERC2P2**	-2.411534305	2.53E-15
**TUBA1A**	-3.493380648	2.64E-15
**TIMP4**	-10.35828778	2.66E-15
**LRRK2**	-3.487581527	2.69E-15
**CDIP1**	-4.509824675	2.72E-15
**RP11-210M15.2**	-7.961094847	2.74E-15
**PNMA2**	-9.704858902	2.96E-15
**MAP3K1**	-4.201957506	3.36E-15
**SPATA18**	-8.537539251	3.80E-15
**RRN3P1**	-10.05930384	3.80E-15
**MEG3**	-9.884091578	3.89E-15
**DSEL**	-4.768684615	3.96E-15
**AFG3L1P**	-3.001978887	4.39E-15
**RAPGEF4**	-6.607849543	4.48E-15
**RP11-161M6.2**	-9.861994023	4.74E-15
**FAM131B**	-8.237770506	5.08E-15
**IGFBP5**	-10.19729231	5.33E-15
**EN2**	-10.45114509	5.51E-15
**MANSC1**	-7.903026856	5.68E-15
**ZNF585A**	-9.42763627	5.98E-15
**PDGFD**	-7.056337249	6.09E-15
**NCAM1**	-7.326499882	6.63E-15
**CHST11**	-3.183656018	7.04E-15

LINC00937	8.783839089	8.75E-12	ZNF629	-2.225279491	8.15E-15
SNTG2	6.807679703	9.17E-12	LBH	-10.08479512	8.24E-15
LINC01615	8.243950135	1.01E-11	PHLPP1	-3.848510032	8.98E-15
DLK2	5.946646145	1.02E-11	D2HGDH	-10.12616631	9.56E-15
SNAP91	8.220812466	1.04E-11	DACT1	-9.543800149	9.59E-15
FAM129A	2.374203625	1.05E-11	GPM6A	-4.887951774	9.76E-15
FAAP24	2.666230167	1.13E-11	SLC16A9	-10.22003955	1.05E-14
GPATCH1	1.971018955	1.16E-11	GPX7	-10.41985536	1.09E-14
CEBPG	2.700543542	1.20E-11	EPHA3	-7.074658212	1.20E-14
IL18R1	5.756643411	1.32E-11	ZSCAN16	-9.661877953	1.36E-14
PIWIL4	3.814574611	1.37E-11	ZNF502	-7.840554377	1.38E-14
CTD-2162K18.4	3.338334789	1.38E-11	AGPAT4	-2.101653927	1.40E-14
AHNAK2	2.884554936	1.54E-11	LCTL	-8.342543906	1.62E-14
SLFN13	8.597945999	1.62E-11	SUGCT	-4.222257183	1.63E-14
RAB32	3.783890141	1.68E-11	UNC5C	-8.446723159	1.67E-14
ACVR1C	4.38929114	1.70E-11	PIM2	-4.257253211	1.67E-14
SH3RF2	6.545880405	1.76E-11	BMP7	-9.001588407	1.76E-14
C5orf38	8.731947638	1.94E-11	RLBP1	-9.386454877	1.78E-14
HYLS1	3.339636784	1.95E-11	CADM2	-5.915072343	1.94E-14
ITGBL1	6.954455607	1.95E-11	UNC5D	-9.606742574	2.14E-14
LSR	4.782358779	1.97E-11	LAYN	-8.206920928	2.15E-14
CYP1B1	3.102717418	2.12E-11	ZNF528-AS1	-10.33612284	2.16E-14
SYPL2	3.680256023	2.31E-11	HTRA1	-3.634436826	2.25E-14
MOXD1	6.016829671	2.41E-11	PROX1	-9.558855829	2.47E-14
PAX2	8.646906415	2.45E-11	COL9A1	-10.68593224	2.62E-14
TAP2	2.389164376	2.48E-11	CTNNA3	-9.274033093	2.75E-14
RAD21L1	6.88404503	2.52E-11	SLC16A4	-5.396769728	2.81E-14
TBX1	6.295838857	2.57E-11	ID2	-3.718737131	2.82E-14
CTD-2054N24.2	8.481067491	2.59E-11	RAMP1	-7.671859269	2.95E-14
STOM	3.04993815	2.64E-11	ZNF501	-9.124117562	3.11E-14
SCFD1	1.869988954	3.05E-11	GRID1	-9.147065955	3.15E-14
HSD17B7	2.263053874	3.22E-11	RP11-275H4.1	-9.076431963	3.29E-14
RIMS2	5.801715661	3.30E-11	NYNRIN	-9.748043583	3.77E-14
RP11-1149O23.3	8.06830903	3.64E-11	QKI	-2.873719689	4.43E-14
LINC00704	8.789363096	4.84E-11	CHST3	-2.172466205	4.46E-14
TREM1	8.84915168	4.89E-11	ARHGAP20	-6.330070706	4.68E-14
TMEM56	4.632607028	4.89E-11	RHBDL3	-9.394730482	4.78E-14
LAMA3	4.531572023	5.51E-11	ZKSCAN7	-9.551872719	4.80E-14
JAKMIP1	8.486903626	5.74E-11	BACE1	-2.538914293	4.81E-14
NCEH1	2.635097116	5.80E-11	SARM1	-4.854439691	5.29E-14
STARD10	3.220951235	6.92E-11	ENG	-9.407434652	6.08E-14
B3GNTL1	2.81932632	7.08E-11	GAREM2	-9.527203178	6.11E-14
STEAP1	6.699685619	7.55E-11	VSTM2B	-4.142350575	7.38E-14
ANKRD20A7P	5.747945417	7.68E-11	TANC1	-2.836249914	7.73E-14
ASNS	2.0213876	7.88E-11	PCDH7	-5.016483528	7.83E-14
RUNDC3B	5.662783935	8.15E-11	POM121	-2.615466504	8.67E-14
KCTD15	1.991483107	8.64E-11	TRIO	-2.359186565	1.01E-13
FENDRR	8.871195576	8.73E-11	ADCY1	-9.958402246	1.06E-13
MCL1	2.043310787	8.90E-11	TMEM255A	-10.15856111	1.11E-13
SLC44A3	4.801732463	1.03E-10	CRISPLD1	-2.36138726	1.16E-13
DLX4	5.605708081	1.04E-10	TMEM169	-3.73587433	1.21E-13
TRPM2	8.127330888	1.12E-10	LRP6	-2.387109171	1.27E-13
PLAGL1	6.907773222	1.13E-10	LRRTM3	-10.16304446	1.37E-13
PRDM6	5.357796962	1.15E-10	CHL1	-8.664921802	1.42E-13
IL22RA1	6.270520045	1.25E-10	TRIM71	-9.558260951	1.45E-13
ADAMTS16	7.971275535	1.27E-10	SPSB4	-9.108004162	1.52E-13
DYNC2H1	2.176882976	1.49E-10	DGKG	-5.995938399	1.70E-13
HTR4	8.278498155	1.62E-10	FCGR2A	-7.848040645	1.81E-13
CDC42EP1	3.893832613	1.63E-10	RP11-508N22.2	-4.021098732	1.89E-13
LINC02163	8.256808714	1.70E-10	ZNF793	-7.107591698	1.97E-13
RP11-66B24.4	8.411213248	1.78E-10	SLC38A3	-9.315850306	2.16E-13
SQRDL	3.78406253	1.95E-10	XYLT1	-6.404403193	2.29E-13
PARM1	5.76649706	1.96E-10	REEP2	-9.587509125	2.44E-13
PAWR	5.694403024	2.04E-10	ZNF350	-9.344411185	2.51E-13
PITPNM1	3.342526777	2.11E-10	KCNH2	-9.049089005	2.81E-13
PPIC	2.63486009	2.12E-10	SESN3	-4.662226537	2.89E-13
CEP55	1.781176612	2.28E-10	EN1	-9.470049184	2.89E-13
C1QTNF12	8.566225282	2.40E-10	SHC4	-4.204050352	3.06E-13

**MET**	4.964316953	2.68E-10	**LOX**	-9.595329966	3.09E-13
**HLX**	8.32616275	2.94E-10	**STAC2**	-9.498233452	3.17E-13
**KLC2**	2.363450549	2.99E-10	**FGL2**	-9.087055866	3.27E-13
**NOX3**	8.950231655	3.13E-10	**DTNA**	-7.133382993	3.43E-13
**HPSE**	3.841066439	3.35E-10	**ZNF568**	-4.321616907	3.53E-13
**CTC-308K20.1**	4.217809789	3.39E-10	**RASL10B**	-8.773796533	3.54E-13
**MSMO1**	2.104822324	3.44E-10	**MLC1**	-9.919336826	3.84E-13
**TCIRG1**	5.547952219	3.44E-10	**SRSF12**	-8.947413633	4.03E-13
**RP11-268G12.1**	4.871952911	3.91E-10	**DCHS1**	-7.789766224	4.62E-13
**CD109**	5.115971602	3.98E-10	**SPATA6**	-4.438571329	4.74E-13
**CSAG2**	8.209689102	4.01E-10	**BEND5**	-8.950317877	5.12E-13
**SAMD3**	8.241270508	4.11E-10	**LRRC37A2**	-5.296736398	5.33E-13
**NAGS**	4.321507059	4.13E-10	**NKX2-2**	-9.630164842	5.42E-13
**PKP3**	5.624990457	4.20E-10	**SOBP**	-3.061564279	5.54E-13
**LPIN3**	5.542145588	4.30E-10	**LINC01896**	-8.717255997	5.77E-13
**GADD45B**	3.506902648	4.44E-10	**CD302**	-9.487738425	5.83E-13
**NUDT19**	2.322905383	4.50E-10	**HILS1**	-9.081856382	5.86E-13
**CDK15**	8.483118047	4.56E-10	**SEPT7**	-2.503100507	6.00E-13
**SPTB**	4.068049916	4.77E-10	**ITGB8**	-4.02595054	6.13E-13
**MAFK**	3.048115348	4.79E-10	**CLN5**	-2.411325908	6.77E-13
**RP4-756H11.3**	3.053415812	4.84E-10	**SPARCL1**	-9.751046978	6.93E-13
**ICAM1**	4.553629258	4.92E-10	**AMPH**	-8.840481822	7.46E-13
**TMEM65**	2.609586651	5.00E-10	**TMEM200C**	-8.883351685	8.01E-13
**P4HA3**	6.022250026	5.15E-10	**HAND2**	-9.26807746	8.19E-13
**DUSP1**	3.286689697	5.55E-10	**YWHAG**	-2.719357957	8.83E-13
**FCHSD1**	2.542522501	5.64E-10	**CACNG4**	-9.330201339	9.32E-13
**INHBE**	5.439184056	5.77E-10	**CA14**	-9.090733215	1.00E-12
**DMTN**	4.237842151	5.83E-10	**CSMD2**	-8.919807686	1.01E-12
**NUDT2**	2.454110123	6.04E-10	**ZNF613**	-7.44015856	1.05E-12
**KCNJ11**	5.858381609	6.04E-10	**CAND2**	-8.970632865	1.07E-12
**STXBP5-AS1**	4.832831339	6.31E-10	**PPARGC1A**	-8.12470585	1.11E-12
**PCDHGB4**	5.331412664	6.42E-10	**DAAM2**	-9.243406812	1.12E-12
**MAGEA2B**	8.073498741	6.56E-10	**NKAIN1**	-6.859791618	1.16E-12
**SLCO1A2**	7.854010996	6.88E-10	**CASC15**	-9.336123802	1.20E-12
**SLC9A2**	5.285059646	7.06E-10	**PLPPR5**	-8.882810232	1.21E-12
**LRP11**	2.168077483	7.06E-10	**COL22A1**	-8.535900913	1.22E-12
**STEAP3**	2.615728039	7.06E-10	**FAM168B**	-1.779746693	1.22E-12
**RP11-423H2.1**	1.799410715	7.08E-10	**RP3-525N10.2**	-6.660068513	1.23E-12
**INO80C**	2.350599073	7.35E-10	**SHC3**	-2.691407268	1.44E-12
**PTTG1**	2.509301668	7.36E-10	**ZNF708**	-2.62627655	1.52E-12
**ATF7IP2**	7.706519694	8.31E-10	**ZNF730**	-8.739699902	1.60E-12
**DGAT2**	3.211915537	8.32E-10	**BAZ1B**	-2.762258786	1.62E-12
**BNC2**	6.47115463	8.34E-10	**ZNF597**	-9.041500888	1.89E-12
**DRAP1**	2.595262103	8.69E-10	**PTPRD**	-6.138160757	1.92E-12
**SLC39A4**	7.931494444	9.21E-10	**ATP1A2**	-8.800575235	2.08E-12
**MICA**	4.090575775	9.23E-10	**ENC1**	-2.964578613	2.18E-12
**SHC1**	3.049894071	9.26E-10	**NOMO1**	-2.07002862	2.28E-12
**EFCAB10**	6.816508256	9.46E-10	**GRASP**	-8.820935872	2.28E-12
**ACSM3**	3.829226204	9.62E-10	**SPOCK1**	-9.259056785	2.33E-12
**IL1R1**	5.977225851	1.01E-09	**PLXNB1**	-3.31053848	2.56E-12
**KDELR3**	3.078509215	1.03E-09	**TGFBR1**	-1.937063821	2.67E-12
**RNF135**	2.03717114	1.07E-09	**DYNLL2**	-2.049706022	2.84E-12
**BLZF1**	2.286235671	1.16E-09	**MREG**	-3.147754199	2.96E-12
**RP11-962G15.1**	5.492674363	1.17E-09	**EVL**	-2.592056101	3.09E-12
**KSR2**	7.934479716	1.23E-09	**WISP1**	-10.04000776	3.34E-12
**RP11-191L9.4**	7.544550047	1.24E-09	**LURAP1**	-8.932546904	3.59E-12
**NDUFB9**	2.297508066	1.32E-09	**BEX4**	-10.68838961	3.83E-12
**FOXA1**	8.296014433	1.32E-09	**FN1**	-4.475818544	3.95E-12
**ADAP2**	3.360071655	1.37E-09	**ING5**	-2.56375819	4.09E-12
**STOX1**	2.308681879	1.42E-09	**ZNF610**	-5.579433024	4.45E-12
**TUBA1C**	3.494690159	1.46E-09	**MOCS1**	-9.461182549	4.60E-12
**GCNT2**	2.558151097	1.52E-09	**PCDH9**	-4.972407095	4.66E-12
**MYO1D**	6.093075883	1.52E-09	**NF2**	-1.93103303	4.81E-12
**PAPLN**	5.08331233	1.60E-09	**SLC22A23**	-2.670555266	5.01E-12
**KCNG3**	5.224748569	1.73E-09	**ING3**	-1.920348482	5.24E-12
**CALB2**	8.803131501	1.76E-09	**ACSBG1**	-8.637936478	5.29E-12
**GPR176**	2.096464143	1.79E-09	**DNAH11**	-6.336125387	5.60E-12
**FSTL3**	2.678035027	1.90E-09	**SLC8A3**	-8.98646579	6.19E-12

HJURP	1.609170662	1.92E-09
RSPH3	1.979736713	1.93E-09
TMEM74	6.599369178	2.05E-09
CCDC69	4.491225879	2.15E-09
SLC5A12	3.495395949	2.16E-09
ERVMER34-1	6.97777023	2.21E-09
SUN3	8.842694345	2.25E-09
GATA6	7.960430528	2.45E-09
RP11-419I17.1	5.188552613	2.55E-09
GLT8D2	3.528624475	2.68E-09
CD274	3.414469829	2.86E-09
FADS3	2.588280271	2.88E-09
MNX1-AS1	4.331028456	2.88E-09
OSCAR	6.070837964	2.92E-09
AC116614.1	5.717797695	3.06E-09
IQCG	2.723182171	3.06E-09
COL6A4P1	7.77648113	3.07E-09
ARHGEF28	4.940166241	3.11E-09
ITGB2	8.433974296	3.31E-09
LPIN1	2.365586945	3.53E-09
HRK	3.464099169	3.89E-09
TSPEAR-AS2	7.861341045	4.06E-09
GATA2-AS1	7.511897668	4.17E-09
MSX2	7.854637092	4.22E-09
TRABD2A	4.547917093	4.49E-09
LINC01106	7.775865143	4.51E-09
ARHGAP40	6.48402718	4.53E-09
PPP1R13L	4.301383932	4.60E-09
N4BP3	4.528783286	4.64E-09
LINC00941	4.901685682	5.11E-09
SLC29A1	1.920056266	5.26E-09
PTGER4	7.785381826	5.29E-09
MNX1	3.125025732	5.42E-09
SMS	2.102905405	5.53E-09
TSPEAR-AS1	7.720834221	5.64E-09
RP11-359K18.4	3.529648568	5.78E-09
AURKB	2.363529693	5.90E-09
OTX1	3.284793985	6.08E-09
AC141928.1	6.975413428	6.36E-09
HOXB6	7.306240165	6.46E-09
EFHD2	3.113296975	6.64E-09
RP1-102E24.8	7.947629154	6.95E-09
ADAMTSL4	4.74600961	7.04E-09
CXCL3	7.715941373	7.11E-09
TTC39B	3.361909153	7.13E-09
RP11-408B11.2	7.274106998	7.61E-09
YIF1A	2.578775453	7.78E-09
NBPF8	2.38466	8.48E-09
LBX2-AS1	4.21313293	8.53E-09
DNAAF3	5.303352744	8.56E-09
RP4-639F20.1	2.662459679	8.82E-09
TMEM171	5.96635433	8.97E-09
PARD3	1.788626557	9.72E-09
CLCF1	3.814585842	1.05E-08
EML2-AS1	4.336272386	1.09E-08
RP11-63G10.4	7.212949803	1.11E-08
AJAP1	8.711525395	1.18E-08
RP11-809O17.1	4.287316525	1.19E-08
EFHC1	2.799524328	1.29E-08
RHBDF2	4.081740817	1.30E-08
BAG2	3.89029467	1.30E-08
PLEKHF1	3.814623752	1.30E-08
PAQR7	2.272751283	1.33E-08
PDGFB	8.448542423	1.33E-08
DNAH5	4.547241587	1.38E-08
RP11-328K4.1	7.624906303	1.43E-08
PATJ	2.919373071	1.43E-08
XAGE1B	7.222530678	1.47E-08

PDPN	-9.474467004	6.32E-12
TGFA	-6.037124927	7.26E-12
MIDN	-2.23557856	7.29E-12
TENM1	-7.601857637	7.83E-12
PDZRN3	-9.905967086	8.74E-12
VGLL4	-2.113415267	9.17E-12
POM121C	-2.53890947	9.25E-12
SIPA1L2	-3.833376088	9.27E-12
PLEKHA7	-5.426444875	9.33E-12
RP11-629G13.1	-8.379396214	1.00E-11
ZNF253	-3.346594707	1.03E-11
RP11-403A3.3	-9.883416675	1.05E-11
PIANP	-10.25838693	1.06E-11
MIR99AHG	-3.934916524	1.08E-11
DCLK1	-9.305260473	1.08E-11
ZNF132	-9.138988243	1.09E-11
TAS2R14	-3.171611974	1.10E-11
NRDE2	-1.863926192	1.11E-11
CECR2	-2.933356988	1.12E-11
MAMLD1	-2.539879194	1.13E-11
ZMIZ1	-2.719191971	1.15E-11
EXOC4	-1.869811181	1.23E-11
APCDD1	-9.562531603	1.23E-11
CASD1	-2.900277409	1.28E-11
NOTCH3	-8.153628127	1.28E-11
ARHGAP42	-4.016082445	1.28E-11
FCGR2C	-8.443591396	1.38E-11
MARC1	-4.341247758	1.41E-11
FADS2	-3.083241594	1.47E-11
AC108142.1	-8.919909338	1.48E-11
CDKN1B	-2.410553199	1.50E-11
FAM120C	-3.415820507	1.51E-11
WWC3	-2.606629707	1.60E-11
ST5	-3.325106723	1.61E-11
RGR	-8.660629444	1.72E-11
LHFPL3	-8.914018515	1.74E-11
AC005154.6	-2.818045991	1.77E-11
SCUBE2	-5.496805625	1.86E-11
LMO7	-3.435356769	1.95E-11
SORBS2	-7.416761466	2.11E-11
CECR6	-4.563177647	2.13E-11
ANKRD10	-2.292872501	2.15E-11
LUC7L2	-1.977428472	2.21E-11
ZNF3	-1.958961924	2.23E-11
ZNF10	-2.637146165	2.31E-11
PGAP1	-3.788491919	2.33E-11
ABCA1	-7.763159382	2.49E-11
PAX6	-9.115436937	2.65E-11
VASH1	-2.201840561	2.69E-11
DCC	-9.774836514	2.74E-11
WLS	-3.153694785	2.75E-11
SLC40A1	-9.266258679	2.91E-11
PKNOX2	-4.070588837	2.92E-11
KIF5A	-8.617260508	2.92E-11
RIMS4	-9.34069477	3.16E-11
LINC01268	-8.650840613	3.20E-11
TIMP3	-7.153875308	3.21E-11
TMEM176B	-8.555184998	3.44E-11
LINC00888	-2.387346492	3.44E-11
RP11-436K8.1	-9.1534206	3.66E-11
NDRG2	-6.381420359	4.04E-11
SUSD6	-2.126698254	4.08E-11
ZNF572	-8.619138989	4.10E-11
CAMK1	-9.994859989	4.23E-11
ZNF713	-4.053929672	4.51E-11
CRB1	-5.505328506	4.53E-11
PRKAR1B	-3.259574342	4.53E-11
FAM212B	-5.595424626	4.87E-11

SQLE	2.524282569	1.51E-08	CDH19	-9.505711618	5.31E-11
NNMT	6.432835991	1.57E-08	TRIM60P18	-8.481672752	5.59E-11
CPNE7	4.899991934	1.61E-08	PLEKHB1	-3.027593713	5.60E-11
TIPRL	1.877169407	1.63E-08	FAXC	-9.168673541	5.80E-11
NUP62CL	4.744812088	1.73E-08	TAGLN3	-4.519613998	6.20E-11
PFDN2	2.058843105	1.80E-08	DMRT2	-9.000753292	6.27E-11
GATA5	7.949669946	1.85E-08	CYP4F29P	-5.689165553	6.45E-11
EML6	3.425680033	1.85E-08	FAT3	-9.339960752	6.50E-11
PCDHB3	3.758112054	1.86E-08	CADM4	-3.963953463	6.66E-11
TMEM144	4.359403781	1.94E-08	CDH7	-9.825580941	6.92E-11
LSM11	1.6528952	1.98E-08	ZNF704	-3.535780593	7.48E-11
AGAP7P	4.35551378	2.05E-08	ASTN1	-8.68251012	7.52E-11
AREG	7.61528496	2.07E-08	GTF2IP4	-3.201276445	7.59E-11
NEUROG2	7.524682836	2.10E-08	CTD-2561J22.2	-8.181471606	7.99E-11
ROR2	6.574301034	2.26E-08	ADGRB3	-4.848981131	8.56E-11
TCOF1	2.715985488	2.38E-08	CRYAB	-9.02962067	9.05E-11
VWA5B2	7.190222738	2.57E-08	ZNF561-AS1	-2.939397831	9.05E-11
GKAP1	2.219373884	2.61E-08	GTF2IP1	-3.267517506	9.27E-11
SNHG8	3.401142657	2.65E-08	VPS41	-2.261133259	9.57E-11
IL15RA	4.472840219	2.73E-08	EPC2	-2.282359641	1.01E-10
AMN1	1.879947855	2.78E-08	GRB10	-3.972812942	1.02E-10
KISS1	7.941750254	2.87E-08	TMPRSS5	-4.684669572	1.08E-10
PEX6	1.916659702	3.16E-08	HOXD9	-6.410263676	1.10E-10
FXYD5	6.520124096	3.17E-08	ISPD	-8.344366595	1.14E-10
RP11-497G19.2	7.584318915	3.32E-08	TIGAR	-2.393431335	1.16E-10
RHOV	3.636398347	3.79E-08	KIF1A	-7.638648178	1.19E-10
HPCAL1	3.3483159	3.99E-08	ARSE	-8.719077726	1.37E-10
RP11-295G20.2	2.273079322	4.32E-08	TNC	-7.489376663	1.49E-10
GPRC5C	6.533455558	4.39E-08	RP5-882C2.2	-3.146614921	1.50E-10
RHOF	4.348262877	4.42E-08	ATMIN	-1.874946624	1.52E-10
MTBP	1.769542495	4.57E-08	DPY19L2P2	-4.952084237	1.52E-10
RSPO4	6.466341757	4.69E-08	NPL	-9.070858855	1.56E-10
IL15	4.619122369	4.74E-08	KCND2	-8.877509591	1.58E-10
SZT2-AS1	7.536631183	4.74E-08	MIAT	-7.634121988	1.63E-10
IFNLR1	7.070643901	4.75E-08	C14orf37	-3.948436124	1.84E-10
CYP26B1	4.713464233	4.90E-08	PELI2	-6.949413329	1.84E-10
LGALS2	7.361977356	5.03E-08	HIST1H2BN	-3.779246657	1.96E-10
CDH23	6.127350939	5.32E-08	FAM196A	-9.293231276	2.04E-10
SPEF2	3.786749972	5.41E-08	ST3GAL6	-2.436643253	2.20E-10
SLC46A3	4.129271264	5.57E-08	LINC00641	-2.919069966	2.50E-10
STAT6	3.171037153	6.07E-08	PDLIM4	-8.372382622	2.63E-10
GLRX	3.569756984	6.16E-08	ZNF844	-6.085858807	2.68E-10
SMAGP	4.50820343	6.23E-08	FOXN3	-2.317824893	2.92E-10
MATK	7.406494039	6.30E-08	ZNF66	-8.179382212	2.92E-10
TBC1D8B	2.272984517	6.35E-08	ELAVL3	-9.381212236	3.04E-10
NDUFV1	2.133710812	6.47E-08	EPHB3	-5.128718708	3.16E-10
OPLAH	5.898682853	6.56E-08	XKR5	-8.089127271	3.27E-10
ZFAND1	2.270683786	6.65E-08	BEX1	-8.951649524	3.33E-10
MID2	3.741921803	6.95E-08	TPT1-AS1	-2.889017128	3.55E-10
EIF3E	2.189769702	6.97E-08	POLR2D	-1.884638485	3.73E-10
CTD-2207P18.2	7.554943496	6.98E-08	AC114730.3	-8.540168498	3.82E-10
UPK1A-AS1	7.064243249	6.99E-08	ZNF630	-8.26587763	3.83E-10
CTD-2587H24.5	7.513565712	7.00E-08	ZBTB8B	-8.177094174	3.96E-10
ITPR3	3.859249955	7.40E-08	DSC2	-8.144468934	3.99E-10
MYH15	3.529512421	7.61E-08	RP11-444D3.1	-8.218665966	4.12E-10
ATP8B3	4.105206884	7.62E-08	PHLDB1	-2.851358214	4.28E-10
TPRG1	4.362960397	7.82E-08	PLEKHA4	-3.202731542	4.50E-10
FAM86JP	2.88795307	7.83E-08	MOB3B	-3.794909886	4.68E-10
FLNC	5.17668967	7.89E-08	TTLL6	-8.027738674	4.77E-10
CTD-2066L21.3	6.285733224	7.90E-08	NES	-6.06911089	4.89E-10
SEMA3F	3.825659501	8.02E-08	ATP9B	-2.419285766	5.32E-10
NBPF26	2.644124877	8.36E-08	CBX3	-1.863023118	5.44E-10
RP11-1038A11.1	7.25275581	8.52E-08	RGS7BP	-8.748284792	5.58E-10
PLEKHG4	5.867395301	8.60E-08	ST8SIA1	-8.861677802	5.73E-10
INSIG1	1.385068067	9.07E-08	CXXC4	-4.496589375	6.10E-10
RAD21	1.785180942	9.28E-08	CTC-559E9.5	-8.344951059	6.27E-10
LINC00623	3.533898273	9.56E-08	RNF180	-8.866709877	6.69E-10
RP11-89C3.4	7.943650619	1.01E-07	PCDHB5	-8.079950987	6.87E-10

**MSR1**	7.541335162	1.01E-07
**UHMK1**	1.692010055	1.08E-07
**BLVRB**	2.876808058	1.18E-07
**CYB561**	3.092550208	1.23E-07
**DNM1P46**	6.052414083	1.31E-07
**CENPW**	2.491538787	1.42E-07
**LY6E**	2.951091292	1.46E-07
**TMEM134**	2.341927806	1.48E-07
**LINC00689**	7.919931953	1.49E-07
**LINC01162**	7.206055523	1.50E-07
**CCND1**	1.809733394	1.51E-07
**RIN1**	4.552124496	1.54E-07
**RGS10**	2.795463949	1.57E-07
**LINC00702**	6.819882379	1.58E-07
**CTD-2311M21.3**	7.220608546	1.59E-07
**SMTN**	2.681079236	1.62E-07
**P4HA2**	2.693749571	1.76E-07
**AC060834.3**	7.227385695	1.77E-07
**RNF6**	1.582813696	1.81E-07
**RNF212**	6.803707261	1.85E-07
**CTD-2303H24.2**	4.704207831	1.86E-07
**RP11-15A1.2**	6.787720725	1.88E-07
**AADAC**	7.315291823	1.89E-07
**BOK**	2.559645467	1.94E-07
**SMCO4**	4.126781577	1.96E-07
**PRDX6**	1.645290329	2.00E-07
**EOMES**	7.192390853	2.01E-07
**PCED1B**	4.281590813	2.03E-07
**ATP2A3**	4.039885023	2.04E-07
**RP5-965G21.4**	4.610304614	2.04E-07
**RNF169**	2.001124756	2.06E-07
**CDKN3**	1.688374591	2.12E-07
**CXCL2**	6.887738653	2.13E-07
**FBXO6**	3.1018191	2.16E-07
**EGFR-AS1**	7.467470445	2.19E-07
**RALBP1**	1.778190423	2.26E-07
**SEC23A**	1.558129817	2.26E-07
**CXCL1**	6.977692859	2.35E-07
**ZNF233**	3.070973205	2.42E-07
**GLDN**	4.760803465	2.45E-07
**C3orf67**	6.177947413	2.46E-07
**FAM26E**	5.676871117	2.53E-07
**FASN**	3.050996704	2.54E-07
**ZDHHC11B**	2.826765098	2.57E-07
**CYSTM1**	2.171272826	2.61E-07
**CLMP**	3.030158787	2.85E-07
**KCTD13**	2.033754176	2.95E-07
**SLFN5**	4.700280359	3.08E-07
**NKX2-5**	7.317834473	3.14E-07
**FAM169A**	2.008542279	3.20E-07
**BARX2**	7.522678667	3.27E-07
**CGB7**	7.223634926	3.32E-07
**CEP72**	1.882470845	3.36E-07
**FAAHP1**	5.416285714	3.44E-07
**AF241725.6**	7.924878636	3.66E-07
**GINS4**	2.031489432	3.91E-07
**EHBP1L1**	1.792657484	4.05E-07
**DNAJC4**	2.04947275	4.07E-07
**RAP1GAP2**	4.292559392	4.12E-07
**MYO1C**	1.882132997	4.16E-07
**DSCC1**	2.31735151	4.26E-07
**CASZ1**	3.693027205	4.28E-07
**MICE**	3.083714381	4.31E-07
**SLC44A3-AS1**	5.830589531	4.44E-07
**LINC01583**	6.789227152	4.51E-07
**LURAP1L-AS1**	3.789069238	4.54E-07
**SCN5A**	4.505321824	4.61E-07
**ACSL4**	2.253050982	4.65E-07

**COTL1**	-3.055208784	6.94E-10
**NT5C3A**	-3.028047386	7.01E-10
**ADAMTS9**	-7.090363115	7.01E-10
**ZMAT3**	-2.642406905	7.10E-10
**RNF150**	-3.959658885	7.11E-10
**XKR6**	-8.041604331	7.17E-10
**DYRK1B**	-3.23554565	7.21E-10
**FAM198A**	-8.460612073	7.23E-10
**NRCAM**	-6.213205884	7.44E-10
**RABIF**	-1.955012276	8.39E-10
**GSTM2**	-3.604770705	8.43E-10
**C1orf216**	-2.517013065	9.32E-10
**RTKN**	-2.49647924	9.56E-10
**RP11-398K22.1[illegible]**	-2.943377545	9.86E-10
**RP9**	-2.602651596	9.96E-10
**MSI2**	-2.047638975	1.02E-09
**RP11-159D12.2**	-2.798991565	1.02E-09
**C8orf88**	-4.039531664	1.03E-09
**RP1-40E16.9**	-8.392876913	1.07E-09
**SLC44A5**	-8.087998331	1.24E-09
**TMEM198**	-4.741158452	1.24E-09
**DCHS2**	-9.574562666	1.25E-09
**BBS9**	-2.466197549	1.32E-09
**KIAA1456**	-8.305725189	1.36E-09
**FREM1**	-6.784375309	1.45E-09
**CTC-260E6.6**	-8.030632831	1.49E-09
**HIP1**	-3.591111157	1.55E-09
**MURC**	-2.442062179	1.70E-09
**SLC26A4**	-3.015547134	1.72E-09
**ZNF827**	-2.468447492	1.75E-09
**SOGA1**	-1.855397591	1.77E-09
**GATS**	-3.246205848	1.80E-09
**GULP1**	-2.526194695	1.91E-09
**GPC6**	-2.803358148	1.92E-09
**ANKRD44**	-3.196667075	1.94E-09
**EPHX2**	-8.04684561	1.96E-09
**ELN**	-8.936451314	1.98E-09
**ZNF680**	-2.663909908	1.98E-09
**GPC2**	-4.058467056	2.00E-09
**FRAS1**	-8.252173315	2.05E-09
**MEOX2**	-8.673562863	2.11E-09
**CHI3L2**	-5.647455583	2.12E-09
**GPR37L1**	-8.395592638	2.16E-09
**ZNF454**	-6.438354007	2.17E-09
**SLC18A1**	-8.004944337	2.19E-09
**AC092835.2**	-5.100191558	2.35E-09
**HSBP1**	-1.822454641	2.36E-09
**RFX4**	-7.937190342	2.49E-09
**GPCPD1**	-2.600260486	2.66E-09
**KIAA1211L**	-8.264788865	2.68E-09
**MARCKSL1**	-2.669447732	2.69E-09
**MIR3681HG**	-8.879008143	2.81E-09
**BCL2L11**	-2.97836296	2.82E-09
**ZNF529-AS1**	-5.273696736	2.83E-09
**DUSP26**	-8.525617216	2.95E-09
**PLPP3**	-5.627070568	2.96E-09
**PHLDA3**	-7.570852467	3.02E-09
**FILIP1L**	-7.662232146	3.21E-09
**KCNE4**	-7.935718471	3.38E-09
**FAM212B-AS1**	-6.817557936	3.47E-09
**JARID2**	-1.748312874	3.53E-09
**ZNF724**	-7.444310404	3.66E-09
**NGFR**	-6.462276435	4.07E-09
**TMEM179**	-9.231262094	4.07E-09
**GTF2IRD2B**	-3.719785254	4.12E-09
**ZNF362**	-2.57010904	4.24E-09
**ABAT**	-4.674580115	4.30E-09
**TMEM154**	-8.223809462	4.37E-09

**GSDMD**	3.635684488	4.65E-07
**FGFR4**	4.799967876	4.69E-07
**ELOVL5**	2.212994865	4.89E-07
**FRRS1**	2.964843836	4.93E-07
**KCNK13**	4.974536378	5.17E-07
**CHD5**	5.376680068	5.23E-07
**RP11-656D10.3**	3.355014087	5.27E-07
**CBX7**	2.601754525	5.44E-07
**DARS2**	2.296150259	5.48E-07
**PRR16**	5.600692946	5.50E-07
**RP11-195E2.1**	7.057265458	5.59E-07
**LVCAT1**	5.211867124	6.09E-07
**TUBB6**	3.27415385	6.31E-07
**BIK**	5.971804006	6.31E-07
**TUFT1**	2.06422218	6.32E-07
**APOBEC3C**	2.037104548	6.34E-07
**NOVA1-AS1**	2.538877294	6.37E-07
**SBSN**	5.833322417	6.48E-07
**NDC80**	1.885478018	6.58E-07
**MICB**	4.316847305	6.70E-07
**OSMR-AS1**	4.848799816	7.28E-07
**HLA-C**	2.254181172	7.31E-07
**HCFC1R1**	2.47347224	7.42E-07
**CDC25C**	1.700115583	7.59E-07
**SEC24D**	1.699647125	7.72E-07
**TRMT12**	1.710909702	8.02E-07
**SLFN12L**	6.520704362	8.22E-07
**MIR548XHG**	6.517723896	8.96E-07
**RP11-495K9.9**	6.202264434	8.96E-07
**TFAP2C**	4.732647929	9.35E-07
**CLEC2B**	2.653662736	9.42E-07
**SAMD12**	6.111494055	9.50E-07
**SIGIRR**	4.589014124	9.60E-07
**ETNK2**	1.587366752	9.65E-07
**AC007773.2**	4.236120734	1.03E-06
**AC156455.1**	4.687323369	1.04E-06
**RP11-25H12.1**	6.900994063	1.07E-06
**VAV1**	6.485448416	1.07E-06
**SP8**	6.662064717	1.07E-06
**KIF22**	2.035682696	1.08E-06
**CCBE1**	7.699538682	1.10E-06
**AP3S1**	1.833769151	1.11E-06
**DCAF6**	1.605474642	1.12E-06
**WIPI1**	2.199242472	1.12E-06
**FCHO1**	2.776417965	1.13E-06
**MT2A**	3.685100979	1.13E-06
**GCLM**	2.924993181	1.14E-06
**NOTUM**	5.056976923	1.15E-06
**PER1**	3.098570984	1.20E-06
**SEH1L**	2.020635887	1.21E-06
**SHCBP1**	1.510681798	1.22E-06
**CYP4F26P**	5.220397301	1.28E-06
**PLEKHN1**	5.383533565	1.28E-06
**COPA**	1.36073993	1.32E-06
**PDIA5**	2.017252978	1.36E-06
**PLAG1**	3.499824371	1.37E-06
**BATF3**	2.312783236	1.38E-06
**FAM131C**	4.525550992	1.44E-06
**LINC00659**	6.365318386	1.52E-06
**ASL**	2.032431243	1.53E-06
**TM4SF1**	2.042896698	1.53E-06
**ICAM5**	4.104295751	1.55E-06
**CLDN1**	4.924402347	1.56E-06
**ESYT2**	1.269970749	1.59E-06
**NKD2**	4.543978384	1.60E-06
**RAB27A**	1.793352869	1.61E-06
**RP13-463N16.6**	7.120883305	1.64E-06
**REEP6**	2.747845644	1.64E-06

**ATL1**	-3.003787884	4.39E-09
**MTTP**	-5.89335954	4.46E-09
**ABHD17B**	-1.733436895	4.83E-09
**C1orf61**	-6.822194754	4.91E-09
**TMEM108**	-4.299944648	4.96E-09
**CALCRL**	-5.410894749	5.14E-09
**AMER2**	-8.862580022	5.22E-09
**LINC02199**	-7.534188611	5.40E-09
**NEDD9**	-7.188475463	5.53E-09
**RHBDD2**	-3.845511535	5.83E-09
**TECTA**	-7.743850242	5.96E-09
**ZIC4**	-8.146380871	6.18E-09
**RP11-428P16.2**	-7.86775702	6.18E-09
**PLXNA4**	-8.48024317	6.30E-09
**FBN1**	-4.137277214	6.33E-09
**RP11-71N10.1**	-8.619040808	6.40E-09
**ZNF781**	-7.818880084	6.49E-09
**RP11-15H20.6**	-5.437567657	6.65E-09
**C1orf94**	-8.827077991	6.75E-09
**TCF7L1**	-1.887735669	7.13E-09
**NEU4**	-8.102452312	7.29E-09
**FGF13**	-7.459756937	7.48E-09
**LINC01550**	-8.085630697	7.55E-09
**FOXO1**	-1.923665028	7.86E-09
**RNF157**	-3.845677675	8.58E-09
**NOMO3**	-1.97768309	8.59E-09
**NSUN5P1**	-2.567205263	9.04E-09
**HFM1**	-3.707314173	9.08E-09
**YPEL3**	-5.0035072	9.09E-09
**EFNB2**	-3.784330275	9.60E-09
**EIF4EBP2**	-1.583543798	9.86E-09
**ZNF582-AS1**	-7.903489168	9.86E-09
**ART3**	-8.92987239	9.90E-09
**DYRK1A**	-1.57302506	1.10E-08
**ARPP21**	-8.037769413	1.10E-08
**RP11-255H23.2**	-6.803708505	1.10E-08
**HOXA7**	-6.678710098	1.11E-08
**LINC01535**	-7.587569134	1.12E-08
**HYDIN2**	-7.68485573	1.26E-08
**NOVA1**	-3.14922289	1.31E-08
**FBN3**	-9.076354861	1.35E-08
**MRPS17**	-2.813500722	1.36E-08
**TSC22D1**	-2.753393057	1.38E-08
**HOXD8**	-8.425698446	1.39E-08
**NALCN**	-7.485100974	1.39E-08
**THBS2**	-6.224305204	1.46E-08
**ATP10A**	-9.044377242	1.50E-08
**RAD54L2**	-1.652976576	1.59E-08
**URM1**	-2.854212069	1.63E-08
**EMILIN3**	-8.295601114	1.65E-08
**TRIM24**	-2.676643359	1.70E-08
**LINC00403**	-7.699092752	1.72E-08
**GPC3**	-8.395769478	1.75E-08
**RIC3**	-7.784436663	1.90E-08
**PIP5K1B**	-8.099273	1.95E-08
**NPR2**	-3.536623637	1.98E-08
**MTURN**	-2.353547582	1.99E-08
**RCOR2**	-2.846955055	2.15E-08
**BCORL1**	-1.797160233	2.18E-08
**KCND3**	-8.744996697	2.18E-08
**LZTS1**	-4.845174792	2.20E-08
**PCDHB11**	-6.626659834	2.21E-08
**TRIM9**	-3.555181903	2.26E-08
**CDC42SE1**	-1.423495394	2.34E-08
**AVL9**	-2.031096303	2.38E-08
**CTD-2245F17.3**	-8.32753367	2.40E-08
**AHCYL2**	-3.107472138	2.54E-08
**C1orf56**	-5.000468537	2.65E-08

CSAG4	6.892698854	1.65E-06
CFH	4.434859221	1.65E-06
GPR39	5.688826339	1.65E-06
CLINT1	1.459013568	1.67E-06
ICOSLG	4.677805147	1.73E-06
ZBTB7B	1.825271449	1.77E-06
GRIP1	6.814249783	1.79E-06
DERL1	1.81614536	1.81E-06
DHRS3	6.676793674	1.84E-06
C10orf35	3.689413158	1.84E-06
IER2	2.046300321	1.87E-06
PLBD1-AS1	6.911744437	1.87E-06
NUDT8	3.004048672	1.91E-06
ZC3H12A	2.846977729	1.98E-06
ERRFI1	2.245704251	1.99E-06
MRPL13	2.013787244	2.00E-06
FAM95C	6.86653314	2.08E-06
ISL1	6.806063501	2.12E-06
ZNF692	1.745599356	2.27E-06
COL17A1	6.70896012	2.34E-06
OSBPL10	1.898111641	2.34E-06
LINC01036	6.818139675	2.35E-06
CCDC7	2.031794083	2.35E-06
LINC01273	5.680137692	2.37E-06
RNF139-AS1	2.092008006	2.49E-06
TCAM1P	7.293955208	2.51E-06
ATAD2	1.758292203	2.57E-06
RP11-284F21.7	4.128882055	2.63E-06
CTC1	1.799988321	2.65E-06
ALOX12B	5.352522856	2.66E-06
PRSS16	4.846260439	2.66E-06
CGB2	6.747292713	2.68E-06
RP11-402J6.1	6.757541223	2.72E-06
LMLN-AS1	5.648446421	2.82E-06
RP11-519M16.1	6.69772685	2.85E-06
LACTB2	1.79429672	2.86E-06
CEP295	1.572774351	2.95E-06
SLC7A5	2.344790914	3.00E-06
LINC00460	3.729442416	3.14E-06
ASPH	2.246488196	3.23E-06
RP11-49I11.1	3.617485545	3.44E-06
SPRYD3	2.01858611	3.45E-06
MTND6P21	6.767640478	3.51E-06
EDARADD	4.151564967	3.53E-06
RP11-66B24.1	6.774858729	3.65E-06
PSMG2	1.600887281	3.68E-06
RP11-575F12.1	6.731795954	3.72E-06
LRRC73	3.648509954	3.74E-06
GRK3	2.903328225	3.76E-06
P2RY2	4.956249106	3.85E-06
TSPAN2	6.937036044	3.93E-06
RP11-148O21.4	6.908853839	3.99E-06
RP11-326A19.4	4.369169949	4.03E-06
PLP2	2.453788251	4.12E-06
IFT57	1.45190077	4.23E-06
MGAT4B	1.432445544	4.32E-06
C8orf59	1.726836254	4.36E-06
HCG25	2.351298305	4.45E-06
PRDM15	1.324954751	4.56E-06
RP11-268G12.3	4.200333373	4.56E-06
WNT2B	3.059556555	4.99E-06
METTL23	1.355119318	5.01E-06
RP11-403I13.5	6.607682454	5.12E-06
RP11-802E16.3	3.829069566	5.16E-06
HOXB4	4.02657126	5.22E-06
LBX2	5.427944399	5.25E-06
MCUB	2.459460478	5.40E-06
ABHD14B	1.769971385	5.59E-06

HOXD11	-6.65021582	2.73E-08
CTD-2291D10.4	-7.697930084	2.78E-08
IDUA	-7.665887637	2.81E-08
RP11-448P19.1	-8.466375954	2.88E-08
BDH2	-2.602569511	2.91E-08
RGS6	-8.116777337	2.91E-08
TOR1A	-2.750079149	3.11E-08
FARP1	-2.829871658	3.18E-08
DAB1	-8.110970716	3.31E-08
PXDN	-1.571777527	3.34E-08
UGGT1	-1.915996479	3.57E-08
SSBP3	-1.912885399	3.64E-08
GNAI1	-2.266042737	3.70E-08
ATAT1	-1.772771174	3.82E-08
ZC3H4	-1.829162559	3.83E-08
MCAM	-2.717888117	3.92E-08
KLRC4-KLRK1	-7.827849964	3.93E-08
GPX3	-7.586653029	4.02E-08
TECPR2	-2.859290907	4.14E-08
ANKRD7	-6.584471336	4.16E-08
APBB2	-2.016174325	4.24E-08
CASP2	-1.930698257	4.25E-08
RP11-472N13.3	-7.835931094	4.56E-08
LINC01105	-8.210805291	4.78E-08
HIST1H2AC	-2.827217506	4.87E-08
RP4-756G23.5	-7.97797169	5.04E-08
SNTG1	-8.861365597	5.27E-08
PTPRD-AS1	-7.51830869	5.39E-08
FKBP9	-4.452799388	5.47E-08
CALM1	-3.04562266	5.56E-08
RP11-496D24.2	-7.031095606	5.62E-08
C1orf54	-6.838687744	5.86E-08
MYO16	-8.760883412	5.90E-08
NAA16	-1.696138497	6.02E-08
CREB3L1	-8.518968892	6.58E-08
TAOK3	-2.790647027	6.63E-08
PLCG1	-2.005319028	6.65E-08
MMP16	-2.885515091	6.65E-08
KCNAB3	-3.517345913	6.77E-08
GLI3	-2.693784032	6.78E-08
SYT11	-3.310530806	6.79E-08
PLEKHB2	-1.469004499	6.89E-08
TNS3	-5.113245881	7.14E-08
MAMDC2-AS1	-3.933905485	7.18E-08
STARD9	-1.495835056	7.29E-08
TMEM170A	-1.92049578	7.48E-08
HIBADH	-1.630515359	7.53E-08
NEB	-5.359146876	7.60E-08
TSPAN11	-8.615608089	7.61E-08
LINC00265	-1.778749128	7.71E-08
DOK5	-7.796575297	7.73E-08
DPP6	-7.448093195	7.77E-08
TSN	-1.817214094	8.25E-08
SRD5A1	-1.953186578	8.28E-08
STK33	-8.564881991	8.35E-08
ARHGAP21	-1.782212896	8.36E-08
RPE65	-7.032590115	8.47E-08
LDLRAD3	-2.164772284	8.53E-08
RAMP2-AS1	-8.60775794	8.64E-08
PARP11	-2.454731806	8.87E-08
ZSCAN21	-1.697076628	9.29E-08
ITGA11	-8.065481368	1.00E-07
HOXB13	-8.108059774	1.08E-07
MEX3A	-1.907301753	1.09E-07
CNTFR	-7.594799677	1.15E-07
DDN	-6.485112482	1.16E-07
IPO9	-1.691754838	1.18E-07
FTX	-2.006892193	1.18E-07

AKAP12	3.582451821	5.60E-06	IL6ST	-2.547443899	1.18E-07
RP1-28O10.1	6.740643804	5.65E-06	FHIT	-3.500481858	1.19E-07
NFKB2	1.785925269	5.75E-06	DSCAML1	-7.267352456	1.21E-07
CA13	1.724515939	5.85E-06	MOAP1	-1.649148768	1.25E-07
PQLC3	1.511373337	6.02E-06	GPR75	-3.191995755	1.26E-07
ZNF280A	6.254061821	6.03E-06	LMO4	-2.141140155	1.26E-07
INTS8	1.768235684	6.10E-06	ZNF117	-4.120366935	1.28E-07
SLC9A7	2.387908572	6.12E-06	CLEC4F	-5.476451894	1.28E-07
RNF19B	2.087396167	6.12E-06	RFC2	-2.343146498	1.32E-07
LINC01037	6.270344811	6.20E-06	KATNAL2	-3.382314823	1.36E-07
RP11-64D22.5	6.670237088	6.25E-06	ID3	-3.408057246	1.45E-07
MSRB3	2.358771729	6.61E-06	FMNL2	-2.0567261	1.48E-07
PTHLH	4.026781908	6.74E-06	LINC01415	-8.371321319	1.48E-07
RHBDD1	1.633073166	7.03E-06	DACT3	-4.782381239	1.49E-07
RP3-522D1.1	3.049267853	7.07E-06	ZKSCAN3	-2.032009982	1.50E-07
PLGRKT	1.75562491	7.40E-06	TOX3	-8.513050805	1.50E-07
DOC2GP	4.240544738	7.46E-06	MAPRE2	-2.258173208	1.55E-07
CHTF18	2.729195271	7.49E-06	PHTF2	-2.110360977	1.56E-07
CAV2	2.607289905	7.52E-06	ZNF429	-4.22913794	1.58E-07
LINC01703	3.063371686	7.75E-06	ETV1	-2.039385166	1.60E-07
LINC01186	6.337343389	7.82E-06	DPYSL5	-2.41613775	1.60E-07
PRKAG2	3.10829687	7.97E-06	PCDHB12	-4.445327016	1.60E-07
SACS	2.252386019	8.15E-06	RAMP2	-7.946178774	1.62E-07
HSD17B14	3.767280143	8.33E-06	C7orf49	-1.897189854	1.68E-07
DHRS2	3.877385968	8.48E-06	CRABP1	-8.200511103	1.72E-07
STK3	1.842450934	8.49E-06	GSK3B	-1.865462316	1.81E-07
BCRP2	6.089677043	8.92E-06	MLYCD	-2.358479961	1.84E-07
DNAH17-AS1	6.671372493	8.96E-06	ADCYAP1R1	-8.568914004	1.84E-07
AADACP1	7.060145573	9.01E-06	EP300	-1.649613285	1.87E-07
GAPLINC	6.488946566	9.04E-06	SLC25A29	-2.248481258	1.87E-07
GGH	1.690024189	9.13E-06	KMT2C	-1.997615406	1.89E-07
LL22NC03-86G7	4.727001344	9.25E-06	HAND2-AS1	-7.871059918	1.90E-07
NUF2	1.692206242	9.42E-06	CHRM1	-7.240024674	1.90E-07
AC073130.3	6.442162012	9.95E-06	TSPAN12	-3.791941242	2.02E-07
PCDHB6	4.284167385	1.01E-05	KLHDC8A	-7.188530969	2.07E-07
VAT1L	7.489768673	1.05E-05	TSPAN14	-2.315003229	2.08E-07
SLC7A11	2.225019393	1.05E-05	NINJ2	-6.911686183	2.15E-07
CRNDE	1.661141577	1.07E-05	LRRC4C	-4.841782525	2.19E-07
XXyac-YM21GA	6.615020672	1.08E-05	SNN	-4.293138139	2.19E-07
SYNGR2	3.255089625	1.09E-05	HES1	-6.804834893	2.20E-07
GATA3	4.587389185	1.10E-05	CTC-543D15.8	-7.521818143	2.35E-07
CH17-472G23.2	3.37406725	1.11E-05	ZNF790-AS1	-7.2818115	2.38E-07
RP11-54A9.1	7.48207227	1.12E-05	MTFR1L	-1.630826464	2.48E-07
PPP2R3A	1.839795585	1.12E-05	ATP2B1	-2.479504465	2.50E-07
MTFR1	1.679659048	1.12E-05	GPR156	-5.196448605	2.51E-07
KHDRBS2	6.558804198	1.13E-05	GPR155	-3.944168043	2.57E-07
ALDH7A1	1.64177603	1.13E-05	PIPOX	-6.204250135	2.58E-07
UTP23	1.74213718	1.15E-05	SCN3A	-6.130215416	2.60E-07
RP5-1139I1.1	6.931226549	1.15E-05	CA8	-7.615048346	2.67E-07
SNHG6	1.619245894	1.16E-05	BAG5	-1.463471935	2.69E-07
RILP	2.948389258	1.16E-05	CDKN2C	-2.585768632	2.71E-07
ADAMTS2	6.835618425	1.19E-05	PLEKHH2	-2.333159992	2.95E-07
MSC	6.418676483	1.20E-05	CTD-2017D11.1	-3.960102808	2.97E-07
PTPRF	2.346133855	1.20E-05	ARGLU1	-1.907938535	2.99E-07
TMEM244	6.446484348	1.22E-05	PTCH1	-2.850126841	2.99E-07
CORO1B	2.39135338	1.23E-05	PMP22	-1.686197621	3.16E-07
RECQL	1.531731314	1.24E-05	VAMP5	-7.736934812	3.24E-07
ARHGAP29	4.987765888	1.26E-05	CDH2	-3.41840142	3.32E-07
RAET1E	5.356798537	1.26E-05	RP11-290O12.2	-7.111607088	3.32E-07
TTPA	1.908082052	1.27E-05	GAS2	-5.162235767	3.33E-07
DCUN1D5	2.108779238	1.32E-05	MAP1A	-4.195995788	3.39E-07
CWF19L2	1.61379868	1.33E-05	NAALAD2	-7.935443856	3.40E-07
RP11-162D16.2	6.403115552	1.33E-05	RASSF2	-2.877848345	3.47E-07
IGSF1	5.118692232	1.34E-05	LRP4	-3.769580609	3.49E-07
BTNL8	7.586761091	1.36E-05	RP9P	-3.261884755	3.67E-07
MAP3K14	2.040029072	1.39E-05	IFI27L1	-2.498331197	3.69E-07
RP11-60A8.1	7.059636969	1.42E-05	PPP1R1B	-8.430361447	3.74E-07
MYL12A	2.238812181	1.42E-05	RP11-329B9.4	-3.828846578	3.74E-07

**DYRK3**	1.566193026	1.43E-05
**RP11-462L8.1**	2.684911141	1.43E-05
**SLC1A5**	2.455219237	1.45E-05
**SLC22A5**	3.356202981	1.46E-05
**CCT4**	1.474237762	1.51E-05
**RNMT**	1.890106209	1.51E-05
**RP11-535M15.1**	6.836140007	1.53E-05
**SPOCK3**	6.329898052	1.53E-05
**MSL3P1**	6.358039667	1.60E-05
**HSD11B2**	5.026868	1.60E-05
**ADCY7**	2.979349539	1.61E-05
**MPP3**	1.949467635	1.61E-05
**NUDT22**	1.845685503	1.64E-05
**PLSCR1**	1.572589187	1.64E-05
**CD7**	7.099517001	1.67E-05
**CFD**	4.508607275	1.67E-05
**METTL4**	1.536763995	1.67E-05
**RP11-134G8.5**	3.127231423	1.71E-05
**NEBL**	4.522355671	1.73E-05
**NEK2**	1.397007791	1.74E-05
**LINC01694**	4.575615416	1.74E-05
**RPL7**	1.459415794	1.83E-05
**TMEM14A**	2.036706921	1.83E-05
**MARCH3**	2.209893142	1.84E-05
**ITGB4**	3.640100631	1.84E-05
**NRIP3**	4.999236115	1.84E-05
**CEP57**	1.613756419	1.93E-05
**RHPN2**	1.431673136	1.93E-05
**FBXL8**	1.733355167	1.93E-05
**IGFN1**	5.37807095	1.93E-05
**ANKRD34A**	1.846622896	1.95E-05
**RP11-148O21.3**	6.106927561	2.05E-05
**NOXA1**	4.590671454	2.06E-05
**CHCHD10**	1.685787785	2.07E-05
**JMJD6**	1.53374278	2.07E-05
**TPD52**	2.862086799	2.08E-05
**CIB1**	1.975761493	2.09E-05
**FAM227A**	2.020644878	2.13E-05
**YARS2**	1.801488105	2.18E-05
**EGFL7**	3.38536721	2.19E-05
**RP13-977J11.2**	4.165965675	2.22E-05
**HIVEP2**	2.651215167	2.24E-05
**TRAF1**	2.355712267	2.24E-05
**GPRC5A**	2.739553481	2.26E-05
**PGM5P2**	1.910117592	2.30E-05
**XKR9**	3.57442536	2.41E-05
**ENO1-IT1**	2.386887831	2.46E-05
**IRX3**	2.442739043	2.47E-05
**FAM72D**	2.224275534	2.49E-05
**RGS5**	2.484751525	2.49E-05
**SENCR**	6.08542527	2.56E-05
**LDOC1**	1.759039104	2.59E-05
**SYCP2L**	4.087907768	2.59E-05
**CARMIL2**	3.323888319	2.66E-05
**FAM91A1**	1.674245702	2.72E-05
**ZNF438**	1.506994198	2.75E-05
**GDPD5**	3.08011749	2.81E-05
**EPS8L2**	3.084997859	2.89E-05
**NAP1L3**	3.01901456	2.91E-05
**AK7**	3.84364141	2.99E-05
**ASAP2**	1.226637135	3.03E-05
**TRIB3**	2.504197396	3.09E-05
**PRELID2**	2.769968727	3.11E-05
**HSPA1L**	2.084791325	3.12E-05
**LAS1L**	1.782298979	3.15E-05
**C6orf1**	2.608738581	3.16E-05
**HIF1A-AS2**	6.404846376	3.20E-05
**LAMP3**	4.373717262	3.20E-05

**PREX1**	-3.119859533	3.81E-07
**C3AR1**	-7.681887624	3.88E-07
**SECISBP2L**	-1.795157252	4.09E-07
**C21orf62**	-8.318048444	4.12E-07
**ELAVL4**	-7.968105052	4.13E-07
**EIF4H**	-1.658449733	4.28E-07
**TSC22D4**	-2.930594811	4.35E-07
**SETBP1**	-7.623236886	4.36E-07
**DDR2**	-2.897682051	4.44E-07
**STMN3**	-4.766113492	4.59E-07
**RP11-488C13.5**	-2.246146887	4.77E-07
**NNT-AS1**	-2.064197224	5.02E-07
**CHRNA1**	-8.095522574	5.03E-07
**CLASP1**	-1.87962099	5.14E-07
**STXBP6**	-3.537600572	5.23E-07
**GRIK1**	-8.236882397	5.27E-07
**TCF12**	-1.720337644	5.29E-07
**SELENOP**	-6.646577824	5.44E-07
**SRGAP3**	-3.750123528	5.48E-07
**DGKB**	-7.637144541	5.55E-07
**LINC00391**	-7.146426634	5.71E-07
**CTA-293F17.1**	-4.702427301	5.76E-07
**AC010729.1**	-7.260223812	5.96E-07
**IGF2R**	-2.053806072	6.01E-07
**FABP3**	-7.747128444	6.13E-07
**ZNF428**	-2.728824689	6.19E-07
**CDHR1**	-7.499802815	6.31E-07
**MEGF9**	-2.374344931	6.31E-07
**YAE1D1**	-1.510469401	6.41E-07
**IRS2**	-1.791708871	6.76E-07
**USP11**	-1.909865811	6.84E-07
**RP11-617F23.1**	-3.124766954	6.90E-07
**UPF3A**	-1.593880475	6.92E-07
**CBX2**	-1.938858457	7.31E-07
**MARVELD1**	-1.944570686	7.37E-07
**KLRC4**	-7.755224737	7.61E-07
**WIPF3**	-6.084224839	7.77E-07
**GTF2IRD2**	-3.802822639	7.78E-07
**USP32P3**	-2.482787701	8.01E-07
**ACP2**	-2.20655722	8.09E-07
**C6orf52**	-3.754987247	8.14E-07
**RP11-480I12.9**	-3.362314911	8.24E-07
**WDR91**	-1.961959135	8.27E-07
**GOLIM4**	-1.766557423	8.38E-07
**ZNF726**	-5.148445832	8.73E-07
**RP11-611L7.2**	-4.304779814	8.76E-07
**RAB39A**	-2.63991549	9.06E-07
**DIP2B**	-1.988339997	9.50E-07
**PNMAL2**	-7.418551756	9.58E-07
**RNASE1**	-6.964960939	9.83E-07
**EYA4**	-8.961759465	9.99E-07
**RP11-277P12.20**	-2.86194715	1.03E-06
**RNF122**	-2.087607451	1.03E-06
**SATB2**	-2.653640328	1.04E-06
**ARHGAP31**	-2.013985984	1.04E-06
**ZNF582**	-3.09410555	1.06E-06
**SPP1**	-8.555276201	1.07E-06
**RP11-966I7.2**	-7.196263744	1.09E-06
**ZKSCAN2**	-1.963998383	1.10E-06
**PTPRA**	-1.998838714	1.10E-06
**B4GALNT3**	-4.457473266	1.12E-06
**DOLPP1**	-2.518584887	1.15E-06
**ZNF157**	-5.307676041	1.20E-06
**WWTR1**	-3.078049584	1.22E-06
**PLCH1**	-4.604689477	1.27E-06
**ENTPD1**	-5.669436679	1.28E-06
**H2AFV**	-1.989108523	1.28E-06
**RP11-25K19.1**	-5.405367611	1.31E-06

**FMN2**	2.896931178	3.22E-05
**SLU7**	1.473450764	3.22E-05
**HEBP2**	1.588958192	3.35E-05
**RP11-527N22.2**	5.339134282	3.42E-05
**TMED3**	1.539236874	3.59E-05
**IL6R**	2.767996341	3.61E-05
**ARFGEF1**	1.772181883	3.69E-05
**GOT1**	1.431330006	3.74E-05
**SDC4**	1.475645055	3.74E-05
**PSTPIP2**	2.035974703	3.75E-05
**CTC-378H22.1**	6.534646783	3.76E-05
**SCNN1A**	6.306270098	3.76E-05
**MYH9**	1.611472476	3.78E-05
**MRE11**	1.47349534	3.78E-05
**KCTD8**	4.173064287	3.79E-05
**CT45A1**	6.249095502	3.82E-05
**SPAG5**	1.444661329	3.84E-05
**FAM72B**	2.151622872	3.85E-05
**RP11-686D22.8**	6.198503793	3.99E-05
**C19orf12**	1.45752136	4.01E-05
**KHNYN**	1.433067726	4.02E-05
**MFSD13B**	5.779481009	4.10E-05
**SLC22A4**	3.393892488	4.12E-05
**LIF**	3.366762223	4.20E-05
**CTDSPL**	1.34538877	4.21E-05
**FBXO28**	1.629398402	4.25E-05
**NSMCE2**	1.72261495	4.29E-05
**CTF1**	3.875202164	4.31E-05
**DDX10**	1.725076038	4.31E-05
**MAGEA2**	6.225964748	4.32E-05
**RP11-531A24.5**	2.303603847	4.33E-05
**BIRC3**	4.058512438	4.34E-05
**GABRD**	4.418032104	4.42E-05
**LINC00706**	6.476287527	4.44E-05
**PTPN2**	2.132937987	4.57E-05
**HSD17B11**	1.424828471	4.58E-05
**SERPINA1**	5.951116786	4.60E-05
**F8**	1.958369945	4.61E-05
**ZFYVE19**	2.195482656	4.68E-05
**FSIP2-AS1**	3.101133259	4.68E-05
**ANKRD20A21P**	5.8115734	4.74E-05
**AC012485.2**	5.923739888	4.79E-05
**ARHGAP19**	1.214350342	4.81E-05
**BAIAP2**	2.052515418	4.82E-05
**COA6**	1.770294751	4.90E-05
**AC007879.5**	3.074641483	4.97E-05
**TMEM151A**	4.304951232	5.04E-05
**WDYHV1**	1.76547478	5.05E-05
**AARS**	1.429283699	5.07E-05
**RPL30**	1.484595814	5.11E-05
**ABCB7**	1.540121436	5.11E-05
**KCNH3**	4.130173125	5.11E-05
**TRIM7**	2.819998212	5.32E-05
**LINC01006**	3.63077232	5.60E-05
**SLC16A7**	5.556371159	5.66E-05
**SYNPO**	2.573724643	5.76E-05
**GPR18**	6.108106477	5.76E-05
**CMBL**	1.123094417	5.88E-05
**RAB3B**	2.334459043	5.93E-05
**CEP126**	4.765219743	5.93E-05
**LRRFIP1**	1.871229807	5.98E-05
**AC079922.3**	1.956902709	5.98E-05
**WDR4**	1.877400742	6.14E-05
**DPM3**	2.452919188	6.16E-05
**CH17-472G23.4**	1.693241182	6.19E-05
**SLC22A31**	6.404361955	6.21E-05
**TESC**	4.034938023	6.24E-05
**TRIM6**	3.341998393	6.33E-05

**SSPN**	-5.155750258	1.33E-06
**SNX18**	-2.917330113	1.33E-06
**A2M**	-7.803565545	1.36E-06
**FAM69C**	-7.049023183	1.36E-06
**E2F3**	-2.939029264	1.38E-06
**STIM2**	-1.92396479	1.38E-06
**CPVL**	-6.316671589	1.39E-06
**PCDHGA9**	-6.37877329	1.43E-06
**ZNF570**	-2.405767216	1.44E-06
**RP11-4O1.2**	-4.615001879	1.45E-06
**GS1-24F4.2**	-6.779841943	1.45E-06
**IQGAP2**	-6.45493982	1.51E-06
**ZNF436**	-2.105896892	1.55E-06
**MVB12B**	-1.836275973	1.57E-06
**FNTB**	-1.997061983	1.60E-06
**MMP11**	-4.666912154	1.60E-06
**MAP1LC3A**	-7.379274997	1.78E-06
**AGMO**	-6.104132665	1.81E-06
**MYEF2**	-2.082989992	1.84E-06
**AP002954.3**	-3.351638835	1.87E-06
**UTP14C**	-1.529938232	1.92E-06
**ZSWIM6**	-2.403045418	2.02E-06
**H19**	-5.533234603	2.07E-06
**ABCA12**	-6.827329406	2.15E-06
**ZNF430**	-1.528009521	2.21E-06
**LANCL2**	-2.013564318	2.23E-06
**KCNIP2**	-2.6298532	2.25E-06
**PEAK1**	-1.85409197	2.26E-06
**RP11-480I12.5**	-2.908564525	2.28E-06
**NR2F1-AS1**	-2.868865851	2.32E-06
**LYNX1**	-7.396014617	2.35E-06
**AK1**	-2.930008372	2.39E-06
**PHACTR1**	-6.182714971	2.39E-06
**ZNF425**	-3.506601368	2.43E-06
**PRSS35**	-7.592427111	2.44E-06
**DENND5A**	-1.518925004	2.45E-06
**SEMA4A**	-3.958432841	2.46E-06
**ADGRL2**	-4.221479784	2.51E-06
**CXCR4**	-5.419354816	2.53E-06
**ABTB2**	-2.737344682	2.57E-06
**NCOA5**	-1.940734617	2.57E-06
**CDK2AP1**	-2.00372139	2.57E-06
**LY6G5C**	-4.083229345	2.57E-06
**NUDT3**	-1.637251599	2.61E-06
**ZC3H7A**	-1.556624236	2.63E-06
**SORCS3**	-7.475381403	2.64E-06
**GBGT1**	-7.258447304	2.72E-06
**DNM3OS**	-7.790383001	2.74E-06
**TRIM46**	-2.255560482	2.76E-06
**FAM110C**	-6.868585317	2.79E-06
**DAGLA**	-2.215702492	2.83E-06
**PNMA6A**	-6.664836396	2.84E-06
**SERHL2**	-6.70343082	2.88E-06
**RP11-1217F2.1**	-2.58704717	2.93E-06
**KRCC1**	-1.591256356	2.97E-06
**ZDHHC15**	-2.442360766	3.07E-06
**TIA1**	-1.927848441	3.09E-06
**KLRC3**	-6.660664937	3.13E-06
**SLC25A25**	-2.564863044	3.18E-06
**GOLGA2**	-2.170339054	3.30E-06
**JAG1**	-4.421832899	3.35E-06
**ZHX2**	-2.734538351	3.39E-06
**NNT**	-1.688072149	3.50E-06
**CLK4**	-1.941223901	3.54E-06
**TICAM1**	-6.409461793	3.65E-06
**MIER3**	-2.020480536	3.67E-06
**NRIP1**	-1.552155897	3.74E-06
**ACAD8**	-1.751444729	3.84E-06

MESP2	6.064005094	6.39E-05
RP11-564D11.3	2.831109385	6.41E-05
DDIT3	2.215673428	6.49E-05
ATM	1.287728998	6.59E-05
SLC50A1	1.565384987	6.64E-05
ACTN1	1.184788077	6.70E-05
CLNS1A	1.126096736	6.84E-05
AP003900.6	6.142594363	7.05E-05
FHL2	3.387355058	7.06E-05
AC073130.1	6.12655016	7.07E-05
SIL1	1.740002194	7.11E-05
QSOX1	1.834396104	7.19E-05
FAM86B3P	1.958737718	7.41E-05
RP11-280G9.1	6.171046787	7.41E-05
TLL2	4.082117802	7.48E-05
CYB5R2	4.606681285	7.55E-05
RPL12	1.429467442	7.62E-05
FBXO43	2.380969891	7.84E-05
RP11-495P10.1(	1.936887807	8.03E-05
FAR2P1	4.936531953	8.05E-05
HSPA9	1.249272179	8.05E-05
PEPD	2.039501264	8.10E-05
KLC3	6.165012543	8.13E-05
HLA-L	2.063913934	8.15E-05
SIRPB1	4.675312778	8.27E-05
RP11-465B22.3	2.976147091	8.27E-05
ERO1B	2.100009409	8.28E-05
MYOSLID	6.251556237	8.30E-05
RP11-122G18.1	4.018316325	8.30E-05
ACD	1.882616365	8.31E-05
OAF	2.390448949	8.33E-05
RP11-148O21.2	6.292163975	8.38E-05
RP11-818F20.5	5.837404855	8.44E-05
APOL1	4.325237844	8.46E-05
ECE2	2.428320432	8.58E-05
DENND3	2.990357638	8.62E-05
AC116609.3	6.271703909	8.65E-05
TRIP10	1.657131428	8.73E-05
SMPDL3B	4.616445423	8.74E-05
DUSP5	3.686129958	8.81E-05
TRIP13	1.600358181	8.90E-05
CES3	3.017303193	8.98E-05
MAP2K3	1.320056939	9.04E-05
KCNN1	3.188418263	9.20E-05
ZC3H15	1.607253748	9.21E-05
CDK7	1.328809493	9.44E-05
NTRK1	3.864659504	9.49E-05
FAM111A	1.434909359	9.61E-05
SLCO1B1	6.200788044	9.65E-05
KRT34	6.175123649	9.66E-05
SNHG3	2.007679738	9.72E-05
RP11-336A10.5	6.036433974	9.75E-05
TEKT4P2	1.652994433	9.76E-05
GUCA1B	2.512679652	9.79E-05
FDPS	1.682649589	9.81E-05
DNAJA2	1.254528871	9.83E-05
SNTA1	1.855026372	9.83E-05
MTHFD2	1.611108297	1.01E-04
MICALCL	5.09568983	1.01E-04
ARRDC1	2.227428125	1.01E-04
RAB27B	4.233431928	1.02E-04
PKD1L2	4.005502551	1.04E-04
RP11-445O3.3	5.996668	1.05E-04
SMCO2	2.552941153	1.05E-04
CMB9-22P13.1	4.696612941	1.06E-04
ALDH9A1	1.167266015	1.08E-04
RP11-789C2.1	6.134136909	1.11E-04
TMC6	2.137676287	1.11E-04

SLC12A8	-5.863900494	3.88E-06
KLHL14	-6.76944669	3.91E-06
ADAMTS15	-6.455448723	3.96E-06
RP11-126K1.6	-3.730455453	3.98E-06
BMF	-4.069333969	4.10E-06
ZSCAN25	-1.732893065	4.23E-06
TBX15	-4.171671145	4.24E-06
PRR18	-6.298117461	4.25E-06
CD207	-5.178703944	4.26E-06
SMURF1	-1.33102264	4.27E-06
KLHL7	-1.719891504	4.28E-06
ZDHHC22	-5.533750026	4.32E-06
CBSL	-3.91480754	4.45E-06
SORCS2	-6.471728621	4.77E-06
CHADL	-6.890250281	4.81E-06
FAM214A	-1.950336965	4.87E-06
BCL7B	-2.769198511	4.94E-06
PEBP1	-1.756583715	5.01E-06
AC078842.3	-6.312706899	5.01E-06
PAQR6	-2.277732777	5.05E-06
KCNN2	-6.800474237	5.19E-06
PLXNB3	-3.407645123	5.28E-06
bP-21264C1.1	-6.335504816	5.33E-06
COL16A1	-4.031738769	5.33E-06
LA16c-306E5.2	-6.609989098	5.38E-06
SDPR	-7.580559368	5.40E-06
MYCN	-7.634149725	5.43E-06
RBM23	-1.47130468	5.57E-06
PARD6G	-2.029138891	5.72E-06
AC064875.2	-8.268709306	5.72E-06
SLC7A3	-6.98685071	5.97E-06
KANK1	-3.055429556	6.04E-06
LEPROTL1	-2.38294778	6.16E-06
PLCB1	-3.154386266	6.19E-06
ESRRG	-7.840914344	6.19E-06
RP11-742D12.2	-6.600456329	6.20E-06
ZNF835	-7.47760205	6.31E-06
ZKSCAN1	-2.017420509	6.33E-06
RND2	-1.916048024	6.35E-06
WSB1	-2.327253738	6.35E-06
RGL1	-1.914148063	6.35E-06
CCDC120	-6.445042338	6.40E-06
BBOX1	-7.257404167	6.45E-06
MBTPS1	-1.246984379	6.45E-06
ATP6V0E2-AS1	-2.617283231	6.45E-06
CHST9	-6.261634078	6.75E-06
TGFB3	-2.174138043	7.01E-06
CDH6	-8.393378183	7.06E-06
CORO2B	-4.079241563	7.06E-06
ADARB2	-6.253261526	7.07E-06
ZFAND5	-1.333078315	7.09E-06
LINCR-0001	-3.583810268	7.15E-06
LINC00909	-1.795020664	7.52E-06
IFITM2	-6.357688366	7.72E-06
AC073343.13	-3.817835458	7.97E-06
LINC01224	-6.942475864	8.32E-06
RASA3	-1.870986963	8.33E-06
ADAMTS3	-2.702139134	8.33E-06
ITM2C	-2.910323702	8.35E-06
REV3L	-1.769411596	8.46E-06
COLEC12	-6.264337315	8.51E-06
SON	-1.19079563	8.55E-06
OPN3	-1.743267409	8.70E-06
ATP6V0E2	-2.493339529	8.70E-06
PLD5	-8.560230933	8.78E-06
CD36	-5.879239698	8.99E-06
KANSL2	-1.278645852	9.00E-06
RGS16	-2.249077285	9.11E-06

C10orf11	3.201052494	1.11E-04
RP11-77H9.2	2.414492258	1.11E-04
ROBO4	5.253815395	1.12E-04
CMTM4	1.509838385	1.13E-04
GOLT1B	1.587493058	1.15E-04
ARHGAP45	3.899697261	1.18E-04
ARHGAP27	2.946301498	1.19E-04
MND1	1.798401823	1.21E-04
TMEM39A	1.114901907	1.23E-04
RP11-126K1.2	2.955735961	1.24E-04
FAM98C	1.451070818	1.25E-04
GTF2H2B	1.695819244	1.28E-04
RP11-61A14.1	5.6362257	1.28E-04
TMEM200B	3.904905657	1.28E-04
FKBP2	1.428743831	1.29E-04
RP11-681B3.4	5.946072551	1.29E-04
AGPAT2	2.30862541	1.30E-04
TMEM40	6.63599404	1.30E-04
LRRC2-AS1	6.032954976	1.31E-04
AIP	2.098549984	1.33E-04
TBC1D32	2.354456866	1.33E-04
CREG1	1.511955171	1.39E-04
PANX2	2.967373077	1.40E-04
RFTN1	4.409310376	1.40E-04
NBL1	3.393181516	1.41E-04
MARS	1.386117466	1.41E-04
AXDND1	3.490919951	1.43E-04
ANXA11	1.540619085	1.44E-04
CTC-281F24.3	3.96588506	1.46E-04
LRRC2	4.249950974	1.49E-04
STYK1	5.307218857	1.49E-04
THG1L	1.125594406	1.52E-04
TPK1	1.484219136	1.53E-04
DEPTOR	3.926036101	1.53E-04
RABGAP1L	1.686217785	1.55E-04
TRIM25	1.290707489	1.56E-04
UQCRB	1.431678816	1.59E-04
CTC-241N9.1	2.085569121	1.62E-04
RP11-745L13.2	5.965543072	1.63E-04
ADRA1B	6.001859259	1.63E-04
EMBP1	4.881064498	1.65E-04
EVA1A	4.734748264	1.65E-04
SHANK2	5.327824646	1.65E-04
EBAG9	1.489276633	1.65E-04
LINC01238	1.855748333	1.66E-04
NRTN	3.205906131	1.66E-04
NMNAT3	2.730653499	1.66E-04
UPK1A	4.837453706	1.67E-04
LINC01293	5.944346668	1.67E-04
STPG2	3.302883306	1.72E-04
DNASE1L1	2.513631086	1.72E-04
DDIT4	2.039723673	1.74E-04
RP11-43A14.1	4.387826573	1.75E-04
DNHD1	1.743757735	1.75E-04
GFOD1	2.586068154	1.78E-04
PRR5	3.722403025	1.79E-04
MYOF	4.747732201	1.79E-04
CAPN12	4.001323879	1.80E-04
PLCB4	2.002095885	1.80E-04
PIK3CD-AS2	2.914291017	1.81E-04
OPTN	1.759683322	1.84E-04
GRAMD1C	2.134171971	1.85E-04
BCAS4	1.852960821	1.88E-04
RP11-445F12.1	3.584080174	1.88E-04
NAP1L1	1.336960592	1.89E-04
TAF1D	1.84307435	1.90E-04
CTD-2128A3.2	5.916777774	1.90E-04
KIAA1522	1.414859377	1.93E-04

CCR1	-7.206672765	9.14E-06
RP5-981L23.7	-6.696184964	9.14E-06
RP11-834C11.4	-6.194158157	9.19E-06
MAML2	-1.79195954	9.28E-06
GLCCI1	-2.205050697	9.29E-06
STXBP5L	-7.422078541	9.37E-06
RALGAPA2	-1.489220243	9.40E-06
CAMK2B	-5.991190257	9.40E-06
NETO1	-7.185569657	9.45E-06
ALK	-6.706011432	9.77E-06
AFDN	-1.359167603	9.79E-06
RGCC	-6.812449545	9.92E-06
ARHGAP6	-7.322137496	1.00E-05
STYXL1	-2.704520263	1.01E-05
MTPN	-1.360651814	1.02E-05
GALNT5	-6.678601559	1.03E-05
ANO7	-6.489876957	1.03E-05
SH3KBP1	-1.573217871	1.03E-05
ABCB6	-3.307001766	1.03E-05
ASB16-AS1	-1.933744054	1.04E-05
WTAP	-1.762099491	1.04E-05
LUM	-6.315667694	1.07E-05
POGLUT1	-1.533434776	1.10E-05
KDM5B	-2.455462867	1.10E-05
USP32P1	-7.126391356	1.11E-05
SYNE2	-2.479573527	1.14E-05
HDLBP	-1.473395602	1.15E-05
AP1S2	-2.574886233	1.17E-05
ITIH6	-6.56496578	1.17E-05
IGDCC4	-5.056808897	1.20E-05
SCARB2	-1.994238576	1.21E-05
ZNF578	-5.785074336	1.21E-05
ACVR2A	-1.850858818	1.23E-05
RAB42	-6.520326766	1.23E-05
TXLNB	-6.662405579	1.23E-05
RCC1L	-2.173430859	1.24E-05
ATOH8	-6.274964139	1.26E-05
LSM5	-1.225545572	1.26E-05
SRGAP2	-2.101690719	1.28E-05
CENPU	-1.734265274	1.29E-05
DRAXIN	-8.939502572	1.30E-05
PSMA2	-1.741675785	1.30E-05
SGK2	-6.779409355	1.32E-05
PMS2P3	-2.299633367	1.32E-05
BPHL	-1.510703854	1.33E-05
CREB5	-5.185225317	1.33E-05
CITED1	-4.037283125	1.35E-05
CELSR2	-1.989719953	1.36E-05
SNHG18	-4.647868931	1.37E-05
NKAIN4	-5.761509818	1.40E-05
LDB1	-1.190045783	1.40E-05
TMEM35A	-6.115730612	1.41E-05
RP11-351J23.2	-7.008216051	1.41E-05
CCNT2-AS1	-2.887192815	1.44E-05
PROX1-AS1	-6.483430831	1.45E-05
PLIN3	-3.321967585	1.49E-05
COL5A3	-5.341963106	1.51E-05
VCAM1	-6.092706226	1.52E-05
WBSCR22	-1.867522876	1.53E-05
RENBP	-5.059132296	1.53E-05
ARHGAP19-SLI'	-5.336643306	1.54E-05
WIPF1	-1.877854311	1.55E-05
WDR60	-1.929350583	1.55E-05
MZF1-AS1	-4.109415081	1.56E-05
U52111.14	-5.209094491	1.56E-05
CYP4F24P	-7.661051506	1.56E-05
VAV3	-8.529394563	1.57E-05
LINC01759	-6.155727325	1.58E-05

**DLX2**	2.865885537	1.93E-04
**MAP4K2**	1.561770013	1.95E-04
**ACAT1**	1.901181734	1.95E-04
**TRMT1L**	1.3881325	1.96E-04
**SSSCA1**	2.207044177	1.97E-04
**ANKRD18DP**	3.798272606	1.97E-04
**TIGD7**	1.261368628	2.00E-04
**RP11-115J23.1**	3.59549855	2.00E-04
**AC058791.1**	2.103776712	2.02E-04
**FAM19A3**	3.17626306	2.02E-04
**TDRKH**	1.509200106	2.04E-04
**SRPX**	1.590762144	2.08E-04
**LINC00397**	5.833030396	2.08E-04
**RP3-414A15.2**	5.833030396	2.08E-04
**MCF2L2**	2.689258539	0.0002088209552
**RP11-553A21.3**	5.668675854	0.0002120600755
**CISH**	2.757082702	0.0002139745216
**ANKRD13D**	1.658325304	0.0002139745216
**P2RX5**	3.68335431	0.0002161277632
**TBC1D2**	2.378117517	0.000217989657
**COQ8A**	1.766127002	0.0002205338588
**INTS3**	1.059356499	0.0002221196089
**RPSAP52**	4.192110637	0.0002231950541
**HRAT17**	6.195418077	0.0002233493012
**CD163L1**	4.532283777	0.0002241113478
**GRIN3B**	5.205836495	0.0002242319126
**KIAA1551**	1.587805706	0.0002265759245
**TRANK1**	3.34275518	0.0002270264672
**RAD21-AS1**	3.167226406	0.0002277363176
**RIDA**	1.471036813	0.0002279195181
**ST8SIA6**	4.701773242	0.0002284750564
**AC007879.2**	4.331446467	0.0002325843762
**AC115522.3**	4.767627014	0.0002331553758
**THAP1**	1.160970462	0.0002344459672
**CCNB3**	1.889676045	0.0002359353223
**RAD51**	1.432194582	0.0002378392159
**C8orf37**	1.402812314	0.0002405864509
**CDS1**	4.593883748	0.0002419520018
**SP6**	3.239145168	0.0002441319584
**DHX38**	1.461842975	0.0002464800835
**FOXP4-AS1**	2.326531954	0.0002478962423
**HSP90AB1**	1.643094068	0.0002505446139
**SGMS2**	4.069633842	0.0002521425125
**RPTOR**	1.680011242	0.0002543925844
**NME3**	3.532225519	0.0002575574722
**FHOD1**	1.938712913	0.0002614833863
**TRPC4**	6.383015553	0.0002631858383
**KNDC1**	4.666180877	0.0002632122149
**ACBD3**	1.232549163	0.0002648191853
**ONECUT3**	6.009196337	0.0002657434757
**LHX1**	3.861006975	0.0002673204673
**KB-1980E6.3**	3.412296271	0.0002681835125
**CENPL**	1.275730176	0.0002735563401
**MTX1**	1.439471995	0.0002755913006
**AP1S3**	3.400879318	0.0002799911503
**PRKCG**	4.282526901	0.0002803721307
**IMPA1**	1.556668686	0.0002806190871
**SHISA2**	6.44072761	0.0002837849483
**FAM210A**	2.304700909	0.0002839375426
**SNX16**	1.61366185	0.0002859528621
**MED8**	1.564165631	0.0002870742793
**CAST**	1.654880843	0.0002882611704
**CPNE3**	1.493605788	0.0002888576008
**STX11**	4.864614093	0.0002891687071
**C1orf204**	3.41048722	0.000289401083
**SLC35E4**	2.065000348	0.0002937047911
**C6orf99**	5.335843395	0.000296850181
**NR4A3**	2.45754144	0.0003046372206

**LMTK2**	-1.254268351	1.58E-05
**PLA2G3**	-6.221722267	1.61E-05
**CABLES1**	-3.085311481	1.64E-05
**TRMT10B**	-2.12178456	1.66E-05
**APC2**	-3.469252438	1.69E-05
**CYP27A1**	-5.862314049	1.73E-05
**PPM1D**	-1.381414749	1.74E-05
**VTI1B**	-1.383130372	1.77E-05
**KLF15**	-2.49576687	1.77E-05
**RPS6KL1**	-7.440406124	1.78E-05
**RP11-268J15.5**	-3.199082895	1.80E-05
**SLAIN1**	-2.38790468	1.81E-05
**PSMA3-AS1**	-1.50008569	1.84E-05
**CDK5RAP2**	-1.249065616	1.84E-05
**SEL1L**	-1.643248111	1.84E-05
**GLIDR**	-2.364688409	1.86E-05
**DENND2A**	-3.682338511	1.86E-05
**GABRA4**	-7.578027863	1.88E-05
**C8orf31**	-6.714240074	1.88E-05
**TLCD2**	-4.302835096	1.94E-05
**RALA**	-1.610496687	1.95E-05
**SFXN5**	-2.49676208	1.95E-05
**ELMO2**	-1.264742299	1.97E-05
**PIK3C3**	-1.622590212	1.98E-05
**KDM2B**	-1.360665572	1.98E-05
**CLCN3P1**	-6.002472608	2.00E-05
**TCTA**	-2.179191107	2.00E-05
**PANK2**	-1.291076364	2.02E-05
**GABBR2**	-6.866019027	2.02E-05
**RP11-174J11.1**	-6.328840649	2.05E-05
**TSPAN3**	-2.151383699	2.06E-05
**ZSWIM7**	-1.5524246	2.08E-05
**TSTD2**	-1.70327186	2.14E-05
**NPNT**	-8.467447428	2.19E-05
**KIAA1841**	-1.605669308	2.24E-05
**SAP130**	-1.314017605	2.33E-05
**NDP**	-7.141590608	2.40E-05
**RP13-942N8.1**	-3.265656338	2.40E-05
**ZNF286A**	-1.547561835	2.41E-05
**PBX1**	-2.793970555	2.52E-05
**SH3PXD2B**	-1.896790533	2.52E-05
**VWCE**	-2.896423675	2.54E-05
**KCTD14**	-5.675366872	2.54E-05
**CH17-408M7.1**	-6.258366558	2.57E-05
**LOXL4**	-6.355158721	2.61E-05
**SLC15A2**	-3.58681522	2.61E-05
**UGGT2**	-1.504955429	2.64E-05
**APOLD1**	-4.099375945	2.66E-05
**MRPS33**	-1.373964755	2.72E-05
**MSRA**	-2.529747678	2.81E-05
**NAA60**	-1.877030786	2.83E-05
**GDPD2**	-7.437552248	2.83E-05
**SRPK3**	-6.234286216	2.83E-05
**TP53RK**	-1.298426852	2.84E-05
**XXYLT1**	-2.230129922	2.87E-05
**SYNPO2L**	-5.332730031	2.88E-05
**LAMP1**	-1.844623723	2.90E-05
**TMEM178A**	-4.806759992	2.90E-05
**TNFRSF1B**	-2.657970399	2.94E-05
**SUSD1**	-3.382783751	2.94E-05
**CNDP2**	-1.794495006	2.95E-05
**COMMD2**	-1.22822191	2.97E-05
**DBNL**	-1.952715567	3.04E-05
**KLF12**	-1.65512703	3.05E-05
**TRIB2**	-4.958139284	3.05E-05
**PTPRN2**	-6.161037669	3.06E-05
**SYNJ1**	-1.590950773	3.08E-05
**APPBP2**	-1.383694247	3.08E-05

IL11	3.632908975	0.0003060368088	TRAF3IP2-AS1	-1.702715019	3.10E-05
FAM157A	2.268478236	0.0003174286696	CREBBP	-1.347635202	3.11E-05
CDK2AP2	2.25631826	0.0003179336575	RP11-120J1.1	-3.974457425	3.13E-05
KREMEN2	3.44683736	0.0003180906123	RBMS3	-2.084815207	3.15E-05
TWIST1	2.072764073	0.00031877083	AC004980.7	-1.951479398	3.15E-05
NUDCD1	1.445088151	0.0003197934527	PELI1	-2.894373961	3.16E-05
EMC2	1.441392204	0.0003226318849	C11orf87	-7.467912664	3.24E-05
LPCAT4	1.318391558	0.0003256612307	LPAR4	-4.813612636	3.33E-05
RP4-660H19.1	5.734910136	0.0003298900161	APAF1	-1.567964156	3.34E-05
RP11-93B14.4	5.670451112	0.0003303264004	BTG2	-2.695127505	3.38E-05
BMI1	1.097282306	0.0003325018199	C2orf80	-6.820461667	3.39E-05
SLC30A3	2.985670228	0.0003348502855	NHSL1	-1.657548669	3.47E-05
METTL18	1.478235135	0.0003348583015	KCNC1	-5.274886501	3.49E-05
TMCO4	2.188888771	0.0003360913493	FAM66C	-1.945290987	3.52E-05
ZNF32-AS2	3.544314316	0.0003360913493	TBC1D7	-1.645422456	3.55E-05
SH3BGRL2	3.173192198	0.0003364128856	RHOU	-3.186389645	3.57E-05
AC017101.10	5.833569888	0.0003380045622	APOM	-2.442445538	3.57E-05
POLR3H	1.702608562	0.0003383808089	L1CAM	-2.681938808	3.61E-05
FAM83B	5.92848296	0.0003413396104	CCDC13	-3.711319491	3.72E-05
CYP11A1	5.812518334	0.0003422847813	RP5-894A10.2	-2.171188768	3.73E-05
CROCCP2	1.589934001	0.0003473772004	LMBR1	-1.907251373	3.80E-05
MRPL41	2.199824598	0.0003509099361	ZNF793-AS1	-6.754784127	3.80E-05
AC006369.2	5.86520115	0.000353289208	RAB7B	-3.086765115	3.82E-05
RP11-289F5.1	4.967720654	0.0003563823617	ZNF786	-1.723777774	3.84E-05
CTC-378H22.2	5.863256032	0.0003609088144	SLC7A8	-4.41206003	3.86E-05
ZFP36	2.259034882	0.0003661364537	VPS45	-1.411453451	3.86E-05
FAM72C	2.873088917	0.0003737272994	SRSF6	-1.135706591	3.91E-05
SNX9	1.165885814	0.0003748318134	KAT2B	-2.244531308	3.96E-05
TSGA10IP	5.724587022	0.0003767621378	MAN1C1	-3.530480386	4.04E-05
TAF7	1.256122997	0.0003800456543	RP11-1263C18.[illegible]	-7.313387613	4.16E-05
DCAF13	1.401547357	0.0003863106457	SULT1A1	-5.492318495	4.17E-05
TNFRSF11A	3.925731058	0.0003954832737	BST2	-6.441679716	4.21E-05
APOBEC3D	5.28173632	0.0003990469086	CTD-2049O4.1	-7.321369781	4.22E-05
SLC13A3	1.318403408	0.0004008600538	PLOD3	-1.681723964	4.25E-05
GHITM	1.264366628	0.0004019056312	MPPED2	-7.737450791	4.31E-05
LYPLA1	1.412389414	0.0004055566677	CCNT2	-1.499508598	4.38E-05
SLC17A7	3.188159766	0.0004118426117	AC004540.4	-6.282644251	4.39E-05
RFESD	1.780747034	0.0004129507907	SHISA7	-4.836924153	4.41E-05
EIF2S2	1.497174808	0.0004143754969	NAA40	-1.315186546	4.50E-05
MSLN	5.79488103	0.0004158964924	ENKUR	-4.544953997	4.50E-05
GRPEL2	1.136061341	0.0004190176426	SFPQ	-1.301935116	4.51E-05
SIRT7	1.280458303	0.000419290467	NOTCH4	-3.352951333	4.57E-05
RP11-195F19.9	4.469005451	0.0004238631088	SPPL3	-1.578143627	4.71E-05
ABCA5	3.479459943	0.0004258580416	GPR107	-1.730129255	4.79E-05
AHNAK	3.86555057	0.0004269202114	YBX2	-4.076146014	4.80E-05
CACNA2D2	3.657926708	0.0004271538083	DRD2	-7.214940803	4.98E-05
DNPH1	1.904916479	0.0004316625232	PXMP2	-2.741124337	4.98E-05
FKBP5	1.443127729	0.0004372348403	PDZD2	-5.08230256	5.00E-05
CDIPT-AS1	3.803998997	0.0004372348403	EZH2	-1.393782233	5.05E-05
CENPA	1.254057718	0.0004419414531	PCDHA10	-4.574259996	5.05E-05
AC009502.4	5.730004875	0.0004430834675	AC009120.6	-1.504138068	5.08E-05
RP11-783K16.1[illegible]	2.343228486	0.000449774211	PRRT4	-4.437228743	5.08E-05
TMEM123	1.481262415	0.0004498855903	NSUN5P2	-2.058475023	5.14E-05
OLMALINC	1.752506758	0.0004498855903	ARL17A	-2.634782824	5.21E-05
TUBE1	1.582715801	0.0004520677542	IFRD1	-1.452438355	5.21E-05
ADAM11	2.858991538	0.0004525825731	CNP	-2.810932937	5.25E-05
RGS4	3.91157089	0.0004567081725	CCDC144CP	-2.84803853	5.28E-05
COG1	1.11675938	0.0004570488584	CACFD1	-2.355884735	5.32E-05
IRF6	6.176392219	0.0004583536955	RARB	-6.197173452	5.54E-05
COPS5	1.325793217	0.0004588084407	ATP6V1G2	-2.12424706	5.58E-05
RP11-146F11.5	3.46808574	0.0004612645846	TMEM181	-1.224017819	5.80E-05
KIAA1429	1.24880511	0.000462955549	SLC19A3	-1.834850488	5.93E-05
SPATS2	1.474620984	0.0004632507756	ADAT1	-1.502652197	6.01E-05
GLTSCR2	1.999321341	0.0004641758216	SLC26A4-AS1	-5.453726901	6.07E-05
IMPA2	1.554706474	0.0004666207193	HEY2	-4.414289421	6.08E-05
BOLA3	1.518398992	0.0004728041371	CHURC1	-1.999784717	6.11E-05
KIF20B	1.703329965	0.0004755326231	TLX1	-6.950689331	6.15E-05
BEND6	2.07869001	0.0004886709483	METTL2B	-1.168119304	6.18E-05

PNPLA6	1.908810733	0.0004961947864
CMTM8	1.566319755	0.0004977234145
KRTAP4-8	6.443207316	0.0004977485223
ULK4	1.312172531	0.0004999601834
ABCC10	1.102596042	0.0005019276307
DUSP11	1.167737455	0.000506676372
LINC01291	5.777375091	0.0005150146816
RPS6KA6	4.176362664	0.0005179845633
NARFL	1.394658197	0.0005220812319
TJP3	4.995679468	0.0005221144978
TRIQK	1.513918681	0.0005225205458
SYT7	3.220055066	0.0005225750323
SHANK1	4.034790192	0.0005341234804
HMGCS1	1.801653223	0.0005392205274
TKFC	1.504730346	0.0005411830668
FAM3D	5.651387957	0.0005411830668
A2M-AS1	4.168786915	0.0005431319448
FAM72A	1.52670555	0.0005460427912
RP11-1149O23.4	5.650212647	0.0005514826019
BTBD1	1.164110292	0.0005561650065
ALDH1L2	1.822030814	0.0005673223049
GRAMD1B	4.141690663	0.0005700008455
EEF1A2	2.505721147	0.0005703344178
BAIAP3	2.92378439	0.0005741814719
CCDC110	2.663756586	0.0005794115432
INTS4	1.03261393	0.0005889573993
TTC9C	1.040712325	0.0005901527529
APOBEC3B	1.462245881	0.0005912597489
RP11-1038A11.3	5.598300563	0.0005962509155
HEBP1	1.097962078	0.0006017518789
CRABP2	3.371708802	0.0006024485267
ZNF550	1.569937387	0.0006024485267
ODF3B	5.538985642	0.0006087419115
LINC01611	5.665276992	0.0006123094382
CYYR1-AS1	5.796277869	0.0006165715929
C4orf22	5.639824024	0.0006184386534
SEMA6B	2.99833995	0.0006242887352
TIPARP-AS1	1.715318172	0.0006259775515
TOMM34	1.484771827	0.0006277886936
MC1R	1.600320028	0.0006305477162
LINC01011	1.594381048	0.0006314346565
RGS9	1.629533345	0.0006322646672
POLR1C	1.532565206	0.0006361256789
CFAP58-AS1	5.230445092	0.0006387733307
XXyac-YX65C7_	3.152361148	0.0006402732459
AP001596.6	3.794530851	0.000642315236
MRPL15	1.303477517	0.0006428825236
NFKBID	1.919576001	0.0006428825236
HLA-DRB1	5.738595578	0.0006457899872
TBC1D3P1	5.291093929	0.000647412725
RP1-46F2.3	5.65892083	0.0006545522028
CTD-2020K17.1	3.574560661	0.0006555385044
C19orf24	2.110828977	0.0006593560176
CTD-2035E11.3	2.266749235	0.0006709769816
TFB1M	1.790036923	0.0006723643113
AP1AR	1.737939287	0.0006747747283
HDDC3	1.759334291	0.0006815891691
DHCR7	2.046923772	0.0006851095909
CHCHD7	1.778174469	0.0006962552906
CCNG1	1.5095986	0.0007141528471
CTD-3162L10.1	3.682695686	0.0007170087374
ALDH1L1	3.991164527	0.0007254732201
ADORA2B	2.014181587	0.0007315146553
VSIR	3.796135419	0.0007328115695
SCN8A	2.419899739	0.0007407571256
SP5	4.554661859	0.0007536343928
EPHX4	2.926726861	0.0007537808265
FAM182B	2.503938656	0.0007599373597

TFAP2A-AS1	-2.578756204	6.18E-05
NOTCH1	-2.38416656	6.32E-05
N6AMT1	-1.464241785	6.41E-05
PTPRJ	-2.995008524	6.50E-05
S1PR2	-2.131228152	6.59E-05
RP11-582J16.4	-4.940195472	6.60E-05
PRKAB1	-1.386382525	6.65E-05
FAAH	-3.063708142	6.66E-05
ZP3	-4.245213998	6.70E-05
ERV3-1	-2.923616752	6.71E-05
MTSS1	-2.902033457	6.74E-05
NHLRC3	-1.972023136	6.78E-05
RP11-625L16.1	-6.239954724	6.85E-05
CCDC188	-6.771855543	6.86E-05
CDS2	-1.517824367	6.97E-05
EGF	-5.910481677	7.11E-05
LRRC37A	-4.076792221	7.24E-05
TET2	-1.726384486	7.26E-05
PIAS2	-1.903096706	7.28E-05
RCOR3	-1.339422034	7.59E-05
TRPC3	-1.952000887	7.69E-05
TSHZ1	-1.843380294	7.79E-05
TPGS2	-1.401330585	7.91E-05
ZMYM2	-1.450774501	7.91E-05
DIRC3	-6.029398735	8.08E-05
PIK3AP1	-6.231266927	8.13E-05
ZNF681	-4.664779331	8.20E-05
BCL9	-1.522688419	8.23E-05
C1R	-4.054879308	8.35E-05
TEKT2	-6.281671406	8.36E-05
UBN2	-1.42492328	8.42E-05
PHKG1	-3.559632841	8.42E-05
POMT2	-1.693584754	8.68E-05
RP11-1114A5.4	-2.634229881	8.80E-05
PCDH18	-2.946862256	8.89E-05
SLC24A2	-4.93774687	8.93E-05
KLF3-AS1	-4.575331248	9.04E-05
LINC00326	-6.314611164	9.07E-05
LGI3	-5.896132289	9.07E-05
ARHGAP15	-6.128386239	9.19E-05
CDK14	-2.799083317	9.22E-05
TMEM221	-7.015319196	9.22E-05
LINC00664	-7.036373964	9.29E-05
LRIG1	-2.651161315	9.30E-05
CYP24A1	-6.52652621	9.39E-05
MED4	-1.051860378	9.42E-05
CCNE2	-2.351715611	9.60E-05
CPT1C	-1.802348229	9.70E-05
TBC1D13	-2.139992088	9.72E-05
ACADL	-6.34097609	9.79E-05
CABYR	-5.855067283	9.87E-05
ZNF397	-1.473815447	9.91E-05
MDH2	-1.625473508	0.0001007438938
CIPC	-1.381691935	0.0001016672288
ZNF195	-1.767649668	0.0001017868071
RP5-942I16.1	-6.014181696	0.0001022728821
LSMEM1	-1.933097674	0.0001035192066
RP11-1191J2.2	-6.395026286	0.000105034038
RP2	-1.494590892	0.0001097058338
PLGLB1	-6.174394792	0.0001098830558
FGF11	-4.064848348	0.000110963311
PLCD4	-4.835138045	0.0001127538413
MEF2C	-2.528773143	0.000113082075
BCL6	-3.223438576	0.0001146074646
AC108025.2	-6.017357396	0.0001211827081
ZNF326	-1.548570916	0.0001216444844
COL24A1	-5.802589958	0.0001217649257
GUCY1A2	-3.926729462	0.000126960291

**SLC10A3**	1.800831718	0.0007610381976
**MRPL39**	1.038188436	0.000767811906
**LINC01385**	5.213778355	0.0007681225893
**KLC4**	1.596923805	0.00077108709
**LINC00887**	4.768162992	0.0007718938547
**CTC-296K1.4**	3.998608616	0.0007742883328
**VDAC3**	1.190327297	0.0007761568429
**WT1**	3.054803397	0.0007794766914
**ATF1**	1.260589816	0.0007796596635
**SERPINE1**	4.000771324	0.0007828209038
**PTBP3**	1.157095553	0.0007908280334
**SH3TC1**	3.332567684	0.0007908280334
**MRPL14**	1.632865686	0.0007918457095
**AFG3L2**	1.335459631	0.0007931669319
**QPCTL**	1.613619578	0.0007952536066
**CENPQ**	1.289494425	0.0007956586358
**IGFBP6**	3.173399727	0.000800734132
**WNT9A**	2.56043785	0.0008139272622
**RP11-255I10.2**	5.566707451	0.0008192694951
**MMP1**	5.633896566	0.0008244235812
**ERC2**	3.784584233	0.0008292004769
**RP11-288C17.1**	3.627892416	0.0008293602346
**ZFP64**	1.346982885	0.0008300294422
**RP11-756A22.7**	2.907268163	0.0008300294422
**ERO1A**	1.418742592	0.0008327648629
**KCNQ1**	4.077196153	0.0008396977699
**SERTAD4-AS1**	4.408977222	0.0008421646925
**GINM1**	1.248458477	0.0008472979903
**KIF12**	4.465577191	0.0008712928593
**ICAM3**	3.65704382	0.0008724535267
**CCDC114**	2.64997732	0.0008784063149
**CHORDC1**	1.807189398	0.0008981652702
**NVL**	1.127588771	0.0009049303891
**ASH1L-AS1**	1.385697423	0.0009050364661
**USP32P2**	3.895944784	0.0009065648927
**RPS27**	1.340957822	0.0009072209424
**AMIGO2**	3.23171473	0.0009230832358
**ELL**	1.648829891	0.0009297164672
**PMFBP1**	3.089186111	0.0009411070239
**LINC01763**	5.073877759	0.0009411342309
**FAM86C2P**	1.718322423	0.0009413369038
**SPINT2**	5.145401442	0.0009486656075
**DMBT1**	4.86532157	0.0009512461699
**CA3**	2.793056217	0.0009560913198
**HOMER2**	2.272314876	0.0009628237861
**HMG20B**	1.561510133	0.0009635275437
**ZFPL1**	1.636337777	0.0009682942101
**NFS1**	1.197655844	0.000968524647
**VPS51**	1.870147358	0.000976192413
**RP11-1094H24.4**	3.435986713	0.000978736911
**PCDHGA6**	1.536393755	0.0009884439744
**RP11-399K21.14**	3.580469233	0.0009919611781
**LYN**	2.426066615	0.0009969719692
**OCLN**	2.151543405	0.001010326866
**SLC7A7**	3.608463611	0.001013418859
**EXPH5**	3.583238705	0.001020974983
**CAMSAP3**	3.450092755	0.001025413497
**AMOT**	1.330270957	0.001026221568
**TSPAN19**	5.293806069	0.001028420529
**HSPA2**	2.78288696	0.001032441753
**GALK1**	2.036385407	0.001052722926
**RP11-337C18.8**	1.595992218	0.001058565184
**RP5-1071N3.1**	3.232444987	0.001062444777
**RP11-1C8.4**	5.648537776	0.001063952771
**PRICKLE3**	1.544015044	0.001073125705
**NEU3**	1.881210749	0.001073452665
**HERC6**	1.668345692	0.001076415138
**COL18A1**	2.550279121	0.001091350925

**BRINP2**	-7.485391959	0.0001280409123
**LINC01571**	-6.934581678	0.0001285817886
**TNR**	-6.04713422	0.0001303132065
**RUFY3**	-2.021686763	0.0001331939762
**CTD-2380F24.1**	-5.961929438	0.000133566291
**SEPT7P2**	-1.5492593	0.0001381117819
**DBI**	-1.932590626	0.0001413593038
**STK36**	-1.786876929	0.0001429509668
**CPNE5**	-4.985076934	0.0001435921825
**DCUN1D2**	-1.610155966	0.0001436026314
**P2RX6**	-3.114256558	0.0001437275736
**CROT**	-1.498122017	0.0001447607742
**HAS2-AS1**	-4.085607506	0.000145027447
**LINC00844**	-6.506214183	0.0001455942328
**USP6NL**	-2.094789533	0.0001476601159
**SLC6A1**	-6.291388637	0.0001492338217
**IGFBPL1**	-6.916218781	0.0001494838741
**ZNF665**	-4.534523102	0.0001497526294
**CPEB2**	-2.183944531	0.0001499956786
**CASK**	-1.218153687	0.0001505001211
**EFHB**	-3.885577778	0.0001505001211
**TET3**	-1.227875609	0.0001534002706
**AGAP9**	-2.033008178	0.0001537526836
**ATP5S**	-1.128715878	0.000153854396
**RP11-75C10.7**	-6.823811427	0.000155208407
**MN1**	-4.443220461	0.0001558878363
**LINC00908**	-6.481107341	0.0001560257212
**KCNH8**	-6.433689519	0.0001566449822
**VPS26B**	-1.518328242	0.0001570580839
**ADGRL3**	-2.625659858	0.0001581451265
**CKB**	-2.672190752	0.000162358273
**NKIRAS2**	-1.596413971	0.0001624482971
**TP53INP1**	-1.653609753	0.0001654590983
**DCTN5**	-1.055875566	0.0001655936404
**SETD4**	-1.309390555	0.0001660571308
**GATSL2**	-2.222107936	0.0001667032109
**DNAJC28**	-1.930911085	0.0001672499089
**SH3BGR**	-1.595741591	0.0001689731608
**PTBP2**	-1.71189802	0.0001693405314
**ANTXR1**	-2.029216227	0.00016971677
**LUC7L**	-1.204263891	0.0001700296867
**THRA**	-1.577056733	0.0001700296867
**FBLN7**	-2.776087263	0.0001700296867
**ARC**	-4.32165699	0.0001700296867
**SH3D21**	-2.287195531	0.0001700296867
**RARRES1**	-6.300998123	0.0001720297713
**HSDL1**	-1.271066674	0.0001741519536
**SCN2A**	-5.720489701	0.0001741935815
**RP11-13A1.1**	-5.656338899	0.0001779992895
**TBX21**	-5.887691991	0.0001783490544
**LMO1**	-5.950475905	0.0001783490544
**HOXD-AS2**	-5.941264646	0.0001805001733
**ZC3H14**	-1.091280277	0.0001805689676
**EML1**	-2.320998196	0.0001829236705
**SGCB**	-2.150964053	0.0001837833549
**RP11-498P14.3**	-2.986561638	0.0001840136747
**CPT2**	-1.274499213	0.0001844144916
**SLC43A2**	-2.551602162	0.0001846792081
**KIAA0825**	-2.649464357	0.000184905333
**LINC00977**	-6.219898602	0.0001857006558
**ABHD3**	-1.355213982	0.0001874053284
**C9orf78**	-1.167024087	0.0001896006876
**TTC3**	-1.411619257	0.000190155777
**CLEC2D**	-1.568440398	0.0001917764615
**STARD7**	-1.282435027	0.0001925264545
**SCG3**	-6.18740641	0.0001934401174
**LRRC34**	-6.126019086	0.0001934924996
**AGMAT**	-6.047277268	0.0001945278792

YIPF2	1.920728219	0.00110347967
TMEM107	1.387412532	0.00110347967
GOLGA2P5	2.611589384	0.00110347967
AARS2	1.236828906	0.001104496593
EMSY	1.149535574	0.001106280874
ULBP2	1.906203493	0.001114037869
AP5B1	1.572753811	0.001118883462
CNGA1	4.085622422	0.001121380329
RP11-585P4.5	2.303163707	0.001122770321
RP11-379B18.5	3.018498083	0.001126284887
SRD5A3	2.072714019	0.001129209441
MYBL1	1.582278739	0.00113156829
RP11-192H23.4	1.642989186	0.001131674007
LRRC3-AS1	5.440915985	0.001134270019
RP11-320G24.1	5.44144726	0.001135271104
PRX	2.644822701	0.00113863024
ZNF600	3.226472752	0.001142282318
COL4A3	3.498714231	0.001150444182
LINC01096	2.569687218	0.001152849704
FAM155A	4.173376415	0.001157937365
MTX1P1	1.849713279	0.001179256358
MVD	1.930819022	0.001183642154
RP11-666A8.8	2.71470148	0.001185413762
PAGR1	2.054124732	0.00119263882
WDR36	1.412308003	0.001200896585
RP11-377G16.2	5.05269672	0.001208141242
LBHD1	2.095812149	0.001214530668
ZNF699	1.418449388	0.001218901552
FDX1	1.238674322	0.001223941869
CUL7	3.804967793	0.001226173149
LHX4	3.122548295	0.001238668146
RP11-397A16.1	5.144165161	0.001252642336
CCDC144B	2.487217816	0.001260722306
MTUS2-AS1	4.327983052	0.00126518416
RP11-6D1.3	5.100909506	0.001274578173
RNLS	3.679139786	0.001294940526
ALPK1	2.727274259	0.001304598993
GCLC	1.755625482	0.001306281919
PCBP3-OT1	4.988139472	0.001306281919
ICA1	1.844884327	0.001320099891
AC007319.1	3.659058233	0.001320099891
VIM-AS1	1.900326091	0.001325357573
SMC1B	3.540512488	0.001345851591
RAB11FIP5	1.509011443	0.001345851591
HLA-E	1.805837696	0.001356580259
RIN2	1.964168698	0.00137101705
CTSL	2.568745398	0.001372251799
AMPD2	2.127534674	0.001378832489
CCNY	1.011801848	0.001382140891
LINC01137	3.235447082	0.001389626545
CASC10	1.933984767	0.001406626481
EIF2A	1.213278665	0.001411032512
KLHL17	1.912875085	0.001411819751
TMEM255B	3.626085184	0.001426567056
CDIPT	1.777987381	0.001438414948
AC023590.1	5.440417548	0.001449039108
HAX1	1.389235591	0.001449173312
CEP70	1.735715871	0.001458130752
H2AFY2	4.677174439	0.001461532812
NDUFA2	1.439361836	0.001475587716
FRG1BP	2.113799438	0.001486390724
DTX4	2.332344206	0.001490834852
WHAMMP3	2.426614058	0.001517472151
PRSS12	3.633101179	0.001525613517
ING2	1.244314259	0.001535089415
CKS1B	1.324403927	0.001536760082
CCDC124	1.570229529	0.001536836703
IVNS1ABP	1.034562602	0.001537452728

SMN2	-4.577968467	0.0001945506733
FAM133B	-1.323088203	0.0001946648009
SLC25A25-AS1	-1.773493332	0.0001957125507
NAV2	-2.839371546	0.0001964197515
ZNF271P	-1.387987967	0.0002018118616
UBE2H	-1.906977693	0.0002022208292
TP53I11	-2.521432255	0.0002028835075
PTCHD1	-6.294287283	0.0002039367622
RCOR1	-1.366311799	0.0002045846559
ABCD4	-1.49262165	0.0002056537894
ZNF285	-2.808015336	0.0002067254787
ACTB	-1.628190211	0.0002077109847
MEGF8	-2.3015151	0.0002139745216
ZFP90	-1.284863431	0.0002158593367
RP11-506M12.1	-2.336284409	0.0002171325614
RP11-231C18.1	-6.020036331	0.0002193220442
LRRC8D	-2.426915263	0.0002198754306
SPRY2	-3.438008128	0.0002237498053
SMARCD3	-2.294120396	0.0002241421563
HIPK2	-2.503557211	0.0002263414467
AC073283.4	-5.977321528	0.0002270424433
EPB41L3	-5.72283328	0.0002273300317
ZNF491	-2.385051145	0.0002279195181
ZMYM3	-1.154269972	0.0002302847214
PPP1R3C	-2.640964527	0.0002305850606
CDH4	-5.057197909	0.0002307041976
CTD-2003C8.2	-5.594257181	0.0002325843762
SMAD3	-1.439773094	0.0002334668178
DAG1	-1.728892896	0.0002334668178
ELF1	-1.565520375	0.0002336127853
C11orf95	-1.507457449	0.0002376789918
ABCC6	-4.629763366	0.0002383591646
PROM2	-4.210973149	0.0002385283845
ITPRIPL1	-1.43474733	0.0002397119019
ZNF107	-1.593153479	0.0002398397476
ORAI2	-2.68372196	0.0002400735245
CRIP2	-3.59710229	0.0002401225806
RAC1	-1.355164181	0.0002444102261
SPOCK2	-4.419469512	0.0002474997344
ZSCAN16-AS1	-1.461219801	0.0002479286896
FOXG1-AS1	-6.018204201	0.0002485594216
ZNF345	-1.487476185	0.0002503699054
TNKS2-AS1	-3.728040785	0.0002505536513
RCSD1	-5.846109352	0.000250956634
WDR20	-1.134379684	0.0002543925844
C1S	-3.414174396	0.0002547136878
NOV	-4.19664866	0.0002554299791
SSBP2	-2.282717774	0.0002608338993
AREL1	-1.10195675	0.000261842113
TMEM132B	-5.376276334	0.0002630405374
SGCZ	-6.75768137	0.0002631858383
BPGM	-1.08817451	0.0002632690706
GSTK1	-1.522254375	0.000264418092
LINC01060	-4.844039641	0.0002649837324
TLX2	-6.112251598	0.0002666913165
C7orf73	-1.13432957	0.0002669831816
ALPL	-6.109260454	0.0002684555945
DIRAS1	-1.729016034	0.0002695335243
ITGB3	-4.084554332	0.0002698704952
ALKBH4	-1.826707945	0.0002699207175
GRIP2	-5.616497764	0.0002701641166
BDNF-AS	-2.440499658	0.0002701641166
IFI44L	-6.823180994	0.0002716000772
TMEM71	-3.913995647	0.0002721894682
PLTP	-3.39070818	0.0002735563401
PIK3R1	-1.600638945	0.0002735563401
PTGIS	-7.463248248	0.0002768317054
PCGF3	-1.337546151	0.0002768498244

LRFN5	5.64381574	0.001537452728
RP11-26J3.1	3.195826099	0.001540317144
CTD-3131K8.2	1.618109617	0.001550529686
RIPK2	1.600186203	0.001553100893
ANKRD18CP	3.527253378	0.001554145871
AC098824.6	2.277679948	0.001557595646
RP11-108K3.1	5.359911445	0.001590979682
KCNT2	3.799814021	0.001593418654
FUCA2	1.404027309	0.001596606665
RBL2	1.2240706	0.001610059573
RABGEF1	1.725345461	0.001619465417
JPH3	5.022404975	0.001624836458
PTPRU	2.489698728	0.001652467175
SLC38A10	1.478383016	0.001672292373
ISOC1	2.401780476	0.001677124986
SLC12A7	2.016885754	0.001677124986
TEAD4	1.779013698	0.001677124986
CEP76	1.132219521	0.001681637115
C17orf58	1.031863211	0.001681637115
ZDHHC11	2.532078098	0.00169144635
EMX1	5.699629578	0.00169985767
FAM133A	4.545321191	0.001704653086
CYP51A1	2.527089481	0.00171374841
CASC20	5.373598885	0.001717624055
SERPINB7	5.553020716	0.001719880577
ECSIT	1.639205203	0.0017216827
PYCR1	1.864939242	0.001731836472
GAS5	1.714774757	0.001737349134
MSTO2P	1.003346545	0.001751780518
ERMP1	2.208046787	0.001778247234
LINC00858	5.202331	0.001792908341
NUCB2	1.141075358	0.001818909996
RGL3	3.249799844	0.001823263231
KCNMB3	1.482012857	0.001825882795
CTD-2575K13.6	3.810233118	0.001836237149
RASAL2	1.2850993	0.001839736086
CHCHD6	1.473421929	0.001863256782
SP110	1.142791827	0.001863938051
AQP11	1.622595425	0.001878547573
HERC5	1.393566605	0.001888464442
MRPL2	1.950014643	0.001921733875
ZNF850	1.842531017	0.001923349143
CCDC28A	1.391034511	0.001931643352
ISG20	3.006849006	0.001931643352
RPS6KB2	1.231233459	0.001936588515
TBX6	2.019051451	0.001937940454
MAFF	2.134233766	0.001939639259
OVOS2	2.16237399	0.001943611156
PHOSPHO1	4.98924047	0.001954683979
RP11-273G15.2	4.355439323	0.001960726502
SERPINB9P1	4.380934561	0.001960820253
ARL1	1.058415242	0.002008049768
CLIP4	1.856614128	0.002016757808
CUL2	1.170760209	0.00202539352
PDP2	1.632040955	0.002034066042
GPR160	3.521102476	0.002042541638
CTD-2589M5.5	5.00840386	0.002049313577
CT45A5	5.30697274	0.002068634764
CTA-292E10.6	1.83530987	0.002103897253
EFNA5	3.700532072	0.002104887158
C1orf115	2.761689151	0.002105672566
COL12A1	3.37911517	0.002110433248
TIFA	1.518750588	0.002117810883
CTC-455F18.3	5.356960969	0.002118365116
LMNTD2	3.368227925	0.00214247161
RP11-276H7.2	4.864001821	0.002168345373
DSG2	1.868598967	0.002171615486
RP11-445O3.2	5.297465727	0.002174603479
FKRP	-2.060296406	0.0002794688007
ST6GAL1	-2.173197724	0.0002812994827
ITGB5	-1.654527117	0.0002812994827
RP11-698N11.2	-5.905142346	0.0002839375426
MTSS1L	-1.986879617	0.0002875948151
CUEDC1	-1.785673907	0.0002877944497
RAB9B	-1.996872307	0.0002884489359
RP11-218E20.3	-6.164438919	0.0002884489359
SHROOM3	-2.421218774	0.0002887211469
CARHSP1	-1.442886255	0.00029491203
HIF3A	-6.040929086	0.0002954027247
SBK1	-1.525217342	0.0002963353223
SPRED1	-1.48976821	0.0002999376624
CILP	-6.052061456	0.0003006211507
C12orf76	-1.546014759	0.0003033741668
ASXL3	-6.566258252	0.0003043854911
CCDC144A	-4.443138448	0.0003052356379
VEZF1	-1.071052799	0.0003063860927
COA1	-1.041327704	0.0003164258104
SEMA3A	-2.196764502	0.00031877083
WFDC2	-5.127485028	0.0003198412785
NUPL2	-1.622198945	0.0003211259925
TMEM117	-1.419785806	0.0003253366882
TMEM206	-2.139153035	0.0003281907788
GBAP1	-2.070216454	0.0003301266904
LINC01748	-5.173272614	0.0003304188215
PIFO	-4.663890261	0.0003332156741
ICMT	-1.14326692	0.0003345020124
TMEM189	-1.3539642	0.0003350175392
DCLK2	-2.814230064	0.0003362538146
TADA2A	-1.238555727	0.0003362883058
AC007204.2	-6.208465703	0.000337371729
SPRY1	-3.912389351	0.0003397114271
RP11-384F7.2	-5.770543132	0.0003414203548
GANC	-1.099202034	0.0003416792693
CLIP2	-2.743310964	0.000342114741
BTBD7	-1.364353956	0.0003465261245
RP11-838N2.5	-6.037508918	0.0003469971602
ZNF433	-3.222613454	0.000347826369
GPR162	-3.225098104	0.0003482257229
ZBTB16	-5.496267258	0.0003486629255
DCT	-4.377877722	0.0003535605571
SLC25A18	-5.786427008	0.0003601694872
SRPK2	-1.236213978	0.0003627820659
C9orf170	-5.720069529	0.0003627820659
ERCC3	-1.077530913	0.0003628295109
ZMYM5	-1.420805513	0.0003633304469
LIMK1	-2.803878421	0.0003640081845
CFDP1	-1.457273581	0.0003640081845
FAM47E	-3.61979543	0.0003658306901
GDF11	-2.140199909	0.000367709583
TULP4	-1.110666178	0.0003684769491
OGDH	-1.526948973	0.0003706432143
TMEM178B	-4.884795858	0.0003708921934
RC3H2	-1.025091362	0.0003738298148
UBR7	-1.050748698	0.0003749530334
PYGM	-3.789423953	0.0003749530334
DDX24	-1.195062903	0.0003765252545
ZFP2	-3.585595095	0.0003767621378
TRUB2	-1.977349448	0.0003787311734
BRI3	-1.207237716	0.0003791440969
RPL23AP7	-1.573655905	0.0003792240745
REV1	-1.047698989	0.0003799781326
MASP1	-7.7266062	0.0003823726403
PKIG	-3.052054967	0.0003837702321
CTD-2528L19.6	-1.793558835	0.0003862636578
ROBO1	-1.805351697	0.0003863106457
BACE1-AS	-1.443267009	0.0003869969702

**MUC3A**	3.821755621	0.002175368184
**NPDC1**	1.878743729	0.002187760254
**CFAP57**	3.619133066	0.002193760096
**ERVK-28**	3.507293559	0.002219708396
**HGS**	1.304995872	0.002227426846
**UXT-AS1**	2.187486049	0.002240510999
**B3GAT2**	4.326432392	0.002243405636
**AC007040.11**	3.466808863	0.002252917358
**TNFRSF10C**	2.63441837	0.00225495612
**GXYLT1P5**	5.399280338	0.00225750151
**SLC25A27**	3.59421987	0.002270242234
**MYH16**	4.529989775	0.002315491339
**MYO15B**	2.216823759	0.002319364735
**IDI1**	1.411945221	0.002323803762
**PRSS53**	1.691305524	0.002325730339
**AC137932.6**	4.936227677	0.002326603314
**RNASET2**	1.772774899	0.002330761318
**SYBU**	2.901292934	0.002349839911
**RNASE10**	5.455883562	0.002355302083
**CARD8-AS1**	1.859352843	0.002363507089
**TMF1**	1.43985376	0.002405874455
**ASIC3**	1.971349946	0.002406035247
**RP5-1011O1.2**	3.750543005	0.0024213379
**PIGZ**	1.453323206	0.002424063441
**LINC01024**	1.414845232	0.002426669236
**SQSTM1**	1.3876862	0.002433702749
**TYSND1**	1.019071886	0.002446071019
**ABC13-4748860**	2.813551695	0.002448828568
**PDE11A**	3.453832853	0.002472297346
**RARS**	1.340440911	0.002505096368
**RP11-783K16.5**	2.211442807	0.002505096368
**GOLGA7**	1.083449249	0.00250931742
**FES**	4.199193872	0.002515623008
**ARHGEF10**	1.376884529	0.002520603555
**BUB1**	1.427294233	0.002530619371
**MRPS22**	1.079874409	0.002533151074
**PDE4DIP**	1.048544431	0.002556672903
**PLIN1**	2.986764859	0.002564297106
**NUAK2**	2.415385596	0.00256771684
**RP4-621F18.2**	2.891474187	0.002586145421
**RP11-686D22.5**	5.227165141	0.002588758152
**TUBA4A**	2.110338898	0.002610244378
**SPIDR**	1.202417229	0.00262751235
**TCHH**	4.085335028	0.002644818322
**CDKL1**	1.247930004	0.002650618585
**STRBP**	1.054375095	0.002660599866
**NLRP3**	3.510340128	0.002667442274
**CCDC125**	2.030840177	0.002684419434
**TLCD1**	1.908959851	0.002690394619
**NBEAL2**	1.842945699	0.002691297434
**RP11-982M15.6**	4.795134696	0.002743005064
**GLTSCR1L**	1.038213169	0.002751679981
**CR848007.2**	3.432343229	0.002751826633
**NOB1**	1.39200049	0.002764911897
**GOLPH3L**	1.177245506	0.002768792228
**PANK4**	1.300454609	0.002768792228
**PACSIN1**	3.338448909	0.002770847259
**TARID**	5.131806908	0.002771968959
**CDT1**	1.835515085	0.002772749002
**CTD-2314G24.2**	5.351285335	0.002777985103
**COX6B2**	3.714060817	0.002816058129
**GTF2H5**	1.372123801	0.002844422083
**WWC1**	1.632212632	0.002876683197
**ANG**	2.160063068	0.002890435816
**FAM49B**	1.891508876	0.002892038074
**SDHB**	1.342177147	0.002924637615
**S1PR1**	3.41104212	0.002942048979
**ACBD5**	1.260759956	0.002947921121

**WDR25**	-1.868920238	0.0003873956196
**LIPH**	-6.158177188	0.0003900339272
**CARMIL1**	-2.381485444	0.0003914098214
**ESPN**	-4.848843912	0.0003915218311
**ADGRL1**	-1.685372987	0.0003937163236
**PCDH12**	-6.013656318	0.0003939582907
**DNASE1**	-1.684415537	0.0003939582907
**THEMIS2**	-3.546700876	0.0003948597105
**XK**	-6.163172758	0.0004001814932
**APOL4**	-6.446356989	0.0004006808264
**CAMKV**	-3.402302075	0.0004019778668
**ZKSCAN4**	-1.777717674	0.0004040534732
**LCMT2**	-1.408766587	0.0004046466973
**ZEB2**	-2.988642347	0.0004082361186
**FLRT1**	-5.812831697	0.0004106557432
**LPAR6**	-7.048559134	0.0004144617019
**RP11-216B9.6**	-2.91571161	0.0004165283251
**MKRN7P**	-5.89447407	0.0004172726828
**ABCC6P2**	-5.74302664	0.0004173912658
**SAP18**	-1.211323091	0.0004215602091
**TRIM27**	-1.456792493	0.0004305401852
**TAPT1**	-1.490575276	0.0004309331332
**KCNIP4**	-6.319513904	0.0004316625232
**ZBTB34**	-1.243684454	0.0004317803252
**AKIRIN1**	-1.335905692	0.0004343339933
**LLNLR-245B6.1**	-1.761195398	0.0004349915419
**STAG3L2**	-1.470266106	0.0004350216878
**RAB8B**	-1.343883294	0.0004350522156
**AF127936.9**	-2.501819757	0.0004353287072
**PALMD**	-6.724504384	0.0004437422021
**TMEM198B**	-1.744418197	0.0004437422021
**CDC42EP5**	-3.495141466	0.0004480469678
**TMTC4**	-1.460991474	0.0004490877643
**SERPINF1**	-4.706854681	0.000449774211
**SLITRK3**	-5.239480202	0.0004522480856
**OSCP1**	-1.844143884	0.0004525887744
**RP11-347E10.1**	-5.757603834	0.0004553496524
**UBXN2A**	-1.350383618	0.0004597840065
**COL23A1**	-7.352899084	0.0004605727315
**CTB-113D17.1**	-5.734937449	0.0004607993101
**TTC39A**	-5.479218511	0.0004692761231
**SLC35E3**	-1.158362154	0.0004712153264
**TOE1**	-1.530474944	0.0004740127705
**TERF2IP**	-1.082798588	0.0004755326231
**UBTD2**	-1.262416715	0.0004755326231
**ADAM23**	-2.947333749	0.0004757096053
**ACAP3**	-1.997884781	0.0004761260454
**ILDR2**	-3.688454154	0.0004785281393
**FAR2**	-4.923025966	0.0004855861352
**TTL**	-1.252130018	0.000490028044
**NEUROD1**	-5.863123254	0.0004901415089
**ZSCAN26**	-1.600397702	0.0004901415089
**CHAMP1**	-2.329984077	0.0004946314486
**RHOQ**	-1.664656514	0.0004979391255
**PLPPR3**	-6.030245825	0.0004993375561
**C2orf70**	-5.633195204	0.0004999601834
**CHN1**	-1.329591775	0.0005003871812
**PPIF**	-1.393159109	0.0005048035131
**AMMECR1L**	-1.161534664	0.0005092677503
**MARCKS**	-2.078331531	0.0005124452688
**RP11-227D13.1**	-5.867579877	0.0005170571664
**BMX**	-5.888720786	0.000517729753
**IKZF2**	-3.180650412	0.0005178334728
**SLC25A13**	-1.885490752	0.0005179845633
**THBS4**	-1.731446231	0.0005182361025
**PCDHGB7**	-4.301777807	0.0005182361025
**SMAD4**	-1.262961469	0.0005198886765
**SEMA4C**	-1.503836974	0.0005274199798

RP11-568K15.1	3.239117993	0.002972692154
HLA-H	2.172928841	0.00297946098
CBFA2T3	5.8058382	0.002982156961
SLC16A3	3.766084138	0.002985825474
AP000640.10	5.400424159	0.002995682983
ARL17B	2.118620332	0.002998582038
ITPKA	2.305681724	0.003012175373
ACAP1	4.827890215	0.003023538269
KATNA1	1.437395811	0.003043211262
PDGFRL	2.448051652	0.003045559286
APOL6	2.402125252	0.003052370147
TIGD6	1.090552702	0.003058712393
FLII	1.199308545	0.003062528375
NDUFC1	1.149202519	0.00308601753
LARS	1.0904858	0.003111621492
SCYL1	1.545494851	0.003111621492
AC069277.2	5.147331362	0.003112925884
RPS3	1.164655539	0.00311883998
CTBS	1.238577651	0.003157362787
AP000695.4	3.416925837	0.003157362787
IFIH1	1.636605485	0.003179519061
DSTN	1.057220262	0.003179519061
AC018816.3	5.17125313	0.003190497569
RP11-565A3.2	5.252153328	0.003195230488
BIRC2	1.296061225	0.003233757508
TESMIN	3.937736647	0.003243881235
PRDM9	5.196390509	0.003255958878
PIK3CB	1.190227231	0.003266392816
OSR2	1.816724702	0.003292340746
BTC	4.191286454	0.0032937472
FEZ2	1.606738363	0.003314191503
GTPBP2	1.097211251	0.003319575422
DHX34	1.266315824	0.003350781558
RP11-141C7.5	2.647802042	0.003350781558
USO1	1.222457362	0.003376315235
SPATA4	3.165186094	0.003400696606
GSTP1	1.438224407	0.003408418853
C15orf48	4.012313451	0.003439412021
MPP4	2.945211565	0.00344669011
UCHL1-AS1	3.427530133	0.003512105265
AC129492.6	4.059740766	0.003514426529
F12	2.241611681	0.003514683548
GNAL	3.812435713	0.003517352423
PHLDB3	1.84834765	0.003529503524
AC140912.1	3.255235499	0.003529503524
CHRAC1	1.221376182	0.003531097158
CUL5	1.13192506	0.003545923711
ATAD5	1.107936454	0.003567230275
RP11-626P14.2	2.26797985	0.003569273891
CTD-2126E3.1	2.962621907	0.003576745562
RBM24	2.29393593	0.003611354347
MLF1	1.891544441	0.003629853842
CFAP36	1.228849857	0.003673241321
RNF139	1.54899124	0.003681355462
ROBO3	1.89230183	0.003686703315
COQ2	1.35206452	0.003692141614
EPRS	1.30570885	0.003705242083
LINC01776	3.983772495	0.003706084058
RP11-211G23.2	5.094660684	0.003707479166
CSNK1A1	1.216822539	0.003725529025
RP11-114H21.2	5.281203818	0.003726359863
DHDH	3.430326768	0.003742443548
DNAI1	3.383229077	0.003745839579
CCNA2	1.282386671	0.003748401343
APOBEC3F	1.912420522	0.003751288281
POLH	1.323002951	0.003754433076
ALMS1-IT1	1.478924142	0.003776549229
AC005264.2	5.278574391	0.003777830725

PSMC1	-1.094637772	0.0005311712444
PTGS1	-6.637075475	0.0005370997692
RND1	-2.925855885	0.0005392205274
RP11-428J1.5	-1.543597441	0.0005411830668
AP5S1	-2.031933481	0.0005424818889
CTD-2516F10.2	-3.097854954	0.0005424818889
ARMCX6	-1.865301847	0.0005477570947
LINC01630	-5.607228072	0.0005489052178
DDX11L2	-2.455600847	0.0005489052178
NAPEPLD	-2.582972854	0.0005514826019
MOCS2	-1.528636886	0.0005562311047
SAMD1	-1.137949242	0.0005568899314
CD4	-5.985825446	0.0005569540785
UCK1	-1.638566767	0.0005582483183
PTPMT1	-1.543709706	0.0005589973629
COX11	-1.49664347	0.0005617123892
NOL4L	-2.381079703	0.0005693639673
SOX13	-1.677816412	0.0005699552522
MBTD1	-1.252823664	0.0005716167567
AC017104.6	-3.316715648	0.0005752778421
WNT7A	-6.340549636	0.0005765290809
ZNF79	-1.620314897	0.0005823330771
PCYOX1	-1.349475538	0.0005853651584
RTKN2	-1.972386547	0.0005853651584
ST3GAL5	-3.300851484	0.0005889573993
AGAP4	-1.336505827	0.0005907559172
PCMTD2	-1.106555353	0.0005921613251
ALS2CR12	-2.642484351	0.0005931197565
FOXB1	-6.182157265	0.0005943278771
POTEI	-2.312419967	0.0005943278771
NUP188	-1.449043729	0.0005966540464
ANKHD1-EIF4EI	-1.934119146	0.0005969277368
B3GALT2	-5.292320752	0.0006004662344
PURG	-2.258714086	0.0006004662344
RP11-263K19.6	-2.906382897	0.0006004662344
PLAGL2	-1.067026298	0.0006024485267
MKLN1	-1.227464971	0.0006058288556
SVIL	-2.159804119	0.0006073678833
PLA2G5	-6.415639157	0.0006116149962
STON2	-6.103953149	0.0006116880584
CDKN1C	-1.728690271	0.0006121123269
ERCC8	-1.540618859	0.0006137193714
RP11-507K2.3	-5.740163394	0.0006139981934
AP5M1	-1.362066745	0.0006156871384
CD27-AS1	-1.901100354	0.0006156871384
PDS5B	-1.237664612	0.0006202012226
WI2-1896O14.1	-5.597763786	0.000623047932
ANKFY1	-1.185855761	0.0006305477162
FAM126A	-1.484986907	0.0006322646672
CIRBP	-1.846245573	0.0006366998712
PCDHGC4	-2.834557638	0.0006379300563
KCNJ9	-5.947140709	0.0006401362418
SP9	-5.970839704	0.000642315236
STAT2	-1.017569891	0.0006428825236
FMNL3	-1.62325652	0.0006452305685
ZNF701	-2.042174059	0.000645672792
NAV1	-1.51918148	0.0006458893652
RALGDS	-1.427784417	0.0006472593819
TMEM57	-1.29783017	0.000647412725
ADAMTS5	-4.373592374	0.0006509266293
SEC61G	-1.480040005	0.0006510800115
PTPN4	-1.779165956	0.0006525371977
ZFP30	-4.077732769	0.0006545522028
PRKRIP1	-1.683699027	0.0006566778147
MT3	-7.567759186	0.0006568096718
FGF14	-5.749996286	0.0006607185698
LRRC8A	-2.405116221	0.000666712098
SMTNL2	-5.861382662	0.000671159774

**TMEM63B**	1.061736475	0.003785927863
**SCRN2**	1.171469588	0.003810602659
**MASTL**	1.299320954	0.003834401889
**RCE1**	1.191441511	0.003845918176
**MYADM**	1.73723218	0.003874123523
**AJ003147.9**	3.526535287	0.003895413923
**UFC1**	1.074607799	0.003906506877
**CCDC163**	2.65879604	0.003906506877
**HOXC10**	3.240617099	0.00391914543
**NLRP14**	3.395962833	0.003923245392
**ARMC1**	1.061680502	0.003941858562
**C11orf68**	1.767235805	0.003946119758
**C2orf82**	2.143657279	0.003954404804
**CFHR1**	5.121549009	0.004039683638
**C11orf65**	1.684546621	0.004043590229
**TMBIM1**	2.98133395	0.004057393204
**RPL23AP49**	2.31440418	0.004065703319
**RRAS**	2.350801858	0.004097961974
**VDR**	3.149456286	0.004114630799
**ZUFSP**	1.758944026	0.004122196302
**RPL3**	1.104790604	0.004135022035
**RAC3**	1.538541062	0.00413613322
**GUCY1B3**	2.333102441	0.004168022024
**KRT80**	3.616414346	0.004177499405
**ANKRD49**	1.129734606	0.004215522146
**HACL1**	1.526402891	0.00422753797
**DLGAP4-AS1**	3.802442206	0.004250083204
**RP11-432J22.2**	1.669411574	0.004252190209
**EPS8L1**	1.854089418	0.004254237365
**RP11-504P24.9**	1.962017455	0.004254237365
**NXNL2**	3.751925868	0.004269207885
**MYO1B**	3.725396612	0.004269690228
**PNPLA7**	2.036273424	0.004279685898
**HTT**	1.21345148	0.004283982501
**TXLNA**	1.00075337	0.00428866993
**EBNA1BP2**	1.46270155	0.004326468787
**RP11-98I9.4**	1.842933091	0.004333315917
**TIMM44**	1.110064762	0.004401082052
**PLPP2**	2.606953319	0.004421971241
**HNF4G**	2.965984565	0.004433280276
**RP11-610P16.1**	2.850455675	0.004433280276
**RP11-547D24.1**	4.721708797	0.004476192619
**PVT1**	1.306446058	0.004494224275
**WASHC5**	1.071949136	0.004498910378
**MIIP**	1.563275249	0.004499888838
**NBEAP1**	3.070261518	0.004506808122
**ATL3**	1.035562144	0.004523243843
**LINC01111**	5.511718329	0.004537567949
**EML3**	1.062047184	0.004548928071
**PEX2**	1.294604673	0.004559218413
**RDM1**	1.579161529	0.004566798972
**CLPB**	1.155363054	0.004580350482
**NUDT17**	1.179491928	0.004580350482
**RADIL**	2.48487725	0.004582944642
**BASP1**	3.304094878	0.004583330498
**METTL5**	1.080075828	0.004598870541
**JAK3**	2.839035813	0.004626541908
**RP1-137D17.1**	5.038749263	0.004627321859
**TBC1D9B**	1.363305773	0.004627839498
**SHOX2**	1.181678162	0.004638412076
**RASGEF1C**	2.275630924	0.004640907386
**HLA-F-AS1**	1.5497679	0.004645809335
**MRPL11**	1.16394183	0.004654054476
**C17orf67**	1.822621419	0.004682166281
**EIF4EBP1**	1.225661651	0.004707930298
**HERPUD1**	1.397410583	0.004712102819
**PSD4**	2.965774862	0.004759672167
**DVL1**	1.684340606	0.00486452169

**PHC2**	-2.336265844	0.0006842141664
**DMTF1**	-1.362356312	0.0006842141664
**RP5-912I13.2**	-5.21900265	0.0006875965339
**FBXL3**	-1.540465124	0.000689868259
**TRPM8**	-5.743879581	0.0007069424627
**LIG4**	-1.759531589	0.0007069424627
**RNF38**	-1.043244419	0.0007106349871
**RFLNA**	-5.690737627	0.0007119552039
**THYN1**	-1.457705797	0.0007120292583
**CHIAP3**	-6.260111233	0.0007126999899
**C7orf43**	-1.530748781	0.0007135491804
**HCG17**	-4.4800824	0.0007135491804
**LIFR-AS1**	-3.059186499	0.0007322952109
**AC002454.1**	-5.217373136	0.0007379084536
**HNRNPA2B1**	-1.125577478	0.0007407571256
**EFCAB5**	-4.111277953	0.0007431640847
**DNM3**	-2.772483874	0.0007435643577
**NOTCH2NL**	-2.710624492	0.0007488159176
**BGN**	-5.615044703	0.0007510874363
**WSCD1**	-3.513596317	0.0007536343928
**SNX13**	-1.49318122	0.0007537808265
**ZFP14**	-1.672086188	0.0007567628286
**PDGFA**	-3.631335924	0.0007572727831
**RAP1GDS1**	-1.39906776	0.0007700383063
**FAM107B**	-2.376139257	0.0007711603332
**RP11-307B6.3**	-5.297361506	0.0007717132749
**IPCEF1**	-5.714955828	0.0007718938547
**SRI**	-2.491182572	0.0007773363264
**TBL2**	-1.483785536	0.0007781993894
**MFAP3L**	-1.692346466	0.0007781993894
**ZNF37BP**	-1.528435326	0.0007811011135
**POPDC2**	-5.778403573	0.0007816006471
**CSPG5**	-3.674995611	0.0007828209038
**FGFRL1**	-1.148882151	0.0007861963945
**ADCY8**	-4.317399422	0.000788357724
**ARL8A**	-1.86535076	0.0007928906959
**LYRM4**	-1.177468368	0.0007928906959
**TSNAXIP1**	-3.152207559	0.0007935908597
**MIMT1**	-6.075412883	0.0007965113531
**ALDH6A1**	-1.039424646	0.000800734132
**MINDY1**	-2.12224402	0.000800734132
**TMEM199**	-1.446290608	0.000800734132
**NXPH3**	-3.269626057	0.0008044918826
**KAT14**	-1.451512776	0.0008071111657
**SS18**	-1.026121465	0.0008113693346
**PROSER1**	-1.556303873	0.0008139272622
**C14orf132**	-1.479014586	0.0008142137478
**EGLN2**	-1.245875573	0.0008240334286
**DIP2A**	-1.190813364	0.0008271225309
**SMC5**	-1.272808196	0.0008278016108
**OLFM2**	-3.078028161	0.0008292004769
**NCAN**	-7.567858401	0.0008292004769
**MESTIT1**	-6.156109399	0.000840727958
**STAG3L1**	-1.771892284	0.0008534749287
**LRP1B**	-4.207098573	0.0008552203791
**AC027612.6**	-5.124045217	0.0008662936612
**LINC01102**	-5.979861627	0.0008665028436
**KIAA1161**	-1.411651014	0.0008702760952
**CERKL**	-5.870283045	0.000871424431
**ZBED3**	-1.176978703	0.0008807519926
**ATP6V0A2**	-1.592371826	0.0008893852279
**ADGRG1**	-4.440798232	0.000895656285
**NSG1**	-5.782386613	0.0008964264316
**SCFD2**	-2.162771664	0.0008994181883
**HKDC1**	-4.63898605	0.0009022556453
**LINC01158**	-3.599358667	0.0009065648927
**RP11-644F5.11**	-1.56797691	0.0009066423293
**EFHC2**	-5.800872569	0.0009087708486

RP11-755F10.1	4.290032186	0.004897947718	RAP1GAP	-1.748237365	0.0009118366537
MTFR2	1.643463726	0.004919819189	SCAF4	-1.029350091	0.0009154269133
PARP4P2	2.869266728	0.004929574104	TENM2	-4.729213432	0.0009187397596
XXbac-BPG294I	2.530545502	0.004953593184	ABHD11	-2.239355234	0.0009193049761
AC068831.10	2.283396586	0.004956503438	RP11-78O7.2	-3.269803284	0.0009193049761
FANCD2P2	4.566029376	0.005023118383	SMARCC2	-1.063520582	0.0009264981633
TAF2	1.180512274	0.005026043336	ZNF114	-1.497348512	0.0009297164672
CAPN11	4.393558384	0.005035104753	HEPACAM	-5.76113418	0.0009309387314
TMEM14C	1.133322474	0.00503944412	HOXA2	-4.939077497	0.0009362885439
RAB26	1.469735496	0.005051953101	TTLL11	-1.55986009	0.0009413369038
KIAA1257	2.27128739	0.005127991526	POLR1A	-1.140018193	0.0009425598121
NBPF3	1.012849574	0.005206256381	ITPR2	-2.667634268	0.0009478641548
MAP7D3	1.522062147	0.005224196594	AFDN-AS1	-2.1176868	0.0009529911553
POLR3G	1.456386204	0.005226528405	GBAS	-1.696377425	0.000955493229
RP11-676J12.7	5.871415047	0.005231143184	SHD	-5.432634289	0.0009578666132
RPS20	1.112044108	0.005237988735	NUPR1	-4.420568895	0.0009594141259
CTD-3220F14.1	2.781303803	0.005275095619	ZNF616	-2.103509346	0.0009628237861
TRABD	1.696639361	0.005296480166	MORN3	-3.897658865	0.0009642532481
PPM1F	1.149143685	0.005310336115	AP000289.6	-6.149069418	0.0009735564463
OTUD7A	2.944783315	0.005327635563	RP5-1159O4.2	-3.33637214	0.0009783005669
ADPRHL1	2.254185903	0.005384236398	CTB-118P15.2	-5.481705306	0.0009795451864
HINT3	1.262389731	0.005391968545	CTC-467M3.1	-5.646775277	0.0009841545403
ANXA4	1.080039129	0.005402096904	PLK2	-2.114942395	0.0009889705959
RP11-147I3.1	2.66104179	0.005409339547	ANKRD20A11P	-2.370088227	0.0009919611781
OLA1	1.449919663	0.005459200033	MCF2L	-3.078548474	0.0009967475061
TMEM74B	3.229025508	0.005466152176	TMEM229B	-3.115280397	0.001000179647
MYCBPAP	3.297800959	0.005493085806	YLPM1	-1.017819958	0.001002470897
MRPL24	1.343023087	0.005513507089	TNPO2	-1.564672486	0.001013418859
WDR93	2.381798737	0.005540520446	TRIP4	-1.419664876	0.00102050976
MYL12B	1.615088367	0.0055485532	TSR2	-1.198898478	0.001021421023
HLA-DOB	2.803747692	0.005555689097	GIGYF1	-1.146287782	0.001023528166
FH	1.277212987	0.00556589146	ZNF407	-1.591542975	0.001024460409
ZNF330	1.349936054	0.005611983118	FAM13B	-1.149549599	0.001024926722
SEZ6L2	3.652941782	0.005649555438	MON1B	-1.055730348	0.001026221568
NDUFS7	1.919638148	0.005654947971	TRAF4	-1.517127663	0.001027681666
GNPDA2	1.179762934	0.005679079249	PSAP	-1.654737776	0.001027753675
ACAD11	1.659899111	0.005686184971	LINC00643	-5.798397739	0.001033118589
MLKL	3.117992487	0.005690931267	LAMP5	-6.152030404	0.001033912465
ENKD1	1.593276914	0.00570051321	TRRAP	-1.148486158	0.001039348434
PAPSS2	3.231283632	0.005736179243	SLC35B4	-1.401453987	0.001042435042
ARHGEF4	2.192242113	0.00579894925	PLGLB2	-5.481491181	0.001042665889
RFNG	1.540183514	0.005864186363	CH17-258A22.4	-4.106535203	0.001045535751
WWP1	1.338351949	0.005874097686	ASB6	-1.675661709	0.001047795236
TMEM87B	1.286252218	0.005893794913	HKR1	-1.432214895	0.001068682443
RP6-24A23.3	4.523628477	0.00589811513	CTC-425O23.2	-2.852989458	0.001073961961
NDNF	3.574724585	0.005940539682	JAZF1	-2.551653884	0.001074038028
THAP4	1.375506715	0.005940539682	TCF7L2	-2.759525068	0.001075604654
TBC1D9	1.098874602	0.005964505308	LDOC1L	-1.611056581	0.001080178245
APRT	1.454839965	0.005964505308	ARMCX5-GPRA	-1.407923176	0.00109723521
MYO7A	2.618041536	0.005981245505	PTPA	-2.074087073	0.00110347967
ADGRF5P1	3.719127858	0.006023151681	PSPH	-1.257732618	0.001108589596
SLC25A32	1.234185867	0.006048102946	PSD3	-2.587745319	0.001111568331
RFWD3	1.11757597	0.006083751041	PGBD1	-1.425982376	0.001114511942
HLA-B	1.466645377	0.006089122383	RP1-273N12.4	-4.680797573	0.001114511942
CMSS1	1.237816528	0.006126000265	SNRNP35	-1.116655399	0.001132352051
PAK1	2.269201331	0.006131264834	YKT6	-1.768037349	0.001135622681
CTD-3092A11.1	1.655251826	0.006136020716	C20orf96	-1.142411246	0.001137296259
ACY3	3.489089775	0.006138327497	BMPR2	-1.229577638	0.001139058945
LETM2	1.639964307	0.006151047724	BAMBI	-2.710763062	0.001146776917
PIP4K2C	1.415475899	0.006161049313	FAM185BP	-3.097363335	0.001146776917
ENO1	1.041031541	0.006201122458	RGS2	-2.691446825	0.001149342922
SOCS2	3.062451408	0.006220676822	TMEM201	-1.551406696	0.00115425012
ENTPD6	1.140937453	0.006258935377	ITFG2	-1.343425396	0.001170528924
NPTX2	3.47764829	0.006281074856	ABCD2	-2.983469516	0.001187605143
PRTN3	3.509253943	0.006287758337	KLHL4	-3.007557136	0.001188115036
RP11-167H9.4	3.150506454	0.006292529877	AC002456.2	-3.333117086	0.00119975203
IFT74	1.2144118	0.006299650906	FUS	-1.199979067	0.001206886185
MIR2052HG	3.947255514	0.006300265844	RP11-893F2.13	-2.327250988	0.001208141242

**OSGIN2**	1.231236012	0.006310215433
**LINC01885**	4.994167419	0.006321021797
**AC131056.3**	4.994167419	0.006321021797
**PCNX2**	2.539563468	0.006367894852
**LGALS1**	2.21882012	0.006370787179
**AJ003147.8**	3.284970252	0.006400022459
**PIGBOS1**	1.000880743	0.00641802659
**L3MBTL3**	1.434549104	0.006471405453
**TUBB8**	2.832842369	0.006483562325
**ACBD4**	1.590323333	0.006499035546
**PTPN14**	1.309262648	0.006518512581
**GBP1**	2.465266646	0.006524101989
**COMMD3**	1.009450084	0.006532714084
**C19orf45**	3.240817488	0.006544402877
**DEF6**	3.605515311	0.006557158622
**MYDGF**	1.276268008	0.006579413059
**DGKD**	1.075134311	0.006668917994
**B3GNT4**	2.692529731	0.006716031652
**LINC01202**	5.044433095	0.006718757131
**AIDA**	1.027806676	0.006755697512
**MRPS10**	1.418138402	0.006788305089
**NUDT18**	1.426394419	0.006788647007
**BAK1**	1.32771267	0.006789221904
**LINC01001**	2.271547507	0.006862132394
**BRMS1**	1.413838982	0.006888058205
**MCOLN3**	3.478039877	0.006921858627
**CCDC88B**	2.055545113	0.006967348885
**NDUFS8**	1.679540398	0.006992665117
**ELF4**	3.063217715	0.007009850199
**G2E3**	1.517291239	0.007024580778
**TRMT112**	1.286658906	0.007054145375
**PLCH2**	5.413050575	0.007056366685
**SPATA2L**	1.888749159	0.007077079882
**GNRHR2**	1.733210137	0.007101758843
**LINC01939**	6.274155912	0.007125126266
**ASPSCR1**	1.548665312	0.007131859271
**LRRIQ3**	1.481463817	0.007137382754
**ANAPC5**	1.354446441	0.007216952797
**AC020571.3**	4.550672319	0.007221518883
**NR2E3**	5.133960385	0.007245148391
**WASH7P**	1.870511785	0.007298988956
**YIF1B**	1.541856075	0.007312925369
**E2F5**	1.089922204	0.007348465916
**LRRIQ1**	3.918201538	0.007427381794
**PCOTH**	2.367560965	0.007460598712
**RP3-467K16.2**	4.46813714	0.007460598712
**EML2**	1.16215098	0.007464342372
**AC092614.2**	4.941359329	0.007464342372
**PIM1**	1.451187687	0.007476320772
**PSMB9**	1.835877279	0.007490382249
**CH17-13I23.3**	2.925772483	0.007526322233
**LPAR2**	1.521608389	0.007550979834
**CYB5D1**	1.498431445	0.007600996498
**CAMK4**	3.308483917	0.007607305008
**UNC93B3**	3.698699706	0.00766316423
**RP13-143G15.4**	4.43834854	0.007680267498
**NUDCD2**	1.175689411	0.007700176587
**GAS6**	1.867290279	0.007776963211
**IRF1**	1.374984132	0.007836097101
**RP11-315O6.1**	1.918246088	0.007885067528
**RAB3IP**	1.710010051	0.007890185133
**PLEKHA3**	1.205736867	0.007923721601
**ACTRT3**	3.041886853	0.007923721601
**SLC2A12**	1.408412676	0.007936253088
**RP11-415J8.5**	2.871950971	0.007936253088
**KIF17**	2.864257261	0.0080188601
**HAR1A**	4.666604453	0.008044817922
**EPB41L4B**	1.832952383	0.008062502557

**ASS1**	-4.441350673	0.001209171395
**RAPGEF2**	-1.409371468	0.001218870822
**CFAP44**	-1.662966401	0.001218901552
**LINC00032**	-3.221439138	0.001245355477
**PDE8B**	-3.700553355	0.001245744216
**ZNF273**	-1.542996435	0.001249096089
**VWA1**	-2.698634104	0.001250649091
**AC053503.11**	-5.392283488	0.00125124486
**UBE2F**	-1.537666482	0.001260329777
**SLC35A5**	-1.711519884	0.00127588523
**ZNF703**	-1.504813222	0.001283976059
**CC2D2B**	-4.527423144	0.001295538248
**INPP4B**	-2.030011082	0.001298229868
**CHPF2**	-1.997068952	0.001300539951
**RUNDC3A**	-5.397720084	0.001306188132
**LEMD1**	-5.974540089	0.001313165668
**PSPC1P1**	-4.041619398	0.001313165668
**LINC00945**	-5.54097168	0.001325653116
**CYGB**	-4.87104753	0.001327761303
**PLCG1-AS1**	-2.769857823	0.001345851591
**ATP6V1B2**	-1.567900004	0.001350594139
**ADPGK**	-1.134811002	0.001350594139
**NR6A1**	-1.929138252	0.001365775065
**TSPOAP1**	-5.614674885	0.001372251799
**ZNF510**	-1.429627072	0.001376199344
**GPR88**	-5.994892806	0.001380553291
**CC2D2A**	-1.390230277	0.001385648879
**GTDC1**	-1.111713956	0.001391514196
**HOXD3**	-5.49486847	0.001398162548
**PLEKHA8**	-1.634648153	0.001400125611
**AKR1B1**	-1.555125241	0.00140546207
**RP4-657D16.3**	-3.717522492	0.001415189303
**MTMR9LP**	-2.237656843	0.001420092965
**RRP15**	-1.458057326	0.001427397675
**DOK1**	-1.831450135	0.001437456887
**PGM5**	-4.077048408	0.001439414878
**PITPNA**	-1.191190374	0.001446673682
**RP3-405J10.3**	-2.720753833	0.001451610997
**ORMDL1**	-1.110626883	0.001455323896
**EPB41L5**	-1.761501922	0.00147055378
**SCHIP1**	-1.896485882	0.00147597191
**HOXD4**	-5.415875505	0.001492918482
**KITLG**	-4.343809756	0.001497542055
**CXADR**	-2.190400942	0.001504639423
**CACNB2**	-5.563294232	0.001504639423
**STUM**	-5.470119019	0.001504639423
**GIMAP2**	-3.133219139	0.001510593363
**LINC01948**	-5.764782325	0.001519939855
**IFT88**	-1.256084782	0.001525613517
**TNKS**	-1.461536459	0.001531083517
**LRRC75A**	-2.80130039	0.001532557354
**REPIN1**	-1.461968345	0.001535768797
**ITM2B**	-2.251890341	0.001544067822
**FAM213B**	-2.065431482	0.001544067822
**DUSP6**	-2.19281561	0.001557595646
**TNIK**	-2.903496877	0.001557595646
**BEX3**	-1.215075789	0.001579322253
**RP11-141M1.3**	-5.389178491	0.001586759001
**ZNF398**	-1.098284275	0.00159201165
**TTBK1**	-4.745425428	0.001611613291
**ADAM22**	-1.886876099	0.0016332856
**LFNG**	-4.401100276	0.001641433711
**DDR1**	-3.42640979	0.001650917708
**ING4**	-1.682920315	0.001655799854
**RP11-430H10.1**	-5.529941441	0.001677124986
**PIK3R3**	-2.00032277	0.001680898923
**ATP9A**	-2.26423251	0.00168516782
**BAX**	-1.699565106	0.00169985767

ZNF860	1.983412176	0.008071271146	ZNF618	-1.913083268	0.001704263111
SLC9A3R2	1.824836741	0.008179512043	RHOBTB2	-1.733428781	0.001708120284
SLC3A2	1.460644012	0.00830256746	SERP2	-6.098550393	0.00171374841
ZBTB38	1.447947042	0.008308455737	CD101	-5.39923216	0.001718425449
LONRF3	2.298276317	0.008325519971	GAS1RR	-4.356538262	0.001723439264
ATP1B1	1.762377221	0.008340099615	CPM	-3.507635036	0.001723473239
PPIL6	1.653086843	0.008342269707	MAPK7	-1.335662546	0.001729643191
NR2F2	1.668526158	0.008350048233	AP006222.2	-5.409402215	0.001739006595
EMC9	1.58136355	0.008369481027	RP11-612B6.2	-5.2185601	0.001739823326
CDKN2D	1.469987061	0.008371927113	ELAC1	-1.99132143	0.001743303832
PCSK4	2.193944768	0.008373008701	WDR47	-1.246292028	0.001752505851
NEK5	3.321760242	0.008394822291	C9orf85	-1.363159949	0.001757229442
PPP1CA	1.279899255	0.008451994788	ZNF607	-2.573572163	0.001758080186
TRAM1	1.308153921	0.008484773304	CASQ1	-5.875069705	0.001760278683
PGGHG	1.615200075	0.008488698839	C2orf27A	-2.48725537	0.001760278683
CNFN	4.984643918	0.008494500542	GALNT7	-1.317781676	0.001760688009
STC2	2.130682695	0.008548755807	CAMSAP2	-1.688860712	0.001775311086
ESRRA	1.238431682	0.008555358899	NDUFB2	-1.281500233	0.001782880306
SNPH	1.663377893	0.008609605431	SURF1	-1.417732177	0.001783553302
TRIM47	1.984352732	0.008609605431	PRKACB	-1.399679528	0.001786126136
PRIM2	1.453186284	0.008617399722	TRO	-1.408437789	0.001792908341
NEK8	1.273202264	0.008633506123	TRAPPC2	-1.546873006	0.001798370002
USP14	1.037878714	0.008634750459	RPS6KA2	-3.39763146	0.001799233873
HMGA1	1.952942405	0.008662006849	RP11-81H14.2	-5.286201455	0.001804927089
AC137932.4	2.795844251	0.00869507607	RP11-38M8.1	-5.989338989	0.001825357441
SLC4A9	2.868599661	0.008699825583	AP3B2	-4.743068214	0.00182738135
SLC47A1	1.87458136	0.008743852052	JPH4	-6.141442203	0.001828448329
TNFSF12	2.122352396	0.008786932218	RXFP1	-4.867732018	0.001839736086
CH17-469D17.1	5.140924526	0.008978492472	GPR19	-2.168209255	0.001843030329
BHLHE40	1.844682013	0.009011761543	CBR3-AS1	-2.111612848	0.001848250624
TOR3A	1.417181194	0.009031375209	SUFU	-1.143874065	0.001850765861
GAS2L3	1.006753704	0.009047458163	VTN	-4.957915379	0.001856139089
FAM84B	1.960970582	0.009079418097	KANSL3	-1.087773725	0.001859565864
COMTD1	2.554934906	0.009123856803	USP2	-1.702051063	0.001863938051
TM4SF1-AS1	3.203756647	0.009164230146	PHF23	-1.417662842	0.001868447494
PPM1J	2.576050295	0.009173829677	SNX11	-1.34865531	0.00189522751
TFPI2	3.082933004	0.009260298446	MAP2K6	-2.723085212	0.00189522751
RP11-65E22.2	2.198296345	0.009420437793	BTBD3	-1.310719678	0.00191197591
ANKFN1	4.844348945	0.009432152363	TCTN1	-1.353109434	0.001925945779
RP11-798K23.1	4.808731238	0.0094402494	LRP1	-2.883113987	0.001931643352
FRS3	1.298853299	0.009519040749	COL28A1	-4.494496314	0.001939639259
COA4	1.008632045	0.0095553037	NME5	-5.81429289	0.001969691609
VSIG10L	1.612915222	0.009581413625	MIR181A2HG	-3.068725026	0.001971044384
RP11-381K20.2	2.670945807	0.009598075674	PTX3	-4.433381327	0.001981496599
ADAMTS6	2.436437792	0.009602273143	NUDCD3	-1.718038597	0.001993722459
EXOSC5	1.584990276	0.009661869382	ZNF547	-1.914533431	0.001999420215
RNF34	1.042106883	0.009673761724	PTPRG	-1.644973667	0.00200099595
CNN2	1.447195185	0.009674430396	B4GALT5	-1.882836191	0.002027691965
SIPA1L3	1.621798139	0.009677210874	ANGEL2	-1.359643943	0.002028902514
SERTAD4	3.039407178	0.009693058467	LRRC75B	-2.286462868	0.002054883078
RP11-460B17.2	4.468328266	0.009700297729	SPOUT1	-1.57716728	0.002054883078
EXOSC1	1.043694253	0.009705383178	PBRM1	-1.352841199	0.002059250533
UBR2	1.274473497	0.009762832305	RP11-495L19.1	-4.385950377	0.002059250533
SRF	1.150990435	0.00980149363	TRMO	-1.823014284	0.00206196536
SNCA-AS1	4.835756356	0.009801529201	KSR1	-1.079490592	0.002067433228
IL1A	5.009246886	0.009811691222	SLC10A4	-4.641003349	0.002074884951
PRRC1	1.428758241	0.009834703337	SORT1	-1.182434614	0.002084518684
C1orf112	1.271165254	0.009836671994	CTC-301O7.4	-2.520257058	0.002121245305
MAGEA4	5.532911472	0.009842597211	ZNF444	-1.514819889	0.002121680293
RP11-816J6.3	4.80383904	0.009874746881	PHEX	-2.525576304	0.002129980868
LIPE-AS1	1.590611256	0.009882149942	ZFP92	-5.485138638	0.002129980868
PPP2R5A	1.322203669	0.009885444252	COQ7	-1.007032436	0.002132800771
CTD-2540B15.9	4.018448288	0.00988955589	INIP	-1.00002656	0.002136516719
AIM1L	4.457851278	0.009960755221	CTNS	-1.503832073	0.002136917781
MEA1	1.390541957	0.0100518035	EIF5	-1.490435122	0.00214247161
AP003068.23	2.087838199	0.0100518035	PPFIBP1	-1.562329375	0.002152984153
SMIM1	5.034602763	0.01006068444	NASP	-1.200291752	0.002166809433
AXL	2.783081733	0.01025045865	SMARCD1	-1.183170893	0.002186131154

CEP192	1.04466848	0.01026137748
AP3M2	1.290475409	0.01028301278
SFTA1P	3.346689327	0.01028588464
SLFNL1-AS1	1.774575445	0.01032988242
JAK1	1.290805482	0.01035721323
RP11-15A1.3	3.031428648	0.01041824749
TMEM126A	1.06662998	0.01042308435
POLRMT	1.543480099	0.01042935935
VAPA	1.109077985	0.0104518636
GM2A	1.005405175	0.01058741719
RAD9A	1.305442359	0.0105912549
MEIOB	5.090568695	0.01064961141
PHF7	1.178655745	0.01077870112
SF3B6	1.299698683	0.01084435673
HSPA4	1.157494729	0.01088991775
UPP1	2.831806786	0.01090030194
RP4-536B24.4	3.687087304	0.01091224381
GPR157	1.555173699	0.01091927609
FAM189B	1.18974639	0.01094929775
DDX21	1.24954371	0.01096742447
WT1-AS	2.909754676	0.0110624291
RHEBL1	1.247967664	0.01113904435
RP11-403I13.8	2.274308992	0.01115131042
RP11-503E24.2	1.464539684	0.01116260243
RAB4A	1.027069786	0.01119198074
ZNHIT2	2.572180477	0.011284547
SUGT1P1	1.941639646	0.01132040195
CTB-32O4.2	2.07953376	0.01132040195
NUDT10	2.240531042	0.01132728928
ALKBH8	1.114900193	0.01146910437
ACOT11	2.467434737	0.01154972144
SLC29A3	1.60960267	0.01155285193
BRD9P2	3.525458266	0.0115736808
AC013268.3	4.407178048	0.0116057418
AP001628.6	2.652537389	0.01166723233
LINC01021	1.955564405	0.0116975285
PAK4	1.497362863	0.0117030675
ZNF485	1.603917783	0.0117030675
RP11-122G18.1:	1.750285823	0.0117030675
AC034220.3	2.795064426	0.0117410892
CLTB	1.220606303	0.01179654722
MR1	2.059998565	0.01181950615
RPL23A	1.009886092	0.01181950615
RPL4	1.004875584	0.0118496327
NFKBIE	1.137553129	0.0118580468
FAM155B	3.459266388	0.011939212
NDUFA13	1.815466263	0.01194748927
PSTK	1.127310752	0.01195941578
NBPF15	1.127673643	0.01195941578
FABP6	4.357111374	0.01198826389
AC092159.2	2.193511264	0.01210692174
COMMD8	1.219897899	0.01213465645
PPP1R14B	1.256467816	0.01215113757
FMR1-AS1	3.142794257	0.0121972216
GALNT18	2.476880593	0.01222787732
UNC5CL	2.243079792	0.01223221056
LMAN1	1.023409725	0.01224338129
RP11-885N19.6	4.896083994	0.01226465106
IQUB	2.230426676	0.01229921336
CSPP1	1.075755792	0.01232382156
LINC01970	2.18814385	0.01232382156
UHRF1BP1L	1.090652884	0.01234487413
PAOX	2.048870373	0.01237116143
SH2D4A	2.630100743	0.01239851874
TAP1	1.190263155	0.01240232959
PRDX5	1.312170986	0.01246318459
PRKCI	1.031567856	0.01252419199
LURAP1L	2.174431828	0.01253368522

DPP4	-7.081551791	0.002187760254
C5orf22	-1.025136578	0.002193718997
PRSS51	-5.401632613	0.002200142887
VPS13C	-1.625226187	0.002209726013
IQCK	-1.413293854	0.002214891262
PLEKHO1	-1.56099081	0.002242453415
CYP2U1	-1.54916895	0.00225206805
TTLL9	-2.940491598	0.002267169685
FZD3	-2.056433612	0.002274488801
GLYR1	-1.030221614	0.00230004626
WWOX	-1.470254724	0.002302854937
ZNRF2	-1.744668359	0.002304038463
STARD3NL	-1.330645349	0.002308870484
ZSCAN31	-1.453863139	0.002312761048
FCGR2B	-5.301211227	0.002349666099
ZNF418	-1.190358746	0.002355097951
KIF2A	-1.442609524	0.002363777415
LDLRAD4	-2.851598509	0.002367170941
CRYGS	-1.571592478	0.002374161956
SLC4A4	-4.484513962	0.002394862033
WRB	-1.060185092	0.002396782596
AC005083.1	-5.100366778	0.002413394299
ARPP19	-1.185558463	0.002416539789
MAGI1	-1.938859083	0.002418317121
KLRA1P	-1.388054194	0.002431490307
PSPC1	-1.382587729	0.002433042662
CCDC50	-1.836099372	0.002433042662
CTC-255N20.1	-5.90996629	0.002439508492
RUNX1T1	-6.997520152	0.002448828568
ABHD13	-1.602081948	0.002455075867
SOSTDC1	-5.408561818	0.002457337802
B3GNT9	-2.281263144	0.00247372978
RP11-7F17.3	-5.580193964	0.00247372978
MACC1	-3.274386347	0.002476659264
MKL2	-1.04636603	0.002478787465
CARF	-1.718585399	0.002492204581
PMS2P5	-2.158910434	0.002494399158
GMPR	-3.206833988	0.002515623008
AVIL	-3.53253046	0.002533511218
SPPL2A	-1.1827833	0.002553452309
FMO5	-3.417845396	0.002559959598
CLEC1A	-7.037307196	0.002565029261
RP11-455F5.3	-2.775083786	0.002586820684
ATP2B2	-5.266123504	0.002594676316
TMSB4X	-3.35187591	0.002612236842
TBRG1	-1.010654317	0.002622542545
KIAA0391	-1.305759089	0.002626274004
AC104653.1	-3.300505301	0.002628005199
WDR33	-1.143160043	0.002645485956
GPRIN3	-5.649765006	0.002645563332
CLSTN2	-5.721474439	0.002652297541
IMPG2	-2.933315649	0.002654360523
FAM234B	-1.454317713	0.002657696648
LRRN4CL	-5.21719	0.002665502428
UBE2E1	-1.162698735	0.002684419434
ZBED3-AS1	-1.506734757	0.00271888178
RP11-196G18.3	-3.786317056	0.002722278814
TSHZ2	-6.733312498	0.002724450858
SMG6	-1.280044418	0.002746806291
AP000708.1	-5.357866885	0.002751679981
NRXN3	-5.272801432	0.002761862418
ATG9B	-3.589388111	0.002778510077
DNER	-2.999847754	0.002783631869
DGCR2	-1.20415802	0.002786456208
NOX4	-3.833526622	0.00280134791
AOAH	-5.265812186	0.002819850895
TMTC3	-1.355298178	0.002832286109
ATRN	-1.048055287	0.002847404102

METTL7B	2.219113569	0.01254948389
ZNF121	1.050329825	0.01254948389
ITGAD	4.718672953	0.01265679995
LACC1	2.317336734	0.01266671626
RIPK4	3.742394763	0.01266671626
PXK	1.10101024	0.01273246732
LGI4	3.207732691	0.01274098655
AC008592.3	4.691933851	0.01276109017
EIF5A2	1.125817127	0.01276821164
ANXA2R	1.368624168	0.0127717863
KRTCAP2	1.029343212	0.01280530371
SCLT1	1.401555349	0.01282257651
MPG	1.451553521	0.01288545546
ANAPC11	1.453076968	0.012934033
NAA20	1.124383618	0.01294606321
ATP5G3	1.513077704	0.01298827909
SPATA13	1.041755525	0.01302649019
TRIM41	1.089862148	0.01306965592
CCNYL2	3.218215789	0.01309015325
LINC01239	3.6509458	0.01310638174
ISYNA1	1.59172281	0.01311051218
ZCCHC7	1.531475121	0.01314366069
CTD-2319I12.2	2.33651796	0.01327537354
FAM3A	1.15564421	0.01331123808
HCG27	1.663921782	0.01331123808
VNN1	4.279483881	0.01347063473
NAPG	1.055555076	0.01349792432
FLI1	1.814636051	0.0134981105
NDUFB7	1.724841999	0.01351498188
COL13A1	3.051250545	0.01356327445
C2CD4C	2.615641019	0.01358591996
TTC27	1.01618063	0.01381059306
RAB12	1.451656382	0.01382833228
HYKK	2.240949091	0.01387365106
MAOA	1.636824515	0.01388672603
FAM173A	1.790835965	0.01389986998
SLC4A11	1.459091064	0.01396445195
NPM3	1.100605149	0.0139785401
RP11-1398P2.1	4.229813996	0.01403510319
MAPK13	1.818111434	0.01406113973
LINC01881	1.198299415	0.01406693285
IGF2	2.894109053	0.01406798947
AP000442.4	3.40471652	0.01410817025
PPOX	1.082271124	0.01412079581
CACYBP	1.491283369	0.01419799094
ALDOC	3.500023866	0.0142321712
CDC42EP2	1.940784982	0.01427460765
ACOT1	1.936027123	0.014304362
SIPA1	1.88859968	0.01432514328
VPS37C	1.229300215	0.01439310279
HID1	1.562757589	0.01440175027
GACAT2	3.202431636	0.01443440693
SNHG16	1.108452011	0.01444495472
NTSR1	2.362662101	0.0144580115
STN1	1.052906669	0.01453654082
SCUBE1	5.086918573	0.01454779536
WHAMMP2	1.767897285	0.01455453445
LINC00882	2.586730527	0.0145691493
TWIST2	3.54078188	0.01460170189
LINC00493	1.024922823	0.01460294371
SPERT	4.829518267	0.01464747894
ATP23	1.064346127	0.01465495581
FLJ21408	1.739172781	0.01465495581
RSF1-IT1	2.226558023	0.01476475356
DHRS11	1.102447309	0.01483763052
SRFBP1	1.217525587	0.01484868622
NR1D1	1.427238925	0.01491029533
LINC01572	1.39448343	0.0149485061
APH1B	-1.770994603	0.002847404102
KANK2	-1.675140318	0.002861384131
MRM2	-1.239198402	0.002862619913
ZNF638-IT1	-1.964433573	0.002868869478
PLEKHO2	-1.235390528	0.002883267205
KLHL32	-2.678828179	0.002900675185
ZNF891	-1.284413488	0.002908255704
FAM200A	-1.172494878	0.002918172064
TMEM164	-1.223446755	0.002918748577
OLFM3	-6.870260521	0.002924637615
RNF219	-1.551291778	0.002944286573
TBC1D3D	-4.89079955	0.002962870257
MGRN1	-1.610982278	0.002998999055
MUC12	-5.749996543	0.003006732773
RP11-247A12.2	-4.169126105	0.003010826868
COPZ2	-3.444508501	0.003022518273
NGRN	-1.399211402	0.003043211262
BAALC-AS2	-5.621475819	0.003052205524
HIST1H3E	-2.705513947	0.003073410086
GABARAPL2	-1.356573527	0.003078801489
MRPL32	-1.070717167	0.003078801489
GSG1L	-5.655331607	0.003102433649
DPY19L1P1	-1.491951695	0.003106900844
TG	-5.16811765	0.00311883998
KRIT1	-1.068110366	0.003123809116
TMEM253	-2.741413488	0.003145646479
C7orf61	-3.717244455	0.00314651588
ALG9	-1.252130947	0.003147345933
ZKSCAN8	-1.416130327	0.003157362787
ADAM32	-3.173090326	0.003159530003
MFSD9	-1.349341051	0.003162814829
ZC3HC1	-1.198346157	0.003176572458
GINS2	-1.414972173	0.003183069205
LIFR	-2.318622349	0.003191589008
RP11-620J15.1	-5.318300474	0.003237995702
TP53	-1.484196512	0.003245564641
ZNF141	-1.858099581	0.003260757943
AC097662.2	-1.687064586	0.0032674624
CLCN2	-1.577793902	0.00326854886
RBBP4	-1.015227957	0.00326854886
PTOV1-AS1	-1.975556696	0.003281406381
HIVEP3	-2.754662634	0.003286301156
N4BP2	-1.350727574	0.003290573016
LINC00601	-3.655881329	0.003293974386
TMEM51	-2.307598675	0.003295663095
WDR7	-1.052464064	0.003313960837
MGAT5	-1.713395288	0.003319575422
AKT1	-1.277894445	0.003332183403
TUBB2BP1	-4.758732598	0.003346107893
ZBTB7C	-5.577719834	0.003399716967
SPATA22	-5.151983746	0.003400853304
NDFIP2	-1.225079092	0.00340616981
TRAF6	-1.471911578	0.003406754276
C9orf50	-5.277628932	0.003419623425
KLRK1	-6.813386098	0.003438214437
ALAD	-1.247897782	0.003497147531
NUP210	-1.497317033	0.003499844693
FST	-3.116680368	0.003507713563
CMTM1	-2.110482737	0.003530184084
AC079584.3	-5.146737373	0.003569273891
SP2	-1.212603048	0.003572919198
ADD1	-1.324669409	0.003595515826
ITGA2B	-2.983063795	0.003598927211
BRSK1	-1.497702774	0.003610092026
LRRC3B	-5.546849316	0.003628677402
RAB38	-2.408044613	0.00363585349
RP5-1024C24.1	-5.999230278	0.003644599512
ADAMTS9-AS2	-5.996907911	0.003644678278

FAM92A1P1	1.537147414	0.01497220412
RP11-247L20.4	1.297895405	0.01499724708
MIF	1.836275922	0.01502694145
RUSC2	1.17464369	0.01507705015
SBNO2	1.447993006	0.01511098396
CCZ1B	1.009681098	0.01511098396
S100A13	1.392327852	0.01521717001
CHMP5	1.263280854	0.01524579701
GLIS3	2.245227673	0.01531899732
EIF4A2	1.027788198	0.01535765521
GDF9	1.234405124	0.01542850194
KLHL29	1.298807423	0.01544212058
RP11-482G13.1	4.725877673	0.01544297214
NCR3LG1	2.257380401	0.01550717836
PLAUR	2.13184167	0.0155146524
GNA15	3.586551437	0.01558921968
MUT	1.115681414	0.01568411776
OXLD1	1.113127281	0.01570929141
MFSD10	1.631706667	0.01571849455
SARDH	4.458753304	0.01578269765
FAM175A	1.108223786	0.0158683899
TOP1MT	1.130399805	0.01595215506
BCAR3	2.544640885	0.01597247322
XAB2	1.301967349	0.01598622458
WNT4	3.129300295	0.01610828401
FAAP100	1.221033362	0.01613011237
KCNJ14	1.658584444	0.01619642901
AC093732.1	4.611877005	0.01625136653
WASH2P	1.286103579	0.01628826123
OSMR	4.93512791	0.01635436406
FBXL2	1.808411585	0.01647970542
CEP89	1.412626602	0.01650476732
SREBF1	1.511079099	0.01662573772
RECQL4	1.431947871	0.0166434539
FAM114A1	1.219219448	0.0167783071
UCK2	1.034370667	0.01679082456
ULBP1	3.129788652	0.01684234547
ZFPM2-AS1	2.865300646	0.01698869866
MAPKAPK3	1.584800926	0.0170506469
GRK6	1.100039681	0.01705819225
STC1	2.420088322	0.01735882349
KLK1	3.400247202	0.01737274577
FBXO32	1.69479155	0.01739309656
DUSP2	2.573539817	0.01744102166
ELOVL4	1.861673353	0.01781338723
RP11-535M15.2	3.929105412	0.01787343677
NOS3	3.876076561	0.01790888322
C10orf25	2.148915277	0.01794711604
BMP4	1.221114222	0.01794855886
RP3-467K16.4	2.82104963	0.01807675308
TOX2	1.841117966	0.01826355756
AC145343.2	3.750887357	0.01832311228
ALG10	1.193737109	0.01840696425
TTC6	4.235383389	0.0184246866
AC002076.10	3.84241051	0.01842606347
RP11-134G8.7	1.650136783	0.01842740977
HSPA4L	1.167002966	0.0185318009
EVA1B	2.989108463	0.01854690629
HAUS4	1.0448497	0.01855474263
EFR3A	1.351535902	0.01857446034
IMPDH2	1.068117651	0.01861473125
FAM208A	1.166974884	0.0186427278
GLIPR1L2	1.692223458	0.01872135097
ANAPC10	1.022415053	0.01885973881
PSEN2	1.393372932	0.01888964793
CGN	1.255766417	0.01890152822
ATP6V1B1-AS1	3.433189361	0.01890187137
IGF2BP1	4.685445109	0.01894746024

BTK	-4.69252084	0.003654835156
GNB4	-1.994316203	0.003662183227
WFDC1	-4.69535704	0.003674131656
ATP6V1G1	-1.133491421	0.003686156411
PCDHB9	-3.203210781	0.003700407369
HERPUD2	-1.239676053	0.003707479166
CNPY4	-1.663794402	0.003707479166
SEC14L5	-4.376461958	0.003708139987
ZFAND2A	-1.817010382	0.003714018982
BEX2	-5.066748804	0.003720183558
TEAD2	-2.08855701	0.003726359863
CHL1-AS2	-5.65075024	0.003741768487
ASB4	-5.252805382	0.003742443548
ABHD17C	-2.535920025	0.003749303409
TMED10	-1.068832266	0.003776549229
EFCAB2	-1.493264648	0.003777830725
TRIM22	-3.201786811	0.003809392918
NECAB2	-3.695586677	0.003821188309
C3orf62	-1.202992092	0.003822747738
RP11-204L24.2	-3.122698041	0.003824633773
C14orf159	-1.018135535	0.0038468482
TOR1B	-1.205636552	0.0038468482
HECTD4	-1.04526215	0.003863815683
TOP3A	-1.042855151	0.0038682073
EFTUD1P1	-3.234812208	0.003874123523
LINC00506	-5.295590616	0.00387448858
SOD2	-1.265259247	0.003877286368
COLQ	-2.075582557	0.003894387102
AMY2B	-2.211568306	0.003894387102
GUCY1A3	-5.988752041	0.003895413923
BMS1P2	-4.052098732	0.003901674228
ZNF268	-1.958203452	0.003907286282
STAT5A	-3.313644331	0.003923227954
TMOD1	-3.398457435	0.003926137547
SH3BP2	-2.246801789	0.003933020251
GFI1B	-3.727546609	0.003950897647
ZNF91	-2.985322129	0.003950897647
PSKH1	-1.449828295	0.003954404804
NCS1	-1.477361497	0.004010450174
PLLP	-2.169383487	0.004015854903
TBCE	-1.388628023	0.004059057399
SLC25A40	-1.147453659	0.004064043798
NDUFA9	-1.189861271	0.004103782492
RGMA	-3.452668704	0.004103782492
CA12	-2.935371191	0.004112074955
POLDIP3	-1.1845829	0.00411630455
SLC25A34	-2.793612554	0.004122196302
CEMIP	-6.080204997	0.004129390959
DGCR8	-1.271239475	0.004165638026
FSBP	-3.788140782	0.004167144363
RPS27L	-1.163187218	0.004197117081
EIF1AX	-1.34560509	0.004202077406
RP11-429G19.3	-5.180231863	0.004224718689
LHFP	-2.745093962	0.004227378322
ELOVL2-AS1	-5.065880739	0.004229916802
LRRC37A3	-2.799352769	0.004236224549
DDX56	-1.259142347	0.00424171754
ZNF423	-4.874175865	0.00426192226
ACSF2	-1.254972269	0.004279685898
PER3	-1.903743332	0.004294537408
RCBTB1	-1.066634271	0.004303880939
RP11-281O15.4	-5.048132452	0.004303880939
GPBP1	-1.335598319	0.004324841391
GAB2	-1.502713089	0.004325200767
FBXO41	-1.187585919	0.004325200767
IGDCC3	-2.949762438	0.004336012166
MAEL	-5.464500876	0.004338551551
FAM117B	-1.563920686	0.004359959699

FAM86HP	1.751332613	0.01896799307
LDLR	1.241552419	0.01911814173
NDUFV2	1.586314944	0.01918657031
RP11-650K20.3	3.615188719	0.01921205146
LRFN4	1.592714353	0.01921625364
LINC02057	4.12147217	0.01929912714
TMA16	1.194177342	0.01931921015
CCNO	2.057851353	0.01934800273
RP11-677M14.3	3.392028617	0.01952092811
HECA	1.06119965	0.01963206607
THOC1	1.006603965	0.01964208744
PTER	4.609702103	0.01970721479
AC106786.1	4.191157465	0.01977993331
PCLO	2.516770863	0.01989108627
EPHB6	4.067710728	0.01992255906
PCGF5	1.304516557	0.01998900498
SLIT2	2.562782214	0.02002261143
CCDC71L	1.213161228	0.02002261143
PIK3CG	4.336444541	0.020048825
RABEPK	1.007530426	0.02016004222
BCAR1	1.687367185	0.02021182386
CH507-154B10.[illegible]	1.455764363	0.02028470541
CTD-2337A12.1	4.537149604	0.02032954731
TNKS1BP1	1.485608127	0.02040811731
VARS	1.143985836	0.02052514182
EEF2	1.180576562	0.02058990351
WASHC1	1.102188977	0.0207091044
TBX19	1.586289407	0.02071502199
FAM86C1	1.275477784	0.02080754254
LINC02086	2.70406007	0.0208398748
HPRT1	1.491272772	0.02111368115
VASP	1.087737242	0.02118515044
RP3-460G2.2	2.392090466	0.02127348231
GADD45GIP1	1.207094011	0.02148357022
PRPS2	1.300665286	0.02152290263
CEP57L1	1.105833171	0.02166298656
ITGB2-AS1	4.593664851	0.0216808998
RHBDL1	2.500206027	0.02171493037
DUSP10	1.36033127	0.0218259368
SEMA6A-AS1	1.611079106	0.02188255209
UBE2S	1.394583147	0.02194888456
DLGAP2	4.52102113	0.02195686522
MYLK	1.500267615	0.02198623706
NKX6-1	2.950053556	0.02199967924
TMEM267	1.174500718	0.02200779639
RPL21	1.16409132	0.02213664948
AJUBA	1.700124755	0.02217522739
RP11-286H14.4	1.271120736	0.02221796274
RP11-14N7.2	4.478142569	0.02225577315
OR2L13	4.477934074	0.02225733432
ZNF404	3.215989816	0.02230053156
CTC-338M12.4	1.275170231	0.02234600247
RP11-297A16.2	4.19827412	0.02245840739
FAM110D	2.097575172	0.02249236466
RP11-84D1.1	4.552179696	0.02250465587
TTC7A	1.236208095	0.02253544339
LRP5	1.356248105	0.02254183327
PRKCQ-AS1	1.210084497	0.02254512085
NFKBIL1	1.388376684	0.02264509758
COL4A4	2.247917666	0.02269664849
CAMKK1	2.098885569	0.02307761215
SHB	1.408419158	0.02317633592
FAM117A	1.188743276	0.02318523575
AP001372.2	1.140514682	0.02328766813
ERN1	1.04968285	0.02331227598
RNF32	2.667459828	0.02334372343
AC053503.4	2.784715264	0.02356068188
SOCS2-AS1	2.678361743	0.02356068188

ZNF391	-1.385817452	0.004364708063
ADPRH	-5.433206133	0.004368055373
DISP3	-2.169585591	0.004377850978
GPR173	-1.703163255	0.00440903211
ANKRD10-IT1	-1.541373901	0.004425009632
LINC00877	-5.162567193	0.004430997495
BLOC1S2	-1.091436053	0.004435476071
PNMA3	-4.283594923	0.004451324321
ZDHHC3	-1.015206125	0.004460942112
IKBKE	-2.583830452	0.00446594411
LMAN2L	-1.398350486	0.004470000054
TMEM176A	-5.895689635	0.004528325101
LINC00606	-5.915208686	0.004529393032
DACT3-AS1	-4.634377855	0.004559218413
STRIP1	-1.069424467	0.00457864508
IFT81	-1.152316531	0.004582483218
LINC00205	-1.177100916	0.00459041247
PLCB2	-4.620534843	0.0045964445
CUX1	-1.119175674	0.004604822623
AGAP1	-1.972480215	0.004616489308
ARID5B	-2.115980148	0.004640907386
MGP	-5.119008872	0.004658574498
LRIG2	-1.142181047	0.004663445946
EVI5	-1.39108446	0.004666592538
ZNF322	-1.195794908	0.004666592538
TRIM13	-1.082299016	0.004672968424
AFF3	-4.850708475	0.004683221846
ZNRF1	-1.083219305	0.004684760128
RPRD1A	-1.049058634	0.004705397279
PGM5P4	-5.473616854	0.004726158734
ASAP3	-1.798129361	0.00473771552
MGAM	-3.875615188	0.004741665923
IFNAR1	-1.118767274	0.004742904452
TJP1	-1.630312674	0.004748682988
MAPT	-1.08660315	0.004757739311
C6orf89	-1.0134314	0.004815699227
TMEM184C	-1.025054577	0.004817898663
STAG3L3	-1.758547837	0.004817898663
ZNF763	-2.878455955	0.004817898663
TRAK2	-1.842841422	0.004852126212
DNMT3A	-1.269829176	0.004865294804
MRPL57	-1.408108246	0.004880726723
POTEE	-1.632164519	0.004929574104
HHATL	-5.354682809	0.004949183226
MTHFSD	-1.160669355	0.004956092823
KLHDC10	-1.045219994	0.004961398181
GPR12	-5.213071627	0.004963393387
KMT2E	-1.203849336	0.004965091321
SLC39A6	-1.466502006	0.004991306023
SOCS6	-1.404263663	0.005019916908
ZNF84	-1.253175728	0.005026043336
ATP6V0D1	-1.286942125	0.005062311635
EXTL3	-2.476230764	0.005104970887
TAF1A-AS1	-1.711780788	0.005107535178
MMP15	-1.877414676	0.005118873902
APP	-1.88228629	0.005137694534
ASCL1	-6.422209399	0.005145285726
EBF4	-2.348431562	0.00523631933
RP11-758P17.2	-3.714195116	0.00523631933
LINC02001	-1.277770011	0.005245208224
WNK3	-1.224242877	0.005272581938
STRADB	-1.45114737	0.005282551095
SCAMP5	-1.747422611	0.005298695264
ST7	-1.361638941	0.005310336115
RP4-625H18.2	-4.934561949	0.005310336115
EMILIN1	-3.364557113	0.005315343912
DIRAS3	-3.872198124	0.005327635563
SFRP4	-5.279322847	0.005387453413

EPOR	1.607168607	0.02369126955
MIR4500HG	3.170335241	0.02370021912
LIPA	1.489928127	0.02374382983
RB1CC1	1.242945521	0.02377184471
USH1G	3.502674536	0.02387122837
LLGL2	3.468753111	0.02387366844
DMBX1	3.059766211	0.02403996129
PFAS	1.077632119	0.02410976757
RPL29	1.20357207	0.02450292679
ENGASE	1.344762342	0.02455274659
RASL11A	1.871102945	0.02460933712
CTD-2540B15.1(	3.480146378	0.02462087006
SLC19A2	1.446280035	0.02463571257
HELZ2	1.626916829	0.02472205589
GOLGA3	1.029643975	0.02472449783
TCEA1	1.021016919	0.02475288636
TXNRD2	1.460290662	0.02475964209
FAM166B	3.24192301	0.02476612505
RP11-122K13.12	1.829609781	0.02484619539
RBKS	1.814333277	0.02484654259
LARP1B	1.141526501	0.02490957687
HSD17B1	1.85547278	0.02491647428
CASP8	2.371285742	0.02496524068
UGCG	1.291875187	0.02500634755
LTBP4	1.425418433	0.0250517973
RP11-736K20.5	2.270516973	0.02522601544
DDO	2.732498667	0.02524786372
ARAP3	2.200094909	0.02526481134
RP11-423H2.3	2.618659719	0.02526481134
NHP2	1.190595491	0.02529063821
PRORSD1P	2.69412074	0.02543442697
TXNRD1	1.49172468	0.02544450274
RAB1B	1.181542574	0.0254483105
MIOX	4.449188315	0.02557995605
GOLGA2P7	2.857246491	0.02558465875
YJEFN3	1.470108105	0.02561824967
CNBD2	1.056901655	0.025642613
THSD4	3.086262806	0.025642613
REEP5	1.157430091	0.02576280535
RP11-297N6.4	3.098554344	0.02583155998
ST6GALNAC2	2.699145091	0.02589483178
PML	1.021428802	0.02594461184
ADGRE2	2.634192958	0.0259800486
RP13-455A7.1	4.448132981	0.02606312664
AC012358.4	4.444920068	0.02616117794
RP1-266L20.2	4.485001717	0.0262392154
AC116609.2	2.735095742	0.02625603682
RP11-89K11.1	3.443333393	0.02625755707
C4orf50	4.712446567	0.02630366597
GAA	1.240166167	0.0264254815
MOV10L1	4.444118524	0.02649441971
MRPL4	1.306785942	0.02651802535
C1RL	1.429504006	0.02653790596
MRPS35	1.005748141	0.02664116115
DLC1	1.317641176	0.02668564577
EBF2	3.605722265	0.02671588276
E2F8	1.031957423	0.02674096747
FGF17	3.981645443	0.02704254102
RPS18	1.143236991	0.02704254102
UBXN6	1.338209262	0.02742119653
CCDC62	1.469654237	0.02749417207
ADAM15	1.068667253	0.02750839065
KCND1	1.648977835	0.02758436408
SH2D3C	3.584609517	0.02760236855
SLC25A35	1.069666021	0.0276067638
CTC-453G23.8	2.202087822	0.02768265983
DNAJC5G	2.738367277	0.02769752914
SLC29A4P2	4.505728154	0.02788244635

CINP	-1.431986774	0.005409339547
CCT6P3	-1.35492557	0.005435119535
ATE1-AS1	-2.633969664	0.005454328544
MRPS31P5	-1.635780829	0.005466152176
SERPING1	-5.394287071	0.005470351194
RAB11B-AS1	-2.034888011	0.005484735777
RP11-216B9.9	-3.234881605	0.00551751757
SLC44A2	-1.871872122	0.005544398093
SLC4A2	-1.574651094	0.005547422294
RP11-136C24.3	-5.298529017	0.005547422294
SPATA42	-5.071871399	0.005567303863
GPR153	-1.727413411	0.005579646203
NCKAP5L	-1.303268217	0.005617076307
BTN2A3P	-1.727781991	0.005643170577
RBMS3-AS3	-2.808902002	0.005653211982
LTK	-3.388892373	0.005681758137
GNG3	-5.027208951	0.005684921355
ZNF561	-1.101989568	0.005696688325
TMSB15A	-1.702155627	0.005715438568
PTGER4P2-CDK	-2.953415243	0.005780874326
ZNF580	-1.214847721	0.00581547482
RP11-620J15.4	-4.943769079	0.00581547482
CXXC5	-2.221080117	0.005823144848
YBEY	-1.207543828	0.005829622156
PKD1L1	-2.745934646	0.005834575571
C11orf49	-1.685126938	0.005878006534
DZANK1	-1.213273265	0.005928809403
ARHGDIG	-5.322135219	0.005944883797
CIART	-1.872670573	0.005952085852
PTGFRN	-3.442098665	0.005964505308
TMX1	-1.259130896	0.005964505308
KIAA1755	-3.416720388	0.00599194589
ITGB1BP2	-1.65738193	0.006026702556
SNCAIP	-2.928106546	0.006030695839
CENPF	-1.33805948	0.006030695839
C6orf141	-5.129777171	0.006036570855
GTF2IRD1	-1.792596473	0.006063890623
TMEM216	-3.434415906	0.006073305984
GRK5	-1.485800323	0.006098625766
AASS	-1.460635917	0.006107761749
LINC01977	-5.32261339	0.006111137619
NEDD4L	-1.248186939	0.006136482573
EBF1	-3.85546155	0.006140889725
SDC3	-2.259838459	0.006150186689
RP11-241F15.1	-4.700815893	0.006161049313
CLSPN	-1.495364896	0.006189936577
RP11-110I1.12	-2.361724833	0.006242885529
HARBI1	-1.136532334	0.006268883221
GRIA1	-4.638861346	0.006283049894
DICER1	-1.036318558	0.006300467461
ZEB2-AS1	-4.099506045	0.006310215433
ZMYND11	-1.033284575	0.006310351727
RNF41	-1.054053974	0.006322627221
LRRC37B	-1.151313638	0.006355494958
RP4-635E18.8	-2.277617836	0.006381108126
LINC01778	-2.722293474	0.006404109579
KLRC2	-6.228398516	0.006410093941
CCT6B	-2.03058941	0.006428037019
ALOX12P2	-5.095230955	0.006447121808
GDF7	-5.293838915	0.00645975598
OAZ2	-1.187357799	0.006499035546
KIF21A	-1.865969966	0.00651053706
C6orf62	-1.127655932	0.006545012497
GPR135	-1.161234495	0.006565965774
CACNA1D	-4.457495342	0.006569160096
JMJD1C	-1.082624334	0.006579413059
NIFK-AS1	-1.50979	0.006581792306
PLXNA2	-3.707638384	0.006618121071

IL27RA	1.972693484	0.02801953559
HSP90AB2P	1.286271622	0.02809119485
ELFN1	2.717904802	0.02817111397
CTB-131B5.5	1.750013657	0.02837920971
FRA10AC1	1.122346196	0.02849619811
TLR5	2.220447221	0.02860894651
RP11-77P6.2	1.167662649	0.02866036952
RP11-235E17.6	2.055833607	0.02879479414
CNBD1	2.968543564	0.02881649906
ST20	1.093934916	0.02882289017
C11orf70	1.875325323	0.02886202535
MKNK2	1.110012591	0.02886266936
FAM53A	2.620544423	0.02886469305
JAG2	1.877050586	0.02900291738
EBLN3P	1.252644577	0.02907481304
RPL13P5	1.432700187	0.0292484033
TMLHE-AS1	3.187322844	0.02935365836
NDST3	4.420464372	0.02943598537
RP11-402G3.3	4.417007063	0.02959428294
APBA3	1.104362129	0.02964744196
CDC42BPG	3.249485587	0.02964744196
MFSD13A	1.287378137	0.02965218063
SNHG12	1.162502945	0.0297142304
RP11-54C4.3	1.722094744	0.02973853888
TMEM145	1.089310047	0.03012363356
DUS1L	1.350888534	0.03015202888
GATA6-AS1	3.995721576	0.03019054622
MSLNL	4.414125529	0.03030087138
RP3-467K16.7	4.411508278	0.03040004781
DPP7	1.603242981	0.03045632509
TUT1	1.072338265	0.03057907622
TMEM9B-AS1	1.529967684	0.03058264342
TCTE1	3.942206796	0.03062300989
NAT6	1.053434812	0.03062300989
STARD4-AS1	1.96123643	0.03065540496
NACAP1	1.034900651	0.0307217102
SLC2A4RG	1.213604184	0.03077495083
ARL2	1.333342336	0.03101619364
RP11-501C14.5	4.024117001	0.03129581396
CRYM-AS1	2.390710586	0.03146325358
CAMTA2	1.011549198	0.03148317724
SLC29A2	2.729380933	0.03156376466
MCMDC2	3.536732217	0.03159987824
CYP39A1	2.849863087	0.03162380654
POLB	1.710448332	0.03165134489
ADAMTS12	3.197889811	0.03165134489
GPAT3	3.317589671	0.03169334889
HERC2P10	2.652305393	0.03175654797
TSPAN4	1.47852995	0.03182322762
RP11-239A17.1	4.321337041	0.0318658036
FOXC2-AS1	4.53987788	0.0319408992
OXTR	1.969510352	0.03204349082
PIEZO1	1.152245111	0.03217150924
WASH5P	1.55629634	0.032183519
SLC16A6	1.889646526	0.03223780035
UNC5A	2.900876876	0.03231884527
BCRP7	4.434811519	0.03239631346
BCL2L12	1.036374197	0.03262397763
LYSMD3	1.146260471	0.03265971689
MCRIP2	1.157305055	0.03284819752
IRAK1	1.029966	0.03286665052
RASD2	2.249821986	0.03296000835
UBE2W	1.104073456	0.03299355355
WFDC21P	4.438280554	0.03299355355
LINC02210	1.019602373	0.03303933055
ERGIC2	1.046840657	0.03304121069
OTUD6B-AS1	1.051572853	0.03304121069
AP001258.4	1.046833372	0.03309785096
PODN	-5.717730119	0.006634694516
LINC01089	-1.256446448	0.006649216337
PCDHA3	-5.707672322	0.006655107343
RAP2A	-2.388459348	0.006668303842
FIGN	-1.310633238	0.006685984479
RERE	-1.189308685	0.006706069916
SLC25A33	-1.04901577	0.006754455841
AGK	-1.222104795	0.006760970495
FKBP14	-1.343572894	0.006764061764
MYO18A	-1.18297083	0.006777988076
CDK5R1	-1.701945974	0.006852079505
OLFML2A	-3.143219955	0.006855833043
CNIH3	-1.982384192	0.00687410851
GLS	-1.265784768	0.006881038643
SELENOV	-5.256844569	0.006881038643
CHD7	-1.219221779	0.006911578283
FERMT3	-2.510695406	0.006921858627
C2orf66	-4.315048385	0.006934827397
TMBIM4	-1.036313056	0.006941580647
C1QTNF1	-2.224858531	0.006969056909
DGUOK	-1.026493349	0.006999614181
AC091133.1	-1.942806565	0.007032716316
CNTLN	-1.707947443	0.00709396116
FAM110B	-1.90209877	0.007127110212
TMEM80	-1.310460343	0.007157746281
NOP14-AS1	-1.292889805	0.0071673344
ZNF841	-1.641010051	0.007209203249
TRIM67	-4.97399557	0.007222763671
ATG2B	-2.122035986	0.007314229283
MINDY3	-1.208613357	0.007328234251
GABPB1-AS1	-1.12794287	0.007344285083
ZNF518A	-1.091974294	0.007385267707
SDK2	-4.777173557	0.007422900564
PCDHGB2	-3.160451764	0.007422900564
OLFM1	-6.227339927	0.007442543374
DISP1	-1.790612397	0.007464342372
LINC01816	-4.92710743	0.00747520279
ABCC9	-5.719689475	0.007479423969
RHOBTB1	-1.365892438	0.007487304493
RP11-282O18.3	-1.395987859	0.007566092326
DPY19L1	-1.162918818	0.007671262437
UCP2	-1.582867376	0.007671262437
ETV5	-1.430772337	0.00768203319
DUSP15	-1.945630796	0.007689829314
MFSD5	-1.491431773	0.007689829314
AC004893.11	-1.505077532	0.00774465452
TFDP1	-1.671615367	0.007794169846
ANKRD45	-3.236919385	0.007803202587
MUTYH	-1.087860648	0.007820749729
SLC6A16	-2.079988901	0.007836097101
GTF3C6	-2.08547215	0.007846086901
ARL4A	-1.808872899	0.007965219414
NR2E1	-4.998425318	0.008078625944
CHRNA4	-5.67091649	0.008107543827
RP11-277P12.9	-4.971186188	0.008118670935
SLC30A1	-1.043399911	0.008135180032
BCL2L15	-3.02854094	0.008137511493
POR	-1.608275558	0.008145464157
CASP3	-2.007195593	0.008183761794
LINC01833	-5.271226429	0.008201056469
THAP5	-1.078117063	0.008204994159
ERVK13-1	-1.346821894	0.008245782331
BMPR1A	-1.449981754	0.008262197588
KIAA1549	-1.228877654	0.00827401152
CDK6	-2.460414467	0.008276424262
TYW5	-1.089644667	0.008303749361
COMMD9	-1.216817124	0.008340099615
DNAJB2	-1.039933749	0.008352247292

ARHGAP22	1.852843606	0.033131005	BTD	-1.109892061	0.008352247292
RP11-95P2.3	4.311069376	0.033131005	ACTN2	-3.088266622	0.008359029552
FBXL17	1.251150204	0.03314059298	RAPGEF5	-2.78974019	0.008447942644
ABC7-42389800	1.064830219	0.0333193869	RP11-472N13.2	-4.464710927	0.008450219104
RP11-497E19.1	4.428545803	0.03337487315	GTF2IP13	-1.396123789	0.008526508601
RP5-901A4.1	1.916525277	0.03360784306	GPR158-AS1	-4.848467894	0.008553432204
PICK1	2.541143568	0.03361843736	IGFBP4	-2.982805565	0.008596466917
CRLF3	1.162553519	0.03363422176	AC009506.1	-1.829064139	0.008609605431
FBXO24	1.407262694	0.03379967997	CTC-429P9.5	-1.447075865	0.008652922341
SP140	2.069142568	0.03385740527	RORA	-3.129045506	0.008655684644
SNX29P1	4.040861205	0.03386266392	CTD-2116N20.1	-2.493858948	0.008759872539
LRGUK	2.291963264	0.03390716001	AC003991.3	-4.831684984	0.008761441907
ZMYND15	2.453982143	0.03391007681	STAR	-5.182937317	0.008793743218
THOC6	1.169586913	0.0339520237	PCDHB15	-2.388931659	0.008840830186
PSMD6-AS2	1.072555648	0.03431800531	S100A4	-2.87993749	0.00886444768
RP11-351I24.1	2.562296038	0.03437842433	TNFRSF19	-3.867091943	0.008924069337
ZNF569	1.016084274	0.03452300258	TYRO3	-1.264610557	0.00895432122
CDC26	1.003115016	0.03457984822	DMRTA2	-4.828751997	0.009035238128
DDX54	1.052762139	0.03464229929	DIXDC1	-1.616748419	0.009042714686
ESYT3	2.023447446	0.0346486406	ATP2A2	-1.061903328	0.009085993475
MAGEB3	4.302391807	0.03473447582	LYPD1	-3.739508984	0.009102181056
NOA1	1.181236046	0.03473473425	MTMR10	-1.242104465	0.009145037318
SDF2L1	1.509234598	0.03507454094	RP11-318C24.2	-4.828048624	0.009147507666
ALKBH7	1.708166414	0.03513903195	AGBL3	-1.061288194	0.009173829677
PEX3	1.101002375	0.03515749002	TIAM1	-2.07132173	0.00924211985
RP11-888D10.3	2.41165358	0.03533741854	TMX3	-1.238228024	0.00926465335
RP11-366F6.2	4.34276227	0.03550164201	KLHL24	-1.345531734	0.009388624109
RUVBL2	1.281350723	0.03551359427	OS9	-1.120775826	0.00939927667
RCCD1	1.821092189	0.03554808692	MBD1	-1.128847187	0.009399627428
BMS1P10	2.802872297	0.03567976326	PHF12	-1.088155462	0.009408449871
ANKRD37	1.659281687	0.0357312581	AC005220.3	-5.551988115	0.009426543604
SELENOH	1.470682877	0.03583679679	VPS33A	-1.057034115	0.009462820097
RP11-1228E12.1	1.830938095	0.03593843095	RNF128	-5.182990908	0.009488958547
RAB3IL1	1.574331572	0.03605493733	SNX1	-2.163906944	0.009498479923
TRAM2	1.155605364	0.03608807348	CDKAL1	-1.816706617	0.009498479923
TK1	1.326923442	0.03611814988	LHX2	-1.135520715	0.0095553037
IGSF8	1.164869832	0.03614910135	LPAR1	-1.49994187	0.00956113669
HERC2P3	1.978943424	0.03620001818	SFMBT2	-1.187935395	0.00956113669
ROPN1B	4.105995045	0.03620890119	PDIA4	-1.158319336	0.009565871393
PUS1	1.23610431	0.03622741263	L3HYPDH	-1.752081738	0.009658583907
CHIC1	1.314888946	0.0362703262	MAP1LC3B	-1.460463461	0.00966229218
RP13-895J2.3	3.993426306	0.03644455666	PRRT3	-1.848819964	0.009683793108
GSTO1	1.028699177	0.03657556116	ARHGAP35	-1.16773525	0.009683864136
TMEM190	4.368570096	0.03700932462	C21orf58	-1.187242538	0.009693058467
BFSP1	1.947666971	0.03723757524	KCNJ15	-5.525751705	0.009794806531
PTK6	3.41126913	0.03731249048	LIPG	-5.093382783	0.00980149363
RIBC2	2.018656952	0.03749598744	GABRB3	-5.54822997	0.009810522744
RTN4R	2.274178385	0.03751848322	ABHD2	-1.24510971	0.009834703337
RP11-406H4.1	4.286387948	0.03756156167	LINC01578	-1.334542649	0.009873474105
CTD-2341M24.1	1.475568801	0.03769661099	ITGA9	-4.922385084	0.009885444252
TKT	1.141137656	0.0377038072	SLC26A1	-4.844346393	0.009926816565
ZNF318	1.100741946	0.03784257957	CPSF4	-1.13349966	0.009927863387
CCNE1	1.359604972	0.03822066458	SATB2-AS1	-2.516153774	0.009929319141
AC092168.2	3.869228131	0.03823486444	SVILP1	-2.605414226	0.00997183135
GMDS	1.60585824	0.03824853784	GABRA2	-4.956357453	0.01002012745
F2RL1	3.143916323	0.03850898767	CTD-2506P8.6	-2.007486644	0.0100673699
ADAT2	1.5829078	0.03850898767	RP11-259K15.2	-5.018307458	0.01012902648
PPP2R5D	1.029706322	0.03857188783	NAXD	-1.193327903	0.01013055487
SCOC-AS1	1.415130662	0.0385734574	SETMAR	-1.224699386	0.01022691535
RP11-551L14.4	2.068696538	0.03878982026	RP11-495P10.1	-2.427570824	0.01023459117
C19orf33	4.23279734	0.03881257332	NXPH2	-5.502533381	0.01027794582
HLA-V	3.181607782	0.03884900345	GOLM1	-1.853170757	0.01033799195
CSF3	4.273837827	0.03888023559	UBE2N	-1.010149092	0.01037461648
TMEM161A	1.158196105	0.03903221258	STAT3	-1.406207472	0.01052727282
GMIP	1.243604304	0.03907621375	LMOD1	-4.857411798	0.01057241416
VEPH1	2.564739522	0.03915655096	AFAP1L1	-1.667286633	0.01068536734
ARL13A	4.270617367	0.03934339155	PDCD4-AS1	-1.479755948	0.01069589917
LINP1	3.012624307	0.03934463347	LSP1	-3.952276757	0.01073264238

**AP000442.1**	1.819045455	0.03934463347
**KIF7**	1.737705301	0.03934937729
**SIDT1**	3.841068065	0.03937217257
**ERICH6-AS1**	1.601404055	0.03938598822
**CSMD3**	2.749939768	0.03941735517
**RP11-44N22.3**	2.451911166	0.03951878733
**METRNL**	2.174270526	0.03955328855
**MED15P9**	4.418146045	0.03958973964
**BOP1**	1.261122679	0.03964881747
**LYAR**	1.550537407	0.03966314585
**CATSPER2P1**	1.263821367	0.03966314585
**C3**	2.880806208	0.03979115124
**CTC-529P8.1**	2.519627515	0.03979660975
**RP13-349O20.2**	1.41595205	0.04007012692
**STAT4**	2.806143704	0.04009804403
**PCDHGA7**	1.518863482	0.04014914625
**SPSB2**	1.166286291	0.04030232701
**STRCP1**	2.900765237	0.04030232701
**SLC25A19**	1.289248701	0.04036255058
**RP11-54O7.18**	4.304917175	0.0404314925
**RP11-380G5.2**	3.144009804	0.04066267001
**RP11-438D8.2**	4.218401032	0.04088791089
**RP11-66N24.3**	1.113943438	0.04105688984
**CTD-2008P7.6**	2.71907958	0.04123771566
**RP11-150C16.1**	3.321742726	0.04124693278
**RP4-607I7.1**	2.803939029	0.04132568467
**IFI6**	1.495670824	0.04135770208
**RP11-108M9.3**	4.217755779	0.04135778809
**KIFC2**	1.17950932	0.04139550145
**EREG**	4.297083774	0.04148729096
**SLC25A22**	1.073915209	0.04186119758
**AC012456.4**	3.658464197	0.04192439519
**ZNF526**	1.073495395	0.04207796074
**RP11-350J20.5**	3.028697357	0.04207796074
**DGAT1**	1.291767371	0.04227784969
**ARMC2**	1.260488581	0.04237059544
**PRDM16**	3.202462642	0.0424175539
**TMEM191A**	3.315083554	0.04242712125
**ZNF23**	1.318167936	0.04256980227
**RP11-525A16.4**	3.533212305	0.04257278328
**DNAJB13**	2.833472192	0.04280364845
**AC091878.1**	4.248277103	0.04292114517
**MORC4**	1.467772928	0.04302988467
**BCL6B**	2.879423633	0.04314804473
**MAP3K15**	1.418234632	0.04320500181
**ELMOD1**	2.511119716	0.04320818754
**IL12A**	1.072305826	0.04320818754
**ZBED8**	1.054307621	0.04320955143
**GMNC**	4.393328017	0.04337788541
**FGF7P8**	4.364164945	0.04337788541
**LINC01376**	1.365574836	0.04341651479
**RTBDN**	2.219928515	0.04343269985
**MYL2**	4.391155455	0.04344809764
**PYY**	3.33794033	0.04344809764
**AIFM2**	1.225566695	0.04355187067
**UVRAG**	1.205152265	0.0437845838
**ULBP3**	1.203934904	0.04383406315
**DNAJC12**	1.142199416	0.04436566611
**DCST2**	1.596935293	0.04437787618
**WDR3**	1.018149225	0.04440133557
**CTD-2616J11.16**	3.779180972	0.04453057161
**OAS3**	1.24788139	0.04457426128
**RP11-666A20.4**	4.199150077	0.04500514622
**DRC3**	1.56777941	0.04502153531
**WDR18**	1.350059104	0.04508854596
**POLD1**	1.223461571	0.04540326691
**LMNA**	1.117541231	0.0454063878
**LINC00466**	2.60243764	0.04580605729

**SLC38A7**	-1.253544202	0.01074385483
**PLEK**	-4.941306972	0.01080182827
**RP11-131L12.2**	-4.429400759	0.01081304619
**CUBN**	-2.516740013	0.0108455522
**POLR2J4**	-1.531158764	0.01085000352
**EGR3**	-3.21776498	0.01086501954
**MAP4K4**	-1.042399508	0.01087233698
**SKINT1L**	-4.598385595	0.01088255378
**SCRN1**	-1.933484303	0.01090030194
**PCDHGA10**	-3.261688889	0.01095545531
**SNAP25**	-3.574188949	0.01099160421
**TMEM130**	-4.634888698	0.01099160421
**SRR**	-1.264081587	0.01100692949
**NEO1**	-1.368746629	0.0110537643
**BRMS1L**	-1.229905563	0.01106363478
**CLDN4**	-4.465142947	0.01106725497
**RBM26-AS1**	-1.372105486	0.01108391598
**AGFG2**	-1.337603735	0.01110980512
**INPP5D**	-4.72493494	0.01120034087
**UPF3AP2**	-1.011411975	0.0112120212
**CTSK**	-2.152043768	0.01124353895
**TRPV4**	-4.541485172	0.01126003085
**RIMKLA**	-1.557459297	0.01131328688
**LINC01727**	-5.094783739	0.01133708271
**VWA5A**	-3.888056191	0.01135056792
**CTSO**	-2.034259644	0.01135572716
**TEX261**	-1.164053081	0.0113687258
**IGSF5**	-4.859755467	0.01142943634
**AC009403.2**	-1.503408273	0.01143030468
**SMAD2**	-1.047772627	0.01145145868
**HMGB1**	-1.053785111	0.01154972144
**TMEM2**	-2.390735082	0.0115684589
**ZNF24**	-1.11489381	0.01159288801
**RP11-46H11.12**	-4.730923494	0.01160296881
**LINC02104**	-4.951757049	0.01162481901
**L3MBTL2**	-1.25829594	0.01163297633
**IP6K1**	-1.219627994	0.01163297633
**WDR86-AS1**	-5.442248239	0.01163761728
**STARD13**	-2.333491404	0.01164344034
**NLGN2**	-1.133591966	0.01166482751
**ZNF571-AS1**	-4.974764195	0.01170403198
**BTBD6**	-1.59387741	0.0117398187
**ZNF138**	-1.236745911	0.0117410892
**AC005519.4**	-1.765263623	0.01181083695
**RABGAP1**	-1.418405523	0.01182730268
**RP11-456K23.1**	-5.421627125	0.0118534843
**ZMYND8**	-1.458418308	0.01187922224
**GTF2IRD2P1**	-4.502661551	0.01188075219
**ZMPSTE24**	-1.12253204	0.01202078226
**COLCA2**	-4.715198645	0.01207939496
**ATF7IP**	-1.117082972	0.01210692174
**C21orf33**	-1.673534053	0.01211841854
**C18orf32**	-2.032558263	0.01213050375
**LHFPL4**	-1.457515424	0.01215705758
**MYOZ3**	-4.137125699	0.01217059243
**CCDC140**	-5.103303613	0.01218791759
**HLF**	-1.867360276	0.0121972216
**CD34**	-5.415531733	0.01219909409
**PCSK5**	-3.568429859	0.01222787732
**COX5BP6**	-2.155563195	0.01228379554
**OBSL1**	-1.403701046	0.01240023565
**CTC-498J12.1**	-4.715600867	0.01241394295
**RTN1**	-3.752368536	0.01243144008
**NBEA**	-1.674659534	0.01243144008
**GDF6**	-5.419686649	0.01249898506
**CHRNA9**	-4.98906756	0.01255874903
**MAOB**	-5.532764789	0.012634349
**EPN2**	-1.058710327	0.01264815875

PYGB	1.101456114	0.04598559352
ZEB1-AS1	1.400077752	0.0460241598
RP11-799D4.4	2.484736958	0.04605572258
LINC01232	1.268280286	0.04613359129
MROH1	1.215027084	0.0461733871
RP11-763B22.4	4.23049027	0.04623083657
CCDC169	1.55615072	0.04636223328
TMEM261	1.0554852	0.04642602013
RPS6	1.004693928	0.04651237249
CTD-2616J11.2	2.356376529	0.0466112716
RP11-360O19.4	4.227441386	0.04688807227
RP11-429J17.7	3.84532819	0.04698622317
ABCG4	2.265925842	0.04709894339
SHROOM1	1.872767263	0.04734839073
RP3-465N24.5	1.668474974	0.04751939906
RP11-498P14.5	2.081135395	0.04765588714
AC007879.3	2.990029833	0.04801605006
RP11-473M10.3	4.224174443	0.04813156201
CC2D1A	1.005138503	0.04821400833
SYNE3	2.357888273	0.04832024572
RP11-321L2.1	3.216843551	0.04848793487
RP11-526D8.7	4.217815787	0.04853946633
RP1-179N16.6	1.310811807	0.04867548494
CFAP54	2.730732716	0.04892529335
HSPA1A	4.162493763	0.04910030017
CFAP45	2.1429671	0.04910570688
CTD-2334D19.1	4.331422054	0.04916671913
SMKR1	1.684159571	0.0493066603
RP11-89F3.2	3.75747999	0.04956146826
TNFRSF12A	2.04597705	0.04984552332
KLF4	1.96858706	0.04985425972
RP11-300E4.2	4.126787633	0.04989447671
HECW1	1.639764714	0.04991663415
TRPM6	3.347037706	0.04997195198

SERPINH1	-2.201737213	0.01265483939
SLC12A9	-1.420691004	0.01266671626
FIS1	-1.122048494	0.01266671626
PDIA2	-4.90016734	0.01267762998
TIGD4	-1.361237215	0.0127164305
ETV6	-1.779954798	0.01273246732
ACTR1A	-1.068640004	0.01276985639
KRBA1	-1.121816959	0.0127717863
PCDH1	-2.73880449	0.01279173593
INHBB	-3.97827568	0.01282362396
HACD3	-1.023127296	0.01285832088
ACTR3C	-5.066091268	0.01289228634
HCG22	-5.376293427	0.01290859019
TCN2	-2.373127321	0.01292083837
KANSL1L	-1.09224532	0.01299767714
CIDECP	-1.25557719	0.01300758988
STRIP2	-1.923999899	0.01302249865
ZNF784	-1.537091366	0.01304528106
TLE4	-1.632946777	0.0130706618
NRM	-1.41478861	0.01307985467
NIPAL4	-4.815582117	0.01311051218
LINC01505	-3.330138445	0.01312200277
GSC	-5.372186312	0.01312203969
PDCD6IPP2	-1.590166055	0.01312203969
MYO5B	-2.989582383	0.01316241238
LEF1-AS1	-4.864527245	0.01317131543
GS1-259H13.2	-3.156508562	0.01318921898
ARID5A	-1.517718623	0.01321606205
RP11-496I9.1	-2.981307848	0.01322938674
FNIP2	-1.346893651	0.01328457477
ATP6V0E1	-1.066929508	0.01336824616
PAQR4	-1.343392509	0.0134282859
ZNF836	-1.578500416	0.01345613209
DNAJA4	-5.125224956	0.01347063473
CHST6	-1.909169059	0.0134862283
ISCA1	-1.040601339	0.01364840762
LINC01132	-4.971933474	0.01375764852
VPS37D	-2.311506937	0.01382833228
RP11-50D16.4	-4.651213362	0.01382833228
CNPY2	-1.45389995	0.01383247188
RP11-848P1.5	-2.669653108	0.01383247188
CCDC160	-4.087525217	0.01385019035
AKR7A2	-1.29363271	0.01386987368
TUBB2A	-1.597020733	0.01388183374
CRYBB1	-5.455885259	0.01392924946
MAP7D1	-1.084772101	0.01396760647
COPG2	-1.081491684	0.01397250807
TSPAN31	-1.16005719	0.01400256042
ANKLE1	-2.4666407	0.01401653329
RASL10A	-4.645904155	0.01412309898
CYP4F25P	-2.5169652	0.01420853731
SEPT2	-1.010013484	0.0142321712
GSTM1	-4.612987802	0.01432514328
NPIPA1	-1.074379241	0.01444495472
HN1L	-1.375791573	0.01450753403
LINC00648	-4.645172685	0.01450753403
CDH3	-3.769199448	0.01462086309
GPER1	-3.245480098	0.01471688999
PARD3B	-1.350809921	0.01473880184
AP000473.5	-3.704294737	0.01474654732
AC027612.4	-5.315763392	0.01476901052
DBNDD1	-1.435023233	0.01502694145
LMCD1-AS1	-2.605152147	0.01511098396
MROH8	-1.737143816	0.01520324938
CUL4A	-1.105717857	0.0154294165
USP54	-2.161500499	0.01553958042
ZNF480	-1.319612695	0.01558938453
XRCC2	-1.080519715	0.01565827956

**MEG9**	-5.272678764	0.01572805843
**ARHGEF26**	-1.52033562	0.01573856063
**CPA2**	-5.294565689	0.01573856063
**MYH7**	-4.862240757	0.01586121556
**FMC1**	-2.232695125	0.01590144024
**ZNF180**	-1.11388579	0.01595483866
**UNC13A**	-3.218726903	0.01598231002
**UCP3**	-1.752307867	0.01598900147
**SELENON**	-1.258213644	0.0159939912
**ZMYM4**	-1.073665877	0.0160645195
**TOMM5**	-1.152875408	0.01608499587
**AE000661.37**	-5.261994157	0.01613549692
**BMP1**	-1.898669967	0.01614389603
**THSD7A**	-3.652561692	0.0161488789
**WARS**	-1.0592577	0.01615281415
**SIDT2**	-1.229256527	0.01615281415
**LOH12CR2**	-1.67691758	0.01636598428
**GRM2**	-4.427570348	0.01644954715
**PPM1L**	-1.03094081	0.01649610118
**ADCK1**	-1.507689762	0.01649658618
**SMIM7**	-1.089195785	0.01656341556
**GLIPR2**	-1.635939354	0.01660478679
**RP11-27I1.4**	-1.559549043	0.01667587774
**MCUR1**	-1.048329386	0.01671446453
**CIZ1**	-1.126964478	0.01685817668
**C6orf223**	-2.803776968	0.01688864763
**AC010883.5**	-2.094717492	0.01690504352
**LANCL3**	-3.676289883	0.01716366508
**SWAP70**	-1.00314323	0.01722553773
**RP11-796E2.4**	-2.760083635	0.01723144724
**PPIA**	-1.182175414	0.0173059176
**JAM2**	-3.00487969	0.01743735981
**LINC01410**	-1.678064504	0.0174400882
**RPP40**	-1.061085731	0.01746395472
**TSPAN5**	-2.215451125	0.01755012935
**FKBP9P1**	-2.786653961	0.01763436275
**PPP1R12B**	-1.091803732	0.01764074781
**TMEM132C**	-5.280471444	0.01764074781
**TAX1BP1**	-1.020367875	0.01765393428
**PROCA1**	-1.879371455	0.01774208688
**RP11-428J1.4**	-2.3268122	0.01789964346
**KAT7**	-1.112235302	0.01793708259
**PAXBP1-AS1**	-1.862069318	0.01803800624
**DBX2**	-4.985128784	0.01803947302
**FGF1**	-2.194756496	0.01809873022
**ZADH2**	-1.010574957	0.01814637299
**RP11-627G23.1**	-4.609350113	0.01814637299
**RSU1P2**	-5.225366186	0.01818090749
**RP11-257P3.3**	-4.876959069	0.01823445458
**LINC01085**	-3.70779577	0.01823959845
**FBLN5**	-4.702085663	0.01828660283
**PTCH2**	-2.74398405	0.01836790408
**HORMAD2**	-5.188698527	0.01839180083
**POLR2J**	-1.083909056	0.01842346686
**SLC26A10**	-1.571036973	0.01842346686
**TRIM62**	-1.30990185	0.01847844345
**CCDC175**	-5.185300482	0.01848685316
**ATP8A2**	-4.291460198	0.01851106605
**SEC14L2**	-1.335052376	0.0185284686
**TENM3**	-3.492901526	0.01855474263
**SERF1B**	-2.038929624	0.01857871248
**RP11-274B21.13**	-1.036207664	0.01858357895
**FAP**	-4.835194214	0.01863198518
**ZFYVE16**	-1.625023493	0.01864231613
**ADCY6**	-1.377884627	0.01864612585
**TEF**	-1.438246803	0.0186734286
**CLK1**	-1.121236214	0.01885873773
**RBP1**	-4.740776138	0.01886058615

**TPP1**	-1.118401138	0.01887313444
**ADAMTS7**	-2.2195974	0.01896799307
**TCTN2**	-1.430464998	0.01907818385
**SERINC5**	-1.141579865	0.01919392089
**FMN1**	-2.440974978	0.01923363864
**AC108488.4**	-1.73751869	0.01934800273
**RECK**	-1.095674901	0.01938349282
**LIMA1**	-1.110009956	0.01949806652
**PITHD1**	-1.049148389	0.01949968909
**RABL2A**	-1.062556597	0.01956697924
**KLC1**	-1.135311744	0.01962575376
**RP11-351J23.1**	-4.495981675	0.0196358903
**NMRAL2P**	-3.568712178	0.01965706005
**GIPC3**	-4.757185092	0.01970721479
**NAGLU**	-1.421840041	0.0197509011
**UBA7**	-1.808348574	0.01975944203
**RTP4**	-3.567992139	0.01977993331
**CYSLTR2**	-5.180562209	0.01977993331
**RP11-54D18.2**	-2.897950156	0.0198209433
**SMARCB1**	-1.03897791	0.01982550254
**FBXO39**	-5.166830435	0.02001968978
**ZNF596**	-1.980658695	0.02007730268
**WWP2**	-1.97774029	0.02010486098
**LINC01549**	-4.572397451	0.02015732158
**NAPA**	-1.170732703	0.02016004222
**MFSD14C**	-1.388260464	0.02016884735
**ZDHHC17**	-1.032897555	0.02019774548
**SMDT1**	-1.227006232	0.02028639716
**LINC01503**	-3.739534837	0.02029372027
**MYSM1**	-1.008475942	0.0203993544
**ARHGEF40**	-1.48529973	0.02048618293
**TRAC**	-5.207150108	0.02051717342
**AC074391.1**	-3.969413731	0.02052388547
**HIST1H3D**	-3.066695655	0.02062658511
**SCN2B**	-4.612587548	0.02067453567
**RP11-263K19.4**	-2.442177897	0.02069675234
**CHST14**	-1.362274446	0.02076114267
**LINC00856**	-3.714459878	0.02076114267
**MMD2**	-4.703942691	0.02095120897
**PBLD**	-1.511055991	0.0210456504
**CMYA5**	-2.199542165	0.0211731849
**NFE2L1**	-1.284855613	0.02143014669
**EGFEM1P**	-1.60304294	0.02143829914
**EPHB4**	-1.451703659	0.02152335606
**RP11-1055B8.4**	-1.750344036	0.02152849722
**ZSCAN23**	-1.278905726	0.02154150772
**ZIM2-AS1**	-5.728487225	0.02157254241
**KCNAB1**	-4.634241237	0.02159080678
**ELAVL2**	-2.680505056	0.02170893029
**DUSP4**	-2.846789594	0.02173576314
**SEPT7P3**	-2.286634191	0.02175672944
**TNN**	-3.052157608	0.02187928191
**CEP85L**	-1.700958305	0.02196281938
**FLOT2**	-1.291724195	0.02199967924
**APBB1**	-1.097763018	0.02214553784
**AC006026.13**	-4.479051824	0.02214956939
**SLC6A11**	-4.73300233	0.0222210514
**C20orf196**	-1.653755861	0.02229771785
**NRXN1**	-4.878613041	0.02236941137
**AC147651.4**	-2.346840133	0.02236941137
**IQCA1L**	-3.961663442	0.02236941137
**TIMP2**	-1.605633935	0.02246955709
**RASA4**	-2.23875188	0.02251500341
**GFRA3**	-4.716226762	0.02251632639
**LCA5L**	-1.622065567	0.02252697764
**KIF9-AS1**	-2.115428858	0.02255149604
**LINC01579**	-5.63619742	0.02270412626
**HIST1H4H**	-2.053581368	0.02296145971

**ANKRD9**	-1.609927333	0.02298963557
**SPIN3**	-1.02601748	0.02299982084
**CTC-471J1.2**	-2.610533659	0.02304810569
**SEZ6L**	-5.600237477	0.02307067542
**ZBTB1**	-1.036029902	0.02315678997
**AC013460.1**	-3.941300567	0.02315678997
**DNM1**	-1.940673073	0.02316430587
**COQ4**	-1.166664683	0.02321509016
**MEIS3**	-1.979511956	0.02331342304
**RASGRP1**	-2.255421973	0.02333289846
**NPR1**	-3.261858369	0.02353109919
**STKLD1**	-1.593759505	0.02353526342
**C20orf194**	-1.095541521	0.02358174193
**ATP6V1B1**	-1.639697141	0.0235821543
**ENDOU**	-4.481845887	0.02358390116
**GPSM3**	-1.593961387	0.02358390116
**SAMD14**	-3.210717561	0.02361794864
**RTP1**	-5.119707558	0.02361794864
**SMC5-AS1**	-1.897243206	0.02369980112
**CDK4**	-1.080127317	0.02382768186
**PLOD2**	-1.094339997	0.02385448137
**FAM182A**	-3.610013261	0.02387122837
**MEPCE**	-1.418934641	0.02387366844
**RSPH14**	-3.872224168	0.02404210977
**SSH1**	-1.210330621	0.02404780723
**RP11-266K4.9**	-4.240484463	0.02410366745
**SEMA6A-AS2**	-3.304025762	0.02410976757
**PNCK**	-2.012182039	0.02413654138
**IGLON5**	-5.574917337	0.0242550133
**ERMAP**	-1.565357325	0.02430926605
**LINC01176**	-2.557288743	0.02431191476
**KANTR**	-1.274402115	0.02433597946
**IGFBP3**	-3.485180675	0.02436418194
**PTPN9**	-1.009102308	0.02443376064
**RP1-274L7.4**	-3.830894524	0.02450292679
**NFKBIB**	-1.165949929	0.0245438539
**MAFTRR**	-4.19633104	0.02455506531
**C5orf67**	-5.027492741	0.02460976606
**SPX**	-3.521762736	0.0246260443
**RP11-402D21.2**	-2.199728472	0.02464133448
**DBP**	-1.253145196	0.0246848529
**C1orf21**	-1.66415185	0.02473822744
**ZNF702P**	-4.678485478	0.02480551964
**TENM4**	-5.697139054	0.02483349446
**MGARP**	-3.460228809	0.02484654259
**TEKT3**	-2.570098095	0.02496524068
**ACSS1**	-1.001498873	0.0250473989
**AC083867.4**	-4.653553283	0.02528750154
**POMZP3**	-1.390858878	0.02538695052
**HERC2**	-1.107655719	0.02539201053
**CXCL11**	-4.886617656	0.0254483105
**LA16c-313D11.1**	-2.757496698	0.0254956113
**RNF208**	-1.949666981	0.02554610578
**YWHAH**	-1.16405029	0.02558465875
**MATN3**	-4.59424317	0.02558909313
**VOPP1**	-1.468894317	0.02577933295
**ZNF165**	-1.943188722	0.02577933295
**RP11-767N6.7**	-1.718742998	0.02577956814
**TOB1**	-1.404346425	0.02578653601
**MYCBP2**	-1.320982412	0.0258168125
**TPTE2P5**	-3.062938455	0.02589483178
**RP11-465C12.1**	-4.999887627	0.02589483178
**ENO3**	-1.927766405	0.0259800486
**AC009473.1**	-2.9445404	0.02598977373
**MEOX2-AS1**	-5.001717284	0.02608338642
**TAF1C**	-1.124258077	0.02609705691
**PHLDA1**	-1.151633818	0.02620723528
**LINC01351**	-2.878391493	0.0262392154

**GRM7**	-3.468244654	0.02629536729
**SNHG11**	-1.126572362	0.02635014089
**THSD1**	-2.519072703	0.02637928321
**COLCA1**	-4.48488505	0.02648659538
**PTCD1**	-1.174756734	0.026520623
**BBOX1-AS1**	-4.343046929	0.026520623
**PAQR9**	-2.369702211	0.02671071193
**TBCK**	-1.366191321	0.02671588276
**RP11-386G11.8**	-4.980436546	0.02682216905
**FRMD6**	-1.249605373	0.02693390969
**RGS12**	-1.549395277	0.02693442205
**GSTZ1**	-1.140401851	0.02696471224
**RP11-103J8.1**	-4.62662294	0.02702386982
**PTPRN**	-3.977913973	0.02736436108
**NPEPL1**	-1.520914885	0.02737289541
**RBPMS**	-2.56712388	0.02742119653
**CUBNP2**	-4.970357227	0.02746794017
**TFCP2L1**	-2.711430953	0.02749247229
**C12orf49**	-1.404722565	0.02751362305
**ZNF184**	-1.215098531	0.02755787246
**EMC8**	-1.024324004	0.02758436408
**RGMB-AS1**	-2.82462874	0.02763328829
**MFSD2A**	-1.608454625	0.02766165192
**TAF6L**	-1.054566535	0.02769866225
**ANK1**	-3.324685999	0.02784548501
**HOXC4**	-2.415318087	0.02784548501
**HIST2H4A**	-2.194569767	0.02788244635
**C16orf45**	-3.139798339	0.02790567306
**C11orf96**	-2.612570619	0.02797058924
**ARMC7**	-1.334944859	0.02797615493
**COA3**	-1.147274974	0.02809119485
**TBKBP1**	-1.015032544	0.0280926059
**LINC01679**	-4.497326429	0.02812941459
**AP000662.4**	-2.885341154	0.02812941459
**LAMTOR4**	-1.171464262	0.02817940831
**ZNF410**	-1.642313258	0.02835847673
**C5**	-2.197702672	0.02837920971
**RP11-615I2.2**	-4.365796099	0.02857671418
**LDB2**	-5.615356655	0.02860894651
**GADD45G**	-4.623560495	0.02865309745
**SLC22A17**	-1.4546415	0.02866036952
**SLC35E2**	-1.22683416	0.02876714954
**RP11-762I7.4**	-2.278249563	0.02883168565
**FAM53B**	-1.032313943	0.02886202535
**RP11-4B14.3**	-4.355487064	0.02886202535
**FLT1**	-5.331703573	0.02886266936
**AKIRIN2**	-1.093749503	0.02886266936
**RP11-631N16.2**	-1.085770273	0.02886266936
**RP11-728K20.1**	-4.929964944	0.02886469305
**CTD-2010I16.1**	-3.238439289	0.02887140667
**ZNF432**	-1.261372899	0.02918046129
**C8orf33**	-1.143859349	0.02919718426
**DICER1-AS1**	-1.364817281	0.0292484033
**ZNF664**	-1.115615316	0.02930132666
**PCDHGC5**	-2.233557493	0.02936653155
**LINC01207**	-4.708937508	0.02946120168
**RAPGEF4-AS1**	-4.937860174	0.02950265689
**SNAI2**	-3.034326106	0.02964744196
**SEZ6**	-5.337512154	0.02964744196
**LACTB2-AS1**	-4.286377731	0.02964744196
**PRAG1**	-2.397735907	0.02964744196
**GRIN2D**	-2.929729894	0.02965218063
**CHMP3**	-1.099229998	0.02973509386
**RP11-1280N14.3**	-2.45280853	0.02986669296
**USP5**	-1.10345883	0.02993405952
**TMEM88**	-3.931908579	0.03020942305
**LHX6**	-3.032134934	0.03061656041
**AC007255.8**	-3.502363755	0.03069365847

**NFIA**	-1.101880345	0.03121421558
**AQP4**	-5.303845444	0.03128250069
**RP11-977G19.5**	-1.031585015	0.03128250069
**ZNF497**	-2.121990603	0.03144826689
**MEX3B**	-1.068023807	0.03152127912
**PRR26**	-4.360719055	0.03165134489
**RP11-536C5.2**	-4.323396763	0.03165134489
**SPON1**	-4.383978921	0.03169829402
**DYNC1I1**	-1.592384778	0.03173060018
**SLC38A9**	-1.102937651	0.03174089186
**RP11-4L24.4**	-1.128201374	0.03186785628
**ARHGAP24**	-2.994552185	0.03194872269
**TTLL7**	-1.276972724	0.03197829912
**RP11-156P1.3**	-1.092878188	0.03206607327
**DTX2**	-1.479157327	0.03208360245
**ARPC4**	-1.03693576	0.03217150924
**RP5-981O7.2**	-2.847910582	0.03218753862
**GBP2**	-2.065336421	0.03219449816
**AP000704.5**	-2.044727445	0.03219449816
**LINC01080**	-4.462752051	0.03219449816
**AP001626.2**	-2.759241704	0.03227373944
**AC025171.1**	-1.521519352	0.03242846035
**RPH3A**	-5.387875211	0.0324331169
**RNASE6**	-4.434991614	0.03248716944
**CD9**	-1.311068127	0.03250234054
**RP11-445N18.5**	-4.872461677	0.03250247295
**UST**	-1.585044001	0.03258029553
**SLC2A11**	-1.269476672	0.03262397763
**KCNQ4**	-4.048889727	0.03263057323
**SKIL**	-1.363466368	0.03273332038
**C1orf122**	-1.423379664	0.03299355355
**SMIM3**	-2.59175709	0.03306528749
**NAPSA**	-3.887056667	0.03314059298
**SIX3**	-4.386238009	0.0332906455
**NR1D2**	-1.065319917	0.03358664258
**RP11-283G6.4**	-3.20224338	0.03358664258
**KB-318B8.7**	-1.944256244	0.03362102375
**FAM13A-AS1**	-1.68370732	0.0336246098
**ARHGEF37**	-1.487682197	0.03379650228
**HOXD13**	-3.154095298	0.03392010609
**CCDC159**	-1.294502763	0.03393469197
**ZNF663P**	-4.320542161	0.03395267252
**RP11-15I11.3**	-3.567386603	0.03422589494
**ROCK1P1**	-2.039979695	0.03423169291
**RP11-576I22.2**	-2.829342613	0.03429533444
**CHRFAM7A**	-3.315962818	0.03431800531
**CTD-2339F6.1**	-5.01173296	0.03435862537
**HSF2BP**	-1.615555654	0.03447360378
**HIF1A-AS1**	-2.767681274	0.03452866598
**BHLHB9**	-1.214364914	0.03466837556
**ST6GALNAC6**	-1.40334193	0.03470165226
**RP11-182J1.14**	-3.303699488	0.0347027342
**FOXRED2**	-1.059887226	0.03470882196
**ARHGAP42P2**	-4.285735307	0.0347683869
**CTD-2267D19.3**	-2.037278158	0.03477260771
**MIR181A1HG**	-3.345085444	0.03482068087
**PDLIM5**	-1.044069279	0.03484552108
**PINK1-AS**	-1.057937569	0.0349061461
**ADCY2**	-3.90805118	0.03505702405
**ZFP37**	-1.191089625	0.03531694235
**CNGA3**	-4.457249307	0.0353337303
**LRP2BP**	-1.857891393	0.03534109477
**SCD5**	-1.247348129	0.03539622341
**PTGDS**	-2.914017724	0.03539875304
**RP11-649E7.5**	-2.509432048	0.0354452056
**RP11-138I1.3**	-4.407859811	0.0354452056
**ZSCAN22**	-1.142902928	0.03545060999
**CEP162**	-1.670415613	0.03553978026

**RP11-624M8.1**	-3.838254014	0.03567976326
**PODXL**	-1.022715717	0.0356894481
**TIMELESS**	-1.025764368	0.03568986748
**ABCB4**	-3.485831353	0.03586087542
**G6PC3**	-1.080659601	0.03590447464
**TBC1D20**	-1.092307016	0.03600027876
**RP11-798M19.3**	-1.580474485	0.03616799772
**DHX29**	-1.017302754	0.03620890119
**LINC00342**	-1.17401864	0.0362703262
**LAMA2**	-2.232943913	0.03644377349
**TACC1**	-1.073020901	0.03644455666
**RP11-109E12.1**	-4.346789663	0.03650719876
**RAD9B**	-1.53490995	0.03660662791
**ZSCAN12P1**	-2.824494825	0.03660662791
**RIPPLY3**	-4.279496184	0.03664590129
**H2AFX**	-1.32837733	0.03678170336
**RBM26**	-1.069181615	0.03681120708
**RP11-146D12.2**	-2.33503296	0.03686205144
**CPNE4**	-4.865033835	0.03694665305
**TNFSF13**	-2.944961944	0.03695774867
**ZNF554**	-1.071057791	0.03698008006
**WNT7B**	-2.993345368	0.03698862136
**IL20RA**	-4.927124666	0.03699888875
**ITGA1**	-1.060674415	0.03703453328
**MYBPHL**	-3.910745144	0.03722460185
**NBPF10**	-1.079349352	0.03733945011
**LINC00598**	-3.25721974	0.03740130574
**WBP1L**	-1.199936575	0.03751585839
**ZCCHC18**	-1.226255666	0.03751585839
**POM121B**	-1.835226599	0.03753094183
**SMAD7**	-1.220665672	0.03755200236
**GRID2IP**	-3.025782384	0.03767317655
**PACS2**	-1.114402437	0.03768606891
**CSRP1**	-1.197304934	0.03803866331
**RHBDD3**	-1.133610052	0.03806597832
**EFHD1**	-2.136805664	0.03825883665
**SPRED2**	-1.132952794	0.03834267084
**FBXW2**	-1.108008763	0.03835017869
**AKAP3**	-1.737265058	0.03837795254
**CAMK2D**	-1.860134917	0.03850898767
**ZMAT1**	-1.271681481	0.03854992543
**TNFSF4**	-2.047753265	0.03875376185
**STX1B**	-1.896603968	0.03875464921
**DPM2**	-1.183995468	0.03880813815
**CTB-179K24.3**	-3.105323631	0.03897929109
**KCNJ16**	-4.291577987	0.03898190613
**SH3RF1**	-1.323076361	0.03938598822
**SLC38A6**	-1.146823099	0.03948874047
**ACSL6**	-4.332264302	0.03948874047
**ZNF324**	-1.076614657	0.03955312061
**PLCL1**	-3.087375714	0.03955655056
**COL4A3BP**	-1.373986406	0.03973000025
**ZNF33B**	-1.738782497	0.03981548942
**RBM6**	-1.011106047	0.03993438768
**CTSS**	-2.548455445	0.03993438768
**FAM174B**	-2.454478594	0.04000782695
**CA3-AS1**	-3.408899148	0.04007012692
**PITX2**	-3.176344797	0.04011675102
**SATB1-AS1**	-4.265492318	0.04023208631
**MGAT4A**	-1.86885204	0.04036255058
**CH507-9B2.9**	-1.414574764	0.04036255058
**FGFR2**	-1.679881674	0.0404314925
**NDE1**	-1.00806691	0.04055652833
**CTD-2619J13.3**	-3.870494129	0.04066267001
**SEPT7-AS1**	-1.273688927	0.04070666908
**ADAM19**	-3.106171353	0.04072612632
**C22orf24**	-3.362164712	0.04078140421
**HAVCR2**	-2.701801774	0.04078140421

**SSPO**	-2.781909787	0.04104117359
**ATP11A**	-1.141711817	0.04120840326
**ZER1**	-1.199783197	0.04120840326
**MSS51**	-1.103355636	0.04123771566
**TTLL1**	-1.001596167	0.04129163677
**CTC-325H20.8**	-4.379620268	0.04129163677
**CLN6**	-1.011937542	0.04139550145
**DOPEY1**	-1.404679516	0.04143512895
**HIRA**	-1.078028655	0.04146879641
**IL1RAP**	-2.900354913	0.04148729096
**RP13-644M16.5**	-4.354222882	0.04152622899
**LRRC9**	-4.700008534	0.04183496363
**RP11-431M7.3**	-4.378161653	0.04183496363
**FAM19A5**	-4.065415292	0.04186174727
**PON2**	-1.368842646	0.04192275078
**CTD-2514C3.1**	-2.222142808	0.04194898996
**RP11-84G21.1**	-1.530244352	0.04196204402
**BORCS7**	-1.703255918	0.04204337993
**SLC35D3**	-4.46357639	0.04210636138
**CLCN4**	-1.017580389	0.04216759745
**CNTNAP1**	-1.348060327	0.04218039325
**LINC01206**	-4.551707684	0.04221773341
**C15orf59**	-2.09205171	0.04257278328
**LINC01852**	-1.687920867	0.0426691845
**IGSF9**	-3.833904903	0.04272012445
**WEE2-AS1**	-1.371126783	0.04272802559
**ZSCAN30**	-1.133090654	0.04287216576
**MIPEPP3**	-1.459318041	0.0429307971
**AP000432.1**	-1.255746825	0.0431367002
**ZBTB20**	-1.134427569	0.04320818754
**HYAL1**	-3.016854124	0.04326354761
**NFIA-AS2**	-4.690934248	0.04341261462
**HSD11B1L**	-1.169662346	0.04341275038
**RP11-685N10.1**	-1.873748264	0.04343756073
**RP5-884C9.2**	-4.67028136	0.04372566592
**RP11-770J1.3**	-1.978003662	0.04376810314
**CCDC157**	-1.924673812	0.04387464067
**RP11-369K16.1**	-4.378477827	0.0439055597
**LINC00519**	-4.174203244	0.04414826492
**RP11-712B9.2**	-1.681867447	0.04438495977
**ZSCAN9**	-1.143102764	0.04440547237
**CORO1C**	-1.371073466	0.04444309768
**NRXN2**	-3.265152575	0.04446223563
**KALRN**	-2.765885882	0.04446223563
**POC1B-AS1**	-1.487207304	0.04446223563
**ROBO2**	-3.104746093	0.04512684457
**MCTP2**	-3.767260804	0.04523505001
**NREP**	-2.461486964	0.04536548297
**AKAP9**	-1.053334069	0.04537460484
**OTOGL**	-3.202638704	0.04566219905
**AGBL4**	-3.874891035	0.04569855501
**FGFR1**	-1.811983801	0.04574920331
**MYO5A**	-1.8759313	0.0457881529
**RP11-359B12.2**	-1.014373795	0.04585671536
**TPST2**	-1.635293308	0.04596908844
**CTC-344H19.4**	-3.534171212	0.046018976
**AC007566.10**	-1.014212726	0.04603914804
**RNF219-AS1**	-4.287346862	0.04607192115
**HEY1**	-1.938937907	0.04610567765
**CCDC93**	-1.053906336	0.04613359129
**IL1RAPL2**	-4.641402639	0.046158938
**SLC2A10**	-1.896922159	0.04618397365
**RANBP3L**	-4.054300833	0.04627817694
**MSI1**	-1.269715637	0.04628176266
**COL4A5**	-1.165371807	0.0462852139
**LINC02082**	-2.0766378	0.04636223328
**ADAMTS14**	-3.460689324	0.04636286823
**HES6**	-1.291320261	0.04637935409

**PTPN13**	-1.817108422	0.04651237249
**NCK1-AS1**	-1.304319419	0.04651237249
**CCND2-AS1**	-3.947812765	0.04700024178
**AC018804.6**	-3.27395259	0.04707095723
**VEZF1P1**	-1.223038487	0.04722431029
**THRB**	-2.247924883	0.04737934441
**FAM189A1**	-3.061011094	0.04790594259
**GFAP**	-4.644488325	0.04797167238
**KLHDC1**	-1.635443325	0.0484250351
**NRBP2**	-1.153527038	0.04855267055
**BCL11A**	-2.418681084	0.04862494178
**LINC01252**	-2.073475688	0.04871208694
**ZBTB21**	-1.040193851	0.04872465371
**SKIDA1**	-1.19451375	0.04872465371
**PTP4A1**	-1.179304474	0.0489195171
**RGS11**	-4.442310826	0.04898101713
**ZBBX**	-3.102530499	0.04909249068
**RP5-994D16.3**	-2.841919319	0.04942585422
**HS6ST1**	-1.229941371	0.04945159219
**TCEAL8**	-1.00031066	0.04956146826
**MAGED2**	-1.077604338	0.04959084528
**STAC3**	-1.509375238	0.04968037008
**BTBD19**	-2.424382464	0.04970254872
**ALDH3A1**	-3.812078314	0.0497637965
**SHISA9**	-4.285136647	0.04994722536

Supplementary Table 6: Highly expressed (multi-exon) genes in mesenchymal (MES) and proneural (PN) GSCs (log2FC > 3 and FDR < 0.05), with expression differences between MES and PN GBM (|log2FC|>0).

Highly expressed genes in MES			
gene	GSCs.log2FC	GSCs.FDR	GBM.log2FC
ANPEP	10.3767	1.99E-70	2.09953
CDCP1	10.4075	4.38E-70	1.38869
SLFN11	10.6064	5.23E-70	0.11886
FOSL1	6.6273	6.26E-67	1.55408
TGM2	8.0496	2.47E-62	1.21532
ALDH1A3	9.6937	4.46E-58	1.68304
CTSZ	9.2329	3.20E-56	1.47754
FGF5	9.9146	9.77E-56	0.53909
MDFIC	8.2457	1.49E-53	1.36954
DNAH14	9.9210	1.48E-51	0.35166
HTATIP2	9.5804	1.73E-51	0.50266
PROCR	6.3594	3.17E-47	0.81380
C4orf32	9.2232	2.46E-46	0.57075
PLBD1	9.3612	1.24E-45	1.47594
MAGEB2	10.4771	4.67E-45	0.46958
TNFRSF10D	9.8447	5.15E-42	1.22471
EGR1	5.8680	5.36E-42	0.71143
STEAP2	10.0367	3.45E-41	1.10119
TUSC3	10.1375	3.85E-41	0.17589
BAIAP2L1	6.3678	1.04E-40	0.93680
MAGEA12	8.8877	2.63E-40	0.05420
SSH3	6.3416	1.85E-39	0.53649
ME1	9.9955	1.63E-37	0.49086
AIM1	4.4846	2.27E-37	1.72706
KRT8	9.6747	7.60E-37	1.07739
DSP	8.7318	1.50E-36	0.48866
ADIRF-AS1	8.1469	3.93E-36	0.30912
EFEMP1	10.9410	1.18E-35	1.50093
PRAME	11.3030	2.28E-34	1.27959
LY6K	9.0424	1.69E-33	0.27941
NFE4	9.9765	6.41E-33	0.97714
KCNC4	5.7273	8.91E-32	0.04717
LRRK1	8.5715	1.06E-31	0.65678
VRK2	8.8949	2.36E-30	0.63973
FAT4	6.4036	2.76E-30	0.72110
CDYL2	4.0989	3.66E-30	0.17782
DOCK5	9.9519	4.36E-30	0.62253
KATNAL1	3.9237	2.47E-29	0.14296
RAB11FIP1	7.7864	5.32E-29	1.16974
RP11-29H23.5	5.5037	1.10E-28	0.27002
EGFR	8.0550	2.38E-28	0.87717
GALNT6	9.5208	5.22E-28	0.90483
KRT18	10.4865	8.62E-28	1.19541
STEAP1B	7.7408	1.01E-27	0.12919

Highly expressed genes in PN			
gene	GSCs.log2FC	GSCs.FDR	GBM.log2FC
GPM6B	10.7109	3.53E-127	0.0319
PDE4B	9.6990	7.76E-74	0.0007
PMP2	10.5275	1.69E-69	0.2675
COL11A1	9.8527	2.15E-68	1.0411
ARNT2	9.1545	4.87E-65	0.1831
PTPRZ1	10.2884	1.65E-64	0.4263
PRTFDC1	10.0067	1.29E-63	0.3787
KCNJ10	9.8793	2.84E-63	0.4904
KCNA2	10.1004	1.60E-61	0.9668
FEZ1	10.1715	4.86E-57	0.3374
BCAN	10.2891	4.13E-56	1.3340
EDNRB	10.4157	1.47E-55	0.6797
ZNF532	10.7090	7.97E-54	0.2351
ARL10	8.2031	9.15E-54	0.5634
PCDH15	9.3152	3.32E-52	2.7574
FXYD6	9.9132	1.22E-51	1.1235
OLIG2	10.2669	3.17E-50	1.7045
NLGN3	7.8820	1.09E-49	0.6115
TUBB2B	10.3490	4.63E-46	0.5740
LSAMP	11.0931	2.38E-45	0.8173
JAM3	10.0985	3.23E-45	0.1004
VCAN	10.5979	6.34E-45	0.0602
GNG2	10.3773	2.35E-44	0.8572
SEMA5A	8.6201	2.59E-44	0.4534
SLC35F1	9.9135	1.09E-43	0.9902
OLIG1	10.4094	1.77E-43	1.6634
CSPG4	9.9819	1.87E-43	0.3887
SOX2-OT	9.1016	5.55E-43	0.3283
ANGPTL2	10.2187	2.80E-42	0.1308
MMP2	10.5826	1.13E-41	0.1237
ZNF608	8.8905	3.60E-41	0.0877
ZNF354C	6.9885	3.01E-40	0.3125
FREM2	5.3429	1.36E-39	0.3714
ZNF300	11.0075	4.33E-39	1.1409
MAP2	8.5508	1.24E-37	1.0706
MPHOSPH8	9.9602	2.46E-37	0.0527
ZNF334	9.1671	2.52E-37	0.0701
MDFI	9.4470	2.61E-37	0.7509
CELF2	9.1976	3.31E-37	0.2632
ZNF736	10.0130	3.31E-37	0.3164
SULF2	9.4323	3.40E-37	0.7024
ZNF542P	8.2413	1.08E-36	0.0470
FGFBP3	4.8909	1.31E-36	1.1660
SETD6	7.3817	1.68E-36	0.2128

ZDHHC23	5.8260	1.38E-27	0.58893
ADM2	8.1786	3.47E-27	0.18935
RUNX3	7.9180	1.00E-26	0.46877
FAM83G	8.1384	2.19E-26	0.91705
TES	11.5896	2.98E-26	0.90344
CASP4	5.4741	5.91E-26	1.14022
MIPEP	3.6301	6.82E-26	0.42856
ANKRD27	3.6217	2.55E-25	0.05869
NIPAL2	8.3632	1.11E-24	1.10676
CADPS2	8.1288	1.94E-24	1.17465
STAMBPL1	6.8045	2.61E-24	0.00945
MAP3K21	4.4245	5.59E-24	0.40366
SPAG1	4.6764	1.16E-23	0.77369
MAT1A	7.7902	1.41E-23	0.45222
MIR222HG	4.0647	3.46E-23	1.40482
IER3	8.7015	4.32E-23	1.17211
CHAC1	5.2816	4.60E-23	0.37037
PRKCH	6.3346	7.52E-23	0.39253
DUSP23	7.1703	7.62E-23	0.98536
RP11-66B24.7	8.9782	8.13E-23	0.19592
GBP3	5.9100	1.02E-22	1.18576
CATSPER1	7.9869	1.24E-22	1.26298
ECHDC2	6.6816	1.60E-22	0.68730
DENND2C	5.8353	2.82E-22	0.21653
ALS2CL	6.8183	3.48E-22	1.41048
LTBR	11.6376	4.79E-22	0.89730
G0S2	7.1054	5.12E-22	1.13372
AOX1	7.8808	9.45E-22	0.56697
TPM2	4.7180	1.31E-21	0.70770
DMKN	10.8580	2.40E-21	1.43401
NME7	3.1848	4.21E-21	0.03588
RASGEF1A	11.3283	6.39E-21	0.12246
SELENOM	6.4010	7.38E-21	0.92268
SIK1	4.7753	1.51E-20	0.61608
IL4R	8.4823	1.84E-20	1.01604
EMB	11.6151	2.13E-20	1.54406
SERPINB9	8.0080	2.24E-20	0.75261
PPARG	8.8885	2.51E-20	0.61278
KCNG1	6.4498	2.70E-20	0.68860
MPP7	5.7031	3.90E-20	0.06112
TNFRSF10A	11.1730	6.54E-20	1.06264
CTGF	5.8601	1.69E-19	0.61500
PMAIP1	5.7864	1.71E-19	0.64524
CSAG1	10.3953	2.24E-19	0.08240
IL7	6.9248	2.84E-19	0.83074
RERG	6.4640	2.84E-19	0.70168
NIPAL1	5.7869	3.62E-19	0.09044
PTPRH	6.8849	3.71E-19	0.93175
PARP4	3.6642	3.83E-19	0.53202
SCRG1	11.0053	2.13E-36	0.4437
ANK3	5.4055	3.28E-36	0.5443
VANGL2	10.0599	4.66E-36	0.4403
DZIP1	10.4170	7.91E-36	0.2864
MLLT11	3.6619	1.41E-35	0.9375
LINC00511	4.7276	8.94E-35	0.6536
PCDHGC3	8.3531	1.06E-34	0.4938
ARMCX4	9.5542	1.39E-34	0.6346
ZNF883	7.8610	2.51E-34	0.3449
CPXM1	10.5931	2.90E-34	0.1185
TTYH1	9.2750	3.37E-34	0.5267
RFTN2	9.4866	3.83E-34	0.5545
SALL3	9.4843	7.54E-34	1.3368
SOX5	10.4488	8.88E-34	0.7518
TNS2	5.2750	1.11E-33	0.0346
LINC00665	6.5084	2.47E-32	0.3358
ZNF93	6.9929	5.30E-32	0.4982
ZCCHC24	3.6333	5.98E-32	0.2863
DISC1	7.7497	6.46E-32	0.5254
SALL2	10.1305	7.18E-32	0.1669
CHFR	4.3815	1.70E-31	0.2096
ZNF585B	5.8969	2.98E-31	0.0772
GNG7	3.5925	5.58E-31	0.3424
COL9A3	10.8461	7.89E-31	0.6293
SV2A	10.5071	8.45E-31	0.4885
PNMAL1	10.6681	1.01E-30	0.4353
GPRC5B	6.2540	1.37E-30	0.2128
PCDH17	7.3463	1.62E-30	0.4427
PPP1R9A	10.2710	3.18E-30	0.2278
CGREF1	8.2704	5.04E-30	0.5471
PROM1	9.5944	2.08E-29	0.5557
ZNF431	10.5750	5.15E-29	0.5580
SOX8	10.6199	6.31E-29	1.9157
CCDC149	9.6059	1.74E-28	0.0466
FRMD5	3.4701	2.15E-28	0.3661
AIF1L	6.5808	5.14E-28	0.6226
DDX19B	7.0008	1.38E-27	0.0338
ZNF43	10.4614	1.44E-27	0.3268
PAG1	6.0137	1.28E-26	0.3382
FLRT3	9.4041	1.57E-26	0.4117
ELMO1	10.3865	1.57E-26	0.9021
HUNK	8.5989	1.94E-26	0.7926
GTF2I	3.5369	1.97E-26	0.1669
ARHGEF7	3.1696	2.19E-26	0.8176
CERS6	11.6372	2.20E-26	0.1270
FAM222A	4.4462	2.27E-26	1.8273
GREB1	4.5814	6.44E-26	0.3667
COL20A1	8.5074	2.20E-25	2.6896
RP11-43F13.1	5.4935	2.46E-25	0.0034

TPBG	10.9432	3.92E-19	1.78491
RP11-420A23.1	3.6846	4.13E-19	0.05179
TRIM58	9.1570	5.22E-19	0.41985
PCK2	4.0535	9.70E-19	0.52767
SERPINB1	6.8758	1.82E-18	1.17338
BCL2A1	9.5784	2.28E-18	1.25019
CHMP4C	10.6741	4.88E-18	0.67970
LINC00707	8.8163	5.03E-18	0.24621
RCN3	4.9144	5.39E-18	1.15529
SLFN12	9.8596	6.30E-18	0.68571
PLA2G16	3.4570	6.33E-18	0.41644
MGST1	8.3238	7.62E-18	0.92754
CT45A10	9.8426	8.52E-18	0.57785
CD40	9.8483	9.59E-18	0.80510
MLPH	9.9790	1.03E-17	0.99098
SPAG4	4.8318	1.24E-17	0.49310
H2AFJ	10.1085	1.58E-17	0.68030
TNFAIP2	4.5793	1.82E-17	1.63649
ELL2	4.3705	1.84E-17	0.70862
TBX18	10.5513	1.88E-17	0.86630
LDHC	7.8273	2.32E-17	0.12632
AC003092.1	10.5004	3.23E-17	0.97824
MAN1A1	5.9947	3.81E-17	1.56757
LINC00857	8.3517	5.01E-17	1.19285
HS3ST3A1	8.2866	5.75E-17	0.49443
WDR66	6.4268	7.31E-17	0.68652
RP5-884M6.1	9.7256	8.11E-17	0.50697
MME	7.5809	1.13E-16	2.08298
IL31RA	10.2933	1.31E-16	2.41949
GFPT2	3.7890	2.39E-16	1.13101
CXCL5	9.8873	2.48E-16	2.06286
RP11-366H4.1	10.0323	3.19E-16	1.78157
DMC1	4.5325	3.59E-16	0.81462
ZNF215	10.0254	3.62E-16	0.70861
EXT1	3.4344	3.92E-16	0.19825
CCDC68	8.2244	3.99E-16	0.44872
TMEM156	9.5593	5.31E-16	0.23005
IRF5	7.0097	5.34E-16	0.66357
FBXL13	4.0736	7.04E-16	0.46210
RP11-108M9.4	6.8084	8.17E-16	0.65201
TM4SF19	8.1077	1.12E-15	0.83681
UBR5-AS1	3.5689	1.20E-15	0.21289
ARNTL2	3.1121	1.27E-15	0.53968
CD55	6.4161	1.55E-15	0.60302
HLA-F	3.7857	1.74E-15	0.76844
ECHDC3	9.9163	1.95E-15	0.40381
LINC00944	9.8211	2.02E-15	0.31916
HHEX	9.2847	2.09E-15	0.64843
RP11-148B18.4	9.2308	2.71E-15	0.96897

LINC00461	4.0718	3.20E-25	0.3884
TCF4	6.3969	9.15E-25	0.3696
PCYT1B	9.7393	1.14E-24	0.2838
STK32B	9.9483	1.30E-24	0.6871
C8orf46	7.5529	1.30E-24	0.7258
MAPK10	5.5778	2.41E-24	0.4253
SATB1	8.1516	2.44E-24	0.8926
SLIT1	8.8588	2.97E-24	1.3022
C3orf70	9.8798	4.32E-24	0.6956
DGKI	8.0565	4.98E-24	1.0900
ID4	9.6761	5.08E-24	0.4220
ASIC4	7.3633	6.89E-24	1.6741
KCNIP1	10.4082	1.00E-23	0.4133
FAM49A	9.7027	1.06E-23	0.0765
TMEM246	9.9217	1.46E-23	1.2458
NLGN4X	11.2397	2.53E-23	0.0599
DTX3	9.4179	2.67E-23	1.7209
PPP1R14C	11.0118	2.80E-23	0.4694
TET1	9.2650	4.27E-23	0.8858
ZNF738	11.1390	6.12E-23	1.0439
ZNF382	7.3153	6.23E-23	0.3182
SEMA5B	8.9971	6.86E-23	0.9562
LGI2	9.6623	6.86E-23	0.0194
HSD17B7P2	4.5378	7.26E-23	0.2911
NIM1K	6.8967	7.95E-23	0.2594
PEG3	9.7716	9.70E-23	0.8884
KLHL13	7.7314	1.55E-22	0.5893
ZNF506	9.6657	3.80E-22	0.3161
JAKMIP2	9.2861	4.60E-22	0.0455
HR	8.4776	4.64E-22	1.4023
B3GAT1	10.7366	7.25E-22	0.9428
BCHE	6.7738	2.14E-21	0.4914
GOLGA8A	4.2395	2.24E-21	0.3784
PDGFRA	7.7900	3.32E-21	2.7554
UNC80	6.8929	3.32E-21	0.8055
RNF175	10.6522	7.91E-21	0.1974
CD200	7.1215	1.07E-20	0.5397
ATCAY	8.8905	1.24E-20	1.9143
TBCEL	10.5117	1.63E-20	0.0253
LRRTM2	8.2571	1.96E-20	0.9006
PTPRO	9.4924	2.01E-20	0.5242
SEMA6A	3.7219	2.06E-20	0.3107
BRSK2	9.1250	2.23E-20	1.7043
DPYSL2	3.2855	3.22E-20	0.0300
ZNF577	4.7199	4.14E-20	0.2319
AC074289.1	6.6117	5.13E-20	0.2217
DOCK10	5.3177	5.21E-20	0.2855
ZNF254	3.3001	5.92E-20	0.2384
SHISA4	8.2521	6.07E-20	0.2450

TEC	9.7881	2.87E-15	1.38344
MSC-AS1	7.9784	5.06E-15	0.23046
SRGN	8.9991	5.33E-15	1.23161
HTR7	9.0756	8.97E-15	1.20624
KIRREL2	5.6129	9.91E-15	0.14202
LINC01605	7.7660	1.00E-14	2.15906
TOR4A	5.5083	1.13E-14	0.90684
CASC9	9.5504	1.20E-14	0.62265
RP11-66B24.2	9.6835	1.23E-14	0.44563
ANKRD29	5.0472	1.75E-14	0.95849
CTB-140J7.2	9.0559	1.87E-14	0.75484
PLCG2	9.5905	2.05E-14	0.77150
MIR137HG	9.4635	2.91E-14	0.02217
RP4-647C14.2	9.4408	3.12E-14	0.05569
ADGRE1	9.9151	3.80E-14	1.50048
WBP2NL	6.0319	3.81E-14	0.11438
LINC00910	4.0521	4.78E-14	0.16762
ZNF185	4.0386	4.79E-14	0.93264
MVP	4.6077	6.03E-14	1.09301
VEGFC	8.8362	6.20E-14	1.18112
LRRC8E	8.8584	6.47E-14	2.36695
XAGE1A	8.8426	6.51E-14	0.14350
MTMR11	3.0034	8.69E-14	0.62618
ADTRP	9.3155	9.03E-14	1.08193
CRLF1	8.3375	1.05E-13	0.98678
ANKRD33B	3.5772	1.07E-13	0.01018
SPOCD1	6.7388	1.11E-13	1.70761
ENPEP	7.7342	1.37E-13	0.50792
AC009237.8	9.3297	2.55E-13	0.39299
CHRNB1	3.3819	2.84E-13	0.28296
POMP	3.0772	2.86E-13	0.28585
PI15	9.2873	3.53E-13	0.90130
UAP1	3.9178	3.56E-13	0.51545
TYMP	7.7895	4.06E-13	1.06827
TRDN	7.4584	4.29E-13	0.03248
SYTL3	4.4968	4.53E-13	0.61531
ADAMTSL5	4.5414	6.83E-13	0.53055
NEXN	3.0647	7.17E-13	0.61634
CYR61	5.2672	7.41E-13	1.02943
STARD8	4.2152	8.20E-13	0.72133
TINAGL1	9.5074	8.94E-13	0.56862
C9orf84	8.6571	9.74E-13	0.02998
XDH	9.8222	1.07E-12	1.69047
POLD4	4.0833	1.07E-12	0.80954
MICAL2	3.5865	1.11E-12	0.61134
SULT1B1	9.0664	1.36E-12	0.81093
PDLIM1	6.2795	1.36E-12	0.66582
PIK3CD	4.1589	1.58E-12	0.60764
MELTF	3.4979	1.83E-12	0.52782

BAALC	9.8395	8.66E-20	0.3276
SLITRK2	10.0475	1.22E-19	1.1851
ZNF737	7.7906	1.36E-19	0.7210
MAGI2	4.6993	1.77E-19	0.3501
EFS	11.7172	2.19E-19	0.6064
ZNF529	10.3714	2.19E-19	0.2753
ZNF85	8.9232	2.41E-19	0.4293
KLHDC8B	5.1776	2.70E-19	0.2149
SHROOM2	9.2579	3.70E-19	0.1916
ZNF853	9.4885	4.76E-19	0.4280
ZNF100	10.1631	6.51E-19	0.4029
ZNF813	5.7554	8.26E-19	0.1357
DPYSL3	5.0577	1.22E-18	0.0746
GRIA3	9.6760	1.44E-18	0.5356
HS6ST2	11.4114	1.53E-18	0.0266
ZNF714	4.2606	2.14E-18	0.6874
LGR5	7.8707	2.81E-18	2.1117
SCN1A	9.3570	2.95E-18	0.5056
ZNF677	9.1889	3.86E-18	0.4288
NACAD	10.5509	4.41E-18	0.3061
ZNF528	10.1801	8.36E-18	0.0845
CTNND2	9.1880	1.14E-17	0.2969
ZNF649	4.2395	1.14E-17	0.6281
C1orf106	10.5447	1.19E-17	1.2267
ZNF493	9.7959	1.61E-17	0.5381
GAB1	3.0072	1.63E-17	0.4226
LINGO1	7.2119	1.66E-17	1.5168
TNFRSF21	3.4698	2.52E-17	0.2299
TMEM100	10.9300	2.82E-17	1.2110
GRIA2	6.4154	3.08E-17	1.0503
RNF165	7.2100	3.16E-17	1.8203
RNF182	10.3365	3.24E-17	0.5468
LRRC4	10.7217	3.37E-17	1.2425
NR2F1	4.2153	3.50E-17	0.0510
KCNK2	7.2431	3.93E-17	0.8776
NPAS3	7.2149	5.70E-17	0.1145
NDRG4	6.0767	6.00E-17	0.3645
PHYHIPL	10.8925	6.17E-17	1.2116
IL17D	4.9250	7.23E-17	0.0384
ZNF90	10.2101	8.19E-17	0.8103
NCALD	8.9305	9.75E-17	0.8427
HOXD10	6.5274	1.09E-16	0.4700
AC018647.3	10.6295	1.29E-16	0.4354
EHD3	10.1748	1.42E-16	0.5472
L3MBTL1	8.2847	1.44E-16	0.8110
AUTS2	6.3802	1.64E-16	0.1232
NLGN1	5.0599	1.80E-16	0.2593
HAGLR	10.3761	1.88E-16	1.2307
CLIP3	5.9451	1.96E-16	0.5690

MARVELD2	6.1006	1.88E-12	0.79659	CSGALNACT1	8.2324	2.10E-16	0.1920
NPAS1	4.6011	1.93E-12	0.08459	ZNF682	10.4208	3.19E-16	0.6207
S100A11	7.0098	1.93E-12	1.20691	NTRK3	9.1973	3.19E-16	0.3906
RP11-221N13.3	5.2213	1.97E-12	2.31656	EFNB3	8.5914	3.21E-16	0.7890
SPHK1	4.4370	2.09E-12	0.63231	ZNF660	10.0700	3.32E-16	0.2745
ARMC4	5.5312	2.15E-12	1.15290	RAB33A	8.5766	3.40E-16	1.6957
AP001065.15	8.3707	2.71E-12	0.52286	P2RX7	9.1876	3.74E-16	0.9403
CASC8	7.3916	2.71E-12	1.33385	SOX21-AS1	10.6784	4.24E-16	0.3024
RSPH9	6.5050	2.87E-12	0.15116	PPFIBP2	3.6217	4.66E-16	0.0988
STK26	6.4454	3.42E-12	0.69332	UGT2B7	8.9569	6.58E-16	4.1225
RBP7	7.1129	3.84E-12	0.15239	ST8SIA5	7.8498	7.58E-16	0.9420
KC6	8.7319	4.18E-12	1.18096	ZNF329	6.0038	9.07E-16	0.2118
SP100	5.2944	4.33E-12	1.00819	SDHAP3	3.9054	9.40E-16	0.2353
PERP	4.8009	4.47E-12	1.08670	GRIA4	11.2092	1.27E-15	0.9964
VSTM1	8.8560	4.48E-12	1.60418	ZNF829	9.8596	1.31E-15	0.3077
DAW1	9.2501	4.94E-12	0.25744	SNX32	7.3539	1.40E-15	0.9148
SLC27A6	8.4641	5.81E-12	1.35163	COL9A2	4.6757	1.53E-15	0.3980
RP11-879F14.2	7.1677	5.85E-12	0.55834	ZNF626	10.1387	1.63E-15	0.0446
FMNL1	5.7755	6.05E-12	0.93437	ZNF513	4.8104	2.35E-15	0.2064
GFI1	3.3699	6.06E-12	0.65206	C2orf72	3.5854	2.43E-15	0.3489
FOXL1	8.9092	7.98E-12	0.30968	TUBA1A	3.4934	2.64E-15	0.7098
LINC01615	8.2440	1.01E-11	1.64958	TIMP4	10.3583	2.66E-15	0.7220
IL18R1	5.7566	1.32E-11	1.39850	LRRK2	3.4876	2.69E-15	0.9987
PIWIL4	3.8146	1.37E-11	0.38059	CDIP1	4.5098	2.72E-15	0.0397
SLFN13	8.5979	1.62E-11	0.36084	RP11-210M15.	7.9611	2.74E-15	0.0025
RAB32	3.7839	1.68E-11	0.75663	PNMA2	9.7049	2.96E-15	0.0734
SH3RF2	6.5459	1.76E-11	0.91868	MAP3K1	4.2020	3.36E-15	0.4219
ITGBL1	6.9545	1.95E-11	1.50077	RRN3P1	10.0593	3.80E-15	0.3782
CYP1B1	3.1027	2.12E-11	2.70550	MEG3	9.8841	3.89E-15	0.4281
MOXD1	6.0168	2.41E-11	1.93569	DSEL	4.7687	3.96E-15	0.6453
PAX2	8.6469	2.45E-11	1.13829	AFG3L1P	3.0020	4.39E-15	0.2170
RAD21L1	6.8840	2.52E-11	0.12328	RAPGEF4	6.6078	4.48E-15	1.0553
TBX1	6.2958	2.57E-11	0.66479	RP11-161M6.2	9.8620	4.74E-15	2.0987
CTD-2054N24.	8.4811	2.59E-11	0.84929	FAM131B	8.2378	5.08E-15	0.6709
STOM	3.0499	2.64E-11	1.13344	EN2	10.4511	5.51E-15	0.2974
RP11-1149O23	8.0683	3.64E-11	1.04664	ZNF585A	9.4276	5.98E-15	0.2671
LINC00704	8.7894	4.84E-11	0.52666	NCAM1	7.3265	6.63E-15	1.0888
TREM1	8.8492	4.89E-11	1.76455	CHST11	3.1837	7.04E-15	0.3314
TMEM56	4.6326	4.89E-11	0.36539	LBH	10.0848	8.24E-15	0.2082
LAMA3	4.5316	5.51E-11	1.18632	PHLPP1	3.8485	8.98E-15	0.6619
STEAP1	6.6997	7.55E-11	1.36881	D2HGDH	10.1262	9.56E-15	0.2232
FENDRR	8.8712	8.73E-11	0.35790	DACT1	9.5438	9.59E-15	0.0176
SLC44A3	4.8017	1.03E-10	0.45702	GPM6A	4.8880	9.76E-15	0.6292
DLX4	5.6057	1.04E-10	0.32022	SLC16A9	10.2200	1.05E-14	0.2550
TRPM2	8.1273	1.12E-10	0.93405	ZSCAN16	9.6619	1.36E-14	0.5897
PLAGL1	6.9078	1.13E-10	1.32230	ZNF502	7.8406	1.38E-14	0.1671
PRDM6	5.3578	1.15E-10	0.03644	BMP7	9.0016	1.76E-14	0.7346
IL22RA1	6.2705	1.25E-10	0.65651	RLBP1	9.3865	1.78E-14	0.7819
ADAMTS16	7.9713	1.27E-10	0.46750	CADM2	5.9151	1.94E-14	1.2857

HTR4	8.2785	1.62E-10	0.35549	ZNF528-AS1	10.3361	2.16E-14	0.6251
RP11-66B24.4	8.4112	1.78E-10	0.40107	PROX1	9.5589	2.47E-14	0.4543
SQRDL	3.7841	1.95E-10	0.97608	COL9A1	10.6859	2.62E-14	1.5901
PARM1	5.7665	1.96E-10	0.71548	CTNNA3	9.2740	2.75E-14	1.3380
PAWR	5.6944	2.04E-10	0.08806	ID2	3.7187	2.82E-14	0.0559
PITPNM1	3.3425	2.11E-10	0.33007	ZNF501	9.1241	3.11E-14	0.3065
MET	4.9643	2.68E-10	1.08407	GRID1	9.1471	3.15E-14	0.3637
HLX	8.3262	2.94E-10	0.97581	RP11-275H4.1	9.0764	3.29E-14	1.5444
NOX3	8.9502	3.13E-10	0.03972	RHBDL3	9.3947	4.78E-14	1.2899
HPSE	3.8411	3.35E-10	0.28960	ZKSCAN7	9.5519	4.80E-14	0.5157
TCIRG1	5.5480	3.44E-10	1.17082	SARM1	4.8544	5.29E-14	0.5753
CD109	5.1160	3.98E-10	1.47040	GAREM2	9.5272	6.11E-14	1.1055
SAMD3	8.2413	4.11E-10	1.12324	VSTM2B	4.1424	7.38E-14	2.1009
NAGS	4.3215	4.13E-10	0.53373	PCDH7	5.0165	7.83E-14	0.8293
PKP3	5.6250	4.20E-10	0.14105	ADCY1	9.9584	1.06E-13	0.9362
LPIN3	5.5421	4.30E-10	0.98350	TMEM169	3.7359	1.21E-13	1.1852
GADD45B	3.5069	4.44E-10	0.81728	LRRTM3	10.1630	1.37E-13	0.6713
CDK15	8.4831	4.56E-10	0.53994	TRIM71	9.5583	1.45E-13	2.0498
MAFK	3.0481	4.79E-10	0.43280	SPSB4	9.1080	1.52E-13	1.2024
RP4-756H11.3	3.0534	4.84E-10	0.17550	DGKG	5.9959	1.70E-13	0.4023
ICAM1	4.5536	4.92E-10	1.42324	ZNF793	7.1076	1.97E-13	0.4239
P4HA3	6.0223	5.15E-10	1.61087	XYLT1	6.4044	2.29E-13	0.9167
DUSP1	3.2867	5.55E-10	0.96551	REEP2	9.5875	2.44E-13	0.0539
INHBE	5.4392	5.77E-10	0.26902	KCNH2	9.0491	2.81E-13	0.4182
STXBP5-AS1	4.8328	6.31E-10	0.12152	SESN3	4.6622	2.89E-13	0.0207
MAGEA2B	8.0735	6.56E-10	0.10503	EN1	9.4700	2.89E-13	0.3339
SLC9A2	5.2851	7.06E-10	0.14593	ZNF568	4.3216	3.53E-13	0.0487
ATF7IP2	7.7065	8.31E-10	0.34324	RASL10B	8.7738	3.54E-13	0.9736
BNC2	6.4712	8.34E-10	1.85641	SRSF12	8.9474	4.03E-13	0.8772
SLC39A4	7.9315	9.21E-10	0.67107	DCHS1	7.7898	4.62E-13	0.3531
MICA	4.0906	9.23E-10	0.91722	SPATA6	4.4386	4.74E-13	0.0022
SHC1	3.0499	9.26E-10	1.32064	BEND5	8.9503	5.12E-13	0.3565
EFCAB10	6.8165	9.46E-10	0.38571	LRRC37A2	5.2967	5.33E-13	0.3784
ACSM3	3.8292	9.62E-10	0.44102	NKX2-2	9.6302	5.42E-13	1.5421
IL1R1	5.9772	1.01E-09	1.43425	SOBP	3.0616	5.54E-13	0.2811
KDELR3	3.0785	1.03E-09	1.84643	LINC01896	8.7173	5.77E-13	1.0336
KSR2	7.9345	1.23E-09	0.08929	HILS1	9.0819	5.86E-13	1.1825
ADAP2	3.3601	1.37E-09	0.86774	AMPH	8.8405	7.46E-13	0.6070
TUBA1C	3.4947	1.46E-09	0.66020	TMEM200C	8.8834	8.01E-13	0.2599
MYO1D	6.0931	1.52E-09	0.21667	HAND2	9.2681	8.19E-13	1.6791
PAPLN	5.0833	1.60E-09	1.11819	CACNG4	9.3302	9.32E-13	1.3925
CALB2	8.8031	1.76E-09	0.37906	CA14	9.0907	1.00E-12	0.5940
TMEM74	6.5994	2.05E-09	0.12750	CSMD2	8.9198	1.01E-12	0.8205
CCDC69	4.4912	2.15E-09	0.78682	CAND2	8.9706	1.07E-12	0.3045
SUN3	8.8427	2.25E-09	0.82984	DAAM2	9.2434	1.12E-12	1.1223
GATA6	7.9604	2.45E-09	1.65716	NKAIN1	6.8598	1.16E-12	2.5212
GLT8D2	3.5286	2.68E-09	1.15556	CASC15	9.3361	1.20E-12	0.9993
CD274	3.4145	2.86E-09	1.48593	PLPPR5	8.8828	1.21E-12	1.1660
OSCAR	6.0708	2.92E-09	1.11755	RP3-525N10.2	6.6601	1.23E-12	0.9506

COL6A4P1	7.7765	3.07E-09	0.19604	ZNF730	8.7397	1.60E-12	1.1191
ITGB2	8.4340	3.31E-09	1.06362	PTPRD	6.1382	1.92E-12	0.6246
TSPEAR-AS2	7.8613	4.06E-09	0.36782	ATP1A2	8.8006	2.08E-12	0.2700
TRABD2A	4.5479	4.49E-09	0.75556	SPOCK1	9.2591	2.33E-12	0.3359
ARHGAP40	6.4840	4.53E-09	0.84930	PLXNB1	3.3105	2.56E-12	0.6963
PPP1R13L	4.3014	4.60E-09	0.88316	LURAP1	8.9325	3.59E-12	0.0707
LINC00941	4.9017	5.11E-09	2.07034	BEX4	10.6884	3.83E-12	0.3494
PTGER4	7.7854	5.29E-09	1.41492	ZNF610	5.5794	4.45E-12	0.5136
TSPEAR-AS1	7.7208	5.64E-09	0.72689	MOCS1	9.4612	4.60E-12	0.0860
RP11-359K18.4	3.5296	5.78E-09	0.38844	ACSBG1	8.6379	5.29E-12	0.2461
OTX1	3.2848	6.08E-09	0.74048	SLC8A3	8.9865	6.19E-12	1.9222
HOXB6	7.3062	6.46E-09	2.46748	TGFA	6.0371	7.26E-12	0.3010
EFHD2	3.1133	6.64E-09	0.21980	TENM1	7.6019	7.83E-12	0.0166
RP1-102E24.8	7.9476	6.95E-09	0.69862	PDZRN3	9.9060	8.74E-12	0.3727
ADAMTSL4	4.7460	7.04E-09	0.97874	RP11-629G13.	8.3794	1.00E-11	0.2281
CXCL3	7.7159	7.11E-09	2.25093	ZNF253	3.3466	1.03E-11	0.5556
TTC39B	3.3619	7.13E-09	0.40113	PIANP	10.2584	1.06E-11	0.9088
LBX2-AS1	4.2131	8.53E-09	1.01622	TAS2R14	3.1716	1.10E-11	0.2839
DNAAF3	5.3034	8.56E-09	1.17521	APCDD1	9.5625	1.23E-11	0.4033
TMEM171	5.9664	8.97E-09	0.42340	ARHGAP42	4.0161	1.28E-11	0.2582
CLCF1	3.8146	1.05E-08	1.92885	MARC1	4.3412	1.41E-11	0.0629
EML2-AS1	4.3363	1.09E-08	0.39496	FADS2	3.0832	1.47E-11	0.6707
RP11-809O17.	4.2873	1.19E-08	0.54437	AC108142.1	8.9199	1.48E-11	0.3438
RHBDF2	4.0817	1.30E-08	0.93501	RGR	8.6606	1.72E-11	1.7076
PLEKHF1	3.8146	1.30E-08	0.87114	LHFPL3	8.9140	1.74E-11	1.3649
PDGFB	8.4485	1.33E-08	0.09174	SCUBE2	5.4968	1.86E-11	0.1648
DNAH5	4.5472	1.38E-08	0.87709	LMO7	3.4354	1.95E-11	0.1850
RP11-328K4.1	7.6249	1.43E-08	1.42516	SORBS2	7.4168	2.11E-11	0.0332
XAGE1B	7.2225	1.47E-08	0.48974	CECR6	4.5632	2.13E-11	0.9829
NNMT	6.4328	1.57E-08	1.40321	PGAP1	3.7885	2.33E-11	0.5285
NUP62CL	4.7448	1.73E-08	0.56300	DCC	9.7748	2.74E-11	1.7475
EML6	3.4257	1.85E-08	0.00246	WLS	3.1537	2.75E-11	0.0592
AREG	7.6153	2.07E-08	2.10910	PKNOX2	4.0706	2.92E-11	0.6154
ROR2	6.5743	2.26E-08	1.68472	KIF5A	8.6173	2.92E-11	1.9538
IL15RA	4.4728	2.73E-08	0.93722	RIMS4	9.3407	3.16E-11	1.0504
FXYD5	6.5201	3.17E-08	0.72357	LINC01268	8.6508	3.20E-11	1.0059
RHOV	3.6364	3.79E-08	0.02555	RP11-436K8.1	9.1534	3.66E-11	0.9481
HPCAL1	3.3483	3.99E-08	0.25860	NDRG2	6.3814	4.04E-11	0.7366
GPRC5C	6.5335	4.39E-08	0.07639	ZNF572	8.6191	4.10E-11	0.3987
RSPO4	6.4663	4.69E-08	0.06196	CRB1	5.5053	4.53E-11	1.1383
IL15	4.6191	4.74E-08	1.30654	PRKAR1B	3.2596	4.53E-11	0.3520
IFNLR1	7.0706	4.75E-08	0.15995	FAM212B	5.5954	4.87E-11	0.8427
LGALS2	7.3620	5.03E-08	0.74371	CDH19	9.5057	5.31E-11	1.0267
CDH23	6.1274	5.32E-08	0.19479	TRIM60P18	8.4817	5.59E-11	0.4083
SPEF2	3.7867	5.41E-08	0.61324	PLEKHB1	3.0276	5.60E-11	0.3401
SLC46A3	4.1293	5.57E-08	0.53603	FAXC	9.1687	5.80E-11	0.8637
STAT6	3.1710	6.07E-08	0.92170	TAGLN3	4.5196	6.20E-11	1.3246
GLRX	3.5698	6.16E-08	0.90019	DMRT2	9.0008	6.27E-11	2.1949
SMAGP	4.5082	6.23E-08	1.07167	CYP4F29P	5.6892	6.45E-11	0.5092

OPLAH	5.8987	6.56E-08	0.88836	FAT3	9.3400	6.50E-11	0.4182
MID2	3.7419	6.95E-08	0.18382	CADM4	3.9640	6.66E-11	0.4446
CTD-2207P18.	7.5549	6.98E-08	1.20368	CDH7	9.8256	6.92E-11	2.1405
UPK1A-AS1	7.0642	6.99E-08	0.34326	ZNF704	3.5358	7.48E-11	0.7691
CTD-2587H24.	7.5136	7.00E-08	0.81242	ASTN1	8.6825	7.52E-11	0.3163
ITPR3	3.8592	7.40E-08	1.17806	GTF2IP4	3.2013	7.59E-11	0.3442
ATP8B3	4.1052	7.62E-08	0.65853	CTD-2561J22.	8.1815	7.99E-11	0.3625
TPRG1	4.3630	7.82E-08	0.93908	ADGRB3	4.8490	8.56E-11	0.8661
FLNC	5.1767	7.89E-08	0.06530	CRYAB	9.0296	9.05E-11	1.1423
CTD-2066L21.3	6.2857	7.90E-08	0.45914	GTF2IP1	3.2675	9.27E-11	0.1797
LINC00623	3.5339	9.56E-08	0.45178	TMPRSS5	4.6847	1.08E-10	1.0040
RP11-89C3.4	7.9437	1.01E-07	0.46369	HOXD9	6.4103	1.10E-10	0.9914
MSR1	7.5413	1.01E-07	1.11755	KIF1A	7.6386	1.19E-10	0.9688
CYB561	3.0926	1.23E-07	0.67475	RP5-882C2.2	3.1466	1.50E-10	0.2545
DNM1P46	6.0524	1.31E-07	0.81795	DPY19L2P2	4.9521	1.52E-10	0.2943
LINC01162	7.2061	1.50E-07	0.16915	KCND2	8.8775	1.58E-10	1.3305
RIN1	4.5521	1.54E-07	1.30710	MIAT	7.6341	1.63E-10	1.3584
LINC00702	6.8199	1.58E-07	0.66971	C14orf37	3.9484	1.84E-10	0.1785
CTD-2311M21.	7.2206	1.59E-07	1.16875	PELI2	6.9494	1.84E-10	0.2385
RNF212	6.8037	1.85E-07	2.20456	HIST1H2BN	3.7792	1.96E-10	0.0916
CTD-2303H24.	4.7042	1.86E-07	0.37253	ZNF844	6.0859	2.68E-10	0.2944
AADAC	7.3153	1.89E-07	1.18256	ZNF66	8.1794	2.92E-10	0.8251
SMCO4	4.1268	1.96E-07	0.47627	ELAVL3	9.3812	3.04E-10	1.3824
EOMES	7.1924	2.01E-07	1.46346	EPHB3	5.1287	3.16E-10	0.6567
PCED1B	4.2816	2.03E-07	0.94027	XKR5	8.0891	3.27E-10	0.3656
ATP2A3	4.0399	2.04E-07	0.81821	BEX1	8.9516	3.33E-10	0.8387
CXCL2	6.8877	2.13E-07	1.27004	AC114730.3	8.5402	3.82E-10	1.4959
FBXO6	3.1018	2.16E-07	0.35175	ZBTB8B	8.1771	3.96E-10	1.2613
EGFR-AS1	7.4675	2.19E-07	0.50939	RP11-444D3.1	8.2187	4.12E-10	0.5984
CXCL1	6.9777	2.35E-07	1.70101	MOB3B	3.7949	4.68E-10	1.1832
GLDN	4.7608	2.45E-07	0.40223	RGS7BP	8.7483	5.58E-10	1.0360
FAM26E	5.6769	2.53E-07	0.44452	ST8SIA1	8.8617	5.73E-10	0.0034
CLMP	3.0302	2.85E-07	0.85470	CXXC4	4.4966	6.10E-10	1.2176
SLFN5	4.7003	3.08E-07	0.31697	CTC-559E9.5	8.3450	6.27E-10	0.2682
NKX2-5	7.3178	3.14E-07	0.45745	PCDHB5	8.0800	6.87E-10	0.1035
CGB7	7.2236	3.32E-07	0.53395	NT5C3A	3.0280	7.01E-10	0.0929
RAP1GAP2	4.2926	4.12E-07	0.27833	RNF150	3.9597	7.11E-10	0.7011
CASZ1	3.6930	4.28E-07	0.44149	XKR6	8.0416	7.17E-10	0.4426
MICE	3.0837	4.31E-07	0.57472	DYRK1B	3.2355	7.21E-10	0.2726
SLC44A3-AS1	5.8306	4.44E-07	0.51389	FAM198A	8.4606	7.23E-10	0.2534
GSDMD	3.6357	4.65E-07	1.13360	C8orf88	4.0395	1.03E-09	0.0678
KCNK13	4.9745	5.17E-07	0.53223	SLC44A5	8.0880	1.24E-09	0.6897
PRR16	5.6007	5.50E-07	0.32534	TMEM198	4.7412	1.24E-09	0.5905
RP11-195E2.1	7.0573	5.59E-07	0.00305	FREM1	6.7844	1.45E-09	0.5807
TUBB6	3.2742	6.31E-07	0.62360	CTC-260E6.6	8.0306	1.49E-09	1.0055
BIK	5.9718	6.31E-07	0.49101	HIP1	3.5911	1.55E-09	0.4058
SBSN	5.8333	6.48E-07	1.36809	GATS	3.2462	1.80E-09	0.7425
MICB	4.3168	6.70E-07	0.45382	ANKRD44	3.1967	1.94E-09	0.1252
OSMR-AS1	4.8488	7.28E-07	0.71496	GPC2	4.0585	2.00E-09	2.1590

SLFN12L	6.5207	8.22E-07	0.54868	FRAS1	8.2522	2.05E-09	0.4777
TFAP2C	4.7326	9.35E-07	1.64091	ZNF454	6.4384	2.17E-09	0.5752
SIGIRR	4.5890	9.60E-07	0.33416	SLC18A1	8.0049	2.19E-09	1.8850
AC156455.1	4.6873	1.04E-06	0.09661	AC092835.2	5.1002	2.35E-09	0.5628
VAV1	6.4854	1.07E-06	0.97678	DUSP26	8.5256	2.95E-09	1.5113
SP8	6.6621	1.07E-06	1.09518	FAM212B-AS1	6.8176	3.47E-09	1.1393
MT2A	3.6851	1.13E-06	1.02600	ZNF724	7.4443	3.66E-09	0.7671
NOTUM	5.0570	1.15E-06	0.23494	NGFR	6.4623	4.07E-09	0.2548
PER1	3.0986	1.20E-06	0.05608	TMEM179	9.2313	4.07E-09	0.8530
PLEKHN1	5.3835	1.28E-06	0.95595	GTF2IRD2B	3.7198	4.12E-09	0.1266
PLAG1	3.4998	1.37E-06	0.28752	ABAT	4.6746	4.30E-09	0.5108
LINC00659	6.3653	1.52E-06	0.36044	ATL1	3.0038	4.39E-09	0.5209
CLDN1	4.9244	1.56E-06	0.74963	C1orf61	6.8222	4.91E-09	1.1464
NKD2	4.5440	1.60E-06	0.15834	TMEM108	4.2999	4.96E-09	0.7762
RP13-463N16.(	7.1209	1.64E-06	2.53901	CALCRL	5.4109	5.14E-09	0.4515
CSAG4	6.8927	1.65E-06	0.23911	AMER2	8.8626	5.22E-09	0.7588
CFH	4.4349	1.65E-06	1.99961	LINC02199	7.5342	5.40E-09	1.4736
GPR39	5.6888	1.65E-06	1.29320	TECTA	7.7439	5.96E-09	0.6624
ICOSLG	4.6778	1.73E-06	0.79175	PLXNA4	8.4802	6.30E-09	0.3292
DHRS3	6.6768	1.84E-06	0.78840	ZNF781	7.8189	6.49E-09	0.6775
PLBD1-AS1	6.9117	1.87E-06	0.80311	NEU4	8.1025	7.29E-09	2.0569
NUDT8	3.0040	1.91E-06	0.25522	FGF13	7.4598	7.48E-09	0.6600
COL17A1	6.7090	2.34E-06	0.95513	LINC01550	8.0856	7.55E-09	0.1503
LINC01036	6.8181	2.35E-06	0.04642	RNF157	3.8457	8.58E-09	0.6021
LINC01273	5.6801	2.37E-06	0.63868	HFM1	3.7073	9.08E-09	0.8667
PRSS16	4.8463	2.66E-06	0.33127	YPEL3	5.0035	9.09E-09	0.0207
CGB2	6.7473	2.68E-06	0.09861	ZNF582-AS1	7.9035	9.86E-09	0.0312
RP11-402J6.1	6.7575	2.72E-06	0.00690	ARPP21	8.0378	1.10E-08	1.1399
RP11-519M16.	6.6977	2.85E-06	0.10229	RP11-255H23.	6.8037	1.10E-08	0.1298
LINC00460	3.7294	3.14E-06	2.34298	HOXA7	6.6787	1.11E-08	0.6904
RP11-49I11.1	3.6175	3.44E-06	1.23603	LINC01535	7.5876	1.12E-08	0.4357
LRRC73	3.6485	3.74E-06	0.07918	HYDIN2	7.6849	1.26E-08	0.7603
P2RY2	4.9562	3.85E-06	0.76596	NOVA1	3.1492	1.31E-08	0.8639
TSPAN2	6.9370	3.93E-06	0.86914	FBN3	9.0764	1.35E-08	2.0368
RP11-326A19.4	4.3692	4.03E-06	0.27005	HOXD8	8.4257	1.39E-08	1.3032
WNT2B	3.0596	4.99E-06	0.03846	NALCN	7.4851	1.39E-08	0.8309
RP11-403I13.5	6.6077	5.12E-06	0.73730	EMILIN3	8.2956	1.65E-08	0.5605
RP11-802E16.3	3.8291	5.16E-06	1.32311	LINC00403	7.6991	1.72E-08	1.1060
HOXB4	4.0266	5.22E-06	0.77482	RIC3	7.7844	1.90E-08	0.6547
LBX2	5.4279	5.25E-06	0.37418	KCND3	8.7450	2.18E-08	0.5605
AKAP12	3.5825	5.60E-06	0.55671	TRIM9	3.5552	2.26E-08	0.3551
RP1-28O10.1	6.7406	5.65E-06	0.78494	C1orf56	5.0005	2.65E-08	0.0309
ZNF280A	6.2541	6.03E-06	0.50701	HOXD11	6.6502	2.73E-08	0.3152
LINC01037	6.2703	6.20E-06	0.26267	CTD-2291D10	7.6979	2.78E-08	0.9282
RP11-64D22.5	6.6702	6.25E-06	0.86965	RP11-448P19.	8.4664	2.88E-08	1.0048
PTHLH	4.0268	6.74E-06	0.65102	DAB1	8.1110	3.31E-08	0.9916
LINC01703	3.0634	7.75E-06	0.32373	KLRC4-KLRK1	7.8278	3.93E-08	2.4113
LINC01186	6.3373	7.82E-06	0.77576	RP11-472N13.	7.8359	4.56E-08	0.4240
PRKAG2	3.1083	7.97E-06	0.08830	LINC01105	8.2108	4.78E-08	1.7989

HSD17B14	3.7673	8.33E-06	0.18341	SNTG1	8.8614	5.27E-08	0.7946
DNAH17-AS1	6.6714	8.96E-06	0.02288	PTPRD-AS1	7.5183	5.39E-08	0.6054
AADACP1	7.0601	9.01E-06	0.78872	CALM1	3.0456	5.56E-08	0.0535
GAPLINC	6.4889	9.04E-06	0.91808	MYO16	8.7609	5.90E-08	0.8936
LL22NC03-86G	4.7270	9.25E-06	0.06886	KCNAB3	3.5173	6.77E-08	0.5775
AC073130.3	6.4422	9.95E-06	0.41210	SYT11	3.3105	6.79E-08	0.3429
PCDHB6	4.2842	1.01E-05	0.02358	TNS3	5.1132	7.14E-08	0.1412
SYNGR2	3.2551	1.09E-05	0.66154	NEB	5.3591	7.60E-08	0.1503
GATA3	4.5874	1.10E-05	1.71327	TSPAN11	8.6156	7.61E-08	0.2543
CH17-472G23.	3.3741	1.11E-05	0.22474	DPP6	7.4481	7.77E-08	0.4283
RP11-54A9.1	7.4821	1.12E-05	0.84593	RPE65	7.0326	8.47E-08	0.0959
RP5-1139I1.1	6.9312	1.15E-05	0.87108	RAMP2-AS1	8.6078	8.64E-08	0.8693
ADAMTS2	6.8356	1.19E-05	1.59596	CNTFR	7.5948	1.15E-07	1.5436
MSC	6.4187	1.20E-05	1.33238	DDN	6.4851	1.16E-07	0.5959
TMEM244	6.4465	1.22E-05	0.05625	FHIT	3.5005	1.19E-07	0.3433
ARHGAP29	4.9878	1.26E-05	0.96156	DSCAML1	7.2674	1.21E-07	2.1167
RAET1E	5.3568	1.26E-05	0.51016	ZNF117	4.1204	1.28E-07	0.2252
RP11-162D16.	6.4031	1.33E-05	0.17109	CLEC4F	5.4765	1.28E-07	0.9937
IGSF1	5.1187	1.34E-05	0.14123	ID3	3.4081	1.45E-07	0.0594
BTNL8	7.5868	1.36E-05	0.63913	LINC01415	8.3713	1.48E-07	0.2638
RP11-60A8.1	7.0596	1.42E-05	0.51943	DACT3	4.7824	1.49E-07	0.6125
SLC22A5	3.3562	1.46E-05	0.32027	TOX3	8.5131	1.50E-07	1.7679
MSL3P1	6.3580	1.60E-05	0.21881	ZNF429	4.2291	1.58E-07	0.3861
CD7	7.0995	1.67E-05	1.06078	PCDHB12	4.4453	1.60E-07	0.1313
CFD	4.5086	1.67E-05	0.76351	RAMP2	7.9462	1.62E-07	0.3166
RP11-134G8.5	3.1272	1.71E-05	0.41683	CRABP1	8.2005	1.72E-07	0.9403
NEBL	4.5224	1.73E-05	0.39882	ADCYAP1R1	8.5689	1.84E-07	0.3659
ITGB4	3.6401	1.84E-05	0.93519	HAND2-AS1	7.8711	1.90E-07	2.2214
NOXA1	4.5907	2.06E-05	0.40712	CHRM1	7.2400	1.90E-07	1.3130
EGFL7	3.3854	2.19E-05	0.07168	TSPAN12	3.7919	2.02E-07	0.3340
RP13-977J11.2	4.1660	2.22E-05	0.10188	LRRC4C	4.8418	2.19E-07	0.3902
XKR9	3.5744	2.41E-05	0.03847	SNN	4.2931	2.19E-07	0.3341
SENCR	6.0854	2.56E-05	0.54951	CTC-543D15.8	7.5218	2.35E-07	0.3541
GDPD5	3.0801	2.81E-05	0.04114	ZNF790-AS1	7.2818	2.38E-07	0.2634
EPS8L2	3.0850	2.89E-05	0.56857	GPR156	5.1964	2.51E-07	0.4145
AK7	3.8436	2.99E-05	1.62802	GPR155	3.9442	2.57E-07	0.2467
HIF1A-AS2	6.4048	3.20E-05	0.93856	SCN3A	6.1302	2.60E-07	1.3950
LAMP3	4.3737	3.20E-05	0.54071	CTD-2017D11	3.9601	2.97E-07	0.3026
RP11-527N22.	5.3391	3.42E-05	1.86058	MAP1A	4.1960	3.39E-07	0.2694
CTC-378H22.1	6.5346	3.76E-05	0.77472	LRP4	3.7696	3.49E-07	0.5343
SCNN1A	6.3063	3.76E-05	1.06684	RP9P	3.2619	3.67E-07	0.1943
CT45A1	6.2491	3.82E-05	0.04096	PREX1	3.1199	3.81E-07	0.1445
RP11-686D22.	6.1985	3.99E-05	0.63035	ELAVL4	7.9681	4.13E-07	1.8819
SLC22A4	3.3939	4.12E-05	0.88710	SETBP1	7.6232	4.36E-07	0.1660
LIF	3.3668	4.20E-05	2.20341	STMN3	4.7661	4.59E-07	0.3862
CTF1	3.8752	4.31E-05	0.90155	STXBP6	3.5376	5.23E-07	0.5613
MAGEA2	6.2260	4.32E-05	0.13708	SRGAP3	3.7501	5.48E-07	0.6684
BIRC3	4.0585	4.34E-05	1.60311	DGKB	7.6371	5.55E-07	1.1143
SERPINA1	5.9511	4.60E-05	1.55661	LINC00391	7.1464	5.71E-07	0.0841

AC012485.2	5.9237	4.79E-05	0.17227	AC010729.1	7.2602	5.96E-07	1.2802
AC007879.5	3.0746	4.97E-05	0.64791	CDHR1	7.4998	6.31E-07	1.4757
GPR18	6.1081	5.76E-05	0.86830	RP11-617F23.	3.1248	6.90E-07	0.1808
CEP126	4.7652	5.93E-05	0.24304	KLRC4	7.7552	7.61E-07	2.6217
SLC22A31	6.4044	6.21E-05	0.34994	GTF2IRD2	3.8028	7.78E-07	0.1810
TESC	4.0349	6.24E-05	0.14992	C6orf52	3.7550	8.14E-07	0.3741
TRIM6	3.3420	6.33E-05	0.83816	RP11-480I12.9	3.3623	8.24E-07	0.4134
MESP2	6.0640	6.39E-05	0.36358	ZNF726	5.1484	8.73E-07	1.0421
FHL2	3.3874	7.06E-05	1.20276	RP11-611L7.2	4.3048	8.76E-07	0.4454
AC073130.1	6.1266	7.07E-05	1.04707	PNMAL2	7.4186	9.58E-07	0.4397
RP11-280G9.1	6.1710	7.41E-05	1.05615	RP11-966I7.2	7.1963	1.09E-06	0.2390
CYB5R2	4.6067	7.55E-05	0.17376	ZNF157	5.3077	1.20E-06	0.9451
KLC3	6.1650	8.13E-05	0.49981	RP11-25K19.1	5.4054	1.31E-06	0.5767
SIRPB1	4.6753	8.27E-05	0.82802	FAM69C	7.0490	1.36E-06	0.5424
MYOSLID	6.2516	8.30E-05	2.13958	RP11-4O1.2	4.6150	1.45E-06	0.0759
RP11-818F20.5	5.8374	8.44E-05	1.07220	GS1-24F4.2	6.7798	1.45E-06	0.9853
APOL1	4.3252	8.46E-05	1.32488	AP002954.3	3.3516	1.87E-06	0.7650
SMPDL3B	4.6164	8.74E-05	0.37931	H19	5.5332	2.07E-06	1.7596
DUSP5	3.6861	8.81E-05	0.51111	PHACTR1	6.1827	2.39E-06	0.3109
CES3	3.0173	8.98E-05	0.89508	PRSS35	7.5924	2.44E-06	0.3953
NTRK1	3.8647	9.49E-05	0.83309	LY6G5C	4.0832	2.57E-06	0.1481
KRT34	6.1751	9.66E-05	0.00412	SORCS3	7.4754	2.64E-06	1.0075
RP11-336A10.5	6.0364	9.75E-05	0.52207	PNMA6A	6.6648	2.84E-06	0.7500
MICALCL	5.0957	0.0001	0.59789	KLRC3	6.6607	3.13E-06	2.2619
PKD1L2	4.0055	0.0001	0.62697	RP11-126K1.6	3.7305	3.98E-06	0.5977
C10orf11	3.2011	0.0001	0.77456	PRR18	6.2981	4.25E-06	0.4771
ROBO4	5.2538	0.0001	0.12160	CD207	5.1787	4.26E-06	0.1910
ARHGAP45	3.8997	0.0001	0.65857	ZDHHC22	5.5338	4.32E-06	2.1002
RP11-61A14.1	5.6362	0.0001	1.12198	CBSL	3.9148	4.45E-06	0.7452
TMEM200B	3.9049	0.0001	1.14820	CHADL	6.8903	4.81E-06	0.8103
TMEM40	6.6360	0.0001	1.03200	AC078842.3	6.3127	5.01E-06	0.0614
LRRC2-AS1	6.0330	0.0001	1.11174	KCNN2	6.8005	5.19E-06	0.2585
RFTN1	4.4093	0.0001	0.80631	PLXNB3	3.4076	5.28E-06	0.6982
NBL1	3.3932	0.0001	0.56873	bP-21264C1.1	6.3355	5.33E-06	0.5720
AXDND1	3.4909	0.0001	0.29095	LA16c-306E5.2	6.6100	5.38E-06	0.0146
LRRC2	4.2500	0.0001	1.51988	SDPR	7.5806	5.40E-06	0.0461
DEPTOR	3.9260	0.0002	0.56669	MYCN	7.6341	5.43E-06	1.6800
RP11-745L13.2	5.9655	0.0002	1.38492	AC064875.2	8.2687	5.72E-06	0.3105
EMBP1	4.8811	0.0002	0.82084	SLC7A3	6.9869	5.97E-06	0.2299
EVA1A	4.7347	0.0002	1.43955	KANK1	3.0554	6.04E-06	0.5487
RP11-43A14.1	4.3878	0.0002	0.03395	PLCB1	3.1544	6.19E-06	0.8093
PRR5	3.7224	0.0002	0.60505	RP11-742D12.	6.6005	6.20E-06	0.8636
MYOF	4.7477	0.0002	1.15184	ZNF835	7.4776	6.31E-06	0.1419
CAPN12	4.0013	0.0002	0.59287	CCDC120	6.4450	6.40E-06	0.5659
CTD-2128A3.2	5.9168	0.0002	2.28349	CHST9	6.2616	6.75E-06	0.7348
RP11-115J23.1	3.5955	0.0002	1.95127	ADARB2	6.2533	7.07E-06	1.0284
FAM19A3	3.1763	0.0002	0.56508	LINCR-0001	3.5838	7.15E-06	1.0618
RP3-414A15.2	5.8330	0.0002	0.56476	AC073343.13	3.8178	7.97E-06	0.6155
RP11-553A21.3	5.6687	0.0002	0.90011	LINC01224	6.9425	8.32E-06	1.7384

RPSAP52	4.1921	0.0002	2.23202	PLD5	8.5602	8.78E-06	0.0531
HRAT17	6.1954	0.0002	1.61930	RP11-834C11.	6.1942	9.19E-06	0.2289
CD163L1	4.5323	0.0002	0.60903	STXBP5L	7.4221	9.37E-06	1.0328
GRIN3B	5.2058	0.0002	0.17320	CAMK2B	5.9912	9.40E-06	0.2237
TRANK1	3.3428	0.0002	0.63481	NETO1	7.1856	9.45E-06	2.2945
AC007879.2	4.3314	0.0002	0.76861	RGCC	6.8124	9.92E-06	0.5416
CDS1	4.5939	0.0002	0.46412	ABCB6	3.3070	1.03E-05	0.3453
SP6	3.2391	0.0002	1.14649	USP32P1	7.1264	1.11E-05	0.7514
SGMS2	4.0696	0.0003	1.30459	ITIH6	6.5650	1.17E-05	2.3300
NME3	3.5322	0.0003	0.25691	ZNF578	5.7851	1.21E-05	1.0256
KNDC1	4.6662	0.0003	0.31460	ATOH8	6.2750	1.26E-05	0.5653
AP1S3	3.4009	0.0003	0.49872	DRAXIN	8.9395	1.30E-05	0.6659
STX11	4.8646	0.0003	0.77466	SGK2	6.7794	1.32E-05	0.6031
C1orf204	3.4105	0.0003	0.37009	CREB5	5.1852	1.33E-05	0.4064
IL11	3.6329	0.0003	1.25543	NKAIN4	5.7615	1.40E-05	1.7634
CYP11A1	5.8125	0.0003	1.43145	TMEM35A	6.1157	1.41E-05	1.2180
AC006369.2	5.8652	0.0004	1.04352	RP11-351J23.[illegible]	7.0082	1.41E-05	0.3510
RP11-289F5.1	4.9677	0.0004	0.76085	PROX1-AS1	6.4834	1.45E-05	0.0064
CTC-378H22.2	5.8633	0.0004	0.71676	ARHGAP19-Sl	5.3366	1.54E-05	1.9297
TSGA10IP	5.7246	0.0004	0.34301	U52111.14	5.2091	1.56E-05	0.2236
TNFRSF11A	3.9257	0.0004	1.21691	CYP4F24P	7.6611	1.56E-05	0.1064
APOBEC3D	5.2817	0.0004	1.20233	PLA2G3	6.2217	1.61E-05	0.2056
MSLN	5.7949	0.0004	1.41620	CABLES1	3.0853	1.64E-05	0.3929
RP11-195F19.[illegible]	4.4690	0.0004	0.37952	APC2	3.4693	1.69E-05	0.6474
ABCA5	3.4795	0.0004	0.54194	RPS6KL1	7.4404	1.78E-05	1.4148
AHNAK	3.8656	0.0004	0.91749	RP11-268J15.[illegible]	3.1991	1.80E-05	0.0660
AC009502.4	5.7300	0.0004	0.87633	GABRA4	7.5780	1.88E-05	0.0433
RGS4	3.9116	0.0005	0.79709	C8orf31	6.7142	1.88E-05	0.2861
IRF6	6.1764	0.0005	0.41778	CLCN3P1	6.0025	2.00E-05	0.4349
FAM3D	5.6514	0.0005	0.19854	GABBR2	6.8660	2.02E-05	0.6836
A2M-AS1	4.1688	0.0005	0.55337	RP11-174J11.[illegible]	6.3288	2.05E-05	0.6565
CRABP2	3.3717	0.0006	0.86578	RP13-942N8.1	3.2657	2.40E-05	0.5635
ODF3B	5.5390	0.0006	0.73959	CH17-408M7.[illegible]	6.2584	2.57E-05	0.2412
LINC01611	5.6653	0.0006	1.37590	SRPK3	6.2343	2.83E-05	0.7928
CYYR1-AS1	5.7963	0.0006	0.68461	SYNPO2L	5.3327	2.88E-05	1.6004
C4orf22	5.6398	0.0006	0.72873	TMEM178A	4.8068	2.90E-05	0.2724
CFAP58-AS1	5.2304	0.0006	0.14104	TRIB2	4.9581	3.05E-05	0.5500
XXyac-YX65C[illegible]	3.1524	0.0006	0.47664	PTPRN2	6.1610	3.06E-05	0.2378
AP001596.6	3.7945	0.0006	0.67843	RP11-120J1.1	3.9745	3.13E-05	0.8404
HLA-DRB1	5.7386	0.0006	1.22954	C11orf87	7.4679	3.24E-05	0.2894
TBC1D3P1	5.2911	0.0006	1.09975	LPAR4	4.8136	3.33E-05	1.1740
CTD-2020K17.	3.5746	0.0007	0.87396	C2orf80	6.8205	3.39E-05	1.0319
ALDH1L1	3.9912	0.0007	0.56721	KCNC1	5.2749	3.49E-05	1.0930
VSIR	3.7961	0.0007	0.61088	RHOU	3.1864	3.57E-05	0.0450
LINC01385	5.2138	0.0008	1.07309	ZNF793-AS1	6.7548	3.80E-05	0.9097
LINC00887	4.7682	0.0008	0.75053	CTD-2049O4.[illegible]	7.3214	4.22E-05	0.8453
WT1	3.0548	0.0008	0.98865	MPPED2	7.7375	4.31E-05	1.0711
SERPINE1	4.0008	0.0008	1.81472	AC004540.4	6.2826	4.39E-05	0.5345
SH3TC1	3.3326	0.0008	0.71729	SHISA7	4.8369	4.41E-05	2.3274

IGFBP6	3.1734	0.0008	2.25711
MMP1	5.6339	0.0008	0.73956
ERC2	3.7846	0.0008	0.03046
RP11-288C17.	3.6279	0.0008	0.13041
KCNQ1	4.0772	0.0008	0.73603
SERTAD4-AS1	4.4090	0.0008	1.03537
ICAM3	3.6570	0.0009	0.75673
USP32P2	3.8959	0.0009	0.50473
AMIGO2	3.2317	0.0009	1.31796
SPINT2	5.1454	0.0009	0.56944
RP11-1094H24	3.4360	0.0010	0.82410
RP11-399K21.	3.5805	0.0010	0.43195
SLC7A7	3.6085	0.0010	0.93479
EXPH5	3.5832	0.0010	0.19545
TSPAN19	5.2938	0.0010	0.61481
RP5-1071N3.1	3.2324	0.0011	0.20494
CNGA1	4.0856	0.0011	0.01097
RP11-379B18.	3.0185	0.0011	0.25385
RP11-320G24.	5.4414	0.0011	0.27479
ZNF600	3.2265	0.0011	0.77642
CUL7	3.8050	0.0012	0.74510
MTUS2-AS1	4.3280	0.0013	0.64259
RNLS	3.6791	0.0013	0.68962
SMC1B	3.5405	0.0013	0.13500
LINC01137	3.2354	0.0014	0.56905
TMEM255B	3.6261	0.0014	0.59492
AC023590.1	5.4404	0.0014	0.58251
RP11-26J3.1	3.1958	0.0015	0.16760
RP11-108K3.1	5.3599	0.0016	0.28687
SERPINB7	5.5530	0.0017	1.60606
RGL3	3.2498	0.0018	0.72499
ISG20	3.0068	0.0019	0.70550
PHOSPHO1	4.9892	0.0020	0.37387
RP11-273G15.	4.3554	0.0020	0.53436
SERPINB9P1	4.3809	0.0020	1.10637
GPR160	3.5211	0.0020	0.91619
CTD-2589M5.5	5.0084	0.0020	0.93880
CT45A5	5.3070	0.0021	0.07938
EFNA5	3.7005	0.0021	0.72212
COL12A1	3.3791	0.0021	1.97470
LMNTD2	3.3682	0.0021	0.75230
RP11-276H7.2	4.8640	0.0022	1.09268
RP11-445O3.2	5.2975	0.0022	0.61157
MUC3A	3.8218	0.0022	0.19107
CFAP57	3.6191	0.0022	0.65972
AC137932.6	4.9362	0.0023	0.04071
RNASE10	5.4559	0.0024	0.48354
FES	4.1992	0.0025	1.14158
RP11-686D22.	5.2272	0.0026	0.75118

NOTCH4	3.3530	4.57E-05	0.0190
YBX2	4.0761	4.80E-05	0.8540
DRD2	7.2149	4.98E-05	1.4385
PDZD2	5.0823	5.00E-05	0.3173
PCDHA10	4.5743	5.05E-05	0.7852
PRRT4	4.4372	5.08E-05	0.4625
RARB	6.1972	5.54E-05	0.1622
SLC26A4-AS1	5.4537	6.07E-05	0.4367
HEY2	4.4143	6.08E-05	0.7923
TLX1	6.9507	6.15E-05	1.4355
RP11-582J16.	4.9402	6.60E-05	0.1800
FAAH	3.0637	6.66E-05	0.8473
RP11-625L16.	6.2400	6.85E-05	1.8692
CCDC188	6.7719	6.86E-05	0.7646
LRRC37A	4.0768	7.24E-05	0.2365
DIRC3	6.0294	8.08E-05	0.6085
ZNF681	4.6648	8.20E-05	0.8585
SLC24A2	4.9377	8.93E-05	0.2471
LINC00326	6.3146	9.07E-05	0.1436
LGI3	5.8961	9.07E-05	0.9421
LINC00664	7.0364	9.29E-05	0.8471
CABYR	5.8551	9.87E-05	0.6687
PLGLB1	6.1744	0.0001	0.6562
FGF11	4.0648	0.0001	0.2315
PLCD4	4.8351	0.0001	0.2321
AC108025.2	6.0174	0.0001	1.2788
GUCY1A2	3.9267	0.0001	0.4330
BRINP2	7.4854	0.0001	0.4023
TNR	6.0471	0.0001	2.2137
CTD-2380F24.	5.9619	0.0001	1.4868
CPNE5	4.9851	0.0001	0.8456
P2RX6	3.1143	0.0001	0.0903
HAS2-AS1	4.0856	0.0001	0.1556
LINC00844	6.5062	0.0001	0.8353
SLC6A1	6.2914	0.0001	0.4046
IGFBPL1	6.9162	0.0001	2.4912
EFHB	3.8856	0.0002	0.4369
MN1	4.4432	0.0002	1.2035
KCNH8	6.4337	0.0002	1.8099
ARC	4.3217	0.0002	0.5959
SCN2A	5.7205	0.0002	0.5109
TBX21	5.8877	0.0002	0.9926
LMO1	5.9505	0.0002	1.8365
HOXD-AS2	5.9413	0.0002	0.7560
LINC00977	6.2199	0.0002	0.1053
SCG3	6.1874	0.0002	1.1116
RP11-231C18.	6.0200	0.0002	1.6517
AC073283.4	5.9773	0.0002	1.1089
CDH4	5.0572	0.0002	0.2821

TCHH	4.0853	0.0026	0.96130	PROM2	4.2110	0.0002	1.3758
NLRP3	3.5103	0.0027	0.58435	FOXG1-AS1	6.0182	0.0002	0.4371
TARID	5.1318	0.0028	0.68985	TNKS2-AS1	3.7280	0.0003	0.3557
RP11-568K15.1	3.2391	0.0030	0.92734	TMEM132B	5.3763	0.0003	1.4219
SLC16A3	3.7661	0.0030	0.86781	SGCZ	6.7577	0.0003	0.2330
ACAP1	4.8279	0.0030	0.66589	TLX2	6.1123	0.0003	0.8312
AP000695.4	3.4169	0.0032	0.55724	ALPL	6.1093	0.0003	0.0626
AC018816.3	5.1713	0.0032	0.82565	GRIP2	5.6165	0.0003	1.3666
RP11-565A3.2	5.2522	0.0032	0.22361	IFI44L	6.8232	0.0003	0.2389
TESMIN	3.9377	0.0032	0.78776	RP11-698N11.	5.9051	0.0003	0.7841
PRDM9	5.1964	0.0033	0.00884	HIF3A	6.0409	0.0003	0.2935
BTC	4.1913	0.0033	0.50962	ASXL3	6.5663	0.0003	1.0015
SPATA4	3.1652	0.0034	0.37639	CCDC144A	4.4431	0.0003	0.8893
C15orf48	4.0123	0.0034	1.24892	WFDC2	5.1275	0.0003	0.2908
LINC01776	3.9838	0.0037	0.56968	LINC01748	5.1733	0.0003	0.7891
RP11-114H21.	5.2812	0.0037	0.05373	AC007204.2	6.2085	0.0003	1.1527
DNAI1	3.3832	0.0037	0.17461	RP11-838N2.5	6.0375	0.0003	0.4641
AC005264.2	5.2786	0.0038	0.98056	ZNF433	3.2226	0.0003	0.3425
NLRP14	3.3960	0.0039	0.09753	GPR162	3.2251	0.0003	0.4771
VDR	3.1495	0.0041	2.20966	ZBTB16	5.4963	0.0003	0.6482
KRT80	3.6164	0.0042	1.60467	DCT	4.3779	0.0004	1.7277
NXNL2	3.7519	0.0043	1.21408	TMEM178B	4.8848	0.0004	0.6946
MYO1B	3.7254	0.0043	0.51001	ZFP2	3.5856	0.0004	0.2171
NBEAP1	3.0703	0.0045	0.30936	MASP1	7.7266	0.0004	0.0686
LINC01111	5.5117	0.0045	1.66477	ESPN	4.8488	0.0004	0.4437
RP1-137D17.1	5.0387	0.0046	0.21997	APOL4	6.4464	0.0004	0.0385
RP11-755F10.1	4.2900	0.0049	0.01045	CAMKV	3.4023	0.0004	1.9006
CAPN11	4.3936	0.0050	1.08441	FLRT1	5.8128	0.0004	1.7925
RP11-676J12.7	5.8714	0.0052	0.46775	KCNIP4	6.3195	0.0004	0.3244
MYCBPAP	3.2978	0.0055	0.79495	ILDR2	3.6885	0.0005	0.4312
MLKL	3.1180	0.0057	0.80525	NEUROD1	5.8631	0.0005	1.1665
PAPSS2	3.2313	0.0057	0.61965	PLPPR3	6.0302	0.0005	2.4924
NDNF	3.5747	0.0059	0.36658	IKZF2	3.1807	0.0005	0.1809
SOCS2	3.0625	0.0062	0.18757	LINC01630	5.6072	0.0005	1.7337
NPTX2	3.4776	0.0063	1.28670	AC017104.6	3.3167	0.0006	0.4561
AC131056.3	4.9942	0.0063	0.38697	WNT7A	6.3405	0.0006	0.7564
DEF6	3.6055	0.0066	0.88804	ST3GAL5	3.3009	0.0006	0.2505
MCOLN3	3.4780	0.0069	0.38890	B3GALT2	5.2923	0.0006	1.1476
ELF4	3.0632	0.0070	0.85559	STON2	6.1040	0.0006	0.0009
LINC01939	6.2742	0.0071	0.41921	RP11-507K2.3	5.7402	0.0006	0.5577
AC020571.3	4.5507	0.0072	1.23355	WI2-1896O14.	5.5978	0.0006	0.2526
LRRIQ1	3.9182	0.0074	1.53367	KCNJ9	5.9471	0.0006	1.7324
RP3-467K16.2	4.4681	0.0075	0.56022	SP9	5.9708	0.0006	2.1380
AC092614.2	4.9414	0.0075	0.48941	ADAMTS5	4.3736	0.0007	0.0579
UNC93B3	3.6987	0.0077	0.37359	ZFP30	4.0777	0.0007	0.3282
ACTRT3	3.0419	0.0079	1.15920	FGF14	5.7500	0.0007	0.3414
NEK5	3.3218	0.0084	0.75995	RFLNA	5.6907	0.0007	0.8682
CNFN	4.9846	0.0085	0.49720	HCG17	4.4801	0.0007	0.3996
TFPI2	3.0829	0.0093	2.02646	LIFR-AS1	3.0592	0.0007	0.3076

SERTAD4	3.0394	0.0097	0.75212	EFCAB5	4.1113	0.0007	1.2357
RP11-460B17.2	4.4683	0.0097	2.16402	WSCD1	3.5136	0.0008	0.7507
IL1A	5.0092	0.0098	0.66339	RP11-307B6.3	5.2974	0.0008	0.6533
MAGEA4	5.5329	0.0098	0.33787	IPCEF1	5.7150	0.0008	0.3759
CTD-2540B15.	4.0184	0.0099	0.77401	CSPG5	3.6750	0.0008	1.1222
SMIM1	5.0346	0.0101	1.09564	ADCY8	4.3174	0.0008	0.2693
SFTA1P	3.3467	0.0103	0.70429	MIMT1	6.0754	0.0008	1.6634
RP11-885N19.	4.8961	0.0123	1.61159	NXPH3	3.2696	0.0008	0.8535
ITGAD	4.7187	0.0127	1.45897	OLFM2	3.0780	0.0008	0.1989
LGI4	3.2077	0.0127	0.01996	NCAN	7.5679	0.0008	0.5057
AC008592.3	4.6919	0.0128	0.93842	MESTIT1	6.1561	0.0008	0.1114
LINC01239	3.6509	0.0131	1.49952	LRP1B	4.2071	0.0009	0.3226
VNN1	4.2795	0.0135	1.06338	LINC01102	5.9799	0.0009	1.0991
COL13A1	3.0513	0.0136	2.05599	ADGRG1	4.4408	0.0009	0.5186
RP11-1398P2.	4.2298	0.0140	1.10989	NSG1	5.7824	0.0009	0.1387
AP000442.4	3.4047	0.0141	0.28090	LINC01158	3.5994	0.0009	0.3068
GACAT2	3.2024	0.0144	0.11248	TENM2	4.7292	0.0009	0.1128
TWIST2	3.5408	0.0146	1.66808	HOXA2	4.9391	0.0009	1.3967
SPERT	4.8295	0.0146	0.94762	SHD	5.4326	0.0010	2.0781
GNA15	3.5866	0.0156	1.27382	AP000289.6	6.1491	0.0010	1.6762
SARDH	4.4588	0.0158	0.79471	CTB-118P15.2	5.4817	0.0010	1.0792
WNT4	3.1293	0.0161	0.88294	CTC-467M3.1	5.6468	0.0010	0.5689
OSMR	4.9351	0.0164	1.22204	MCF2L	3.0785	0.0010	0.8547
RP11-535M15.	3.9291	0.0179	0.03152	TMEM229B	3.1153	0.0010	0.2452
NOS3	3.8761	0.0179	0.28895	LINC00643	5.7984	0.0010	1.4408
AC145343.2	3.7509	0.0183	0.53261	LAMP5	6.1520	0.0010	0.0670
AC002076.10	3.8424	0.0184	0.86434	CH17-258A22.	4.1065	0.0010	0.0446
RP11-650K20.3	3.6152	0.0192	0.92055	FAM185BP	3.0974	0.0011	0.0482
LINC02057	4.1215	0.0193	0.39344	PDE8B	3.7006	0.0012	0.2282
PTER	4.6097	0.0197	0.16752	AC053503.11	5.3923	0.0013	1.8829
AC106786.1	4.1912	0.0198	0.46195	CC2D2B	4.5274	0.0013	0.1065
PIK3CG	4.3364	0.0200	0.71098	RUNDC3A	5.3977	0.0013	1.4718
CTD-2337A12.	4.5371	0.0203	0.58038	LEMD1	5.9745	0.0013	0.8117
ITGB2-AS1	4.5937	0.0217	0.60709	PSPC1P1	4.0416	0.0013	0.2474
RP11-14N7.2	4.4781	0.0223	0.24367	LINC00945	5.5410	0.0013	0.7030
RP11-297A16.2	4.1983	0.0225	0.39012	TSPOAP1	5.6147	0.0014	0.6303
LLGL2	3.4688	0.0239	0.41053	HOXD3	5.4949	0.0014	1.4608
AC012358.4	4.4449	0.0262	0.00672	RP4-657D16.3	3.7175	0.0014	0.3317
RP11-89K11.1	3.4433	0.0263	0.31701	HOXD4	5.4159	0.0015	1.3408
MOV10L1	4.4441	0.0265	0.63408	STUM	5.4701	0.0015	0.4918
EBF2	3.6057	0.0267	0.15407	TTBK1	4.7454	0.0016	0.7564
GATA6-AS1	3.9957	0.0302	1.26542	LFNG	4.4011	0.0016	0.1920
RP3-467K16.7	4.4115	0.0304	1.50772	DDR1	3.4264	0.0017	0.0309
ADAMTS12	3.1979	0.0317	1.13029	RP11-430H10.	5.5299	0.0017	0.3254
GPAT3	3.3176	0.0317	1.28396	SERP2	6.0986	0.0017	0.6553
FOXC2-AS1	4.5399	0.0319	1.07089	CD101	5.3992	0.0017	0.2487
WFDC21P	4.4383	0.0330	1.31817	AP006222.2	5.4094	0.0017	0.1647
RP11-95P2.3	4.3111	0.0331	1.02435	CASQ1	5.8751	0.0018	0.3069
SNX29P1	4.0409	0.0339	0.04275	RPS6KA2	3.3976	0.0018	0.0586

RP11-366F6.2	4.3428	0.0355	0.40639
TMEM190	4.3686	0.0370	0.99047
AC092168.2	3.8692	0.0382	0.68960
F2RL1	3.1439	0.0385	1.20420
C19orf33	4.2328	0.0388	0.19382
HLA-V	3.1816	0.0388	0.07377
CSF3	4.2738	0.0389	2.51381
SIDT1	3.8411	0.0394	0.03443
MED15P9	4.4181	0.0396	0.25970
RP11-54O7.18	4.3049	0.0404	0.22346
RP11-438D8.2	4.2184	0.0409	0.21019
RP11-150C16.	3.3217	0.0412	0.49684
EREG	4.2971	0.0415	3.12461
RP11-350J20.5	3.0287	0.0421	0.14878
PRDM16	3.2025	0.0424	0.12259
TMEM191A	3.3151	0.0424	0.08724
RP11-525A16.4	3.5332	0.0426	1.12813
GMNC	4.3933	0.0434	0.68632
FGF7P8	4.3642	0.0434	0.03762
PYY	3.3379	0.0434	0.05025
RP11-666A20.4	4.1992	0.0450	0.13444
RP11-360O19.	4.2274	0.0469	0.20909
HSPA1A	4.1625	0.0491	0.39271
CTD-2334D19.	4.3314	0.0492	0.02606
RP11-89F3.2	3.7575	0.0496	0.15480
TRPM6	3.3470	0.0500	0.01057

RP11-38M8.1	5.9893	0.0018	0.2782
AP3B2	4.7431	0.0018	0.9162
JPH4	6.1414	0.0018	1.3734
RXFP1	4.8677	0.0018	0.7205
COL28A1	4.4945	0.0019	0.4136
MIR181A2HG	3.0687	0.0020	0.8415
PRSS51	5.4016	0.0022	0.9272
CTC-255N20.1	5.9100	0.0024	0.4790
RUNX1T1	6.9975	0.0024	0.4504
SOSTDC1	5.4086	0.0025	0.0885
AVIL	3.5325	0.0025	1.2415
FMO5	3.4178	0.0026	0.2359
ATP2B2	5.2661	0.0026	0.8111
CLSTN2	5.7215	0.0027	1.1158
AP000708.1	5.3579	0.0028	0.1114
NRXN3	5.2728	0.0028	0.3186
ATG9B	3.5894	0.0028	0.3798
OLFM3	6.8703	0.0029	0.9459
TBC1D3D	4.8908	0.0030	0.7413
RP11-247A12.	4.1691	0.0030	0.3506
GSG1L	5.6553	0.0031	0.5406
C7orf61	3.7172	0.0031	0.6755
RP11-620J15.1	5.3183	0.0032	2.0189
TUBB2BP1	4.7587	0.0033	0.5226
SPATA22	5.1520	0.0034	0.1093
KLRK1	6.8134	0.0034	2.0787
LRRC3B	5.5468	0.0036	0.8352
RP5-1024C24.	5.9992	0.0036	0.8969
WFDC1	4.6954	0.0037	0.7572
PCDHB9	3.2032	0.0037	0.6081
SEC14L5	4.3765	0.0037	1.0790
BEX2	5.0667	0.0037	0.7579
ASB4	5.2528	0.0037	0.6381
NECAB2	3.6956	0.0038	0.9083
EFTUD1P1	3.2348	0.0039	0.3442
LINC00506	5.2956	0.0039	0.1917
BMS1P2	4.0521	0.0039	0.4080
GFI1B	3.7275	0.0040	0.5945
RGMA	3.4527	0.0041	0.0208
FSBP	3.7881	0.0042	0.9495
ZNF423	4.8742	0.0043	0.3785
RP11-281O15.	5.0481	0.0043	0.6800
LINC00877	5.1626	0.0044	0.2446
PNMA3	4.2836	0.0045	1.2794
LINC00606	5.9152	0.0045	0.4150
DACT3-AS1	4.6344	0.0046	0.8437
AFF3	4.8507	0.0047	0.6721
HHATL	5.3547	0.0049	0.9503
GPR12	5.2131	0.0050	1.4840

ASCL1	6.4222	0.0051	1.5635
RP11-758P17.	3.7142	0.0052	0.1701
RP11-216B9.9	3.2349	0.0055	0.5805
RP11-136C24.	5.2985	0.0055	0.5999
SPATA42	5.0719	0.0056	0.5594
LTK	3.3889	0.0057	0.6213
GNG3	5.0272	0.0057	0.7093
RP11-620J15.4	4.9438	0.0058	1.7478
ARHGDIG	5.3221	0.0059	0.9620
KIAA1755	3.4167	0.0060	0.4759
LINC01977	5.3226	0.0061	1.0575
RP11-241F15.	4.7008	0.0062	0.9812
ZEB2-AS1	4.0995	0.0063	0.1863
KLRC2	6.2284	0.0064	2.8325
GDF7	5.2938	0.0065	0.9583
CACNA1D	4.4575	0.0066	0.6191
PLXNA2	3.7076	0.0066	0.2782
PODN	5.7177	0.0066	0.6198
PCDHA3	5.7077	0.0067	0.8190
SELENOV	5.2568	0.0069	1.1943
TRIM67	4.9740	0.0072	2.6221
SDK2	4.7772	0.0074	0.0914
OLFM1	6.2273	0.0074	0.9495
LINC01816	4.9271	0.0075	0.8473
CHRNA4	5.6709	0.0081	2.2968
RP11-277P12.	4.9712	0.0081	2.2566
BCL2L15	3.0285	0.0081	0.5070
ACTN2	3.0883	0.0084	0.2396
RP11-472N13.	4.4647	0.0085	0.7597
GPR158-AS1	4.8485	0.0086	0.0642
AC003991.3	4.8317	0.0088	0.4532
RP11-318C24.	4.8280	0.0091	0.5283
AC005220.3	5.5520	0.0094	0.1061
RNF128	5.1830	0.0095	0.0309
GABRB3	5.5482	0.0098	1.3130
SLC26A1	4.8443	0.0099	0.6689
GABRA2	4.9564	0.0100	0.4802
RP11-259K15.	5.0183	0.0101	1.1989
NXPH2	5.5025	0.0103	1.1442
SKINT1L	4.5984	0.0109	0.5295
PCDHGA10	3.2617	0.0110	0.6541
SNAP25	3.5742	0.0110	0.8426
TMEM130	4.6349	0.0110	0.6108
IGSF5	4.8598	0.0114	0.2773
ZNF571-AS1	4.9748	0.0117	0.8521
MYOZ3	4.1371	0.0122	0.3432
RTN1	3.7524	0.0124	0.4498
GDF6	5.4197	0.0125	0.9618
PDIA2	4.9002	0.0127	1.4515

HCG22	5.3763	0.0129	0.2231
LINC01505	3.3301	0.0131	0.7594
GS1-259H13.2	3.1565	0.0132	0.0925
DNAJA4	5.1252	0.0135	0.0129
RP11-50D16.4	4.6512	0.0138	0.6064
RASL10A	4.6459	0.0141	0.8780
LINC00648	4.6452	0.0145	1.5474
CDH3	3.7692	0.0146	0.6309
GPER1	3.2455	0.0147	0.1122
AC027612.4	5.3158	0.0148	0.4684
MEG9	5.2727	0.0157	0.0300
CPA2	5.2946	0.0157	0.1324
MYH7	4.8622	0.0159	0.4765
UNC13A	3.2187	0.0160	1.1579
THSD7A	3.6526	0.0161	1.0109
GRM2	4.4276	0.0164	0.9824
TMEM132C	5.2805	0.0176	1.0937
RP11-627G23.	4.6094	0.0181	0.2903
RSU1P2	5.2254	0.0182	0.3793
ATP8A2	4.2915	0.0185	0.5791
RP11-351J23.	4.4960	0.0196	0.5468
LINC01549	4.5724	0.0202	0.4761
SCN2B	4.6126	0.0207	0.2847
MMD2	4.7039	0.0210	1.6718
ZIM2-AS1	5.7285	0.0216	1.0882
KCNAB1	4.6342	0.0216	0.3873
AC006026.13	4.4791	0.0221	0.2633
SLC6A11	4.7330	0.0222	0.0728
NRXN1	4.8786	0.0224	1.0610
GFRA3	4.7162	0.0225	0.6031
SEZ6L	5.6002	0.0231	1.3996
AC013460.1	3.9413	0.0232	1.3381
SAMD14	3.2107	0.0236	0.6566
FAM182A	3.6100	0.0239	1.0859
RP11-266K4.9	4.2405	0.0241	0.4630
SEMA6A-AS2	3.3040	0.0241	0.1935
IGLON5	5.5749	0.0243	1.3758
RP1-274L7.4	3.8309	0.0245	0.8858
C5orf67	5.0275	0.0246	0.1530
AC083867.4	4.6536	0.0253	1.6979
TPTE2P5	3.0629	0.0259	0.2563
GRM7	3.4682	0.0263	1.0132
RP11-103J8.1	4.6266	0.0270	0.0646
C16orf45	3.1398	0.0279	0.4463
LINC01679	4.4973	0.0281	0.9819
RP11-615I2.2	4.3658	0.0286	1.2044
GADD45G	4.6236	0.0287	1.8836
RP11-728K20.	4.9300	0.0289	0.1546
CTD-2010I16.1	3.2384	0.0289	0.4354

LINC01207	4.7089	0.0295	1.5553
RAPGEF4-AS1	4.9379	0.0295	0.4769
SEZ6	5.3375	0.0296	1.4494
TMEM88	3.9319	0.0302	0.0721
AC007255.8	3.5024	0.0307	0.7794
PRR26	4.3607	0.0317	0.8069
RP11-536C5.2	4.3234	0.0317	0.8584
SPON1	4.3840	0.0317	0.2559
RPH3A	5.3879	0.0324	0.2942
KCNQ4	4.0489	0.0326	0.2564
NAPSA	3.8871	0.0331	0.0376
HOXD13	3.1541	0.0339	0.2672
ZNF663P	4.3205	0.0340	0.6102
RP11-15I11.3	3.5674	0.0342	0.8645
CTD-2339F6.1	5.0117	0.0344	1.9449
RP11-182J1.14	3.3037	0.0347	1.2716
MIR181A1HG	3.3451	0.0348	1.0008
ADCY2	3.9081	0.0351	0.6100
RP11-138I1.3	4.4079	0.0354	0.2193
RP11-624M8.1	3.8383	0.0357	0.1144
RP11-109E12.	4.3468	0.0365	0.5169
RIPPLY3	4.2795	0.0366	0.5124
MYBPHL	3.9107	0.0372	0.3339
LINC00598	3.2572	0.0374	0.0892
GRID2IP	3.0258	0.0377	0.7852
ACSL6	4.3323	0.0395	0.6218
PLCL1	3.0874	0.0396	0.7133
SATB1-AS1	4.2655	0.0402	1.0831
CTD-2619J13.	3.8705	0.0407	0.1729
C22orf24	3.3622	0.0408	0.4299
RP13-644M16	4.3542	0.0415	0.2604
FAM19A5	4.0654	0.0419	0.8651
SLC35D3	4.4636	0.0421	0.7549
IGSF9	3.8339	0.0427	1.3024
HYAL1	3.0169	0.0433	0.1436
RP11-369K16.	4.3785	0.0439	0.2091
LINC00519	4.1742	0.0441	0.0473
NRXN2	3.2652	0.0445	1.1900
ROBO2	3.1047	0.0451	0.7539
OTOGL	3.2026	0.0457	0.9311
CTC-344H19.4	3.5342	0.0460	0.2764
RNF219-AS1	4.2873	0.0461	1.2373
IL1RAPL2	4.6414	0.0462	0.7757
CCND2-AS1	3.9478	0.0470	0.4098
FAM189A1	3.0610	0.0479	0.1377
RGS11	4.4423	0.0490	0.1674
SHISA9	4.2851	0.0499	0.6837

Supplementary Table 7: Alternatively spliced and differentially expressed genes between mesenchymal (MES) and proneural (PN) GSCs.

ASS (alternative splice site 5' or 3'): genomic coordinates of long and short exons (columns g.coord

MXE (mutually exclusive exon):genomic coordinates of first and second exons (columns g.coord_1

RI (retained intron): genomic coordinates of retained intron (column g.coord_1)

ES (exon skipping): genomic coordinates of skipped exon (column g.coord_1)

	Differential expression		Differential splicing					
**gene**	**log2FoldChange**	**FDR**	**event.type**	**strand**	**g.coord_1**	**g.coord_2**	**inc.level.difference**	**FDR**
ABCB6	-3.307001766	1.03E-05	RI	-	chr2:219210380-219210823	NA	0.538	0.001910688849
ABCD4	-1.49262165	0.000205653789	ES	-	chr14:74295147-74295198	NA	-0.467	0.000418686846
			ES	-	chr14:74297638-74298069	NA	0.168	0.000507655253
			ES	-	chr14:74299547-74299675	NA	0.145	0.001061603759
			ES	-	chr14:74300149-74300268	NA	0.162	0.004938847597
			ES	-	chr14:74288206-74288259	NA	-0.172	0.01087266728
			ES	-	chr14:74299547-74299675	NA	-0.244	0.01184031581
			ES	-	chr14:74300149-74300268	NA	-0.157	0.04822228261
ABHD11	-2.239355234	0.000919304976	ES	-	chr7:73737220-73737360	NA	0.193	0.007980648665
			RI	-	chr7:73737220-73737735	NA	-0.285	0.0236309712
ABHD14B	1.769971385	5.59E-06	ES	-	chr3:51971811-51971892	NA	-0.435	4.64E-06
ABHD3	-1.355213982	0.000187405328	A5SS	-	chr18:21663677-21664230	chr18:21664117-21664230	0.388	4.71E-07
ABL2	1.946649981	3.09E-13	ES	-	chr1:179133311-179133374	NA	-0.173	0.001458185891
AC005154.6	-2.818045991	1.77E-11	MXE	-	chr7:30576563-30576710	chr7:30584710-30584808	0.278	0.02127345004
AC007040.11	3.466808863	0.002252917358	ES	-	chr2:70991829-70991983	NA	-0.101	0.01868342172
AC007566.10	-1.014212726	0.04603914804	ES	+	chr7:92462957-92463026	NA	0.228	3.06E-06
AC108488.4	-1.73751869	0.01934800273	ES	-	chr2:3533107-3533190	NA	0.211	0.02986419007
ACAD11	1.659899111	0.005686184971	RI	-	chr3:132578795-132579558	NA	-0.11	0.00157883001
ACAT1	1.901181734	0.000194905780	ES	+	chr11:108129102-108129201	NA	0.17	0.04997449951
ACSS1	-1.001498873	0.0250473989	ES	-	chr20:25015137-25015230	NA	-0.146	0.02664983959
ACTN1	1.184788077	6.70E-05	ES	-	chr14:68878988-68879069	NA	0.131	2.38E-08
ADAM22	-1.886876099	0.0016332856	ES	+	chr7:88186614-88186701	NA	-0.22	4.44E-07
ADAMTS6	2.436437792	0.009602273143	ES	-	chr5:65470777-65471142	NA	-0.295	6.69E-05
ADAP2	3.360071655	1.37E-09	MXE	+	chr17:30926826-30926918	chr17:30931888-30931968	0.411	3.40E-05
ADAT1	-1.502652197	6.01E-05	ES	-	chr16:75618585-75618698	NA	0.123	0.009096233477
ADCY6	-1.377884627	0.01864612585	ES	-	chr12:48773939-48774098	NA	-0.182	0.001421550924
			A3SS	-	chr12:48773468-48774098	chr12:48773468-48773647	-0.286	0.002706536394
ADD1	-1.324669409	0.003595515826	RI	+	chr4:2908514-2909431	NA	0.284	0.01160406502
ADGRG1	-4.440798232	0.000895656285	ES	+	chr16:57641590-57641708	NA	0.318	0.005039214886
			ES	+	chr16:57641586-57641708	NA	0.443	0.009505385205
ADGRL1	-1.685372987	0.000393716323	ES	-	chr19:14167015-14167030	NA	-0.204	0.002071693895
ADGRL2	-4.221479784	2.51E-06	ES	+	chr1:81986900-81987029	NA	0.227	3.49E-10
			ES	+	chr1:81987288-81987341	NA	-0.256	1.66E-05
			ES	+	chr1:81952986-81953025	NA	0.368	0.004113255113
			ES	+	chr1:81987259-81987341	NA	-0.144	0.0439700115
ADGRL3	-2.625659858	0.000158145126	ES	+	chr4:61730621-61730636	NA	-0.224	0.001796423469
			MXE	+	chr4:61676825-61676935	chr4:61732753-61733554	-0.138	0.008473719203
AFDN	-1.359167603	9.79E-06	ES	+	chr6:167913402-167913423	NA	-0.579	4.69E-10
AGAP1	-1.972480215	0.004616489308	ES	+	chr2:235750353-235750488	NA	-0.18	3.67E-06
AGAP4	-1.336505827	0.000590755917	MXE	-	chr10:45841652-45841687	chr10:45844325-45844394	0.785	1.11E-09
			ES	-	chr10:45834015-45834116	NA	-0.8	0.000114900623
			MXE	-	chr10:45834015-45834116	chr10:45841652-45841687	0.708	0.000194493061
AGPAT4	-2.101653927	1.40E-14	ES	-	chr6:161165576-161165713	NA	0.184	1.73E-06
			MXE	-	chr6:161154148-161154310	chr6:161165576-161165713	0.108	0.000617106267
			ES	-	chr6:161165576-161165713	NA	0.441	0.001023745599
ALG9	-1.252130947	0.003147345933	A3SS	-	chr11:111838248-111838399	chr11:111838248-11183837	0.2	0.009557061151
ALKBH8	1.114900193	0.01146910437	ES	-	chr11:107549752-107549823	NA	-0.271	0.000185782203
ALPK1	2.727274259	0.001304598993	ES	+	chr4:112315800-112315852	NA	-0.153	0.004367829994
AMMECR1L	-1.161534664	0.000509267750	ES	-	chr2:127871248-127871359	NA	0.193	0.02137621621
AMPD2	2.127534674	0.001378832489	ES	+	chr1:109622184-109622353	NA	-0.554	0.000293523243
ANAPC10	1.022415053	0.01885973881	ES	-	chr4:145097687-145097760	NA	0.592	0.006742072944

ANAPC11	1.453076968	0.012934033	ES	+	chr17:81899241-81899542	NA	0.106	0
ANAPC5	1.354446441	0.007216952797	RI	-	chr12:121345838-121347005	NA	-0.144	0
			RI	-	chr12:121327095-121328497	NA	-0.144	0
			A3SS	-	chr12:121328315-121328497	chr12:121328315-12132845	0.198	5.53E-11
ANK3	-5.405543463	3.28E-36	ES	-	chr10:60200128-60200263	NA	0.135	0.002225080387
			A3SS	-	chr10:60059339-60060006	chr10:60059339-60059430	0.164	0.003617031386
			ES	-	chr10:60059697-60060033	NA	0.119	0.00550678331
			ES	-	chr10:60145967-60146021	NA	0.18	0.02371629734
ANKHD1-EIF4E	-1.934119146	0.000596927736	ES	+	chr5:140496519-140497278	NA	0.155	2.70E-05
			A3SS	+	chr5:140537337-140537589	chr5:140537388-140537589	0.176	0.000113258999
ANKRD10	-2.292872501	2.15E-11	A3SS	-	chr13:110900529-110900694	chr13:110900529-11090066	0.144	0.000443820326
			ES	-	chr13:110894284-110894393	NA	-0.459	0.01041046655
ANKRD20A11F	-2.370088227	0.000991961178	MXE	-	chr21:13915186-13915409	chr21:13916746-13916958	0.288	0.002470228289
ANKRD27	3.621652629	2.55E-25	MXE	-	chr19:32607634-32607832	chr19:32615657-32615780	-0.209	0.004476786939
			MXE	-	chr19:32605834-32605954	chr19:32607634-32607832	0.209	0.01128144421
ANXA4	1.080039129	0.005402096904	A5SS	+	chr2:69806384-69806498	chr2:69806384-69806432	-0.17	2.79E-05
AP1AR	1.737939287	0.000674774728	ES	+	chr4:112262987-112263086	NA	0.121	0.002927110877
AP1S2	-2.574886233	1.17E-05	ES	-	chrX:15833443-15833524	NA	0.284	0.03860979484
APAF1	-1.567964156	3.34E-05	ES	+	chr12:98703370-98703499	NA	-0.278	0.001135475143
APBB2	-2.016174325	4.24E-08	ES	-	chr4:40935076-40935139	NA	0.711	0
APOM	-2.442445538	3.57E-05	ES	+	chr6:31656471-31656626	NA	0.162	0.000963896981
APRT	1.454839965	0.005964505308	A3SS	-	chr16:88809468-88809840	chr16:88809468-88809706	0.167	1.57E-06
AREL1	-1.10195675	0.000261842113	ES	-	chr14:74701668-74701774	NA	0.502	0.001735749298
ARHGAP21	-1.782212896	8.36E-08	ES	-	chr10:24622732-24622762	NA	-0.367	1.48E-07
ARHGEF10	1.376884529	0.002520603555	ES	+	chr8:1926376-1926463	NA	-0.138	1.97E-05
			ES	+	chr8:1880047-1880164	NA	-0.324	0.000880675005
ARHGEF40	-1.48529973	0.02048618293	MXE	+	chr14:21087000-21087105	chr14:21087319-21087463	-0.249	6.58E-05
			RI	+	chr14:21087000-21087463	NA	-0.404	0.000594384868
			ES	+	chr14:21075644-21075765	NA	-0.169	0.000780430881
			ES	+	chr14:21075622-21075765	NA	-0.248	0.000884318560
			A5SS	+	chr14:21075331-21075765	chr14:21075331-21075499	-0.301	0.001128835062
ARHGEF7	-3.169599061	2.19E-26	MXE	+	chr13:111205288-111205373	chr13:111209871-11121000	0.312	7.62E-10
			ES	+	chr13:111288353-111288443	NA	-0.162	8.37E-06
			MXE	+	chr13:111205288-111205373	chr13:111209871-11121000	0.219	1.07E-05
			ES	+	chr13:111209871-111210002	NA	-0.238	1.63E-05
			ES	+	chr13:111209871-111210002	NA	-0.205	0.007873662431
			MXE	+	chr13:111153904-111153991	chr13:111209871-11121000	0.14	0.008703836928
ARL4A	-1.808872899	0.007965219414	ES	+	chr7:12687634-12687728	NA	-0.602	0
			ES	+	chr7:12687634-12687728	NA	-0.499	7.00E-10
ARMC4	5.531166913	2.15E-12	ES	-	chr10:27968922-27969018	NA	-0.369	0.01357652802
ARMCX5-GPRA	-1.407923176	0.00109723521	ES	+	chrX:102601879-102601977	NA	0.266	1.98E-07
			ES	+	chrX:102601463-102601509	NA	0.358	0.000666033342
			MXE	+	chrX:102601463-102601509	chrX:102601879-102601977	-0.371	0.001699714909
			MXE	+	chrX:102601879-102601977	chrX:102639399-10263953:	0.154	0.02227107106
ARNTL2	3.112129098	1.27E-15	ES	+	chr12:27370128-27370230	NA	-0.173	0.000297930725
			MXE	+	chr12:27370128-27370230	chr12:27376345-27376387	-0.224	0.002977500621
			ES	+	chr12:27376345-27376387	NA	0.204	0.005462739449
			ES	+	chr12:27370128-27370230	NA	-0.309	0.02166060778
ARPC4	-1.03693576	0.03217150924	MXE	+	chr3:9797658-9797777	chr3:9800184-9800296	0.15	9.96E-07
ARPP19	-1.185558463	0.002416539789	ES	-	chr15:52564189-52564246	NA	-0.288	3.04E-05
			ES	-	chr15:52568847-52568902	NA	-0.12	0.000174792228
			ES	-	chr15:52568847-52568900	NA	-0.143	0.000749563285
ARRDC1	2.227428125	0.000101178293	RI	+	chr9:137612895-137613510	NA	-0.299	1.29E-06
ASAP2	1.226637135	3.03E-05	ES	+	chr2:9391061-9391196	NA	-0.539	6.77E-06
ASPH	2.246488196	3.23E-06	ES	-	chr8:61646749-61646878	NA	0.112	0
			MXE	-	chr8:61651049-61651124	chr8:61653567-61653660	0.132	0
			MXE	-	chr8:61618977-61619019	chr8:61633682-61633727	0.21	2.91E-07
			MXE	-	chr8:61583943-61584029	chr8:61633682-61633727	0.178	1.90E-06
ASPSCR1	1.548665312	0.007131859271	RI	+	chr17:82016799-82017113	NA	0.125	0.002774257415
			MXE	+	chr17:82016475-82016527	chr17:82016799-82016869	-0.327	0.03891190073

ATAD2	1.758292203	2.57E-06	ES	-	chr8:123400742-123401175	NA	0.254	0.02776636468
ATAD5	1.107936454	0.003567230275	ES	+	chr17:30879422-30879487	NA	-0.194	1.38E-05
ATAT1	-1.772771174	3.82E-08	RI	+	chr6:30642767-30644679	NA	0.271	2.34E-08
			MXE	+	chr6:30642767-30643011	chr6:30645894-30645974	0.4	0.000570966894
			ES	+	chr6:30642767-30643011	NA	0.236	0.01637108017
			ES	+	chr6:30640534-30640603	NA	-0.265	0.02320561358
ATP11A	-1.141711817	0.04120840326	A3SS	+	chr13:112881812-112887168	chr13:112881875-11288716	0.39	0.001121865795
			ES	+	chr13:112878216-112878303	NA	-0.461	0.002993621228
ATP2B1	-2.479504465	2.50E-07	MXE	-	chr12:89627677-89627716	chr12:89630504-89630645	0.152	0.007378547302
ATP6V1B1	-1.639697141	0.0235821543	ES	+	chr2:70963161-70963261	NA	0.253	0.000812214171
			ES	+	chr2:70963161-70963312	NA	0.147	0.001312492684
ATP9B	-2.419285766	5.32E-10	ES	+	chr18:79375393-79375426	NA	-0.2	0.000136276856
			MXE	+	chr18:79154503-79154555	chr18:79176812-79176907	-0.167	0.001097782761
AURKB	2.363529693	5.90E-09	ES	-	chr17:8210176-8210249	NA	-0.232	1.97E-11
			ES	-	chr17:8210176-8210225	NA	-0.33	1.60E-09
			A3SS	-	chr17:8207737-8207901	chr17:8207737-8207840	-0.343	0.007397341977
B3GNTL1	2.81932632	7.08E-11	MXE	-	chr17:82965650-82965746	chr17:83005114-83005210	-0.181	0.000398856445
BAIAP2	2.052515418	4.82E-05	ES	+	chr17:81036862-81036961	NA	-0.133	0.03537587177
BAX	-1.699565106	0.00169985767	RI	+	chr19:48960809-48961798	NA	0.233	0
			ES	+	chr19:48955547-48955599	NA	0.209	0.02365356143
BCAS4	1.852960821	0.000187599744	ES	+	chr20:50840536-50840930	NA	-0.605	0
BCHE	-6.773798862	2.14E-21	ES	-	chr3:165829516-165831041	NA	-0.372	1.84E-13
			ES	-	chr3:165777716-165777795	NA	0.11	1.52E-06
BCL2L11	-2.97836296	2.82E-09	ES	+	chr2:111124049-111124139	NA	0.168	0.000244558941
			A5SS	+	chr2:111123745-111124139	chr2:111123745-111123869	0.154	0.000276218368
			ES	+	chr2:111123732-111123869	NA	0.111	0.01404100243
BCL2L12	1.036374197	0.03262397763	ES	+	chr19:49667018-49667161	NA	-0.105	0.01389440019
			ES	+	chr19:49667021-49667161	NA	-0.145	0.02383511682
BDH2	-2.602569511	2.91E-08	A5SS	-	chr4:103085698-103086540	chr4:103086479-103086540	-0.241	0
			RI	-	chr4:103085348-103086540	NA	-0.253	0
			RI	-	chr4:103085348-103086540	NA	0.223	8.25E-05
			A5SS	-	chr4:103085348-103086540	chr4:103086479-103086540	-0.231	0.04209827853
BDNF-AS	-2.440499658	0.000270164116	ES	+	chr11:27658240-27658462	NA	0.274	1.00E-06
			ES	+	chr11:27675576-27676140	NA	0.343	0.001406429329
			ES	+	chr11:27675296-27676140	NA	0.368	0.00177508727
BEND6	2.07869001	0.000488670948	ES	+	chr6:57018420-57018557	NA	-0.22	0.02050644615
BMP1	-1.898669967	0.01614389603	A3SS	+	chr8:22201117-22201928	chr8:22201802-22201928	0.321	0.000398575518
			A3SS	+	chr8:22176923-22177139	chr8:22176960-22177139	0.119	0.01003596905
BPHL	-1.510703854	1.33E-05	ES	+	chr6:3119284-3119512	NA	0.475	1.12E-05
			A3SS	+	chr6:3138040-3140509	chr6:3140385-3140509	0.145	0.004748089327
			RI	+	chr6:3138040-3140509	NA	-0.198	0.01313973159
BTBD3	-1.310719678	0.00191197591	RI	+	chr20:11917864-11918601	NA	-0.352	0.000314242349
C11orf49	-1.685126938	0.005878006534	ES	+	chr11:47157022-47157218	NA	-0.276	1.06E-10
			RI	+	chr11:47161126-47162247	NA	0.261	0.000656008530
C11orf65	1.684546621	0.004043590229	ES	-	chr11:108431745-108431838	NA	0.536	0.001578796476
			MXE	-	chr11:108405428-108405559	chr11:108431745-10843183	0.19	0.01613726558
C14orf159	-1.018135535	0.0038468482	A5SS	+	chr14:91215404-91215793	chr14:91215404-91215556	0.236	0.03523702781
C19orf12	1.45752136	4.01E-05	ES	-	chr19:29708253-29708423	NA	-0.347	0.000170947032
C1orf112	1.271165254	0.009836671994	A3SS	+	chr1:169804074-169804240	chr1:169804111-169804240	0.17	0.003340729773
C1QTNF1	-2.224858531	0.006969056909	ES	+	chr17:79043954-79044123	NA	0.132	0.003512996048
C1RL	1.429504006	0.02653790596	ES	-	chr12:7108250-7108479	NA	0.435	2.43E-05
C20orf194	-1.095541521	0.02358174193	ES	-	chr20:3343675-3343731	NA	-0.169	0.0087482388
C21orf58	-1.187242538	0.009693058467	RI	-	chr21:46301129-46302154	NA	0.265	3.09E-06
			A3SS	-	chr21:46314715-46315049	chr21:46314715-46314880	0.528	0.01015531981
C5orf22	-1.025136578	0.002193718997	ES	+	chr5:31548489-31548687	NA	0.15	9.78E-06
C6orf52	-3.754987247	8.14E-07	ES	-	chr6:10686965-10687056	NA	0.117	0.0084424949
			ES	-	chr6:10672546-10672636	NA	0.505	0.01885208174
			MXE	-	chr6:10683186-10683232	chr6:10686965-10687164	0.145	0.02470719013
			MXE	-	chr6:10683186-10683232	chr6:10686965-10687164	0.123	0.03336528488
C9orf85	-1.363159949	0.001757229442	ES	+	chr9:71971504-71971618	NA	0.131	0.004011400497

CADM2	-5.915072343	1.94E-14	ES	+	chr3:85979163-85979283	NA	0.535	5.93E-06
CAMK1	-9.994859989	4.23E-11	ES	-	chr3:9765758-9765890	NA	0.122	0.001499769913
CAMK2D	-1.860134917	0.03850898767	A3SS	-	chr4:113509637-113509675	chr4:113509637-113509672	0.216	0.004100974342
CAMTA2	1.011549198	0.03148317724	ES	-	chr17:4982088-4982160	NA	-0.239	0.000390645863
			ES	-	chr17:4982088-4982227	NA	-0.274	0.002049001138
			ES	-	chr17:4982088-4982175	NA	-0.265	0.003568838564
CARF	-1.718585399	0.002492204581	ES	+	chr2:202974333-202974496	NA	0.101	0.003230019984
			MXE	+	chr2:202924356-202924415	chr2:202941859-202941980	-0.149	0.004239967383
CASK	-1.218153687	0.000150500121	ES	-	chrX:41555599-41555635	NA	-0.278	1.34E-11
			ES	-	chrX:41557031-41557100	NA	-0.608	4.65E-08
			MXE	-	chrX:41555599-41555635	chrX:41557031-41557100	0.134	0.001407486282
CASP4	5.47407291	5.91E-26	ES	-	chr11:104951895-104952063	NA	-0.144	0.005857179572
CASP8	2.371285742	0.02496524068	A3SS	+	chr2:201266460-201266791	chr2:201266486-201266791	-0.127	0.002459197716
CAST	1.654880843	0.000288261170	ES	+	chr5:96726793-96726859	NA	-0.698	0
			ES	+	chr5:96726753-96726859	NA	-0.557	0
			MXE	+	chr5:96727488-96727530	chr5:96729152-96729209	0.368	6.23E-14
			ES	+	chr5:96740744-96740783	NA	0.662	2.11E-09
			ES	+	chr5:96729152-96729209	NA	-0.178	4.62E-07
			ES	+	chr5:96726793-96726859	NA	-0.543	0.000103588851
			MXE	+	chr5:96722638-96722698	chr5:96729152-96729209	0.177	0.00121263946
CC2D2A	-1.390230277	0.001385648879	ES	+	chr4:15479218-15479328	NA	0.369	0.01564723304
			ES	+	chr4:15479222-15479328	NA	0.366	0.01617330057
CCDC125	2.030840177	0.002684419434	ES	-	chr5:69294792-69294900	NA	-0.188	0.004507877005
CCDC159	-1.294502763	0.03393469197	RI	+	chr19:11354579-11354943	NA	0.208	0.01046368681
CCDC7	2.031794083	2.35E-06	ES	+	chr10:32567669-32567891	NA	0.407	0.00357102587
			MXE	+	chr10:32463016-32463049	chr10:32471063-32471230	-0.421	0.0054461904
			ES	+	chr10:32463016-32463049	NA	-0.147	0.01351304657
			ES	+	chr10:32471063-32471230	NA	0.41	0.03915855325
CCNE1	1.359604972	0.03822066458	ES	+	chr19:29821995-29822130	NA	-0.164	1.69E-05
			ES	+	chr19:29812531-29812578	NA	-0.23	0.000898392038
CCNT2	-1.499508598	4.38E-05	ES	+	chr2:134945786-134945982	NA	-0.132	0.02298175873
CD27-AS1	-1.901100354	0.000615687138	MXE	-	chr12:6448639-6448737	chr12:6450340-6450980	-0.223	0.000313158994
			ES	-	chr12:6443545-6443717	NA	0.321	0.000758051464
			ES	-	chr12:6450340-6450980	NA	-0.27	0.01669192307
			ES	-	chr12:6450869-6450980	NA	-0.313	0.01675843214
			ES	-	chr12:6450892-6450980	NA	-0.337	0.02475961249
			ES	-	chr12:6450473-6450708	NA	0.232	0.0496313164
CD274	3.414469829	2.86E-09	RI	+	chr9:5462833-5463121	NA	-0.167	0.000447323618
CDC25C	1.700115583	7.59E-07	ES	-	chr5:138329552-138329647	NA	0.277	4.21E-10
			MXE	-	chr5:138325814-138325904	chr5:138328483-138328529	-0.357	4.51E-08
			ES	-	chr5:138325814-138325904	NA	0.312	1.94E-06
			ES	-	chr5:138326020-138326054	NA	0.343	8.74E-06
			ES	-	chr5:138289479-138289563	NA	0.118	0.01782449163
CDC42SE1	-1.423495394	2.34E-08	ES	-	chr1:151056650-151056786	NA	-0.108	8.21E-09
CDK4	-1.080127317	0.02382768186	ES	-	chr12:57750441-57750563	NA	0.44	0.008685505951
CDK5RAP2	-1.249065616	1.84E-05	ES	-	chr9:120460571-120460667	NA	-0.271	3.89E-05
			ES	-	chr9:120460571-120460747	NA	-0.404	7.74E-05
CDK7	1.328809493	9.44E-05	ES	+	chr5:69254601-69254669	NA	0.114	0.01973663004
			ES	+	chr5:69254601-69254669	NA	0.131	0.02599262688
CDKL1	1.247930004	0.002650618585	ES	-	chr14:50332261-50332432	NA	0.34	2.97E-05
CENPA	1.254057718	0.000441941453	ES	+	chr2:26792755-26792833	NA	-0.12	5.47E-05
			ES	+	chr2:26792501-26792833	NA	-0.254	0.000514199217
CENPU	-1.734265274	1.29E-05	ES	-	chr4:184700819-184700881	NA	-0.484	0
			MXE	-	chr4:184697646-184697803	chr4:184700819-184700881	-0.15	1.65E-07
CENPV	6.07890014	5.41E-34	A3SS	-	chr17:16348615-16349333	chr17:16348615-16348685	-0.676	2.77E-12
			ES	-	chr17:16349139-16349333	NA	-0.682	5.11E-08
CEP192	1.04466848	0.01026137748	RI	+	chr18:13057584-13059312	NA	-0.287	0
			ES	+	chr18:13058293-13058435	NA	-0.104	1.04E-05
CEP41	-2.976284071	2.58E-19	ES	-	chr7:130400038-130400254	NA	0.189	7.13E-06
CEP57L1	1.105833171	0.02166298656	ES	+	chr6:109106034-109106090	NA	-0.427	0.000731961416

			ES	+	chr6:109155790-109155877	NA	-0.116	0.001692970305
			ES	+	chr6:109145218-109145381	NA	0.161	0.04168586939
CEP70	1.735715871	0.001458130752	A3SS	-	chr3:138572858-138572984	chr3:138572858-138572932	0.362	0.000477228309
CEP72	1.882470845	3.36E-07	ES	+	chr5:665940-666099	NA	-0.538	1.80E-07
CEP85L	-1.700958305	0.02196281938	MXE	-	chr6:118491685-118491865	chr6:118511297-118511415	0.153	0.04132591343
CFAP44	-1.662966401	0.001218901552	ES	-	chr3:113344515-113344712	NA	0.248	0.001534015222
			ES	-	chr3:113409105-113409322	NA	0.236	0.003084990753
			ES	-	chr3:113333405-113333583	NA	0.174	0.02483827348
CHFR	-4.3815182	1.70E-31	ES	-	chr12:132869618-132869834	NA	0.261	0.000138240809
			ES	-	chr12:132869618-132869798	NA	0.231	0.000242143471
			ES	-	chr12:132870723-132870783	NA	0.113	0.009067303247
CHN1	-1.329591775	0.000500387181	ES	-	chr2:174945169-174945261	NA	-0.138	0.009304957146
CHORDC1	1.807189398	0.000898165270	ES	-	chr11:90206747-90206810	NA	-0.12	3.29E-05
CHPF2	-1.997068952	0.001300539951	ES	+	chr7:151236955-151237172	NA	0.137	0.000336201988
CHURC1	-1.999784717	6.11E-05	ES	+	chr14:64926009-64926080	NA	0.109	9.60E-08
CIB1	1.975761493	2.09E-05	MXE	-	chr15:90232218-90232327	chr15:90233668-90233703	0.112	0.01287962329
CIRBP	-1.846245573	0.000636699871	RI	+	chr19:1271980-1274440	NA	-0.142	5.48E-11
			ES	+	chr19:1273493-1273715	NA	-0.369	9.30E-07
			ES	+	chr19:1273600-1273715	NA	-0.456	1.72E-06
			RI	+	chr19:1271980-1273172	NA	0.145	7.01E-05
CITED1	-4.037283125	1.35E-05	ES	-	chrX:72305301-72305422	NA	-0.169	0.00029790281
CIZ1	-1.126964478	0.01685817668	MXE	-	chr9:128187862-128187934	chr9:128190328-128190444	0.137	0.0168551311
CLASP1	-1.87962099	5.14E-07	ES	-	chr2:121409023-121409047	NA	-0.444	9.89E-07
			ES	-	chr2:121445448-121445496	NA	0.3	4.70E-05
CLCN2	-1.577793902	0.00326854886	A3SS	-	chr3:184354547-184354714	chr3:184354547-184354658	-0.555	3.27E-05
CLEC2B	2.653662736	9.42E-07	ES	-	chr12:9862498-9862573	NA	0.149	4.64E-06
CLEC2D	-1.568440398	0.000191776461	ES	+	chr12:9693824-9693948	NA	0.461	5.17E-05
			ES	+	chr12:9693415-9693948	NA	0.21	0.000432013183
			ES	+	chr12:9692827-9693948	NA	0.153	0.000987478066
			ES	+	chr12:9692827-9692931	NA	0.167	0.001097891832
			ES	+	chr12:9693033-9693115	NA	0.395	0.02024804826
CLK1	-1.121236214	0.01885873773	RI	-	chr2:200859679-200861466	NA	-0.38	5.85E-11
CLK4	-1.941223901	3.54E-06	RI	-	chr5:178616881-178618778	NA	-0.481	1.16E-13
			RI	-	chr5:178617343-178618778	NA	-0.336	9.82E-08
			A3SS	-	chr5:178619837-178620673	chr5:178619837-178619896	0.302	4.40E-05
			ES	-	chr5:178619837-178619896	NA	-0.17	0.000432013183
			ES	-	chr5:178619837-178620673	NA	-0.125	0.000805592955
			RI	-	chr5:178616881-178618778	NA	-0.325	0.00639004427
CLN6	-1.011937542	0.04139550145	A5SS	-	chr15:68211555-68211863	chr15:68211674-68211863	0.117	0.0269999003
CNTLN	-1.707947443	0.00709396116	MXE	+	chr9:17273732-17273866	chr9:17298189-17298352	-0.54	6.62E-10
COA1	-1.041327704	0.000316425810	ES	-	chr7:43650611-43650712	NA	0.256	0
			ES	-	chr7:43650492-43650712	NA	0.171	0
			ES	-	chr7:43656032-43656153	NA	-0.166	6.41E-13
			ES	-	chr7:43665657-43665722	NA	-0.299	5.25E-12
			ES	-	chr7:43650611-43650712	NA	0.536	8.02E-12
			MXE	-	chr7:43656032-43656153	chr7:43665657-43665722	0.198	1.72E-06
			ES	-	chr7:43650611-43650712	NA	0.425	1.83E-06
			ES	-	chr7:43665657-43665722	NA	0.29	1.78E-05
			ES	-	chr7:43656032-43656153	NA	-0.194	8.02E-05
			A5SS	-	chr7:43650492-43650712	chr7:43650611-43650712	-0.196	0.007807705261
COL12A1	3.37911517	0.002110433248	ES	-	chr6:75138319-75138340	NA	-0.133	0.006341438047
COL4A3BP	-1.373986406	0.03973000025	ES	-	chr5:75399309-75399387	NA	-0.146	2.38E-05
COMMD3	1.009450084	0.006532714084	RI	+	chr10:22318271-22318713	NA	-0.116	0.000248055687
COX11	-1.49664347	0.000561712389	A3SS	-	chr17:54961118-54962915	chr17:54961118-54961312	0.114	7.79E-06
CPEB2	-2.183944531	0.000149995678	A5SS	+	chr4:15007304-15007595	chr4:15007304-15007586	-0.18	0.000151410115
			ES	+	chr4:15040463-15040487	NA	-0.139	0.002054465018
CPM	-3.507635036	0.001723473239	A5SS	-	chr12:68933141-68933202	chr12:68933159-68933202	-0.432	5.28E-05
			A5SS	-	chr12:68933141-68933199	chr12:68933169-68933199	-0.431	0.01433723906
CPNE3	1.493605788	0.000288857600	ES	+	chr8:86524876-86524997	NA	0.406	4.50E-07
CROCCP2	1.589934001	0.000347377200	ES	-	chr1:16642766-16642850	NA	-0.292	0.0125683966

			MXE	-	chr1:16635026-16635168	chr1:16642766-16642850	-0.24	0.01637989376
			ES	-	chr1:16643027-16643145	NA	-0.19	0.0290614872
CSPP1	1.075755792	0.01232382156	ES	+	chr8:67172768-67172973	NA	0.222	2.44E-07
CTC-308K20.1	4.217809789	3.39E-10	RI	-	chr5:172957153-172958257	NA	-0.484	1.27E-07
CTC1	1.799988321	2.65E-06	RI	-	chr17:8229890-8230468	NA	0.204	0.001464590268
CTD-2341M24.	1.475568801	0.03769661099	ES	+	chr14:86128664-86128773	NA	-0.612	5.70E-05
CUL5	1.13192506	0.003545923711	ES	+	chr11:108104189-108104902	NA	-0.466	0
CYB5R2	4.606681285	7.55E-05	RI	-	chr11:7668477-7672523	NA	-0.382	0.003244746418
DAG1	-1.728892896	0.000233466817	ES	+	chr3:49470964-49471049	NA	0.562	2.12E-05
			ES	+	chr3:49487253-49487415	NA	0.199	0.002308161594
			A5SS	+	chr3:49476848-49476905	chr3:49476848-49476900	0.565	0.008667503029
			MXE	+	chr3:49470964-49471049	chr3:49476848-49476905	0.277	0.03468750359
DCLK2	-2.814230064	0.000336253814	ES	+	chr4:150253473-150253556	NA	0.488	7.92E-10
DDIT3	2.215673428	6.49E-05	RI	-	chr12:57517268-57517753	NA	0.147	0.00342269305
			A3SS	-	chr12:57517268-57517595	chr12:57517268-57517424	0.149	0.0401411959
			RI	-	chr12:57517268-57517753	NA	0.12	0.04122650279
DDR1	-3.42640979	0.001650917708	ES	+	chr6:30885624-30885680	NA	0.447	0.000114666020
			ES	+	chr6:30884537-30884710	NA	0.449	0.000141064512
			ES	+	chr6:30895403-30895514	NA	0.192	0.01899892488
DENND5A	-1.518925004	2.45E-06	ES	-	chr11:9176771-9176902	NA	0.13	3.99E-12
			ES	-	chr11:9143402-9143485	NA	-0.105	0.02294288612
			ES	-	chr11:9142721-9143485	NA	-0.117	0.02602112914
DGUOK	-1.026493349	0.006999614181	ES	+	chr2:73950626-73950732	NA	-0.113	1.12E-08
			MXE	+	chr2:73946718-73946906	chr2:73957124-73957240	0.163	3.19E-05
			ES	+	chr2:73950600-73950732	NA	-0.168	0.000113494923
			MXE	+	chr2:73946718-73946906	chr2:73950584-73950732	0.172	0.000147455171
			MXE	+	chr2:73946718-73946906	chr2:73957124-73957240	0.158	0.000238790189
			MXE	+	chr2:73938909-73939022	chr2:73957124-73957240	0.133	0.00191291694
			ES	+	chr2:73957124-73957240	NA	-0.204	0.002786142805
			ES	+	chr2:73950584-73950732	NA	-0.101	0.007035617093
			MXE	+	chr2:73938909-73939022	chr2:73950584-73950732	0.157	0.00925802358
DHX34	1.266315824	0.003350781558	ES	+	chr19:47376914-47376989	NA	-0.308	0.008437561811
DMTF1	-1.362356312	0.000684214166	ES	+	chr7:87194683-87194828	NA	0.205	4.66E-08
DNAJC4	2.04947275	4.07E-07	ES	+	chr11:64231031-64231147	NA	-0.445	0.03833779699
DNHD1	1.743757735	0.000175070153	RI	+	chr11:6497584-6498961	NA	-0.604	1.49E-08
DNM1	-1.940673073	0.02316430587	MXE	+	chr9:128224250-128224389	chr9:128226034-128226173	-0.438	2.11E-05
			ES	+	chr9:128240986-128241047	NA	-0.22	0.04612861764
DNM3	-2.772483874	0.000743564357	ES	+	chr1:172093695-172093725	NA	-0.149	0.001174848614
DNMT3A	-1.269829176	0.004865294804	ES	-	chr2:25239314-25239513	NA	0.13	0.001496409421
DOCK10	-5.317664304	5.21E-20	A3SS	-	chr2:224805407-224805529	chr2:224805407-224805502	0.187	0.004936945245
DOK1	-1.831450135	0.001437456887	ES	+	chr2:74555893-74556078	NA	0.164	5.43E-05
DOLPP1	-2.518584887	1.15E-06	ES	+	chr9:129086138-129086267	NA	0.101	0.01074850663
DOPEY1	-1.404679516	0.04143512895	MXE	+	chr6:83147235-83147291	chr6:83148758-83148863	-0.179	0.01456348202
DPM2	-1.183995468	0.03880813815	RI	-	chr9:127936552-127937523	NA	0.183	9.37E-07
DUS1L	1.350888534	0.03015202888	RI	-	chr17:82061208-82061721	NA	-0.208	0.002439817932
DYNC2H1	2.176882976	1.49E-10	ES	+	chr11:103287532-103287605	NA	-0.228	1.53E-05
DZANK1	-1.213273265	0.005928809403	A3SS	-	chr20:18412645-18412853	chr20:18412645-18412835	-0.791	1.24E-12
ECHDC2	6.681644148	1.60E-22	ES	-	chr1:52905033-52906611	NA	-0.209	0.000864396411
ECSIT	1.639205203	0.0017216827	ES	-	chr19:11519074-11519193	NA	-0.11	0.001119481664
EFHC1	2.799524328	1.29E-08	A3SS	+	chr6:52454061-52454287	chr6:52454094-52454287	0.24	0.000251187674
			ES	+	chr6:52488556-52488675	NA	-0.223	0.000645485187
			RI	+	chr6:52452687-52454287	NA	0.172	0.006772555752
EIF1AX	-1.34560509	0.004202077406	ES	-	chrX:20132181-20132263	NA	0.231	0.02363008235
EIF3E	2.189769702	6.97E-08	ES	-	chr8:108242111-108242346	NA	-0.195	0.000158041518
EIF4A2	1.027788198	0.01535765521	ES	+	chr3:186784961-186785101	NA	0.29	0
			RI	+	chr3:186784563-186786051	NA	0.153	4.57E-06
			MXE	+	chr3:186784961-186785101	chr3:186786163-186786273	0.108	3.42E-05
			ES	+	chr3:186788309-186788416	NA	-0.195	0.000992879108
			ES	+	chr3:186788309-186788420	NA	-0.222	0.00365172218
ELOVL5	2.212994865	4.89E-07	ES	-	chr6:53273219-53273344	NA	0.12	0

			MXE	-	chr6:53290094-53290189	chr6:53290400-53290535	-0.319	0.000453694653
			MXE	-	chr6:53287861-53287942	chr6:53290094-53290189	0.217	0.000926774312
			ES	-	chr6:53290400-53290535	NA	-0.395	0.003902246943
EMC8	-1.024324004	0.02758436408	ES	-	chr16:85780378-85780473	NA	0.164	1.58E-13
EMILIN2	-9.817925615	1.18E-35	MXE	+	chr18:2884963-2885139	chr18:2890560-2892486	-0.309	9.76E-05
EML1	-2.320998196	0.000182923670	ES	+	chr14:99914565-99914697	NA	0.141	0.01019963675
EMSY	1.149535574	0.001106280874	ES	+	chr11:76535894-76536059	NA	0.231	1.35E-06
ENGASE	1.344762342	0.02455274659	A5SS	+	chr17:79081897-79082877	chr17:79081897-79082063	0.365	1.43E-06
			ES	+	chr17:79083481-79083590	NA	0.201	0.0149867324
ENO3	-1.927766405	0.0259800486	ES	+	chr17:4952024-4952147	NA	0.139	0.00490899262
ENTPD1	-5.669436679	1.28E-06	ES	+	chr10:95842343-95842494	NA	0.413	0.000150335610
ENTPD6	1.140937453	0.006258935377	ES	+	chr20:25207075-25207397	NA	0.27	1.31E-05
			ES	+	chr20:25207078-25207397	NA	0.274	1.54E-05
			ES	+	chr20:25221231-25221333	NA	-0.148	0.000203737327
EOGT	2.54815863	4.75E-13	RI	-	chr3:68988505-68989017	NA	-0.162	0.04266975874
EPS8L2	3.084997859	2.89E-05	RI	+	chr11:709552-710486	NA	-0.571	1.48E-06
ERCC3	-1.077530913	0.000362829510	RI	-	chr2:127289336-127289824	NA	0.142	8.44E-05
ESYT2	1.269970749	1.59E-06	MXE	-	chr7:158748193-158748280	chr7:158749648-158749723	0.142	0
			ES	-	chr7:158752780-158752843	NA	0.166	0
			MXE	-	chr7:158737047-158737179	chr7:158739022-158739121	0.124	0.000613981788
			ES	-	chr7:158752780-158752843	NA	0.346	0.001829980388
ETNK2	1.587366752	9.65E-07	ES	-	chr1:204134514-204134694	NA	-0.161	0.03472346189
ETV1	-2.039385166	1.60E-07	ES	-	chr7:13989267-13989464	NA	0.291	9.08E-05
EVI5	-1.39108446	0.004666592538	ES	-	chr1:92663419-92663452	NA	-0.515	0.000226627216
EZH2	-1.393782233	5.05E-05	ES	-	chr7:148836821-148837000	NA	0.269	0.000373023368
FADS3	2.588280271	2.88E-09	RI	-	chr11:61876358-61876963	NA	0.185	0.009886221094
FAM107B	-2.376139257	0.000771160333	ES	-	chr10:14553321-14553387	NA	-0.202	0.01165258035
FAM111A	1.434909359	9.61E-05	A3SS	+	chr11:59148703-59148953	chr11:59148835-59148953	0.327	0.000441187003
			A3SS	+	chr11:59148796-59148953	chr11:59148835-59148953	0.215	0.003383676778
FAM120C	-3.415820507	1.51E-11	ES	-	chrX:54087754-54087964	NA	0.163	0.002895587267
			ES	-	chrX:54085714-54085916	NA	0.142	0.005509184309
FAM13B	-1.149549599	0.001024926722	ES	-	chr5:138018954-138019146	NA	-0.522	3.97E-08
			ES	-	chr5:137946227-137946311	NA	-0.339	3.76E-05
			ES	-	chr5:137956476-137956542	NA	-0.265	0.002442117802
FAM175A	1.108223786	0.0158683899	ES	-	chr4:83470202-83470396	NA	0.107	0.03069639954
FAM189B	1.18974639	0.01094929775	ES	-	chr1:155250275-155250929	NA	-0.18	0.001414702212
			ES	-	chr1:155250275-155250821	NA	-0.133	0.004324272947
			ES	-	chr1:155251094-155251164	NA	0.295	0.005179405283
			ES	-	chr1:155253858-155253978	NA	-0.24	0.008470168692
FAM208A	1.166974884	0.0186427278	A3SS	-	chr3:56633043-56633966	chr3:56633043-56633843	-0.155	2.29E-06
			A3SS	-	chr3:56633043-56633966	chr3:56633043-56633843	-0.224	4.33E-06
			ES	-	chr3:56638705-56638765	NA	-0.106	0.000113089307
FAM210A	2.304700909	0.000283937542	ES	-	chr18:13671861-13671973	NA	0.142	0.000175916401
FAM3A	1.15564421	0.01331123808	MXE	-	chrX:154511847-154511871	chrX:154512822-15451293[illegible]	-0.138	0.000112173190
			RI	-	chrX:154515759-154516242	NA	-0.192	0.00491760265
FAM49B	1.891508876	0.002892038074	MXE	-	chr8:129904498-129904585	chr8:129970942-129970995	0.151	0.001100968158
			ES	-	chr8:129904498-129904585	NA	-0.157	0.006151912774
			MXE	-	chr8:129879596-129879789	chr8:129896748-129896854	0.146	0.01128144421
FAM66C	-1.945290987	3.52E-05	ES	+	chr12:8195276-8195412	NA	0.396	0.02043424899
			ES	+	chr12:8187731-8188225	NA	-0.229	0.03557962837
FAM86B3P	1.958737718	7.41E-05	ES	+	chr8:8234482-8234584	NA	0.64	0.01525821969
FAM86C1	1.275477784	0.02080754254	MXE	+	chr11:71791738-71791819	chr11:71793379-71793481	-0.395	0.00664473448
			MXE	+	chr11:71791735-71791819	chr11:71793379-71793481	-0.231	0.01960597291
FAXDC2	-8.26761745	6.82E-33	A5SS	-	chr5:154823307-154823592	chr5:154823386-154823592	0.969	1.44E-12
FBXL2	1.808411585	0.01647970542	MXE	+	chr3:33297868-33297962	chr3:33364624-33364719	0.214	0.001031064798
			ES	+	chr3:33297868-33297962	NA	0.342	0.002142500663
			MXE	+	chr3:33297868-33297962	chr3:33359282-33359357	0.262	0.01098908084
FBXO24	1.407262694	0.03379967997	ES	+	chr7:100595101-100595223	NA	-0.414	6.11E-06
FCHSD1	2.542522501	5.64E-10	A3SS	-	chr5:141644858-141644942	chr5:141644858-141644938	-0.113	0.003195651006
FEZ2	1.606738363	0.003314191503	MXE	-	chr2:36555682-36555748	chr2:36558437-36558513	0.176	0

			MXE	-	chr2:36558437-36558513	chr2:36560784-36560865	0.131	0
			ES	-	chr2:36560784-36560865	NA	0.225	0
			ES	-	chr2:36560784-36560865	NA	-0.201	0.000209617447
			MXE	-	chr2:36555682-36555748	chr2:36558437-36558513	-0.108	0.003486529079
			ES	-	chr2:36560784-36560865	NA	0.386	0.006549900596
FGFR2	-1.679881674	0.0404314925	ES	-	chr10:121565636-121565704	NA	-0.539	0.008407926613
			ES	-	chr10:121565437-121565704	NA	-0.411	0.01160808791
FHL2	3.387355058	7.06E-05	ES	-	chr2:105396646-105396697	NA	-0.659	1.87E-07
			ES	-	chr2:105396646-105396697	NA	-0.59	0.02228946003
FIS1	-1.122048494	0.01266671626	ES	-	chr7:101244006-101244139	NA	0.152	0
FNIP2	-1.346893651	0.01328457477	ES	+	chr4:158833800-158833890	NA	-0.447	0.000114599384
FNTB	-1.997061983	1.60E-06	ES	+	chr14:65032358-65032521	NA	0.679	5.36E-09
FRG1BP	2.113799438	0.001486390724	ES	+	chr20:30391196-30391308	NA	0.796	0
FRG1CP	5.838530862	2.66E-23	MXE	-	chr20:28588642-28588757	chr20:28591393-28591519	-0.364	6.65E-05
FUS	-1.199979067	0.001206886185	RI	+	chr16:31184938-31188357	NA	-0.141	6.33E-13
FUZ	-5.489171703	6.01E-24	A5SS	-	chr19:49812446-49812736	chr19:49812614-49812736	0.806	1.10E-09
			A5SS	-	chr19:49812446-49812736	chr19:49812637-49812736	0.508	1.43E-06
			A5SS	-	chr19:49812614-49812736	chr19:49812637-49812736	0.214	0.000503434802
			ES	-	chr19:49812446-49812685	NA	0.212	0.001680905192
			ES	-	chr19:49812446-49812736	NA	0.142	0.001793405002
			MXE	-	chr19:49812250-49812335	chr19:49812446-49812736	0.511	0.006041335787
			A3SS	-	chr19:49811362-49811491	chr19:49811362-49811467	0.594	0.03982276621
FYN	-2.80300083	3.86E-21	MXE	-	chr6:111699514-111699670	chr6:111700103-111700268	-0.349	0
			MXE	-	chr6:111696276-111696456	chr6:111700103-111700268	-0.155	1.01E-06
			ES	-	chr6:111846588-111846629	NA	0.25	1.07E-06
GAA	1.240166167	0.0264254815	ES	+	chr17:80101810-80101890	NA	-0.367	8.37E-06
			A5SS	+	chr17:80101810-80101925	chr17:80101810-80101890	0.217	0.000394146704
GAB1	-3.007152327	1.63E-17	ES	+	chr4:143457679-143457769	NA	0.485	1.31E-05
GALNT7	-1.317781676	0.001760688009	MXE	+	chr4:173297794-173297977	chr4:173298114-173298297	-0.185	0.000488484178
			MXE	+	chr4:173297794-173297977	chr4:173298114-173298297	-0.152	0.008807758448
GAS5	1.714774757	0.001737349134	MXE	-	chr1:173865470-173865547	chr1:173865856-173865894	-0.266	0
			MXE	-	chr1:173865856-173865894	chr1:173866527-173866796	0.254	0
			MXE	-	chr1:173865856-173865894	chr1:173866176-173866206	0.232	0
			RI	-	chr1:173864256-173865282	NA	0.15	0
			MXE	-	chr1:173865509-173865547	chr1:173866176-173866206	0.233	0
			RI	-	chr1:173865228-173865547	NA	0.436	0
			A5SS	-	chr1:173865470-173865547	chr1:173865509-173865547	0.477	0
			MXE	-	chr1:173864674-173864704	chr1:173865228-173865282	-0.224	0
			MXE	-	chr1:173865509-173865547	chr1:173865856-173866206	0.274	0
			MXE	-	chr1:173864483-173864506	chr1:173865228-173865282	-0.124	6.96E-12
			MXE	-	chr1:173865509-173865547	chr1:173865856-173865894	0.154	2.88E-11
			MXE	-	chr1:173864674-173864704	chr1:173865228-173865282	0.206	5.01E-11
			RI	-	chr1:173864483-173864704	NA	0.206	4.03E-10
			MXE	-	chr1:173865470-173865547	chr1:173866176-173866206	-0.232	1.87E-09
			RI	-	chr1:173864256-173864506	NA	0.171	1.09E-08
			MXE	-	chr1:173865856-173865894	chr1:173866760-173866796	0.229	1.36E-07
			MXE	-	chr1:173865228-173865282	chr1:173865470-173865547	0.121	1.59E-05
			MXE	-	chr1:173865228-173865547	chr1:173866176-173866206	-0.198	2.31E-05
			MXE	-	chr1:173865509-173865547	chr1:173866527-173866567	0.214	6.74E-05
			MXE	-	chr1:173865470-173865547	chr1:173866527-173866567	-0.194	8.27E-05
			MXE	-	chr1:173865856-173865894	chr1:173866527-173866567	0.25	0.000377180693
			ES	-	chr1:173864674-173864704	NA	-0.223	0.000528708466
			MXE	-	chr1:173866760-173866796	chr1:173866990-173867043	-0.166	0.008854126289
GLS	-1.265784768	0.006881038643	ES	+	chr2:190913192-190913364	NA	-0.119	2.51E-07
			ES	+	chr2:190920214-190920248	NA	-0.395	0.000238932773
			MXE	+	chr2:190913192-190913364	chr2:190920214-190920248	0.17	0.000383469829
			ES	+	chr2:190913192-190913364	NA	0.115	0.001084209801
GMIP	1.243604304	0.03907621375	ES	-	chr19:19634506-19634703	NA	0.187	0.02076062402
GNRHR2	1.733210137	0.007101758843	RI	+	chr1:145919343-145919900	NA	-0.257	0.002073595908
GOLGA2	-2.170339054	3.30E-06	RI	-	chr9:128262562-128263092	NA	0.313	2.48E-07

			ES	-	chr9:128272784-128272865	NA	-0.197	0.04068278322
GOLT1B	1.587493058	0.000115319109	ES	+	chr12:21508382-21508561	NA	-0.101	6.35E-09
			ES	+	chr12:21507943-21508109	NA	-0.315	5.26E-07
GPBP1	-1.335598319	0.004324841391	ES	+	chr5:57237112-57237172	NA	-0.19	1.14E-08
GPCPD1	-2.600260486	2.66E-09	ES	-	chr20:5586193-5586269	NA	-0.46	0.003724555554
GPM6A	-4.887951774	9.76E-15	ES	-	chr4:175812190-175812249	NA	-0.649	2.67E-10
GPR155	-3.944168043	2.57E-07	ES	-	chr2:174482872-174482918	NA	-0.246	8.71E-05
			ES	-	chr2:174482872-174482918	NA	-0.231	0.000149459279
GPR157	1.555173699	0.01091927609	ES	-	chr1:9105485-9105680	NA	-0.412	0.00512657653
GPR158	-2.873095031	4.71E-17	MXE	+	chr10:25412249-25412473	chr10:25466650-25466719	-0.612	4.45E-12
GRB10	-3.972812942	1.02E-10	ES	-	chr7:50732271-50732368	NA	0.158	0.002538626055
			ES	-	chr7:50710874-50710990	NA	0.197	0.00375709255
			MXE	-	chr7:50732271-50732368	chr7:50755886-50756056	0.133	0.003775870552
			MXE	-	chr7:50710874-50710990	chr7:50755886-50756056	0.18	0.03407818423
GTF2IP1	-3.267517506	9.27E-11	ES	-	chr7:75202541-75202613	NA	0.111	0.0239620042
GULP1	-2.526194695	1.91E-09	ES	+	chr2:188584264-188584403	NA	-0.787	0
HACL1	1.526402891	0.00422753797	ES	-	chr3:15574976-15575082	NA	0.111	0.000315974689
HAUS4	1.0448497	0.01855474263	A5SS	-	chr14:22956745-22957094	chr14:22956915-22957094	0.171	0.03020364092
HCFC1R1	2.47347224	7.42E-07	ES	-	chr16:3023473-3023530	NA	-0.28	0.007123014945
HDLBP	-1.473395602	1.15E-05	ES	-	chr2:241269360-241269561	NA	0.129	2.19E-07
			A5SS	-	chr2:241269200-241269295	chr2:241269205-241269295	0.332	0.02387795607
			RI	-	chr2:241268953-241269295	NA	-0.137	0.04657411543
HECTD4	-1.04526215	0.003863815683	A5SS	-	chr12:112264083-112264212	chr12:112264089-11226421	0.156	0.04804249068
HIVEP2	2.651215167	2.24E-05	ES	-	chr6:142769551-142775125	NA	-0.156	0.000781848142
HJURP	1.609170662	1.92E-09	MXE	-	chr2:233844204-233844283	chr2:233849762-233849859	0.145	0.000382262424
			MXE	-	chr2:233845727-233845820	chr2:233847396-233847461	0.167	0.000982592194
HKR1	-1.432214895	0.001068682443	ES	+	chr19:37344609-37344774	NA	0.782	7.85E-07
			ES	+	chr19:37346887-37347052	NA	0.712	1.03E-06
			ES	+	chr19:37322184-37322232	NA	-0.262	0.001068047104
HLA-C	2.254181172	7.31E-07	MXE	-	chr6:31270209-31270485	chr6:31271072-31271331	0.117	5.70E-06
HLA-F	3.785726115	1.74E-15	RI	+	chr6:29726010-29726526	NA	0.379	0.000124961378
HLA-L	2.063913934	8.15E-05	ES	+	chr6:30262294-30262342	NA	-0.674	0.000182498683
HMG20B	1.561510133	0.000963527543	MXE	+	chr19:3575539-3575660	chr19:3576260-3576307	0.118	0.001067898129
HMGCS1	1.801653223	0.000539220527	ES	-	chr5:43307765-43307824	NA	-0.166	5.14E-13
HNRNPA2B1	-1.125577478	0.000740757125	ES	-	chr7:26190992-26191128	NA	-0.402	0
HSD11B1L	-1.169662346	0.04341275038	MXE	+	chr19:5684818-5684905	chr19:5684964-5685119	-0.557	0.007513422672
			MXE	+	chr19:5684818-5684905	chr19:5684964-5685119	-0.541	0.01372157933
HYI	4.717152425	4.55E-29	ES	-	chr1:43452902-43453004	NA	-0.24	0.02445759739
HYKK	2.240949091	0.01387365106	ES	+	chr15:78513083-78513425	NA	-0.122	0.01097352416
ICA1	1.844884327	0.001320099891	ES	-	chr7:8138984-8139047	NA	-0.216	5.39E-06
IFI27L1	-2.498331197	3.69E-07	ES	+	chr14:94097494-94097678	NA	0.265	0.006289332786
			ES	+	chr14:94097491-94097678	NA	0.249	0.007405164285
			MXE	+	chr14:94100738-94100771	chr14:94101813-94102059	-0.137	0.02602007324
IFRD1	-1.452438355	5.21E-05	MXE	+	chr7:112450507-112450782	chr7:112455762-112455867	0.193	0.000362523698
IFT88	-1.256084782	0.001525613517	MXE	+	chr13:20625749-20625849	chr13:20631015-20631102	0.315	2.93E-06
			ES	+	chr13:20663262-20663386	NA	-0.116	2.64E-05
			ES	+	chr13:20615792-20615879	NA	-0.231	0.000290026541
			ES	+	chr13:20631015-20631102	NA	-0.152	0.002607315509
			ES	+	chr13:20589810-20589867	NA	-0.139	0.02953166679
IGSF8	1.164869832	0.03614910135	A3SS	-	chr1:160091339-160091631	chr1:160091339-160091608	0.108	0.01220499947
IMPA2	1.554706474	0.000466620719	A5SS	+	chr18:12012169-12012349	chr18:12012169-12012215	-0.136	0.02239688901
ING4	-1.682920315	0.001655799854	ES	-	chr12:6655637-6655913	NA	0.405	0.001631986303
			RI	-	chr12:6652270-6652770	NA	0.184	0.001649350636
			RI	-	chr12:6652661-6653050	NA	0.142	0.004201701249
INIP	-1.00002656	0.002136516719	ES	-	chr9:112701787-112701880	NA	0.115	0.000135175814
			ES	-	chr9:112689526-112689617	NA	-0.193	0.000853548371
INO80C	2.350599073	7.35E-10	A3SS	-	chr18:35487385-35487823	chr18:35487385-35487439	0.679	3.18E-10
INTS3	1.059356499	0.000222119608	ES	+	chr1:153761019-153761109	NA	0.275	0
			ES	+	chr1:153753700-153753818	NA	-0.218	0.01176581464
INTS4	1.03261393	0.000588957399	ES	-	chr11:77910969-77911152	NA	0.154	0.000923038976

IQCK	-1.413293854	0.002214891262	MXE	+	chr16:19735352-19735450	chr16:19788837-19788922	-0.216	0.04786024144
ISG20	3.006849006	0.001931643352	A3SS	+	chr15:88650234-88652310	chr15:88652169-88652310	-0.128	0.004538961798
			A3SS	+	chr15:88650234-88652310	chr15:88652109-88652310	-0.225	0.0436367351
ISYNA1	1.59172281	0.01311051218	MXE	-	chr19:18436972-18437105	chr19:18437598-18437760	-0.472	3.96E-05
			ES	-	chr19:18437598-18437760	NA	-0.257	0.01175358981
ITPRIPL1	-1.43474733	0.000239711901	ES	+	chr2:96325746-96325849	NA	0.45	4.21E-05
JAM2	-3.00487969	0.01743735981	ES	+	chr21:25711366-25711568	NA	0.238	0
JMJD1C	-1.082624334	0.006579413059	ES	-	chr10:63215552-63215617	NA	-0.149	0.000182600247
			ES	-	chr10:63215552-63215696	NA	-0.103	0.000344036754
KANK1	-3.055429556	6.04E-06	A3SS	+	chr9:732377-734835	chr9:734747-734835	0.169	0.000542092763
KANK2	-1.675140318	0.002861384131	ES	-	chr19:11196049-11196227	NA	0.329	0.00167889463
KANSL2	-1.278645852	9.00E-06	RI	-	chr12:48681381-48682225	NA	-0.201	4.32E-05
			ES	-	chr12:48679035-48679150	NA	0.132	0.000684907752
			ES	-	chr12:48679035-48679238	NA	0.226	0.005663637832
KATNAL1	3.923730963	2.47E-29	ES	-	chr13:30255446-30255615	NA	0.107	0.000264591864
KATNAL2	-3.382314823	1.36E-07	ES	+	chr18:47046390-47046527	NA	0.121	0.01677792067
KCNAB3	-3.517345913	6.77E-08	RI	-	chr17:7923967-7924265	NA	-0.341	0.002842735607
			ES	-	chr17:7925096-7925183	NA	0.145	0.003016887814
KIAA1429	1.24880511	0.000462955549	MXE	-	chr8:94530962-94531085	chr8:94534838-94535007	0.112	0.02615574639
KIAA1551	1.587805706	0.000226575924	ES	+	chr12:31987238-31987322	NA	-0.143	0.000575324216
			ES	+	chr12:31970188-31970356	NA	-0.106	0.002983483307
			MXE	+	chr12:31960776-31960871	chr12:31980877-31985957	0.111	0.01001908489
KIF21A	-1.865969966	0.00651053706	ES	-	chr12:39330241-39330262	NA	-0.766	0
			A5SS	-	chr12:39321347-39322882	chr12:39322667-39322882	-0.441	0
			ES	-	chr12:39318072-39318201	NA	0.303	2.13E-08
			ES	-	chr12:39318072-39318201	NA	0.269	2.19E-06
			MXE	-	chr12:39315931-39315970	chr12:39318072-39318201	0.172	2.61E-06
			ES	-	chr12:39315931-39315970	NA	-0.222	7.86E-05
			MXE	-	chr12:39311414-39311553	chr12:39319905-39320013	-0.432	0.002295330497
KIF2A	-1.442609524	0.002363777415	A5SS	+	chr5:62352587-62352710	chr5:62352587-62352653	0.164	0.004282582856
KLC1	-1.135311744	0.01962575376	A5SS	+	chr14:103679383-103679545	chr14:103679383-10367951	-0.222	0.002423306488
			ES	+	chr14:103692358-103692425	NA	-0.228	0.003905473379
			ES	+	chr14:103684985-103685036	NA	0.248	0.01272844939
			A5SS	+	chr14:103679383-103679545	chr14:103679383-10367951	-0.171	0.02000464033
KLHDC1	-1.635443325	0.0484250351	MXE	+	chr14:49725685-49725769	chr14:49728925-49729009	0.409	0.001455592303
			ES	+	chr14:49728925-49729009	NA	-0.363	0.04492330258
KLHL24	-1.345531734	0.009388624109	ES	+	chr3:183636220-183636311	NA	-0.354	7.54E-08
			ES	+	chr3:183636220-183636311	NA	-0.322	0.000150335610
KLHL7	-1.719891504	4.28E-06	ES	+	chr7:23117833-23117964	NA	0.334	0.002563569625
KMT2C	-1.997615406	1.89E-07	A3SS	-	chr7:152156204-152156346	chr7:152156204-152156274	0.104	0.001747876875
KMT2E	-1.203849336	0.004965091321	ES	+	chr7:105110476-105110602	NA	0.199	3.54E-09
KRIT1	-1.068110366	0.003123809116	RI	-	chr7:92235402-92236542	NA	-0.14	9.18E-06
			ES	-	chr7:92244001-92244149	NA	0.288	1.62E-05
			MXE	-	chr7:92235402-92236542	chr7:92237666-92237759	0.128	0.006841950359
KRTCAP2	1.029343212	0.01280530371	MXE	-	chr1:155169790-155169857	chr1:155172564-155172892	0.12	6.32E-05
L3HYPDH	-1.752081738	0.009658583907	ES	-	chr14:59474438-59474526	NA	0.268	0.006032195647
			ES	-	chr14:59474438-59474526	NA	0.206	0.01072927059
L3MBTL3	1.434549104	0.006471405453	ES	+	chr6:130049650-130049830	NA	0.114	0.03879465813
LAS1L	1.782298979	3.15E-05	A5SS	-	chrX:65520701-65523707	chrX:65523559-65523707	-0.247	0
			MXE	-	chrX:65524055-65524262	chrX:65524563-65524614	-0.225	3.21E-08
			ES	-	chrX:65528259-65528369	NA	0.125	2.61E-07
			ES	-	chrX:65524563-65524614	NA	-0.365	2.58E-06
LCA5L	-1.622065567	0.02252697764	ES	-	chr21:39427758-39427905	NA	0.462	0.001839490346
			ES	-	chr21:39428171-39428503	NA	0.499	0.0264779633
LDLR	1.241552419	0.01911814173	MXE	+	chr19:11123173-11123344	chr19:11128007-11128085	0.134	0.008448894889
LETM2	1.639964307	0.006151047724	ES	+	chr8:38406945-38407038	NA	-0.437	1.41E-06
LIG4	-1.759531589	0.000706942462	ES	-	chr13:108214537-108214807	NA	0.237	7.32E-05
			ES	-	chr13:108214537-108214610	NA	0.167	0.000500771164
LINC00910	4.052061325	4.78E-14	ES	-	chr17:43387935-43388057	NA	0.294	2.51E-05
LINC01001	2.271547507	0.006862132394	ES	-	chr11:134563-134796	NA	0.533	0.01082847443

LINC01021	1.955564405	0.0116975285	ES	+	chr5:27477638-27477801	NA	-0.121	0.03820251916
LINC01778	-2.722293474	0.006404109579	MXE	+	chr1:30826554-30826652	chr1:30829747-30829882	-0.203	0.04357236015
LINC02210	1.019602373	0.03303933055	ES	+	chr17:45640388-45640455	NA	0.754	3.26E-07
LMAN2L	-1.398350486	0.004470000054	ES	-	chr2:96737136-96737217	NA	0.169	1.72E-06
LMNA	1.117541231	0.0454063878	RI	+	chr1:156137653-156138757	NA	-0.152	0.001867343234
LPCAT4	1.318391558	0.000325661230	ES	-	chr15:34361399-34361532	NA	0.164	0.000190521206
LPIN1	2.365586945	3.53E-09	ES	+	chr2:11776085-11776193	NA	0.162	0.000381103110
LRP2BP	-1.857891393	0.03534109477	ES	-	chr4:185378080-185378207	NA	0.311	0.01178140079
LRRC37B	-1.151313638	0.006355494958	ES	+	chr17:32045699-32045818	NA	0.181	0.000129012653
LRRC75B	-2.286462868	0.002054883078	ES	-	chr22:24588678-24589265	NA	-0.438	0.000475420331
			A3SS	-	chr22:24588213-24589393	chr22:24588213-24588329	-0.394	0.000962875699
LRRFIP1	1.871229807	5.98E-05	ES	+	chr2:237751199-237751271	NA	-0.265	0
LRRK2	-3.487581527	2.69E-15	ES	+	chr12:40313971-40314173	NA	0.211	0.000176395956
LTBP4	1.425418433	0.0250517973	ES	+	chr19:40622949-40623021	NA	-0.229	4.13E-11
			ES	+	chr19:40622949-40623021	NA	-0.236	1.52E-05
			ES	+	chr19:40617099-40617225	NA	0.159	0.005927429303
			ES	+	chr19:40616888-40617042	NA	0.21	0.03749535962
LUC7L2	-1.977428472	2.21E-11	ES	+	chr7:139375508-139375583	NA	-0.459	0.003536476151
MAGED2	-1.077604338	0.04959084528	RI	+	chrX:54815247-54816007	NA	0.171	3.60E-08
MAGI1	-1.938859083	0.002418317121	ES	-	chr3:65387142-65387226	NA	-0.5	4.47E-07
MAP4K4	-1.042399508	0.01087233698	MXE	+	chr2:101860824-101860986	chr2:101863820-101864051	0.393	0
			ES	+	chr2:101868028-101868037	NA	-0.441	0
			ES	+	chr2:101860824-101860986	NA	0.35	1.98E-11
			ES	+	chr2:101863820-101864051	NA	-0.319	2.64E-09
			ES	+	chr2:101871493-101871685	NA	0.161	7.79E-08
			A5SS	+	chr2:101887087-101887261	chr2:101887087-101887237	-0.102	2.13E-07
			ES	+	chr2:101860824-101860986	NA	0.384	6.13E-05
MAP7D1	-1.084772101	0.01396760647	ES	+	chr1:36174897-36175008	NA	-0.155	0.001511022672
MAPT	-1.08660315	0.004757739311	MXE	+	chr17:45971858-45971945	chr17:45978374-45978440	0.149	0.000529692575
			ES	+	chr17:45971858-45971945	NA	0.225	0.001677676425
MBD1	-1.128847187	0.009399627428	ES	-	chr18:50271468-50271540	NA	-0.415	0.000907332278
			ES	-	chr18:50273333-50273471	NA	0.124	0.009485303594
			ES	-	chr18:50273333-50273426	NA	0.161	0.01539580262
MBTD1	-1.252823664	0.000571616756	ES	-	chr17:51202021-51202077	NA	-0.178	0.008345359488
			ES	-	chr17:51193427-51193510	NA	-0.119	0.01245768692
MCOLN3	3.478039877	0.006921858627	ES	-	chr1:85036709-85036868	NA	0.648	3.05E-06
MCRIP2	1.157305055	0.03284819752	ES	+	chr16:646471-646608	NA	-0.358	0.003349315801
MEIS3	-1.979511956	0.02331342304	ES	-	chr19:47415050-47415101	NA	0.259	0.02092964191
MELTF	3.497884867	1.83E-12	MXE	-	chr3:197021403-197021471	chr3:197022956-197023113	-0.268	0.02020366208
METTL2B	-1.168119304	6.18E-05	ES	+	chr7:128477081-128477173	NA	0.255	4.97E-11
METTL4	1.536763995	1.67E-05	ES	-	chr18:2544652-2544759	NA	-0.245	4.20E-07
			ES	-	chr18:2544194-2544286	NA	-0.201	1.41E-06
			ES	-	chr18:2547354-2547529	NA	0.114	0.01235782639
MFSD10	1.631706667	0.01571849455	RI	-	chr4:2931374-2931936	NA	0.328	5.32E-07
			ES	-	chr4:2931554-2931663	NA	0.108	0.000747529734
MGAT5	-1.713395288	0.003319575422	ES	+	chr2:134253094-134253218	NA	0.373	0.01190796755
MGRN1	-1.610982278	0.002998999055	A3SS	+	chr16:4686258-4690974	chr16:4688795-4690974	-0.593	3.75E-10
			ES	+	chr16:4686258-4686303	NA	-0.582	1.44E-07
MICAL2	3.586544564	1.11E-12	ES	+	chr11:12249183-12249246	NA	0.136	0.02165860992
MIIP	1.563275249	0.004499888838	RI	+	chr1:12029222-12030124	NA	-0.328	0.008465546508
MINDY3	-1.208613357	0.007328234251	ES	-	chr10:15838227-15838279	NA	-0.52	2.85E-13
			ES	-	chr10:15816834-15816915	NA	0.114	0.03671992905
MIR99AHG	-3.934916524	1.08E-11	ES	+	chr21:16537378-16537439	NA	0.119	0.003127023316
MLF1	1.891544441	0.003629853842	ES	+	chr3:158588851-158588924	NA	-0.118	8.38E-08
			ES	+	chr3:158588851-158588924	NA	-0.16	3.81E-05
MRM2	-1.239198402	0.002862619913	ES	-	chr7:2238014-2238937	NA	-0.164	0.008753276415
MROH8	-1.737143816	0.01520324938	ES	-	chr20:37155062-37155167	NA	0.609	0.001277328111
MSI2	-2.047638975	1.02E-09	A3SS	+	chr17:57674917-57675126	chr17:57674971-57675126	-0.372	0.009154824571
MSRA	-2.529747678	2.81E-05	MXE	+	chr8:10207832-10207901	chr8:10245103-10245223	-0.15	2.77E-06
			MXE	+	chr8:10207832-10207901	chr8:10245103-10245223	-0.14	5.58E-06

MSRB3	2.358771729	6.61E-06	MXE	+	chr12:65308528-65308655	chr12:65326825-65326934	0.241	0.0416374328
MTMR11	3.003439045	8.69E-14	RI	-	chr1:149930364-149930965	NA	-0.421	0.000164408632
			ES	-	chr1:149931259-149931426	NA	-0.184	0.0223742272
MTMR9LP	-2.237656843	0.001420092965	ES	-	chr1:32234035-32234301	NA	0.18	0.04339536956
MTSS1L	-1.986879617	0.000287594815	ES	-	chr16:70665465-70665540	NA	-0.25	0.001167925376
MTTP	-5.89335954	4.46E-09	ES	+	chr4:99574808-99574970	NA	-0.745	3.17E-09
MTX1	1.439471995	0.000275591300	ES	+	chr1:155212126-155212219	NA	-0.196	0
			A5SS	+	chr1:155210547-155212219	chr1:155210547-155210627	-0.6	0
			RI	+	chr1:155210547-155212219	NA	-0.118	0.000173568272
MUTYH	-1.087860648	0.007820749729	ES	-	chr1:45332917-45332959	NA	0.303	0.01134847022
			RI	-	chr1:45332762-45333324	NA	0.151	0.01473339108
			A5SS	-	chr1:45332917-45333324	chr1:45333284-45333324	0.149	0.03557564081
MVD	1.930819022	0.001183642154	ES	-	chr16:88657145-88657247	NA	-0.523	0.000142670014
			A3SS	-	chr16:88656104-88656561	chr16:88656104-88656304	-0.376	0.01930354929
MYCBP2	-1.320982412	0.0258168125	ES	-	chr13:77103208-77103310	NA	-0.138	0.007740262278
MYEF2	-2.082989992	1.84E-06	ES	-	chr15:48151099-48151171	NA	0.243	2.46E-08
			MXE	-	chr15:48151472-48151571	chr15:48151873-48151942	0.163	0.001055792768
MYLK	1.500267615	0.02198623706	ES	-	chr3:123732895-123733102	NA	-0.244	0.000111282462
MYO15B	2.216823759	0.002319364735	RI	+	chr17:75590906-75591246	NA	-0.606	0.000440194364
MYO18A	-1.18297083	0.006777988076	ES	-	chr17:29085603-29085648	NA	-0.652	0
MYO5A	-1.8759313	0.0457881529	ES	-	chr15:52337809-52337884	NA	-0.362	0
N4BP2	-1.350727574	0.003290573016	MXE	+	chr4:40097226-40097569	chr4:40100035-40100126	0.26	0.01590946898
			ES	+	chr4:40100035-40100126	NA	-0.335	0.03898426062
N6AMT1	-1.464241785	6.41E-05	RI	-	chr21:28872190-28876491	NA	-0.205	0.01873364361
NAA16	-1.696138497	6.02E-08	ES	+	chr13:41336649-41336756	NA	0.191	0.000130332972
			ES	+	chr13:41336649-41336756	NA	0.165	0.005376307953
			ES	+	chr13:41355143-41355216	NA	0.106	0.009473724616
NAA60	-1.877030786	2.83E-05	A5SS	+	chr16:3484698-3485083	chr16:3484698-3485061	-0.189	0.000245554480
NAGLU	-1.421840041	0.0197509011	ES	+	chr17:42537397-42537545	NA	-0.267	0.002270954097
NAGS	4.321507059	4.13E-10	RI	+	chr17:44006528-44007494	NA	-0.583	2.47E-09
NAP1L1	1.336960592	0.000189389238	ES	-	chr12:76074202-76074239	NA	-0.129	1.61E-08
NAPEPLD	-2.582972854	0.000551482601	RI	-	chr7:103099775-103103554	NA	-0.376	0.002761239845
NARFL	1.394658197	0.000522081231	ES	-	chr16:740021-740103	NA	0.156	6.49E-08
NASP	-1.200291752	0.002166809433	ES	+	chr1:45607320-45608337	NA	-0.258	0.01542938513
			MXE	+	chr1:45594693-45594773	chr1:45600384-45600457	0.277	0.03478906968
NBPF26	2.644124877	8.36E-08	MXE	+	chr1:120834494-120834546	chr1:120836516-120836568	0.438	0.03649123668
NBPF8	2.38466	8.48E-09	MXE	+	chr1:120447093-120447166	chr1:120447629-120447841	0.575	1.47E-06
			ES	+	chr1:120447093-120447166	NA	0.448	4.38E-05
NDE1	-1.00806691	0.04055652833	MXE	+	chr16:15687374-15687511	chr16:15691143-15691323	-0.145	9.27E-05
			MXE	+	chr16:15651468-15651584	chr16:15651930-15652050	-0.124	0.03549421484
NDUFB2	-1.281500233	0.001782880306	ES	+	chr7:140700869-140700959	NA	0.623	7.31E-06
			ES	+	chr7:140700869-140700959	NA	0.561	1.31E-05
NDUFC1	1.149202519	0.00308601753	ES	-	chr4:139297384-139297443	NA	-0.122	0.02506932821
NDUFS8	1.679540398	0.006992665117	ES	+	chr11:68035627-68035788	NA	-0.114	1.32E-06
NEK8	1.273202264	0.008633506123	RI	+	chr17:28737305-28737736	NA	-0.113	0.0180385365
NEO1	-1.368746629	0.0110537643	ES	+	chr15:73293389-73293548	NA	0.392	1.17E-08
			A3SS	+	chr15:73270009-73270233	chr15:73270057-73270233	-0.215	0.000102151128
NEXN	3.064731182	7.17E-13	ES	+	chr1:77925187-77925229	NA	-0.687	4.41E-07
NF2	-1.93103303	4.81E-12	MXE	+	chr22:29668332-29668446	chr22:29673268-29673486	-0.623	0
NFE2L1	-1.284855613	0.02143014669	ES	+	chr17:48057031-48057121	NA	0.245	1.71E-08
NHLRC3	-1.972023136	6.78E-05	ES	+	chr13:39042104-39042305	NA	-0.36	5.79E-08
NHSL1	-1.657548669	3.47E-05	ES	-	chr6:138441982-138442114	NA	0.476	0.007436656522
NID2	-10.74102599	8.89E-21	RI	-	chr14:52005736-52006660	NA	0.884	1.29E-06
NKIRAS2	-1.596413971	0.000162448297	RI	+	chr17:42022398-42022640	NA	-0.163	5.64E-05
NMNAT3	2.730653499	0.000166470975	ES	-	chr3:139637962-139638062	NA	-0.251	0.0425199426
NOVA1-AS1	2.538877294	6.37E-07	ES	+	chr14:26797913-26798030	NA	-0.122	0.01371870462
NPR2	-3.536623637	1.98E-08	RI	+	chr9:35808508-35808853	NA	0.544	0.001284150908
NR2F1-AS1	-2.868865851	2.32E-06	ES	-	chr5:93438702-93438741	NA	0.288	0.004687272022
NRBP2	-1.153527038	0.04855267055	RI	-	chr8:143839508-143840030	NA	0.219	1.41E-12
NRM	-1.41478861	0.01307985467	ES	-	chr6:30689275-30689452	NA	-0.259	8.50E-12

			A5SS	-	chr6:30690024-30690243	chr6:30690046-30690243	-0.66	7.95E-05
NSUN5P1	-2.567205263	9.04E-09	ES	+	chr7:75411654-75411759	NA	-0.317	0.003212828364
			ES	+	chr7:75410605-75410726	NA	-0.256	0.005608000496
			A5SS	+	chr7:75415591-75415778	chr7:75415591-75415770	0.186	0.02639140838
NT5C3A	-3.028047386	7.01E-10	ES	-	chr7:33035933-33035988	NA	-0.264	0.008891469354
			MXE	-	chr7:33024038-33024108	chr7:33026816-33026915	0.177	0.04140530147
NUDT17	1.179491928	0.004580350482	RI	+	chr1:145846432-145846690	NA	-0.388	0.000299396112
			MXE	+	chr1:145846012-145846196	chr1:145846597-145846690	0.18	0.004181133627
NUDT2	2.454110123	6.04E-10	ES	+	chr9:34336228-34336341	NA	-0.193	0
NUDT22	1.845685503	1.64E-05	ES	+	chr11:64227567-64227666	NA	-0.367	3.01E-09
			ES	+	chr11:64229849-64230070	NA	0.145	0.000222018372
NUDT8	3.004048672	1.91E-06	RI	-	chr11:67628918-67629921	NA	-0.504	0.000634117199
NVL	1.127588771	0.000904930389	MXE	-	chr1:224232088-224232182	chr1:224232894-224232972	0.615	0.000384726908
OBSL1	-1.403701046	0.01240023565	RI	-	chr2:219550728-219551798	NA	0.388	0.03714903893
OGDH	-1.526948973	0.000370643214	MXE	+	chr7:44647443-44647534	chr7:44647656-44647759	0.358	0
			MXE	+	chr7:44647443-44647534	chr7:44647656-44647759	0.363	0
			MXE	+	chr7:44645326-44645518	chr7:44647656-44647759	0.168	0.002859687472
OPTN	1.759683322	0.000184078235	A3SS	+	chr10:13109096-13109288	chr10:13109111-13109288	-0.305	0.02544833074
ORMDL1	-1.110626883	0.001455323896	ES	-	chr2:189783013-189783123	NA	-0.422	0
			RI	-	chr2:189782421-189783123	NA	-0.168	0.002200528562
PACS2	-1.114402437	0.03768606891	RI	+	chr14:105383358-105384463	NA	-0.184	1.17E-07
PAK4	1.497362863	0.0117030675	ES	+	chr19:39168229-39168312	NA	-0.166	0.000241931653
			ES	+	chr19:39168229-39168302	NA	-0.16	0.000290026541
PANK4	1.300454609	0.002768792228	A3SS	-	chr1:2510677-2510782	chr1:2510677-2510748	0.32	0.004748089327
PAQR6	-2.277732777	5.05E-06	ES	-	chr1:156247509-156247632	NA	0.619	4.35E-07
PARD3	1.788626557	9.72E-09	ES	-	chr10:34336198-34336243	NA	0.427	1.53E-12
			ES	-	chr10:34372497-34372536	NA	-0.263	8.40E-06
PATJ	2.919373071	1.43E-08	ES	+	chr1:62100318-62100408	NA	-0.5	0.01919460471
PBRM1	-1.352841199	0.002059250533	A3SS	-	chr3:52679573-52679726	chr3:52679573-52679723	0.207	0.0011645449
			ES	-	chr3:52554723-52554879	NA	0.487	0.002131254622
			A3SS	-	chr3:52587352-52587510	chr3:52587352-52587435	0.106	0.007750127607
			ES	-	chr3:52554723-52554879	NA	0.286	0.008665320904
PCDHGC3	-8.35313208	1.06E-34	ES	+	chr5:141478393-141478546	NA	0.206	0.007191723509
PCGF3	-1.337546151	0.000276849824	ES	+	chr4:732399-732498	NA	-0.258	0.03122303962
			ES	+	chr4:732443-732498	NA	-0.204	0.04316493501
PCNX2	2.539563468	0.006367894852	RI	-	chr1:232984135-232986540	NA	-0.155	0.006928555429
			ES	-	chr1:233160282-233160433	NA	-0.131	0.01676038414
PDGFRA	-7.789961156	3.32E-21	MXE	+	chr4:54258756-54258817	chr4:54261094-54261412	-0.168	0.000306191906
PDLIM5	-1.044069279	0.03484552108	ES	+	chr4:94584985-94585003	NA	-0.172	2.58E-07
			A3SS	+	chr4:94575615-94576034	chr4:94575942-94576034	0.428	1.38E-06
PER3	-1.903743332	0.004294537408	RI	+	chr1:7829836-7830161	NA	0.148	0.000143573687
			ES	+	chr1:7787197-7787246	NA	-0.168	0.00357297092
			ES	+	chr1:7787197-7787246	NA	-0.575	0.003807338828
PEX2	1.294604673	0.004559218413	ES	-	chr8:76988306-76988338	NA	0.111	3.97E-08
PEX3	1.101002375	0.03515749002	ES	+	chr6:143472159-143472328	NA	-0.146	1.34E-05
PFAS	1.077632119	0.02410976757	A3SS	+	chr17:8266500-8266898	chr17:8266752-8266898	0.201	0.009613703767
PGAP1	-3.788491919	2.33E-11	ES	-	chr2:196892345-196892401	NA	-0.204	0.02951502951
PGGHG	1.615200075	0.008488698839	RI	+	chr11:290677-292095	NA	0.381	3.71E-07
			A3SS	+	chr11:290657-291113	chr11:290677-291113	0.517	2.86E-06
PHF7	1.178655745	0.01077870112	RI	+	chr3:52421647-52422881	NA	-0.515	0.000497736380
			MXE	+	chr3:52422221-52422338	chr3:52422759-52422881	0.14	0.00222168987
PHTF2	-2.110360977	1.56E-07	ES	+	chr7:77937709-77937838	NA	0.121	1.21E-07
PKIG	-3.052054967	0.000383770232	ES	+	chr20:44582584-44582731	NA	-0.416	0
			ES	+	chr20:44585099-44585204	NA	-0.289	0.002174269931
PLCB4	2.002095885	0.000180340697	ES	+	chr20:9373046-9373104	NA	0.482	0.000163314841
PLCD4	-4.835138045	0.000112753841	RI	+	chr2:218633604-218634221	NA	0.245	8.18E-06
PLEKHA4	-3.202731542	4.50E-10	MXE	-	chr19:48839204-48839263	chr19:48841148-48841310	0.396	0.01313028342
PLEKHA8	-1.634648153	0.001400125611	ES	+	chr7:30050433-30050474	NA	-0.397	4.16E-05
PLEKHH2	-2.333159992	2.95E-07	RI	+	chr2:43710488-43712383	NA	-0.327	0.000589824088
PLGRKT	1.75562491	7.40E-06	ES	-	chr9:5381862-5382031	NA	0.183	0.001850489502

PLOD2	-1.094339997	0.02385448137	ES	-	chr3:146077861-146077924	NA	-0.18	0.007990153349
PLSCR1	1.572589187	1.64E-05	RI	-	chr3:146521543-146522053	NA	-0.279	1.65E-09
			ES	-	chr3:146528613-146528831	NA	0.271	0.000434706440
			ES	-	chr3:146536539-146536565	NA	-0.146	0.03266616291
PLXNB1	-3.31053848	2.56E-12	RI	-	chr3:48410454-48411036	NA	0.641	0.01297814223
PNPLA6	1.908810733	0.000496194786	ES	+	chr19:7541983-7542067	NA	-0.137	0.002534240441
POGLUT1	-1.533434776	1.10E-05	ES	+	chr3:119478333-119478474	NA	0.363	0.01651821476
POLB	1.710448332	0.03165134489	ES	+	chr8:42352518-42352568	NA	-0.146	0.000121552777
POLD1	1.223461571	0.04540326691	RI	+	chr19:50401998-50402373	NA	-0.509	0.003199637451
			RI	+	chr19:50415723-50416528	NA	-0.266	0.005472713134
POLDIP3	-1.1845829	0.00411630455	ES	-	chr22:42601969-42602056	NA	-0.229	0.005634242534
			ES	-	chr22:42601969-42602107	NA	-0.263	0.007773580358
POLR1C	1.532565206	0.000636125678	RI	+	chr6:43519705-43520185	NA	-0.137	0.006280059241
POMZP3	-1.390858878	0.02538695052	RI	-	chr7:76618182-76618615	NA	0.259	0.01055453037
			A3SS	-	chr7:76611453-76611627	chr7:76611453-76611591	0.25	0.02937565073
POP4	2.558240582	3.06E-16	MXE	+	chr19:29610408-29610457	chr19:29611861-29611939	0.229	0.007333096958
PPFIBP1	-1.562329375	0.002152984153	ES	+	chr12:27658815-27658848	NA	-0.773	0
PPOX	1.082271124	0.01412079581	RI	+	chr1:161170906-161171218	NA	0.241	0.003244746418
			ES	+	chr1:161167370-161167486	NA	-0.156	0.04131087203
PPP1R12B	-1.091803732	0.01764074781	ES	+	chr1:202445045-202445228	NA	0.302	0.001936037329
			ES	+	chr1:202501858-202502005	NA	0.611	0.03813114754
			ES	+	chr1:202443086-202443127	NA	0.25	0.03843822808
PQLC3	1.511373337	6.02E-06	ES	+	chr2:11174967-11175009	NA	-0.307	6.68E-05
			ES	+	chr2:11174967-11175009	NA	-0.106	0.00284845948
			ES	+	chr2:11172750-11172824	NA	-0.284	0.005969129601
			MXE	+	chr2:11164203-11164261	chr2:11171924-11172045	-0.19	0.0108490355
PRKAB1	-1.386382525	6.65E-05	ES	+	chr12:119671494-119671668	NA	0.152	0.007309215227
PRKRIP1	-1.683699027	0.000656677814	ES	+	chr7:102395976-102396537	NA	0.361	2.37E-05
			MXE	+	chr7:102395976-102396537	chr7:102397619-102397698	0.196	0.000102901206
			ES	+	chr7:102395976-102396537	NA	0.301	0.000271826994
			ES	+	chr7:102397619-102397698	NA	0.122	0.0140171851
PRMT2	-3.181143107	5.87E-16	ES	+	chr21:46636438-46636547	NA	-0.729	0
			RI	+	chr21:46660832-46661936	NA	0.205	0.006535676432
PROCA1	-1.879371455	0.01774208688	ES	-	chr17:28706679-28706763	NA	0.381	0.000336973187
			A3SS	-	chr17:28704306-28704616	chr17:28704306-28704435	0.396	0.01603843118
PSMA3-AS1	-1.50008569	1.84E-05	ES	-	chr14:58293065-58293138	NA	0.417	1.58E-13
			ES	-	chr14:58288717-58288813	NA	-0.432	7.31E-06
			ES	-	chr14:58285590-58285756	NA	0.308	1.18E-05
			ES	-	chr14:58293765-58295811	NA	0.201	4.24E-05
			ES	-	chr14:58273978-58274135	NA	0.237	7.74E-05
			ES	-	chr14:58285590-58285756	NA	0.289	0.000525528662
			MXE	-	chr14:58273978-58274010	chr14:58285590-58285756	0.377	0.001136202548
			A3SS	-	chr14:58273978-58274135	chr14:58273978-58274010	0.23	0.00508214036
			MXE	-	chr14:58285590-58285756	chr14:58291634-58291707	-0.248	0.005483540331
			ES	-	chr14:58293313-58293544	NA	0.102	0.005554562012
PSTK	1.127310752	0.01195941578	ES	+	chr10:122986299-122986375	NA	0.104	0.04261741669
PTBP2	-1.71189802	0.000169340531	A3SS	+	chr1:96804784-96804939	chr1:96804799-96804939	0.389	0.03982276621
PTPMT1	-1.543709706	0.000558997362	ES	+	chr11:47569699-47569891	NA	0.32	0
PTPN4	-1.779165956	0.000652537197	ES	+	chr2:119881785-119881830	NA	0.222	5.64E-05
PTPRA	-1.998838714	1.10E-06	ES	+	chr20:2947981-2948024	NA	0.158	0.004696244476
PTPRD	-6.138160757	1.92E-12	ES	-	chr9:8437197-8437239	NA	-0.283	6.82E-05
PVT1	1.306446058	0.004494224275	ES	+	chr8:127983903-127984204	NA	0.173	1.02E-05
			ES	+	chr8:128099369-128099573	NA	0.182	2.16E-05
			MXE	+	chr8:127939507-127939676	chr8:127983903-127984204	-0.137	0.000100701606
			ES	+	chr8:127983903-127984204	NA	0.228	0.001136224867
			ES	+	chr8:128070159-128070272	NA	-0.138	0.003876184563
PXMP2	-2.741124337	4.98E-05	ES	+	chr12:132695883-132696046	NA	-0.16	0.03033077178
QKI	-2.873719689	4.43E-14	ES	+	chr6:163455278-163455421	NA	-0.424	0
			A3SS	+	chr6:163565945-163566795	chr6:163566720-163566795	-0.573	0
			RI	+	chr6:163563419-163570145	NA	0.602	3.34E-14

			A3SS	+	chr6:163564666-163570145	chr6:163565945-163570145	0.545	3.14E-09
			MXE	+	chr6:163534981-163535125	chr6:163561981-163562069	0.124	1.07E-08
			RI	+	chr6:163563419-163570145	NA	0.403	4.78E-07
			MXE	+	chr6:163534981-163535125	chr6:163561981-163562069	0.124	2.92E-06
			A3SS	+	chr6:163563419-163563719	chr6:163563443-163563719	0.127	1.90E-05
			MXE	+	chr6:163455278-163455421	chr6:163534981-163535125	-0.168	3.05E-05
RAB26	1.469735496	0.005051953101	ES	+	chr16:2151129-2151315	NA	0.161	0.002964538658
RAB3IP	1.710010051	0.007890185133	ES	+	chr12:69812777-69812877	NA	-0.262	6.76E-08
RABEPK	1.007530426	0.02016004222	ES	+	chr9:125213369-125213522	NA	0.256	2.92E-10
			ES	+	chr9:125220538-125220700	NA	0.194	3.05E-07
			MXE	+	chr9:125220538-125220700	chr9:125227909-125228059	0.116	0.004332221339
			MXE	+	chr9:125220538-125220700	chr9:125227909-125228059	0.102	0.01742082734
RABGAP1	-1.418405523	0.01182730268	ES	+	chr9:122945331-122945432	NA	0.112	0.001312112384
RABGEF1	1.725345461	0.001619465417	MXE	+	chr7:66739995-66740792	chr7:66771882-66772078	-0.192	7.65E-05
RAC1	-1.355164181	0.000244410226	ES	+	chr7:6398661-6398718	NA	0.261	4.82E-06
RAD51	1.432194582	0.000237839215	A5SS	+	chr15:40695173-40695425	chr15:40695173-40695321	-0.159	0.000245554480
RAD9A	1.305442359	0.0105912549	A3SS	+	chr11:67393690-67393790	chr11:67393722-67393790	0.407	0.02636430401
RALGAPA2	-1.489220243	9.40E-06	ES	-	chr20:20396694-20396734	NA	-0.421	0.0255521065
RAP1GAP	-1.748237365	0.000911836653	A3SS	-	chr1:21602803-21603912	chr1:21602803-21602913	-0.117	7.66E-09
			A3SS	-	chr1:21597960-21598095	chr1:21597960-21598040	0.321	0.001576710913
			A3SS	-	chr1:21597960-21598064	chr1:21597960-21598040	0.156	0.002071148324
RAPGEF2	-1.409371468	0.001218870822	ES	+	chr4:159344035-159344059	NA	-0.824	0
RB1CC1	1.242945521	0.02377184471	ES	-	chr8:52685936-52686156	NA	0.105	0.000140901881
RBM23	-1.47130468	5.57E-06	ES	-	chr14:22905605-22905659	NA	0.136	6.52E-08
			ES	-	chr14:22908332-22908380	NA	-0.228	1.15E-05
RBM26-AS1	-1.372105486	0.01108391598	ES	+	chr13:79417834-79417916	NA	0.207	0.0179359004
RBM6	-1.011106047	0.03993438768	ES	+	chr3:49972058-49972148	NA	-0.301	1.54E-10
			RI	+	chr3:50061461-50062108	NA	-0.347	1.98E-07
			ES	+	chr3:49975322-49975392	NA	-0.313	2.49E-06
			ES	+	chr3:49967197-49968748	NA	0.109	2.69E-05
			MXE	+	chr3:50048244-50048319	chr3:50054334-50054395	0.131	0.000205403459
			A3SS	+	chr3:49967197-49968748	chr3:49967469-49968748	0.154	0.02566195299
RCBTB2	-4.409763263	2.01E-15	ES	-	chr13:48524658-48524757	NA	-0.139	0.007914741791
RECQL	1.531731314	1.24E-05	ES	-	chr12:21501169-21501372	NA	-0.113	0.002987512401
			A3SS	-	chr12:21501169-21501372	chr12:21501169-21501215	-0.117	0.02462972179
			ES	-	chr12:21501169-21501215	NA	-0.14	0.03400412644
RFWD3	1.11757597	0.006083751041	ES	-	chr16:74664623-74664745	NA	0.289	0.04080879163
RGL1	-1.914148063	6.35E-06	MXE	+	chr1:183806374-183806485	chr1:183847565-183847774	-0.141	0.02946769912
RGMB-AS1	-2.82462874	0.02763328829	ES	-	chr5:98771655-98771766	NA	0.667	2.10E-07
RGS5	2.484751525	2.49E-05	ES	-	chr1:163319181-163319291	NA	-0.123	0.001702146197
			ES	-	chr1:163248529-163248598	NA	0.186	0.03328327983
RHOQ	-1.664656514	0.000497939125	ES	+	chr2:46543753-46543812	NA	-0.264	0.005789128017
RIDA	1.471036813	0.000227919518	ES	-	chr8:98108645-98108713	NA	0.106	0.00140484798
RIN2	1.964168698	0.00137101705	ES	+	chr20:19964951-19965024	NA	-0.162	0.001397451056
RIPK2	1.600186203	0.001553100893	ES	+	chr8:89762828-89762982	NA	-0.303	0.007952215332
RNF157	-3.845677675	8.58E-09	ES	-	chr17:76155251-76155317	NA	0.122	0.0145371809
RNF19B	2.087396167	6.12E-06	MXE	-	chr1:32942251-32942459	chr1:32944018-32944159	0.179	0.03033891155
RNF32	2.667459828	0.02334372343	A3SS	+	chr7:156676294-156677130	chr7:156676418-156677130	-0.385	0.002902268724
RNMT	1.890106209	1.51E-05	ES	+	chr18:13759941-13760208	NA	-0.162	0.000409380521
ROBO1	-1.805351697	0.000386310645	ES	-	chr3:78693347-78693356	NA	0.306	2.98E-07
RP11-1228E12	1.830938095	0.03593843095	ES	-	chr17:71365-71556	NA	0.667	0.001952422279
RP11-156P1.3	-1.092878188	0.03206607327	ES	-	chr17:47072471-47072559	NA	0.494	0.000788106904
			ES	-	chr17:47033277-47033381	NA	0.171	0.02472987573
RP4-639F20.1	2.662459679	8.82E-09	A3SS	+	chr1:94962924-94963270	chr1:94962978-94963270	0.159	0.02193666938
RPL12	1.429467442	7.62E-05	RI	-	chr9:127449609-127451405	NA	0.177	0.000119603100
RPL21	1.16409132	0.02213664948	MXE	+	chr13:27253764-27253843	chr13:27255241-27255349	0.201	0.006700381109
			MXE	+	chr13:27253764-27253843	chr13:27255241-27255354	0.205	0.006823004384
RPL3	1.104790604	0.004135022035	RI	-	chr22:39313190-39314208	NA	-0.286	0.000796534118
RPP40	-1.061085731	0.01746395472	ES	-	chr6:4998715-4998841	NA	0.204	6.11E-06
RPS27	1.340957822	0.000907220942	RI	+	chr1:153990762-153991034	NA	-0.166	2.22E-10

			ES	+	chr1:153990913-153991034	NA	0.186	0.04298664594
RPS3	1.164655539	0.00311883998	ES	+	chr11:75401639-75401733	NA	-0.255	0.001839342785
RPS6KB2	1.231233459	0.001936588515	MXE	+	chr11:67431367-67431515	chr11:67432352-67432492	0.107	0.000575271427
			MXE	+	chr11:67431367-67431515	chr11:67432355-67432492	0.108	0.000729447841
			ES	+	chr11:67432355-67432492	NA	-0.228	0.003857761711
			ES	+	chr11:67432352-67432492	NA	-0.222	0.00513160654
RPTOR	1.680011242	0.000254392584	ES	+	chr17:80925369-80925480	NA	0.262	0.04458274527
RTKN	-2.49647924	9.56E-10	ES	-	chr2:74439550-74439977	NA	-0.359	0.002527404038
RUFY3	-2.021686763	0.000133193976	MXE	+	chr4:70789494-70789592	chr4:70793764-70793904	-0.252	0.005649545712
SACS	2.252386019	8.15E-06	ES	-	chr13:23341444-23341690	NA	-0.213	1.17E-10
SBNO2	1.447993006	0.01511098396	ES	-	chr19:1147308-1147420	NA	-0.3	0.001484505828
SCHIP1	-1.896485882	0.00147597191	MXE	+	chr3:159866162-159866281	chr3:159886206-159886324	-0.247	0
SCLT1	1.401555349	0.01282257651	ES	-	chr4:128942995-128943188	NA	0.136	0.03286776765
SCYL1	1.545494851	0.003111621492	RI	+	chr11:65538269-65538704	NA	-0.15	0.005191038741
SEC14L2	-1.335052376	0.0185284686	ES	+	chr22:30415947-30416087	NA	0.154	0.000126474028
			ES	+	chr22:30415758-30416087	NA	0.117	0.000292565238
			ES	+	chr22:30415758-30415865	NA	0.109	0.000366701875
SEPT2	-1.010013484	0.0142321712	ES	+	chr2:241324408-241324662	NA	0.718	0
			MXE	+	chr2:241317498-241317599	chr2:241324408-241324662	-0.404	1.58E-05
			ES	+	chr2:241324408-241324662	NA	0.489	0.000119680357
			ES	+	chr2:241316421-241316540	NA	-0.447	0.001538786731
			ES	+	chr2:241317498-241317599	NA	-0.488	0.02613073178
SEPT7	-2.503100507	6.00E-13	ES	+	chr7:35831491-35832034	NA	-0.326	0
			ES	+	chr7:35831352-35831496	NA	-0.389	8.87E-05
			ES	+	chr7:35831424-35831496	NA	-0.371	0.000802562723
SEPT7P2	-1.5492593	0.000138111781	ES	-	chr7:45728831-45729026	NA	0.255	7.82E-11
SERHL2	-6.70343082	2.88E-06	ES	+	chr22:42573633-42573745	NA	0.73	1.31E-09
			A5SS	+	chr22:42571120-42571901	chr22:42571120-42571203	0.324	1.14E-05
			RI	+	chr22:42555663-42556083	NA	0.355	8.44E-05
SETD4	-1.309390555	0.000166057130	A3SS	-	chr21:36058815-36058973	chr21:36058815-36058924	0.195	0.03505499569
SETD9	-4.251404359	3.77E-17	ES	+	chr5:56911168-56911536	NA	0.363	3.97E-05
SH3BP2	-2.246801789	0.003933020251	A3SS	+	chr4:2831922-2832412	chr4:2832330-2832412	-0.38	0.01237140713
SIDT2	-1.229256527	0.01615281415	ES	+	chr11:117186989-117187001	NA	-0.102	0.001234249565
SIL1	1.740002194	7.11E-05	ES	-	chr5:139127943-139128052	NA	0.447	4.68E-06
SIPA1L3	1.621798139	0.009677210874	MXE	+	chr19:38119305-38119882	chr19:38130497-38130772	-0.122	0.008360219703
SIRT7	1.280458303	0.000419290467	RI	-	chr17:81914293-81915512	NA	-0.388	8.29E-05
SLC16A4	-5.396769728	2.81E-14	ES	-	chr1:110378852-110379356	NA	0.19	0.000400911488
SLC22A17	-1.4546415	0.02866036952	A5SS	-	chr14:23347459-23347731	chr14:23347513-23347731	-0.149	0.00896509776
SLC25A19	1.289248701	0.04036255058	A5SS	-	chr17:75288387-75288591	chr17:75288497-75288591	-0.227	8.47E-05
			A5SS	-	chr17:75288497-75288591	chr17:75288501-75288591	0.165	0.000713535124
SLC25A22	1.073915209	0.04186119758	ES	-	chr11:796042-796367	NA	-0.298	0.000390645863
SLC25A29	-2.248481258	1.87E-07	ES	-	chr14:100298026-100298190	NA	0.417	0.001127335634
SLC25A40	-1.147453659	0.004064043798	ES	-	chr7:87860071-87860169	NA	0.144	0.02566581821
SLC29A1	1.920056266	5.26E-09	MXE	+	chr6:44227262-44227342	chr6:44229389-44229471	-0.303	0
			MXE	+	chr6:44227262-44227342	chr6:44229389-44229471	0.274	4.53E-11
			ES	+	chr6:44225972-44226167	NA	-0.228	3.37E-06
			ES	+	chr6:44226060-44226167	NA	-0.312	1.18E-05
			ES	+	chr6:44226060-44226167	NA	-0.22	0.003873995416
			ES	+	chr6:44226060-44226167	NA	-0.201	0.01275158129
SLC2A11	-1.269476672	0.03262397763	ES	+	chr22:23877384-23877869	NA	-0.319	9.42E-06
			A3SS	+	chr22:23875084-23875241	chr22:23875120-23875241	-0.59	0.004351922584
SLC38A6	-1.146823099	0.03948874047	ES	+	chr14:61034018-61034113	NA	-0.104	0.001528473657
SLC38A9	-1.102937651	0.03174089186	MXE	-	chr5:55656714-55656774	chr5:55664692-55664863	0.106	0.02053550709
SLC3A2	1.460644012	0.00830256746	ES	+	chr11:62870744-62870837	NA	0.475	0
			ES	+	chr11:62871576-62871669	NA	0.132	0.03843822808
SLC47A1	1.87458136	0.008743852052	ES	+	chr17:19571477-19571572	NA	0.191	0.001141438719
SLC50A1	1.565384987	6.64E-05	ES	+	chr1:155136827-155136951	NA	0.332	1.70E-06
			ES	+	chr1:155136827-155136951	NA	0.296	0.000110375191
			ES	+	chr1:155137560-155137722	NA	0.133	0.000352212672
			ES	+	chr1:155137578-155137722	NA	0.148	0.000665949902

SLC7A11	2.225019393	1.05E-05	ES	-	chr4:138185120-138185244	NA	-0.542	9.09E-08
			ES	-	chr4:138223198-138223324	NA	-0.163	0.000199745017
SMARCC2	-1.063520582	0.000926498163	A5SS	-	chr12:56164302-56164731	chr12:56164647-56164731	0.43	1.46E-05
			ES	-	chr12:56172936-56173029	NA	-0.191	2.01E-05
			ES	-	chr12:56164302-56164368	NA	0.483	0.000311022291
SMTN	2.681079236	1.62E-07	A3SS	+	chr22:31088453-31088606	chr22:31088512-31088606	0.149	0.00185771104
			ES	+	chr22:31080449-31080754	NA	-0.259	0.002442197371
SNHG11	-1.126572362	0.02635014089	ES	+	chr20:38447929-38448093	NA	-0.161	0.02738999534
SNHG3	2.007679738	9.72E-05	ES	+	chr1:28508127-28508160	NA	0.173	0.0111445589
SNHG6	1.619245894	1.16E-05	ES	-	chr8:66922613-66922725	NA	-0.151	0
SNHG8	3.401142657	2.65E-08	A5SS	+	chr4:118278708-118279137	chr4:118278708-118278792	-0.114	0.000990167683
SNX11	-1.34865531	0.00189522751	ES	+	chr17:48110707-48110767	NA	-0.122	4.93E-05
SON	-1.19079563	8.55E-06	ES	+	chr21:33572550-33572630	NA	-0.263	0
SP110	1.142791827	0.001863938051	ES	-	chr2:230216780-230216821	NA	-0.134	0.04267665388
SP140L	2.932572231	1.28E-12	ES	+	chr2:230328756-230328831	NA	-0.363	0.000167484406
SPAG1	4.676396214	1.16E-23	ES	+	chr8:100220278-100220431	NA	-0.111	0.003664403339
			MXE	+	chr8:100213818-100213918	chr8:100220278-100220431	0.178	0.03855224577
SPAG5	1.444661329	3.84E-05	RI	-	chr17:28579750-28580120	NA	-0.101	2.69E-09
SPATA4	3.165186094	0.003400696606	ES	-	chr4:176192957-176193076	NA	-0.321	0.00010612589
SPATS2	1.474620984	0.000463250775	ES	+	chr12:49371227-49371290	NA	-0.28	1.33E-05
SPEF2	3.786749972	5.41E-08	ES	+	chr5:35644354-35644525	NA	-0.32	9.18E-05
SPIN3	-1.02601748	0.02299982084	A3SS	-	chrX:56978579-56978762	chrX:56978579-56978660	0.322	5.15E-06
			A3SS	-	chrX:56978579-56978747	chrX:56978579-56978660	0.322	1.29E-05
			A3SS	-	chrX:56978579-56978751	chrX:56978579-56978660	0.318	1.35E-05
SPPL2A	-1.1827833	0.002553452309	ES	-	chr15:50726072-50726126	NA	0.135	0.01789049185
SPRY1	-3.912389351	0.000339711427	ES	+	chr4:123397610-123397856	NA	0.155	0.000870311140
SREBF1	1.511079099	0.01662573772	ES	-	chr17:17817547-17817591	NA	0.375	0.0013265261
			ES	-	chr17:17823517-17823607	NA	0.302	0.008461191506
			RI	-	chr17:17817547-17817916	NA	-0.223	0.01127882976
SRSF6	-1.135706591	3.91E-05	ES	+	chr20:43459152-43459420	NA	-0.11	0.000938808030
SS18	-1.026121465	0.000811369334	MXE	-	chr18:26080304-26080423	chr18:26084051-26084175	-0.557	9.01E-05
			MXE	-	chr18:26080304-26080423	chr18:26082362-26082528	-0.419	0.000165960160
			ES	-	chr18:26035830-26035923	NA	0.123	0.003304618959
SSH1	-1.210330621	0.02404780723	ES	-	chr12:108802321-108802368	NA	-0.175	0.0250286001
ST20	1.093934916	0.02882289017	ES	-	chr15:79907628-79907684	NA	-1	0
ST3GAL5	-3.300851484	0.000588957399	MXE	-	chr2:85851263-85851703	chr2:85852859-85853099	-0.153	0.000619848871
			MXE	-	chr2:85851263-85851703	chr2:85852859-85853091	-0.148	0.000836217543
			MXE	-	chr2:85851263-85851703	chr2:85852859-85853000	-0.212	0.001463632539
			ES	-	chr2:85870071-85870258	NA	0.324	0.02209414026
ST3GAL6	-2.436643253	2.20E-10	ES	+	chr3:98756368-98756540	NA	0.151	0.02805536072
ST5	-3.325106723	1.61E-11	ES	-	chr11:8729949-8731209	NA	0.325	0.000714324807
			A3SS	-	chr11:8702571-8703611	chr11:8702571-8702720	-0.104	0.01220499947
STAG3L3	-1.758547837	0.004817898663	ES	-	chr7:73000357-73000481	NA	-0.569	0.000438379529
STARD4	2.836661212	7.11E-13	ES	-	chr5:111501961-111502088	NA	0.133	0.02770700481
STAT2	-1.017569891	0.000642882523	RI	-	chr12:56354777-56355351	NA	-0.129	0.0174877641
STYXL1	-2.704520263	1.01E-05	ES	-	chr7:76021850-76021992	NA	0.112	0.003498861351
			ES	-	chr7:76013741-76013887	NA	0.105	0.003523797492
SUSD1	-3.382783751	2.94E-05	ES	-	chr9:112042100-112042165	NA	-0.11	0.01062440517
SVIL	-2.159804119	0.000607367883	ES	-	chr10:29532001-29532172	NA	-0.376	2.63E-06
			ES	-	chr10:29526960-29527056	NA	-0.202	7.64E-06
			ES	-	chr10:29535988-29536069	NA	-0.202	0.04292658489
SYNE2	-2.479573527	1.14E-05	ES	+	chr14:64098746-64098821	NA	0.232	0.000938468137
SYNJ1	-1.590950773	3.08E-05	ES	-	chr21:32634860-32634884	NA	-0.634	4.13E-11
			ES	-	chr21:32634860-32634881	NA	-0.289	6.83E-05
TADA2A	-1.238555727	0.000336288305	MXE	+	chr17:37426949-37427009	chr17:37437737-37437922	-0.353	0.000772029136
			ES	+	chr17:37423508-37423615	NA	0.112	0.007108285273
TAF1D	1.84307435	0.000189951980	A5SS	-	chr11:93736693-93737239	chr11:93737063-93737239	-0.127	1.32E-10
TAF2	1.180512274	0.005026043336	ES	-	chr8:119744880-119745036	NA	0.283	1.62E-08
TANC1	-2.836249914	7.73E-14	RI	+	chr2:159219237-159219867	NA	-0.182	0.01003146764
TAPT1	-1.490575276	0.000430933133	ES	-	chr4:16170652-16170729	NA	-0.159	0.01024382028

TATDN1	3.26461441	1.65E-12	ES	-	chr8:124522150-124522200	NA	0.101	0.002762253283
TAX1BP1	-1.020367875	0.01765393428	A5SS	+	chr7:27816348-27816520	chr7:27816348-27816394	0.103	5.39E-05
TBC1D31	2.526802507	8.82E-14	ES	+	chr8:123082701-123082817	NA	-0.101	0.000452081202
TBC1D32	2.354456866	0.000132871583	ES	-	chr6:121090852-121091041	NA	-0.271	1.21E-05
			ES	-	chr6:121299445-121299505	NA	-0.161	0.000571601245
			ES	-	chr6:121255327-121255410	NA	-0.174	0.000938515980
			A3SS	-	chr6:121115171-121115747	chr6:121115171-121115241	0.264	0.03686690828
TBC1D7	-1.645422456	3.55E-05	ES	-	chr6:13325093-13325174	NA	0.194	0.01448293753
TBC1D8B	2.272984517	6.35E-08	ES	+	chrX:106839325-106839457	NA	-0.271	4.34E-05
			ES	+	chrX:106839307-106839457	NA	-0.193	0.000257758414
TBC1D9B	1.363305773	0.004627839498	ES	-	chr5:179867444-179867495	NA	-0.158	0
TBL2	-1.483785536	0.000778199389	ES	-	chr7:73576540-73576701	NA	-0.221	0.006525933172
TCEA1	1.021016919	0.02475288636	ES	-	chr8:54002891-54003092	NA	-0.308	0.000990626250
			ES	-	chr8:54002891-54003086	NA	-0.261	0.0075854768
			ES	-	chr8:54002891-54002940	NA	-0.315	0.01533786035
TCEAL8	-1.00031066	0.04956146826	ES	-	chrX:103254604-103254677	NA	0.212	6.41E-13
TCF4	-6.396944488	9.15E-25	A5SS	-	chr18:55232508-55232671	chr18:55232520-55232671	0.431	5.01E-06
			RI	-	chr18:55228846-55232671	NA	0.753	7.37E-05
			RI	-	chr18:55228846-55232671	NA	-0.136	0.000666479782
TCF7L2	-2.759525068	0.001075604654	ES	+	chr10:113160618-113160691	NA	0.805	0.000153796721
			MXE	+	chr10:113159919-113159992	chr10:113160618-11316069	-0.684	0.000707600869
			ES	+	chr10:113159919-113159992	NA	-0.415	0.002988281976
TCOF1	2.715985488	2.38E-08	ES	+	chr5:150379228-150379408	NA	0.127	2.19E-11
			ES	+	chr5:150372005-150372236	NA	0.13	0.003432257142
TCTN1	-1.353109434	0.001925945779	ES	+	chr12:110644966-110645129	NA	0.225	0.000867862258
			ES	+	chr12:110644966-110645143	NA	0.283	0.002401590692
			ES	+	chr12:110634395-110634463	NA	0.353	0.005171587045
			A3SS	+	chr12:110647195-110647336	chr12:110647210-11064733	0.456	0.009968353808
TCTN2	-1.430464998	0.01907818385	ES	+	chr12:123694841-123694976	NA	0.273	5.84E-06
TEAD2	-2.08855701	0.003726359863	ES	-	chr19:49355958-49355970	NA	-0.145	0.000154946153
			ES	-	chr19:49359843-49360081	NA	-0.101	0.000362805466
TEAD4	1.779013698	0.001677124986	ES	+	chr12:2959947-2960353	NA	-0.59	7.68E-06
TET2	-1.726384486	7.26E-05	ES	+	chr4:105241338-105241429	NA	0.147	0.005143745186
			ES	+	chr4:105190359-105190505	NA	0.155	0.02398359823
TFDP1	-1.671615367	0.007794169846	MXE	+	chr13:113610995-113611062	chr13:113631622-11363174	-0.13	0.000762542582
			MXE	+	chr13:113610995-113611062	chr13:113623179-11362328	-0.147	0.001750191041
			ES	+	chr13:113631622-113631905	NA	0.13	0.01092216805
TGFB3	-2.174138043	7.01E-06	ES	-	chr14:75960268-75960386	NA	-0.128	0.009293494939
THAP5	-1.078117063	0.008204994159	ES	-	chr7:108565829-108566022	NA	0.157	0.000545223317
			ES	-	chr7:108565951-108566022	NA	0.278	0.000942157194
THRB	-2.247924883	0.04737934441	ES	-	chr3:24297225-24297371	NA	-0.304	0.005335128453
TIA1	-1.927848441	3.09E-06	ES	-	chr2:70229058-70229091	NA	-0.349	1.08E-13
			ES	-	chr2:70228343-70228462	NA	-0.465	4.53E-11
			A5SS	-	chr2:70216752-70216994	chr2:70216885-70216994	0.191	1.98E-07
TIAM1	-2.07132173	0.00924211985	ES	-	chr21:31276731-31276908	NA	-0.388	0.02905195337
TJP1	-1.630312674	0.004748682988	ES	-	chr15:29719776-29720016	NA	-0.238	2.38E-05
TKFC	1.504730346	0.000541183066	MXE	+	chr11:61335839-61335937	chr11:61336203-61336390	-0.446	0.000306133701
			ES	+	chr11:61343341-61343458	NA	-0.109	0.002348355715
			ES	+	chr11:61335839-61335937	NA	-0.113	0.03969952441
TKT	1.141137656	0.0377038072	A3SS	-	chr3:53237796-53237921	chr3:53237796-53237820	-0.501	1.07E-07
TMA16	1.194177342	0.01931921015	MXE	+	chr4:163514073-163514158	chr4:163515312-163515461	-0.108	0.005271854269
TMEM117	-1.419785806	0.000325336688	ES	+	chr12:44143524-44143624	NA	-0.285	0.006170354981
TMEM161A	1.158196105	0.03903221258	ES	-	chr19:19134783-19134916	NA	-0.602	4.20E-05
			MXE	-	chr19:19133129-19133210	chr19:19134783-19134887	0.142	0.002295330497
			ES	-	chr19:19134783-19134887	NA	-0.228	0.03346111353
TMEM178A	-4.806759992	2.90E-05	ES	+	chr2:39707048-39707186	NA	0.269	2.40E-05
TMEM198B	-1.744418197	0.000443742202	RI	+	chr12:55830840-55831349	NA	0.33	1.84E-08
			A3SS	+	chr12:55832870-55833014	chr12:55832958-55833014	0.387	4.38E-06
			A5SS	+	chr12:55832958-55833038	chr12:55832958-55833014	0.385	0.000399128713
			RI	+	chr12:55832958-55833541	NA	0.151	0.000400561250

TMEM267	1.174500718	0.02200779639	ES	-	chr5:43479889-43479987	NA	-0.131	0.04751393873
TMEM39A	1.114901907	0.000123257389	ES	-	chr3:119435754-119435935	NA	-0.321	2.44E-10
TMEM63B	1.061736475	0.003785927863	ES	+	chr6:44135327-44135366	NA	0.149	2.00E-05
			MXE	+	chr6:44150563-44150629	chr6:44151845-44152008	-0.191	0.002658984414
TMEM80	-1.310460343	0.007157746281	RI	+	chr11:698868-700235	NA	0.241	9.73E-06
TMEM87B	1.286252218	0.005893794913	ES	+	chr2:112074911-112074962	NA	-0.152	0.004874200486
			ES	+	chr2:112077191-112077282	NA	-0.121	0.007276760501
TMTC4	-1.460991474	0.000449087764	ES	-	chr13:100670359-100670569	NA	0.222	1.46E-06
			ES	-	chr13:100670282-100670569	NA	0.327	1.64E-06
TNC	-7.489376663	1.49E-10	MXE	-	chr9:115048259-115048532	chr9:115059729-115060002	0.464	3.13E-05
			MXE	-	chr9:115048259-115048532	chr9:115059729-115060002	0.416	5.63E-05
			ES	-	chr9:115048259-115048532	NA	-0.601	0.000541520201
			MXE	-	chr9:115048259-115048532	chr9:115064646-115064919	0.187	0.03274388609
			MXE	-	chr9:115046409-115046682	chr9:115048259-115048532	-0.285	0.04224761506
TNFRSF12A	2.04597705	0.04984552332	ES	+	chr16:3021188-3021319	NA	-0.144	0.001101883147
			ES	+	chr16:3021214-3021319	NA	-0.123	0.001898049618
TNFSF13	-2.944961944	0.03695774867	MXE	+	chr17:7559623-7559702	chr17:7559850-7559893	-0.476	3.35E-06
			MXE	+	chr17:7559623-7559702	chr17:7559845-7559893	-0.405	9.38E-06
TNIK	-2.903496877	0.001557595646	ES	-	chr3:171139469-171139556	NA	-0.604	3.94E-11
			ES	-	chr3:171128713-171128878	NA	0.129	0.00168795208
TOP1MT	1.130399805	0.01595215506	ES	-	chr8:143332486-143332657	NA	-0.317	1.32E-09
			ES	-	chr8:143334107-143334285	NA	-0.35	1.38E-06
			ES	-	chr8:143332486-143332601	NA	-0.232	8.45E-05
TOX2	1.841117966	0.01826355756	ES	+	chr20:44065711-44066107	NA	-0.576	0.02126672239
TPGS2	-1.401330585	7.91E-05	ES	-	chr18:36805373-36805397	NA	0.211	9.62E-09
TPK1	1.484219136	0.000152871539	ES	-	chr7:144681806-144681864	NA	-0.484	0.01202735009
TPM2	4.718007161	1.31E-21	MXE	-	chr9:35684731-35684807	chr9:35685063-35685139	0.365	8.39E-13
			RI	-	chr9:35684487-35685139	NA	-0.633	3.04E-07
TPT1-AS1	-2.889017128	3.55E-10	MXE	+	chr13:45378529-45378671	chr13:45383090-45383235	-0.625	6.05E-07
			MXE	+	chr13:45379194-45379335	chr13:45383090-45383235	-0.547	3.37E-06
			A3SS	+	chr13:45390713-45390902	chr13:45390757-45390902	0.12	1.61E-05
			ES	+	chr13:45390757-45390902	NA	0.148	1.99E-05
			ES	+	chr13:45390713-45390902	NA	0.109	2.67E-05
			MXE	+	chr13:45379194-45379335	chr13:45383090-45383235	-0.37	3.21E-05
			MXE	+	chr13:45379925-45380001	chr13:45383090-45383235	-0.293	8.84E-05
			MXE	+	chr13:45379925-45380001	chr13:45383090-45383235	-0.308	9.27E-05
			MXE	+	chr13:45379194-45380001	chr13:45383090-45383235	-0.377	0.000188143965
			A3SS	+	chr13:45377060-45377215	chr13:45377139-45377215	0.22	0.00019763891
			MXE	+	chr13:45379194-45380001	chr13:45383090-45383235	-0.397	0.000310183700
			ES	+	chr13:45360140-45360239	NA	0.619	0.004168487247
			MXE	+	chr13:45383090-45383235	chr13:45389734-45389820	0.107	0.018188216
			MXE	+	chr13:45379898-45380001	chr13:45383090-45383235	-0.116	0.03030437867
TRABD	1.696639361	0.005296480166	RI	+	chr22:50197822-50198186	NA	-0.409	0.000651342608
TRABD2A	4.547917093	4.49E-09	MXE	-	chr2:84839148-84839323	chr2:84841860-84842007	-0.25	0.03053270545
TRAF3IP2-AS1	-1.702715019	3.10E-05	ES	+	chr6:111574620-111574658	NA	-0.317	0.01210955425
TRIM22	-3.201786811	0.003809392918	RI	+	chr11:5697247-5698545	NA	0.236	0.03643105134
TRIM41	1.089862148	0.01306965592	ES	+	chr5:181233412-181233435	NA	-0.132	0.00683098948
TRIM9	-3.555181903	2.26E-08	ES	-	chr14:51000682-51000828	NA	0.253	0.000645221412
TRIQK	1.513918681	0.000522520545	MXE	-	chr8:92921502-92921660	chr8:92954405-92954564	-0.132	1.64E-09
			ES	-	chr8:92921502-92921660	NA	0.286	0.000185566505
			A3SS	-	chr8:92885319-92886735	chr8:92885319-92886667	-0.188	0.003596342349
			ES	-	chr8:92921502-92921660	NA	0.19	0.00409329242
TRMO	-1.823014284	0.00206196536	ES	-	chr9:97916163-97916338	NA	0.189	0.001827589487
			ES	-	chr9:97912872-97912975	NA	-0.12	0.01516291276
TSPAN4	1.47852995	0.03182322762	ES	+	chr11:850287-850367	NA	-0.239	3.83E-05
TTC3	-1.411619257	0.000190155777	ES	+	chr21:37150819-37150884	NA	-0.141	2.10E-11
			ES	+	chr21:37094004-37094090	NA	0.175	1.50E-06
TTC7A	1.236208095	0.02253544339	ES	+	chr2:46958494-46958552	NA	-0.432	0.004065365915
TTLL11	-1.55986009	0.000941336903	ES	-	chr9:122031722-122031856	NA	0.221	0.03455480269
TXLNA	1.00075337	0.00428866993	MXE	+	chr1:32184524-32184616	chr1:32187953-32188124	-0.196	0.001031064798

TXNRD1	1.49172468	0.02544450274	MXE	+	chr12:104290992-104291104	chr12:104309758-10430995	-0.697	4.20E-05
TYSND1	1.019071886	0.002446071019	ES	-	chr10:70143841-70143972	NA	-0.289	0.000311701948
UBA7	-1.808348574	0.01975944203	ES	-	chr3:49809389-49809464	NA	0.212	0.03434244633
UBE2E1	-1.162698735	0.002684419434	ES	+	chr3:23811459-23811510	NA	-0.124	0
UBE2F	-1.537666482	0.001260329777	ES	+	chr2:237987962-237987992	NA	0.108	0.001458988257
			ES	+	chr2:237994743-237994809	NA	0.194	0.001936623701
			ES	+	chr2:237987962-237987992	NA	0.164	0.002623975802
UBE2W	1.104073456	0.03299355355	ES	-	chr8:73870268-73870301	NA	-0.19	4.28E-08
UGGT1	-1.915996479	3.57E-08	ES	+	chr2:128156391-128156415	NA	-0.114	0.002140032246
UGGT2	-1.504955429	2.64E-05	ES	-	chr13:95860787-95860883	NA	-0.462	1.74E-10
			MXE	-	chr13:95859590-95859675	chr13:95860787-95860883	-0.253	0.000129838985
			MXE	-	chr13:95856157-95856340	chr13:95859590-95859675	-0.204	0.001400276335
ULBP2	1.906203493	0.001114037869	ES	+	chr6:149947319-149947451	NA	0.15	0.005873187661
ULK4	1.312172531	0.000499960183	ES	-	chr3:41819422-41819506	NA	-0.301	8.15E-05
			ES	-	chr3:41915976-41916052	NA	0.403	0.01803272989
UPF3A	-1.593880475	6.92E-07	ES	+	chr13:114298839-114299000	NA	0.129	3.01E-05
			ES	+	chr13:114286301-114286400	NA	-0.217	0.000867862258
UPP1	2.831806786	0.01090030194	ES	+	chr7:48103296-48103411	NA	-0.155	0.001796423469
URI1	2.834677087	1.93E-13	ES	+	chr19:29971192-29971227	NA	-0.253	0.002066660603
USP32P3	-2.482787701	8.01E-07	ES	+	chr17:20420378-20420605	NA	0.582	1.10E-08
USP54	-2.161500499	0.01553958042	ES	-	chr10:73504849-73504990	NA	0.122	0.0168943985
VCAN	-10.59791853	6.34E-45	ES	+	chr5:83537006-83542268	NA	0.471	3.38E-05
VEPH1	2.564739522	0.03915655096	ES	-	chr3:157495211-157495506	NA	-0.107	0.003593871272
VPS41	-2.261133259	9.57E-11	ES	-	chr7:38796414-38796526	NA	0.11	1.23E-08
VTI1B	-1.383130372	1.77E-05	ES	-	chr14:67663028-67663190	NA	-0.183	1.67E-10
WARS	-1.0592577	0.01615281415	ES	-	chr14:100375282-100375403	NA	-0.232	1.37E-10
			ES	-	chr14:100375282-100375406	NA	-0.236	2.43E-10
			ES	-	chr14:100375282-100375350	NA	-0.205	7.15E-10
			ES	-	chr14:100375282-100375350	NA	-0.573	9.26E-05
			ES	-	chr14:100375282-100375406	NA	-0.432	0.001631060496
			ES	-	chr14:100375282-100375350	NA	-0.109	0.003593871272
			ES	-	chr14:100375282-100375406	NA	-0.185	0.04751030201
WASH2P	1.286103579	0.01628826123	ES	+	chr2:113595062-113595161	NA	-0.555	0.000350375615
WBSCR22	-1.867522876	1.53E-05	MXE	+	chr7:73693623-73693669	chr7:73693991-73694050	-0.103	0.03980685599
WDR20	-1.134379684	0.000254392584	ES	+	chr14:102197754-102197847	NA	0.174	0.009240772418
WDR4	1.877400742	6.14E-05	MXE	-	chr21:42859661-42859722	chr21:42862281-42862394	-0.158	0.02770680963
			MXE	-	chr21:42855681-42855780	chr21:42859661-42859722	-0.189	0.04529641663
WEE2-AS1	-1.371126783	0.04272802559	ES	-	chr7:141714188-141714374	NA	0.275	0.000891391094
WIPF1	-1.877854311	1.55E-05	ES	-	chr2:174677044-174677124	NA	0.321	0.004005348732
WIPI1	2.199242472	1.12E-06	ES	-	chr17:68434555-68434626	NA	0.101	0.00192549168
WSB1	-2.327253738	6.35E-06	A3SS	+	chr17:27307743-27309272	chr17:27309099-27309272	-0.171	0
WWP2	-1.97774029	0.02010486098	ES	+	chr16:69783031-69783152	NA	-0.18	0.000659813177
XKR9	3.57442536	2.41E-05	MXE	+	chr8:70674817-70674899	chr8:70680780-70681330	0.185	0.002440740444
YLPM1	-1.017819958	0.001002470897	ES	+	chr14:74824255-74824307	NA	-0.119	0.000506087906
ZBED3-AS1	-1.506734757	0.00271888178	ES	+	chr5:77100235-77100391	NA	0.276	0.009747054499
ZBTB1	-1.036029902	0.02315678997	ES	+	chr14:64516599-64516751	NA	0.31	6.58E-06
ZBTB21	-1.040193851	0.04872465371	ES	-	chr21:42002896-42002961	NA	-0.134	0.03287761207
			ES	-	chr21:42002349-42002503	NA	0.3	0.03885696071
ZBTB38	1.447947042	0.008308455737	ES	+	chr3:141438242-141438365	NA	0.146	0.04507777634
ZC3H14	-1.091280277	0.000180568967	ES	+	chr14:88602827-88603060	NA	0.23	6.94E-06
			ES	+	chr14:88601923-88602083	NA	0.325	0.000154138242
			ES	+	chr14:88602827-88603045	NA	0.265	0.000317236808
			ES	+	chr14:88596733-88596808	NA	0.164	0.002711949392
			ES	+	chr14:88601923-88602083	NA	0.154	0.04519826484
ZDHHC15	-2.442360766	3.07E-06	ES	-	chrX:75429077-75429198	NA	-0.334	0.01080928689
ZDHHC17	-1.032897555	0.02019774548	ES	+	chr12:76821057-76821167	NA	0.148	0.002782691887
ZFAND1	2.270683786	6.65E-08	ES	-	chr8:81717248-81717288	NA	-0.224	0.04922594537
ZFAND2A	-1.817010382	0.003714018982	A3SS	-	chr7:1152906-1153241	chr7:1152906-1153224	0.139	0.02999909818
ZFAND5	-1.333078315	7.09E-06	MXE	-	chr9:72356930-72357056	chr9:72360109-72360221	0.19	0
			ES	-	chr9:72363469-72363606	NA	-0.426	0

			MXE	-	chr9:72359417-72359521	chr9:72360109-72360221	0.191	1.68E-12
ZFYVE19	2.195482656	4.68E-05	ES	+	chr15:40809155-40809240	NA	-0.239	1.17E-08
			ES	+	chr15:40809118-40809240	NA	-0.147	3.27E-08
			RI	+	chr15:40813942-40814569	NA	0.164	0.03791551547
ZKSCAN3	-2.032009982	1.50E-07	ES	+	chr6:28363691-28363815	NA	0.198	0.000515678164
ZMAT1	-1.271681481	0.03854992543	MXE	-	chrX:101887197-101887304	chrX:101895813-10189588[illegible]	0.262	0.001875902704
			A3SS	-	chrX:101895813-101895882	chrX:101895813-10189587[illegible]	0.243	0.02099159975
ZMIZ1	-2.719191971	1.15E-11	ES	+	chr10:79162052-79162133	NA	0.14	0.000864542017
ZMYM5	-1.420805513	0.000363330446	ES	-	chr13:19838699-19838985	NA	0.124	0.000225754383
			A5SS	-	chr13:19851354-19852190	chr13:19851688-19852190	0.216	0.000928201364
			ES	-	chr13:19837655-19837821	NA	0.131	0.009523405635
			ES	-	chr13:19851354-19851448	NA	0.105	0.01973663004
ZMYND8	-1.458418308	0.01187922224	ES	-	chr20:47212641-47212725	NA	-0.25	0.000358771617
			MXE	-	chr20:47236325-47236516	chr20:47249286-47249439	-0.139	0.005434224012
ZNF10	-2.637146165	2.31E-11	RI	+	chr12:133130610-133131014	NA	-0.378	0.02210096143
ZNF107	-1.593153479	0.000239839747	ES	+	chr7:64691864-64691960	NA	0.133	0.004266924805
ZNF141	-1.858099581	0.003260757943	ES	+	chr4:343781-343908	NA	0.248	0.000379898374
ZNF180	-1.11388579	0.01595483866	ES	-	chr19:44485035-44485157	NA	0.207	0.04841923256
ZNF195	-1.767649668	0.000101786807	ES	-	chr11:3373576-3373656	NA	-0.149	0.000377219785
			ES	-	chr11:3361975-3362073	NA	-0.117	0.004491791827
			ES	-	chr11:3373576-3373656	NA	-0.579	0.006463478574
			ES	-	chr11:3361975-3362032	NA	-0.142	0.01403856436
			ES	-	chr11:3362545-3362602	NA	-0.142	0.02225064706
ZNF253	-3.346594707	1.03E-11	ES	+	chr19:19880050-19880146	NA	0.377	0.000181045047
			ES	+	chr19:19878480-19878607	NA	0.297	0.00201485337
ZNF254	-3.300071439	5.92E-20	ES	+	chr19:24105939-24106066	NA	0.269	8.74E-05
			ES	+	chr19:24106547-24106643	NA	0.187	0.000963896981
ZNF271P	-1.387987967	0.000201811861	ES	+	chr18:35291009-35291232	NA	0.134	0.01506849521
ZNF286A	-1.547561835	2.41E-05	A3SS	+	chr17:15700047-15700366	chr17:15700134-15700366	-0.398	0.00692806339
ZNF3	-1.958961924	2.23E-11	A5SS	-	chr7:100077228-100077433	chr7:100077302-100077433	0.106	0.01848702629
			ES	-	chr7:100077302-100077433	NA	-0.173	0.02672533686
ZNF326	-1.548570916	0.000121644484	ES	+	chr1:90007611-90007750	NA	-0.173	5.06E-06
			ES	+	chr1:90007344-90007750	NA	-0.107	6.21E-05
ZNF33B	-1.738782497	0.03981548942	ES	-	chr10:42636919-42636972	NA	-0.245	5.20E-06
			ES	-	chr10:42632294-42632439	NA	-0.176	0.02109524081
ZNF37BP	-1.528435326	0.000781101113	ES	-	chr10:42525539-42525660	NA	0.132	0.001151705187
ZNF391	-1.385817452	0.004364708063	ES	+	chr6:27395016-27395095	NA	-0.698	7.31E-07
ZNF444	-1.514819889	0.002121680293	ES	+	chr19:56142275-56142386	NA	-0.162	0.02259035035
ZNF480	-1.319612695	0.01558938453	MXE	+	chr19:52300393-52300484	chr19:52314152-52314279	0.278	0.001318100309
ZNF485	1.603917783	0.0117030675	ES	+	chr10:43609254-43609350	NA	-0.164	0.006739092745
			ES	+	chr10:43608613-43608740	NA	-0.166	0.00723312539
ZNF506	-9.665650226	3.80E-22	ES	-	chr19:19793833-19795660	NA	0.147	0.001824574982
ZNF507	2.576833563	5.28E-14	ES	+	chr19:32347244-32347338	NA	-0.214	0.004718480332
ZNF561	-1.101989568	0.005696688325	ES	-	chr19:9617620-9617758	NA	0.122	0.002940491554
			ES	-	chr19:9617620-9617768	NA	0.105	0.006032195647
ZNF561-AS1	-2.939397831	9.05E-11	ES	+	chr19:9627715-9627806	NA	-0.149	0.02302160623
ZNF569	1.016084274	0.03452300258	ES	-	chr19:37454833-37455073	NA	-0.129	0.003350576238
ZNF582-AS1	-7.903489168	9.86E-09	RI	+	chr19:56398276-56399168	NA	0.809	0
ZNF596	-1.980658695	0.02007730268	A5SS	+	chr8:243721-243858	chr8:243721-243805	-0.172	0.001472187424
			A3SS	+	chr8:242886-243013	chr8:242917-243013	0.109	0.02146154903
ZNF664	-1.115615316	0.02930132666	A5SS	+	chr12:123973218-123973352	chr12:123973218-12397330	0.12	0.0156075419
ZNF680	-2.663909908	1.98E-09	ES	-	chr7:64543706-64543802	NA	0.377	4.99E-05
			ES	-	chr7:64544305-64544432	NA	0.37	6.13E-05
			ES	-	chr7:64560945-64561273	NA	0.153	0.04800831293
ZNF692	1.745599356	2.27E-06	ES	-	chr1:248856372-248856422	NA	0.103	0.001282264471
ZNF786	-1.723777774	3.84E-05	ES	-	chr7:149080590-149080717	NA	-0.354	0.04214164797
ZNF90	-10.21007468	8.19E-17	ES	+	chr19:20125005-20126166	NA	0.155	3.70E-05
ZSCAN23	-1.278905726	0.02154150772	ES	-	chr6:28435858-28436343	NA	0.305	0.000418220202
			ES	-	chr6:28435459-28435607	NA	0.301	0.000960374064
ZSCAN26	-1.600397702	0.000490141508	A5SS	+	chr6:28271853-28272339	chr6:28271853-28272336	0.153	0.004252062559

ZSCAN31	-1.453863139	0.002312761048	ES	-	chr6:28329302-28329714	NA	0.38	8.71E-05
			ES	-	chr6:28329302-28329778	NA	0.367	0.000114900623
ZSWIM7	-1.5524246	2.08E-05	ES	-	chr17:15986799-15986892	NA	0.184	0.000434850546
			ES	-	chr17:15986799-15986886	NA	0.15	0.003676103307
			RI	-	chr17:15977578-15977913	NA	-0.191	0.02434367906
ZUFSP	1.758944026	0.004122196302	MXE	-	chr6:116660735-116660846	chr6:116666633-116666912	-0.12	0.03791778075

Supplementary Table 8: Alternatively spliced and differentially expressed protein-coding genes in GSCs significantly associated with prognosis in mesenchymal and proneural GBM.

PART A. Univariate analysis of protein-coding genes

		MES GBM samples		PN GBM samples	
**gene.symbol**	**higher.expression.GSCs**	**log-rank.pvalue**	**prognosis**	**log-rank.pvalue**	**prognosis**
**LRRFIP1**	MES	**0.0089**	poor	**0.0209**	poor
**WBSCR22**	PN	0.4699	----	**<0.001**	poor
**EMILIN2**	PN	0.5632	----	**0.0198**	poor
**CC2D2A**	PN	0.5877	----	**0.0208**	better
**RABGAP1**	PN	0.8292	----	**0.0279**	better
**FAXDC2**	PN	0.2130	----	**0.0289**	better
**TIAM1**	PN	0.9708	----	**0.0318**	better
**ATP6V1B1**	PN	0.4047	----	**0.0350**	poor
**SLC25A40**	PN	0.4172	----	**0.0406**	poor
**TCTN2**	PN	0.5923	----	**0.0419**	poor
**SH3BP2**	PN	0.5459	----	**0.0495**	poor
**DOCK10**	PN	0.3564	----	**0.0499**	better

PART B. Multivariate analysis of protein-coding genes and clinical parameters in mesenchymal GBM

	LRRFIP1		
**variable**	**beta.coef**	**Wald.statistic**	**Cox.pvalue**
**gene**	0.3576	2.1732	**0.0298**
**age at diagnosis**	1.0235	2.1386	**0.0325**
**gender**	-0.5672	-0.9263	0.3543
**CIMP status**	----	----	----
**IDH1 mutation**	----	----	----
**MGMT methylation**	0.6664	1.0655	0.2866
**chr 19/20 co-gain**	-0.3824	-0.4919	0.6228
**chr 7 gain/chr 10 loss**	1.6639	3.0849	**0.0020**

***significant variables (p<0.05)**

PART C. Multivariate analysis of protein-coding genes and clinical parameters in proneural GBM

	WBSCR22			EMILIN2			CC2D2A		
**variable**	**beta.coef**	**Wald.statistic**	**Cox.pvalue**	**beta.coef**	**Wald.statistic**	**Cox.pvalue**	**beta.coef**	**Wald.statistic**	**Cox.pvalue**
**gene**	0.4371	2.8880	**0.0039**	0.3057	2.2905	**0.0220**	-0.6509	-2.1904	**0.0285**
**age at diagnosis**	0.6586	0.7622	0.4459	1.7669	1.7764	0.0757	0.9869	1.2168	0.2237
**gender**	2.0447	2.1251	**0.0336**	1.3444	1.5675	0.1170	1.4648	1.6988	0.0894
**CIMP status**	-0.4641	-0.2689	0.7880	-1.3122	-0.9068	0.3645	-1.6557	-0.9882	0.3230
**IDH1 mutation**	0.6683	0.3387	0.7348	1.1980	0.7027	0.4823	2.4601	1.3693	0.1709
**MGMT methylation**	-0.6517	-0.8580	0.3909	-0.2245	-0.3230	0.7467	-0.5978	-0.8284	0.4075
**chr 19/20 co-gain**	2.5858	2.0582	**0.0396**	3.0716	2.3222	**0.0202**	1.9142	1.6647	0.0960
**chr 7 gain/chr 10 loss**	-3.1405	-2.4756	**0.0133**	-3.2788	-3.3061	**0.0009**	-3.0877	-2.7108	**0.0067**

	RABGAP1			FAXDC2			TIAM1		
**variable**	**beta.coef**	**Wald.statistic**	**Cox.pvalue**	**beta.coef**	**Wald.statistic**	**Cox.pvalue**	**beta.coef**	**Wald.statistic**	**Cox.pvalue**
**gene**	-0.4898	-2.6637	**0.0077**	-0.3867	-2.3636	**0.0181**	-0.4516	-2.3453	**0.0190**
**age at diagnosis**	1.5329	1.6473	0.0995	1.0947	1.2361	0.2164	0.9877	1.1502	0.2500
**gender**	1.0491	1.3994	0.1617	1.5410	1.9416	0.0522	0.9314	1.2902	0.1970
**CIMP status**	-0.7617	-0.5088	0.6109	0.5045	0.2817	0.7781	-0.8702	-0.6871	0.4920
**IDH1 mutation**	1.3375	0.7748	0.4385	0.4471	0.2210	0.8251	1.9105	1.2648	0.2059
**MGMT methylation**	-0.1112	-0.1626	0.8708	-0.5255	-0.7487	0.4541	-0.2856	-0.4097	0.6820
**chr 19/20 co-gain**	3.6093	2.6960	**0.0070**	3.8169	2.7339	**0.0063**	3.2484	2.5389	**0.0111**
**chr 7 gain/chr 10 loss**	-2.9092	-2.6597	**0.0078**	-3.6507	-3.1298	**0.0017**	-2.7238	-2.4488	**0.0143**

	ATP6V1B1			SLC25A40			TCTN2		
**variable**	**beta.coef**	**Wald.statistic**	**Cox.pvalue**	**beta.coef**	**Wald.statistic**	**Cox.pvalue**	**beta.coef**	**Wald.statistic**	**Cox.pvalue**
**gene**	0.9800	2.7176	**0.0066**	-0.6519	-1.9933	**0.0462**	-0.5079	-2.1521	**0.0314**

**age at diagnosis**	1.2171	1.2241	0.2209	0.0986	0.1209	0.9038	1.3442	1.3018	0.1930
**gender**	1.3306	1.7217	0.0851	0.8510	1.2075	0.2272	1.9354	2.0847	**0.0371**
**CIMP status**	-1.0483	-0.4788	0.6321	-1.0927	-0.9923	0.3211	-0.3113	-0.2644	0.7915
**IDH1 mutation**	1.2353	0.5064	0.6126	3.4524	2.1729	**0.0298**	2.3793	1.5883	0.1122
**MGMT methylation**	-0.1912	-0.2778	0.7811	0.2864	0.3885	0.6977	-0.7888	-1.0348	0.3008
**chr 19/20 co-gain**	2.4281	2.1981	**0.0279**	1.7522	1.4073	0.1593	3.2235	2.5165	**0.0119**
**chr 7 gain/chr 10 loss**	-3.8004	-3.4037	**0.0007**	-3.4784	-3.1967	**0.0014**	-3.6597	-3.1296	**0.0018**

	**SH3BP2**			**DOCK10**		
**variable**	**beta.coef**	**Wald.statistic**	**Cox.pvalue**	**beta.coef**	**Wald.statistic**	**Cox.pvalue**
**gene**	0.4703	2.9020	**0.0037**	-0.4336	-2.5625	**0.0104**
**age at diagnosis**	-0.1829	-0.1705	0.8646	2.4709	2.1732	**0.0298**
**gender**	1.6646	2.1857	**0.0288**	0.6621	0.9648	0.3346
**CIMP status**	-1.2888	-0.4183	0.6757	-1.2697	-0.9045	0.3657
**IDH1 mutation**	4.4787	1.2527	0.2103	1.0125	0.5902	0.5551
**MGMT methylation**	0.9293	1.1462	0.2517	0.0835	0.1115	0.9112
**chr 19/20 co-gain**	1.1558	0.9661	0.3340	3.7678	2.9061	**0.0037**
**chr 7 gain/chr 10 loss**	-2.6520	-2.9495	**0.0032**	-2.8759	-2.6857	**0.0072**

Supplementary Table 9: Co-expression of protein-coding genes associated with survival in PN GBM.

	RABGAP1	CC2D2A	WBSCR22	SLC25A40	SH3BP2	ATP6V1B1	EMILIN2	DOCK10	TIAM1	TCTN2	FAXDC2
RABGAP1	1	----	----	----	----	----	----	----	----	----	----
CC2D2A	0.3778 *	1	----	----	----	----	----	----	----	----	----
WBSCR22	-0.4728 **	-0.5614 ***	1	----	----	----	----	----	----	----	----
SLC25A40	0.1489	0.0202	0.1651	1	----	----	----	----	----	----	----
SH3BP2	-0.1133	-0.1089	0.2537	0.0130	1	----	----	----	----	----	----
ATP6V1B1	-0.2706	-0.2227	0.4765 **	0.0485	0.5485 ***	1	----	----	----	----	----
EMILIN2	-0.4391 **	-0.4684 **	0.4113 *	-0.0629	0.2745	0.3251 *	1	----	----	----	----
DOCK10	0.4522 **	0.5113 **	-0.3949 *	-0.2087	-0.1564	-0.0277	-0.5242 ***	1	----	----	----
TIAM1	0.5437 ***	0.3678 *	-0.4822 **	-0.1319	-0.0248	-0.0955	-0.2969	0.6133 ***	1	----	----
TCTN2	0.0143	0.2205	-0.0137	0.2529	0.1962	-0.0242	0.1408	-0.0546	-0.0513	1	----
FAXDC2	0.3104	0.2907	-0.5157 ***	-0.0152	-0.0399	-0.2645	-0.4730 **	0.4783 **	0.4010 *	-0.0701	1

* p<0.05 ** p<0.01 *** p<0.001

Supplementary Table 10: List of cancer-related genes.

proto.oncogenes	tumor.suppressor.genes
AKT1	ACVR1B
BRAF	APC
CTNNB1	ARID1A
DNMT3A	ARID2
EGFR	ASXL1
ERBB2	ATM
EZH2	B2M
FGFR2	BAP1
FGFR3	BCOR
FLT3	BRCA1
HRAS	CASP8
IDH1	CDH1
IDH2	CDKN2A
KIT	CEBPA
KRAS	EP300
MED12	FBXW7
NFE2L2	GATA3
NOTCH1	KDM5C
NPM1	KDM6A
NRAS	MAP3K1
PIK3CA	NCOR1
PTPN11	NF1
SF3B1	PBRM1
SPOP	PHF6
U2AF1	PIK3R1
ALK	PPP2R1A
CARD11	PTEN
HIST1H3B	RB1
MAP2K1	RUNX1
MET	SETD2
MYD88	SMAD2
ABL1	SMAD4
AR	SMARCA4
GNAS	STAG2
JAK2	STK11
PDGFRA	TET2
AJUBA	TP53
ARID5B	VHL
CBFB	WT1
CCND1	CREBBP
CHD4	PRDM1
ELF3	SMARCB1
ERCC2	TSC1
FOXA1	ATRX
MTOR	BRCA2

MYB	CDC73
RAC1	CIC
RAD21	CYLD
RASA1	RNF43
RHEB	ARHGAP35
RHOA	AXIN2
RXRA	BCLAF1
SGK1	CDK12
SMC1A	CDKN1A
SMC3	CDKN1B
SOS1	CHD8
SPEN	CTCF
TBL1XR1	DDX5
TBX3	GPS2
TCF7L2	HLA-A
BCL2	HLA-B
CBL	HSP90AB1
CRLF2	KEAP1
CSF1R	MAP2K4
DNMT1	NSD1
FOXL2	PCBP1
FUBP1	RPL5
GATA1	SOX17
GATA2	TCF7L2
GNA11	TGFBR2
GNAQ	TP53BP1
H3F3A	ZFHX3
JAK1	ARID1B
JAK3	AXIN1
KLF4	DAXX
MPL	HNF1A
NOTCH2	MEN1
PAX5	MLH1
RET	MSH2
SETBP1	MSH6
SMO	NF2
SOX9	PAX5
SRSF2	PTCH1
TSHR	SOCS1
ABCB1	TNFAIP3
ADAM10	TRAF7
AKAP9	ANAPC1
AMPH	ANKRD12
ARHGEF6	APAF1
ASPM	ATG13
BUB1B	ATR
CAD	BARD1

CARM1	BLM
CAST	BUB1B
CLIP1	CCAR1
CLTC	CDKN2C
CREB1	CHEK2
CRTC1	CLTC
CRTC3	COL18A1
CTNND1	DMD
CTTN	EIF4A2
CUL1	FANCA
DICER1	FBN2
DOCK2	FBXO11
EEF1A1	FOXA2
ERBB2IP	KALRN
ESR1	KIAA1109
EWSR1	LIFR
FES	MAP3K4
FN1	MATK
GOLGA5	MAX
GOT2	MST1
HGF	NAV3
HSPA8	NEDD4L
HUWE1	NLRP3
IRS2	NR1H2
ITGA6	PABPC1
KIFC3	PCM1
LCP1	PHLDA1
LEPR	POTEF
LPP	PRKCD
LUM	RBMX
LYN	RPL22
MALAT1	SIN3A
MAP3K3	SYNE1
MAPK8IP1	TJP2
MAST2	TOP3A
MDC1	TSC2
MECOM	TXNIP
MEF2A	ZFP36L1
MLLT10	ZFP36L2
MLLT3	ZNF132
MLLT4	ACO1
MMP2	ALPK2
MN1	ATP5B
MSN	AZGP1
MYH9	CCDC6
NCOA3	FAT1
NCOR2	ING1

NONO	IRF6
NR4A2	MED23
NUMA1	MGA
NUP214	MPO
NUP98	MUC17
PCM1	MYOCD
PIK3CB	NBPF1
PIK3CG	NTN4
PIP5K1A	ODAM
PLCG1	OMA1
PLEC	OTUD7A
PRKAR1A	PDSS2
PRKCD	PPP6C
STAT3	QKI
SUZ12	RBM10
TAF1	SERPINB13
TCF12	SIRT4
TFDP1	SLC26A3
TGIF1	TAP1
TNFRSF10C	TRAF3
TPR	KMT2C
TRIO	KMT2D
WHSC1	AMER1
WHSC1L1	
WRN	
ZMYM2	
ZNF300	
ACVR2B	
BRE	
CAP2	
CD1D	
CD70	
CDC27	
CDK4	
CNKSR1	
COL5A1	
CUL4B	
CUX1	
DDX3X	
DIAPH1	
DIS3	
EPHA2	
ERBB3	
EZR	
FAT1	
FGFBP1	
FOXQ1	

GNA13
GNB1
IL7R
INPPL1
IRF4
ITGB7
MAP4K3
MBD1
MED23
MORC4
MUC17
MYCN
NTN4
ODAM
PLCG2
POU2F2
PPM1D
RIT1
RPS15
RPS2
SETDB1
STX2
TCEB1
TIMM17A
TNF
TNFRSF14
TPX2
TRAF3
WASF3
XPO1
KMT2D
KMT2B
SRSF2
KMT2A

Supplementary Table 11: Differentially spliced and expressed cancer-related genes in GSCs.

	AS		AS & up-regulated		AS & down-regulated	
	**MES**	**PN**	**MES**	**PN**	**MES**	**PN**
**Proto-oncogenes**	22	17	1	1	3	8
**Tumor suppressor genes**	19	14	1	2	5	5
**Total cancer genes**	**41**	**31**	**2**	**3**	**8**	**13**

* MES: mesenchymal; PN: proneural; AS: alternative splicing

Supplementary Table 12: Functional annotation of genes exclusively undergoing splicing alterations in mesenchymal (MES) and proneural (PN) GSCs samples based on gene ontology (GO) terms for biological process (BP), cellular component (CC), and molecular function (MF).

GO category	GO term	# genes	% genes	FDR
GOTERM_BP_DIRECT	GO:0006281~DNA repair	55	2.819067145	3.70E-06
GOTERM_BP_DIRECT	GO:0060271~cilium morphogenesis	38	1.947719118	1.12E-05
GOTERM_BP_DIRECT	GO:0042384~cilium assembly	35	1.79395182	3.69E-05
GOTERM_BP_DIRECT	GO:0051301~cell division	65	3.331624808	0.001310342791
GOTERM_BP_DIRECT	GO:0000398~mRNA splicing, via spliceosome	45	2.306509482	0.008430104661
GOTERM_BP_DIRECT	GO:0000086~G2/M transition of mitotic cell cycle	32	1.640184521	0.01364712908
GOTERM_BP_DIRECT	GO:0007062~sister chromatid cohesion	26	1.332649923	0.0285266066
GOTERM_CC_DIRECT	GO:0005654~nucleoplasm	464	23.78267555	6.82E-35
GOTERM_CC_DIRECT	GO:0005737~cytoplasm	657	33.67503844	6.59E-15
GOTERM_CC_DIRECT	GO:0005634~nucleus	676	34.648898	9.83E-15
GOTERM_CC_DIRECT	GO:0005829~cytosol	442	22.65504869	7.31E-12
GOTERM_CC_DIRECT	GO:0005813~centrosome	91	4.664274731	5.32E-10
GOTERM_CC_DIRECT	GO:0016607~nuclear speck	47	2.409021015	2.51E-05
GOTERM_CC_DIRECT	GO:0005814~centriole	32	1.640184521	7.03E-05
GOTERM_CC_DIRECT	GO:0005730~nucleolus	131	6.714505382	9.41E-05
GOTERM_CC_DIRECT	GO:0016020~membrane	278	14.24910302	0.0006209350894
GOTERM_CC_DIRECT	GO:0036064~ciliary basal body	27	1.383905689	0.001692829306
GOTERM_CC_DIRECT	GO:0005739~mitochondrion	176	9.021014864	0.009257314479
GOTERM_CC_DIRECT	GO:0000922~spindle pole	27	1.383905689	0.01426062834
GOTERM_CC_DIRECT	GO:1904115~axon cytoplasm	14	0.7175807278	0.0162211246
GOTERM_MF_DIRECT	GO:0005515~protein binding	1088	55.76627371	4.14E-27
GOTERM_MF_DIRECT	GO:0044822~poly(A) RNA binding	199	10.19989749	3.66E-13
GOTERM_MF_DIRECT	GO:0003723~RNA binding	87	4.459251666	0.01536239243

Supplementary Table 13: Functional annotation of genes exclusively undergoing expression alterations between mesenchymal (MES) and proneural (PN) GSCs samples based on KEGG pathways gene ontology (GO) terms for biological process (BP), cellular component (CC), and molecular function (MF).

Up-regulated genes in MES

Category	Term	# genes	% genes	FDR
GOTERM_BP_DIRECT	GO:0098609~cell-cell adhesion	46	2.212602213	0.04799407647
GOTERM_MF_DIRECT	GO:0005515~protein binding	866	41.65464165	0.04744372776

Up-regulated genes in PN

Category	Term	# genes	% genes	FDR
GOTERM_BP_DIRECT	GO:0006351~transcription, DNA-templated	323	12.75167785	3.58E-08
GOTERM_BP_DIRECT	GO:0007399~nervous system development	72	2.842479274	9.92E-07
GOTERM_BP_DIRECT	GO:0006355~regulation of transcription, DNA-templated	250	9.8697197	6.38E-06
GOTERM_BP_DIRECT	GO:0007156~homophilic cell adhesion via plasma membrane adhesion molecules	46	1.816028425	1.56E-05
GOTERM_BP_DIRECT	GO:0007155~cell adhesion	95	3.750493486	6.75E-05
GOTERM_BP_DIRECT	GO:0000122~negative regulation of transcription from RNA polymerase II promoter	128	5.053296486	0.00235541617
GOTERM_BP_DIRECT	GO:0007158~neuron cell-cell adhesion	11	0.4342676668	0.00375030318
GOTERM_BP_DIRECT	GO:0045665~negative regulation of neuron differentiation	21	0.8290564548	0.004961836942
GOTERM_BP_DIRECT	GO:0010977~negative regulation of neuron projection development	18	0.7106198184	0.008066097786
GOTERM_BP_DIRECT	GO:0007411~axon guidance	40	1.579155152	0.009417216454
GOTERM_BP_DIRECT	GO:0030198~extracellular matrix organization	46	1.816028425	0.01217008478
GOTERM_BP_DIRECT	GO:0007416~synapse assembly	21	0.8290564548	0.02206267871
GOTERM_BP_DIRECT	GO:0051965~positive regulation of synapse assembly	21	0.8290564548	0.02900744251
GOTERM_CC_DIRECT	GO:0005578~proteinaceous extracellular matrix	69	2.724042637	3.28E-07
GOTERM_CC_DIRECT	GO:0030054~cell junction	94	3.711014607	5.88E-05
GOTERM_CC_DIRECT	GO:0009986~cell surface	103	4.066324516	0.0005953719362
GOTERM_CC_DIRECT	GO:0005622~intracellular	204	8.053691275	0.02365275485
GOTERM_MF_DIRECT	GO:0003700~transcription factor activity, sequence-specific DNA binding	174	6.869324911	1.48E-06
GOTERM_MF_DIRECT	GO:0046872~metal ion binding	320	12.63324122	1.34E-05
GOTERM_MF_DIRECT	GO:0005509~calcium ion binding	130	5.132254244	0.0002364379719
GOTERM_MF_DIRECT	GO:0003676~nucleic acid binding	161	6.356099487	0.005817301938
GOTERM_MF_DIRECT	GO:0050840~extracellular matrix binding	13	0.5132254244	0.01829692888
GOTERM_MF_DIRECT	GO:0005518~collagen binding	20	0.789577576	0.04420150625
KEGG_PATHWAY	hsa05200:Pathways in cancer	77	3.039873668	7.38E-05
KEGG_PATHWAY	hsa04360:Axon guidance	32	1.263324122	0.006546766066
KEGG_PATHWAY	hsa04350:TGF-beta signaling pathway	23	0.9080142124	0.04864576473

Supplementary Table 14: RNA binding proteins (RBPs) mapped to spliceosome components.

RBP	up in MES	up in PN	AS.events	spliceosome
DHX38	yes	----	----	2nd step factor
SLU7	yes	----	----	2nd step factor
DDX50	----	----	MES	B act
CACTIN	----	----	MES	Complex C
GPATCH1	yes	----	----	Complex C
PPIL3	----	----	MES	Complex C
PPWD1	----	----	MES	Complex C
WDR83	----	----	PN	Complex C
TFIP11	----	----	MES	Complex C2
FUS	----	yes	PN	Complex A
LUC7L	----	yes	----	Complex A
PRPF40A	----	----	MES	Complex A
RBM23	----	yes	MES & PN	Complex A
RBM25	----	----	PN	Complex A
RBM39	----	----	MES	Complex A
RBM5	----	----	MES	Complex A
SF1	----	----	MES	Complex A
PRPF38A	----	----	PN	Complex B
PRPF4B	----	----	MES & PN	Complex B
THRAP3	----	----	MES	Complex B
ACIN1	----	----	MES	EJC/TREX
SAP18	----	yes	----	EJC/TREX
THOC1	yes	----	----	EJC/TREX
THOC2	----	----	MES	EJC/TREX
THOC5	----	----	MES & PN	EJC/TREX
THOC6	yes	----	----	EJC/TREX
HNRNPA1	----	----	MES	hnRNP
HNRNPA2B1	----	yes	MES	hnRNP
HNRNPD	----	----	MES & PN	hnRNP
HNRNPDL	----	----	PN	hnRNP
HNRNPH1	----	----	MES	hnRNP
HNRNPH3	----	----	PN	hnRNP
HNRNPL	----	----	MES	hnRNP
HNRNPM	----	----	PN	hnRNP
HNRNPR	----	----	PN	hnRNP
HNRNPUL1	----	----	PN	hnRNP
PTBP2	----	yes	MES	hnRNP
PTBP3	yes	----	----	hnRNP
RALY	----	----	PN	hnRNP
LSM1	----	----	PN	Lsm protein
LSM5	----	yes	----	Lsm protein
CELF1	----	----	MES	MISC
DHX9	----	----	PN	MISC

ILF3	----	----	MES & PN	MISC
NSRP1	----	----	MES & PN	MISC
PABPC1	yes	----	----	MISC
QKI	----	yes	MES & PN	MISC
RBFOX2	----	----	PN	MISC
XAB2	yes	----	----	Complex Prp19 associated
SNRPG	----	----	PN	Sm protein
SNRPN	----	----	MES	Sm protein
SREK1	----	----	MES	SR protein
SRRM1	----	----	MES	SR protein
SRSF12	----	yes	----	SR protein
SRSF6	----	yes	MES	SR protein
SRSF7	----	----	MES & PN	SR protein
SNRNP70	----	----	MES	snRNP U1
SNRNP35	----	yes	----	snRNP U11/U12
PHF5A	----	----	PN	snRNP U2
SF3B6	yes	----	----	snRNP U2
DDX46	----	----	PN	snRNP U2 associated
PUF60	----	----	MES	snRNP U2 associated
U2AF1	----	yes	----	snRNP U2 associated
PRPF31	----	----	MES	snRNP U4/U6
SNU13	----	----	PN	snRNP U4/U6
SRPK2	----	yes	----	snRNP U5oU4/U6

Supplementary Table 15: Differentially expressed splicing-related RNA binding proteins (RBPs) between mesenchymal (MES) and proneural (PN) GSCs and GBM samples from TCGA.

GSCs			
**RBP**	**log2FoldChange**	**FDR**	**up.regulated**
CELF2	9.197588986	3.31E-37	PN
U2AF1	4.008162325	1.11E-22	PN
MBNL3	7.840253782	2.85E-20	PN
POP4	2.558240582	3.06E-16	MES
RBM11	9.634986879	3.26E-14	MES
QKI	2.873719689	4.43E-14	PN
MVP	4.607741975	6.03E-14	MES
SRSF12	8.947413633	4.03E-13	PN
PPARGC1A	8.12470585	1.11E-12	PN
PABPC1	2.380206452	6.33E-12	MES
GPATCH1	1.971018955	1.16E-11	MES
POLR2D	1.884638485	3.73E-10	PN
RP9	2.602651596	9.96E-10	PN
NOVA1	3.14922289	1.31E-08	PN
UPF3A	1.593880475	6.92E-07	PN
TIA1	1.927848441	3.09E-06	PN
CLK4	1.941223901	3.54E-06	PN
RBM23	1.47130468	5.57E-06	PN
SON	1.19079563	8.55E-06	PN
LSM5	1.225545572	1.26E-05	PN
RBMS3	2.084815207	3.15E-05	PN
SLU7	1.473450764	3.22E-05	MES
SRSF6	1.135706591	3.91E-05	PN
CCNT2	1.499508598	4.38E-05	PN
SFPQ	1.301935116	4.51E-05	PN
ADAT1	1.502652197	6.01E-05	PN
ZNF326	1.548570916	0.0001216444844	PN
PTBP2	1.71189802	0.0001693405314	PN
LUC7L	1.204263891	0.0001700296867	PN
SMN2	4.577968467	0.0001945506733	PN
TRMT1L	1.3881325	0.0001964197515	MES
DHX38	1.461842975	0.0002464800835	MES
SRPK2	1.236213978	0.0003627820659	PN
SAP18	1.211323091	0.0004215602091	PN
DUSP11	1.167737455	0.000506676372	MES
HNRNPA2B1	1.125577478	0.0007407571256	PN
PTBP3	1.157095553	0.0007908280334	MES
SCAF4	1.029350091	0.0009154269133	PN
SNRNP35	1.116655399	0.001132352051	PN
FUS	1.199979067	0.001206886185	PN
SMG6	1.280044418	0.002746806291	PN
POLDIP3	1.1845829	0.00411630455	PN
SF3B6	1.299698683	0.01084435673	MES
XAB2	1.301967349	0.01598622458	MES
POLR2J	1.083909056	0.01842346686	PN
CLK1	1.121236214	0.01885873773	PN
THOC1	1.006603965	0.01964208744	MES
ERN1	1.04968285	0.02331227598	MES

GBM from TCGA			
**RBP**	**diff**	**p.adj**	**up.regulated**
MVP	1.12	0	MES
QKI	0.63	0	PN
POLR2D	0.28	0	PN
NOVA1	1.13	0	PN
TIA1	0.46	0	PN
RBM23	0.27	0	PN
CCNT2	0.35	0	PN
SFPQ	0.48	0	PN
ZNF326	0.27	0	PN
PTBP2	0.92	0	PN
LUC7L	0.44	0	PN
SRPK2	0.31	0	PN
SNRNP35	0.26	0	PN
FUS	0.37	0	PN
POLDIP3	0.4	0	PN
CLK1	0.46	0	PN
HNRNPA2B1	0.21	0.01	PN
CELF2	0.26	0.02	PN
UPF3A	0.26	0.03	PN

RBPMS	2.56712388	0.02742119653	PN
THOC6	1.169586913	0.0339520237	MES

Supplementary Table 16: RNA Binding Protein (RBPs)-associated splicing events enriched in mesenchymal (MES) and proneural (PN) GSCs samples.

ASS (alternative 5'or 3'splice site): genomic coordinates of long and short exons (columns g.coord_1 and g.coord_

MXE (mutually exclusive exon): genomic coordinates of first and EScond exons (columns g.coord_1 and g.coord_

RI (retained introns): genomic coordinates of retained intron (column g.coord_1)

ES (exon skipping): genomic coordinates of skipped exon (column g.coord_1)

MES						
event.type	RBP	strand	g.coord_1	g.coord_2	inc.level.difference	FDR
ES	RBM5	+	chr3:50104247-50104308	NA	0.637	0
ES	RBM5	+	chr3:50110378-50110463	NA	0.247	0
ES	SNRNP70	+	chr19:49102113-49103587	NA	0.141	0
ES	QKI	+	chr6:163455278-163455421	NA	0.424	0
ES	HNRNPA2B1	-	chr7:26190992-26191128	NA	0.402	0
ASS	THOC2	-	chrX:123620374-123620965	chrX:123620906-123620965	0.231	0
ES	DBR1	-	chr3:138163777-138163858	NA	0.426	0
ES	SON	+	chr21:33572550-33572630	NA	0.263	0
ES	PRPF40A	-	chr2:152679055-152679109	NA	0.309	0
RI	QKI	+	chr6:163563419-163570145	NA	0.602	3.34E-14
ES	CCAR2	+	chr8:22613016-22613136	NA	0.147	8.25E-14
ES	TIA1	-	chr2:70229058-70229091	NA	0.349	1.08E-13
ES	U2AF1L4	-	chr19:35744625-35744692	NA	0.725	9.67E-13
ES	EEF1D	-	chr8:143592646-143592893	NA	0.522	1.10E-11
ES	EEF1D	-	chr8:143592646-143592942	NA	0.497	1.12E-11
ES	ILF3	+	chr19:10684415-10684476	NA	0.323	1.34E-11
ES	U2AF1L4	-	chr19:35744625-35744738	NA	0.662	2.54E-11
ES	TIA1	-	chr2:70228343-70228462	NA	0.465	4.53E-11
ES	PPIL3	-	chr2:200882341-200882435	NA	0.454	5.63E-11
ES	NSUN2	-	chr5:6631872-6631977	NA	0.189	1.23E-10
ES	PUF60	-	chr8:143820665-143820716	NA	0.435	2.28E-10
ES	HNRNPD	-	chr4:82371527-82371584	NA	0.29	3.50E-10
ASS	QKI	+	chr6:163564666-163570145	chr6:163565945-163570145	0.545	3.14E-09
ES	LUC7L3	+	chr17:50738136-50738199	NA	0.274	3.31E-09
ES	HNRNPH1	-	chr5:179619268-179619360	NA	0.129	5.52E-09
ES	SRSF7	-	chr2:38748897-38749346	NA	0.118	8.21E-09
MXE	QKI	+	chr6:163534981-163535125	chr6:163561981-163562069	0.124	1.07E-08
ES	EEF1D	-	chr8:143592646-143592893	NA	0.499	1.57E-08
RI	ARL6IP4	+	chr12:122981128-122981879	NA	0.401	1.61E-08
ASS	SNRPN	+	chr15:24974287-24974456	chr15:24974310-24974456	0.392	2.09E-08
ES	THOC5	-	chr22:29550727-29550843	NA	0.218	2.53E-08
ES	PPWD1	+	chr5:65571838-65572286	NA	0.179	4.50E-08
ES	MBNL1	+	chr3:152456266-152456361	NA	0.13	5.88E-08
ES	SF1	-	chr11:64771463-64771537	NA	0.208	1.08E-07
ASS	SFSWAP	+	chr12:131714070-131714240	chr12:131714070-131714193	0.163	1.54E-07
ASS	TIA1	-	chr2:70216752-70216994	chr2:70216885-70216994	0.191	1.98E-07
ASS	GEMIN8	-	chrX:14026051-14026221	chrX:14026139-14026221	0.327	3.93E-07
RI	QKI	+	chr6:163563419-163570145	NA	0.403	4.78E-07
ES	ACO1	+	chr9:32386370-32386438	NA	0.159	9.98E-07
ES	PPIL3	-	chr2:200882341-200882382	NA	0.525	1.32E-06
ES	SREK1	+	chr5:66156064-66158932	NA	0.327	1.42E-06
ES	DDX50	+	chr10:68906225-68906337	NA	0.106	1.74E-06
ES	ZCCHC8	-	chr12:122490461-122490567	NA	0.176	1.79E-06
MXE	HNRNPH1	-	chr5:179620891-179621397	chr5:179623036-179623164	0.114	2.46E-06
MXE	QKI	+	chr6:163534981-163535125	chr6:163561981-163562069	0.124	2.92E-06
MXE	PPIL3	-	chr2:200882341-200882435	chr2:200885261-200885343	0.104	3.05E-06
ES	EEF1D	-	chr8:143592646-143592797	NA	0.512	4.25E-06
MXE	LUC7L3	+	chr17:50737286-50737479	chr17:50738140-50738199	0.106	4.40E-06
ES	ZNF326	+	chr1:90007611-90007750	NA	0.173	5.06E-06
ES	SRRM1	+	chr1:24663182-24663224	NA	0.254	5.46E-06
MXE	LUC7L3	+	chr17:50737286-50737479	chr17:50738136-50738199	0.109	5.64E-06
ES	RBM23	-	chr14:22908332-22908380	NA	0.228	1.15E-05
MXE	PPIL3	-	chr2:200882341-200882435	chr2:200885261-200885367	0.153	1.17E-05
ES	EEF1D	-	chr8:143590607-143590729	NA	0.51	1.39E-05
ASS	QKI	+	chr6:163563419-163563719	chr6:163563443-163563719	0.127	1.90E-05
ES	PPIL3	-	chr2:200885261-200885367	NA	0.339	1.93E-05
ES	EEF1D	-	chr8:143590607-143590735	NA	0.471	1.99E-05
ASS	HNRNPH1	-	chr5:179623594-179623671	chr5:179623635-179623671	0.141	2.06E-05
ES	CACTIN	-	chr19:3611228-3611364	NA	0.444	2.72E-05
ASS	CLK4	-	chr5:178619837-178620673	chr5:178619837-178619896	0.302	4.40E-05
ES	EEF1D	-	chr8:143590607-143590738	NA	0.346	5.02E-05
ES	SNRPN	+	chr15:24974641-24974957	NA	0.514	5.03E-05
ES	ZNF326	+	chr1:90007344-90007750	NA	0.107	6.21E-05
MXE	HNRNPD	-	chr4:82355303-82355401	chr4:82356536-82356683	0.127	8.41E-05
RI	U2AF1L4	-	chr19:35744322-35744738	NA	0.397	9.78E-05
RI	PRPF31	+	chr19:54129056-54129370	NA	0.131	0.000126791982
ASS	CASC3	+	chr17:40167370-40167612	chr17:40167497-40167612	0.116	0.000143821555
RI	PRPF4B	+	chr6:4056320-4057187	NA	0.118	0.000170267772
ES	PRPF4B	+	chr6:4062556-4062760	NA	0.163	0.000193544491
ES	SMG7	+	chr1:183547102-183547252	NA	0.216	0.000268228621
MXE	HNRNPH1	-	chr5:179621241-179621397	chr5:179623036-179623164	0.106	0.000298680430
ES	CLK4	-	chr5:178619837-178619896	NA	0.17	0.000432013183
RI	RBM5	+	chr3:50100531-50103166	NA	0.214	0.000549559422
ES	TRMU	+	chr22:46355989-46356072	NA	0.401	0.000653105210
ES	HNRNPA1	+	chr12:54281385-54281502	NA	0.112	0.000749873320
ASS	SART3	-	chr12:108544426-108544532	chr12:108544426-108544478	0.142	0.000777512798
ES	PUF60	-	chr8:143827384-143827536	NA	0.128	0.000780430881
ES	EEF1D	-	chr8:143590644-143590738	NA	0.311	0.000791141303
ES	CLK4	-	chr5:178619837-178620673	NA	0.125	0.000805592955
ES	UPF3A	+	chr13:114286301-114286400	NA	0.217	0.000867862258
ES	SRSF6	+	chr20:43459152-43459420	NA	0.11	0.000938808030
ES	PRPF40B	+	chr12:49632595-49632623	NA	0.329	0.001138009614
MXE	DEK	-	chr6:18258303-18258405	chr6:18263842-18263996	0.121	0.001323759902
ES	EEF1D	-	chr8:143590644-143590735	NA	0.479	0.001367554607
ES	NSRP1	+	chr17:30118079-30118173	NA	0.239	0.002111221232
ASS	THOC2	-	chrX:123610917-123610963	chrX:123610917-123610957	0.113	0.002286623134
ES	CPSF7	-	chr11:61420469-61420530	NA	0.111	0.002471171293
ASS	SECISBP2	+	chr9:89334521-89334730	chr9:89334635-89334730	0.158	0.002486846193

PN						
event.type	RBP	strand	g.coord_1	g.coord_2	inc.level.difference	FDR
ASS	QKI	+	chr6:163565945-163566795	chr6:163566720-163566795	-0.573	0
ASS	DAP3	+	chr1:155689090-155689243	chr1:155689090-155689174	-0.185	0
RI	TSEN2	+	chr3:12529761-12531659	NA	-0.169	0
RI	CLK4	-	chr5:178616881-178618778	NA	-0.481	1.16E-13
RI	SUGP2	-	chr19:18994365-18995280	NA	-0.322	1.97E-13
ASS	ARL6IP4	+	chr12:122981570-122981879	chr12:122981594-122981879	-0.303	5.86E-13
RI	FUS	+	chr16:31184938-31188357	NA	-0.141	6.33E-13
ES	PRPF4B	+	chr6:4056526-4056688	NA	-0.255	6.63E-13
ES	NONO	+	chrX:71284983-71285097	NA	-0.185	7.49E-12
ES	SNU13	-	chr22:41682355-41682587	NA	-0.101	1.11E-11
RI	CLK1	-	chr2:200859679-200861466	NA	-0.38	5.85E-11
ES	DHX9	+	chr1:182855576-182855713	NA	-0.121	3.15E-10
ASS	ARL6IP4	+	chr12:122981570-122981879	chr12:122981594-122981879	-0.254	3.67E-10
ES	U2AF1L4	-	chr19:35744490-35744540	NA	-0.55	1.56E-09
ES	UBTF	-	chr17:44208618-44209001	NA	-0.104	6.26E-08
ES	RBM23	-	chr14:22905605-22905659	NA	-0.136	6.52E-08
RI	HNRNPM	+	chr19:8465323-8465515	NA	-0.161	7.93E-08
RI	CLK4	-	chr5:178617343-178618778	NA	-0.336	9.82E-08
ES	RBFOX2	-	chr22:35836319-35836439	NA	-0.189	1.49E-07
RI	ILF3	+	chr19:10681218-10682192	NA	-0.143	1.78E-07
ES	HNRNPDL	-	chr4:82425562-82425667	NA	-0.185	3.12E-07
MXE	EEF1D	-	chr8:143581228-143581328	chr8:143586218-143586290	-0.184	1.14E-06
ES	HNRNPD	-	chr4:82356536-82356683	NA	-0.181	1.83E-06
RI	CLK3	+	chr15:74622119-74622560	NA	-0.15	1.92E-06
RI	WDR83	+	chr19:12669754-12670080	NA	-0.576	5.75E-06
ES	SNRPG	-	chr2:70289349-70289372	NA	-0.177	8.30E-06
ES	LSM1	-	chr8:38169801-38169917	NA	-0.102	2.05E-05
ES	ADARB1	+	chr21:45184473-45184593	NA	-0.343	2.24E-05
ES	THOC5	-	chr22:29531077-29531110	NA	-0.132	2.46E-05
RI	SRSF7	-	chr2:38748578-38749705	NA	-0.138	2.58E-05
ES	UPF3A	+	chr13:114298839-114299000	NA	-0.129	3.01E-05
MXE	QKI	+	chr6:163455278-163455421	chr6:163534981-163535125	-0.168	3.05E-05
RI	PRPF40B	+	chr12:49631859-49632880	NA	-0.355	4.01E-05
ES	HNRNPR	-	chr1:23340851-23341017	NA	-0.125	4.88E-05
RI	PRPF4B	+	chr6:4060412-4061147	NA	-0.11	4.91E-05
ES	HNRNPR	-	chr1:23340851-23341020	NA	-0.127	6.33E-05
ES	PHF5A	-	chr22:41468019-41468147	NA	-0.127	8.80E-05
ASS	PRPF38A	+	chr1:52405679-52405839	chr1:52405679-52405713	-0.175	0.000108494166
RI	ZCCHC8	-	chr12:122483278-122483563	NA	-0.216	0.000110570446
MXE	NSRP1	+	chr17:30118079-30118173	chr17:30163066-30163162	-0.106	0.000111351818
RI	DDX46	+	chr5:134816429-134817714	NA	-0.105	0.000396894854
MXE	SUGP2	-	chr19:18995143-18995280	chr19:19001612-19001674	-0.153	0.000401550801
ES	RALY	+	chr20:34065043-34065160	NA	-0.352	0.000541806568
ES	HNRNPUL	+	chr19:41305831-41305867	NA	-0.111	0.000558909809
MXE	TRMT2B	-	chrX:101042041-101042312	chrX:101051643-101051733	-0.147	0.000818605483
RI	CCAR2	+	chr8:22619149-22620468	NA	-0.169	0.001791555223
RI	ARL6IP4	+	chr12:122981128-122981879	NA	-0.104	0.001961330751
RI	DDX39A	-	chr19:14409741-14410334	NA	-0.119	0.002219711602
RI	ADARB1	+	chr21:45222017-45226560	NA	-0.208	0.002219711602
ASS	CPSF7	-	chr11:61420469-61421608	chr11:61421389-61421608	-0.151	0.002518662501
ES	GFM2	-	chr5:74730259-74730398	NA	-0.123	0.003000752999
ASS	ADARB1	+	chr21:45222017-45223577	chr21:45222017-45222107	-0.212	0.003150772569
ASS	HNRNPH3	+	chr10:68338502-68338687	chr10:68338502-68338642	-0.114	0.004040130954
MXE	TRMT2B	-	chrX:101051250-101051366	chrX:101051643-101051733	-0.474	0.004271075024
ASS	U2AF1L4	-	chr19:35744322-35744738	chr19:35744322-35744421	-0.35	0.004351922584
ES	CCNT1	-	chr12:48695998-48696162	NA	-0.117	0.005099606958
RI	CLK4	-	chr5:178616881-178618778	NA	-0.325	0.00639004427
ES	ADAT1	-	chr16:75618585-75618698	NA	-0.123	0.009096233477
RI	U2AF1L4	-	chr19:35744322-35745187	NA	-0.332	0.0095896582
MXE	FASTK	-	chr7:151076912-151077027	chr7:151077100-151077236	-0.121	0.01029295253
ASS	U2AF1L4	-	chr19:35744322-35744981	chr19:35744322-35744421	-0.329	0.01277456951
ES	GFM2	-	chr5:74738501-74738642	NA	-0.102	0.01693677111
RI	RBM25	+	chr14:73110830-73111802	NA	-0.103	0.01817881467
ES	ZCCHC8	-	chr12:122483278-122483344	NA	-0.213	0.0247351972
ES	NSRP1	+	chr17:30163066-30163162	NA	-0.453	0.02613073178
ES	GEMIN4	-	chr17:749813-749909	NA	-0.119	0.03462897539
MXE	PRPF4B	+	chr6:4061654-4061700	chr6:4062212-4062299	-0.24	0.03970723868
ASS	WDR83	+	chr19:12670518-12670611	chr19:12670562-12670611	-0.299	0.04408854106
ES	MBNL1	+	chr3:152446703-152446757	NA	-0.235	0.04669502336

ES	RBM39	-	chr20:35740524-35740597	NA	0.154	0.003153225159
ASS	CPSF7	-	chr11:61421053-61421130	chr11:61421053-61421127	0.301	0.003195651006
RI	ARL6IP4	+	chr12:122981128-122981879	NA	0.229	0.00421474628
MXE	PPIL3	-	chr2:200882341-200882382	chr2:200885261-200885343	0.286	0.004322405941
ES	POLDIP3	-	chr22:42601969-42602056	NA	0.229	0.005634242534
RI	U2AF1L4	-	chr19:35744322-35745187	NA	0.282	0.005964038928
MXE	POP4	+	chr19:29610408-29610457	chr19:29611861-29611939	0.229	0.007333096958
ES	POLDIP3	-	chr22:42601969-42602107	NA	0.263	0.007773580358
ES	PRPF4B	+	chr6:4062556-4062633	NA	0.151	0.007818435325
ES	TSEN2	+	chr3:12516610-12516661	NA	0.173	0.007851399504
ES	PUF60	-	chr8:143824312-143824396	NA	0.228	0.008185219315
MXE	DBR1	-	chr3:138163777-138163858	chr3:138167080-138167305	0.171	0.01047188361
MXE	CPSF7	-	chr11:61421053-61421127	chr11:61421191-61421257	0.378	0.01173911421
MXE	MBNL1	+	chr3:152455541-152455577	chr3:152456266-152456361	0.163	0.01194955016
ES	THRAP3	+	chr1:36225411-36225463	NA	0.218	0.01278202333
ES	TFIP11	-	chr22:26511118-26511317	NA	0.127	0.0127859952
ES	EXOSC10	-	chr1:11080498-11080514	NA	0.137	0.01358618517
ES	SREK1	+	chr5:66158808-66158932	NA	0.212	0.01430870942
ES	RBM5	+	chr3:50100531-50100605	NA	0.228	0.02074446813
ES	CCNT2	+	chr2:134945786-134945982	NA	0.132	0.02298175873
ES	CELF1	-	chr11:47500860-47500932	NA	0.237	0.02326580329
ES	TSEN2	+	chr3:12505153-12505231	NA	0.135	0.02398359823
ES	RBMS1	-	chr2:160282256-160282304	NA	0.217	0.02751345708
ES	ACIN1	-	chr14:23089981-23090101	NA	0.233	0.02847082484
MXE	PPIL3	-	chr2:200882341-200882382	chr2:200885261-200885367	0.277	0.03684497474
ES	PUF60	-	chr8:143824312-143824399	NA	0.166	0.0374094293
ES	HNRNPL	-	chr19:38841612-38841682	NA	0.156	0.03764037285
ASS	PTBP2	+	chr1:96804784-96804939	chr1:96804799-96804939	0.389	0.03982276621
MXE	CPSF7	-	chr11:61421053-61421130	chr11:61421191-61421257	0.32	0.0423514077
ES	ACIN1	-	chr14:23071124-23071186	NA	0.194	0.04533534914
ES	TFIP11	-	chr22:26511118-26511195	NA	0.144	0.04611867135

Supplementary Table 17: Differentially spliced/expressed RNA binding proteins (RBPs) in GSCs with splice-site-creating mutations reported at Jayasinghe et al.

	**GSCs**		**Mutations Jayasinghe *et al.***							
**RBP**	**Mesenchy**	**AS.events**	**Exon**	**Intron**	**Site**	**Chromosome**	**Position**	**MutationType**	**NovelJunctionPosition**	**Cancer**
**ACIN1**	----	MES	14/19	.	TCGA-D1-A2G0_14_23532257_G_A	14	23532257	Missense	23532247	UCEC
			12/19	.	TCGA-CF-A27C_14_23533388_C_G	14	23533388	Missense	23533386	BLCA
**DDX46**	----	PN	18/23	.	TCGA-YG-AA3O_5_134147386_G_A	5	134147386	Missense	134147388	SKCM
**DHX38**	up	----	.	15/26	TCGA-DG-A2KM_16_72138925_G_C	16	72138925	Splice Site	72138934	CESC
**GEMIN8**	----	MES	5/5	.	TCGA-BF-AAP1_X_14027019_C_T	X	14027019	Other	14027017	SKCM
			.	4/4	TCGA-G3-A7M6_X_14027290_T_A	X	14027290	Splice Site	14027281	LIHC
**HNRNPL**	----	MES	.	9/12	TCGA-CV-A45Q_19_39329240_T_C	19	39329240	Splice Site	39329226	HNSC
**LUC7L**	down	----	6/10	.	TCGA-ZF-A9R7_16_249172_G_T	16	249172	Silent	249174	BLCA
**NONO**	----	PN	3/12	.	TCGA-YC-A9TC_X_70510515_G_C	X	70510515	Missense	70510517	BLCA
**RBFOX2**	----	PN	.	2/13	TCGA-34-5241_22_36236390_T_C	22	36236390	Other	36236394	LUSC
**RBM39**	----	MES	.	2/16	TCGA-34-A5IX_20_34328745_C_A	20	34328745	Splice Site	34328727	LUSC
**RBM5**	----	MES	.	13/24	TCGA-50-5068_3_50145581_G_A	3	50145581	Splice Site	50145585	LUAD
			.	3/24	TCGA-UY-A78L_3_50131152_G_A	3	50131152	Splice Site	50131154	BLCA
**SCAF4**	down	----	.	7/19	TCGA-G2-A3IE_21_33069064_C_T	21	33069064	Splice Site	33069061	BLCA
**SMG7**	----	MES	.	18/22	TCGA-D5-6924_1_183518342_G_A	1	183518342	Splice Site	183518360	COAD
			9/23	.	TCGA-EE-A2GM_1_183502377_C_T	1	183502377	Missense	183502390	SKCM
**SRRM1**	----	MES	9/17	8/16	TCGA-BR-8686_1_24981344_AGA_-	1	24981344	Splice Site	24981348	STAD
**SRSF6**	down	MES	.	5/5	TCGA-85-A4JC_20_42089342_G_T	20	42089342	Splice Site	42089348	LUSC
**THOC5**	----	MES & PN	.	4/19	TCGA-22-5472_22_29938946_C_A	22	29938946	Splice Site	29938937	LUSC
**THRAP3**	----	MES	5/12	.	TCGA-J2-A4AD_1_36755327_C_A	1	36755327	Silent	36755325	LUAD
**UBTF**	----	PN	.	10/20	TCGA-P3-A6T5_17_42288700_C_G	17	42288700	Splice Site	42288694	HNSC
			20/21	.	TCGA-AJ-A8CT_17_42284837_G_A	17	42284837	Silent	42284838	UCEC
**ZNF326**	down	MES	8/12	.	TCGA-FS-A1Z0_1_90483007_T_G	1	90483007	Missense	90483003	SKCM

Supplementary Table 18: Differentially spliced/expressed RBPs in GSCs frequently mutated according to Seiler *et al.*

	GSCs		Mutations Seiler et al.			
**RBP**	**Mesenchymal**	**AS.events**	**percentage.non.silent.mutation**	**percentage.hotspot**	**percentage.LoF**	**tcga.cohort**
**CLK4**	down	MES & PN	1.14, 0.43, 0.9, 0.95, 0.94, 0.17, 0.35, 0.35, 1.72, 0.35, 1.21, 0.68, 0.14, 0.31, 0.43, 0.61, 0.58, 0.32, 0.95, 1, 3.19	40, 20, 0, 0, 0, 0, 0, 0, 0, 50, 0, 25, 0, 50, 100, 16, 0, 0, 20, 20, 16.67	0, 40, 0, 40, 0, 100, 0, 0, 60, 50, 0, 0, 0, 50, 0, 22.67, 0, 0, 40, 20, 22.22	BLCA, BRCA, CESC, COAD, ESCA, **GBM**, HNSC, KIRC, KIRP, LGG, LIHC, LUAD, LUSC, OV, PAAD, pancan, READ, SARC, SKCM, STAD, UCEC
**DDX50**	----	MES	1.09, 1.14, 0.43, 0.6, 1.53, 1.42, 0.17, 0.18, 1.77, 0.71, 0.69, 0.5, 0.17, 1.7, 2.05, 0.29, 0.16, 0.43, 0.98, 0.59, 1.75, 1.13, 2.59, 6.38	0, 0, 0, 0, 12.5, 0, 0, 0, 0, 0, 0, 0, 0, 0, 0, 0, 0, 0, 6.67, 0, 33.33, 0, 0, 16.67	100, 20, 20, 0, 37.5, 0, 100, 0, 0, 25, 0, 100, 0, 0, 16.67, 50, 0, 100, 30, 0, 66.67, 0, 46.15, 41.67	ACC, BLCA, BRCA, CESC, COAD, ESCA, **GBM**, HNSC, KICH, KIRC, KIRP, LAML, LGG, LIHC, LUAD, LUSC, OV, PAAD, pancan, PRAD, READ, SKCM, STAD, UCEC
**DHX9**	----	PN	4.34, 1.04, 2.1, 1.96, 3.82, 1.89, 0.33, 0.88, 0.53, 1.37, 0.35, 2.91, 2.56, 1.15, 0.47, 0.43, 1.73, 2.34, 3.4, 2.99, 9.57, 3.51	0, 0, 14.29, 0, 15, 0, 0, 0, 0, 0, 50, 0, 0, 0, 0, 0, 5.69, 0, 5.56, 6.67, 9.26, 0	10.53, 8.33, 0, 0, 30, 25, 50, 0, 33.33, 0, 0, 16.67, 6.67, 12.5, 33.33, 100, 21.33, 0, 11.11, 40, 35.19, 0	BLCA, BRCA, CESC, CHOL, COAD, ESCA, **GBM**, HNSC, KIRC, KIRP, LGG, LIHC, LUAD, LUSC, OV, PAAD, pancan, READ, SKCM, STAD, UCEC, UCS
**HNRNPD**	----	MES & PN	0.91, 0.17, 0.3, 1.15, 1.79, 0.47, 0.88, 0.18, 0.34, 0.17, 0.24, 0.51, 0.43, 0.57, 0.2, 0.64, 1.13, 0.4, 0.19, 4.43, 1.75	0, 0, 0, 0, 0, 0, 0, 0, 0, 0, 0, 0, 0, 0, 0, 0, 0, 0, 0, 0, 0	50, 50, 100, 0, 50, 0, 40, 0, 0, 100, 100, 33.33, 66.67, 33.33, 100, 0, 0, 0, 0, 40, 0	BLCA, BRCA, CESC, COAD, DLBC, ESCA, HNSC, KIRC, KIRP, LGG, LIHC, LUAD, LUSC, pancan, PRAD, SARC, SKCM, STAD, THCA, UCEC, UCS
**HNRNPDL**	----	PN	1.09, 1.37, 0.35, 0.9, 2.48, 0.47, 0.83, 0.88, 0.18, 0.69, 0.5, 0.17, 0.17, 0.72, 0.16, 0.67, 0.2, 0.58, 0.32, 0.38, 1.8, 0.38, 2.84	0, 0, 0, 0, 0, 0, 0, 20, 0, 0, 0, 0, 0, 20, 0, 3.66, 0, 0, 0, 0, 0, 0, 6.25	0, 0, 50, 33.33, 46.15, 0, 20, 60, 0, 0, 0, 100, 100, 0, 100, 32.93, 0, 0, 0, 100, 55.56, 0, 25	ACC, BLCA, BRCA, CESC, COAD, ESCA, **GBM**, HNSC, KIRC, KIRP, LAML, LGG, LUAD, LUSC, OV, pancan, PRAD, READ, SARC, SKCM, STAD, THCA, UCEC
**HNRNPH3**	----	PN	0.68, 0.43, 0.95, 0.47, 0.33, 0.18, 0.34, 0.5, 0.17, 0.58, 0.43, 0.45, 0.39, 0.58, 1.32, 1, 2.66	0, 0, 0, 0, 0, 0, 0, 0, 0, 25, 0, 7.27, 0, 0, 0, 0, 20	0, 20, 20, 100, 0, 0, 0, 100, 0, 25, 0, 16.36, 0, 0, 28.57, 0, 13.33	BLCA, BRCA, COAD, ESCA, **GBM**, HNSC, KIRP, LAML, LUAD, LUSC, PAAD, pancan, PRAD, READ, SKCM, STAD, UCEC
**HNRNPL**	----	MES	0.68, 0.35, 0.6, 4.01, 0.47, 0.33, 1.23, 0.18, 0.17, 0.73, 0.43, 0.31, 0.75, 1.17, 2.46, 1.4, 3.55	0, 0, 0, 4.76, 0, 50, 0, 0, 0, 0, 0, 0, 3.26, 0, 0, 14.29, 0	33.33, 25, 50, 57.14, 100, 0, 42.86, 0, 0, 33.33, 0, 50, 31.52, 50, 7.69, 14.29, 25	BLCA, BRCA, CESC, COAD, ESCA, **GBM**, HNSC, KIRC, LGG, LIHC, LUSC, OV, pancan, READ, SKCM, STAD, UCEC
**NOVA1**	down	----	1.09, 0.91, 0.17, 0.9, 3.63, 0.89, 0.94, 0.7, 0.34, 0.17, 1.46, 3.76, 2.16, 0.31, 0.43, 1.47, 0.39, 2.92, 0.32, 6.05, 2.79, 0.81, 6.91, 1.75	0, 0, 50, 0, 0, 0, 0, 0, 0, 0, 0, 13.64, 13.33, 0, 0, 9.5, 0, 20, 100, 6.25, 28.57, 0, 7.69, 0	0, 0, 0, 0, 52.63, 0, 0, 0, 0, 100, 33.33, 13.64, 6.67, 0, 100, 21.23, 0, 0, 0, 21.88, 14.29, 0, 28.21, 0	ACC, BLCA, BRCA, CESC, COAD, DLBC, ESCA, HNSC, KIRP, LGG, LIHC, LUAD, LUSC, OV, PAAD, pancan, PRAD, READ, SARC, SKCM, STAD, THYM, UCEC, UCS
**NSRP1**	----	MES & PN	0.68, 0.26, 0.6, 1.53, 0.33, 0.53, 0.34, 0.73, 1.03, 0.29, 0.31, 0.63, 0.2, 0.58, 0.32, 1.89, 1.2, 4.08	0, 0, 0, 0, 0, 0, 0, 0, 0, 0, 0, 0, 0, 0, 0, 0, 0, 0	33.33, 0, 0, 37.5, 0, 0, 0, 66.67, 0, 0, 0, 18.18, 0, 0, 0, 0, 33.33, 26.09	BLCA, BRCA, CESC, COAD, **GBM**, HNSC, KIRP, LIHC, LUAD, LUSC, OV, pancan, PRAD, READ, SARC, SKCM, STAD, UCEC
**PABPC1**	up	----	1.09, 1.6, 0.26, 0.3, 2.29, 1.79, 0.47, 0.18, 0.35, 0.35, 0.24, 0.85, 0.86, 0.31, 0.72, 0.58, 0.32, 2.08, 1, 0.19, 3.9, 1.75	0, 0, 0, 100, 16.67, 0, 0, 0, 0, 50, 0, 0, 0, 0, 17.05, 100, 0, 9.09, 0, 0, 40.91, 0	0, 14.29, 0, 0, 16.67, 100, 0, 0, 0, 50, 0, 0, 0, 0, 12.5, 100, 0, 0, 0, 0, 18.18, 0	ACC, BLCA, BRCA, CESC, COAD, DLBC, ESCA, HNSC, KIRC, LGG, LIHC, LUAD, LUSC, OV, pancan, READ, SARC, SKCM, STAD, THCA, UCEC, UCS
**PHF5A**	----	PN	0.23, 0.17, 0.35, 0.88, 0.35, 0.35, 0.43, 0.16, 0.2, 0.57, 0.4, 0.18	0, 100, 0, 100, 0, 100, 66.67, 47.37, 0, 66.67, 50, 0	0, 0, 50, 0, 0, 0, 33.33, 10.53, 0, 0, 0, 0	BLCA, **GBM**, HNSC, KICH, KIRC, LGG, LUSC, pancan, PRAD, SKCM, STAD, UCEC
**PRPF40B**	----	MES & PN	0.91, 0.43, 1.2, 2.86, 0.94, 1.16, 1.58, 0.88, 0.53, 0.69, 0.52, 1.71, 0.86, 0.16, 1.17, 0.2, 2.92, 0.32, 3.21, 2.99, 5.67	0, 0, 0, 6.67, 0, 0, 0, 0, 33.33, 0, 0, 10, 16.67, 0, 6.29, 100, 0, 0, 0, 0, 12.5	0, 20, 50, 26.67, 0, 14.29, 11.11, 0, 0, 0, 0, 10, 33.33, 0, 16.08, 0, 0, 100, 0, 33.33, 15.62	BLCA, BRCA, CESC, COAD, ESCA, **GBM**, HNSC, KICH, KIRC, KIRP, LGG, LUAD, LUSC, OV, pancan, PRAD, READ, SARC, SKCM, STAD, UCEC
**PRPF4B**	----	MES & PN	1.09, 1.83, 1.04, 0.9, 2.67, 0.47, 0.33, 1.23, 0.35, 1.37, 0.35, 0.73, 1.88, 0.86, 1.14, 0.47, 1.26, 0.59, 0.58, 0.64, 3.02, 2.79, 0.38, 6.21, 1.75	0, 0, 8.33, 0, 0, 0, 0, 14.29, 0, 0, 0, 0, 0, 0, 0, 33.33, 1.95, 0, 0, 0, 0, 0, 0, 0, 0	100, 25, 0, 33.33, 50, 100, 50, 0, 0, 25, 50, 33.33, 9.09, 0, 0, 33.33, 27.27, 0, 100, 0, 6.25, 78.57, 0, 31.43, 0	ACC, BLCA, BRCA, CESC, COAD, ESCA, **GBM**, HNSC, KIRC, KIRP, LGG, LIHC, LUAD, LUSC, MESO, OV, pancan, PRAD, READ, SARC, SKCM, STAD, THCA, UCEC, UCS
**QKI**	down	MES & PN	1.09, 0.68, 0.17, 0.3, 1.53, 1.16, 0.88, 0.69, 0.5, 0.17, 0.68, 0.72, 0.43, 0.74, 0.39, 0.58, 0.32, 1.89, 2.59, 3.72, 1.25	0, 0, 0, 0, 0, 0, 20, 0, 0, 0, 0, 0, 0, 4.44, 0, 100, 0, 0, 7.69, 4.76, 0	0, 33.33, 50, 0, 50, 57.14, 0, 0, 0, 0, 0, 20, 0, 42.22, 50, 0, 0, 60, 61.54, 52.38, 100	ACC, BLCA, BRCA, CESC, COAD, **GBM**, HNSC, KIRP, LAML, LGG, LUAD, LUSC, PAAD, pancan, PRAD, READ, SARC, SKCM, STAD, UCEC, UVM
**RBM25**	----	PN	1.83, 0.86, 2.1, 1.96, 2.1, 0.47, 0.5, 0.53, 0.88, 0.18, 0.69, 0.24, 0.85, 1.3, 0.31, 0.43, 1.22, 0.39, 2.34, 0.32, 3.78, 2.4, 0.67, 7.09, 1.75	12.5, 10, 14.29, 0, 0, 0, 0, 0, 100, 0, 0, 0, 0, 0, 50, 0, 16.78, 50, 0, 0, 5, 41.67, 0, 32.5, 0	12.5, 10, 0, 0, 9.09, 0, 33.33, 33.33, 0, 0, 25, 0, 0, 22.22, 50, 0, 12.75, 0, 0, 100, 0, 8.33, 0, 20, 0	BLCA, BRCA, CESC, CHOL, COAD, ESCA, **GBM**, HNSC, KICH, KIRC, LGG, LIHC, LUAD, LUSC, OV, PAAD, pancan, PRAD, READ, SARC, SKCM, STAD, TGCT, UCEC, UCS
**SF1**	----	MES	2.28, 0.35, 1.8, 2.1, 0.47, 0.5, 0.88, 0.35, 0.73, 2.56, 0.58, 0.31, 0.43, 0.95, 0.2, 0.58, 0.64, 2.08, 2.59, 3.72	40, 25, 50, 9.09, 0, 33.33, 20, 0, 0, 13.33, 0, 50, 0, 19.83, 0, 0, 0, 0, 23.08, 28.57	0, 0, 16.67, 27.27, 100, 0, 60, 0, 0, 26.67, 0, 0, 100, 16.38, 100, 0, 0, 0, 23.08, 9.52	BLCA, BRCA, CESC, COAD, ESCA, **GBM**, HNSC, LGG, LIHC, LUAD, LUSC, OV, PAAD, pancan, PRAD, READ, SARC, SKCM, STAD, UCEC
**SNRNP35**	down	----	1.09, 0.46, 0.26, 1.15, 0.47, 0.17, 0.35, 0.34, 0.17, 0.14, 0.43, 0.37, 0.58, 0.38, 0.6, 0.19, 0.81, 3.01	0, 0, 0, 0, 0, 0, 0, 0, 0, 0, 0, 0, 0, 0, 0, 0, 0, 0	0, 0, 66.67, 33.33, 100, 0, 0, 0, 0, 0, 0, 26.67, 0, 0, 66.67, 0, 0, 29.41	ACC, BLCA, BRCA, COAD, ESCA, **GBM**, HNSC, KIRP, LGG, LUSC, PAAD, pancan, READ, SKCM, STAD, THCA, THYM, UCEC
**SNRPN**	----	MES	1.09, 0.91, 0.26, 0.3, 1.53, 0.89, 1.42, 1.05, 0.35, 0.17, 0.49, 2.39, 1.44, 0.31, 0.43, 0.84, 0.58, 3.02, 2.2, 2.84	0, 25, 33.33, 100, 50, 0, 33.33, 16.67, 0, 0, 50, 14.29, 10, 0, 0, 20.39, 0, 12.5, 45.45, 6.25	0, 0, 33.33, 0, 12.5, 0, 0, 33.33, 0, 0, 0, 0, 20, 0, 0, 8.74, 0, 6.25, 0, 12.5	ACC, BLCA, BRCA, CESC, COAD, DLBC, ESCA, HNSC, KIRC, LGG, LIHC, LUAD, LUSC, OV, PAAD, pancan, READ, SKCM, STAD, UCEC

**THOC5**	----	MES & PN	2.05, 0.26, 0.9, 1.96, 2.48, 0.89, 0.35, 0.18, 0.34, 0.35, 0.49, 0.34, 1.15, 0.47, 1.3, 0.83, 0.39, 0.58, 0.32, 2.08, 1, 0.67, 0.38, 4.08, 1.75	0, 33.33, 0, 0, 15.38, 0, 0, 0, 0, 0, 0, 0, 0, 0, 0, 5.94, 0, 0, 0, 0, 0, 0, 0, 13.04, 0	11.11, 33.33, 33.33, 0, 7.69, 0, 0, 100, 0, 0, 0, 0, 25, 0, 0, 12.87, 50, 0, 0, 18.18, 0, 100, 0, 8.7, 0	BLCA, BRCA, CESC, CHOL, COAD, DLBC, HNSC, KIRC, KIRP, LGG, LIHC, LUAD, LUSC, OV, PAAD, pancan, PRAD, READ, SARC, SKCM, STAD, TGCT, THCA, UCEC, UCS
**THOC6**	up	----	0.46, 0.3, 0.57, 0.17, 0.35, 0.18, 0.35, 0.24, 1.03, 0.47, 0.43, 0.48, 0.2, 0.32, 1.32, 0.6, 4.08	0, 100, 0, 0, 0, 0, 0, 0, 0, 0, 0, 12.07, 0, 0, 0, 0, 26.09	0, 0, 0, 0, 0, 0, 0, 0, 0, 0, 6.9, 0, 0, 28.57, 33.33, 4.35	BLCA, CESC, COAD, **GBM**, HNSC, KIRC, LGG, LIHC, LUAD, OV, PAAD, pancan, PRAD, SARC, SKCM, STAD, UCEC
**THRAP3**	----	MES	3.88, 0.78, 1.5, 1.96, 2.67, 0.33, 0.7, 0.35, 0.69, 1.49, 0.52, 0.49, 1.88, 1.15, 1.14, 1.09, 0.87, 1.48, 0.39, 2.34, 0.32, 4.73, 3.39, 6.91	0, 0, 20, 0, 21.43, 50, 0, 0, 50, 33.33, 0, 0, 0, 0, 0, 0, 0, 11.05, 0, 0, 0, 8, 23.53, 17.95	41.18, 0, 40, 100, 21.43, 50, 25, 50, 50, 33.33, 0, 50, 18.18, 37.5, 100, 28.57, 0, 28.73, 50, 0, 100, 28, 41.18, 23.08	BLCA, BRCA, CESC, CHOL, COAD, **GBM**, HNSC, KIRC, KIRP, LAML, LGG, LIHC, LUAD, LUSC, MESO, OV, PAAD, pancan, PRAD, READ, SARC, SKCM, STAD, UCEC
**TIA1**	down	MES	0.23, 0.52, 0.3, 1.15, 0.47, 0.5, 0.18, 0.18, 0.17, 0.17, 1.3, 0.48, 0.2, 0.58, 0.32, 1.51, 1, 1.95, 1.75	0, 0, 0, 0, 0, 0, 0, 0, 0, 0, 0, 0, 0, 0, 0, 0, 0, 0, 0	0, 33.33, 100, 66.67, 0, 66.67, 100, 0, 100, 0, 0, 30.51, 0, 0, 100, 37.5, 40, 9.09, 0	BLCA, BRCA, CESC, COAD, ESCA, **GBM**, HNSC, KIRC, LGG, LUAD, LUSC, pancan, PRAD, READ, SARC, SKCM, STAD, UCEC, UCS
**U2AF1**	down	----	1.09, 1.83, 0.17, 0.6, 0.76, 0.17, 0.35, 0.03, 0.35, 2.22, 0.14, 0.16, 1.3, 0.63, 0.2, 0.58, 0.95, 1, 0.19, 3.01, 3.51	100, 12.5, 0, 100, 0, 0, 50, 0.86, 0, 92.31, 0, 0, 100, 48.05, 100, 0, 0, 0, 100, 52.94, 100	0, 0, 0, 0, 0, 0, 0, 0, 0, 0, 0, 0, 1.3, 0, 0, 0, 20, 0, 0, 0	ACC, BLCA, BRCA, CESC, COAD, **GBM**, HNSC, LAML, LGG, LUAD, LUSC, OV, PAAD, pancan, PRAD, READ, SKCM, STAD, THCA, UCEC, UCS
**ZCCHC8**	----	MES & PN	1.09, 2.74, 0.6, 1.8, 1.96, 1.72, 1.79, 0.94, 0.7, 0.88, 0.35, 0.34, 0.17, 0.24, 1.2, 0.58, 0.16, 0.43, 0.9, 1.75, 0.64, 1.32, 1.4, 0.19, 4.79	0, 16.67, 0, 0, 0, 0, 0, 0, 25, 0, 0, 0, 0, 0, 14.29, 25, 0, 0, 8.18, 0, 0, 14.29, 14.29, 0, 7.41	0, 8.33, 14.29, 0, 0, 11.11, 0, 0, 0, 0, 0, 100, 0, 100, 28.57, 0, 100, 0, 18.18, 0, 0, 14.29, 14.29, 0, 37.04	ACC, BLCA, BRCA, CESC, CHOL, COAD, DLBC, ESCA, HNSC, KICH, KIRC, KIRP, LGG, LIHC, LUAD, LUSC, OV, PAAD, pancan, READ, SARC, SKCM, STAD, THCA, UCEC

Supplementary Table 19: Differentially expressed lncRNAs between mesenchymal (MES) and proneural (PN) GSCs samples.

Up-regulated lncRNAs in MES (down-regulated in PN)		
**lncRNA**	**log2FoldChange**	**FDR**
ADIRF-AS1	8.146945972	3.93E-36
RP11-169F17.1	9.033911847	1.37E-30
HCG11	6.647723412	3.53E-25
MIR222HG	4.064738431	3.46E-23
RP11-66B24.7	8.978200339	8.13E-23
LINC00960	10.26330846	2.66E-19
RP11-420A23.1	3.684649121	4.13E-19
LINC00470	10.06050549	1.29E-18
LINC01123	7.706435703	1.45E-18
LINC01436	9.962790407	3.64E-18
LINC00707	8.816289946	5.03E-18
CTC-459F4.3	2.972995332	7.19E-18
AC005301.9	10.40930029	1.21E-17
RP11-30P6.6	5.681131516	2.14E-17
AC003092.1	10.50044544	3.23E-17
LINC00857	8.351658274	5.01E-17
RP5-884M6.1	9.72557049	8.11E-17
RP11-366H4.1	10.03231099	3.19E-16
FAM201A	9.935176618	5.54E-16
LINC01249	9.35697513	5.98E-16
RP11-497G19.1	7.366430786	7.63E-16
RP11-108M9.4	6.808368938	8.17E-16
UBR5-AS1	3.568858858	1.20E-15
RP11-575F12.3	7.440012405	1.80E-15
LINC00944	9.821144795	2.02E-15
RP11-148B18.4	9.230758474	2.71E-15
LINC01139	9.226548995	3.83E-15
MSC-AS1	7.978350409	5.06E-15
LINC01605	7.765975334	1.00E-14
CASC9	9.550356712	1.20E-14
RP11-66B24.2	9.683498973	1.23E-14
CTB-140J7.2	9.055864721	1.87E-14
MIR137HG	9.463462249	2.91E-14
RP4-647C14.2	9.440803875	3.12E-14
LINC00910	4.052061325	4.78E-14
TRHDE-AS1	9.426571735	5.89E-14
RP11-129M6.1	9.283071256	1.02E-13
PCAT7	7.012366968	1.83E-13
RP11-390F4.3	4.822466039	6.69E-13
LINC01533	9.351719175	6.69E-13
LINC00662	2.658992173	1.17E-12
RP11-262H14.3	4.33804083	1.57E-12

Down-regulated lncRNAs in MES (up-regulated in PN)		
**lncRNA**	**log2FoldChange**	**FDR**
XIST	-10.22135421	1.92E-94
SOX2-OT	-9.101572388	5.55E-43
LINC00511	-4.72758947	8.94E-35
LINC00665	-6.508442983	2.47E-32
LINC00461	-4.071773964	3.20E-25
MIR34AHG	-7.151118111	2.77E-21
RP11-196G18.22	-9.157734844	1.05E-20
AC074289.1	-6.611724699	5.13E-20
LINC01963	-5.64863106	1.60E-17
AC018647.3	-10.62948487	1.29E-16
HAGLR	-10.37607784	1.88E-16
SOX21-AS1	-10.67844791	4.24E-16
RP11-288C18.1	-3.662952762	5.00E-16
RP11-210M15.2	-7.961094847	2.74E-15
MEG3	-9.884091578	3.89E-15
RP11-566E18.1	-6.093151216	3.97E-15
RP11-161M6.2	-9.861994023	4.74E-15
ZNF528-AS1	-10.33612284	2.16E-14
RP11-275H4.1	-9.076431963	3.29E-14
CTD-2353F22.2	-4.084734896	1.33E-13
LINC01896	-8.717255997	5.77E-13
AF131215.2	-9.011129608	6.69E-13
CASC15	-9.336123802	1.20E-12
RP3-525N10.2	-6.660068513	1.23E-12
RP11-119F7.5	-8.593043845	1.63E-12
RP11-629G13.1	-8.379396214	1.00E-11
RP11-403A3.3	-9.883416675	1.05E-11
MIR99AHG	-3.934916524	1.08E-11
AC108142.1	-8.919909338	1.48E-11
AC005154.6	-2.818045991	1.77E-11
LINC01268	-8.650840613	3.20E-11
RP11-436K8.1	-9.1534206	3.66E-11
RP11-893F2.14	-8.211591997	4.55E-11
AF131215.9	-8.346895896	7.37E-11
RP1-140K8.5	-7.58448199	8.15E-11
ZNF561-AS1	-2.939397831	9.05E-11
RP11-104N10.2	-2.659618794	1.14E-10
RP5-882C2.2	-3.146614921	1.50E-10
MIAT	-7.634121988	1.63E-10
LINC00641	-2.919069966	2.50E-10
CTC-260E6.4	-8.239041606	2.69E-10
TPT1-AS1	-2.889017128	3.55E-10

RP11-221N13.3	5.221276331	1.97E-12
AP001065.15	8.370716353	2.71E-12
CASC8	7.391618522	2.71E-12
RP11-121P12.1	9.043223723	2.97E-12
KC6	8.731874238	4.18E-12
LINC00668	8.742740586	4.45E-12
RP11-879F14.2	7.167672853	5.85E-12
LINC00937	8.783839089	8.75E-12
LINC01615	8.243950135	1.01E-11
CTD-2162K18.4	3.338334789	1.38E-11
CTD-2054N24.2	8.481067491	2.59E-11
RP11-1149O23.3	8.06830903	3.64E-11
CTD-2574D22.4	2.185932751	4.70E-11
LINC00704	8.789363096	4.84E-11
FENDRR	8.871195576	8.73E-11
AC006946.16	6.245377065	1.48E-10
LINC02163	8.256808714	1.70E-10
RP11-66B24.4	8.411213248	1.78E-10
CTC-308K20.1	4.217809789	3.39E-10
RP11-268G12.1	4.871952911	3.91E-10
RP4-756H11.3	3.053415812	4.84E-10
STXBP5-AS1	4.832831339	6.31E-10
RP13-270P17.3	4.51796317	6.97E-10
RP4-555D20.4	3.567185961	8.62E-10
RP11-962G15.1	5.492674363	1.17E-09
RP11-471M2.3	7.569974173	1.21E-09
RP11-191L9.4	7.544550047	1.24E-09
RP11-419I17.1	5.188552613	2.55E-09
MNX1-AS1	4.331028456	2.88E-09
AC116614.1	5.717797695	3.06E-09
TSPEAR-AS2	7.861341045	4.06E-09
GATA2-AS1	7.511897668	4.17E-09
LINC01106	7.775865143	4.51E-09
RP11-440D17.3	7.420510911	4.73E-09
LINC00941	4.901685682	5.11E-09
TSPEAR-AS1	7.720834221	5.64E-09
RP11-359K18.4	3.529648568	5.78E-09
AC141928.1	6.975413428	6.36E-09
RP1-102E24.8	7.947629154	6.95E-09
RP11-408B11.2	7.274106998	7.61E-09
LBX2-AS1	4.21313293	8.53E-09
RP4-639F20.1	2.662459679	8.82E-09
EML2-AS1	4.336272386	1.09E-08
RP11-63G10.4	7.212949803	1.11E-08
RP11-809O17.1	4.287316525	1.19E-08
RP11-328K4.1	7.624906303	1.43E-08
SNHG8	3.401142657	2.65E-08

AC114730.3	-8.540168498	3.82E-10
RP11-444D3.1	-8.218665966	4.12E-10
RP11-981G7.6	-4.471913906	5.13E-10
CTC-559E9.5	-8.344951059	6.27E-10
RP11-398K22.12	-2.943377545	9.86E-10
RP11-159D12.2	-2.798991565	1.02E-09
RP1-40E16.9	-8.392876913	1.07E-09
RP11-196G18.24	-7.657098272	1.30E-09
CTC-260E6.6	-8.030632831	1.49E-09
FGF14-AS2	-7.851104231	2.11E-09
MIR3681HG	-8.879008143	2.81E-09
ZNF529-AS1	-5.273696736	2.83E-09
FAM212B-AS1	-6.817557936	3.47E-09
LINC00997	-2.566414249	5.29E-09
LINC02199	-7.534188611	5.40E-09
CTD-2619J13.17	-7.692717819	6.18E-09
RP11-71N10.1	-8.619040808	6.40E-09
RP11-15H20.6	-5.437567657	6.65E-09
LINC01550	-8.085630697	7.55E-09
ZNF582-AS1	-7.903489168	9.86E-09
LINC01535	-7.587569134	1.12E-08
LINC00403	-7.699092752	1.72E-08
RP11-159D12.8	-2.225130911	1.75E-08
RP11-713M15.2	-7.552931119	2.17E-08
ATP2B1-AS1	-4.769950165	2.53E-08
CTD-2291D10.4	-7.697930084	2.78E-08
RP11-448P19.1	-8.466375954	2.88E-08
RP4-751H13.7	-4.908088963	3.87E-08
RP11-223I10.1	-4.899060475	4.30E-08
RP11-472N13.3	-7.835931094	4.56E-08
LINC01105	-8.210805291	4.78E-08
RP4-756G23.5	-7.97797169	5.04E-08
PTPRD-AS1	-7.51830869	5.39E-08
RP11-496D24.2	-7.031095606	5.62E-08
ST7-AS1	-4.374864479	6.18E-08
MAMDC2-AS1	-3.933905485	7.18E-08
LINC00265	-1.778749128	7.71E-08
RP1-193H18.2	-4.664498604	8.02E-08
RAMP2-AS1	-8.60775794	8.64E-08
FTX	-2.006892193	1.18E-07
LINC01415	-8.371321319	1.48E-07
RP11-481J2.4	-7.144945655	1.76E-07
RP11-395L14.18	-2.820322349	1.77E-07
HAND2-AS1	-7.871059918	1.90E-07
C6orf3	-3.798519763	1.94E-07
RP11-57H14.4	-2.092178357	2.09E-07
RP5-1024N4.4	-7.130551155	2.15E-07

RP11-416N2.4	5.710982828	2.69E-08
RP11-497G19.2	7.584318915	3.32E-08
RP11-295G20.2	2.273079322	4.32E-08
SZT2-AS1	7.536631183	4.74E-08
CTD-2207P18.2	7.554943496	6.98E-08
UPK1A-AS1	7.064243249	6.99E-08
CTD-2587H24.5	7.513565712	7.00E-08
CTD-2066L21.3	6.285733224	7.90E-08
RP11-1038A11.1	7.25275581	8.52E-08
LINC00623	3.533898273	9.56E-08
RP11-89C3.4	7.943650619	1.01E-07
LINC00689	7.919931953	1.49E-07
LINC01162	7.206055523	1.50E-07
LINC00702	6.819882379	1.58E-07
CTD-2311M21.3	7.220608546	1.59E-07
AC060834.3	7.227385695	1.77E-07
CTD-2303H24.2	4.704207831	1.86E-07
RP11-15A1.2	6.787720725	1.88E-07
RP5-965G21.4	4.610304614	2.04E-07
EGFR-AS1	7.467470445	2.19E-07
RP11-467L13.7	3.326213921	3.44E-07
AF241725.6	7.924878636	3.66E-07
LINC01583	6.789227152	4.51E-07
LURAP1L-AS1	3.789069238	4.54E-07
RP11-656D10.3	3.355014087	5.27E-07
LVCAT1	5.211867124	6.09E-07
NOVA1-AS1	2.538877294	6.37E-07
RP11-440D17.4	7.058620585	6.76E-07
OSMR-AS1	4.848799816	7.28E-07
MIR548XHG	6.517723896	8.96E-07
RP11-495K9.9	6.202264434	8.96E-07
AC007773.2	4.236120734	1.03E-06
AC156455.1	4.687323369	1.04E-06
RP11-25H12.1	6.900994063	1.07E-06
CYP4F26P	5.220397301	1.28E-06
LINC00659	6.365318386	1.52E-06
RP13-463N16.6	7.120883305	1.64E-06
PLBD1-AS1	6.911744437	1.87E-06
FAM95C	6.86653314	2.08E-06
LINC01036	6.818139675	2.35E-06
LINC01273	5.680137692	2.37E-06
RNF139-AS1	2.092008006	2.49E-06
RP11-284F21.7	4.128882055	2.63E-06
RP11-402J6.1	6.757541223	2.72E-06
LMLN-AS1	5.648446421	2.82E-06
RP11-519M16.1	6.69772685	2.85E-06
LINC00460	3.729442416	3.14E-06

CTC-543D15.8	-7.521818143	2.35E-07
ZNF790-AS1	-7.2818115	2.38E-07
RP11-471B22.3	-7.2094215	2.84E-07
CTD-2017D11.1	-3.960102808	2.97E-07
RP11-290O12.2	-7.111607088	3.32E-07
RP11-329B9.4	-3.828846578	3.74E-07
RP11-488C13.5	-2.246146887	4.77E-07
NNT-AS1	-2.064197224	5.02E-07
LINC00391	-7.146426634	5.71E-07
CTA-293F17.1	-4.702427301	5.76E-07
AC010729.1	-7.260223812	5.96E-07
RP11-710C12.1	-6.584402572	6.24E-07
CTD-2587H19.2	-6.980442654	6.68E-07
RP11-617F23.1	-3.124766954	6.90E-07
XXbac-BPG283O16	-2.719804735	7.12E-07
RP11-1148L6.8	-2.711128153	7.16E-07
RP11-274B21.10	-7.017479016	1.01E-06
RP11-277P12.20	-2.86194715	1.03E-06
RP11-966I7.2	-7.196263744	1.09E-06
RP11-25K19.1	-5.405367611	1.31E-06
CTD-2541J13.1	-3.859070692	1.42E-06
RP11-4O1.2	-4.615001879	1.45E-06
GS1-24F4.2	-6.779841943	1.45E-06
RP11-679B19.1	-8.107790291	1.51E-06
RP11-225B17.2	-2.395268743	1.81E-06
AP002954.3	-3.351638835	1.87E-06
LPP-AS2	-3.390293858	1.92E-06
H19	-5.533234603	2.07E-06
NR2F1-AS1	-2.868865851	2.32E-06
RP11-823E8.3	-4.921400554	2.60E-06
DNM3OS	-7.790383001	2.74E-06
RP11-16P6.1	-4.139371016	3.47E-06
RP11-126K1.6	-3.730455453	3.98E-06
RP11-737O24.5	-6.683925926	4.25E-06
AC078842.3	-6.312706899	5.01E-06
RP11-283I3.6	-2.064384119	5.27E-06
RP11-257O5.2	-1.980800207	5.32E-06
AC064875.2	-8.268709306	5.72E-06
RP11-715J22.6	-3.397527777	5.97E-06
RP11-742D12.2	-6.600456329	6.20E-06
ATP6V0E2-AS1	-2.617283231	6.45E-06
LINCR-0001	-3.583810268	7.15E-06
LINC00909	-1.795020664	7.52E-06
RP11-251G23.5	-2.710204464	7.65E-06
AC073343.13	-3.817835458	7.97E-06
LINC01224	-6.942475864	8.32E-06
RP11-399B17.1	-3.845322242	8.48E-06

RP11-49I11.1	3.617485545	3.44E-06
RP11-66B24.1	6.774858729	3.65E-06
RP11-575F12.1	6.731795954	3.72E-06
RP11-148O21.4	6.908853839	3.99E-06
RP11-326A19.4	4.369169949	4.03E-06
HCG25	2.351298305	4.45E-06
RP11-268G12.3	4.200333373	4.56E-06
RP11-403I13.5	6.607682454	5.12E-06
RP11-802E16.3	3.829069566	5.16E-06
RP11-888D10.4	2.615126859	5.27E-06
RP1-28O10.1	6.740643804	5.65E-06
LINC01037	6.270344811	6.20E-06
RP3-522D1.1	3.049267853	7.07E-06
LINC01703	3.063371686	7.75E-06
LINC01186	6.337343389	7.82E-06
DNAH17-AS1	6.671372493	8.96E-06
CTC-480C2.1	6.476276773	9.00E-06
GAPLINC	6.488946566	9.04E-06
LL22NC03-86G7.1	4.727001344	9.25E-06
AC073130.3	6.442162012	9.95E-06
CRNDE	1.661141577	1.07E-05
XXyac-YM21GA2.4	6.615020672	1.08E-05
RP11-54A9.1	7.48207227	1.12E-05
RP5-1139I1.1	6.931226549	1.15E-05
SNHG6	1.619245894	1.16E-05
RP11-490G2.2	6.510949254	1.22E-05
RP11-162D16.2	6.403115552	1.33E-05
RP11-60A8.1	7.059636969	1.42E-05
RP11-462L8.1	2.684911141	1.43E-05
RP11-535M15.1	6.836140007	1.53E-05
RP11-134G8.5	3.127231423	1.71E-05
LINC01694	4.575615416	1.74E-05
RP11-148O21.3	6.106927561	2.05E-05
RP13-977J11.2	4.165965675	2.22E-05
ENO1-IT1	2.386887831	2.46E-05
SENCR	6.08542527	2.56E-05
CEBPA-AS1	3.857018567	3.16E-05
HIF1A-AS2	6.404846376	3.20E-05
RP11-527N22.2	5.339134282	3.42E-05
CTC-378H22.1	6.534646783	3.76E-05
RP11-686D22.8	6.198503793	3.99E-05
RP11-148O21.6	6.315889341	4.01E-05
RP11-531A24.5	2.303603847	4.33E-05
LINC00706	6.476287527	4.44E-05
FSIP2-AS1	3.101133259	4.68E-05
AC012485.2	5.923739888	4.79E-05
AC007879.5	3.074641483	4.97E-05

RP11-999E24.3	-5.359679484	9.02E-06
RP5-981L23.7	-6.696184964	9.14E-06
RP11-834C11.4	-6.194158157	9.19E-06
RP11-212P7.2	-2.845628074	9.90E-06
ASB16-AS1	-1.933744054	1.04E-05
RP11-420L9.5	-1.908570577	1.10E-05
RP11-403A3.2	-7.184113199	1.17E-05
RP11-348P10.2	-1.86151317	1.23E-05
SNHG18	-4.647868931	1.37E-05
RP11-351J23.2	-7.008216051	1.41E-05
RP11-329B9.3	-5.279490129	1.42E-05
CCNT2-AS1	-2.887192815	1.44E-05
PROX1-AS1	-6.483430831	1.45E-05
RP11-395I6.3	-3.734767077	1.53E-05
MZF1-AS1	-4.109415081	1.56E-05
U52111.14	-5.209094491	1.56E-05
LINC01759	-6.155727325	1.58E-05
RP11-375N15.2	-5.839939169	1.74E-05
RP11-268J15.5	-3.199082895	1.80E-05
PSMA3-AS1	-1.50008569	1.84E-05
GLIDR	-2.364688409	1.86E-05
C8orf31	-6.714240074	1.88E-05
RP11-46H11.11	-5.332381628	1.88E-05
RP11-174J11.1	-6.328840649	2.05E-05
RP11-731C17.2	-3.268225973	2.30E-05
RP5-967N21.11	-2.324336409	2.30E-05
RP11-51F16.1	-4.936587761	2.35E-05
RP13-942N8.1	-3.265656338	2.40E-05
GS1-358P8.4	-1.446336752	2.47E-05
CH17-408M7.1	-6.258366558	2.57E-05
RP5-1039K5.19	-1.852020323	2.86E-05
TRAF3IP2-AS1	-1.702715019	3.10E-05
RP11-120J1.1	-3.974457425	3.13E-05
FAM66C	-1.945290987	3.52E-05
RP5-894A10.2	-2.171188768	3.73E-05
RP5-1054A22.4	-6.344397026	3.74E-05
FLJ16779	-6.872421382	3.78E-05
ZNF793-AS1	-6.754784127	3.80E-05
RP11-70D24.2	-6.968508177	4.04E-05
RP11-1263C18.2	-7.313387613	4.16E-05
CTD-2049O4.1	-7.321369781	4.22E-05
AC004540.4	-6.282644251	4.39E-05
AC009120.6	-1.504138068	5.08E-05
SLC26A4-AS1	-5.453726901	6.07E-05
RP11-298I3.6	-4.958802067	6.14E-05
TFAP2A-AS1	-2.578756204	6.18E-05
RP11-563K23.1	-6.952420153	6.18E-05

LINC01006	3.63077232	5.60E-05
AC079922.3	1.956902709	5.98E-05
KB-68A7.1	6.166678949	6.29E-05
AP003900.6	6.142594363	7.05E-05
AC073130.1	6.12655016	7.07E-05
RP11-280G9.1	6.171046787	7.41E-05
MYOSLID	6.251556237	8.30E-05
RP11-122G18.11	4.018316325	8.30E-05
RP11-148O21.2	6.292163975	8.38E-05
RP11-818F20.5	5.837404855	8.44E-05
AC116609.3	6.271703909	8.65E-05
RP11-796E10.1	6.028707466	8.66E-05
CTC-459F4.9	2.311443761	9.18E-05
RP4-561L24.3	2.730763156	9.65E-05
SNHG3	2.007679738	9.72E-05
RP11-336A10.5	6.036433974	9.75E-05
RP5-906A24.1	6.163773382	0.0001015202373
RP11-445O3.3	5.996668	0.0001047885947
RP11-789C2.1	6.134136909	0.000110518313
RP11-77H9.2	2.414492258	0.0001109149926
RP11-126K1.2	2.955735961	0.0001237668845
RP11-61A14.1	5.6362257	0.0001275645902
RP11-64C12.1	6.037386758	0.0001287785648
RP11-681B3.4	5.946072551	0.0001294839333
LRRC2-AS1	6.032954976	0.0001313836569
RP1-80N2.3	6.076198882	0.0001424505241
CTC-241N9.1	2.085569121	0.0001624255994
RP11-745L13.2	5.965543072	0.0001629744574
LINC01238	1.855748333	0.0001655287567
RP11-284F21.10	3.759829959	0.0001668059475
LINC01293	5.944346668	0.0001670993592
RP11-43A14.1	4.387826573	0.0001750318637
PIK3CD-AS2	2.914291017	0.0001805689676
RP11-445F12.1	3.584080174	0.0001880725427
CTD-2128A3.2	5.916777774	0.0001900227398
CAPN10-AS1	1.275388447	0.0001929530167
RP11-115J23.1	3.59549855	0.0002004776747
AC058791.1	2.103776712	0.0002022208292
LINC00397	5.833030396	0.0002076789859
RP3-414A15.2	5.833030396	0.0002076789859
RP11-553A21.3	5.668675854	0.0002120600755
HRAT17	6.195418077	0.0002233493012
RAD21-AS1	3.167226406	0.0002277363176
AC007879.2	4.331446467	0.0002325843762
AC115522.3	4.767627014	0.0002331553758
FOXP4-AS1	2.326531954	0.0002478962423
KB-1980E6.3	3.412296271	0.0002681835125

CTB-92J24.2	-5.314069527	6.34E-05
LINC01003	-2.501040256	6.39E-05
RP11-645C24.5	-6.087222557	6.49E-05
RP11-582J16.4	-4.940195472	6.60E-05
CTC-260E6.3	-6.190075006	6.62E-05
AC083843.4	-4.139200705	6.70E-05
RP11-625L16.1	-6.239954724	6.85E-05
RP11-73M18.8	-1.594714807	8.36E-05
RP11-479G22.8	-2.85231467	8.58E-05
RP11-546D6.3	-1.665107563	8.65E-05
RP11-1114A5.4	-2.634229881	8.80E-05
KLF3-AS1	-4.575331248	9.04E-05
LINC00326	-6.314611164	9.07E-05
LINC00664	-7.036373964	9.29E-05
RP11-1191J2.5	-6.320824449	9.92E-05
RP5-942I16.1	-6.014181696	0.0001022728821
RP11-565F19.2	-2.237585259	0.0001032761953
RP11-1191J2.2	-6.395026286	0.000105034038
AC108025.2	-6.017357396	0.0001211827081
RP11-873E20.1	-6.226084246	0.0001229669432
RP11-177H2.1	-6.058860997	0.0001237316106
RP11-172H24.4	-3.04103295	0.0001265054452
AP000473.8	-5.81538309	0.0001278147999
LINC01571	-6.934581678	0.0001285817886
CTD-2380F24.1	-5.961929438	0.000133566291
RP11-713C5.1	-6.876356427	0.0001355681751
HAS2-AS1	-4.085607506	0.000145027447
LINC00844	-6.506214183	0.0001455942328
RP11-75C10.7	-6.823811427	0.000155208407
LINC00908	-6.481107341	0.0001560257212
CTC-490E21.11	-2.791691542	0.0001629744574
RP11-13A1.1	-5.656338899	0.0001779992895
RP3-412A9.16	-5.700218034	0.000178039151
RP11-399J13.2	-3.953294004	0.000179960132
HOXD-AS2	-5.941264646	0.0001805001733
RP11-498P14.3	-2.986561638	0.0001840136747
LINC00977	-6.219898602	0.0001857006558
SLC25A25-AS1	-1.773493332	0.0001957125507
RP11-378A13.1	-3.932969614	0.0001961897978
CTB-12A17.2	-4.347383965	0.0002094631782
RP11-506M12.1	-2.336284409	0.0002171325614
RP11-231C18.1	-6.020036331	0.0002193220442
AC073283.4	-5.977321528	0.0002270424433
CTD-2003C8.2	-5.594257181	0.0002325843762
RP11-797A18.5	-2.60626251	0.0002400735245
ZSCAN16-AS1	-1.461219801	0.0002479286896
FOXG1-AS1	-6.018204201	0.0002485594216

RP11-85I17.2	1.952760501	0.0002794556833
C6orf99	5.335843395	0.000296850181
RP4-660H19.1	5.734910136	0.0003298900161
RP11-93B14.4	5.670451112	0.0003303264004
RP11-848P1.2	2.476435257	0.0003354439189
ZNF32-AS2	3.544314316	0.0003360913493
AC017101.10	5.833569888	0.0003380045622
AC006369.2	5.86520115	0.000353289208
RP11-289F5.1	4.967720654	0.0003563823617
CTC-378H22.2	5.863256032	0.0003609088144
RP11-195F19.9	4.469005451	0.0004238631088
AC009502.4	5.730004875	0.0004430834675
RP11-783K16.13	2.343228486	0.000449774211
OLMALINC	1.752506758	0.0004498855903
RP11-146F11.5	3.46808574	0.0004612645846
LINC01023	3.312964937	0.00049292833
LINC01291	5.777375091	0.0005150146816
A2M-AS1	4.168786915	0.0005431319448
RP11-1149O23.4	5.650212647	0.0005514826019
RP11-1038A11.3	5.598300563	0.0005962509155
LINC01611	5.665276992	0.0006123094382
CYYR1-AS1	5.796277869	0.0006165715929
TIPARP-AS1	1.715318172	0.0006259775515
LINC01011	1.594381048	0.0006314346565
CFAP58-AS1	5.230445092	0.0006387733307
XXyac-YX65C7_A	3.152361148	0.0006402732459
AP001596.6	3.794530851	0.000642315236
RP1-46F2.3	5.65892083	0.0006545522028
RP11-686D22.4	5.215504474	0.0006545533053
CTD-2020K17.1	3.574560661	0.0006555385044
CTD-2035E11.3	2.266749235	0.0006709769816
CTD-3162L10.1	3.682695686	0.0007170087374
LINC01385	5.213778355	0.0007681225893
LINC00887	4.768162992	0.0007718938547
CTC-296K1.4	3.998608616	0.0007742883328
RP11-255I10.2	5.566707451	0.0008192694951
RP11-313L6.2	5.542958397	0.0008296757669
SERTAD4-AS1	4.408977222	0.0008421646925
ASH1L-AS1	1.385697423	0.0009050364661
LINC01763	5.073877759	0.0009411342309
RP11-1094H24.4	3.435986713	0.000978736911
RP11-399K21.14	3.580469233	0.0009919611781
CTD-2527I21.5	5.707627463	0.001009396059
RP11-33E12.2	2.942980229	0.001049733793
RP11-337C18.8	1.595992218	0.001058565184
RP5-1071N3.1	3.232444987	0.001062444777
RP11-1C8.4	5.648537776	0.001063952771

TNKS2-AS1	-3.728040785	0.0002505536513
LINC01060	-4.844039641	0.0002649837324
BDNF-AS	-2.440499658	0.0002701641166
RP11-698N11.2	-5.905142346	0.0002839375426
RP11-218E20.3	-6.164438919	0.0002884489359
RP3-394A18.1	-6.54873568	0.0003054275043
LINC01748	-5.173272614	0.0003304188215
AC007204.2	-6.208465703	0.000337371729
RP11-384F7.2	-5.770543132	0.0003414203548
RP11-797A18.6	-2.336037687	0.0003469052726
RP11-838N2.5	-6.037508918	0.0003469971602
C9orf170	-5.720069529	0.0003627820659
RP11-77K12.9	-4.828734775	0.0003678902314
RP11-180M15.7	-2.099645006	0.0003789415833
CTD-2528L19.6	-1.793558835	0.0003862636578
BACE1-AS	-1.443267009	0.0003869969702
RP11-266K4.14	-4.242588344	0.000397881714
RP11-216B9.6	-2.91571161	0.0004165283251
RP5-1042I8.7	-3.113608267	0.0004197058299
LLNLR-245B6.1	-1.761195398	0.0004349915419
AF127936.9	-2.501819757	0.0004353287072
RP11-347E10.1	-5.757603834	0.0004553496524
CTB-113D17.1	-5.734937449	0.0004607993101
RP3-368A4.6	-1.449611238	0.0004745898413
RP11-227D13.1	-5.867579877	0.0005170571664
RP11-644A7.2	-5.790196387	0.0005276721712
RP11-428J1.5	-1.543597441	0.0005411830668
CTD-2516F10.2	-3.097854954	0.0005424818889
LINC01630	-5.607228072	0.0005489052178
AC017104.6	-3.316715648	0.0005752778421
CTD-3014M21.1	-4.237842183	0.0005861190858
RP11-263K19.6	-2.906382897	0.0006004662344
RP11-507K2.3	-5.740163394	0.0006139981934
CD27-AS1	-1.901100354	0.0006156871384
RP11-340F14.6	-6.094730703	0.0006597727109
RP5-912I13.2	-5.21900265	0.0006875965339
HCG17	-4.4800824	0.0007135491804
LIFR-AS1	-3.059186499	0.0007322952109
AC002454.1	-5.217373136	0.0007379084536
CTD-2017D11.2	-3.427087233	0.0007380361314
RP11-815I9.4	-1.355214018	0.0007654657756
RP5-1024N4.5	-6.026962016	0.000770880823
RP11-307B6.3	-5.297361506	0.0007717132749
MIMT1	-6.075412883	0.0007965113531
MESTIT1	-6.156109399	0.000840727958
LINC01102	-5.979861627	0.0008665028436
RP1-153G14.4	-3.352936442	0.0008724535267

RP11-800A3.4	3.836645469	0.001103710061
CTA-14H9.5	5.444560449	0.001111568331
RP11-585P4.5	2.303163707	0.001122770321
RP11-379B18.5	3.018498083	0.001126284887
LRRC3-AS1	5.440915985	0.001134270019
RP11-320G24.1	5.44144726	0.001135271104
RP11-344P13.6	5.019574774	0.001135717299
LINC01096	2.569687218	0.001152849704
RP11-666A8.8	2.71470148	0.001185413762
RP11-377G16.2	5.05269672	0.001208141242
RP11-397A16.1	5.144165161	0.001252642336
CTA-280A3.2	5.43109425	0.001261036766
MTUS2-AS1	4.327983052	0.00126518416
RP11-6D1.3	5.100909506	0.001274578173
PCBP3-OT1	4.988139472	0.001306281919
AC007319.1	3.659058233	0.001320099891
VIM-AS1	1.900326091	0.001325357573
LINC01137	3.235447082	0.001389626545
AC023590.1	5.440417548	0.001449039108
RP11-26J3.1	3.195826099	0.001540317144
CTD-3131K8.2	1.618109617	0.001550529686
AC006946.17	5.357788586	0.001586759001
RP11-108K3.1	5.359911445	0.001590979682
WWC2-AS2	3.74645957	0.00169144635
CASC20	5.373598885	0.001717624055
CTB-60B18.12	3.931782788	0.001731460144
GAS5	1.714774757	0.001737349134
LINC00858	5.202331	0.001792908341
RP11-273G15.2	4.355439323	0.001960726502
SERPINB9P1	4.380934561	0.001960820253
RP11-495K9.10	4.068974459	0.001973420312
CTD-2589M5.5	5.00840386	0.002049313577
RP11-190A12.8	4.239058034	0.00209242228
CTA-292E10.6	1.83530987	0.002103897253
RP11-276H7.2	4.864001821	0.002168345373
RP11-445O3.2	5.297465727	0.002174603479
ERVK-28	3.507293559	0.002219708396
UXT-AS1	2.187486049	0.002240510999
AC137932.6	4.936227677	0.002326603314
RP11-222K16.1	4.830431392	0.002339860971
CARD8-AS1	1.859352843	0.002363507089
RP11-91J19.3	3.229595167	0.00237987829
RP5-1011O1.2	3.750543005	0.0024213379
LINC01024	1.414845232	0.002426669236
RP11-783K16.5	2.211442807	0.002505096368
RP4-621F18.2	2.891474187	0.002586145421
RP11-686D22.5	5.227165141	0.002588758152

LINC01158	-3.599358667	0.0009065648927
RP11-644F5.11	-1.56797691	0.0009066423293
RP4-761J14.10	-5.526895507	0.0009086436691
RP11-78O7.2	-3.269803284	0.0009193049761
AFDN-AS1	-2.1176868	0.0009529911553
RP11-731J8.2	-6.106345858	0.00096371095
AP000289.6	-6.149069418	0.0009735564463
RP5-1159O4.2	-3.33637214	0.0009783005669
CTB-118P15.2	-5.481705306	0.0009795451864
CTC-467M3.1	-5.646775277	0.0009841545403
CTC-425O23.2	-2.852989458	0.001073961961
ARMCX5-GPRASP2	-1.407923176	0.00109723521
RP1-273N12.4	-4.680797573	0.001114511942
RP11-142E9.1	-2.148972429	0.001153749262
AC002456.2	-3.333117086	0.00119975203
CAHM	-2.066462593	0.001205708899
RP11-893F2.13	-2.327250988	0.001208141242
RP5-894A10.6	-1.410084025	0.00121887929
RP11-324E6.10	-5.020937672	0.001218901552
AP000254.8	-2.895415786	0.001234586927
AC053503.11	-5.392283488	0.00125124486
LINC00945	-5.54097168	0.001325653116
PLCG1-AS1	-2.769857823	0.001345851591
RP4-657D16.3	-3.717522492	0.001415189303
RP11-723O4.9	-2.963186604	0.00144225339
RP3-405J10.3	-2.720753833	0.001451610997
RP11-549B18.1	-2.066701039	0.001453948792
RP11-303E16.9	-2.946717768	0.001498140987
LINC01948	-5.764782325	0.001519939855
CTD-2621I17.3	-2.433641132	0.001532216372
CTC-429P9.3	-1.61425528	0.001584564787
RP11-141M1.3	-5.389178491	0.001586759001
RP11-1109F11.3	-1.584739307	0.001588949572
RP11-51J9.6	-4.941437238	0.001664504684
RP11-430H10.1	-5.529941441	0.001677124986
RP11-178H8.7	-1.996689343	0.001691175555
GAS1RR	-4.356538262	0.001723439264
AP006222.2	-5.409402215	0.001739006595
RP11-612B6.2	-5.2185601	0.001739823326
RP11-44F14.6	-2.711064773	0.001784799015
RP11-81H14.2	-5.286201455	0.001804927089
RP11-38M8.1	-5.989338989	0.001825357441
CBR3-AS1	-2.111612848	0.001848250624
RP11-1060J15.9	-5.057482317	0.001897250751
RP11-88E10.4	-2.970796319	0.001899397513
MIR181A2HG	-3.068725026	0.001971044384
RP11-495L19.1	-4.385950377	0.002059250533

RP11-982M15.6	4.795134696	0.002743005064
TARID	5.131806908	0.002771968959
CTD-2314G24.2	5.351285335	0.002777985103
CTD-2574D22.3	1.219204514	0.002957277444
AP000640.10	5.400424159	0.002995682983
RP11-533E19.5	2.854644329	0.003073410086
AC069277.2	5.147331362	0.003112925884
AP000695.4	3.416925837	0.003157362787
AC018816.3	5.17125313	0.003190497569
RP11-565A3.2	5.252153328	0.003195230488
RP11-930P14.2	4.416514667	0.003244161404
RP4-806M20.4	4.969661965	0.003280006796
RP11-284F21.9	2.870204056	0.003382508368
RP11-121L10.2	1.929467064	0.003489041343
UCHL1-AS1	3.427530133	0.003512105265
AC129492.6	4.059740766	0.003514426529
AC140912.1	3.255235499	0.003529503524
RP11-1334A24.5	2.375697794	0.003558685932
RP11-626P14.2	2.26797985	0.003569273891
CTD-2126E3.1	2.962621907	0.003576745562
RP11-700A24.1	2.393535101	0.003625553534
LINC00115	2.058579187	0.003644678278
LINC01776	3.983772495	0.003706084058
RP11-211G23.2	5.094660684	0.003707479166
RP11-114H21.2	5.281203818	0.003726359863
ALMS1-IT1	1.478924142	0.003776549229
AC005264.2	5.278574391	0.003777830725
AJ003147.9	3.526535287	0.003895413923
DLGAP4-AS1	3.802442206	0.004250083204
RP11-432J22.2	1.669411574	0.004252190209
RP11-504P24.9	1.962017455	0.004254237365
RP11-98I9.4	1.842933091	0.004333315917
RP11-359E10.1	3.480699906	0.004367681772
RP11-610P16.1	2.850455675	0.004433280276
RP11-547D24.1	4.721708797	0.004476192619
PVT1	1.306446058	0.004494224275
LINC01111	5.511718329	0.004537567949
HYMAI	5.040696403	0.004537567949
RP1-137D17.1	5.038749263	0.004627321859
HLA-F-AS1	1.5497679	0.004645809335
LL21NC02-1C16.2	5.890732138	0.004724494364
RP11-755F10.1	4.290032186	0.004897947718
XXbac-BPG294E2	2.530545502	0.004953593184
AC068831.10	2.283396586	0.004956503438
RP11-676J12.7	5.871415047	0.005231143184
CTD-3220F14.1	2.781303803	0.005275095619
PACERR	3.216647238	0.005341262143
RP11-73E17.2	-1.5225864	0.002066691171
RP11-379H18.1	-1.47162864	0.00208566031
RP11-454H19.2	-5.491275053	0.002117863573
CTC-301O7.4	-2.520257058	0.002121245305
CTD-2227E11.1	-5.715709335	0.002187760254
RP1-30M3.5	-2.957415119	0.002318239095
RP5-1085F17.3	-1.699213656	0.002380817185
AC005083.1	-5.100366778	0.002413394299
CTC-255N20.1	-5.90996629	0.002439508492
RP11-7F17.3	-5.580193964	0.00247372978
RP11-571L19.8	-4.096570865	0.002515623008
AP000280.66	-7.07685636	0.002526135113
RP11-517B11.7	-1.948887852	0.002565866236
RP11-49K24.8	-5.084806688	0.002582499042
RP11-455F5.3	-2.775083786	0.002586820684
AC104653.1	-3.300505301	0.002628005199
ZBED3-AS1	-1.506734757	0.00271888178
RP11-196G18.3	-3.786317056	0.002722278814
AP000708.1	-5.357866885	0.002751679981
ZNF638-IT1	-1.964433573	0.002868869478
RP11-70D24.3	-5.505695201	0.002968008946
BLACAT1	-5.30854716	0.002993764745
RP11-247A12.2	-4.169126105	0.003010826868
BAALC-AS2	-5.621475819	0.003052205524
RP11-429B14.4	-2.884900617	0.00313103042
RP11-620J15.1	-5.318300474	0.003237995702
AC097662.2	-1.687064586	0.0032674624
PTOV1-AS1	-1.975556696	0.003281406381
LINC00601	-3.655881329	0.003293974386
AC079584.3	-5.146737373	0.003569273891
RP5-1024C24.1	-5.999230278	0.003644599512
ADAMTS9-AS2	-5.996907911	0.003644678278
CHL1-AS2	-5.65075024	0.003741768487
RP11-204L24.2	-3.122698041	0.003824633773
RP11-447D11.3	-2.285101453	0.003834401889
LINC00638	-2.516873246	0.00386929974
LINC00506	-5.295590616	0.00387448858
RP11-345J18.2	-5.54459906	0.003906506877
RP11-429G19.3	-5.180231863	0.004224718689
ELOVL2-AS1	-5.065880739	0.004229916802
RP11-281O15.4	-5.048132452	0.004303880939
RP11-261P13.5	-5.345587797	0.004311879315
CTD-2310F14.1	-5.097612394	0.004314407439
RP11-333O1.1	-4.797569852	0.004356240433
RP11-229D13.3	-2.522981967	0.004358301733
ANKRD10-IT1	-1.541373901	0.004425009632
LINC00877	-5.162567193	0.004430997495

RP4-794I6.4	4.302509489	0.005583274299
RP6-24A23.3	4.523628477	0.00589811513
CTC-1337H24.4	3.823592819	0.006134441325
RP11-167H9.4	3.150506454	0.006292529877
MIR2052HG	3.947255514	0.006300265844
LINC01885	4.994167419	0.006321021797
AC131056.3	4.994167419	0.006321021797
AJ003147.8	3.284970252	0.006400022459
RP11-49I11.4	3.298447728	0.006716031652
LINC01202	5.044433095	0.006718757131
LINC01001	2.271547507	0.006862132394
LINC01939	6.274155912	0.007125126266
AC020571.3	4.550672319	0.007221518883
RP3-467K16.2	4.46813714	0.007460598712
AC092614.2	4.941359329	0.007464342372
CTD-2382E5.6	2.51016861	0.007465628382
RP13-143G15.4	4.43834854	0.007680267498
RP11-315O6.1	1.918246088	0.007885067528
RP11-415J8.5	2.871950971	0.007936253088
HAR1A	4.666604453	0.008044817922
LINC00526	1.255142528	0.008534755491
AC137932.4	2.795844251	0.00869507607
LINC01124	4.820811423	0.00877107676
TM4SF1-AS1	3.203756647	0.009164230146
RP11-798K23.1	4.808731238	0.0094402494
RP11-381K20.2	2.670945807	0.009598075674
RP11-460B17.2	4.468328266	0.009700297729
SNCA-AS1	4.835756356	0.009801529201
RP11-816J6.3	4.80383904	0.009874746881
LIPE-AS1	1.590611256	0.009882149942
CTD-2540B15.9	4.018448288	0.00988955589
RP11-107N15.1	4.349087981	0.009996760949
AP003068.23	2.087838199	0.0100518035
SFTA1P	3.346689327	0.01028588464
SLFNL1-AS1	1.774575445	0.01032988242
RP11-15A1.3	3.031428648	0.01041824749
RP4-536B24.4	3.687087304	0.01091224381
WT1-AS	2.909754676	0.0110624291
RP11-445P19.3	4.784105335	0.01113904435
RP11-503E24.2	1.464539684	0.01116260243
CTB-32O4.2	2.07953376	0.01132040195
CTD-2270P14.5	1.611690475	0.01137785432
AP001628.6	2.652537389	0.01166723233
LINC01021	1.955564405	0.0116975285
RP11-122G18.12	1.750285823	0.0117030675
AC034220.3	2.795064426	0.0117410892
AC092159.2	2.193511264	0.01210692174

LINC00606	-5.915208686	0.004529393032
DACT3-AS1	-4.634377855	0.004559218413
LINC00205	-1.177100916	0.00459041247
RP3-428L16.2	-2.843211281	0.004603675121
RP11-478J18.2	-5.016918499	0.004865294804
RP11-884K10.7	-1.241319978	0.005095643322
TAF1A-AS1	-1.711780788	0.005107535178
RP11-758P17.2	-3.714195116	0.00523631933
LINC02001	-1.277770011	0.005245208224
RP4-625H18.2	-4.934561949	0.005310336115
RAB11B-AS1	-2.034888011	0.005484735777
RP11-216B9.9	-3.234881605	0.00551751757
RP11-139H14.5	-4.122590574	0.005561575863
SPATA42	-5.071871399	0.005567303863
RBMS3-AS3	-2.808902002	0.005653211982
RP11-305E6.4	-1.44701567	0.005653211982
PTGER4P2-CDK2A	-2.953415243	0.005780874326
RP11-620J15.4	-4.943769079	0.00581547482
RP11-2E11.5	-5.760933414	0.005964505308
LINC01977	-5.32261339	0.006111137619
RP11-110I1.12	-2.361724833	0.006242885529
CTD-2373N4.3	-3.131337625	0.006244244755
ZEB2-AS1	-4.099506045	0.006310215433
RP4-635E18.8	-2.277617836	0.006381108126
LINC01778	-2.722293474	0.006404109579
NIFK-AS1	-1.50979	0.006581792306
LINC01089	-1.256446448	0.006649216337
RP1-101A2.1	-2.16468442	0.006717658182
RP11-1055B8.1	-4.764731927	0.006756104393
RP11-429P3.8	-2.212538116	0.006911578283
AC091133.1	-1.942806565	0.007032716316
RP11-259N19.1	-1.551587016	0.007164029932
NOP14-AS1	-1.292889805	0.0071673344
RP11-20I23.13	-5.036447104	0.00719956373
GABPB1-AS1	-1.12794287	0.007344285083
RP11-541N10.3	-2.198334071	0.007432138973
RP13-753N3.3	-1.939758783	0.007464342372
LINC01816	-4.92710743	0.00747520279
RP11-282O18.3	-1.395987859	0.007566092326
AC004893.11	-1.505077532	0.00774465452
RP11-303E16.2	-1.116020249	0.007846086901
RP3-425C14.4	-1.847167451	0.008006701946
RP11-277P12.9	-4.971186188	0.008118670935
CTD-2286N8.2	-2.935585777	0.008120622619
LINC01833	-5.271226429	0.008201056469
ERVK13-1	-1.346821894	0.008245782331
RP11-526I2.5	-2.412792585	0.008271190894

FMR1-AS1	3.142794257	0.0121972216	RP11-472N13.2	-4.464710927	0.008450219104
RP11-885N19.6	4.896083994	0.01226465106	GPR158-AS1	-4.848467894	0.008553432204
LINC01970	2.18814385	0.01232382156	AC009506.1	-1.829064139	0.008609605431
RP11-843B15.4	2.063634549	0.01244819334	CTC-429P9.5	-1.447075865	0.008652922341
LINC01239	3.6509458	0.01310638174	CTD-2116N20.1	-2.493858948	0.008759872539
CTD-2319I12.2	2.33651796	0.01327537354	AC003991.3	-4.831684984	0.008761441907
HCG27	1.663921782	0.01331123808	AC002550.6	-2.956937025	0.008784520128
RP11-286N22.14	2.374173794	0.0134862283	RP11-48B3.4	-2.734011924	0.008835094593
RP11-1398P2.1	4.229813996	0.01403510319	RP11-318C24.2	-4.828048624	0.009147507666
AP000442.4	3.40471652	0.01410817025	AC005220.3	-5.551988115	0.009426543604
KB-431C1.5	2.606676792	0.01425787821	CTA-276F8.1	-2.233223989	0.0094402494
GACAT2	3.202431636	0.01443440693	CTB-119C2.1	-2.21113906	0.009598075674
SNHG16	1.108452011	0.01444495472	LINC01578	-1.334542649	0.009873474105
LINC00346	2.516630852	0.01450628056	SATB2-AS1	-2.516153774	0.009929319141
LINC00882	2.586730527	0.0145691493	CTD-2506P8.6	-2.007486644	0.0100673699
FLJ21408	1.739172781	0.01465495581	RP11-259K15.2	-5.018307458	0.01012902648
RSF1-IT1	2.226558023	0.01476475356	RP11-568N6.1	-2.769320265	0.01017272629
LINC01572	1.39448343	0.0149485061	RP11-247A12.7	-5.018047244	0.0104112034
RP11-247L20.4	1.297895405	0.01499724708	RP11-996F15.6	-2.648966799	0.01063016375
RP11-482G13.1	4.725877673	0.01544297214	RP11-182L21.6	-1.329206658	0.01069523342
XXbac-BPG299F1	1.473385639	0.01560910182	PDCD4-AS1	-1.479755948	0.01069589917
LINC00622	2.778829799	0.01561320562	RP11-557L19.1	-4.256984938	0.01075240857
AC093732.1	4.611877005	0.01625136653	RP11-131L12.2	-4.429400759	0.01081304619
ZFPM2-AS1	2.865300646	0.01698869866	DLEU2L	-1.325731708	0.01087992414
C10orf25	2.148915277	0.01794711604	RBM26-AS1	-1.372105486	0.01108391598
RP3-467K16.4	2.82104963	0.01807675308	RP11-111K18.2	-2.32547559	0.01132040195
AC145343.2	3.750887357	0.01832311228	LINC01727	-5.094783739	0.01133708271
AC002076.10	3.84241051	0.01842606347	RP11-343L5.2	-2.09831483	0.01136507759
RP11-134G8.7	1.650136783	0.01842740977	AC009403.2	-1.503408273	0.01143030468
ATP6V1B1-AS1	3.433189361	0.01890187137	RP11-890B15.3	-1.77508915	0.01149966624
RP11-650K20.3	3.615188719	0.01921205146	RP11-46H11.12	-4.730923494	0.01160296881
LINC02057	4.12147217	0.01929912714	RP11-134K13.4	-3.565316139	0.01160795922
RP11-677M14.3	3.392028617	0.01952092811	RP11-582J16.5	-1.954152104	0.01160868639
RP11-7F18.2	1.500458157	0.01960752343	LINC02104	-4.951757049	0.01162481901
AC106786.1	4.191157465	0.01977993331	WDR86-AS1	-5.442248239	0.01163761728
RP11-63E9.1	4.798029568	0.0198209433	ZNF571-AS1	-4.974764195	0.01170403198
CH507-154B10.1	1.455764363	0.02028470541	AC093642.1	-2.599684197	0.01172659291
CTD-2337A12.1	4.537149604	0.02032954731	AC005519.4	-1.765263623	0.01181083695
LINC02086	2.70406007	0.0208398748	RP11-456K23.1	-5.421627125	0.0118534843
RP3-460G2.2	2.392090466	0.02127348231	CTC-498J12.1	-4.715600867	0.01241394295
ITGB2-AS1	4.593664851	0.0216808998	HCG22	-5.376293427	0.01290859019
SEMA6A-AS1	1.611079106	0.02188255209	RP11-395A13.2	-2.110592624	0.01298823947
RP11-14N7.2	4.478142569	0.02225577315	LINC01505	-3.330138445	0.01312200277
CTC-338M12.4	1.275170231	0.02234600247	AL022393.9	-2.869433763	0.0131653149
RP11-297A16.2	4.19827412	0.02245840739	LEF1-AS1	-4.864527245	0.01317131543
RP11-84D1.1	4.552179696	0.02250465587	RP11-496I9.1	-2.981307848	0.01322938674
PRKCQ-AS1	1.210084497	0.02254512085	LINC01132	-4.971933474	0.01375764852

AC005754.8	2.282792454	0.02300016749
AP001372.2	1.140514682	0.02328766813
AC053503.4	2.784715264	0.02356068188
SOCS2-AS1	2.678361743	0.02356068188
MIR4500HG	3.170335241	0.02370021912
XXbac-BPG181B2	3.435231655	0.02374267554
CTD-2540B15.10	3.480146378	0.02462087006
RP11-122K13.12	1.829609781	0.02484619539
RP11-736K20.5	2.270516973	0.02522601544
RP11-423H2.3	2.618659719	0.02526481134
RP11-297N6.4	3.098554344	0.02583155998
RP13-455A7.1	4.448132981	0.02606312664
RP1-266L20.2	4.485001717	0.0262392154
AC116609.2	2.735095742	0.02625603682
RP11-455F15.1	4.180136384	0.0263973349
RP11-259K5.2	1.403386795	0.02701010484
RP11-533E19.7	1.648595301	0.02704133112
CTC-453G23.8	2.202087822	0.02768265983
CTB-131B5.5	1.750013657	0.02837920971
RP11-77P6.2	1.167662649	0.02866036952
RP11-235E17.6	2.055833607	0.02879479414
RP11-375I20.6	2.511867114	0.02888306097
EBLN3P	1.252644577	0.02907481304
TMLHE-AS1	3.187322844	0.02935365836
SNHG12	1.162502945	0.0297142304
RP11-54C4.3	1.722094744	0.02973853888
GATA6-AS1	3.995721576	0.03019054622
AD000864.6	4.372146893	0.03034770741
RP3-467K16.7	4.411508278	0.03040004781
TMEM9B-AS1	1.529967684	0.03058264342
STARD4-AS1	1.96123643	0.03065540496
RP11-481J2.3	1.653053125	0.03115767131
RP11-501C14.5	4.024117001	0.03129581396
CRYM-AS1	2.390710586	0.03146325358
RP11-239A17.1	4.321337041	0.0318658036
FOXC2-AS1	4.53987788	0.0319408992
WFDC21P	4.438280554	0.03299355355
OTUD6B-AS1	1.051572853	0.03304121069
AP001258.4	1.046833372	0.03309785096
RP11-95P2.3	4.311069376	0.033131005
RP11-497E19.1	4.428545803	0.03337487315
RP5-901A4.1	1.916525277	0.03360784306
PSMD6-AS2	1.072555648	0.03431800531
RP11-715F3.2	1.601567836	0.03436347478
RP11-351I24.1	2.562296038	0.03437842433
RP11-79H23.3	4.299504049	0.03483550597
RP11-888D10.3	2.41165358	0.03533741854

RP11-48G14.2	-4.684513252	0.01381711614
RP11-9N12.2	-4.684513252	0.01381711614
RP11-50D16.4	-4.651213362	0.01382833228
RP11-848P1.5	-2.669653108	0.01383247188
RP5-1065J22.8	-1.649678346	0.01386497538
AC000403.4	-2.741311201	0.01391242411
RP11-332H14.2	-1.111480965	0.01399112789
RP5-1136G13.2	-1.599171771	0.01401653329
RP11-350N15.6	-3.047191588	0.01423488913
CTB-105N12.2	-4.983529656	0.0143183238
LINC00648	-4.645172685	0.01450753403
RP11-390E23.6	-1.171112194	0.01455555821
RP11-1134I14.8	-4.652330353	0.01471688999
AP000473.5	-3.704294737	0.01474654732
RP11-231G15.3	-4.811260988	0.0149979938
LMCD1-AS1	-2.605152147	0.01511098396
CTB-36H16.2	-2.200069423	0.01540239574
RP3-333H23.9	-1.682743633	0.01550077249
MEG9	-5.272678764	0.01572805843
RP11-758H9.2	-1.607081423	0.0160937755
AE000661.37	-5.261994157	0.01613549692
LOH12CR2	-1.67691758	0.01636598428
AC010883.5	-2.094717492	0.01690504352
RP11-380B4.3	-2.439577585	0.01705551462
RP11-81H14.1	-4.806787285	0.01719813169
CTC-360G5.9	-2.368500479	0.01720457727
RP11-796E2.4	-2.760083635	0.01723144724
LINC01410	-1.678064504	0.0174400882
RP11-428J1.4	-2.3268122	0.01789964346
CTA-246H3.12	-4.798251726	0.0179619574
PAXBP1-AS1	-1.862069318	0.01803800624
RP11-627G23.1	-4.609350113	0.01814637299
RP11-257P3.3	-4.876959069	0.01823445458
LINC01085	-3.70779577	0.01823959845
AC009166.7	-5.201731791	0.01837383149
LINC00294	-1.095938356	0.01855474263
CTC-366B18.4	-2.093559034	0.01865539229
RP11-260M2.1	-1.318083794	0.01886728818
RP11-749I16.3	-2.217730829	0.01887511345
CTB-152G17.6	-1.643327484	0.01890152822
RP11-448A19.1	-1.219705379	0.01918016334
AC108488.4	-1.73751869	0.01934800273
RP11-351J23.1	-4.495981675	0.0196358903
RP11-16P20.4	-4.810891092	0.01992404833
RP11-1109F11.5	-2.273051173	0.01998900498
RP11-403A3.1	-4.659595147	0.02001479214
LINC01549	-4.572397451	0.02015732158

RP11-366F6.2	4.34276227	0.03550164201
RP11-1228E12.1	1.830938095	0.03593843095
RP11-81A1.6	1.075472397	0.03620001818
RP13-895J2.3	3.993426306	0.03644455666
RP11-73K9.2	1.107972094	0.03647433494
RP11-43F13.4	3.285308497	0.03697037675
RP11-1020A11.2	1.888200393	0.03727368249
RP11-406H4.1	4.286387948	0.03756156167
CTD-2341M24.1	1.475568801	0.03769661099
RP11-277B15.3	2.004144709	0.03781760213
AC092168.2	3.869228131	0.03823486444
SCOC-AS1	1.415130662	0.0385734574
LINP1	3.012624307	0.03934463347
AP000442.1	1.819045455	0.03934463347
ERICH6-AS1	1.601404055	0.03938598822
RP11-44N22.3	2.451911166	0.03951878733
CTC-529P8.1	2.519627515	0.03979660975
RP11-810P12.7	2.392211464	0.03989634644
RP13-349O20.2	1.41595205	0.04007012692
RP11-54O7.18	4.304917175	0.0404314925
RP11-380G5.2	3.144009804	0.04066267001
RP11-438D8.2	4.218401032	0.04088791089
RP11-66N24.3	1.113943438	0.04105688984
LINC00896	4.25843734	0.04124060309
RP11-150C16.1	3.321742726	0.04124693278
RP4-607I7.1	2.803939029	0.04132568467
RP11-108M9.3	4.217755779	0.04135778809
RP11-330O11.3	1.84891761	0.04168350781
AC012456.4	3.658464197	0.04192439519
RP11-350J20.5	3.028697357	0.04207796074
RP11-532M24.1	2.8707048	0.04210636138
RP11-525A16.4	3.533212305	0.04257278328
AC091878.1	4.248277103	0.04292114517
RP3-523K23.2	4.203013618	0.04340980105
LINC01376	1.365574836	0.04341651479
CTD-2616J11.16	3.779180972	0.04453057161
RP11-666A20.4	4.199150077	0.04500514622
SSSCA1-AS1	1.198465235	0.04549432814
LINC00466	2.60243764	0.04580605729
ZEB1-AS1	1.400077752	0.0460241598
RP11-799D4.4	2.484736958	0.04605572258
LINC01232	1.268280286	0.04613359129
RP11-763B22.4	4.23049027	0.04623083657
RP11-255E6.6	4.187792162	0.04629119587
CTD-2616J11.2	2.356376529	0.0466112716
RP11-360O19.4	4.227441386	0.04688807227
RP11-429J17.7	3.84532819	0.04698622317

LINC01503	-3.739534837	0.02029372027
AC074391.1	-3.969413731	0.02052388547
RP1-86C11.7	-2.953936931	0.02056099504
RP11-263K19.4	-2.442177897	0.02069675234
LINC00856	-3.714459878	0.02076114267
CH17-262A2.1	-2.550560831	0.02078054112
RP11-387M24.5	-4.518544328	0.02134639103
AC006116.24	-4.602989578	0.02136451585
RP11-1055B8.4	-1.750344036	0.02152849722
ZIM2-AS1	-5.728487225	0.02157254241
RP11-342K6.4	-4.385682779	0.0221367623
RP11-265E18.1	-3.28672003	0.02214553784
AC006026.13	-4.479051824	0.02214956939
RP11-1263C18.1	-5.088165351	0.0222210514
AC147651.4	-2.346840133	0.02236941137
RP11-632L2.2	-2.839659583	0.0224747888
KIF9-AS1	-2.115428858	0.02255149604
CTA-363E19.2	-2.28344642	0.02266258966
LINC01579	-5.63619742	0.02270412626
CTC-471J1.2	-2.610533659	0.02304810569
AC013460.1	-3.941300567	0.02315678997
RP11-506K6.4	-5.165760808	0.02351283909
RP11-415J8.7	-4.194523533	0.0236786889
SMC5-AS1	-1.897243206	0.02369980112
FAM182A	-3.610013261	0.02387122837
RP11-266K4.9	-4.240484463	0.02410366745
SEMA6A-AS2	-3.304025762	0.02410976757
RP11-690D19.3	-1.169963848	0.02426667977
LINC01176	-2.557288743	0.02431191476
AC005592.3	-2.891919018	0.02450292679
MAFTRR	-4.19633104	0.02455506531
RP11-402D21.2	-2.199728472	0.02464133448
RP11-161M6.6	-2.786670968	0.02470335821
AC083867.4	-4.653553283	0.02528750154
LA16c-313D11.12	-2.757496698	0.0254956113
CTC-487M23.6	-1.69272911	0.02564487108
RP11-767N6.7	-1.718742998	0.02577956814
RP11-465C12.1	-4.999887627	0.02589483178
AC009473.1	-2.9445404	0.02598977373
MEOX2-AS1	-5.001717284	0.02608338642
LINC01351	-2.878391493	0.0262392154
SNHG11	-1.126572362	0.02635014089
COLCA1	-4.48488505	0.02648659538
BBOX1-AS1	-4.343046929	0.026520623
AC008088.4	-4.415163761	0.02682158665
RP11-386G11.8	-4.980436546	0.02682216905
RP11-103J8.1	-4.62662294	0.02702386982

RP11-498P14.5	2.081135395	0.04765588714
AC007879.3	2.990029833	0.04801605006
RP11-473M10.3	4.224174443	0.04813156201
RP11-321L2.1	3.216843551	0.04848793487
RP1-179N16.6	1.310811807	0.04867548494
CTD-2334D19.1	4.331422054	0.04916671913
RP11-89F3.2	3.75747999	0.04956146826
RP11-300E4.2	4.126787633	0.04989447671

RP11-173P15.9	-3.052212839	0.02761667489
RGMB-AS1	-2.82462874	0.02763328829
UG0898H09	-5.411559902	0.02770926109
LINC01679	-4.497326429	0.02812941459
AP000662.4	-2.885341154	0.02812941459
LINC01126	-1.767347444	0.02835847673
RP11-615I2.2	-4.365796099	0.02857671418
RP11-762I7.4	-2.278249563	0.02883168565
RP11-73M18.7	-1.140167638	0.02883168565
RP11-4B14.3	-4.355487064	0.02886202535
RP11-631N16.2	-1.085770273	0.02886266936
DICER1-AS1	-1.364817281	0.0292484033
CTD-2081C10.7	-3.434787641	0.02930132666
RP11-727F15.11	-4.118654448	0.02948834621
RAPGEF4-AS1	-4.937860174	0.02950265689
LACTB2-AS1	-4.286377731	0.02964744196
AC007255.8	-3.502363755	0.03069365847
CTB-25B13.5	-2.348980938	0.03093578474
RP1-79C4.4	-4.442624133	0.03125269414
RP11-977G19.5	-1.031585015	0.03128250069
RP11-594N15.3	-2.363080029	0.03129911619
RP11-329B9.5	-3.129805678	0.03139442107
RP11-101E13.5	-1.556053153	0.03164908581
RP11-536C5.2	-4.323396763	0.03165134489
RP11-4L24.4	-1.128201374	0.03186785628
RP11-426D19.1	-3.920727587	0.03202164348
RP11-156P1.3	-1.092878188	0.03206607327
RP5-981O7.2	-2.847910582	0.03218753862
AP000704.5	-2.044727445	0.03219449816
LINC01080	-4.462752051	0.03219449816
AP001626.2	-2.759241704	0.03227373944
CTC-463A16.1	-2.200716717	0.03229865297
AC025171.1	-1.521519352	0.03242846035
RP11-445N18.5	-4.872461677	0.03250247295
RP11-723J4.3	-2.86558885	0.03250247295
RP11-127B20.2	-3.268284733	0.03252835712
CTD-2017F17.2	-1.890797922	0.03272478463
RP11-372K14.2	-2.734014231	0.03299355355
CTD-2349P21.9	-1.653768305	0.03302221907
RP11-283G6.4	-3.20224338	0.03358664258
KB-318B8.7	-1.944256244	0.03362102375
FAM13A-AS1	-1.68370732	0.0336246098
ZNF663P	-4.320542161	0.03395267252
RP11-15I11.3	-3.567386603	0.03422589494
RP11-576I22.2	-2.829342613	0.03429533444
CTD-2339F6.1	-5.01173296	0.03435862537
HIF1A-AS1	-2.767681274	0.03452866598

RP11-358L22.3	-1.863561675	0.03475279743
CTD-2267D19.3	-2.037278158	0.03477260771
MIR181A1HG	-3.345085444	0.03482068087
PINK1-AS	-1.057937569	0.0349061461
RP11-120K24.3	-4.292761346	0.03502009568
RP11-309L24.10	-4.367741678	0.03528179292
RP11-454E5.4	-1.72255704	0.03528179292
RP11-649E7.5	-2.509432048	0.0354452056
RP11-138I1.3	-4.407859811	0.0354452056
RP11-455O6.8	-3.389333778	0.0354606177
RP11-155D18.13	-2.850027708	0.03549900072
RP11-966I7.4	-4.833089133	0.03566957238
RP11-624M8.1	-3.838254014	0.03567976326
RP11-21L23.2	-1.641763783	0.03587147586
RP11-798M19.3	-1.580474485	0.03616799772
LINC00342	-1.17401864	0.0362703262
RP11-109E12.1	-4.346789663	0.03650719876
DGCR11	-1.522625147	0.03660919963
RP11-146D12.2	-2.33503296	0.03686205144
LINC00598	-3.25721974	0.03740130574
CTC-487M23.5	-1.617480029	0.03788049924
RP11-503P10.1	-2.93979721	0.03808119036
RP11-46C24.7	-1.453625372	0.03839689006
CTB-179K24.3	-3.105323631	0.03897929109
RP11-274H2.5	-1.774968806	0.03902359236
RP11-407G23.5	-1.162246303	0.0391767662
RP11-180M15.6	-3.830464148	0.03990378392
CA3-AS1	-3.408899148	0.04007012692
SATB1-AS1	-4.265492318	0.04023208631
RP11-362K14.6	-1.798464724	0.04032130625
RP11-76I23.7	-2.909154172	0.04051247153
CTD-2619J13.3	-3.870494129	0.04066267001
FBXL19-AS1	-1.214368531	0.04067420231
SEPT7-AS1	-1.273688927	0.04070666908
C22orf24	-3.362164712	0.04078140421
CTC-325H20.8	-4.379620268	0.04129163677
RP11-431M7.3	-4.378161653	0.04183496363
RP11-84G21.1	-1.530244352	0.04196204402
LINC01206	-4.551707684	0.04221773341
LINC01852	-1.687920867	0.0426691845
WEE2-AS1	-1.371126783	0.04272802559
AP000432.1	-1.255746825	0.0431367002
NFIA-AS2	-4.690934248	0.04341261462
RP11-685N10.1	-1.873748264	0.04343756073
RP11-226L15.5	-1.156051126	0.04355089618
RP5-884C9.2	-4.67028136	0.04372566592
RP11-770J1.3	-1.978003662	0.04376810314

RP11-500G22.4	-4.213399087	0.04400849013
LINC00519	-4.174203244	0.04414826492
RP11-712B9.2	-1.681867447	0.04438495977
POC1B-AS1	-1.487207304	0.04446223563
AGPAT4-IT1	-1.387935733	0.04454139494
RP11-359B12.2	-1.014373795	0.04585671536
U47924.31	-2.014400185	0.04592004515
CTC-344H19.4	-3.534171212	0.046018976
AC007566.10	-1.014212726	0.04603914804
RNF219-AS1	-4.287346862	0.04607192115
LINC02082	-2.0766378	0.04636223328
NCK1-AS1	-1.304319419	0.04651237249
CCND2-AS1	-3.947812765	0.04700024178
RP11-445N20.2	-2.679775	0.04734839073
RP11-73M18.6	-1.485525302	0.04735323094
LINC01686	-4.286835057	0.04863535485
LINC01252	-2.073475688	0.04871208694
RP11-390P24.1	-1.435312492	0.04924716103
RP5-994D16.3	-2.841919319	0.04942585422
SPRY4-IT1	-1.766620564	0.04966854642

Supplementary Table 20: Highly expressed lncRNAs in mesenchymal (MES) and proneural (PN) GSCs (log2FC > 3 and FDR < 0.05), with expression differences between MES and PN GBM (|log2FC|>0).

Highly expressed lncRNAs in MES			
lncRNA	log2FC.GSCs	FDR.GSCs	log2FC.GBM
ADIRF-AS1	8.1469	3.93E-36	0.3091
MIR222HG	4.0647	3.46E-23	1.4048
RP11-66B24.7	8.9782	8.13E-23	0.1959
RP11-420A23.1	3.6846	4.13E-19	0.0518
LINC00707	8.8163	5.03E-18	0.2462
AC003092.1	10.5004	3.23E-17	0.9782
LINC00857	8.3517	5.01E-17	1.1929
RP5-884M6.1	9.7256	8.11E-17	0.5070
RP11-366H4.1	10.0323	3.19E-16	1.7816
RP11-108M9.4	6.8084	8.17E-16	0.6520
UBR5-AS1	3.5689	1.20E-15	0.2129
LINC00944	9.8211	2.02E-15	0.3192
RP11-148B18.4	9.2308	2.71E-15	0.9690
MSC-AS1	7.9784	5.06E-15	0.2305
LINC01605	7.7660	1.00E-14	2.1591
CASC9	9.5504	1.20E-14	0.6227
RP11-66B24.2	9.6835	1.23E-14	0.4456
CTB-140J7.2	9.0559	1.87E-14	0.7548
MIR137HG	9.4635	2.91E-14	0.0222
RP4-647C14.2	9.4408	3.12E-14	0.0557
LINC00910	4.0521	4.78E-14	0.1676
RP11-221N13.3	5.2213	1.97E-12	2.3166
AP001065.15	8.3707	2.71E-12	0.5229
CASC8	7.3916	2.71E-12	1.3339
KC6	8.7319	4.18E-12	1.1810
RP11-879F14.2	7.1677	5.85E-12	0.5583
LINC01615	8.2440	1.01E-11	1.6496
CTD-2054N24.2	8.4811	2.59E-11	0.8493
RP11-1149O23.3	8.0683	3.64E-11	1.0466
LINC00704	8.7894	4.84E-11	0.5267
FENDRR	8.8712	8.73E-11	0.3579
RP11-66B24.4	8.4112	1.78E-10	0.4011
RP4-756H11.3	3.0534	4.84E-10	0.1755
STXBP5-AS1	4.8328	6.31E-10	0.1215
TSPEAR-AS2	7.8613	4.06E-09	0.3678
LINC00941	4.9017	5.11E-09	2.0703
TSPEAR-AS1	7.7208	5.64E-09	0.7269
RP11-359K18.4	3.5296	5.78E-09	0.3884
RP1-102E24.8	7.9476	6.95E-09	0.6986
LBX2-AS1	4.2131	8.53E-09	1.0162
EML2-AS1	4.3363	1.09E-08	0.3950
RP11-809O17.1	4.2873	1.19E-08	0.5444
RP11-328K4.1	7.6249	1.43E-08	1.4252
CTD-2207P18.2	7.5549	6.98E-08	1.2037

Highly expressed lncRNAs in PN			
lncRNA	log2FC.GSCs	GSCs.FDR	log2FC.GBM
SOX2-OT	9.1016	5.55E-43	0.3283
LINC00511	4.7276	8.94E-35	0.6536
LINC00665	6.5084	2.47E-32	0.3358
LINC00461	4.0718	3.20E-25	0.3884
AC074289.1	6.6117	5.13E-20	0.2217
AC018647.3	10.6295	1.29E-16	0.4354
HAGLR	10.3761	1.88E-16	1.2307
SOX21-AS1	10.6784	4.24E-16	0.3024
RP11-210M15.2	7.9611	2.74E-15	0.0025
MEG3	9.8841	3.89E-15	0.4281
RP11-161M6.2	9.8620	4.74E-15	2.0987
ZNF528-AS1	10.3361	2.16E-14	0.6251
RP11-275H4.1	9.0764	3.29E-14	1.5444
LINC01896	8.7173	5.77E-13	1.0336
CASC15	9.3361	1.20E-12	0.9993
RP3-525N10.2	6.6601	1.23E-12	0.9506
RP11-629G13.1	8.3794	1.00E-11	0.2281
AC108142.1	8.9199	1.48E-11	0.3438
LINC01268	8.6508	3.20E-11	1.0059
RP11-436K8.1	9.1534	3.66E-11	0.9481
RP5-882C2.2	3.1466	1.50E-10	0.2545
MIAT	7.6341	1.63E-10	1.3584
AC114730.3	8.5402	3.82E-10	1.4959
RP11-444D3.1	8.2187	4.12E-10	0.5984
CTC-559E9.5	8.3450	6.27E-10	0.2682
CTC-260E6.6	8.0306	1.49E-09	1.0055
FAM212B-AS1	6.8176	3.47E-09	1.1393
LINC02199	7.5342	5.40E-09	1.4736
LINC01550	8.0856	7.55E-09	0.1503
ZNF582-AS1	7.9035	9.86E-09	0.0312
LINC01535	7.5876	1.12E-08	0.4357
LINC00403	7.6991	1.72E-08	1.1060
CTD-2291D10.4	7.6979	2.78E-08	0.9282
RP11-448P19.1	8.4664	2.88E-08	1.0048
RP11-472N13.3	7.8359	4.56E-08	0.4240
LINC01105	8.2108	4.78E-08	1.7989
PTPRD-AS1	7.5183	5.39E-08	0.6054
RAMP2-AS1	8.6078	8.64E-08	0.8693
LINC01415	8.3713	1.48E-07	0.2638
HAND2-AS1	7.8711	1.90E-07	2.2214
CTC-543D15.8	7.5218	2.35E-07	0.3541
ZNF790-AS1	7.2818	2.38E-07	0.2634
CTD-2017D11.1	3.9601	2.97E-07	0.3026
LINC00391	7.1464	5.71E-07	0.0841

UPK1A-AS1	7.0642	6.99E-08	0.3433	AC010729.1	7.2602	5.96E-07	1.2802
CTD-2587H24.5	7.5136	7.00E-08	0.8124	RP11-617F23.1	3.1248	6.90E-07	0.1808
CTD-2066L21.3	6.2857	7.90E-08	0.4591	RP11-966I7.2	7.1963	1.09E-06	0.2390
LINC00623	3.5339	9.56E-08	0.4518	RP11-25K19.1	5.4054	1.31E-06	0.5767
RP11-89C3.4	7.9437	1.01E-07	0.4637	RP11-4O1.2	4.6150	1.45E-06	0.0759
LINC01162	7.2061	1.50E-07	0.1691	GS1-24F4.2	6.7798	1.45E-06	0.9853
LINC00702	6.8199	1.58E-07	0.6697	AP002954.3	3.3516	1.87E-06	0.7650
CTD-2311M21.3	7.2206	1.59E-07	1.1687	H19	5.5332	2.07E-06	1.7596
CTD-2303H24.2	4.7042	1.86E-07	0.3725	RP11-126K1.6	3.7305	3.98E-06	0.5977
EGFR-AS1	7.4675	2.19E-07	0.5094	AC078842.3	6.3127	5.01E-06	0.0614
OSMR-AS1	4.8488	7.28E-07	0.7150	AC064875.2	8.2687	5.72E-06	0.3105
AC156455.1	4.6873	1.04E-06	0.0966	RP11-742D12.2	6.6005	6.20E-06	0.8636
LINC00659	6.3653	1.52E-06	0.3604	LINCR-0001	3.5838	7.15E-06	1.0618
RP13-463N16.6	7.1209	1.64E-06	2.5390	AC073343.13	3.8178	7.97E-06	0.6155
PLBD1-AS1	6.9117	1.87E-06	0.8031	LINC01224	6.9425	8.32E-06	1.7384
LINC01036	6.8181	2.35E-06	0.0464	RP11-834C11.4	6.1942	9.19E-06	0.2289
LINC01273	5.6801	2.37E-06	0.6387	RP11-351J23.2	7.0082	1.41E-05	0.3510
RP11-402J6.1	6.7575	2.72E-06	0.0069	PROX1-AS1	6.4834	1.45E-05	0.0064
RP11-519M16.1	6.6977	2.85E-06	0.1023	U52111.14	5.2091	1.56E-05	0.2236
LINC00460	3.7294	3.14E-06	2.3430	RP11-268J15.5	3.1991	1.80E-05	0.0660
RP11-49I11.1	3.6175	3.44E-06	1.2360	C8orf31	6.7142	1.88E-05	0.2861
RP11-326A19.4	4.3692	4.03E-06	0.2700	RP11-174J11.1	6.3288	2.05E-05	0.6565
RP11-403I13.5	6.6077	5.12E-06	0.7373	RP13-942N8.1	3.2657	2.40E-05	0.5635
RP11-802E16.3	3.8291	5.16E-06	1.3231	CH17-408M7.1	6.2584	2.57E-05	0.2412
RP1-28O10.1	6.7406	5.65E-06	0.7849	RP11-120J1.1	3.9745	3.13E-05	0.8404
LINC01037	6.2703	6.20E-06	0.2627	ZNF793-AS1	6.7548	3.80E-05	0.9097
LINC01703	3.0634	7.75E-06	0.3237	CTD-2049O4.1	7.3214	4.22E-05	0.8453
LINC01186	6.3373	7.82E-06	0.7758	AC004540.4	6.2826	4.39E-05	0.5345
DNAH17-AS1	6.6714	8.96E-06	0.0229	SLC26A4-AS1	5.4537	6.07E-05	0.4367
GAPLINC	6.4889	9.04E-06	0.9181	RP11-582J16.4	4.9402	6.60E-05	0.1800
LL22NC03-86G7.1	4.7270	9.25E-06	0.0689	RP11-625L16.1	6.2400	6.85E-05	1.8692
AC073130.3	6.4422	9.95E-06	0.4121	LINC00326	6.3146	9.07E-05	0.1436
RP11-54A9.1	7.4821	1.12E-05	0.8459	LINC00664	7.0364	9.29E-05	0.8471
RP5-1139I1.1	6.9312	1.15E-05	0.8711	AC108025.2	6.0174	0.0001	1.2788
RP11-162D16.2	6.4031	1.33E-05	0.1711	CTD-2380F24.1	5.9619	0.0001	1.4868
RP11-60A8.1	7.0596	1.42E-05	0.5194	HAS2-AS1	4.0856	0.0001	0.1556
RP11-134G8.5	3.1272	1.71E-05	0.4168	LINC00844	6.5062	0.0001	0.8353
RP13-977J11.2	4.1660	2.22E-05	0.1019	HOXD-AS2	5.9413	0.0002	0.7560
SENCR	6.0854	2.56E-05	0.5495	LINC00977	6.2199	0.0002	0.1053
HIF1A-AS2	6.4048	3.20E-05	0.9386	RP11-231C18.1	6.0200	0.0002	1.6517
RP11-527N22.2	5.3391	3.42E-05	1.8606	AC073283.4	5.9773	0.0002	1.1089
CTC-378H22.1	6.5346	3.76E-05	0.7747	FOXG1-AS1	6.0182	0.0002	0.4371
RP11-686D22.8	6.1985	3.99E-05	0.6303	TNKS2-AS1	3.7280	0.0003	0.3557
AC012485.2	5.9237	4.79E-05	0.1723	RP11-698N11.2	5.9051	0.0003	0.7841
AC007879.5	3.0746	4.97E-05	0.6479	LINC01748	5.1733	0.0003	0.7891
AC073130.1	6.1266	7.07E-05	1.0471	AC007204.2	6.2085	0.0003	1.1527
RP11-280G9.1	6.1710	7.41E-05	1.0562	RP11-838N2.5	6.0375	0.0003	0.4641
MYOSLID	6.2516	8.30E-05	2.1396	LINC01630	5.6072	0.0005	1.7337
RP11-818F20.5	5.8374	8.44E-05	1.0722	AC017104.6	3.3167	0.0006	0.4561

RP11-336A10.5	6.0364	9.75E-05	0.5221
RP11-61A14.1	5.6362	0.0001	1.1220
LRRC2-AS1	6.0330	0.0001	1.1117
RP11-745L13.2	5.9655	0.0002	1.3849
RP11-43A14.1	4.3878	0.0002	0.0340
CTD-2128A3.2	5.9168	0.0002	2.2835
RP11-115J23.1	3.5955	0.0002	1.9513
RP3-414A15.2	5.8330	0.0002	0.5648
RP11-553A21.3	5.6687	0.0002	0.9001
HRAT17	6.1954	0.0002	1.6193
AC007879.2	4.3314	0.0002	0.7686
AC006369.2	5.8652	0.0004	1.0435
RP11-289F5.1	4.9677	0.0004	0.7608
CTC-378H22.2	5.8633	0.0004	0.7168
RP11-195F19.9	4.4690	0.0004	0.3795
AC009502.4	5.7300	0.0004	0.8763
A2M-AS1	4.1688	0.0005	0.5534
LINC01611	5.6653	0.0006	1.3759
CYYR1-AS1	5.7963	0.0006	0.6846
CFAP58-AS1	5.2304	0.0006	0.1410
XXyac-YX65C7_A.	3.1524	0.0006	0.4766
AP001596.6	3.7945	0.0006	0.6784
CTD-2020K17.1	3.5746	0.0007	0.8740
LINC01385	5.2138	0.0008	1.0731
LINC00887	4.7682	0.0008	0.7505
SERTAD4-AS1	4.4090	0.0008	1.0354
RP11-1094H24.4	3.4360	0.0010	0.8241
RP11-399K21.14	3.5805	0.0010	0.4319
RP5-1071N3.1	3.2324	0.0011	0.2049
RP11-379B18.5	3.0185	0.0011	0.2539
RP11-320G24.1	5.4414	0.0011	0.2748
MTUS2-AS1	4.3280	0.0013	0.6426
LINC01137	3.2354	0.0014	0.5691
AC023590.1	5.4404	0.0014	0.5825
RP11-26J3.1	3.1958	0.0015	0.1676
RP11-108K3.1	5.3599	0.0016	0.2869
RP11-273G15.2	4.3554	0.0020	0.5344
SERPINB9P1	4.3809	0.0020	1.1064
CTD-2589M5.5	5.0084	0.0020	0.9388
RP11-276H7.2	4.8640	0.0022	1.0927
RP11-445O3.2	5.2975	0.0022	0.6116
AC137932.6	4.9362	0.0023	0.0407
RP11-686D22.5	5.2272	0.0026	0.7512
TARID	5.1318	0.0028	0.6899
AP000695.4	3.4169	0.0032	0.5572
AC018816.3	5.1713	0.0032	0.8256
RP11-565A3.2	5.2522	0.0032	0.2236
LINC01776	3.9838	0.0037	0.5697
RP11-114H21.2	5.2812	0.0037	0.0537
RP11-507K2.3	5.7402	0.0006	0.5577
HCG17	4.4801	0.0007	0.3996
LIFR-AS1	3.0592	0.0007	0.3076
RP11-307B6.3	5.2974	0.0008	0.6533
MIMT1	6.0754	0.0008	1.6634
MESTIT1	6.1561	0.0008	0.1114
LINC01102	5.9799	0.0009	1.0991
LINC01158	3.5994	0.0009	0.3068
AP000289.6	6.1491	0.0010	1.6762
CTB-118P15.2	5.4817	0.0010	1.0792
CTC-467M3.1	5.6468	0.0010	0.5689
AC053503.11	5.3923	0.0013	1.8829
LINC00945	5.5410	0.0013	0.7030
RP4-657D16.3	3.7175	0.0014	0.3317
RP11-430H10.1	5.5299	0.0017	0.3254
AP006222.2	5.4094	0.0017	0.1647
RP11-38M8.1	5.9893	0.0018	0.2782
MIR181A2HG	3.0687	0.0020	0.8415
CTC-255N20.1	5.9100	0.0024	0.4790
AP000708.1	5.3579	0.0028	0.1114
RP11-247A12.2	4.1691	0.0030	0.3506
RP11-620J15.1	5.3183	0.0032	2.0189
RP5-1024C24.1	5.9992	0.0036	0.8969
LINC00506	5.2956	0.0039	0.1917
RP11-281O15.4	5.0481	0.0043	0.6800
LINC00877	5.1626	0.0044	0.2446
LINC00606	5.9152	0.0045	0.4150
DACT3-AS1	4.6344	0.0046	0.8437
RP11-758P17.2	3.7142	0.0052	0.1701
RP11-216B9.9	3.2349	0.0055	0.5805
SPATA42	5.0719	0.0056	0.5594
RP11-620J15.4	4.9438	0.0058	1.7478
LINC01977	5.3226	0.0061	1.0575
ZEB2-AS1	4.0995	0.0063	0.1863
LINC01816	4.9271	0.0075	0.8473
RP11-277P12.9	4.9712	0.0081	2.2566
RP11-472N13.2	4.4647	0.0085	0.7597
GPR158-AS1	4.8485	0.0086	0.0642
AC003991.3	4.8317	0.0088	0.4532
RP11-318C24.2	4.8280	0.0091	0.5283
AC005220.3	5.5520	0.0094	0.1061
RP11-259K15.2	5.0183	0.0101	1.1989
ZNF571-AS1	4.9748	0.0117	0.8521
HCG22	5.3763	0.0129	0.2231
LINC01505	3.3301	0.0131	0.7594
RP11-50D16.4	4.6512	0.0138	0.6064
LINC00648	4.6452	0.0145	1.5474
MEG9	5.2727	0.0157	0.0300
RP11-627G23.1	4.6094	0.0181	0.2903

AC005264.2	5.2786	0.0038	0.9806
LINC01111	5.5117	0.0045	1.6648
RP1-137D17.1	5.0387	0.0046	0.2200
RP11-755F10.1	4.2900	0.0049	0.0104
RP11-676J12.7	5.8714	0.0052	0.4677
AC131056.3	4.9942	0.0063	0.3870
LINC01939	6.2742	0.0071	0.4192
AC020571.3	4.5507	0.0072	1.2336
RP3-467K16.2	4.4681	0.0075	0.5602
AC092614.2	4.9414	0.0075	0.4894
RP11-460B17.2	4.4683	0.0097	2.1640
CTD-2540B15.9	4.0184	0.0099	0.7740
SFTA1P	3.3467	0.0103	0.7043
RP11-885N19.6	4.8961	0.0123	1.6116
LINC01239	3.6509	0.0131	1.4995
RP11-1398P2.1	4.2298	0.0140	1.1099
AP000442.4	3.4047	0.0141	0.2809
GACAT2	3.2024	0.0144	0.1125
AC145343.2	3.7509	0.0183	0.5326
AC002076.10	3.8424	0.0184	0.8643
RP11-650K20.3	3.6152	0.0192	0.9205
LINC02057	4.1215	0.0193	0.3934
AC106786.1	4.1912	0.0198	0.4619
CTD-2337A12.1	4.5371	0.0203	0.5804
ITGB2-AS1	4.5937	0.0217	0.6071
RP11-14N7.2	4.4781	0.0223	0.2437
RP11-297A16.2	4.1983	0.0225	0.3901
GATA6-AS1	3.9957	0.0302	1.2654
RP3-467K16.7	4.4115	0.0304	1.5077
FOXC2-AS1	4.5399	0.0319	1.0709
WFDC21P	4.4383	0.0330	1.3182
RP11-95P2.3	4.3111	0.0331	1.0244
RP11-366F6.2	4.3428	0.0355	0.4064
AC092168.2	3.8692	0.0382	0.6896
RP11-54O7.18	4.3049	0.0404	0.2235
RP11-438D8.2	4.2184	0.0409	0.2102
RP11-150C16.1	3.3217	0.0412	0.4968
RP11-350J20.5	3.0287	0.0421	0.1488
RP11-525A16.4	3.5332	0.0426	1.1281
RP11-666A20.4	4.1992	0.0450	0.1344
RP11-360O19.4	4.2274	0.0469	0.2091
CTD-2334D19.1	4.3314	0.0492	0.0261
RP11-89F3.2	3.7575	0.0496	0.1548

RP11-351J23.1	4.4960	0.0196	0.5468
LINC01549	4.5724	0.0202	0.4761
ZIM2-AS1	5.7285	0.0216	1.0882
AC006026.13	4.4791	0.0221	0.2633
AC013460.1	3.9413	0.0232	1.3381
FAM182A	3.6100	0.0239	1.0859
RP11-266K4.9	4.2405	0.0241	0.4630
SEMA6A-AS2	3.3040	0.0241	0.1935
AC083867.4	4.6536	0.0253	1.6979
RP11-103J8.1	4.6266	0.0270	0.0646
LINC01679	4.4973	0.0281	0.9819
RP11-615I2.2	4.3658	0.0286	1.2044
RAPGEF4-AS1	4.9379	0.0295	0.4769
AC007255.8	3.5024	0.0307	0.7794
RP11-536C5.2	4.3234	0.0317	0.8584
ZNF663P	4.3205	0.0340	0.6102
RP11-15I11.3	3.5674	0.0342	0.8645
CTD-2339F6.1	5.0117	0.0344	1.9449
MIR181A1HG	3.3451	0.0348	1.0008
RP11-138I1.3	4.4079	0.0354	0.2193
RP11-624M8.1	3.8383	0.0357	0.1144
RP11-109E12.1	4.3468	0.0365	0.5169
LINC00598	3.2572	0.0374	0.0892
SATB1-AS1	4.2655	0.0402	1.0831
CTD-2619J13.3	3.8705	0.0407	0.1729
C22orf24	3.3622	0.0408	0.4299
LINC00519	4.1742	0.0441	0.0473
CTC-344H19.4	3.5342	0.0460	0.2764
RNF219-AS1	4.2873	0.0461	1.2373
CCND2-AS1	3.9478	0.0470	0.4098

Supplementary Table 21: TCGA tumor types considered in the lncRNAs survival analyses.

Tumor type	TCGA's abbreviation	Samples
Breast invasive carcinoma	BRCA	1,037
Bladder urothelial carcinoma	BLCA	395
Cervical squamous cell carcinoma and endocervical adenocarcinoma	CESC	287
Colon adenocarcinoma	COAD	350
Glioblastoma multiforme	GBM	153
Head and neck squamous cell carcinoma	HNSC	495
Kidney renal clear cell carcinoma	KIRC	519
Kidney renal papillary cell carcinoma	KIRP	269
Lower grade glioma	LGG	655
Liver hepatocellular carcinoma	LIHC	338
Lung adenocarcinoma	LUAD	476
Lung squamous cell carcinoma	LUSC	475
Ovarian serous cystadenocarcinoma	OV	372
Prostate adenocarcinoma	PRAD	496
Rectum adenocarcinoma	READ	125
Skin cutaneous melanoma	SKCM	85
Stomach adenocarcinoma	STAD	338
Thyroid carcinoma	THCA	498
Uterine corpus endometrioid carcinoma	UCEC	539

Supplementary Table 22: Differentially expressed lncRNAs in GSCs significantly associated with prognosis in GBM.

PART A. Univariate analysis of lncRNAs

		Cox regression				
**lncRNA**	**Mesenchymal-GSCs**	**beta.coef**	**Wald.statistic**	**Cox.pvalue**	**log-rank.pvalue**	**other.tumors**
**CTD-2589M5.5**	up	2.7389	3.7104	0.0002	0.0001	KIRC
**SOX21-AS1**	down	-0.1676	-3.2633	0.0011	0.0047	CESC
**MYOSLID**	up	0.3918	3.1528	0.0016	0.0110	HNSC,KIRC,KIRP,LGG,UCEC
**CRNDE**	up	0.0863	3.5171	0.0004	0.0120	LGG,LUAD,THCA
**AC005264.2**	up	4.9631	3.5559	0.0004	0.0158	HNSC
**RP11-575F12.1**	up	3.9087	2.4723	0.0134	0.0334	----

high expression associated with poor prognosis

high expression associated with better prognosis

PART B. Multivariate analysis of lncRNAs and clinical parameters

	CTD-2589M5.5			SOX21-AS1			MYOSLID		
**variable**	**beta.coef**	**Wald.statistic**	**Cox.pvalue**	**beta.coef**	**Wald.statistic**	**Cox.pvalue**	**beta.coef**	**Wald.statistic**	**Cox.pvalue**
**lncRNA**	2.9512	3.1500	**0.0016**	-0.1790	-2.6102	**0.0090**	0.8050	2.1389	**0.0324**
**age at diagnosis**	0.5335	1.7187	0.0857	0.5917	1.8940	0.0582	0.6910	2.1127	**0.0346**
**gender**	-0.3129	-1.1116	0.2663	0.1605	0.5079	0.6115	-0.2283	-0.8285	0.4074
**CIMP status**	0.2230	0.2378	0.8120	0.3155	0.3541	0.7233	0.4810	0.5311	0.5954
**IDH1 mutation**	1.4575	1.4885	0.1366	1.5631	1.6686	0.0952	1.4187	1.4822	0.1383
**MGMT methylation**	0.2439	0.8588	0.3904	0.0376	0.1227	0.9023	0.3214	1.1492	0.2505
**chr 19/20 co-gain**	1.2870	2.8095	**0.0050**	1.1573	2.5024	**0.0123**	1.2405	2.7213	**0.0065**
**chr 7 gain/chr 10 loss**	0.0805	0.2733	0.7846	0.1482	0.5271	0.5981	0.2086	0.7326	0.4638

***significant variables (p<0.05)**

	CRNDE			AC005264.2			RP11-575F12.1		
**variable**	**beta.coef**	**Wald.statistic**	**Cox.pvalue**	**beta.coef**	**Wald.statistic**	**Cox.pvalue**	**beta.coef**	**Wald.statistic**	**Cox.pvalue**
**lncRNA**	0.0708	2.2929	**0.0219**	6.2901	4.0482	**0.0001**	5.2020	1.9933	**0.0462**
**age at diagnosis**	0.5759	1.8630	0.0625	0.5009	1.6054	0.1084	0.5246	1.6998	0.0892
**gender**	-0.3174	-1.1087	0.2675	-0.0666	-0.2353	0.8140	-0.3786	-1.3188	0.1872
**CIMP status**	0.7011	0.7955	0.4263	0.4377	0.4857	0.6272	0.5731	0.6738	0.5004
**IDH1 mutation**	1.5042	1.5970	0.1103	1.4554	1.5310	0.1258	1.6372	1.7991	0.0720
**MGMT methylation**	0.4147	1.4743	0.1404	0.4312	1.5554	0.1199	0.5118	1.8300	0.0672
**chr 19/20 co-gain**	1.3610	3.0141	**0.0026**	1.2733	2.7621	**0.0057**	1.1554	2.5138	**0.0119**
**chr 7 gain/chr 10 loss**	0.4099	1.4812	0.1386	0.1894	0.6771	0.4984	0.4450	1.5918	0.1114

***significant variables (p<0.05)**

Supplementary Table 23: Differentially expressed lncRNAs in GSCs significantly associated with prognosis in mesenchymal and proneural GBM.

PART A. Univariate analysis of lncRNAs in mesenchymal GBM

		Cox regression				
**lncRNA**	**Mesenchymal-GSCs**	**beta.coef**	**Wald.statistic**	**Cox.pvalue**	**log-rank.pvalue**	**other.tumors**
**CTB-60B18.12**	up	20.5377	3.0619	0.0022	0.0027	----
**AC005264.2**	up	9.1094	3.3256	0.0009	0.0097	HNSC
**CTD-2589M5.5**	up	2.9758	2.8526	0.0043	0.0477	KIRC

high expression associated with poor prognosis

high expression associated with better prognosis

PART B. Univariate analysis of lncRNAs in proneural GBM

		Cox regression				
**lncRNA**	**Mesenchymal-GSCs**	**beta.coef**	**Wald.statistic**	**Cox.pvalue**	**log-rank.pvalue**	**other.tumors**
**RP4-806M20.4**	up	3.7744	3.5038	0.0005	0.0014	BLCA,KIRC
**RP11-676J12.7**	up	8.0575	2.2958	0.0217	0.0029	STAD
**CRNDE**	up	0.1092	2.7879	0.0053	0.0181	LGG,LUAD,THCA
**CTC-459F4.9**	up	0.6691	2.0621	0.0392	0.0204	KIRC,LUSC
**RP11-1334A24.5**	up	2.2393	2.1045	0.0353	0.043	BRCA,KIRC,KIRP,LGG

PART C. Multivariate analysis of lncRNAs and clinical parameters in mesenchymal GBM

	CTB-60B18.12			AC005264.2			CTD-2589M5.5		
**variable**	**beta.coef**	**Wald.statistic**	**Cox.pvalue**	**beta.coef**	**Wald.statistic**	**Cox.pvalue**	**beta.coef**	**Wald.statistic**	**Cox.pvalue**
**lncRNA**	33.0376	2.8358	**0.0046**	7.8433	2.3209	**0.0203**	2.7948	1.9768	**0.0481**
**age at diagnosis**	2.1069	2.4202	**0.0155**	1.2896	1.7938	0.0728	1.1447	1.5948	0.1108
**gender**	-0.0734	-0.0961	0.9235	-0.1417	-0.1815	0.8560	-0.4173	-0.5389	0.5899
**CIMP status**	----	----	----	----	----	----	----	----	----
**IDH1 mutation**	----	----	----	----	----	----	----	----	----
**MGMT methylation**	-0.2769	-0.3597	0.7191	0.0773	0.0979	0.9221	0.4200	0.5347	0.5929
**chr 19/20 co-gain**	1.4125	1.3232	0.1858	-0.4264	-0.4767	0.6336	-0.3758	-0.4180	0.6759
**chr 7 gain/chr 10 loss**	1.5644	2.5044	**0.0123**	1.1077	1.6839	0.0922	1.0507	1.5672	0.1171

***significant variables (p<0.05)**

PART D. Multivariate analysis of lncRNAs and clinical parameters in proneural GBM

	RP4-806M20.4			RP11-676J12.7			CRNDE		
**variable**	**beta.coef**	**Wald.statistic**	**Cox.pvalue**	**beta.coef**	**Wald.statistic**	**Cox.pvalue**	**beta.coef**	**Wald.statistic**	**Cox.pvalue**
**lncRNA**	5.4902	2.3728	**0.0177**	43.5598	6.7626	**<0.0001**	0.1043	2.7750	**0.0055**
**age at diagnosis**	-0.1168	-0.0705	0.9438	1.4617	1.3044	0.1921	0.0185	0.0170	0.9864
**gender**	1.8457	1.3081	0.1909	5.8983	5.0449	**<0.0001**	2.6368	2.7610	**0.0058**
**CIMP status**	----	----	----	----	----	----	----	----	----
**IDH1 mutation**	23.4572	0.0015	0.9988	24.7285	0.0017	0.9987	19.6754	0.0050	0.9960
**MGMT methylation**	-2.3104	-2.0284	**0.0425**	-3.7578	-4.8466	**<0.0001**	-2.2035	-3.1479	**0.0016**
**chr 19/20 co-gain**	21.5606	0.0008	0.9994	21.3173	0.0009	0.9993	18.7554	0.0027	0.9978
**chr 7 gain/chr 10 loss**	-4.2776	-2.3606	**0.0182**	-5.1058	-4.4856	**<0.0001**	-3.1269	-2.6810	**0.0073**

***significant variables (p<0.05)**

	CTC-459F4.9			RP11-1334A24.5		
**variable**	**beta.coef**	**Wald.statistic**	**Cox.pvalue**	**beta.coef**	**Wald.statistic**	**Cox.pvalue**
**lncRNA**	1.4546	2.0460	**0.0408**	8.4904	2.0374	**0.0416**
**age at diagnosis**	-0.1616	-0.1363	0.8916	0.3081	0.2469	0.8050
**gender**	1.9280	1.7567	0.0790	3.9690	2.5544	**0.0106**
**CIMP status**	----	----	----	----	----	----
**IDH1 mutation**	20.9784	0.0013	0.9990	19.2700	0.0012	0.9990

**MGMT methylation**	-2.0482	-2.0364	**0.0417**	1.0608	0.7475	0.4548
**chr 19/20 co-gain**	20.8821	0.0007	0.9994	18.6720	0.0006	0.9995
**chr 7 gain/chr 10 loss**	-3.4659	-2.7186	**0.0066**	-3.9128	-2.5797	**0.0099**

***significant variables (p<0.05)**

Supplementary Table 24: lncRNA-associated splicing events enriched in mesenchymal (MES) and proneural (PN) GSCs samples.

ASS (alternative 5'or 3'splice site): genomic coordinates of long and short exons (columns g.coord_1 and g.coord_2)
MXE (mutually exclusive exon): genomic coordinates of first and EScond exons (columns g.coord_1 and g.coord_2)
RI (retained introns): genomic coordinates of retained intron (column g.coord_1)
ES (exon skipping): genomic coordinates of skipped exon (column g.coord_1)

MES						
event.type	lncRNA	strand	g.coord_1	g.coord_2	inc.level.difference	FDR
MXE	GAS5	-	chr1:173865509-173865547	chr1:173865856-173866206	0.274	0
MXE	GAS5	-	chr1:173865856-173865894	chr1:173866527-173866796	0.254	0
RI	GAS5	-	chr1:173865228-173865547	NA	0.436	0
RI	GAS5	-	chr1:173864256-173865282	NA	0.15	0
ASS	GAS5	-	chr1:173865470-173865547	chr1:173865509-173865547	0.477	0
MXE	GAS5	-	chr1:173865856-173865894	chr1:173866176-173866206	0.232	0
MXE	GAS5	-	chr1:173865509-173865547	chr1:173866176-173866206	0.233	0
ES	SNHG6	-	chr8:66922613-66922725	NA	0.151	0
RI	ZNF582-AS1	+	chr19:56398276-56399168	NA	0.809	0
ES	USP3-AS1	-	chr15:63593748-63593877	NA	0.925	4.00E-12
ES	PCBP1-AS1	-	chr2:70053730-70053815	NA	0.84	2.34E-11
MXE	GAS5	-	chr1:173865509-173865547	chr1:173865856-173865894	0.154	2.88E-11
MXE	GAS5	-	chr1:173864674-173864704	chr1:173865228-173865282	0.206	5.01E-11
ES	RP4-798P15.3	-	chr1:177984205-177984418	NA	0.86	9.70E-11
ES	RASSF8-AS1	-	chr12:25956226-25956347	NA	0.633	2.67E-10
RI	GAS5	-	chr1:173864483-173864704	NA	0.206	4.03E-10
ES	THUMPD3-AS1	-	chr3:9395505-9395579	NA	0.324	4.80E-10
ES	SNHG17	-	chr20:38422091-38422241	NA	0.386	7.64E-10
MXE	SNHG17	-	chr20:38422091-38422241	chr20:38425934-38426052	0.256	5.21E-09
RI	GAS5	-	chr1:173864256-173864506	NA	0.171	1.09E-08
MXE	RP11-421L21.3	+	chr1:101075394-101075716	chr1:101077362-101077508	0.439	3.74E-08
MXE	GAS5	-	chr1:173865856-173865894	chr1:173866760-173866796	0.229	1.36E-07
MXE	PCBP1-AS1	-	chr2:70053730-70053792	chr2:70055713-70055910	0.505	1.79E-07
ASS	SNHG5	-	chr6:85677794-85678032	chr6:85677953-85678032	0.139	3.85E-07
ES	PCBP1-AS1	-	chr2:70053730-70053792	NA	0.227	4.19E-07
MXE	PCBP1-AS1	-	chr2:70053730-70053792	chr2:70055655-70055910	0.392	5.67E-07
RI	TTC28-AS1	+	chr22:27997410-27998838	NA	0.378	9.64E-07
MXE	PCBP1-AS1	-	chr2:70053730-70053792	chr2:70055713-70055910	0.382	1.18E-06
ES	PCBP1-AS1	-	chr2:70053730-70053792	NA	0.359	2.03E-06
MXE	PCBP1-AS1	-	chr2:70053730-70053792	chr2:70059642-70059680	0.407	4.02E-06
MXE	PCBP1-AS1	-	chr2:70053730-70053792	chr2:70083537-70083687	0.148	5.92E-06
MXE	PCBP1-AS1	-	chr2:70053730-70053792	chr2:70083506-70083687	0.166	6.87E-06
ES	PSMA3-AS1	-	chr14:58288717-58288813	NA	0.432	7.31E-06
ES	GUSBP11	-	chr22:23700725-23700845	NA	0.608	8.54E-06
ES	PCBP1-AS1	-	chr2:70053730-70053792	NA	0.315	1.22E-05
MXE	NUTM2A-AS1	-	chr10:87246114-87246212	chr10:87288441-87288495	0.172	1.51E-05
MXE	GAS5	-	chr1:173865228-173865282	chr1:173865470-173865547	0.121	1.59E-05
ASS	TPT1-AS1	+	chr13:45390713-45390902	chr13:45390757-45390902	0.12	1.61E-05
ES	RP11-452F19.3	+	chr1:222823855-222823945	NA	0.305	2.24E-05
ES	CTD-2341M24.1	+	chr14:86128664-86128773	NA	0.612	5.70E-05
MXE	GAS5	-	chr1:173865509-173865547	chr1:173866527-173866567	0.214	6.74E-05
MXE	LINC01184	-	chr5:127956803-127956921	chr5:128061143-128061244	0.313	8.49E-05
ASS	TPT1-AS1	+	chr13:45377060-45377215	chr13:45377139-45377215	0.22	1.98E-04
MXE	GAS5	-	chr1:173865856-173865894	chr1:173866527-173866567	0.25	3.77E-04
ES	GAS5	-	chr1:173864674-173864704	NA	0.223	5.29E-04
ES	RP11-319G6.1	+	chr3:139419556-139419680	NA	0.16	5.72E-04
MXE	PKD1P6-NPIPP1	-	chr16:15118925-15119054	chr16:15125690-15125807	0.146	7.72E-04
MXE	PSMA3-AS1	-	chr14:58273978-58274010	chr14:58285590-58285756	0.377	1.14E-03
ES	WAC-AS1	-	chr10:28525947-28526008	NA	0.149	1.17E-03
ES	TTC28-AS1	+	chr22:27989881-27990026	NA	0.514	1.92E-03
ASS	PCBP1-AS1	-	chr2:70085815-70086273	chr2:70086123-70086273	0.138	1.97E-03
ES	FOXD3-AS1	-	chr1:63321664-63322080	NA	0.279	2.72E-03
MXE	STAG3L5P-PVRIG2P-PILRB	+	chr7:100338772-100338889	chr7:100345864-100345968	0.111	2.96E-03
ES	PCBP1-AS1	-	chr2:70059642-70059680	NA	0.334	3.39E-03
ES	RP11-295P9.3	+	chr10:13654088-13654258	NA	0.33	3.52E-03
ASS	PCBP1-AS1	-	chr2:70086054-70086269	chr2:70086123-70086269	0.295	3.79E-03
ES	PCBP1-AS1	-	chr2:70053730-70053792	NA	0.657	3.87E-03
ES	PVT1	+	chr8:128070159-128070272	NA	0.138	3.88E-03
ES	TTC28-AS1	+	chr22:27935150-27935230	NA	0.145	4.42E-03
ES	DHRS4-AS1	-	chr14:23953773-23954033	NA	0.213	4.87E-03
RI	SNHG17	-	chr20:38421005-38422241	NA	0.299	4.90E-03
ASS	PSMA3-AS1	-	chr14:58273978-58274135	chr14:58273978-58274010	0.23	5.08E-03
ASS	ST3GAL6-AS1	-	chr3:98718418-98718521	chr3:98718423-98718521	0.268	5.49E-03
ES	RP11-580I16.2	-	chr17:47626546-47626627	NA	0.524	6.17E-03
ES	LINC00969	+	chr3:195685817-195685939	NA	0.268	7.19E-03
ES	RP11-499E18.1	-	chr4:102419696-102419794	NA	0.535	9.47E-03
ASS	DUXAP8	+	chr22:15790660-15790798	chr22:15790684-15790798	0.136	0.01002753622
ES	ST3GAL6-AS1	-	chr3:98718423-98718521	NA	0.384	0.01134847022
ES	TRAF3IP2-AS1	+	chr6:111574620-111574658	NA	0.317	0.01210955425
MXE	TMEM161B-AS1	+	chr5:88287435-88287622	chr5:88410072-88410194	0.224	0.0129113373
MXE	PCBP1-AS1	-	chr2:70059642-70059680	chr2:70083537-70083687	0.181	0.01308646593
MXE	RP11-274B21.14	+	chr7:128578889-128578933	chr7:128579998-128580094	0.251	0.01368807562
ES	NOVA1-AS1	+	chr14:26797913-26798030	NA	0.122	0.01371870462
MXE	PCBP1-AS1	-	chr2:70059642-70059680	chr2:70083506-70083687	0.183	0.01562606437
ES	CD27-AS1	-	chr12:6450340-6450980	NA	0.27	0.01669192307
ES	C21orf62-AS1	+	chr21:32788611-32788701	NA	0.385	0.01673793337
ES	CD27-AS1	-	chr12:6450869-6450980	NA	0.313	0.01675843214
MXE	PKD1P6-NPIPP1	-	chr16:15125690-15125807	chr16:15127469-15127596	0.32	0.01727736852
MXE	TPT1-AS1	+	chr13:45383090-45383235	chr13:45389734-45389820	0.107	0.018188216
MXE	AC005154.6	-	chr7:30576563-30576710	chr7:30584710-30584808	0.278	0.02127345004
ES	CTC-444N24.6	-	chr19:57278115-57278182	NA	0.214	0.02156029198
ASS	RP4-639F20.1	+	chr1:94962924-94963270	chr1:94962978-94963270	0.159	0.02193666938
MXE	ARMCX5-GPRASP2	+	chrX:102601879-102601977	chrX:102639399-102639533	0.154	0.02227107106
ES	ZNF561-AS1	+	chr19:9627715-9627806	NA	0.149	0.02302160623
ES	THAP9-AS1	-	chr4:82897988-82898062	NA	0.122	0.02355317783
ES	CD27-AS1	-	chr12:6450892-6450980	NA	0.337	0.02475961249
ES	RP11-295P9.3	+	chr10:13652845-13653148	NA	0.259	0.0255521065
ES	SNHG11	+	chr20:38447929-38448093	NA	0.161	0.02738999534
ES	SNHG14	+	chr15:25111764-25111894	NA	0.173	0.02967749663
ES	FIRRE	-	chrX:131795808-131796331	NA	0.578	0.03072258172
ES	MIATNB	+	chr22:26693680-26693836	NA	0.193	0.0333244848
ES	FAM66C	+	chr12:8187731-8188225	NA	0.229	0.03557962837
ES	THAP9-AS1	-	chr4:82897988-82898062	NA	0.201	0.03668129886
MXE	RP11-421L21.3	+	chr1:101075394-101075716	chr1:101083460-101083574	0.215	0.03685287948
ES	RP11-421L21.3	+	chr1:101077362-101077508	NA	0.196	0.03803544067
ES	LINC01021	+	chr5:27477638-27477801	NA	0.121	0.03820251916
MXE	PCBP1-AS1	-	chr2:70018014-70018131	chr2:70051202-70051305	0.165	0.04040093942
MXE	PCBP1-AS1	-	chr2:70018014-70018131	chr2:70051202-70051305	0.198	0.04071530613

PN						
event.type	lncRNA	strand	g.coord_1	g.coord_2	inc.level.difference	FDR
MXE	GAS5	-	chr1:173865470-173865547	chr1:173865856-173865894	-0.266	0
MXE	GAS5	-	chr1:173864674-173864704	chr1:173865228-173865282	-0.224	0
ES	ZFAS1	+	chr20:49280484-49280570	NA	-0.112	5.59E-14
ES	PSMA3-AS1	-	chr14:58293065-58293138	NA	-0.417	1.58E-13
ES	TMEM161B-AS1	+	chr5:88287435-88287622	NA	-0.534	8.81E-13
MXE	GAS5	-	chr1:173864483-173864506	chr1:173865228-173865282	-0.124	6.96E-12
ES	MAPKAPK5-AS1	-	chr12:111840332-111840477	NA	-0.213	3.75E-11
ES	SNHG5	-	chr6:85677790-85677875	NA	-0.106	4.04E-11
ES	AC074286.1	-	chr2:177332141-177332251	NA	-0.616	6.59E-11
ES	ZNF667-AS1	+	chr19:56491576-56491766	NA	-0.819	1.32E-10
MXE	RASSF8-AS1	-	chr12:25952757-25953076	chr12:25956226-25956347	-0.53	9.32E-10
MXE	GAS5	-	chr1:173865470-173865547	chr1:173866176-173866206	-0.232	1.87E-09
ES	TMEM161B-AS1	+	chr5:88287435-88287622	NA	-0.436	4.27E-09
ES	SNHG5	-	chr6:85677794-85677868	NA	-0.124	3.33E-08
ES	SNHG5	-	chr6:85677794-85677899	NA	-0.128	7.18E-08
RI	CTC-308K20.1	-	chr5:172957153-172958257	NA	-0.484	1.27E-07
ES	HCG18	-	chr6:30314268-30314482	NA	-0.478	1.30E-07
ES	TMEM161B-AS1	+	chr5:88283009-88283086	NA	-0.54	1.95E-07
ES	ARMCX5-GPRASP2	+	chrX:102601879-102601977	NA	-0.266	1.98E-07
ES	RGMB-AS1	-	chr5:98771655-98771766	NA	-0.667	2.10E-07
ES	SNHG14	+	chr15:25053265-25053363	NA	-0.65	2.78E-07
MXE	TPT1-AS1	+	chr13:45378529-45378671	chr13:45383090-45383235	-0.625	6.05E-07
ES	DLEU2	-	chr13:50049583-50049662	NA	-0.345	6.76E-07
ES	BDNF-AS	+	chr11:27658240-27658462	NA	-0.274	1.00E-06
ES	RP11-421L21.3	+	chr1:101075394-101075716	NA	-0.362	1.04E-06
RI	A1BG-AS1	+	chr19:58353713-58355183	NA	-0.563	1.09E-06
ES	DLEU2	-	chr13:50044650-50044768	NA	-0.404	1.13E-06
ES	LINC00969	+	chr3:195663623-195663832	NA	-0.32	2.17E-06
ES	SLCO4A1-AS1	-	chr20:62664923-62665497	NA	-0.273	2.23E-06
ES	RP11-421L21.3	+	chr1:101080388-101080613	NA	-0.634	2.30E-06
ES	RP11-9G1.3	-	chr4:133140585-133140659	NA	-0.468	2.41E-06
ES	PCBP1-AS1	-	chr2:70055713-70055910	NA	-0.436	2.42E-06
ES	TMEM161B-AS1	+	chr5:88285740-88285851	NA	-0.317	2.86E-06
ES	AC007566.10	+	chr7:92462957-92463026	NA	-0.228	3.06E-06
MXE	TPT1-AS1	+	chr13:45379194-45379335	chr13:45383090-45383235	-0.547	3.37E-06
ES	DLGAP1-AS1	+	chr18:3596578-3596675	NA	-0.304	4.07E-06
ES	CCDC18-AS1	-	chr1:93318167-93318228	NA	-0.794	6.70E-06
MXE	SLCO4A1-AS1	-	chr20:62664923-62665497	chr20:62665859-62666010	-0.113	7.86E-06
ES	PVT1	+	chr8:127983903-127984204	NA	-0.173	1.02E-05
ES	PSMA3-AS1	-	chr14:58285590-58285756	NA	-0.308	1.18E-05
ASS	LRRC75A-AS1	+	chr17:16463163-16463317	chr17:16463166-16463317	-0.451	1.54E-05
RI	SNHG14	+	chr15:25087466-25089866	NA	-0.204	1.55E-05
ES	TPT1-AS1	+	chr13:45390757-45390902	NA	-0.148	1.99E-05
ES	PVT1	+	chr8:128099369-128099573	NA	-0.182	2.16E-05
MXE	GAS5	-	chr1:173865228-173865547	chr1:173866176-173866206	-0.198	2.31E-05
ES	LINC00910	-	chr17:43387935-43388057	NA	-0.294	2.51E-05
ES	TPT1-AS1	+	chr13:45390713-45390902	NA	-0.109	2.67E-05
MXE	TPT1-AS1	+	chr13:45379194-45379335	chr13:45383090-45383235	-0.37	3.21E-05
MXE	LINC00630	+	chrX:102816991-102817082	chrX:102825992-102826169	-0.477	4.09E-05
ES	PSMA3-AS1	-	chr14:58293765-58295811	NA	-0.201	4.24E-05
ES	RP11-42I10.1	+	chr16:48671860-48672033	NA	-0.342	6.74E-05
ES	PRR34-AS1	+	chr22:46055598-46055733	NA	-0.455	7.54E-05
ES	PSMA3-AS1	-	chr14:58273978-58274135	NA	-0.237	7.74E-05
MXE	GAS5	-	chr1:173865470-173865547	chr1:173866527-173866567	-0.194	8.27E-05
MXE	TPT1-AS1	+	chr13:45379925-45380001	chr13:45383090-45383235	-0.293	8.84E-05
MXE	TPT1-AS1	+	chr13:45379925-45380001	chr13:45383090-45383235	-0.308	9.27E-05
MXE	PVT1	+	chr8:127939507-127939676	chr8:127983903-127984204	-0.137	0.000100701606
MXE	GUSBP11	-	chr22:23695172-23695517	chr22:23700725-23700845	-0.284	0.000126545078
ES	RNASEH1-AS1	+	chr2:3559447-3559729	NA	-0.187	0.000171719075
RI	RP11-295P9.3	+	chr10:13654352-13655482	NA	-0.475	0.000173568272
MXE	TPT1-AS1	+	chr13:45379194-45380001	chr13:45383090-45383235	-0.377	0.000188143965
ES	LINC00630	+	chrX:102825992-102826169	NA	-0.467	0.000207640945
ES	SOX9-AS1	-	chr17:72074548-72076733	NA	-0.145	0.000265122834
MXE	PCBP1-AS1	-	chr2:70055713-70055910	chr2:70059642-70059680	-0.379	0.000272505190
MXE	TPT1-AS1	+	chr13:45379194-45380001	chr13:45383090-45383235	-0.397	0.000310183700
MXE	CD27-AS1	-	chr12:6448639-6448737	chr12:6450340-6450980	-0.223	0.000313158994
MXE	RP11-421L21.3	+	chr1:101075275-101075394	chr1:101075394-101075716	-0.198	0.000313158994
ES	RP11-10K16.1	-	chr4:173166644-173166790	NA	-0.284	0.000342014704
ES	ST3GAL6-AS1	-	chr3:98715649-98715777	NA	-0.232	0.000373111134
ES	RP4-614C10.2	-	chr2:89580557-89580635	NA	-0.337	0.000398047305
ASS	ZRANB2-AS2	+	chr1:71237129-71237386	chr1:71237129-71237195	-0.531	0.000401095345
ES	RP13-968A2.1	-	chr7:99299463-99299666	NA	-0.667	0.000406792612
MXE	STAG3L5P-PVRIG2P-PILRB	+	chr7:100349798-100349887	chr7:100351251-100351410	-0.142	0.000459483911
ES	PSMA3-AS1	-	chr14:58285590-58285756	NA	-0.289	0.000525528662
MXE	LINC00894	+	chrX:149942183-149942329	chrX:149943650-149943762	-0.445	0.000560198685
ES	ARMCX5-GPRASP2	+	chrX:102601463-102601509	NA	-0.358	0.000666033342
ES	CD27-AS1	-	chr12:6443545-6443717	NA	-0.321	0.000758051464
ES	RP11-156P1.3	-	chr17:47072471-47072559	NA	-0.494	0.000788106904
ES	LINC00174	-	chr7:66400321-66400437	NA	-0.314	0.000859083617
ES	WEE2-AS1	-	chr7:141714188-141714374	NA	-0.275	0.000891391094
ES	PCBP1-AS1	-	chr2:70085815-70085903	NA	-0.291	0.000899841131
ES	RP11-274B21.14	+	chr7:128578889-128578933	NA	-0.501	0.000902998005
ASS	SNHG8	+	chr4:118278708-118279137	chr4:118278708-118278792	-0.114	0.000990167683
ES	RP11-421L21.3	+	chr1:101083460-101083574	NA	-0.185	0.001013602768
ES	PVT1	+	chr8:127983903-127984204	NA	-0.228	0.001136224867
ES	BDNF-AS	+	chr11:27675576-27676140	NA	-0.343	0.001406429329
ES	POLR2J4	-	chr7:43986600-43986677	NA	-0.327	0.001407114613
ES	LINC00969	+	chr3:195696360-195696589	NA	-0.207	0.001482125936
MXE	ARMCX5-GPRASP2	+	chrX:102601463-102601509	chrX:102601879-102601977	-0.371	0.001699714909
ES	BDNF-AS	+	chr11:27675296-27676140	NA	-0.368	0.00177508727
ES	RP11-1228E12.1	-	chr17:71365-71556	NA	-0.667	0.001952422279
MXE	LINC00894	+	chrX:149941789-149941847	chrX:149943650-149943762	-0.4	0.002052545047
MXE	LINC00630	+	chrX:102816991-102817082	chrX:102825992-102826169	-0.413	0.002188454797
ES	KB-1572G7.2	-	chr22:23686837-23686995	NA	-0.382	0.002398430612
ES	RP11-421L21.3	+	chr1:101075394-101075716	NA	-0.238	0.002624894621
ES	ZBED5-AS1	+	chr11:10865260-10865327	NA	-0.129	0.003016790343
ES	MIR99AHG	+	chr21:16537378-16537439	NA	-0.119	0.003127023316
ES	ARF4-AS1	+	chr3:57600058-57600169	NA	-0.46	0.003887141779

ES	LRRC75A-AS1	+	chr17:16460892-16461245	NA	0.218	0.04305333938
MXE	CCDC18-AS1	-	chr1:93315148-93315269	chr1:93318167-93318228	0.386	0.04922057688

ASS	PCBP1-AS1	-	chr2:70083506-70083687	chr2:70083537-70083687	-0.299	0.004120571796
ES	TPT1-AS1	+	chr13:45360140-45360239	NA	-0.619	0.004168487247
ES	NR2F1-AS1	-	chr5:93438702-93438741	NA	-0.288	0.004687272022
ES	UBL7-AS1	+	chr15:74466035-74466130	NA	-0.286	0.00472504306
ASS	KB-1572G7.2	-	chr22:23686830-23686995	chr22:23686837-23686995	-0.224	0.005345968046
ES	ALOX12-AS1	-	chr17:7010058-7010137	NA	-0.167	0.005410559041
MXE	PSMA3-AS1	-	chr14:58285590-58285756	chr14:58291634-58291707	-0.248	0.005483540331
ES	PSMA3-AS1	-	chr14:58293313-58293544	NA	-0.102	0.005554562012
MXE	TTC28-AS1	+	chr22:27935150-27935230	chr22:27993123-27993535	-0.166	0.007663048107
ES	SNHG14	+	chr15:24998969-24999078	NA	-0.19	0.007705740173
MXE	LINC00174	-	chr7:66455658-66455853	chr7:66467681-66467719	-0.442	0.008104896287
ES	KB-1572G7.2	-	chr22:23686830-23686995	NA	-0.362	0.008304198588
MXE	GAS5	-	chr1:173866760-173866796	chr1:173866990-173867043	-0.166	0.008854126289
ES	ZBED3-AS1	+	chr5:77100235-77100391	NA	-0.276	0.009747054499
MXE	PCBP1-AS1	-	chr2:70055655-70055910	chr2:70059642-70059680	-0.27	0.01020578741
ES	LINC01001	-	chr11:134563-134796	NA	-0.533	0.01082847443
ES	RP11-216L13.19	+	chr9:136809254-136809429	NA	-0.207	0.01082847443
ES	SNHG3	+	chr1:28508127-28508160	NA	-0.173	0.0111445589
ES	OSER1-AS1	+	chr20:44214887-44215003	NA	-0.236	0.01202849288
ES	CTD-2651B20.3	-	chr15:45264626-45264809	NA	-0.287	0.01243842924
MXE	THAP9-AS1	-	chr4:82895691-82895774	chr4:82897988-82898062	-0.118	0.01323461605
MXE	PCBP1-AS1	-	chr2:70055713-70055910	chr2:70059642-70059680	-0.246	0.01388670621
MXE	PCBP1-AS1	-	chr2:70055713-70055910	chr2:70059642-70059680	-0.246	0.01388670621
ES	MIR22HG	-	chr17:1714370-1714453	NA	-0.418	0.01461642436
ES	TMEM44-AS1	+	chr3:194589473-194589626	NA	-0.4	0.01519053624
ES	TMEM161B-AS1	+	chr5:88283009-88283086	NA	-0.3	0.01655644229
ES	MIATNB	+	chr22:26718139-26718231	NA	-0.307	0.01784243899
ES	RBM26-AS1	+	chr13:79417834-79417916	NA	-0.207	0.0179359004
ES	HCG18	-	chr6:30314268-30314482	NA	-0.426	0.01832264645
ES	RP11-250B2.3	+	chr6:80454109-80454227	NA	-0.281	0.01900862158
ES	TUG1	+	chr22:30972047-30972172	NA	-0.204	0.02023209143
ES	FAM66C	+	chr12:8195276-8195412	NA	-0.396	0.02043424899
ES	LINC00963	+	chr9:129502396-129503139	NA	-0.244	0.02048193694
ES	CTD-2378E12.1	-	chr15:50890855-50891077	NA	-0.655	0.02204339488
ES	LINC01128	+	chr1:850177-850351	NA	-0.51	0.02372588051
ES	RP11-156P1.3	-	chr17:47033277-47033381	NA	-0.171	0.02472987573
ES	LINC00969	+	chr3:195689338-195689438	NA	-0.151	0.02605534879
ES	TMEM161B-AS1	+	chr5:88285740-88285851	NA	-0.2	0.02616662861
ES	CCDC18-AS1	-	chr1:93318167-93318228	NA	-0.312	0.02759347156
ES	AC108488.4	-	chr2:3533107-3533190	NA	-0.211	0.02986419007
MXE	TPT1-AS1	+	chr13:45379898-45380001	chr13:45383090-45383235	-0.116	0.03030437867
ES	RP11-274B21.14	+	chr7:128579998-128580094	NA	-0.156	0.0314556154
MXE	PCBP1-AS1	-	chr2:70055713-70055910	chr2:70059642-70059680	-0.431	0.03469929876
ES	PSMG3-AS1	+	chr7:1579286-1579404	NA	-0.294	0.03625932837
ES	LINC00963	+	chr9:129502396-129503139	NA	-0.268	0.03797039936
ES	RASSF8-AS1	-	chr12:25947518-25947622	NA	-0.397	0.0415224856
MXE	RP11-421L21.3	+	chr1:101077362-101077508	chr1:101083460-101083574	-0.235	0.04252502176
ES	AC091729.9	+	chr7:1162351-1162543	NA	-0.173	0.04292658489
MXE	LINC01778	+	chr1:30826554-30826652	chr1:30829747-30829882	-0.203	0.04357236015
ES	LINC00630	+	chrX:102839800-102839863	NA	-0.285	0.04393571592
ES	CD27-AS1	-	chr12:6450473-6450708	NA	-0.232	0.0496313164

Supplementary Table 25: Differentially spliced and expressed lncRNAs in mesenchymal (MES) and proneural (PN) GSCs samples.

ASS (alternative 5'or 3'splice site): genomic coordinates of long and short exons (columns g.coord_1 and g.coord_2)

MXE (mutually exclusive exon): genomic coordinates of first and EScond exons (columns g.coord_1 and g.coord_2)

RI (retained introns): genomic coordinates of retained intron (column g.coord_1)

ES (exon skipping): genomic coordinates of skipped exon (column g.coord_1)

	Differential expression		Differential splicing					
**lncRNA**	**log2FoldChange**	**FDR**	**event.type**	**strand**	**g.coord_1**	**g.coord_2**	**inc.level.difference**	**FDR**
AC005154.6	-2.818045991	1.77E-11	MXE	-	chr7:30576563-30576710	chr7:30584710-30584808	0.278	0.02127345004
AC007566.10	-1.014212726	0.04603914804	ES	+	chr7:92462957-92463026	NA	0.228	3.06E-06
AC108488.4	-1.73751869	0.01934800273	ES	-	chr2:3533107-3533190	NA	0.211	0.02986419007
ARMCX5-GPRASP2	-1.407923176	0.00109723521	ES	+	chrX:102601879-102601977	NA	0.266	1.98E-07
			ES	+	chrX:102601463-102601509	NA	0.358	0.000666033342
			MXE	+	chrX:102601463-102601509	chrX:102601879-102601977	-0.371	0.001699714909
			MXE	+	chrX:102601879-102601977	chrX:102639399-102639533	0.154	0.02227107106
BDNF-AS	-2.440499658	0.000270164116	ES	+	chr11:27658240-27658462	NA	0.274	1.00E-06
			ES	+	chr11:27675576-27676140	NA	0.343	0.001406429329
			ES	+	chr11:27675296-27676140	NA	0.368	0.00177508727
CD27-AS1	-1.901100354	0.000615687138	MXE	-	chr12:6448639-6448737	chr12:6450340-6450980	-0.223	0.000313158994
			ES	-	chr12:6443545-6443717	NA	0.321	0.000758051464
			ES	-	chr12:6450340-6450980	NA	-0.27	0.01669192307
			ES	-	chr12:6450869-6450980	NA	-0.313	0.01675843214
			ES	-	chr12:6450892-6450980	NA	-0.337	0.02475961249
			ES	-	chr12:6450473-6450708	NA	0.232	0.0496313164
CTC-308K20.1	4.217809789	3.39E-10	RI	-	chr5:172957153-172958257	NA	-0.484	1.27E-07
CTD-2341M24.1	1.475568801	0.03769661099	ES	+	chr14:86128664-86128773	NA	-0.612	5.70E-05
FAM66C	-1.945290987	3.52E-05	ES	+	chr12:8195276-8195412	NA	0.396	0.02043424899
			ES	+	chr12:8187731-8188225	NA	-0.229	0.03557962837
GAS5	1.714774757	0.001737349134	A5SS	-	chr1:173865470-173865547	chr1:173865509-173865547	0.477	0
			MXE	-	chr1:173865509-173865547	chr1:173866176-173866206	0.233	0
			MXE	-	chr1:173864674-173864704	chr1:173865228-173865282	-0.224	0
			MXE	-	chr1:173865856-173865894	chr1:173866527-173866796	0.254	0
			RI	-	chr1:173864256-173865282	NA	0.15	0
			MXE	-	chr1:173865470-173865547	chr1:173865856-173865894	-0.266	0
			RI	-	chr1:173865228-173865547	NA	0.436	0
			MXE	-	chr1:173865509-173865547	chr1:173865856-173866206	0.274	0
			MXE	-	chr1:173865856-173865894	chr1:173866176-173866206	0.232	0
			MXE	-	chr1:173864483-173864506	chr1:173865228-173865282	-0.124	6.96E-12
			MXE	-	chr1:173865509-173865547	chr1:173865856-173865894	0.154	2.88E-11
			MXE	-	chr1:173864674-173864704	chr1:173865228-173865282	0.206	5.01E-11
			RI	-	chr1:173864483-173864704	NA	0.206	4.03E-10
			MXE	-	chr1:173865470-173865547	chr1:173866176-173866206	-0.232	1.87E-09
			RI	-	chr1:173864256-173864506	NA	0.171	1.09E-08
			MXE	-	chr1:173865856-173865894	chr1:173866760-173866796	0.229	1.36E-07
			MXE	-	chr1:173865228-173865282	chr1:173865470-173865547	0.121	1.59E-05
			MXE	-	chr1:173865228-173865547	chr1:173866176-173866206	-0.198	2.31E-05
			MXE	-	chr1:173865509-173865547	chr1:173866527-173866567	0.214	6.74E-05
			MXE	-	chr1:173865470-173865547	chr1:173866527-173866567	-0.194	8.27E-05
			MXE	-	chr1:173865856-173865894	chr1:173866527-173866567	0.25	0.000377180693
			ES	-	chr1:173864674-173864704	NA	-0.223	0.000528708466
			MXE	-	chr1:173866760-173866796	chr1:173866990-173867043	-0.166	0.008854126289
LINC00910	4.052061325	4.78E-14	ES	-	chr17:43387935-43388057	NA	0.294	2.51E-05
LINC01001	2.271547507	0.006862132394	ES	-	chr11:134563-134796	NA	0.533	0.01082847443
LINC01021	1.955564405	0.0116975285	ES	+	chr5:27477638-27477801	NA	-0.121	0.03820251916
LINC01778	-2.722293474	0.006404109579	MXE	+	chr1:30826554-30826652	chr1:30829747-30829882	-0.203	0.04357236015
MIR99AHG	-3.934916524	1.08E-11	ES	+	chr21:16537378-16537439	NA	0.119	0.003127023316
NOVA1-AS1	2.538877294	6.37E-07	ES	+	chr14:26797913-26798030	NA	-0.122	0.01371870462
NR2F1-AS1	-2.868865851	2.32E-06	ES	-	chr5:93438702-93438741	NA	0.288	0.004687272022
PSMA3-AS1	-1.50008569	1.84E-05	ES	-	chr14:58293065-58293138	NA	0.417	1.58E-13
			ES	-	chr14:58288717-58288813	NA	-0.432	7.31E-06
			ES	-	chr14:58285590-58285756	NA	0.308	1.18E-05
			ES	-	chr14:58293765-58295811	NA	0.201	4.24E-05
			ES	-	chr14:58273978-58274135	NA	0.237	7.74E-05

			ES	-	chr14:58285590-58285756	NA	0.289	0.000525528662
			MXE	-	chr14:58273978-58274010	chr14:58285590-58285756	0.377	0.001136202548
			A3SS	-	chr14:58273978-58274135	chr14:58273978-58274010	0.23	0.00508214036
			MXE	-	chr14:58285590-58285756	chr14:58291634-58291707	-0.248	0.005483540331
			ES	-	chr14:58293313-58293544	NA	0.102	0.005554562012
PVT1	1.306446058	0.004494224275	ES	+	chr8:127983903-127984204	NA	0.173	1.02E-05
			ES	+	chr8:128099369-128099573	NA	0.182	2.16E-05
			MXE	+	chr8:127939507-127939676	chr8:127983903-127984204	-0.137	0.000100701606
			ES	+	chr8:127983903-127984204	NA	0.228	0.001136224867
			ES	+	chr8:128070159-128070272	NA	-0.138	0.003876184563
RBM26-AS1	-1.372105486	0.01108391598	ES	+	chr13:79417834-79417916	NA	0.207	0.0179359004
RGMB-AS1	-2.82462874	0.02763328829	ES	-	chr5:98771655-98771766	NA	0.667	2.10E-07
RP11-1228E12.1	1.830938095	0.03593843095	ES	-	chr17:71365-71556	NA	0.667	0.001952422279
RP11-156P1.3	-1.092878188	0.03206607327	ES	-	chr17:47072471-47072559	NA	0.494	0.000788106904
			ES	-	chr17:47033277-47033381	NA	0.171	0.02472987573
RP4-639F20.1	2.662459679	8.82E-09	A3SS	+	chr1:94962924-94963270	chr1:94962978-94963270	0.159	0.02193666938
SNHG11	-1.126572362	0.02635014089	ES	+	chr20:38447929-38448093	NA	-0.161	0.02738999534
SNHG3	2.007679738	9.72E-05	ES	+	chr1:28508127-28508160	NA	0.173	0.0111445589
SNHG6	1.619245894	1.16E-05	ES	-	chr8:66922613-66922725	NA	-0.151	0
SNHG8	3.401142657	2.65E-08	A5SS	+	chr4:118278708-118279137	chr4:118278708-118278792	-0.114	0.000990167683
TPT1-AS1	-2.889017128	3.55E-10	MXE	+	chr13:45378529-45378671	chr13:45383090-45383235	-0.625	6.05E-07
			MXE	+	chr13:45379194-45379335	chr13:45383090-45383235	-0.547	3.37E-06
			A3SS	+	chr13:45390713-45390902	chr13:45390757-45390902	0.12	1.61E-05
			ES	+	chr13:45390757-45390902	NA	0.148	1.99E-05
			ES	+	chr13:45390713-45390902	NA	0.109	2.67E-05
			MXE	+	chr13:45379194-45379335	chr13:45383090-45383235	-0.37	3.21E-05
			MXE	+	chr13:45379925-45380001	chr13:45383090-45383235	-0.293	8.84E-05
			MXE	+	chr13:45379925-45380001	chr13:45383090-45383235	-0.308	9.27E-05
			MXE	+	chr13:45379194-45380001	chr13:45383090-45383235	-0.377	0.000188143965
			A3SS	+	chr13:45377060-45377215	chr13:45377139-45377215	0.22	0.00019763891
			MXE	+	chr13:45379194-45380001	chr13:45383090-45383235	-0.397	0.000310183700
			ES	+	chr13:45360140-45360239	NA	0.619	0.004168487247
			MXE	+	chr13:45383090-45383235	chr13:45389734-45389820	0.107	0.018188216
			MXE	+	chr13:45379898-45380001	chr13:45383090-45383235	-0.116	0.03030437867
TRAF3IP2-AS1	-1.702715019	3.10E-05	ES	+	chr6:111574620-111574658	NA	-0.317	0.01210955425
WEE2-AS1	-1.371126783	0.04272802559	ES	-	chr7:141714188-141714374	NA	0.275	0.000891391094
ZBED3-AS1	-1.506734757	0.00271888178	ES	+	chr5:77100235-77100391	NA	0.276	0.009747054499
ZNF561-AS1	-2.939397831	9.05E-11	ES	+	chr19:9627715-9627806	NA	-0.149	0.02302160623
ZNF582-AS1	-7.903489168	9.86E-09	RI	+	chr19:56398276-56399168	NA	0.809	0

Supplementary Table 26: List of primers used for validation of splicing events by qRT-PCR.

gene	exon	Primer Forward (5' - 3')	Primer Reverse (5' - 3')
OSBPL6	constitutive	ATGTGGGACTCTCAGTCAT	CTTCGGTGTCAAGGTCTATTC
	alternative	GCTAACTGTGTAGATATTTCAAAGAA	TTTGTATCTTTGCTGACACTTT
RAB18	constitutive	GCTGGTCAAGAGAGGTTTAG	ACCCTGTGCACCTCTATAA
	alternative	TTACTTTGCATCAGCAGACT	CCACTTCAGGATTGGACTATTT
ELMOD3	constitutive	CAAGGAAGGCAAAGGTAGAG	CTATGTGGAGAGCCAGAGAT
	alternative	GAAAGCTGGTCACGATTCT	ACAGGCGGGTATGGTAATA
WASF3	constitutive	ACTACTGATAACTGAGCCAAAG	CATGTAATCGTTGACTATGGGT
	alternative	GCTGAACCCTAACAGAAACC	CCTTCTCTCCCACTCTTCTT
SLC9A5	constitutive	GGGAGCAGTTTGACAAGAA	CCTGTAGTACACATCCCAGA
	alternative	GGATAGAGCTGAGGACAGA	CATGTCAGTCTGGAGGAAAG
GSN	constitutive	CTCTCTTTGTCCAGTGCTTC	TGTGCAGGGTGAAGAGT
	alternative	CAGATACAGCGCTAGGAAA	TCCATTCTAGACCTGAATTACTC
FDFT1	constitutive	CAGAACGTGCCAACTCTATG	TTCCTCCTTGCTGGTCTT
	alternative	GTCCTGGCTGACCTGT	AACCACCGGGAACCA
B2M	endogenous	TGCCGTGTGAACCATGTGA	GCTTACATGTCTCGATCCCACT

Supplementary References

1) Lawrence, Michael S., Petar Stojanov, Craig H. Mermel, James T. Robinson, Levi A. Garraway, Todd R. Golub, Matthew Meyerson, Stacey B. Gabriel, Eric S. Lander, and Gad Getz. 2014. "Discovery and Saturation Analysis of Cancer Genes across 21 Tumour Types." *Nature* 505 (7484): 495–501.

2) Vogelstein, Bert, Nickolas Papadopoulos, Victor E. Velculescu, Shibin Zhou, Luis A. Diaz Jr, and Kenneth W. Kinzler. 2013. "Cancer Genome Landscapes." *Science* 339 (6127): 1546–58.

3) Tamborero, David, Abel Gonzalez-Perez, Christian Perez-Llamas, Jordi Deu-Pons, Cyriac Kandoth, Jüri Reimand, Michael S. Lawrence, et al. 2013. "Comprehensive Identification of Mutational Cancer Driver Genes across 12 Tumor Types." *Scientific Reports* 3 (October): 2650.
